# Supplementary material for: Ductal or Ngn3+ cells do not contribute to adult pancreatic islet beta-cell neogenesis in homeostasis
Source: EMBO J. 2025 Apr 9;44(10):2856–81. doi: 10.1038/s44318-025-00434-z (PMC12084597; doi:10.1038/s44318-025-00434-z)
Supplement: Supplementary file 1 — Appendix [file 44318_2025_434_MOESM1_ESM.pdf]

Appendix Table

Ductal or Ngn3$^+$ cells do not contribute to adult pancreatic islet beta-cell neogenesis in homeostasis

Xiuzhen Huang, Huan Zhao, Hui Chen, Zixin Liu, Kuo Liu, Zan Lv, Xiuxiu Liu, Ximeng Han, Maoying Han, Jie Lu, Qiao Zhou & Bin Zhou

This PDF file includes:

Appendix Table S1...................Page 1
Appendix Table S2...................Page 2
Appendix Table S3...................Page 3
Appendix Table S4...................Page 4
Appendix Table S5...................Page 5
Appendix Table S6...................Page 6
Appendix Table S7...................Page 7
Appendix Table S8...................Page 8
Appendix Table S9...................Page 9
Appendix Table S10.................Page 10
Appendix Table S11.................Page 11

Figure 1F

	Ngn3-CreER;R26-tdT (+2w)										*Ngn3-CreER;R26-tdT* (+12w)							
	Sample1		Sample2		Sample3		Sample4		Sample5		Sample1		Sample2		Sample3		Sample4	
	tdT+Ins+	Ins+	tdT+Ins+	Ins+	tdT+Ins+	Ins+	tdT+Ins+	Ins+	tdT+Ins+	Ins+	tdT+Ins+	Ins+	tdT+Ins+	Ins+	tdT+Ins+	Ins+	tdT+Ins+	Ins+
Section1	129	819	65	598	221	629	58	502	63	689	176	722	79	507	308	1290	169	922
Section2	103	611	102	960	176	523	75	608	71	748	95	435	178	995	177	568	235	998
Section3	121	854	78	925	315	872	81	673	82	892	143	611	185	1132	164	609	307	1279
Section4	130	744	81	978	243	707	98	736	72	639	83	310	151	923	242	885	134	664
Section5	97	627	124	1103	261	724	103	891	79	767	135	495	119	757	205	799	213	901
Section6	183	1065	85	912	170	499	145	1194	43	344	184	673	98	623	236	737	169	853
Section7	165	931	79	860	213	581	122	1046	105	1271	208	846	85	495	175	495	158	702
Section8	69	465	40	344	385	1139	84	594	78	752	145	511	157	830	256	844	107	420
Section9	164	940	72	612	314	864	93	778	92	983	193	1108	134	619	358	1098	206	766
Section10	122	837	54	499	213	518	71	476	73	452	355	977	145	996	153	596	214	951
Total	1283	7893	780	7791	2511	7056	930	7498	758	7537	1717	6688	1331	7877	2274	7921	1912	8456
**Percent**	**16.25%**		**10.01%**		**35.59%**		**12.40%**		**10.06%**		**25.67%**		**16.90%**		**28.71%**		**22.61%**	

	Ngn3-CreER;R26-tdT (+2w)										*Ngn3-CreER;R26-tdT* (+12w)							
	Sample1		Sample2		Sample3		Sample4		Sample5		Sample1		Sample2		Sample3		Sample4	
	tdT+Sst+	Sst+	tdT+Sst+	Sst+	tdT+Sst+	Sst+	tdT+Sst+	Sst+	tdT+Sst+	Sst+	tdT+Sst+	Sst+	tdT+Sst+	Sst+	tdT+Sst+	Sst+	tdT+Sst+	Sst+
Section1	85	87	59	63	51	62	48	54	77	79	67	69	85	91	119	134	80	87
Section2	63	69	75	77	47	54	65	69	62	63	47	48	96	103	48	57	86	91
Section3	68	73	84	89	68	78	68	71	93	95	51	52	50	56	64	69	107	113
Section4	73	79	89	92	56	69	47	52	57	58	38	39	72	78	81	95	61	67
Section5	46	54	115	119	48	58	79	83	81	82	44	45	59	62	63	76	77	81
Section6	102	114	74	95	66	79	106	115	42	44	103	106	64	69	65	67	103	109
Section7	67	72	76	83	55	70	102	106	120	121	62	64	68	73	39	45	72	76
Section8	35	38	51	87	74	86	50	58	71	72	52	53	79	84	78	84	45	49
Section9	84	85	62	64	83	94	69	77	92	93	95	96	67	74	97	105	78	83
Section10	63	64	44	46	53	56	42	43	46	46	71	72	85	95	52	64	74	86
Total	686	735	729	815	601	706	676	728	741	753	630	644	725	785	706	796	783	842
**Percent**	**93.33%**		**89.45%**		**85.13%**		**92.86%**		**98.41%**		**97.83%**		**92.36%**		**88.69%**		**92.99%**	

	Ngn3-CreER;R26-tdT (+2w)										*Ngn3-CreER;R26-tdT* (+12w)							
	Sample1		Sample2		Sample3		Sample4		Sample5		Sample1		Sample2		Sample3		Sample4	
	tdT+Gcg+	Gcg+	tdT+Gcg+	Gcg+	tdT+Gcg+	Gcg+	tdT+Gcg+	Gcg+	tdT+Gcg+	Gcg+	tdT+Gcg+	Gcg+	tdT+Gcg+	Gcg+	tdT+Gcg+	Gcg+	tdT+Gcg+	Gcg+
Section1	19	113	7	89	10	69	8	82	19	99	8	95	16	126	27	176	26	149
Section2	14	83	12	128	12	63	10	103	25	105	3	53	22	144	11	87	21	135
Section3	16	95	13	141	21	118	14	109	21	119	5	68	9	75	16	122	15	88
Section4	22	107	7	97	16	75	12	78	17	89	4	53	15	108	29	154	28	114
Section5	10	76	15	182	9	84	17	127	19	103	6	60	14	83	18	117	33	143
Section6	21	152	8	139	8	66	18	155	22	87	12	126	13	97	14	93	35	158
Section7	27	142	11	125	23	122	20	146	29	133	6	86	18	153	10	69	24	120
Section8	8	59	16	137	13	72	9	87	17	84	4	69	22	126	26	145	16	92
Section9	25	135	7	96	17	116	14	105	26	112	13	115	24	141	22	156	18	112
Section10	11	98	14	178	12	89	8	84	14	78	10	94	18	133	35	178	22	127
Total	173	1060	110	1312	141	874	130	1076	209	1009	71	819	171	1186	208	1297	238	1238
**Percent**	**16.32%**		**8.38%**		**16.13%**		**12.08%**		**20.71%**		**8.67%**		**14.42%**		**16.04%**		**19.22%**	

	Ngn3-CreER;R26-tdT (+2w)										*Ngn3-CreER;R26-tdT* (+12w)							
	Sample1		Sample2		Sample3		Sample4		Sample5		Sample1		Sample2		Sample3		Sample4	
	tdT+Ppy+	Ppy+	tdT+Ppy+	Ppy+	tdT+Ppy+	Ppy+	tdT+Ppy+	Ppy+	tdT+Ppy+	Ppy+	tdT+Ppy+	Ppy+	tdT+Ppy+	Ppy+	tdT+Ppy+	Ppy+	tdT+Ppy+	Ppy+
Section1	31	142	7	76	41	178	22	87	18	69	26	133	34	109	56	212	29	133
Section2	24	106	12	98	20	91	19	76	27	103	13	76	38	113	27	112	35	138
Section3	26	112	13	105	37	147	27	88	22	71	15	79	22	67	35	134	21	106
Section4	28	128	15	110	25	103	16	59	45	152	12	68	27	83	44	181	19	97
Section5	23	89	17	127	21	87	18	71	40	148	18	87	21	73	38	146	30	125
Section6	46	188	15	119	38	158	39	138	24	85	35	169	41	86	36	130	38	156
Section7	37	117	8	84	34	129	31	116	59	206	22	106	33	97	19	91	24	104
Section8	12	61	18	128	32	142	17	54	51	174	16	89	37	110	43	162	18	74
Section9	34	136	9	73	40	163	18	67	26	77	30	154	25	78	61	195	20	82
Section10	33	143	18	101	29	106	30	94	25	83	24	118	34	104	32	124	43	174
Total	294	1222	132	1021	317	1304	237	850	337	1168	211	1079	312	920	391	1487	277	1189
**Percent**	**24.06%**		**12.93%**		**24.31%**		**27.88%**		**28.85%**		**19.56%**		**33.91%**		**26.29%**		**23.30%**	

Figure 1N

	Ngn3-2A-CreER;R26-tdT (+2w)										*Ngn3-2A-CreER;R26-tdT* (+12w)									
	Sample1		Sample2		Sample3		Sample4		Sample5		Sample1		Sample2		Sample3		Sample4		Sample5	
	tdT+Ins+	Ins+	tdT+Ins+	Ins+	tdT+Ins+	Ins+	tdT+Ins+	Ins+	tdT+Ins+	Ins+	tdT+Ins+	Ins+	tdT+Ins+	Ins+	tdT+Ins+	Ins+	tdT+Ins+	Ins+	tdT+Ins+	Ins+
Section1	303	780	298	904	281	823	124	485	266	989	85	995	204	956	199	597	318	731	215	609
Section2	331	859	197	586	276	806	215	823	151	562	134	730	153	712	312	914	229	528	321	881
Section3	197	502	406	1195	177	533	196	732	182	661	131	687	106	490	168	537	388	875	256	710
Section4	174	445	361	1065	325	945	168	654	319	1159	118	619	213	989	255	766	406	963	309	875
Section5	355	908	185	533	123	361	225	863	258	927	162	882	189	875	209	628	164	378	296	850
Section6	404	1026	233	680	146	425	153	589	240	873	194	1003	124	577	104	313	291	669	328	931
Section7	234	598	274	827	185	522	211	825	167	642	150	787	177	806	163	473	135	334	165	468
Section8	322	829	269	796	371	1083	224	814	219	809	116	606	109	532	358	1035	224	503	174	546
Section9	338	865	266	788	299	863	142	572	273	952	218	1117	152	755	279	886	345	783	337	937
Section10	241	593	523	1529	118	353	248	951	203	689	277	857	227	957	263	778	336	751	325	909
Total	2899	7405	3012	8903	2301	6714	1906	7308	2278	8263	1585	8283	1654	7649	2310	6927	2836	6515	2726	7716
**Percent**	**39.15%**		**33.83%**		**34.27%**		**26.08%**		**27.57%**		**19.14%**		**21.62%**		**33.35%**		**43.53%**		**35.33%**	

	Ngn3-2A-CreER;R26-tdT (+2w)										*Ngn3-2A-CreER;R26-tdT* (+12w)									
	Sample1		Sample2		Sample3		Sample4		Sample5		Sample1		Sample2		Sample3		Sample4		Sample5	
	tdT+Sst+	Sst+	tdT+Sst+	Sst+	tdT+Sst+	Sst+	tdT+Sst+	Sst+	tdT+Sst+	Sst+	tdT+Sst+	Sst+	tdT+Sst+	Sst+	tdT+Sst+	Sst+	tdT+Sst+	Sst+	tdT+Sst+	Sst+
Section1	62	63	108	114	53	54	48	65	43	55	74	109	73	98	52	75	65	74	65	77
Section2	85	85	55	59	79	80	53	82	62	79	64	93	58	73	58	81	33	42	59	73
Section3	43	44	83	88	64	64	51	73	50	66	52	78	93	117	30	43	74	78	84	91
Section4	51	52	91	96	41	41	55	75	64	83	67	71	66	82	46	66	78	85	76	87
Section5	59	59	48	53	44	45	62	96	47	61	60	98	59	75	39	57	43	53	56	69
Section6	55	56	73	78	58	59	66	89	44	57	73	114	34	46	26	39	61	68	39	49
Section7	68	69	79	84	50	51	69	92	49	54	64	88	61	80	28	37	46	52	56	58
Section8	86	86	88	95	56	57	70	97	71	78	51	74	85	103	65	93	42	47	72	82
Section9	71	72	85	93	85	86	47	68	65	85	69	95	57	74	52	78	54	59	81	92
Section10	82	83	139	142	55	57	72	103	34	58	66	106	87	94	45	67	60	61	88	90
Total	662	669	849	902	585	594	593	840	529	676	640	926	673	842	441	636	556	619	676	768
**Percent**	**98.95%**		**94.12%**		**98.48%**		**70.60%**		**78.25%**		**69.11%**		**79.93%**		**69.34%**		**89.82%**		**88.02%**	

	Ngn3-2A-CreER;R26-tdT (+2w)										*Ngn3-2A-CreER;R26-tdT* (+12w)									
	Sample1		Sample2		Sample3		Sample4		Sample5		Sample1		Sample2		Sample3		Sample4		Sample5	
	tdT+Gcg+	Gcg+	tdT+Gcg+	Gcg+	tdT+Gcg+	Gcg+	tdT+Gcg+	Gcg+	tdT+Gcg+	Gcg+	tdT+Gcg+	Gcg+	tdT+Gcg+	Gcg+	tdT+Gcg+	Gcg+	tdT+Gcg+	Gcg+	tdT+Gcg+	Gcg+
Section1	9	98	22	208	19	106	16	107	11	103	12	189	21	171	25	148	9	145	13	137
Section2	14	138	10	109	32	166	25	138	19	146	14	160	19	126	29	159	6	88	15	129
Section3	7	64	14	161	22	124	21	121	14	123	9	133	29	206	14	90	11	202	17	162
Section4	8	79	18	176	16	83	24	125	22	153	15	121	20	143	24	132	12	165	19	155
Section5	8	91	11	98	15	90	31	163	13	111	13	169	22	131	21	116	14	197	9	119
Section6	10	86	15	143	27	115	26	150	15	109	17	198	10	78	13	83	7	134	11	86
Section7	12	109	17	154	18	101	23	156	12	105	12	151	17	139	11	80	6	118	12	102
Section8	14	140	19	177	20	112	34	165	21	144	10	126	27	180	38	192	5	97	16	146
Section9	11	115	14	181	31	164	17	112	19	157	16	165	18	128	25	153	8	118	18	164
Section10	7	79	35	259	33	116	36	175	8	108	18	184	25	164	35	134	10	122	16	160
Total	100	999	175	1666	233	1177	253	1412	154	1259	136	1596	208	1466	235	1287	88	1386	146	1360
**Percent**	**10.01%**		**10.50%**		**19.80%**		**17.92%**		**12.23%**		**8.52%**		**14.19%**		**18.26%**		**6.35%**		**10.74%**	

	Ngn3-2A-CreER;R26-tdT (+2w)										*Ngn3-2A-CreER;R26-tdT* (+12w)									
	Sample1		Sample2		Sample3		Sample4		Sample5		Sample1		Sample2		Sample3		Sample4		Sample5	
	tdT+Ppy+	Ppy+	tdT+Ppy+	Ppy+	tdT+Ppy+	Ppy+	tdT+Ppy+	Ppy+	tdT+Ppy+	Ppy+	tdT+Ppy+	Ppy+	tdT+Ppy+	Ppy+	tdT+Ppy+	Ppy+	tdT+Ppy+	Ppy+	tdT+Ppy+	Ppy+
Section1	24	117	13	87	21	107	20	109	11	84	19	104	12	53	14	70	8	59	7	44
Section2	18	88	17	86	12	66	25	106	17	123	12	55	29	126	23	97	13	89	12	82
Section3	33	144	8	47	32	152	28	132	7	55	23	82	35	92	18	83	10	74	10	67
Section4	22	103	14	74	25	128	31	130	9	73	15	94	18	65	20	90	12	97	11	73
Section5	16	81	11	58	29	146	16	93	10	77	11	46	19	63	26	115	9	63	14	83
Section6	11	59	9	49	18	106	14	75	13	81	17	78	34	116	21	111	13	72	8	57
Section7	24	95	7	40	17	87	21	81	15	95	21	97	18	74	29	107	6	62	15	75
Section8	28	127	19	105	14	73	25	120	12	125	19	91	23	84	24	116	11	93	6	64
Section9	19	91	18	85	21	91	32	137	8	113	30	95	28	126	13	80	14	98	15	87
Section10	28	116	14	76	19	99	26	139	19	71	26	138	30	88	35	124	9	67	7	46
Total	223	1021	130	707	208	1055	238	1122	121	897	193	880	246	887	223	993	105	774	105	678
**Percent**	**21.84%**		**18.39%**		**19.72%**		**21.21%**		**13.49%**		**21.93%**		**27.73%**		**22.46%**		**13.57%**		**15.49%**	

Appendix Table S1. Raw cell numbers counted in each section per sample, related to Figure 1.

Figure 2H

Ins2-DreER;Ngn3-CreER;R26-TLR (+2w)																				
	Sample1				Sample2				Sample3				Sample4				Sample5			
	tdT+zsG-Ins+	zsG+Ins+	tdT+zsG+Ins+	Ins+	tdT+zsG-Ins+	zsG+Ins+	tdT+zsG+Ins+	Ins+	tdT+zsG-Ins+	zsG+Ins+	tdT+zsG+Ins+	Ins+	tdT+zsG-Ins+	zsG+Ins+	tdT+zsG+Ins+	Ins+	tdT+zsG-Ins+	zsG+Ins+	tdT+zsG+Ins+	Ins+
Section1	0	832	162	834	0	692	99	695	0	506	63	506	1	948	149	955	0	509	148	509
Section2	1	791	151	792	0	853	143	857	0	959	121	959	0	811	124	817	0	983	255	983
Section3	0	556	113	557	0	414	67	415	0	829	116	829	0	1086	177	1093	0	463	120	463
Section4	0	950	178	954	0	506	81	508	0	311	44	311	0	748	115	754	0	393	108	393
Section5	0	532	145	533	1	724	122	727	0	785	105	785	0	921	144	927	0	584	163	584
Section6	0	679	132	686	0	811	135	815	0	907	126	907	1	798	125	803	0	471	129	471
Section7	1	885	169	889	0	744	18	748	0	671	82	671	0	894	141	901	1	819	217	820
Section8	0	755	197	755	0	632	103	637	0	379	56	379	0	762	119	767	0	774	206	774
Section9	0	1382	183	1390	0	762	202	766	0	906	120	906	1	697	108	698	0	412	113	412
Section10	0	749	174	751	0	1118	196	1123	0	569	79	569	0	891	142	903	0	815	251	815
Total	2	8111	1604	8141	1	7256	1166	7291	0	6822	912	6822	3	8556	1344	8618	1	6223	1710	6224
**Percent**	**0.02%**	**99.63%**	**19.70%**		**0.01%**	**99.52%**	**15.99%**		**0.00%**	**100.00%**	**13.37%**		**0.03%**	**99.28%**	**15.60%**		**0.02%**	**99.98%**	**27.47%**	

Ins2-DreER;Ngn3-CreER;R26-TLR (+12w)																								
	Sample1				Sample2				Sample3				Sample4				Sample5				Sample6			
	tdT+zsG-Ins+	zsG+Ins+	tdT+zsG+Ins+	Ins+	tdT+zsG-Ins+	zsG+Ins+	tdT+zsG+Ins+	Ins+	tdT+zsG-Ins+	zsG+Ins+	tdT+zsG+Ins+	Ins+	tdT+zsG-Ins+	zsG+Ins+	tdT+zsG+Ins+	Ins+	tdT+zsG-Ins+	zsG+Ins+	tdT+zsG+Ins+	Ins+	tdT+zsG-Ins+	zsG+Ins+	tdT+zsG+Ins+	Ins+
Section1	0	820	134	824	1	955	96	956	0	858	135	858	0	593	99	596	0	989	205	989	0	467	88	467
Section2	0	601	106	604	0	1018	132	1018	2	836	141	838	0	723	124	726	3	825	172	828	0	944	198	944
Section3	2	919	162	925	0	553	71	553	0	744	122	744	0	402	68	404	0	471	89	471	0	684	142	684
Section4	0	372	65	374	0	475	58	475	2	620	95	622	0	664	109	667	0	259	57	259	0	1367	270	1367
Section5	0	486	86	488	0	534	64	534	0	933	153	933	0	894	165	898	1	660	141	661	0	702	144	702
Section6	1	937	166	942	0	804	105	804	1	355	58	356	1	645	102	647	0	726	155	726	0	478	91	478
Section7	0	833	147	837	0	891	116	891	2	876	152	878	0	730	114	732	4	922	196	926	0	779	148	779
Section8	0	860	183	864	1	521	68	522	0	402	61	402	0	661	122	663	0	547	104	547	1	426	76	427
Section9	0	765	135	770	0	405	52	405	0	749	123	749	0	674	105	678	2	781	166	783	0	717	152	717
Section10	1	931	145	935	0	511	102	511	0	913	151	913	0	817	146	823	0	636	143	636	0	541	103	541
Total	4	7524	1329	7563	2	6667	864	6669	7	7286	1191	7293	1	6803	1154	6834	10	6816	1428	6826	1	7105	1412	7106
**Percent**	**0.05%**	**99.48%**	**17.57%**		**0.03%**	**99.97%**	**12.96%**		**0.10%**	**99.90%**	**16.33%**		**0.01%**	**99.55%**	**16.89%**		**0.15%**	**99.85%**	**20.92%**		**0.01%**	**99.99%**	**19.87%**	

Figure 2N

Ins2-DreER;Ngn3-2A-CreER;R26-TLR (+2w)																
	Sample1				Sample2				Sample3				Sample4			
	tdT+zsG-Ins+	zsG+Ins+	tdT+zsG+Ins+	Ins+	tdT+zsG-Ins+	zsG+Ins+	tdT+zsG+Ins+	Ins+	tdT+zsG-Ins+	zsG+Ins+	tdT+zsG+Ins+	Ins+	tdT+zsG-Ins+	zsG+Ins+	tdT+zsG+Ins+	Ins+
Section1	0	744	118	750	0	815	81	818	0	294	41	295	0	858	103	862
Section2	0	816	133	821	0	345	39	347	0	354	55	356	0	517	65	518
Section3	0	648	101	653	1	870	95	872	0	928	143	930	0	793	98	793
Section4	1	446	68	449	0	976	104	979	0	525	80	527	2	977	126	980
Section5	0	592	95	596	0	533	55	535	1	274	39	275	0	853	115	854
Section6	0	858	138	864	0	557	62	559	0	726	115	728	0	612	74	613
Section7	0	643	109	648	1	634	69	636	0	377	48	380	0	521	61	521
Section8	0	664	98	669	1	742	78	745	0	815	128	816	1	332	42	334
Section9	0	495	83	499	0	426	45	426	0	731	113	732	0	417	53	418
Section10	0	981	160	988	0	540	54	542	0	571	99	574	2	738	90	740
Total	1	6887	1103	6937	3	6438	682	6459	1	5595	861	5613	5	6618	827	6633
**Percent**	**0.01%**	**99.28%**	**15.90%**		**0.05%**	**99.67%**	**10.56%**		**0.02%**	**99.68%**	**15.34%**		**0.08%**	**99.77%**	**12.47%**	

Ins2-DreER;Ngn3-2A-CreER;R26-TLR (+12w)																								
	Sample1				Sample2				Sample3				Sample4				Sample5				Sample6			
	tdT+zsG-Ins+	zsG+Ins+	tdT+zsG+Ins+	Ins+	tdT+zsG-Ins+	zsG+Ins+	tdT+zsG+Ins+	Ins+	tdT+zsG-Ins+	zsG+Ins+	tdT+zsG+Ins+	Ins+	tdT+zsG-Ins+	zsG+Ins+	tdT+zsG+Ins+	Ins+	tdT+zsG-Ins+	zsG+Ins+	tdT+zsG+Ins+	Ins+	tdT+zsG-Ins+	zsG+Ins+	tdT+zsG+Ins+	Ins+
Section1	0	948	105	955	1	755	47	757	0	418	75	418	0	289	46	289	0	977	175	977	0	772	179	772
Section2	0	622	89	625	0	912	61	919	0	905	159	909	0	603	97	603	1	524	89	525	0	614	156	614
Section3	0	466	51	467	1	467	30	470	0	972	164	975	1	568	91	569	0	548	116	548	0	468	111	468
Section4	0	569	65	573	0	844	55	849	0	941	147	946	0	523	87	523	1	614	124	615	0	332	85	332
Section5	0	522	57	525	1	846	56	852	0	938	169	941	0	433	69	433	0	556	112	556	0	562	148	562
Section6	0	843	102	847	0	773	38	776	0	982	172	983	2	485	84	487	0	921	184	921	0	922	231	922
Section7	1	436	53	438	0	857	59	862	0	549	92	550	0	568	95	568	1	887	169	888	0	767	180	767
Section8	0	457	58	459	0	515	30	518	1	540	84	542	1	494	68	495	0	804	153	804	1	668	144	669
Section9	0	619	65	622	0	838	54	845	0	716	147	716	0	466	74	466	0	723	98	723	0	886	218	886
Section10	0	911	89	916	0	692	55	694	0	651	81	653	0	791	121	791	0	608	148	608	0	858	180	858
Total	1	6393	734	6427	3	7499	485	7542	1	7612	1290	7633	4	5220	832	5224	3	7162	1368	7165	1	6849	1632	6850
**Percent**	**0.02%**	**99.47%**	**11.42%**		**0.04%**	**99.43%**	**6.43%**		**0.01%**	**99.72%**	**16.90%**		**0.08%**	**99.92%**	**15.93%**		**0.04%**	**99.96%**	**19.09%**		**0.01%**	**99.99%**	**23.82%**	

Appendix Table S2. Raw cell numbers counted in each section per sample, related to Figure 2.

Figure 3H

Ins2-DreER;Ngn3-CreER;NR1 (+2w)															
	Sample1			Sample2			Sample3			Sample4			Sample5		
	zsG+Ins+	tdT+Ins+	Ins+	zsG+Ins+	tdT+Ins+	Ins+	zsG+Ins+	tdT+Ins+	Ins+	zsG+Ins+	tdT+Ins+	Ins+	zsG+Ins+	tdT+Ins+	Ins+
Section1	0	946	946	0	719	719	0	709	709	0	616	620	0	586	595
Section2	0	820	820	0	476	476	0	489	489	0	669	671	0	576	576
Section3	0	496	496	0	437	437	0	640	640	0	596	601	0	821	826
Section4	0	625	625	0	524	524	0	451	451	0	747	749	0	896	902
Section5	0	651	651	0	980	980	0	919	919	0	901	906	0	443	443
Section6	0	659	659	0	925	925	0	696	696	0	854	858	0	391	399
Section7	0	937	937	0	772	772	0	595	595	0	786	788	0	641	646
Section8	0	722	722	0	498	498	0	753	753	0	388	392	0	920	928
Section9	0	975	975	0	893	893	0	427	427	0	680	683	0	536	539
Section10	0	617	617	0	689	689	0	741	741	0	596	599	0	721	727
Total	0	7448	7448	0	6913	6913	0	6420	6420	0	6833	6867	0	6531	6581
**Percent**	**0.00%**	**100.00%**		**0.00%**	**100.00%**		**0.00%**	**100.00%**		**0.00%**	**99.50%**		**0.00%**	**99.24%**	

Ins2-DreER;Ngn3-CreER;NR1 (+12w)															
	Sample1			Sample2			Sample3			Sample4			Sample5		
	zsG+Ins+	tdT+Ins+	Ins+	zsG+Ins+	tdT+Ins+	Ins+	zsG+Ins+	tdT+Ins+	Ins+	zsG+Ins+	tdT+Ins+	Ins+	zsG+Ins+	tdT+Ins+	Ins+
Section1	0	633	640	0	518	525	0	830	835	0	781	789	0	895	895
Section2	0	514	522	0	945	957	0	564	570	0	688	694	0	398	398
Section3	0	760	772	0	935	940	0	823	825	0	468	468	0	822	822
Section4	0	677	684	0	817	826	0	477	478	0	937	947	0	894	894
Section5	0	389	394	0	738	745	0	362	363	0	695	699	0	769	769
Section6	0	424	431	0	676	682	0	429	429	0	502	506	0	530	530
Section7	0	509	515	0	701	709	0	836	838	0	738	741	0	597	597
Section8	0	460	468	0	378	382	0	682	686	0	592	594	0	725	725
Section9	0	842	855	0	457	459	0	917	925	0	305	310	0	468	468
Section10	0	489	499	0	840	848	0	981	987	0	731	736	0	598	598
Total	0	5697	5780	0	7005	7073	0	6901	6936	0	6437	6484	0	6696	6696
**Percent**	**0.00%**	**98.56%**		**0.00%**	**99.04%**		**0.00%**	**99.50%**		**0.00%**	**99.28%**		**0.00%**	**100.00%**	

Figure 3N

Ins2-DreER;Ngn3-2A-CreER;NR1 (+2w)												
	Sample1			Sample2			Sample3			Sample4		
	zsG+Ins+	tdT+Ins+	Ins+	zsG+Ins+	tdT+Ins+	Ins+	zsG+Ins+	tdT+Ins+	Ins+	zsG+Ins+	tdT+Ins+	Ins+
Section1	0	726	727	0	351	355	0	886	886	0	669	674
Section2	0	567	569	0	465	466	0	676	676	0	1017	1021
Section3	0	454	457	0	642	645	0	577	577	0	635	635
Section4	0	657	659	0	741	743	0	954	954	0	341	345
Section5	0	892	893	0	668	672	0	986	986	0	608	609
Section6	0	669	669	0	454	456	0	947	947	0	717	730
Section7	0	722	722	0	789	792	0	874	874	0	615	617
Section8	0	823	824	0	932	938	0	858	858	0	985	990
Section9	0	598	598	0	964	971	0	526	526	0	363	365
Section10	0	781	781	0	890	893	0	682	682	0	1295	1297
Total	0	6889	6899	0	6896	6931	0	7966	7966	0	7245	7283
**Percent**	**0.00%**	**99.86%**		**0.00%**	**99.50%**		**0.00%**	**100.00%**		**0.00%**	**99.48%**	

Ins2-DreER;Ngn3-2A-CreER;NR1 (+12w)												
	Sample1			Sample2			Sample3			Sample4		
	zsG+Ins+	tdT+Ins+	Ins+	zsG+Ins+	tdT+Ins+	Ins+	zsG+Ins+	tdT+Ins+	Ins+	zsG+Ins+	tdT+Ins+	Ins+
Section1	0	448	452	0	942	945	0	678	684	0	734	739
Section2	0	543	549	0	781	788	0	846	852	0	941	952
Section3	0	395	398	0	821	823	0	779	782	0	607	611
Section4	0	621	623	0	655	659	0	456	456	0	991	998
Section5	0	824	824	0	505	507	0	930	936	0	601	606
Section6	0	397	402	0	522	522	0	873	879	0	863	868
Section7	0	801	803	0	886	894	0	503	503	0	557	560
Section8	0	605	609	0	863	868	0	915	920	0	859	874
Section9	0	773	775	0	743	747	0	911	917	0	542	547
Section10	0	844	848	0	869	872	0	890	894	0	873	880
Total	0	6251	6283	0	7587	7625	0	7781	7823	0	7568	7635
**Percent**	**0.00%**	**99.49%**		**0.00%**	**99.50%**		**0.00%**	**99.46%**		**0.00%**	**99.12%**	

Appendix Table S3. Raw cell numbers counted in each section per sample, related to Figure 3.

Figure 4J

	Ins2-DreER;R26-R-tdT-DTR (PBS)								*Ins2-DreER;R26-R-tdT-DTR* (DT)							
	Sample1		Sample2		Sample3		Sample4		Sample1		Sample2		Sample3		Sample4	
	Ins+ cell number	counted islet number	Ins+ cell number	counted islet number	Ins+ cell number	counted islet number	Ins+ cell number	counted islet number	Ins+ cell number	counted islet number	Ins+ cell number	counted islet number	Ins+ cell number	counted islet number	Ins+ cell number	counted islet number
Section1	372	2	279	2	607	3	221	2	4	2	1	2	4	2	1	2
Section2	283	2	329	3	965	5	152	2	3	2	2	2	1	2	0	2
Section3	237	2	166	2	378	2	254	2	6	2	2	2	3	2	1	2
Section4	622	2	664	4	365	2	195	2	7	2	0	2	0	2	1	2
Section5	507	2	375	2	399	2	108	2	6	3	1	2	3	4	2	2
Section6	255	2	402	2	386	3	235	2	5	3	2	2	3	4	1	4
Section7	357	2	526	3	404	2	249	2	9	3	3	2	1	4	1	2
Section8	333	2	878	4	702	3	333	2	6	3	3	2	2	4	2	2
Section9	249	2	663	4	759	4	167	2	4	2	0	2	3	2	0	4
Section10	350	2	718	4	894	4	291	2	8	3	1	2	0	4	0	3
Total	3565	20	5000	30	5859	30	2205	20	58	25	15	20	20	30	9	25
**Average**	**178.25**		**166.67**		**195.3**		**110.25**		**2.32**		**0.75**		**0.67**		**0.36**	

Figure 4L

	Ins2-DreER;Ngn3-2A-CreER;NR1;R26-R-tdT-DTR (PBS)								*Ins2-DreER;Ngn3-2A-CreER;NR1;R26-R-tdT-DTR* (DT)							
	Sample1		Sample2		Sample3		Sample4		Sample1		Sample2		Sample3		Sample4	
	zsG+Ins+	Ins+	zsG+Ins+	Ins+	zsG+Ins+	Ins+	zsG+Ins+	Ins+	zsG+Ins+	Ins+	zsG+Ins+	Ins+	zsG+Ins+	Ins+	zsG+Ins+	Ins+
Section1	0	542	0	858	0	795	0	875	17	28	13	22	6	12	11	17
Section2	0	690	0	265	0	942	0	578	6	9	8	17	7	15	5	8
Section3	0	507	0	649	0	444	0	643	16	22	14	35	9	19	6	10
Section4	0	651	0	556	0	638	0	519	13	17	11	23	12	21	8	15
Section5	0	438	0	971	0	318	0	543	27	41	7	13	6	14	10	21
Section6	0	407	0	893	0	546	0	415	10	15	9	18	14	28	13	24
Section7	0	360	0	810	0	697	0	347	11	21	14	24	5	11	11	26
Section8	0	843	0	411	0	834	0	930	10	12	16	31	5	17	13	23
Section9	0	522	0	359	0	382	0	418	5	8	10	29	7	18	14	18
Section10	0	702	0	516	0	237	0	707	7	10	13	28	6	13	9	19
Total	0	5662	0	6288	0	5833	0	5975	122	183	115	240	77	168	100	181
**Average**	**0.00%**		**0.00%**		**0.00%**		**0.00%**		**66.67%**		**47.92%**		**45.83%**		**55.25%**	

Appendix Table S4. Raw cell numbers counted in each section per sample, related to Figure 4.

Figure 5E

	Ins2-DreER;Hnf1b-2A-CreER;R26-TLR (+2w)									
	Sample1		Sample2		Sample3		Sample4		Sample5	
	tdT+CK19+	CK19+	tdT+CK19+	CK19+	tdT+CK19+	CK19+	tdT+CK19+	CK19+	tdT+CK19+	CK19+
Section1	60	60	223	223	106	106	94	94	86	86
Section2	127	127	216	216	82	82	125	125	103	105
Section3	84	84	105	105	61	61	82	82	152	152
Section4	118	118	103	103	86	87	108	108	89	89
Section5	76	76	130	130	73	73	116	116	79	80
Section6	121	122	205	205	92	92	105	105	121	121
Section7	115	115	178	179	118	118	76	76	136	136
Section8	139	139	198	198	76	77	122	122	126	129
Section9	97	97	249	249	57	58	84	84	203	203
Section10	64	64	282	282	65	66	65	65	188	189
Total	1001	1002	1889	1890	816	820	977	977	1283	1290
**Average**	**99.90%**		**99.95%**		**99.51%**		**100.00%**		**99.46%**	

Figure 5J

	Ins2-DreER;Hnf1b-2A-CreER;R26-TLR (+2w)																			
	Sample1				Sample2				Sample3				Sample4				Sample5			
	tdT+zsG-Ins+	tdT+zsG+Ins+	zsG+Ins+	Ins+	tdT+zsG-Ins+	tdT+zsG+Ins+	zsG+Ins+	Ins+	tdT+zsG-Ins+	tdT+zsG+Ins+	zsG+Ins+	Ins+	tdT+zsG-Ins+	tdT+zsG+Ins+	zsG+Ins+	Ins+	tdT+zsG-Ins+	tdT+zsG+Ins+	zsG+Ins+	Ins+
Section1	0	63	724	724	0	48	592	592	0	46	560	560	0	49	726	728	0	62	645	645
Section2	0	75	887	887	0	61	793	793	0	48	587	587	0	37	572	575	0	46	440	440
Section3	0	65	756	756	0	58	727	727	0	80	945	945	0	42	635	637	0	72	764	764
Section4	0	62	702	702	0	52	657	657	0	43	567	567	0	35	425	428	0	52	513	513
Section5	0	69	743	745	0	80	989	989	0	37	480	480	0	62	891	892	0	38	341	341
Section6	0	65	852	852	0	56	688	688	0	69	804	804	0	74	908	909	0	47	584	584
Section7	0	32	455	457	0	45	591	591	0	47	568	568	0	42	642	642	0	81	880	880
Section8	0	71	792	792	0	74	912	912	0	28	309	309	0	30	367	369	0	58	596	596
Section9	0	68	713	713	0	72	929	929	0	51	496	496	0	22	263	264	0	27	359	359
Section10	0	70	831	831	0	42	519	519	0	79	924	924	0	56	690	691	0	62	757	757
Total	0	640	7455	7459	0	588	7397	7397	0	528	6240	6240	0	449	6119	6135	0	545	5879	5879
**Percent**	**0.00%**	**8.58%**	**99.95%**		**0.00%**	**7.95%**	**100.00%**		**0.00%**	**8.46%**	**100.00%**		**0.00%**	**7.32%**	**99.74%**		**0.00%**	**9.27%**	**100.00%**	

	Ins2-DreER;Hnf1b-2A-CreER;R26-TLR (+12w)																			
	Sample1				Sample2				Sample3				Sample4				Sample5			
	tdT+zsG-Ins+	tdT+zsG+Ins+	zsG+Ins+	Ins+	tdT+zsG-Ins+	tdT+zsG+Ins+	zsG+Ins+	Ins+	tdT+zsG-Ins+	tdT+zsG+Ins+	zsG+Ins+	Ins+	tdT+zsG-Ins+	tdT+zsG+Ins+	zsG+Ins+	Ins+	tdT+zsG-Ins+	tdT+zsG+Ins+	zsG+Ins+	Ins+
Section1	0	41	524	524	0	62	522	522	0	66	563	563	0	54	914	914	0	74	922	922
Section2	0	47	704	704	0	59	479	479	0	85	927	927	0	76	812	812	0	55	655	655
Section3	0	39	483	483	0	61	491	491	0	50	496	498	0	64	548	548	0	44	423	423
Section4	0	25	353	353	0	72	645	645	0	55	556	559	0	82	870	870	0	69	731	731
Section5	0	37	461	461	0	62	538	538	0	63	651	651	0	67	643	643	0	40	488	488
Section6	0	58	722	722	0	74	812	812	0	65	802	803	0	89	989	989	0	47	506	506
Section7	0	76	955	955	0	57	699	699	0	68	843	843	0	55	427	427	0	59	735	735
Section8	0	59	714	714	0	78	725	725	0	46	567	569	0	53	532	532	0	74	898	898
Section9	0	52	623	623	0	61	388	388	0	59	856	856	0	39	361	361	0	43	530	530
Section10	0	54	669	669	0	55	434	434	0	76	939	941	0	52	855	855	0	48	589	589
Total	0	488	6208	6208	0	641	5733	5733	0	633	7200	7210	0	631	6951	6951	0	553	6477	6477
**Percent**	**0.00%**	**7.86%**	**100.00%**		**0.00%**	**11.18%**	**100.00%**		**0.00%**	**8.78%**	**99.86%**		**0.00%**	**9.08%**	**100.00%**		**0.00%**	**8.54%**	**100.00%**	

Appendix Table S5. Raw cell numbers counted in each section per sample, related to Figure 5.

Figure 6E

Ins2-DreER;Hnf1b-2A-CreER;NR1 (+2w)										
	Sample1		Sample2		Sample3		Sample4		Sample5	
	zsG+CK19+	CK19+	zsG+CK19+	CK19+	zsG+CK19+	CK19+	zsG+CK19+	CK19+	zsG+CK19+	CK19+
Section1	104	104	87	87	86	86	71	71	130	131
Section2	71	71	101	104	94	94	136	137	211	213
Section3	69	69	84	84	157	157	106	106	116	118
Section4	81	81	93	95	84	84	131	132	103	104
Section5	73	73	106	107	81	81	89	90	136	137
Section6	93	93	105	105	130	130	154	154	234	235
Section7	116	117	73	73	136	136	135	135	186	188
Section8	75	75	109	109	124	124	157	157	102	102
Section9	67	67	87	88	108	108	122	123	166	167
Section1	70	70	64	64	201	201	83	83	119	119
Total	819	820	909	916	1201	1201	1184	1188	1503	1514
**Average**	**99.88%**		**99.24%**		**100.00%**		**99.66%**		**99.27%**	

Figure 6J

Ins2-DreER;Hnf1b-2A-CreER;NR1 (+2w)															
	Sample1			Sample2			Sample3			Sample4			Sample5		
	zsG+Ins+	tdT+Ins+	Ins+	zsG+Ins+	tdT+Ins+	Ins+	zsG+Ins+	tdT+Ins+	Ins+	zsG+Ins+	tdT+Ins+	Ins+	zsG+Ins+	tdT+Ins+	Ins+
Section1	0	384	384	0	664	669	0	297	301	0	621	621	0	495	497
Section2	0	259	259	0	571	573	0	402	405	0	668	668	0	588	589
Section3	0	453	453	0	372	372	0	731	733	0	715	715	0	517	518
Section4	0	281	281	0	604	605	0	553	556	0	647	647	0	783	783
Section5	0	579	579	0	1105	1106	0	580	581	0	746	746	0	850	852
Section6	0	492	492	0	667	668	0	457	460	0	648	648	0	505	508
Section7	0	340	340	0	704	706	0	622	625	0	801	801	0	842	845
Section8	0	595	595	0	1039	1041	0	432	434	0	688	688	0	456	460
Section9	0	690	690	0	790	791	0	843	847	0	425	425	0	778	779
Section1	0	448	448	0	961	964	0	635	638	0	767	767	0	729	729
Total	0	4521	4521	0	7477	7495	0	5552	5580	0	6726	6726	0	6543	6560
**Percent**	**0.00%**	**100.00%**		**0.00%**	**99.76%**		**0.00%**	**99.50%**		**0.00%**	**100.00%**		**0.00%**	**99.74%**	

Ins2-DreER;Hnf1b-2A-CreER;NR1 (+12w)															
	Sample1			Sample2			Sample3			Sample4			Sample5		
	zsG+Ins+	tdT+Ins+	Ins+	zsG+Ins+	tdT+Ins+	Ins+	zsG+Ins+	tdT+Ins+	Ins+	zsG+Ins+	tdT+Ins+	Ins+	zsG+Ins+	tdT+Ins+	Ins+
Section1	0	547	551	0	494	494	0	313	318	0	766	766	0	753	763
Section2	0	371	372	0	758	758	0	671	683	0	571	571	0	640	644
Section3	0	690	692	0	703	703	0	277	285	0	476	476	0	861	871
Section4	0	411	413	0	720	720	0	205	208	0	327	327	0	458	471
Section5	0	445	450	0	396	396	0	526	529	0	760	760	0	515	518
Section6	0	703	705	0	451	451	0	553	556	0	812	812	0	617	622
Section7	0	504	506	0	612	612	0	441	444	0	727	727	0	573	577
Section8	0	331	332	0	566	566	0	485	497	0	496	496	0	582	596
Section9	0	575	578	0	468	468	0	507	507	0	394	394	0	841	852
Section1	0	384	388	0	527	527	0	684	698	0	882	882	0	962	973
Total	0	4961	4987	0	5695	5695	0	4662	4725	0	6211	6211	0	6802	6887
**Percent**	**0.00%**	**99.48%**		**0.00%**	**100.00%**		**0.00%**	**98.67%**		**0.00%**	**100.00%**		**0.00%**	**98.77%**	

Appendix Table S6. Raw cell numbers counted in each section per sample, related to Figure 6.

Figure EV1E

Ngn3-CreER;R26-tdT (Oil)										
	Sample1		Sample2		Sample3		Sample4		Sample5	
	tdT+Ins+	Ins+	tdT+Ins+	Ins+	tdT+Ins+	Ins+	tdT+Ins+	Ins+	tdT+Ins+	Ins+
Section1	0	676	0	514	0	590	0	612	0	865
Section2	0	939	0	942	0	565	1	733	0	619
Section3	0	469	0	695	2	479	0	786	0	521
Section4	0	955	1	902	0	576	1	623	0	327
Section5	0	673	0	594	0	625	1	364	1	576
Section6	0	862	0	989	1	654	0	648	1	615
Section7	1	612	0	699	1	446	0	656	0	632
Section8	0	488	0	869	0	778	0	427	0	906
Section9	0	967	0	516	0	335	0	821	1	738
Section1	0	807	0	469	0	628	0	383	0	814
Total	1	7448	1	7189	4	5676	3	6053	3	6613
**Percent**	**0.01%**		**0.01%**		**0.07%**		**0.05%**		**0.05%**	

Figure EV1J

Ngn3-2A-CreER;R26-tdT (Oil)										
	Sample1		Sample2		Sample3		Sample4		Sample5	
	tdT+Ins+	Ins+	tdT+Ins+	Ins+	tdT+Ins+	Ins+	tdT+Ins+	Ins+	tdT+Ins+	Ins+
Section1	0	757	0	584	0	423	0	809	0	618
Section2	0	478	1	404	0	591	0	386	0	513
Section3	0	466	0	594	0	689	0	318	0	426
Section4	0	663	0	498	0	303	0	629	0	504
Section5	0	364	0	774	0	589	0	765	0	430
Section6	0	739	1	505	0	708	1	608	0	572
Section7	0	517	1	568	0	719	0	591	0	706
Section8	0	715	0	639	0	684	0	704	0	501
Section9	0	469	0	724	1	342	0	634	0	616
Section1	0	558	0	877	0	661	0	505	0	508
Total	0	5726	3	6167	1	5709	1	5949	0	5394
**Percent**	**0.00%**		**0.05%**		**0.02%**		**0.02%**		**0.00%**	

Appendix Table S7. Raw cell numbers counted in each section per sample, related to Figure EV1.

Figure EV2F

Ins2-DreER;R26-TLR (Tam)																				
	Sample1				Sample2				Sample3				Sample4				Sample5			
	tdT+zsG- Ins+	zsG+tdT- Ins+	tdT+zsG+ Ins+	Ins+	tdT+zsG- Ins+	zsG+tdT- Ins+	tdT+zsG+ Ins+	Ins+	tdT+zsG- Ins+	zsG+tdT- Ins+	tdT+zsG+ Ins+	Ins+	tdT+zsG- Ins+	zsG+tdT- Ins+	tdT+zsG+ Ins+	Ins+	tdT+zsG- Ins+	zsG+tdT- Ins+	tdT+zsG+ Ins+	Ins+
Section1	0	808	0	808	0	771	0	771	0	434	0	434	0	263	0	263	0	601	0	601
Section2	0	561	0	563	0	858	0	858	0	793	0	793	0	729	0	729	0	705	0	705
Section3	0	636	0	636	0	648	0	648	0	825	0	825	0	359	0	360	0	355	0	355
Section4	0	869	0	871	0	623	0	623	0	656	0	656	0	505	0	506	0	459	0	459
Section5	0	409	0	409	0	819	0	820	0	221	0	224	0	421	0	421	0	574	0	574
Section6	0	468	0	468	0	408	0	408	0	675	0	675	0	820	0	822	0	632	0	632
Section7	0	672	0	672	0	459	0	459	0	408	0	408	0	732	0	732	0	527	0	527
Section8	0	916	1	921	0	541	0	541	0	736	0	736	0	252	0	254	0	806	0	806
Section9	0	712	0	713	0	302	0	302	0	759	0	759	0	784	0	784	0	312	0	312
Section1	0	654	0	656	0	714	0	716	0	299	0	299	0	487	0	487	0	226	0	226
Total	0	6705	1	6717	0	6143	0	6146	0	5806	0	5809	0	5352	0	5358	0	5197	0	5197
**Percent**	**0.00%**	**99.82%**	**0.01%**		**0.00%**	**99.95%**	**0.00%**		**0.00%**	**99.95%**	**0.00%**		**0.00%**	**99.89%**	**0.00%**		**0.00%**	**100.00%**	**0.00%**	

Figure EV2J

Ins2-DreER;Ngn3-CreER;R26-TLR (Oil)																				
	Sample1				Sample2				Sample3				Sample4				Sample5			
	zsG+tdT- Ins+	tdT+zsG- Ins+	tdT+zsG+ Ins+	Ins+	zsG+tdT- Ins+	tdT+zsG- Ins+	tdT+zsG+ Ins+	Ins+	zsG+tdT- Ins+	tdT+zsG- Ins+	tdT+zsG+ Ins+	Ins+	zsG+tdT- Ins+	tdT+zsG- Ins+	tdT+zsG+ Ins+	Ins+	zsG+tdT- Ins+	tdT+zsG- Ins+	tdT+zsG+ Ins+	Ins+
Section1	0	0	0	485	0	1	0	756	0	0	0	743	0	0	0	427	0	0	0	477
Section2	0	0	0	796	0	0	0	555	1	0	0	584	1	0	0	234	0	0	0	364
Section3	0	0	0	645	0	0	0	962	0	0	0	839	0	0	0	750	0	0	0	428
Section4	0	0	0	563	0	0	0	515	0	1	0	462	0	0	0	880	0	0	0	373
Section5	0	0	0	749	0	0	0	952	0	0	0	844	0	0	0	455	0	0	0	546
Section6	0	0	0	658	0	0	0	890	0	0	0	401	0	0	0	235	0	0	0	737
Section7	1	0	0	930	0	0	0	792	0	0	0	726	0	0	0	478	0	0	0	414
Section8	0	0	0	798	0	0	0	635	0	0	0	875	0	1	0	346	0	0	0	547
Section9	0	1	0	802	0	0	0	669	0	0	0	549	0	0	0	490	0	0	0	221
Section1	0	0	0	412	0	0	0	419	0	0	0	701	0	0	0	323	0	0	0	339
Total	1	1	0	6838	0	1	0	7145	1	1	0	6724	1	1	0	4618	0	0	0	4446
**Percent**	**0.01%**	**0.01%**	**0.00%**		**0.00%**	**0.01%**	**0.00%**		**0.01%**	**0.01%**	**0.00%**		**0.02%**	**0.02%**	**0.00%**		**0.00%**	**0.00%**	**0.00%**	

Figure EV2N

Ins2-DreER;Ngn3-2A-CreER;R26-TLR (Oil)																				
	Sample1				Sample2				Sample3				Sample4				Sample5			
	tdT+zsG- Ins+	zsG+tdT- Ins+	tdT+zsG+ Ins+	Ins+	tdT+zsG- Ins+	zsG+tdT- Ins+	tdT+zsG+ Ins+	Ins+	tdT+zsG- Ins+	zsG+tdT- Ins+	tdT+zsG+ Ins+	Ins+	tdT+zsG- Ins+	zsG+tdT- Ins+	tdT+zsG+ Ins+	Ins+	tdT+zsG- Ins+	zsG+tdT- Ins+	tdT+zsG+ Ins+	Ins+
Section1	0	0	0	866	0	0	0	359	0	0	0	716	0	0	0	507	0	0	0	546
Section2	0	0	0	516	0	0	0	407	0	0	0	438	0	0	0	425	0	0	0	690
Section3	0	1	0	702	0	0	0	638	0	0	0	514	0	0	0	834	0	0	0	649
Section4	0	0	0	405	0	0	0	542	0	0	0	395	0	1	0	782	0	0	0	983
Section5	0	0	0	578	0	0	0	843	0	0	0	543	0	0	0	697	0	0	0	643
Section6	0	1	0	601	0	0	0	648	0	0	0	934	0	0	0	599	0	0	0	475
Section7	0	0	0	526	0	0	0	305	0	1	0	562	0	0	0	707	0	0	0	919
Section8	0	0	0	483	0	0	0	318	0	0	0	646	0	0	0	893	0	0	0	764
Section9	0	0	0	237	0	0	0	411	0	0	0	320	0	0	0	508	0	1	0	604
Section1	0	0	0	746	0	0	0	830	0	0	0	561	0	0	0	940	0	0	0	793
Total	0	2	0	5660	0	0	0	5301	0	1	0	5629	0	1	0	6892	0	1	0	7066
**Percent**	**0.00%**	**0.04%**	**0.00%**		**0.00%**	**0.00%**	**0.00%**		**0.00%**	**0.02%**	**0.00%**		**0.00%**	**0.01%**	**0.00%**		**0.00%**	**0.01%**	**0.00%**	

Appendix Table S8. Raw cell numbers counted in each section per sample, related to Figure EV2.

Figure EV3F

Ins2-DreER;NR1 (Tam)															
	Sample1			Sample2			Sample3			Sample4			Sample5		
	zsG+Ins+	tdT+Ins+	Ins+	zsG+Ins+	tdT+Ins+	Ins+	zsG+Ins+	tdT+Ins+	Ins+	zsG+Ins+	tdT+Ins+	Ins+	zsG+Ins+	tdT+Ins+	Ins+
Section1	0	286	286	0	410	410	0	559	559	0	347	347	0	324	326
Section2	0	683	683	0	387	387	0	358	358	0	697	697	0	512	513
Section3	0	403	403	0	539	540	0	394	394	0	432	432	0	573	579
Section4	0	264	264	0	696	698	0	587	587	0	408	409	0	245	245
Section5	0	505	505	0	309	310	0	407	407	0	440	440	0	450	453
Section6	0	535	535	0	672	672	0	624	624	0	469	469	0	364	367
Section7	0	742	742	0	510	510	0	590	590	0	636	636	0	223	225
Section8	0	350	350	0	597	599	0	286	286	0	421	422	0	566	570
Section9	0	579	579	0	498	498	0	401	401	0	387	388	0	643	644
Section1	0	648	648	0	552	552	0	461	461	0	758	758	0	572	572
Total	0	4995	4995	0	5170	5176	0	4667	4667	0	4995	4998	0	4472	4494
**Percent**	**0.00%**	**100.00%**		**0.00%**	**99.88%**		**0.00%**	**100.00%**		**0.00%**	**99.94%**		**0.00%**	**99.51%**	

Figure EV3J

Ins2-DreER;Ngn3-CreER;NR1 (Oil)															
	Sample1			Sample2			Sample3			Sample4			Sample5		
	zsG+Ins+	tdT+Ins+	Ins+	zsG+Ins+	tdT+Ins+	Ins+	zsG+Ins+	tdT+Ins+	Ins+	zsG+Ins+	tdT+Ins+	Ins+	zsG+Ins+	tdT+Ins+	Ins+
Section1	0	0	731	0	0	730	0	0	361	0	0	702	0	0	609
Section2	0	0	852	0	0	423	0	0	569	0	0	604	0	0	568
Section3	0	0	756	0	0	735	0	0	427	0	0	592	0	0	655
Section4	1	0	831	0	0	785	0	0	989	0	0	728	0	0	591
Section5	0	0	498	0	0	691	0	0	394	1	0	922	0	0	745
Section6	0	0	659	0	0	453	0	0	480	0	0	415	0	0	881
Section7	0	0	713	0	0	560	0	0	517	0	0	964	0	0	596
Section8	0	0	895	0	0	412	0	0	757	0	0	819	0	0	669
Section9	0	0	451	0	0	246	0	0	343	0	0	563	1	0	870
Section1	0	0	590	0	0	394	0	0	487	0	0	479	0	0	765
Total	1	0	6976	0	0	5429	0	0	5324	1	0	6788	1	0	6949
**Percent**	**0.01%**	**0.00%**		**0.00%**	**0.00%**		**0.00%**	**0.00%**		**0.01%**	**0.00%**		**0.01%**	**0.00%**	

Figure EV3N

Ins2-DreER;Ngn3-2A-CreER;NR1 (Oil)															
	Sample1			Sample2			Sample3			Sample4			Sample5		
	zsG+Ins+	tdT+Ins+	Ins+	zsG+Ins+	tdT+Ins+	Ins+	zsG+Ins+	tdT+Ins+	Ins+	zsG+Ins+	tdT+Ins+	Ins+	zsG+Ins+	tdT+Ins+	Ins+
Section1	0	0	865	0	0	576	0	0	298	0	0	705	1	0	673
Section2	0	0	436	0	0	656	0	0	327	0	0	865	0	0	632
Section3	1	0	625	0	0	612	0	0	562	1	0	967	0	0	556
Section4	0	0	682	0	0	468	0	0	619	0	0	514	0	0	692
Section5	0	0	423	0	0	969	0	0	858	0	0	594	0	0	362
Section6	0	0	678	1	0	882	0	0	628	0	0	846	0	0	889
Section7	0	0	806	0	0	638	0	0	771	0	0	778	0	0	648
Section8	0	0	896	0	0	738	0	0	409	0	0	576	0	0	699
Section9	0	0	822	0	0	862	0	0	839	0	0	869	0	0	929
Section1	0	0	513	0	0	883	0	0	375	0	0	673	0	0	722
Total	1	0	6746	1	0	7284	0	0	5686	1	0	7387	1	0	6802
**Percent**	**0.01%**	**0.00%**		**0.01%**	**0.00%**		**0.00%**	**0.00%**		**0.01%**	**0.00%**		**0.01%**	**0.00%**	

Appendix Table S9. Raw cell numbers counted in each section per sample, related to Figure EV3.

Figure EV4E

	Ins2-DreER;Ngn3-2A-CreER;NR1;R26-R-tdT-DTR (PBS)				*Ins2-DreER;Ngn3-2A-CreER;NR1;R26-R-tdT-DTR* (DT)			
	Sample1	Sample2	Sample3	Sample4	Sample1	Sample2	Sample3	Sample4
Section1	5	6	7	1	1	3	6	4
Section2	3	4	5	4	2	6	7	3
Section3	2	1	3	2	4	3	5	2
Section4	5	2	8	5	0	4	3	5
Section5	4	4	7	4	1	7	5	3
Section6	2	3	2	0	3	6	2	1
Section7	5	6	5	1	5	4	6	1
Section8	2	1	6	2	0	3	1	0
Section9	4	5	3	0	1	5	3	2
Section10	2	4	6	3	0	4	5	2
Total	34	36	52	22	17	45	43	23
**Average**	**3.4**	**3.6**	**5.2**	**2.2**	**1.7**	**4.5**	**4.3**	**2.3**

Figure EV4J

	Ins2-DreER;Ngn3-CreER;NR1 (Head)									
	Sample1		Sample2		Sample3		Sample4		Sample5	
	zsG+Ins+	Ins+	zsG+Ins+	Ins+	zsG+Ins+	Ins+	zsG+Ins+	Ins+	zsG+Ins+	Ins+
Section1	0	650	0	526	0	660	0	809	0	770
Section2	0	466	0	708	0	516	0	569	0	537
Section3	0	638	0	458	0	461	0	480	0	675
Section4	0	819	0	632	0	992	0	872	0	526
Section5	0	414	0	572	0	509	0	705	0	692
Section6	0	523	0	505	0	838	0	844	0	839
Section7	0	588	0	397	0	542	0	922	0	621
Section8	0	876	0	441	0	697	0	535	0	568
Section9	0	655	0	459	0	506	0	891	0	662
Section10	0	776	0	520	0	846	0	634	0	537
Total	0	6405	0	5218	0	6567	0	7261	0	6427
**Average**	**0.00%**		**0.00%**		**0.00%**		**0.00%**		**0.00%**	

	Ins2-DreER;Ngn3-CreER;NR1 (Tail)									
	Sample1		Sample2		Sample3		Sample4		Sample5	
	zsG+Ins+	Ins+	zsG+Ins+	Ins+	zsG+Ins+	Ins+	zsG+Ins+	Ins+	zsG+Ins+	Ins+
Section1	0	532	0	589	0	626	0	807	0	415
Section2	0	606	0	802	0	574	0	874	0	784
Section3	0	674	0	770	0	581	0	396	0	494
Section4	0	583	0	753	0	559	0	636	0	645
Section5	0	498	0	536	0	618	0	703	0	842
Section6	0	432	0	638	0	791	0	620	0	517
Section7	0	803	0	583	0	570	0	405	0	878
Section8	0	497	1	593	0	554	0	479	0	830
Section9	0	745	0	717	0	632	0	519	0	701
Section10	0	843	0	750	0	625	0	599	0	319
Total	0	6213	1	6731	0	6130	0	6038	0	6425
**Average**	**0.00%**		**0.01%**		**0.00%**		**0.00%**		**0.00%**	

Figure EV4O

	Ins2-DreER;Ngn3-2A-CreER;NR1 (Head)									
	Sample1		Sample2		Sample3		Sample4		Sample5	
	zsG+Ins+	Ins+	zsG+Ins+	Ins+	zsG+Ins+	Ins+	zsG+Ins+	Ins+	zsG+Ins+	Ins+
Section1	0	826	0	757	0	739	0	746	0	940
Section2	0	732	0	881	0	957	0	662	0	544
Section3	0	715	0	607	0	872	0	709	0	903
Section4	0	680	0	862	0	915	0	959	0	638
Section5	0	703	0	486	0	637	0	568	0	611
Section6	0	867	0	465	0	491	0	601	0	770
Section7	0	506	0	558	0	942	0	426	0	562
Section8	0	641	0	812	0	563	0	875	0	597
Section9	0	388	0	430	0	758	0	483	1	565
Section10	0	694	0	713	0	693	0	730	0	606
Total	0	6752	0	6571	0	7567	0	6759	1	6736
**Average**	**0.00%**		**0.00%**		**0.00%**		**0.00%**		**0.01%**	

	Ins2-DreER;Ngn3-2A-CreER;NR1 (Tail)									
	Sample1		Sample2		Sample3		Sample4		Sample5	
	zsG+Ins+	Ins+	zsG+Ins+	Ins+	zsG+Ins+	Ins+	zsG+Ins+	Ins+	zsG+Ins+	Ins+
Section1	0	439	0	607	0	514	0	850	0	783
Section2	0	857	0	699	0	597	0	643	1	501
Section3	0	365	0	628	0	918	0	816	0	588
Section4	0	794	0	737	0	829	0	578	0	879
Section5	0	388	0	635	0	704	0	729	0	673
Section6	0	504	0	667	0	682	0	563	0	654
Section7	0	456	0	923	1	780	0	520	0	417
Section8	0	364	0	531	0	807	0	934	0	660
Section9	0	647	0	611	0	475	0	616	0	529
Section10	0	461	0	753	0	591	0	769	0	553
Total	0	5275	0	6791	1	6897	0	7018	1	6237
**Average**	**0.00%**		**0.00%**		**0.01%**		**0.00%**		**0.02%**	

Appendix Table S10. Raw cell numbers counted in each section per sample, related to Figure EV4.

Figure EV5D

Ins2-DreER;Hnf1b-2A-CreER;R26-TLR (Oil)										
	Sample1		Sample2		Sample3		Sample4		Sample5	
	tdT+CK19+	CK19+	tdT+CK19+	CK19+	tdT+CK19+	CK19+	tdT+CK19+	CK19+	tdT+CK19+	CK19+
Section1	0	284	0	327	0	138	0	237	0	112
Section2	0	102	0	288	0	214	0	196	0	135
Section3	0	193	0	171	0	169	0	274	0	212
Section4	0	286	0	193	0	164	0	198	0	254
Section5	0	148	0	98	0	117	0	282	0	139
Section6	0	151	0	136	0	105	0	216	0	218
Section7	0	233	0	122	0	118	0	173	0	93
Section8	0	249	0	153	0	154	0	141	0	126
Section9	0	195	0	204	0	167	0	159	0	205
Section1	0	117	0	152	0	246	0	135	0	189
Total	0	1958	0	1844	0	1592	0	2011	0	1683
**Percent**	**0.00%**		**0.00%**		**0.00%**		**0.00%**		**0.00%**	

Figure EV5F

Ins2-DreER;Hnf1b-2A-CreER;R26-TLR (Oil)																				
	Sample1				Sample2				Sample3				Sample4				Sample5			
	tdT+zsG-Ins+	zsG+tdT-Ins+	tdT+zsG+Ins+	Ins+	tdT+zsG-Ins+	zsG+tdT-Ins+	tdT+zsG+Ins+	Ins+	tdT+zsG-Ins+	zsG+tdT-Ins+	tdT+zsG+Ins+	Ins+	tdT+zsG-Ins+	zsG+tdT-Ins+	tdT+zsG+Ins+	Ins+	tdT+zsG-Ins+	zsG+tdT-Ins+	tdT+zsG+Ins+	Ins+
Section1	0	0	0	502	0	0	0	944	0	0	0	481	0	0	0	816	0	0	0	628
Section2	0	0	0	881	0	0	0	679	0	0	0	644	0	0	0	949	0	0	0	822
Section3	0	0	0	974	0	0	0	821	0	0	0	825	0	0	0	470	0	0	0	745
Section4	0	0	0	593	0	0	0	480	0	0	0	592	0	0	0	1047	0	0	0	933
Section5	0	0	0	582	0	0	0	306	0	0	0	505	0	0	0	862	0	0	0	646
Section6	0	0	0	899	0	0	0	803	0	0	0	701	0	1	0	707	0	0	0	620
Section7	0	0	0	317	0	0	0	446	0	0	0	489	0	0	0	651	0	0	0	847
Section8	0	0	0	328	0	0	0	940	0	0	0	1003	0	0	0	842	0	0	0	338
Section9	0	0	0	535	0	0	0	812	0	1	0	852	0	0	0	622	0	0	0	564
Section1	0	0	0	841	0	0	0	505	0	0	0	928	0	0	0	996	0	0	0	665
Total	0	0	0	6452	0	0	0	6736	0	1	0	7020	0	1	0	7962	0	0	0	6808
**Percent**	**0.00%**	**0.00%**	**0.00%**		**0.00%**	**0.00%**	**0.00%**		**0.00%**	**0.01%**	**0.00%**		**0.00%**	**0.01%**	**0.00%**		**0.00%**	**0.00%**	**0.00%**	

Figure EV5J

Ins2-DreER;Hnf1b-2A-CreER;NR1 (Oil)										
	Sample1		Sample2		Sample3		Sample4		Sample5	
	zsG+CK19+	CK19+	zsG+CK19+	CK19+	zsG+CK19+	CK19+	zsG+CK19+	CK19+	zsG+CK19+	CK19+
Section1	2	162	0	159	0	78	0	121	0	89
Section2	0	104	0	140	0	82	0	132	0	147
Section3	1	126	0	112	0	74	0	101	0	142
Section4	2	154	2	101	0	85	0	128	0	82
Section5	4	141	3	164	0	103	0	89	0	93
Section6	4	157	3	98	0	161	0	118	0	85
Section7	3	120	0	103	0	86	0	129	0	162
Section8	1	95	2	134	0	94	0	95	0	95
Section9	0	93	0	159	0	67	0	131	0	70
Section1	0	86	0	117	0	88	0	96	0	92
Total	17	1238	10	1287	0	918	0	1140	0	1057
**Percent**	**1.37%**		**0.78%**		**0.00%**		**0.00%**		**0.00%**	

Figure EV5L

Ins2-DreER;Hnf1b-2A-CreER;NR1 (Oil)															
	Sample1			Sample2			Sample3			Sample4			Sample5		
	zsG+Ins+	tdT+Ins+	Ins+	zsG+Ins+	tdT+Ins+	Ins+	zsG+Ins+	tdT+Ins+	Ins+	zsG+Ins+	tdT+Ins+	Ins+	zsG+Ins+	tdT+Ins+	Ins+
Section1	0	0	823	0	0	712	0	0	909	0	0	726	0	0	346
Section2	0	0	564	0	0	631	0	0	920	0	0	692	0	0	561
Section3	0	0	648	0	0	378	0	0	789	0	0	587	0	0	454
Section4	0	0	556	0	0	817	0	0	856	0	0	783	0	0	339
Section5	0	0	488	0	0	236	0	0	560	0	0	326	0	0	724
Section6	0	0	551	0	0	882	0	0	536	0	0	360	0	0	520
Section7	0	0	535	0	0	540	0	0	412	0	0	962	0	0	329
Section8	0	0	421	0	0	953	0	0	305	0	0	333	0	0	917
Section9	0	0	552	0	0	479	0	0	727	0	0	841	0	0	665
Section1	0	0	354	0	0	575	0	0	819	0	0	359	0	0	861
Total	0	0	5492	0	0	6203	0	0	6833	0	0	5969	0	0	5716
**Percent**	**0.00%**	**0.00%**		**0.00%**	**0.00%**		**0.00%**	**0.00%**		**0.00%**	**0.00%**		**0.00%**	**0.00%**	

Appendix Table S11. Raw cell numbers counted in each section per sample, related to Figure EV5.
