# Supplementary figures and images for: Ductal or Ngn3+ cells do not contribute to adult pancreatic islet beta-cell neogenesis in homeostasis (part 1 of 5)
Source: EMBO J. 2025 Apr 9;44(10):2856–81. doi: 10.1038/s44318-025-00434-z (PMC12084597; doi:10.1038/s44318-025-00434-z)

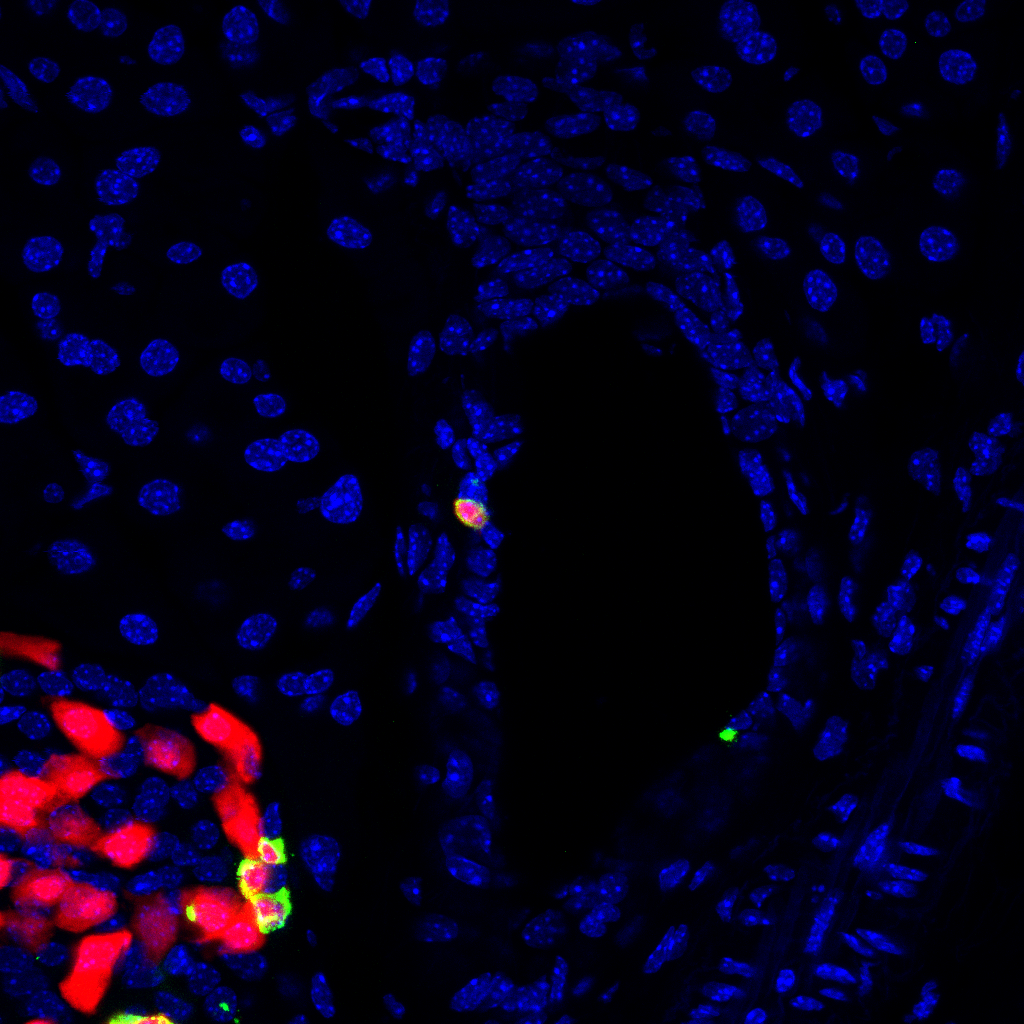

Supplement: Supplementary file 3 — Source data Fig. 1 [file 44318_2025_434_MOESM3_ESM.zip › Figure 1/1L/1L_2w_Sst.tif]

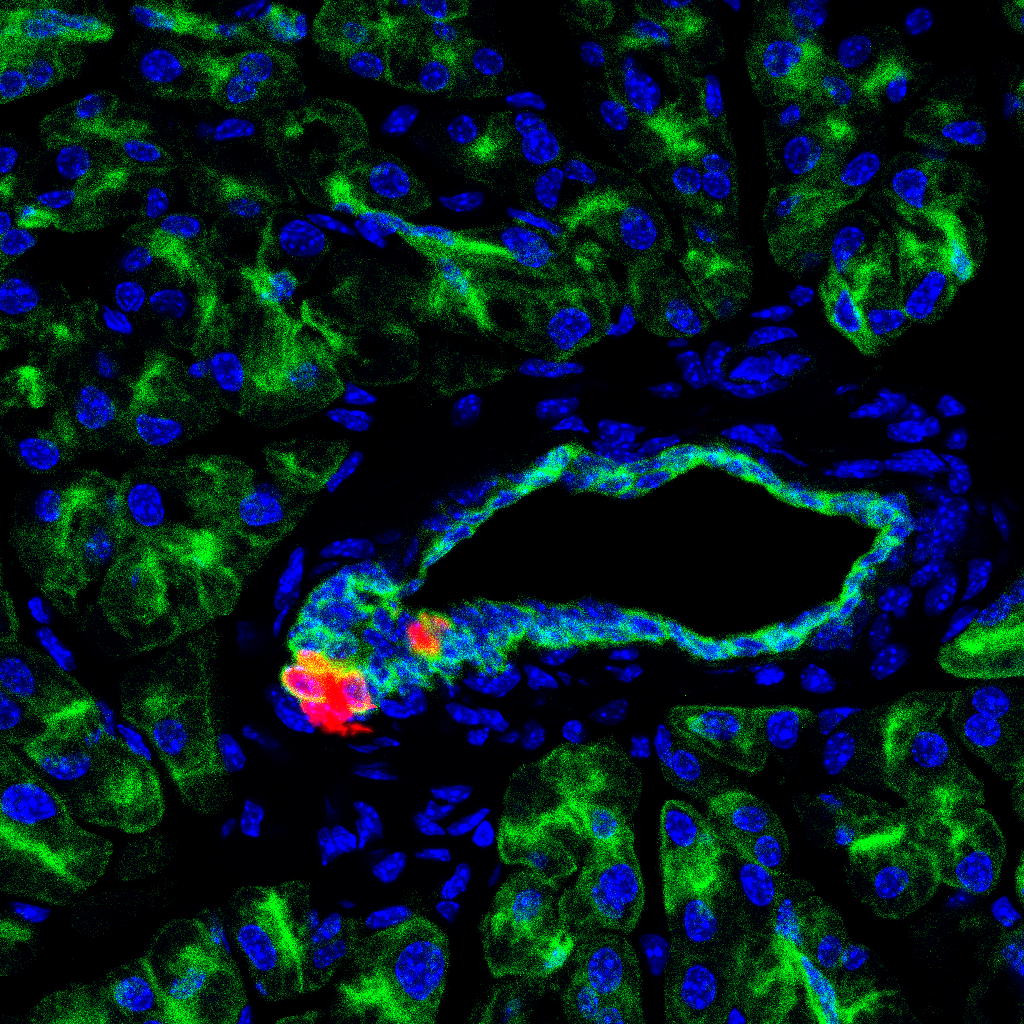

Supplement: Supplementary file 3 — Source data Fig. 1 [file 44318_2025_434_MOESM3_ESM.zip › Figure 1/1L/1L_2w_CK19.tif]

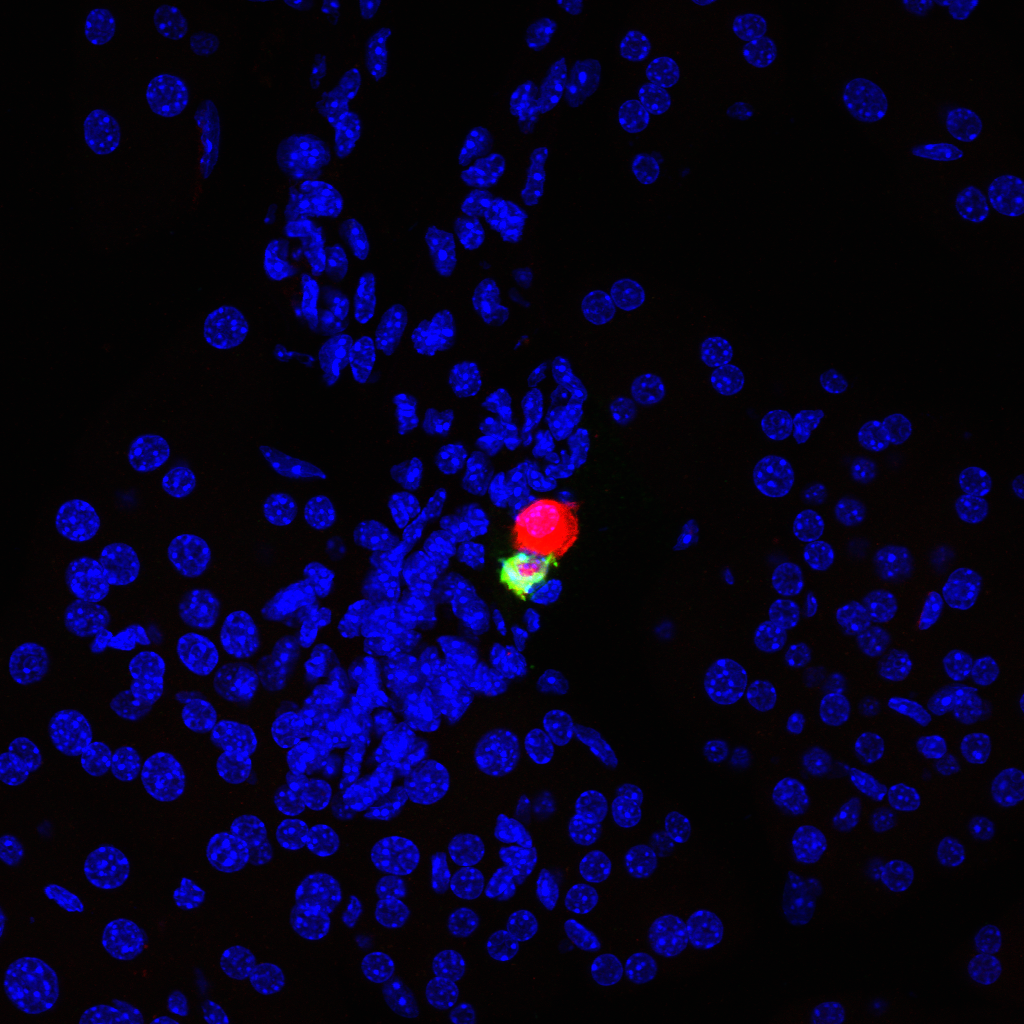

Supplement: Supplementary file 3 — Source data Fig. 1 [file 44318_2025_434_MOESM3_ESM.zip › Figure 1/1L/1L_12w_Sst.tif]

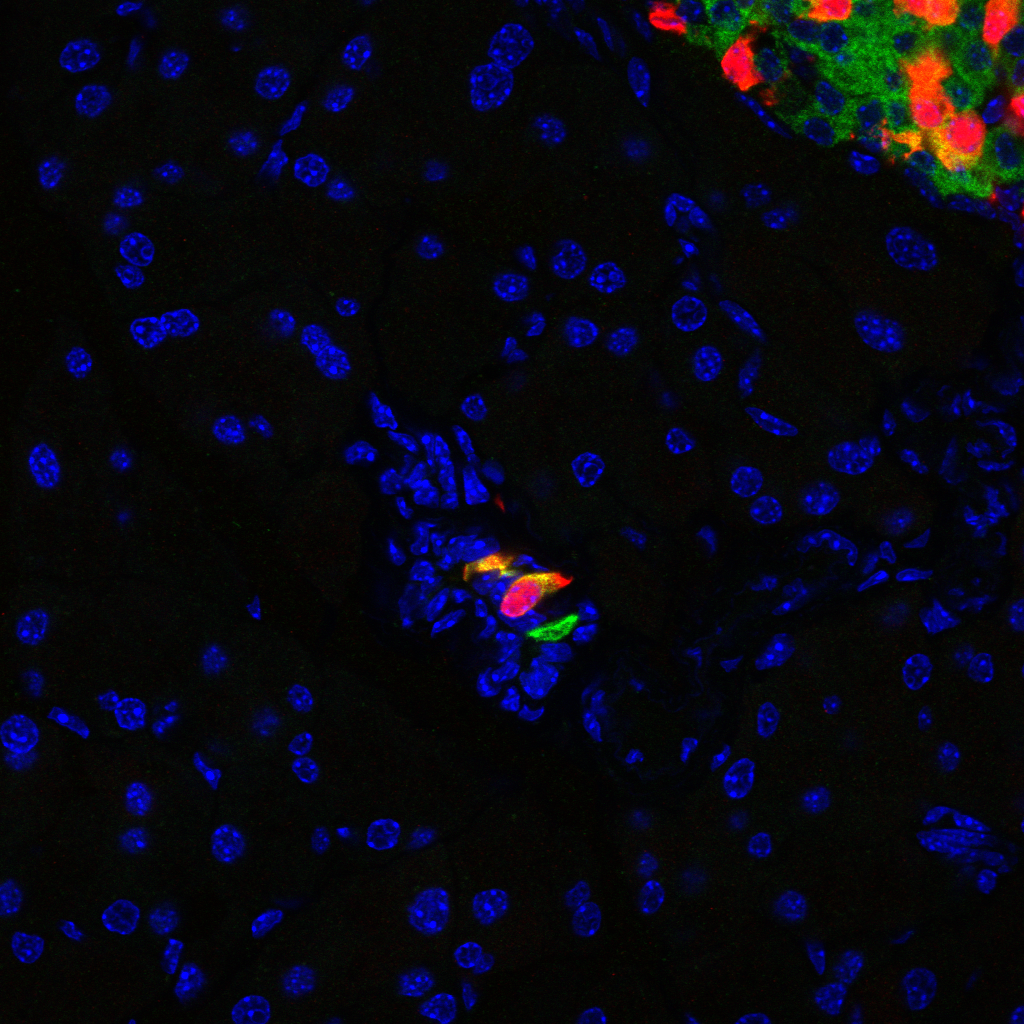

Supplement: Supplementary file 3 — Source data Fig. 1 [file 44318_2025_434_MOESM3_ESM.zip › Figure 1/1L/1L_12w_Ins.tif]

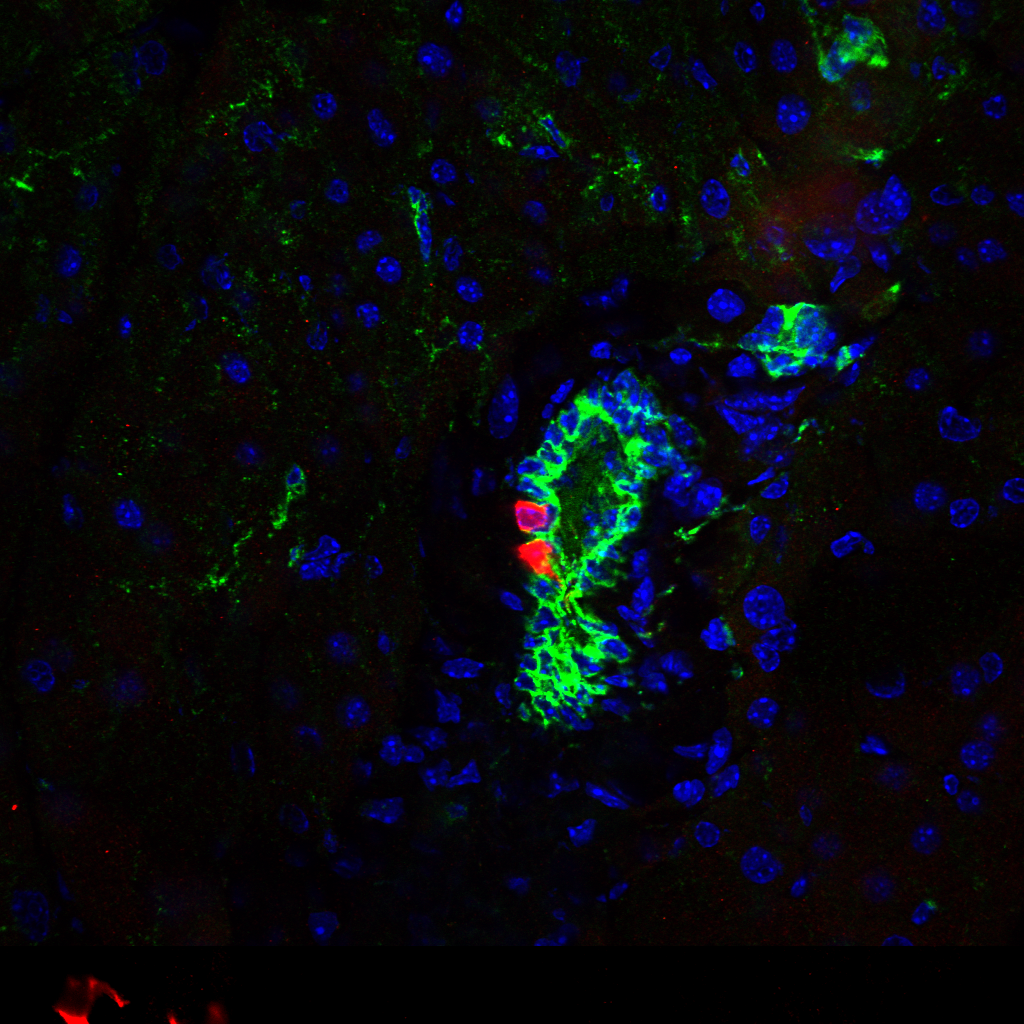

Supplement: Supplementary file 3 — Source data Fig. 1 [file 44318_2025_434_MOESM3_ESM.zip › Figure 1/1L/1L_12w_CK19.tif]

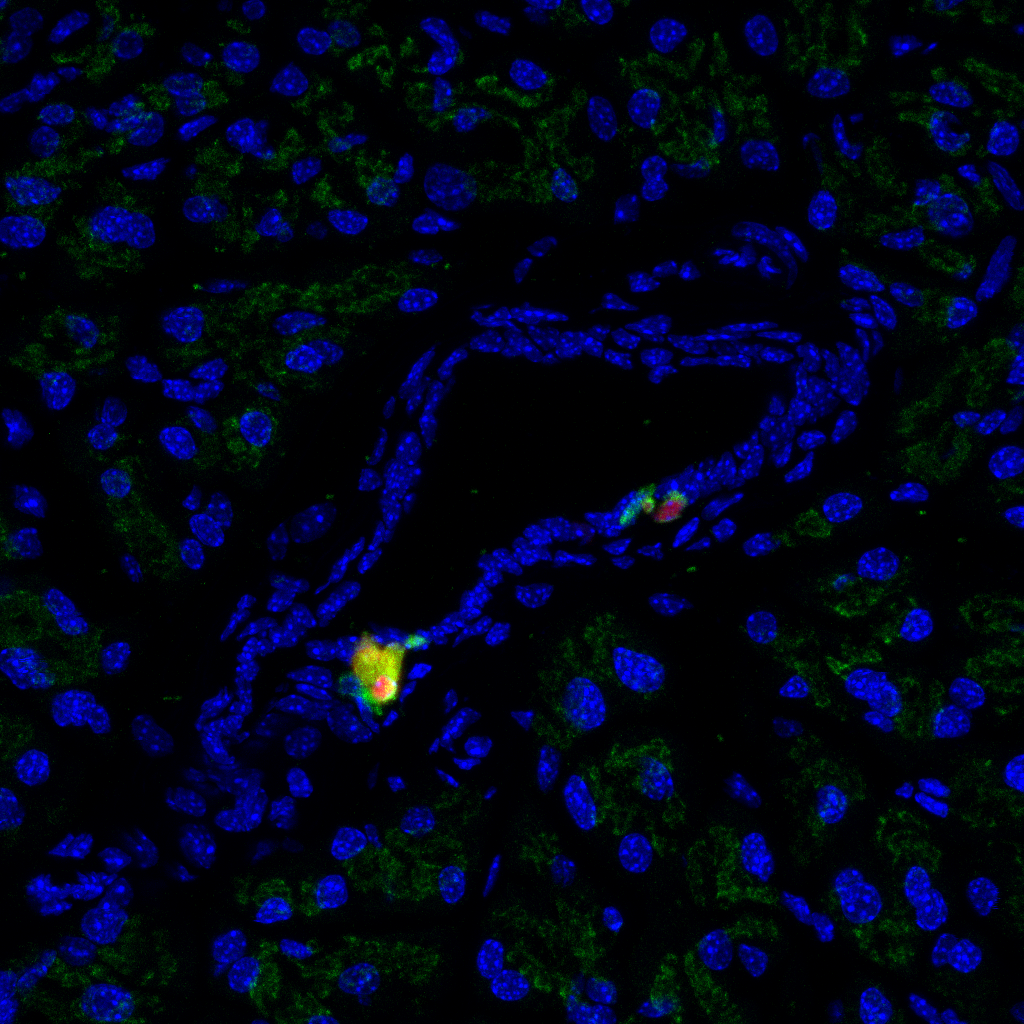

Supplement: Supplementary file 3 — Source data Fig. 1 [file 44318_2025_434_MOESM3_ESM.zip › Figure 1/1L/1L_2w_Ins.tif]

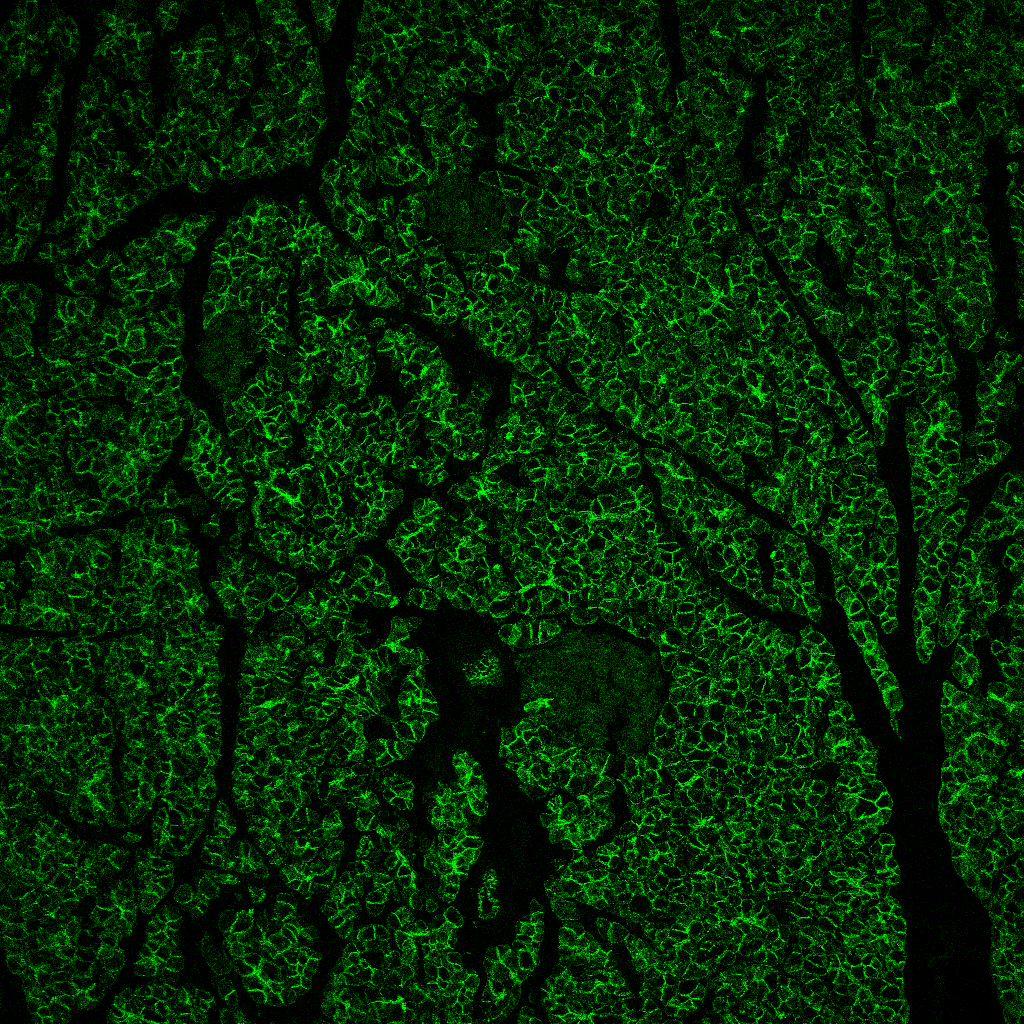

Supplement: Supplementary file 3 — Source data Fig. 1 [file 44318_2025_434_MOESM3_ESM.zip › Figure 1/1K/1K_2w (green).tif]

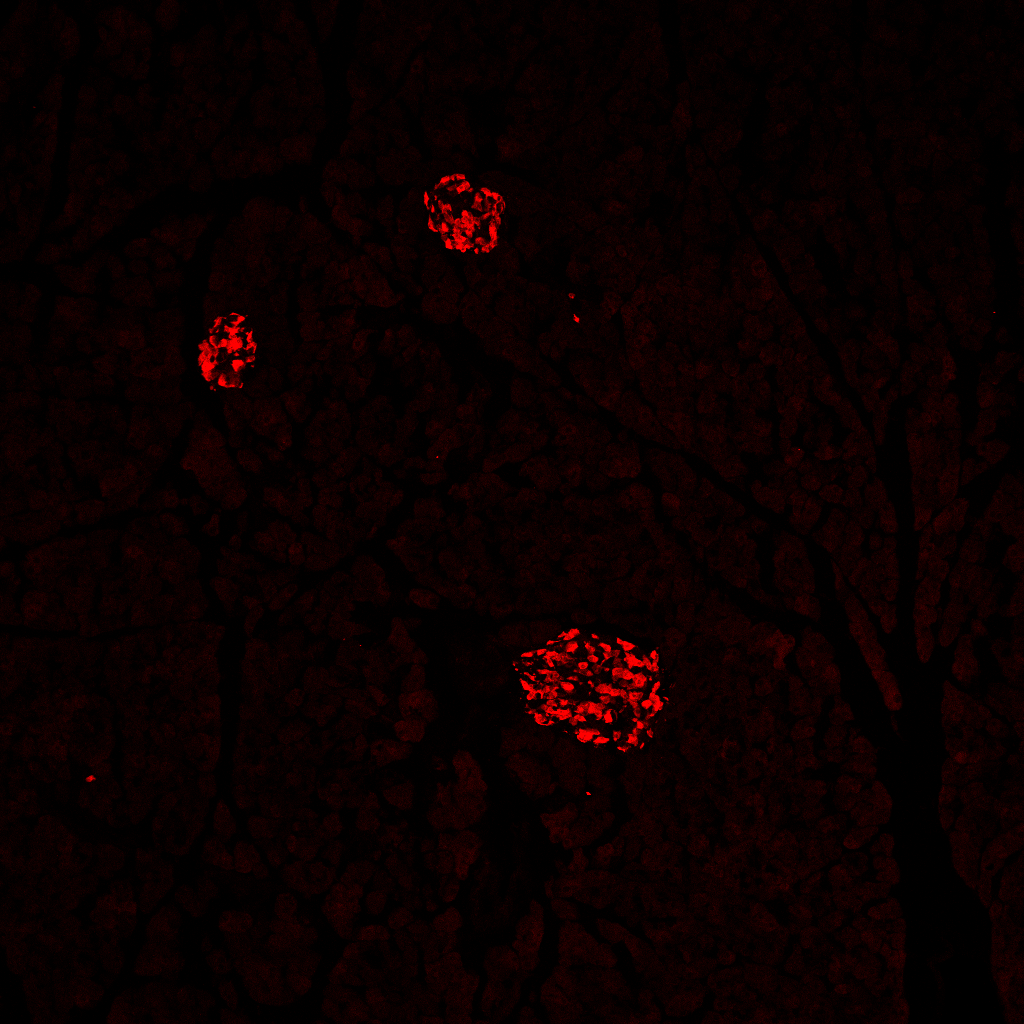

Supplement: Supplementary file 3 — Source data Fig. 1 [file 44318_2025_434_MOESM3_ESM.zip › Figure 1/1K/1K_2w (red).tif]

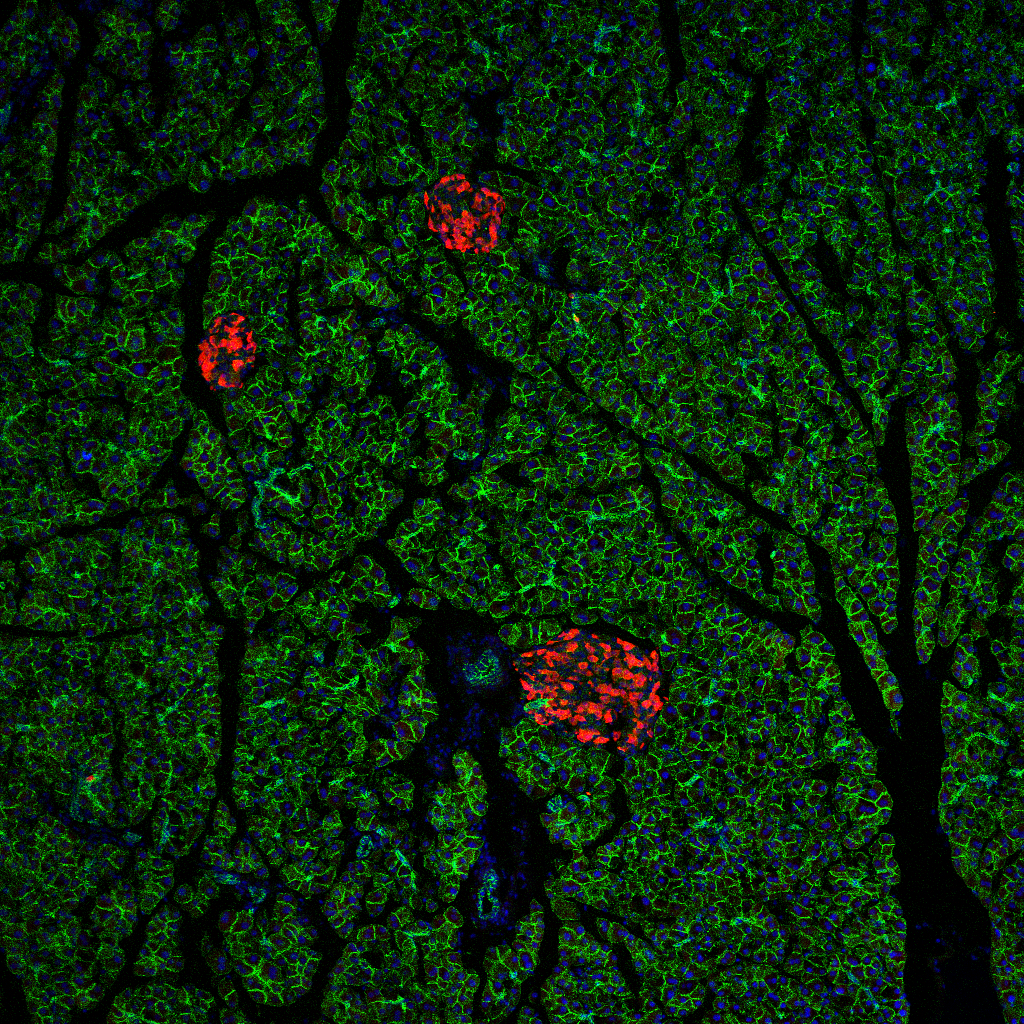

Supplement: Supplementary file 3 — Source data Fig. 1 [file 44318_2025_434_MOESM3_ESM.zip › Figure 1/1K/1K_2w.tif]

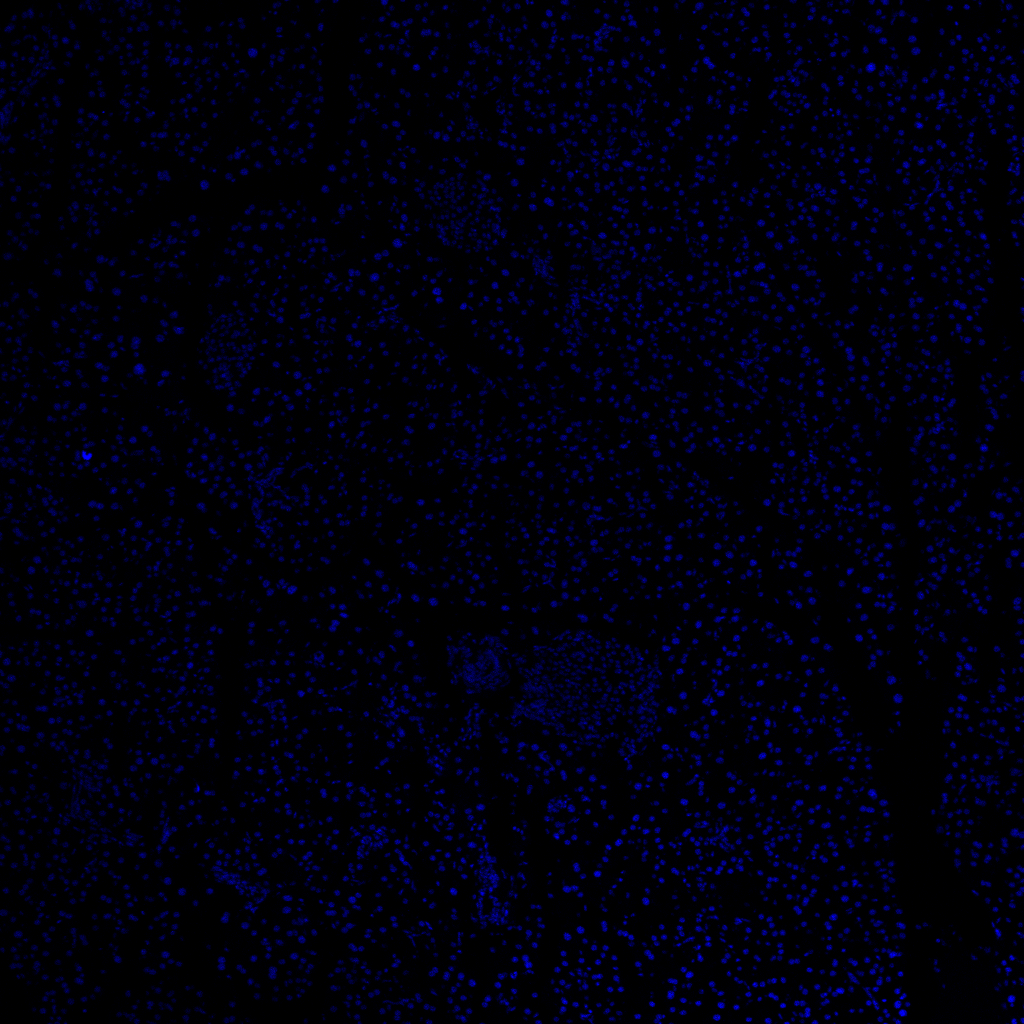

Supplement: Supplementary file 3 — Source data Fig. 1 [file 44318_2025_434_MOESM3_ESM.zip › Figure 1/1K/1K_2w (blue).tif]

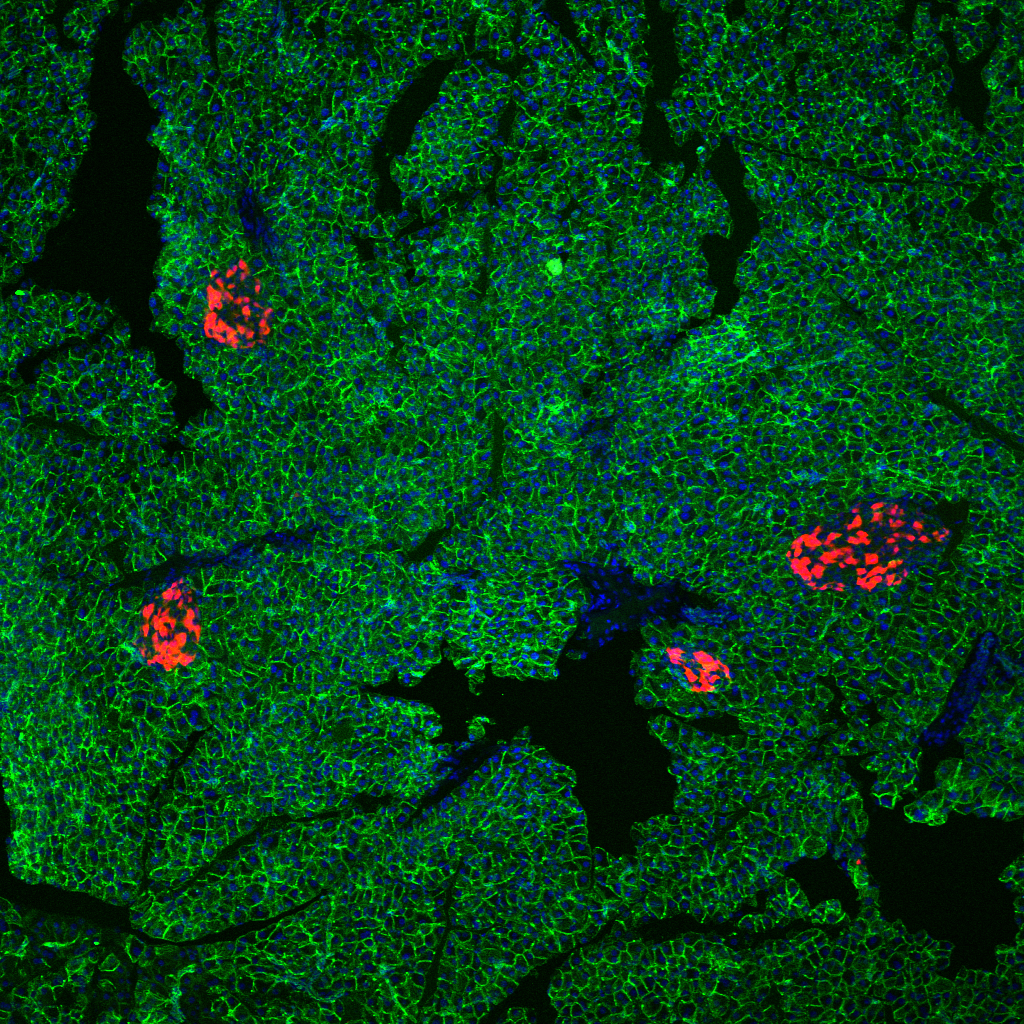

Supplement: Supplementary file 3 — Source data Fig. 1 [file 44318_2025_434_MOESM3_ESM.zip › Figure 1/1K/1K_12w.tif]

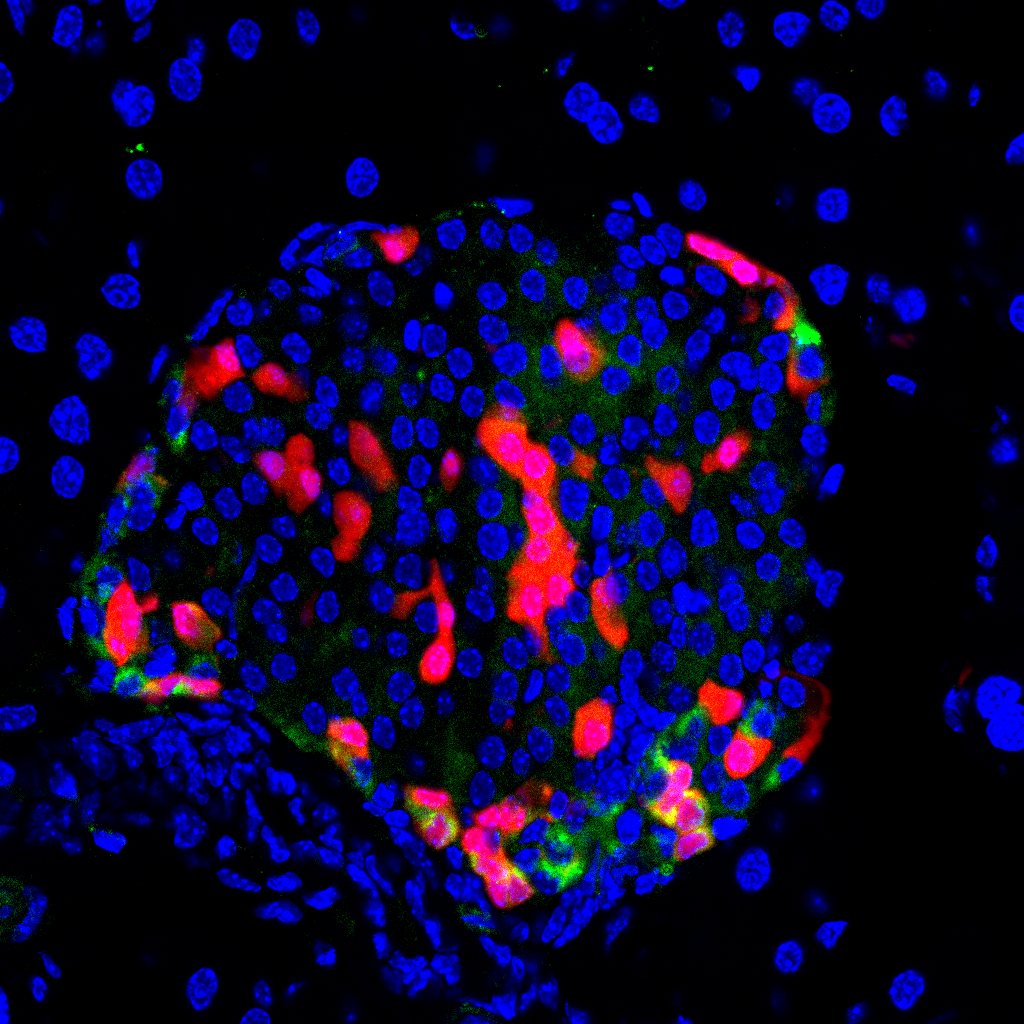

Supplement: Supplementary file 3 — Source data Fig. 1 [file 44318_2025_434_MOESM3_ESM.zip › Figure 1/1E/1E_12w_Ppy.tiff]

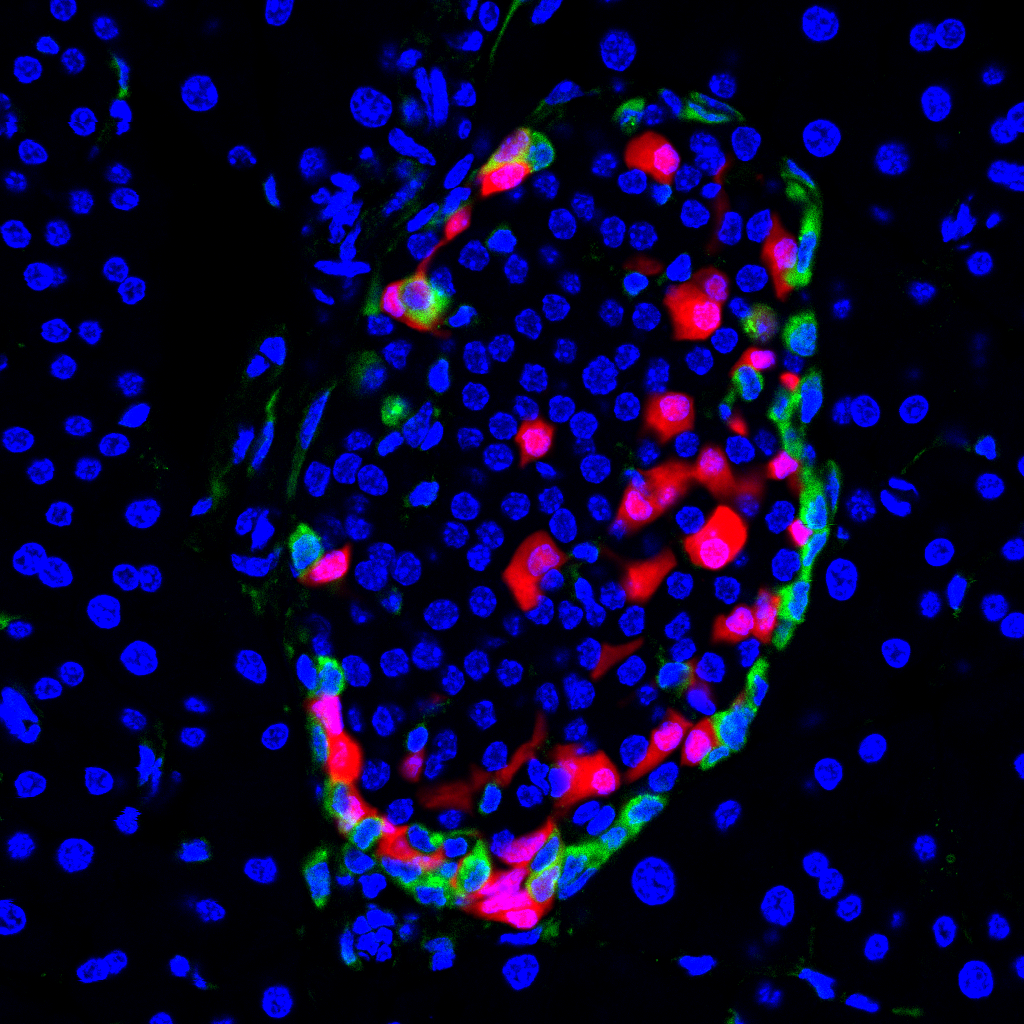

Supplement: Supplementary file 3 — Source data Fig. 1 [file 44318_2025_434_MOESM3_ESM.zip › Figure 1/1E/1E_12w_Gcg.tif]

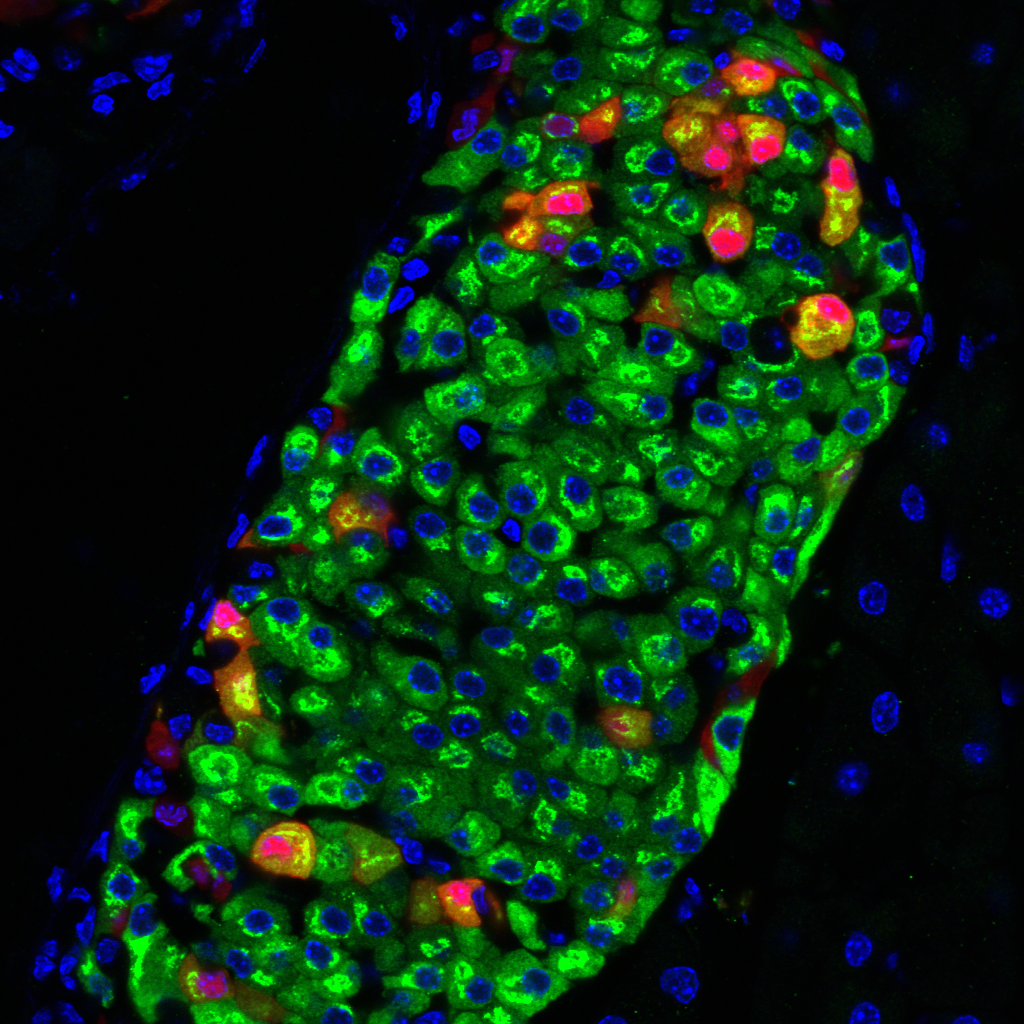

Supplement: Supplementary file 3 — Source data Fig. 1 [file 44318_2025_434_MOESM3_ESM.zip › Figure 1/1E/1E_2w_Ins.tif]

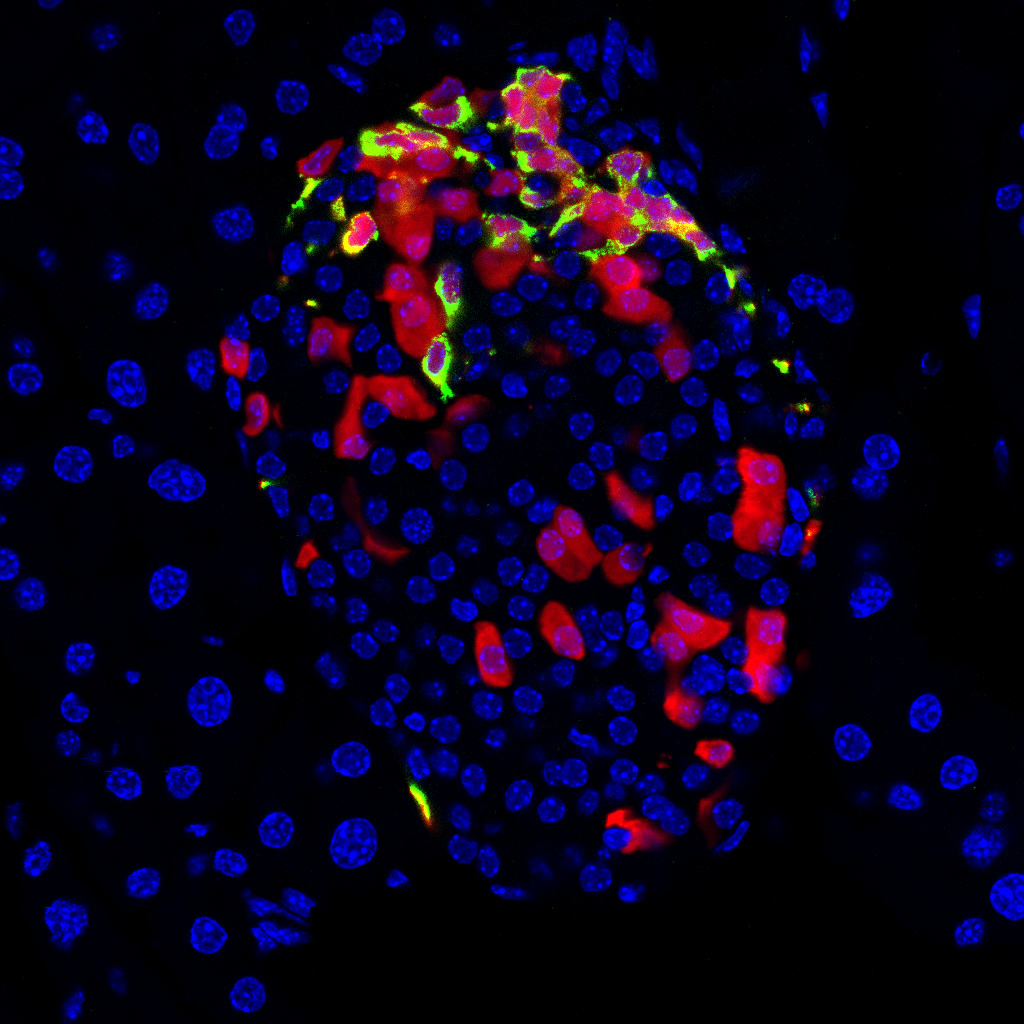

Supplement: Supplementary file 3 — Source data Fig. 1 [file 44318_2025_434_MOESM3_ESM.zip › Figure 1/1E/1E_12w_Sst.tif]

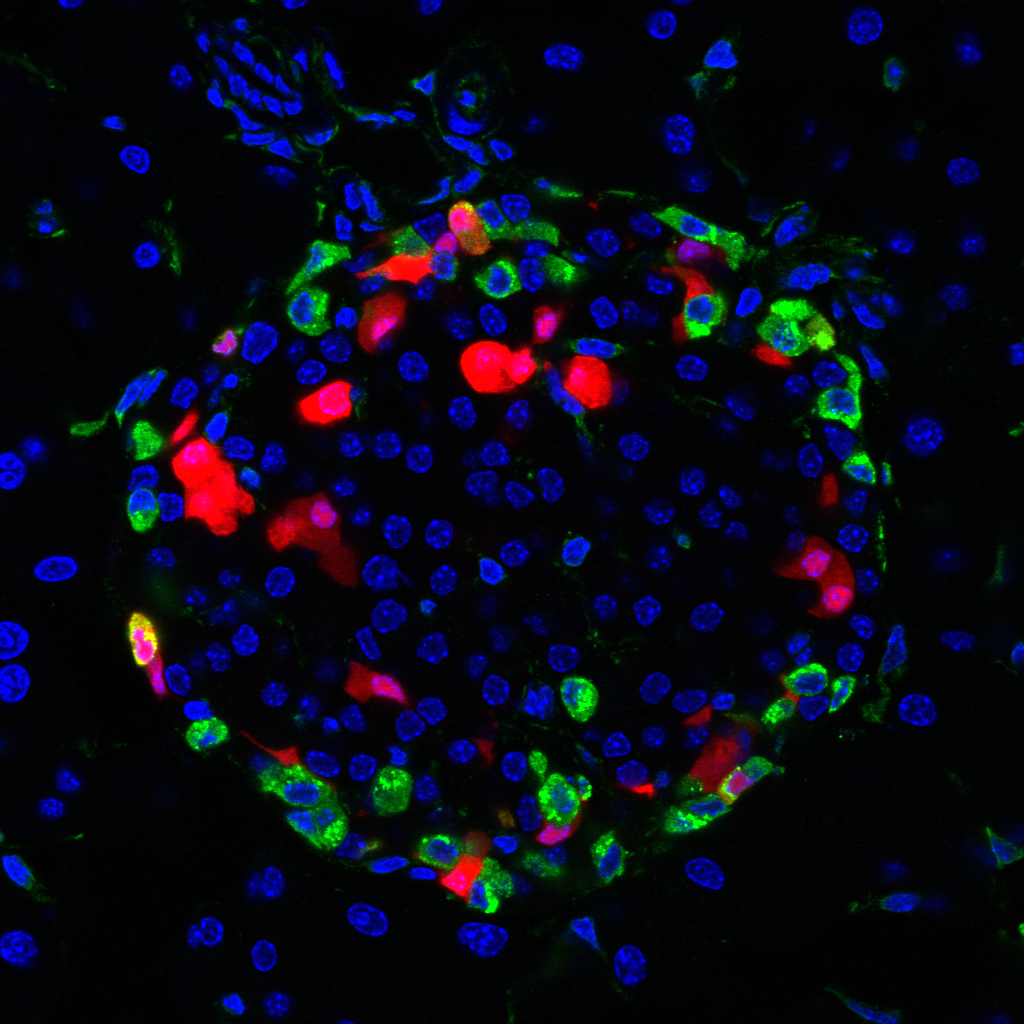

Supplement: Supplementary file 3 — Source data Fig. 1 [file 44318_2025_434_MOESM3_ESM.zip › Figure 1/1E/1E_2w_Gcg.tif]

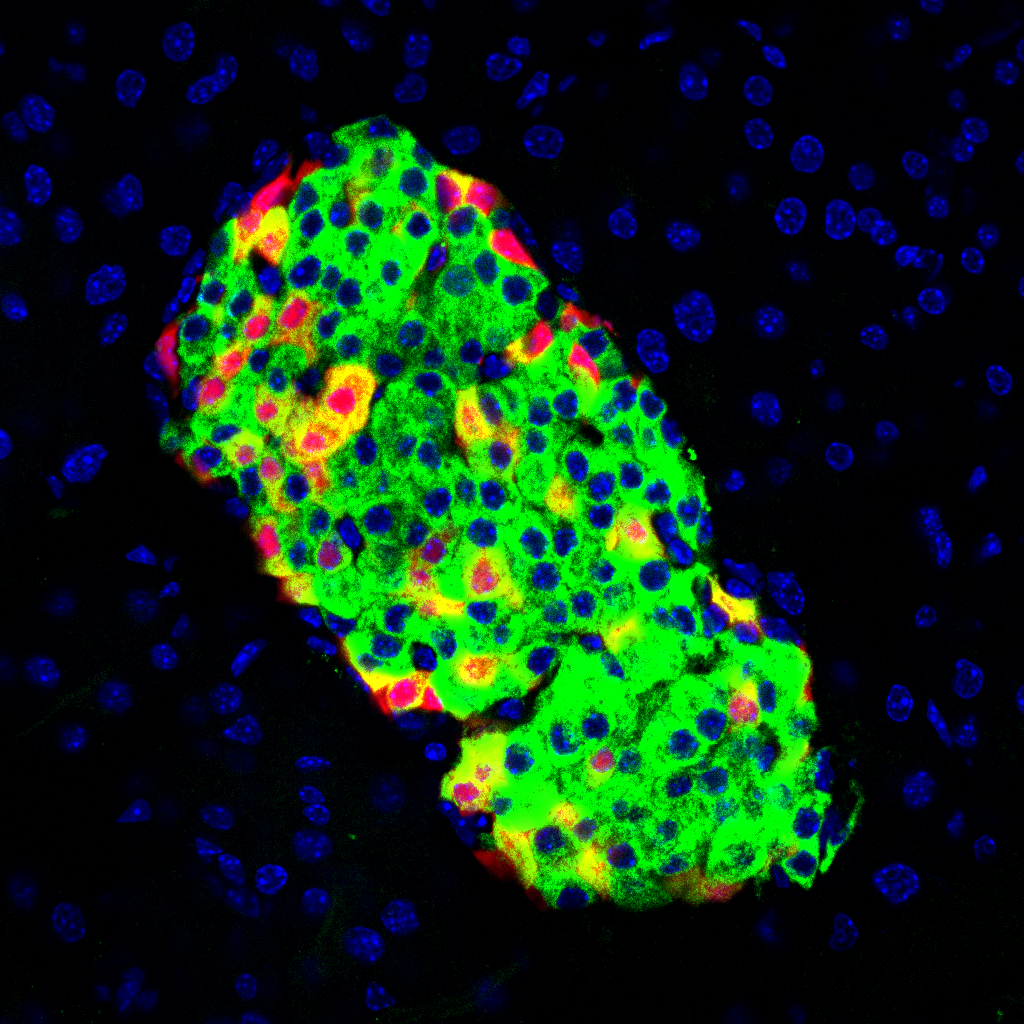

Supplement: Supplementary file 3 — Source data Fig. 1 [file 44318_2025_434_MOESM3_ESM.zip › Figure 1/1E/1E_12w_Ins.tif]

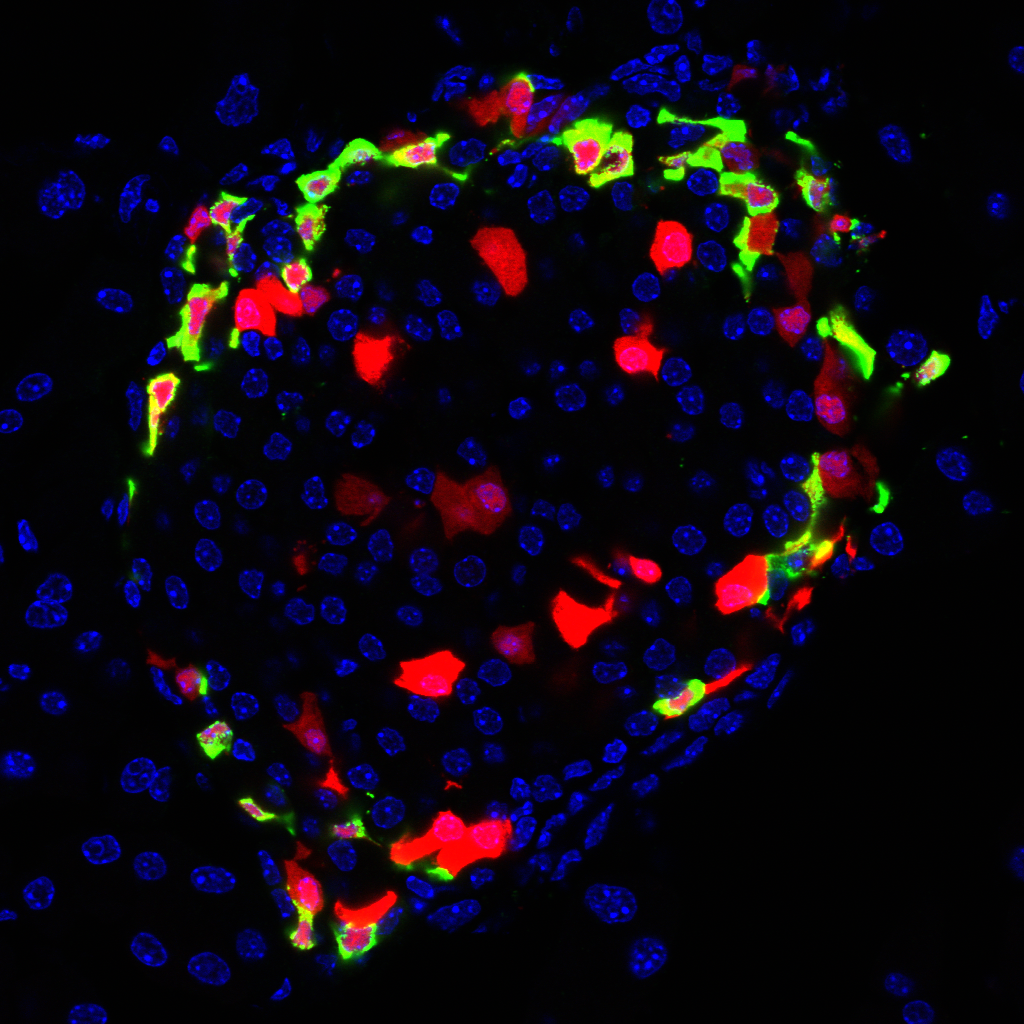

Supplement: Supplementary file 3 — Source data Fig. 1 [file 44318_2025_434_MOESM3_ESM.zip › Figure 1/1E/1E_2w_Sst.tif]

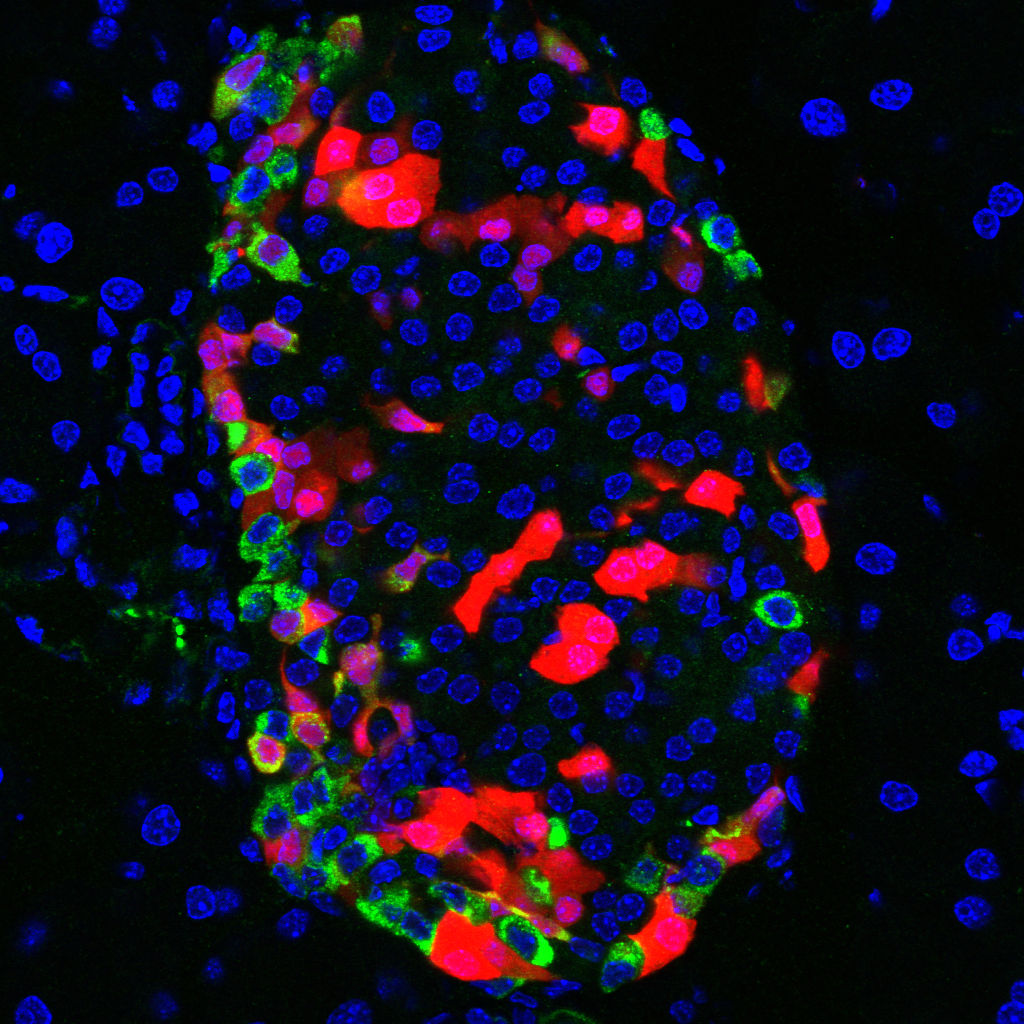

Supplement: Supplementary file 3 — Source data Fig. 1 [file 44318_2025_434_MOESM3_ESM.zip › Figure 1/1E/1E_2w_Ppy.tif]

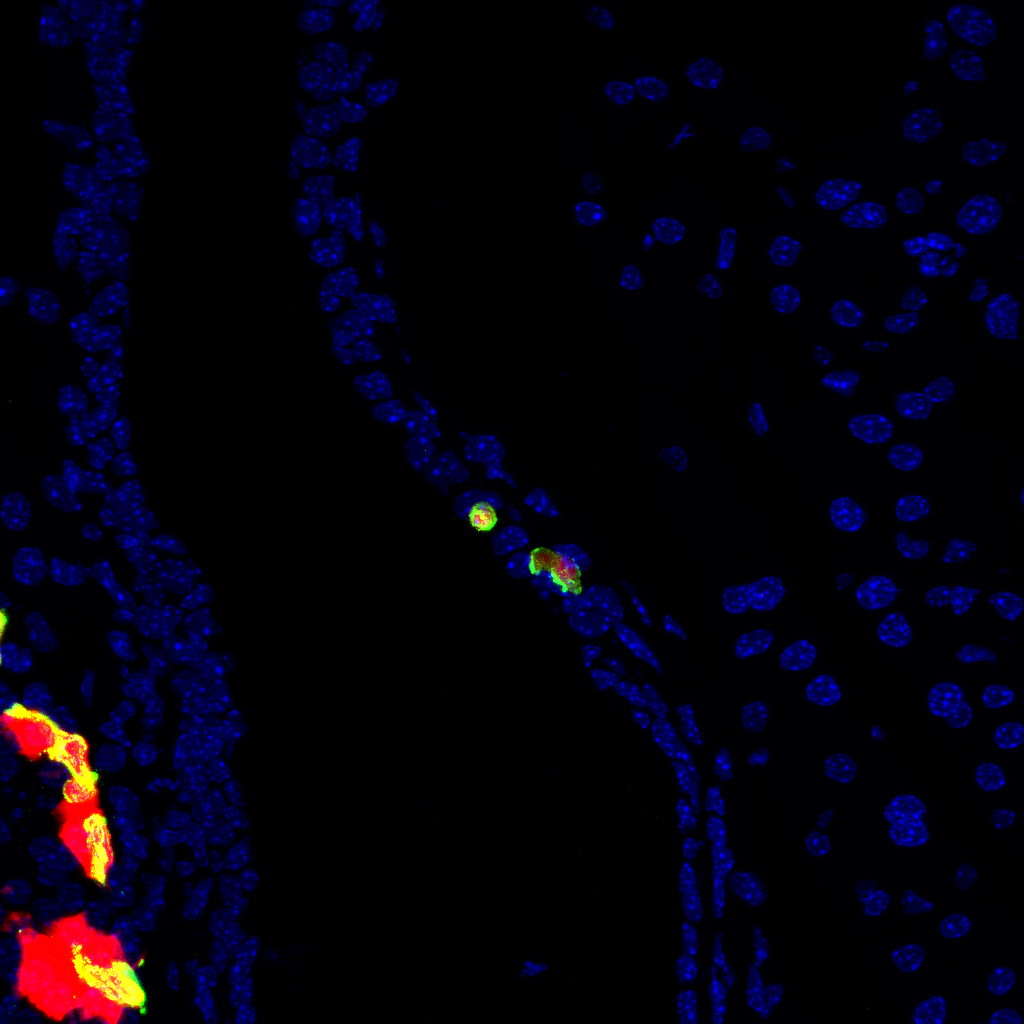

Supplement: Supplementary file 3 — Source data Fig. 1 [file 44318_2025_434_MOESM3_ESM.zip › Figure 1/1D/1D_2w_Sst.tif]

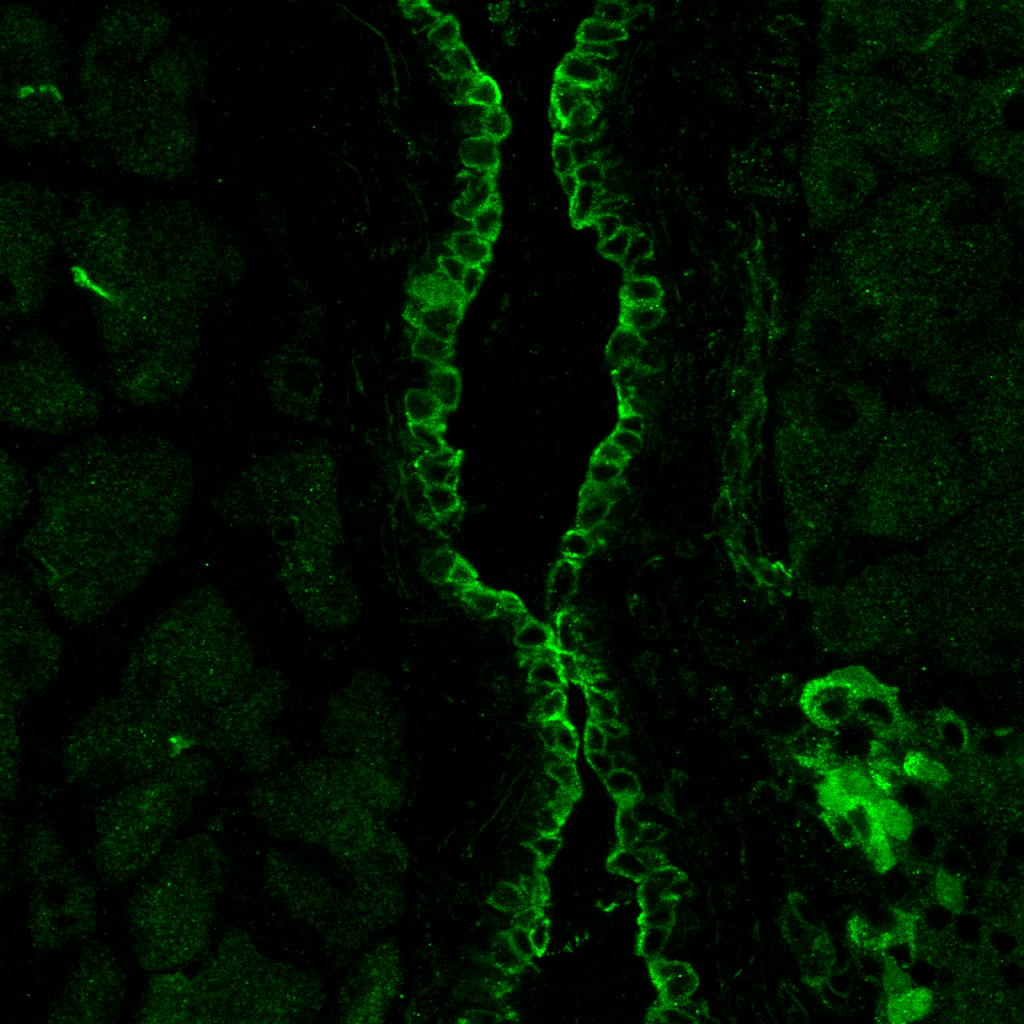

Supplement: Supplementary file 3 — Source data Fig. 1 [file 44318_2025_434_MOESM3_ESM.zip › Figure 1/1D/1D_2w_CK19 (green).tif]

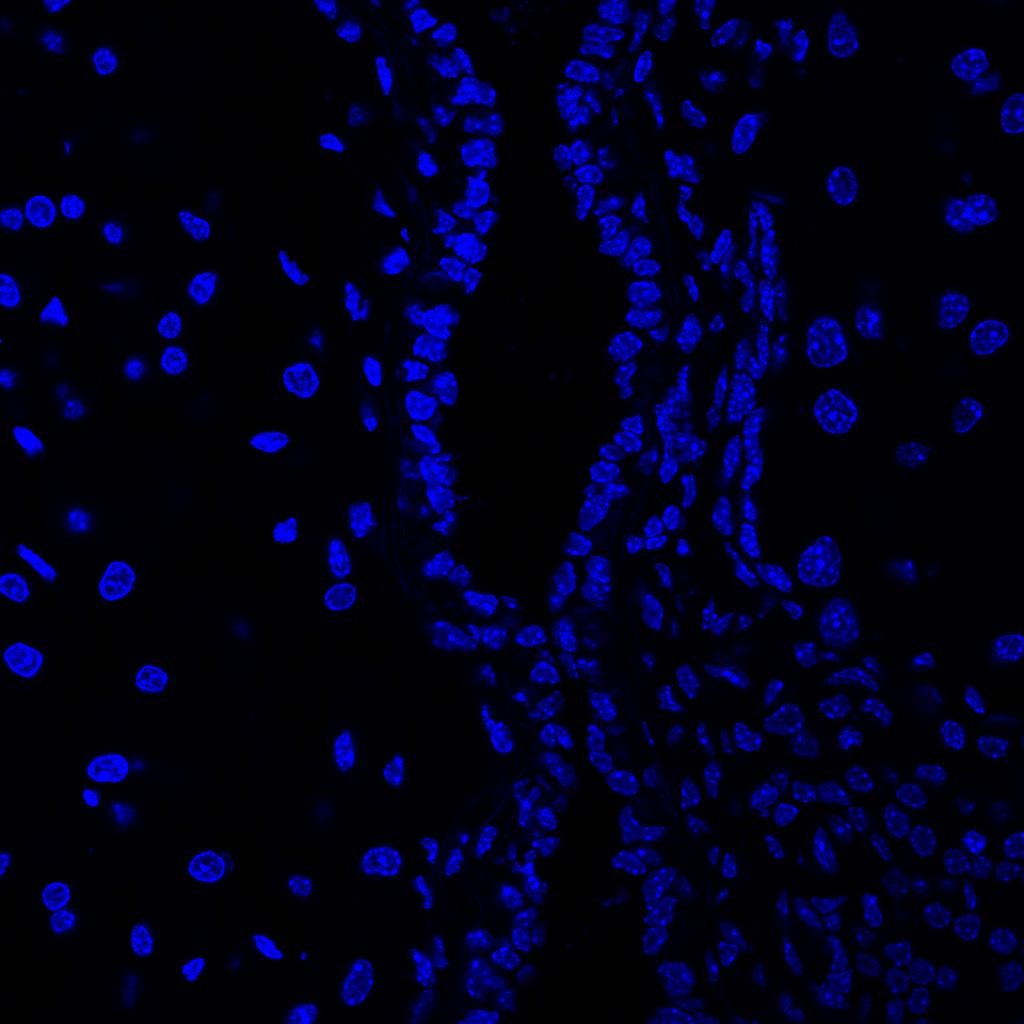

Supplement: Supplementary file 3 — Source data Fig. 1 [file 44318_2025_434_MOESM3_ESM.zip › Figure 1/1D/1D_2w_CK19 (blue).tif]

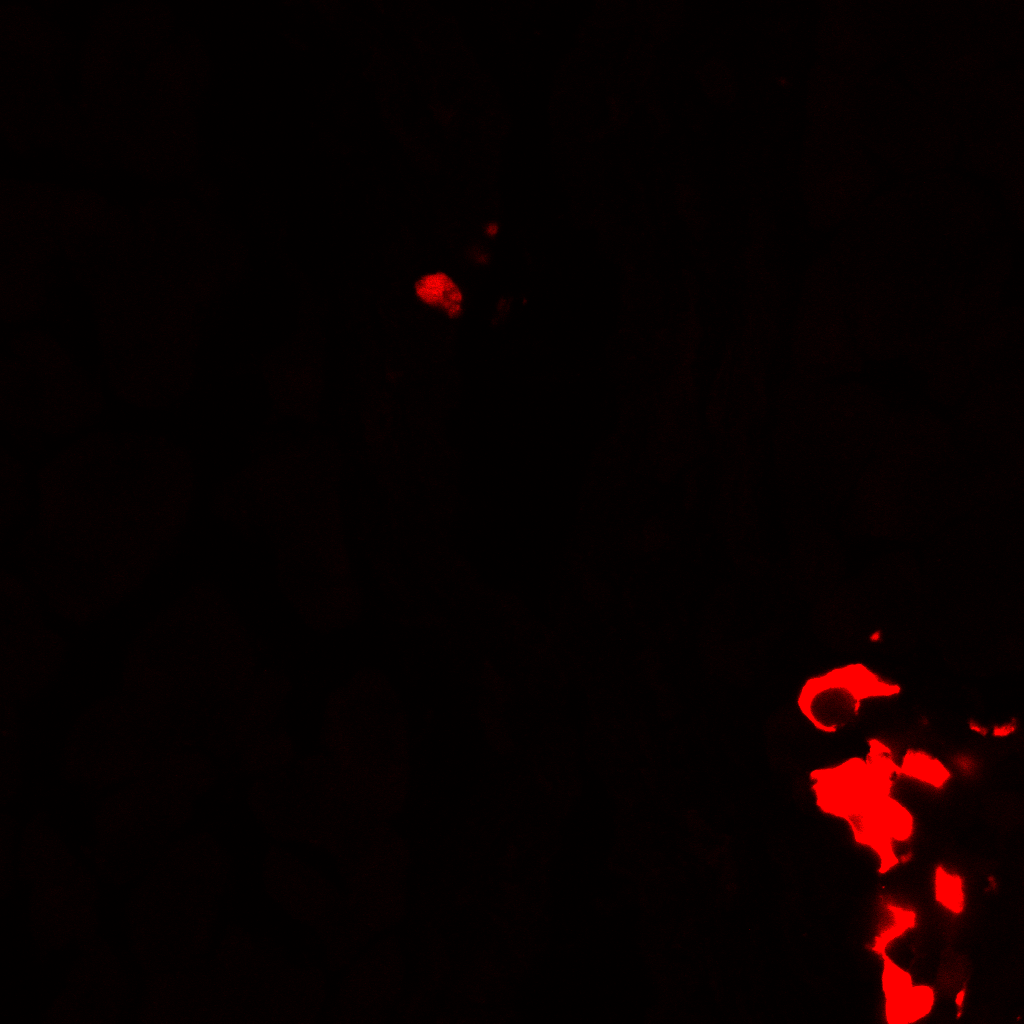

Supplement: Supplementary file 3 — Source data Fig. 1 [file 44318_2025_434_MOESM3_ESM.zip › Figure 1/1D/1D_2w_CK19 (red).tif]

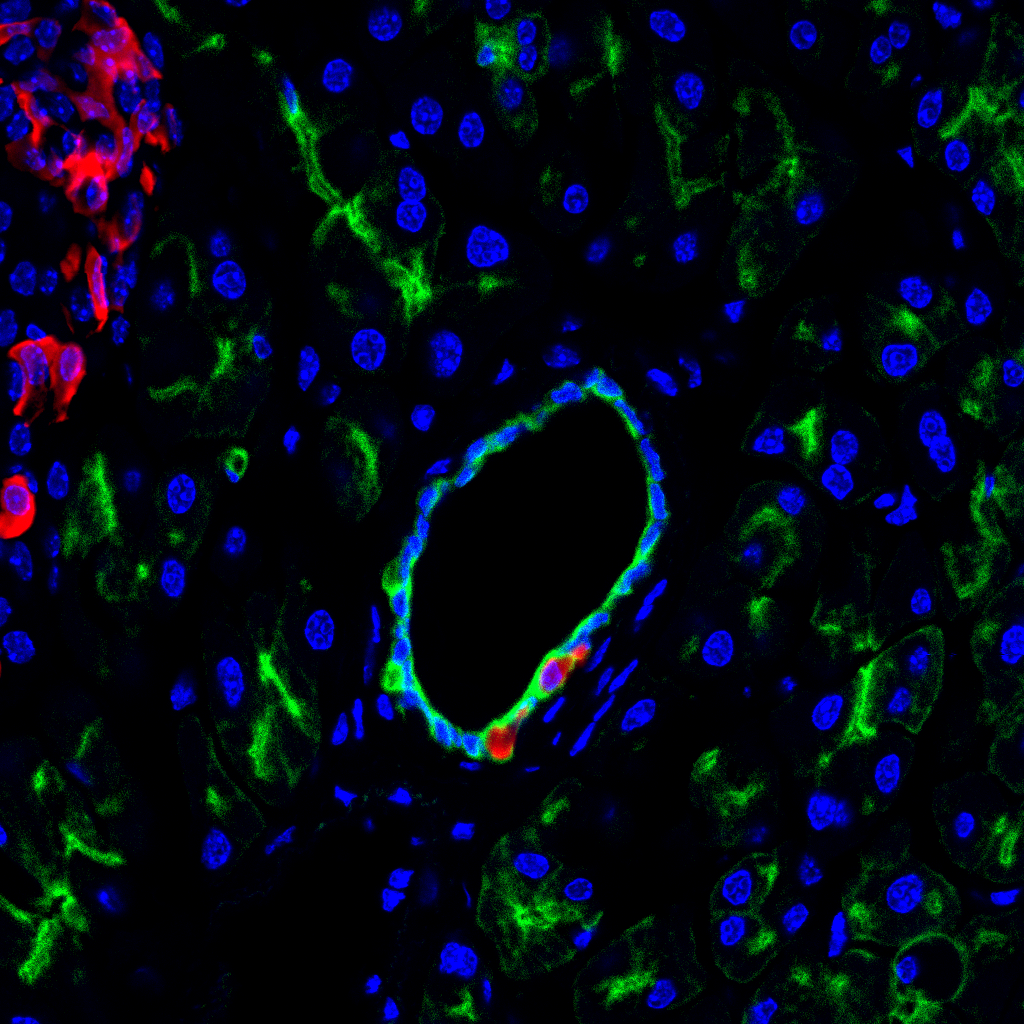

Supplement: Supplementary file 3 — Source data Fig. 1 [file 44318_2025_434_MOESM3_ESM.zip › Figure 1/1D/1D_12w_CK19.tif]

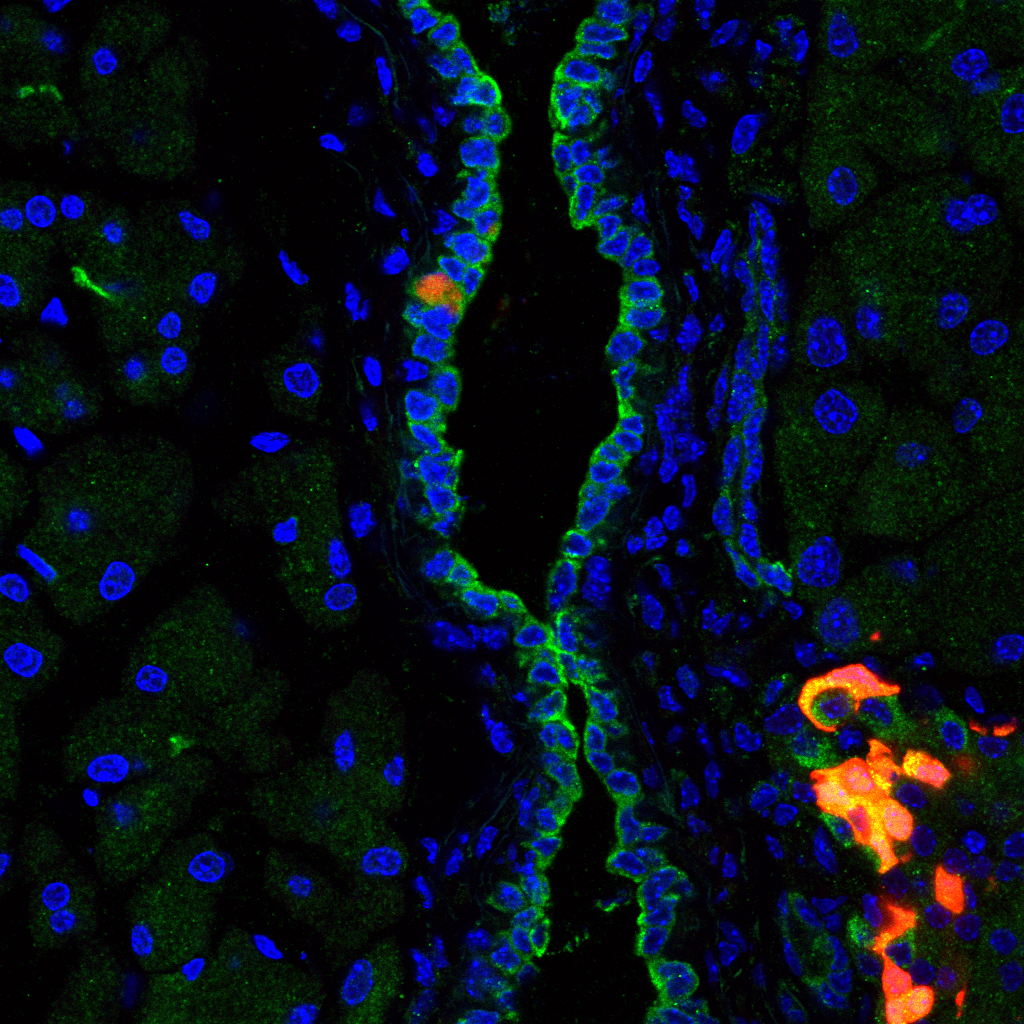

Supplement: Supplementary file 3 — Source data Fig. 1 [file 44318_2025_434_MOESM3_ESM.zip › Figure 1/1D/1D_2w_CK19.tif]

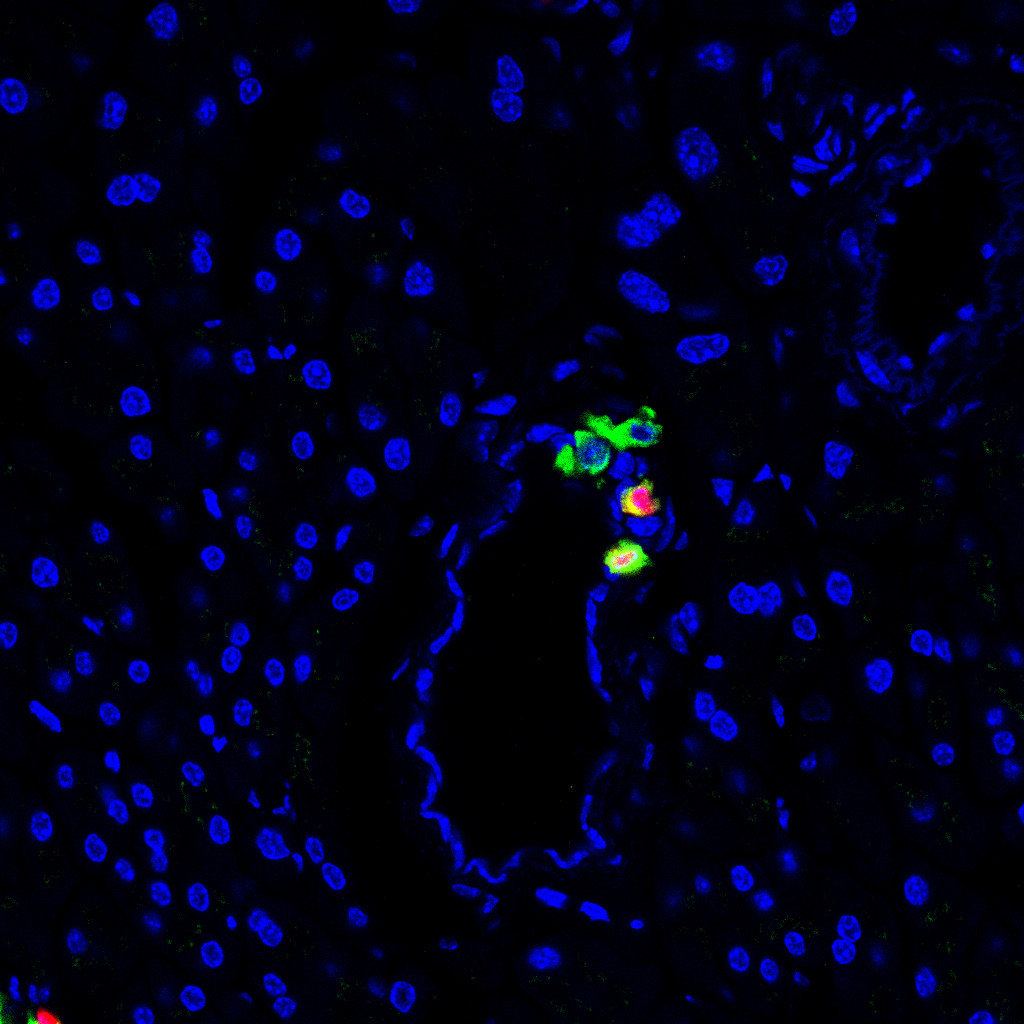

Supplement: Supplementary file 3 — Source data Fig. 1 [file 44318_2025_434_MOESM3_ESM.zip › Figure 1/1D/1D_12w_Ins.tiff]

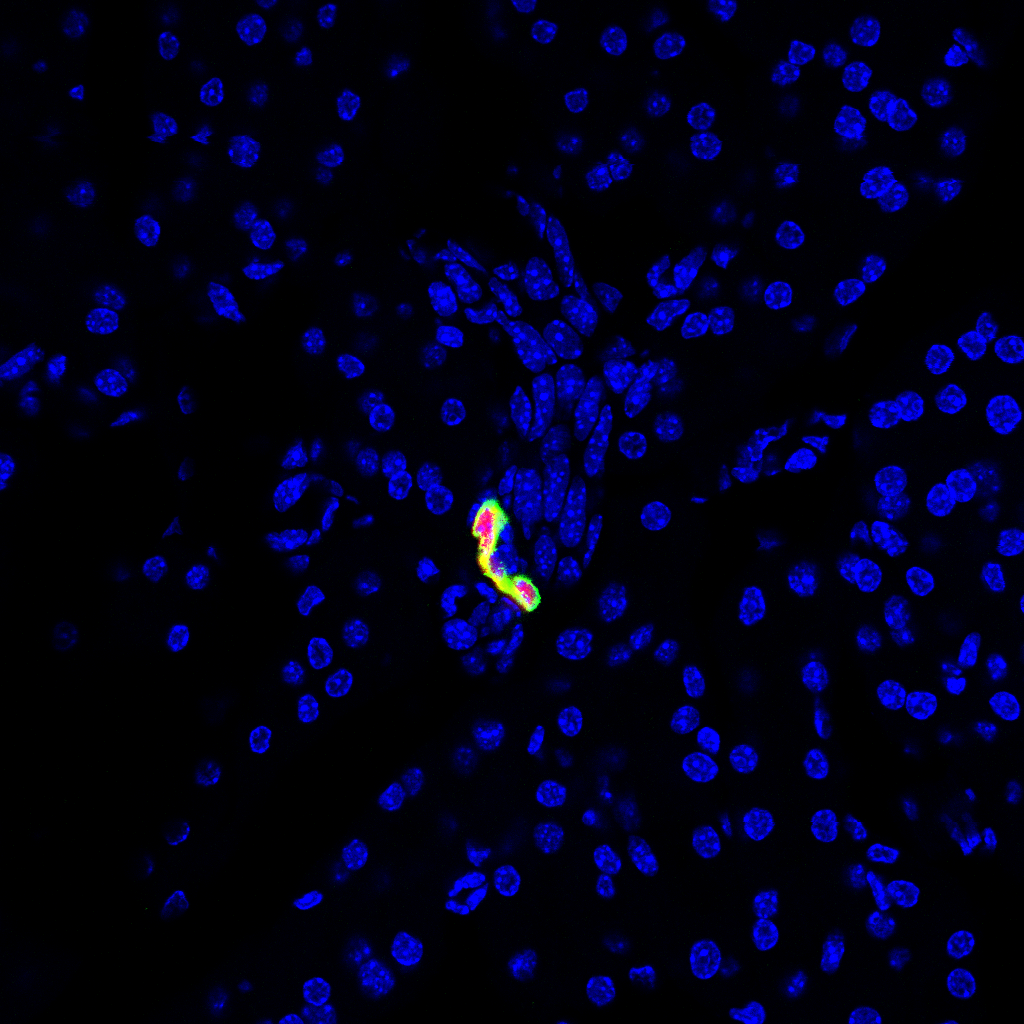

Supplement: Supplementary file 3 — Source data Fig. 1 [file 44318_2025_434_MOESM3_ESM.zip › Figure 1/1D/1D_12w_Sst.tif]

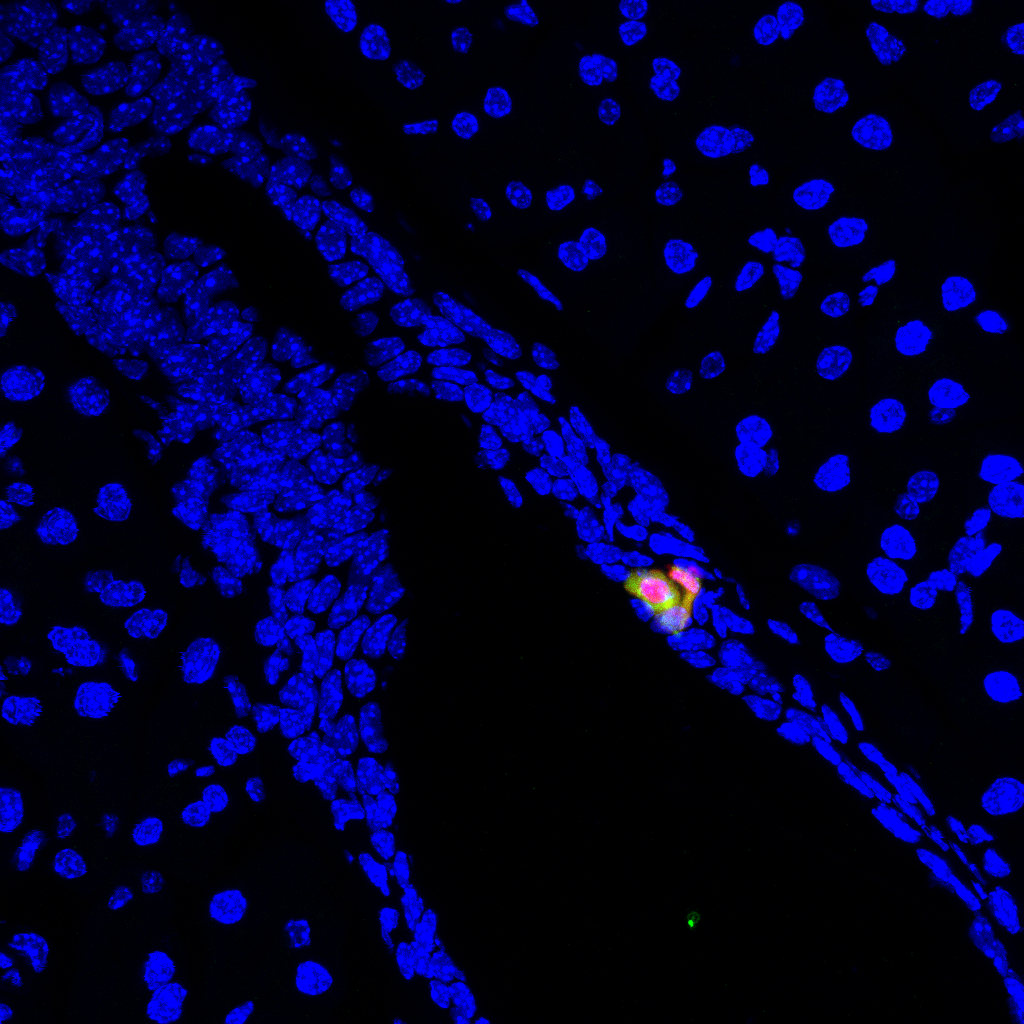

Supplement: Supplementary file 3 — Source data Fig. 1 [file 44318_2025_434_MOESM3_ESM.zip › Figure 1/1D/1D_2w_Ins.tif]

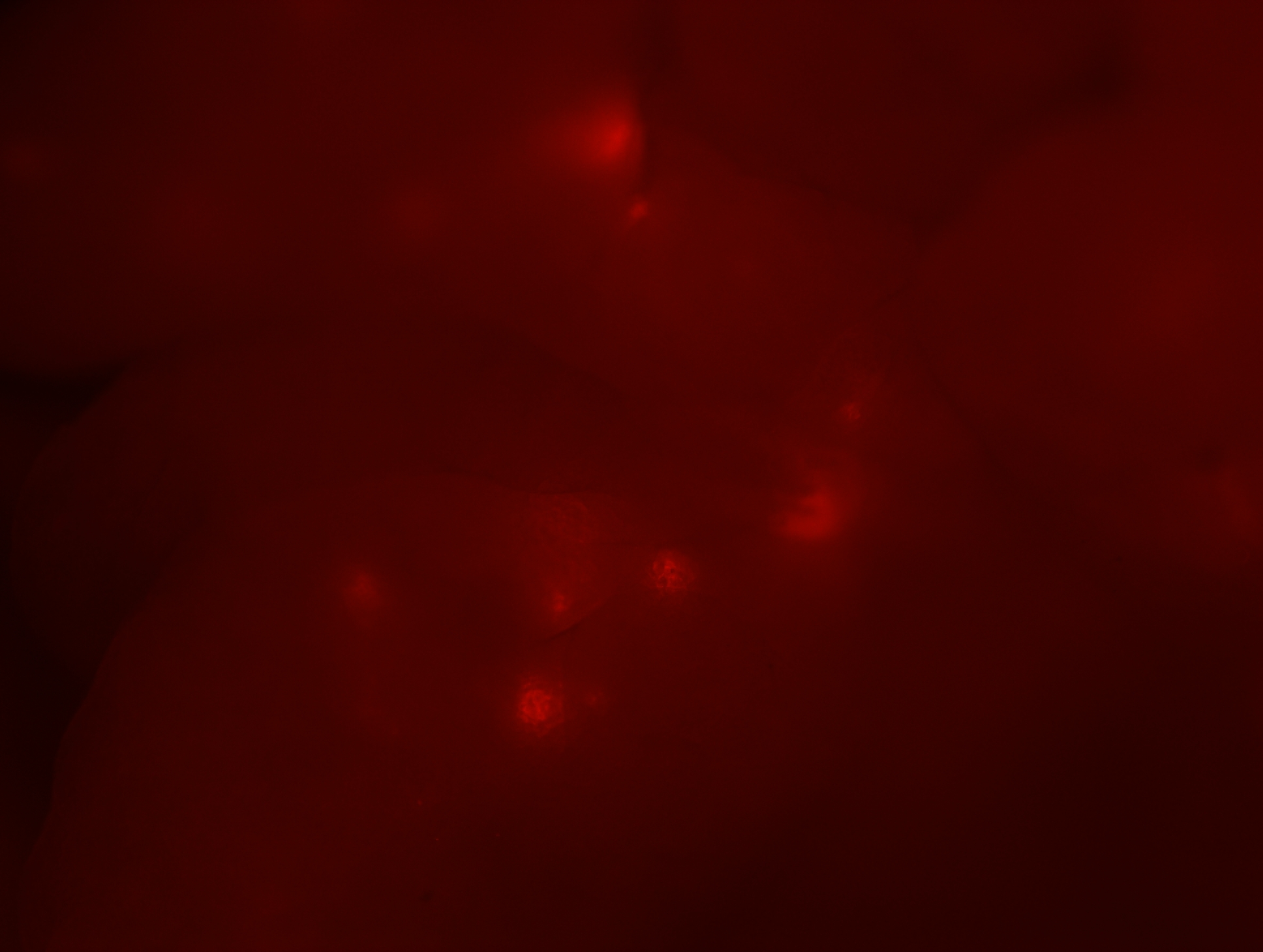

Supplement: Supplementary file 3 — Source data Fig. 1 [file 44318_2025_434_MOESM3_ESM.zip › Figure 1/1C/1C_12w_tdT_mag.tif]

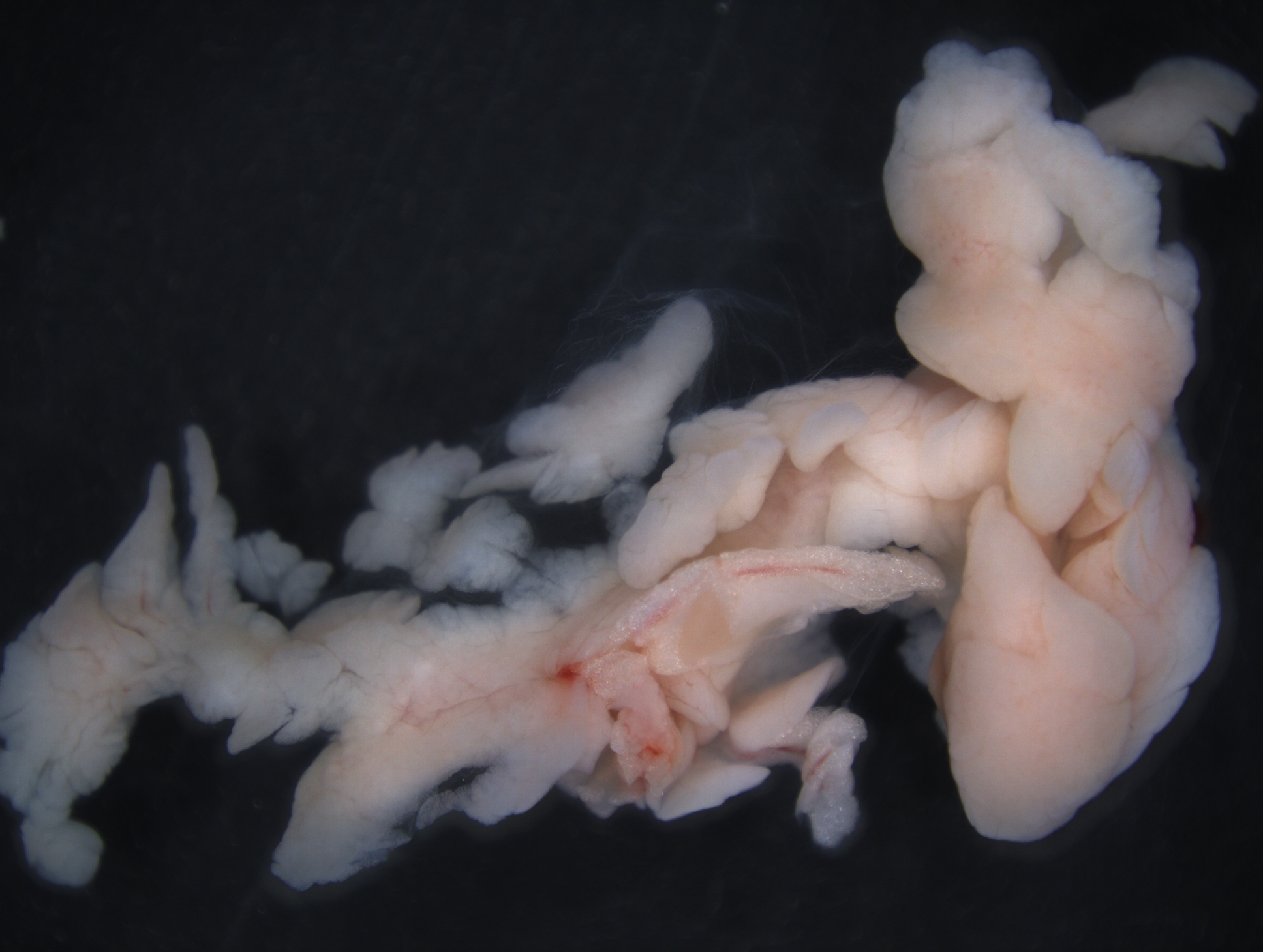

Supplement: Supplementary file 3 — Source data Fig. 1 [file 44318_2025_434_MOESM3_ESM.zip › Figure 1/1C/1C_2w_BF.tif]

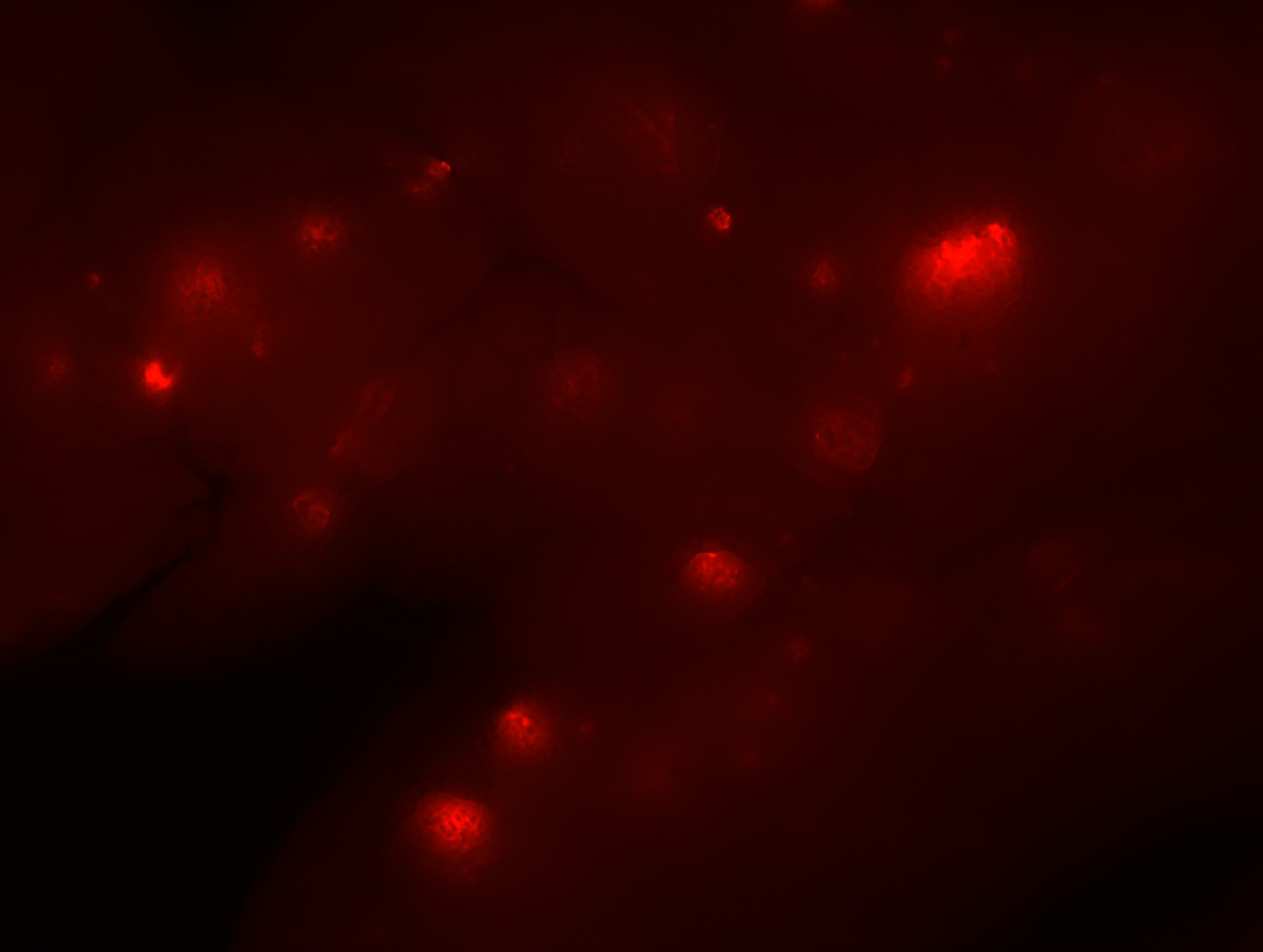

Supplement: Supplementary file 3 — Source data Fig. 1 [file 44318_2025_434_MOESM3_ESM.zip › Figure 1/1C/1C_2w_tdT_mag.tif]

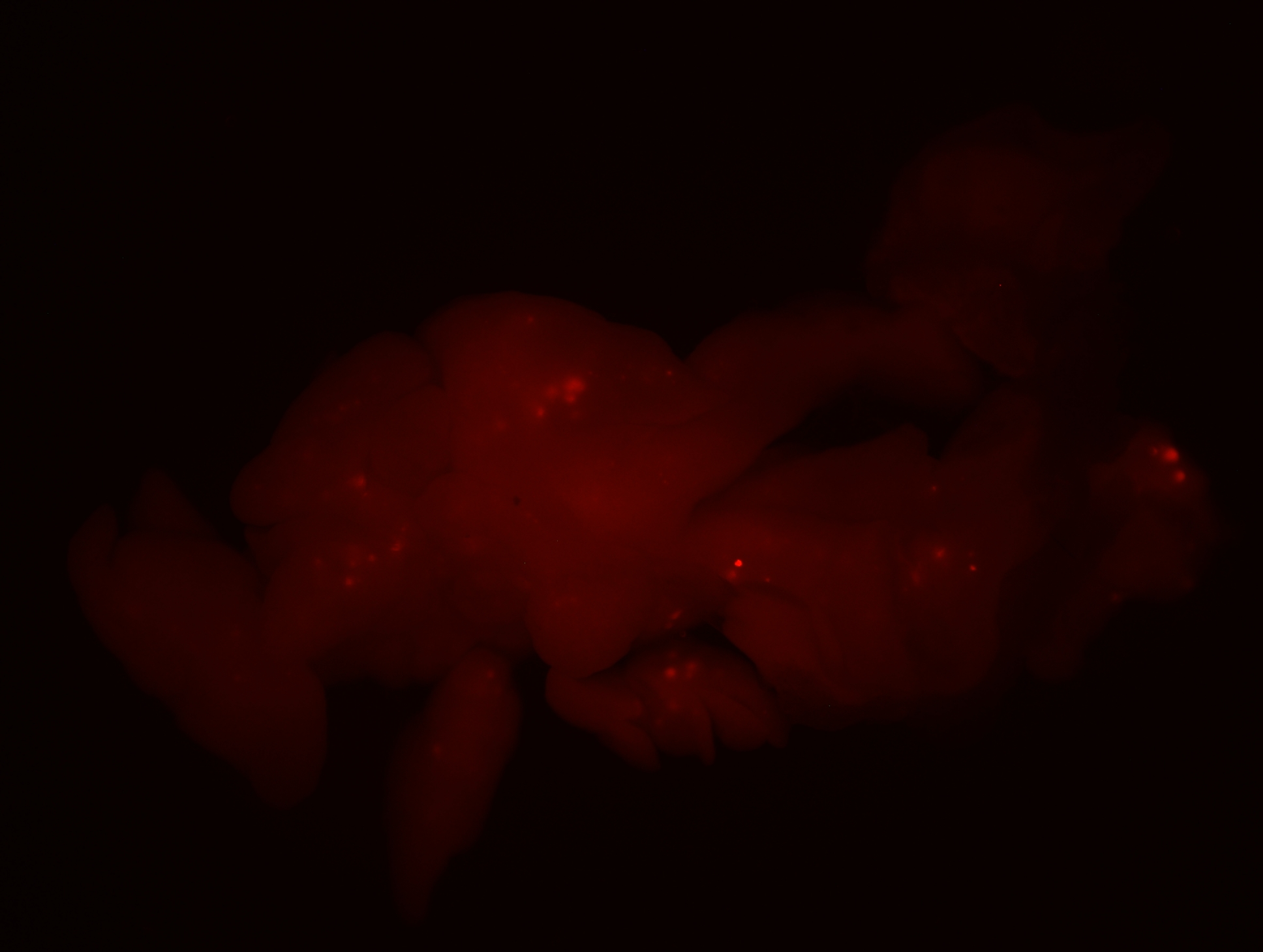

Supplement: Supplementary file 3 — Source data Fig. 1 [file 44318_2025_434_MOESM3_ESM.zip › Figure 1/1C/1C_12w_tdT.tif]

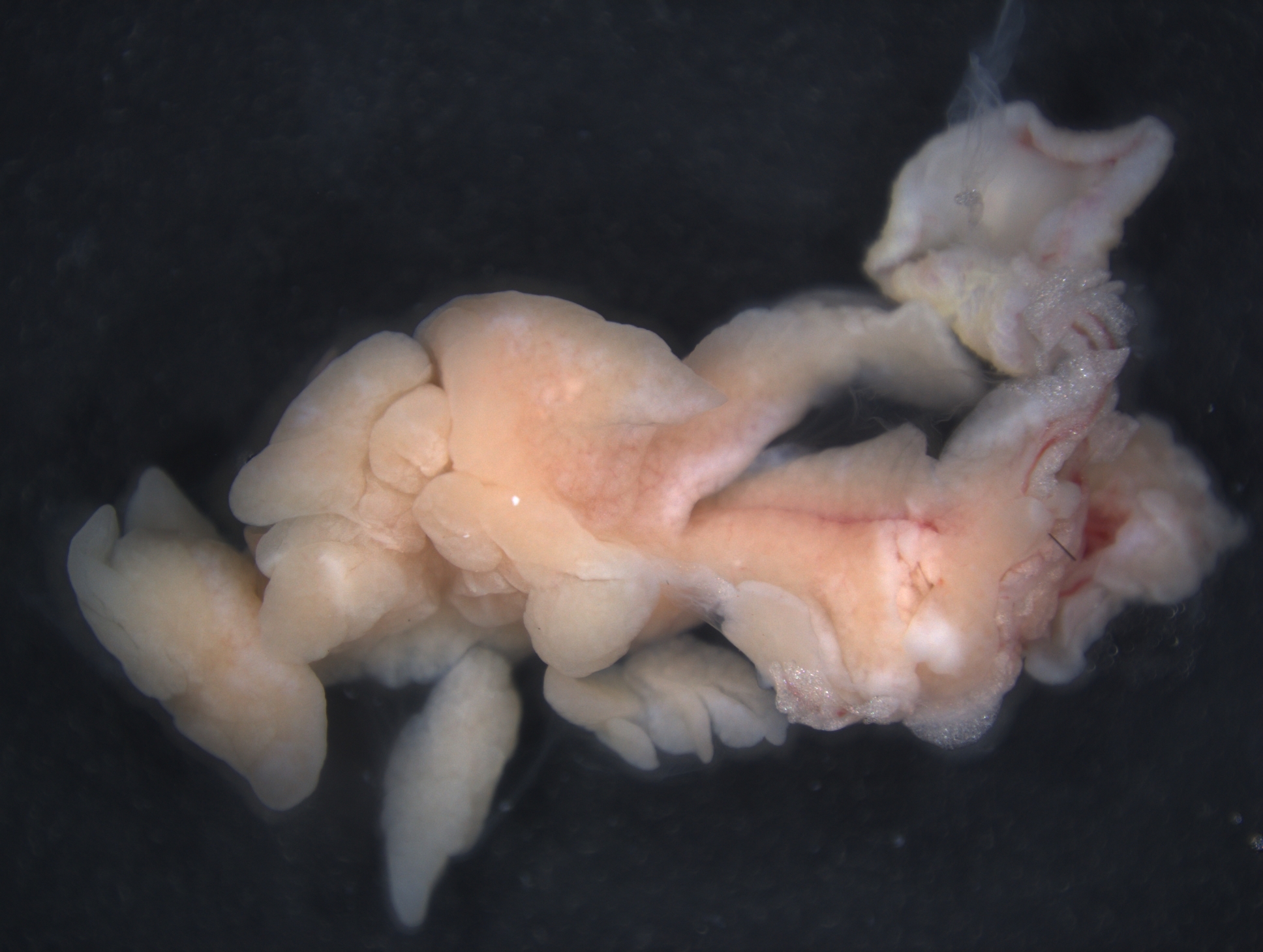

Supplement: Supplementary file 3 — Source data Fig. 1 [file 44318_2025_434_MOESM3_ESM.zip › Figure 1/1C/1C_12w_BF.tif]

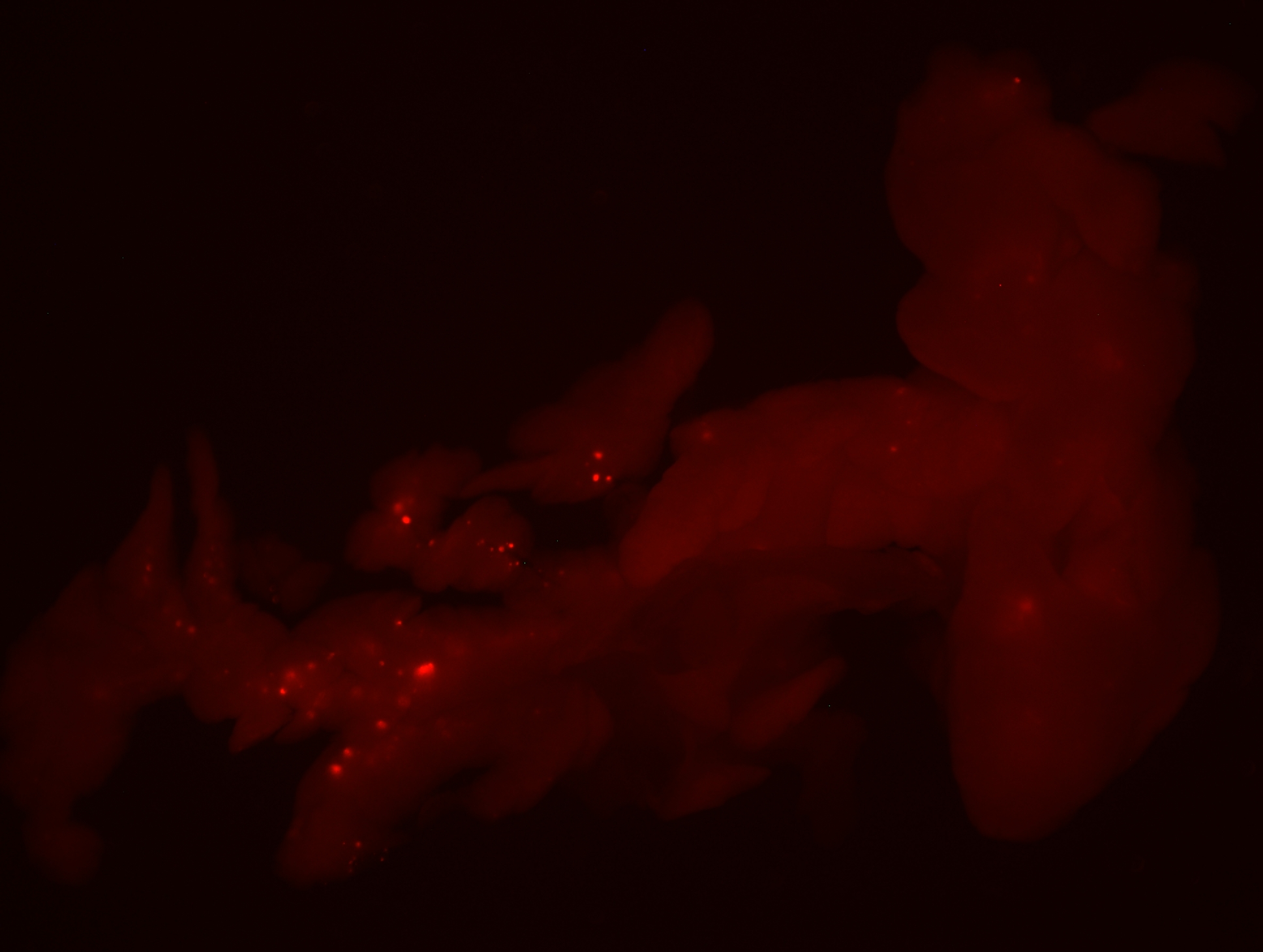

Supplement: Supplementary file 3 — Source data Fig. 1 [file 44318_2025_434_MOESM3_ESM.zip › Figure 1/1C/1C_2w_tdT.tif]

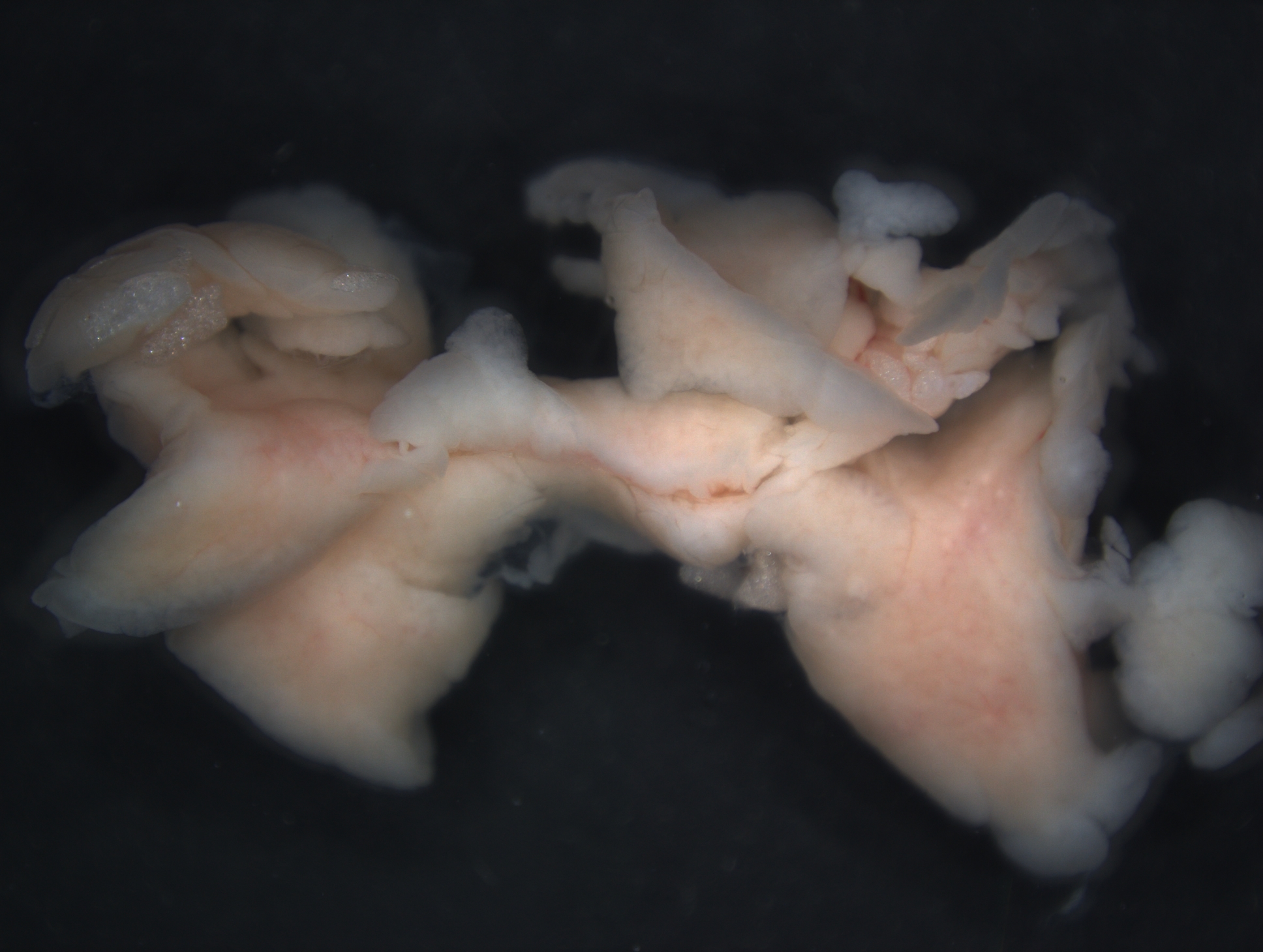

Supplement: Supplementary file 3 — Source data Fig. 1 [file 44318_2025_434_MOESM3_ESM.zip › Figure 1/1J/1J_12w_BF.tif]

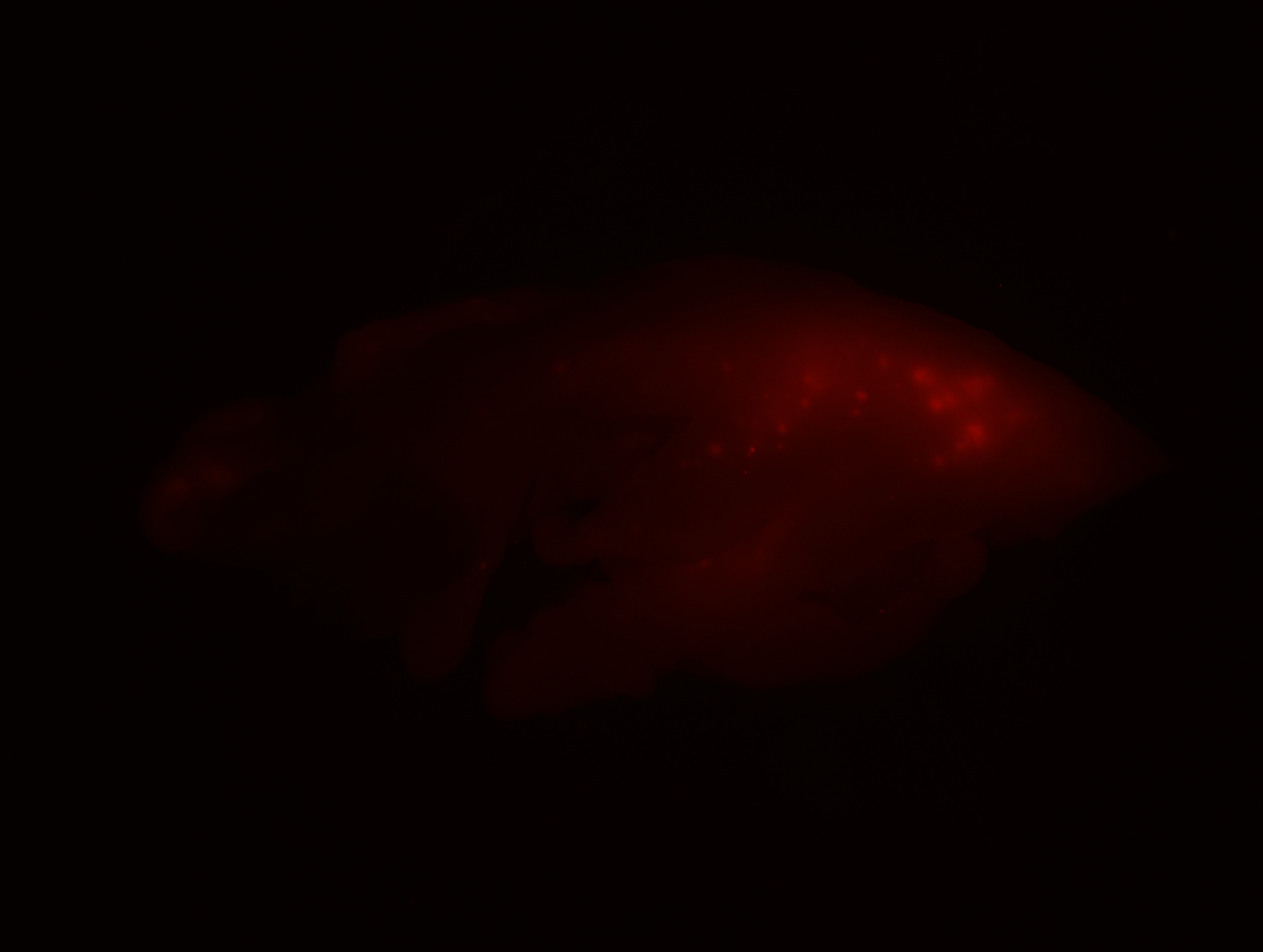

Supplement: Supplementary file 3 — Source data Fig. 1 [file 44318_2025_434_MOESM3_ESM.zip › Figure 1/1J/1J_2w_tdT.tif]

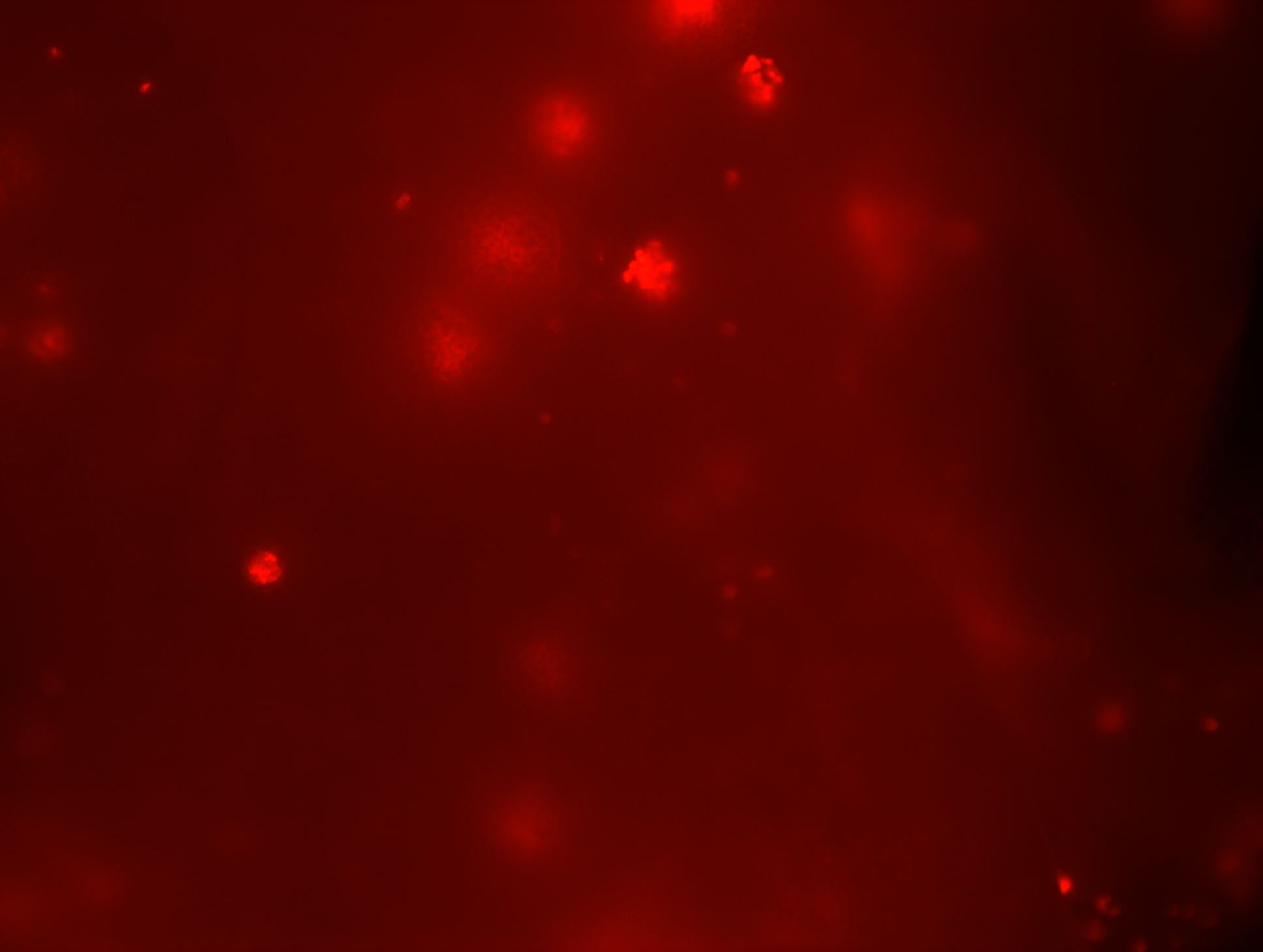

Supplement: Supplementary file 3 — Source data Fig. 1 [file 44318_2025_434_MOESM3_ESM.zip › Figure 1/1J/1J_12w_tdT_mag.tif]

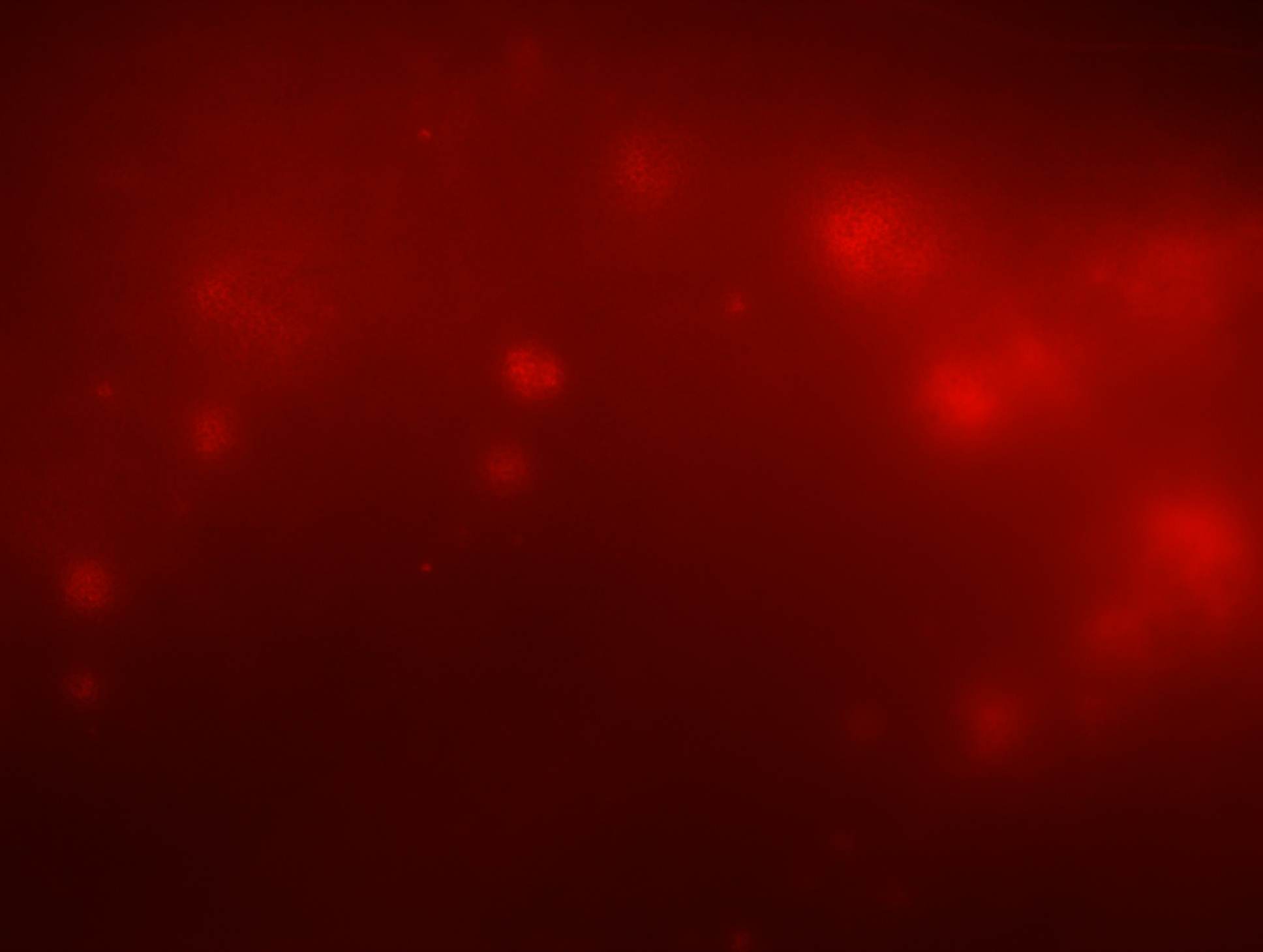

Supplement: Supplementary file 3 — Source data Fig. 1 [file 44318_2025_434_MOESM3_ESM.zip › Figure 1/1J/1J_2w_tdT_mag.tif]

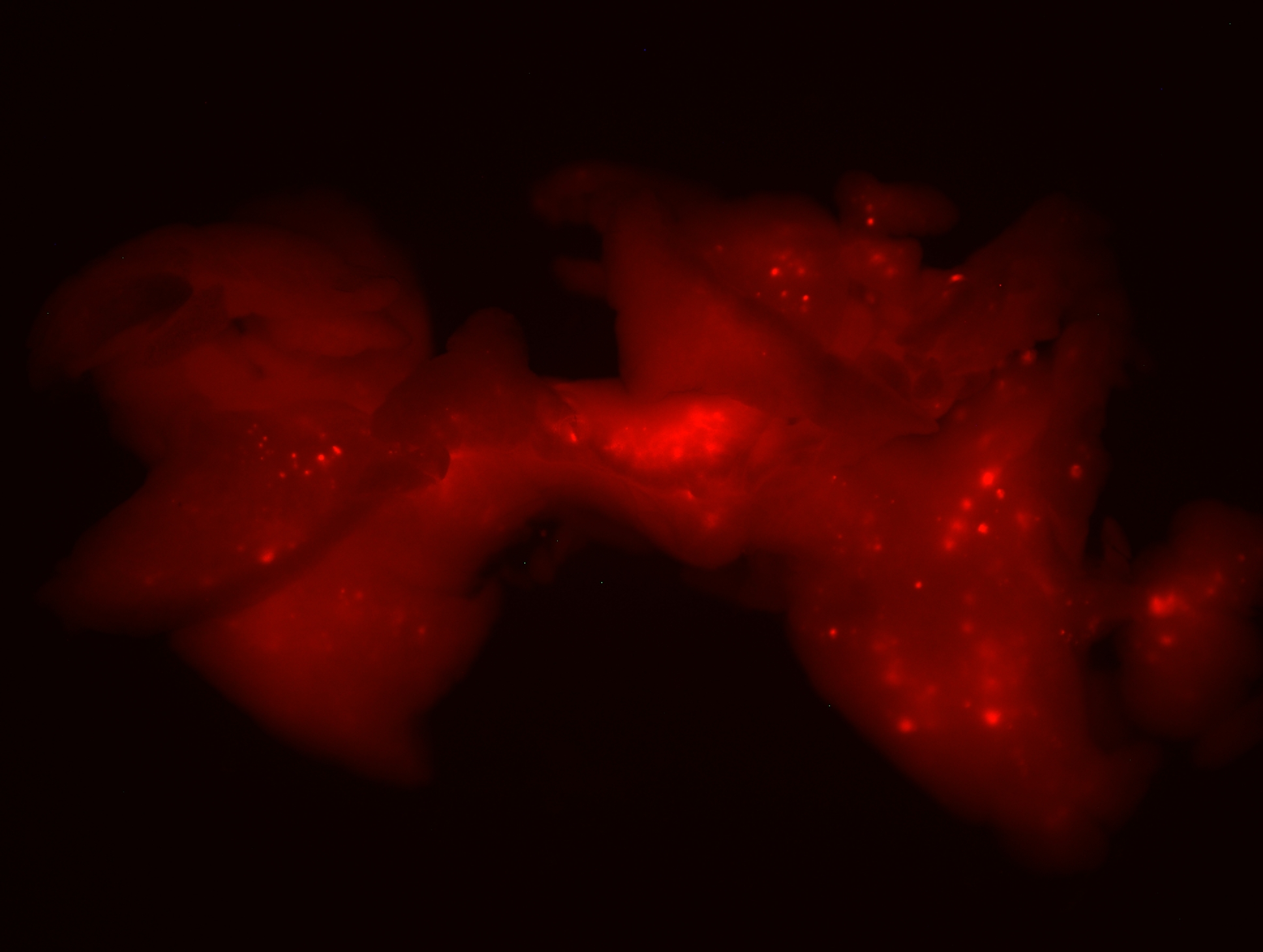

Supplement: Supplementary file 3 — Source data Fig. 1 [file 44318_2025_434_MOESM3_ESM.zip › Figure 1/1J/1J_12w_tdT.tif]

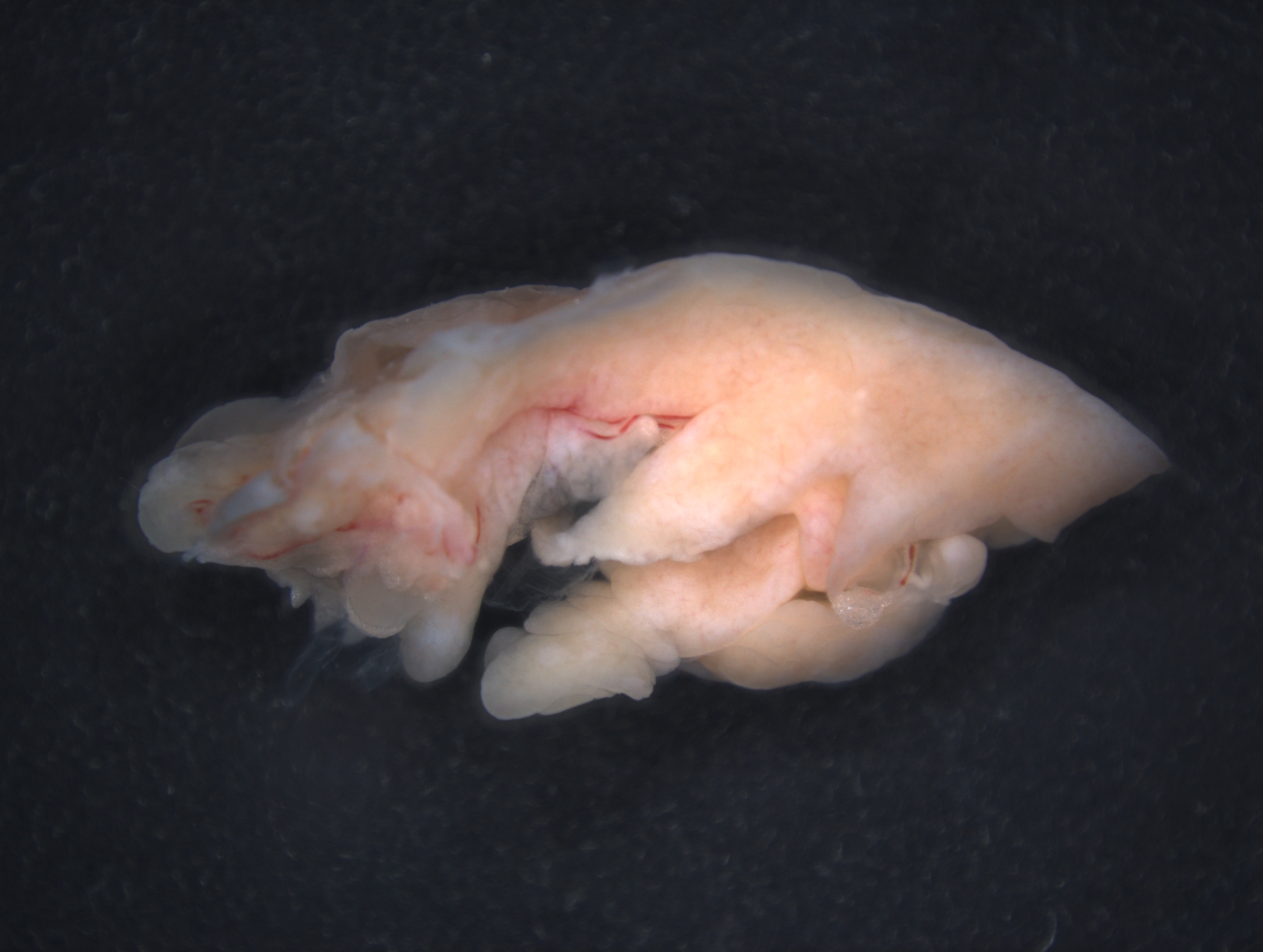

Supplement: Supplementary file 3 — Source data Fig. 1 [file 44318_2025_434_MOESM3_ESM.zip › Figure 1/1J/1J_2w_BF.tif]

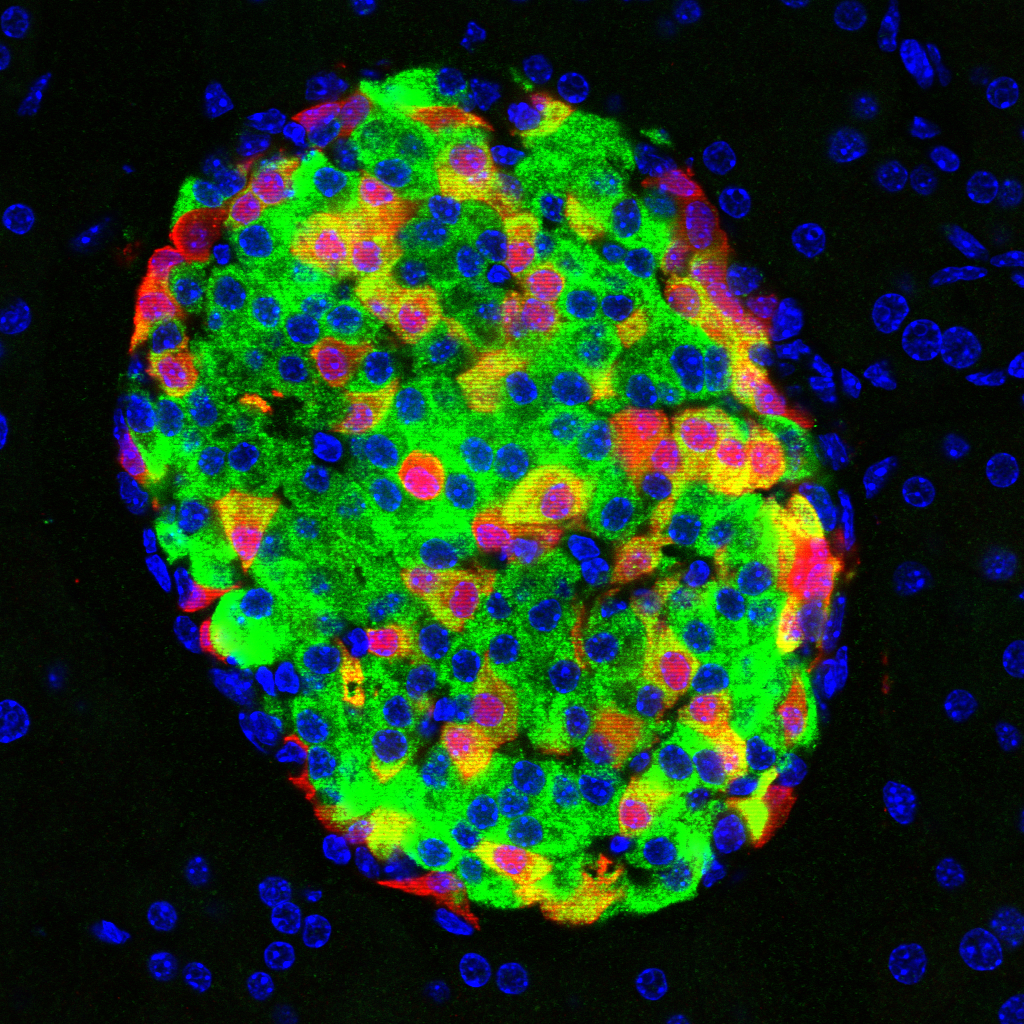

Supplement: Supplementary file 3 — Source data Fig. 1 [file 44318_2025_434_MOESM3_ESM.zip › Figure 1/1M/1M_12w_Ins.tif]

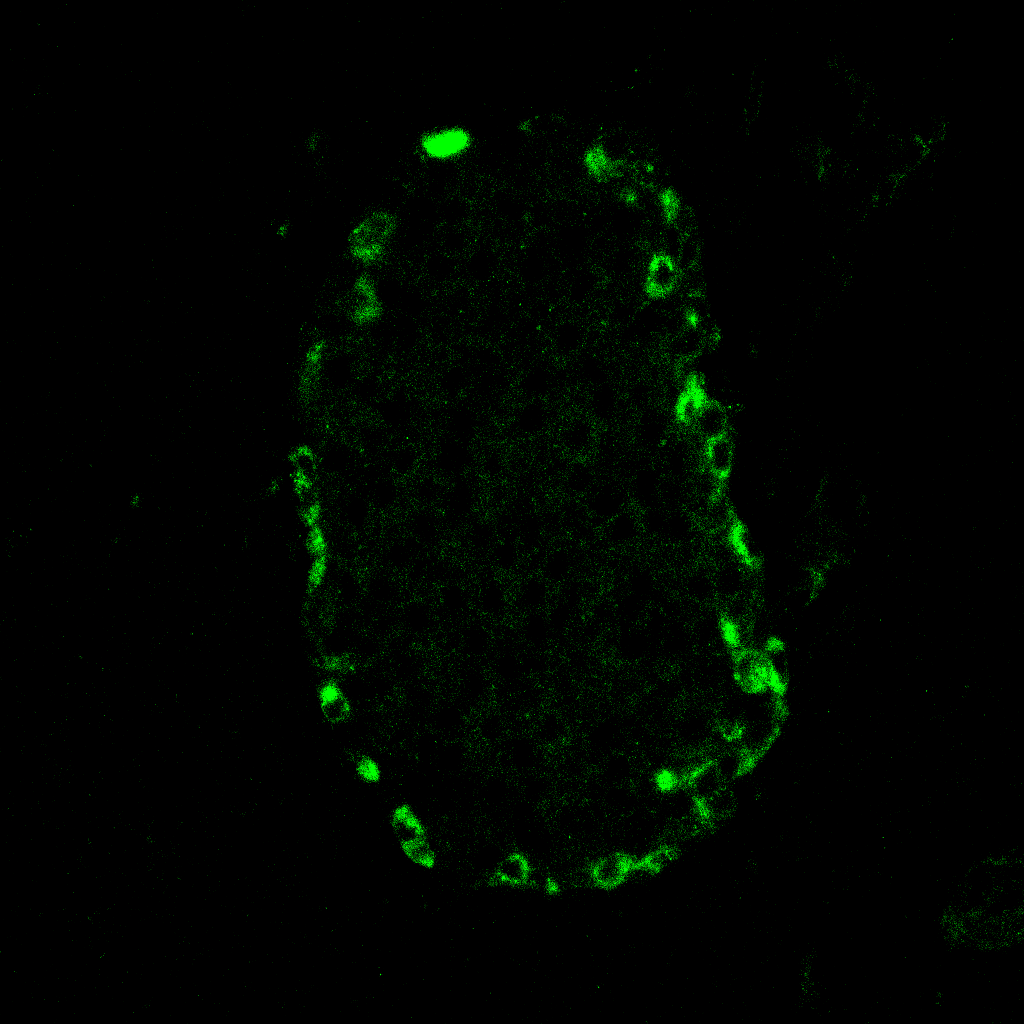

Supplement: Supplementary file 3 — Source data Fig. 1 [file 44318_2025_434_MOESM3_ESM.zip › Figure 1/1M/1M_2w_Ppy (green).tif]

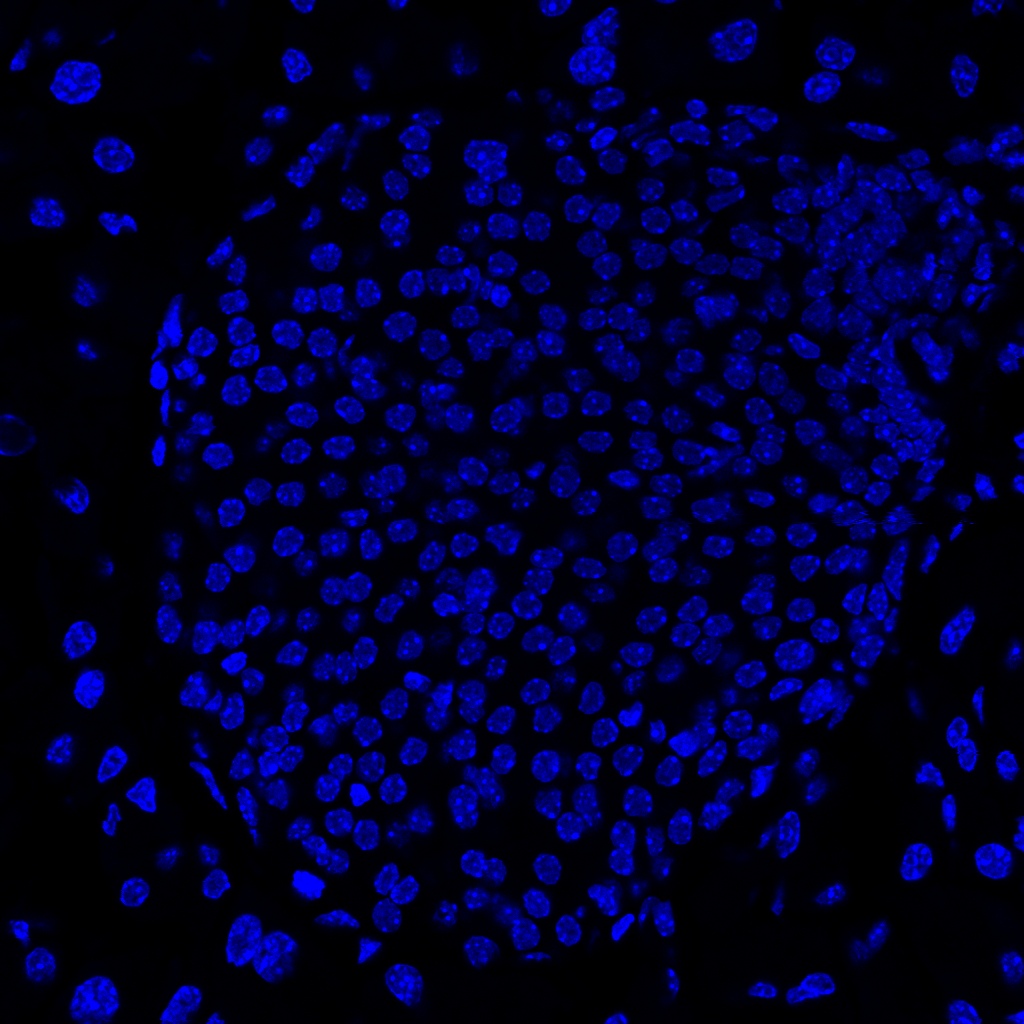

Supplement: Supplementary file 3 — Source data Fig. 1 [file 44318_2025_434_MOESM3_ESM.zip › Figure 1/1M/1M_2w_Sst (blue).tif]

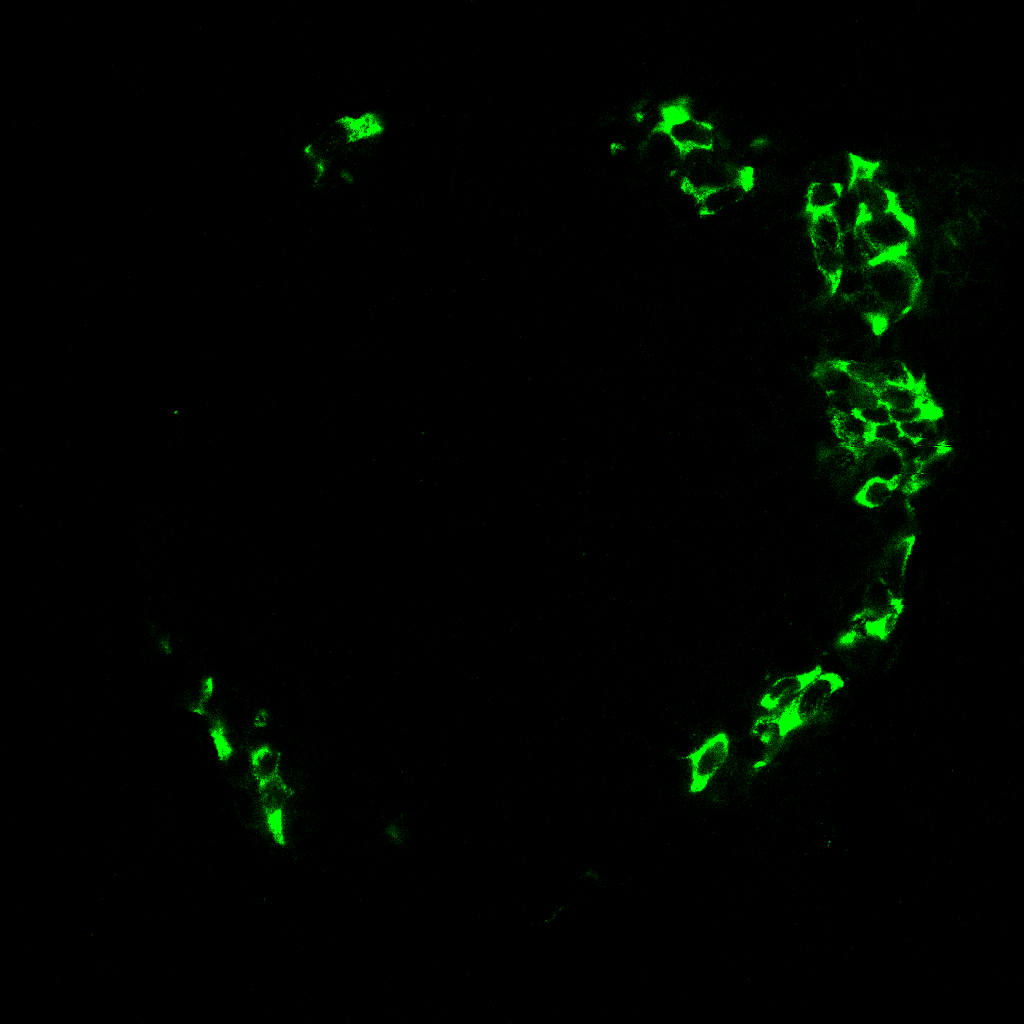

Supplement: Supplementary file 3 — Source data Fig. 1 [file 44318_2025_434_MOESM3_ESM.zip › Figure 1/1M/1M_2w_Sst (green).tif]

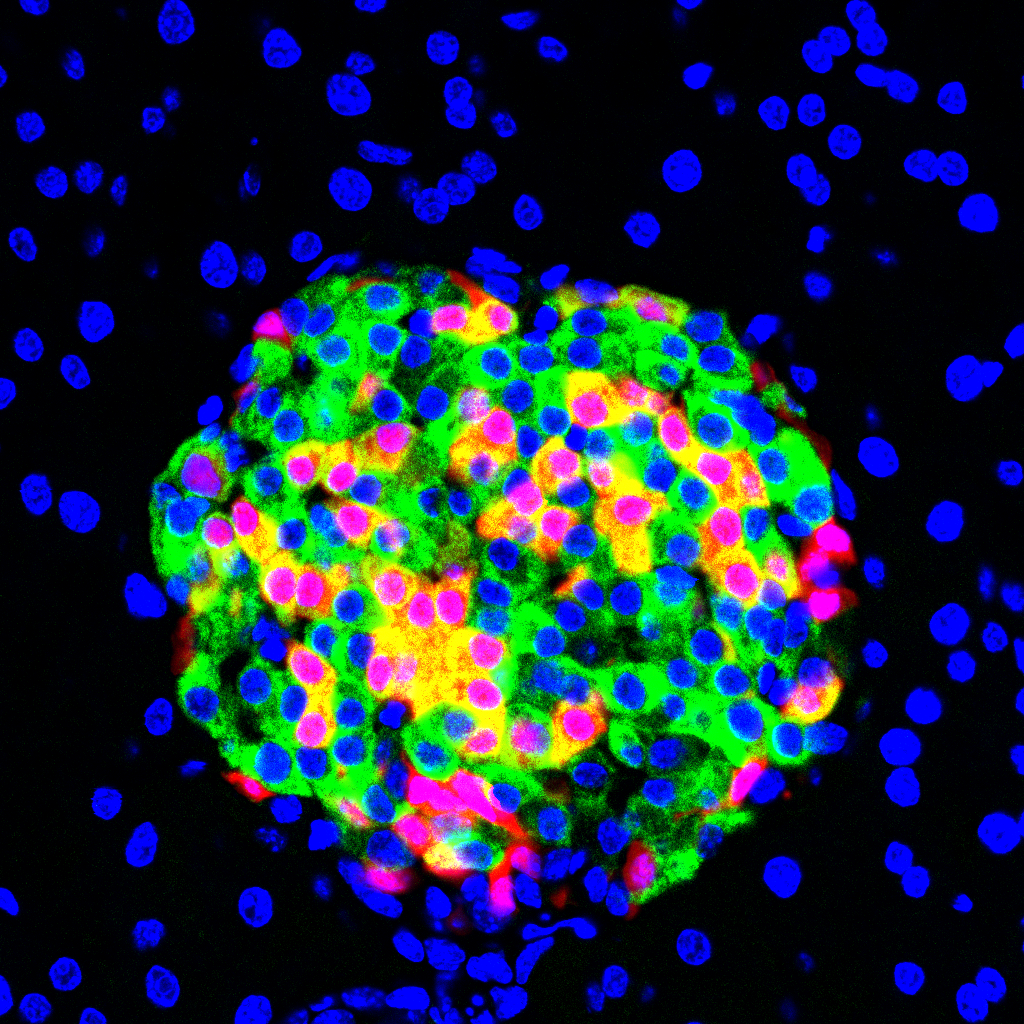

Supplement: Supplementary file 3 — Source data Fig. 1 [file 44318_2025_434_MOESM3_ESM.zip › Figure 1/1M/1M_2w_Ins.tif]

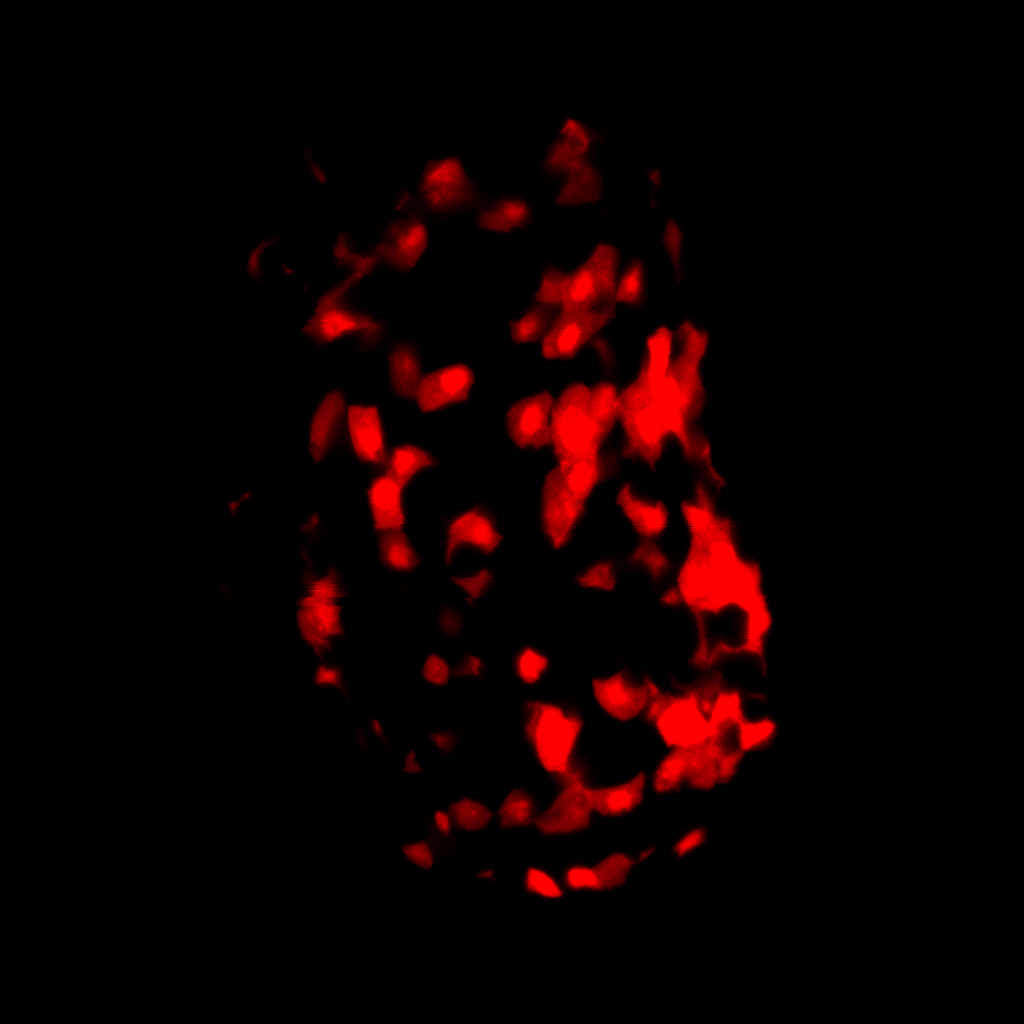

Supplement: Supplementary file 3 — Source data Fig. 1 [file 44318_2025_434_MOESM3_ESM.zip › Figure 1/1M/1M_2w_Ppy (red).tif]

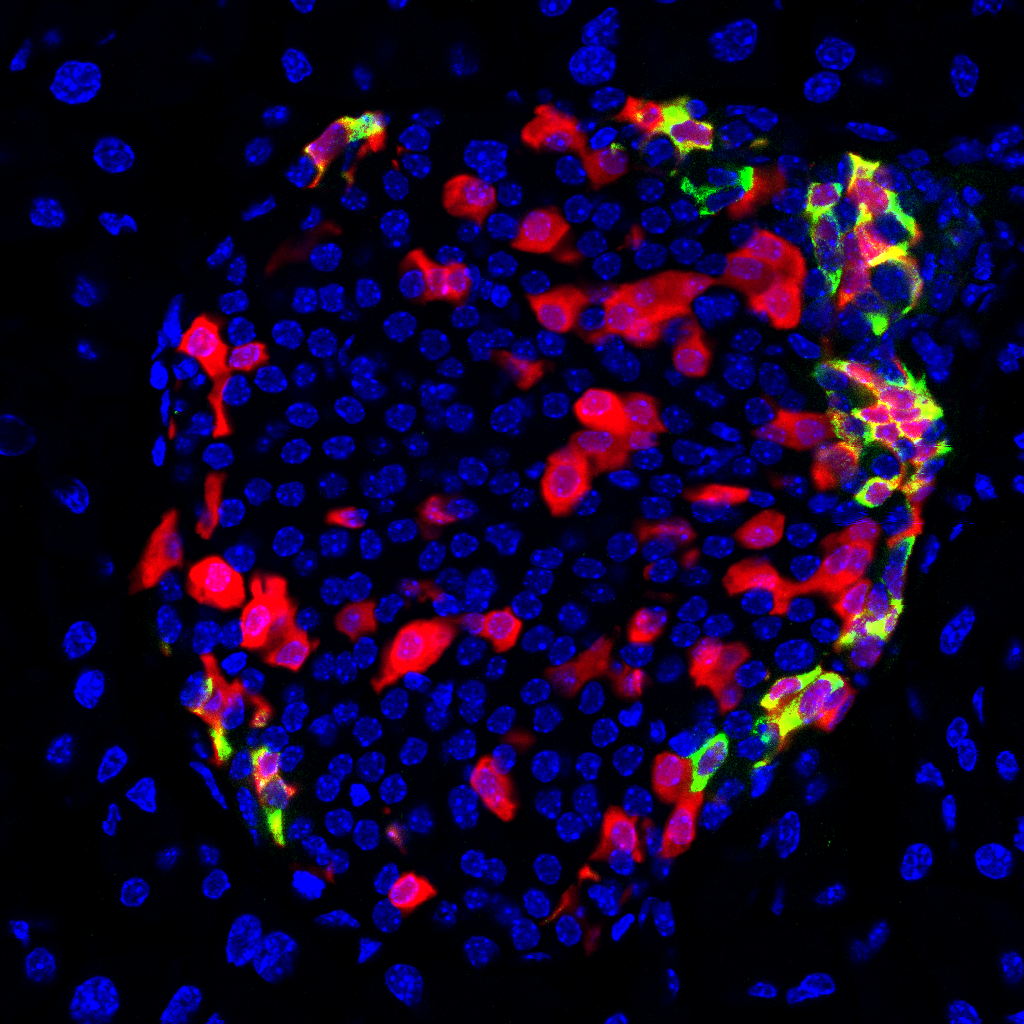

Supplement: Supplementary file 3 — Source data Fig. 1 [file 44318_2025_434_MOESM3_ESM.zip › Figure 1/1M/1M_2w_Sst.tif]

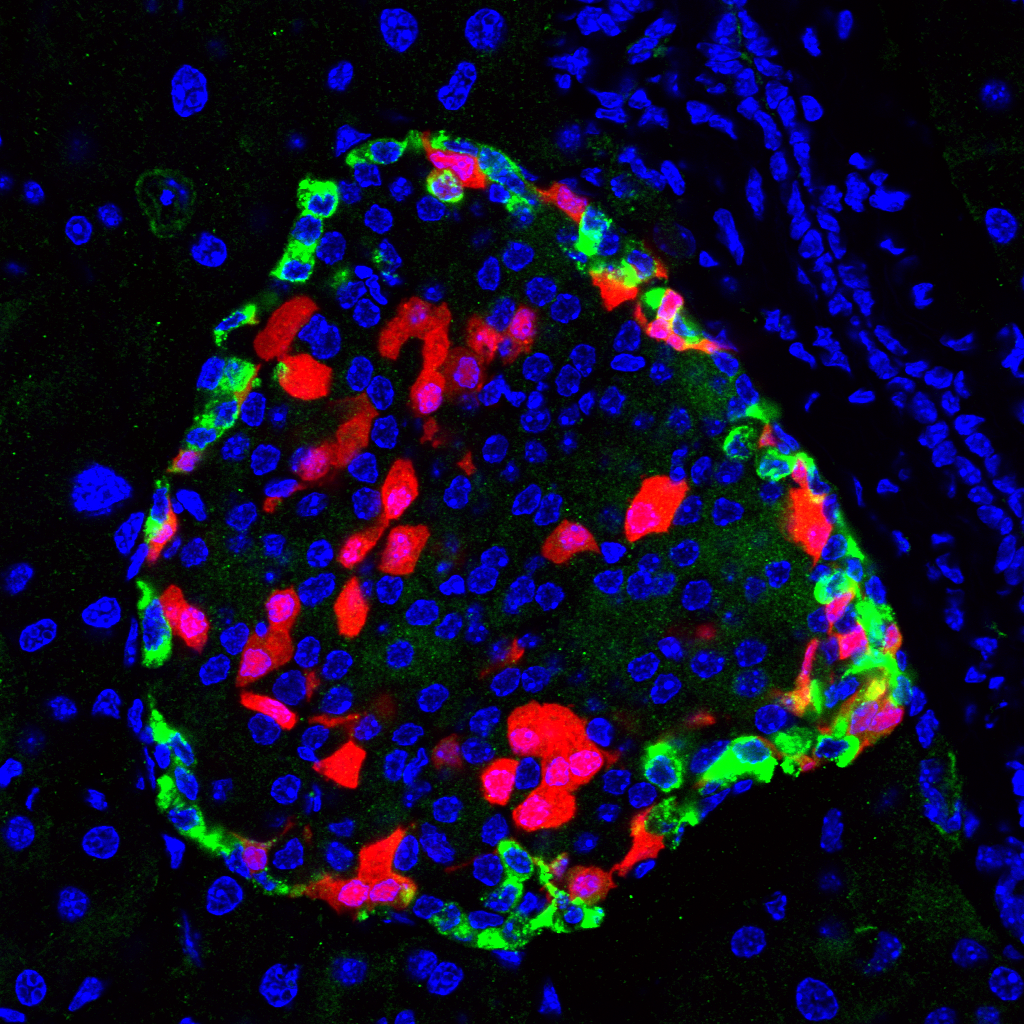

Supplement: Supplementary file 3 — Source data Fig. 1 [file 44318_2025_434_MOESM3_ESM.zip › Figure 1/1M/1M_12w_Ppy.tif]

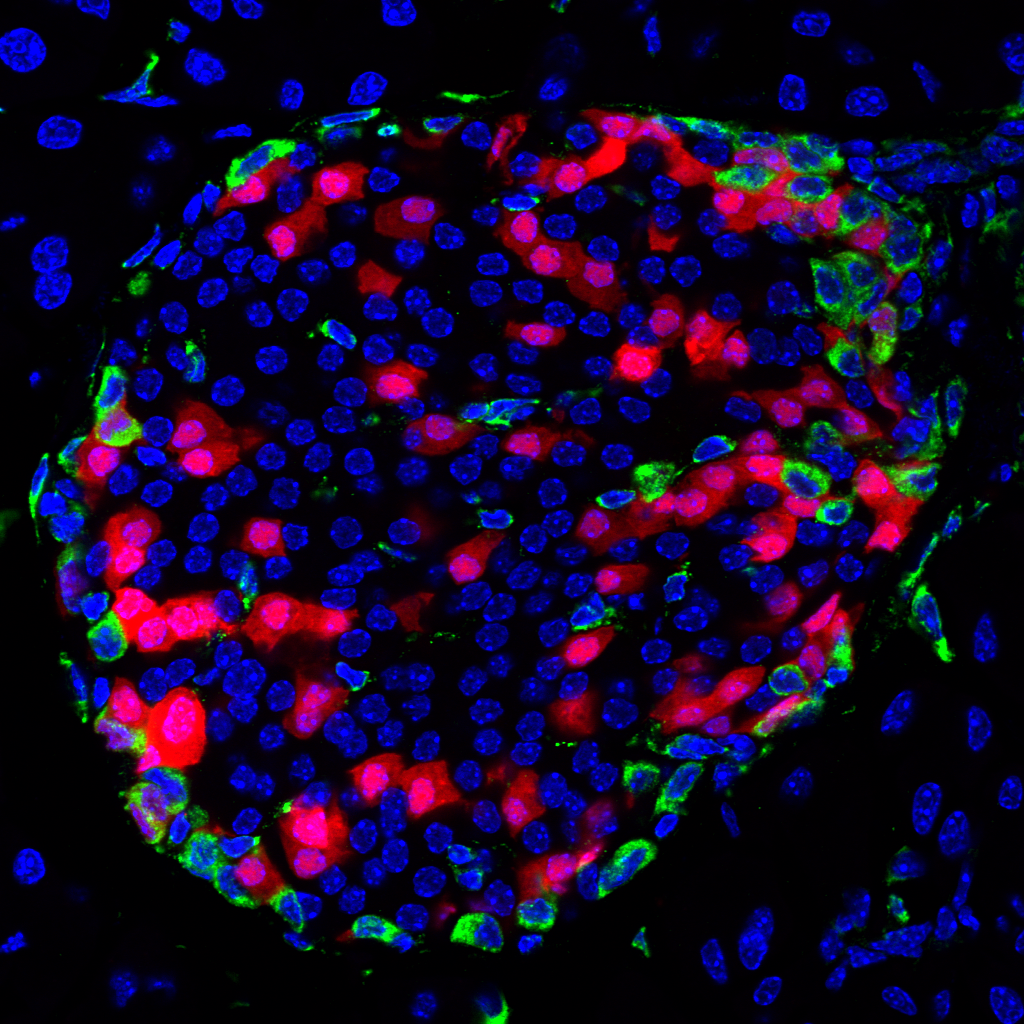

Supplement: Supplementary file 3 — Source data Fig. 1 [file 44318_2025_434_MOESM3_ESM.zip › Figure 1/1M/1M_2w_Gcg.tif]

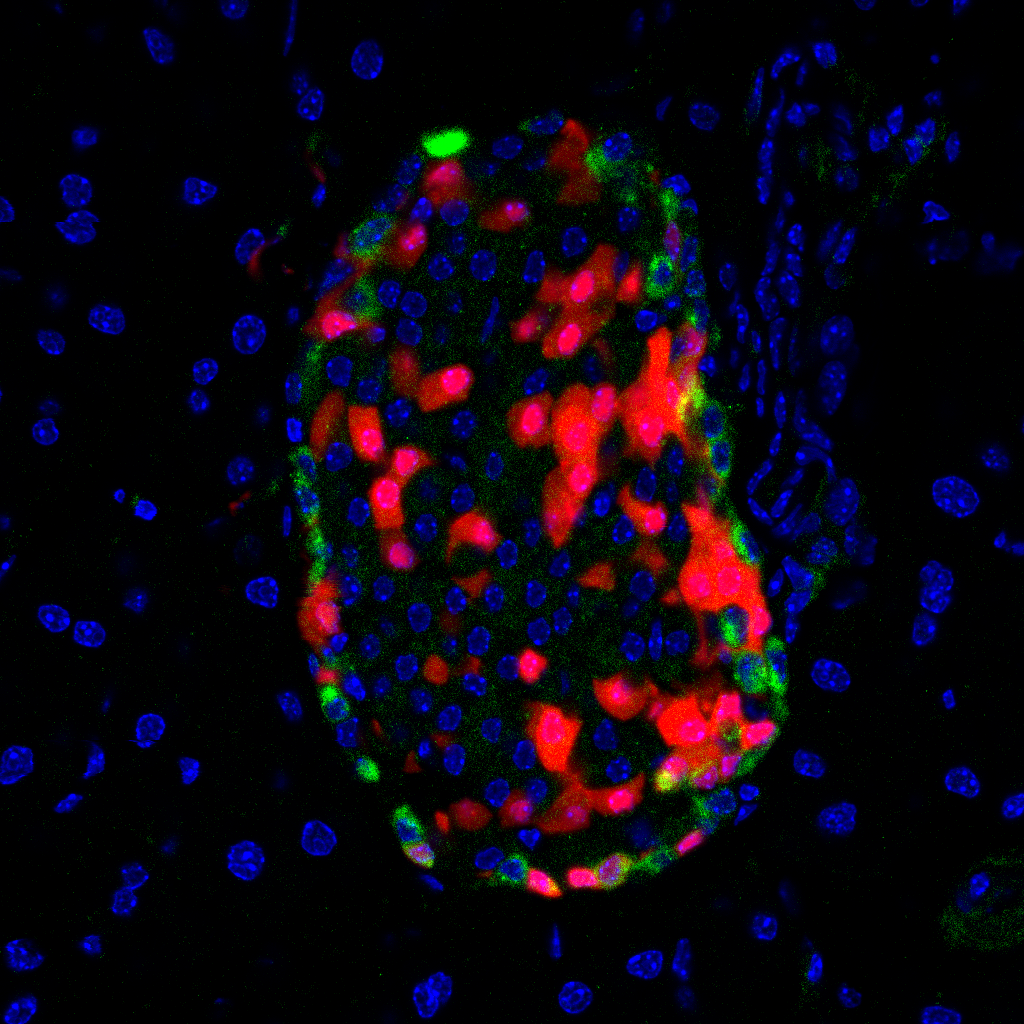

Supplement: Supplementary file 3 — Source data Fig. 1 [file 44318_2025_434_MOESM3_ESM.zip › Figure 1/1M/1M_2w_Ppy.tif]

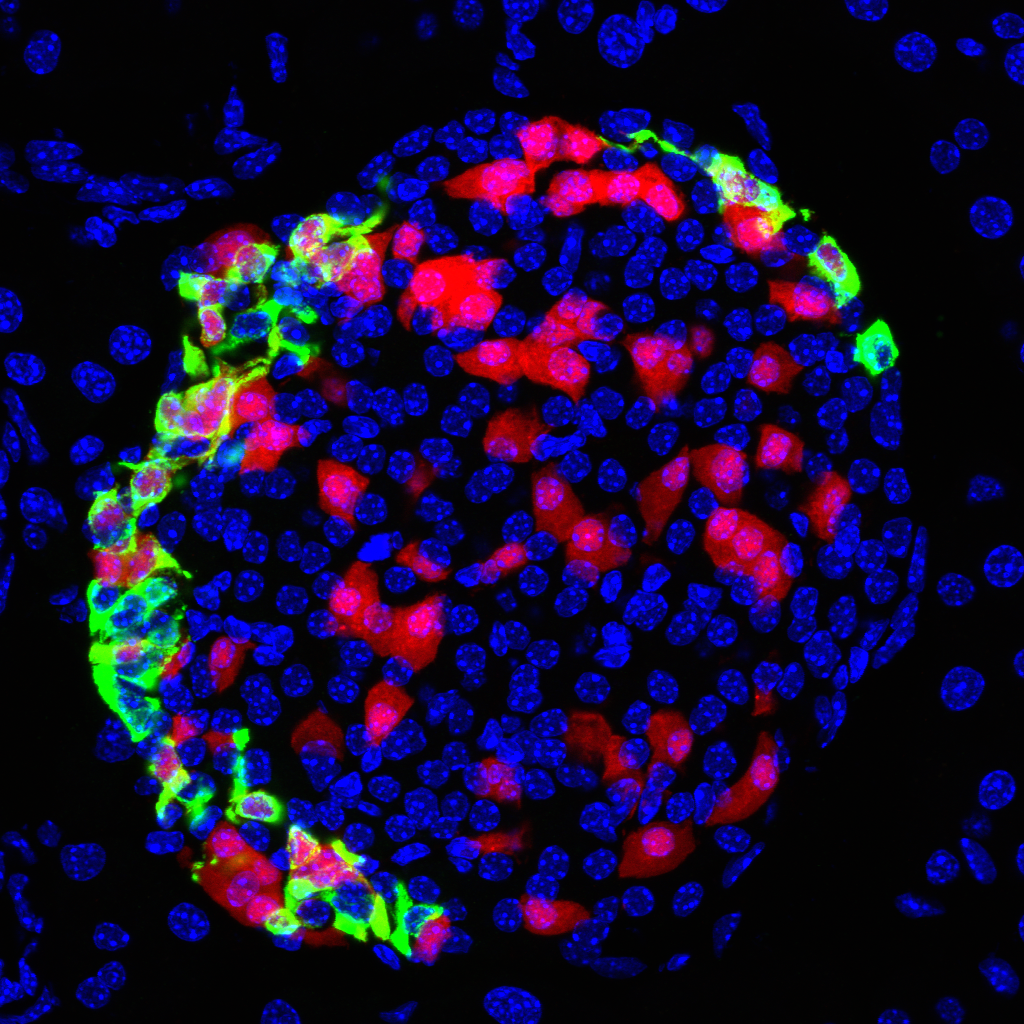

Supplement: Supplementary file 3 — Source data Fig. 1 [file 44318_2025_434_MOESM3_ESM.zip › Figure 1/1M/1M_12w_Sst.tif]

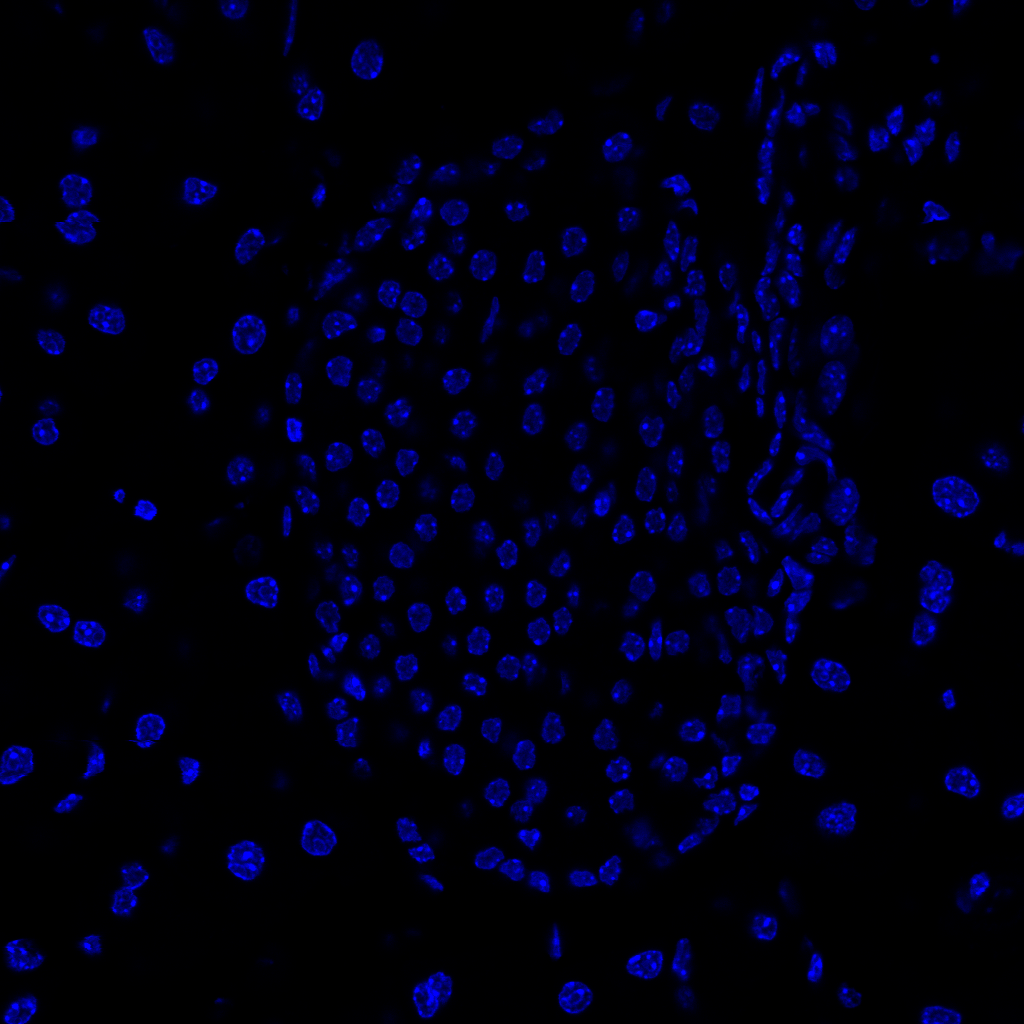

Supplement: Supplementary file 3 — Source data Fig. 1 [file 44318_2025_434_MOESM3_ESM.zip › Figure 1/1M/1M_2w_Ppy (blue).tif]

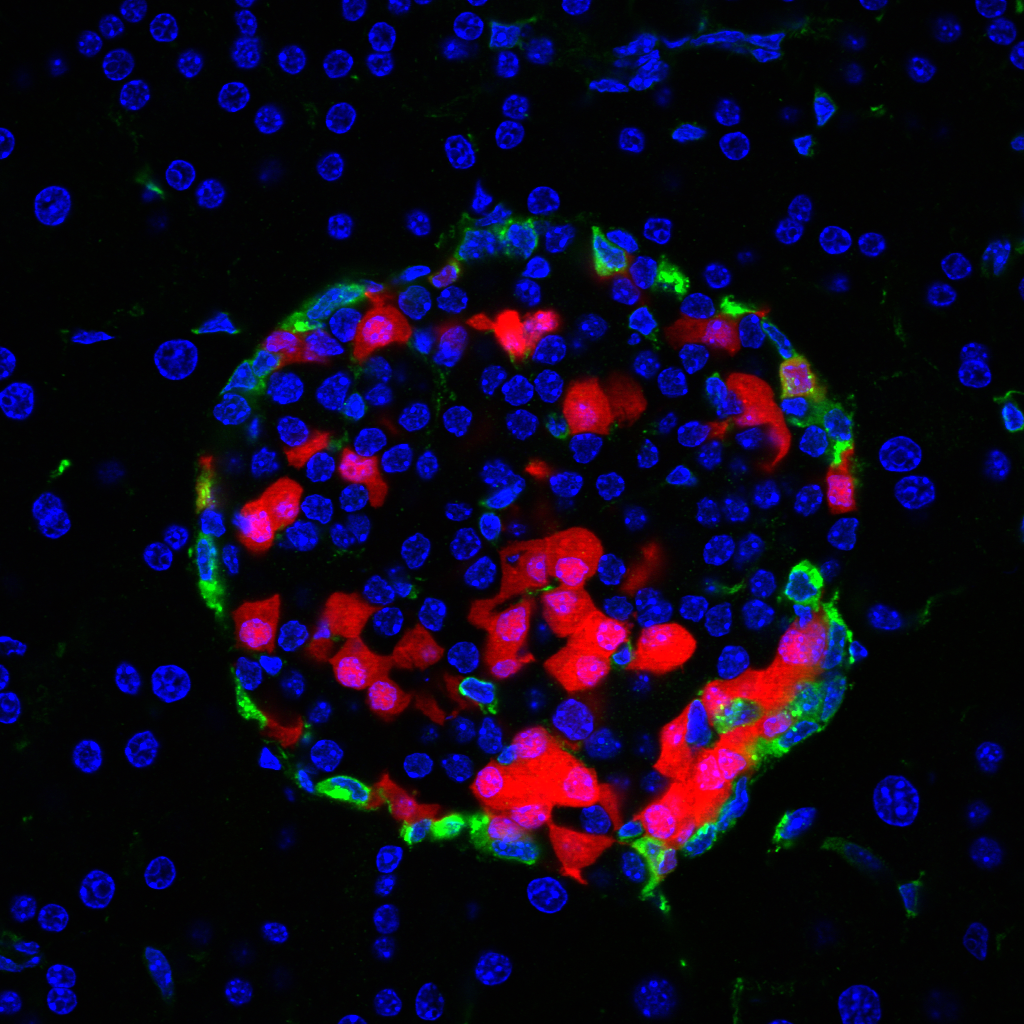

Supplement: Supplementary file 3 — Source data Fig. 1 [file 44318_2025_434_MOESM3_ESM.zip › Figure 1/1M/1M_12w_Gcg.tif]

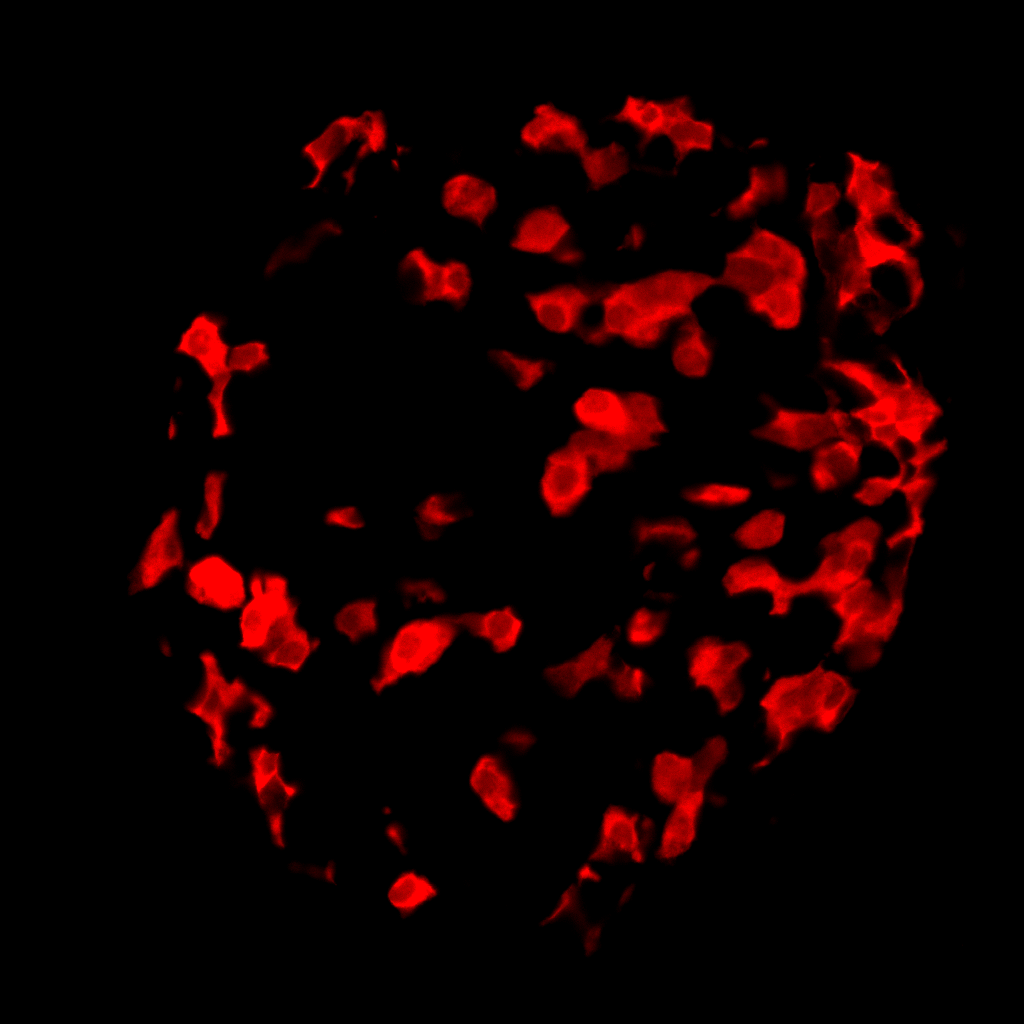

Supplement: Supplementary file 3 — Source data Fig. 1 [file 44318_2025_434_MOESM3_ESM.zip › Figure 1/1M/1M_2w_Sst (red).tif]

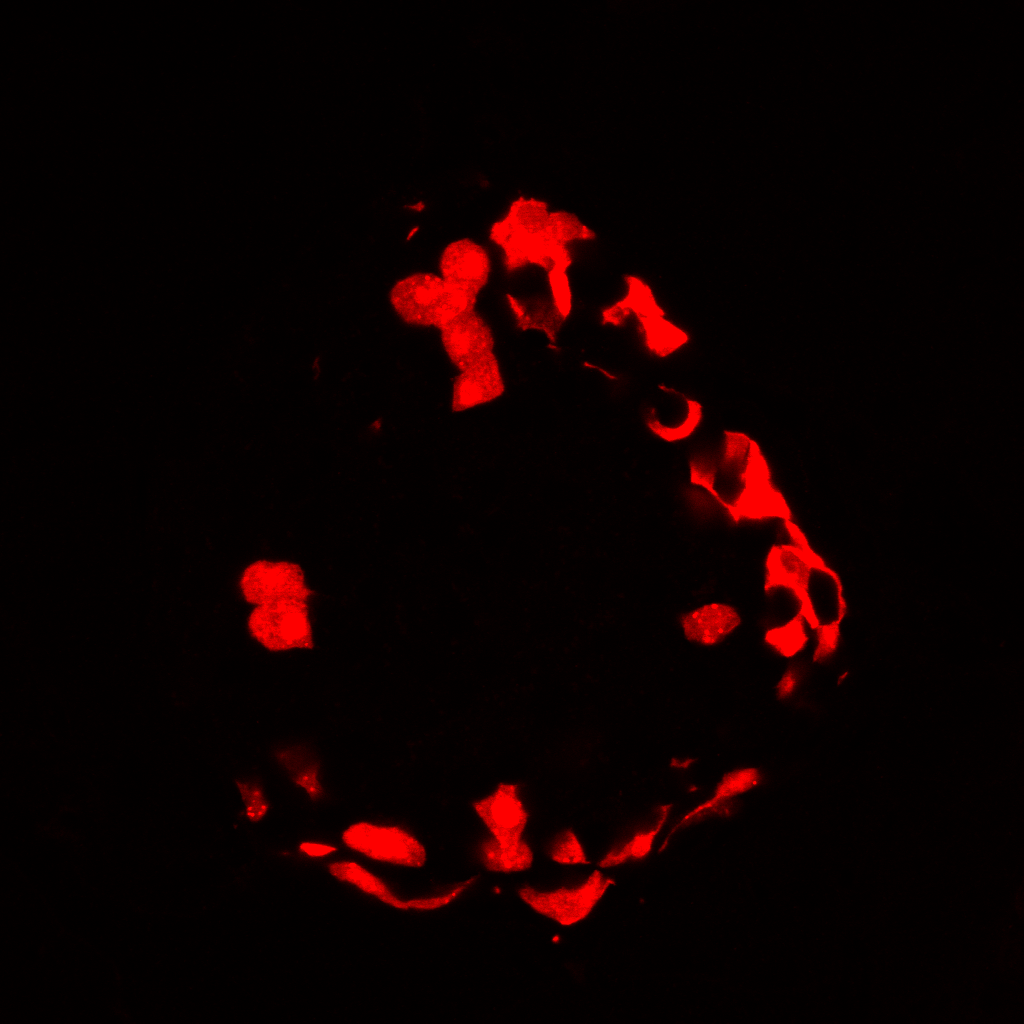

Supplement: Supplementary file 4 — Source data Fig. 2 [file 44318_2025_434_MOESM4_ESM.zip › Figure 2/2G/2G_Merge (red).tif]

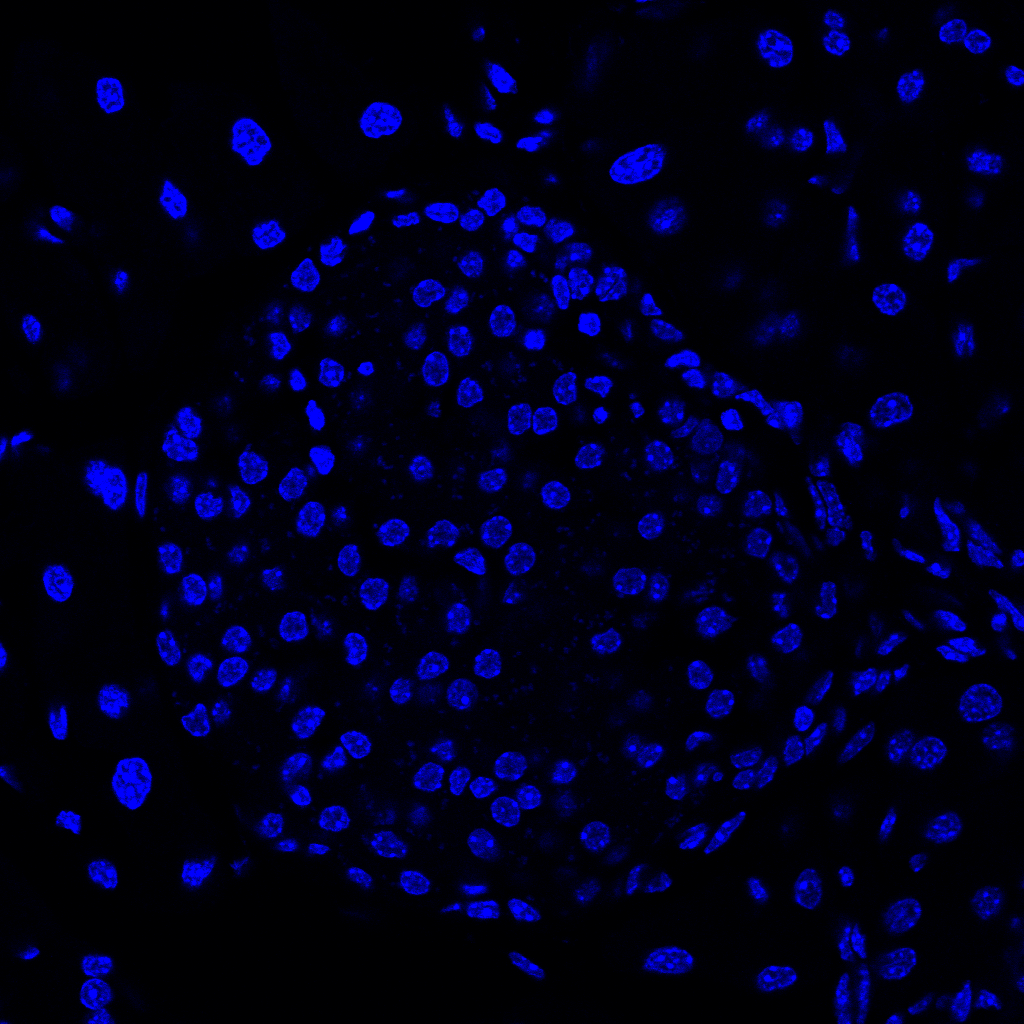

Supplement: Supplementary file 4 — Source data Fig. 2 [file 44318_2025_434_MOESM4_ESM.zip › Figure 2/2G/2G_Merge (blue).tif]

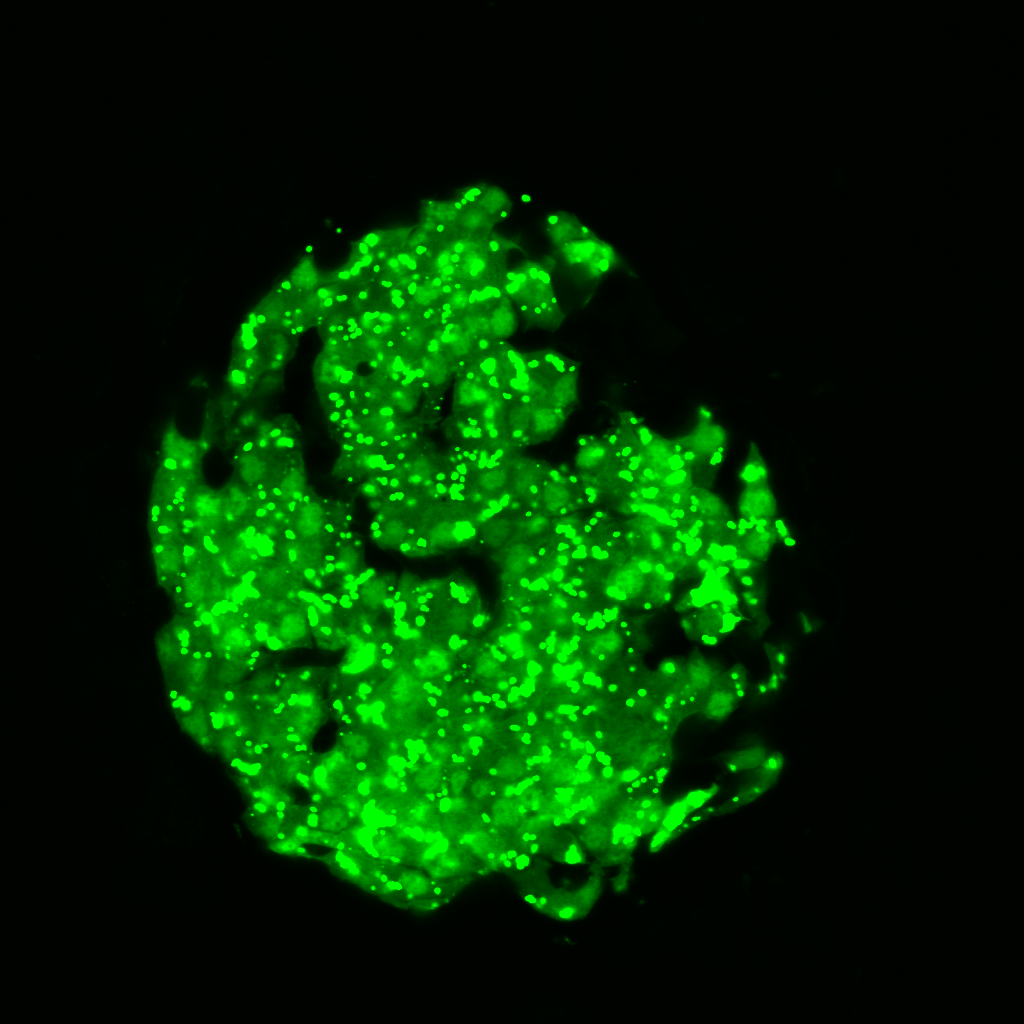

Supplement: Supplementary file 4 — Source data Fig. 2 [file 44318_2025_434_MOESM4_ESM.zip › Figure 2/2G/2G_Merge (green).tif]

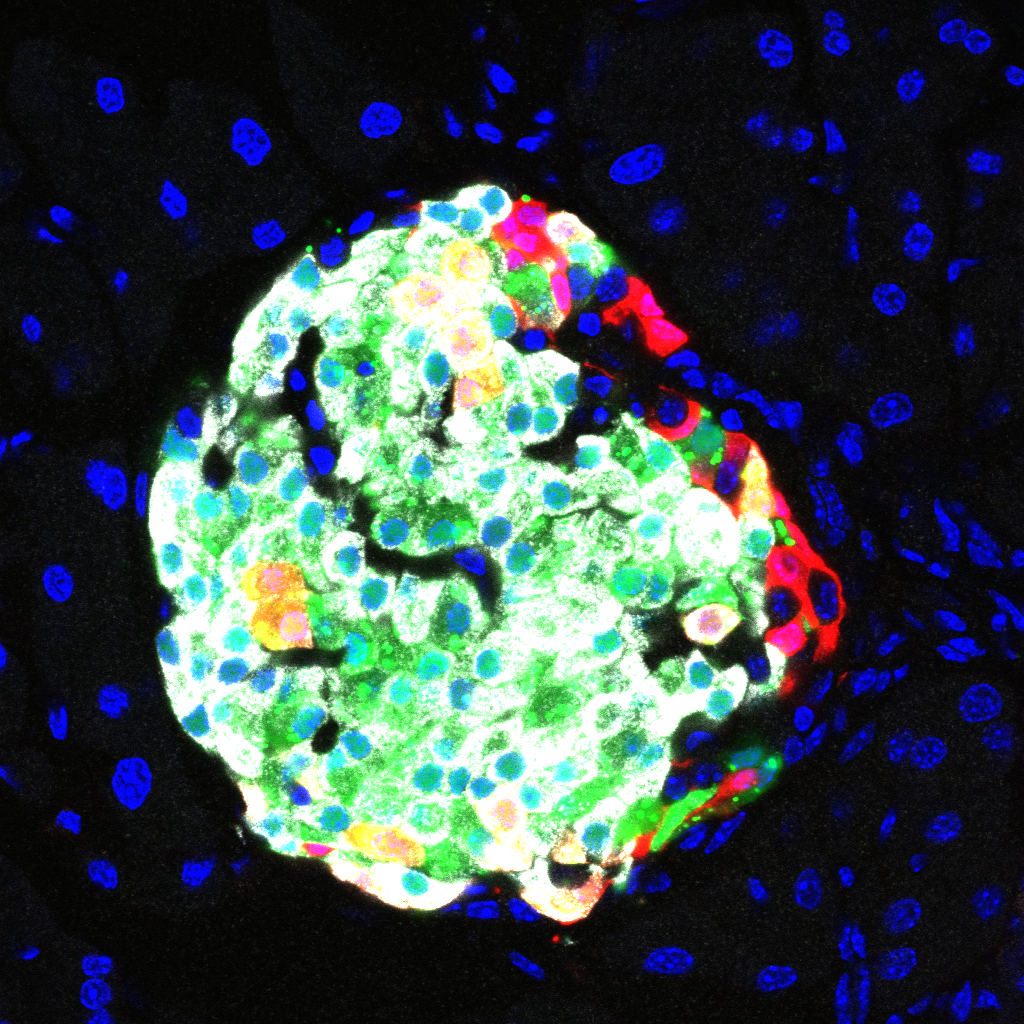

Supplement: Supplementary file 4 — Source data Fig. 2 [file 44318_2025_434_MOESM4_ESM.zip › Figure 2/2G/2G_Merge.tif]

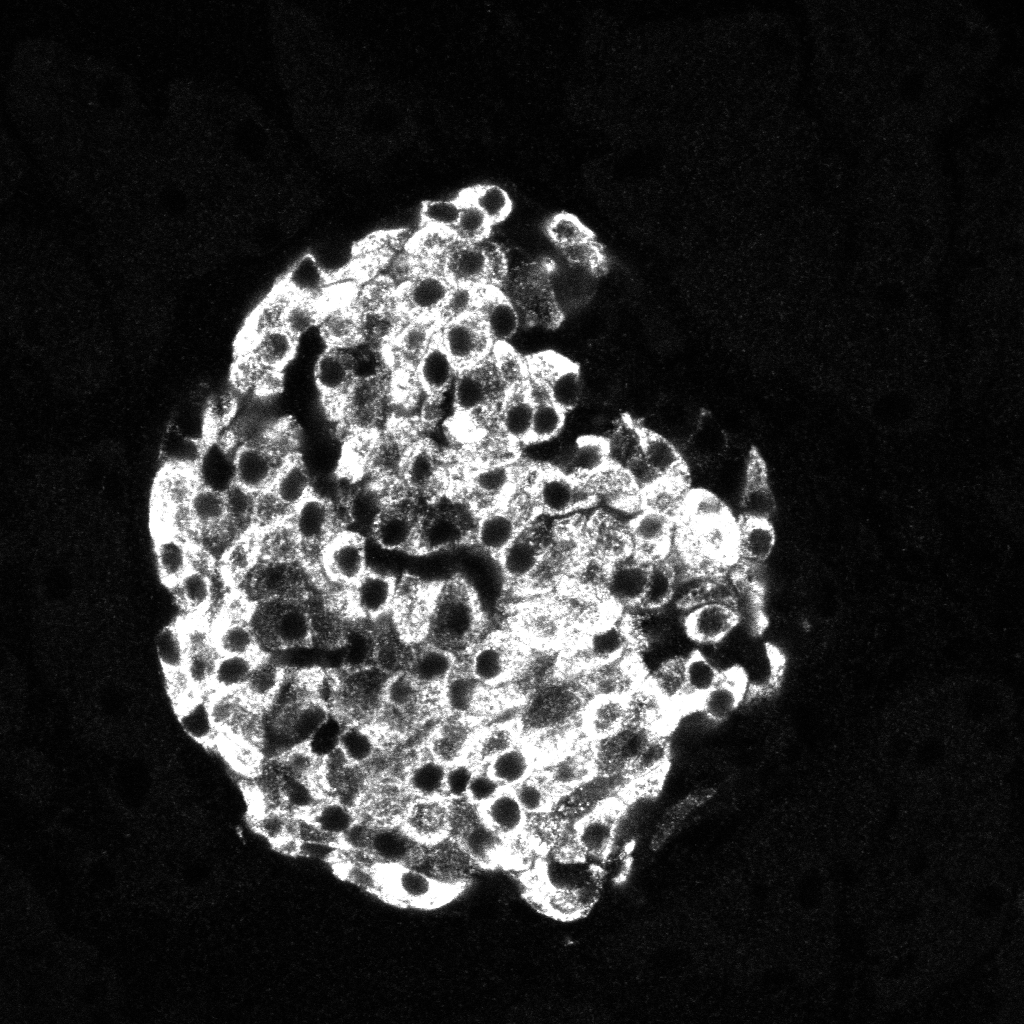

Supplement: Supplementary file 4 — Source data Fig. 2 [file 44318_2025_434_MOESM4_ESM.zip › Figure 2/2G/2G_Merge (gray).tif]

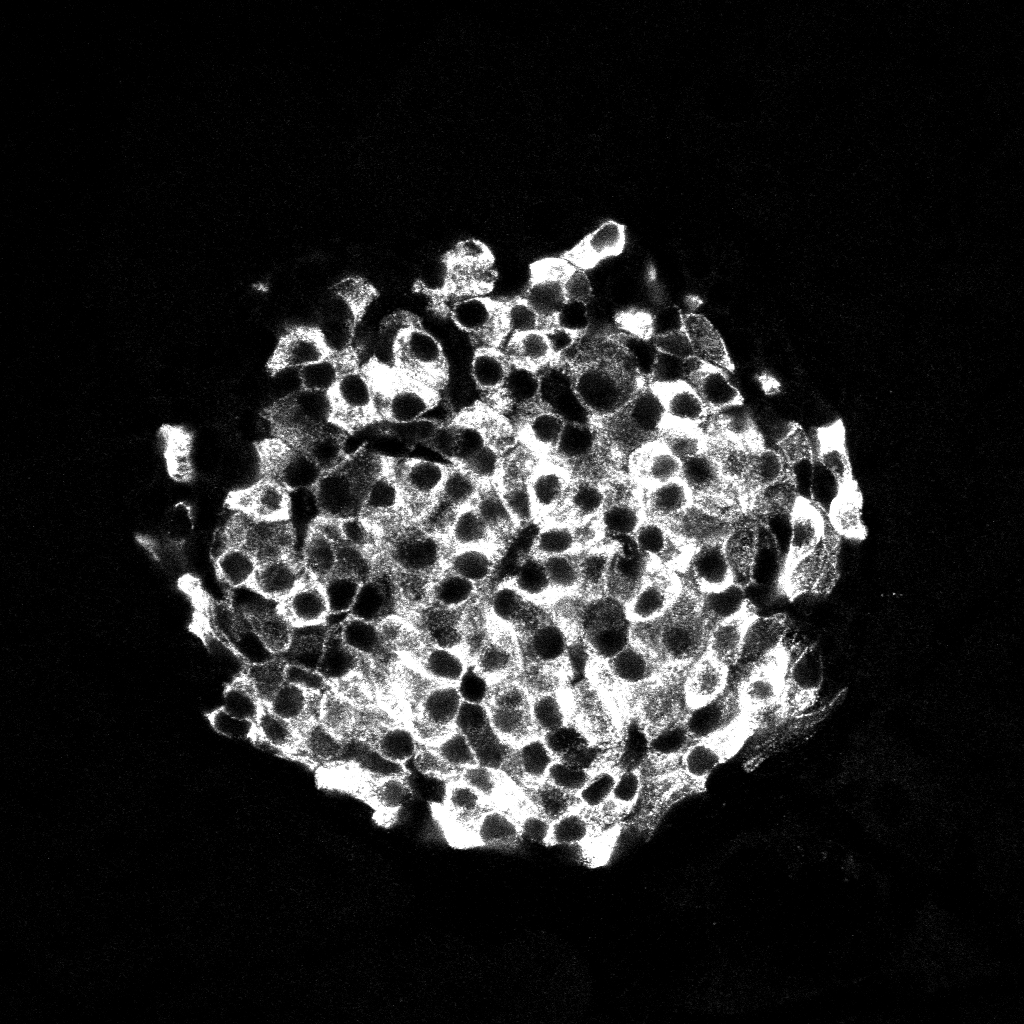

Supplement: Supplementary file 4 — Source data Fig. 2 [file 44318_2025_434_MOESM4_ESM.zip › Figure 2/2F/2F_merge (gray).tif]

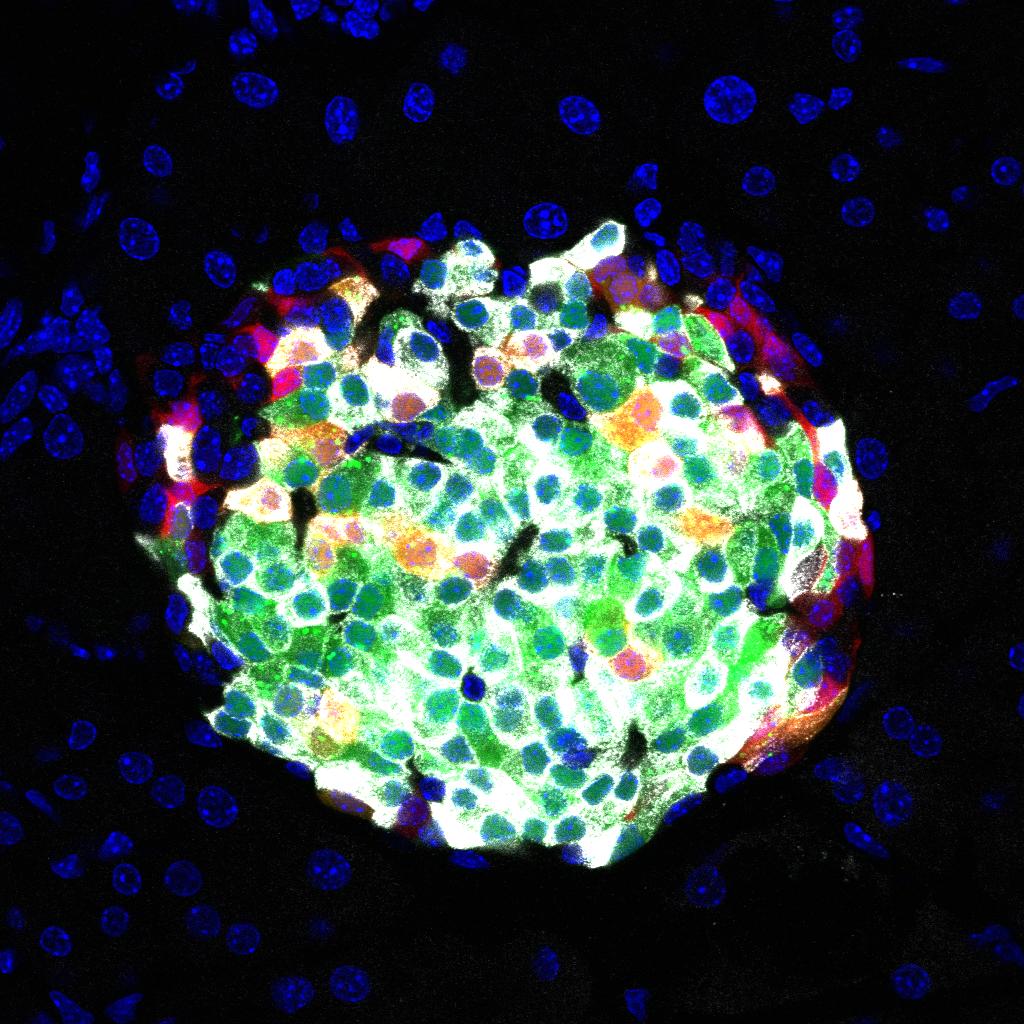

Supplement: Supplementary file 4 — Source data Fig. 2 [file 44318_2025_434_MOESM4_ESM.zip › Figure 2/2F/2F_merge.tif]

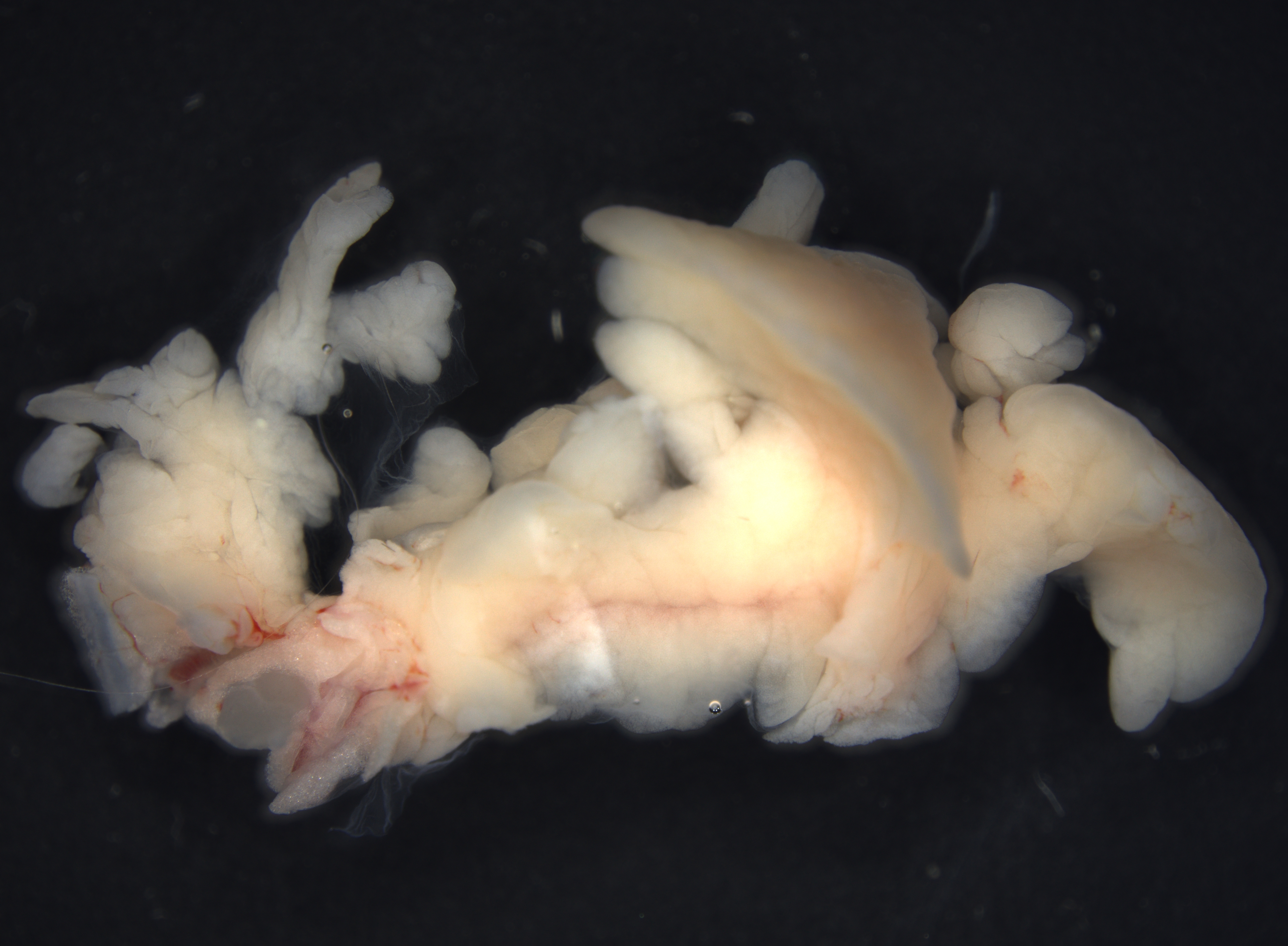

Supplement: Supplementary file 4 — Source data Fig. 2 [file 44318_2025_434_MOESM4_ESM.zip › Figure 2/2C/2C_BF.tif]

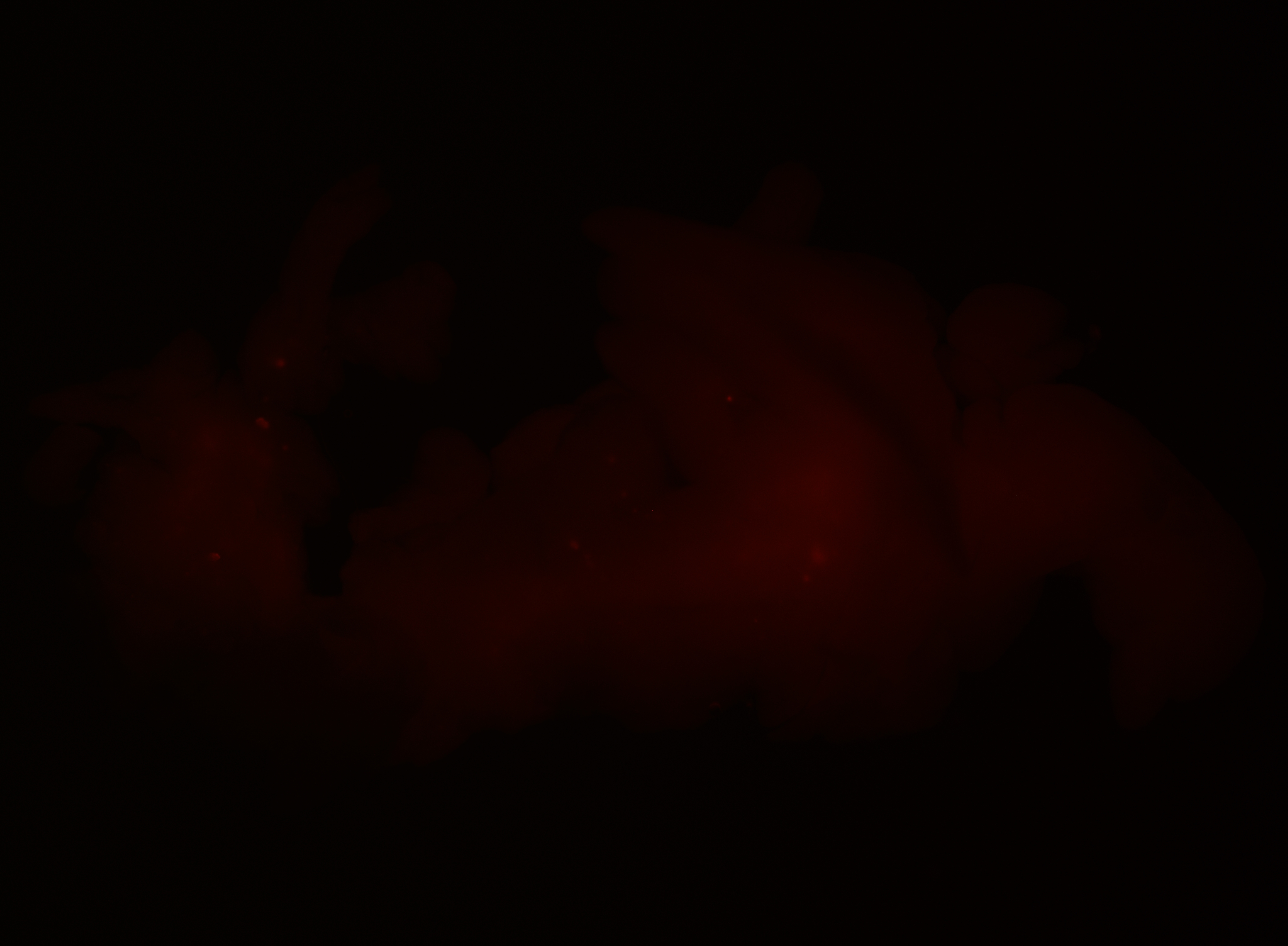

Supplement: Supplementary file 4 — Source data Fig. 2 [file 44318_2025_434_MOESM4_ESM.zip › Figure 2/2C/2C_tdT.tif]

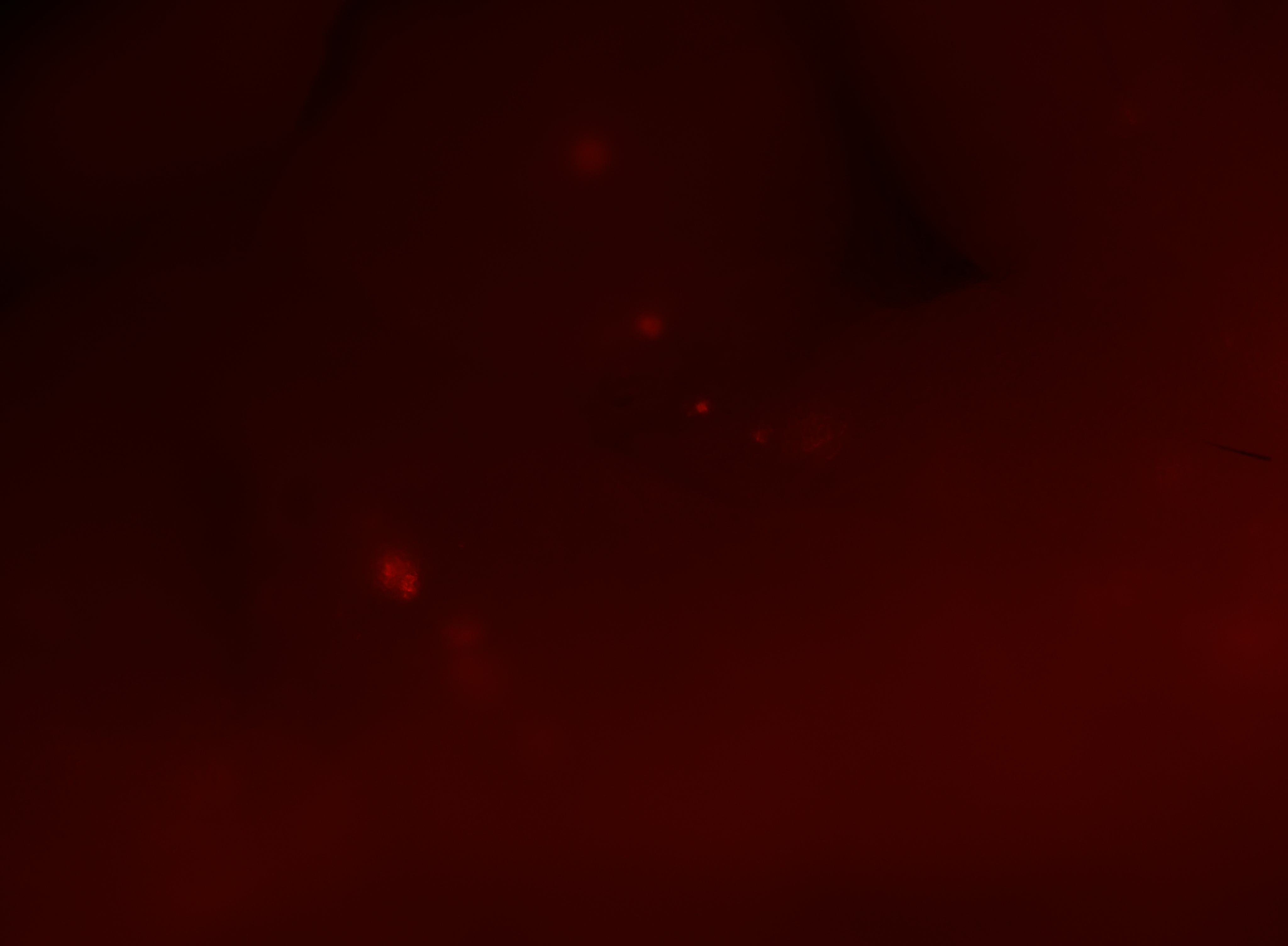

Supplement: Supplementary file 4 — Source data Fig. 2 [file 44318_2025_434_MOESM4_ESM.zip › Figure 2/2C/2C_tdT_mag.tif]

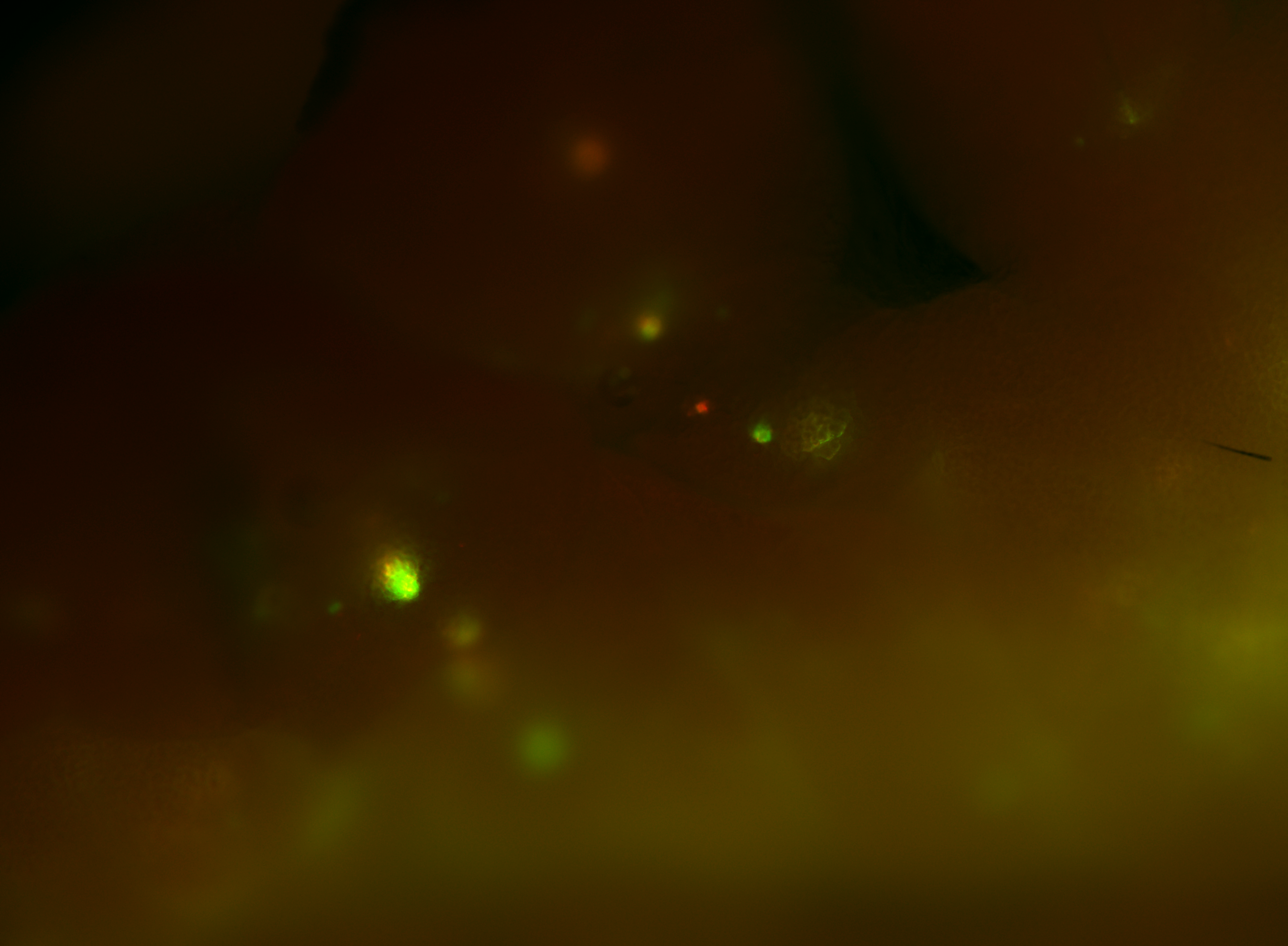

Supplement: Supplementary file 4 — Source data Fig. 2 [file 44318_2025_434_MOESM4_ESM.zip › Figure 2/2C/2C_mag.tif]

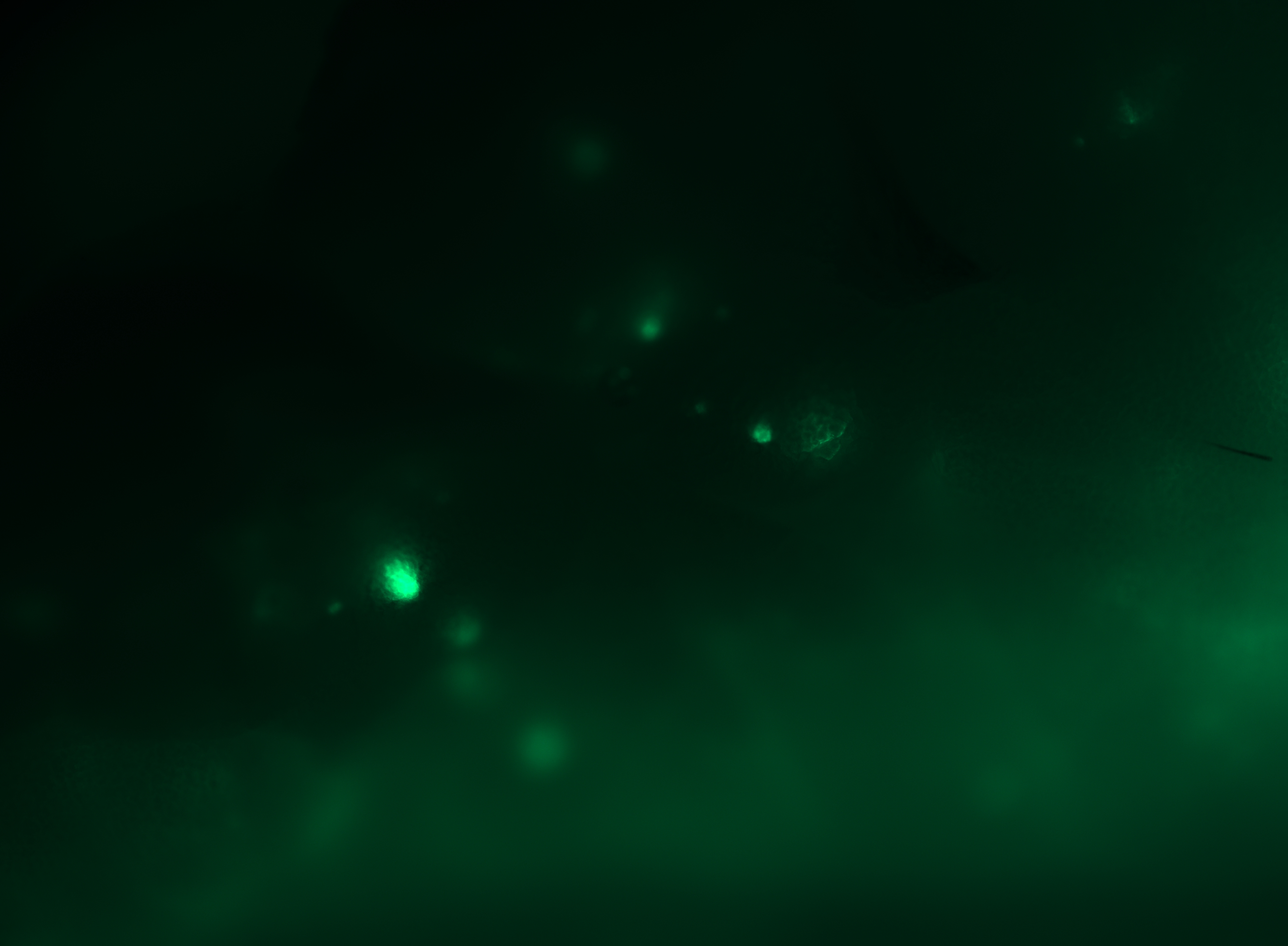

Supplement: Supplementary file 4 — Source data Fig. 2 [file 44318_2025_434_MOESM4_ESM.zip › Figure 2/2C/2C_zsGreen_mag.tif]

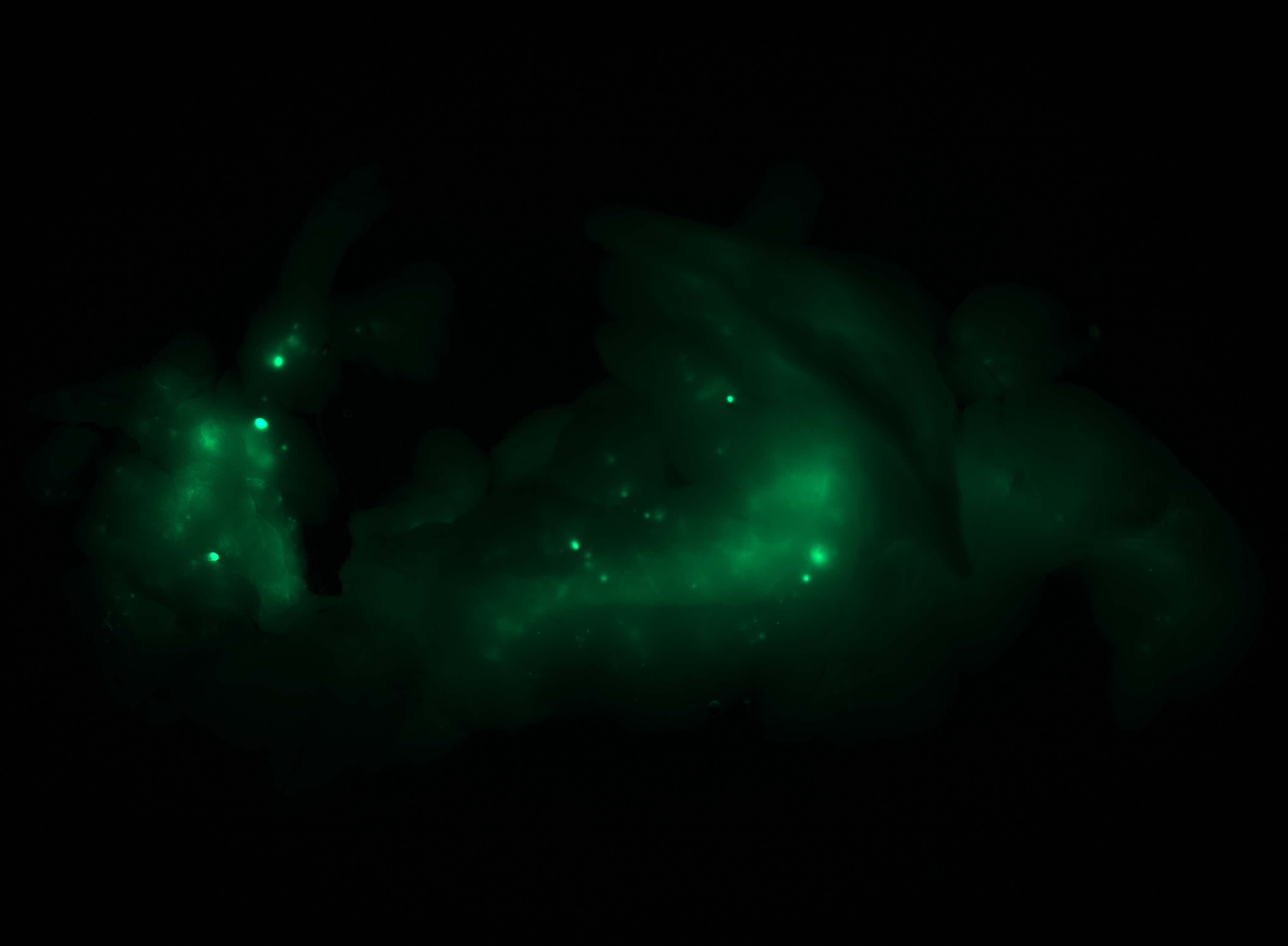

Supplement: Supplementary file 4 — Source data Fig. 2 [file 44318_2025_434_MOESM4_ESM.zip › Figure 2/2C/2C_zsGreen.tif]

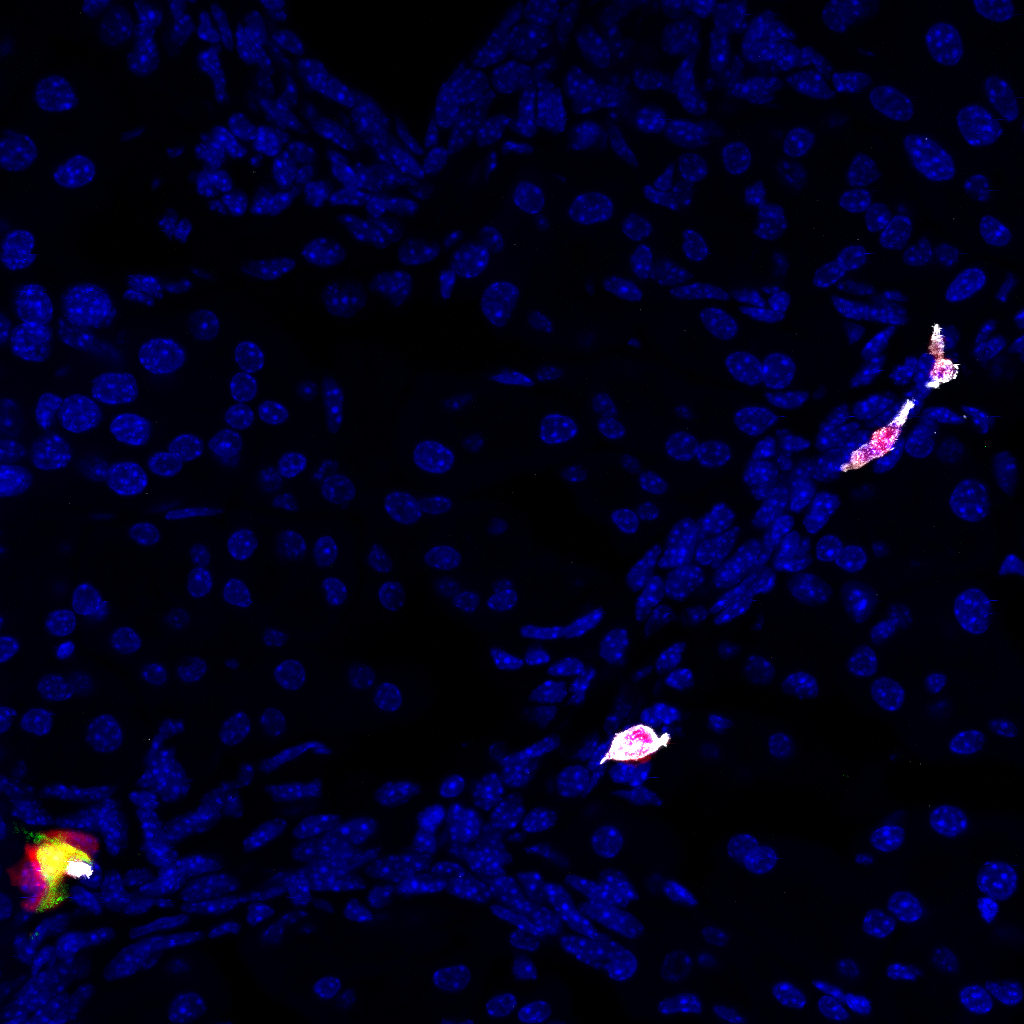

Supplement: Supplementary file 4 — Source data Fig. 2 [file 44318_2025_434_MOESM4_ESM.zip › Figure 2/2D/2D_Sst.tif]

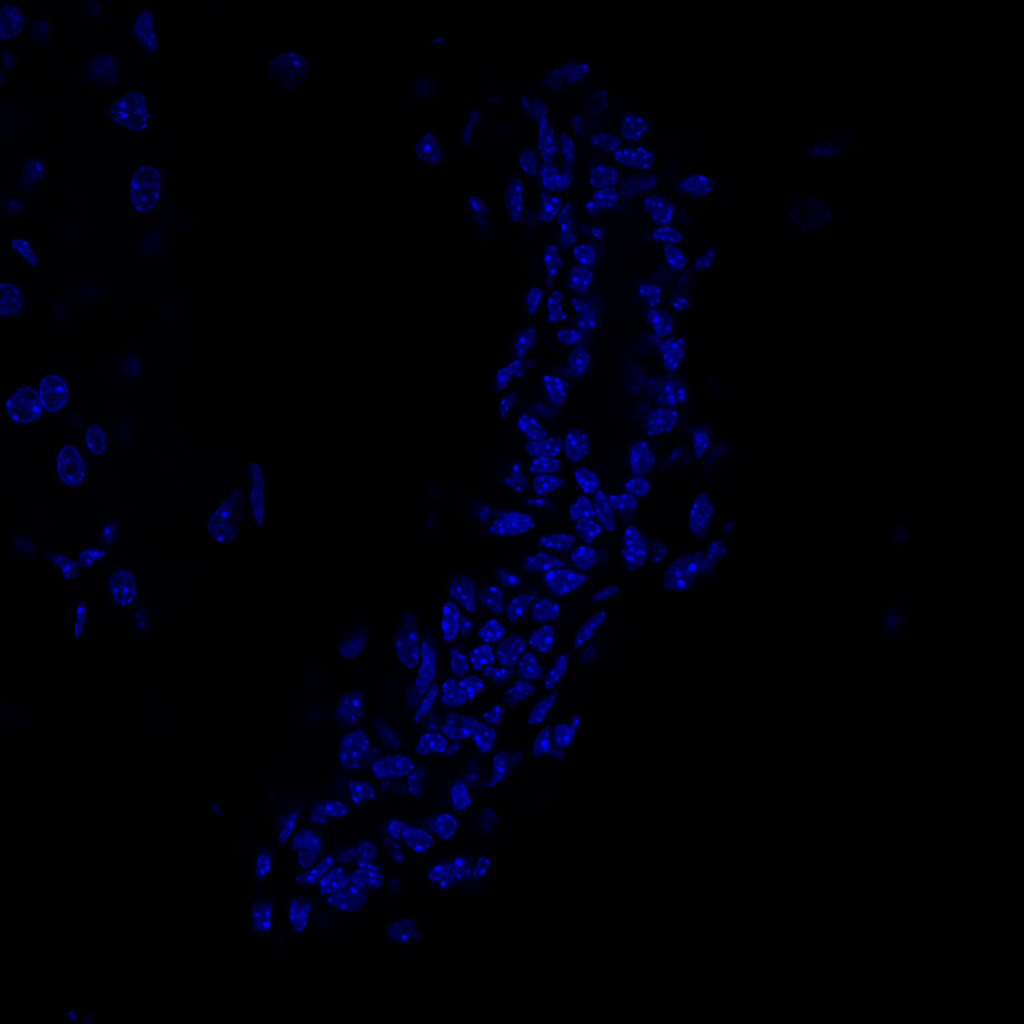

Supplement: Supplementary file 4 — Source data Fig. 2 [file 44318_2025_434_MOESM4_ESM.zip › Figure 2/2D/2D_CK19 (blue).tif]

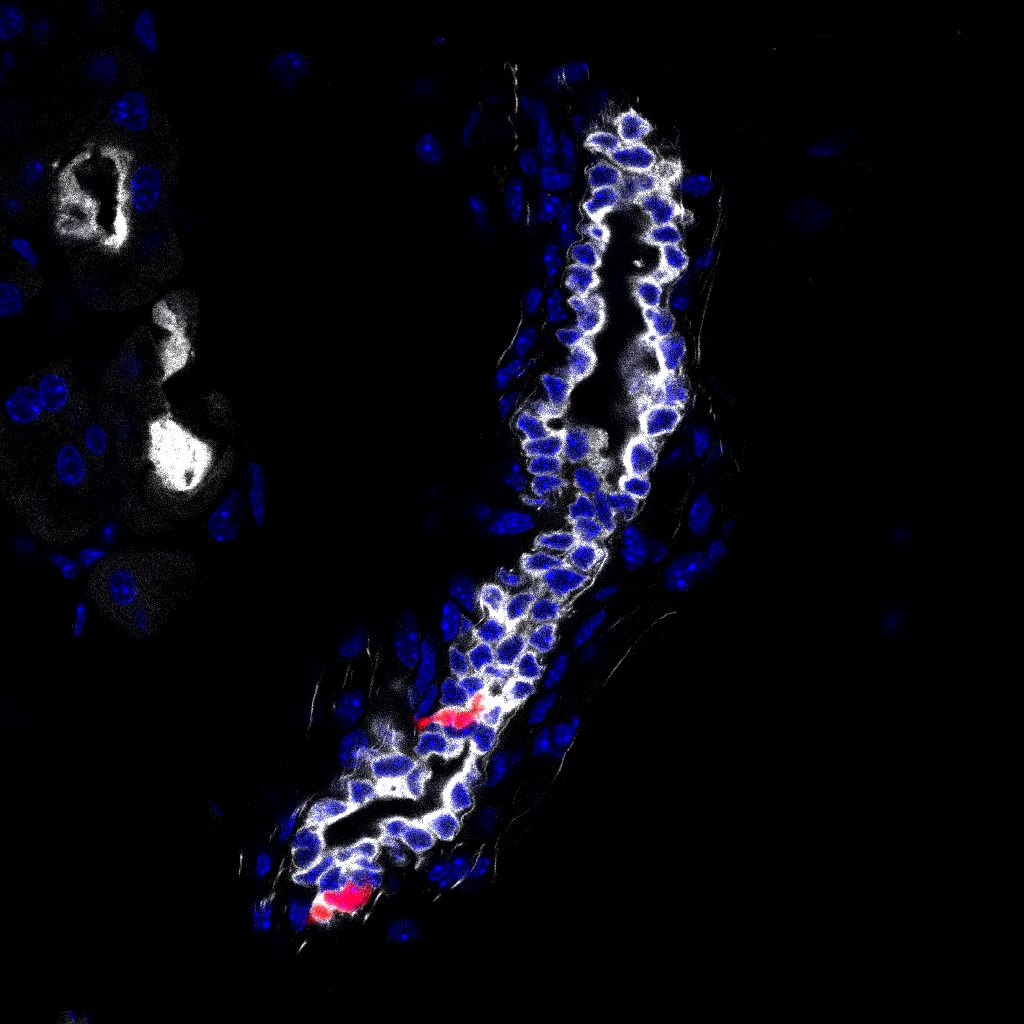

Supplement: Supplementary file 4 — Source data Fig. 2 [file 44318_2025_434_MOESM4_ESM.zip › Figure 2/2D/2D_CK19.tif]

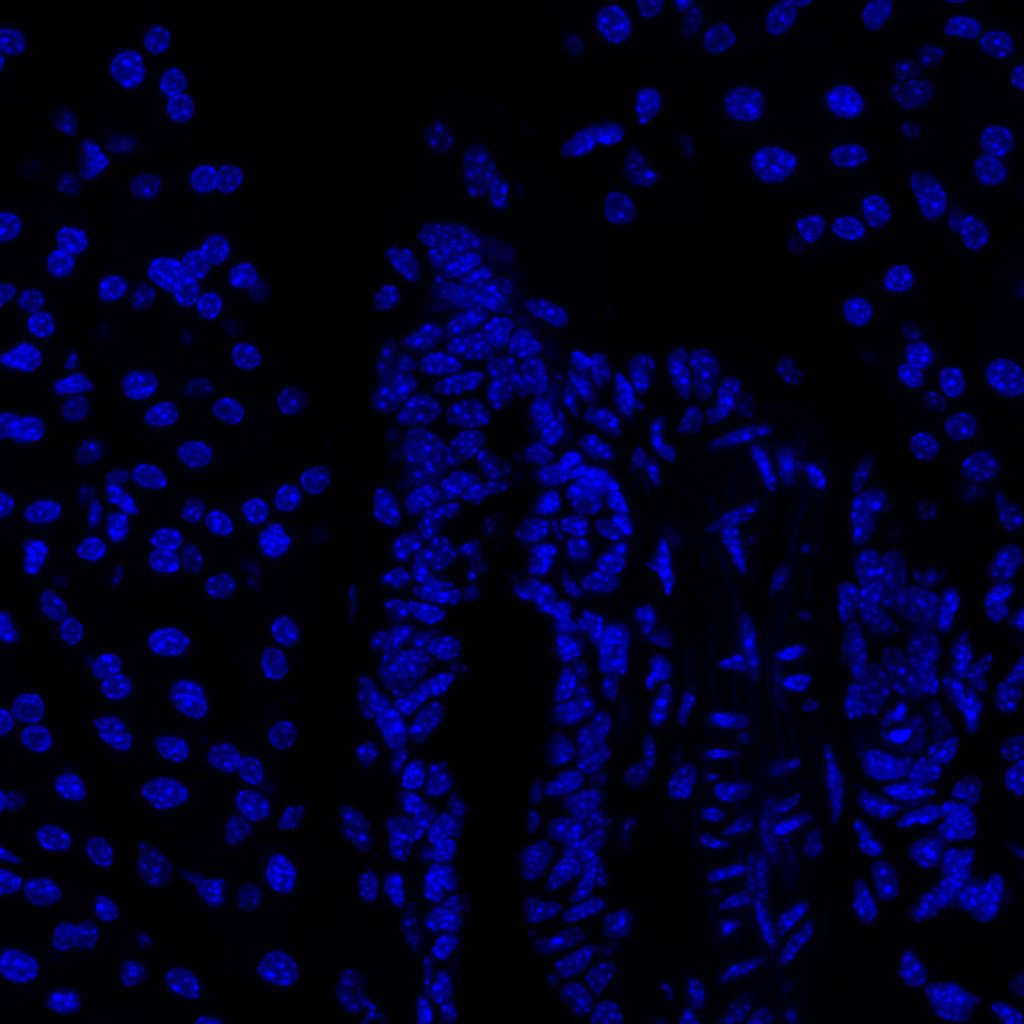

Supplement: Supplementary file 4 — Source data Fig. 2 [file 44318_2025_434_MOESM4_ESM.zip › Figure 2/2D/2D_Ins (blue).tif]

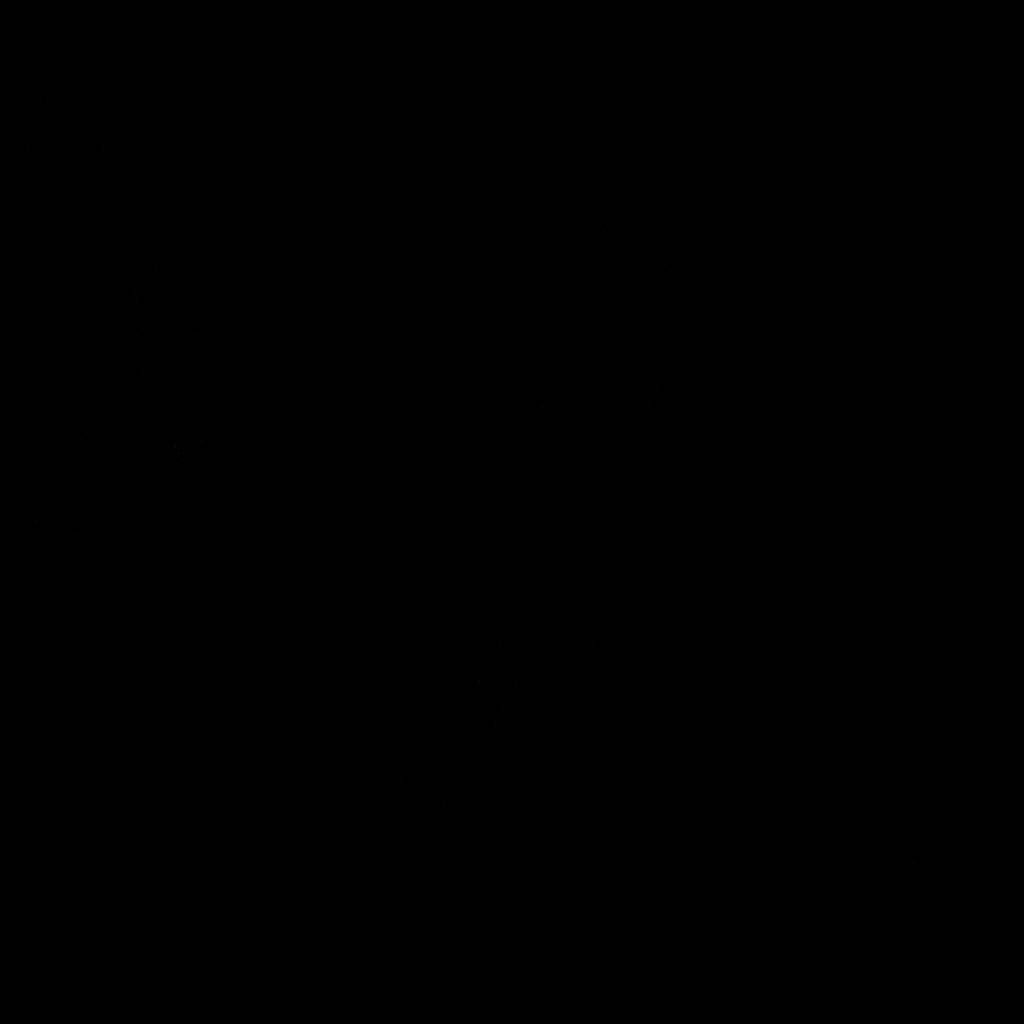

Supplement: Supplementary file 4 — Source data Fig. 2 [file 44318_2025_434_MOESM4_ESM.zip › Figure 2/2D/2D_CK19 (green).tif]

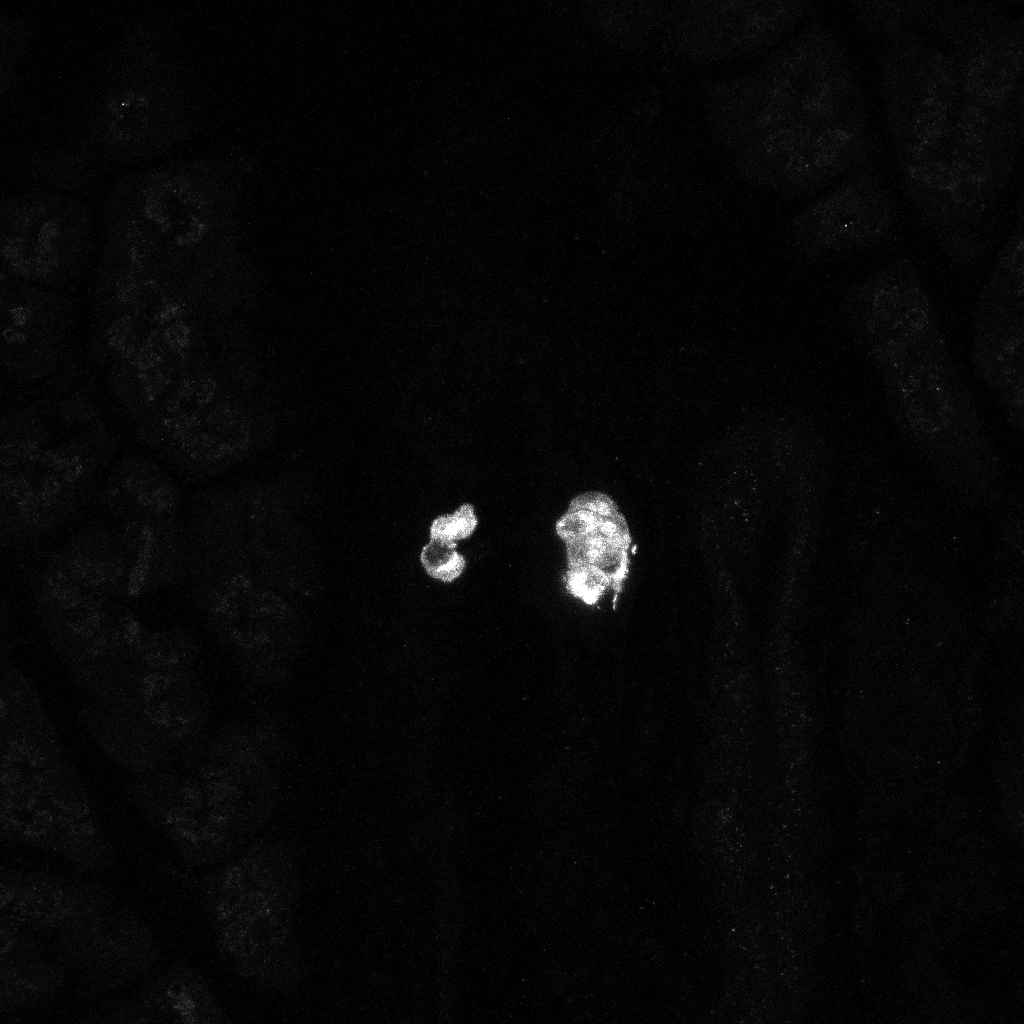

Supplement: Supplementary file 4 — Source data Fig. 2 [file 44318_2025_434_MOESM4_ESM.zip › Figure 2/2D/2D_Ins (gray).tif]

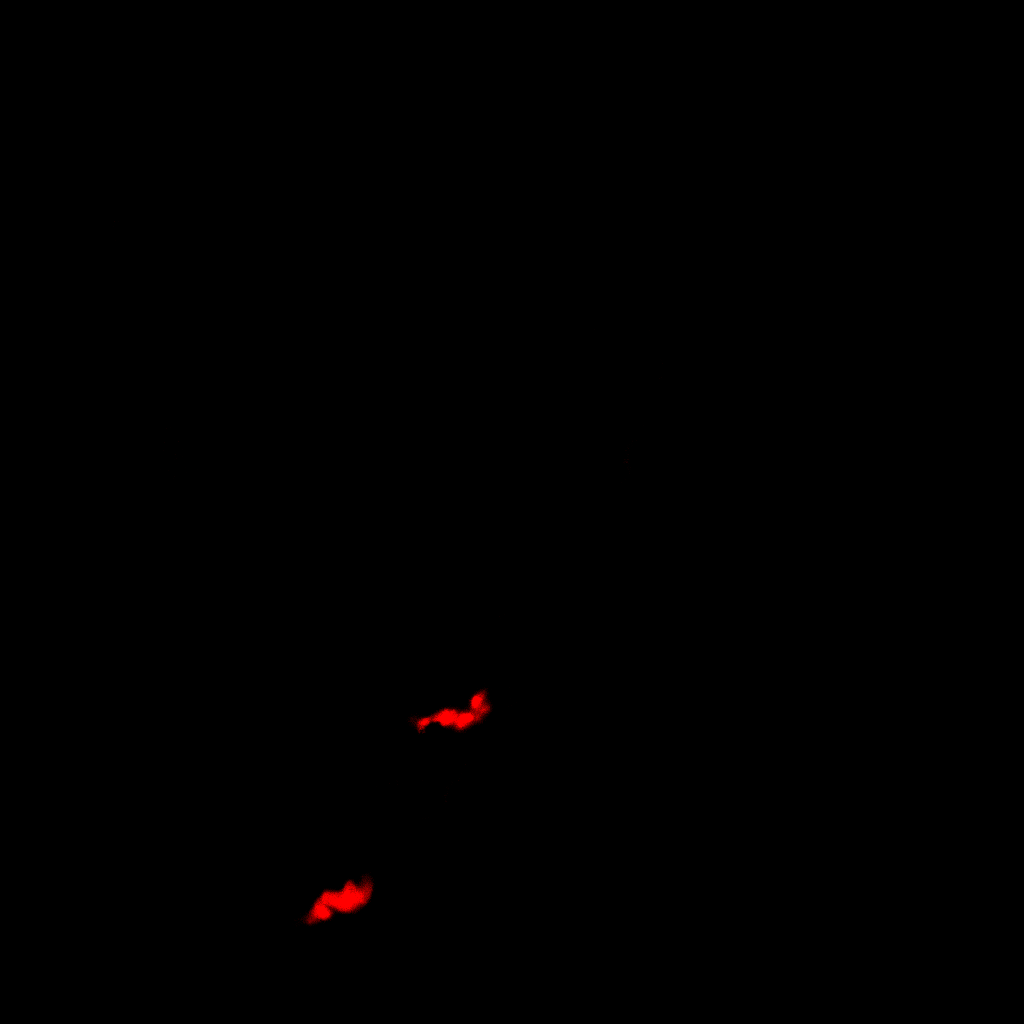

Supplement: Supplementary file 4 — Source data Fig. 2 [file 44318_2025_434_MOESM4_ESM.zip › Figure 2/2D/2D_CK19 (red).tif]

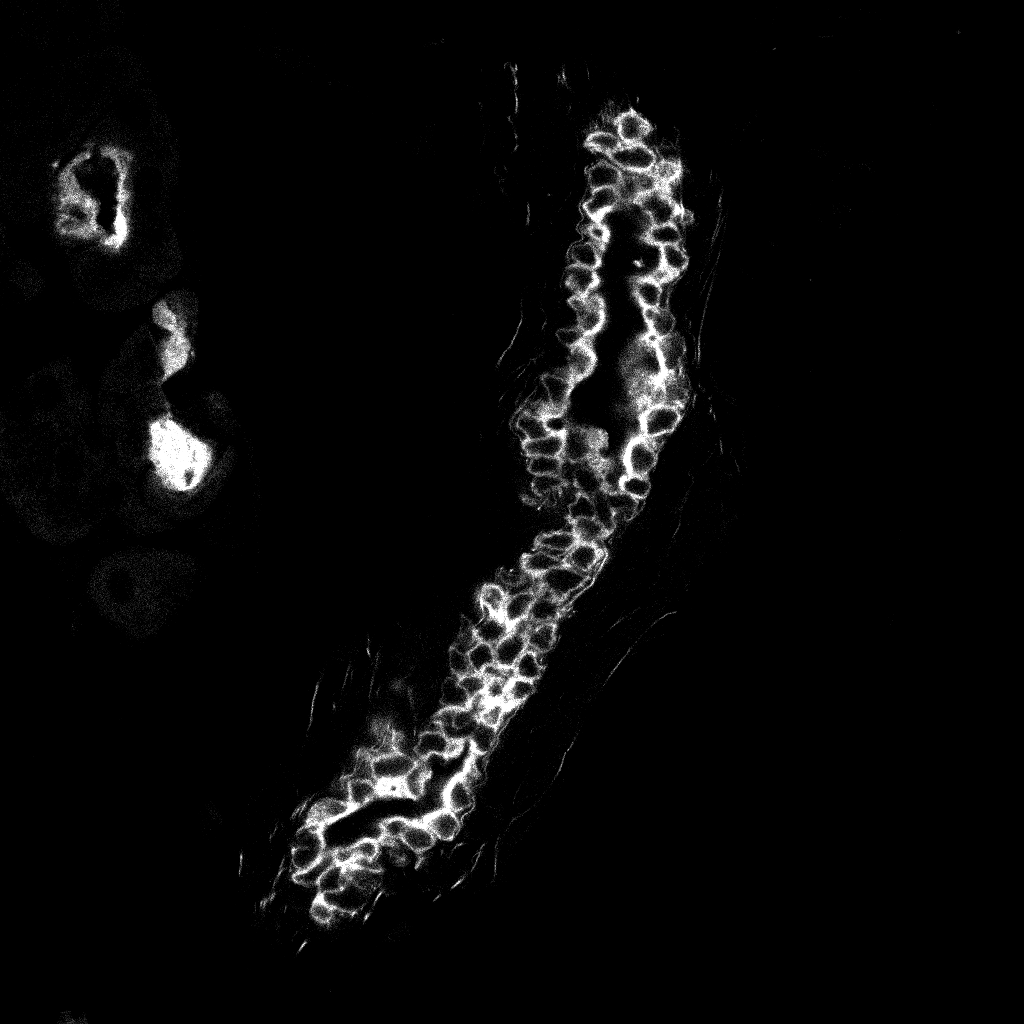

Supplement: Supplementary file 4 — Source data Fig. 2 [file 44318_2025_434_MOESM4_ESM.zip › Figure 2/2D/2D_CK19 (gray).tif]

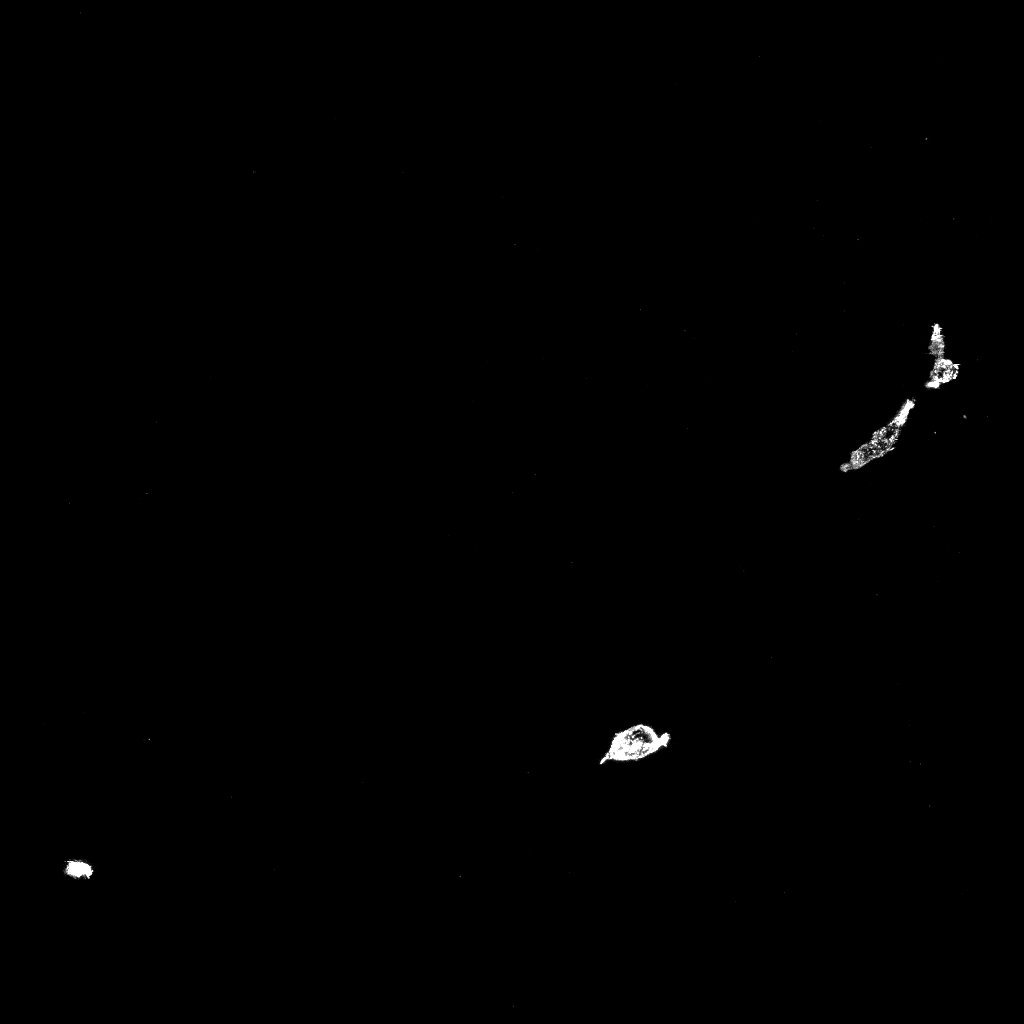

Supplement: Supplementary file 4 — Source data Fig. 2 [file 44318_2025_434_MOESM4_ESM.zip › Figure 2/2D/2D_Sst (gray).tif]

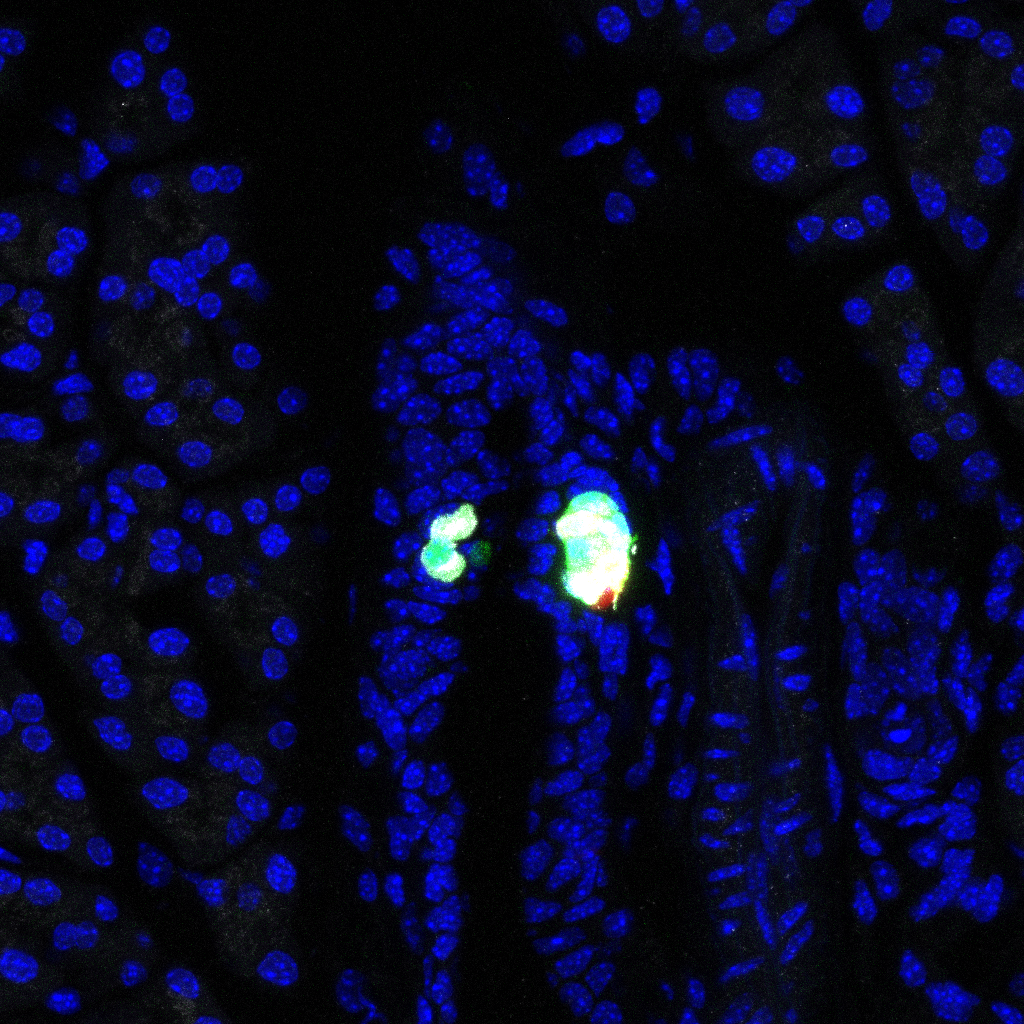

Supplement: Supplementary file 4 — Source data Fig. 2 [file 44318_2025_434_MOESM4_ESM.zip › Figure 2/2D/2D_Ins.tif]

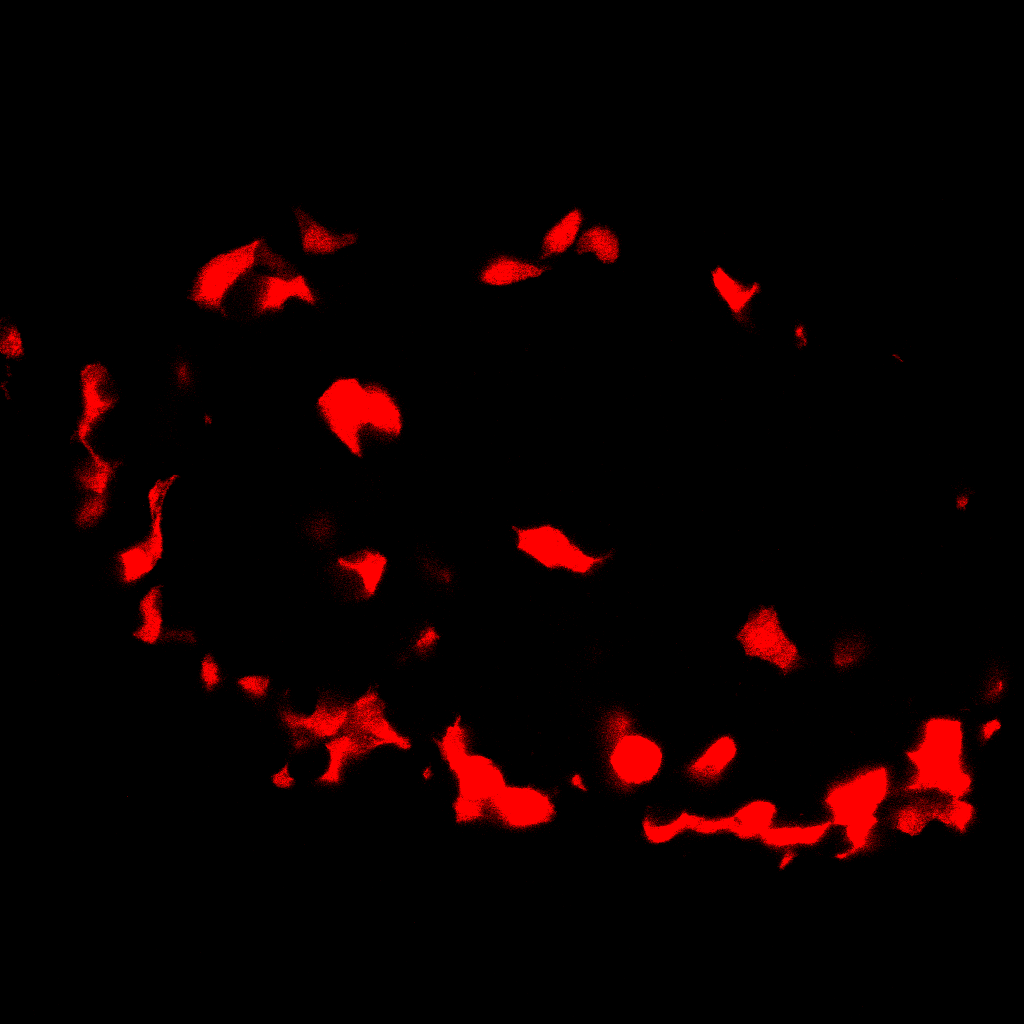

Supplement: Supplementary file 4 — Source data Fig. 2 [file 44318_2025_434_MOESM4_ESM.zip › Figure 2/2M/2M_2w_Merge (red).tif]

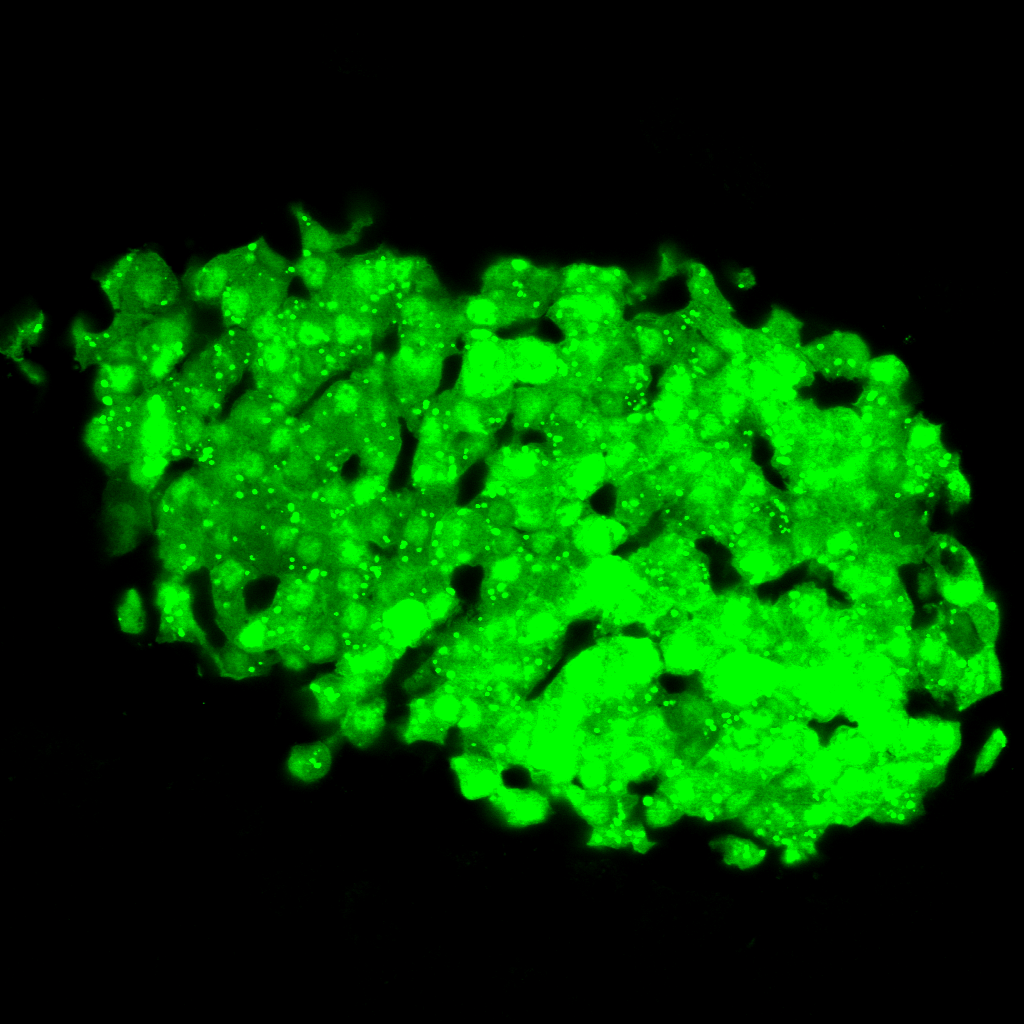

Supplement: Supplementary file 4 — Source data Fig. 2 [file 44318_2025_434_MOESM4_ESM.zip › Figure 2/2M/2M_2w_Merge (green).tif]

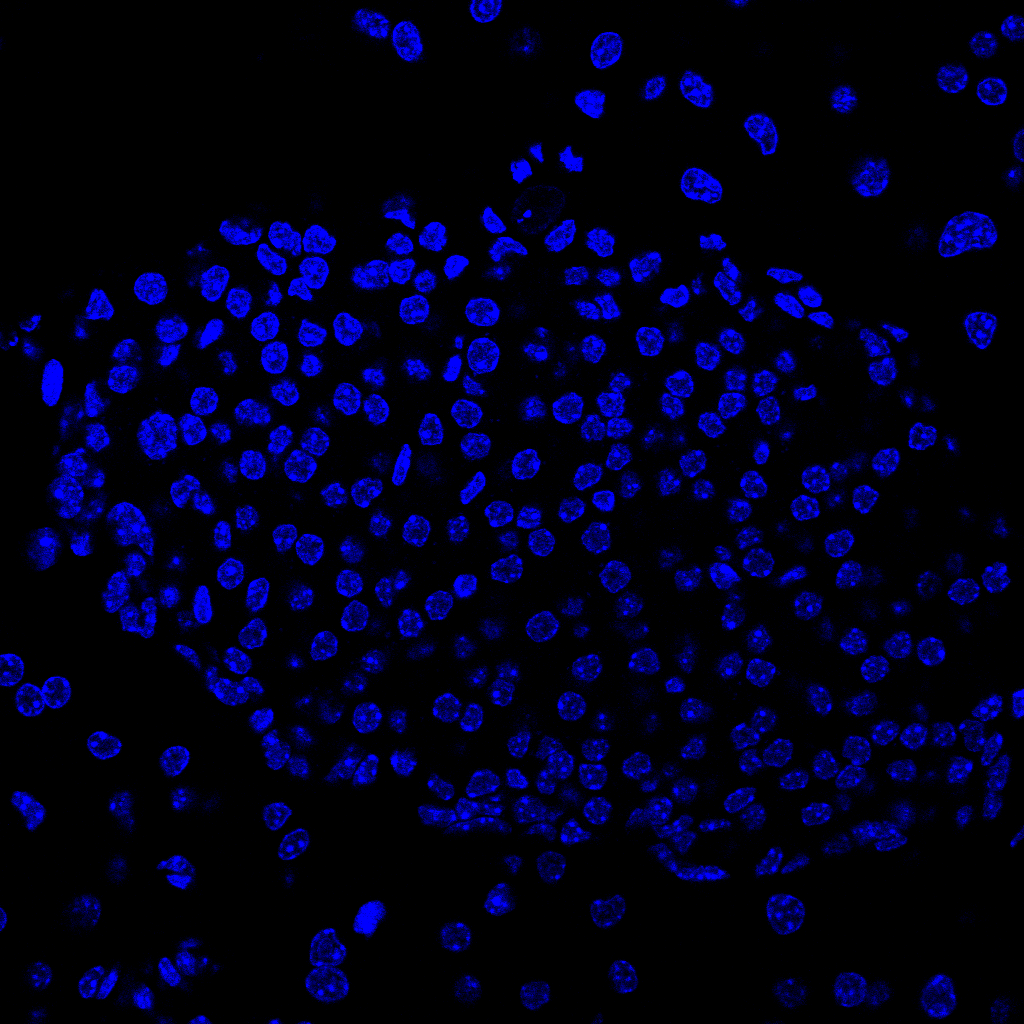

Supplement: Supplementary file 4 — Source data Fig. 2 [file 44318_2025_434_MOESM4_ESM.zip › Figure 2/2M/2M_2w_Merge (blue).tif]

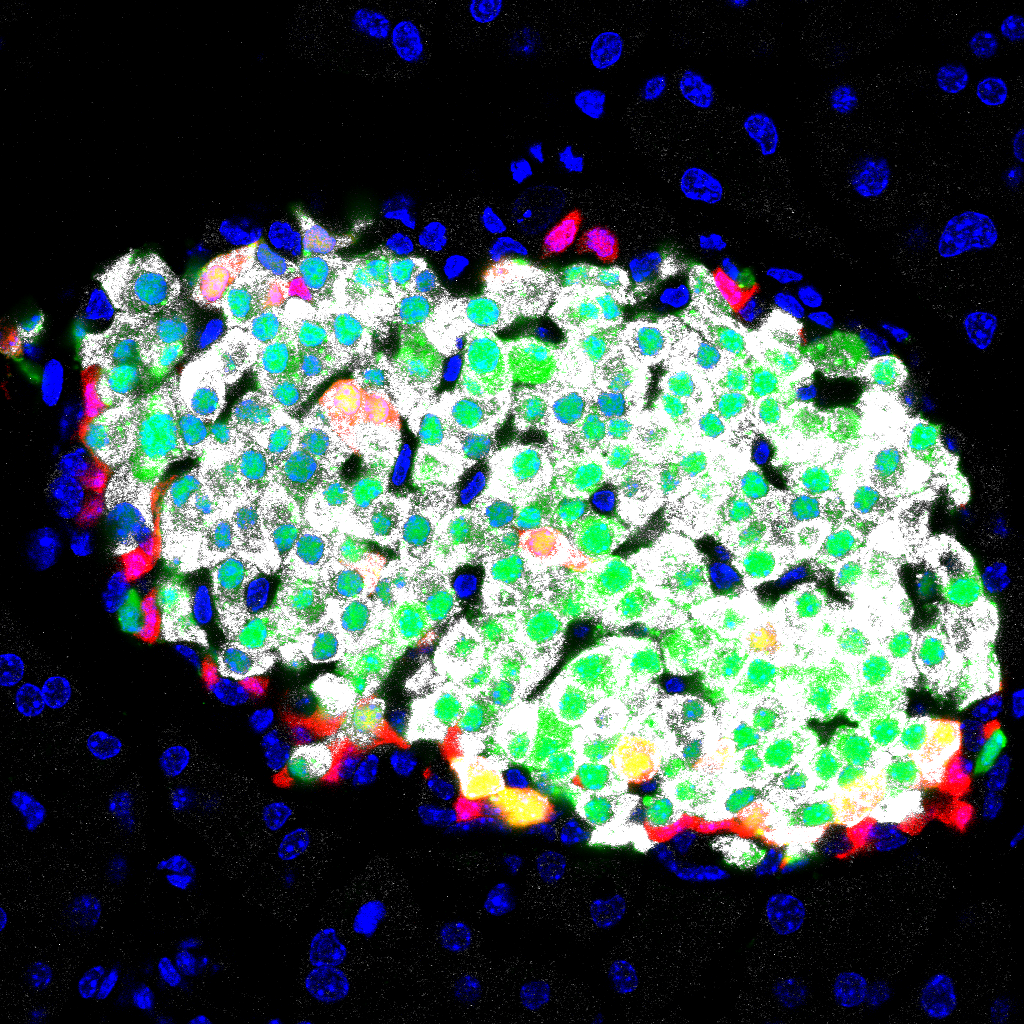

Supplement: Supplementary file 4 — Source data Fig. 2 [file 44318_2025_434_MOESM4_ESM.zip › Figure 2/2M/2M_2w_Merge.tif]

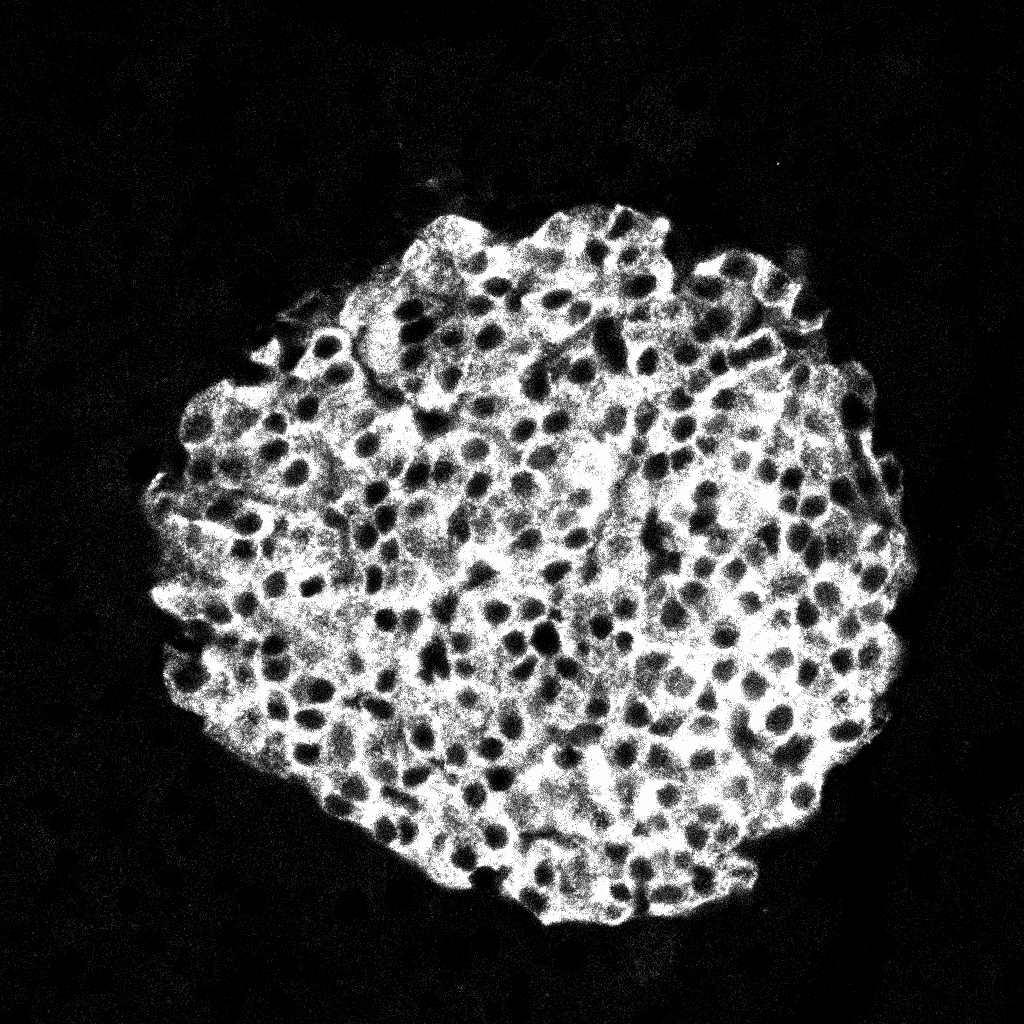

Supplement: Supplementary file 4 — Source data Fig. 2 [file 44318_2025_434_MOESM4_ESM.zip › Figure 2/2M/2M_12w_Merge (gray).tif]

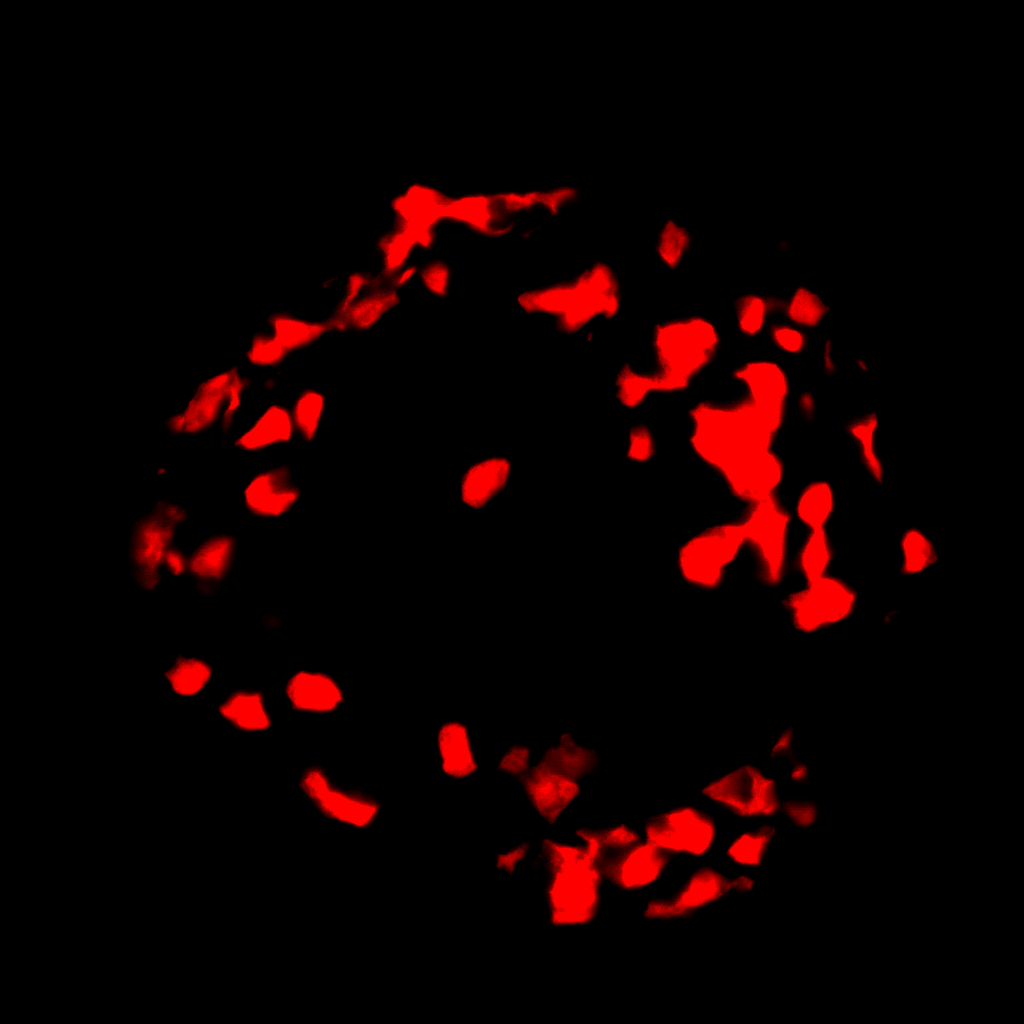

Supplement: Supplementary file 4 — Source data Fig. 2 [file 44318_2025_434_MOESM4_ESM.zip › Figure 2/2M/2M_12w_Merge (red).tif]

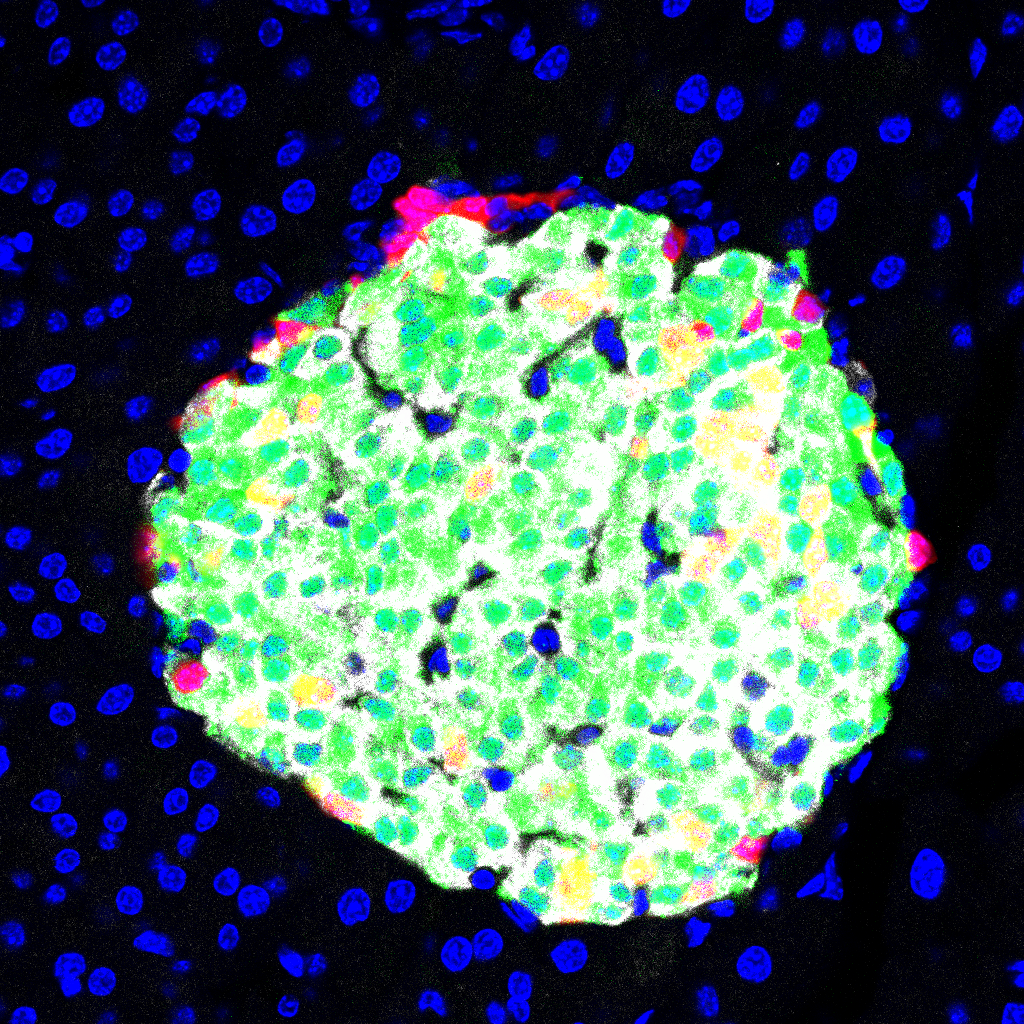

Supplement: Supplementary file 4 — Source data Fig. 2 [file 44318_2025_434_MOESM4_ESM.zip › Figure 2/2M/2M_12w_Merge.tif]

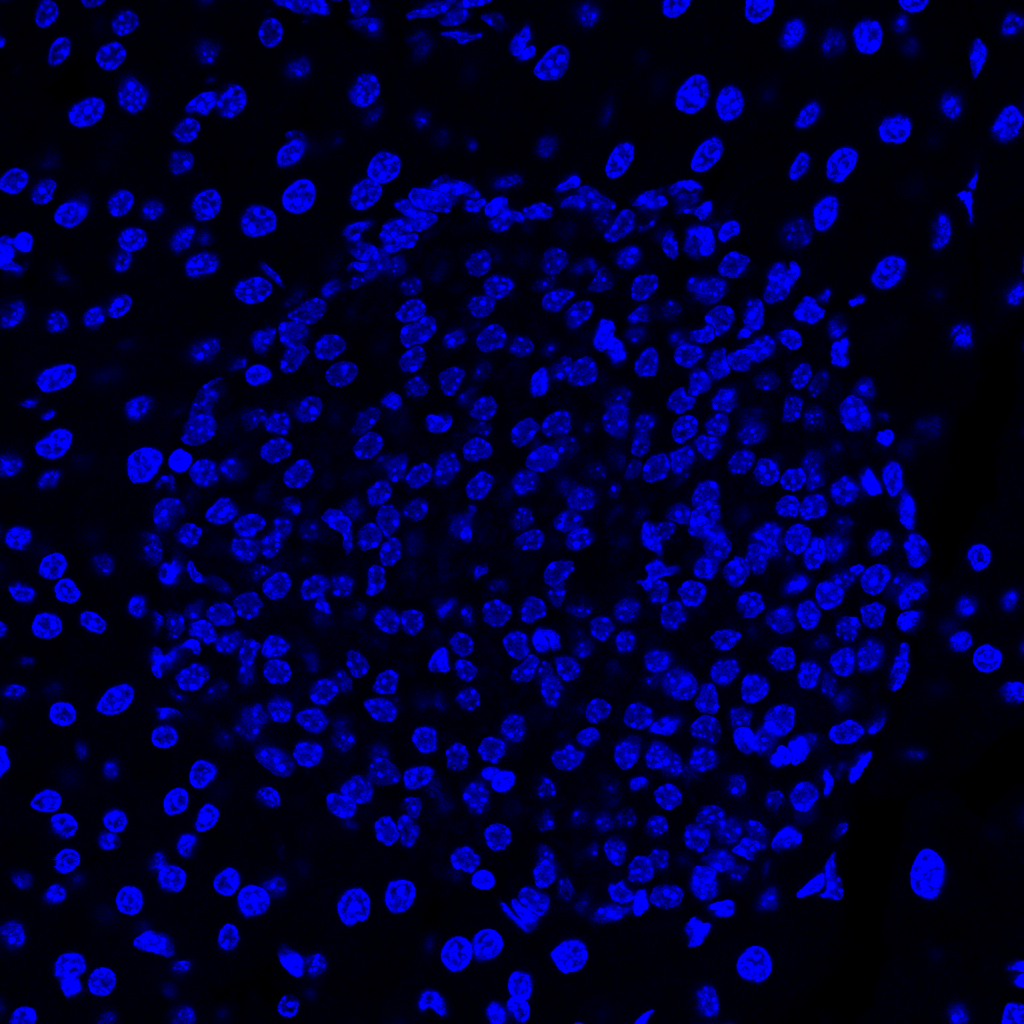

Supplement: Supplementary file 4 — Source data Fig. 2 [file 44318_2025_434_MOESM4_ESM.zip › Figure 2/2M/2M_12w_Merge (blue).tif]

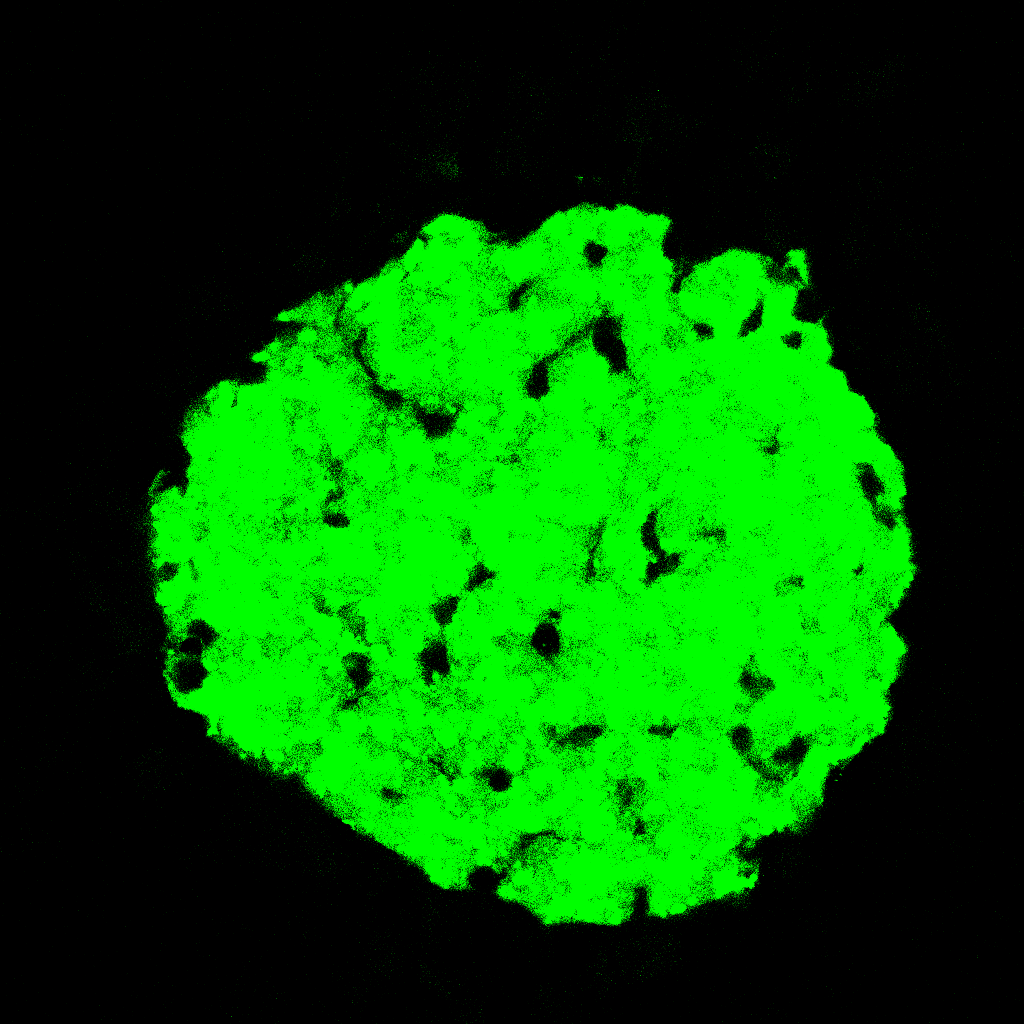

Supplement: Supplementary file 4 — Source data Fig. 2 [file 44318_2025_434_MOESM4_ESM.zip › Figure 2/2M/2M_12w_Merge (green).tif]

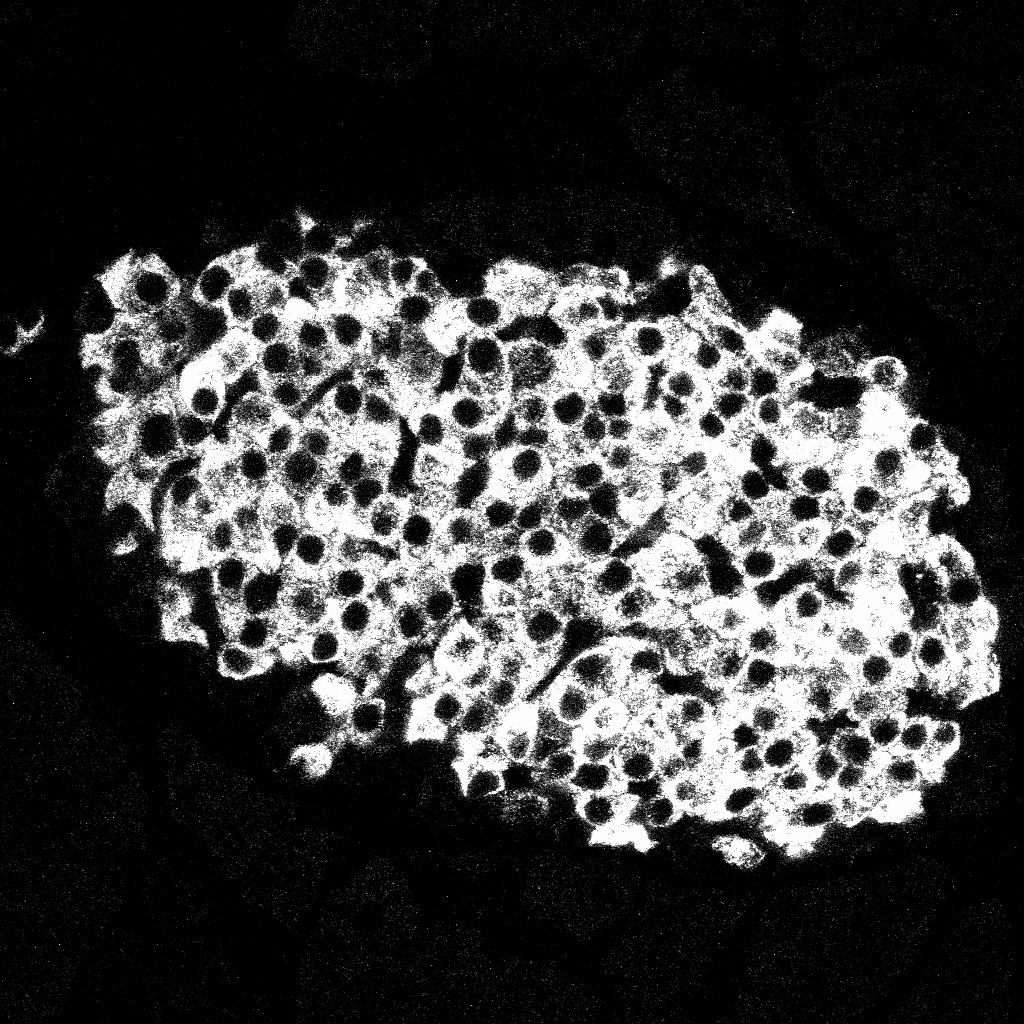

Supplement: Supplementary file 4 — Source data Fig. 2 [file 44318_2025_434_MOESM4_ESM.zip › Figure 2/2M/2M_2w_Merge (gray).tif]

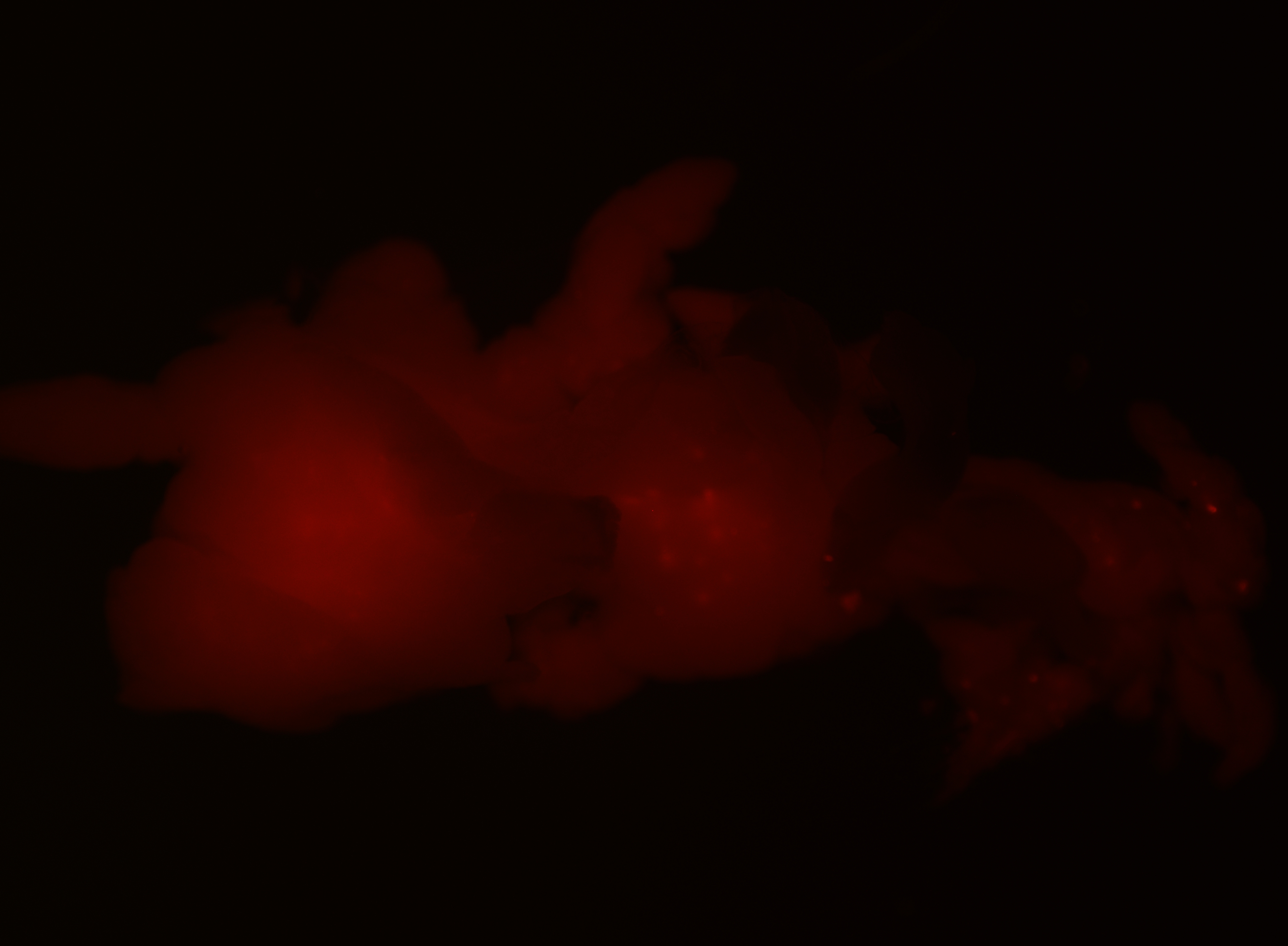

Supplement: Supplementary file 4 — Source data Fig. 2 [file 44318_2025_434_MOESM4_ESM.zip › Figure 2/2J/2J_tdT.tif]

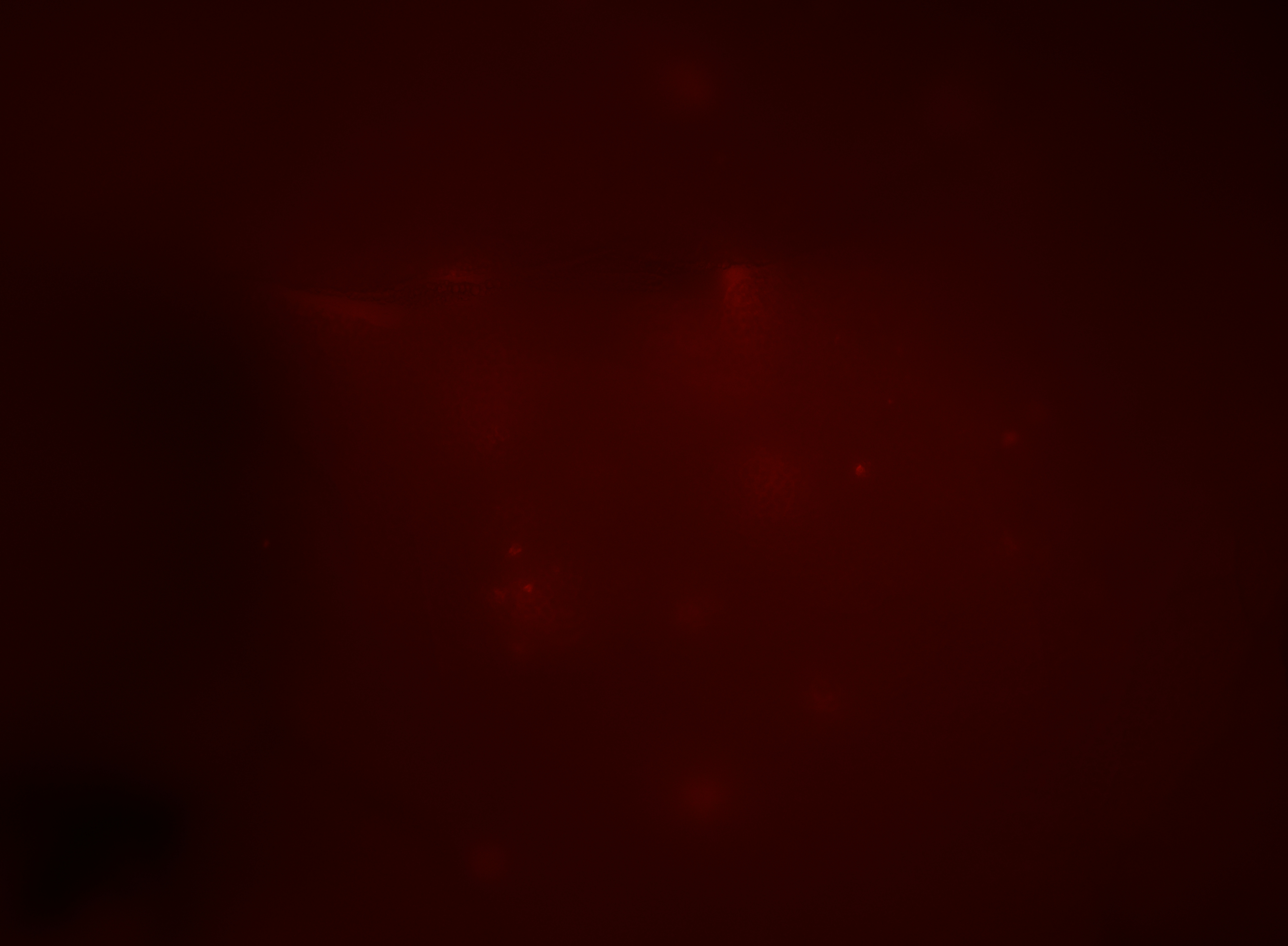

Supplement: Supplementary file 4 — Source data Fig. 2 [file 44318_2025_434_MOESM4_ESM.zip › Figure 2/2J/2J_tdT_mag.tif]

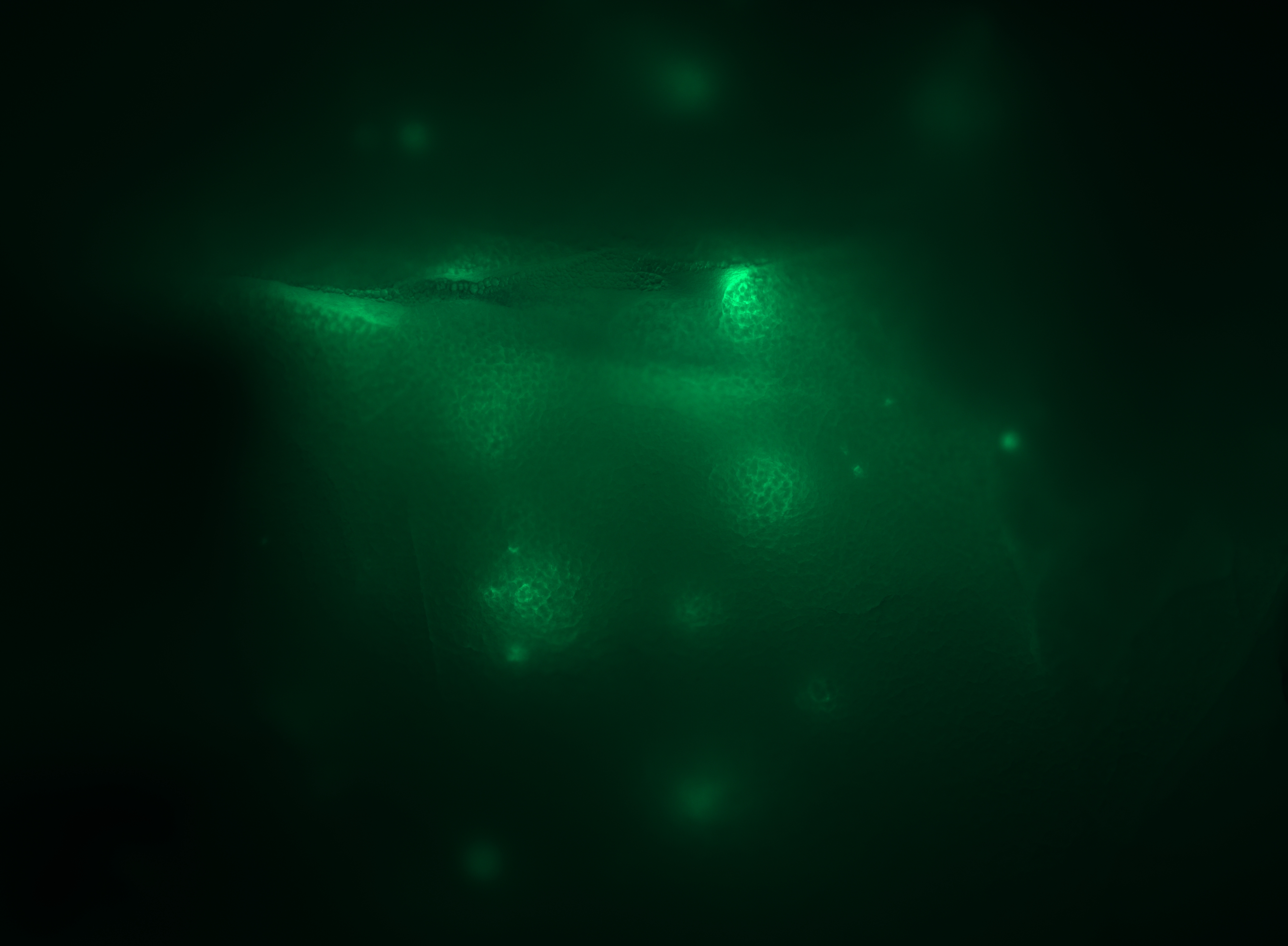

Supplement: Supplementary file 4 — Source data Fig. 2 [file 44318_2025_434_MOESM4_ESM.zip › Figure 2/2J/2J_zsGreen_mag.tif]

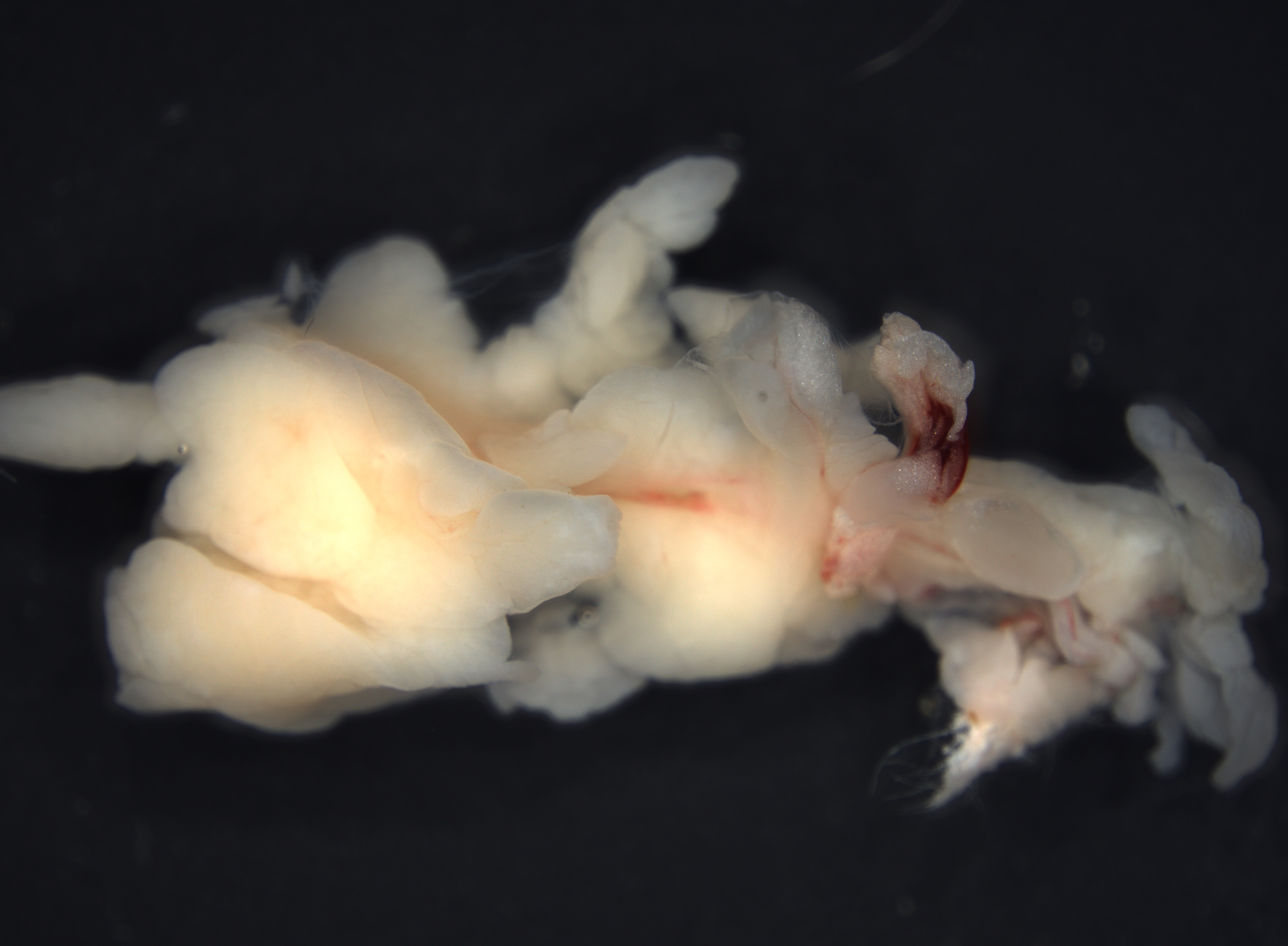

Supplement: Supplementary file 4 — Source data Fig. 2 [file 44318_2025_434_MOESM4_ESM.zip › Figure 2/2J/2J_BF.tif]

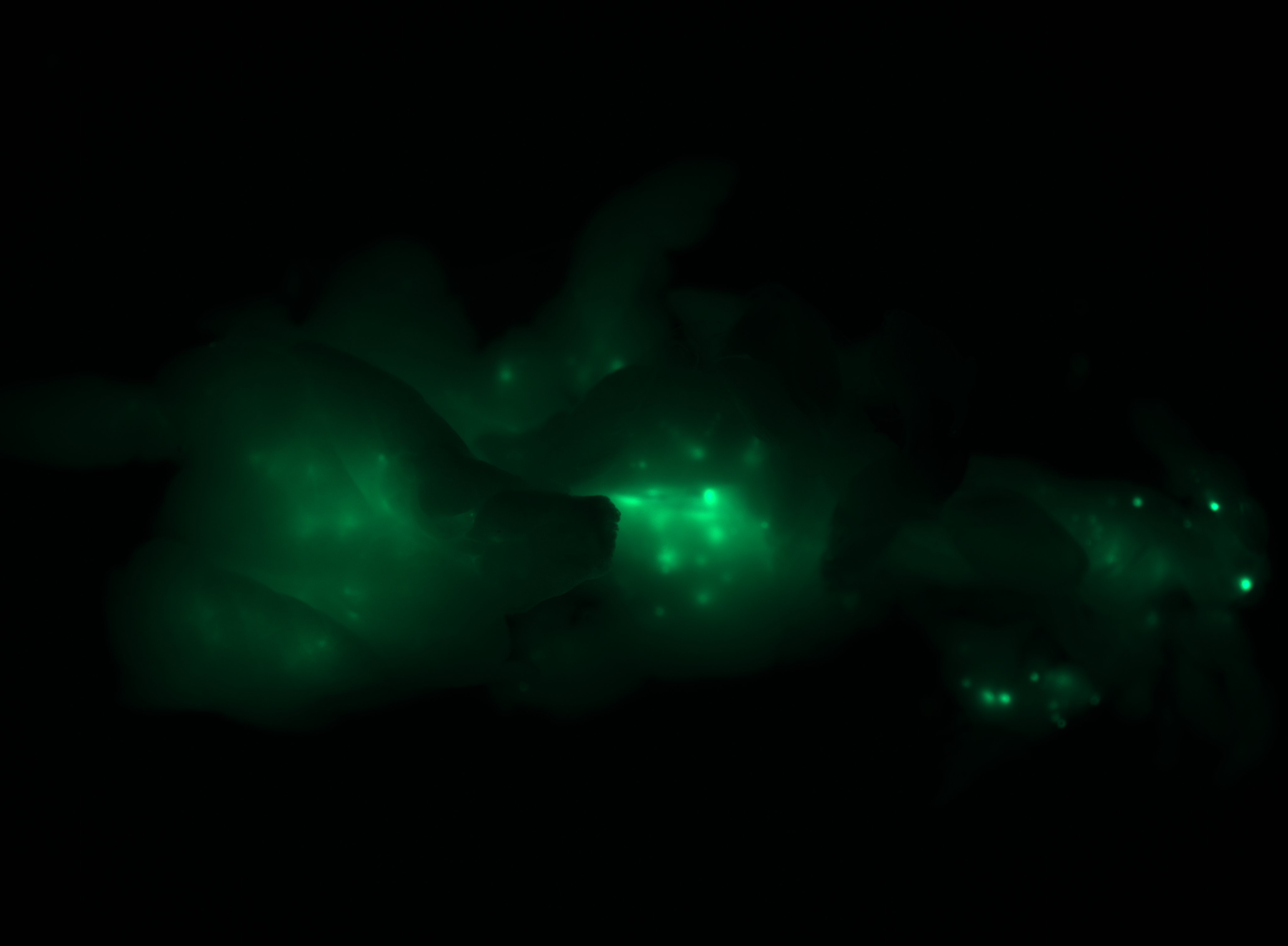

Supplement: Supplementary file 4 — Source data Fig. 2 [file 44318_2025_434_MOESM4_ESM.zip › Figure 2/2J/2J_zsGreen.tif]

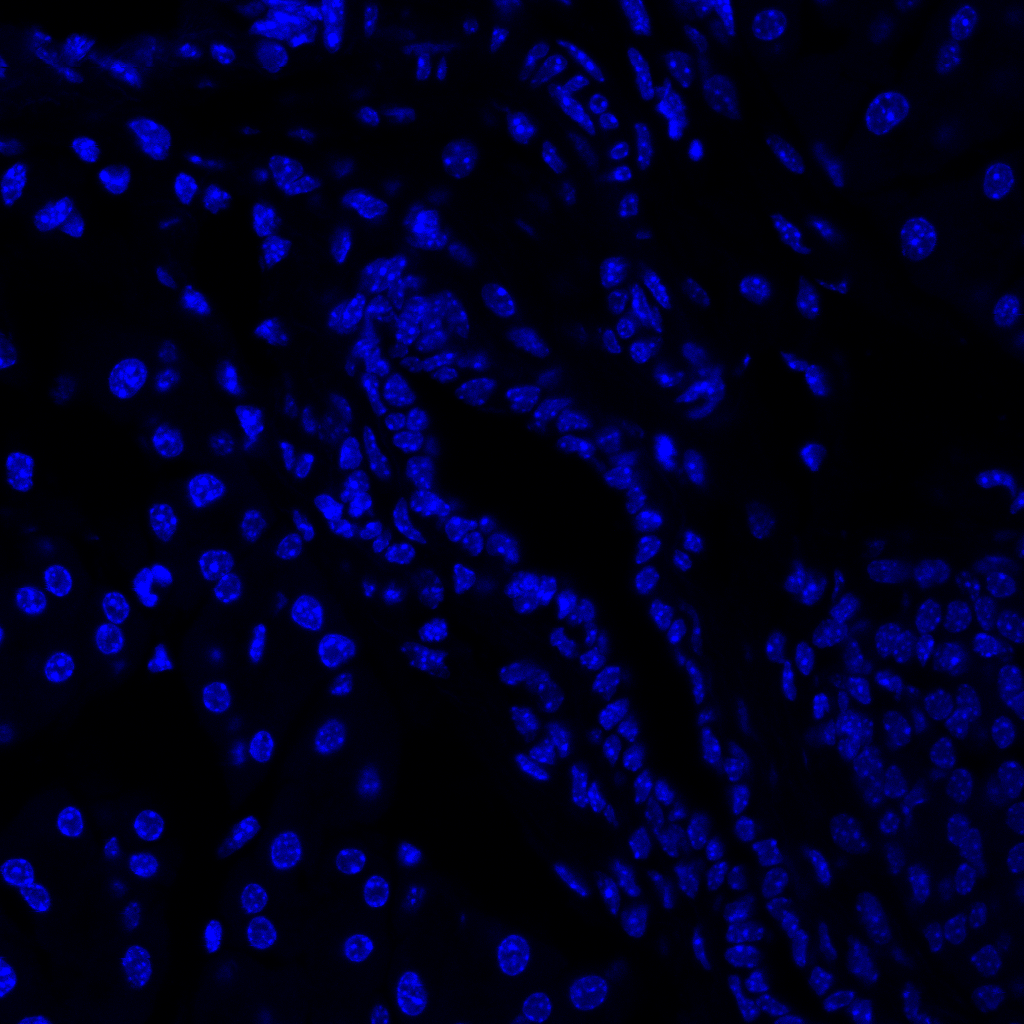

Supplement: Supplementary file 4 — Source data Fig. 2 [file 44318_2025_434_MOESM4_ESM.zip › Figure 2/2K/2K_2w_Sst (blue).tif]

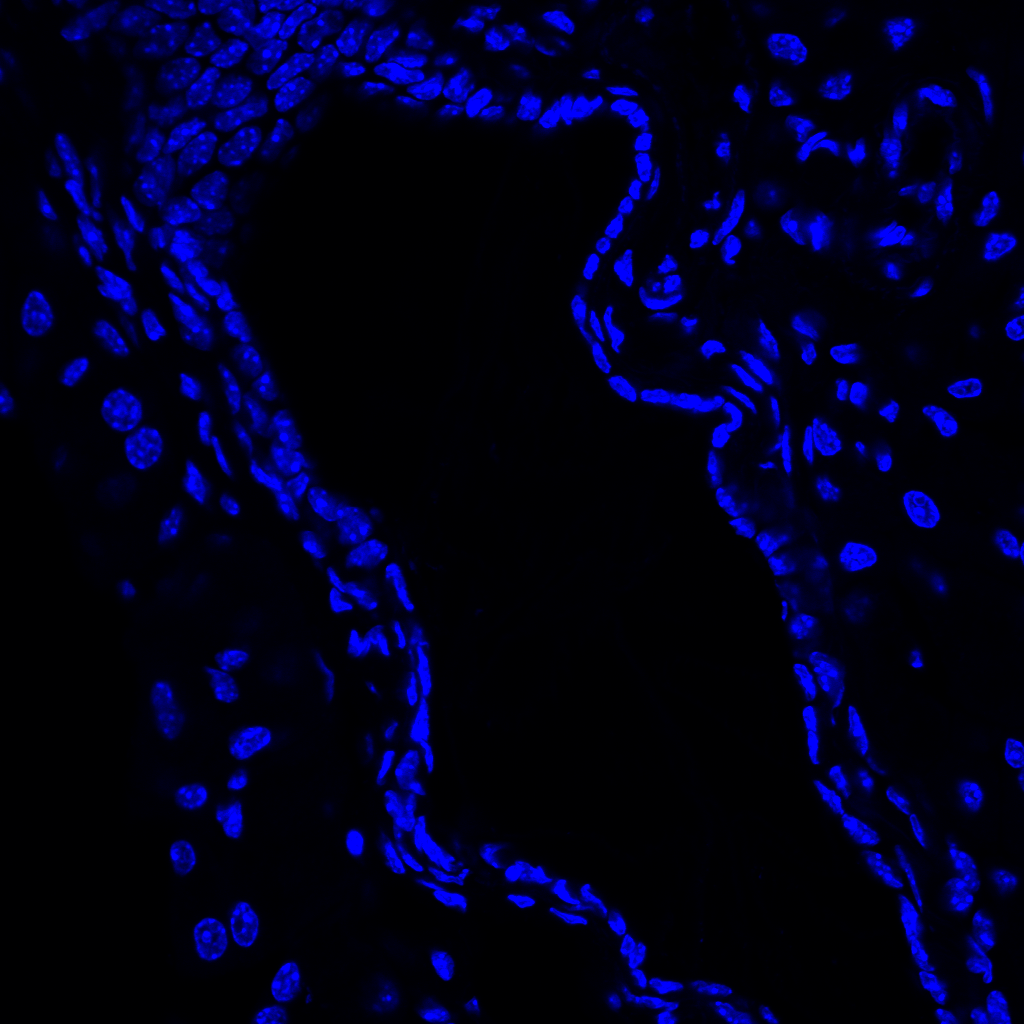

Supplement: Supplementary file 4 — Source data Fig. 2 [file 44318_2025_434_MOESM4_ESM.zip › Figure 2/2K/2K_12w_Ins (blue).tif]

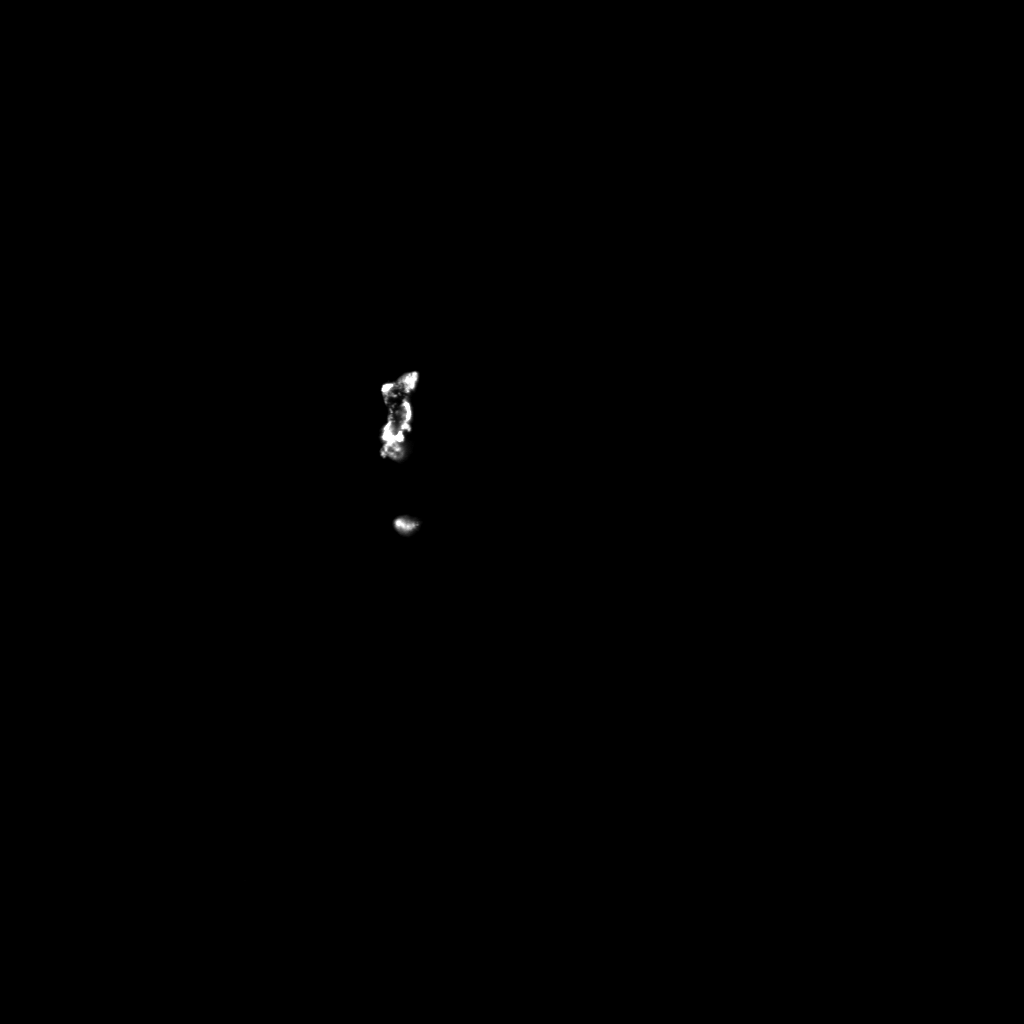

Supplement: Supplementary file 4 — Source data Fig. 2 [file 44318_2025_434_MOESM4_ESM.zip › Figure 2/2K/2K_12w_Sst.(gray).tif]

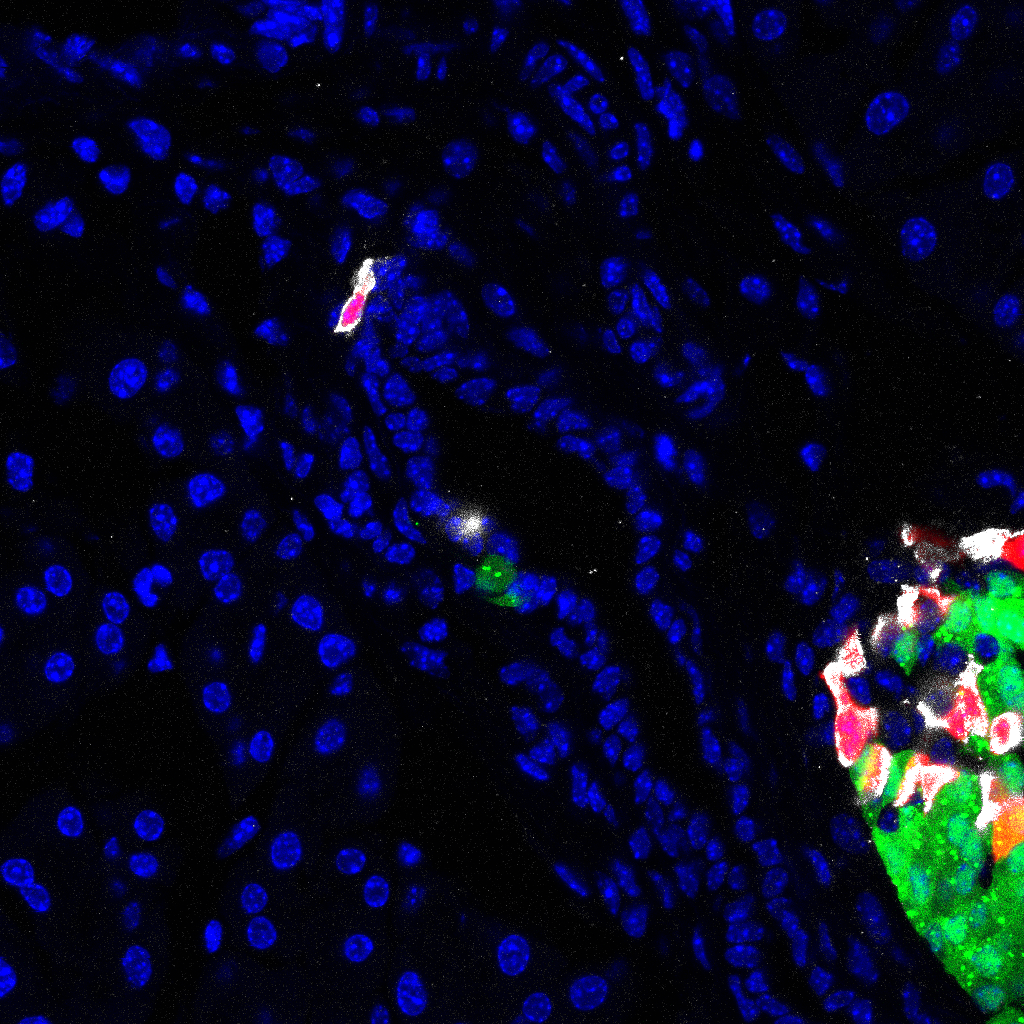

Supplement: Supplementary file 4 — Source data Fig. 2 [file 44318_2025_434_MOESM4_ESM.zip › Figure 2/2K/2K_2w_Sst.tif]

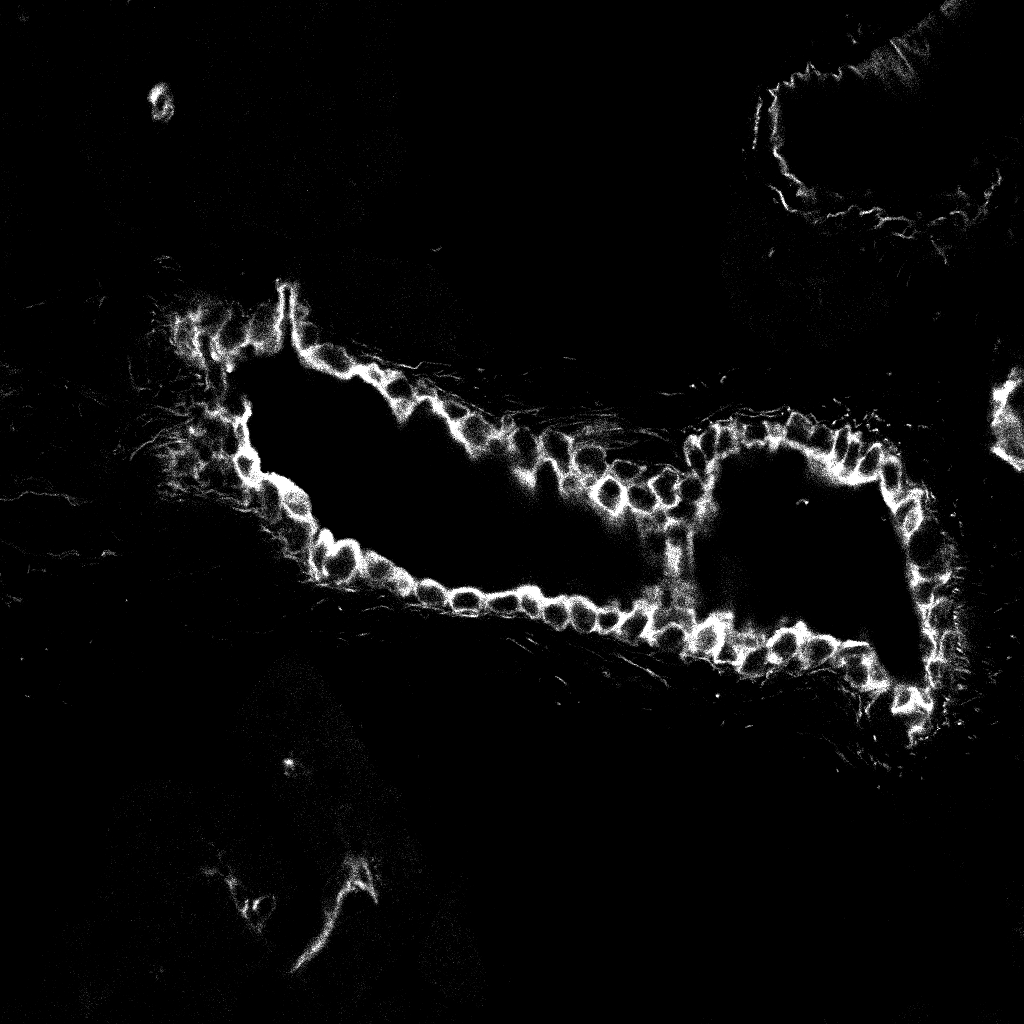

Supplement: Supplementary file 4 — Source data Fig. 2 [file 44318_2025_434_MOESM4_ESM.zip › Figure 2/2K/2K_2w_CK19 (gray).tif]

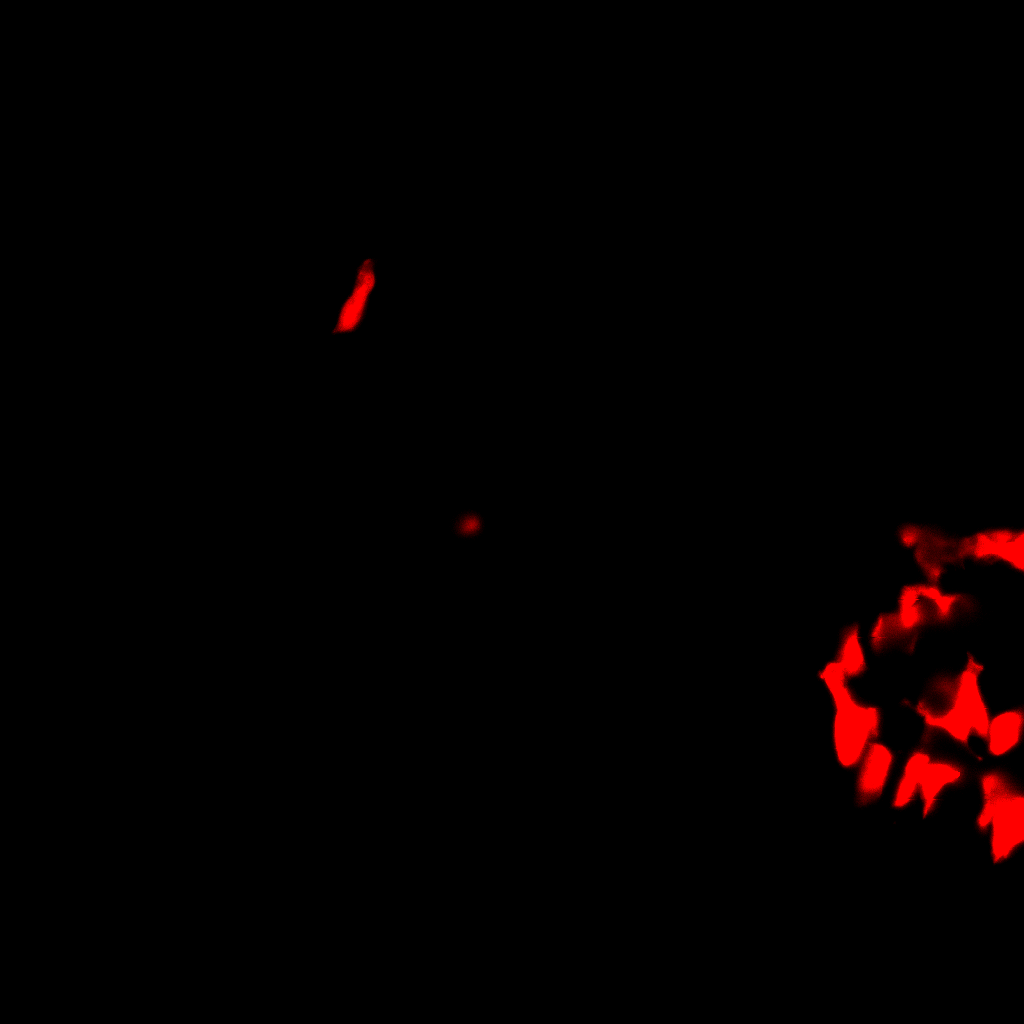

Supplement: Supplementary file 4 — Source data Fig. 2 [file 44318_2025_434_MOESM4_ESM.zip › Figure 2/2K/2K_2w_Sst (red).tif]

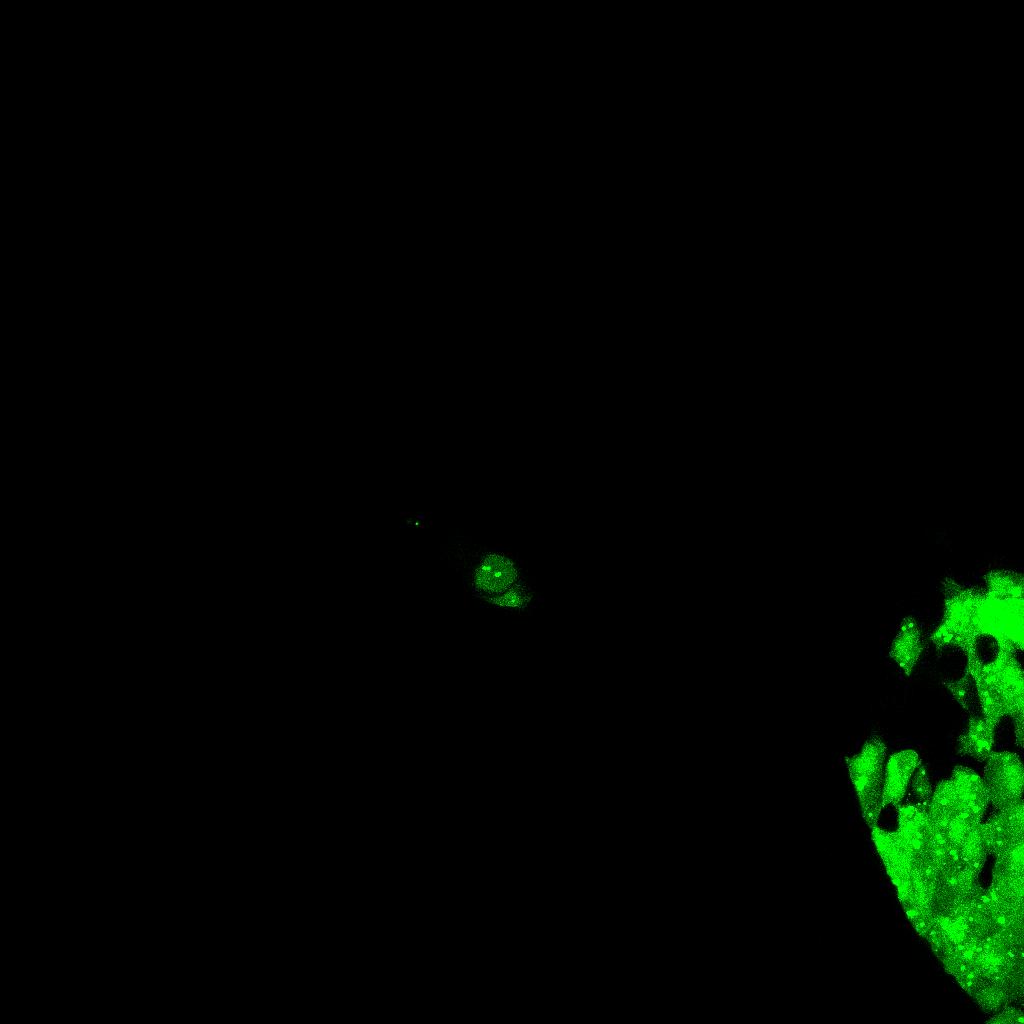

Supplement: Supplementary file 4 — Source data Fig. 2 [file 44318_2025_434_MOESM4_ESM.zip › Figure 2/2K/2K_2w_Sst (green).tif]

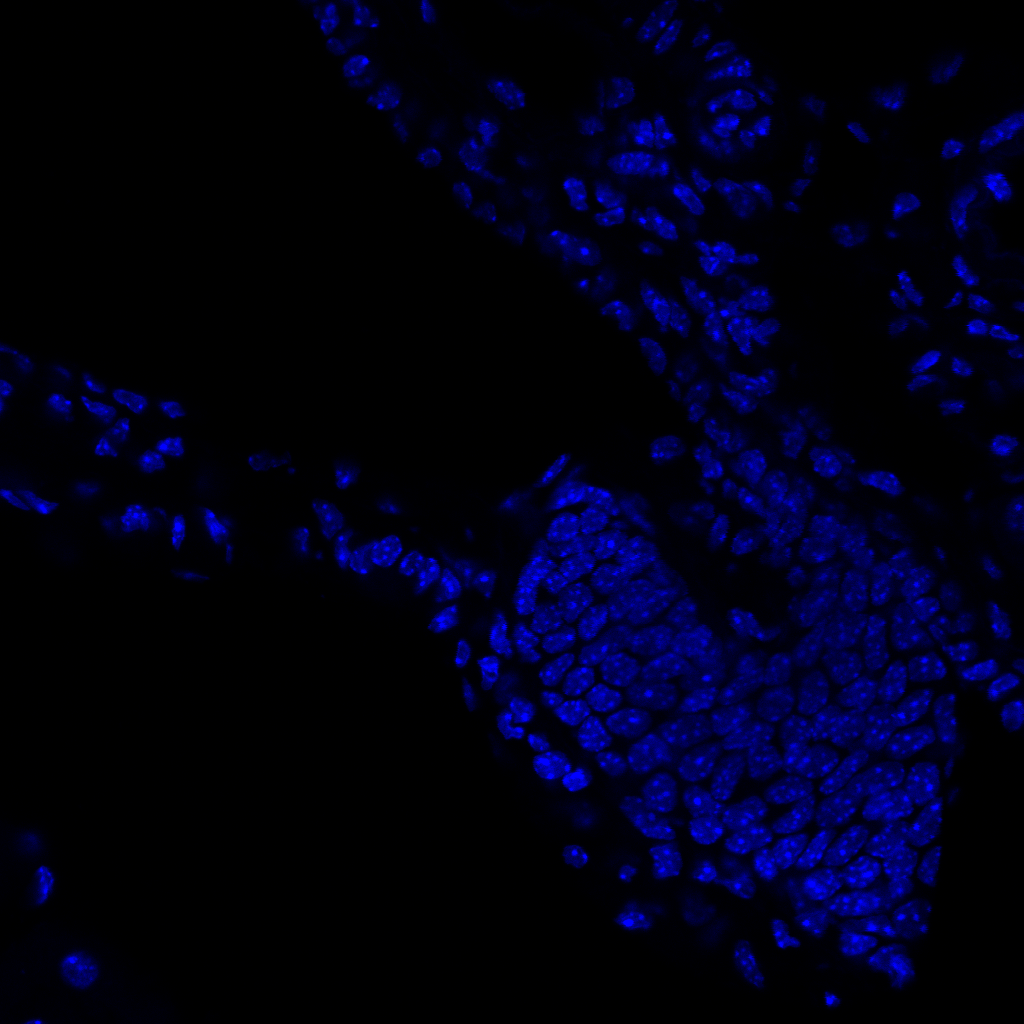

Supplement: Supplementary file 4 — Source data Fig. 2 [file 44318_2025_434_MOESM4_ESM.zip › Figure 2/2K/2K_2w_Ins (blue).tif]
