# Supplementary figures and images for: Ductal or Ngn3+ cells do not contribute to adult pancreatic islet beta-cell neogenesis in homeostasis (part 2 of 5)
Source: EMBO J. 2025 Apr 9;44(10):2856–81. doi: 10.1038/s44318-025-00434-z (PMC12084597; doi:10.1038/s44318-025-00434-z)

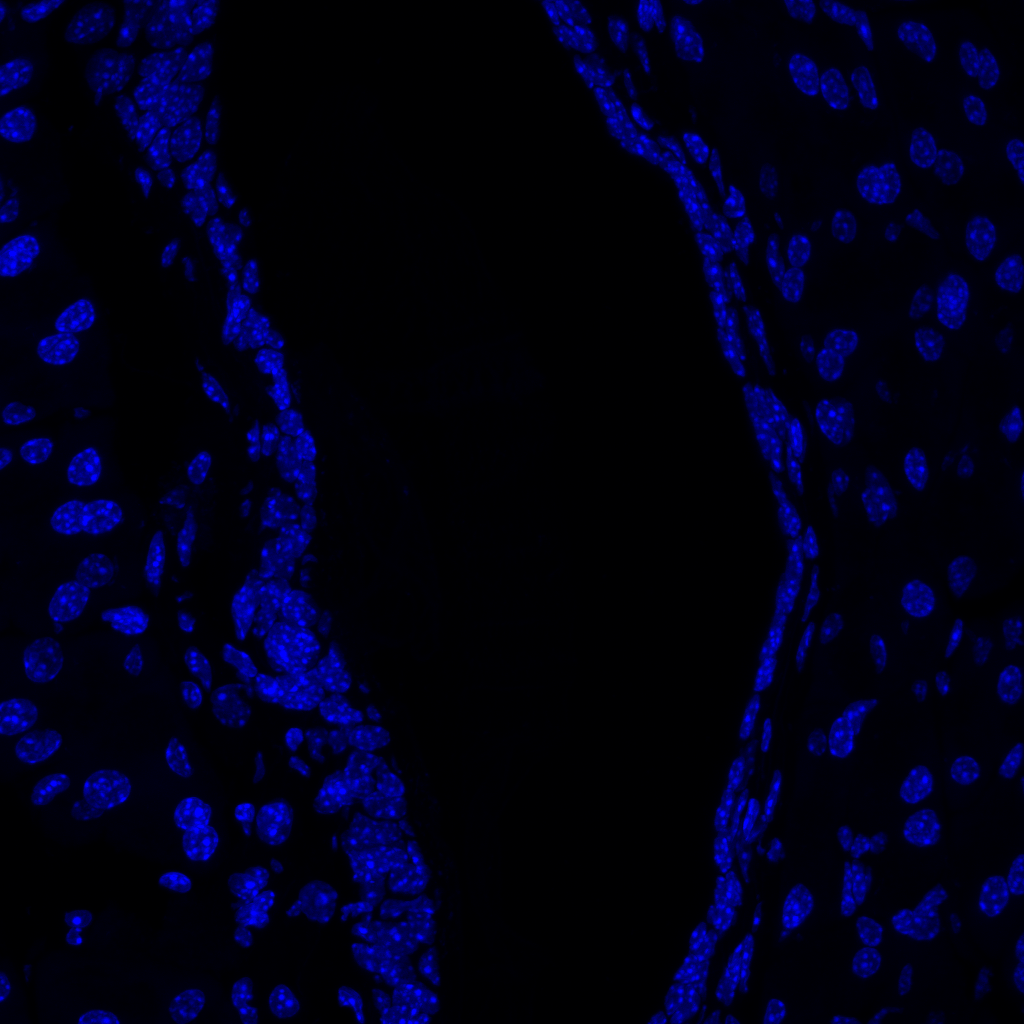

Supplement: Supplementary file 4 — Source data Fig. 2 [file 44318_2025_434_MOESM4_ESM.zip › Figure 2/2K/2K_12w_CK19 (blue).tif]

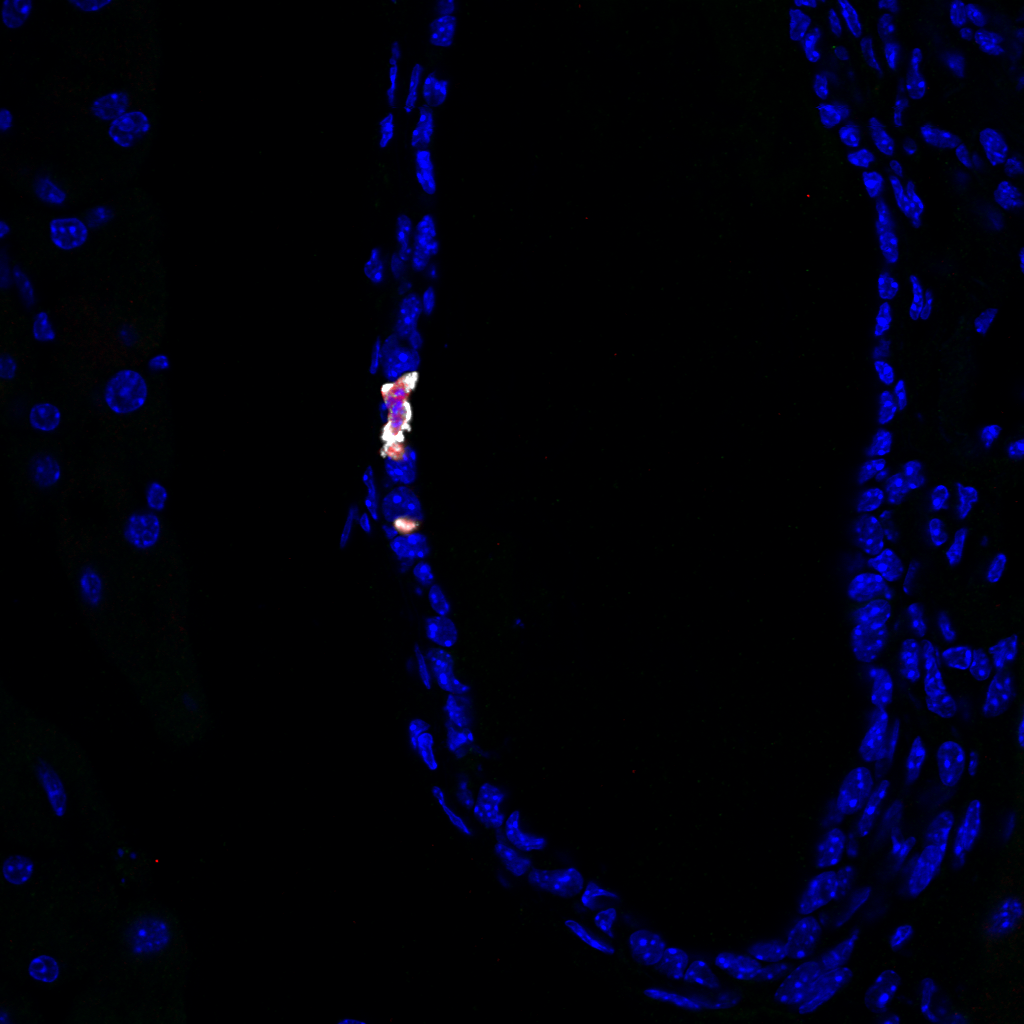

Supplement: Supplementary file 4 — Source data Fig. 2 [file 44318_2025_434_MOESM4_ESM.zip › Figure 2/2K/2K_12w_Sst.tif]

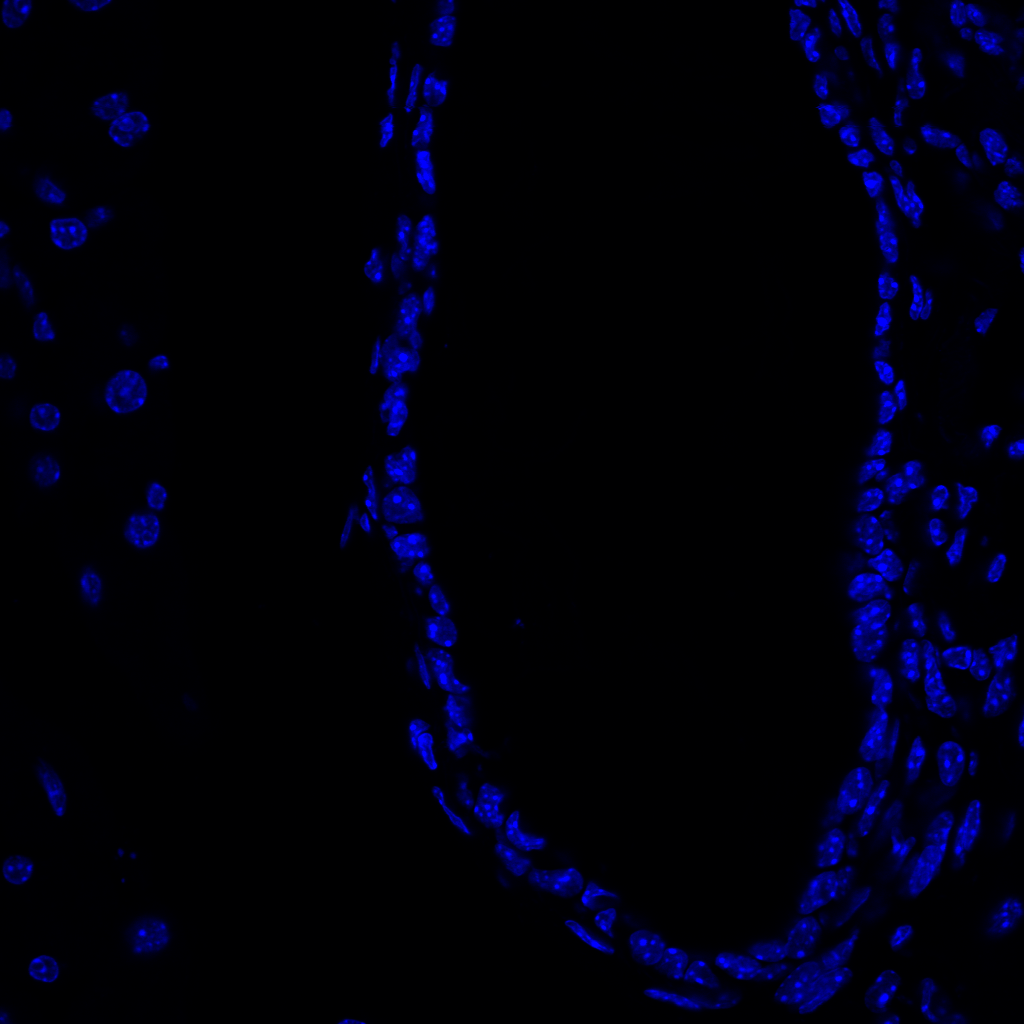

Supplement: Supplementary file 4 — Source data Fig. 2 [file 44318_2025_434_MOESM4_ESM.zip › Figure 2/2K/2K_12w_Sst (blue).tif]

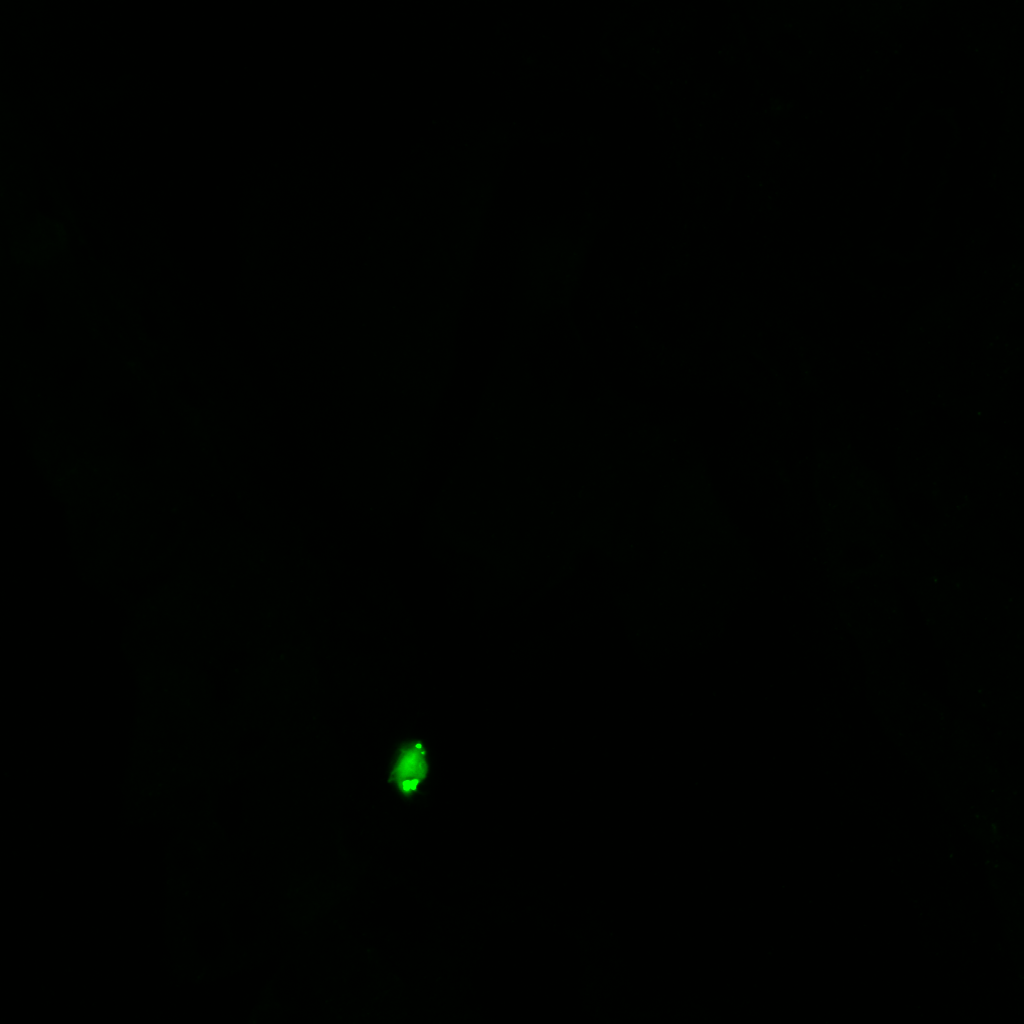

Supplement: Supplementary file 4 — Source data Fig. 2 [file 44318_2025_434_MOESM4_ESM.zip › Figure 2/2K/2K_12w_Ins (green).tif]

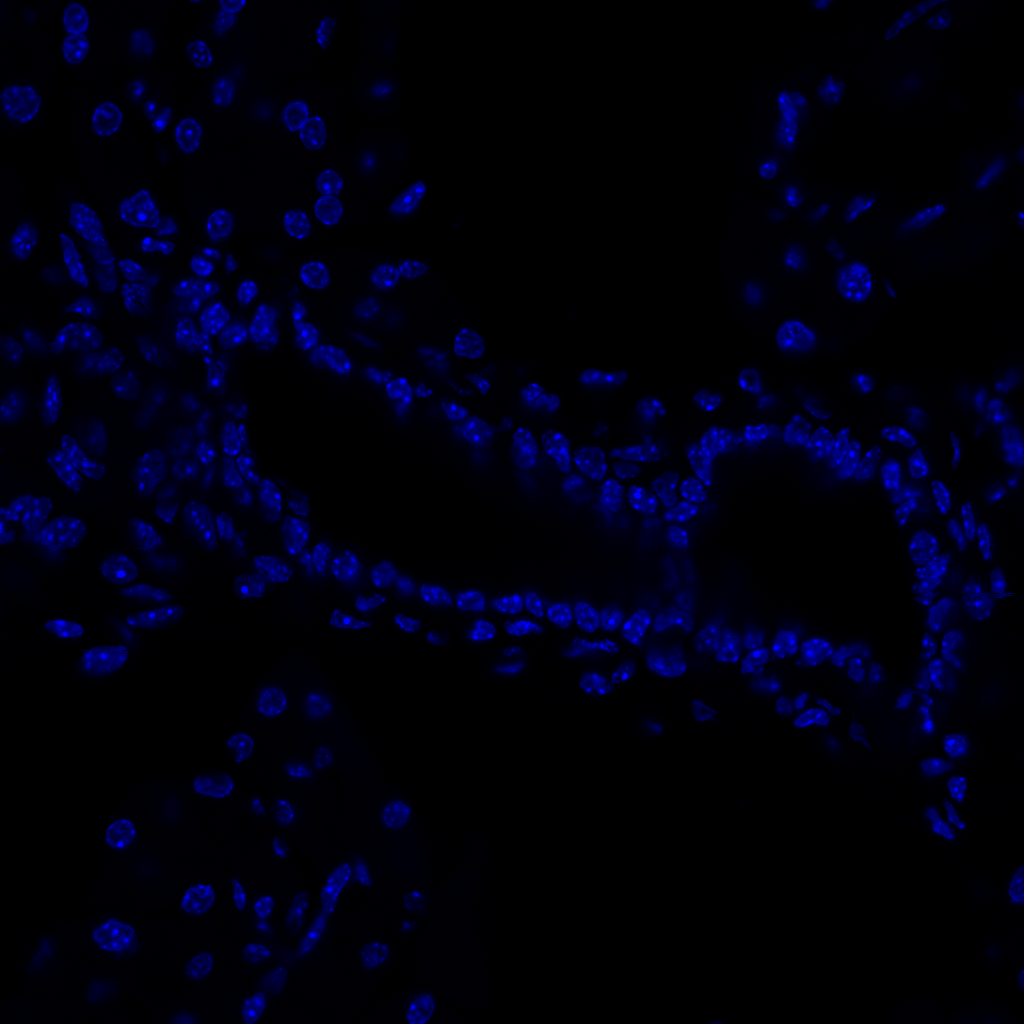

Supplement: Supplementary file 4 — Source data Fig. 2 [file 44318_2025_434_MOESM4_ESM.zip › Figure 2/2K/2K_2w_CK19 (blue).tif]

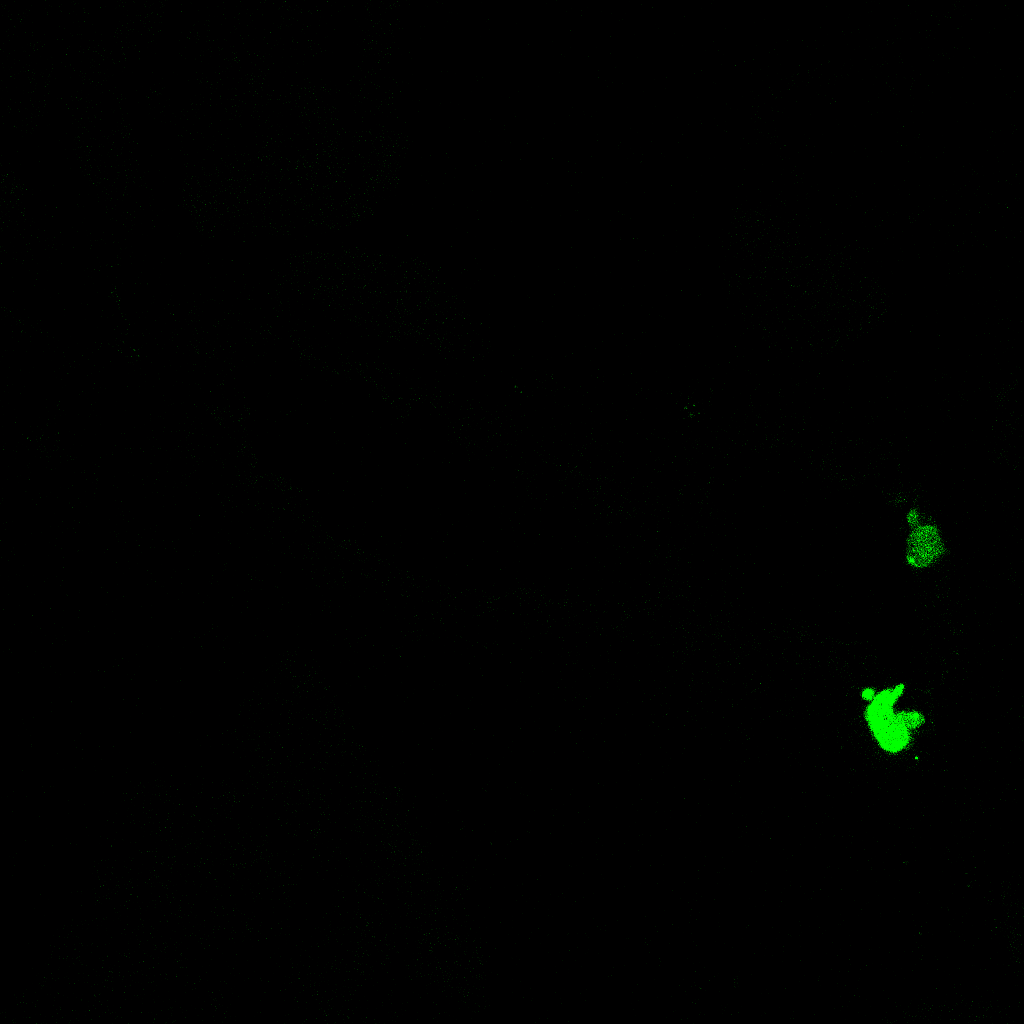

Supplement: Supplementary file 4 — Source data Fig. 2 [file 44318_2025_434_MOESM4_ESM.zip › Figure 2/2K/2K_2w_CK19 (green).tif]

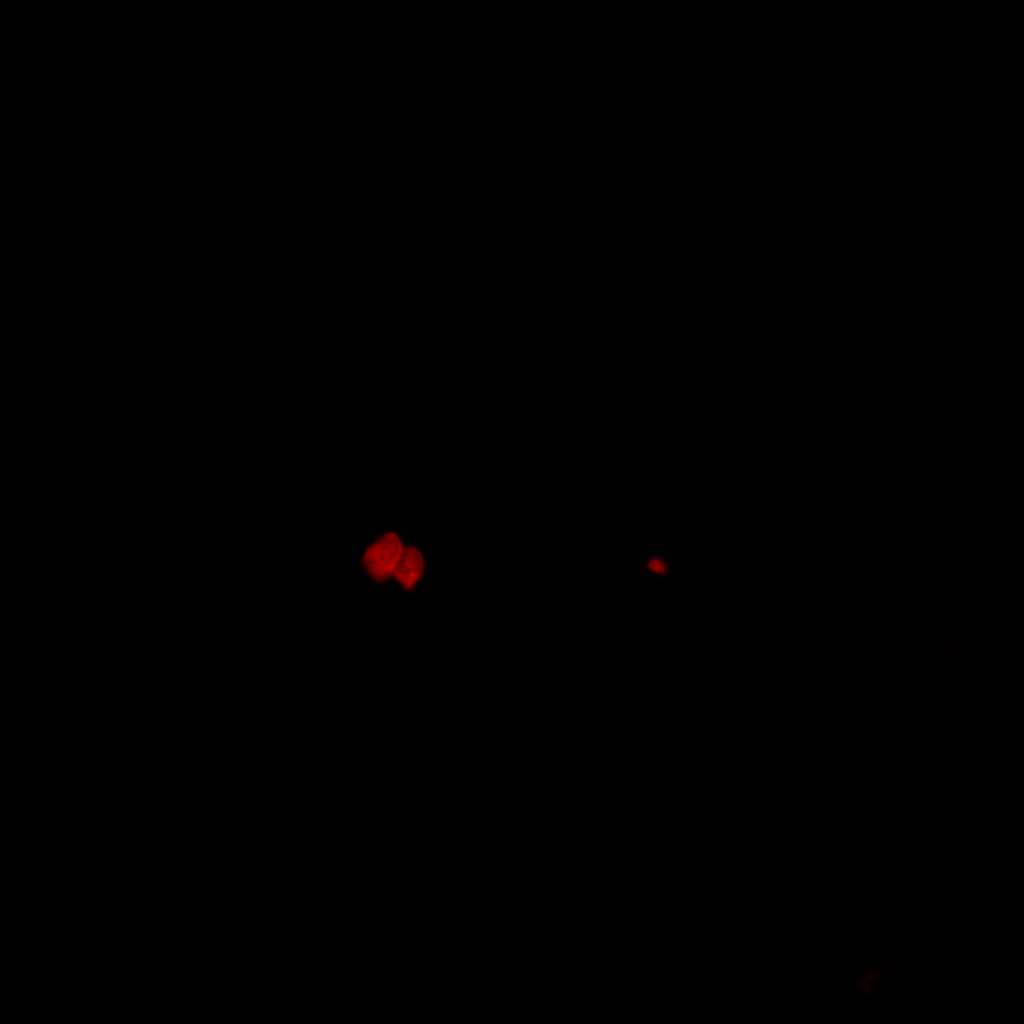

Supplement: Supplementary file 4 — Source data Fig. 2 [file 44318_2025_434_MOESM4_ESM.zip › Figure 2/2K/2K_2w_Ins (red).tif]

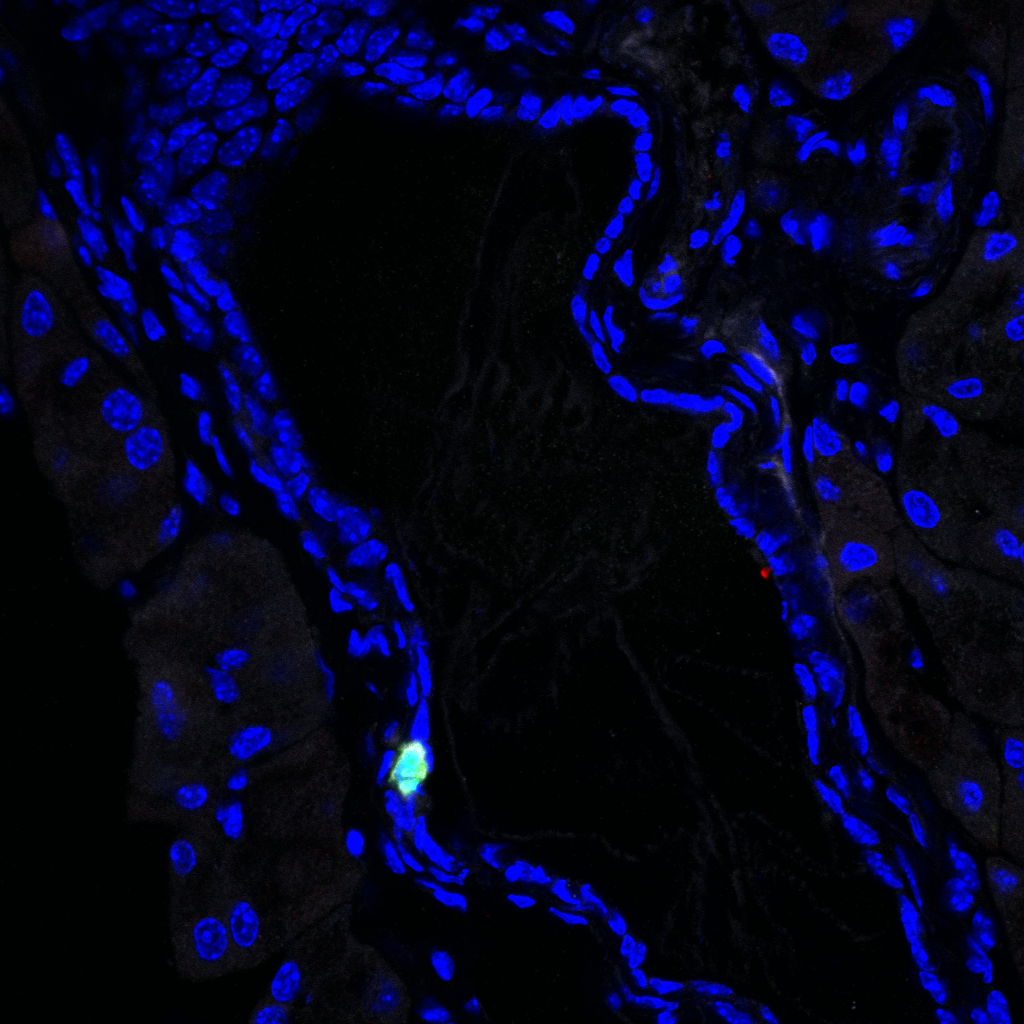

Supplement: Supplementary file 4 — Source data Fig. 2 [file 44318_2025_434_MOESM4_ESM.zip › Figure 2/2K/2K_12w_Ins.tif]

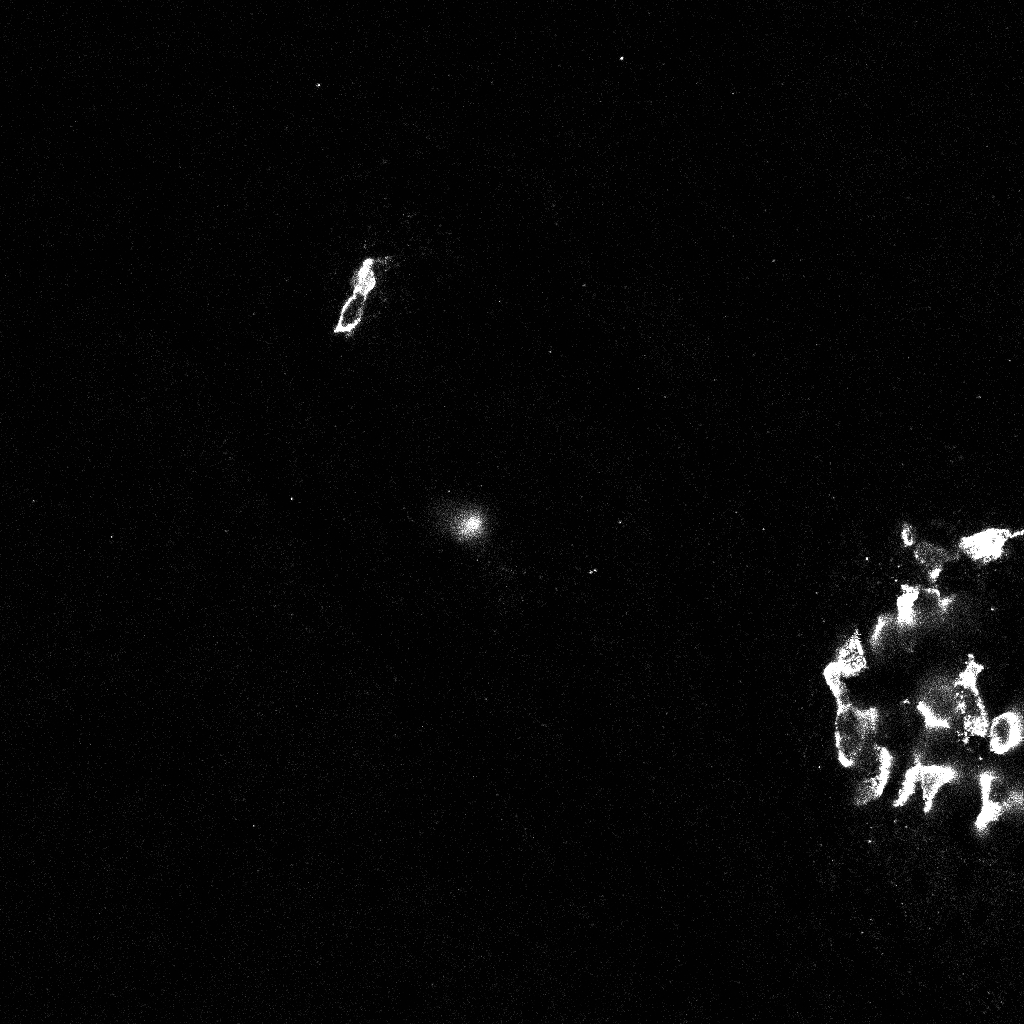

Supplement: Supplementary file 4 — Source data Fig. 2 [file 44318_2025_434_MOESM4_ESM.zip › Figure 2/2K/2K_2w_Sst (gray).tif]

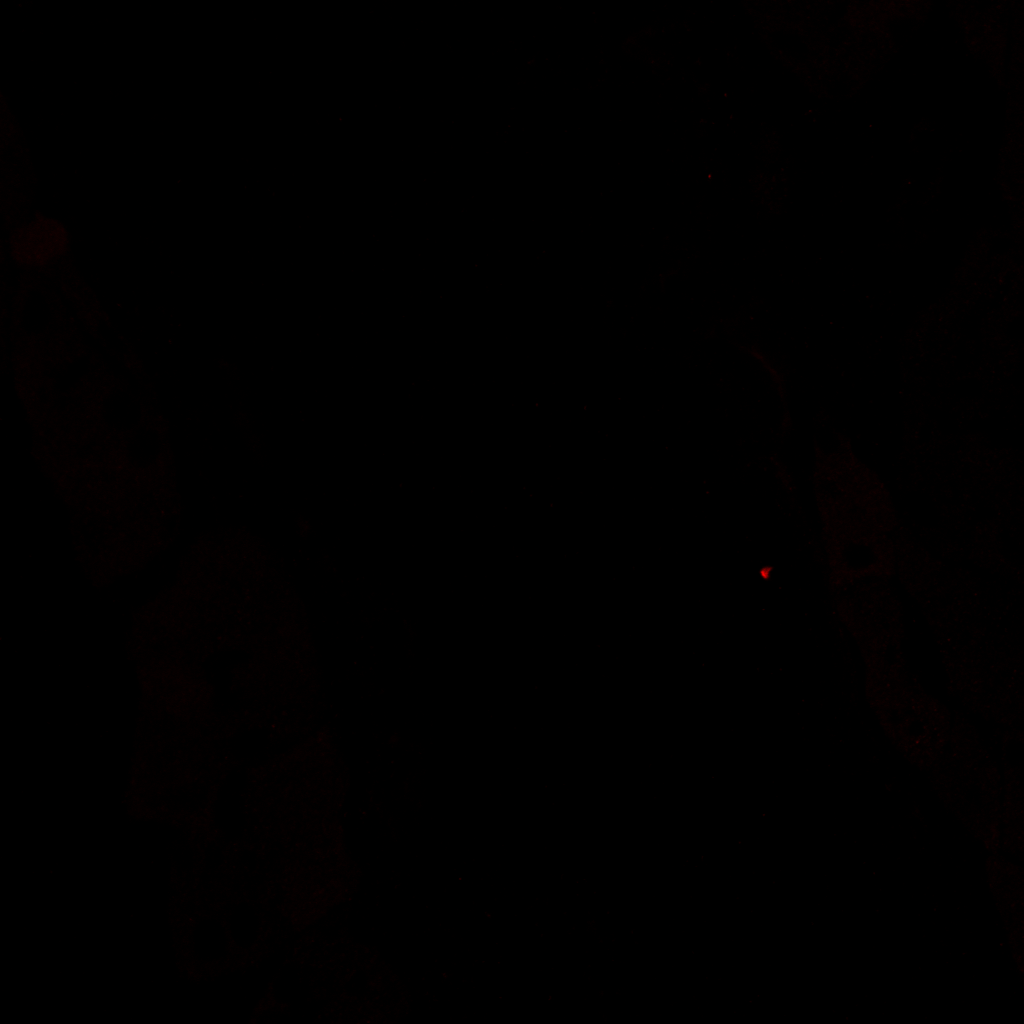

Supplement: Supplementary file 4 — Source data Fig. 2 [file 44318_2025_434_MOESM4_ESM.zip › Figure 2/2K/2K_12w_Ins (red).tif]

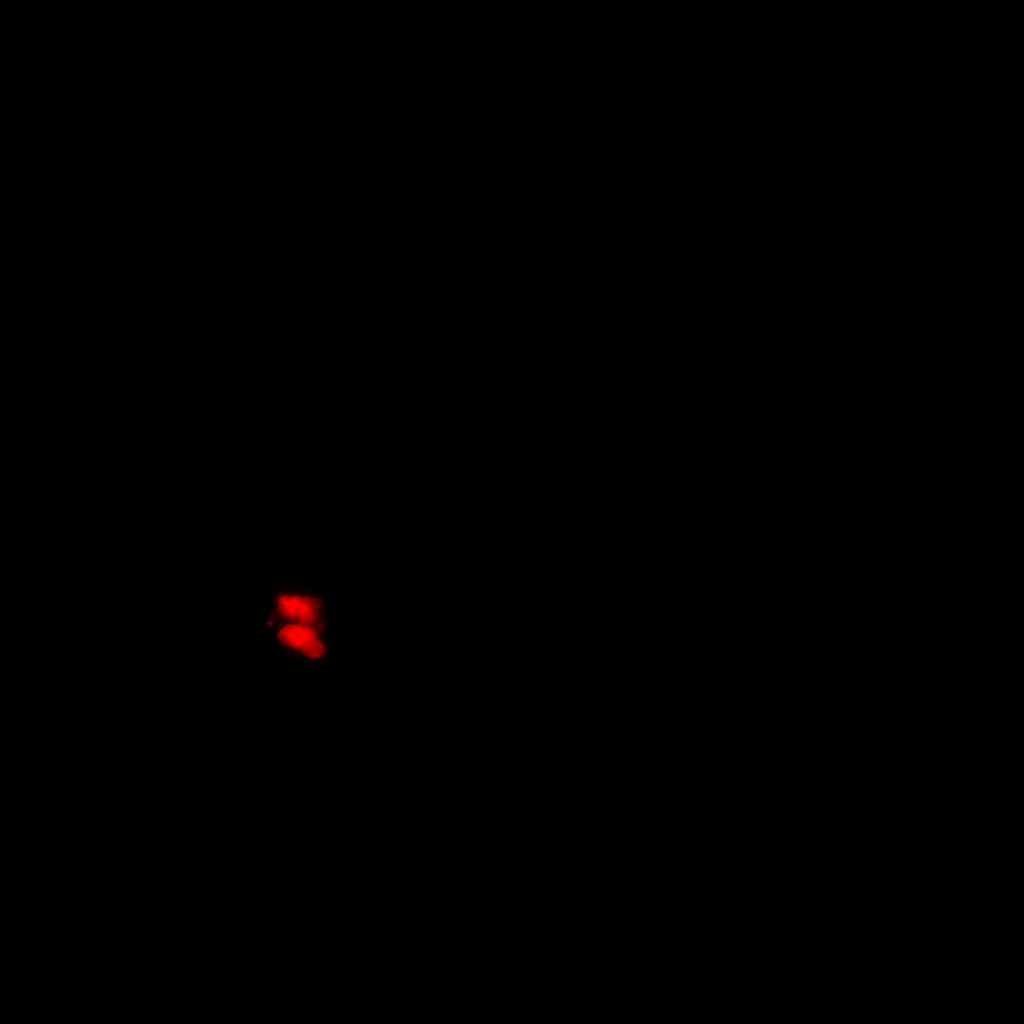

Supplement: Supplementary file 4 — Source data Fig. 2 [file 44318_2025_434_MOESM4_ESM.zip › Figure 2/2K/2K_12w_CK19 (red).tif]

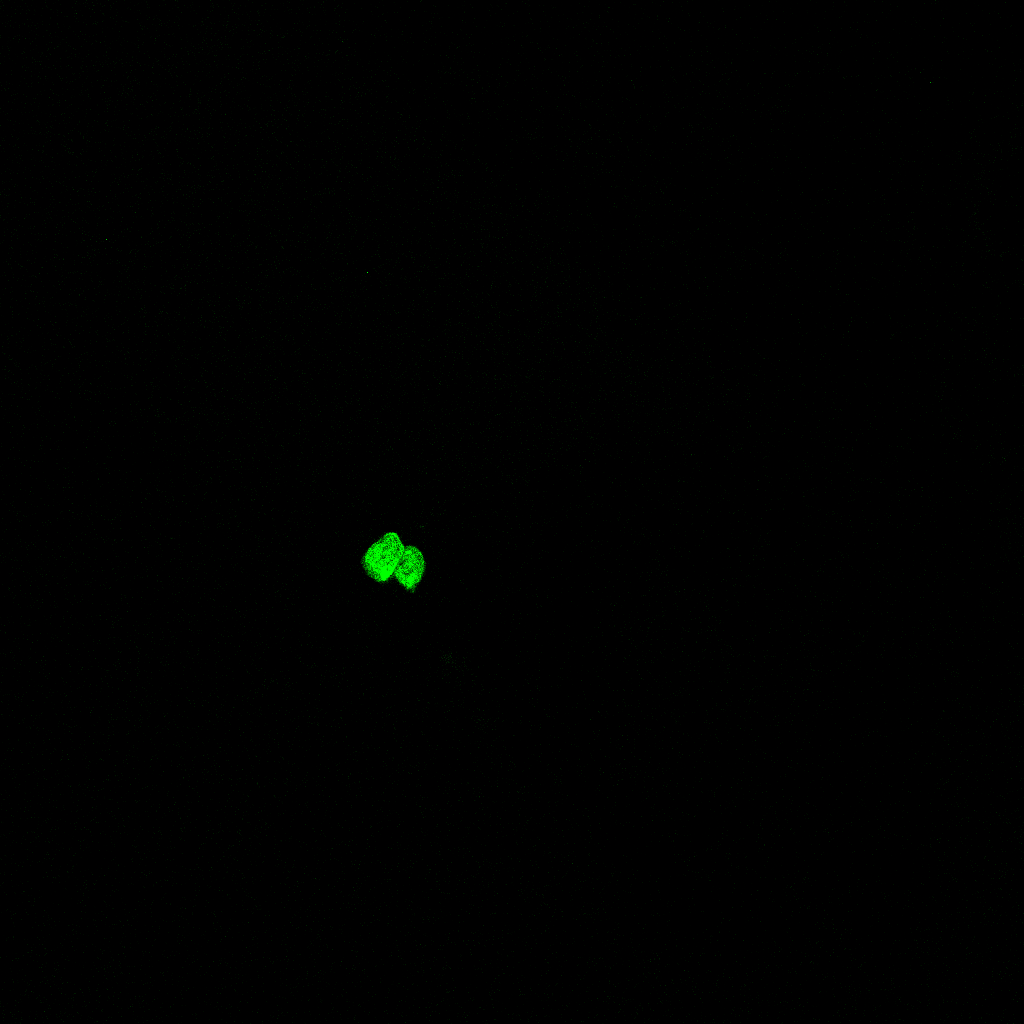

Supplement: Supplementary file 4 — Source data Fig. 2 [file 44318_2025_434_MOESM4_ESM.zip › Figure 2/2K/2K_2w_Ins (green).tif]

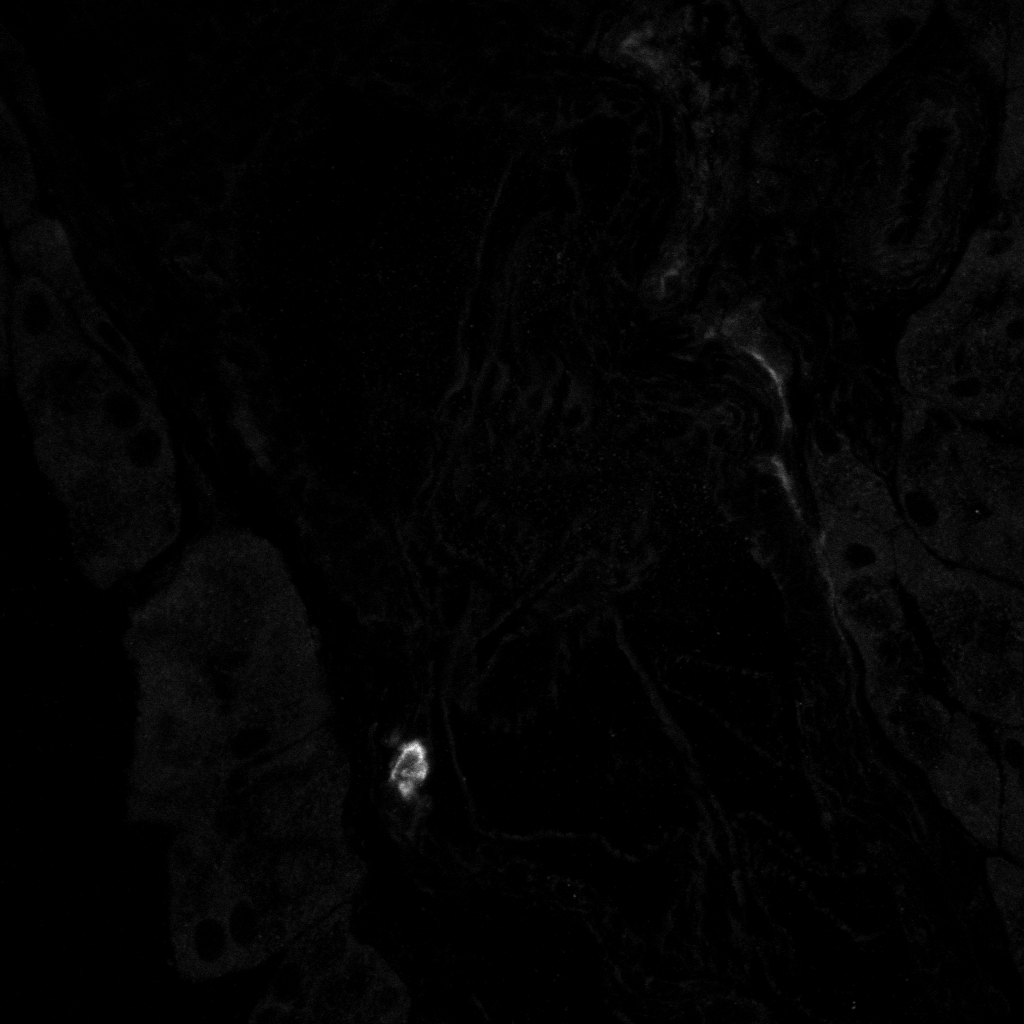

Supplement: Supplementary file 4 — Source data Fig. 2 [file 44318_2025_434_MOESM4_ESM.zip › Figure 2/2K/2K_12w_Ins (gray).tif]

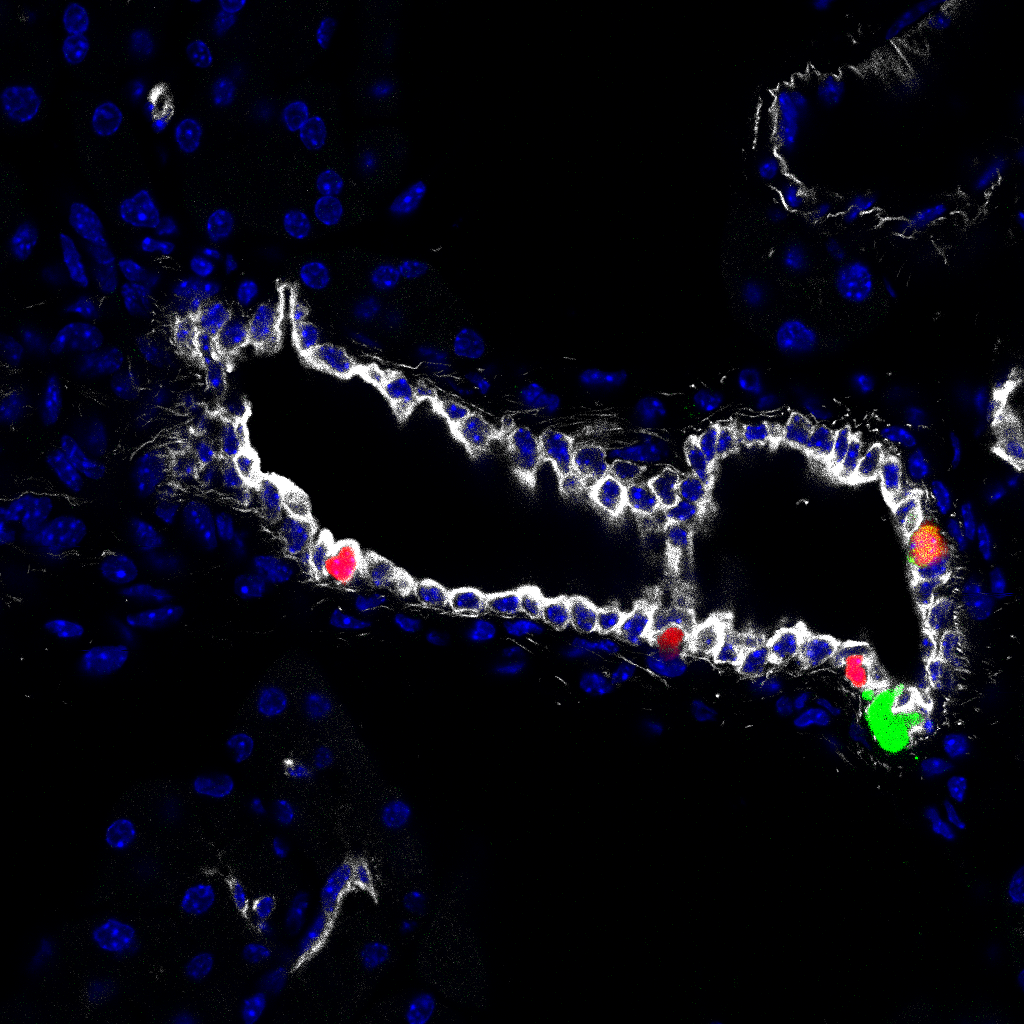

Supplement: Supplementary file 4 — Source data Fig. 2 [file 44318_2025_434_MOESM4_ESM.zip › Figure 2/2K/2K_2w_CK19..tif]

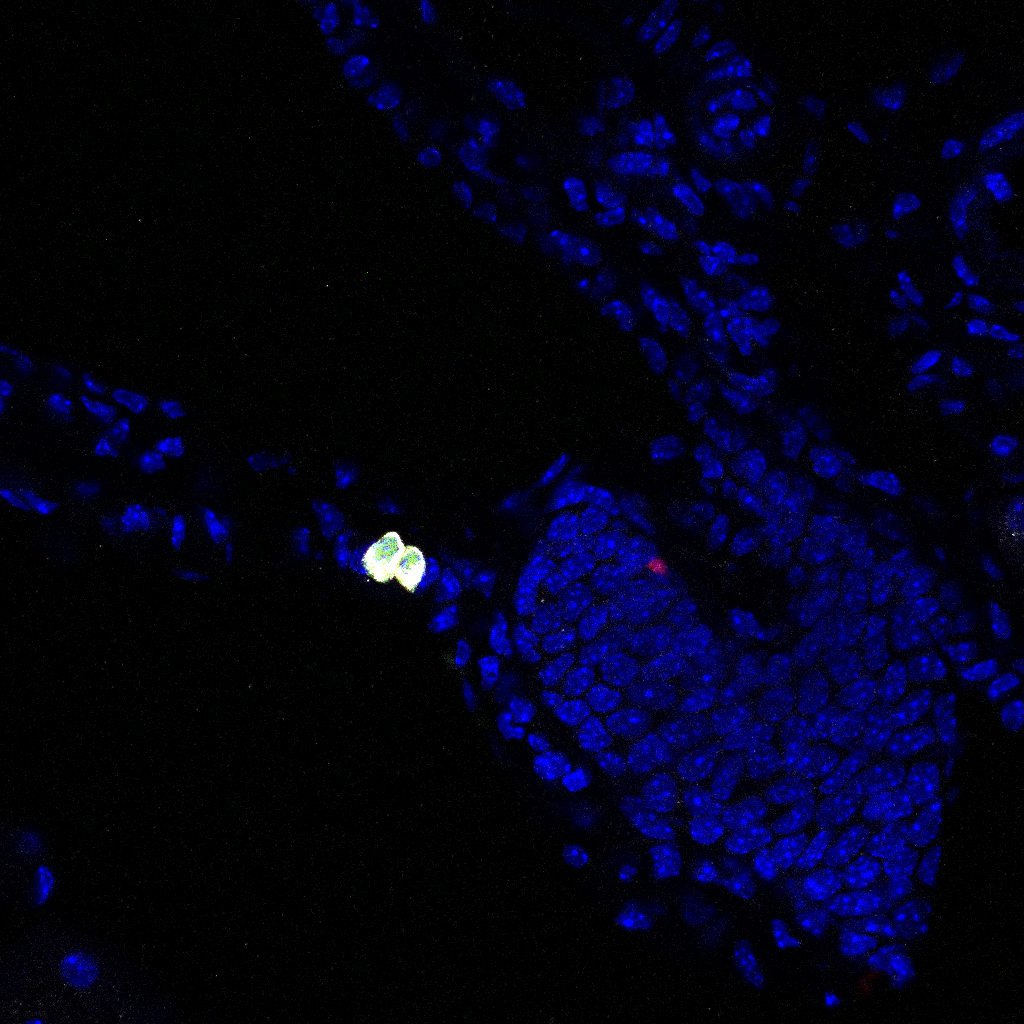

Supplement: Supplementary file 4 — Source data Fig. 2 [file 44318_2025_434_MOESM4_ESM.zip › Figure 2/2K/2K_2w_Ins.tif]

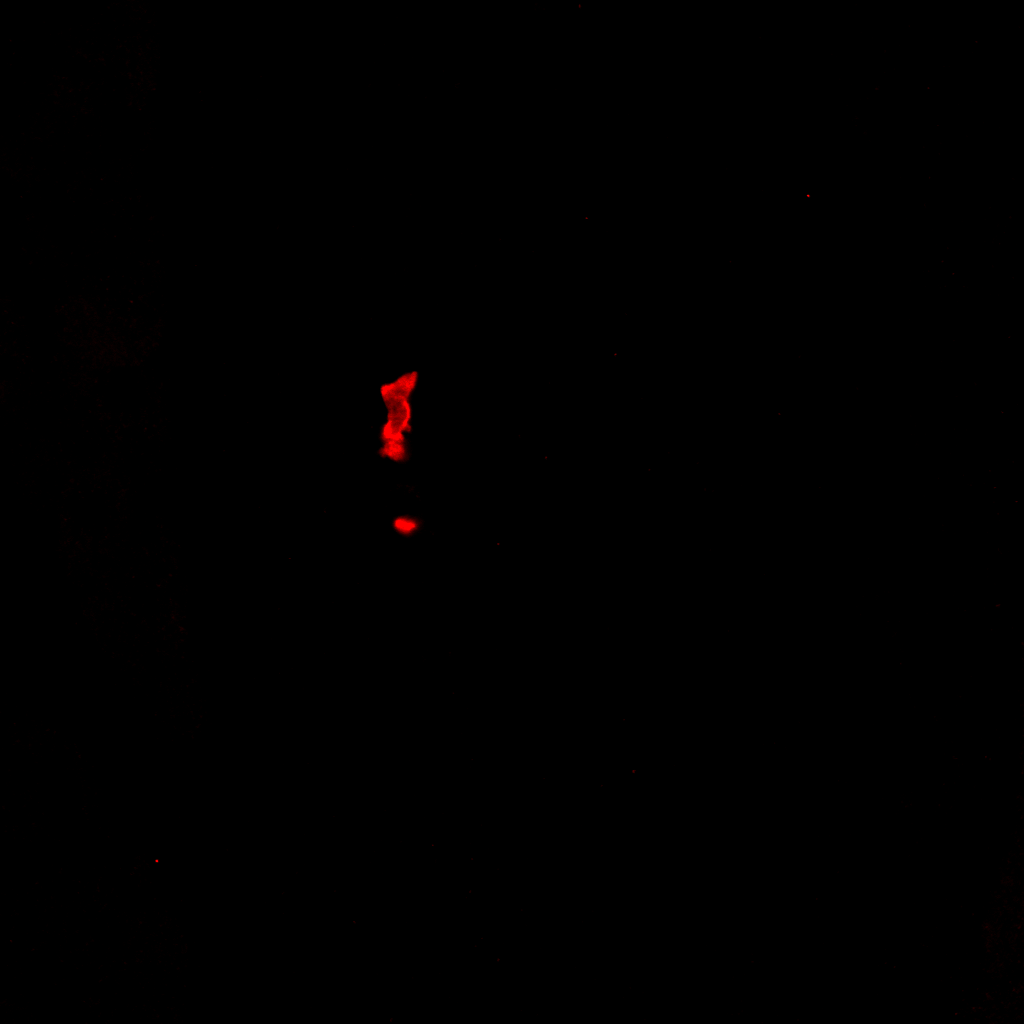

Supplement: Supplementary file 4 — Source data Fig. 2 [file 44318_2025_434_MOESM4_ESM.zip › Figure 2/2K/2K_12w_Sst.(red).tif]

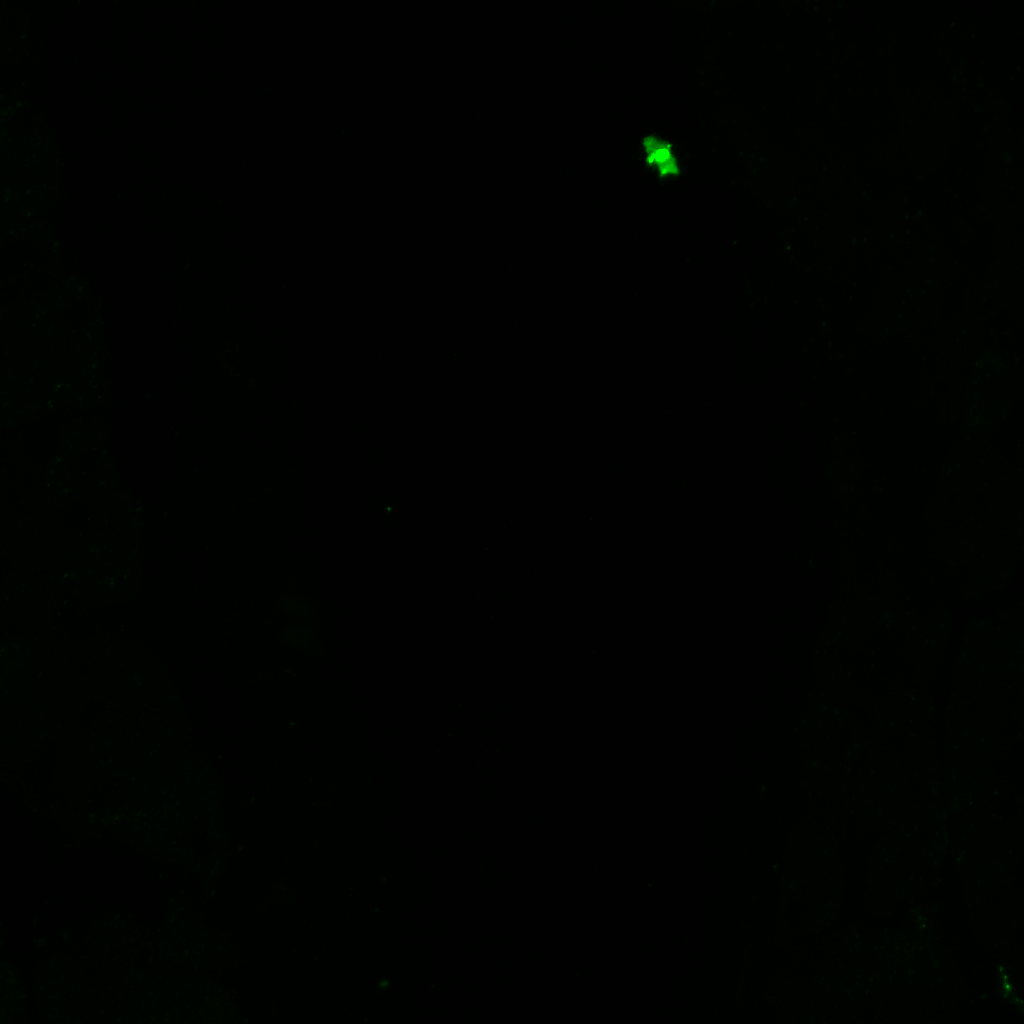

Supplement: Supplementary file 4 — Source data Fig. 2 [file 44318_2025_434_MOESM4_ESM.zip › Figure 2/2K/2K_12w_CK19 (green).tif]

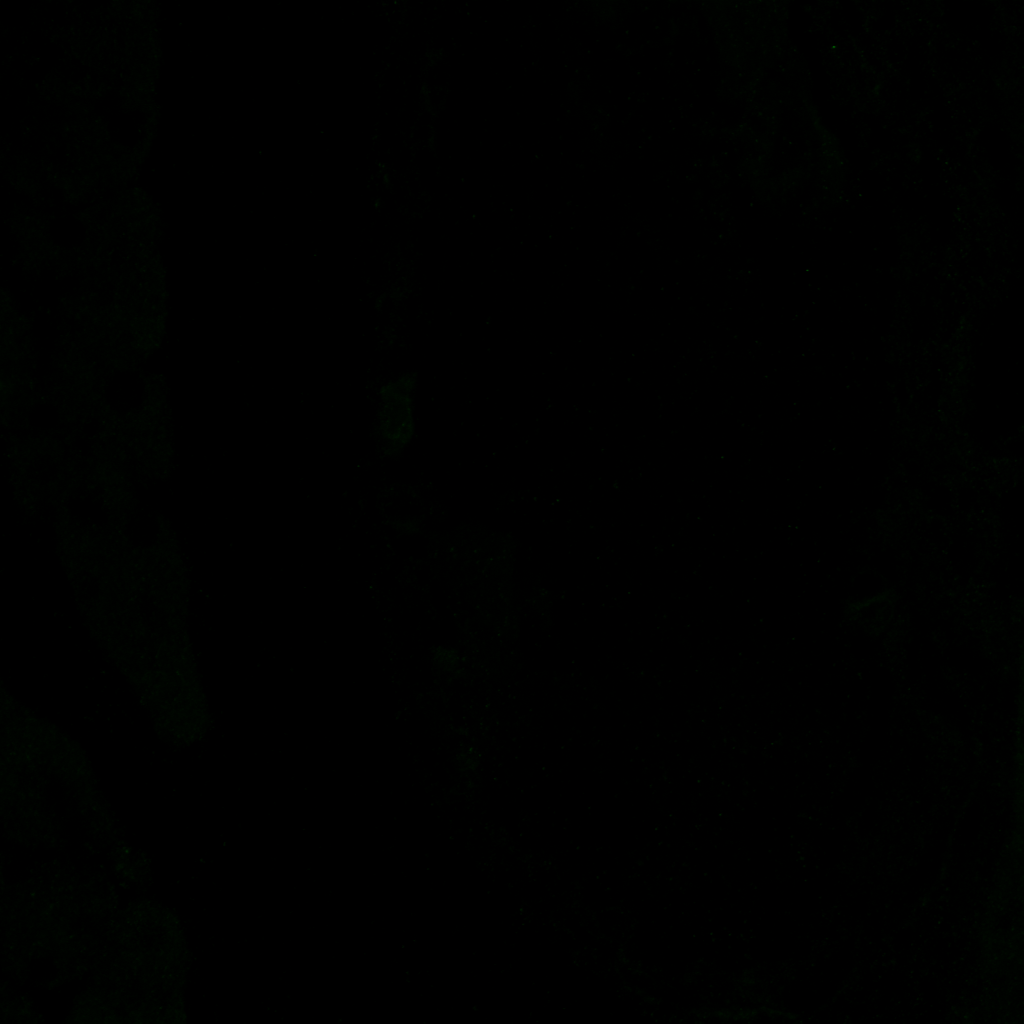

Supplement: Supplementary file 4 — Source data Fig. 2 [file 44318_2025_434_MOESM4_ESM.zip › Figure 2/2K/2K_12w_Sst (green).tif]

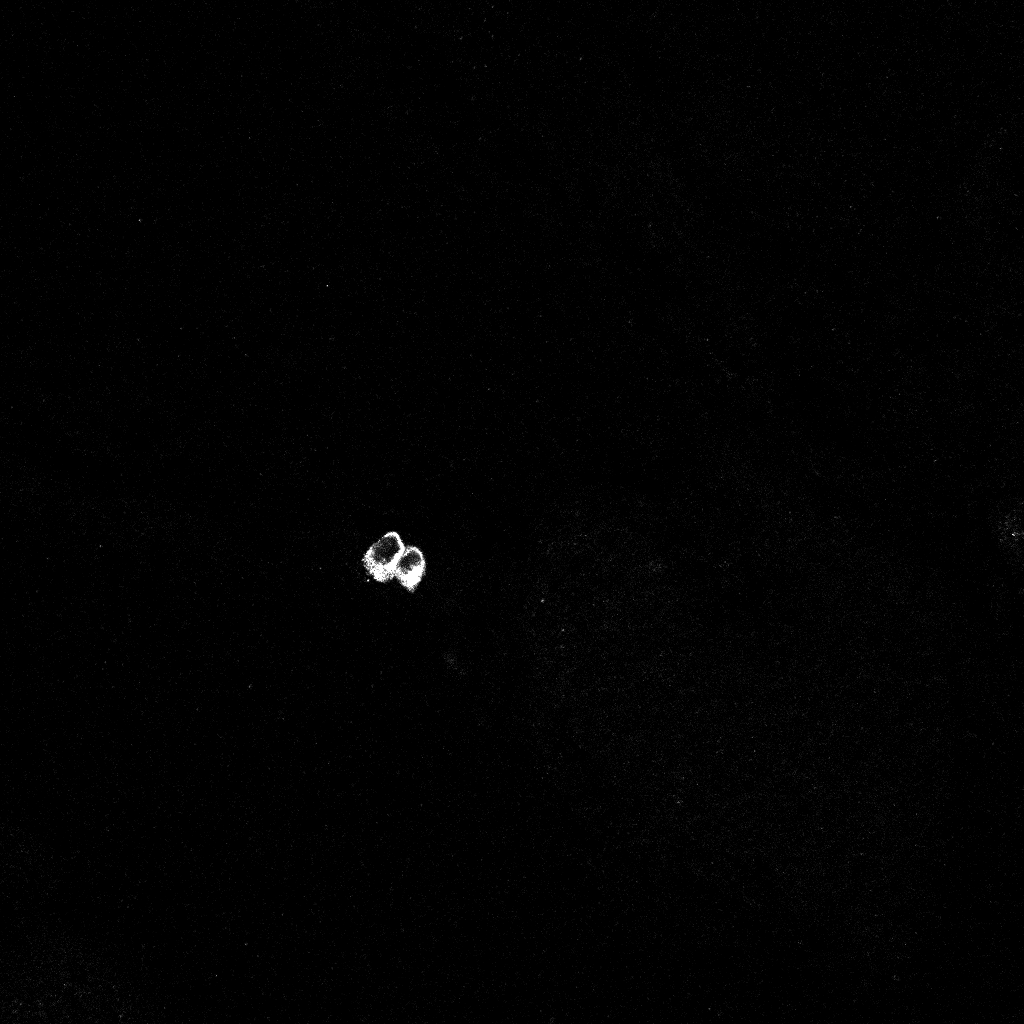

Supplement: Supplementary file 4 — Source data Fig. 2 [file 44318_2025_434_MOESM4_ESM.zip › Figure 2/2K/2K_2w_Ins (gray).tif]

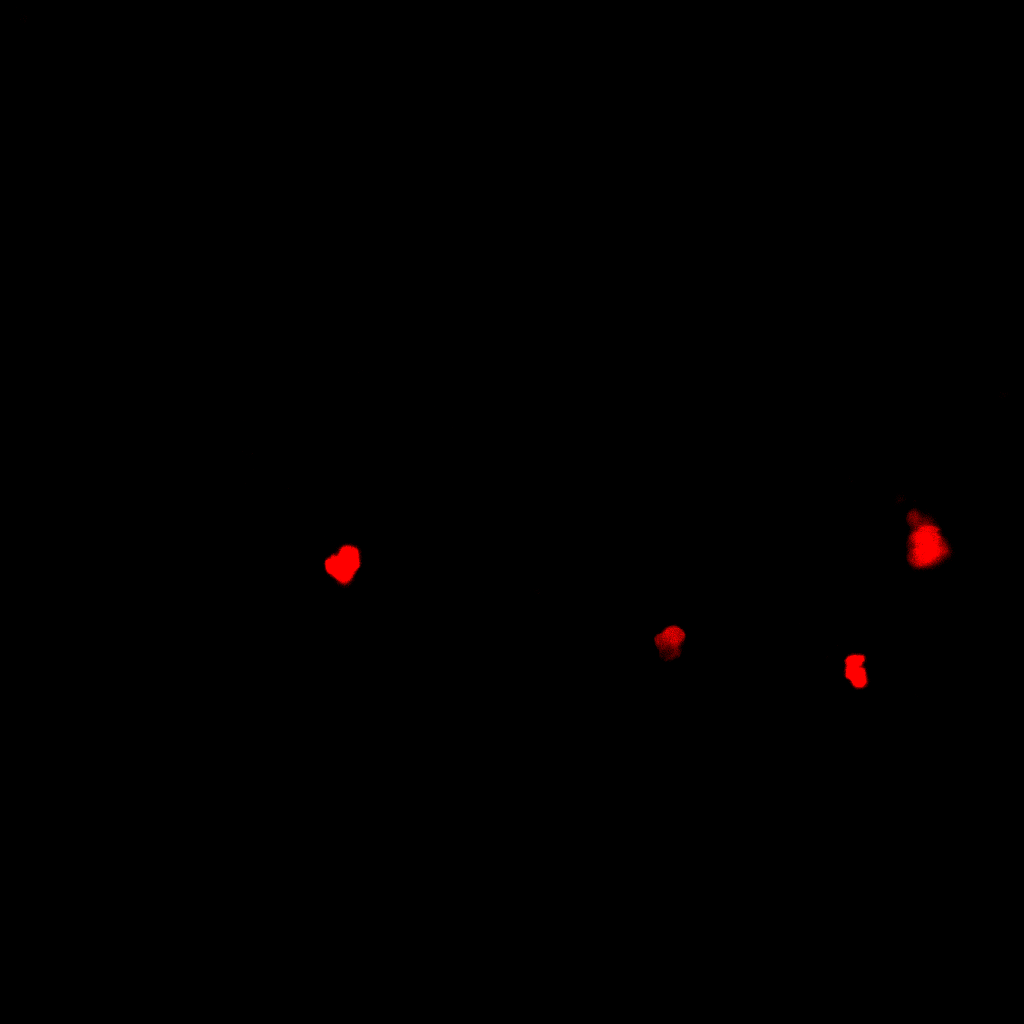

Supplement: Supplementary file 4 — Source data Fig. 2 [file 44318_2025_434_MOESM4_ESM.zip › Figure 2/2K/2K_2w_CK19 (red).tif]

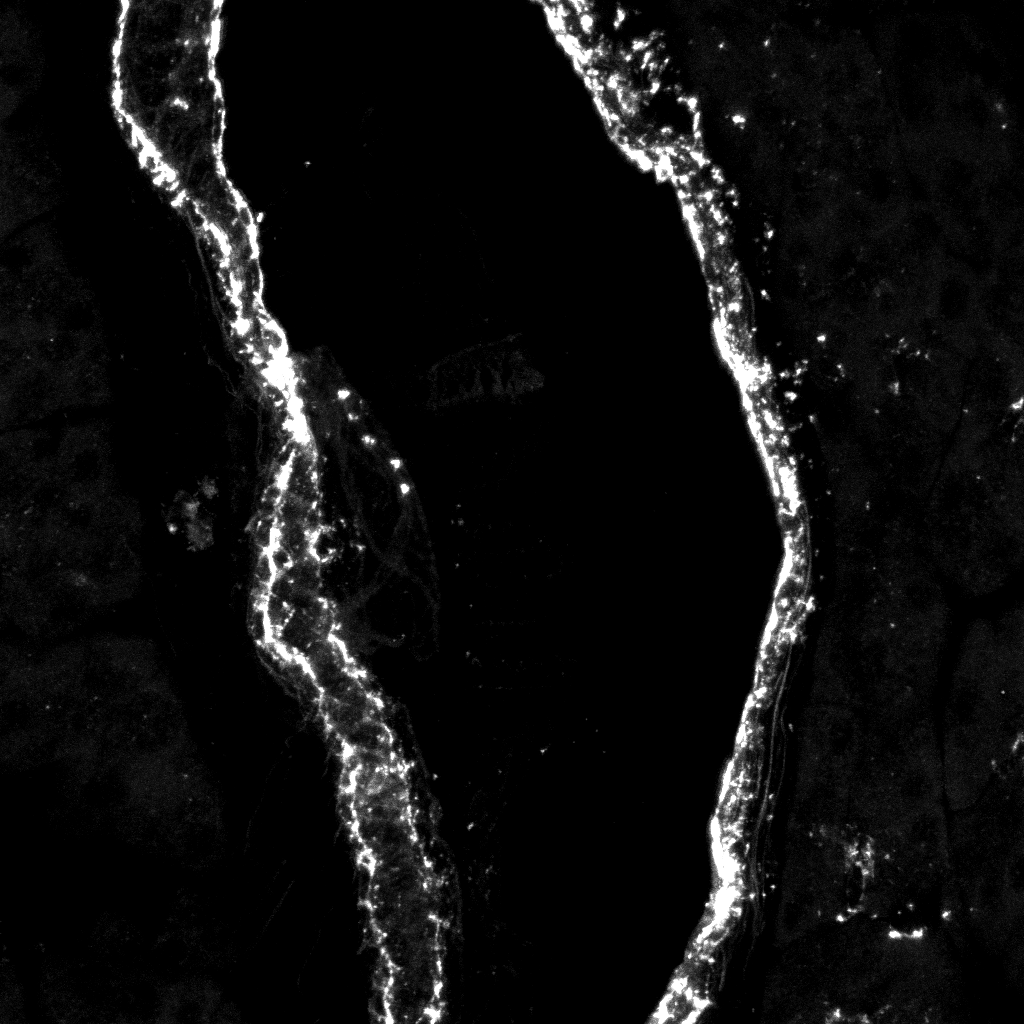

Supplement: Supplementary file 4 — Source data Fig. 2 [file 44318_2025_434_MOESM4_ESM.zip › Figure 2/2K/2K_12w_CK19 (gray).tif]

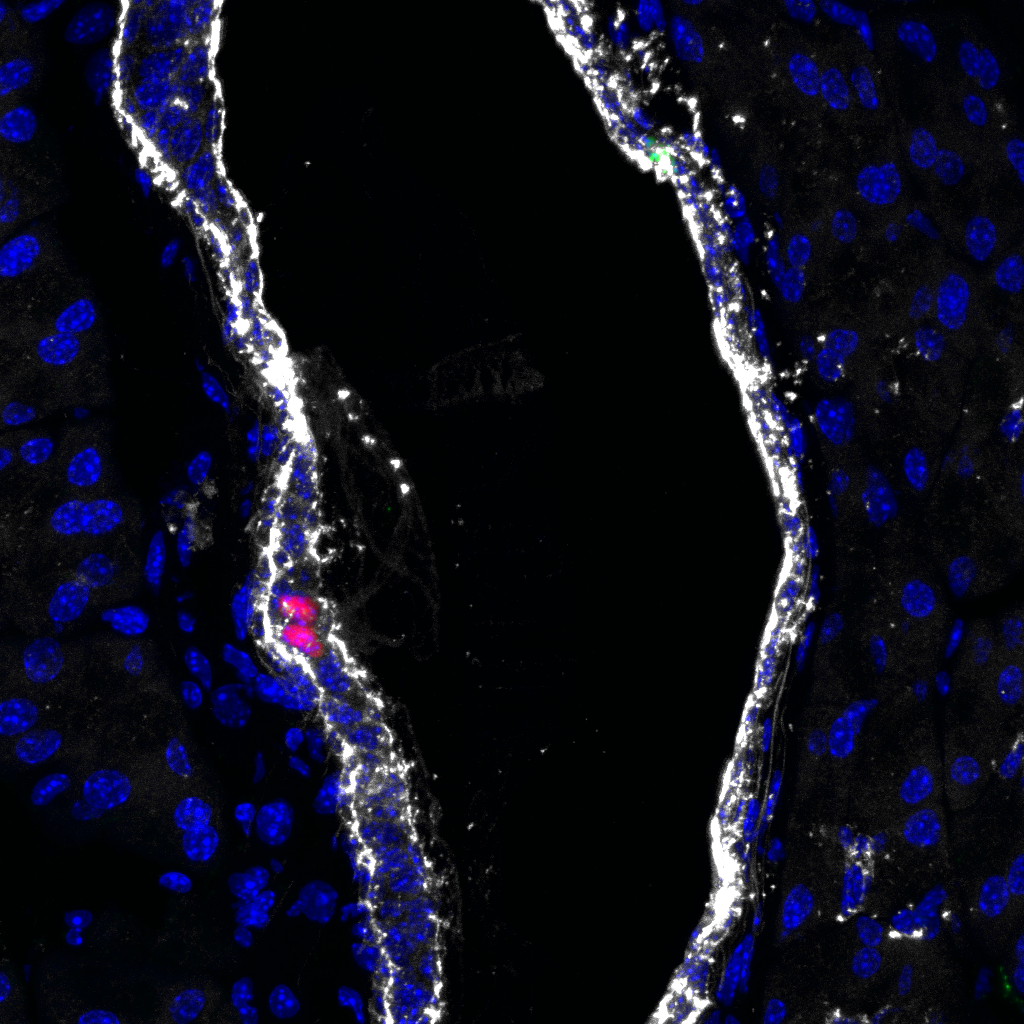

Supplement: Supplementary file 4 — Source data Fig. 2 [file 44318_2025_434_MOESM4_ESM.zip › Figure 2/2K/2K_12w_CK19.tif]

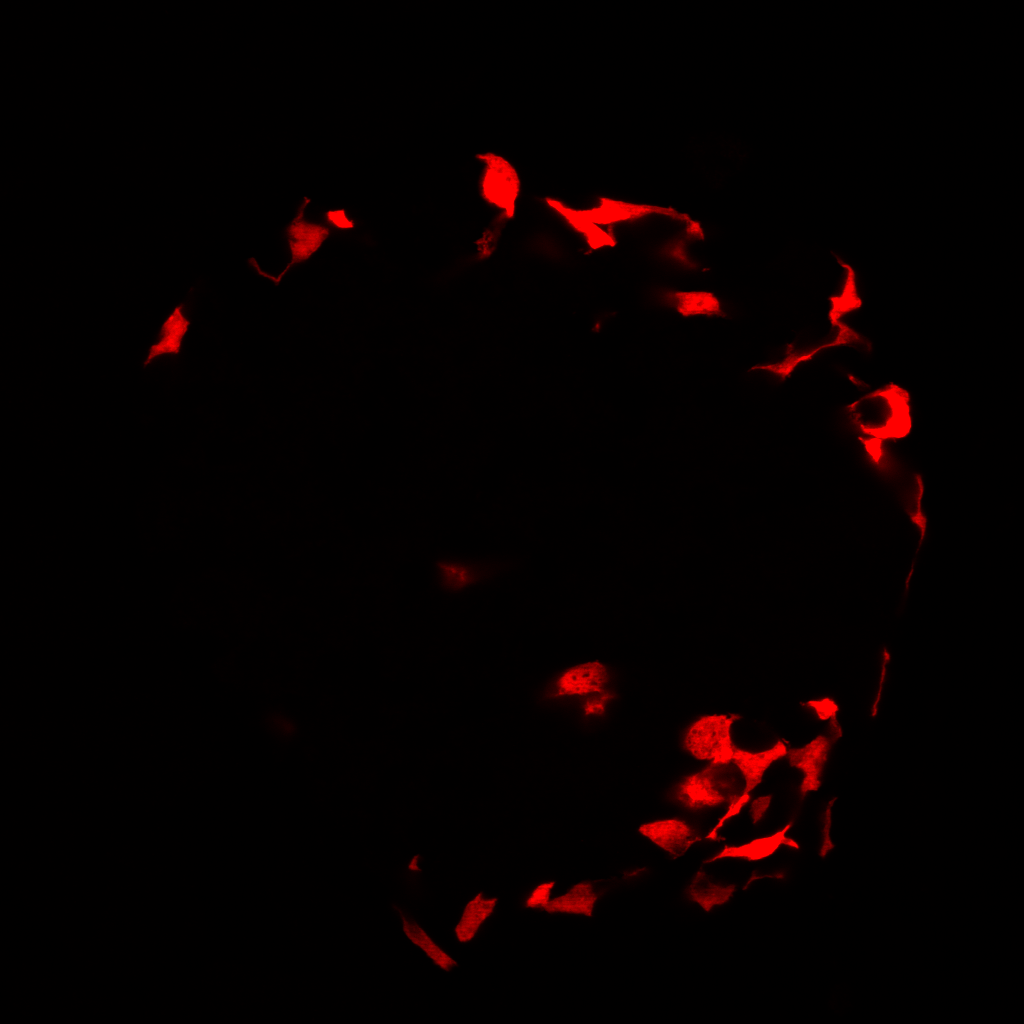

Supplement: Supplementary file 4 — Source data Fig. 2 [file 44318_2025_434_MOESM4_ESM.zip › Figure 2/2L/2L_12w_Sst (red).tif]

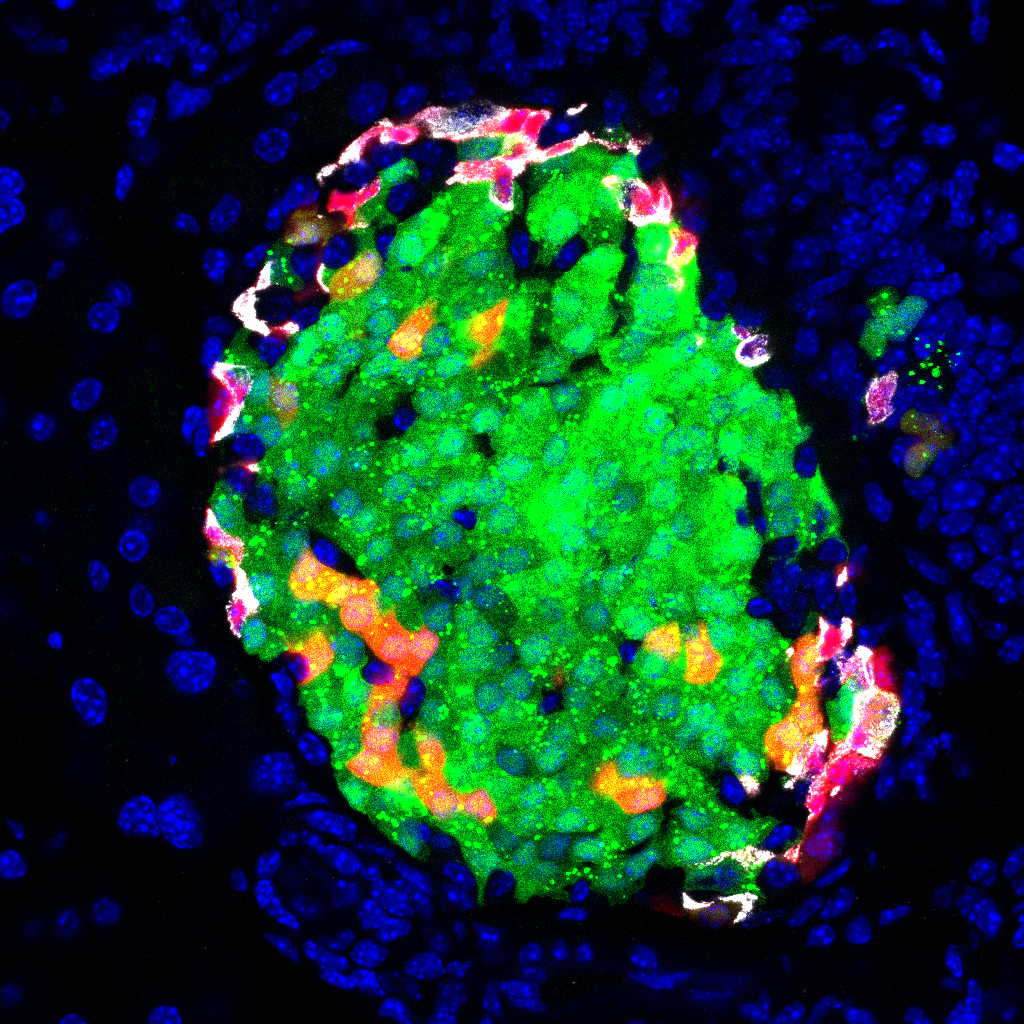

Supplement: Supplementary file 4 — Source data Fig. 2 [file 44318_2025_434_MOESM4_ESM.zip › Figure 2/2L/2L_2w_Sst.tif]

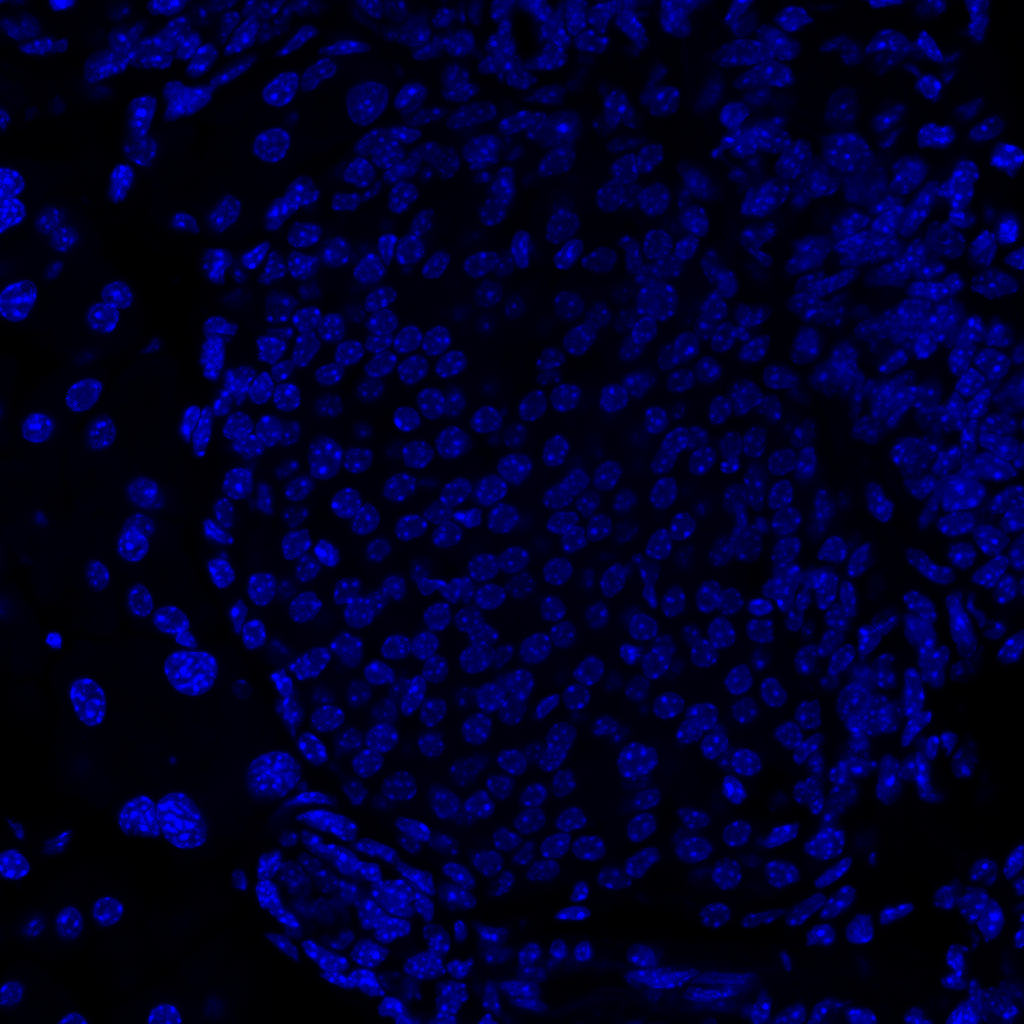

Supplement: Supplementary file 4 — Source data Fig. 2 [file 44318_2025_434_MOESM4_ESM.zip › Figure 2/2L/2L_2w_Sst (blue).tif]

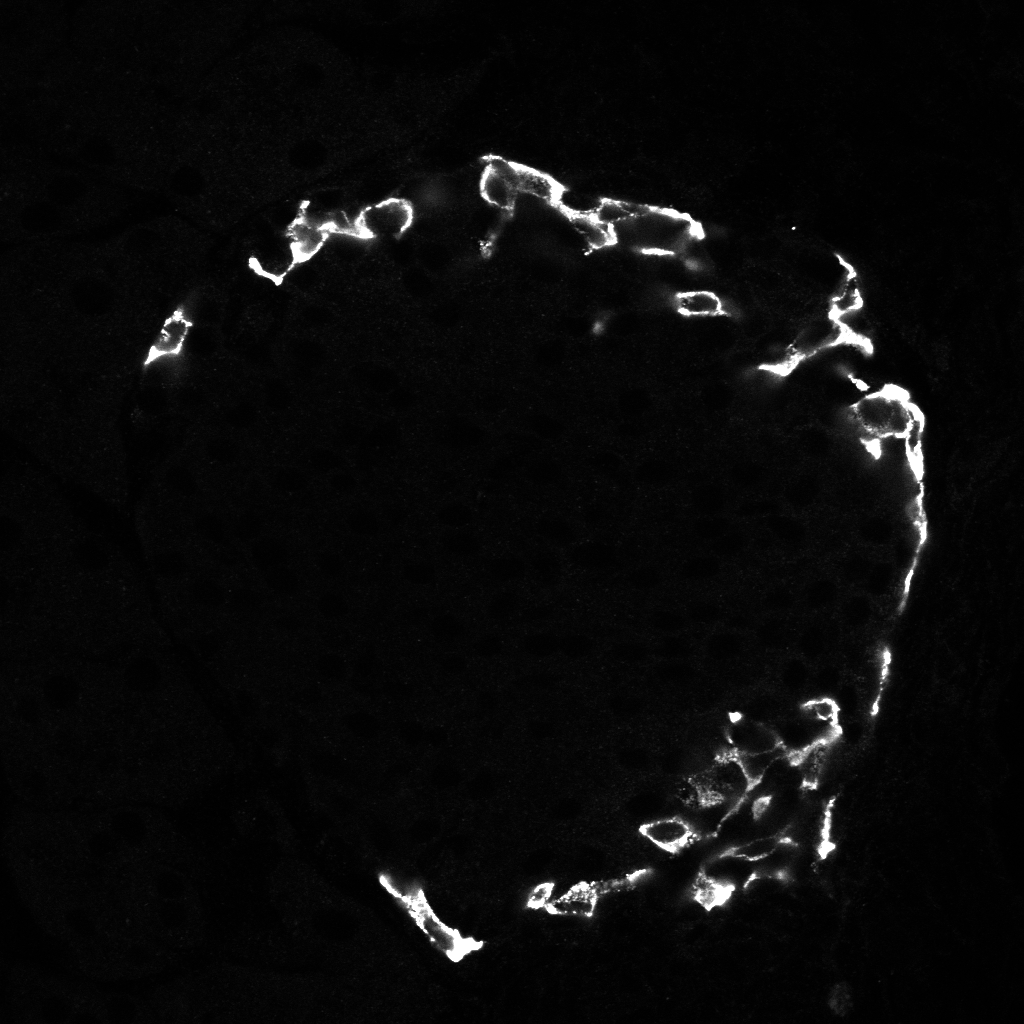

Supplement: Supplementary file 4 — Source data Fig. 2 [file 44318_2025_434_MOESM4_ESM.zip › Figure 2/2L/2L_12w_Sst (gray).tif]

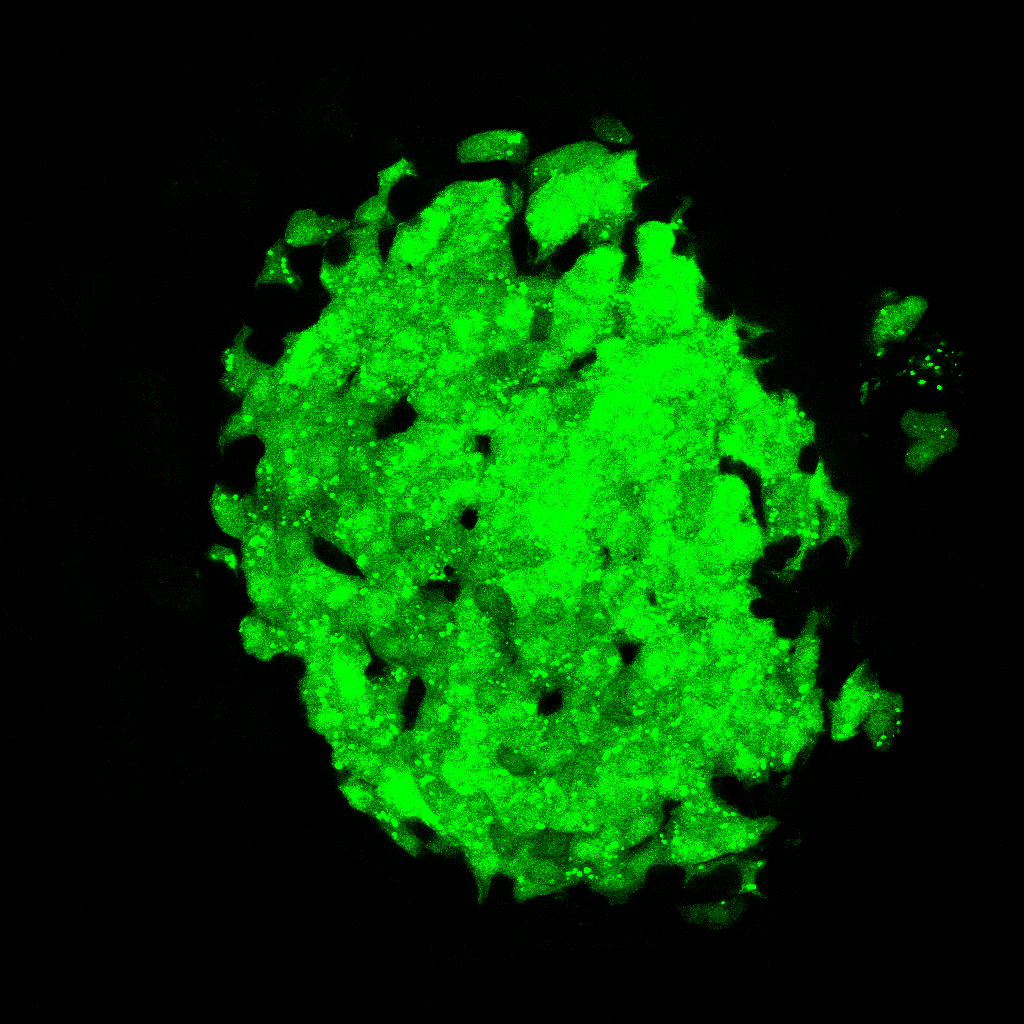

Supplement: Supplementary file 4 — Source data Fig. 2 [file 44318_2025_434_MOESM4_ESM.zip › Figure 2/2L/2L_2w_Sst (green).tif]

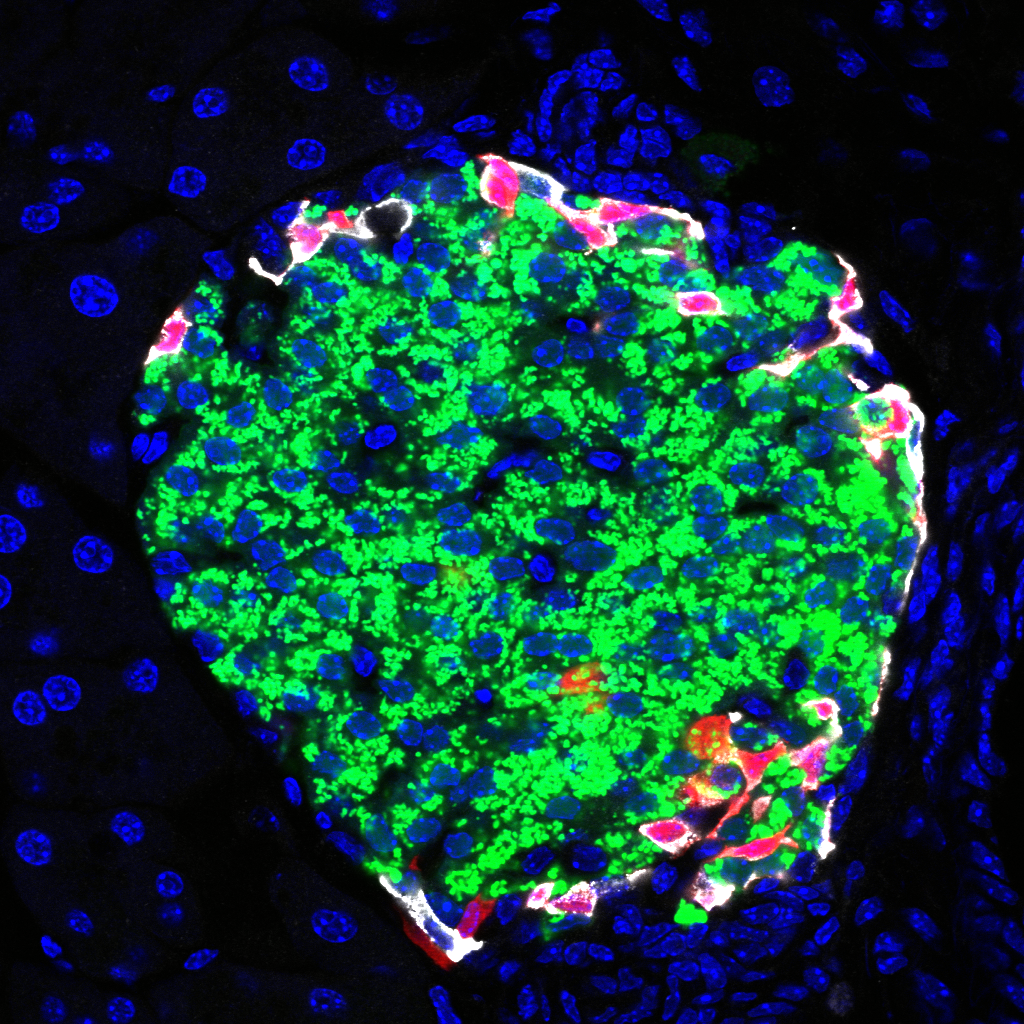

Supplement: Supplementary file 4 — Source data Fig. 2 [file 44318_2025_434_MOESM4_ESM.zip › Figure 2/2L/2L_12w_Sst.tif]

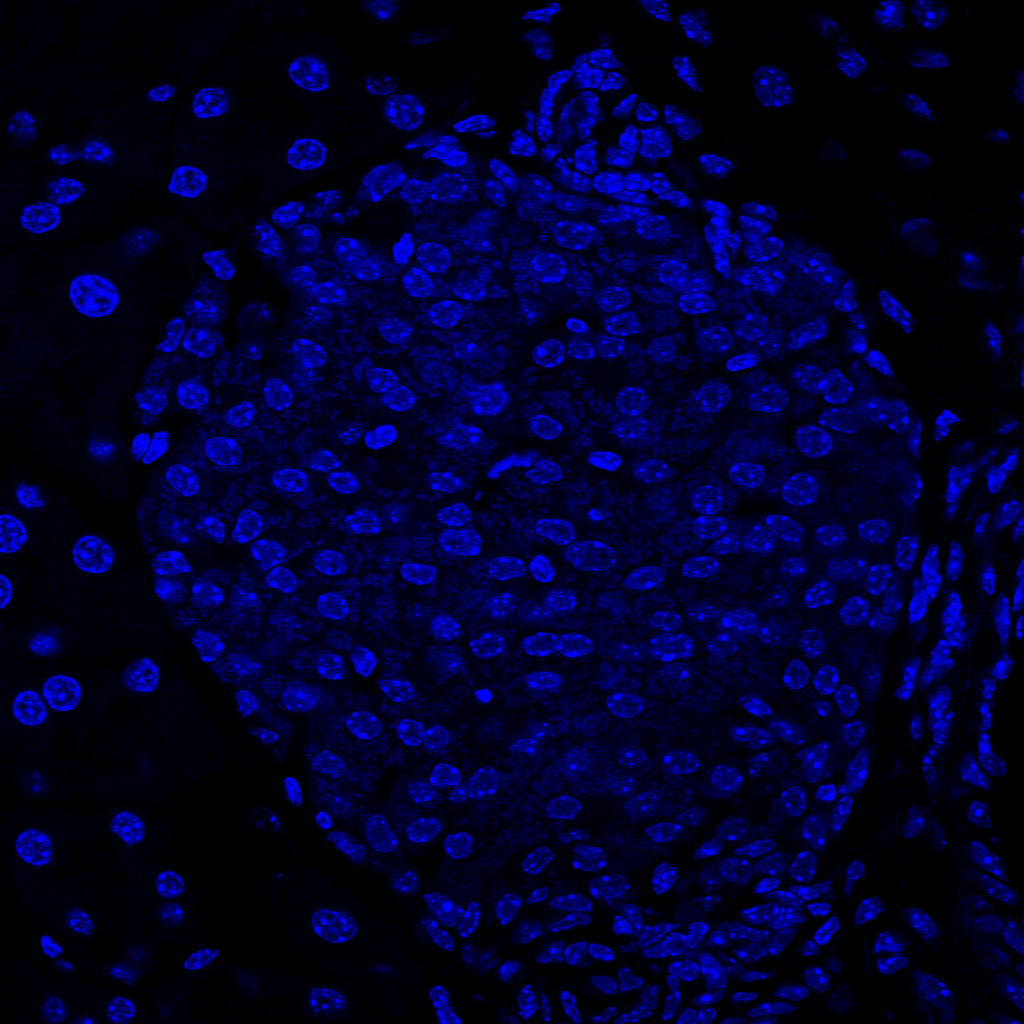

Supplement: Supplementary file 4 — Source data Fig. 2 [file 44318_2025_434_MOESM4_ESM.zip › Figure 2/2L/2L_12w_Sst (blue).tif]

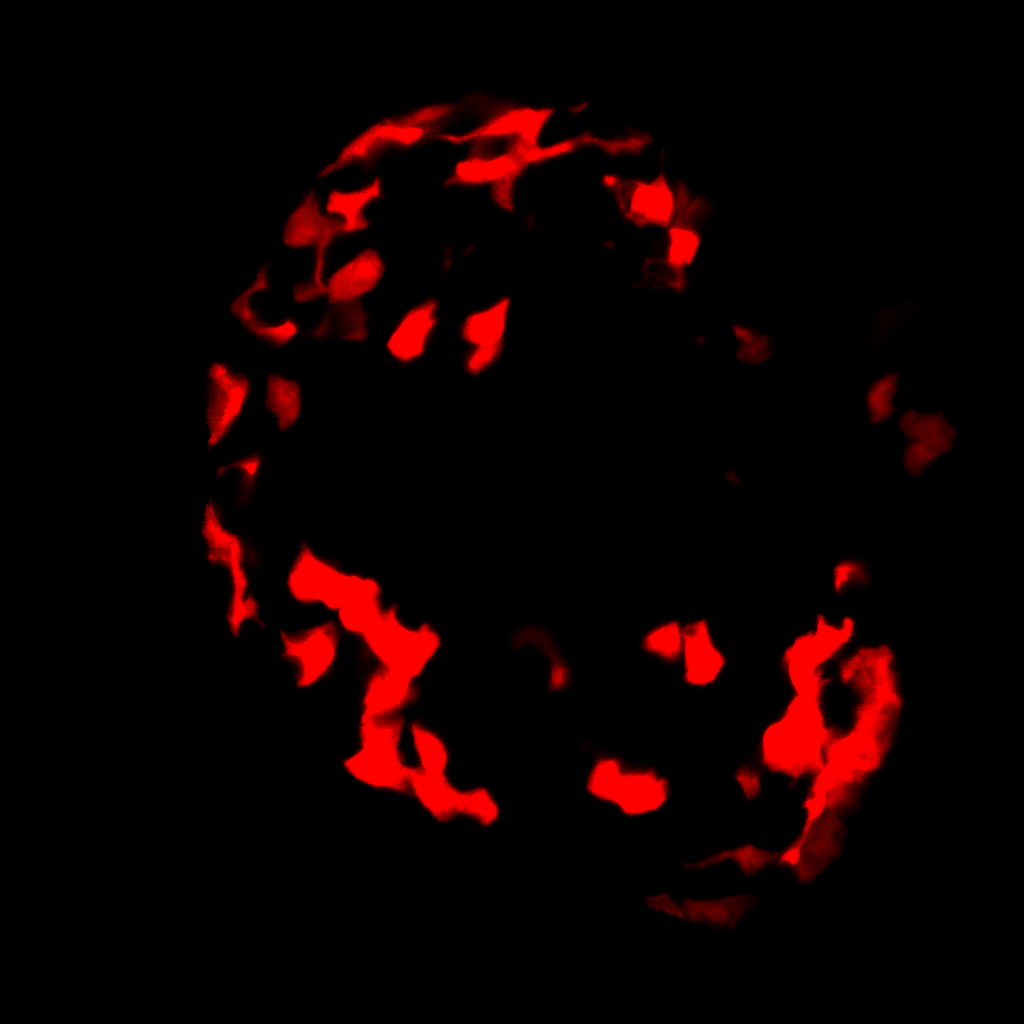

Supplement: Supplementary file 4 — Source data Fig. 2 [file 44318_2025_434_MOESM4_ESM.zip › Figure 2/2L/2L_2w_Sst (red).tif]

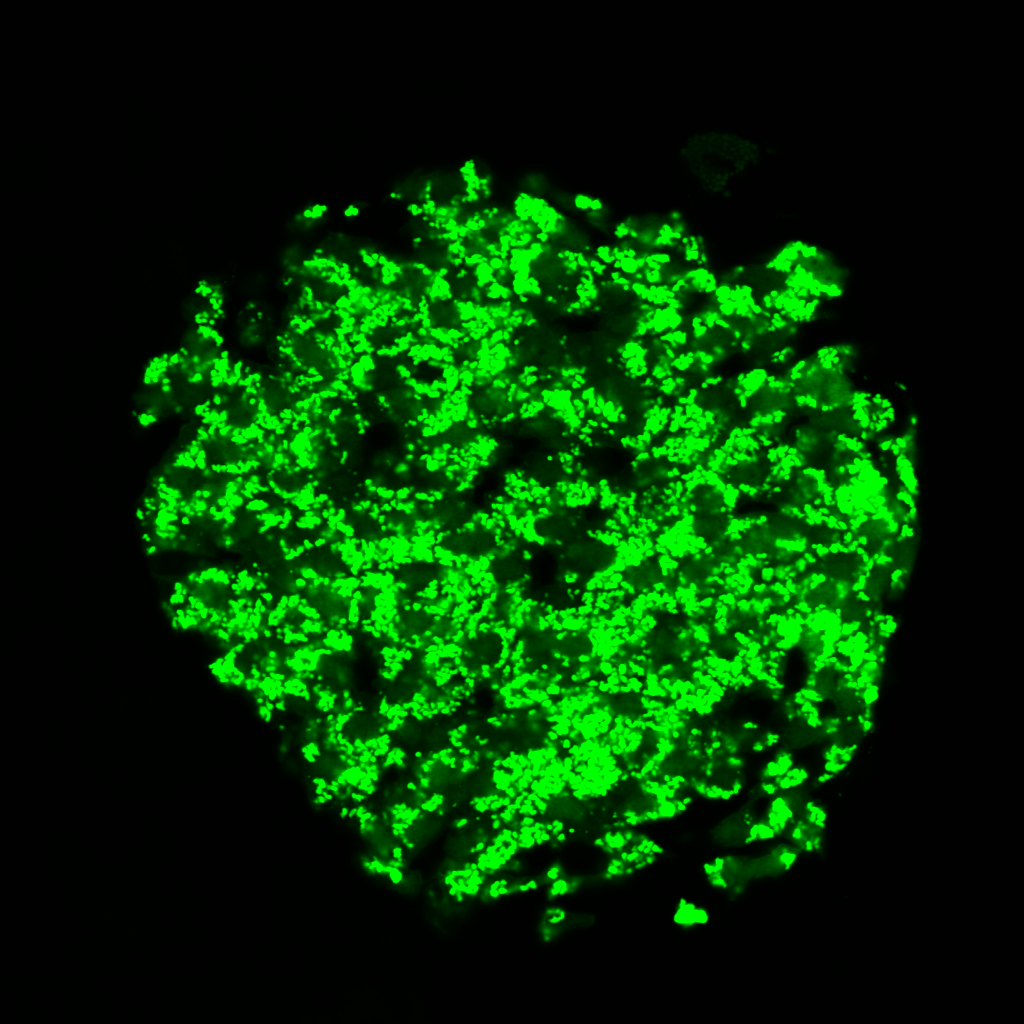

Supplement: Supplementary file 4 — Source data Fig. 2 [file 44318_2025_434_MOESM4_ESM.zip › Figure 2/2L/2L_12w_Sst (green).tif]

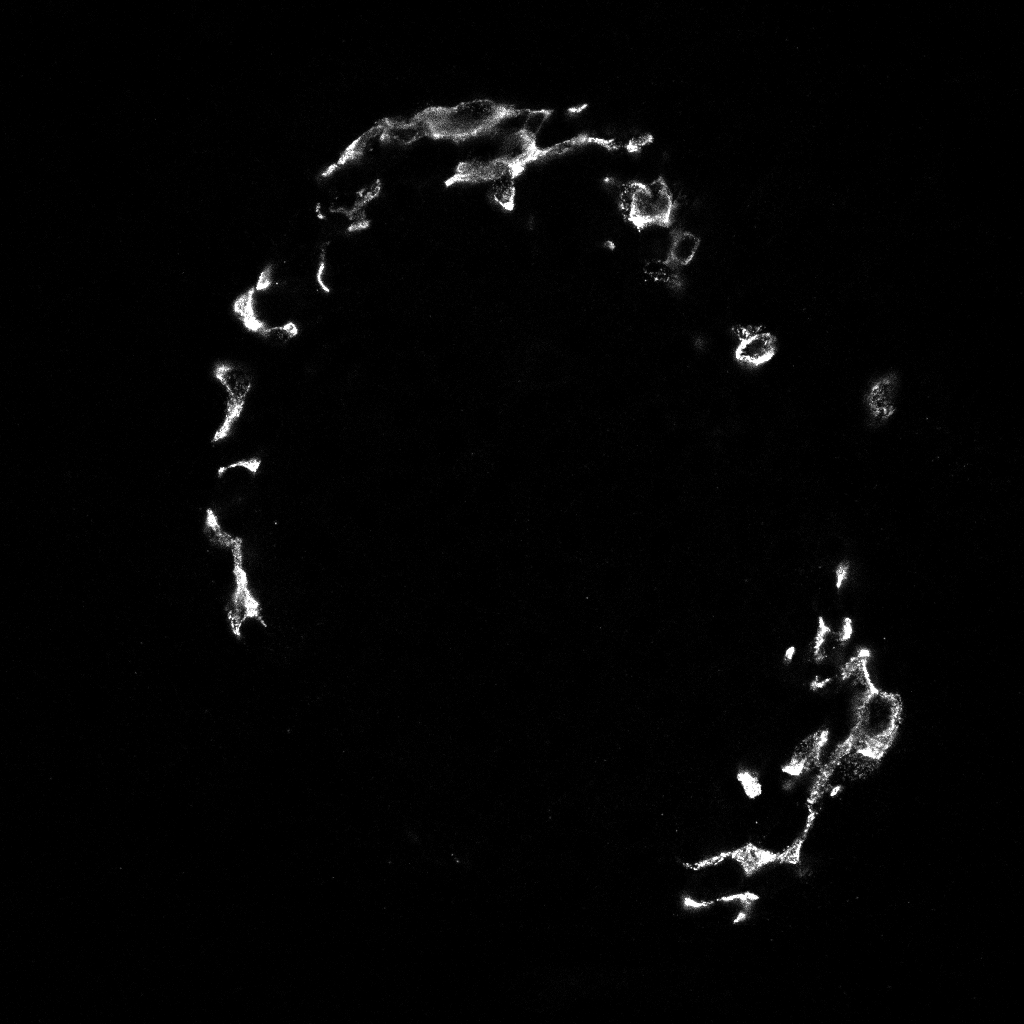

Supplement: Supplementary file 4 — Source data Fig. 2 [file 44318_2025_434_MOESM4_ESM.zip › Figure 2/2L/2L_2w_Sst (gray).tif]

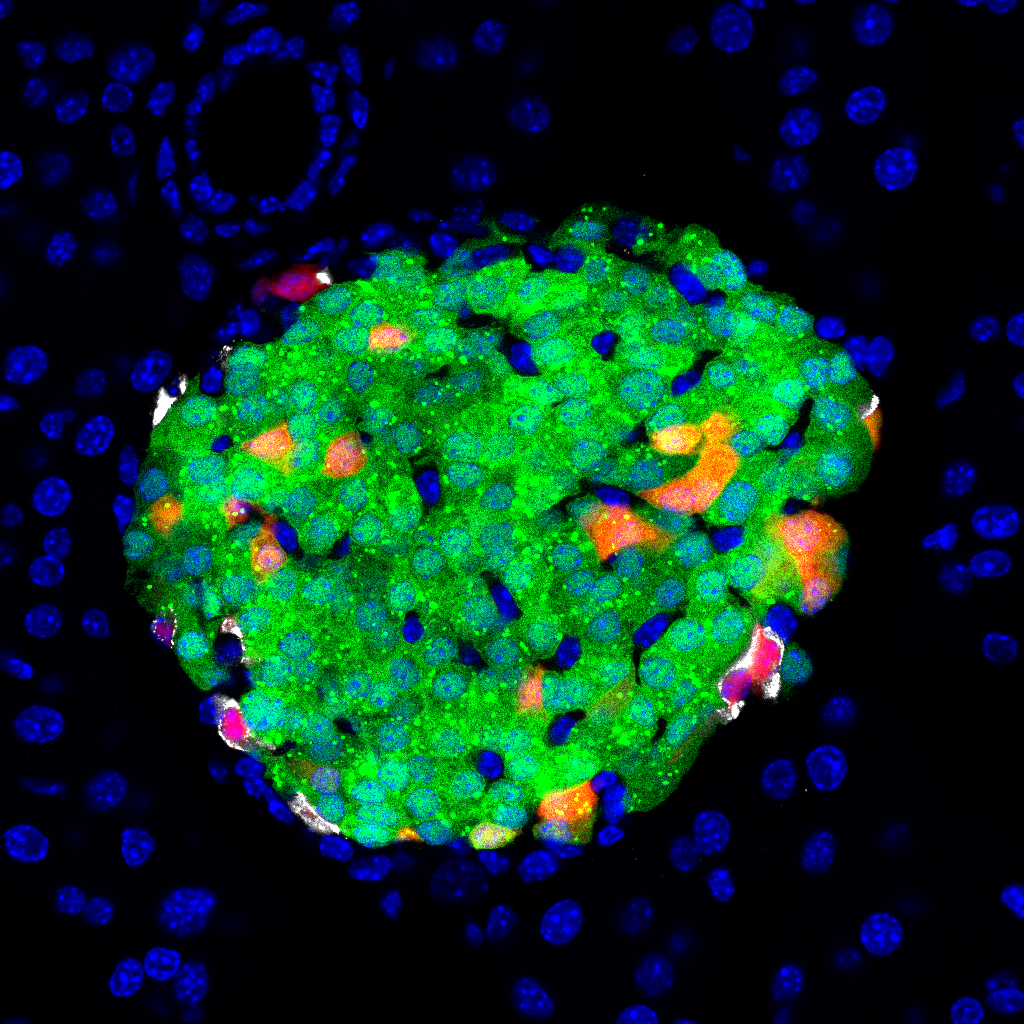

Supplement: Supplementary file 4 — Source data Fig. 2 [file 44318_2025_434_MOESM4_ESM.zip › Figure 2/2E/2E_Merge.tif]

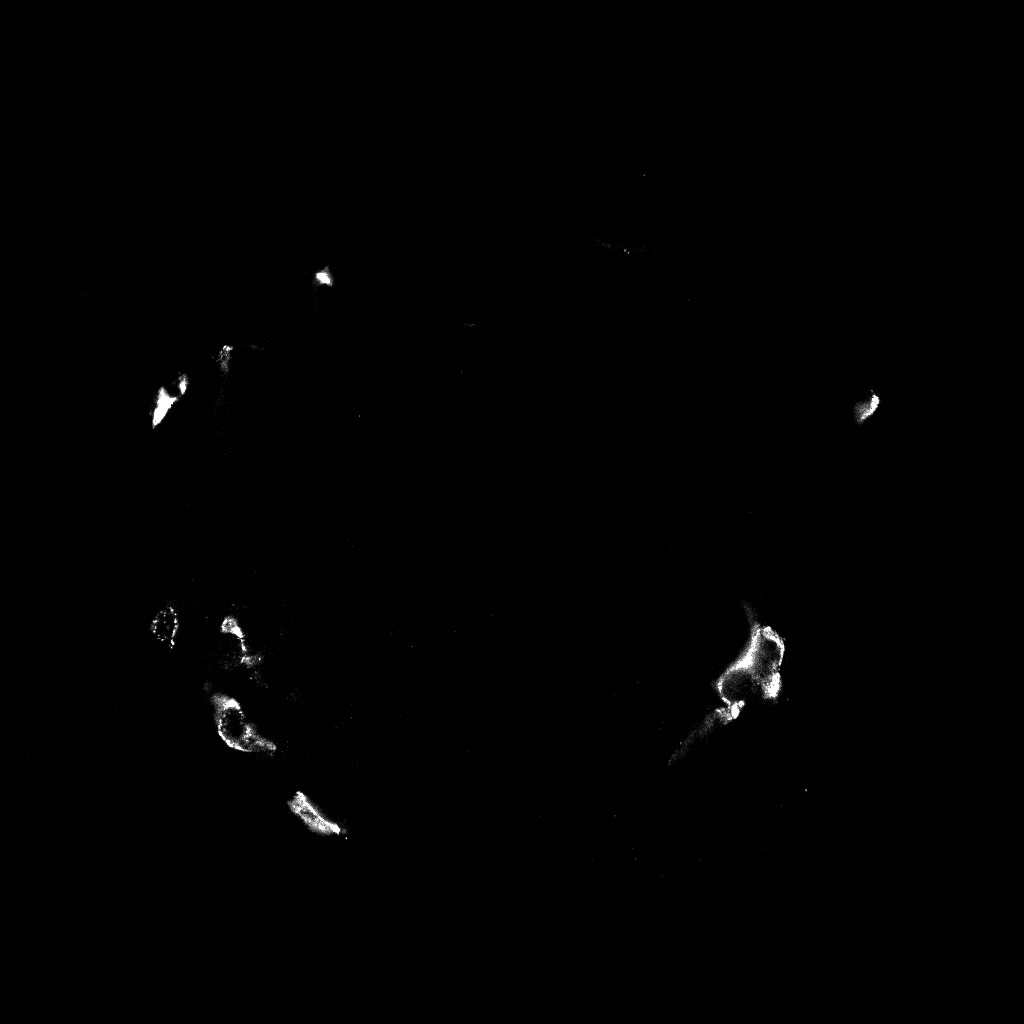

Supplement: Supplementary file 4 — Source data Fig. 2 [file 44318_2025_434_MOESM4_ESM.zip › Figure 2/2E/2E_Merge (gray).tif]

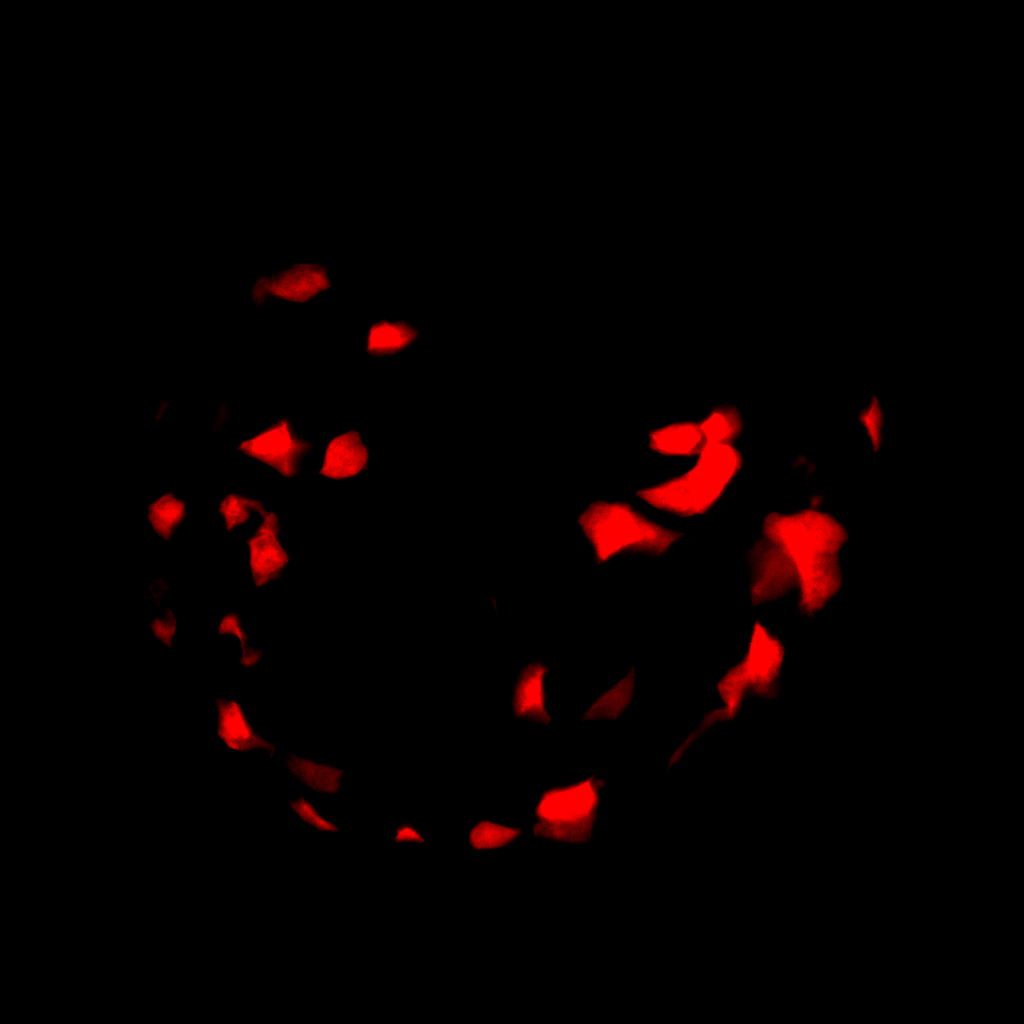

Supplement: Supplementary file 4 — Source data Fig. 2 [file 44318_2025_434_MOESM4_ESM.zip › Figure 2/2E/2E_Merge (red).tif]

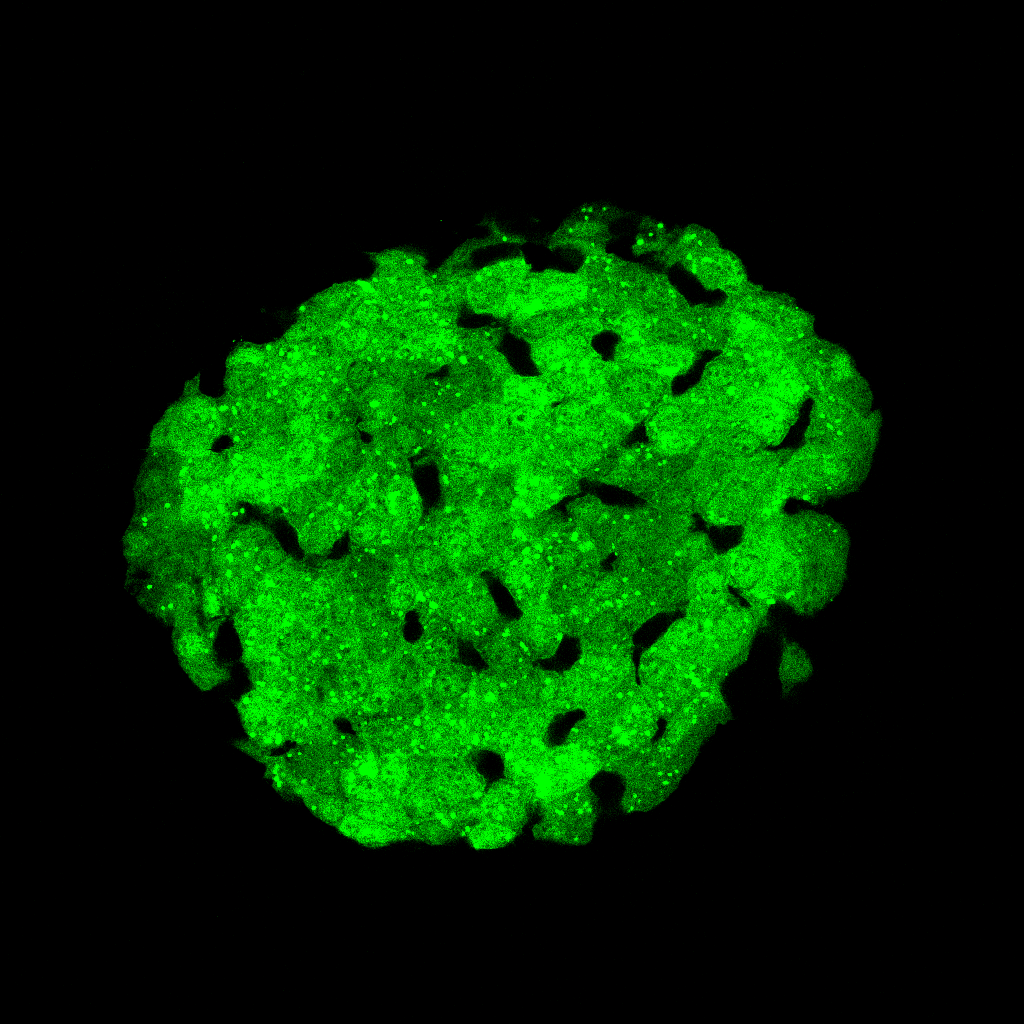

Supplement: Supplementary file 4 — Source data Fig. 2 [file 44318_2025_434_MOESM4_ESM.zip › Figure 2/2E/2E_Merge (green).tif]

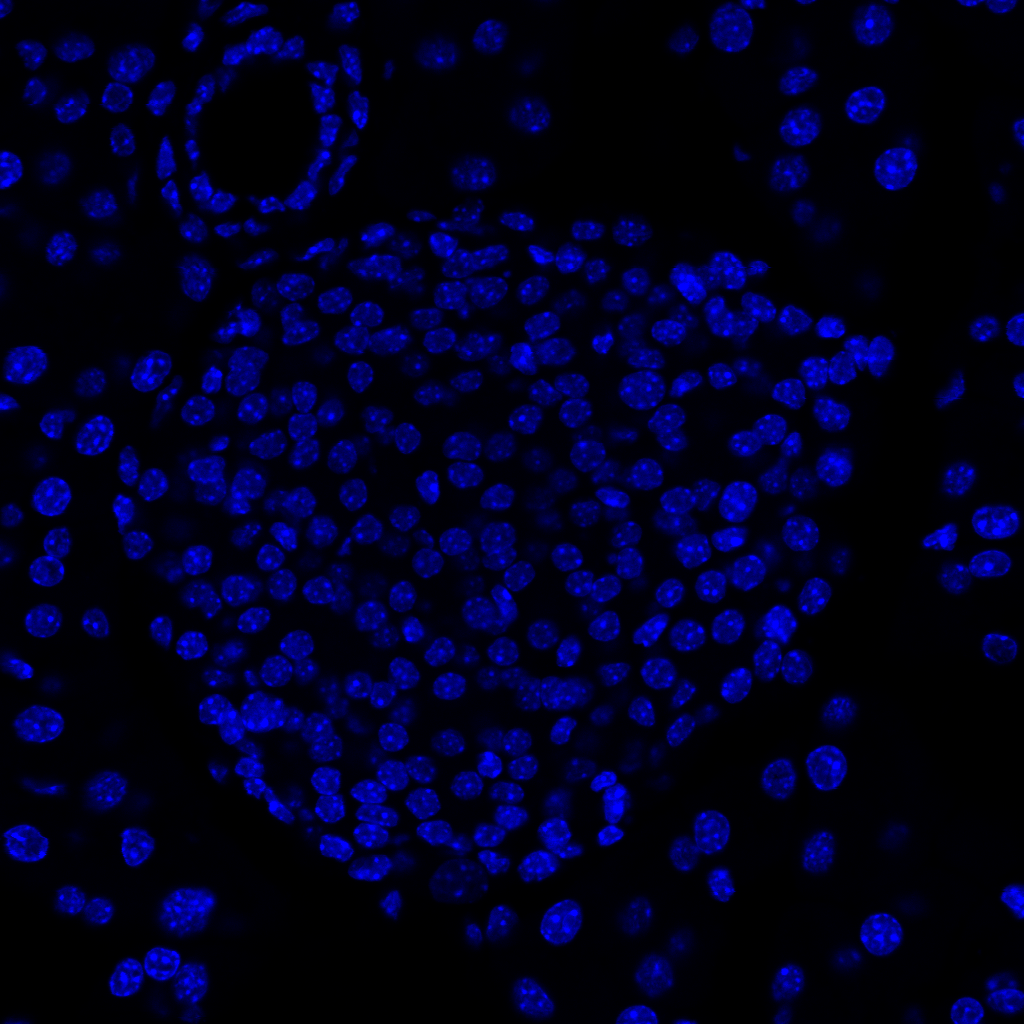

Supplement: Supplementary file 4 — Source data Fig. 2 [file 44318_2025_434_MOESM4_ESM.zip › Figure 2/2E/2E_Merge (blue).tif]

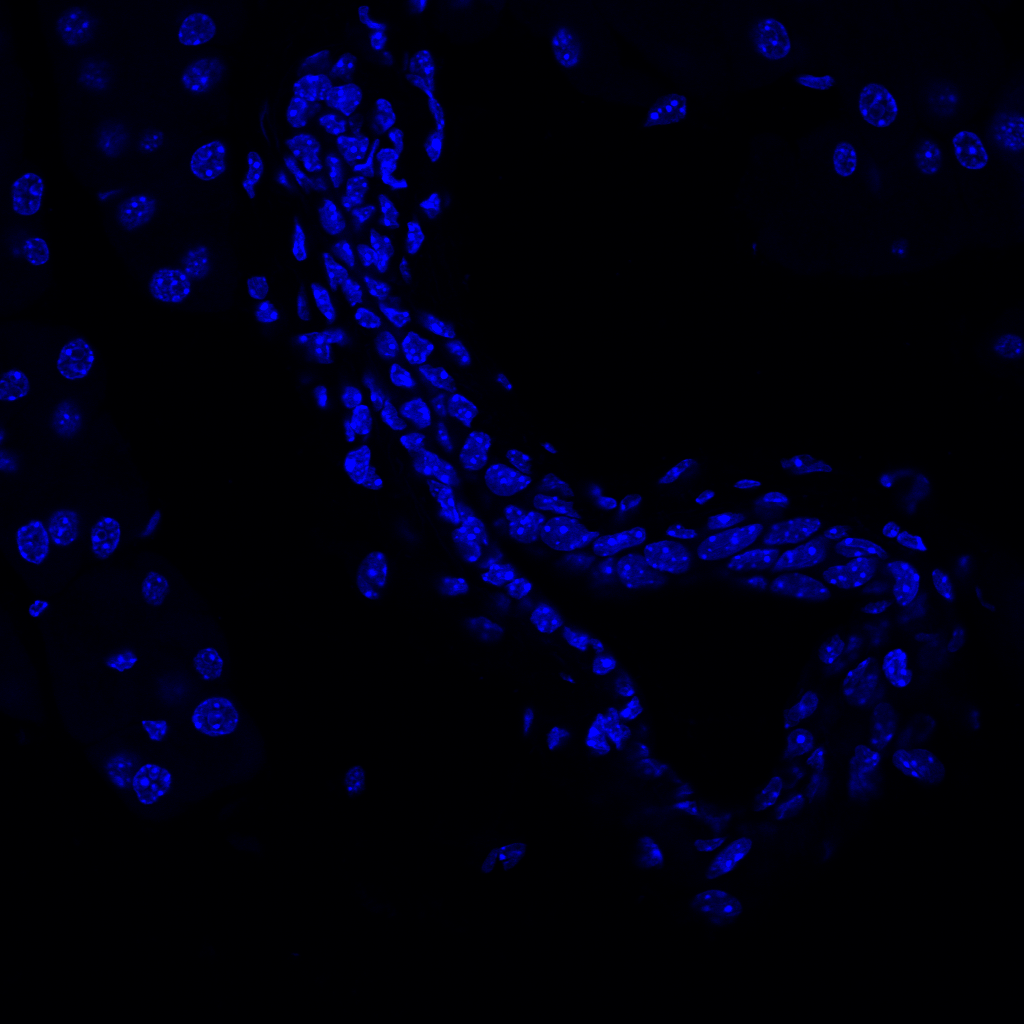

Supplement: Supplementary file 5 — Source data Fig. 3 [file 44318_2025_434_MOESM5_ESM.zip › Figure 3/3K/3K_12w_CK19 (blue).tif]

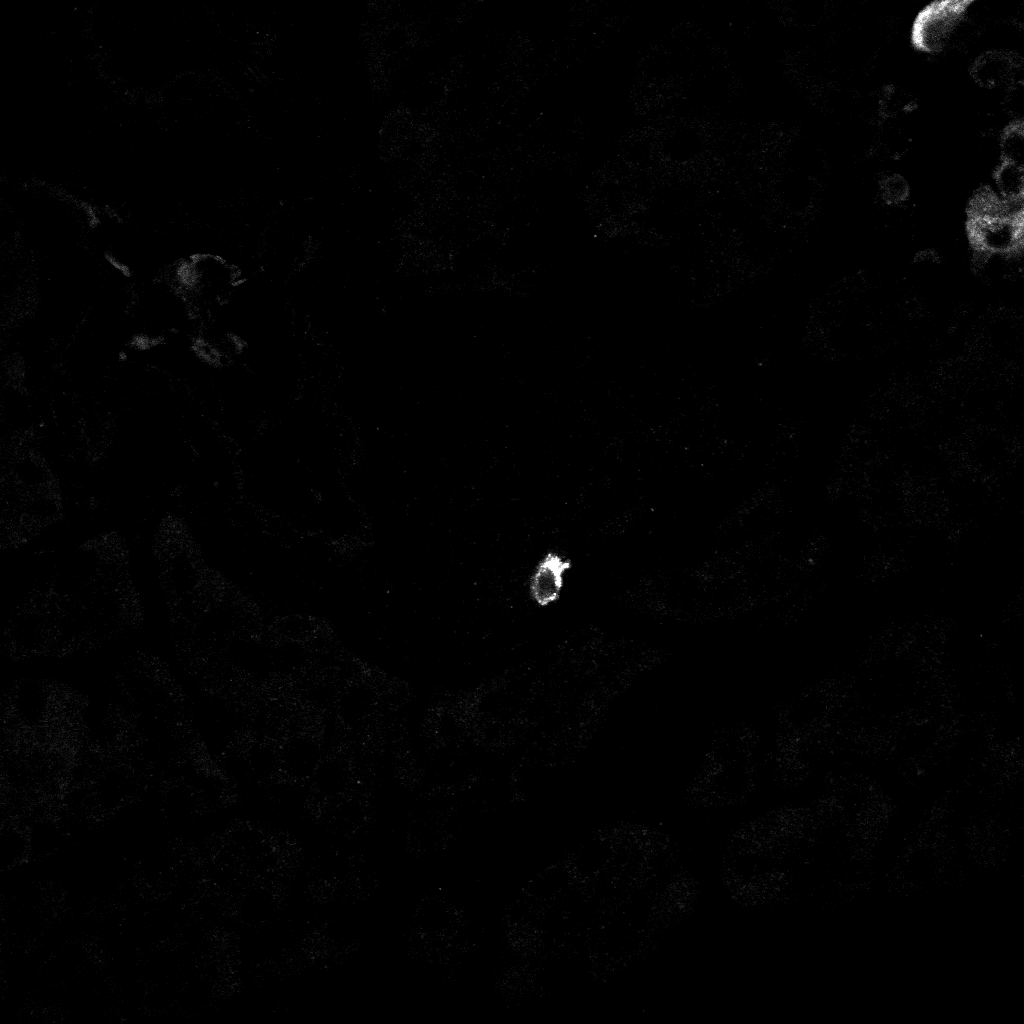

Supplement: Supplementary file 5 — Source data Fig. 3 [file 44318_2025_434_MOESM5_ESM.zip › Figure 3/3K/3K_2w_Ins (gray).tif]

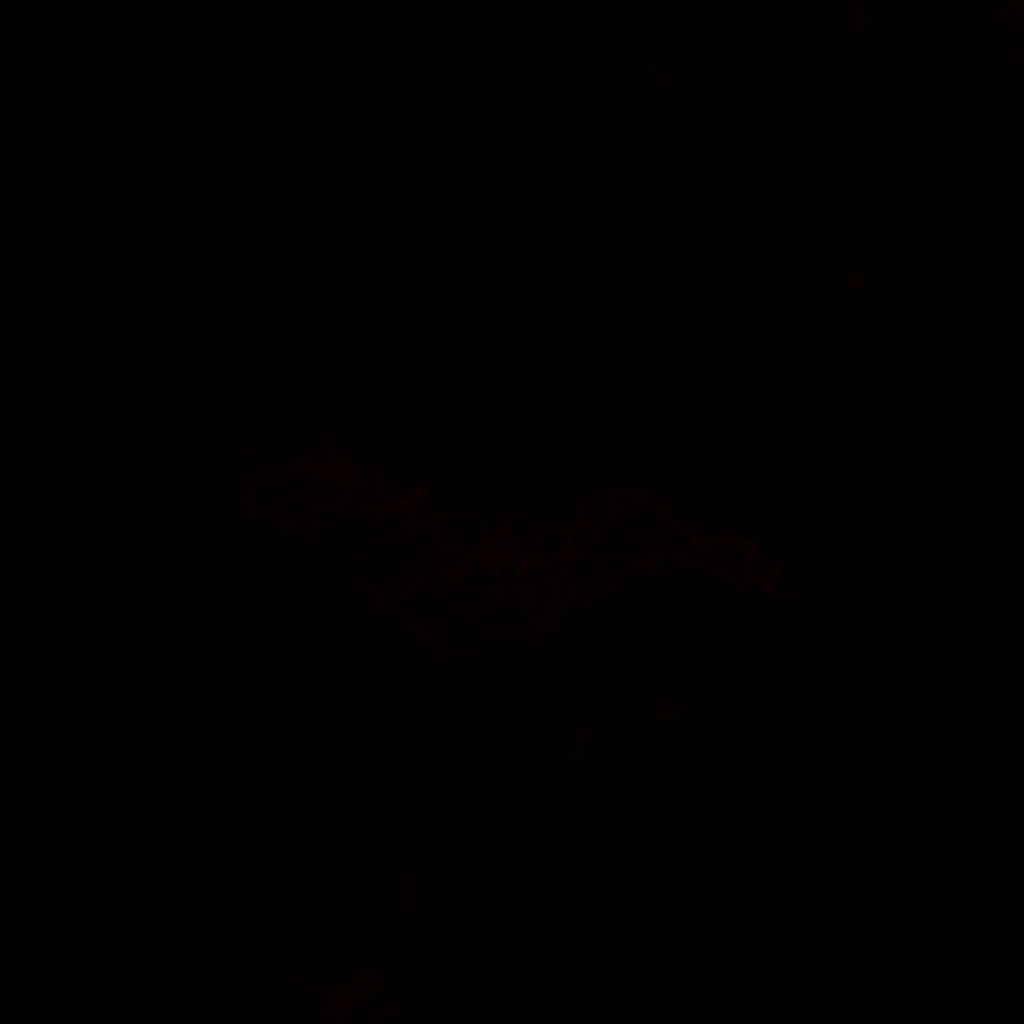

Supplement: Supplementary file 5 — Source data Fig. 3 [file 44318_2025_434_MOESM5_ESM.zip › Figure 3/3K/3K_2w_CK19 (red).tif]

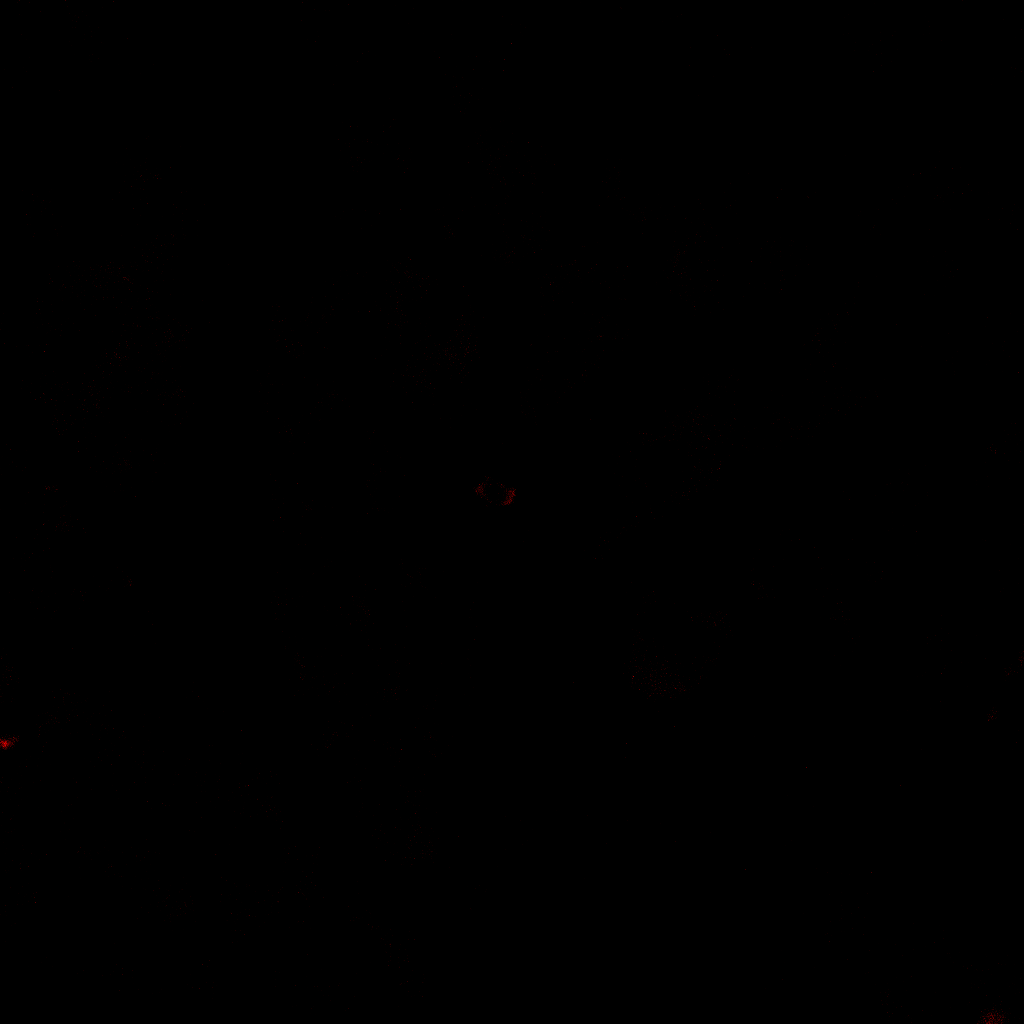

Supplement: Supplementary file 5 — Source data Fig. 3 [file 44318_2025_434_MOESM5_ESM.zip › Figure 3/3K/3K_2w_Sst (red).tif]

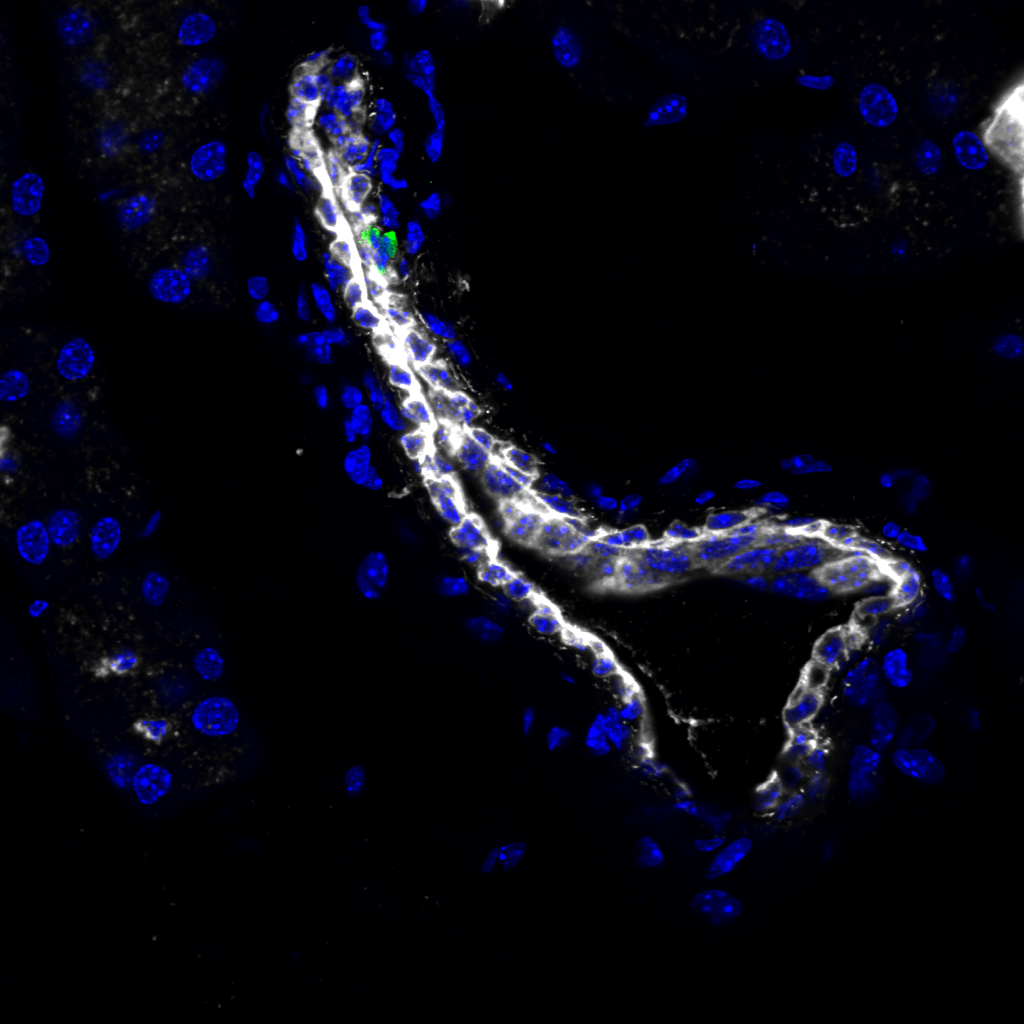

Supplement: Supplementary file 5 — Source data Fig. 3 [file 44318_2025_434_MOESM5_ESM.zip › Figure 3/3K/3K_12w_CK19.tif]

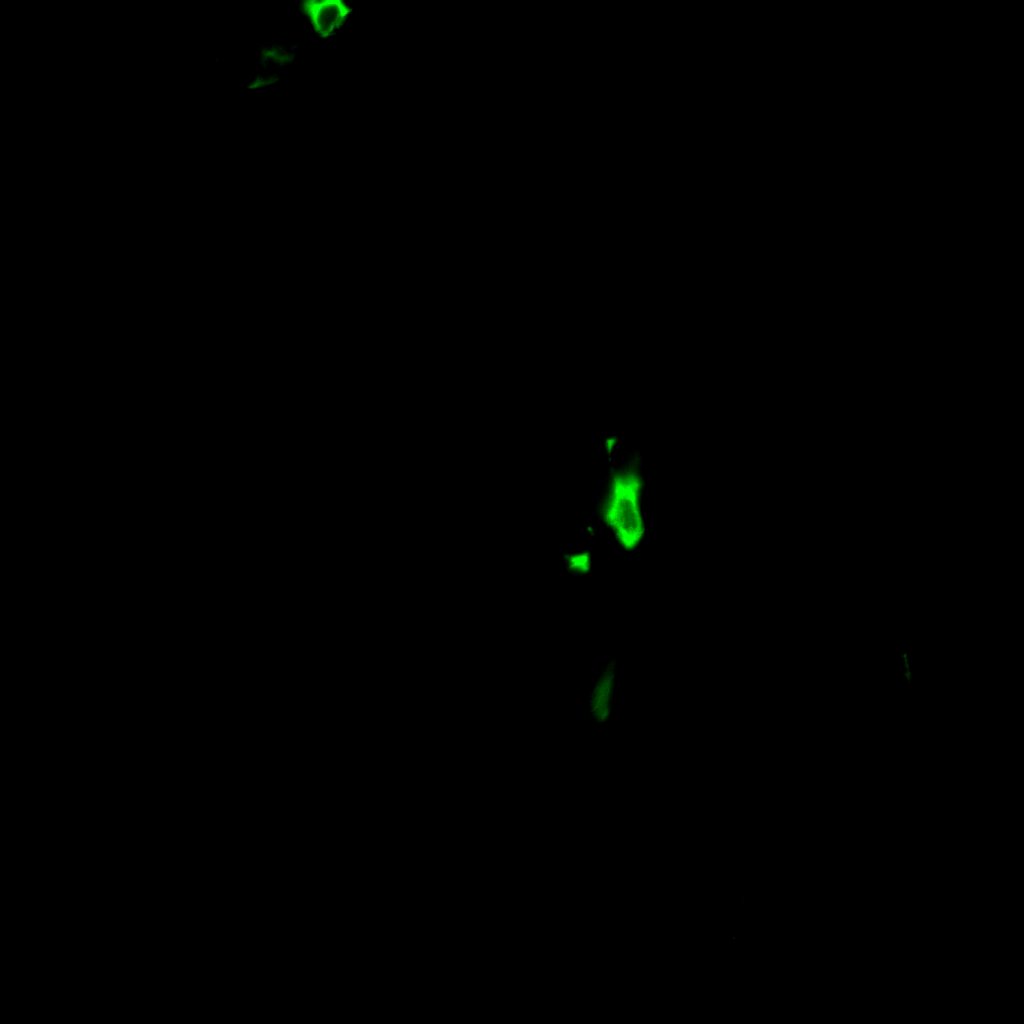

Supplement: Supplementary file 5 — Source data Fig. 3 [file 44318_2025_434_MOESM5_ESM.zip › Figure 3/3K/3K_12w_Ins (green).tiff]

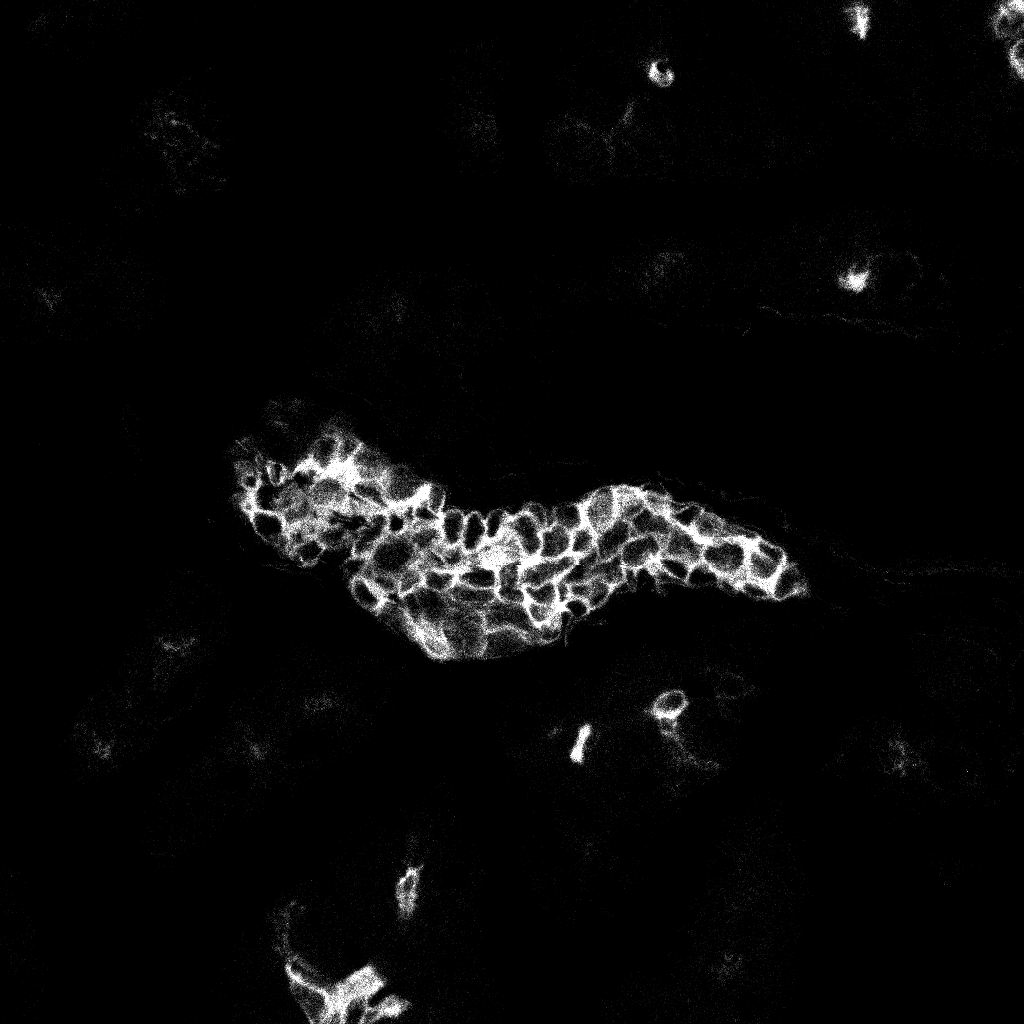

Supplement: Supplementary file 5 — Source data Fig. 3 [file 44318_2025_434_MOESM5_ESM.zip › Figure 3/3K/3K_2w_CK19 (gray).tif]

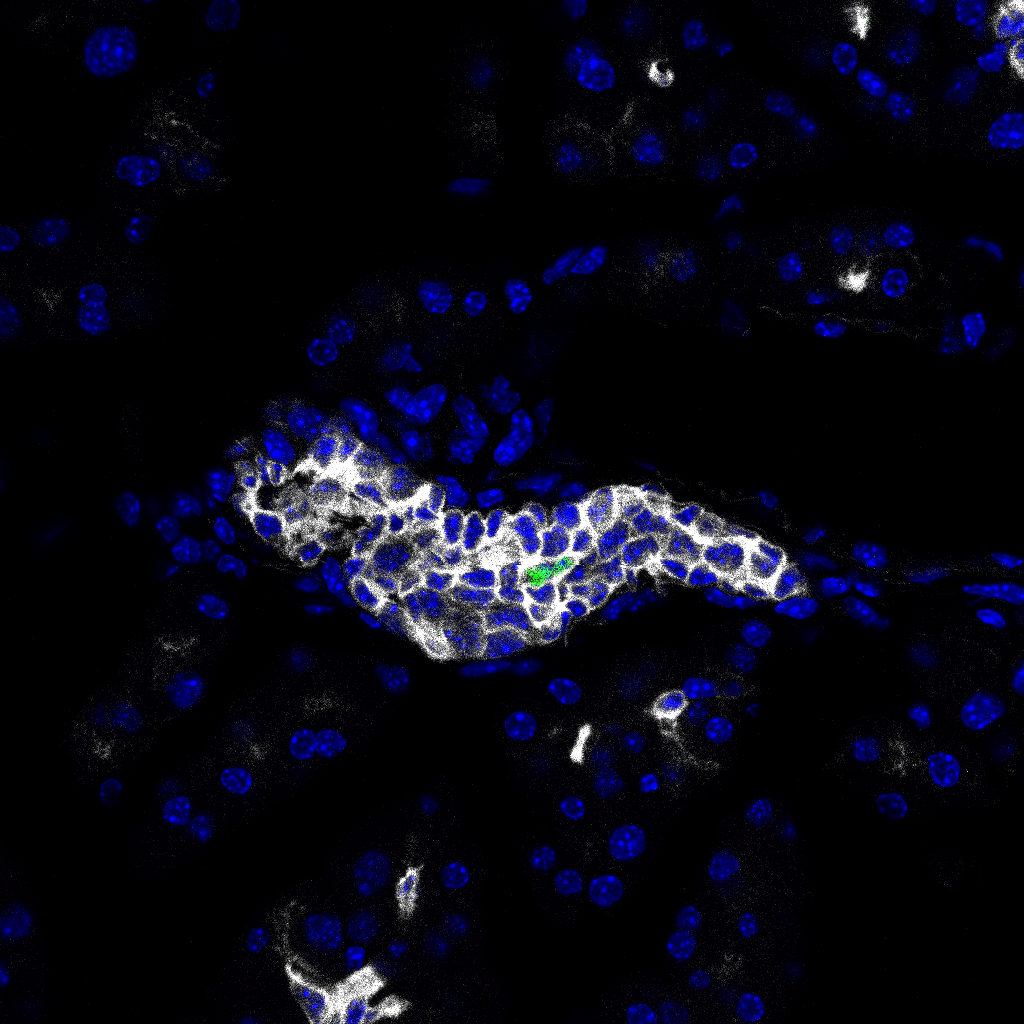

Supplement: Supplementary file 5 — Source data Fig. 3 [file 44318_2025_434_MOESM5_ESM.zip › Figure 3/3K/3K_2w_CK19.tif]

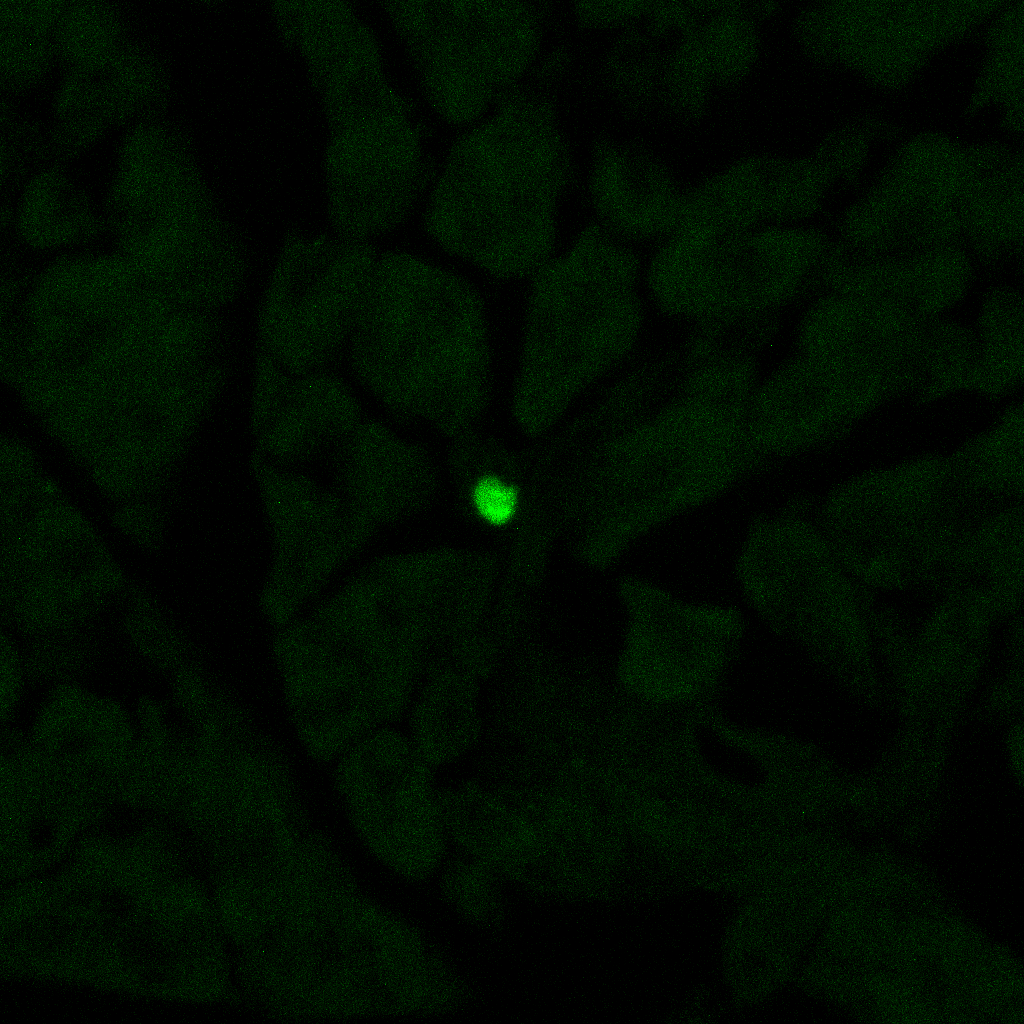

Supplement: Supplementary file 5 — Source data Fig. 3 [file 44318_2025_434_MOESM5_ESM.zip › Figure 3/3K/3K_2w_Sst (green).tif]

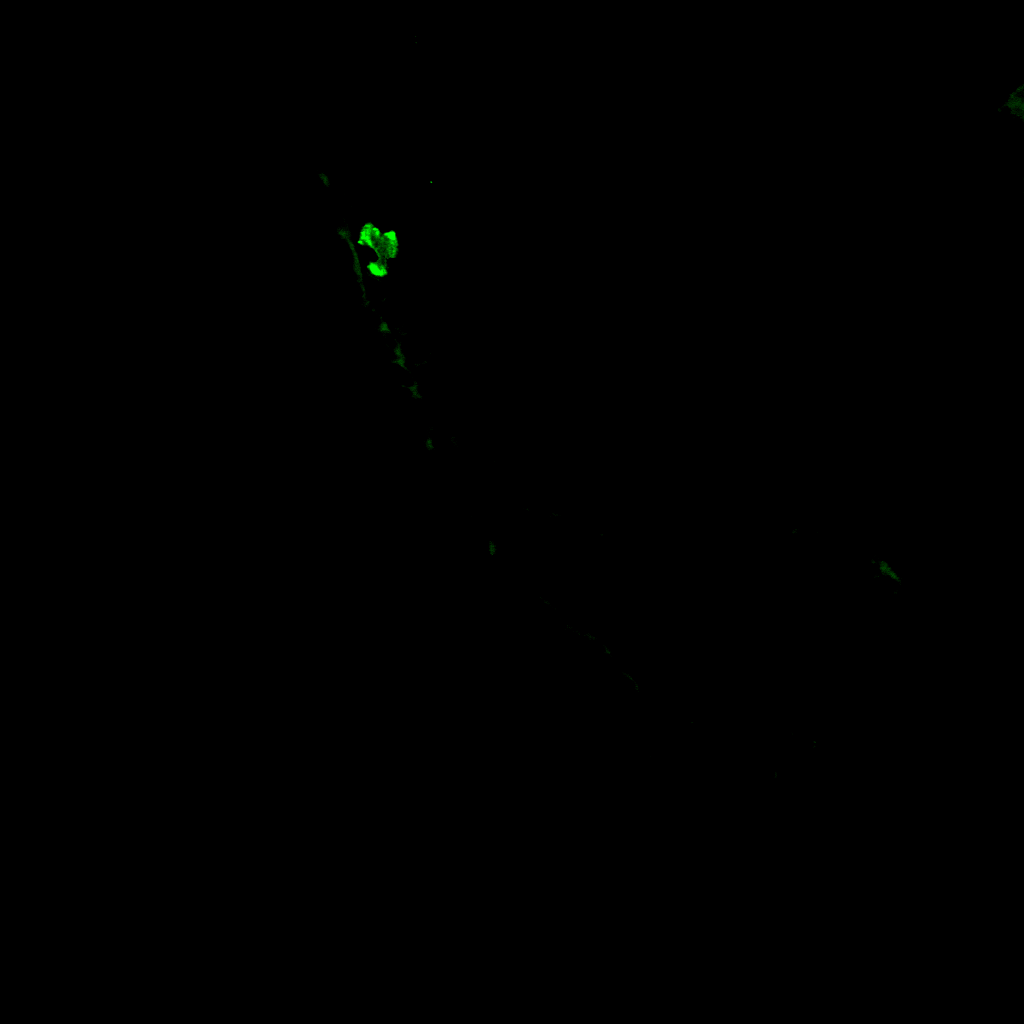

Supplement: Supplementary file 5 — Source data Fig. 3 [file 44318_2025_434_MOESM5_ESM.zip › Figure 3/3K/3K_12w_CK19 (green).tif]

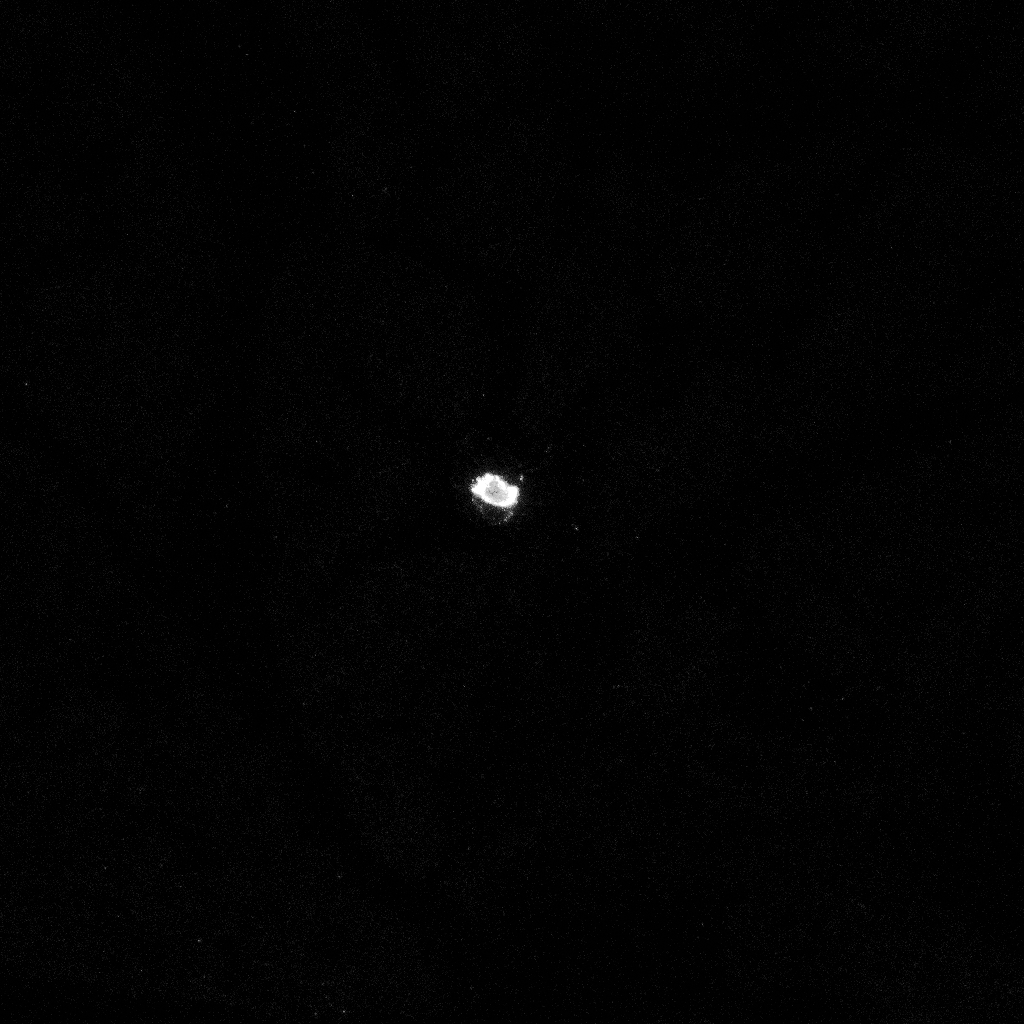

Supplement: Supplementary file 5 — Source data Fig. 3 [file 44318_2025_434_MOESM5_ESM.zip › Figure 3/3K/3K_2w_Sst (gray).tif]

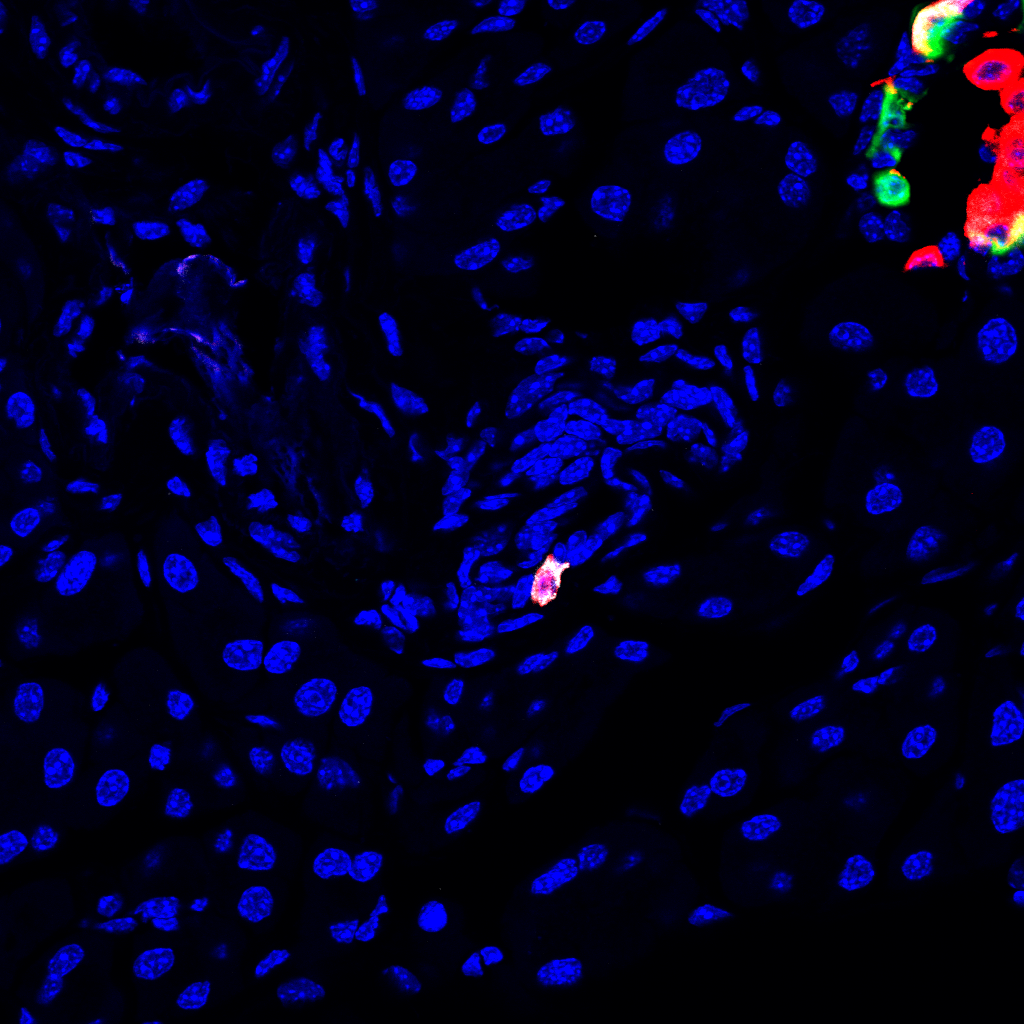

Supplement: Supplementary file 5 — Source data Fig. 3 [file 44318_2025_434_MOESM5_ESM.zip › Figure 3/3K/3K_2w_Ins.tif]

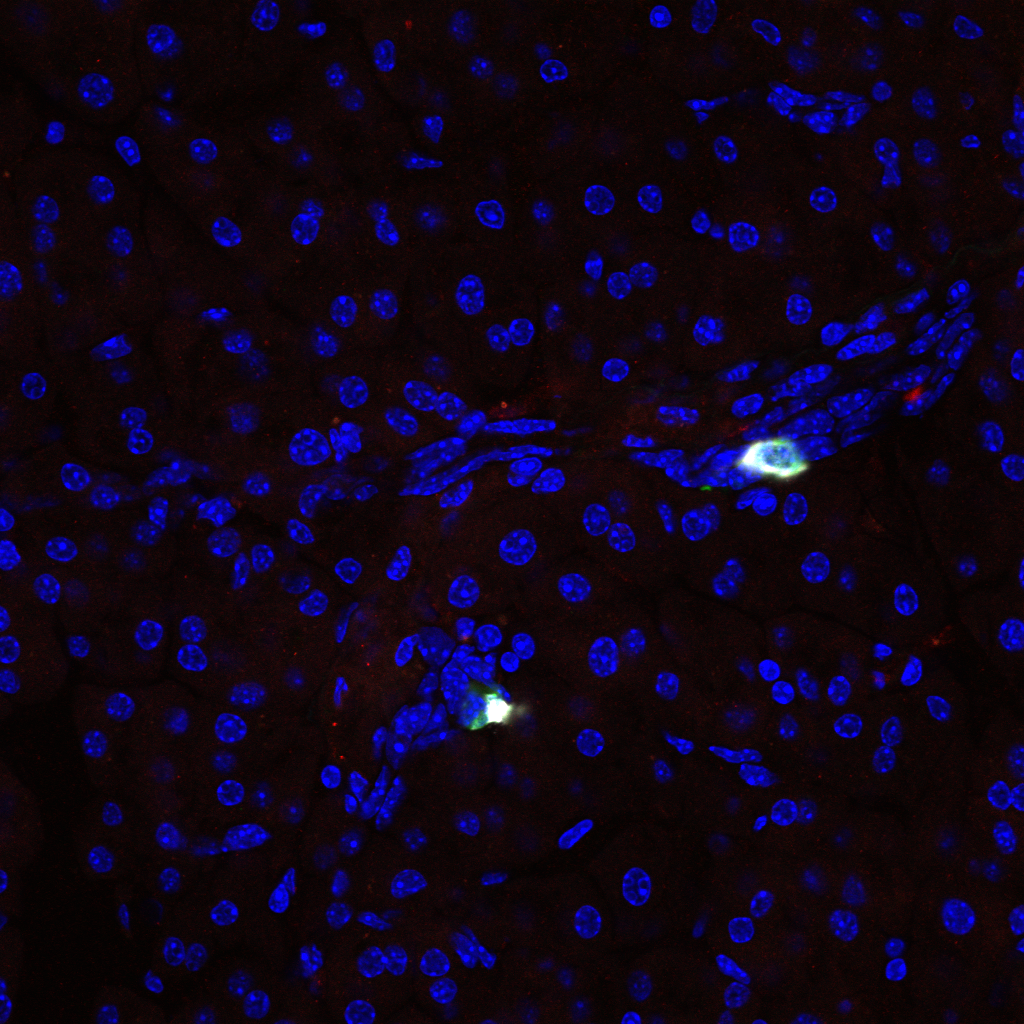

Supplement: Supplementary file 5 — Source data Fig. 3 [file 44318_2025_434_MOESM5_ESM.zip › Figure 3/3K/3K_12w_Sst.tif]

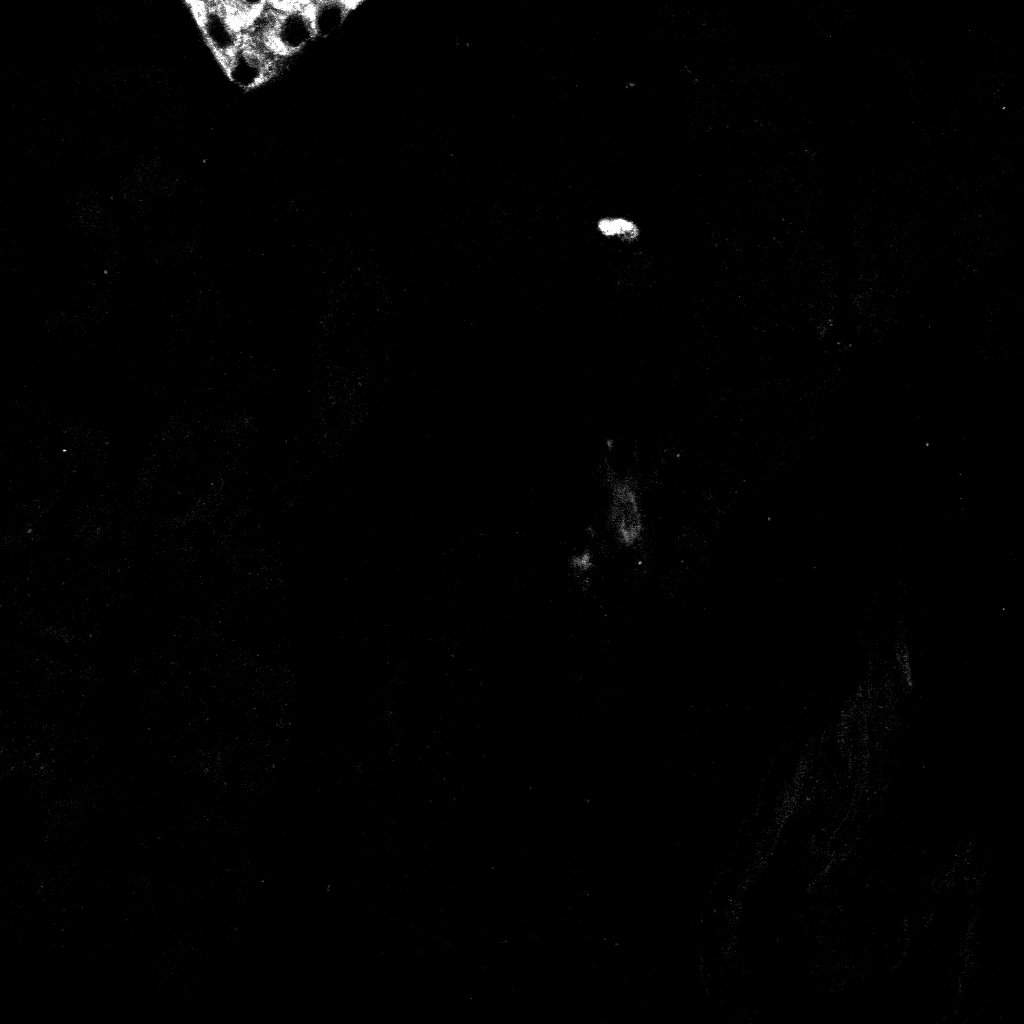

Supplement: Supplementary file 5 — Source data Fig. 3 [file 44318_2025_434_MOESM5_ESM.zip › Figure 3/3K/3K_12w_Ins (gray).tiff]

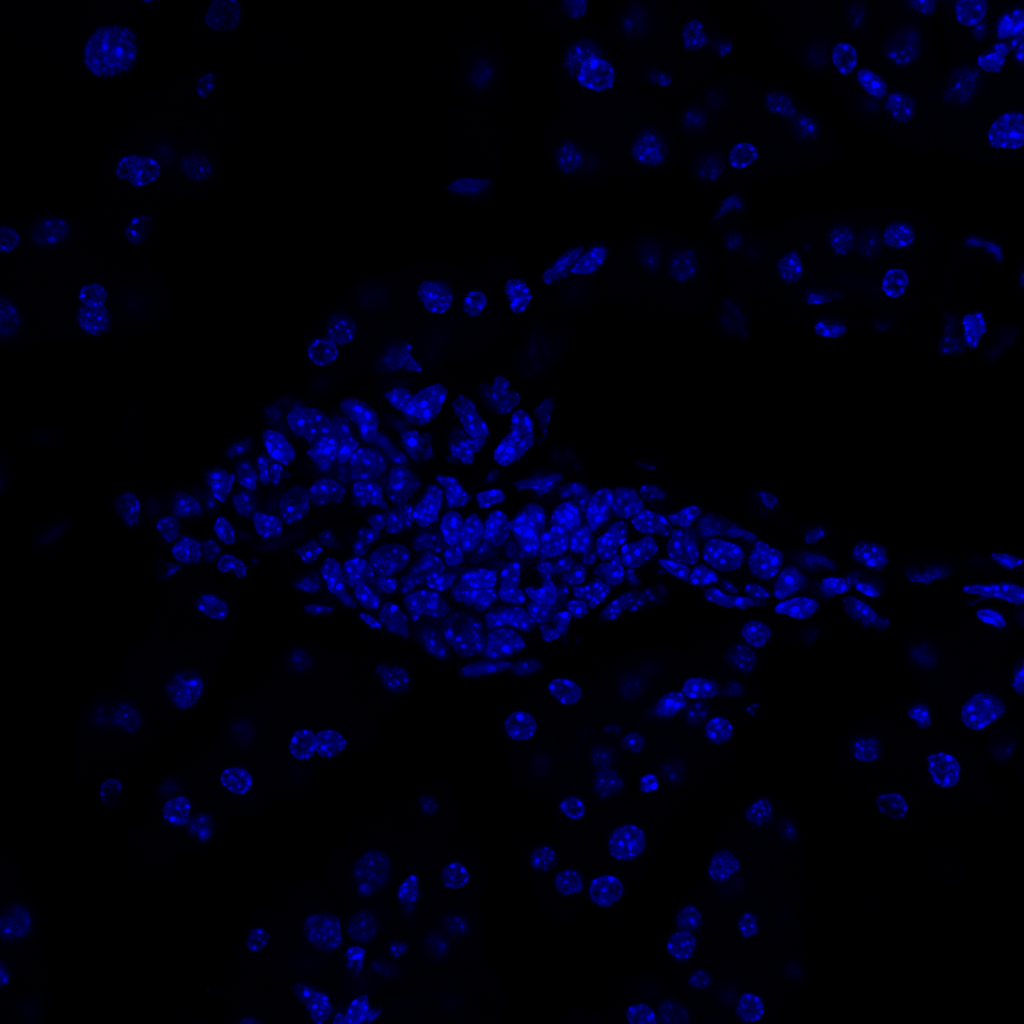

Supplement: Supplementary file 5 — Source data Fig. 3 [file 44318_2025_434_MOESM5_ESM.zip › Figure 3/3K/3K_2w_CK19 (blue).tif]

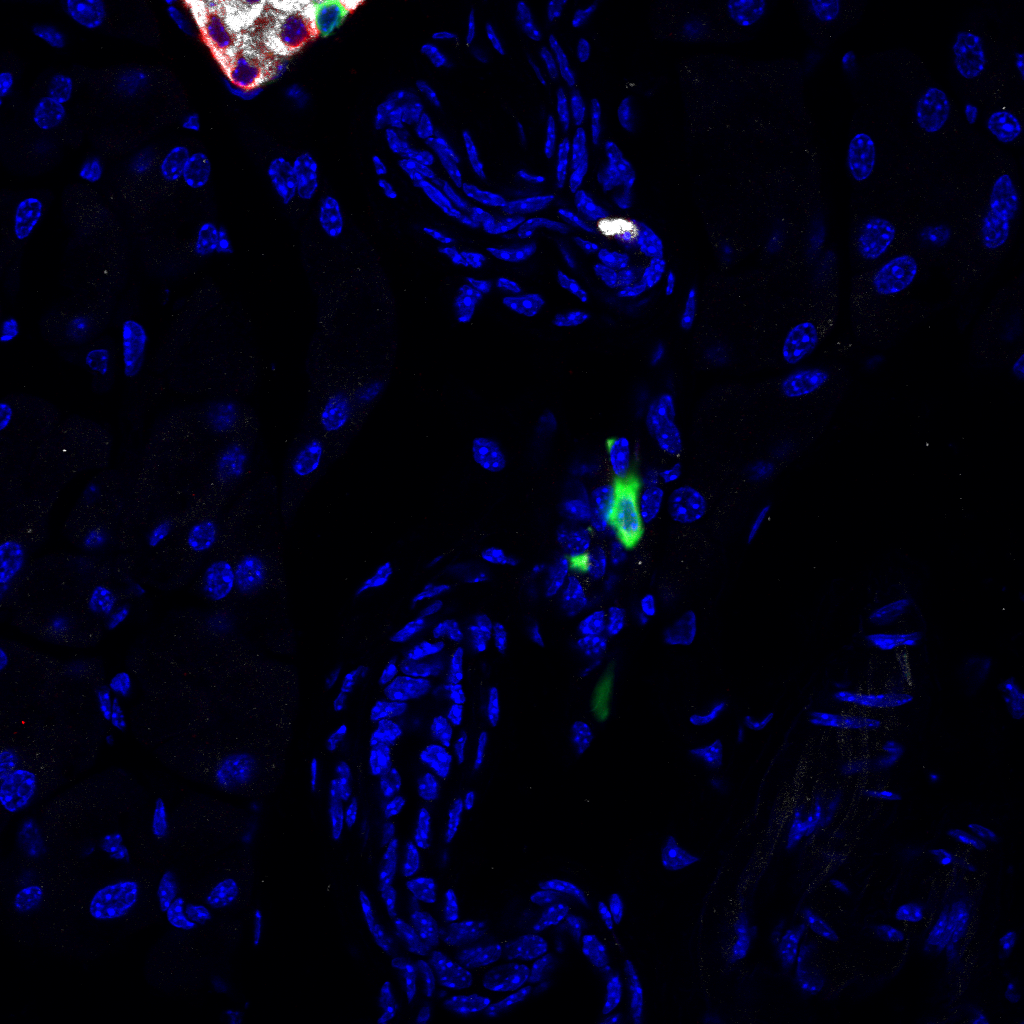

Supplement: Supplementary file 5 — Source data Fig. 3 [file 44318_2025_434_MOESM5_ESM.zip › Figure 3/3K/3K_12w_Ins.tif]

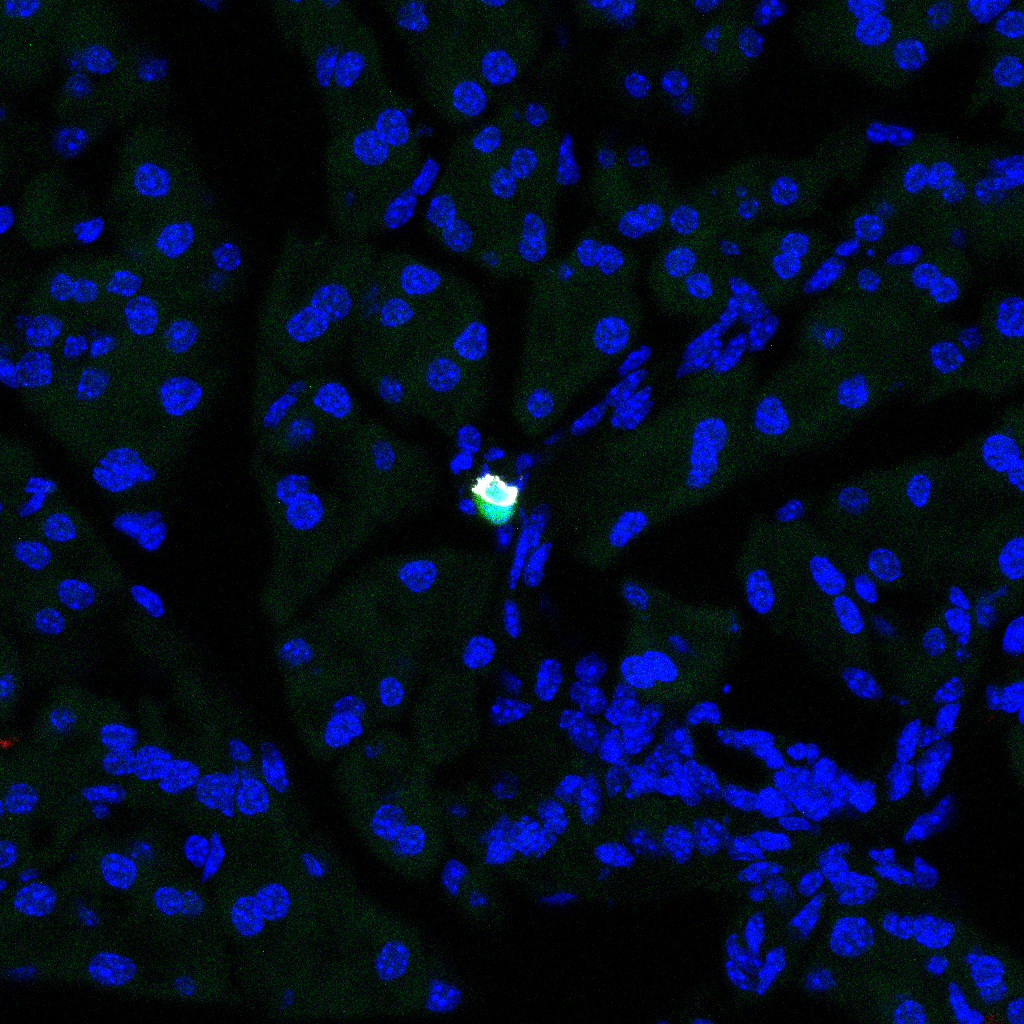

Supplement: Supplementary file 5 — Source data Fig. 3 [file 44318_2025_434_MOESM5_ESM.zip › Figure 3/3K/3K_2w_Sst.tif]

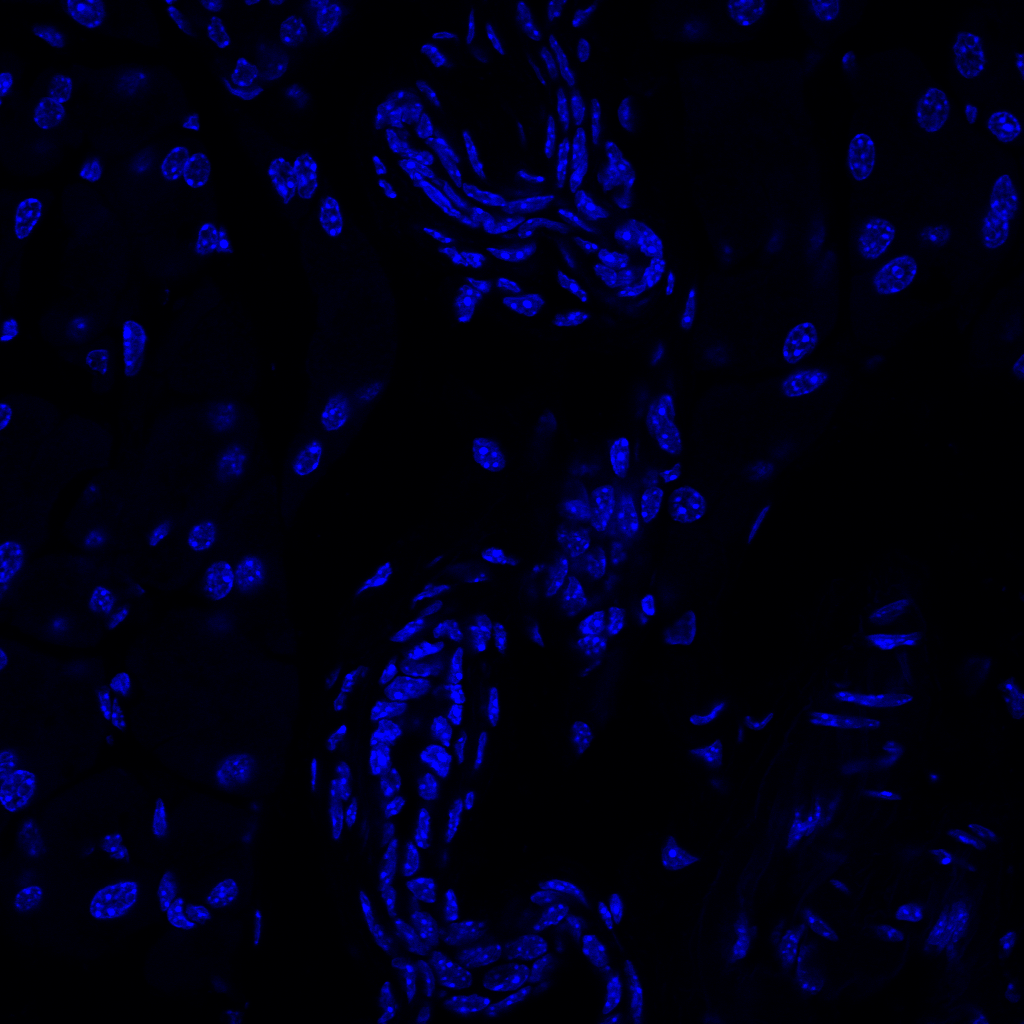

Supplement: Supplementary file 5 — Source data Fig. 3 [file 44318_2025_434_MOESM5_ESM.zip › Figure 3/3K/3K_12w_Ins (blue).tiff]

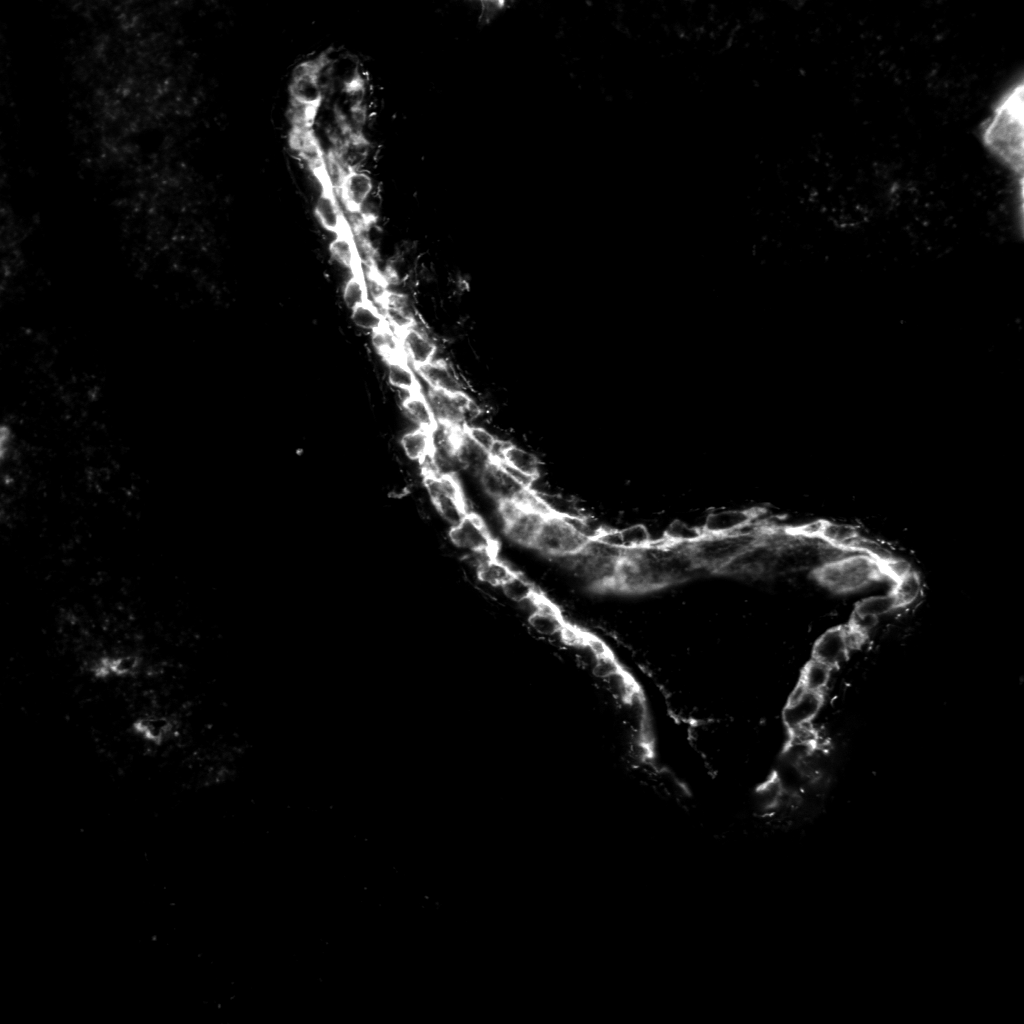

Supplement: Supplementary file 5 — Source data Fig. 3 [file 44318_2025_434_MOESM5_ESM.zip › Figure 3/3K/3K_12w_CK19 (gray).tif]

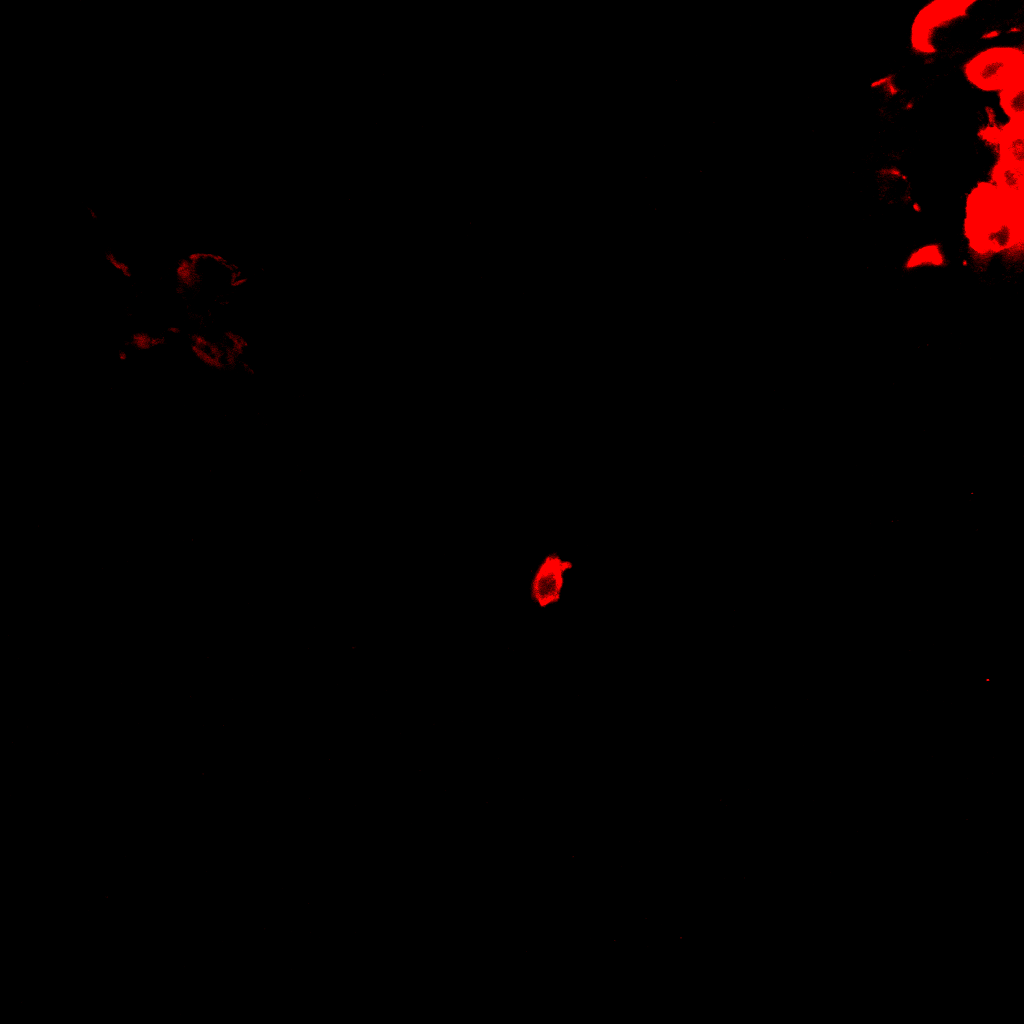

Supplement: Supplementary file 5 — Source data Fig. 3 [file 44318_2025_434_MOESM5_ESM.zip › Figure 3/3K/3K_2w_Ins (red).tif]

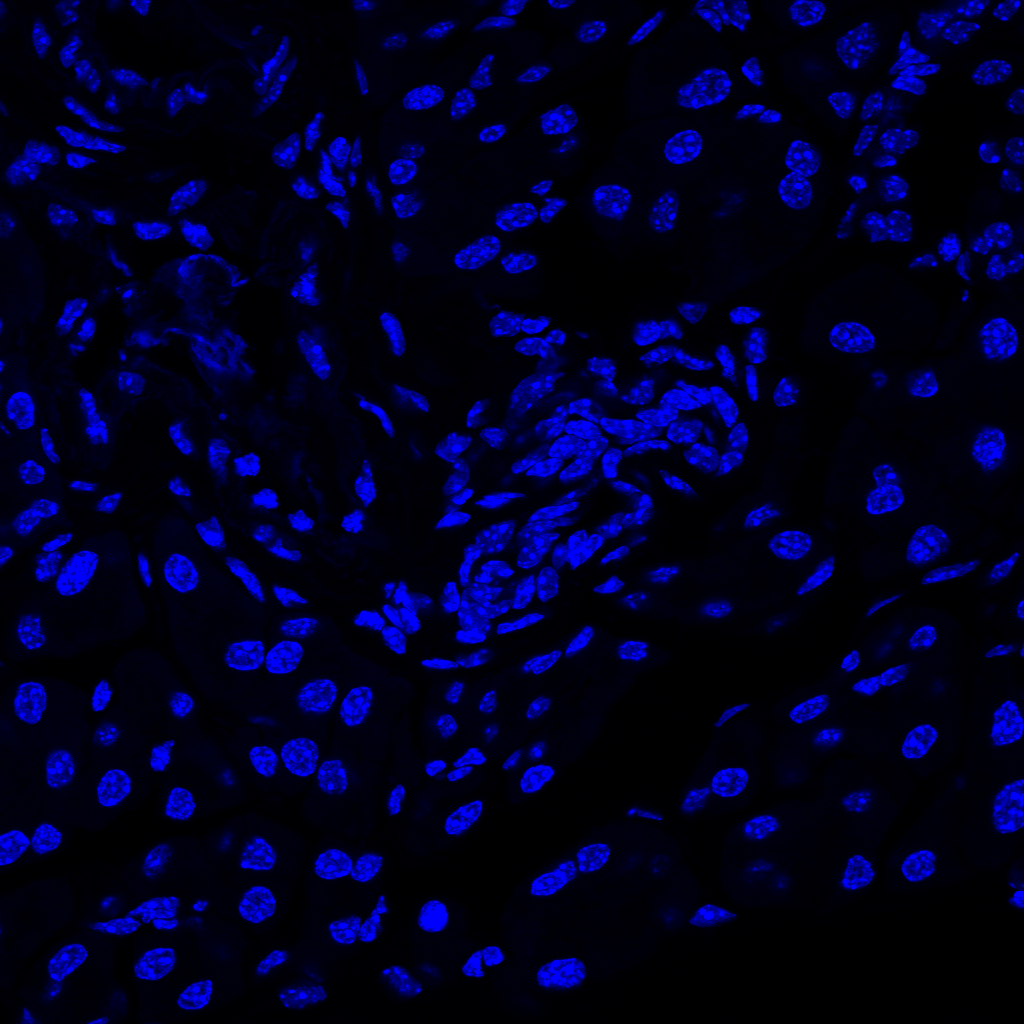

Supplement: Supplementary file 5 — Source data Fig. 3 [file 44318_2025_434_MOESM5_ESM.zip › Figure 3/3K/3K_2w_Ins (blue).tif]

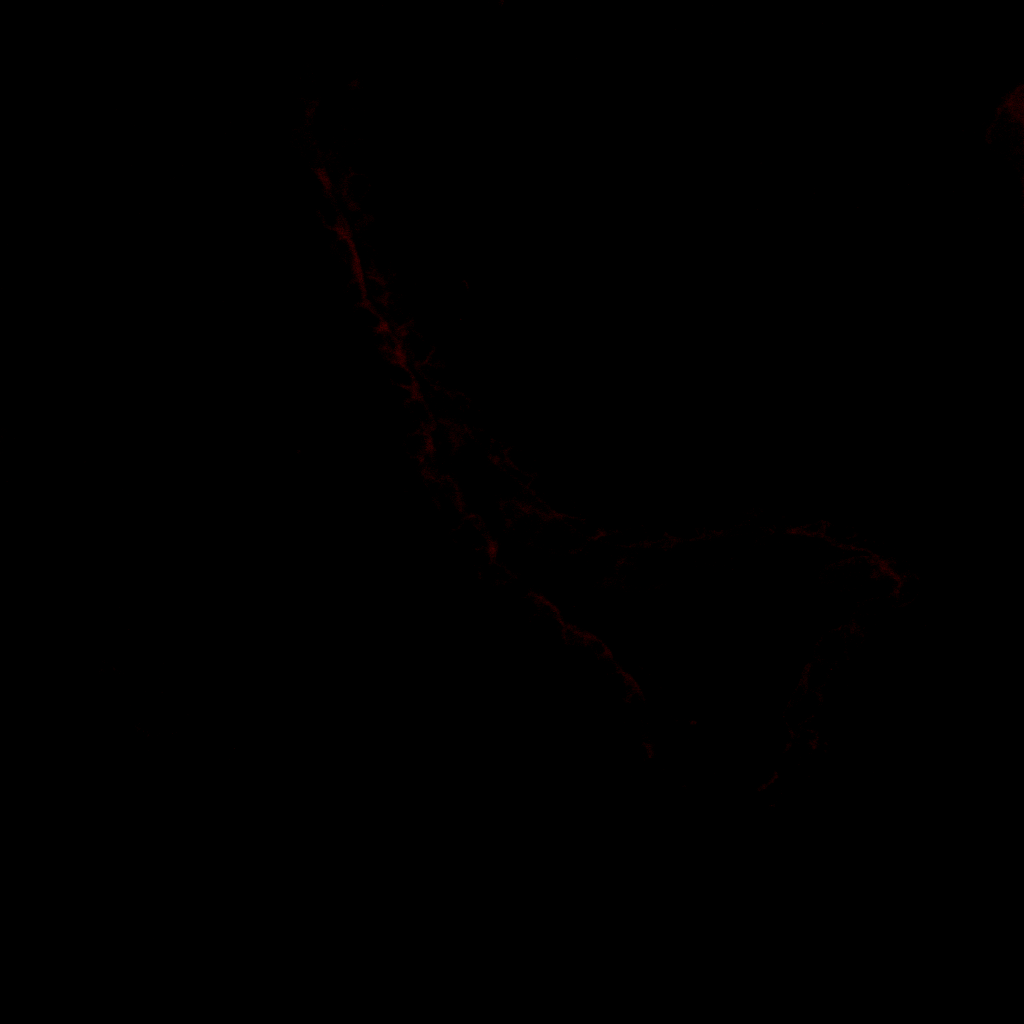

Supplement: Supplementary file 5 — Source data Fig. 3 [file 44318_2025_434_MOESM5_ESM.zip › Figure 3/3K/3K_12w_CK19 (red).tif]

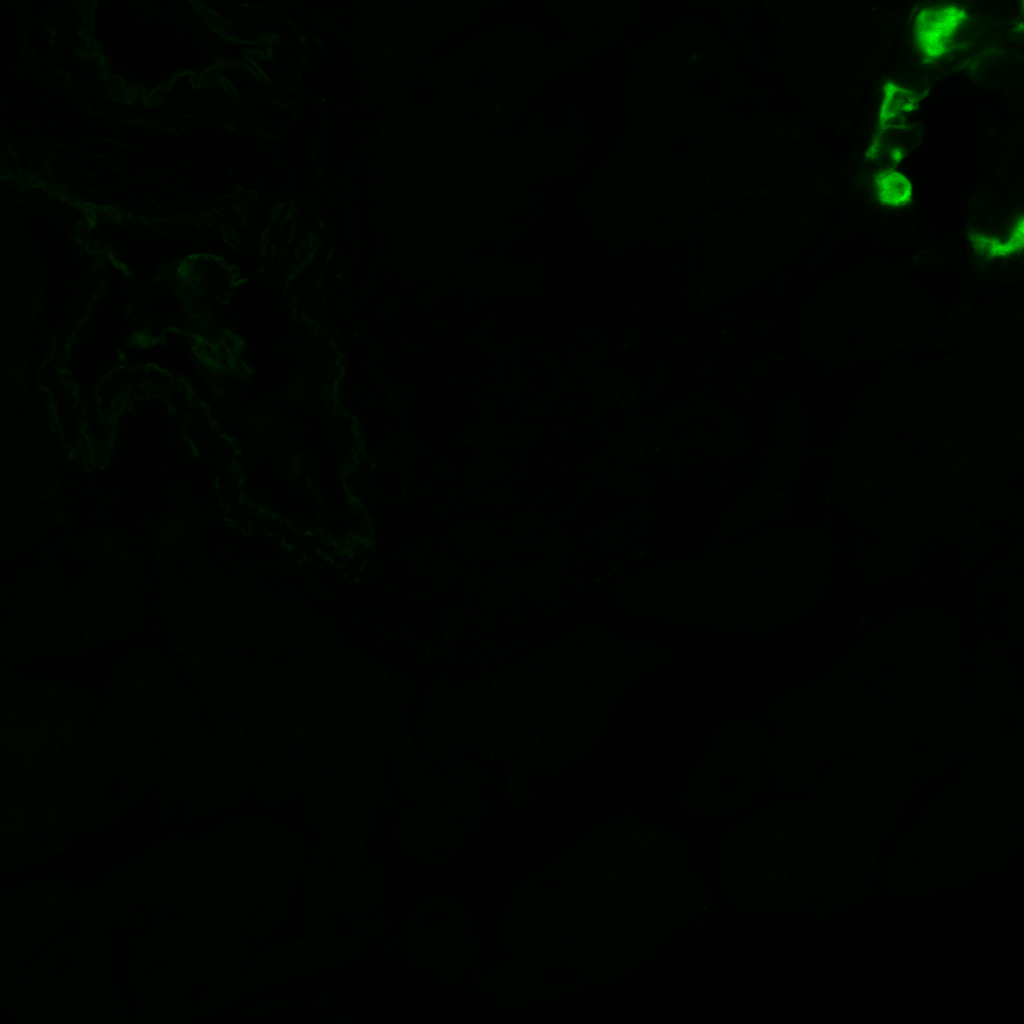

Supplement: Supplementary file 5 — Source data Fig. 3 [file 44318_2025_434_MOESM5_ESM.zip › Figure 3/3K/3K_2w_Ins (green).tif]

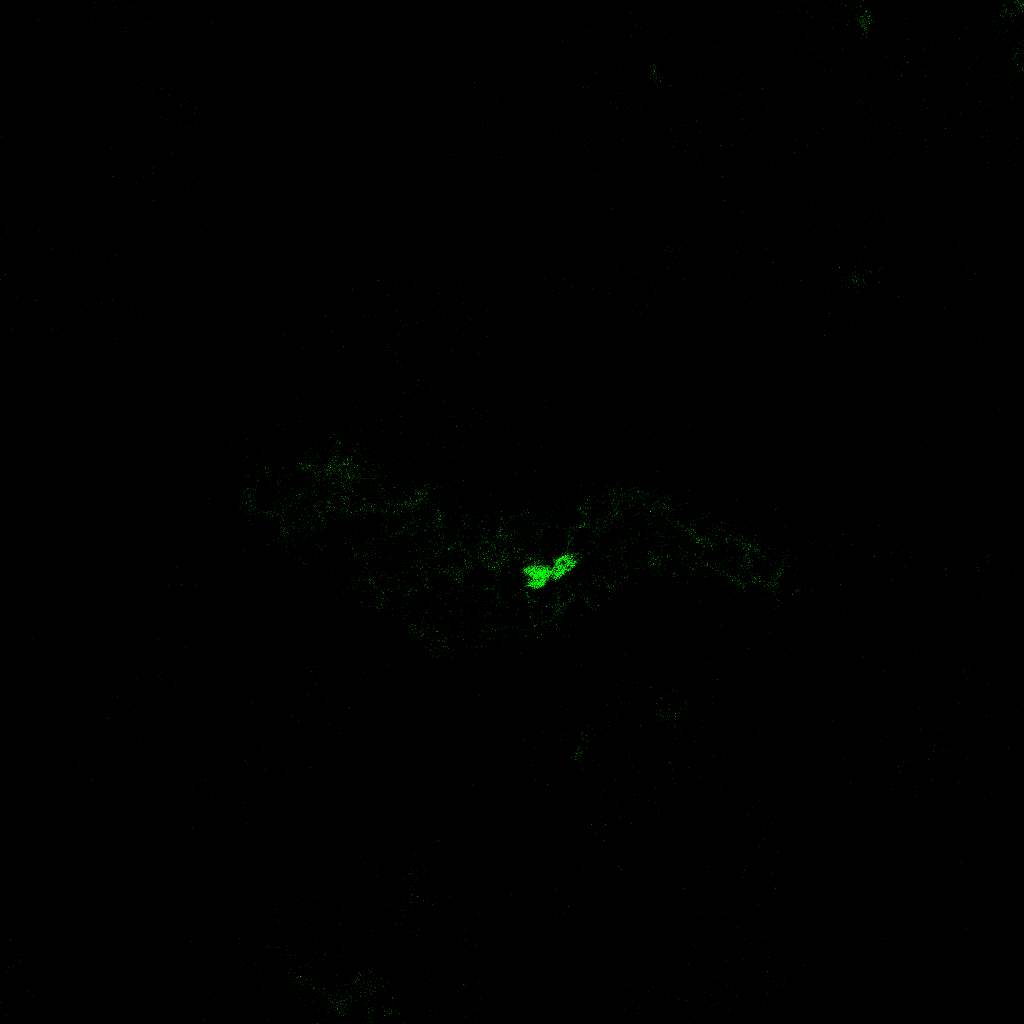

Supplement: Supplementary file 5 — Source data Fig. 3 [file 44318_2025_434_MOESM5_ESM.zip › Figure 3/3K/3K_2w_CK19 (green).tif]

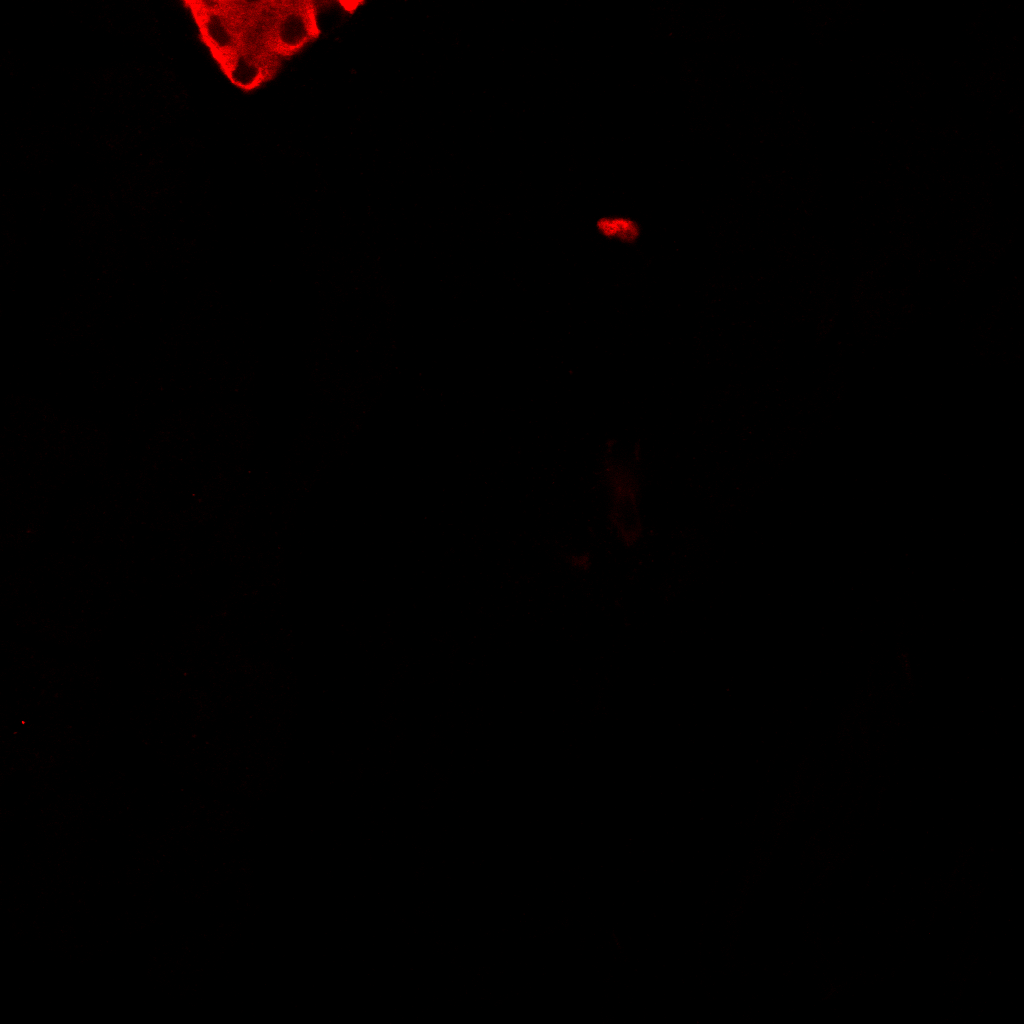

Supplement: Supplementary file 5 — Source data Fig. 3 [file 44318_2025_434_MOESM5_ESM.zip › Figure 3/3K/3K_12w_Ins (red).tiff]

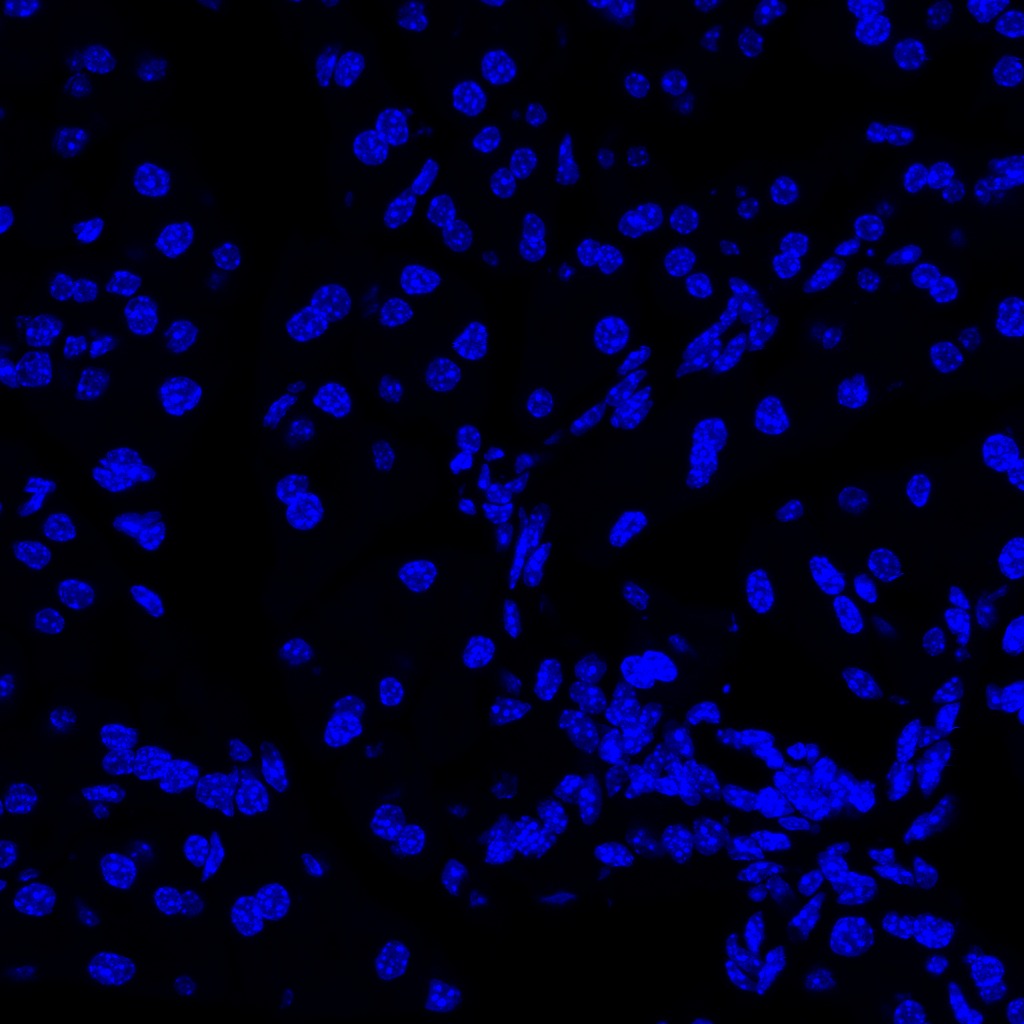

Supplement: Supplementary file 5 — Source data Fig. 3 [file 44318_2025_434_MOESM5_ESM.zip › Figure 3/3K/3K_2w_Sst (blue).tif]

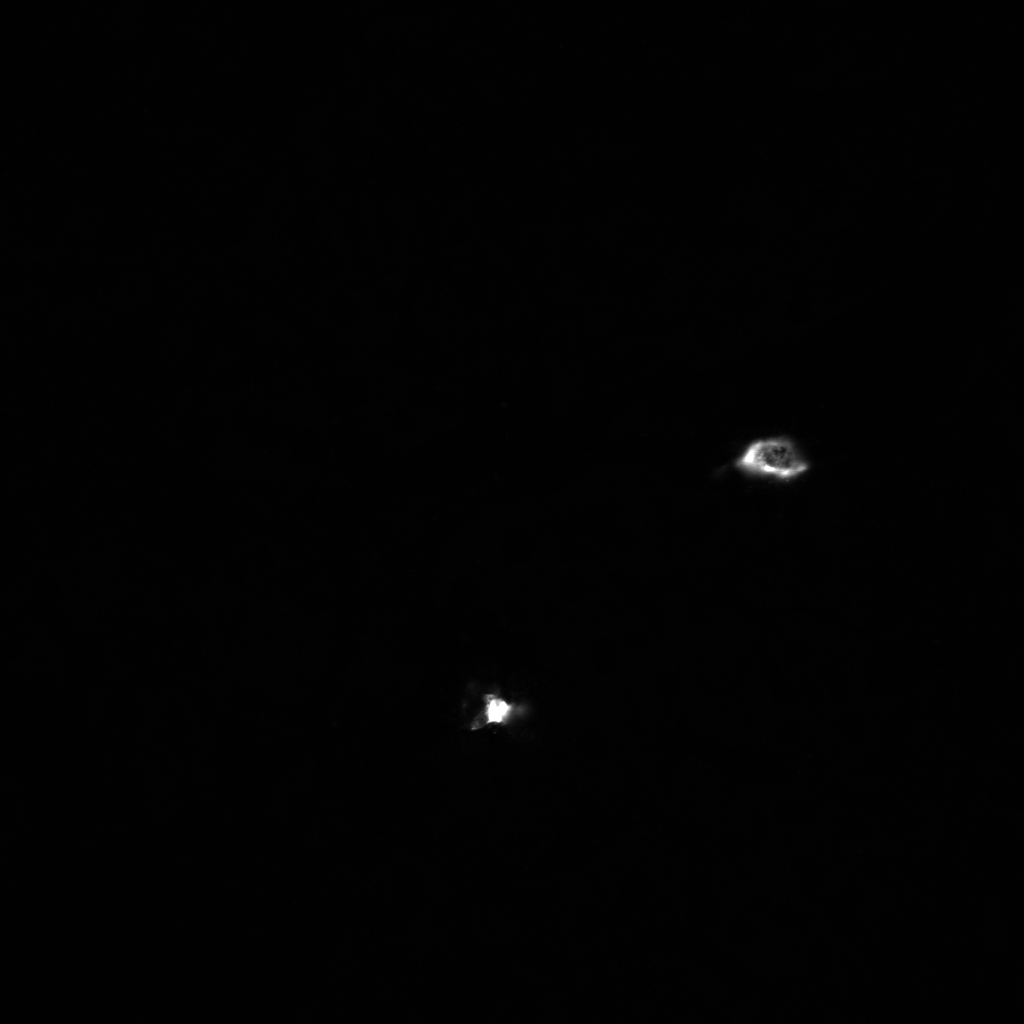

Supplement: Supplementary file 5 — Source data Fig. 3 [file 44318_2025_434_MOESM5_ESM.zip › Figure 3/3K/3K_12w_Sst (gray).tif]

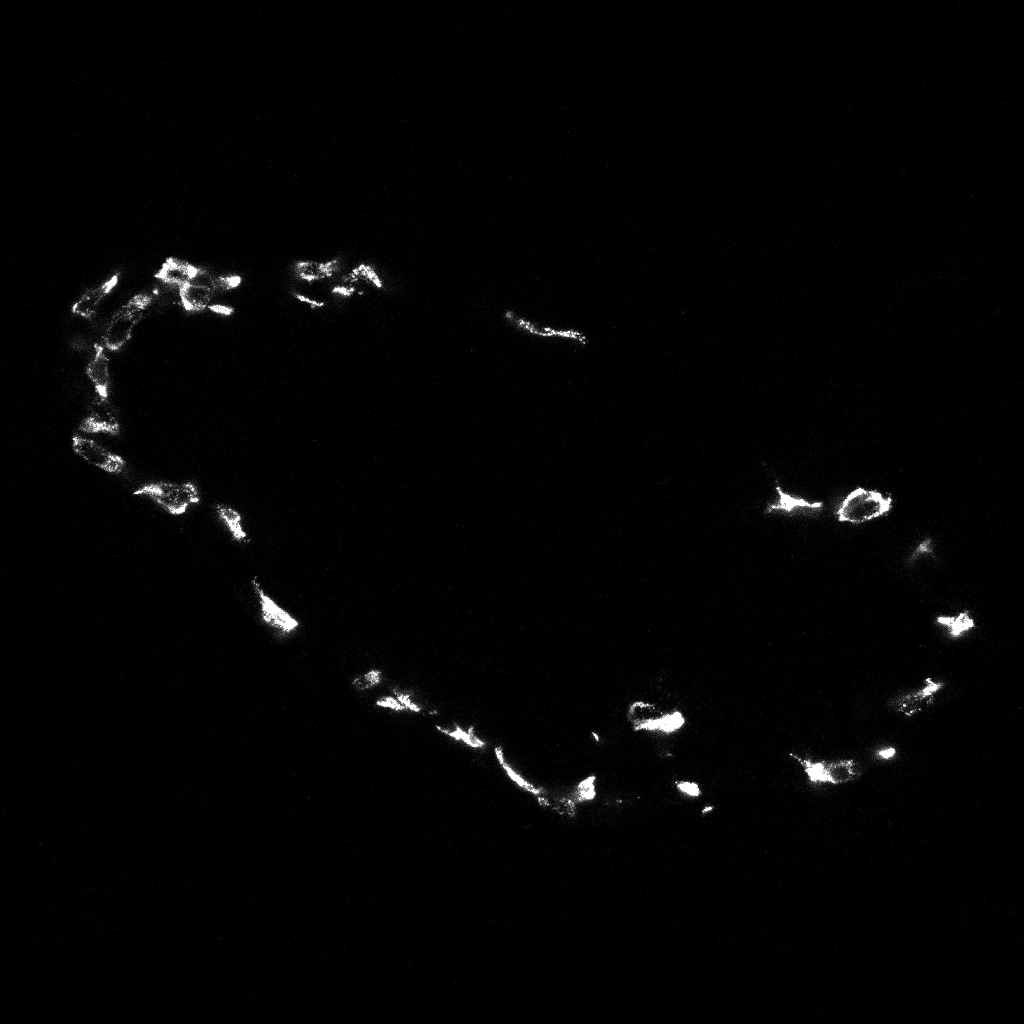

Supplement: Supplementary file 5 — Source data Fig. 3 [file 44318_2025_434_MOESM5_ESM.zip › Figure 3/3L/3L_2w_Sst (gray).tif]

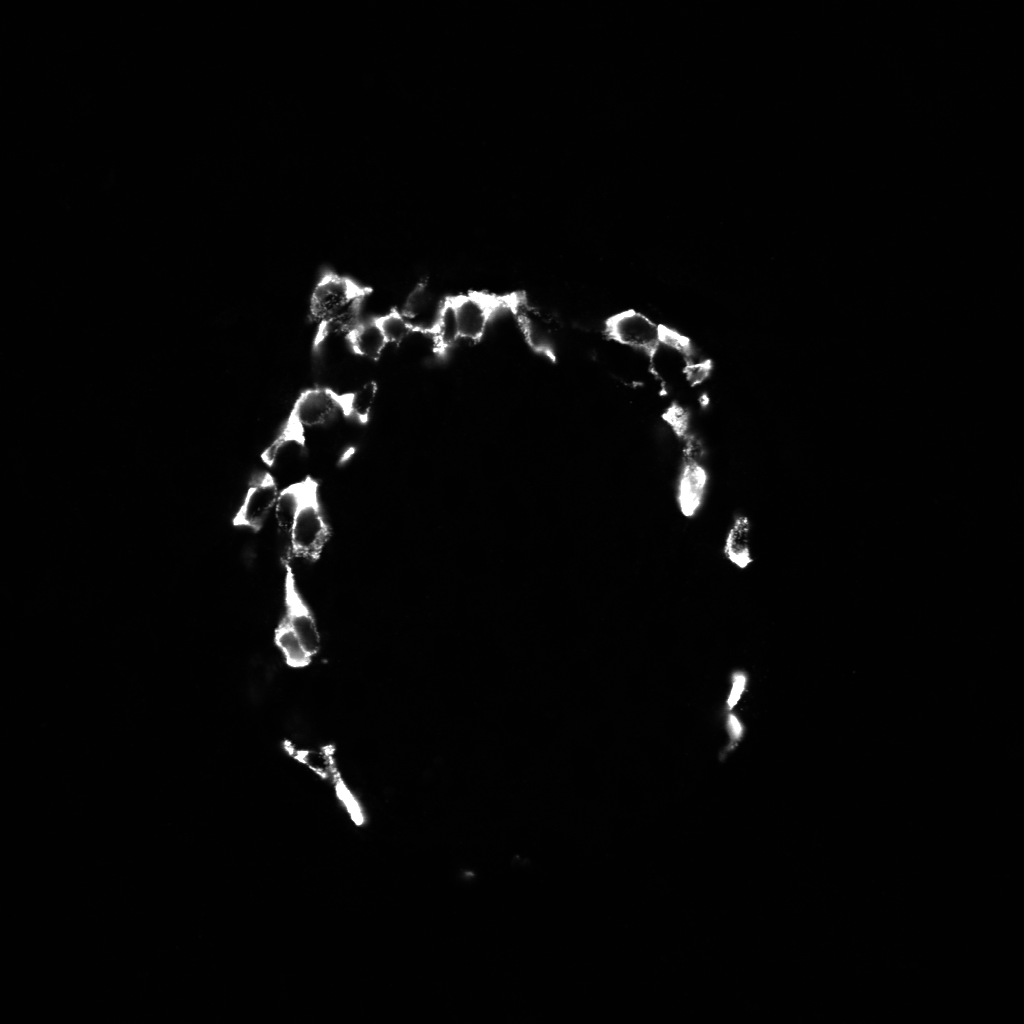

Supplement: Supplementary file 5 — Source data Fig. 3 [file 44318_2025_434_MOESM5_ESM.zip › Figure 3/3L/3L_12w_Sst (gray).tif]

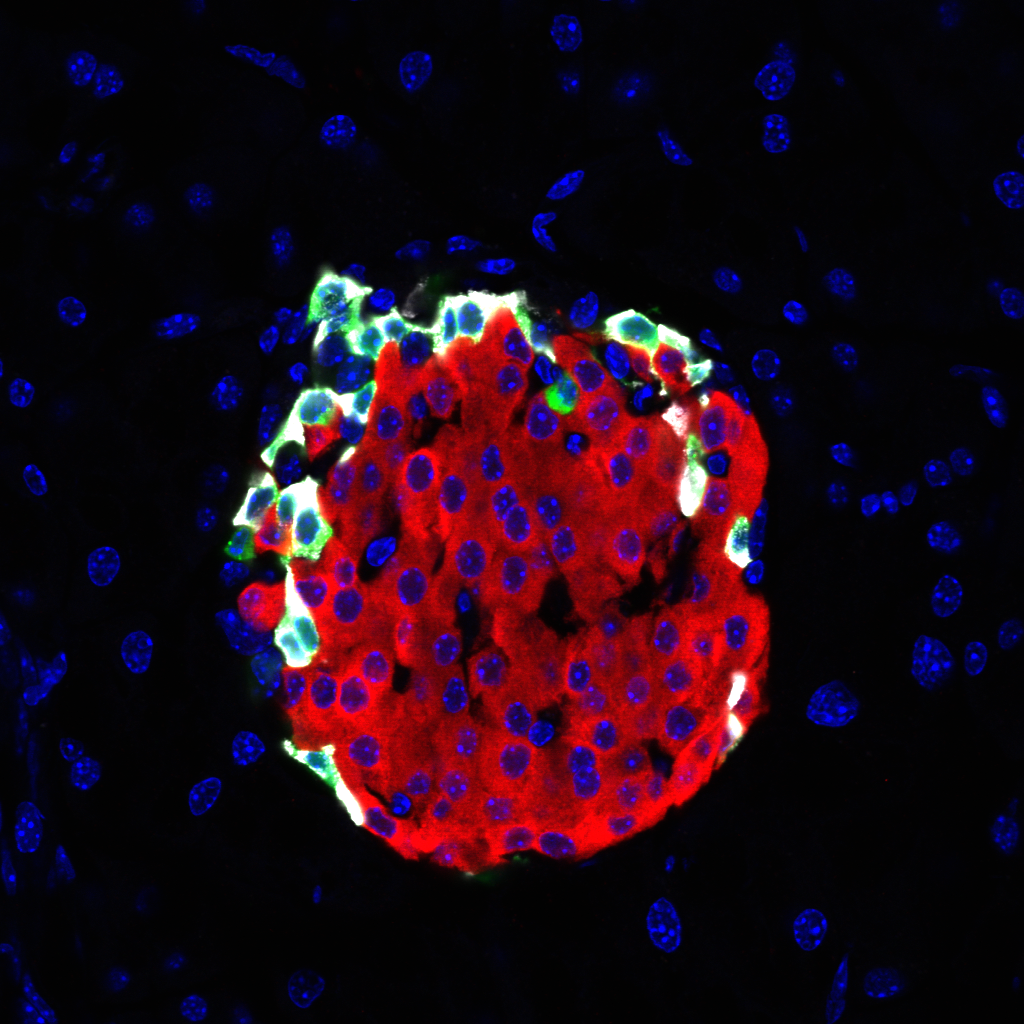

Supplement: Supplementary file 5 — Source data Fig. 3 [file 44318_2025_434_MOESM5_ESM.zip › Figure 3/3L/3L_12w_Sst.tif]

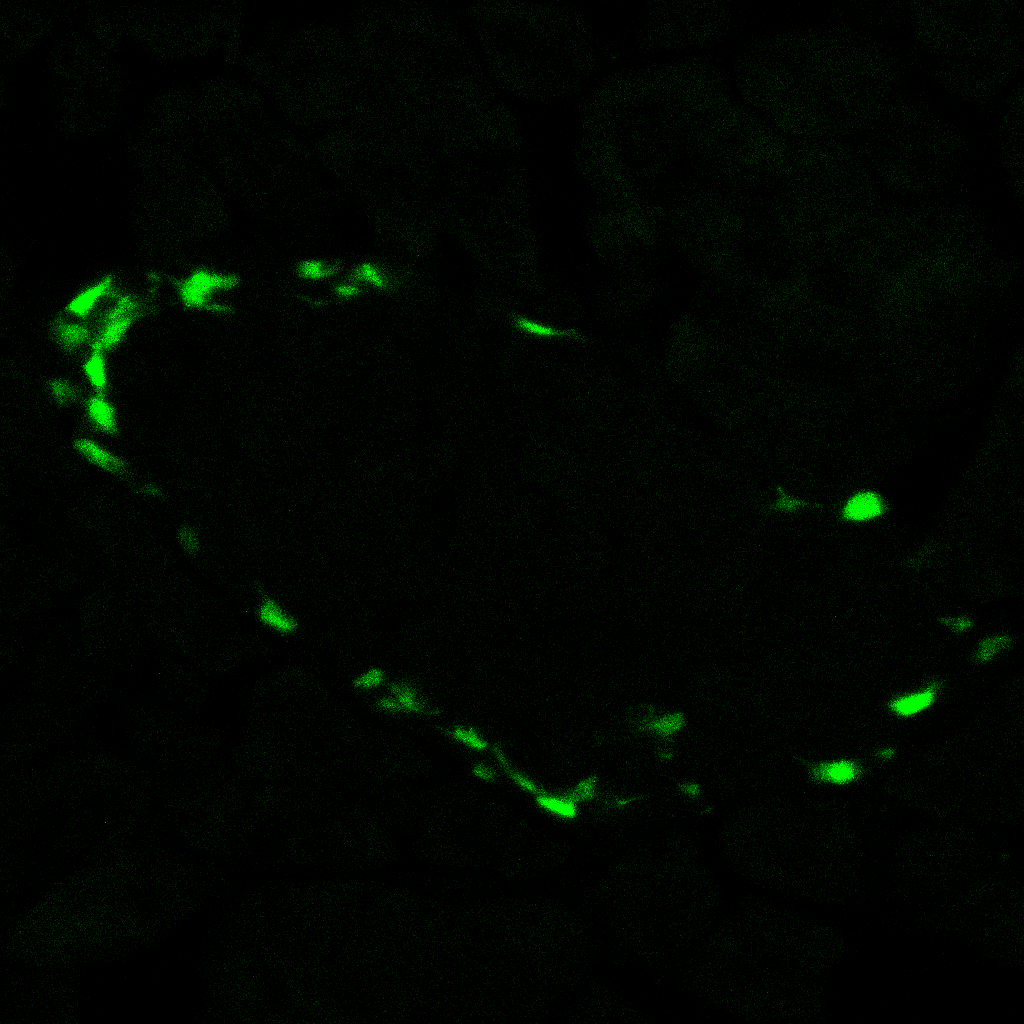

Supplement: Supplementary file 5 — Source data Fig. 3 [file 44318_2025_434_MOESM5_ESM.zip › Figure 3/3L/3L_2w_Sst (green).tif]

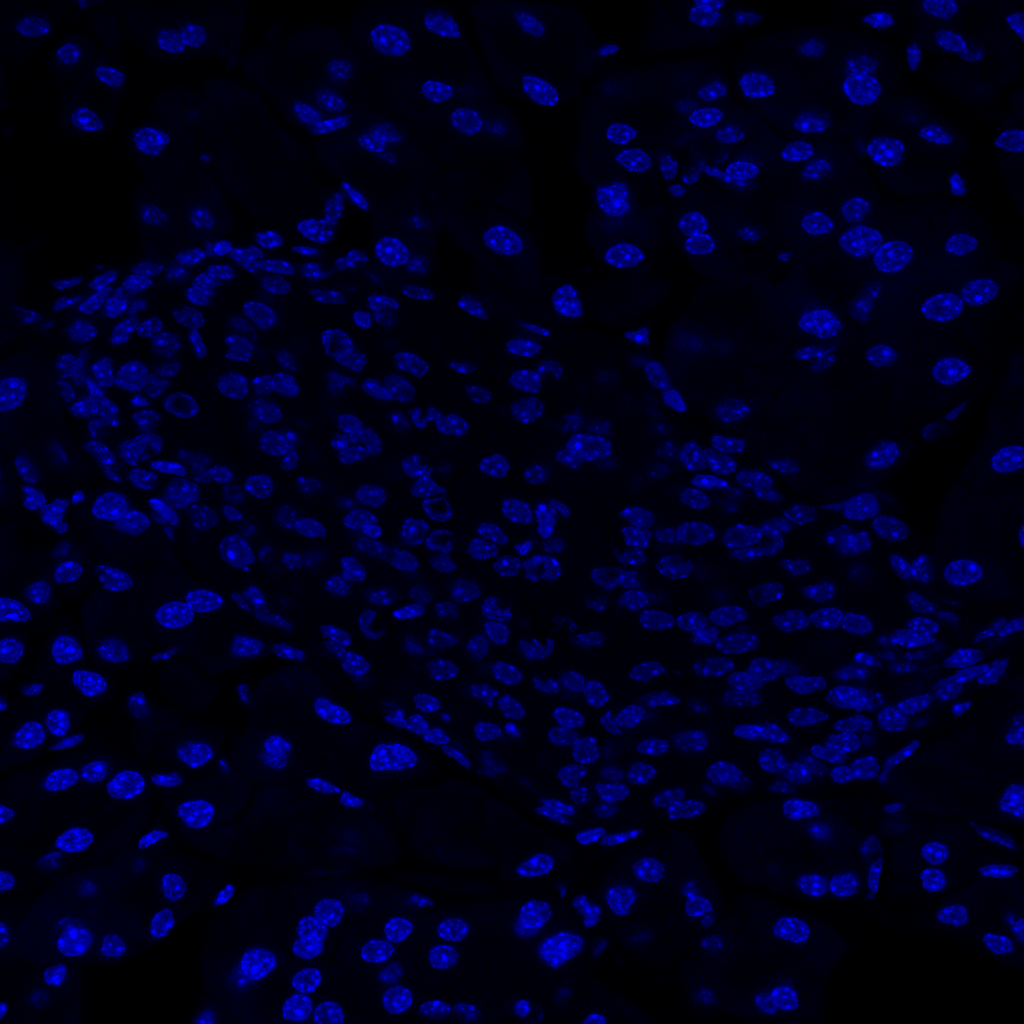

Supplement: Supplementary file 5 — Source data Fig. 3 [file 44318_2025_434_MOESM5_ESM.zip › Figure 3/3L/3L_2w_Sst (blue).tif]

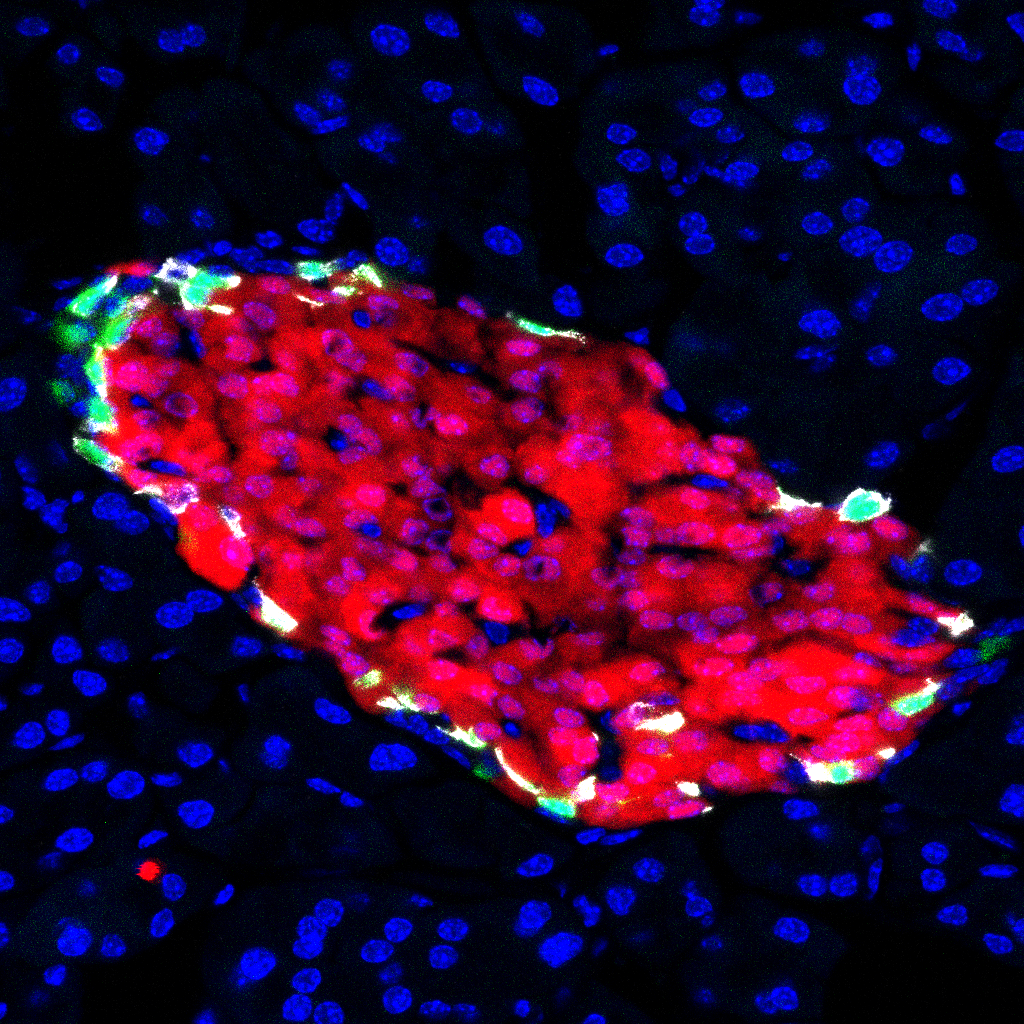

Supplement: Supplementary file 5 — Source data Fig. 3 [file 44318_2025_434_MOESM5_ESM.zip › Figure 3/3L/3L_2w_Sst.tif]

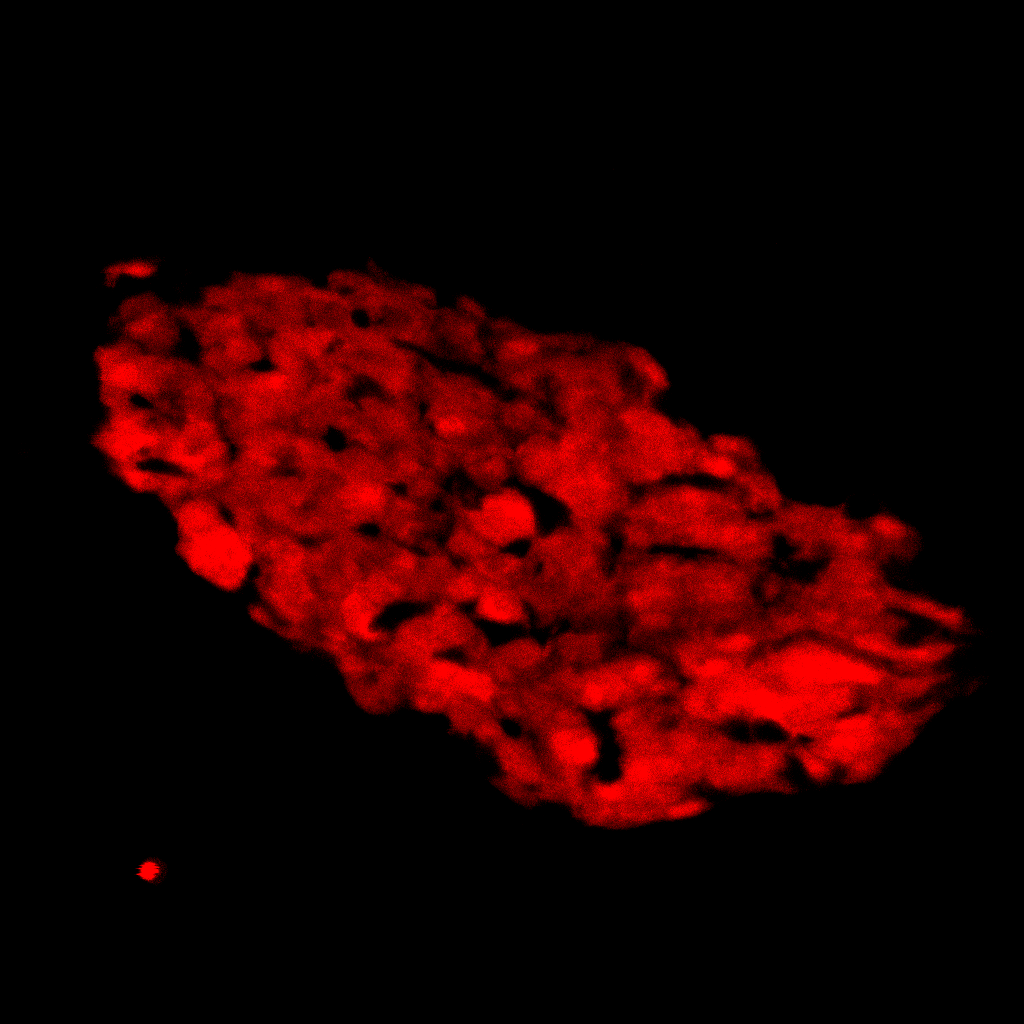

Supplement: Supplementary file 5 — Source data Fig. 3 [file 44318_2025_434_MOESM5_ESM.zip › Figure 3/3L/3L_2w_Sst (red).tif]

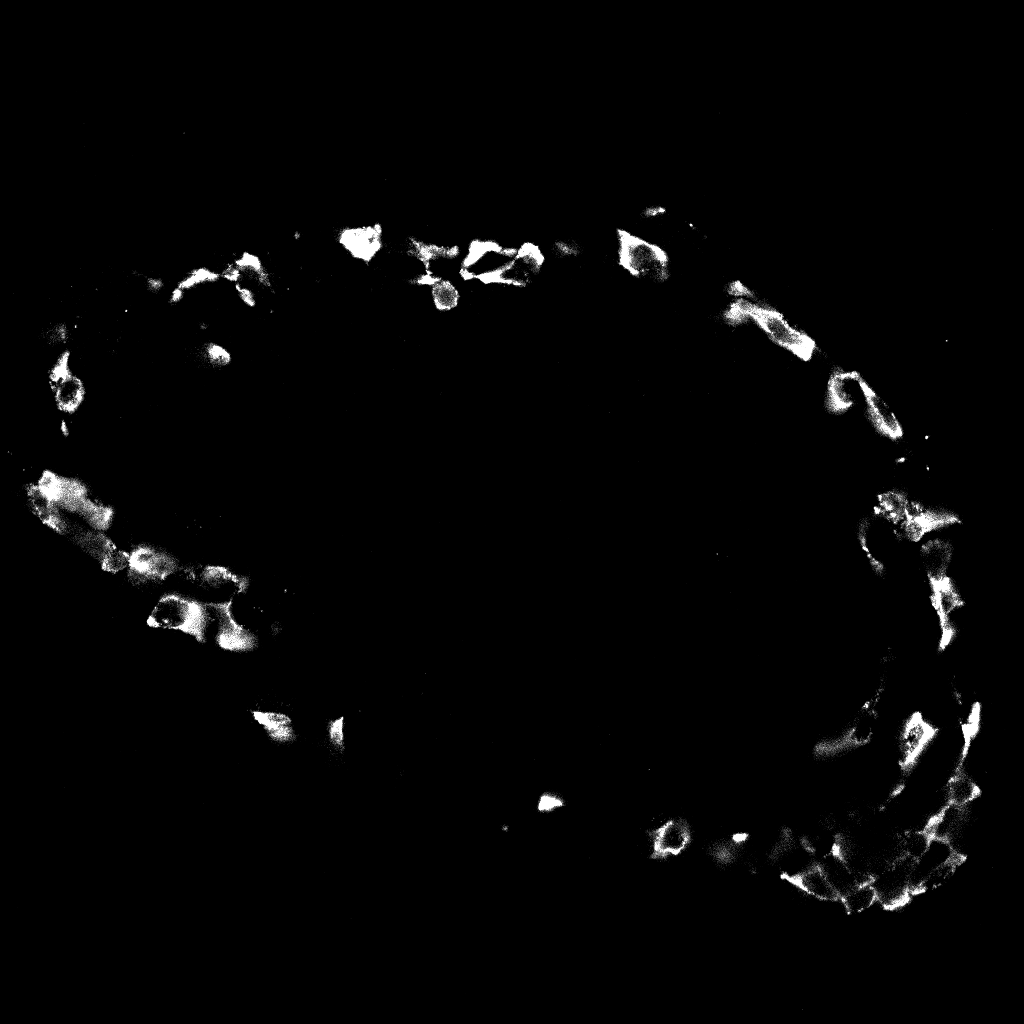

Supplement: Supplementary file 5 — Source data Fig. 3 [file 44318_2025_434_MOESM5_ESM.zip › Figure 3/3E/3E-Merge (gray).tif]

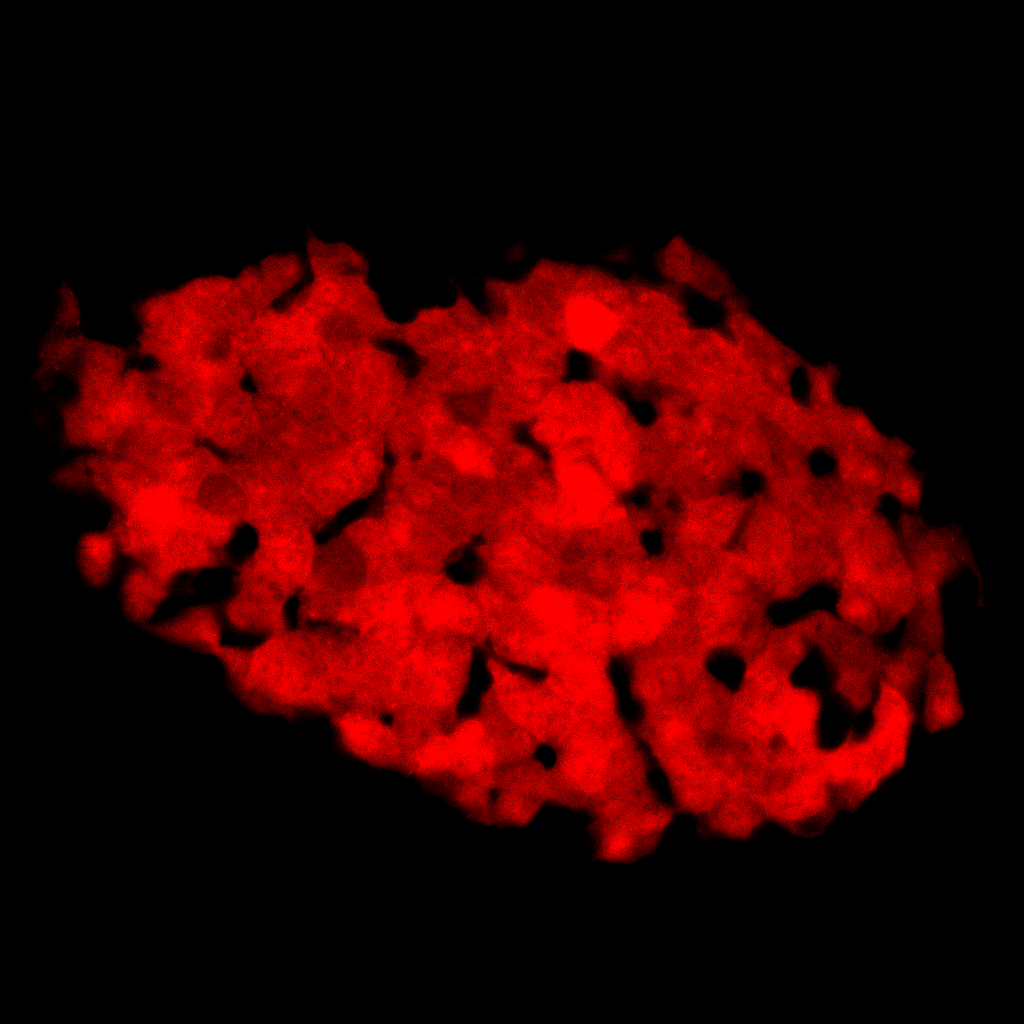

Supplement: Supplementary file 5 — Source data Fig. 3 [file 44318_2025_434_MOESM5_ESM.zip › Figure 3/3E/3E-Merge (red).tif]

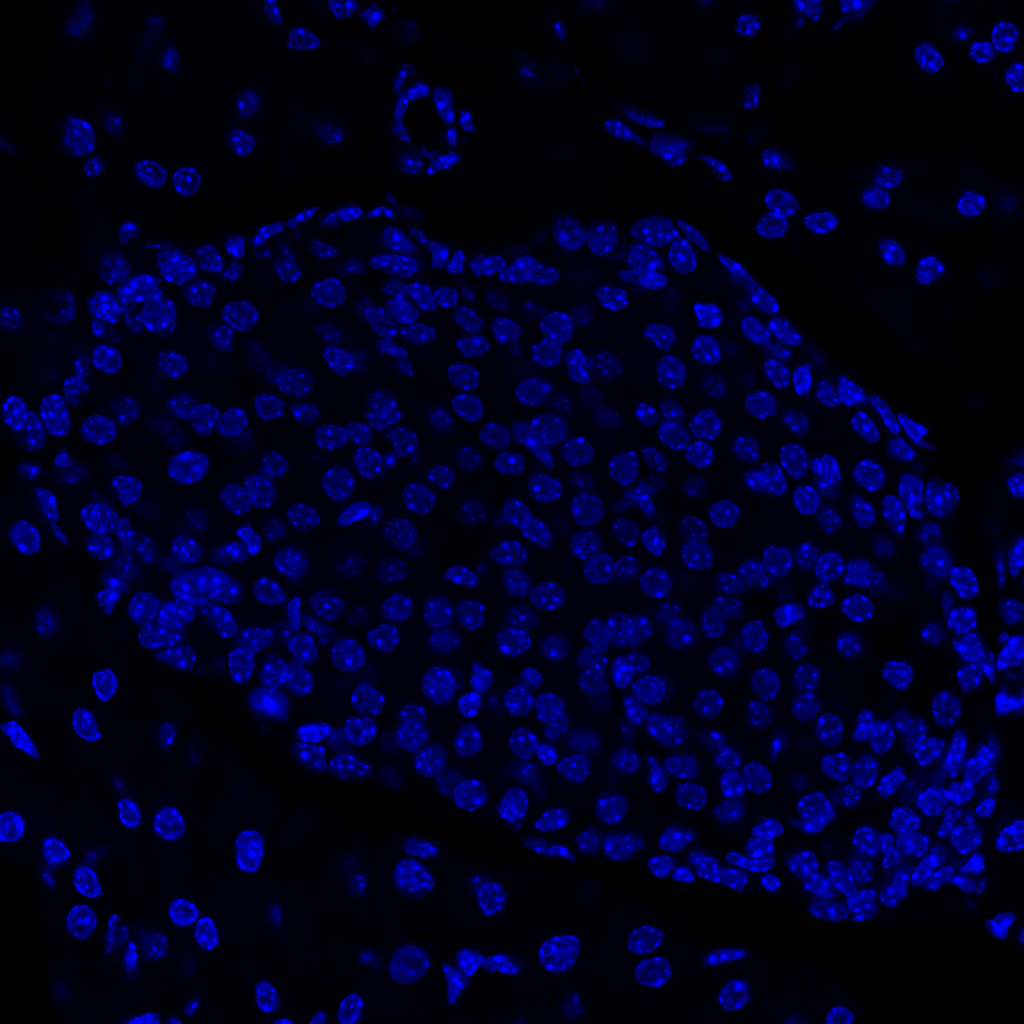

Supplement: Supplementary file 5 — Source data Fig. 3 [file 44318_2025_434_MOESM5_ESM.zip › Figure 3/3E/3E-Merge (blue).tif]

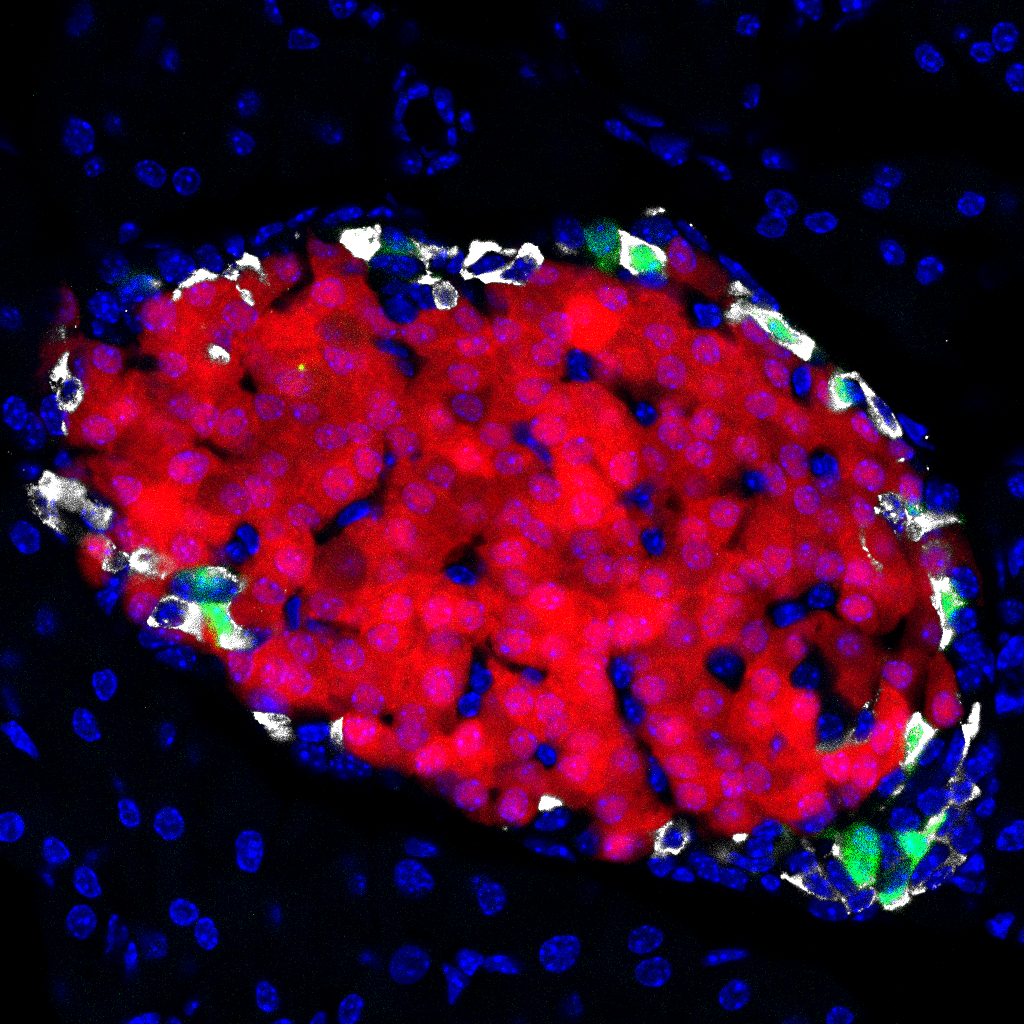

Supplement: Supplementary file 5 — Source data Fig. 3 [file 44318_2025_434_MOESM5_ESM.zip › Figure 3/3E/3E-Merge.tif]

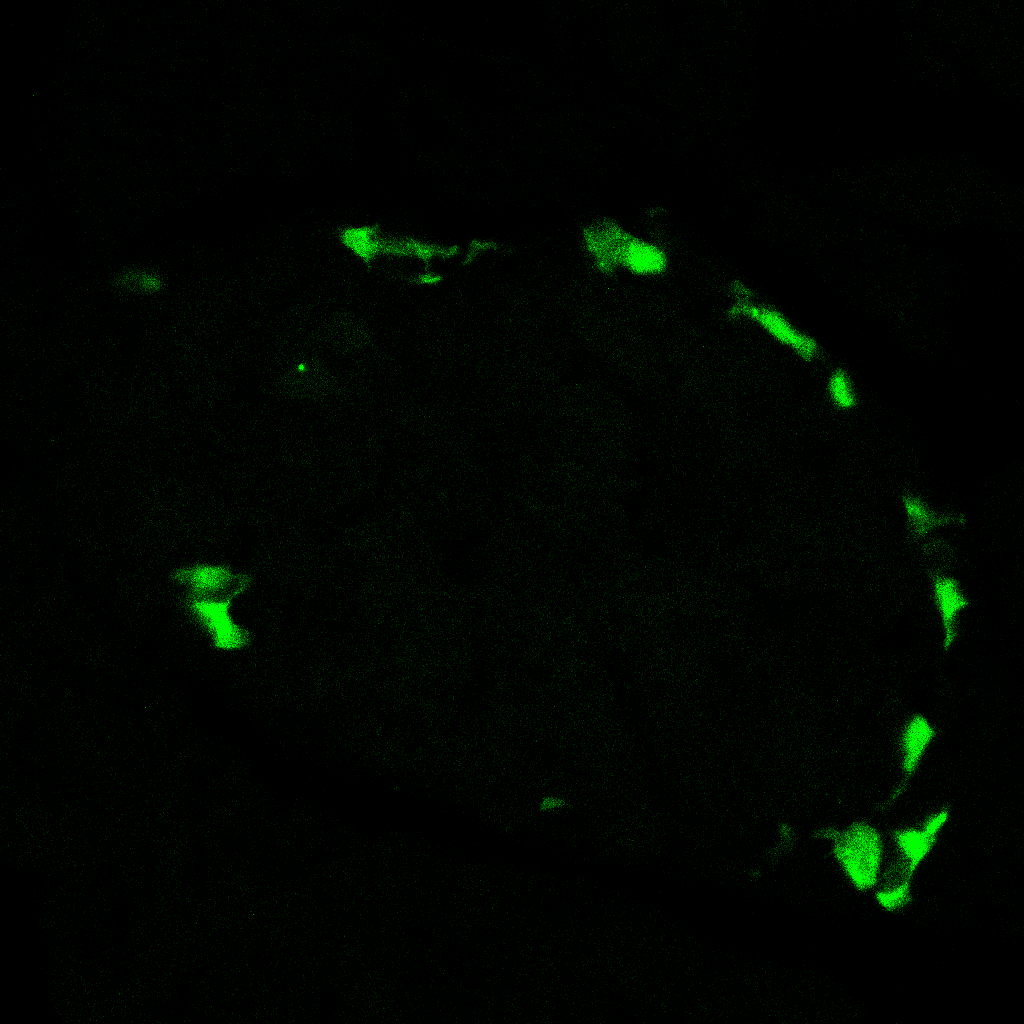

Supplement: Supplementary file 5 — Source data Fig. 3 [file 44318_2025_434_MOESM5_ESM.zip › Figure 3/3E/3E-Merge (green).tif]

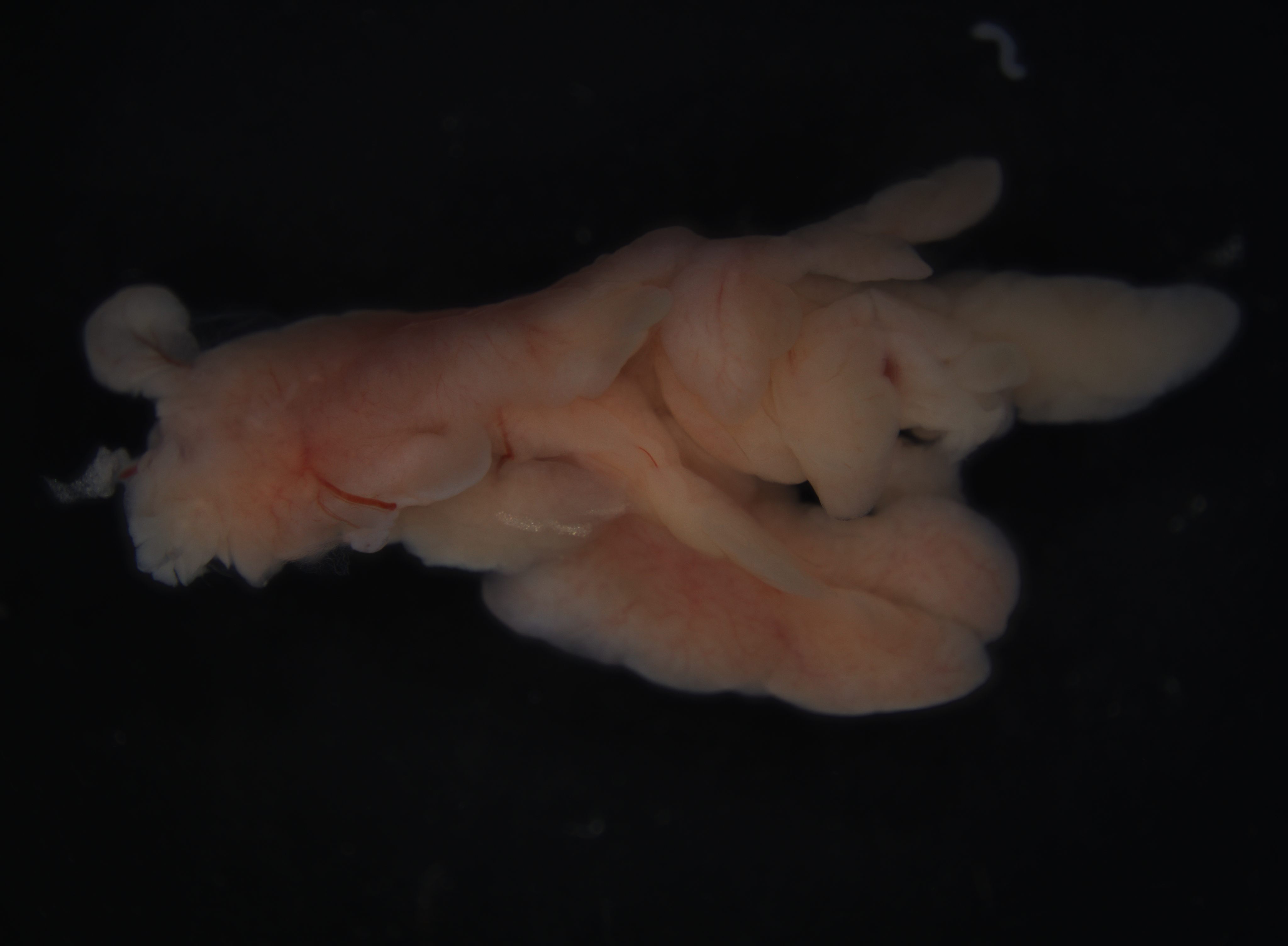

Supplement: Supplementary file 5 — Source data Fig. 3 [file 44318_2025_434_MOESM5_ESM.zip › Figure 3/3C/3C_BF.tif]

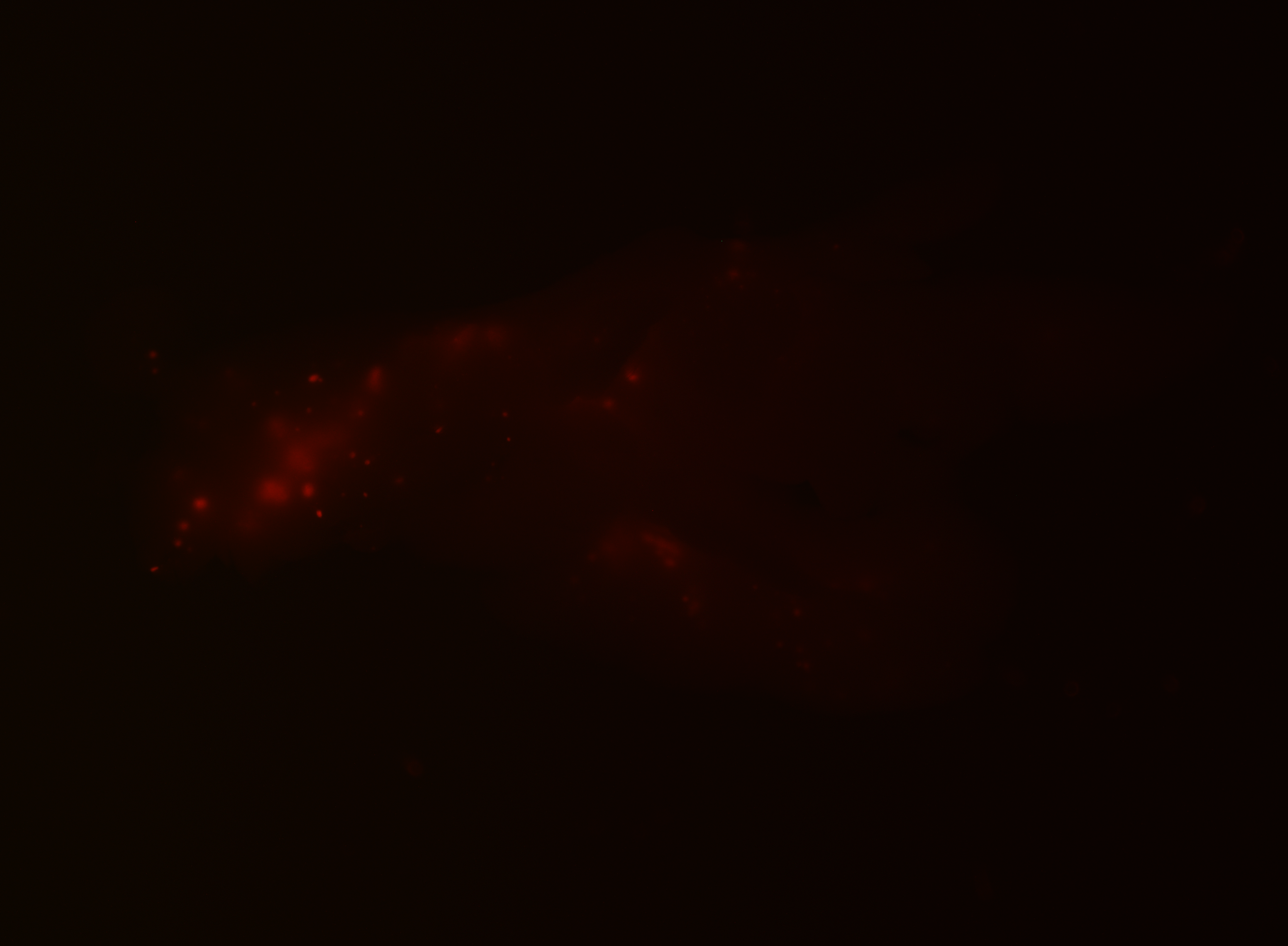

Supplement: Supplementary file 5 — Source data Fig. 3 [file 44318_2025_434_MOESM5_ESM.zip › Figure 3/3C/3C_tdT.tif]

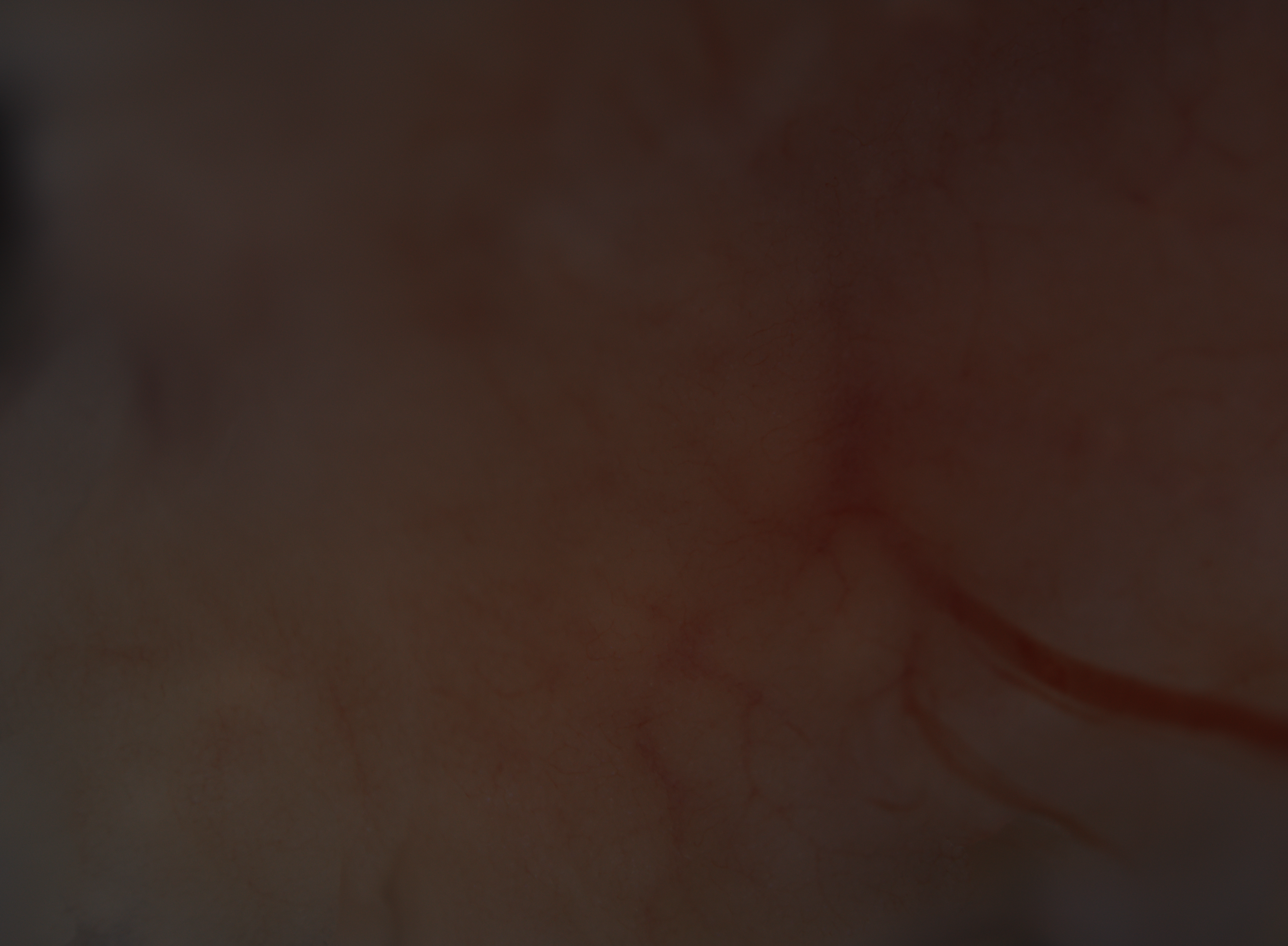

Supplement: Supplementary file 5 — Source data Fig. 3 [file 44318_2025_434_MOESM5_ESM.zip › Figure 3/3C/3C_tdT_mag.tif]

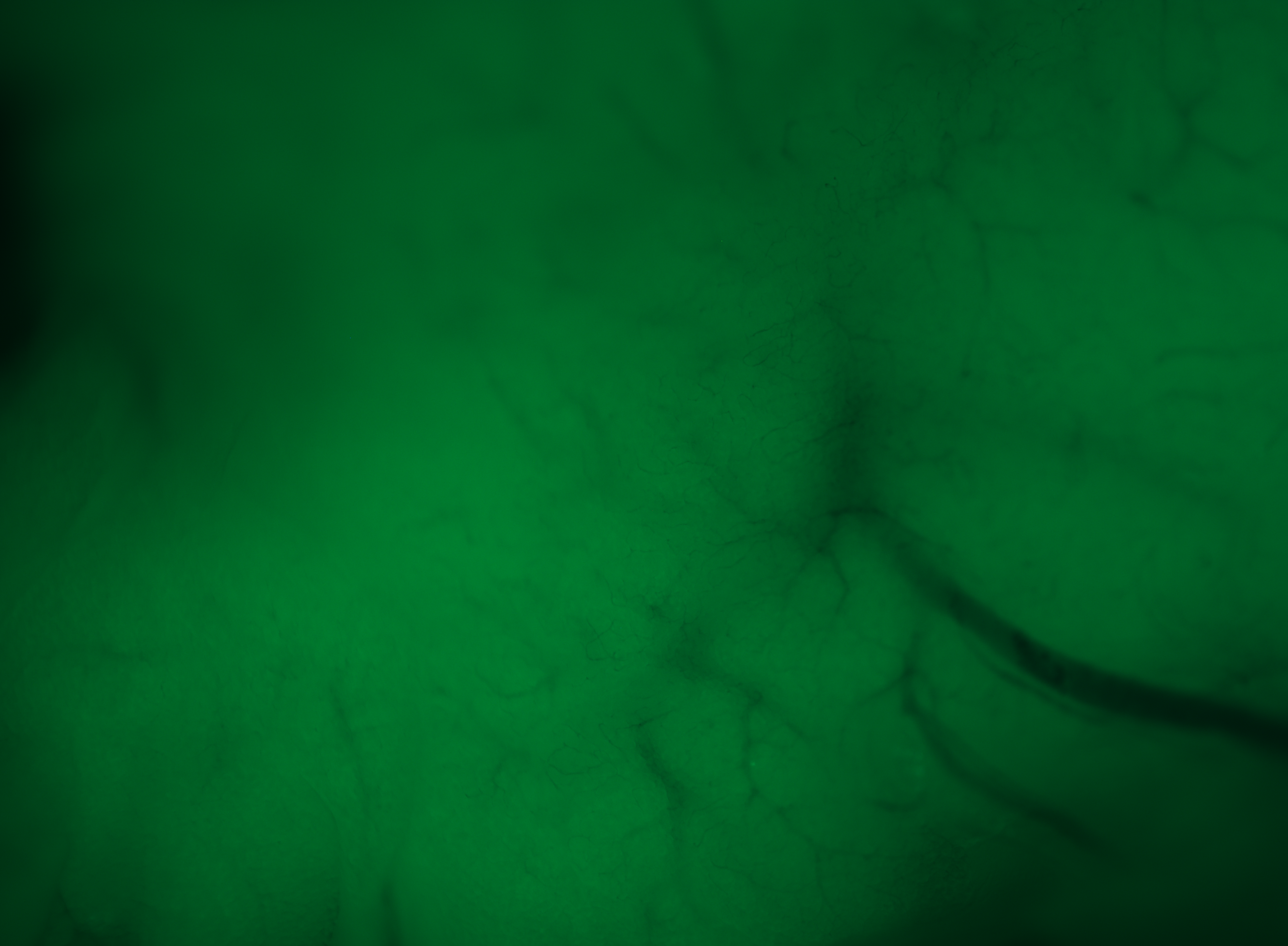

Supplement: Supplementary file 5 — Source data Fig. 3 [file 44318_2025_434_MOESM5_ESM.zip › Figure 3/3C/3C_zsGreen_mag.tif]

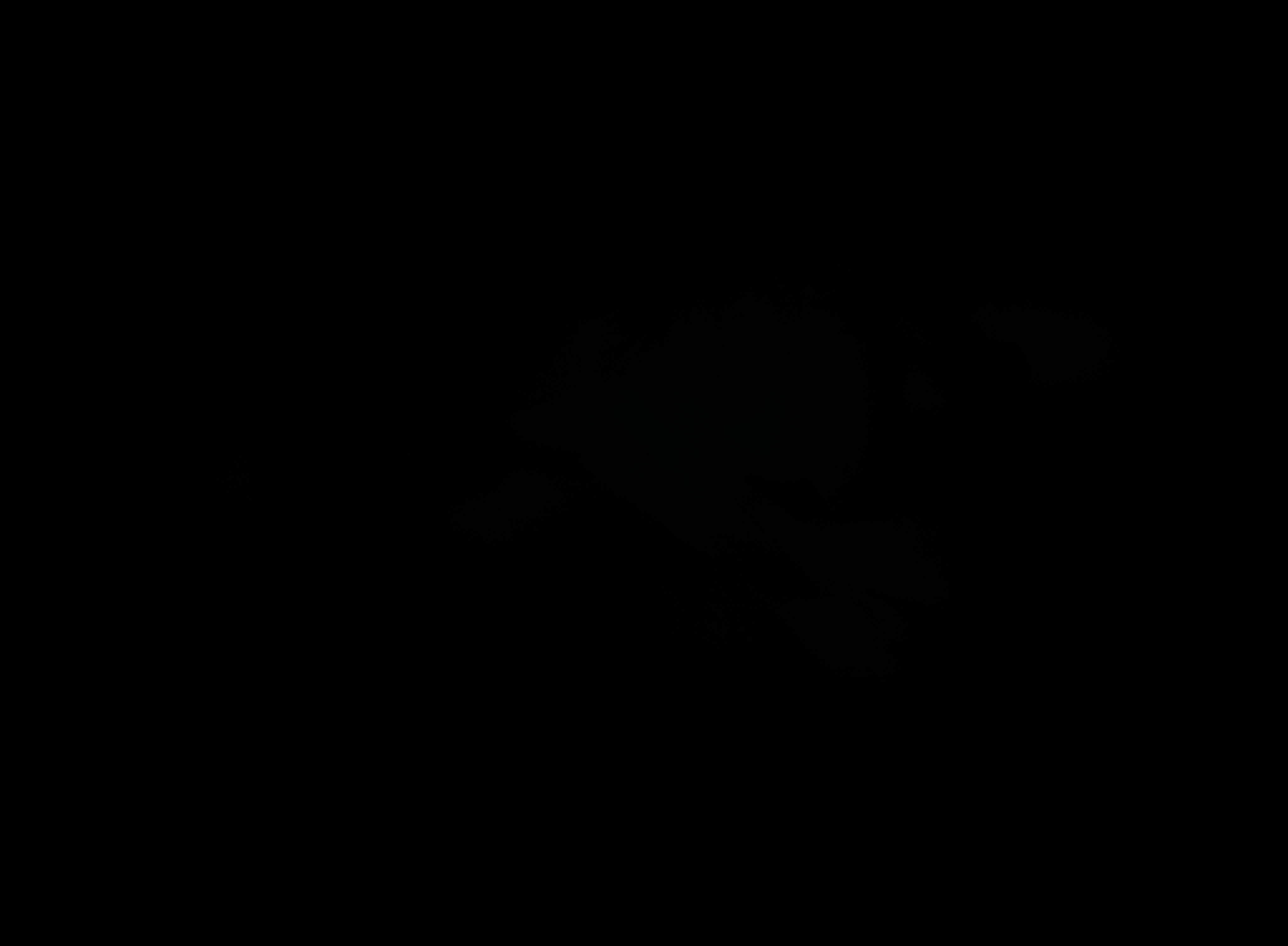

Supplement: Supplementary file 5 — Source data Fig. 3 [file 44318_2025_434_MOESM5_ESM.zip › Figure 3/3C/3C_zsGreen.tif]

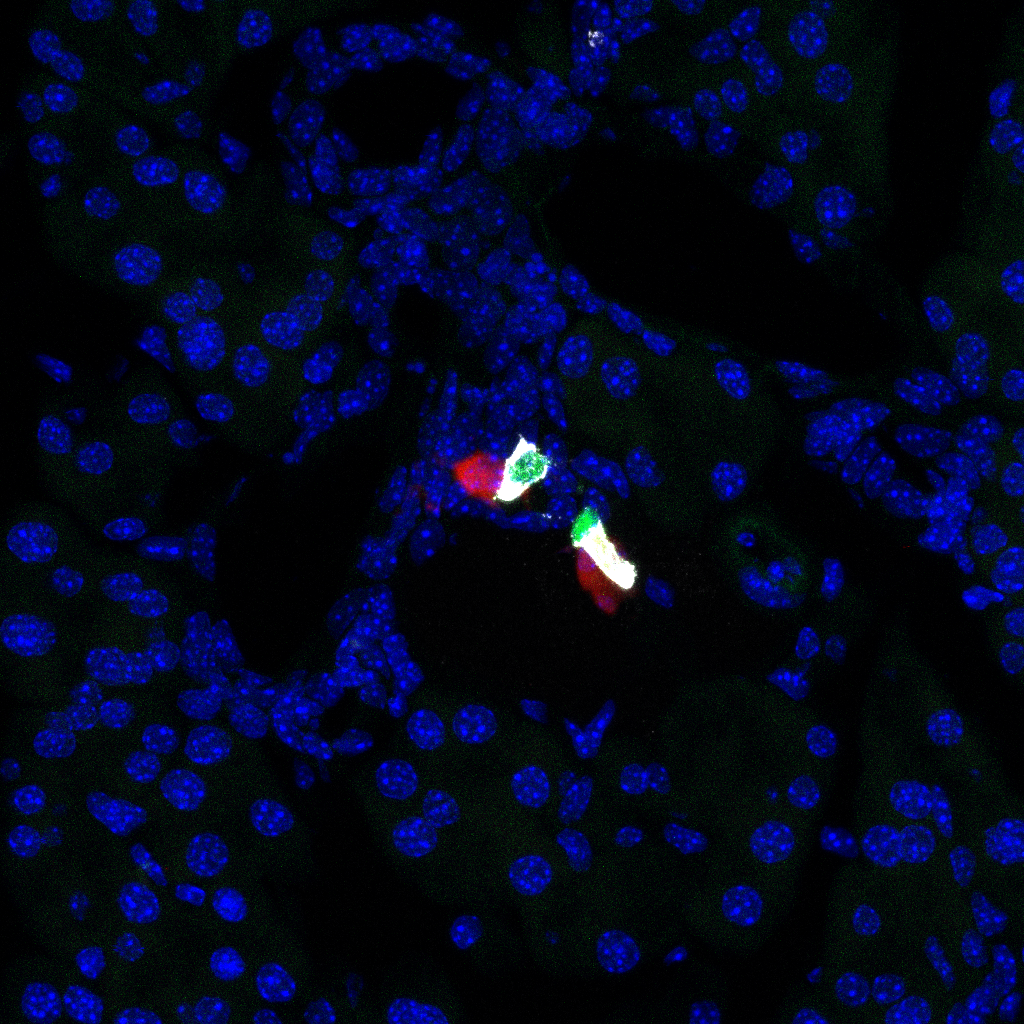

Supplement: Supplementary file 5 — Source data Fig. 3 [file 44318_2025_434_MOESM5_ESM.zip › Figure 3/3D/3D_Sst.tif]

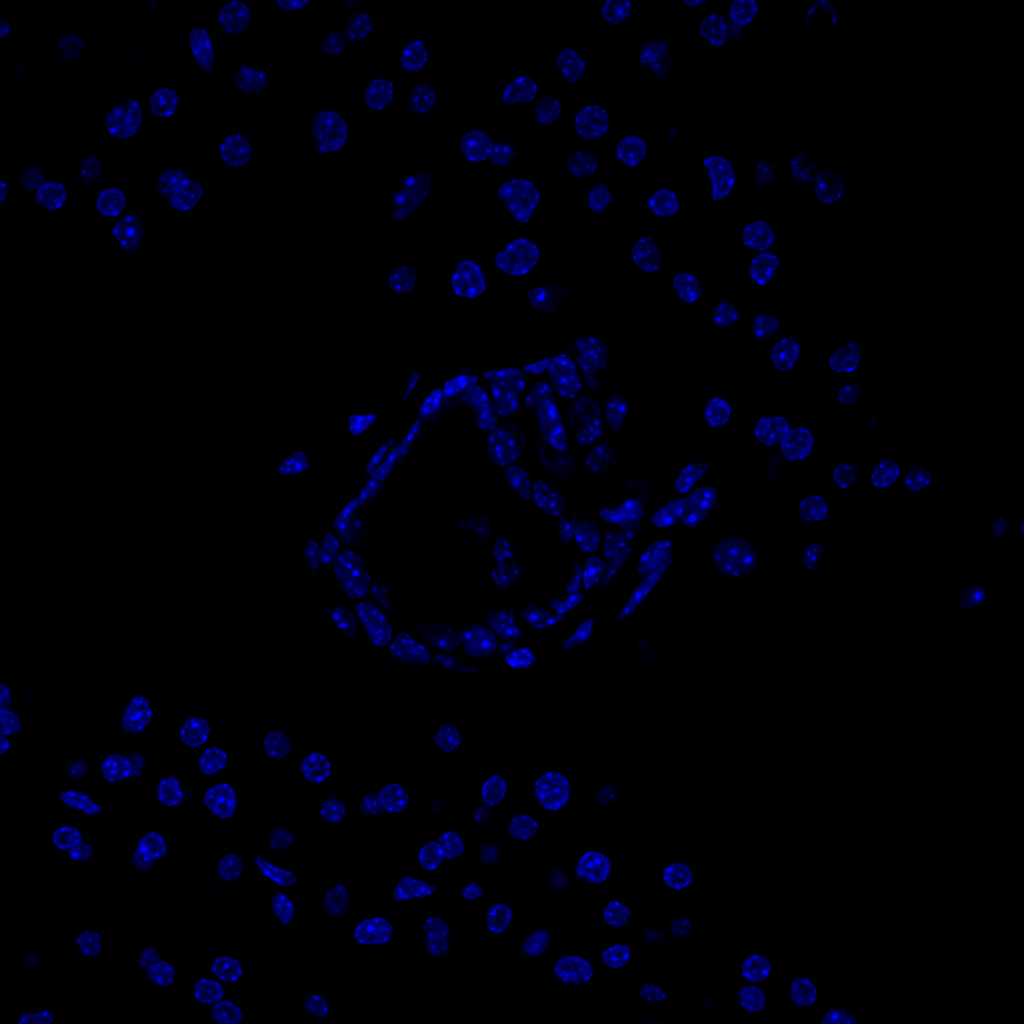

Supplement: Supplementary file 5 — Source data Fig. 3 [file 44318_2025_434_MOESM5_ESM.zip › Figure 3/3D/3D_CK19 (blue).tif]

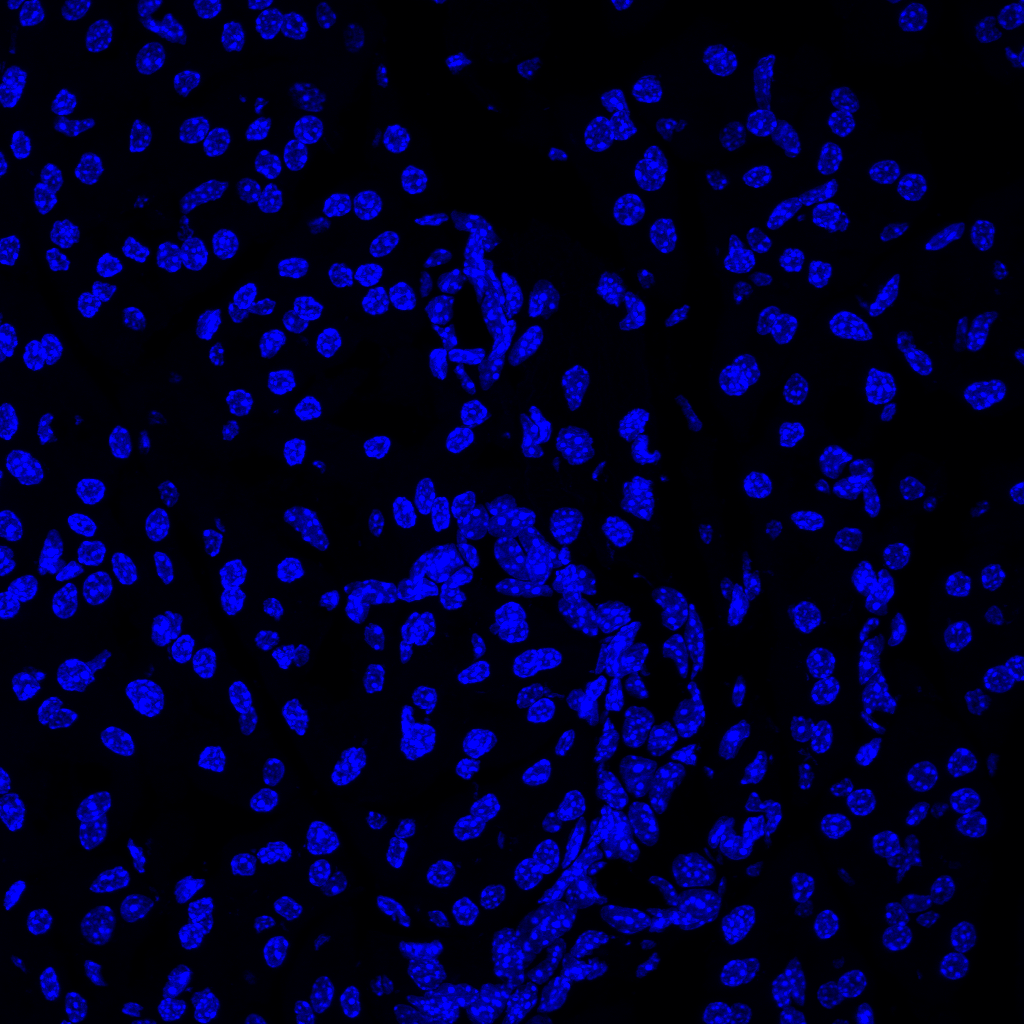

Supplement: Supplementary file 5 — Source data Fig. 3 [file 44318_2025_434_MOESM5_ESM.zip › Figure 3/3D/3D_Ins (blue).tif]

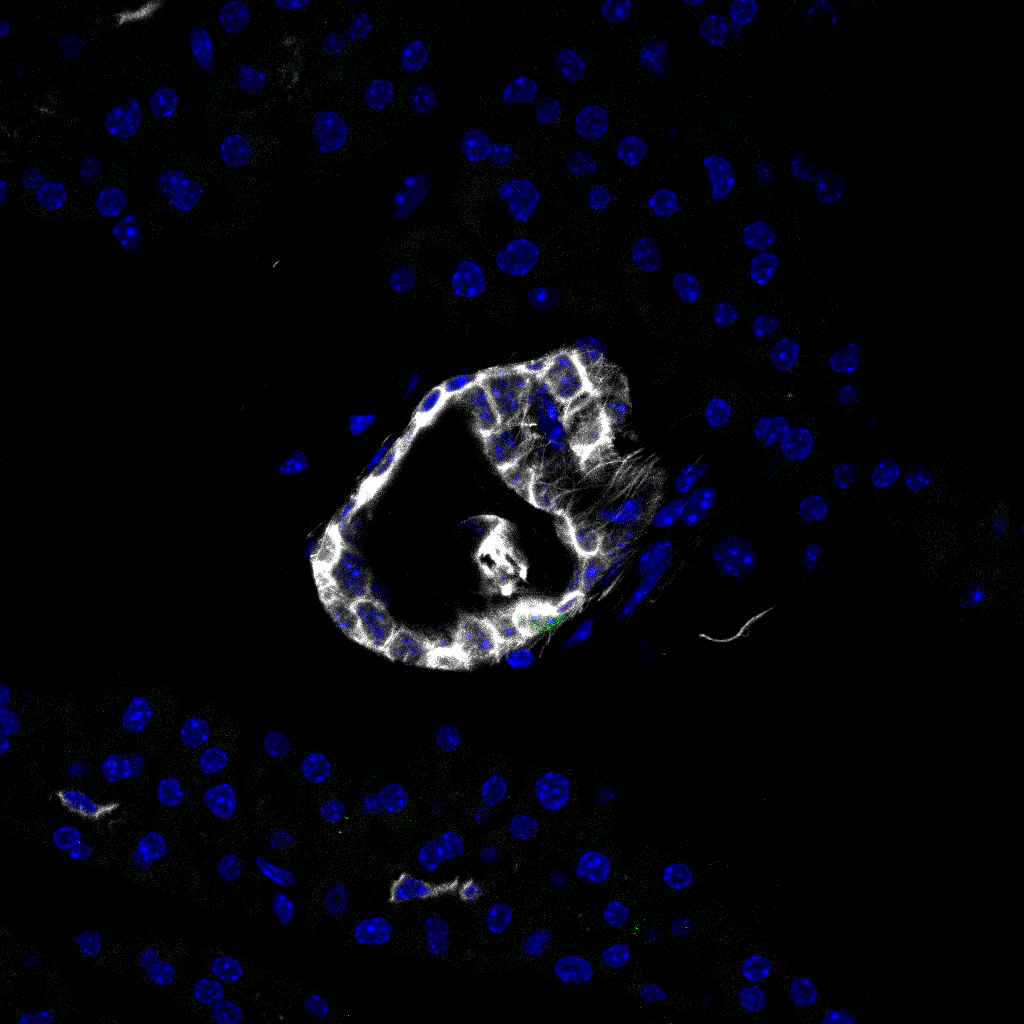

Supplement: Supplementary file 5 — Source data Fig. 3 [file 44318_2025_434_MOESM5_ESM.zip › Figure 3/3D/3D_CK19.tif]

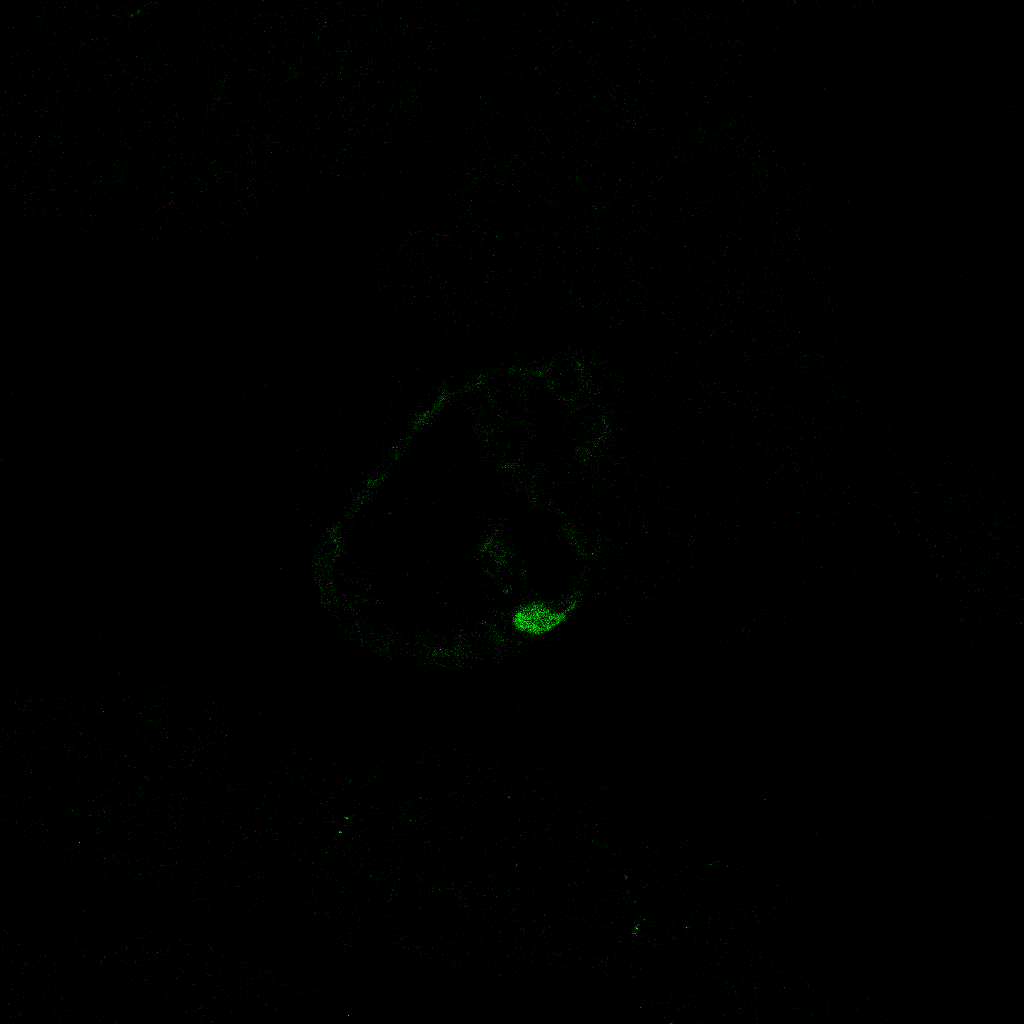

Supplement: Supplementary file 5 — Source data Fig. 3 [file 44318_2025_434_MOESM5_ESM.zip › Figure 3/3D/3D_CK19 (green).tif]

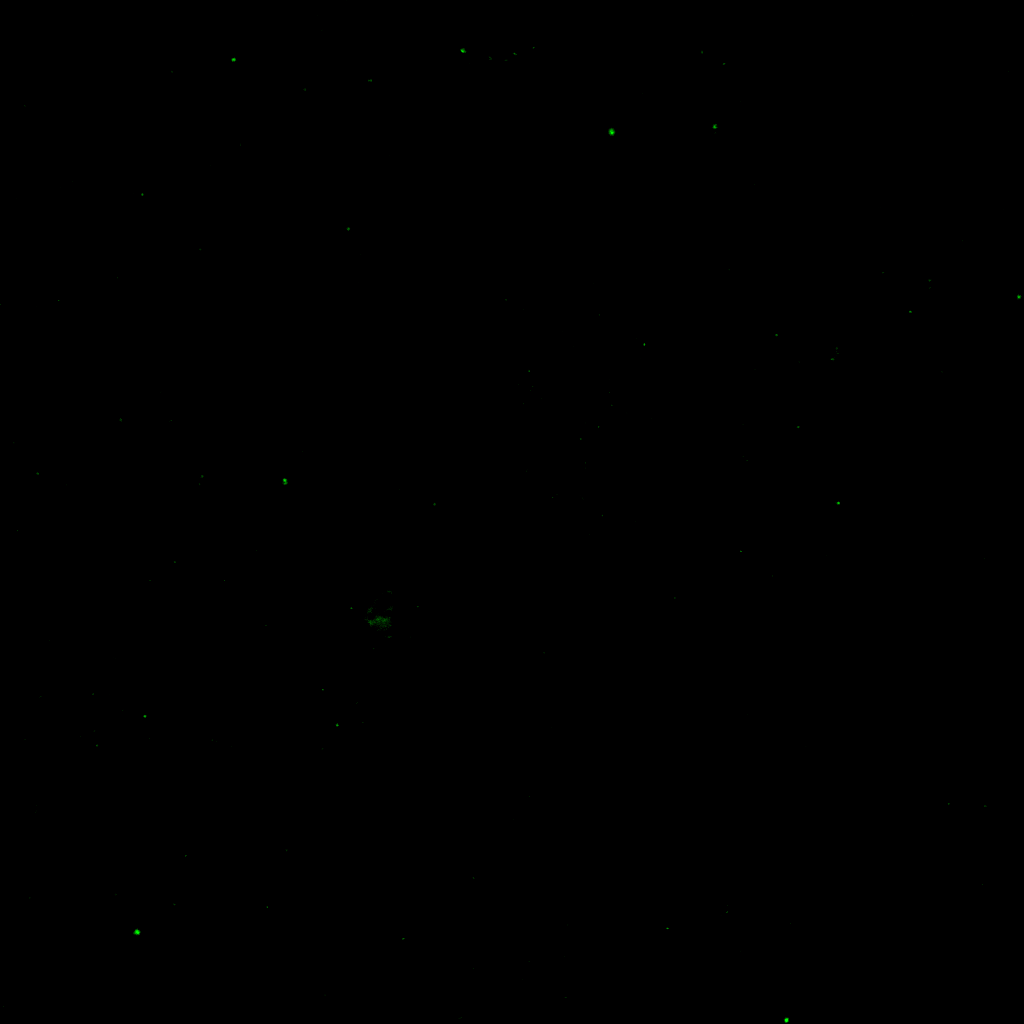

Supplement: Supplementary file 5 — Source data Fig. 3 [file 44318_2025_434_MOESM5_ESM.zip › Figure 3/3D/3D_Ins (green).tif]

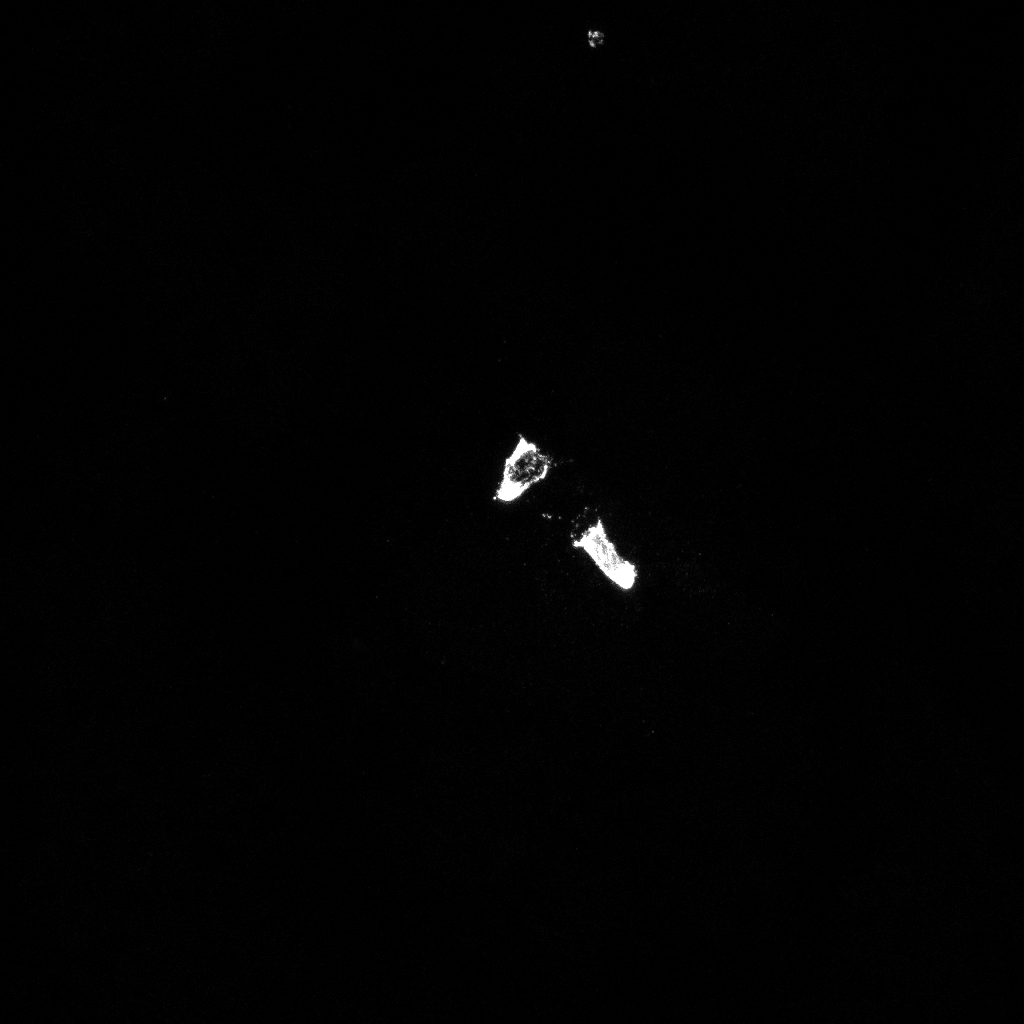

Supplement: Supplementary file 5 — Source data Fig. 3 [file 44318_2025_434_MOESM5_ESM.zip › Figure 3/3D/3D_Sst (gray).tif]

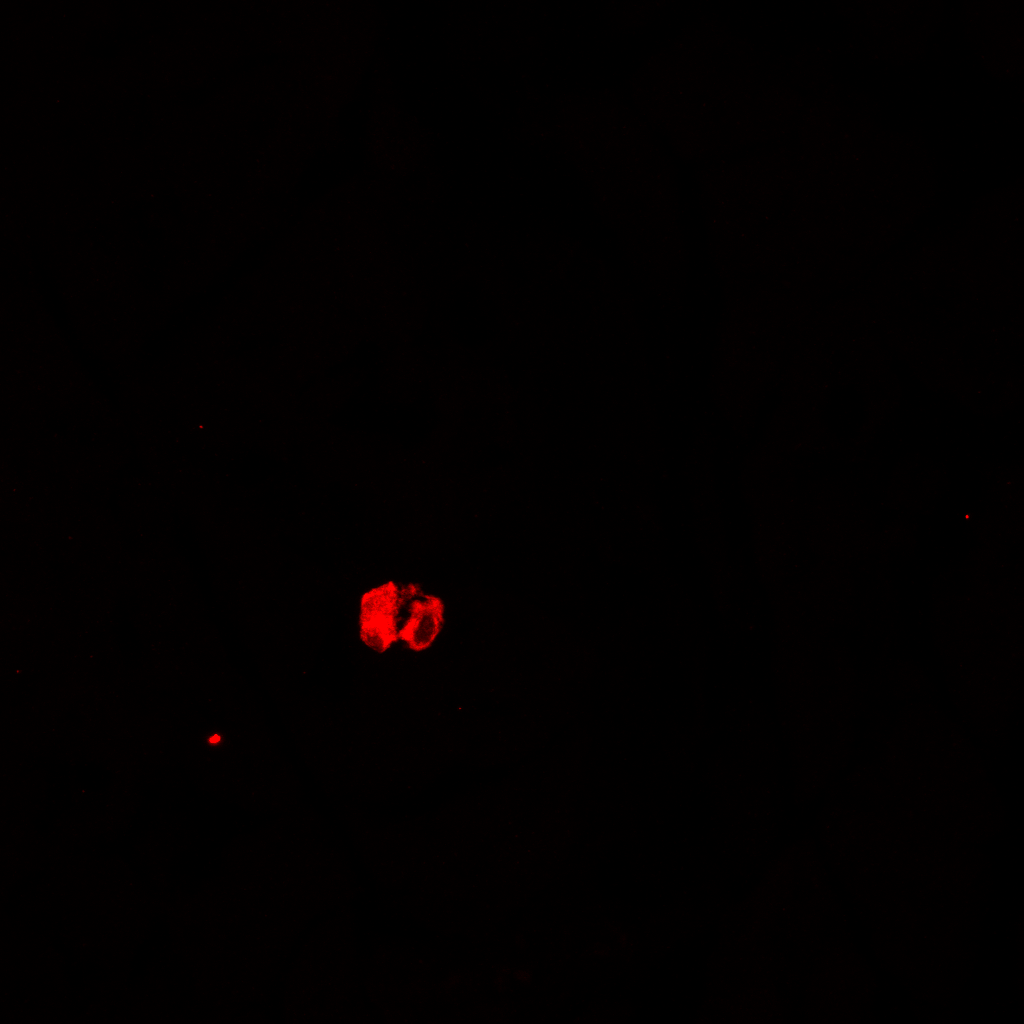

Supplement: Supplementary file 5 — Source data Fig. 3 [file 44318_2025_434_MOESM5_ESM.zip › Figure 3/3D/3D_Ins (tdT).tif]

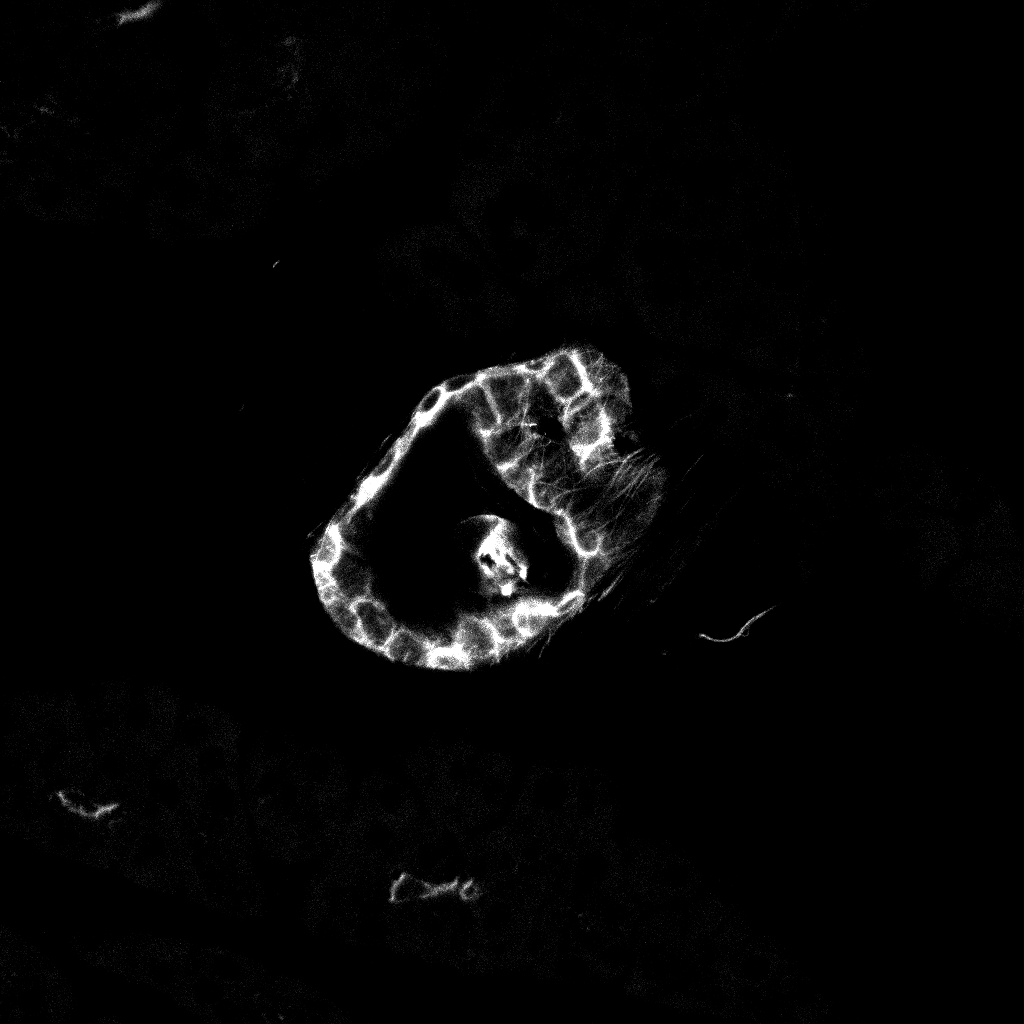

Supplement: Supplementary file 5 — Source data Fig. 3 [file 44318_2025_434_MOESM5_ESM.zip › Figure 3/3D/3D_CK19 (gray).tif]

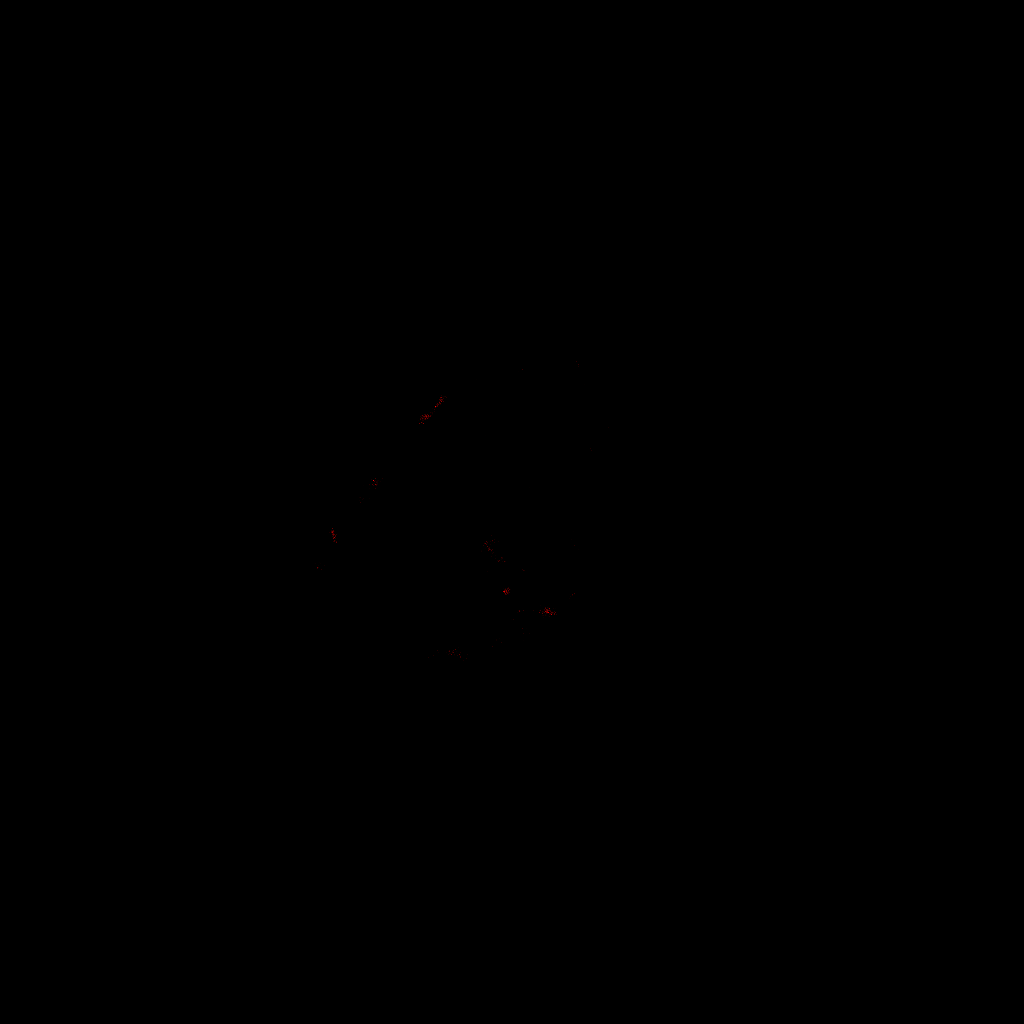

Supplement: Supplementary file 5 — Source data Fig. 3 [file 44318_2025_434_MOESM5_ESM.zip › Figure 3/3D/3D_CK19 (red).tif]

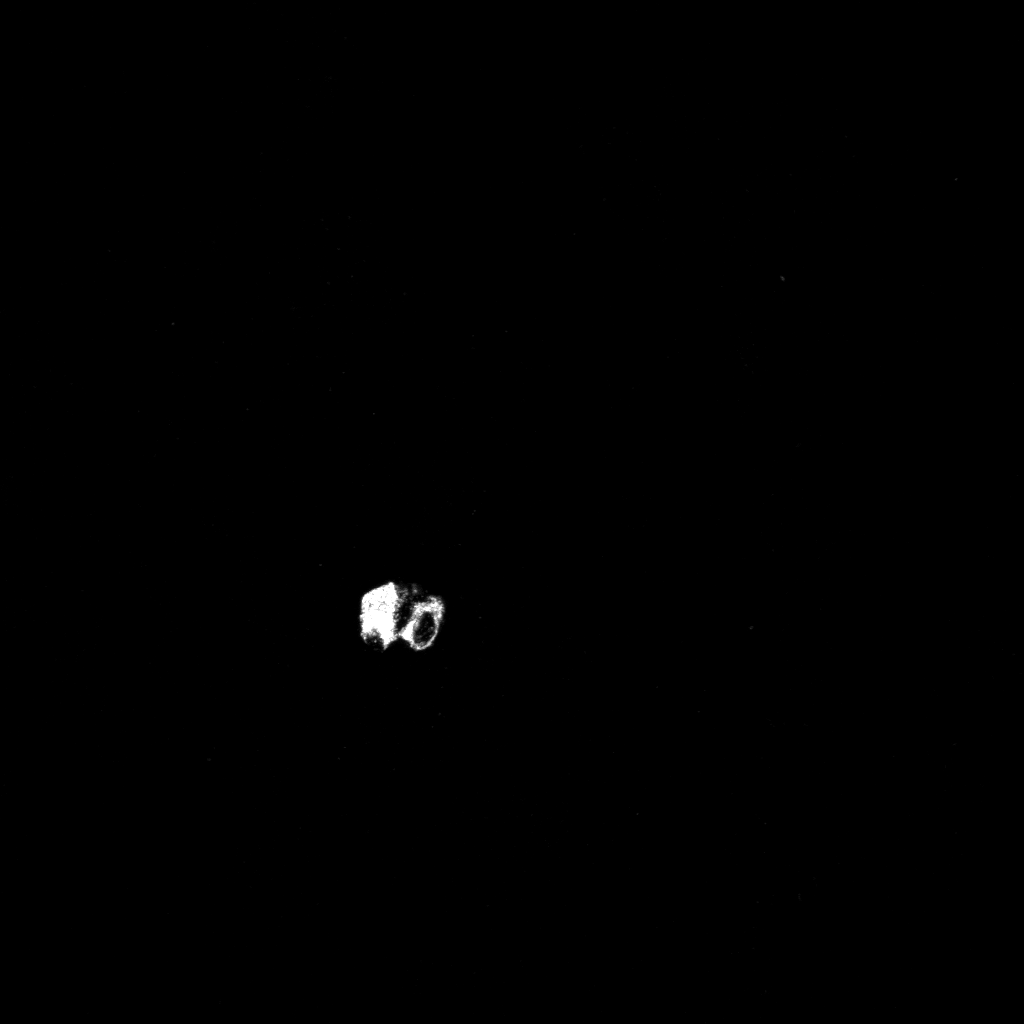

Supplement: Supplementary file 5 — Source data Fig. 3 [file 44318_2025_434_MOESM5_ESM.zip › Figure 3/3D/3D_Ins (gray).tif]

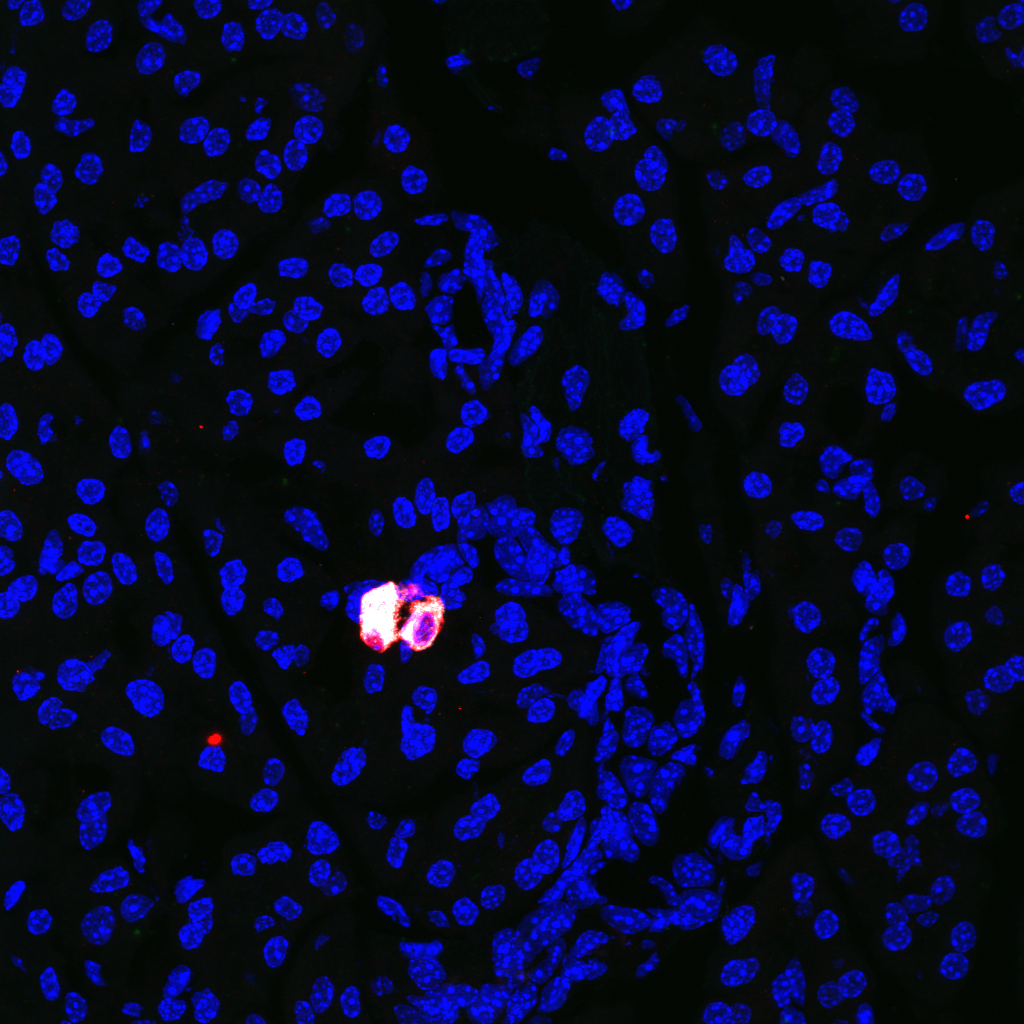

Supplement: Supplementary file 5 — Source data Fig. 3 [file 44318_2025_434_MOESM5_ESM.zip › Figure 3/3D/3D_Ins.tif]

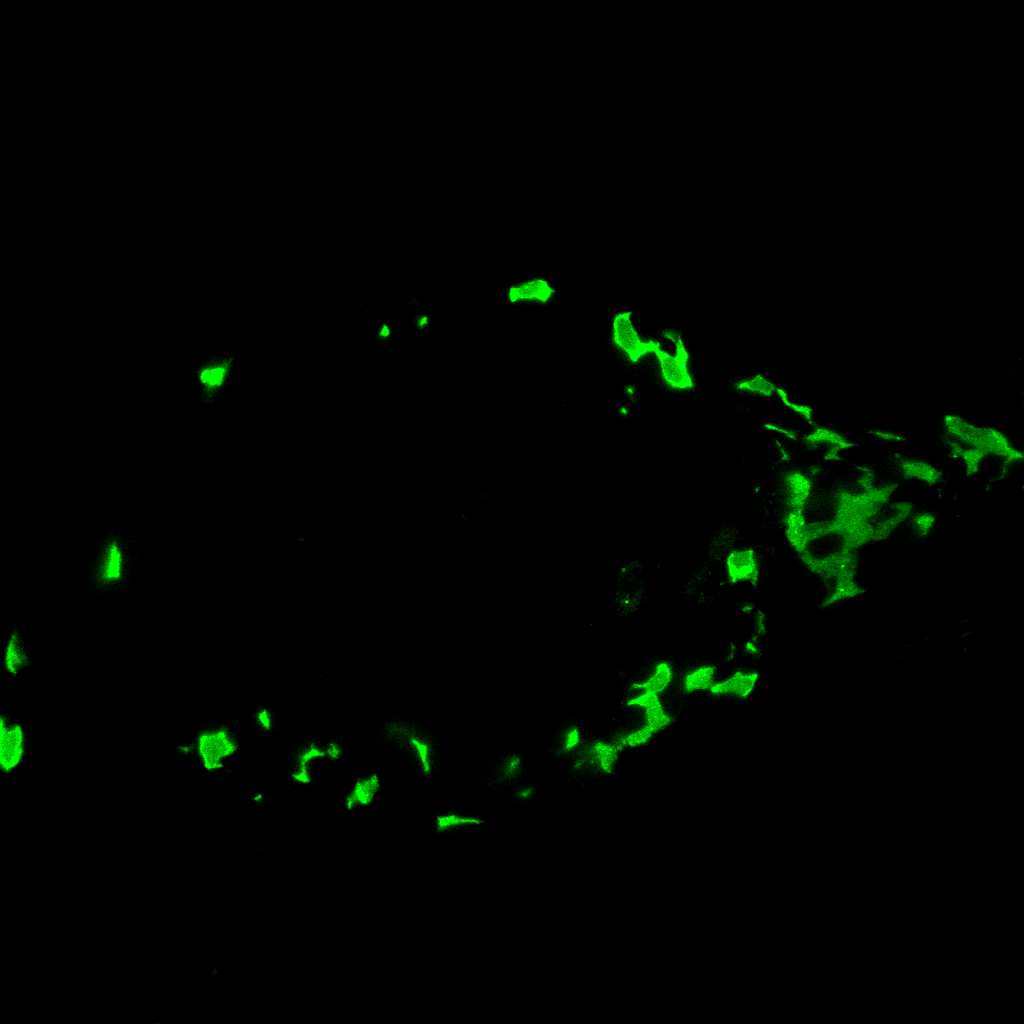

Supplement: Supplementary file 5 — Source data Fig. 3 [file 44318_2025_434_MOESM5_ESM.zip › Figure 3/3M/3M_12w_Ins (green).tif]

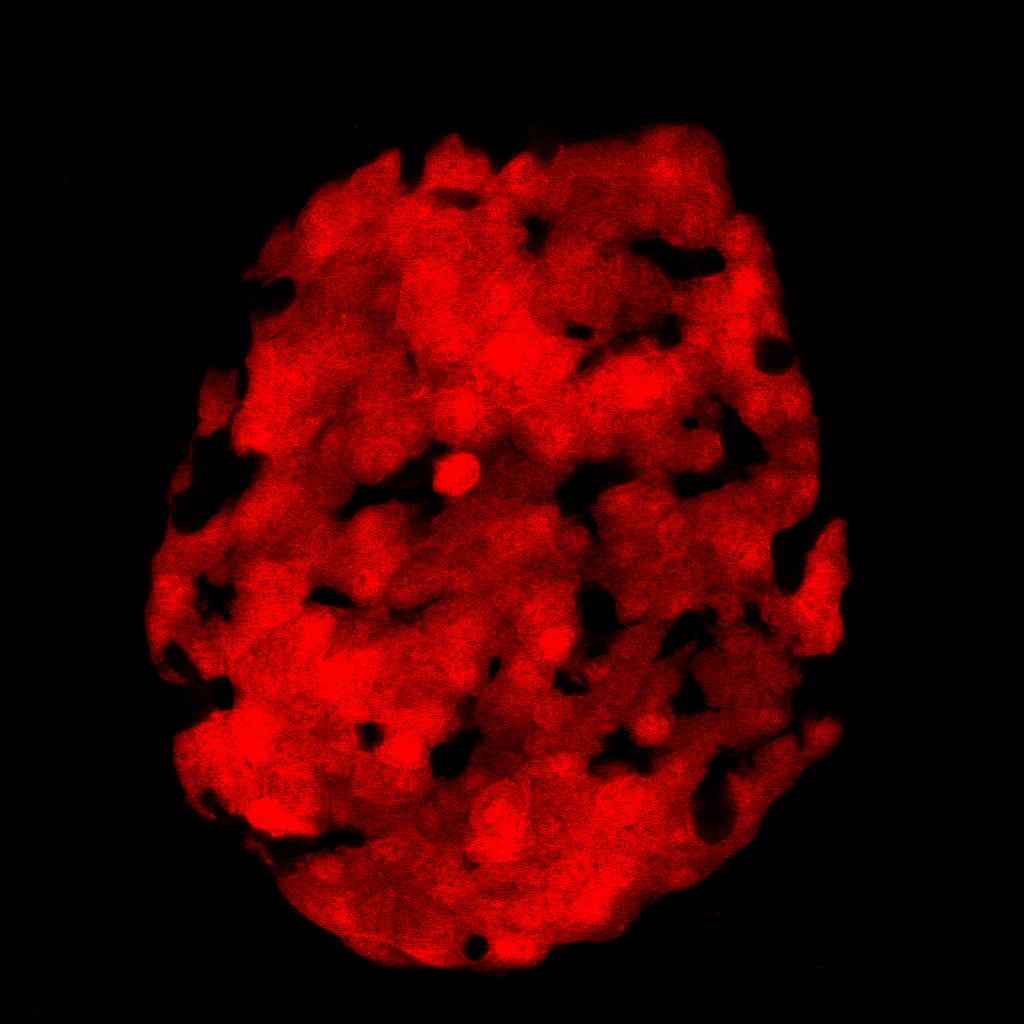

Supplement: Supplementary file 5 — Source data Fig. 3 [file 44318_2025_434_MOESM5_ESM.zip › Figure 3/3M/3M_2w_Ins (red).tif]

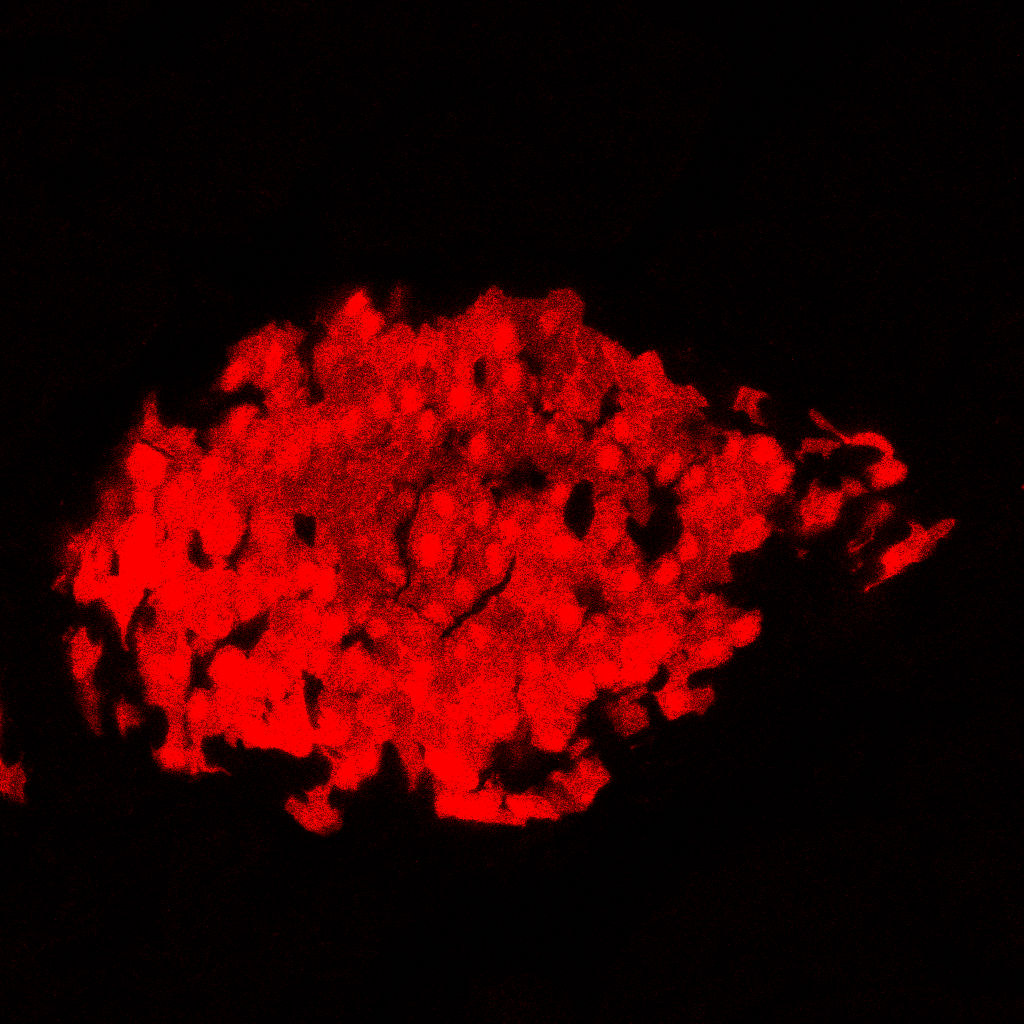

Supplement: Supplementary file 5 — Source data Fig. 3 [file 44318_2025_434_MOESM5_ESM.zip › Figure 3/3M/3M_12w_Ins (red).tif]

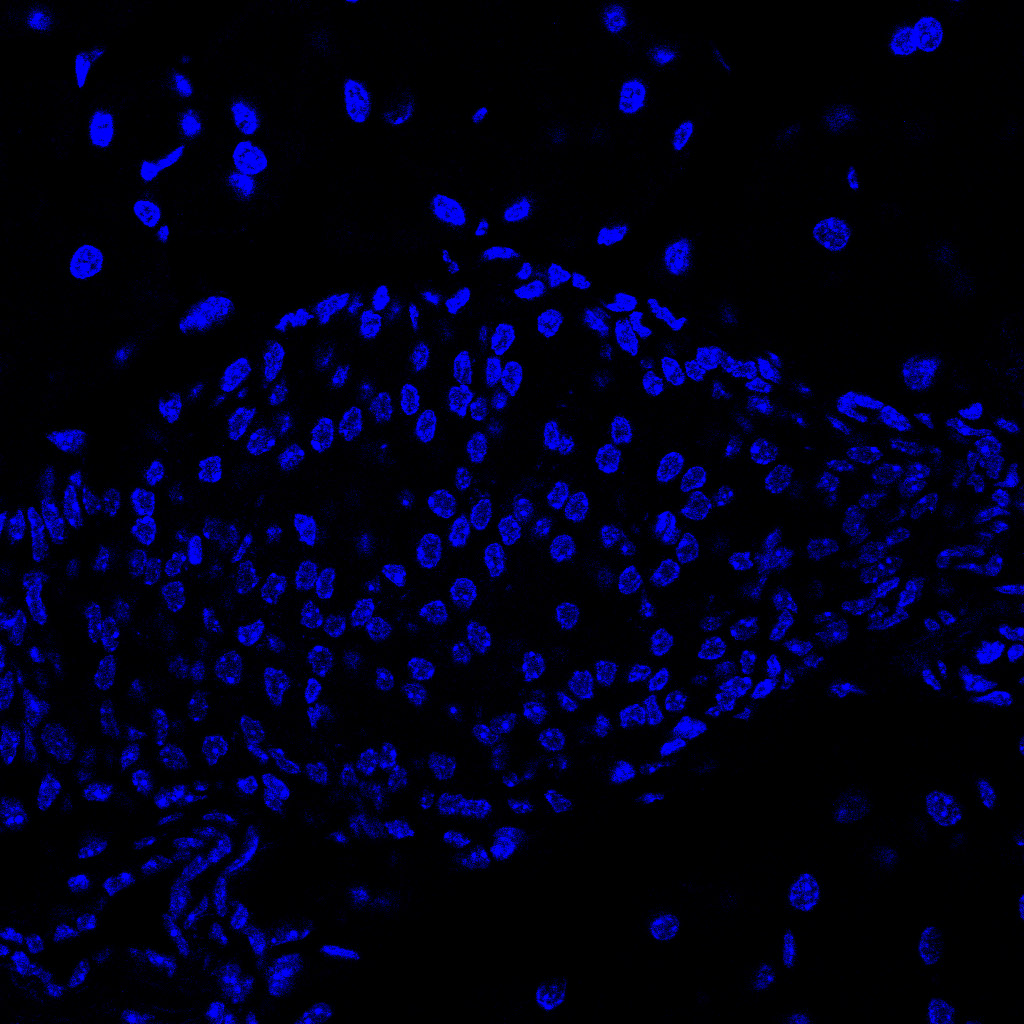

Supplement: Supplementary file 5 — Source data Fig. 3 [file 44318_2025_434_MOESM5_ESM.zip › Figure 3/3M/3M_12w_Ins (blue).tif]

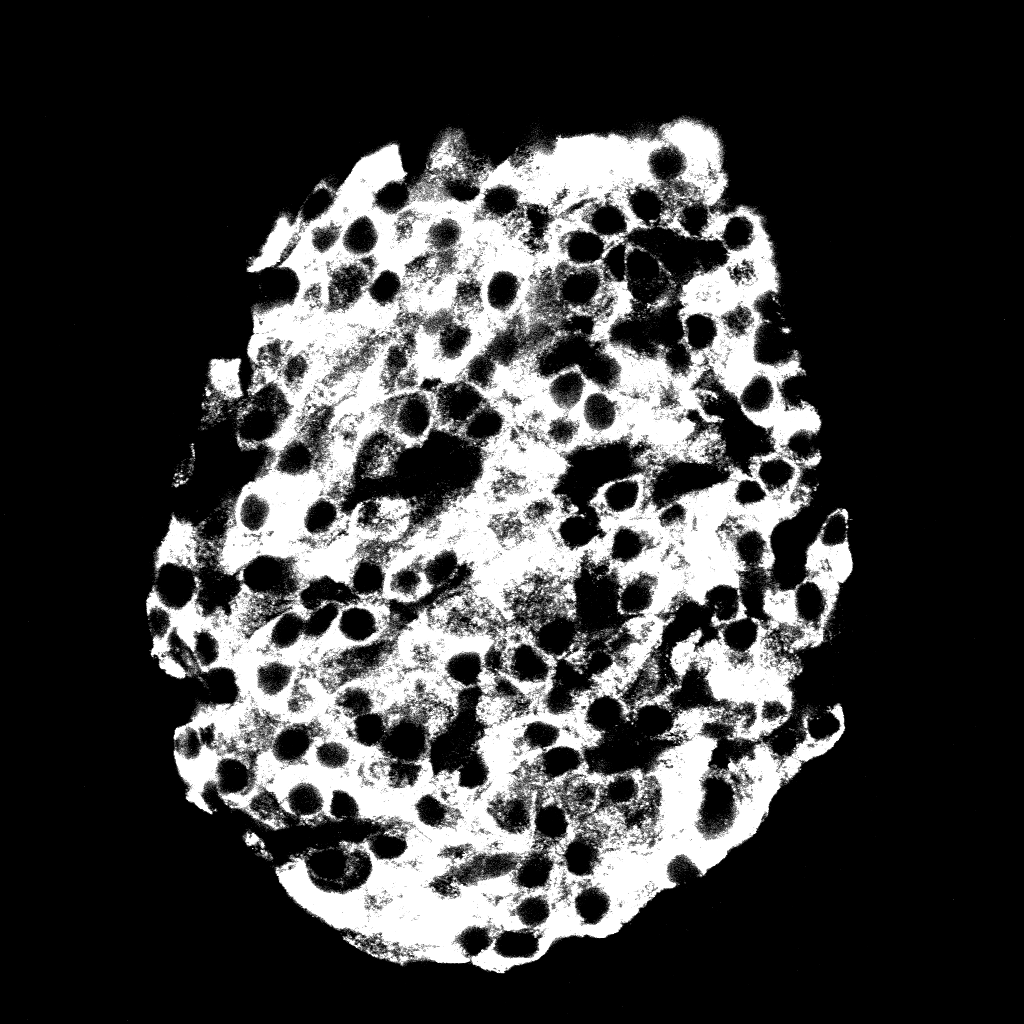

Supplement: Supplementary file 5 — Source data Fig. 3 [file 44318_2025_434_MOESM5_ESM.zip › Figure 3/3M/3M_2w_Ins (gray).tif]

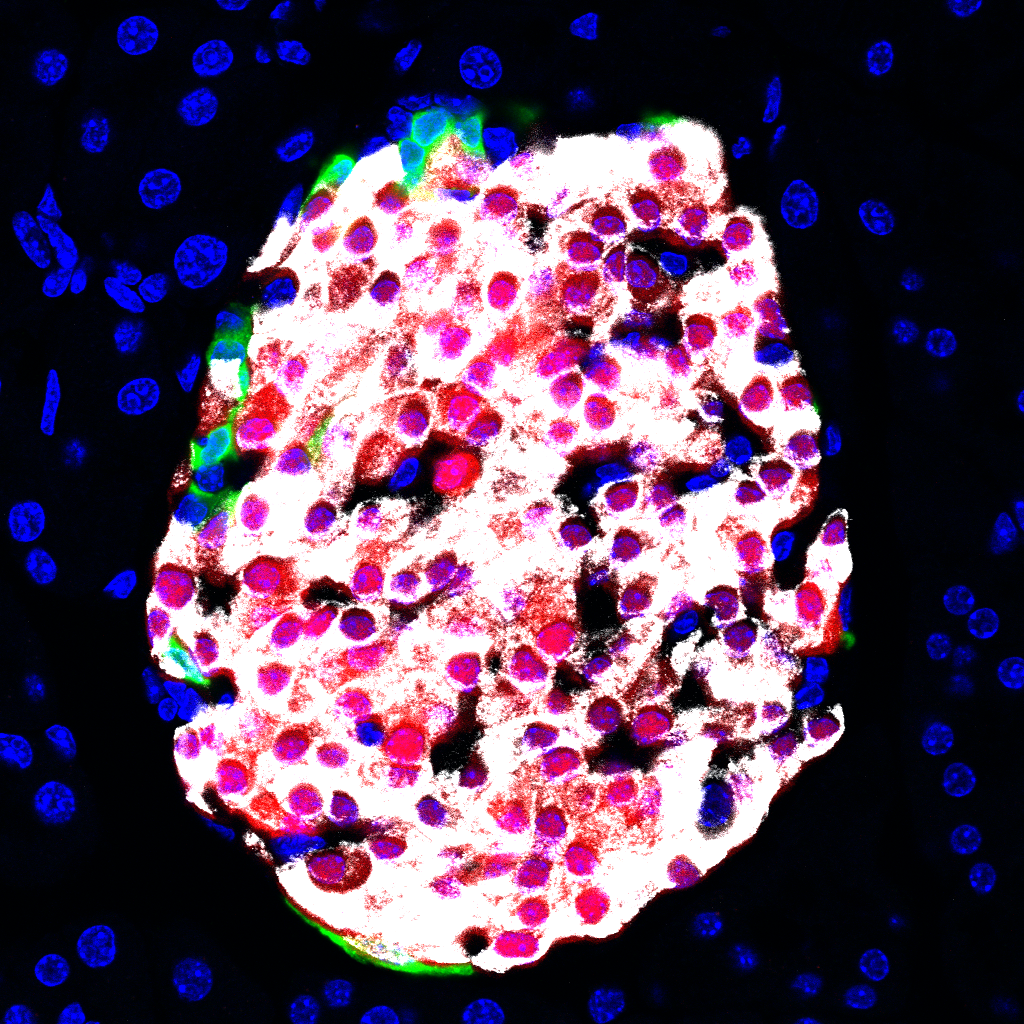

Supplement: Supplementary file 5 — Source data Fig. 3 [file 44318_2025_434_MOESM5_ESM.zip › Figure 3/3M/3M_2w_Ins.tif]

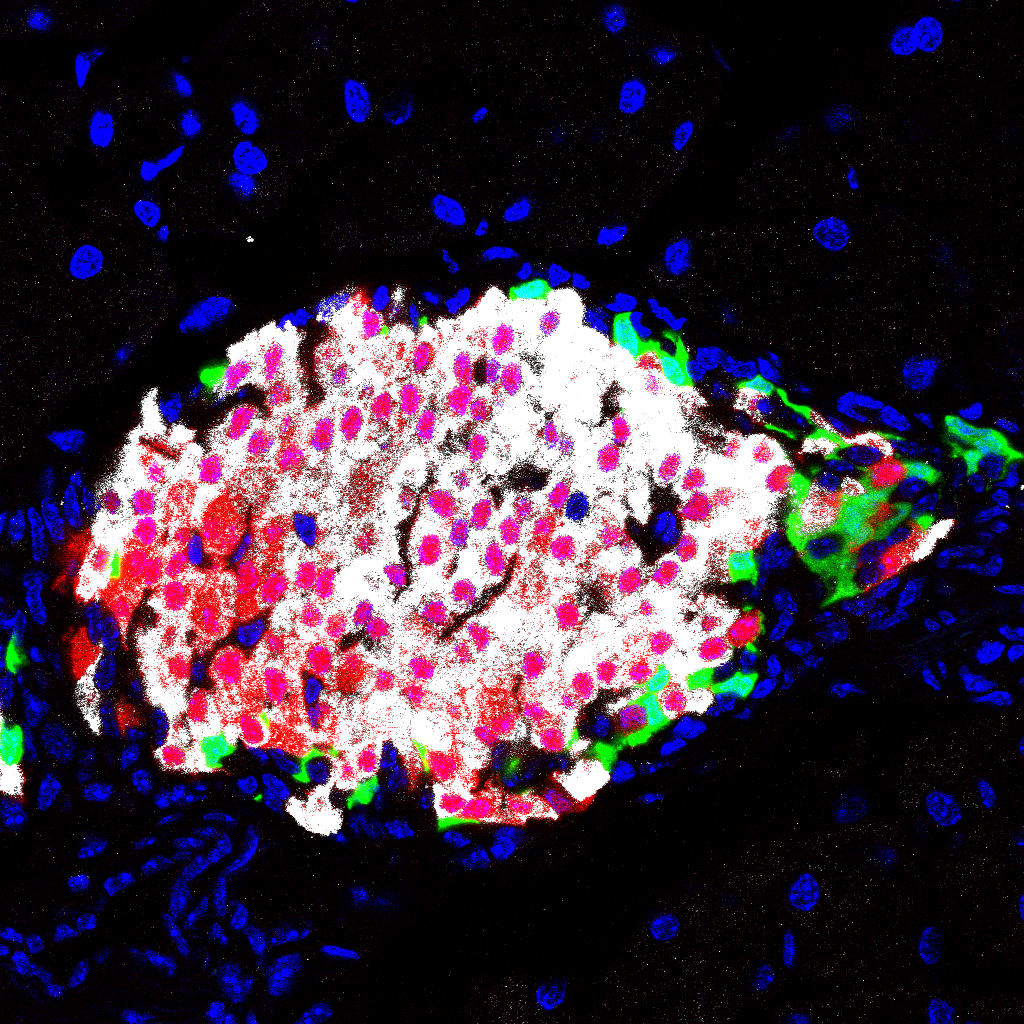

Supplement: Supplementary file 5 — Source data Fig. 3 [file 44318_2025_434_MOESM5_ESM.zip › Figure 3/3M/3M_12w_Ins.tif]
