# Supplementary figures and images for: Ductal or Ngn3+ cells do not contribute to adult pancreatic islet beta-cell neogenesis in homeostasis (part 3 of 5)
Source: EMBO J. 2025 Apr 9;44(10):2856–81. doi: 10.1038/s44318-025-00434-z (PMC12084597; doi:10.1038/s44318-025-00434-z)

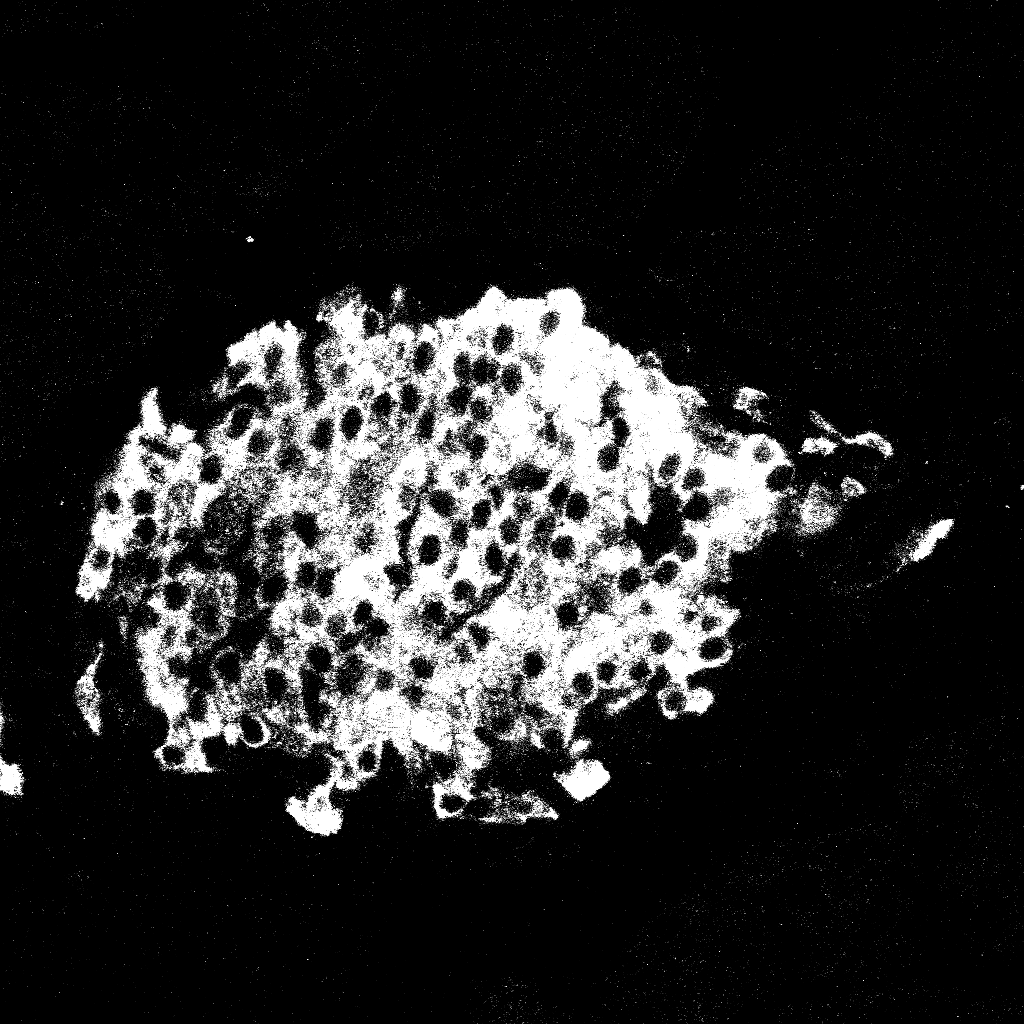

Supplement: Supplementary file 5 — Source data Fig. 3 [file 44318_2025_434_MOESM5_ESM.zip › Figure 3/3M/3M_12w_Ins (gray).tif]

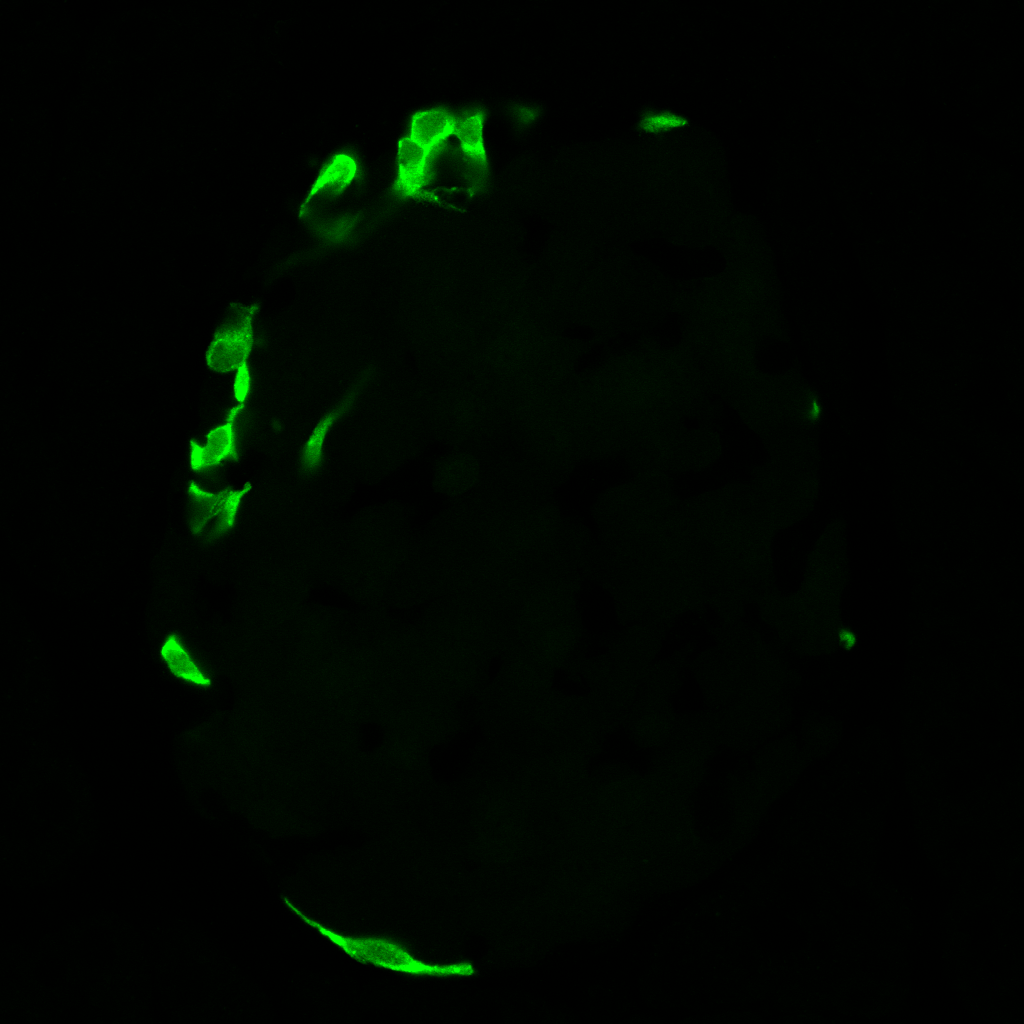

Supplement: Supplementary file 5 — Source data Fig. 3 [file 44318_2025_434_MOESM5_ESM.zip › Figure 3/3M/3M_2w_Ins (green).tif]

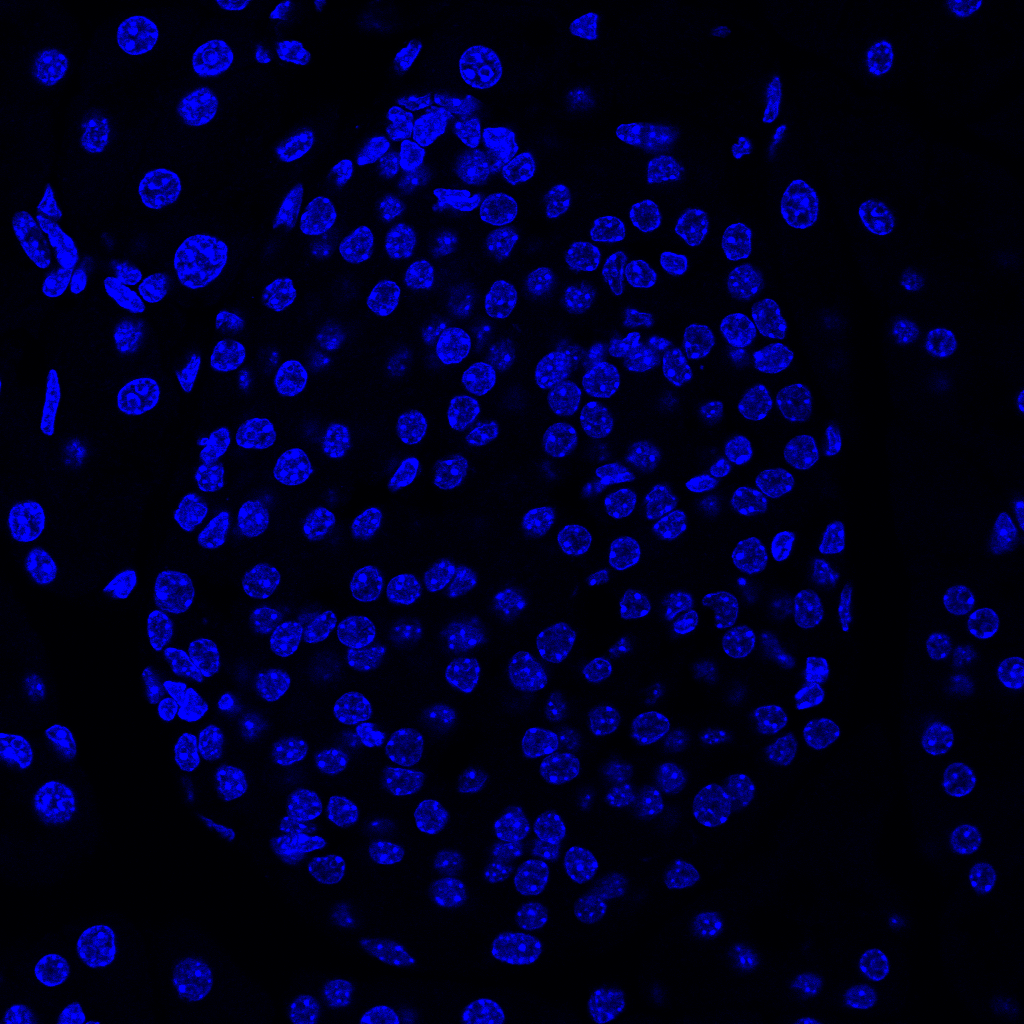

Supplement: Supplementary file 5 — Source data Fig. 3 [file 44318_2025_434_MOESM5_ESM.zip › Figure 3/3M/3M_2w_Ins (blue).tif]

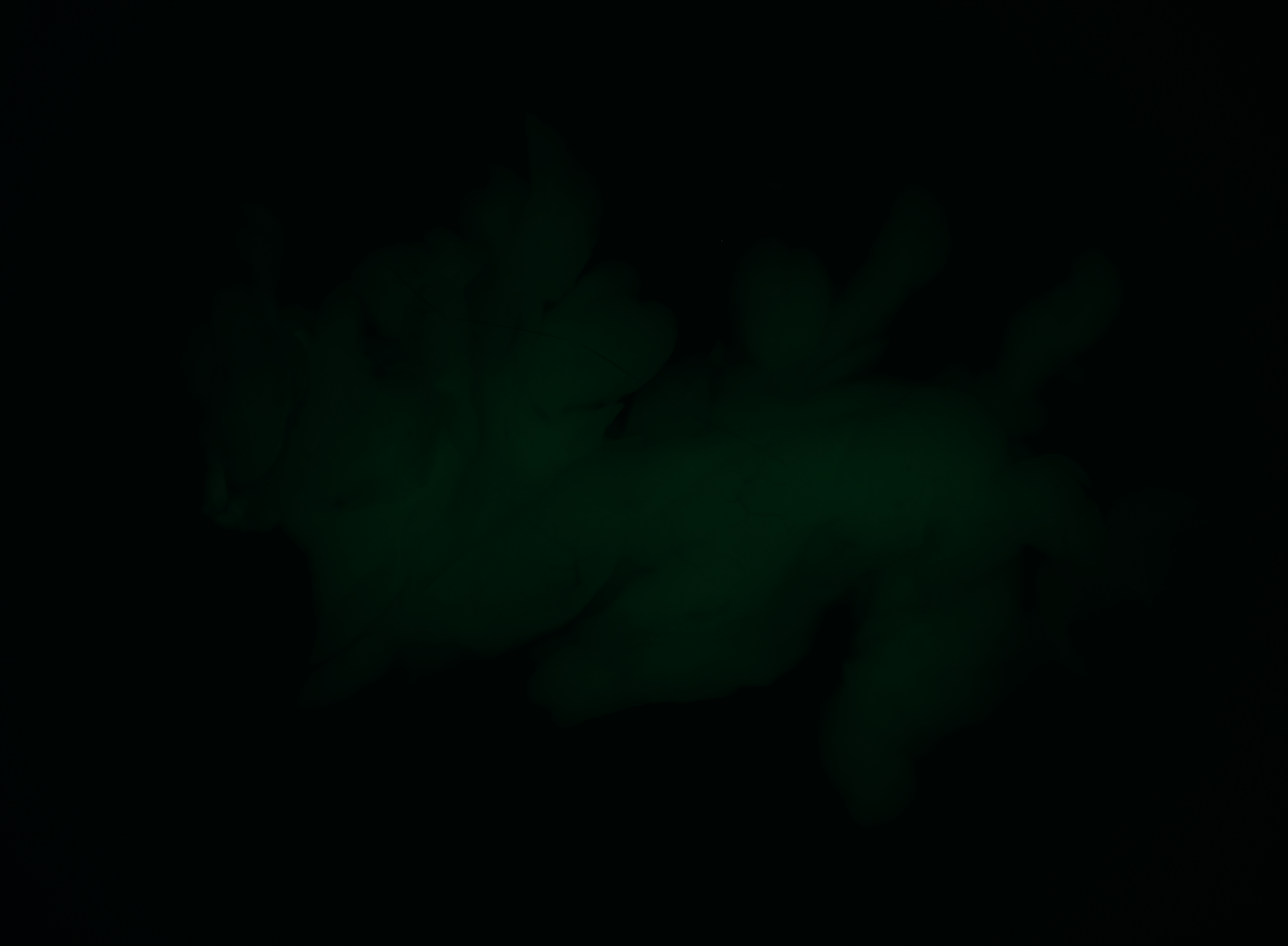

Supplement: Supplementary file 5 — Source data Fig. 3 [file 44318_2025_434_MOESM5_ESM.zip › Figure 3/3J/3J_2w_zsGreen.tif]

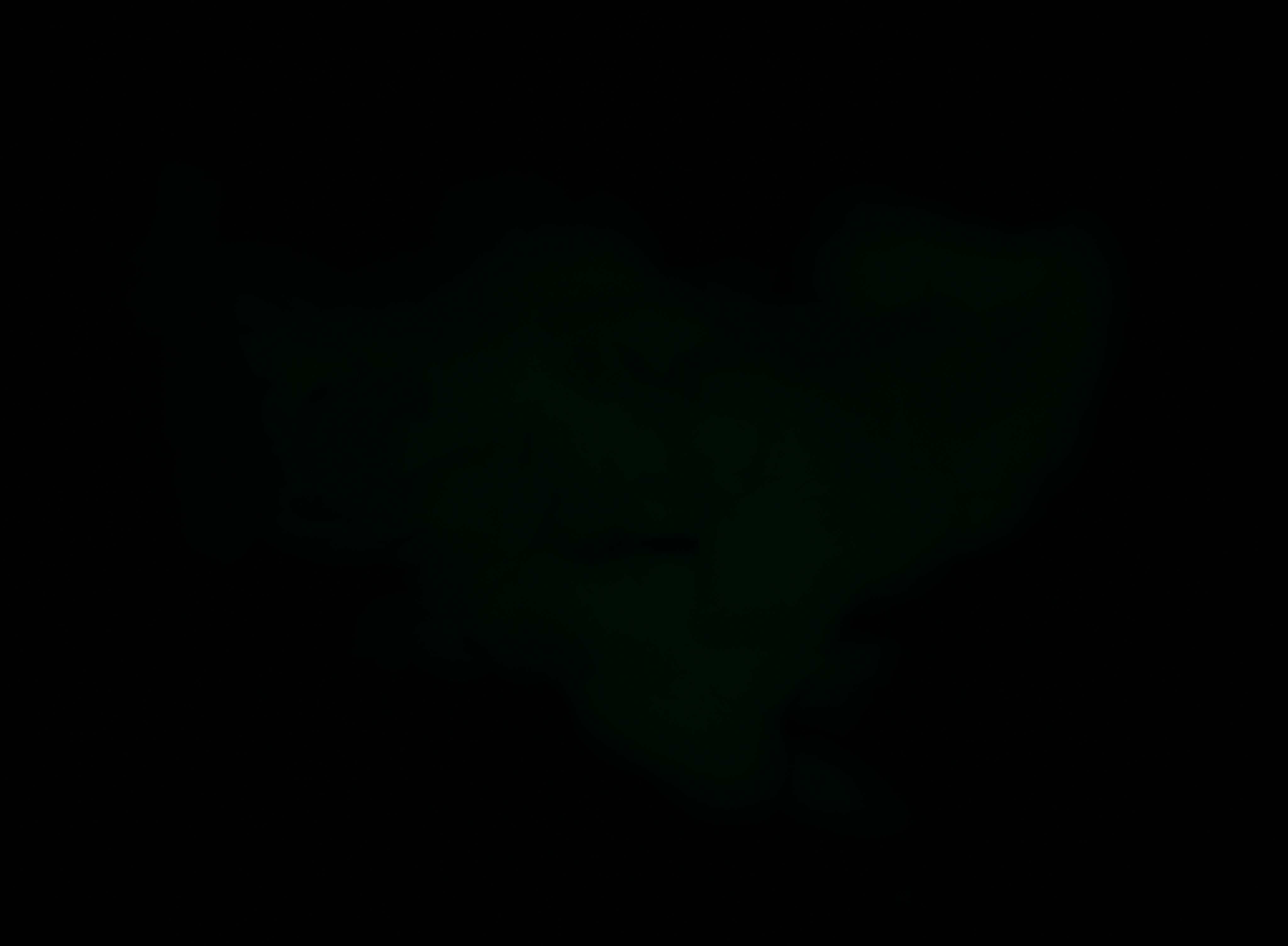

Supplement: Supplementary file 5 — Source data Fig. 3 [file 44318_2025_434_MOESM5_ESM.zip › Figure 3/3J/3J_12w_zsGreen.tif]

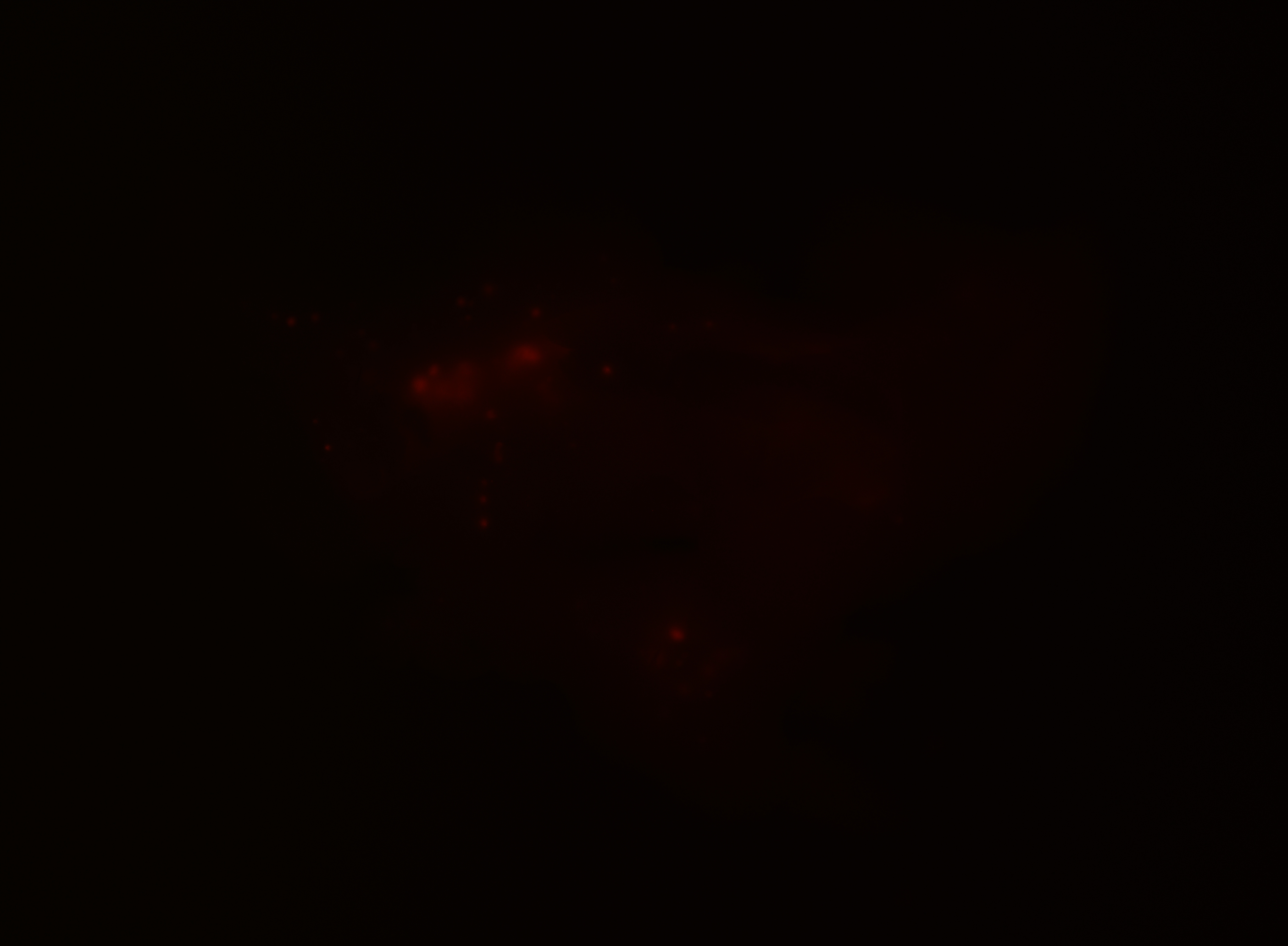

Supplement: Supplementary file 5 — Source data Fig. 3 [file 44318_2025_434_MOESM5_ESM.zip › Figure 3/3J/3J_12w_tdT.tif]

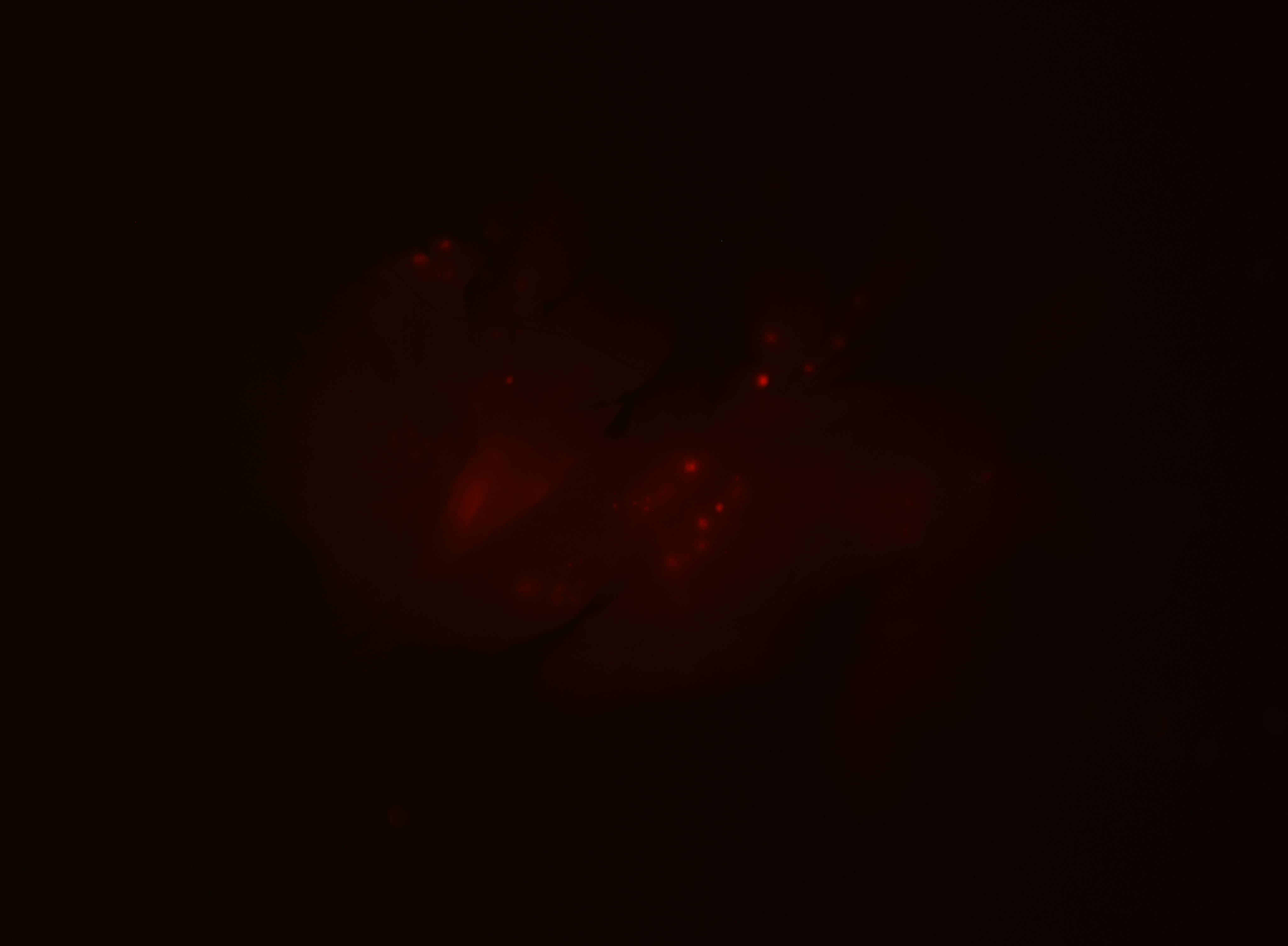

Supplement: Supplementary file 5 — Source data Fig. 3 [file 44318_2025_434_MOESM5_ESM.zip › Figure 3/3J/3J_2w_tdT.tif]

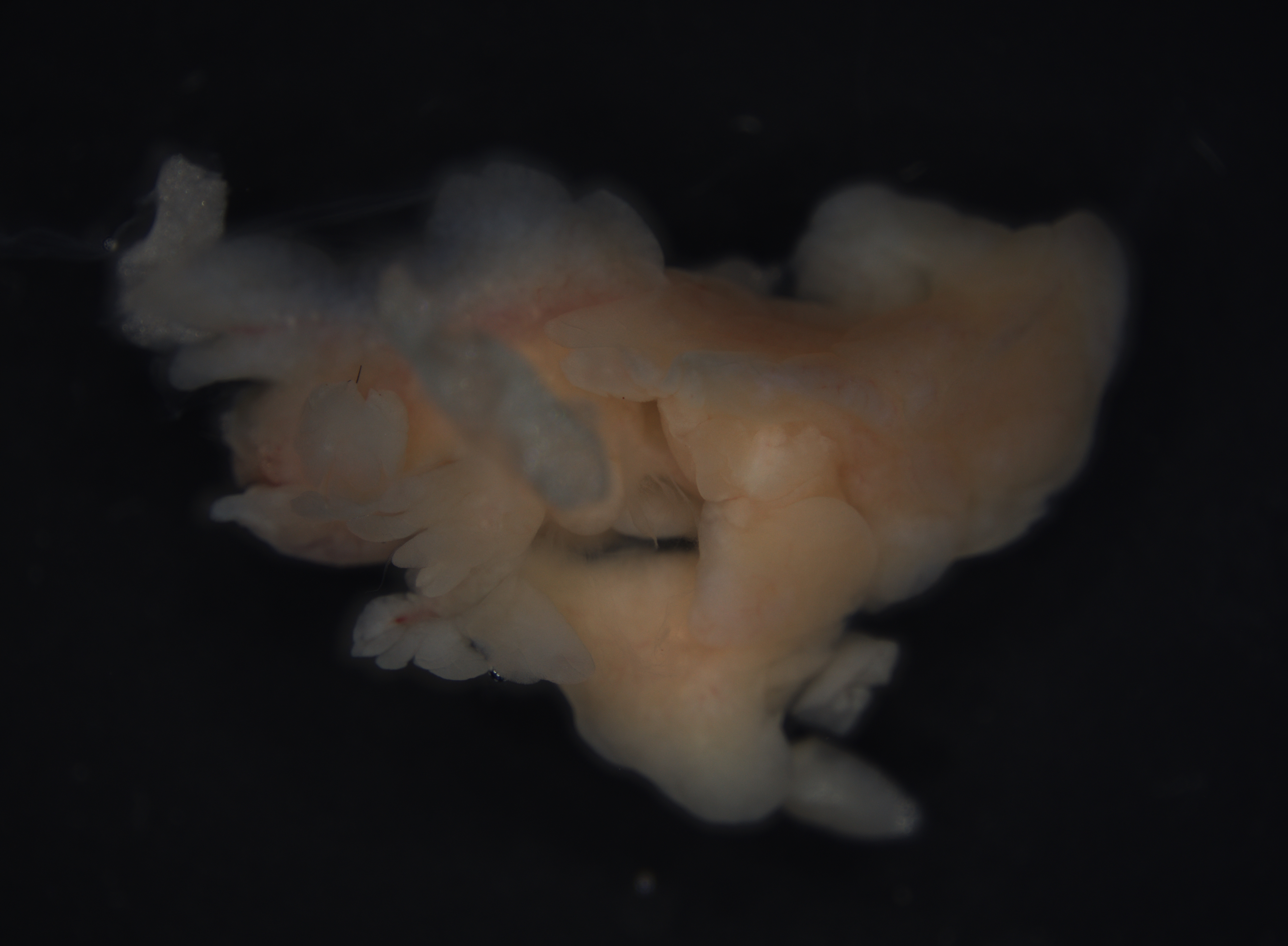

Supplement: Supplementary file 5 — Source data Fig. 3 [file 44318_2025_434_MOESM5_ESM.zip › Figure 3/3J/3J_12w_BF.tif]

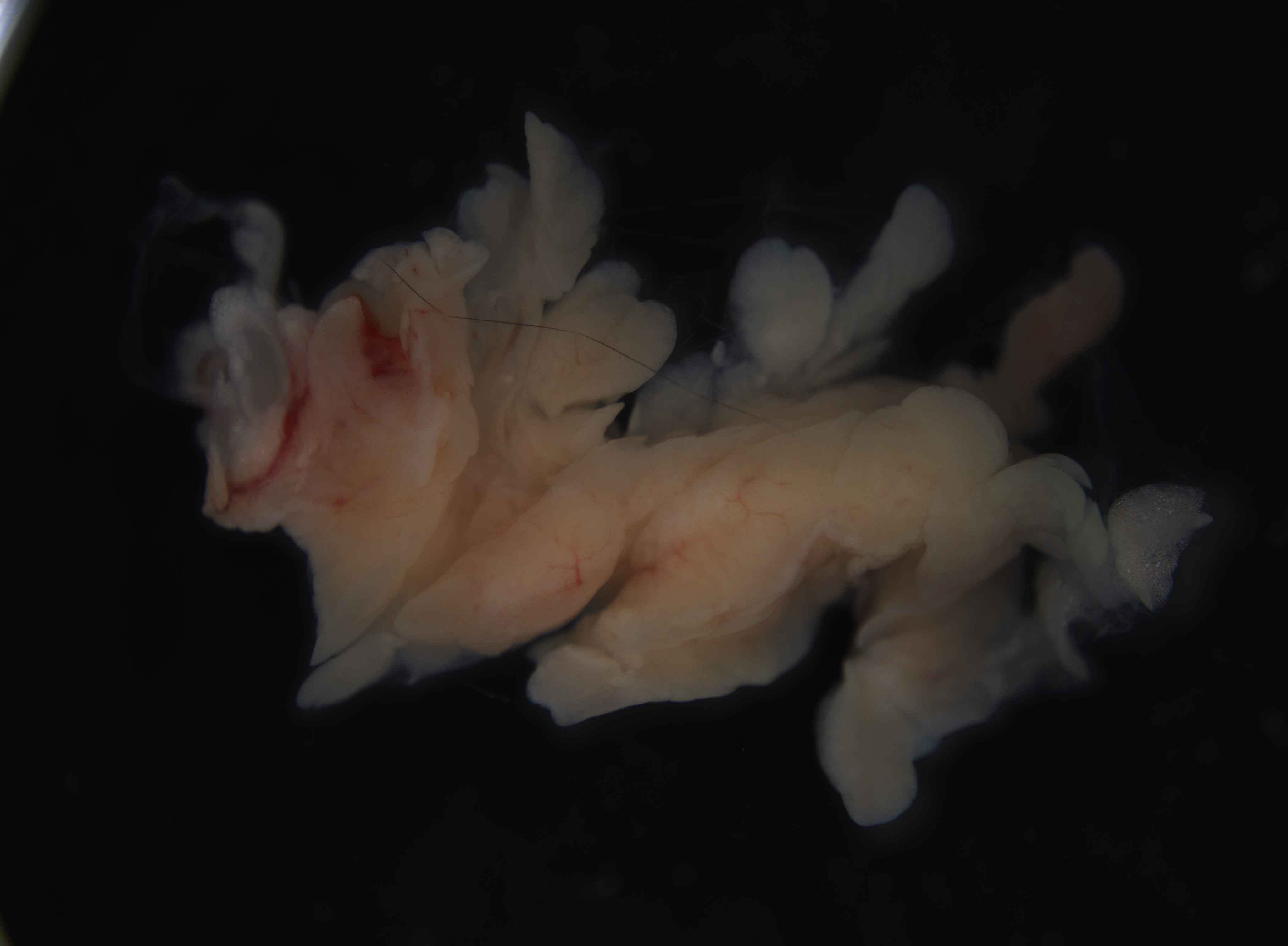

Supplement: Supplementary file 5 — Source data Fig. 3 [file 44318_2025_434_MOESM5_ESM.zip › Figure 3/3J/3J_2w_BF.tif]

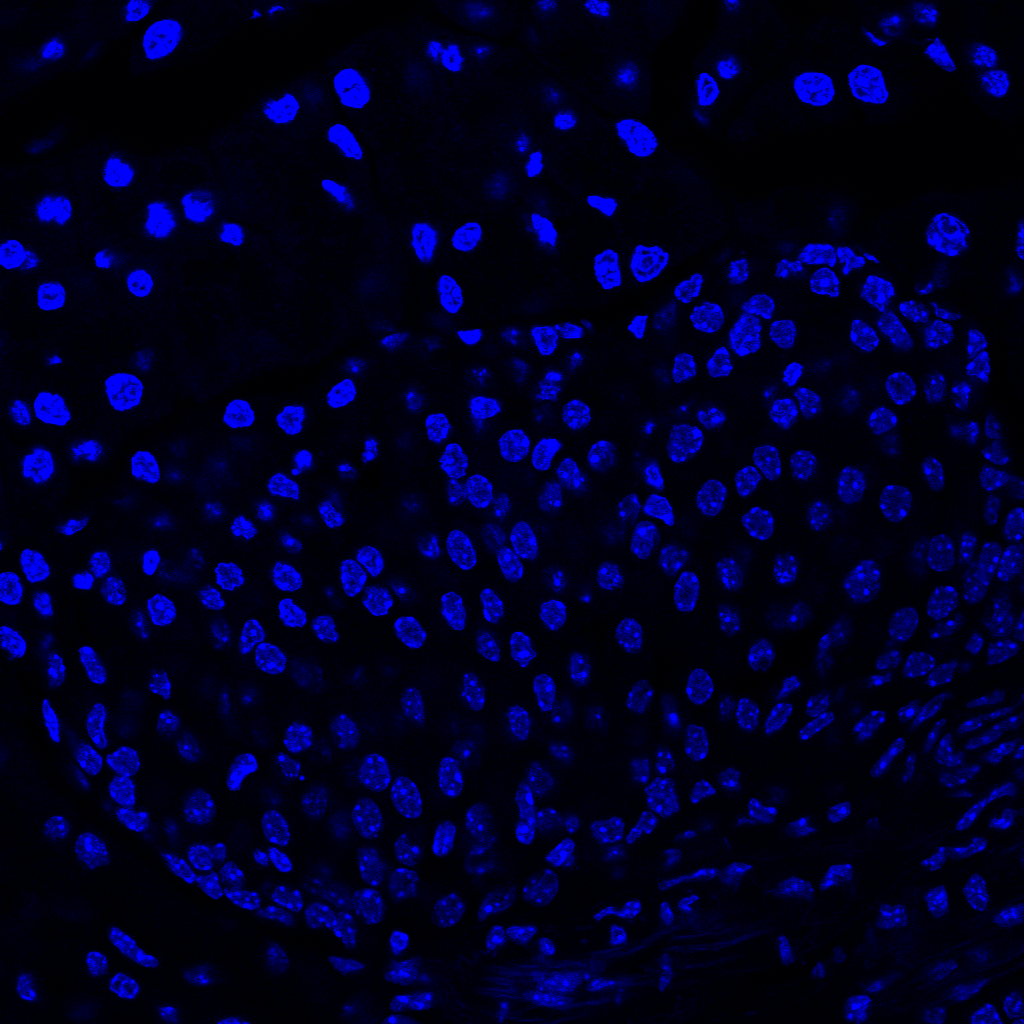

Supplement: Supplementary file 5 — Source data Fig. 3 [file 44318_2025_434_MOESM5_ESM.zip › Figure 3/3F/3F_Merge (blue).tif]

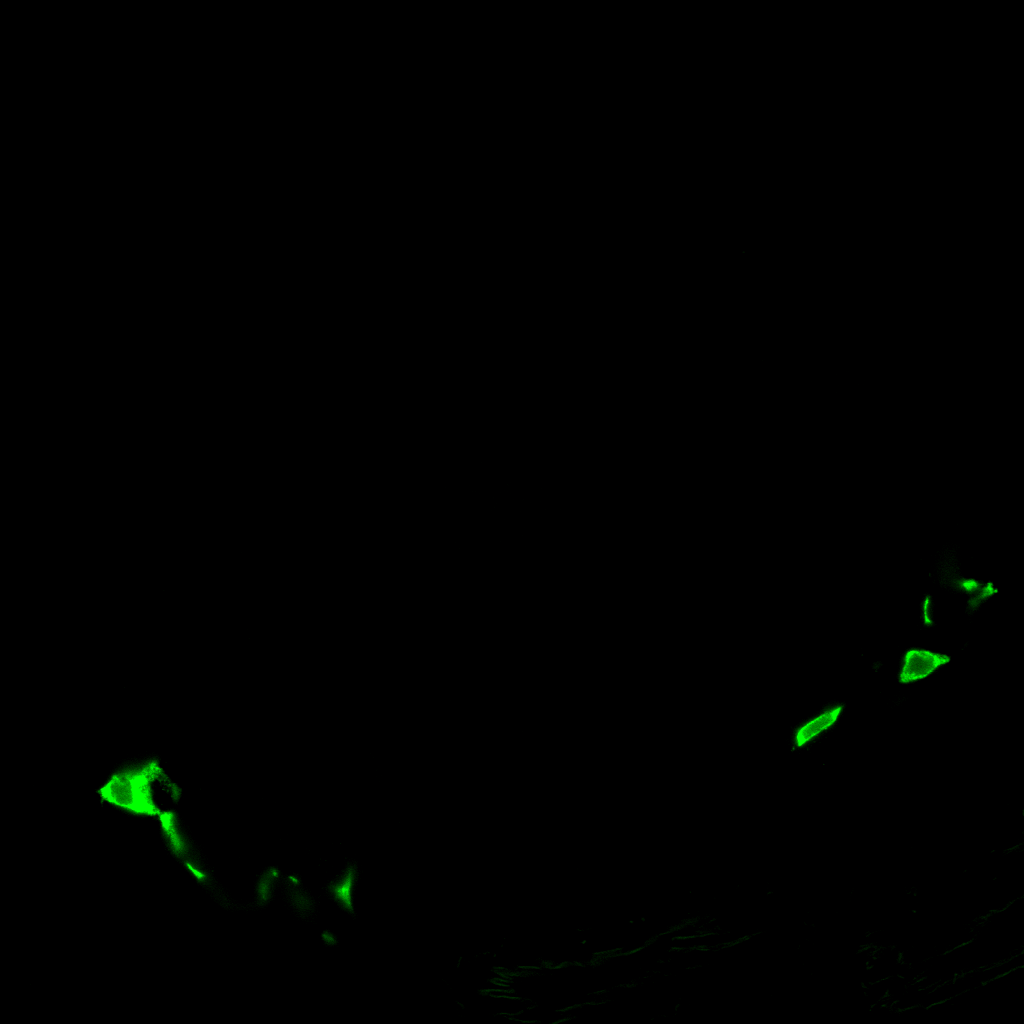

Supplement: Supplementary file 5 — Source data Fig. 3 [file 44318_2025_434_MOESM5_ESM.zip › Figure 3/3F/3F_Merge (green).tif]

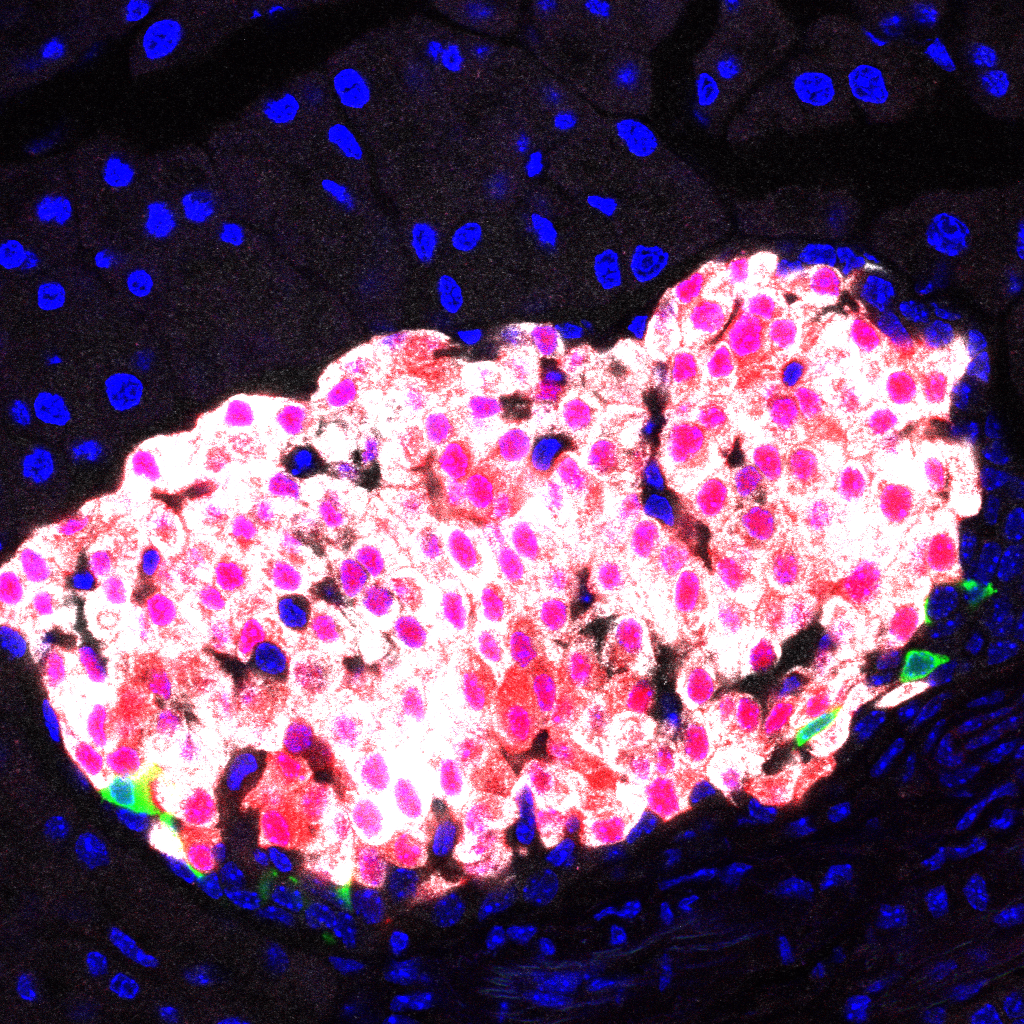

Supplement: Supplementary file 5 — Source data Fig. 3 [file 44318_2025_434_MOESM5_ESM.zip › Figure 3/3F/3F_Merge.tif]

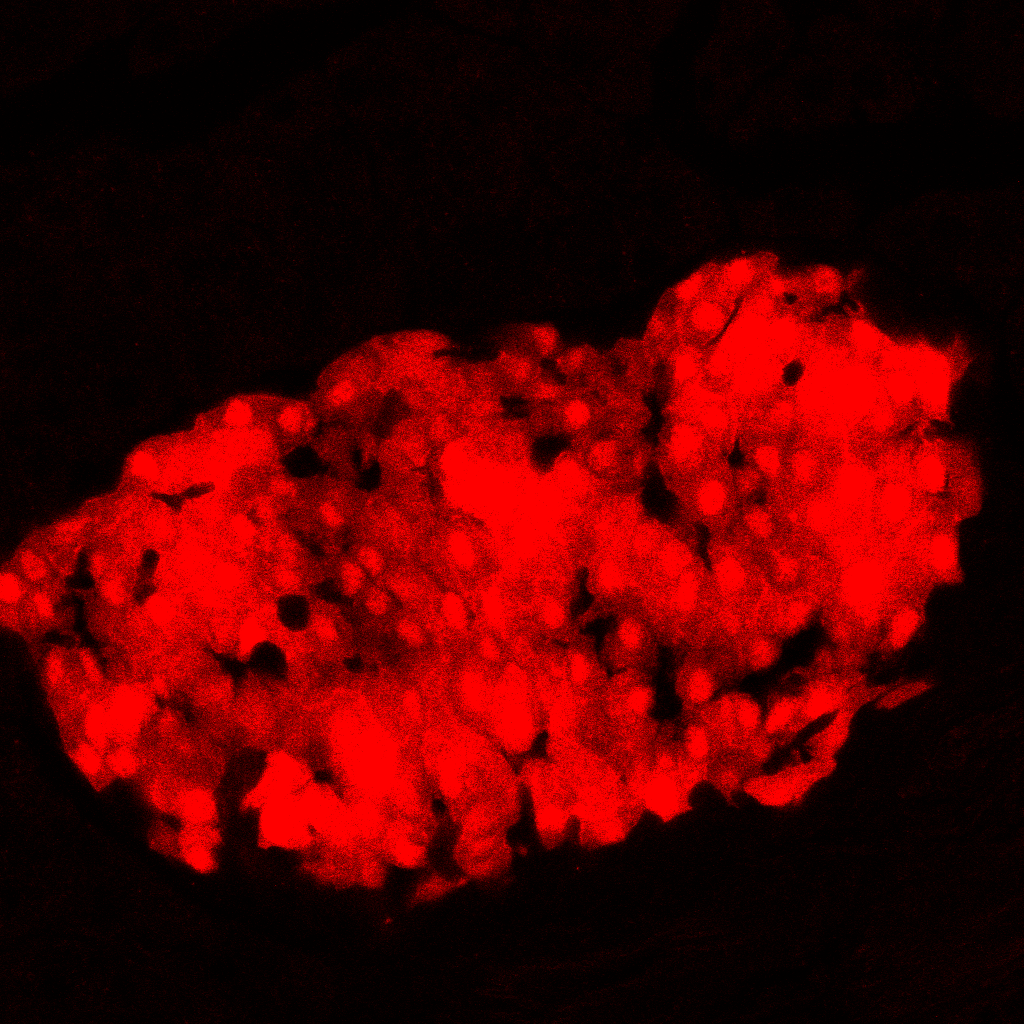

Supplement: Supplementary file 5 — Source data Fig. 3 [file 44318_2025_434_MOESM5_ESM.zip › Figure 3/3F/3F_Merge (red).tif]

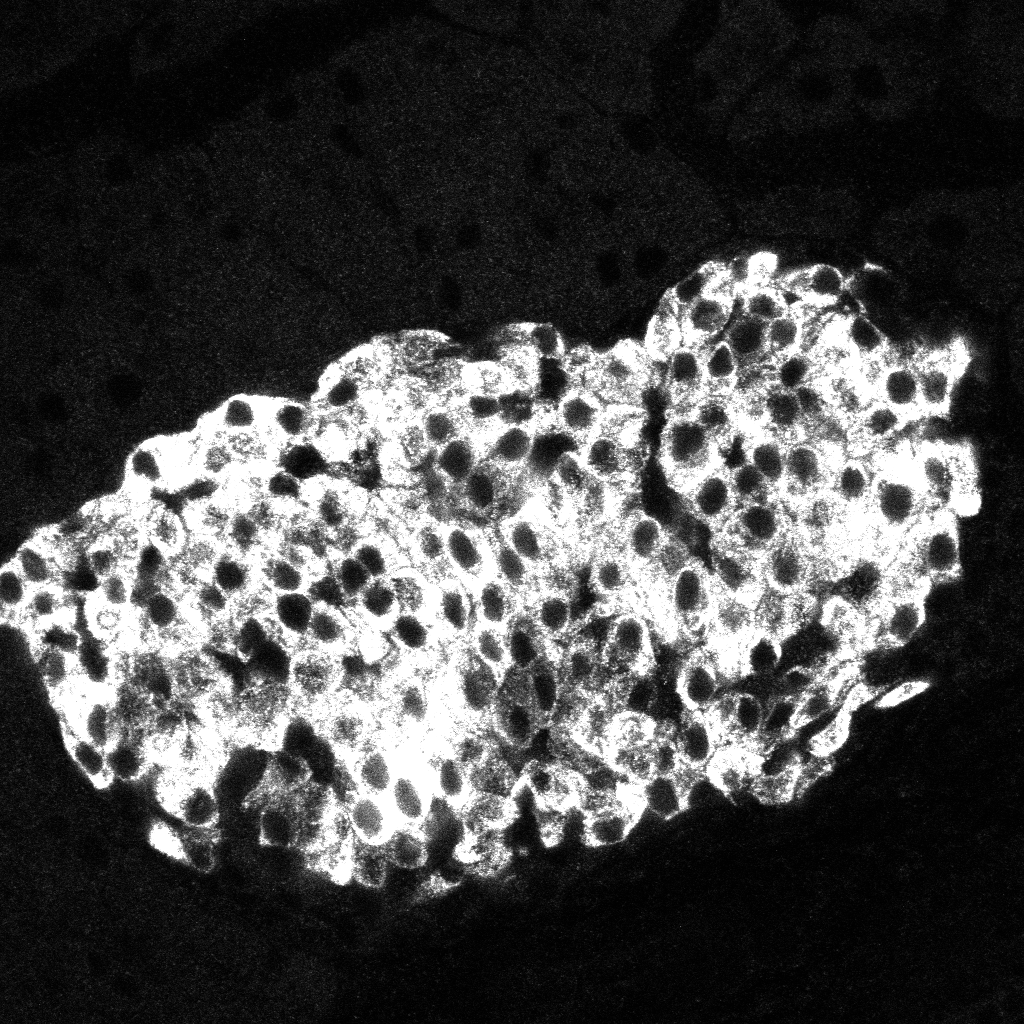

Supplement: Supplementary file 5 — Source data Fig. 3 [file 44318_2025_434_MOESM5_ESM.zip › Figure 3/3F/3F_Merge (gray).tif]

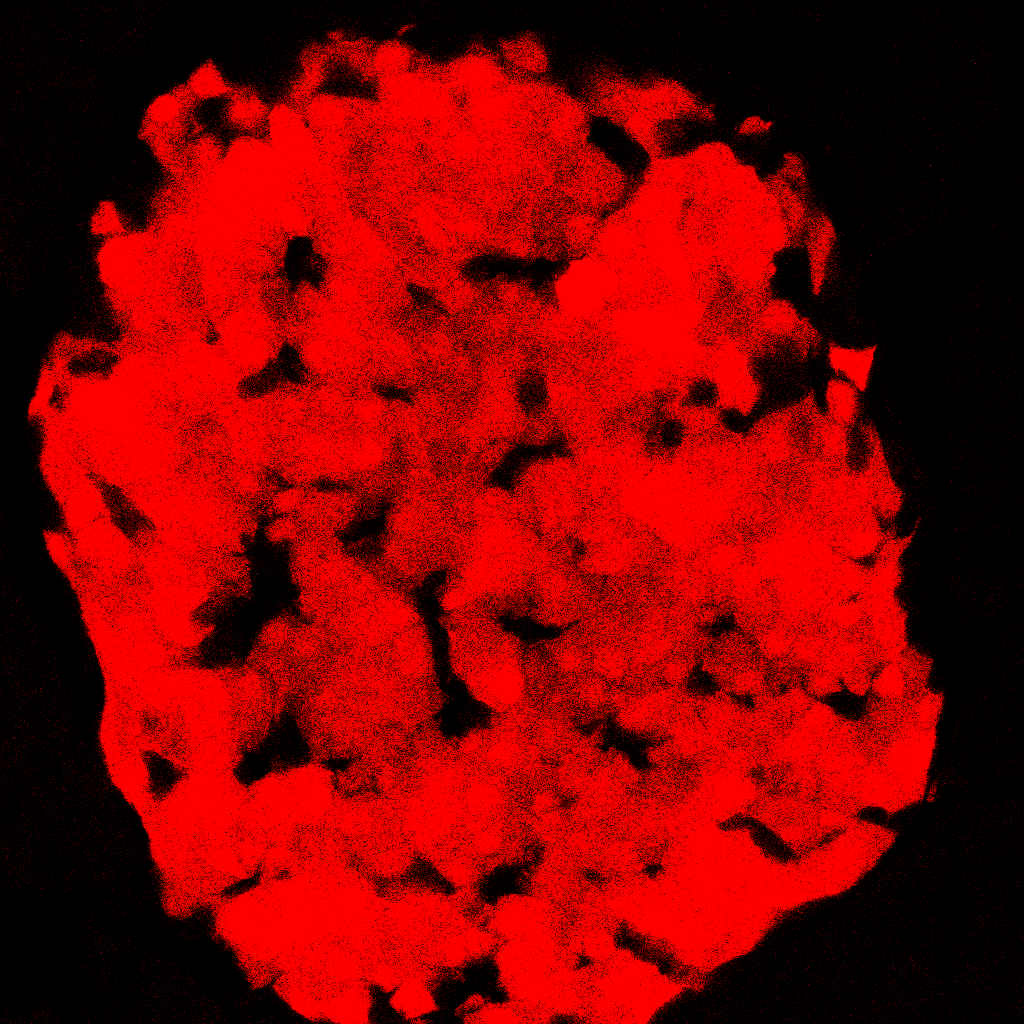

Supplement: Supplementary file 5 — Source data Fig. 3 [file 44318_2025_434_MOESM5_ESM.zip › Figure 3/3G/3G_Merge (red).tif]

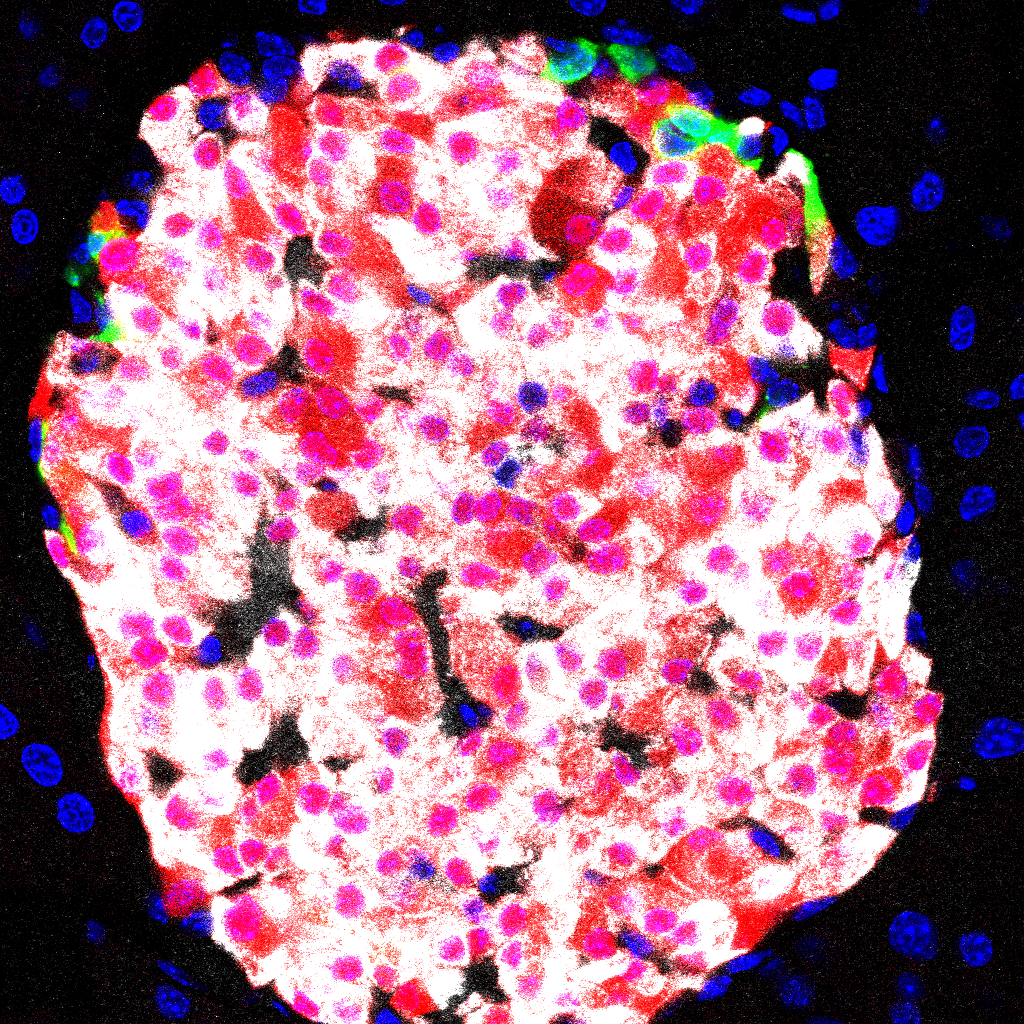

Supplement: Supplementary file 5 — Source data Fig. 3 [file 44318_2025_434_MOESM5_ESM.zip › Figure 3/3G/3G_Merge.tif]

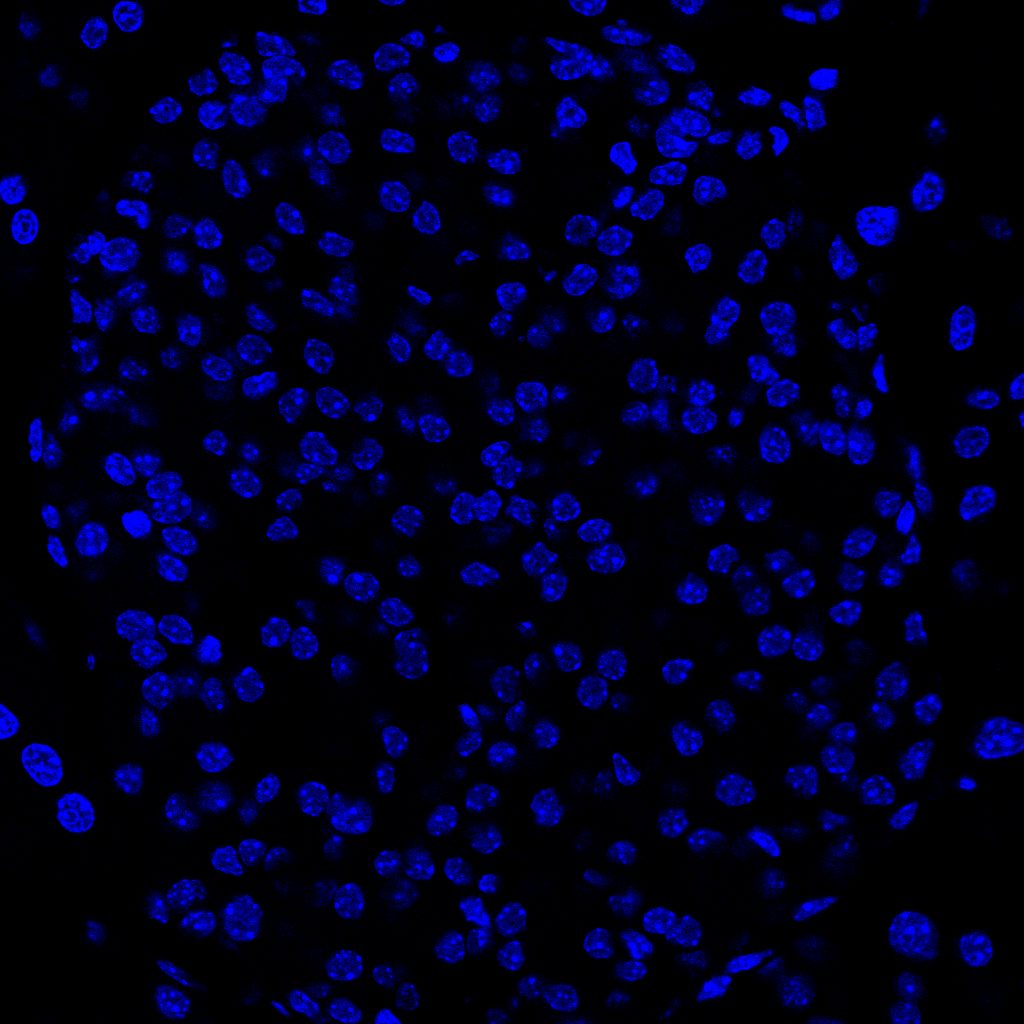

Supplement: Supplementary file 5 — Source data Fig. 3 [file 44318_2025_434_MOESM5_ESM.zip › Figure 3/3G/3G_Merge (blue).tif]

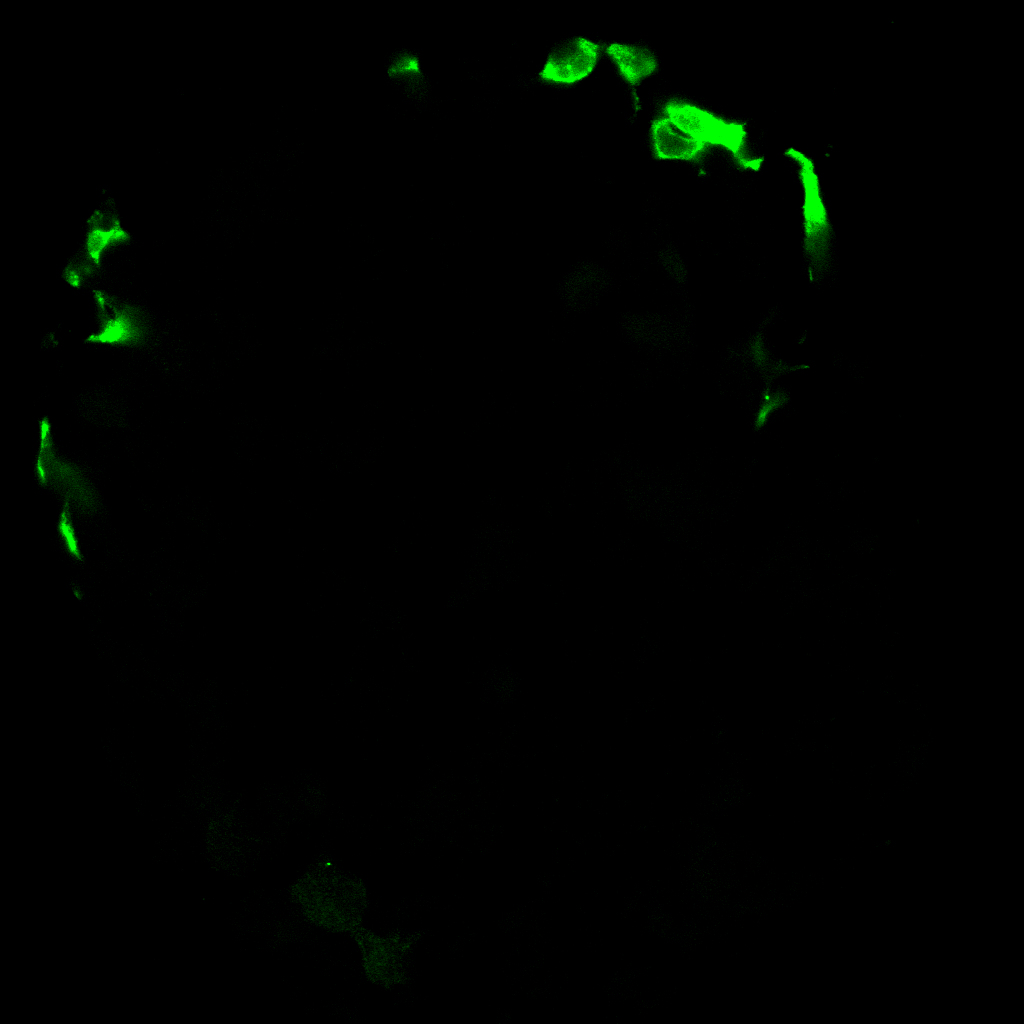

Supplement: Supplementary file 5 — Source data Fig. 3 [file 44318_2025_434_MOESM5_ESM.zip › Figure 3/3G/3G_Merge (green).tif]

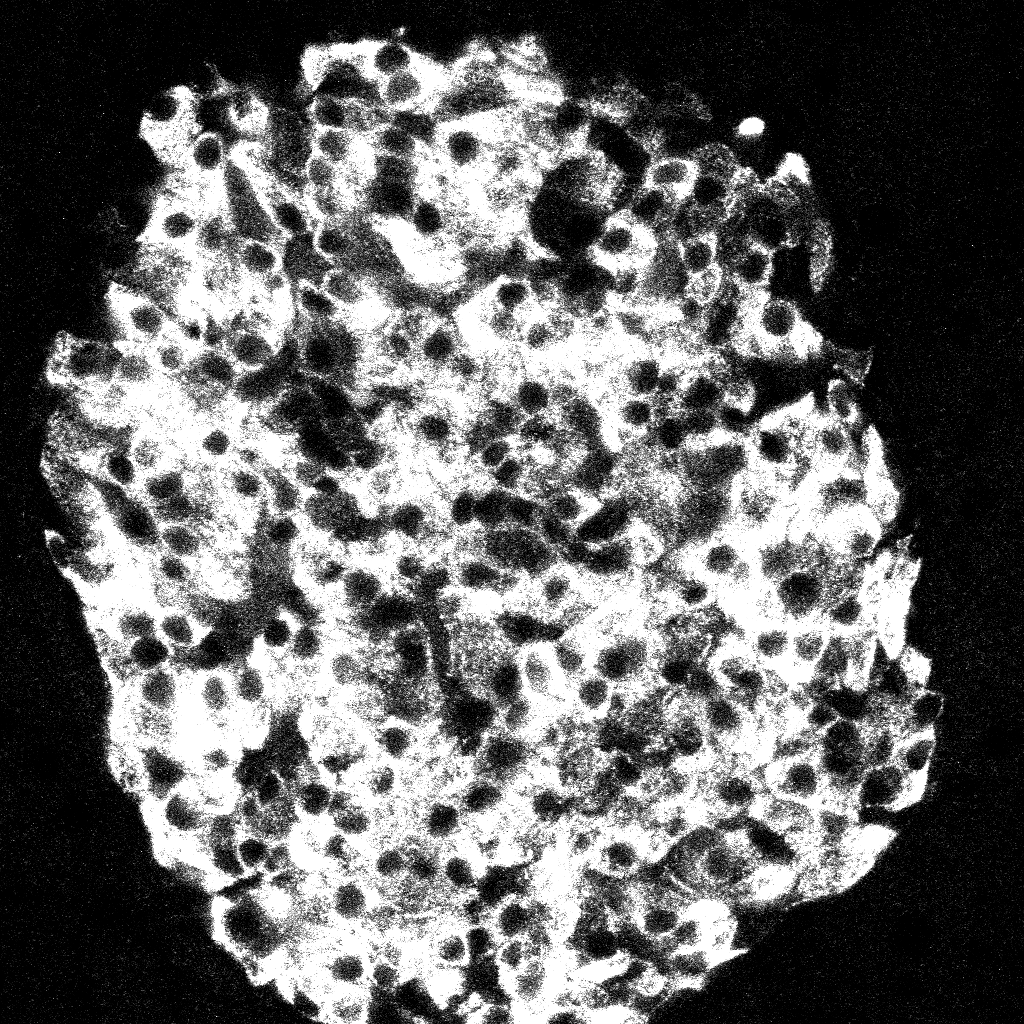

Supplement: Supplementary file 5 — Source data Fig. 3 [file 44318_2025_434_MOESM5_ESM.zip › Figure 3/3G/3G_Merge (gray).tif]

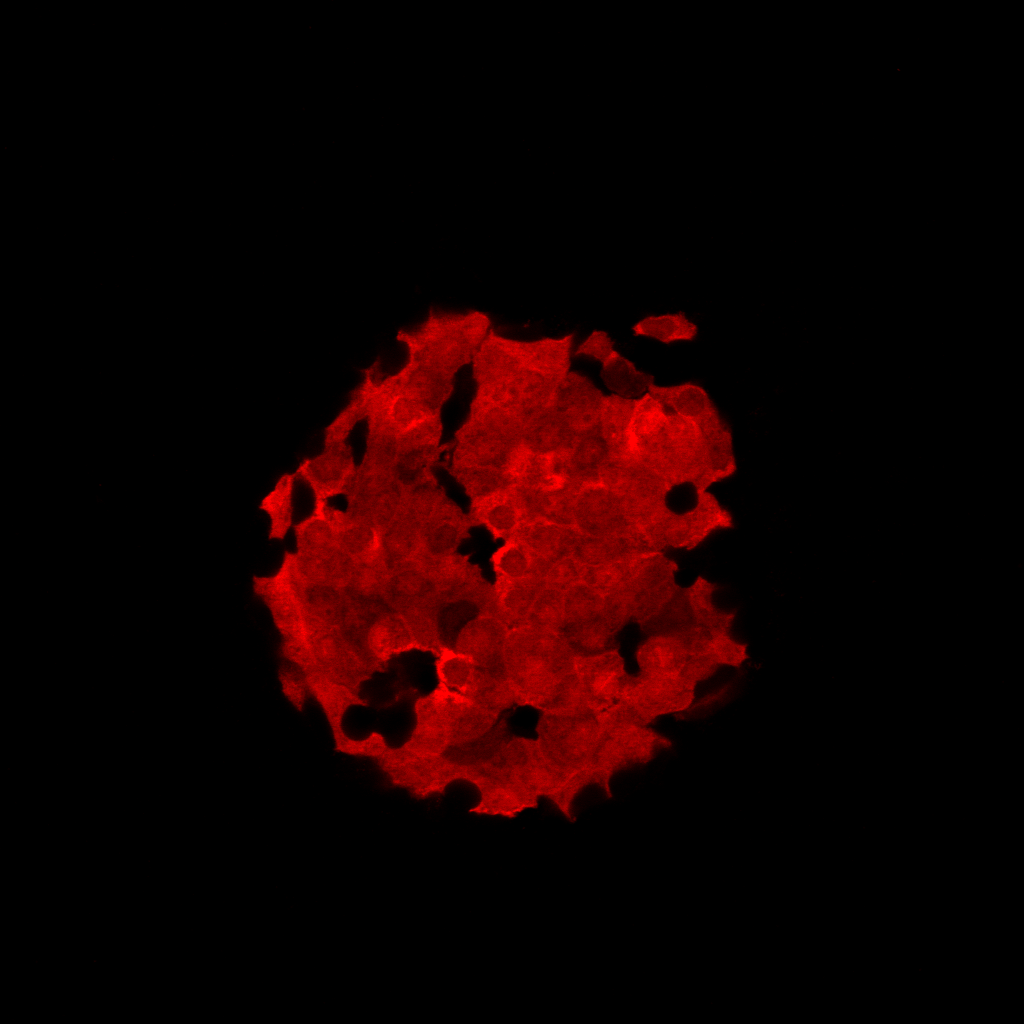

Supplement: Supplementary file 6 — Source data Fig. 4 [file 44318_2025_434_MOESM6_ESM.zip › Figure 4/4E/4E_15.tif (red).tif]

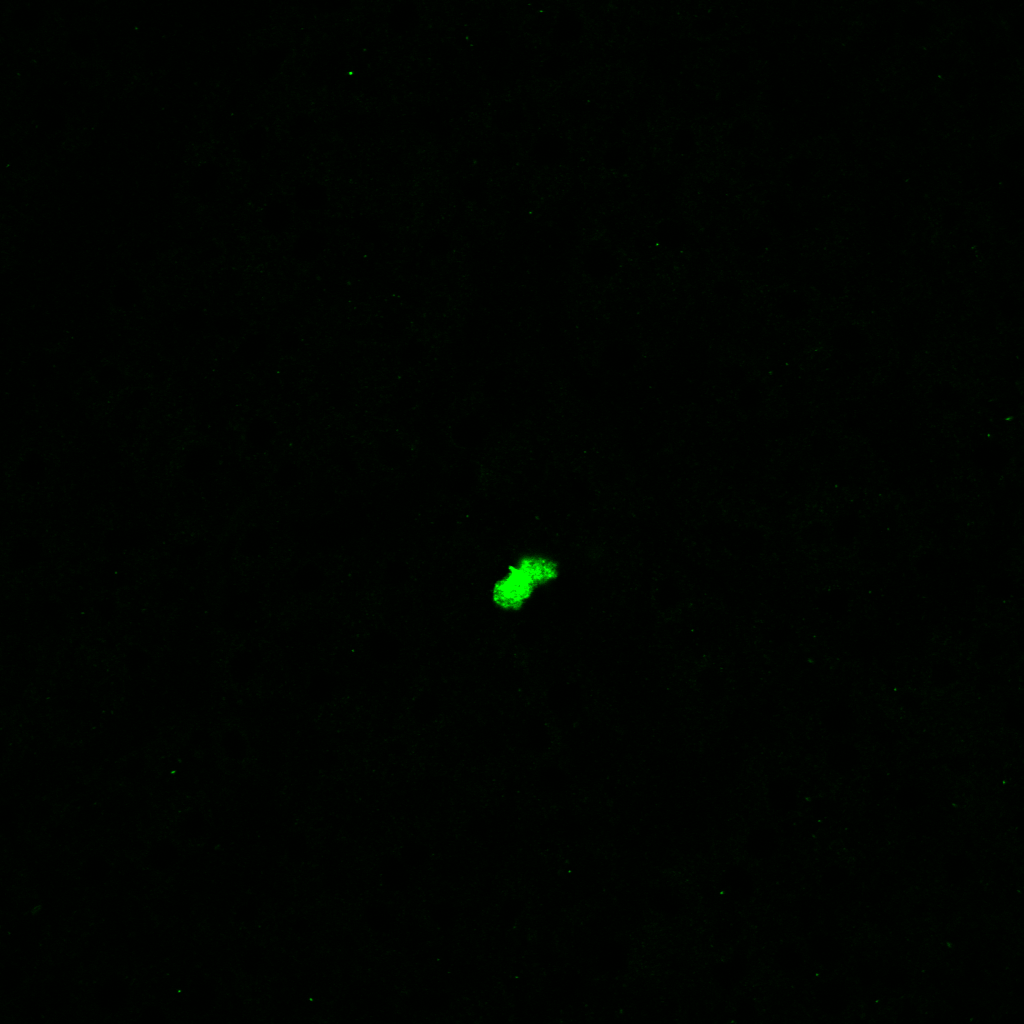

Supplement: Supplementary file 6 — Source data Fig. 4 [file 44318_2025_434_MOESM6_ESM.zip › Figure 4/4E/4E_23.tif (green).tif]

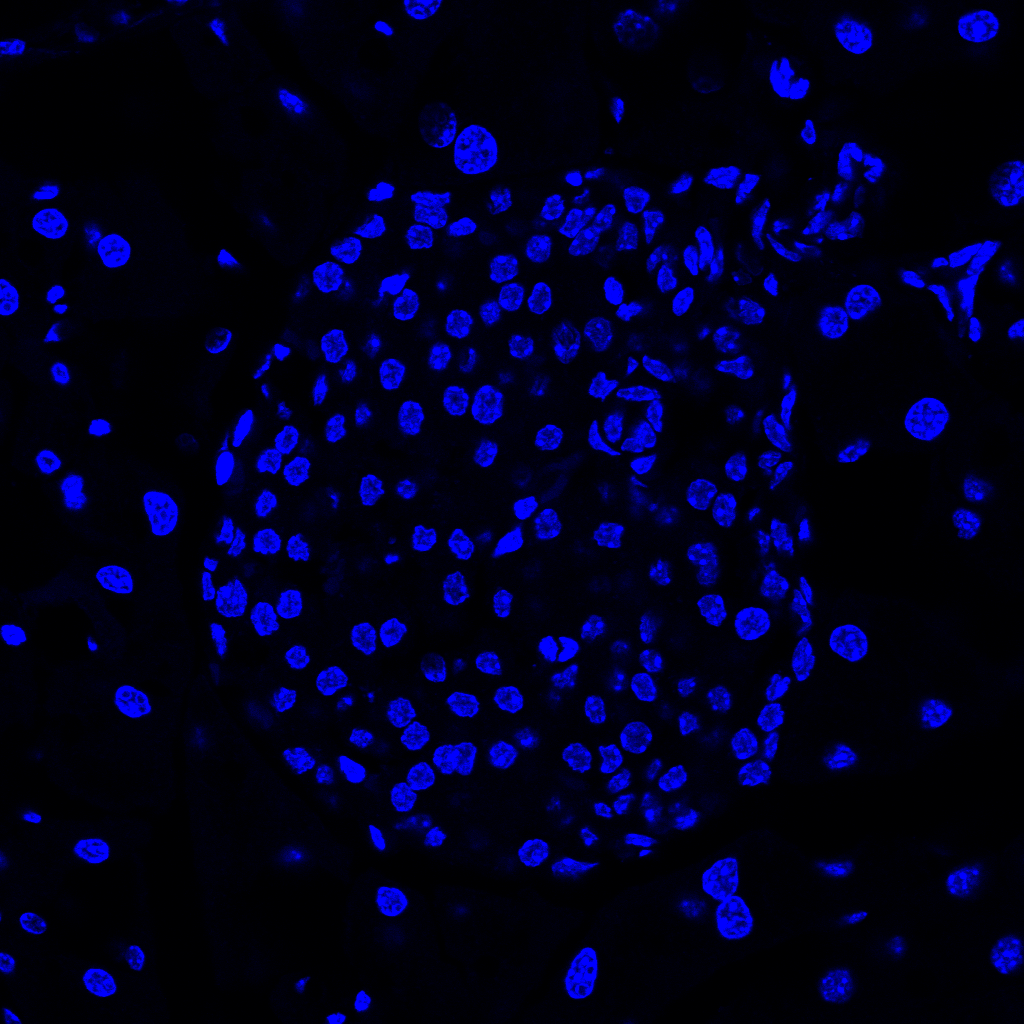

Supplement: Supplementary file 6 — Source data Fig. 4 [file 44318_2025_434_MOESM6_ESM.zip › Figure 4/4E/4E_13.tif (blue).tif]

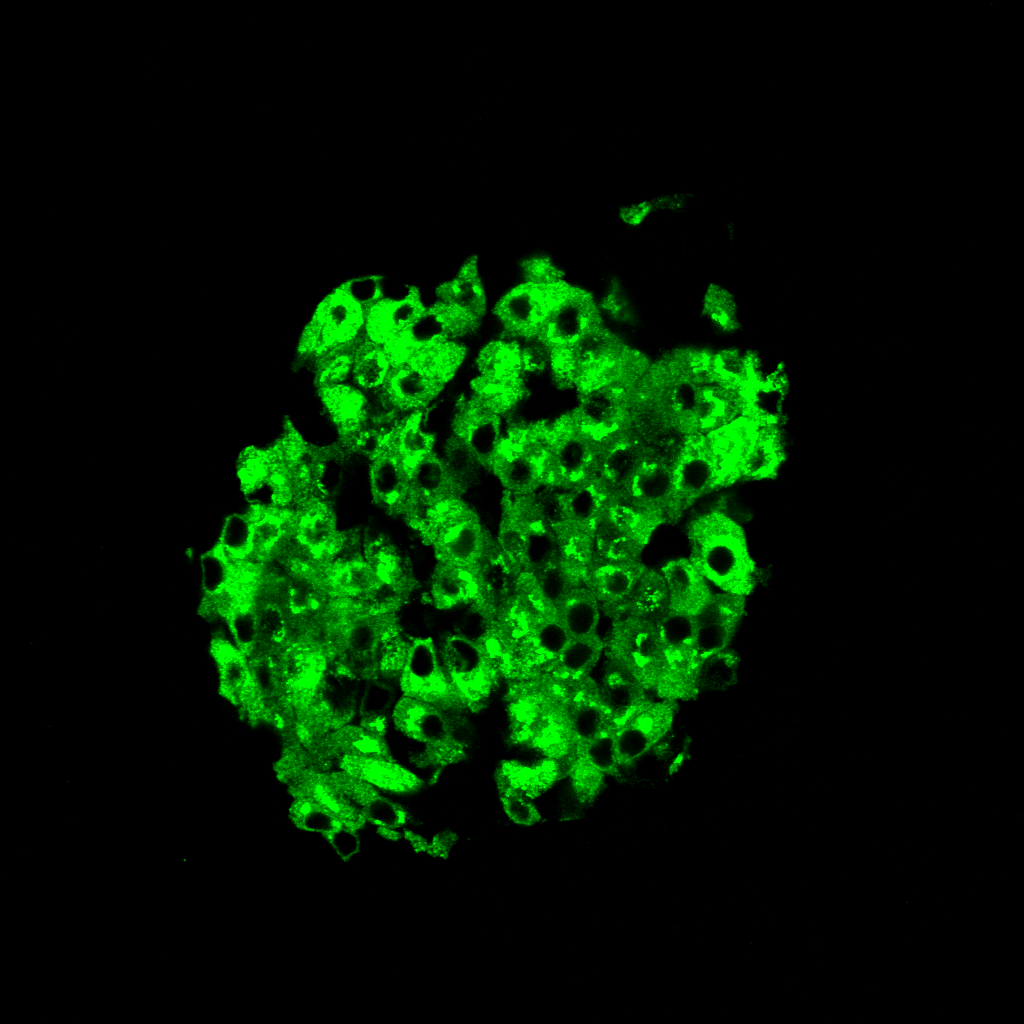

Supplement: Supplementary file 6 — Source data Fig. 4 [file 44318_2025_434_MOESM6_ESM.zip › Figure 4/4E/4E_5.tif (green).tif]

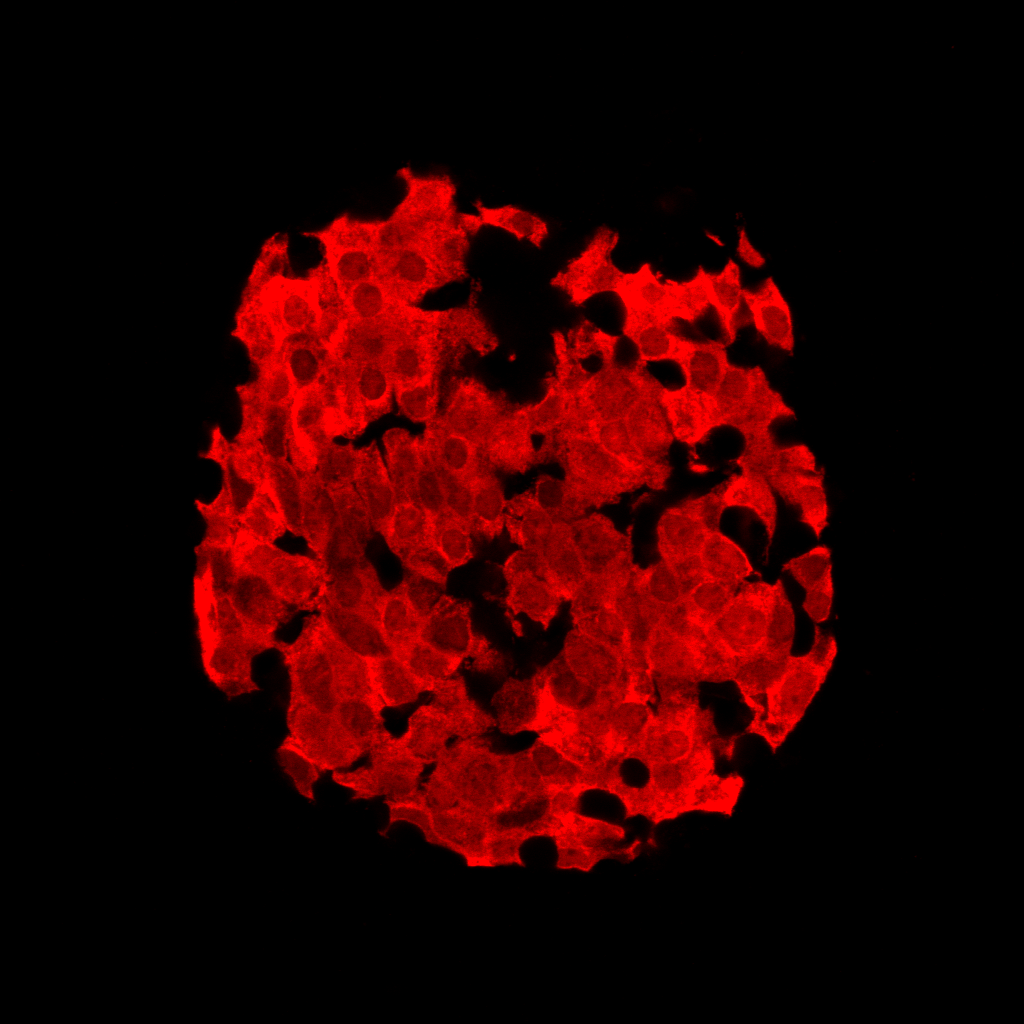

Supplement: Supplementary file 6 — Source data Fig. 4 [file 44318_2025_434_MOESM6_ESM.zip › Figure 4/4E/4E_11.tif (red).tif]

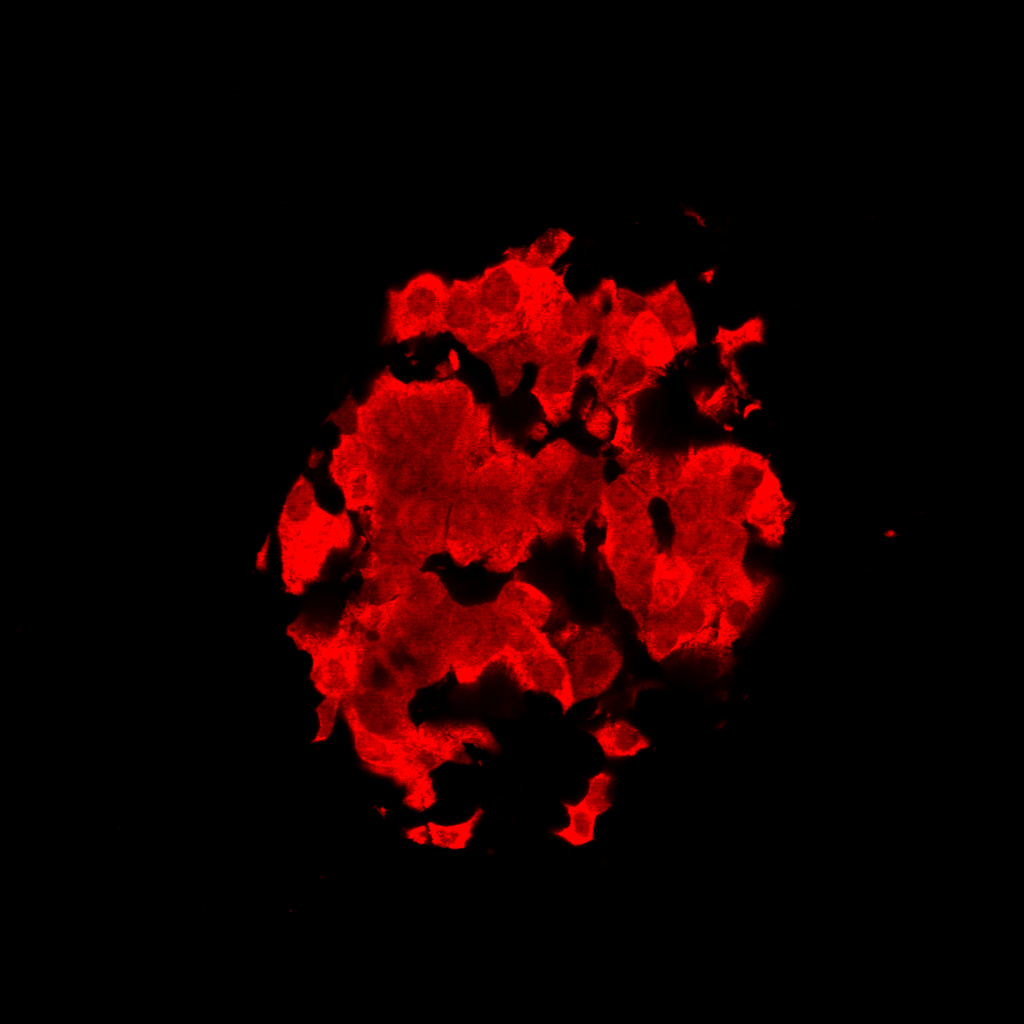

Supplement: Supplementary file 6 — Source data Fig. 4 [file 44318_2025_434_MOESM6_ESM.zip › Figure 4/4E/4E_4.tif (red).tif]

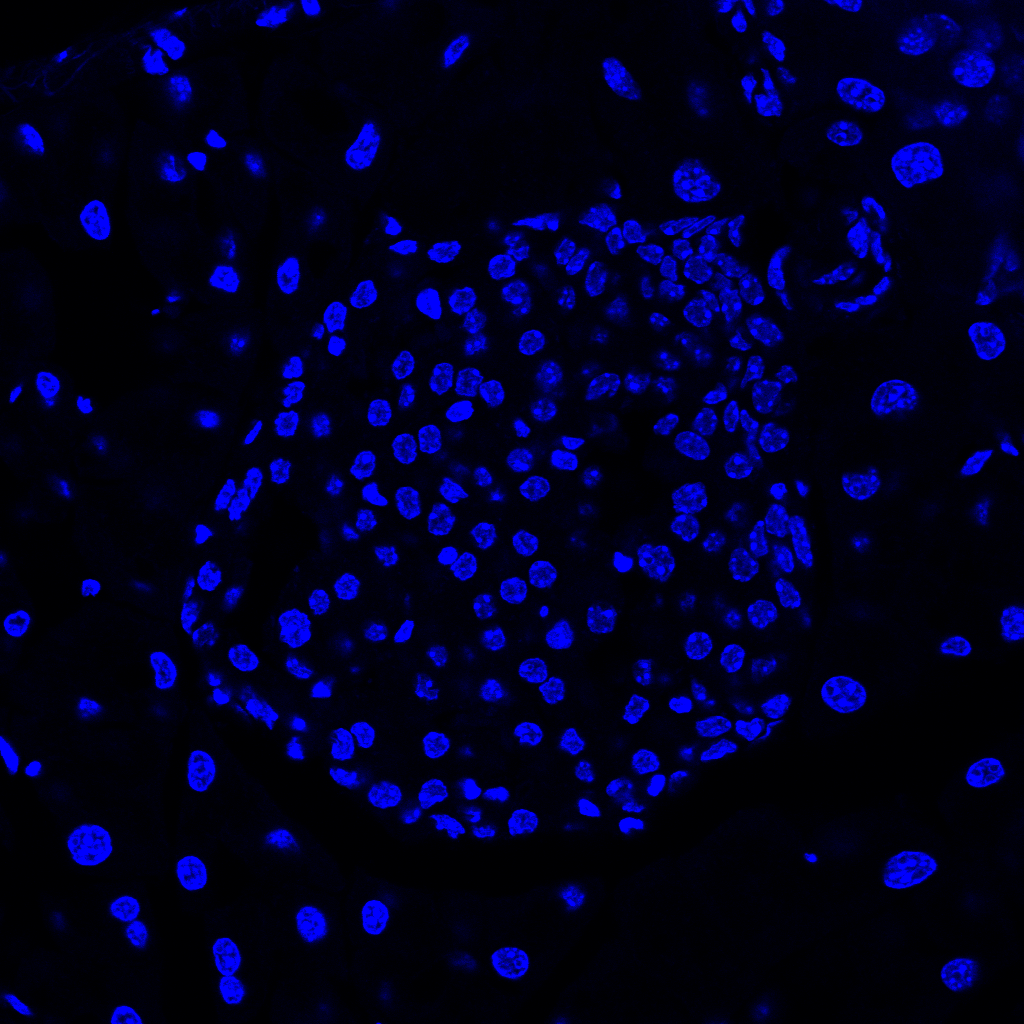

Supplement: Supplementary file 6 — Source data Fig. 4 [file 44318_2025_434_MOESM6_ESM.zip › Figure 4/4E/4E_14.tif (blue).tif]

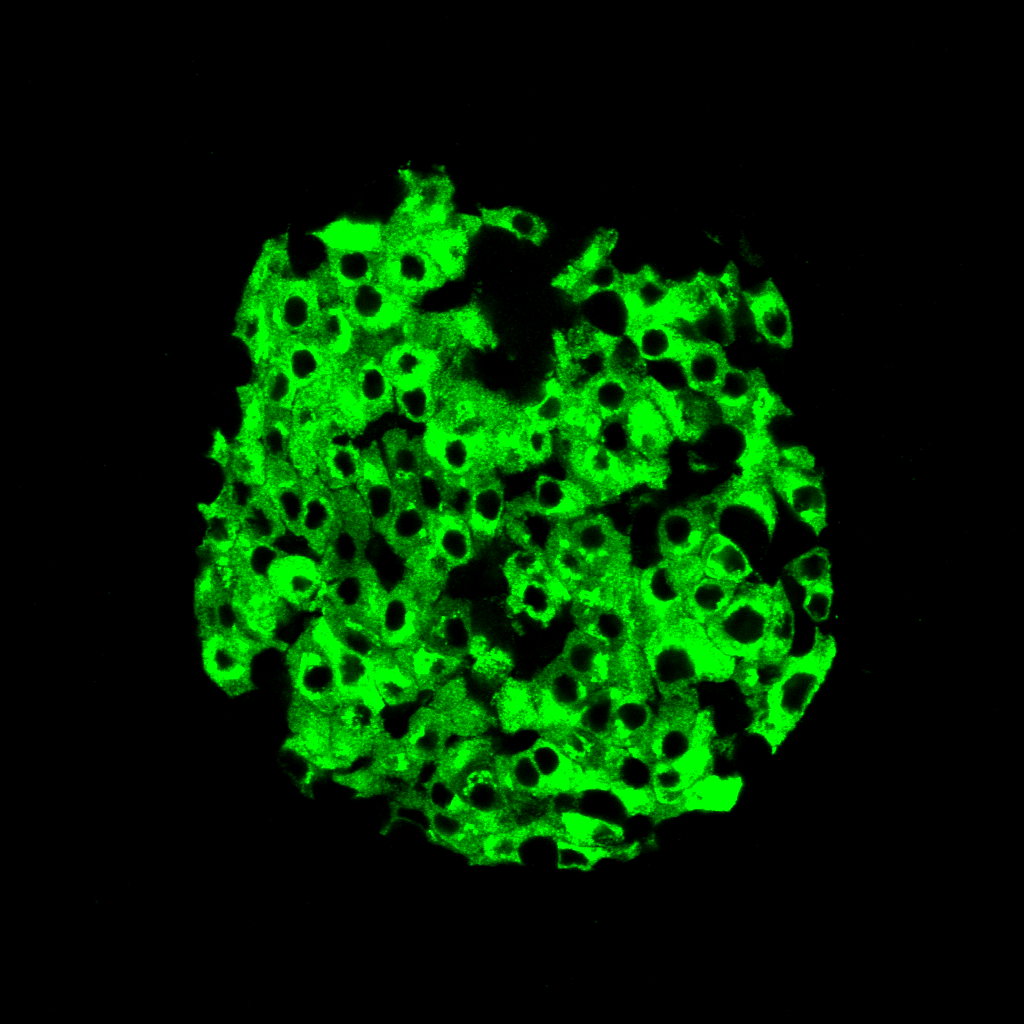

Supplement: Supplementary file 6 — Source data Fig. 4 [file 44318_2025_434_MOESM6_ESM.zip › Figure 4/4E/4E_11.tif (green).tif]

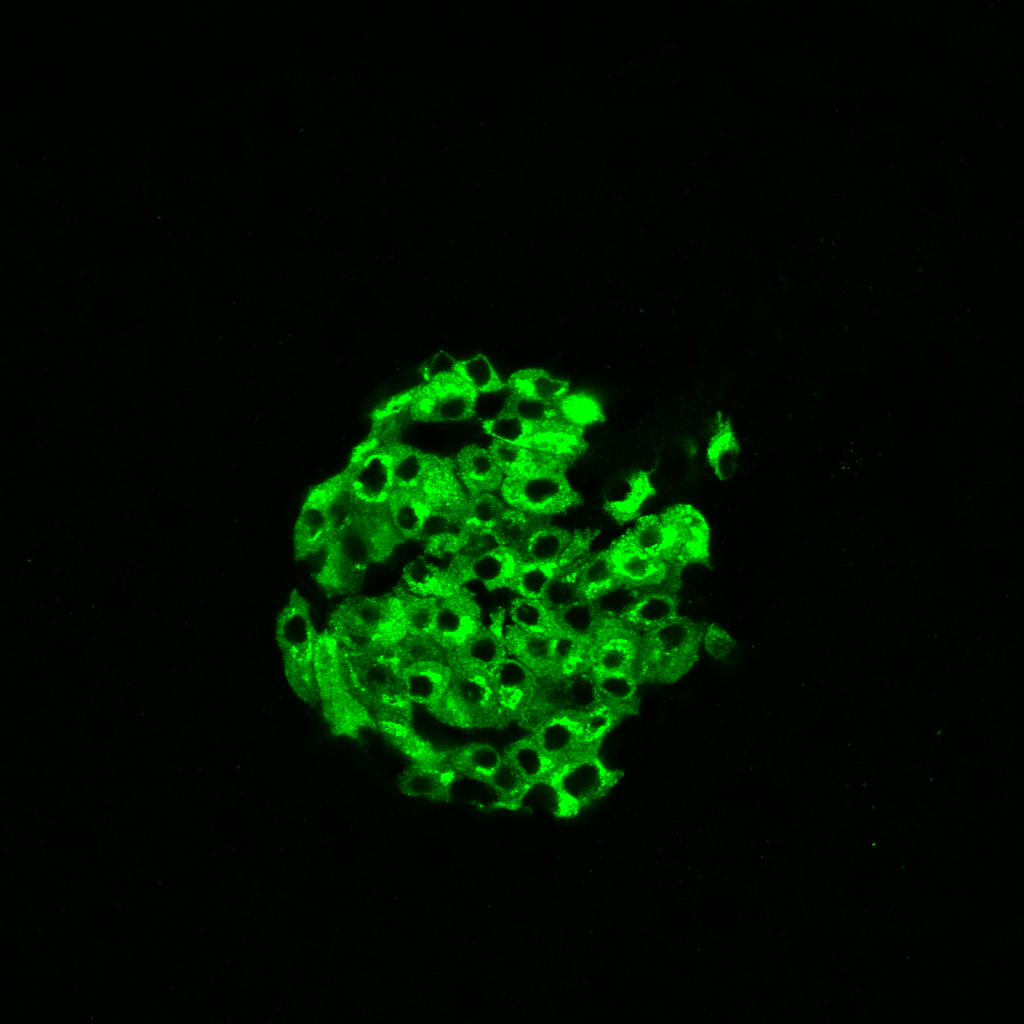

Supplement: Supplementary file 6 — Source data Fig. 4 [file 44318_2025_434_MOESM6_ESM.zip › Figure 4/4E/4E_16.tif (green).tif]

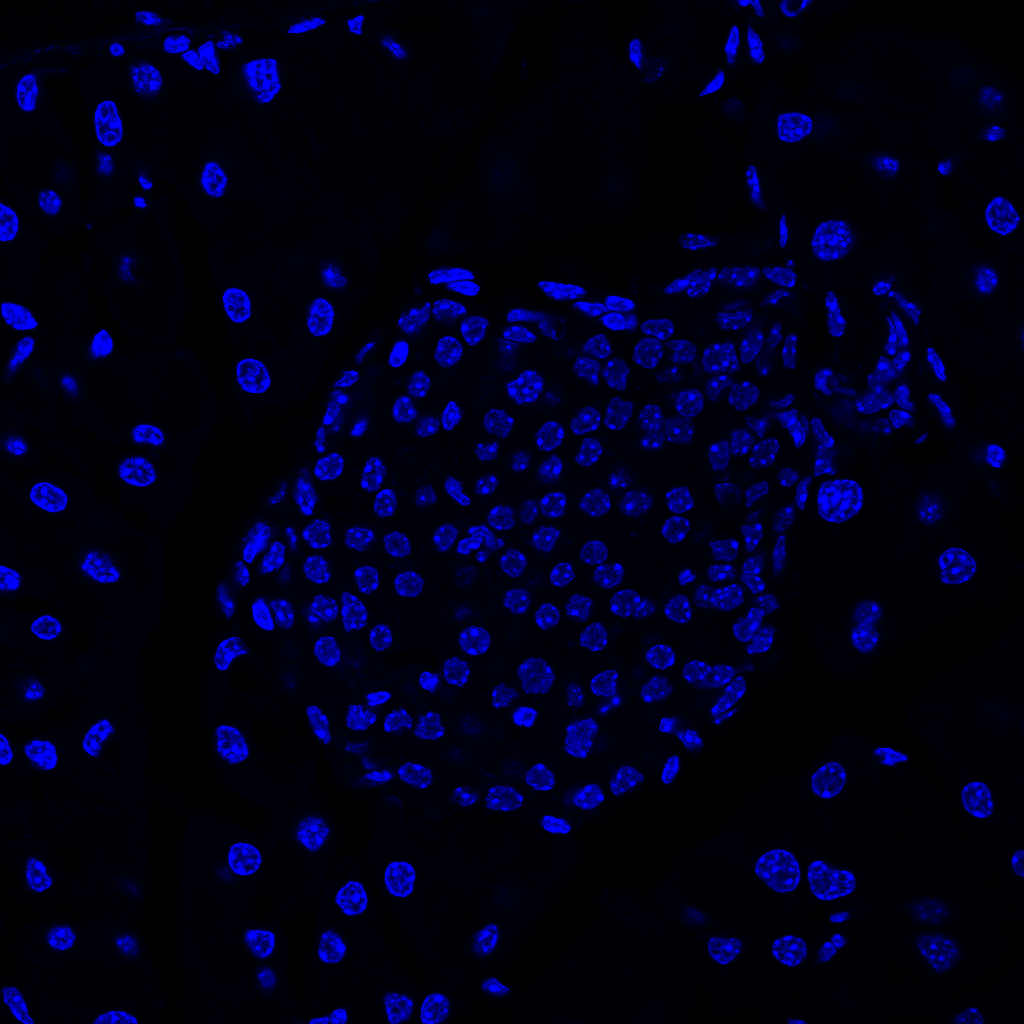

Supplement: Supplementary file 6 — Source data Fig. 4 [file 44318_2025_434_MOESM6_ESM.zip › Figure 4/4E/4E_15.tif (blue).tif]

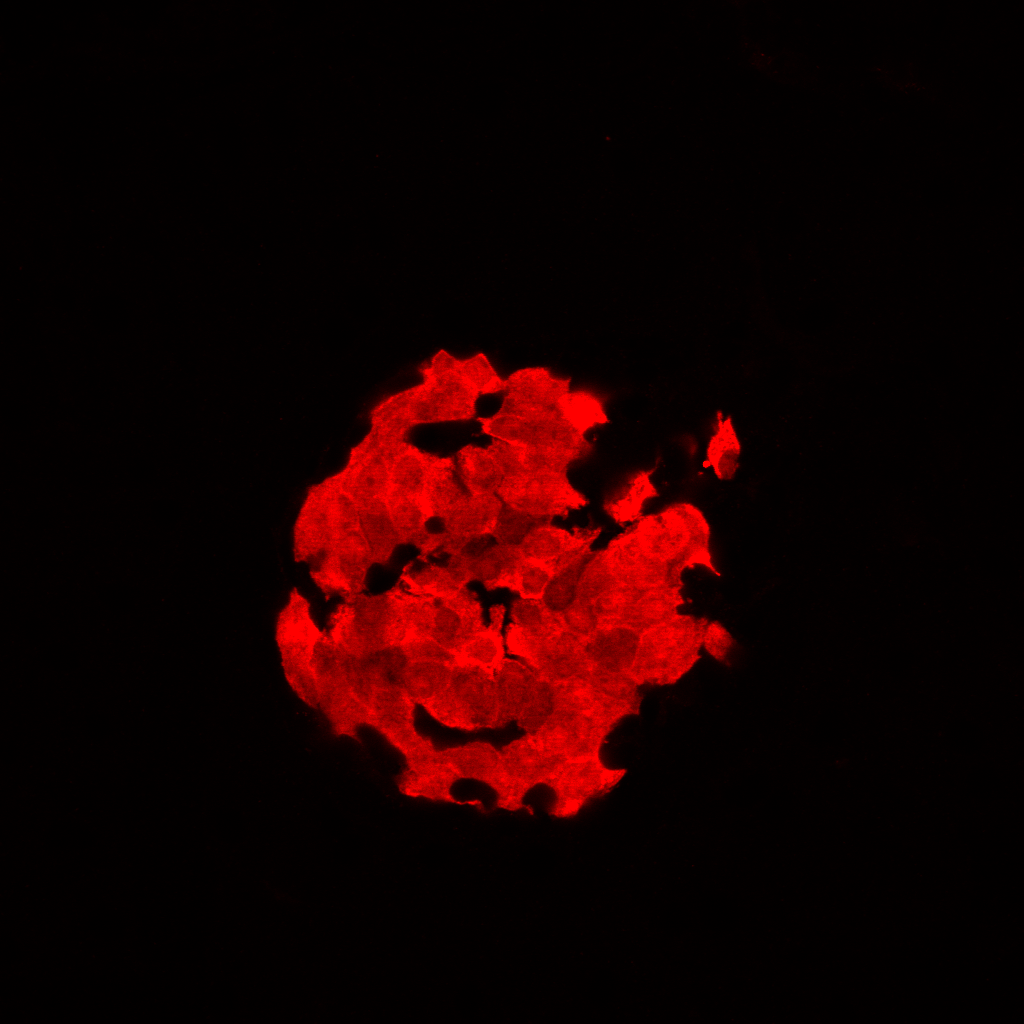

Supplement: Supplementary file 6 — Source data Fig. 4 [file 44318_2025_434_MOESM6_ESM.zip › Figure 4/4E/4E_16.tif (red).tif]

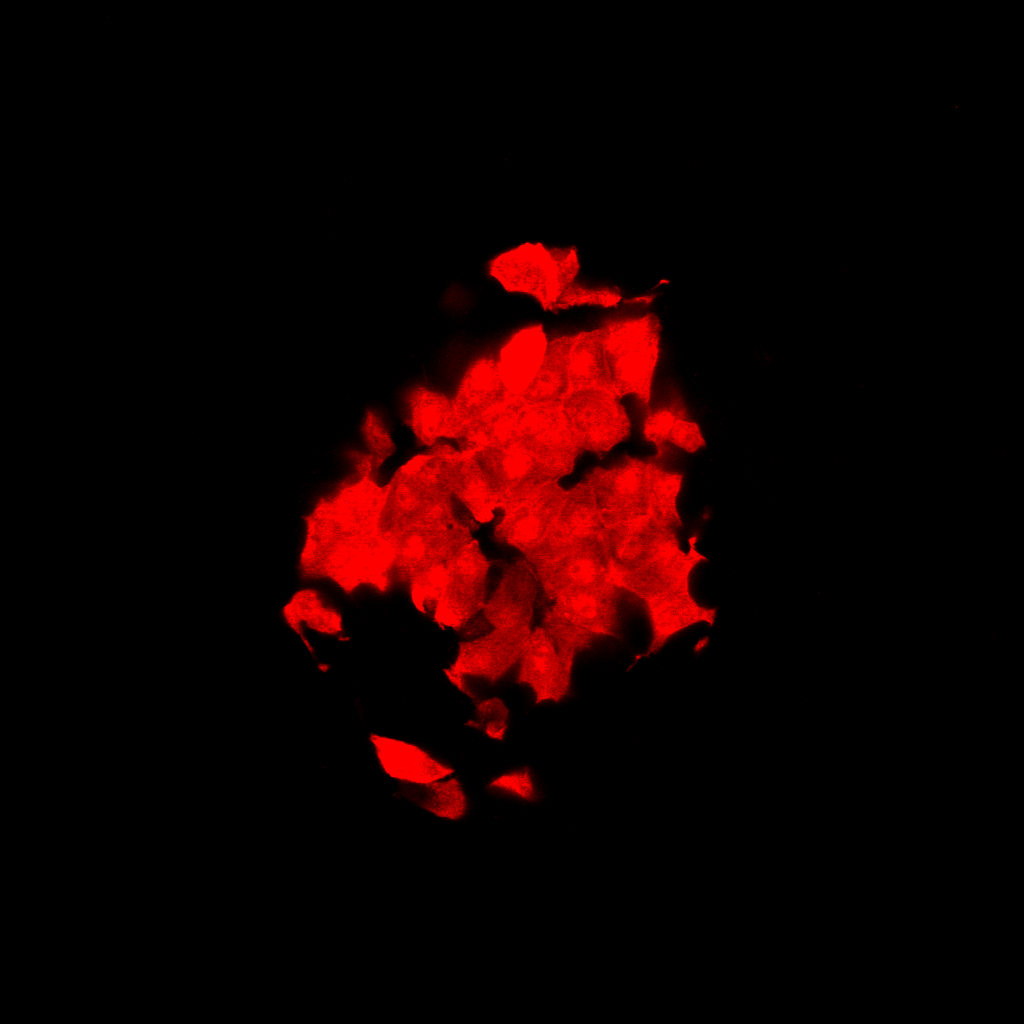

Supplement: Supplementary file 6 — Source data Fig. 4 [file 44318_2025_434_MOESM6_ESM.zip › Figure 4/4E/4E_3.tif (red).tif]

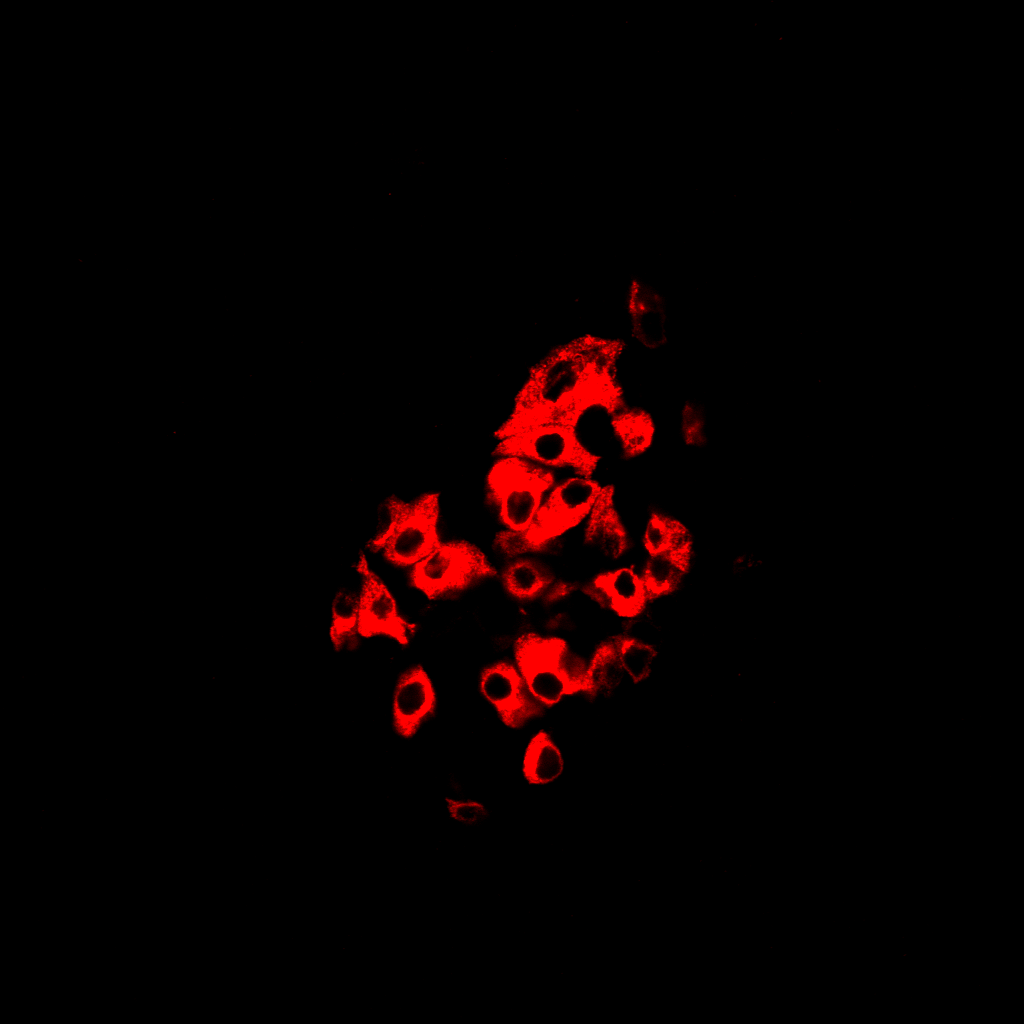

Supplement: Supplementary file 6 — Source data Fig. 4 [file 44318_2025_434_MOESM6_ESM.zip › Figure 4/4E/4E_2.tif (green).tif]

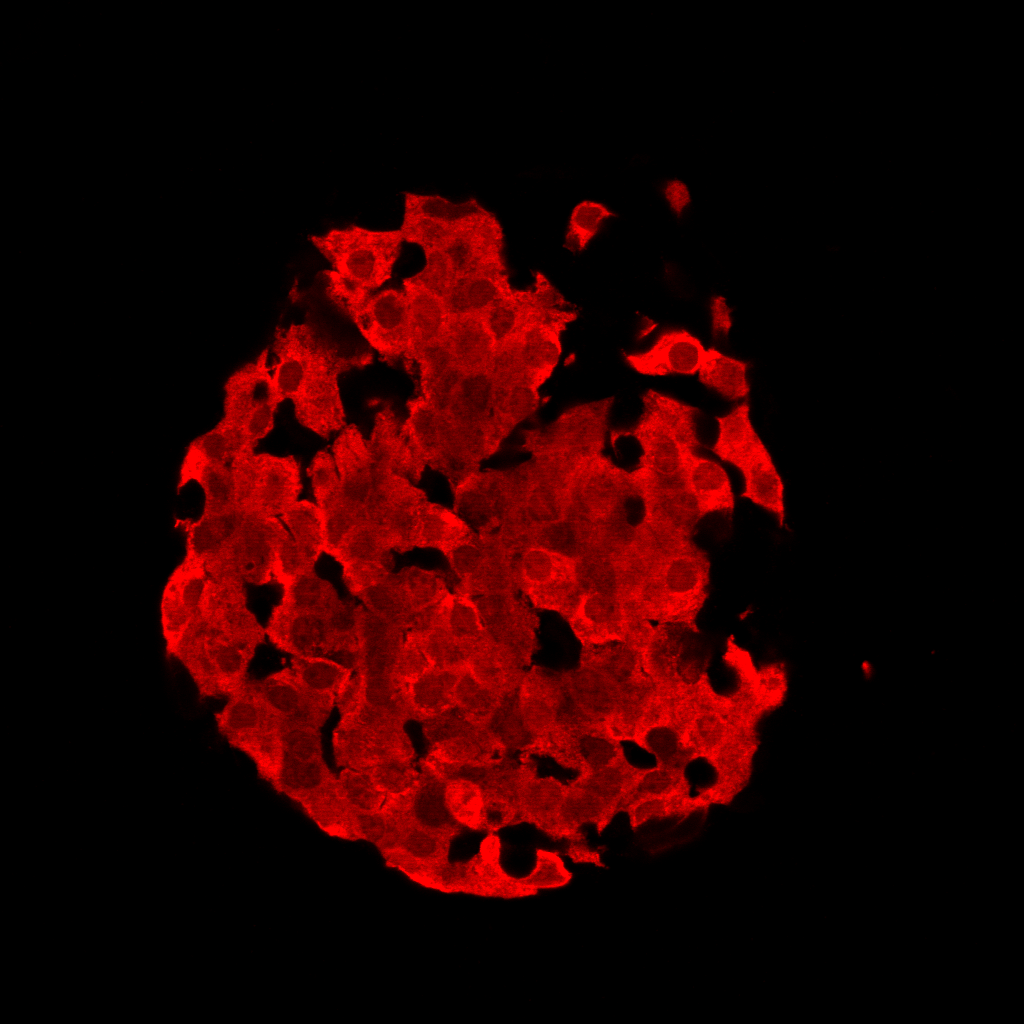

Supplement: Supplementary file 6 — Source data Fig. 4 [file 44318_2025_434_MOESM6_ESM.zip › Figure 4/4E/4E_12.tif (red).tif]

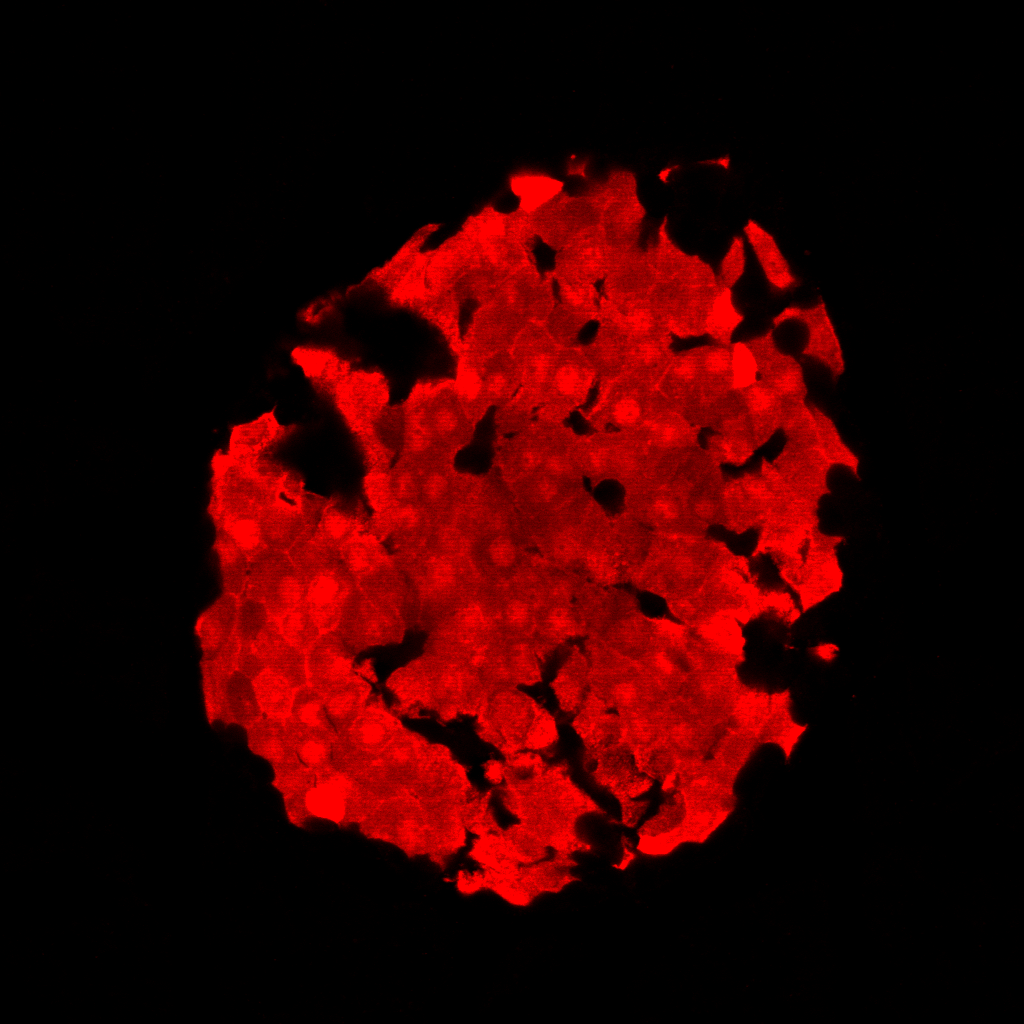

Supplement: Supplementary file 6 — Source data Fig. 4 [file 44318_2025_434_MOESM6_ESM.zip › Figure 4/4E/4E_7.tif (red).tif]

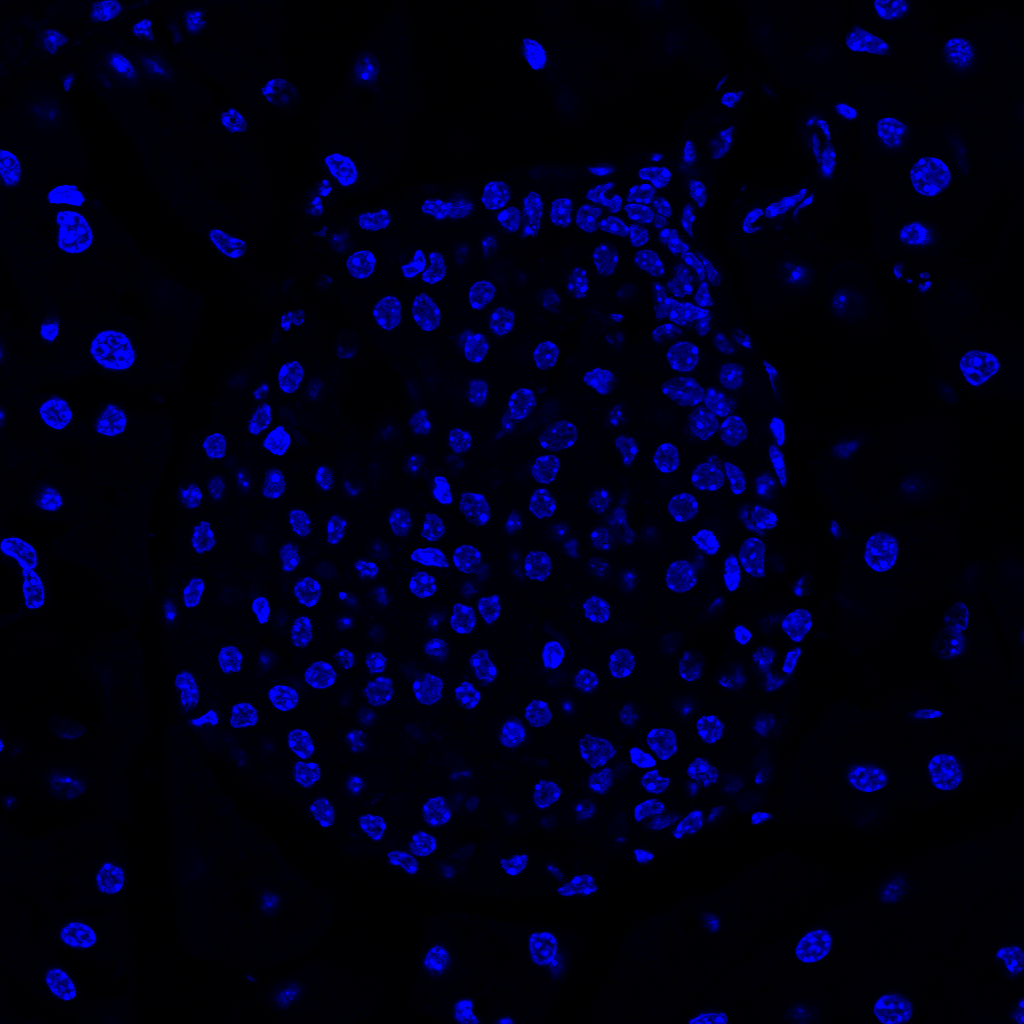

Supplement: Supplementary file 6 — Source data Fig. 4 [file 44318_2025_434_MOESM6_ESM.zip › Figure 4/4E/4E_12.tif (blue).tif]

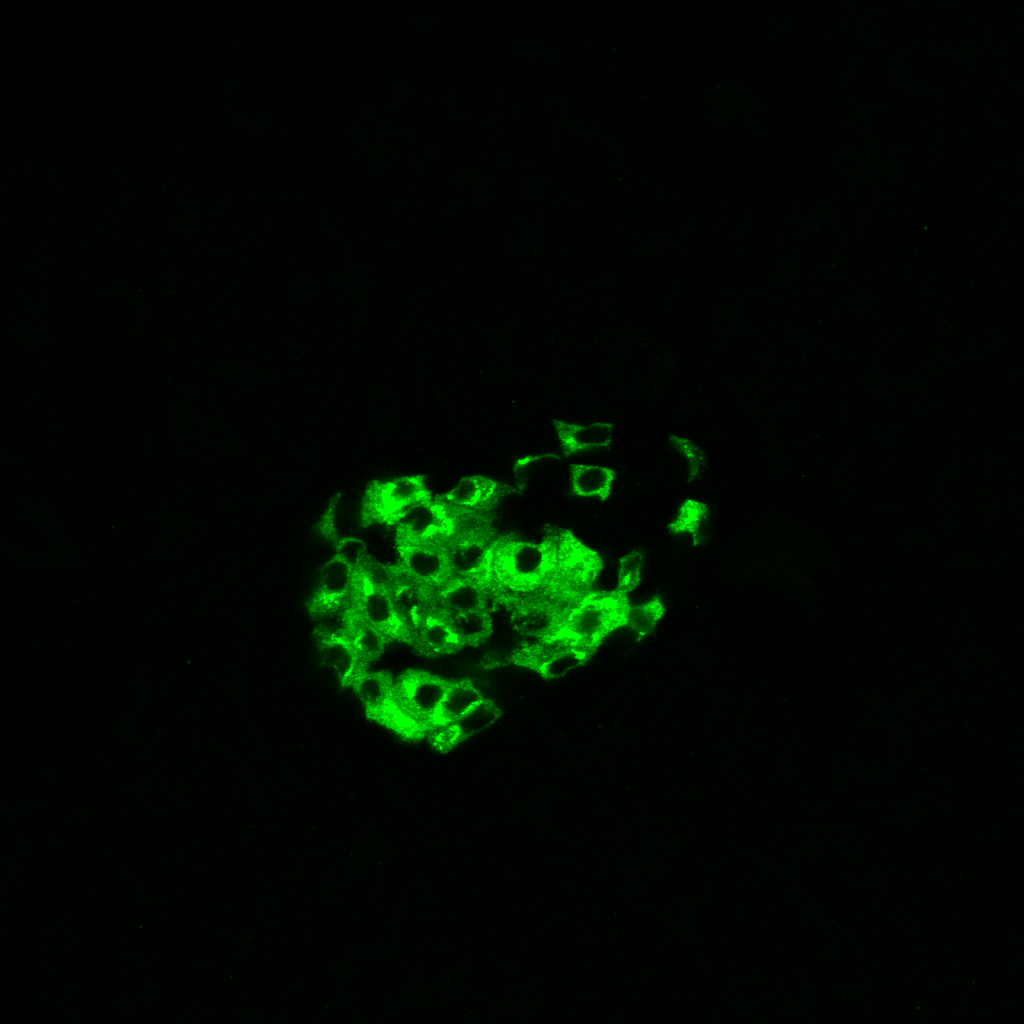

Supplement: Supplementary file 6 — Source data Fig. 4 [file 44318_2025_434_MOESM6_ESM.zip › Figure 4/4E/4E_19.tif (green).tif]

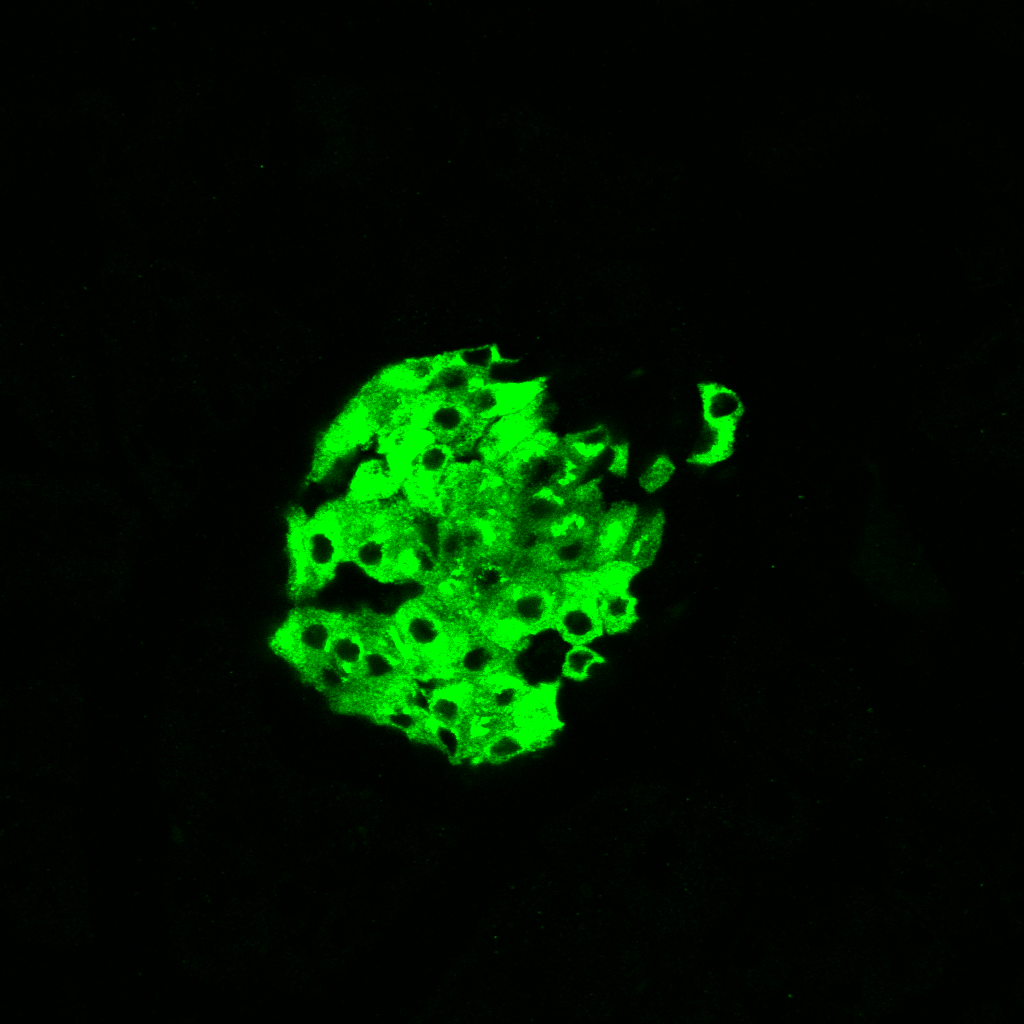

Supplement: Supplementary file 6 — Source data Fig. 4 [file 44318_2025_434_MOESM6_ESM.zip › Figure 4/4E/4E_17.tif (green).tif]

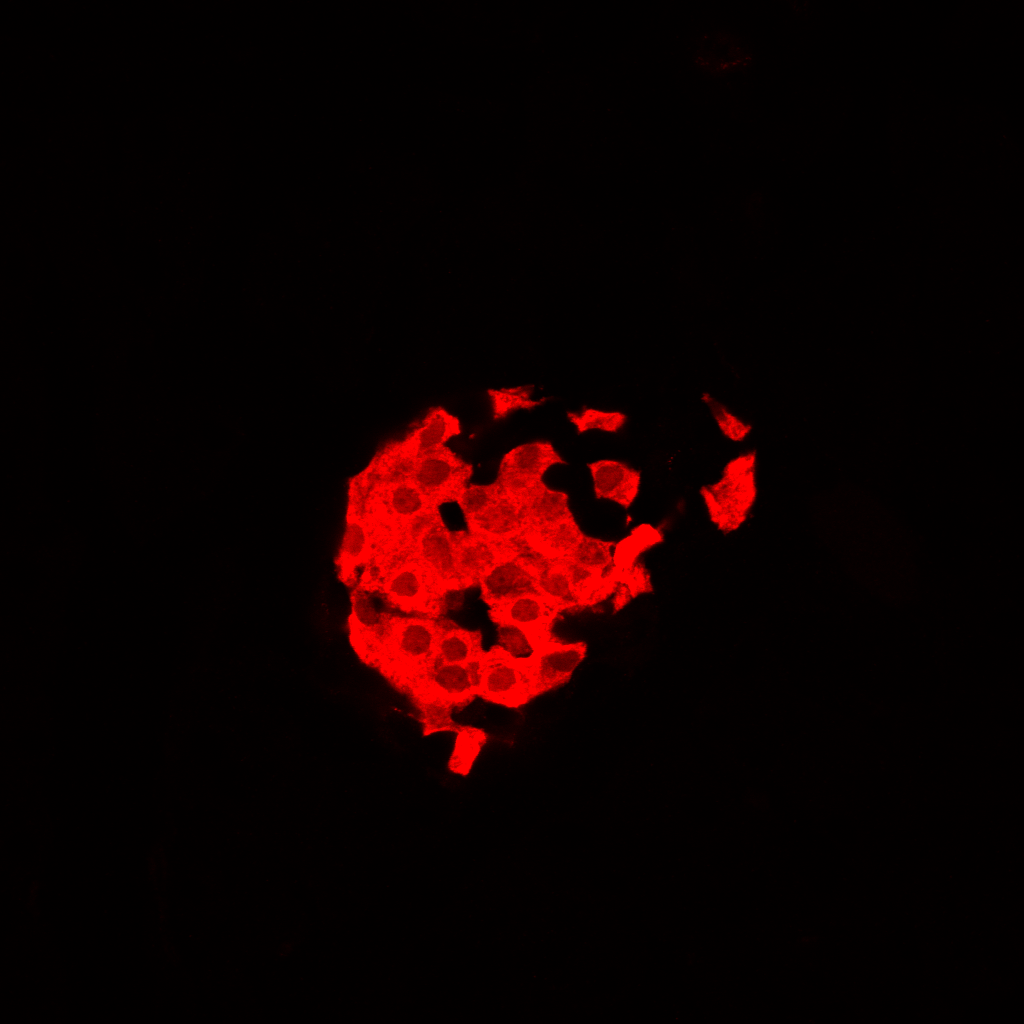

Supplement: Supplementary file 6 — Source data Fig. 4 [file 44318_2025_434_MOESM6_ESM.zip › Figure 4/4E/4E_18.tif (red).tif]

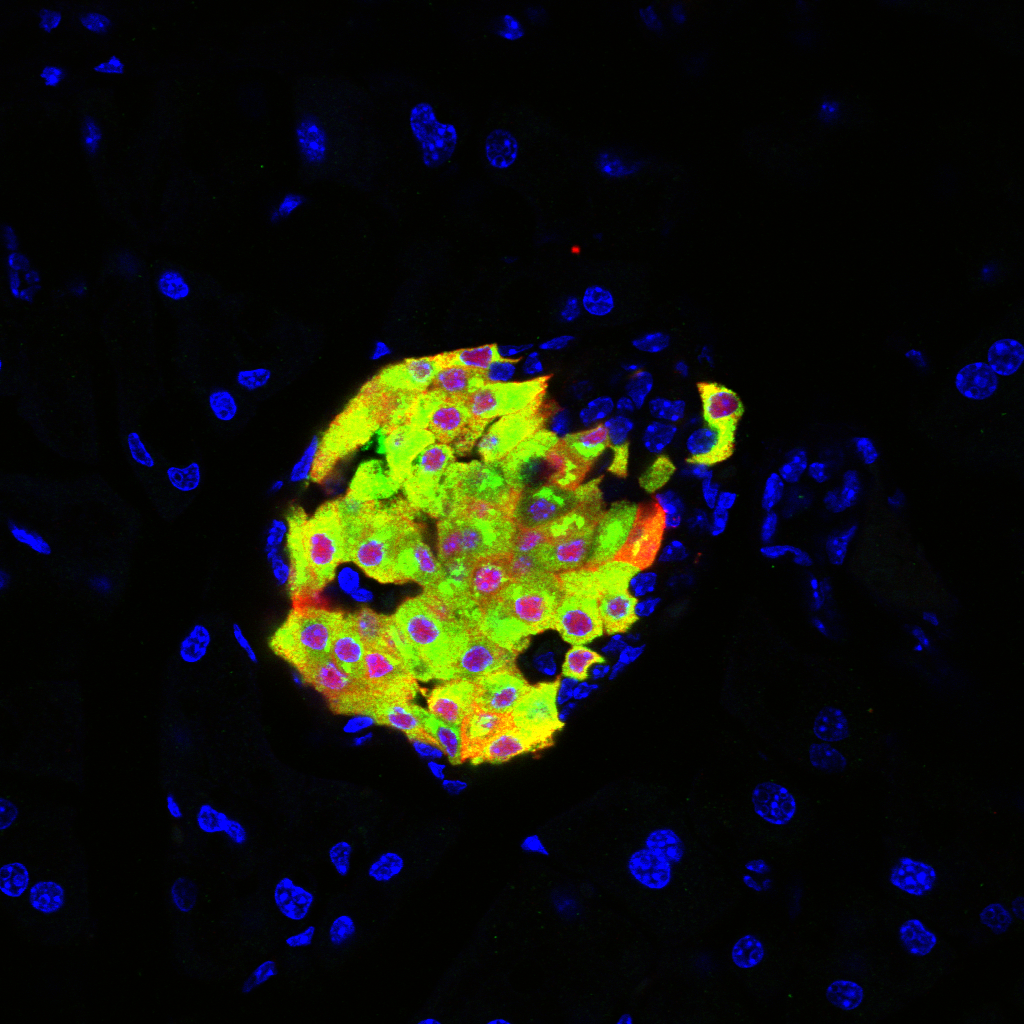

Supplement: Supplementary file 6 — Source data Fig. 4 [file 44318_2025_434_MOESM6_ESM.zip › Figure 4/4E/4E_17.tif]

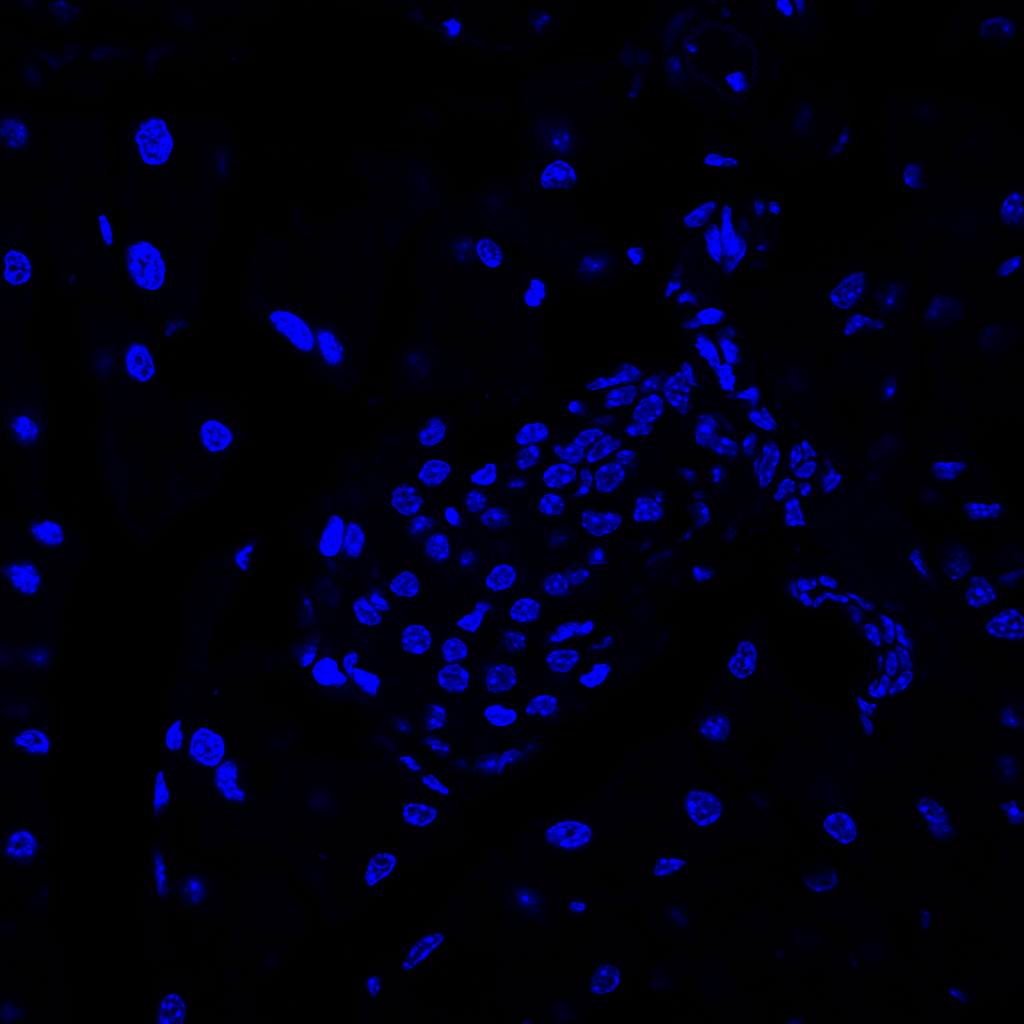

Supplement: Supplementary file 6 — Source data Fig. 4 [file 44318_2025_434_MOESM6_ESM.zip › Figure 4/4E/4E_18.tif (blue).tif]

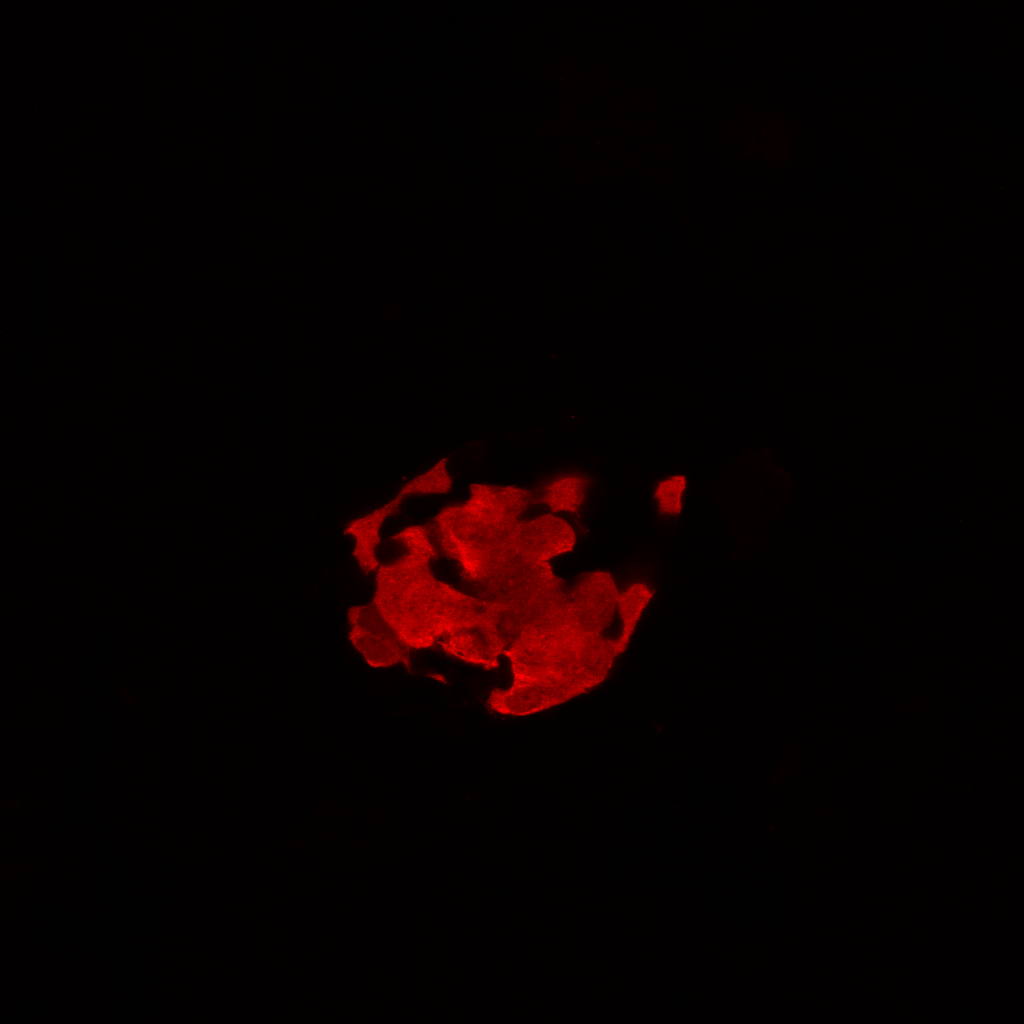

Supplement: Supplementary file 6 — Source data Fig. 4 [file 44318_2025_434_MOESM6_ESM.zip › Figure 4/4E/4E_21.tif (red).tif]

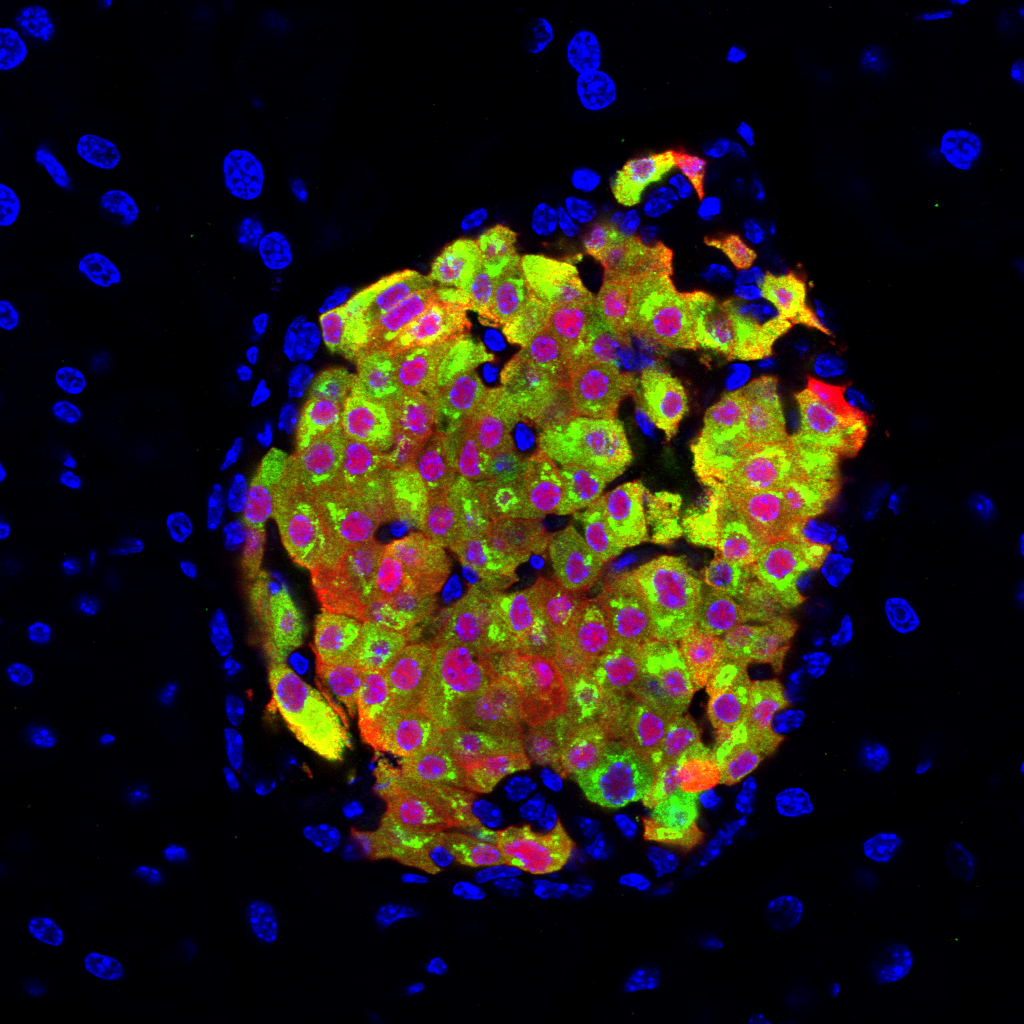

Supplement: Supplementary file 6 — Source data Fig. 4 [file 44318_2025_434_MOESM6_ESM.zip › Figure 4/4E/4E_6.tif]

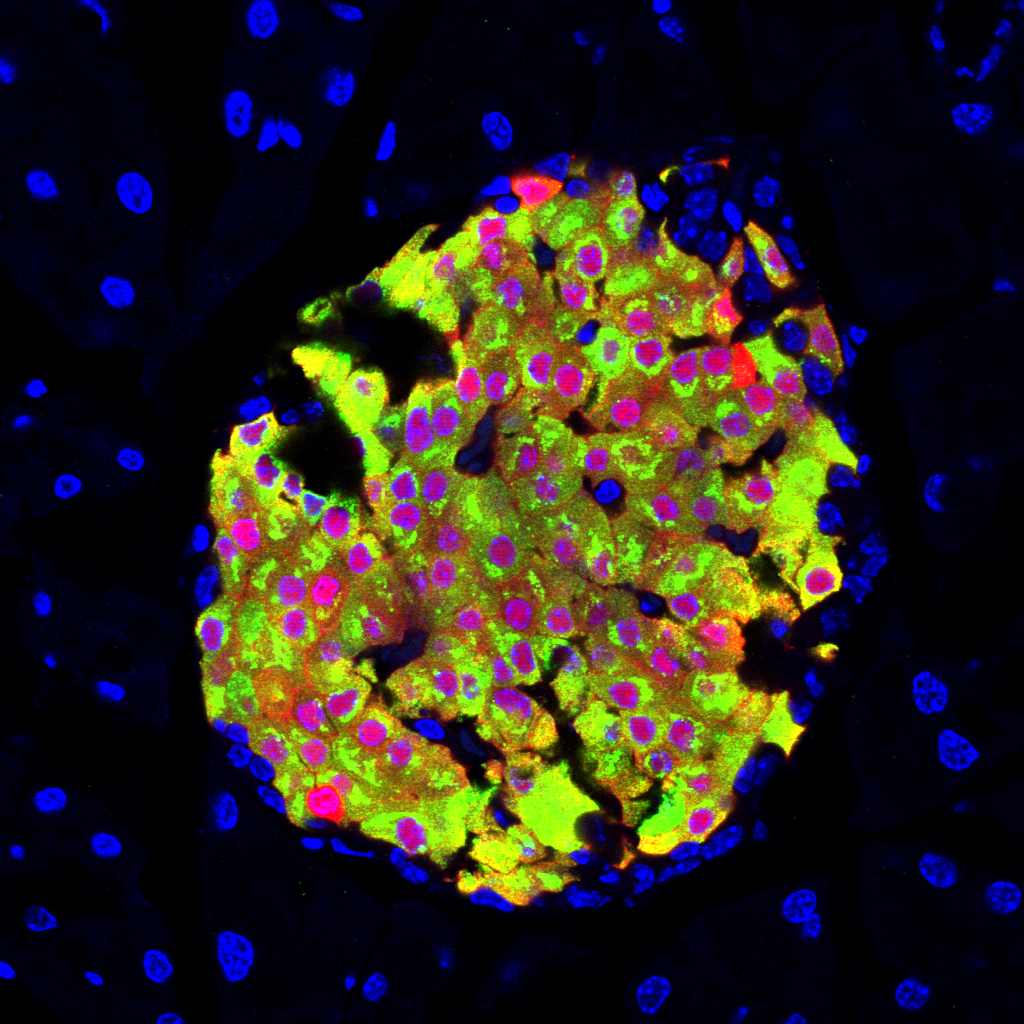

Supplement: Supplementary file 6 — Source data Fig. 4 [file 44318_2025_434_MOESM6_ESM.zip › Figure 4/4E/4E_7.tif]

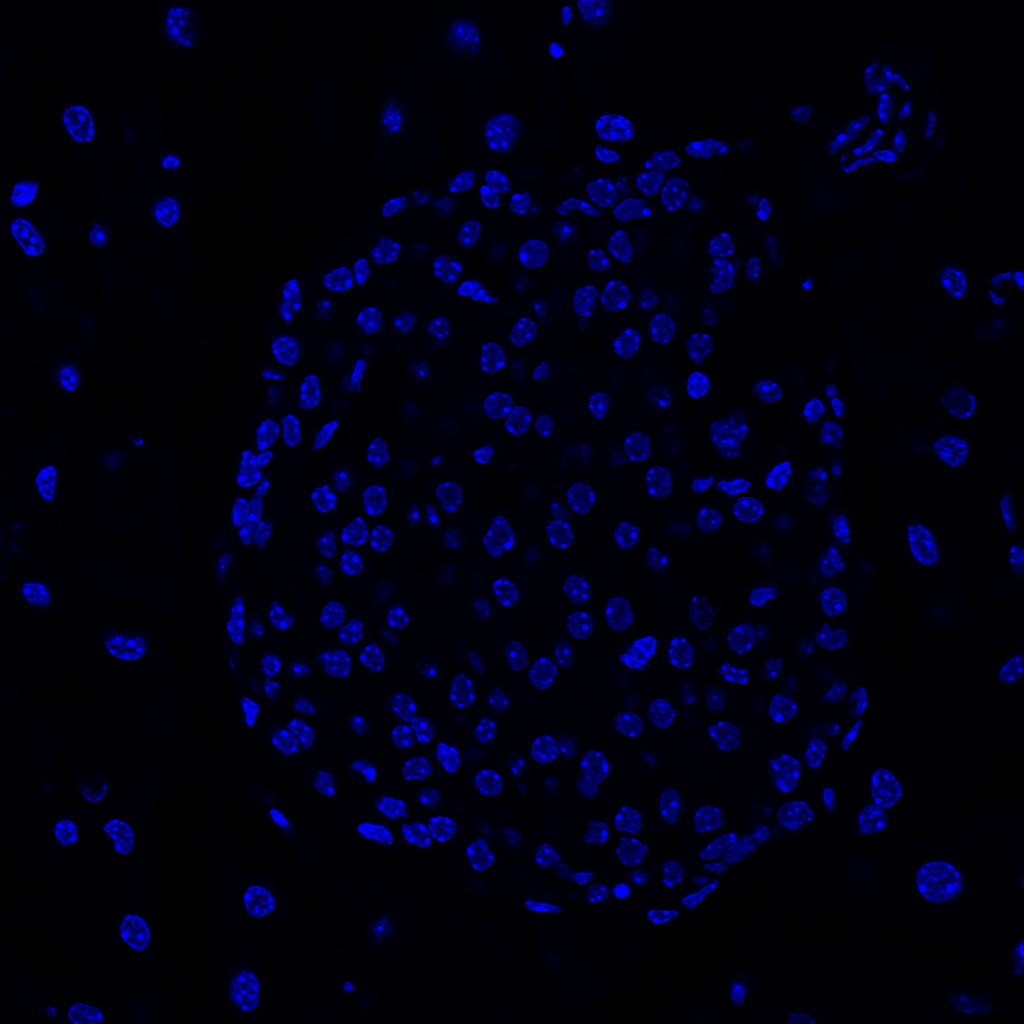

Supplement: Supplementary file 6 — Source data Fig. 4 [file 44318_2025_434_MOESM6_ESM.zip › Figure 4/4E/4E_10.tif (blue).tif]

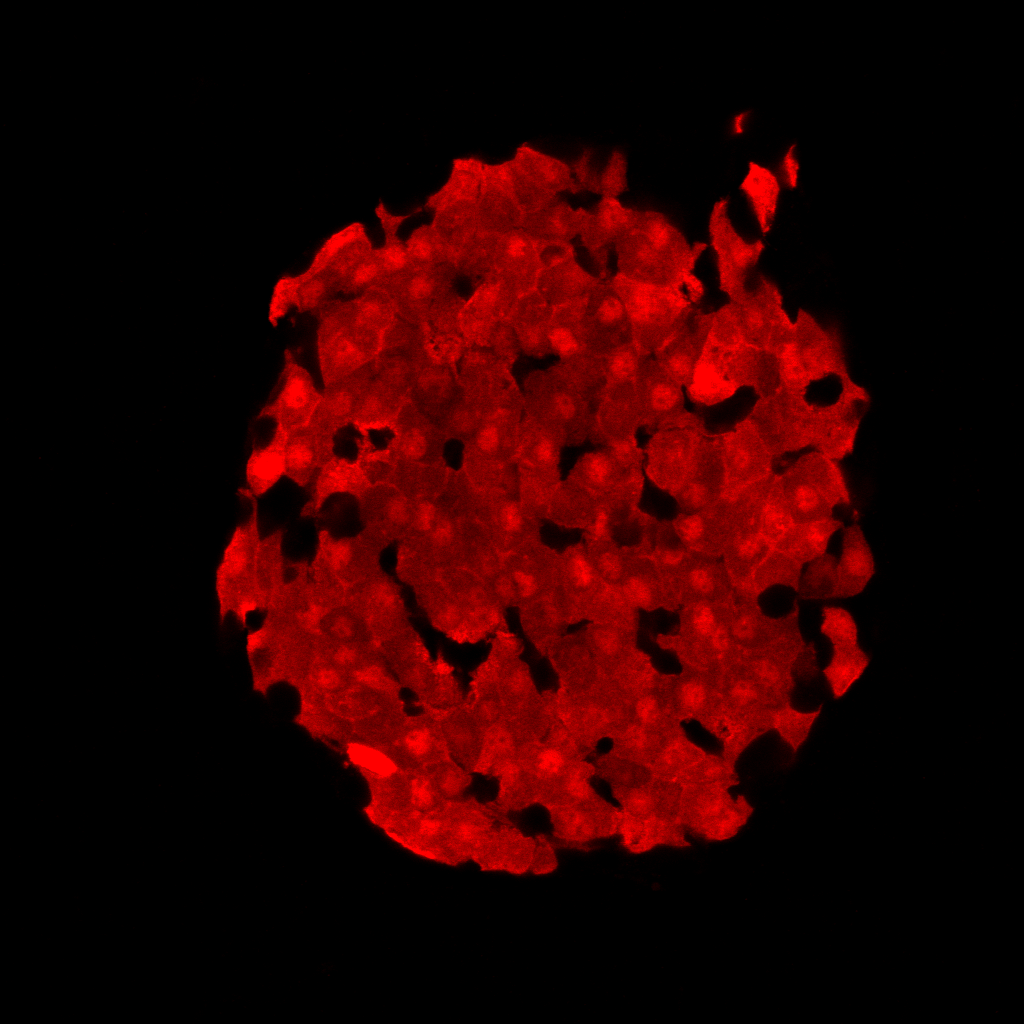

Supplement: Supplementary file 6 — Source data Fig. 4 [file 44318_2025_434_MOESM6_ESM.zip › Figure 4/4E/4E_9.tif (red).tif]

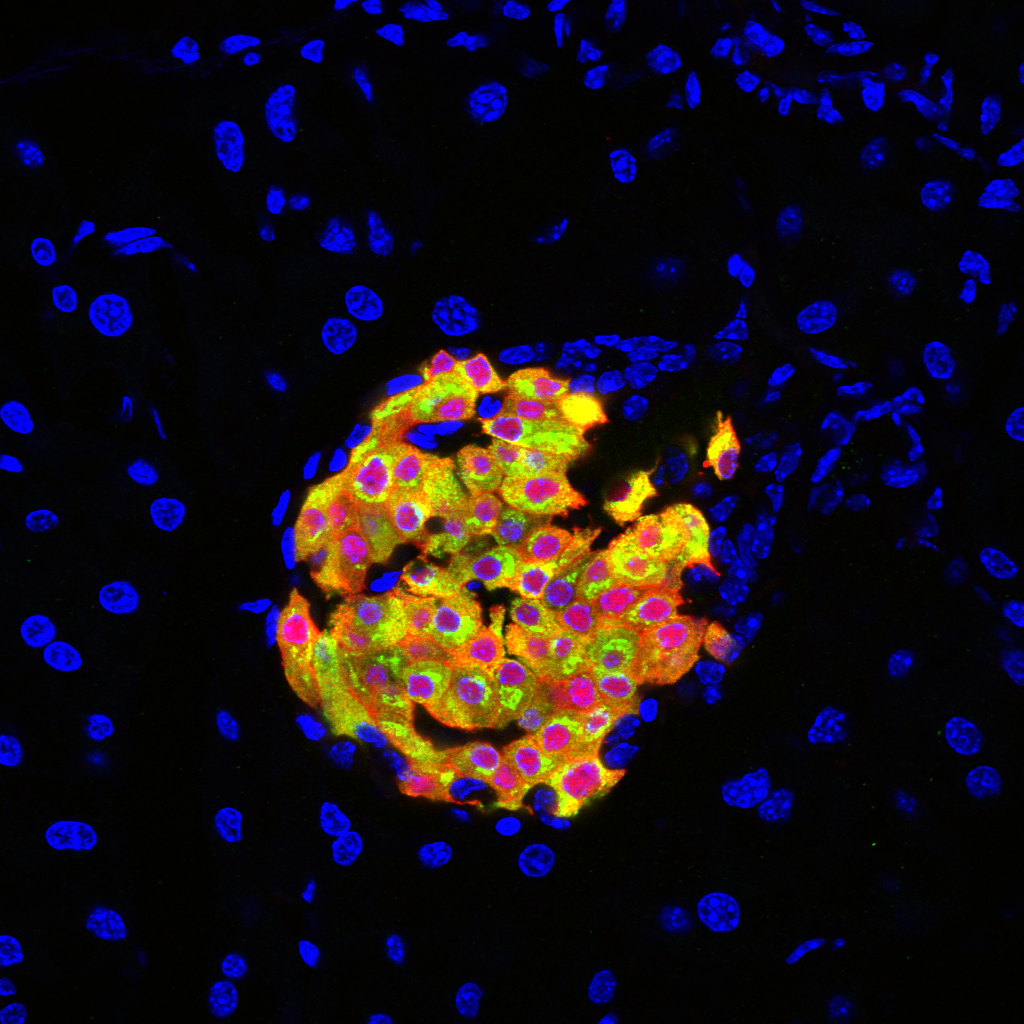

Supplement: Supplementary file 6 — Source data Fig. 4 [file 44318_2025_434_MOESM6_ESM.zip › Figure 4/4E/4E_16.tif]

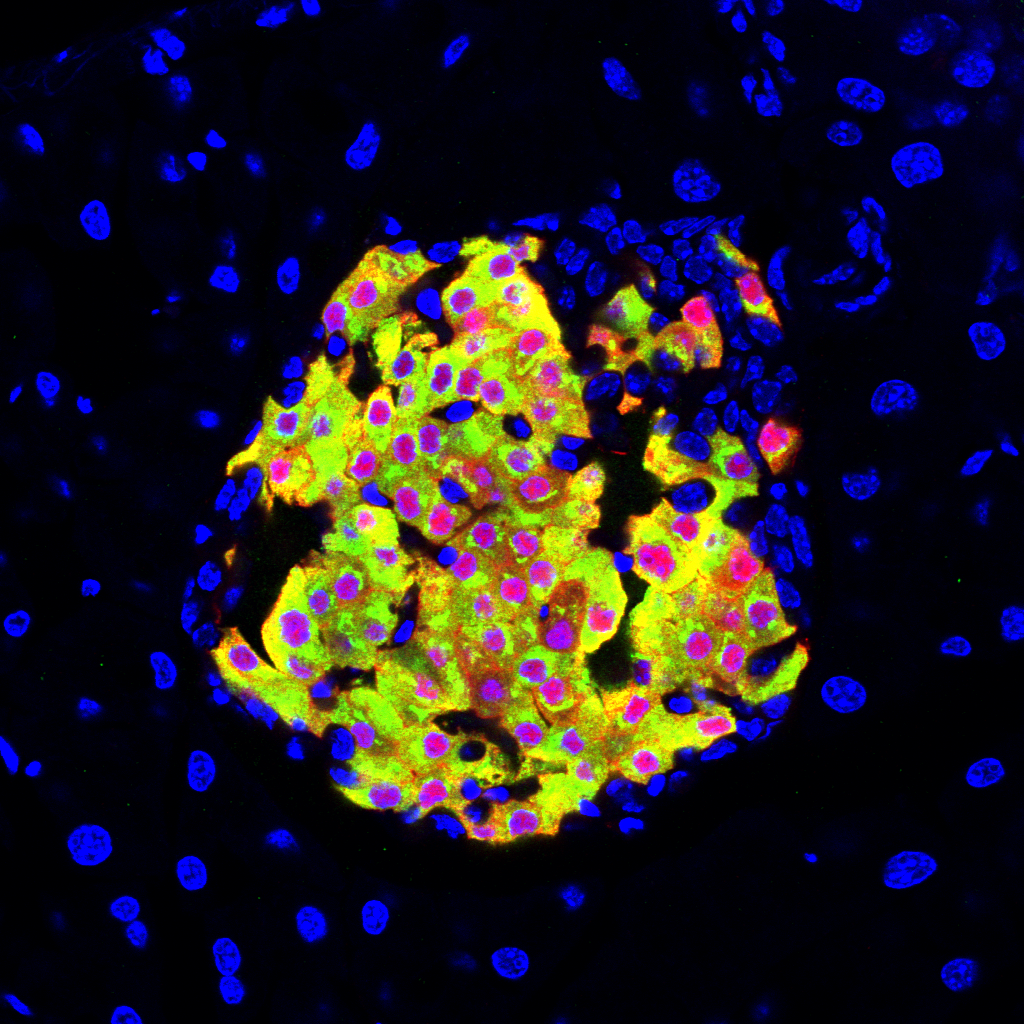

Supplement: Supplementary file 6 — Source data Fig. 4 [file 44318_2025_434_MOESM6_ESM.zip › Figure 4/4E/4E_14.tif]

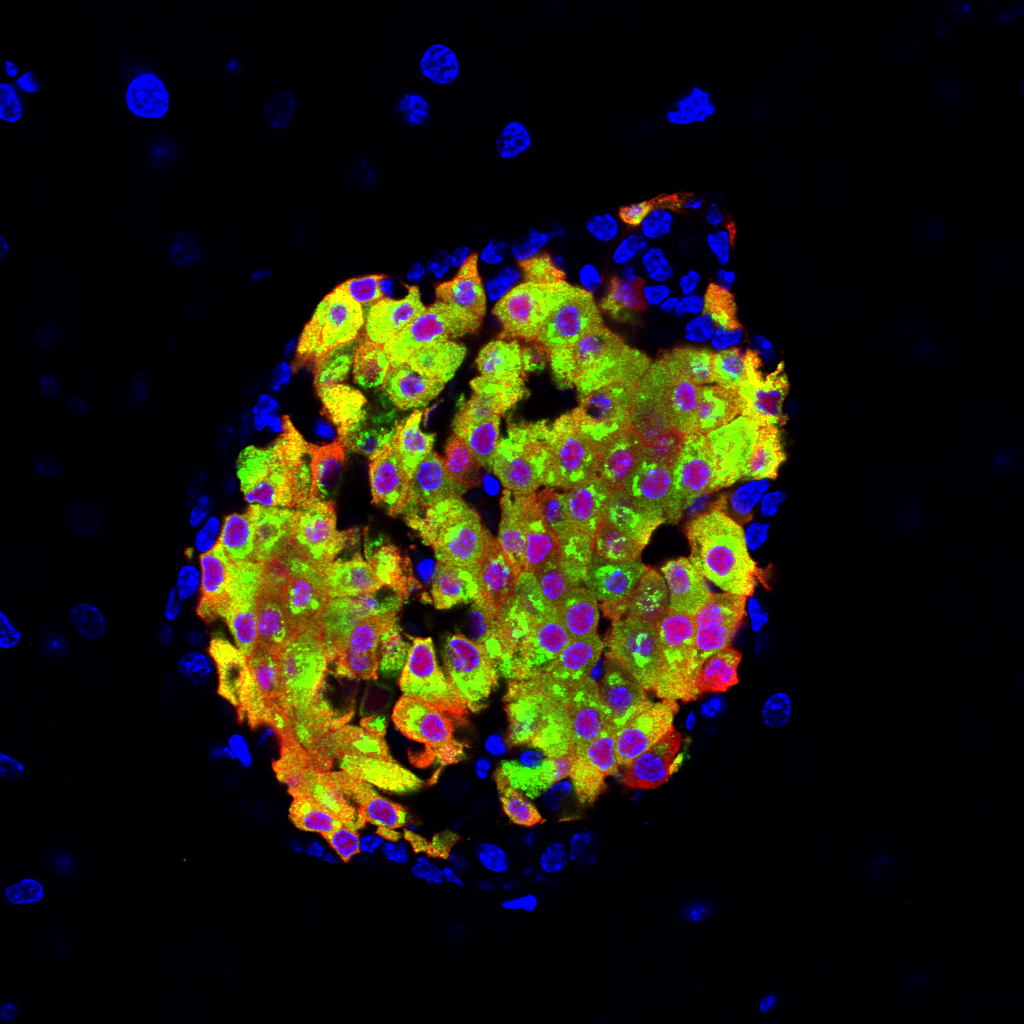

Supplement: Supplementary file 6 — Source data Fig. 4 [file 44318_2025_434_MOESM6_ESM.zip › Figure 4/4E/4E_5.tif]

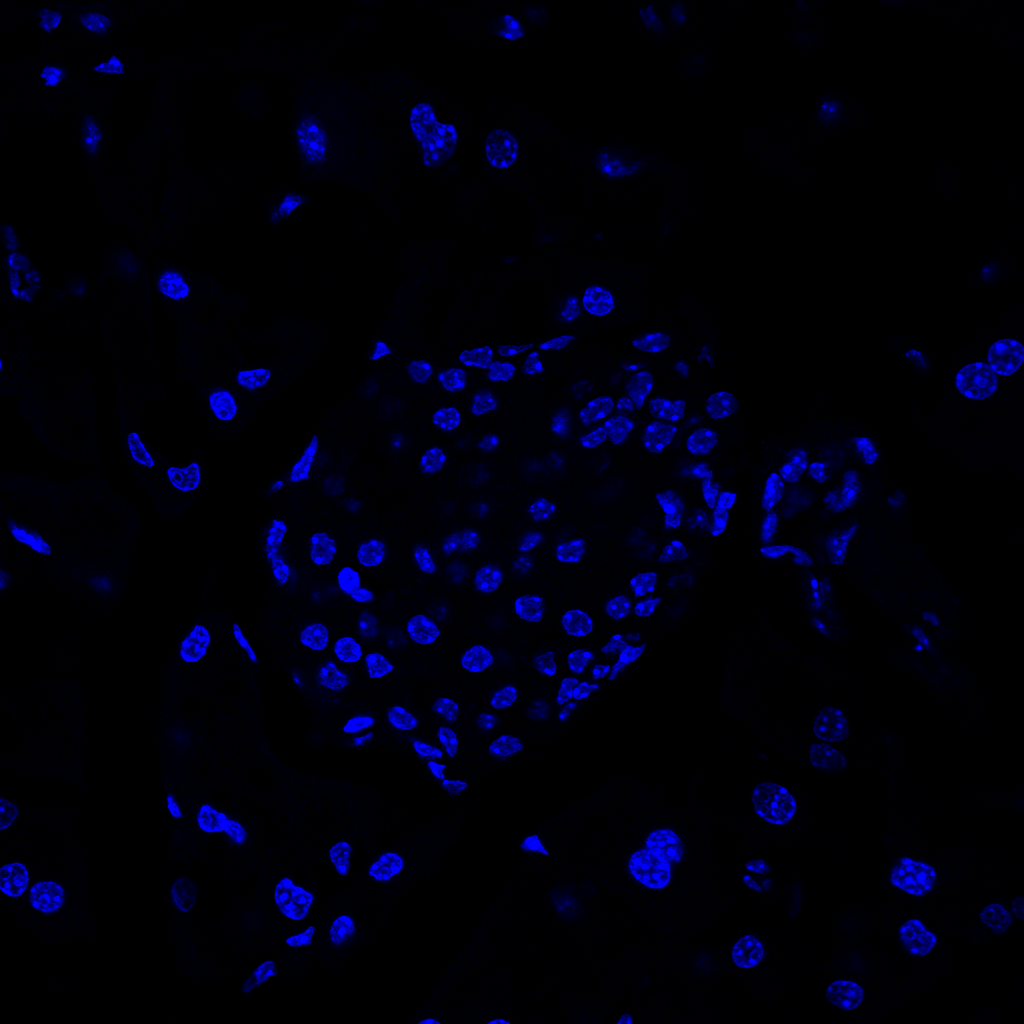

Supplement: Supplementary file 6 — Source data Fig. 4 [file 44318_2025_434_MOESM6_ESM.zip › Figure 4/4E/4E_17.tif (blue).tif]

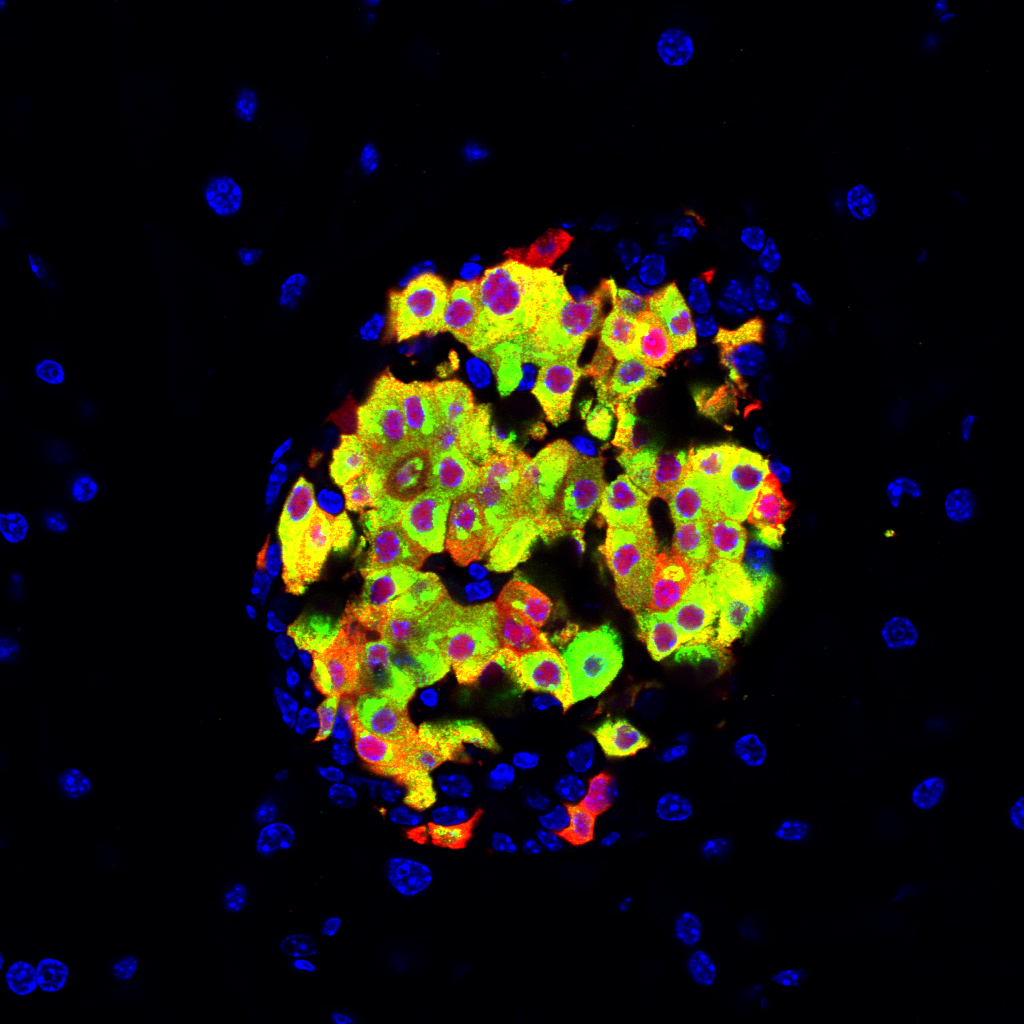

Supplement: Supplementary file 6 — Source data Fig. 4 [file 44318_2025_434_MOESM6_ESM.zip › Figure 4/4E/4E_4.tif]

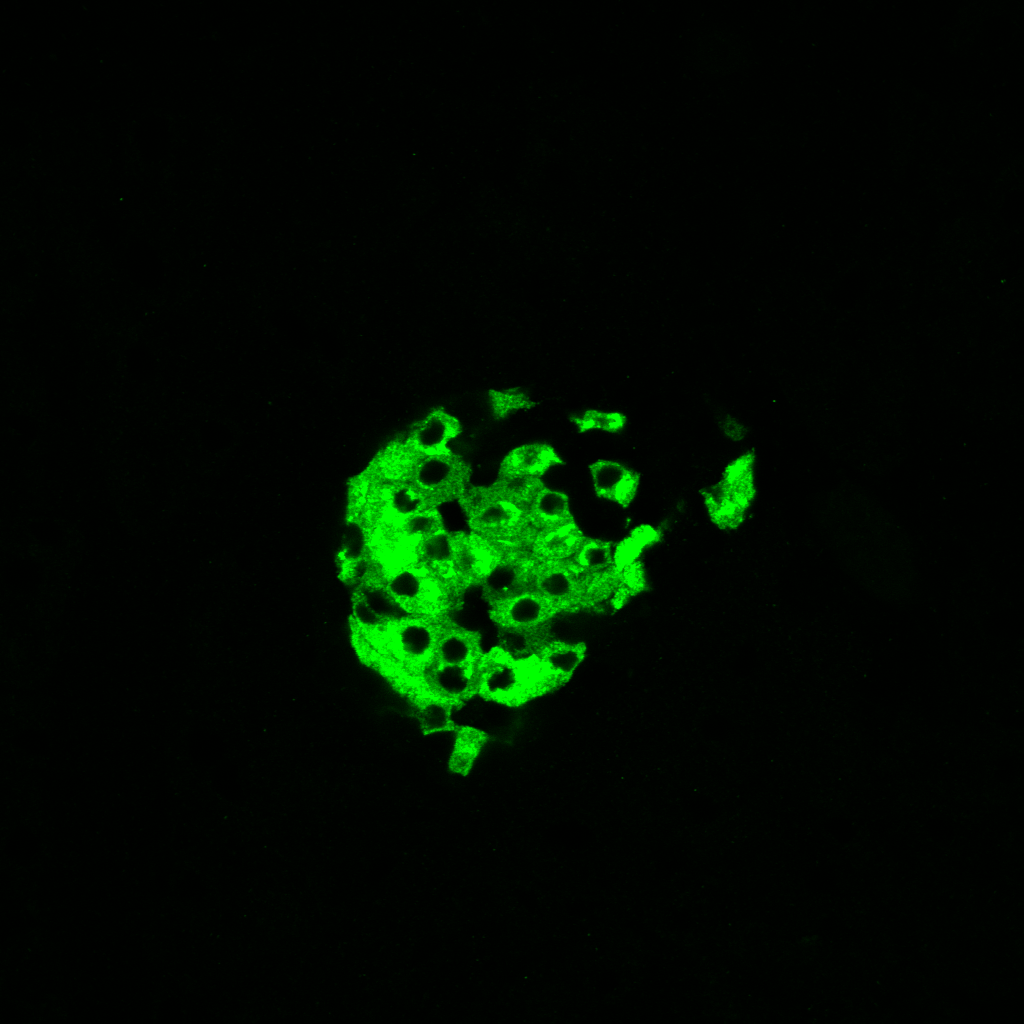

Supplement: Supplementary file 6 — Source data Fig. 4 [file 44318_2025_434_MOESM6_ESM.zip › Figure 4/4E/4E_18.tif (green).tif]

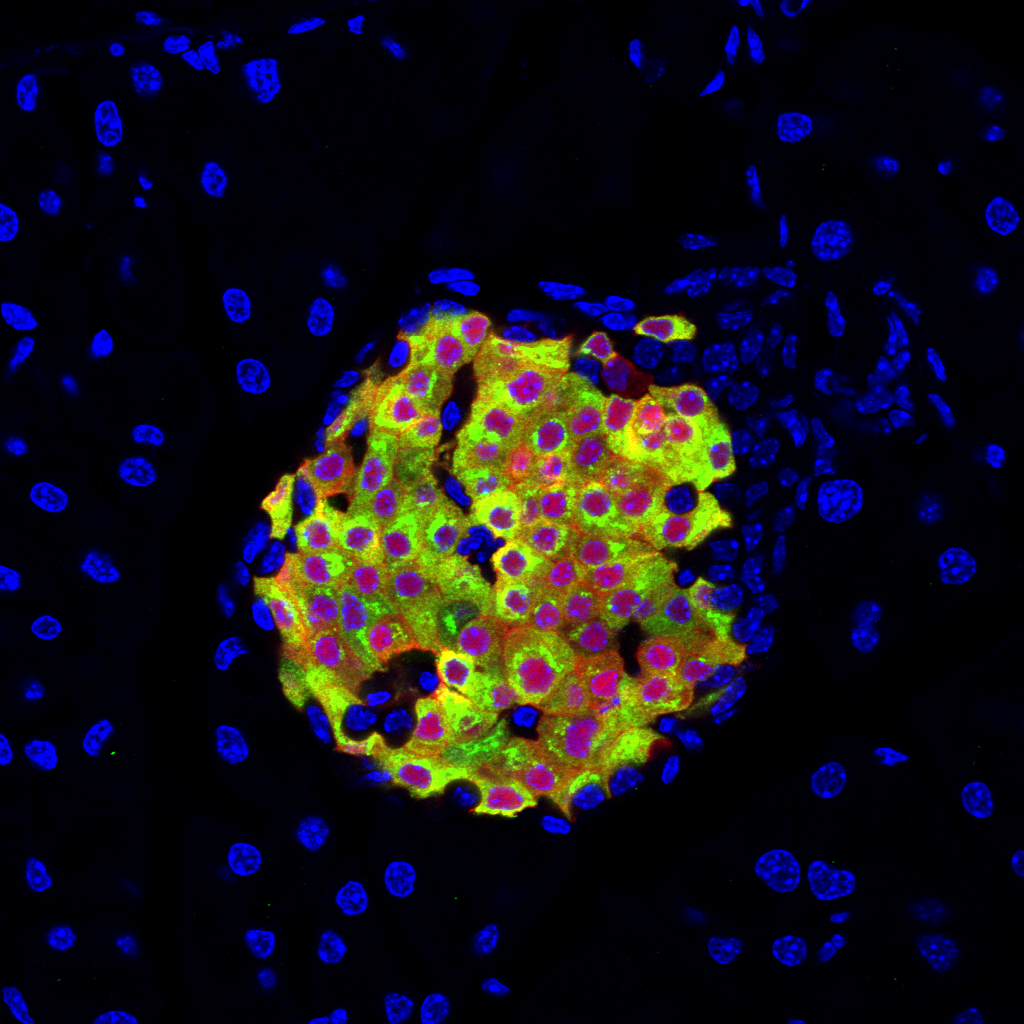

Supplement: Supplementary file 6 — Source data Fig. 4 [file 44318_2025_434_MOESM6_ESM.zip › Figure 4/4E/4E_15.tif]

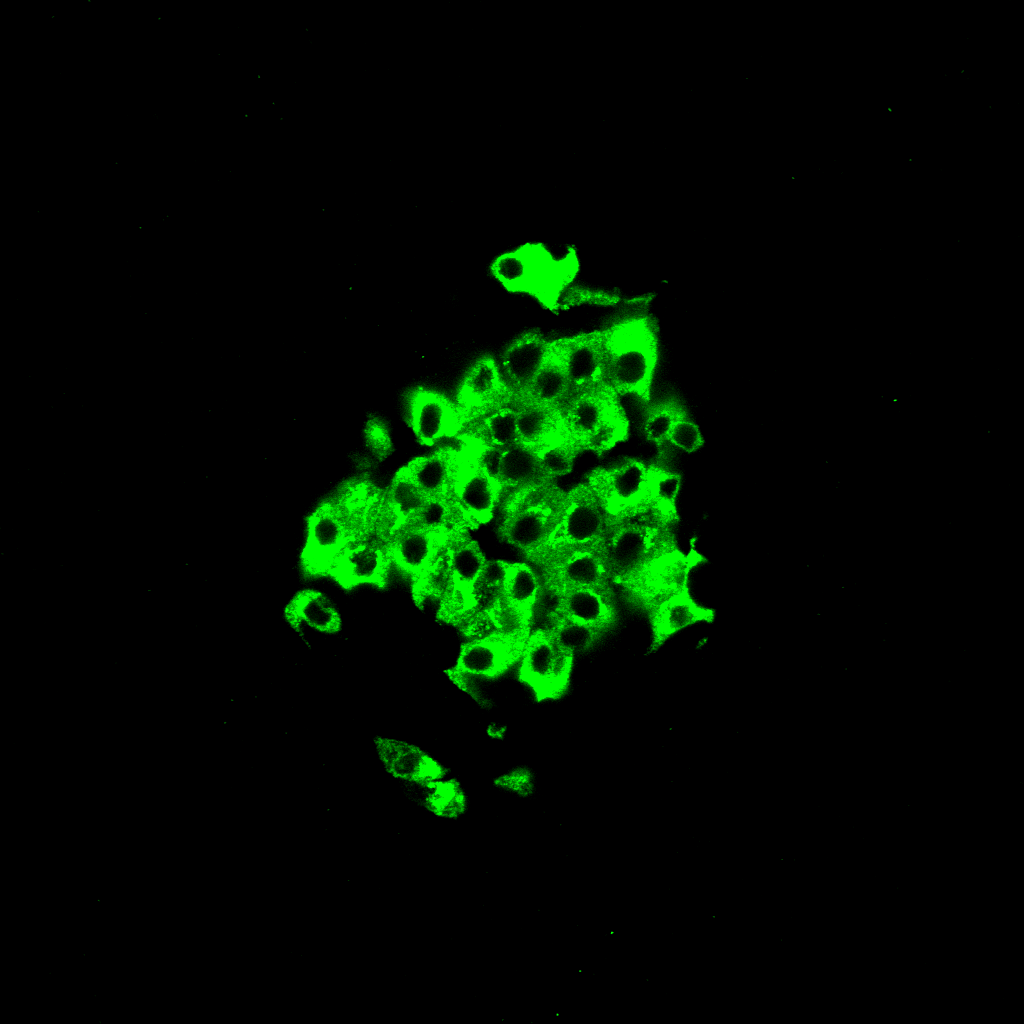

Supplement: Supplementary file 6 — Source data Fig. 4 [file 44318_2025_434_MOESM6_ESM.zip › Figure 4/4E/4E_3.tif (green).tif]

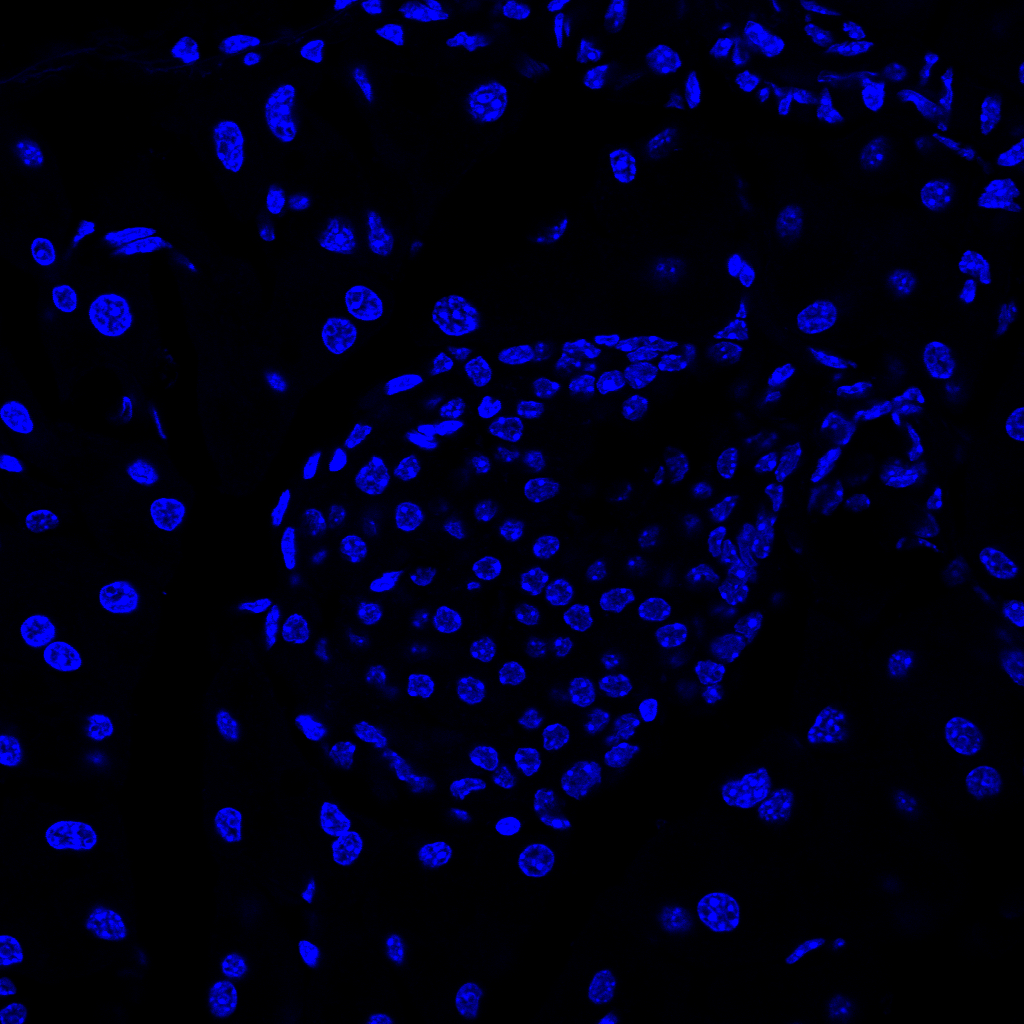

Supplement: Supplementary file 6 — Source data Fig. 4 [file 44318_2025_434_MOESM6_ESM.zip › Figure 4/4E/4E_16.tif (blue).tif]

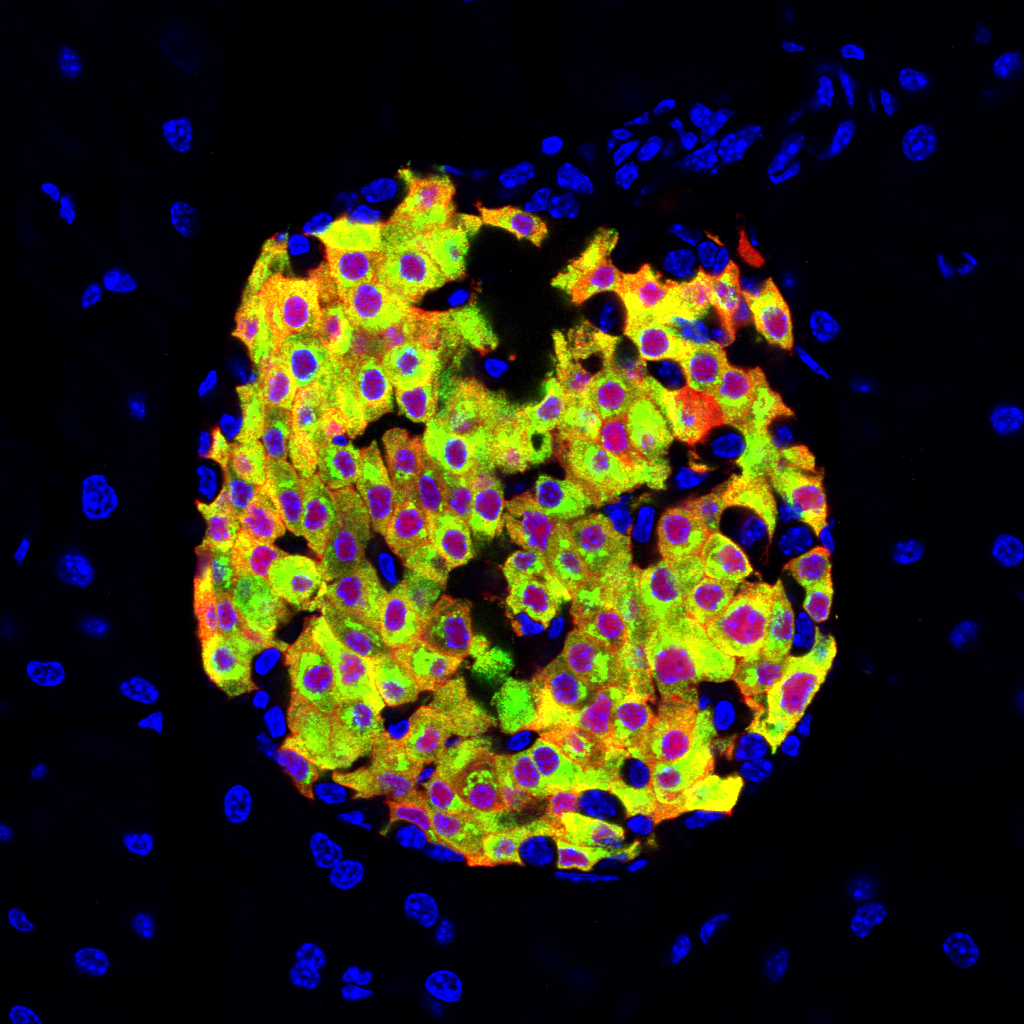

Supplement: Supplementary file 6 — Source data Fig. 4 [file 44318_2025_434_MOESM6_ESM.zip › Figure 4/4E/4E_11.tif]

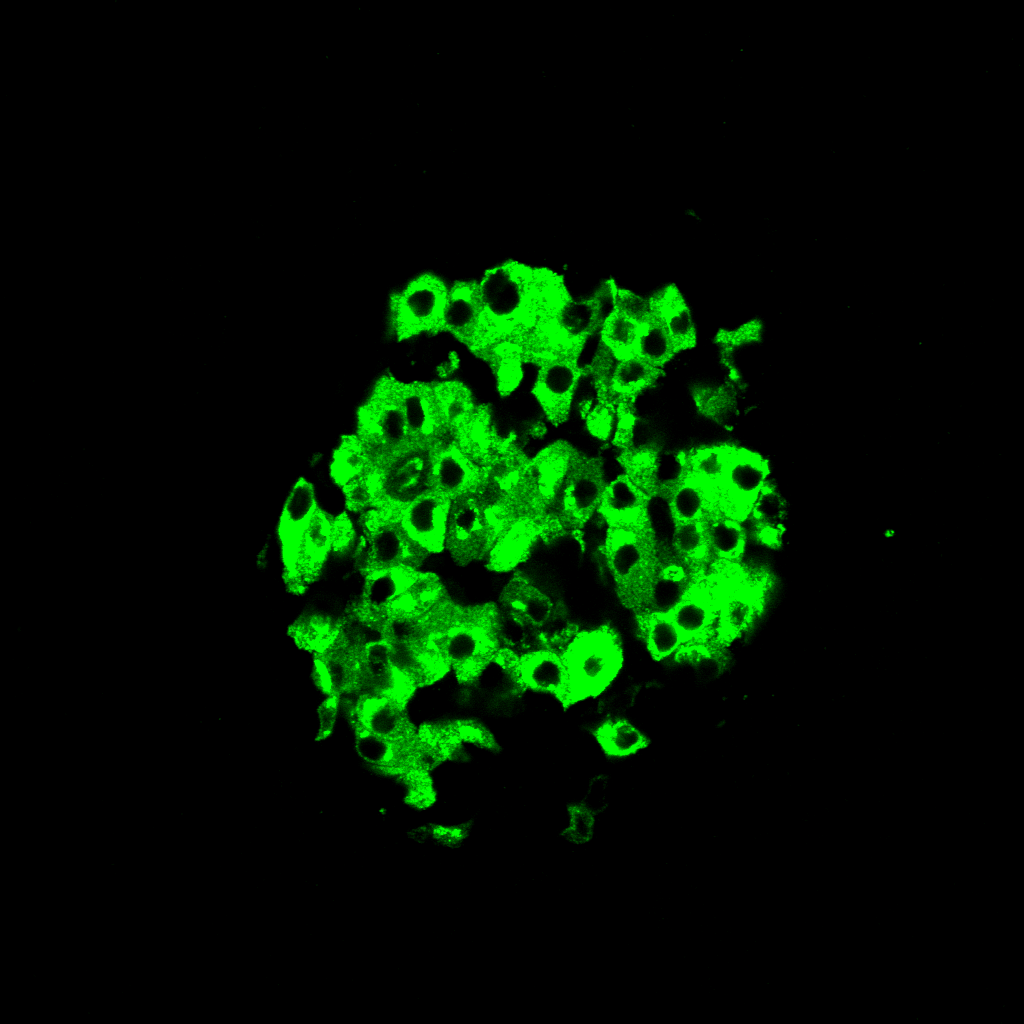

Supplement: Supplementary file 6 — Source data Fig. 4 [file 44318_2025_434_MOESM6_ESM.zip › Figure 4/4E/4E_4.tif (green).tif]

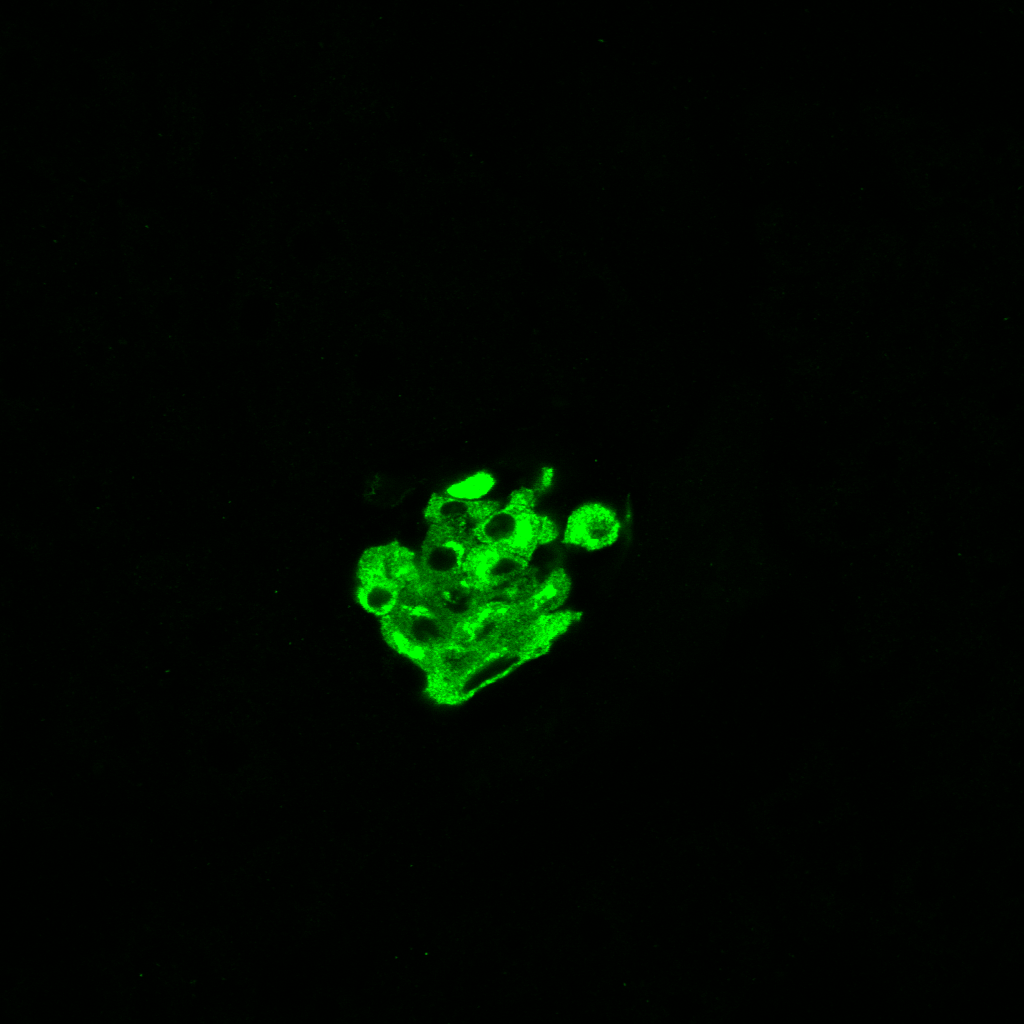

Supplement: Supplementary file 6 — Source data Fig. 4 [file 44318_2025_434_MOESM6_ESM.zip › Figure 4/4E/4E_22.tif (green).tif]

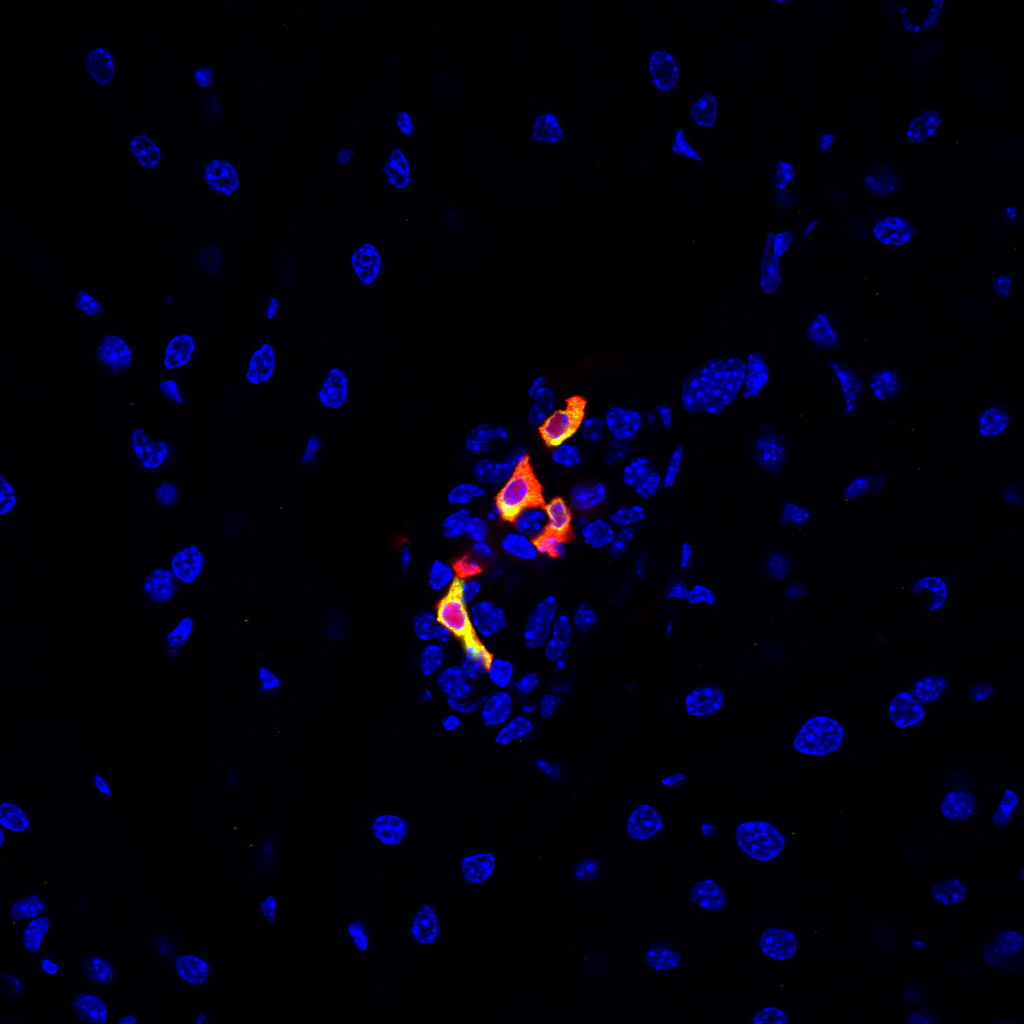

Supplement: Supplementary file 6 — Source data Fig. 4 [file 44318_2025_434_MOESM6_ESM.zip › Figure 4/4E/4E_1.tif]

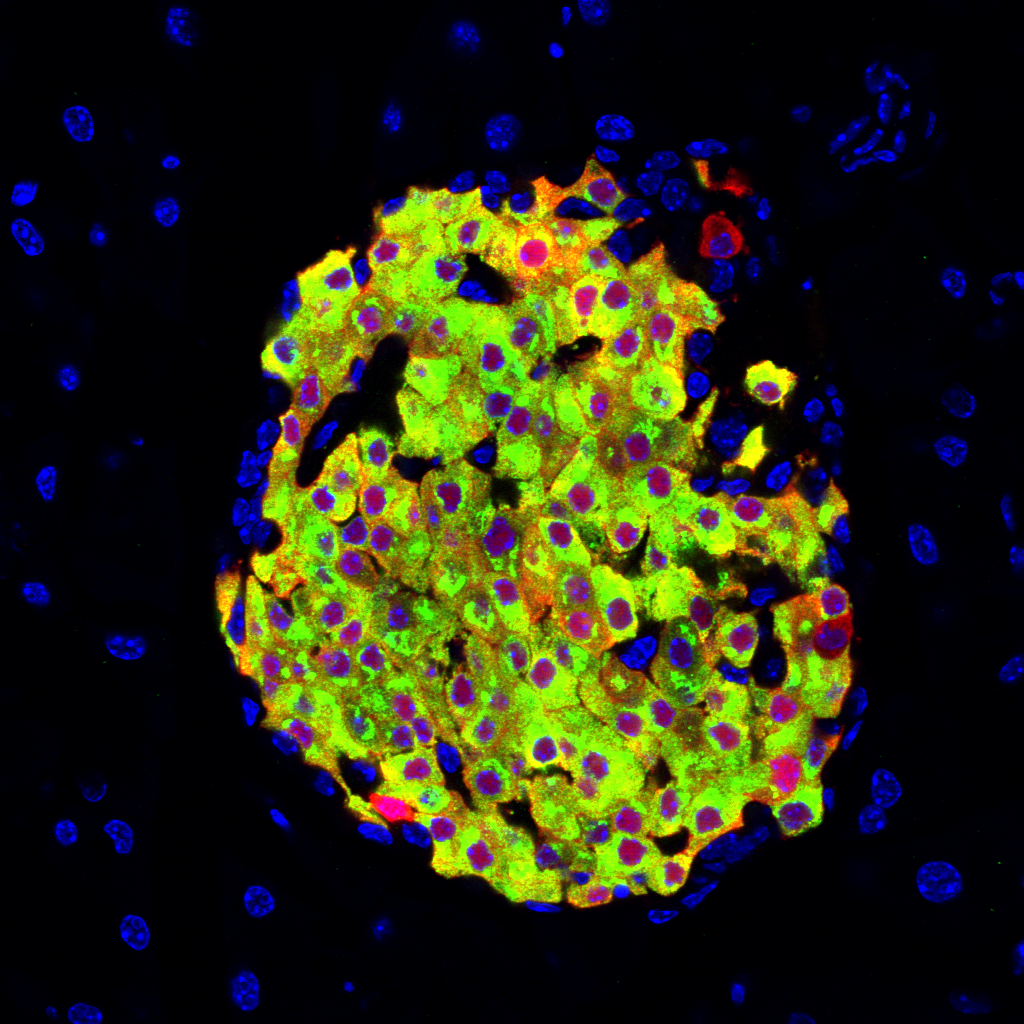

Supplement: Supplementary file 6 — Source data Fig. 4 [file 44318_2025_434_MOESM6_ESM.zip › Figure 4/4E/4E_10.tif]

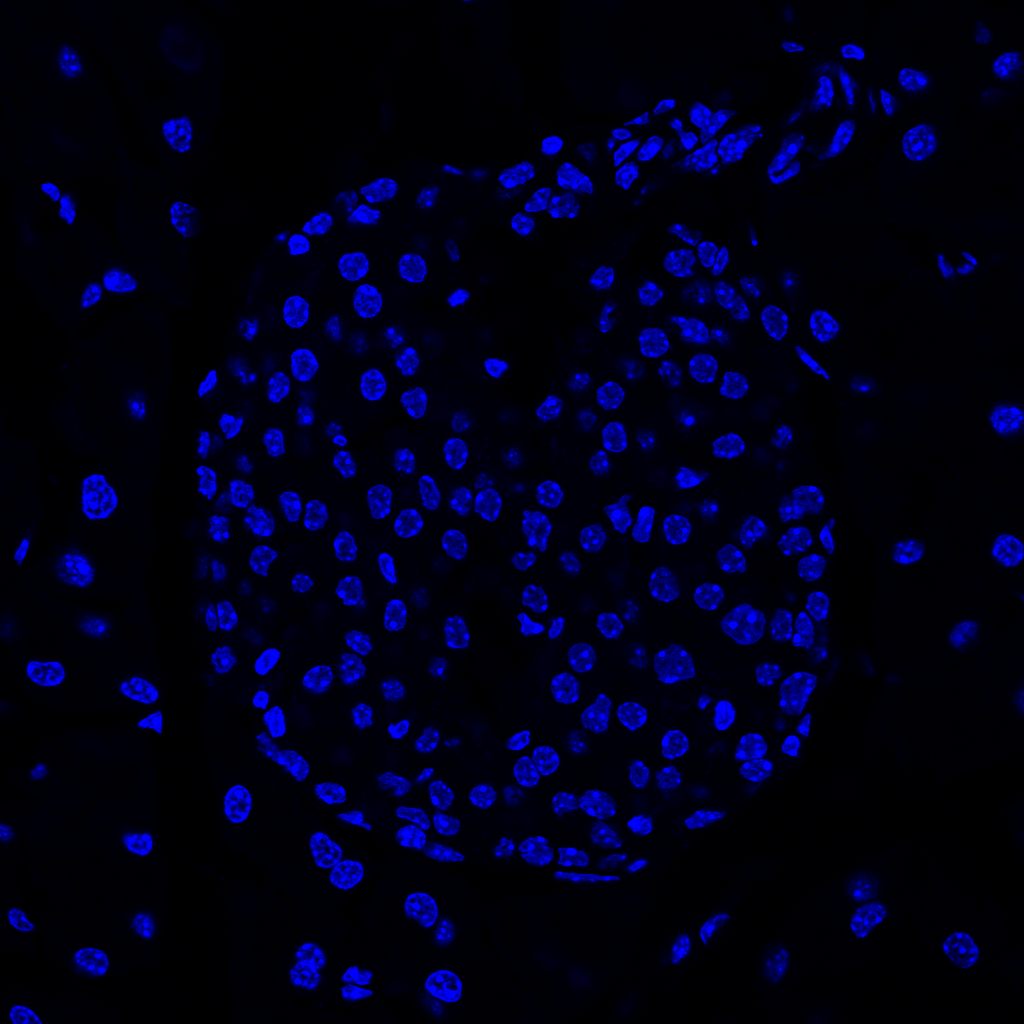

Supplement: Supplementary file 6 — Source data Fig. 4 [file 44318_2025_434_MOESM6_ESM.zip › Figure 4/4E/4E_11.tif (blue).tif]

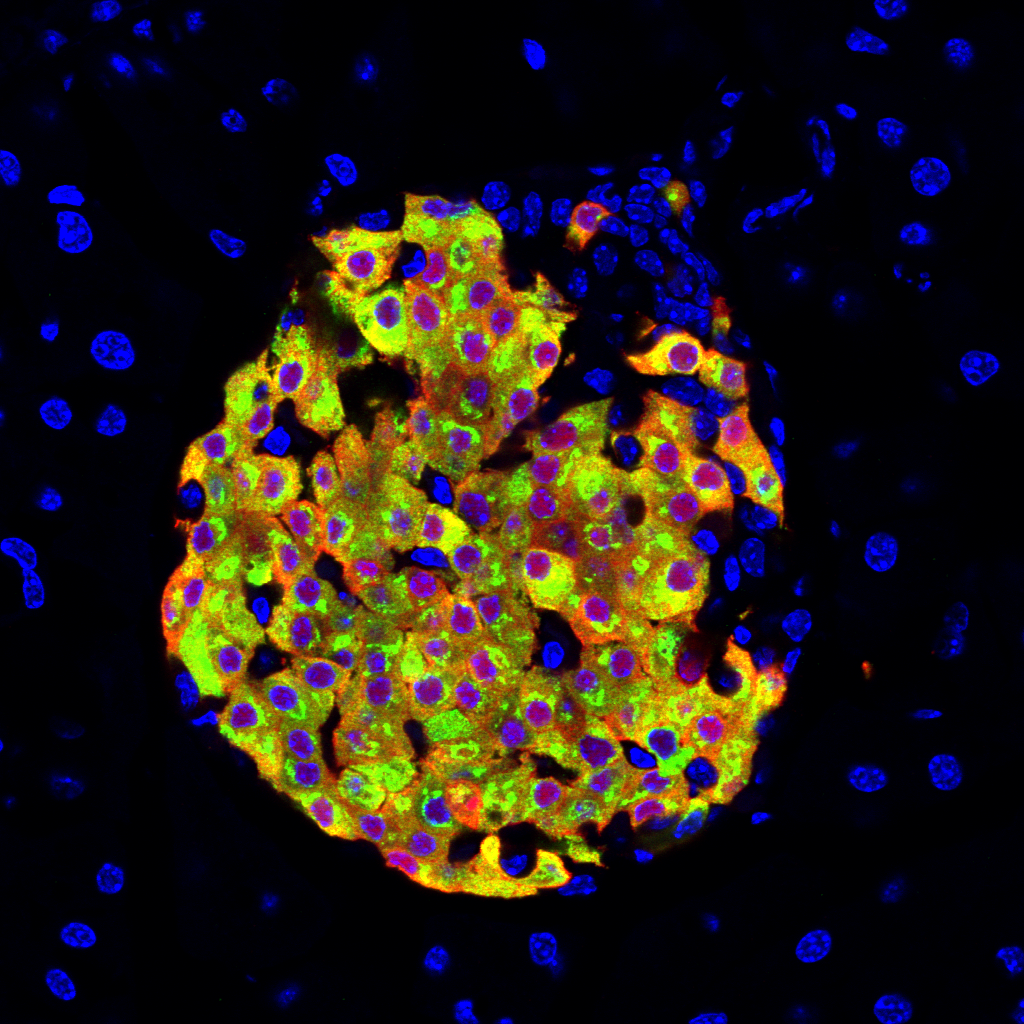

Supplement: Supplementary file 6 — Source data Fig. 4 [file 44318_2025_434_MOESM6_ESM.zip › Figure 4/4E/4E_12.tif]

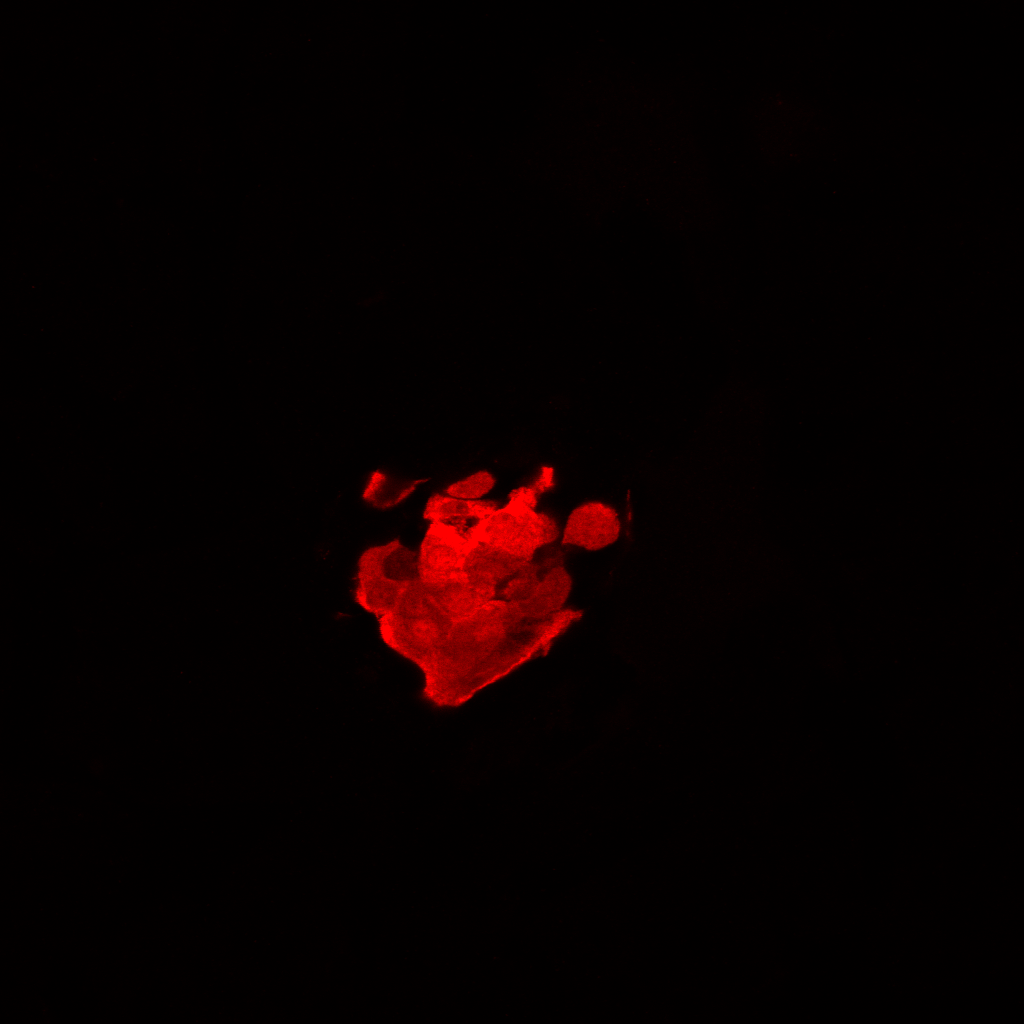

Supplement: Supplementary file 6 — Source data Fig. 4 [file 44318_2025_434_MOESM6_ESM.zip › Figure 4/4E/4E_22.tif (red).tif]

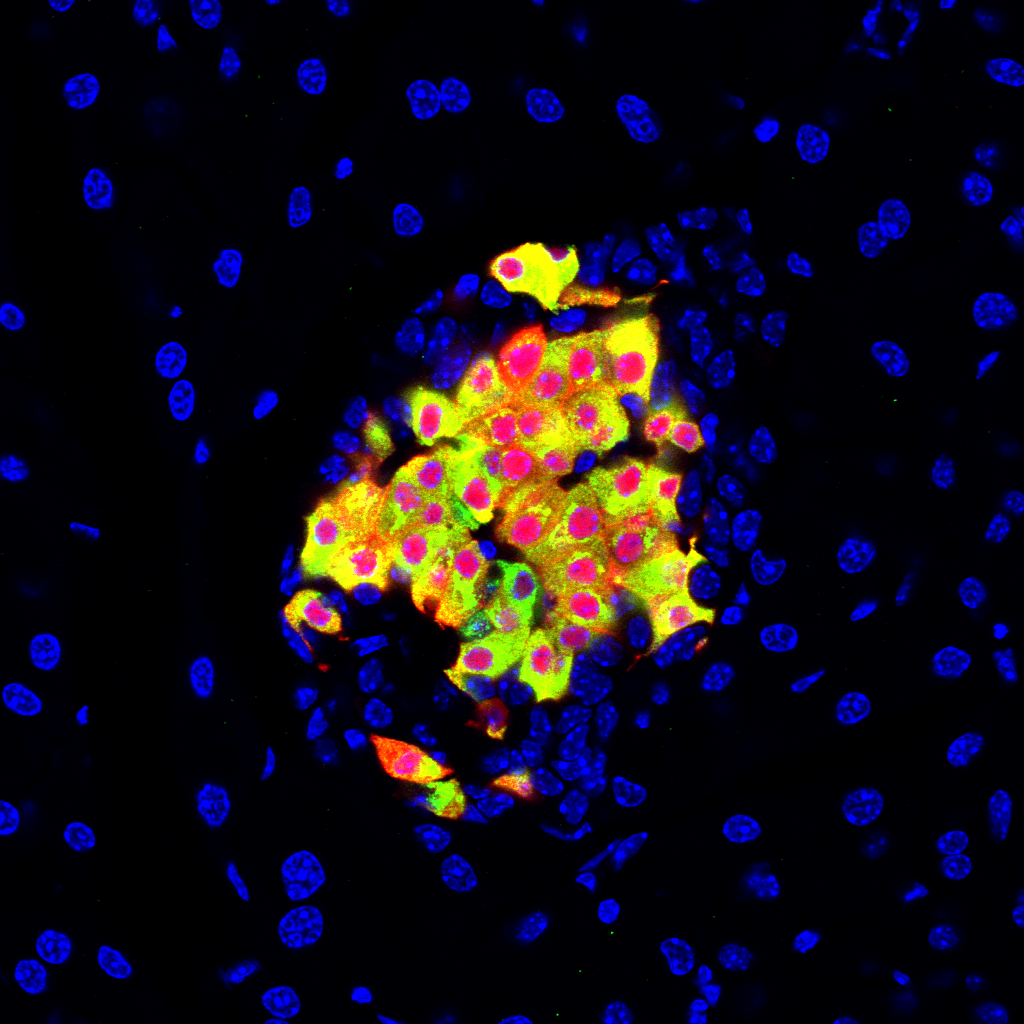

Supplement: Supplementary file 6 — Source data Fig. 4 [file 44318_2025_434_MOESM6_ESM.zip › Figure 4/4E/4E_3.tif]

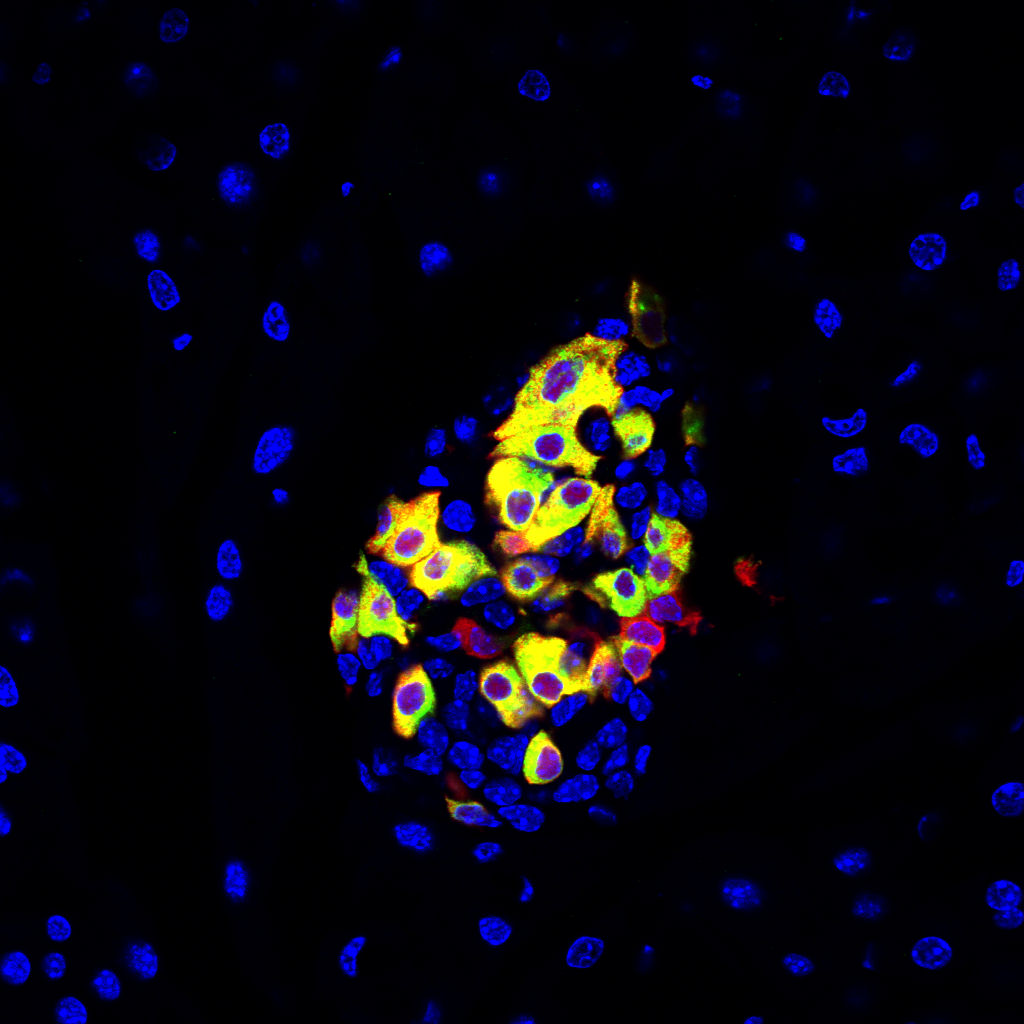

Supplement: Supplementary file 6 — Source data Fig. 4 [file 44318_2025_434_MOESM6_ESM.zip › Figure 4/4E/4E_2.tif]

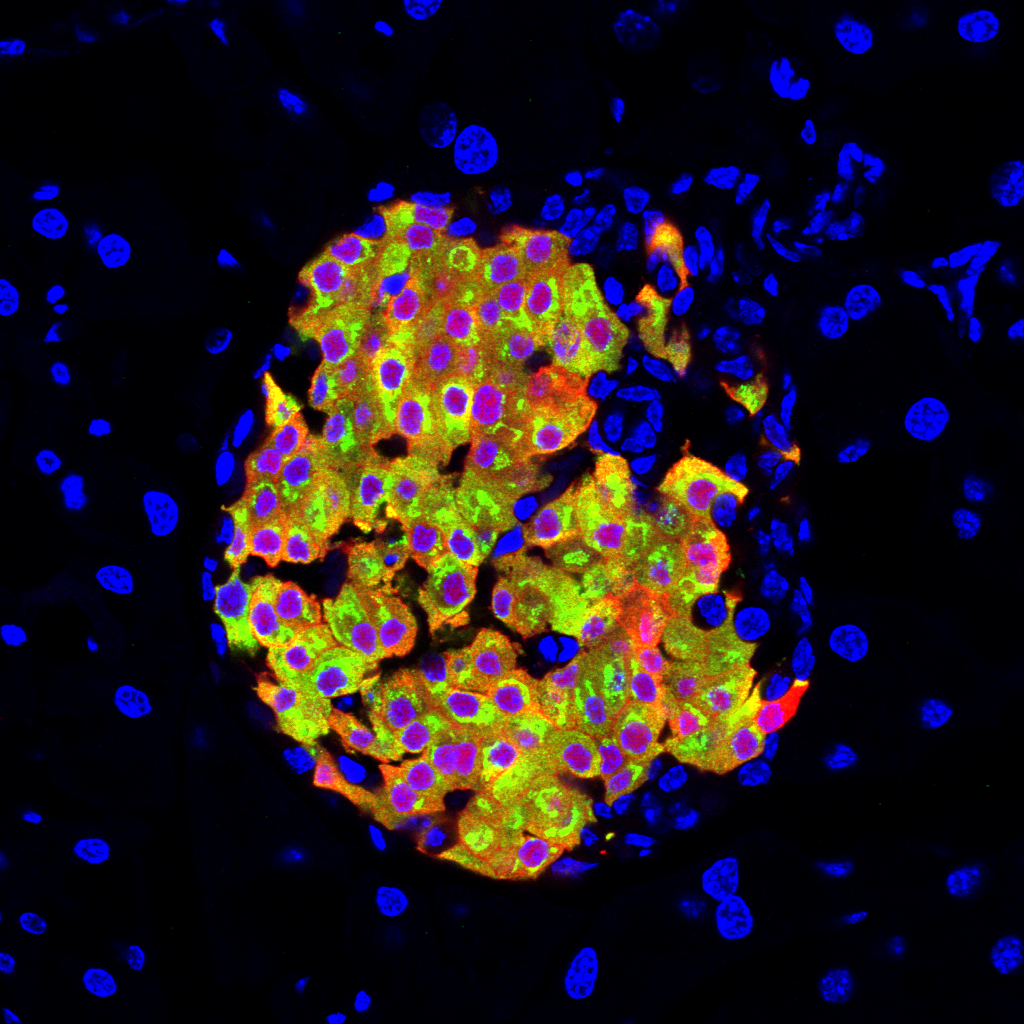

Supplement: Supplementary file 6 — Source data Fig. 4 [file 44318_2025_434_MOESM6_ESM.zip › Figure 4/4E/4E_13.tif]

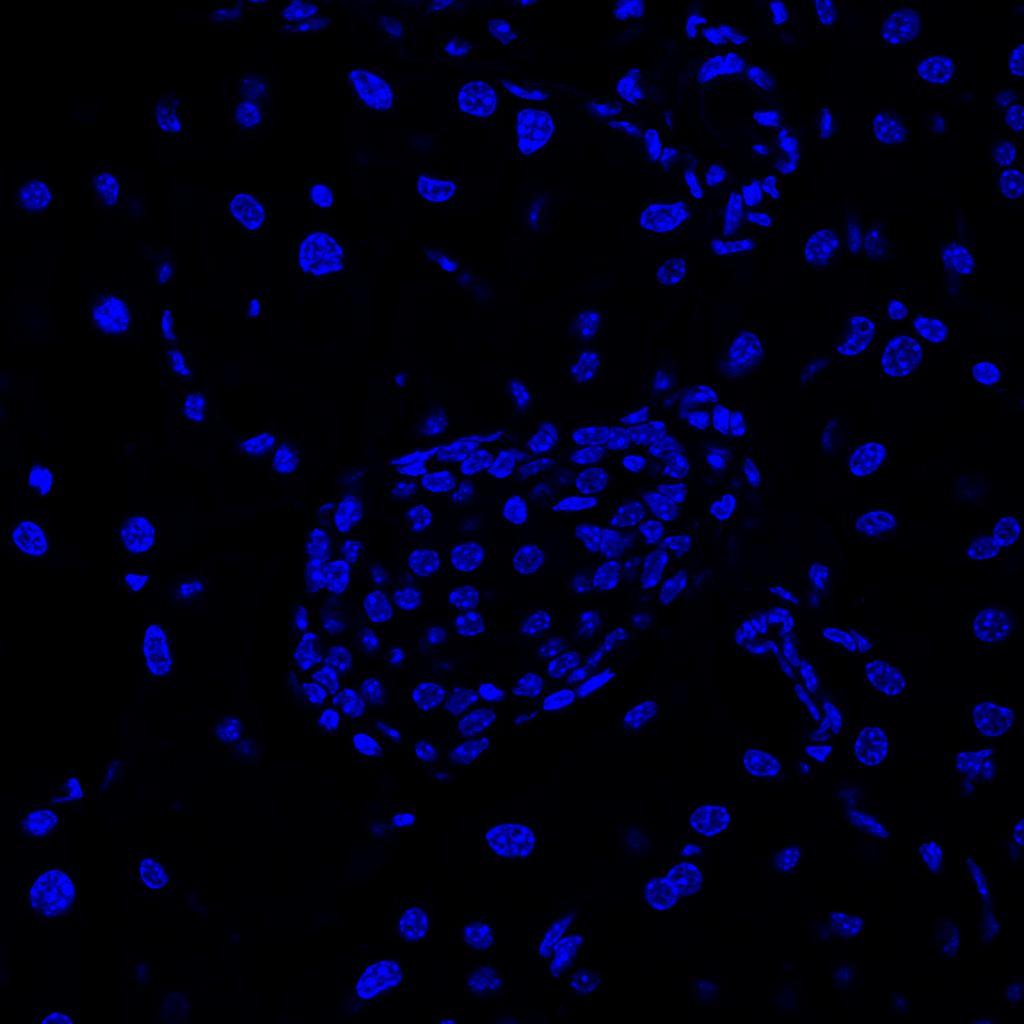

Supplement: Supplementary file 6 — Source data Fig. 4 [file 44318_2025_434_MOESM6_ESM.zip › Figure 4/4E/4E_19.tif (blue).tif]

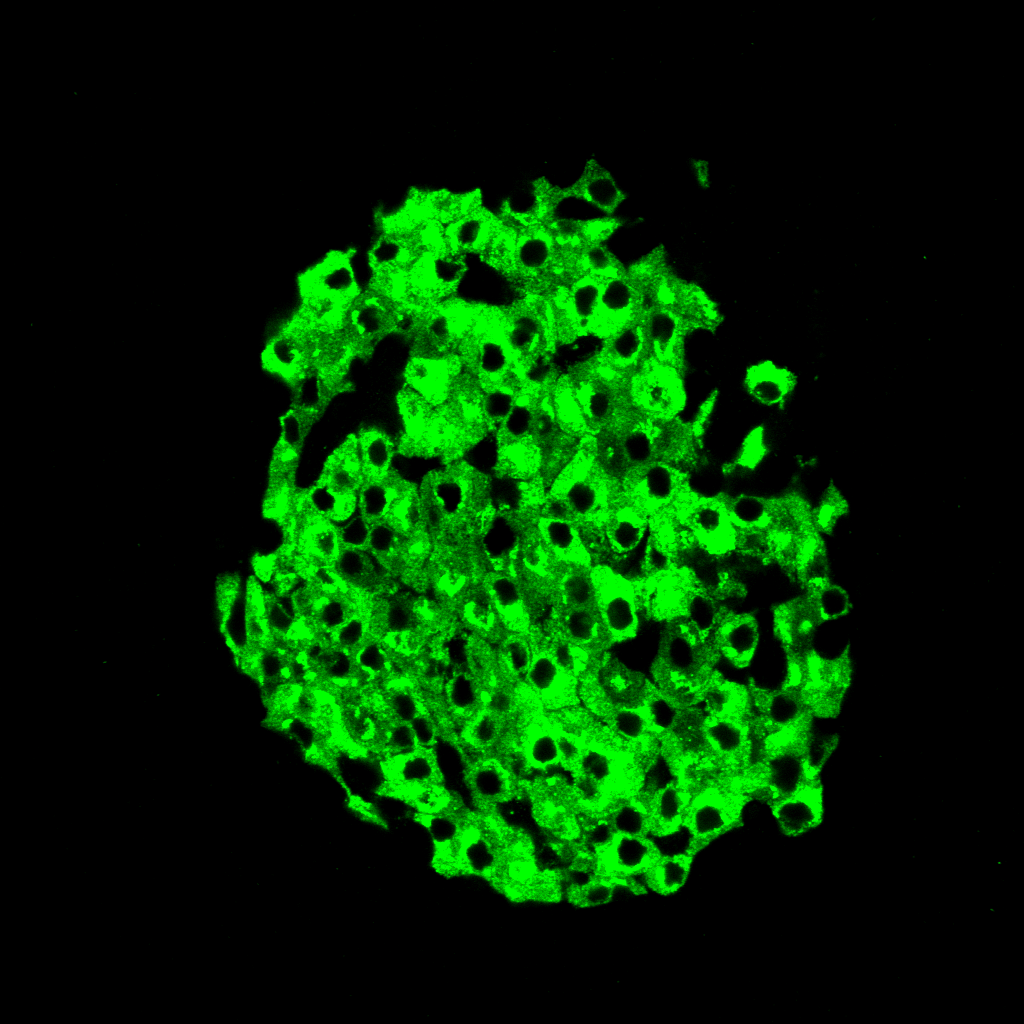

Supplement: Supplementary file 6 — Source data Fig. 4 [file 44318_2025_434_MOESM6_ESM.zip › Figure 4/4E/4E_10.tif (green).tif]

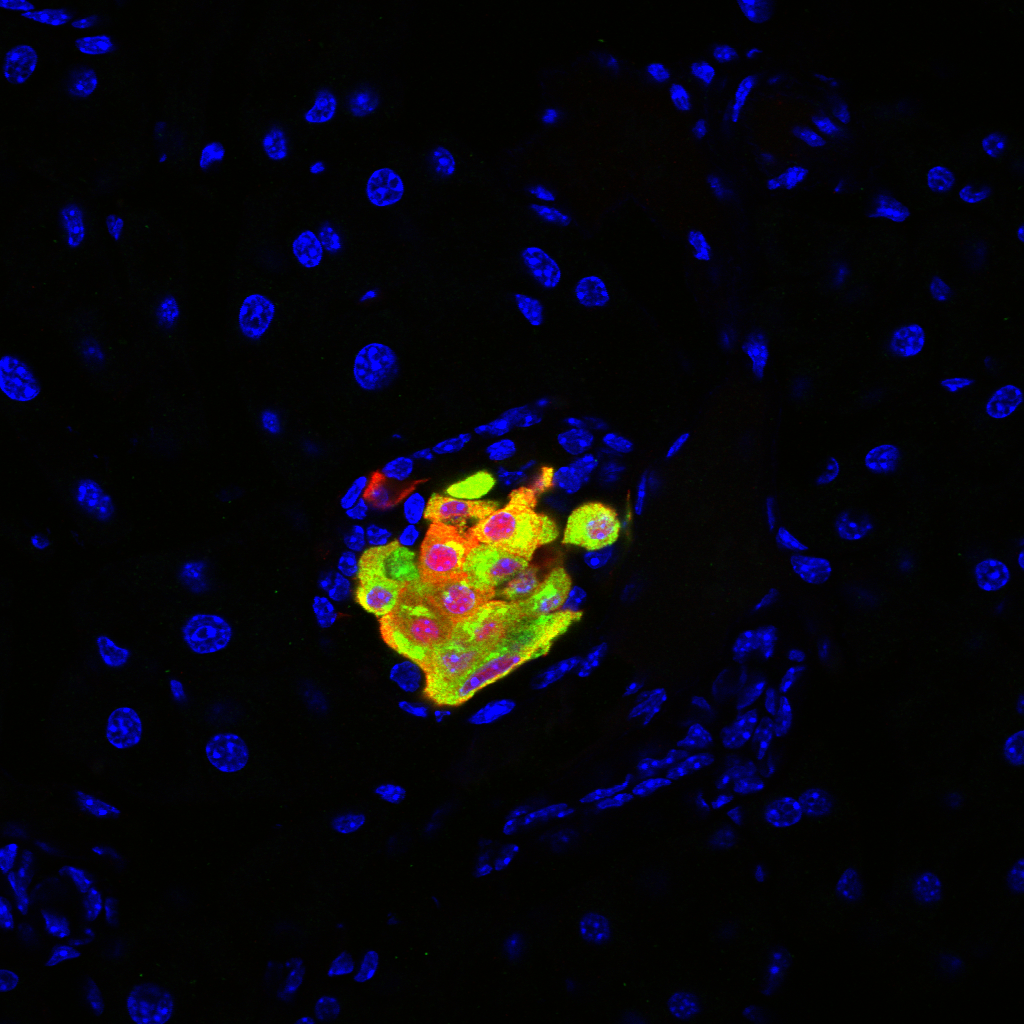

Supplement: Supplementary file 6 — Source data Fig. 4 [file 44318_2025_434_MOESM6_ESM.zip › Figure 4/4E/4E_22.tif]

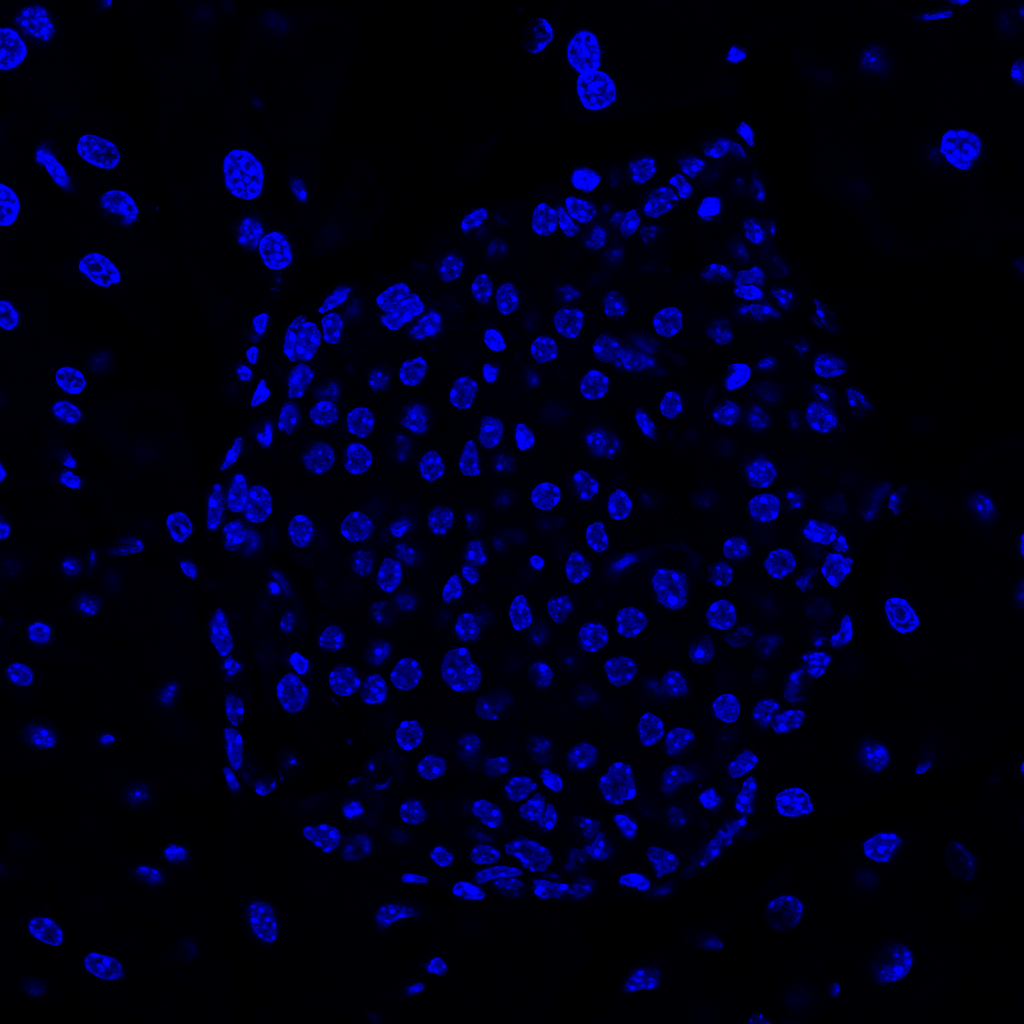

Supplement: Supplementary file 6 — Source data Fig. 4 [file 44318_2025_434_MOESM6_ESM.zip › Figure 4/4E/4E_6.tif (blue).tif]

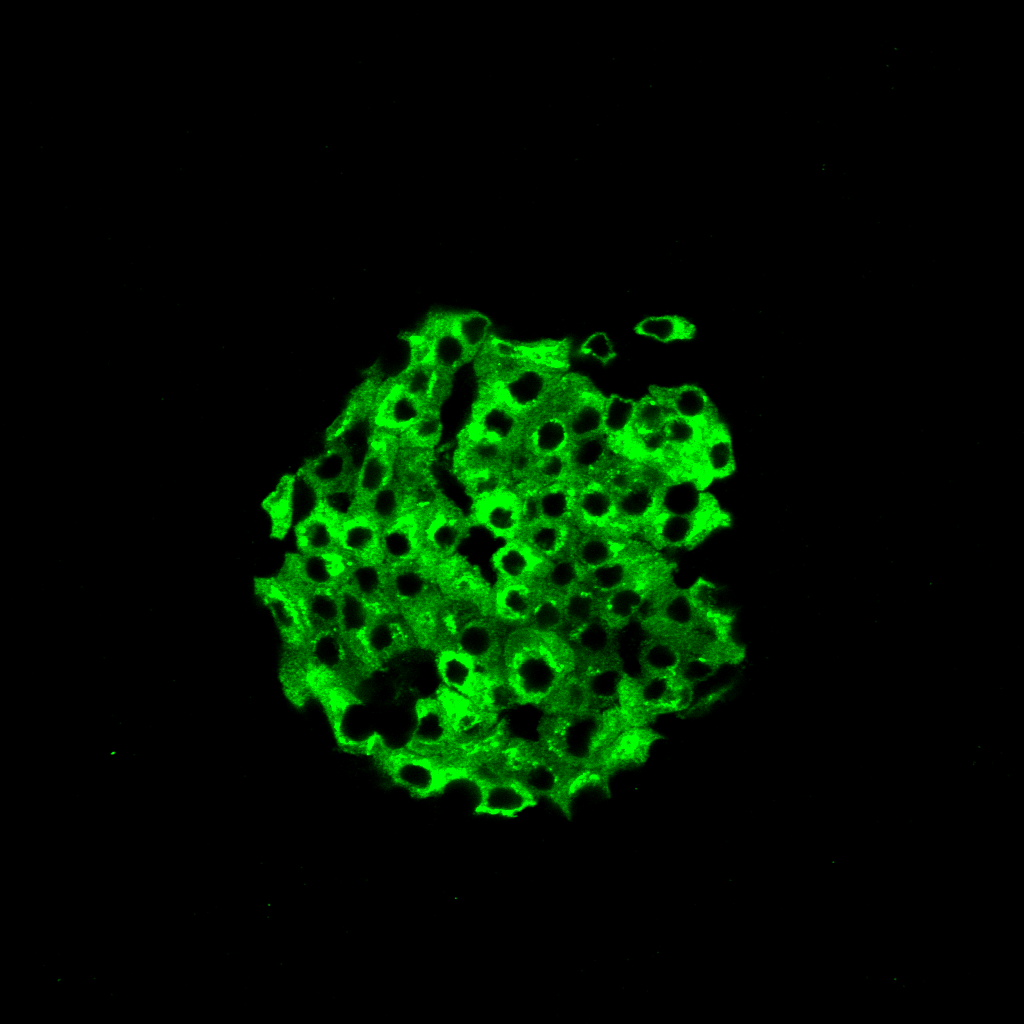

Supplement: Supplementary file 6 — Source data Fig. 4 [file 44318_2025_434_MOESM6_ESM.zip › Figure 4/4E/4E_15.tif (green).tif]

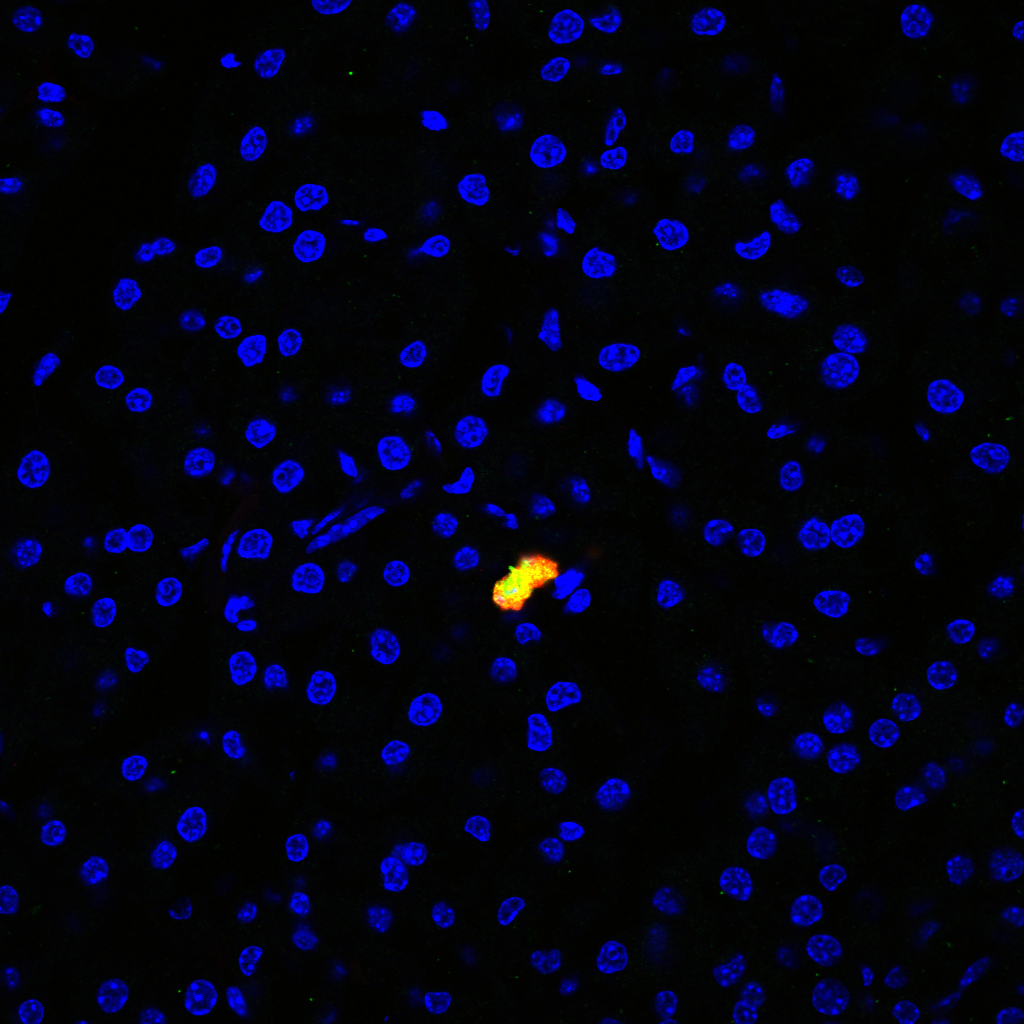

Supplement: Supplementary file 6 — Source data Fig. 4 [file 44318_2025_434_MOESM6_ESM.zip › Figure 4/4E/4E_23.tif]

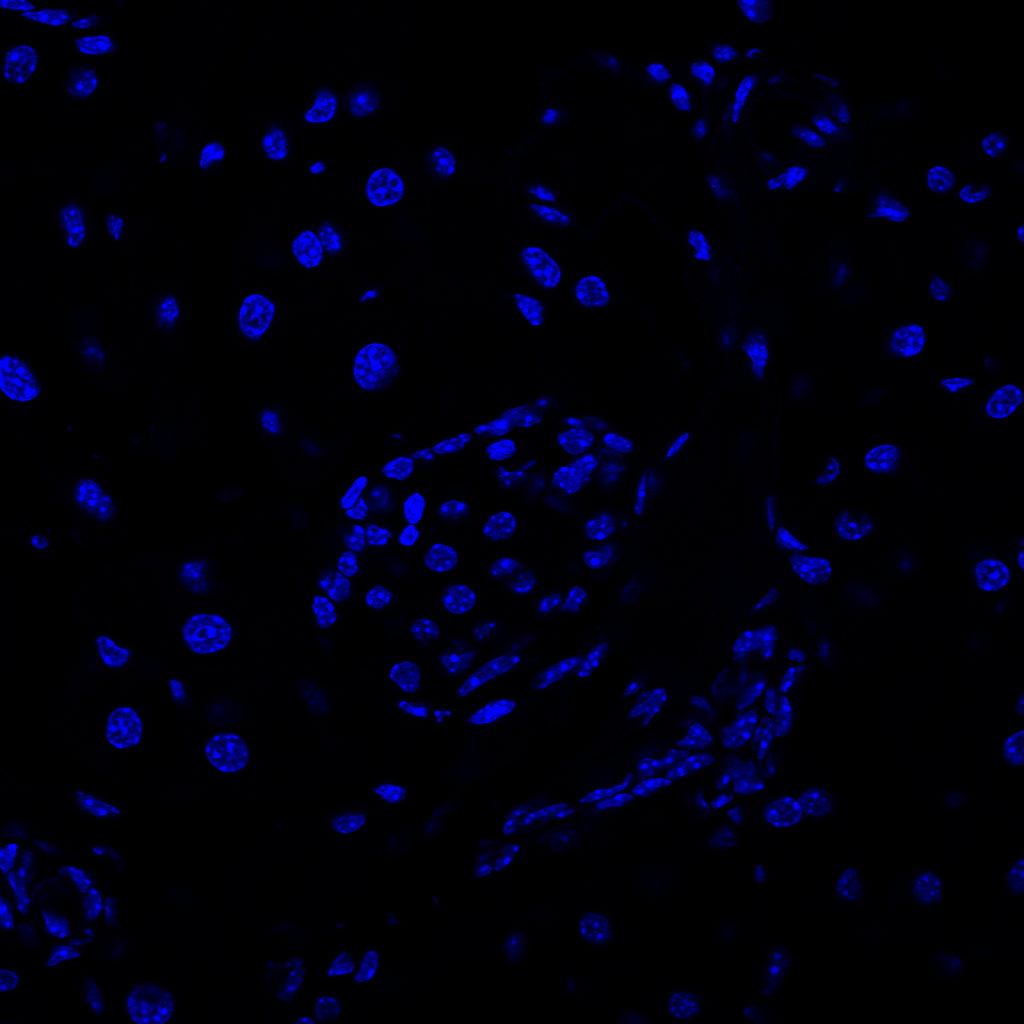

Supplement: Supplementary file 6 — Source data Fig. 4 [file 44318_2025_434_MOESM6_ESM.zip › Figure 4/4E/4E_22.tif (blue).tif]

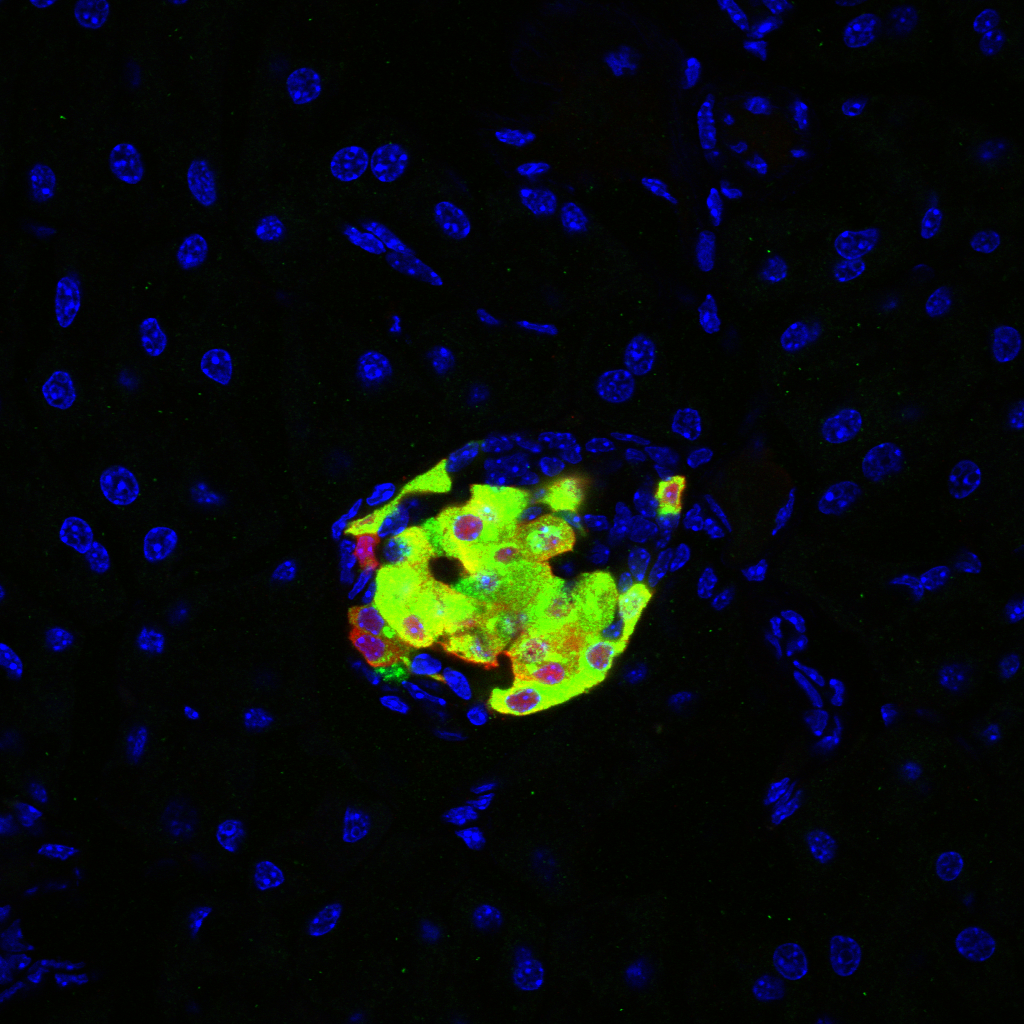

Supplement: Supplementary file 6 — Source data Fig. 4 [file 44318_2025_434_MOESM6_ESM.zip › Figure 4/4E/4E_21.tif]

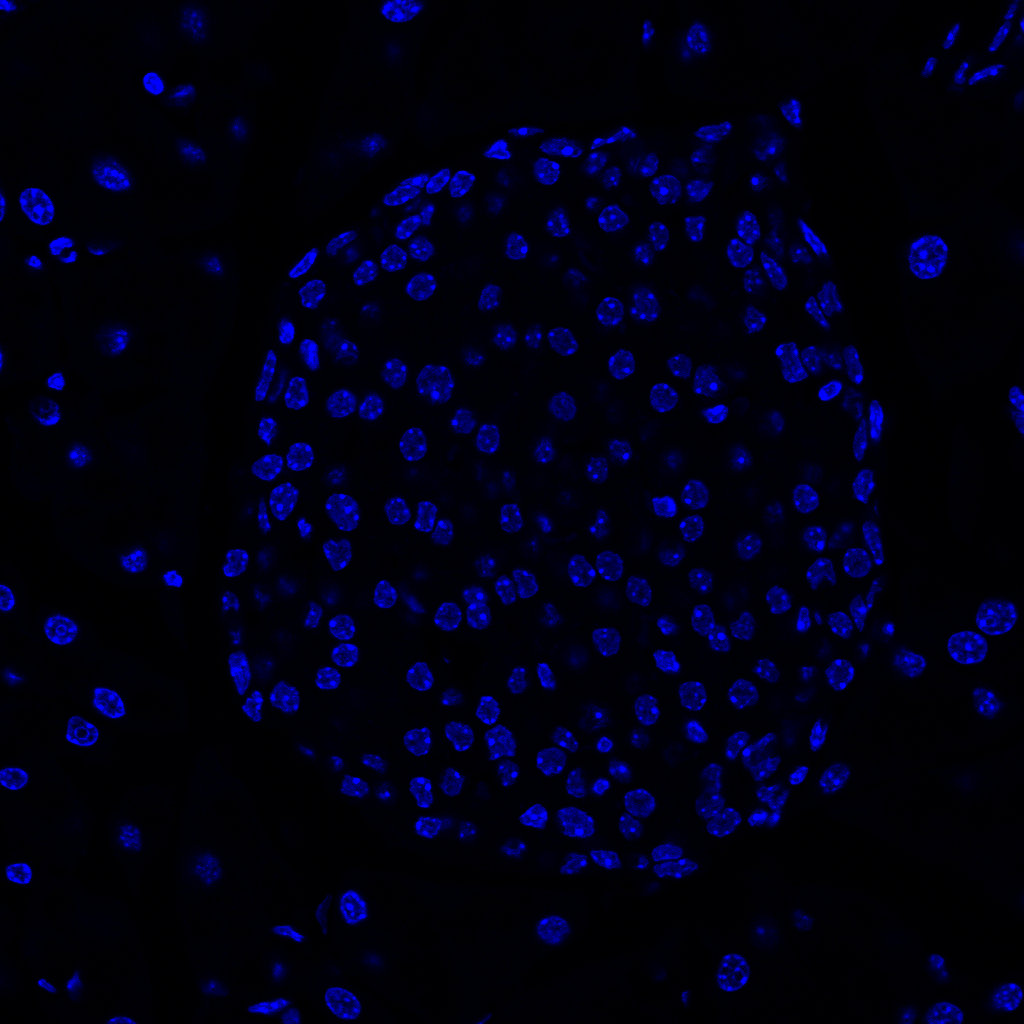

Supplement: Supplementary file 6 — Source data Fig. 4 [file 44318_2025_434_MOESM6_ESM.zip › Figure 4/4E/4E_9.tif (blue).tif]

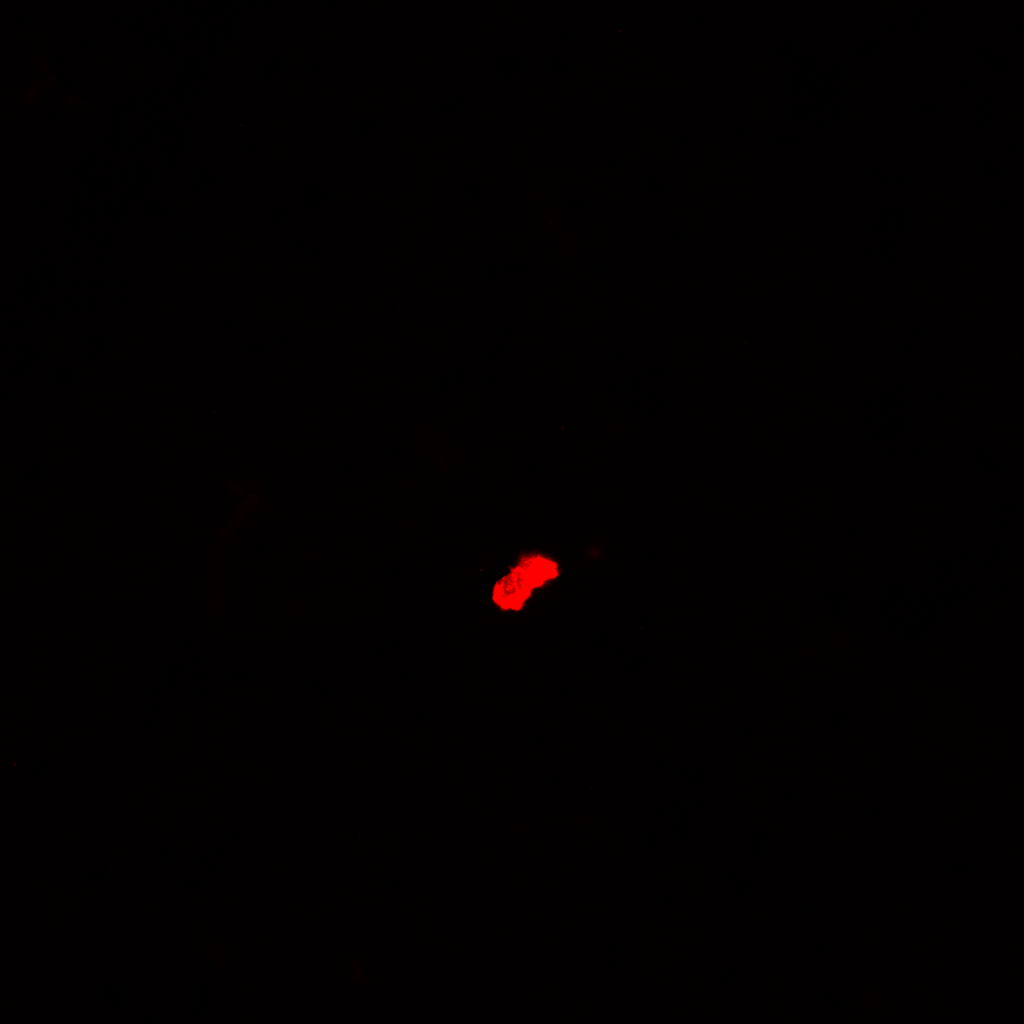

Supplement: Supplementary file 6 — Source data Fig. 4 [file 44318_2025_434_MOESM6_ESM.zip › Figure 4/4E/4E_23.tif (red).tif]

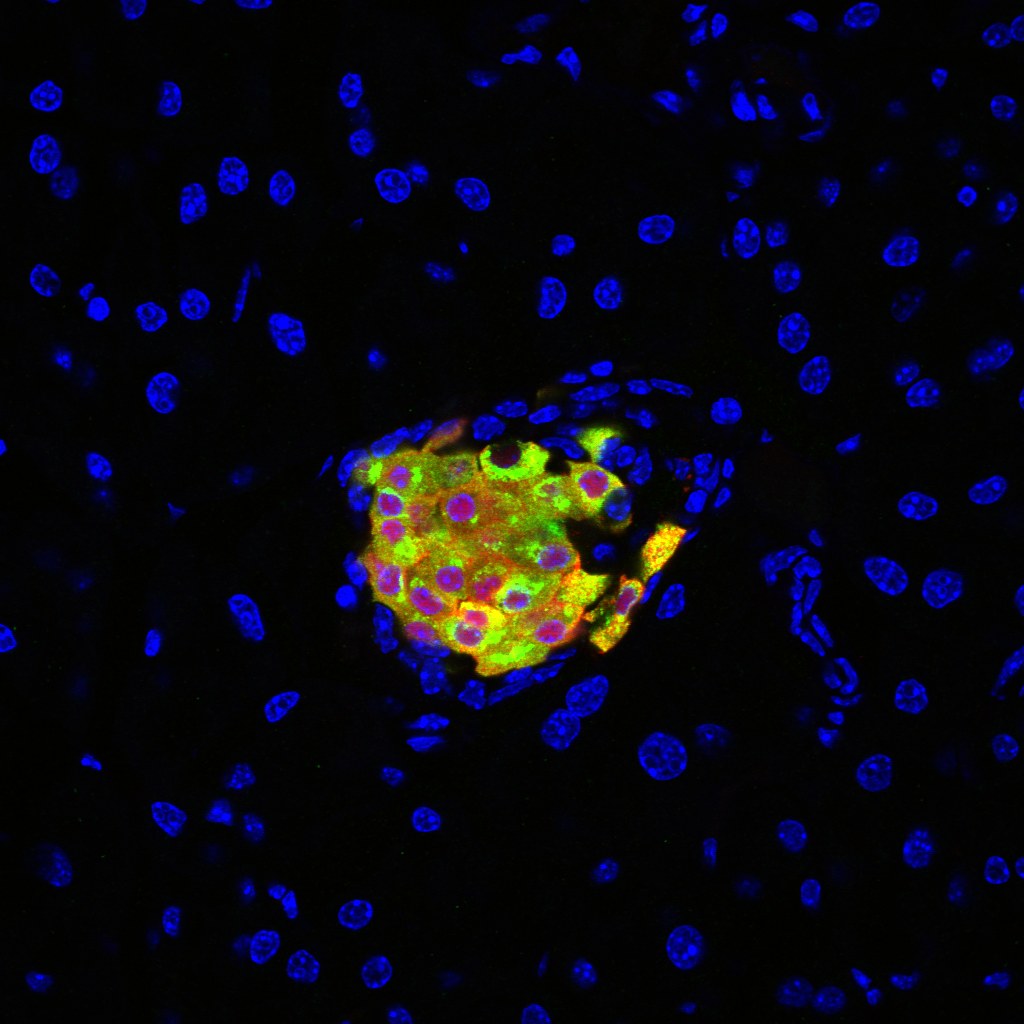

Supplement: Supplementary file 6 — Source data Fig. 4 [file 44318_2025_434_MOESM6_ESM.zip › Figure 4/4E/4E_20.tif]

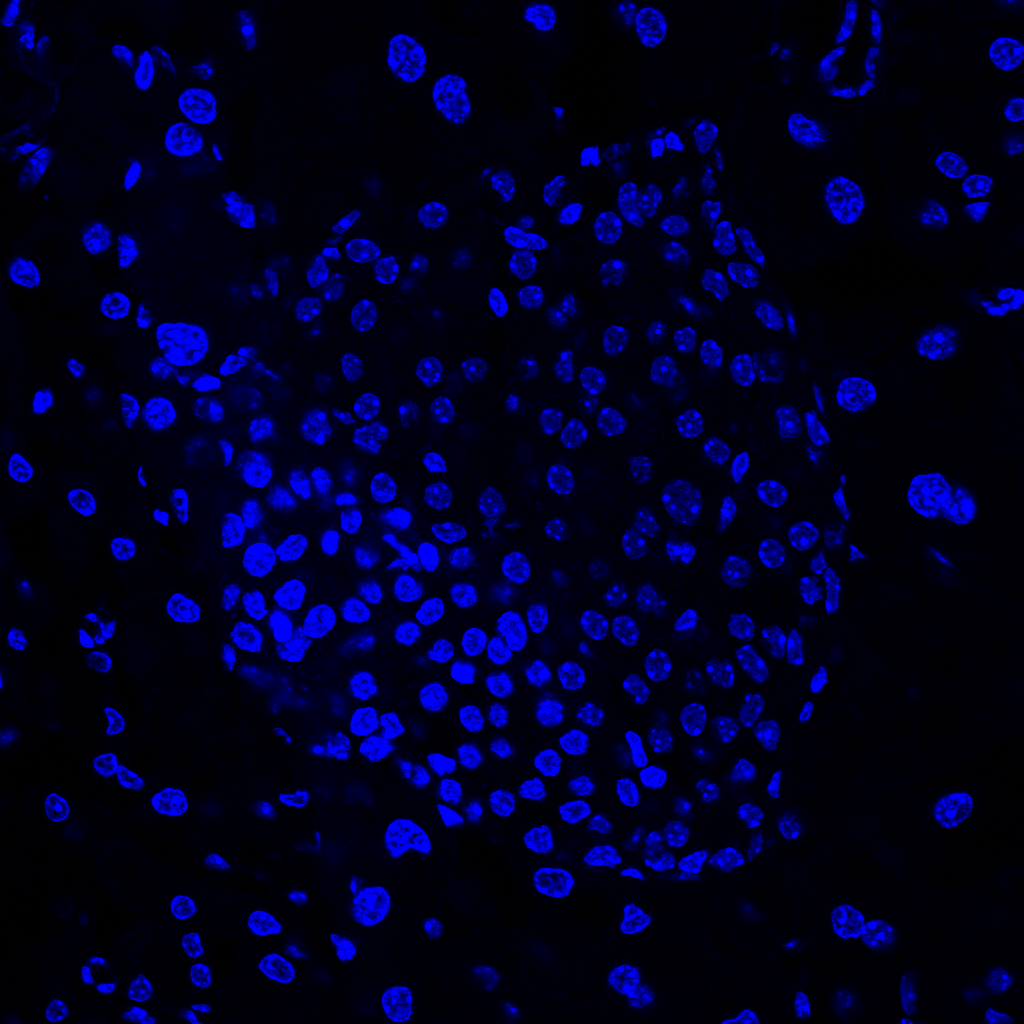

Supplement: Supplementary file 6 — Source data Fig. 4 [file 44318_2025_434_MOESM6_ESM.zip › Figure 4/4E/4E_8.tif (blue).tif]

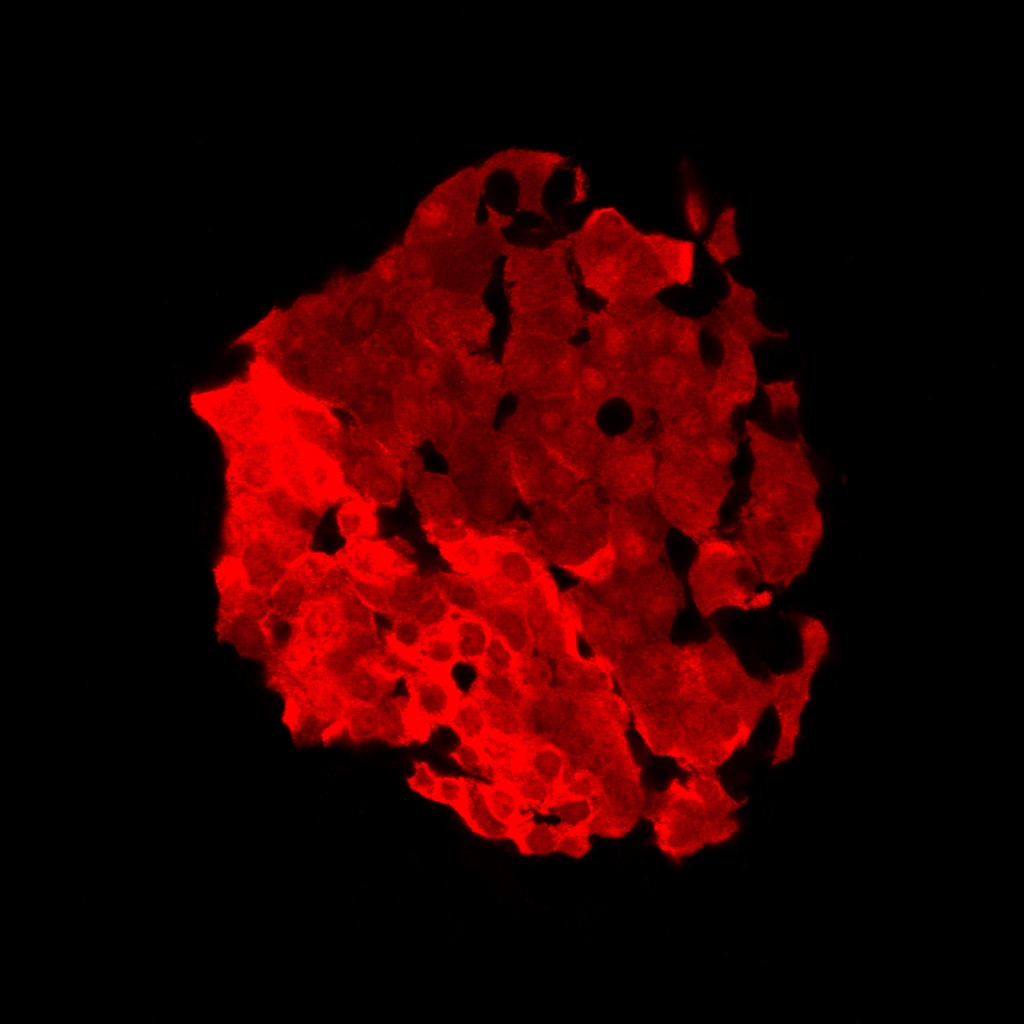

Supplement: Supplementary file 6 — Source data Fig. 4 [file 44318_2025_434_MOESM6_ESM.zip › Figure 4/4E/4E_8.tif (red).tif]

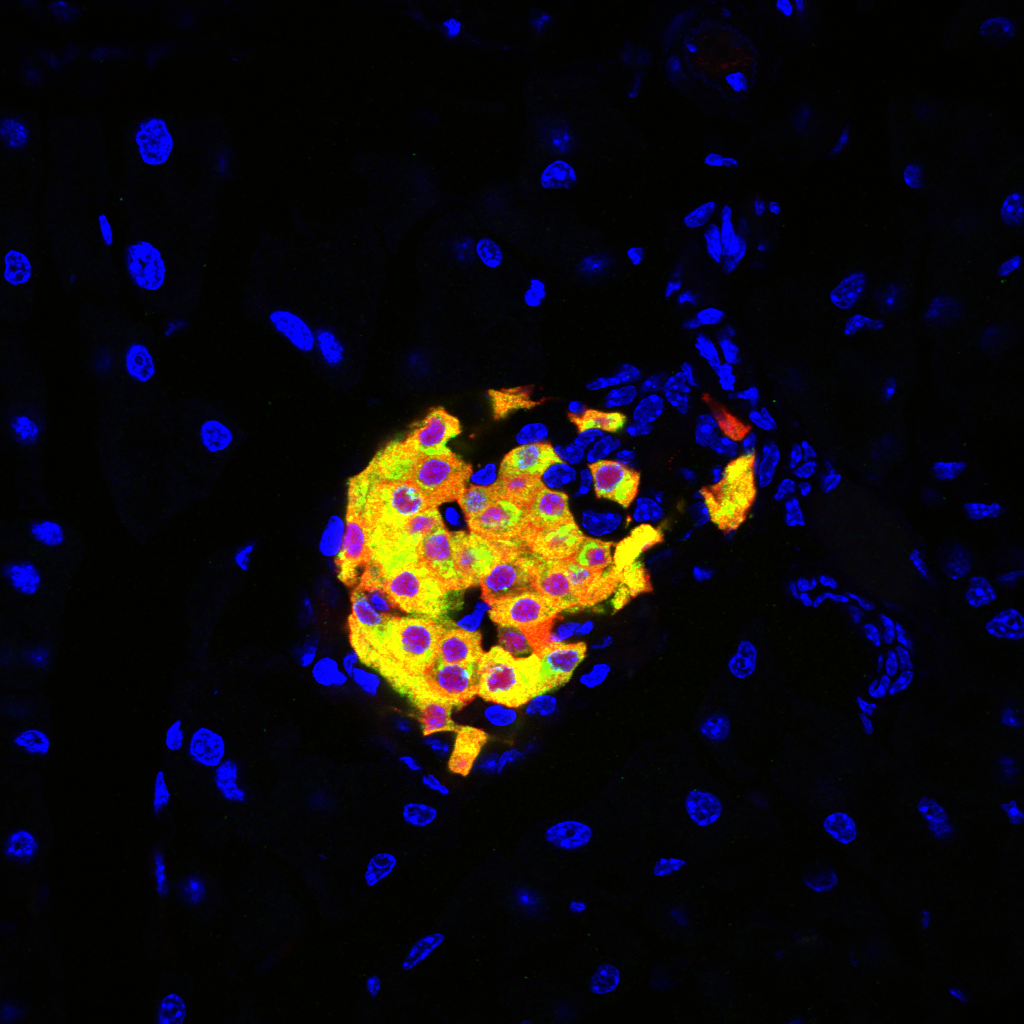

Supplement: Supplementary file 6 — Source data Fig. 4 [file 44318_2025_434_MOESM6_ESM.zip › Figure 4/4E/4E_18.tif]

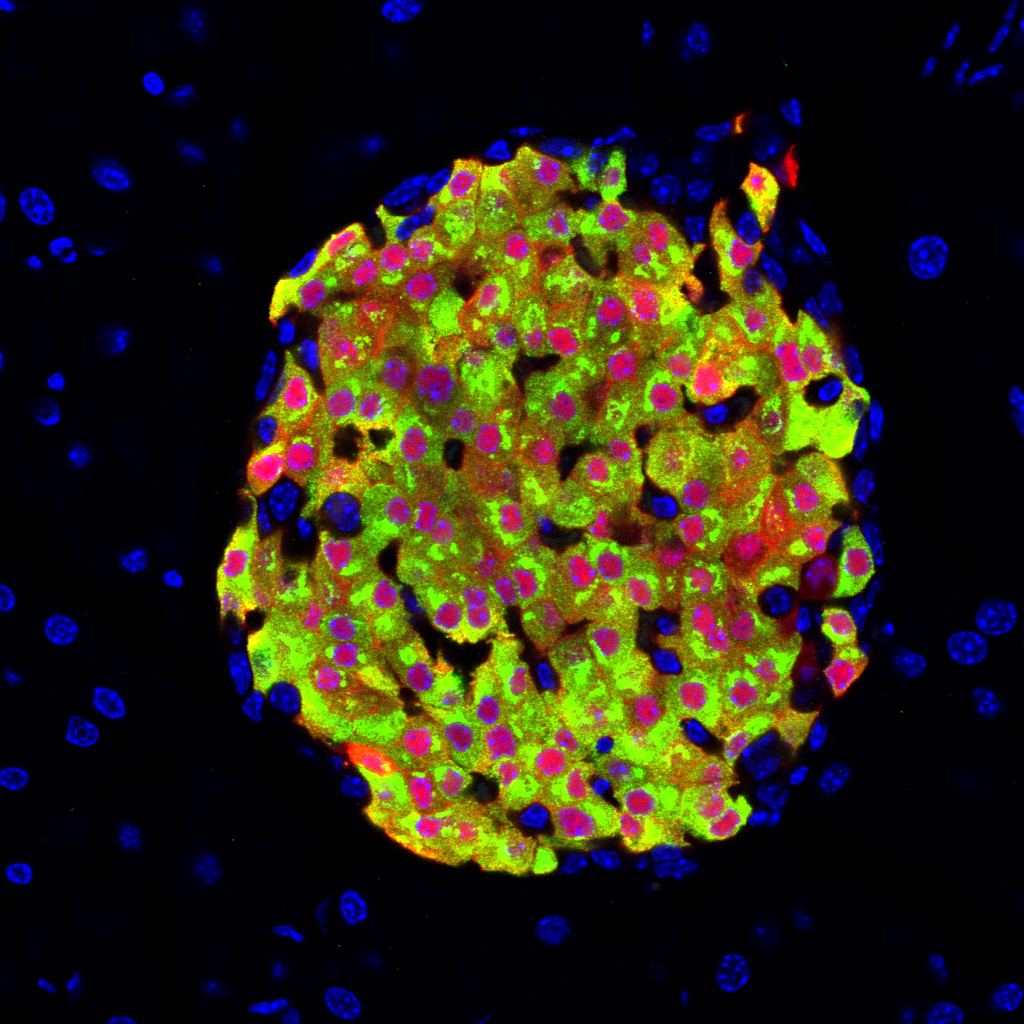

Supplement: Supplementary file 6 — Source data Fig. 4 [file 44318_2025_434_MOESM6_ESM.zip › Figure 4/4E/4E_9.tif]

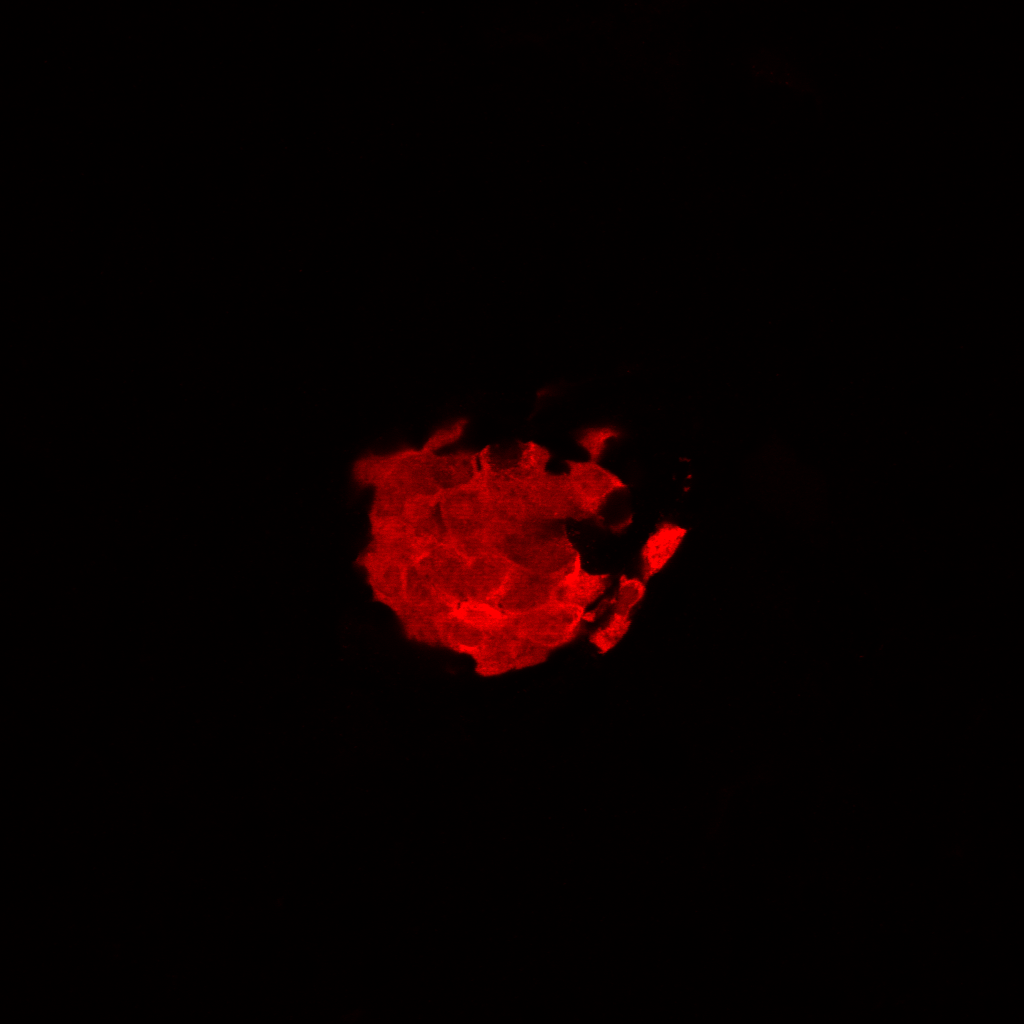

Supplement: Supplementary file 6 — Source data Fig. 4 [file 44318_2025_434_MOESM6_ESM.zip › Figure 4/4E/4E_20.tif (red).tif]

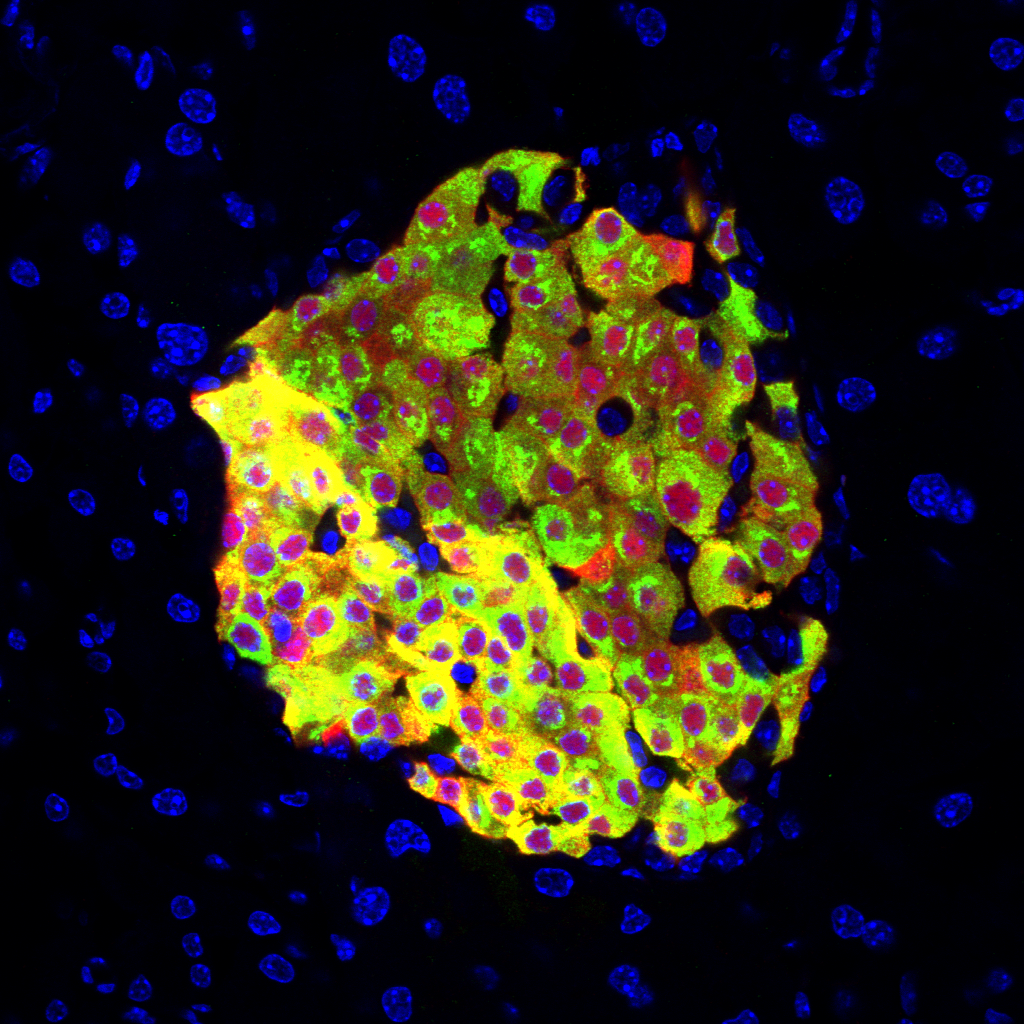

Supplement: Supplementary file 6 — Source data Fig. 4 [file 44318_2025_434_MOESM6_ESM.zip › Figure 4/4E/4E_8.tif]

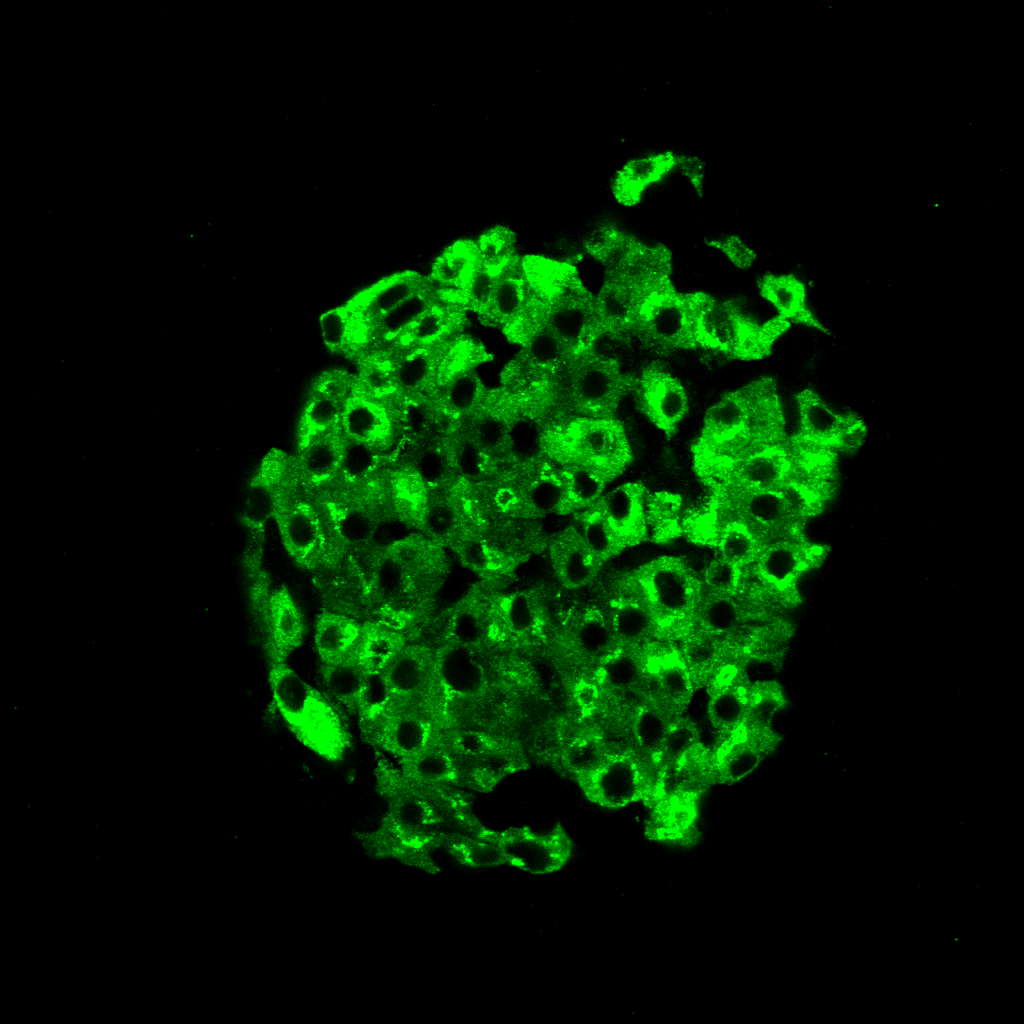

Supplement: Supplementary file 6 — Source data Fig. 4 [file 44318_2025_434_MOESM6_ESM.zip › Figure 4/4E/4E_6.tif (green).tif]

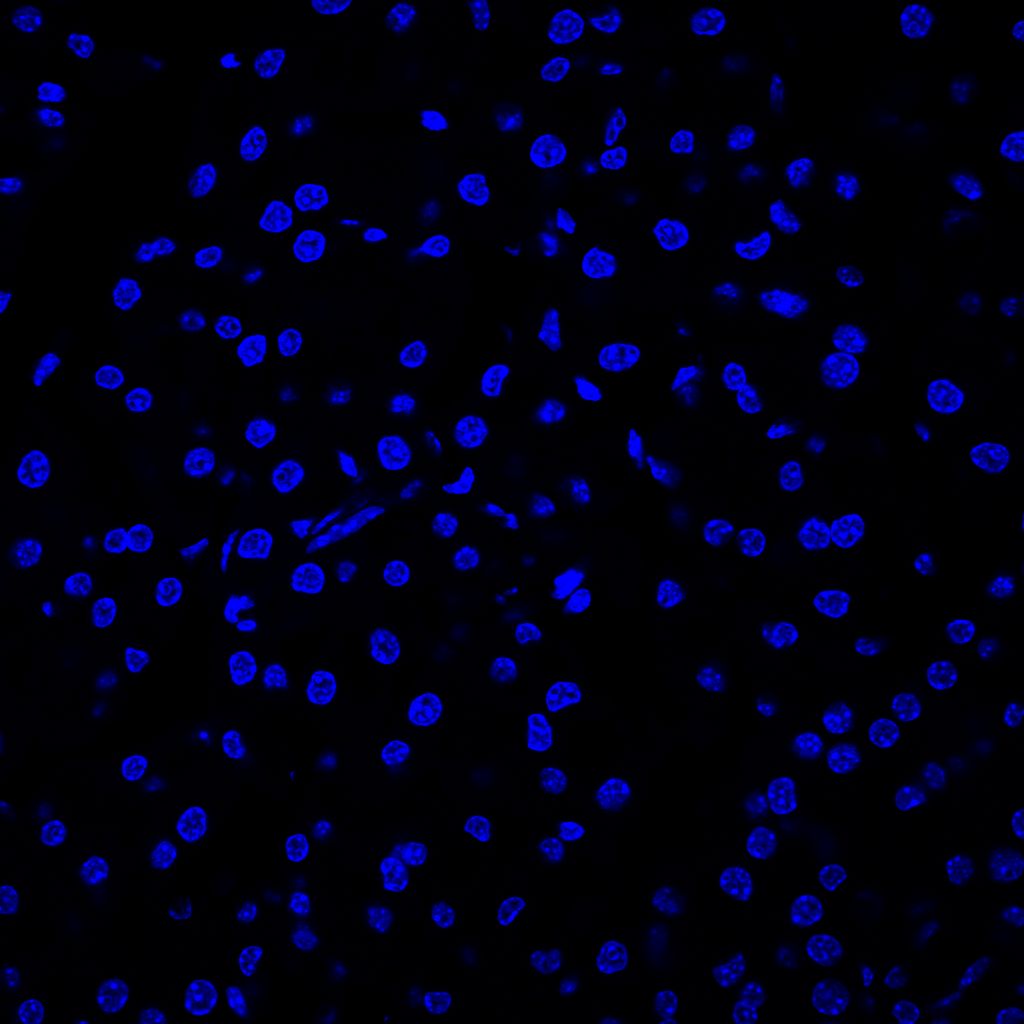

Supplement: Supplementary file 6 — Source data Fig. 4 [file 44318_2025_434_MOESM6_ESM.zip › Figure 4/4E/4E_23.tif (blue).tif]

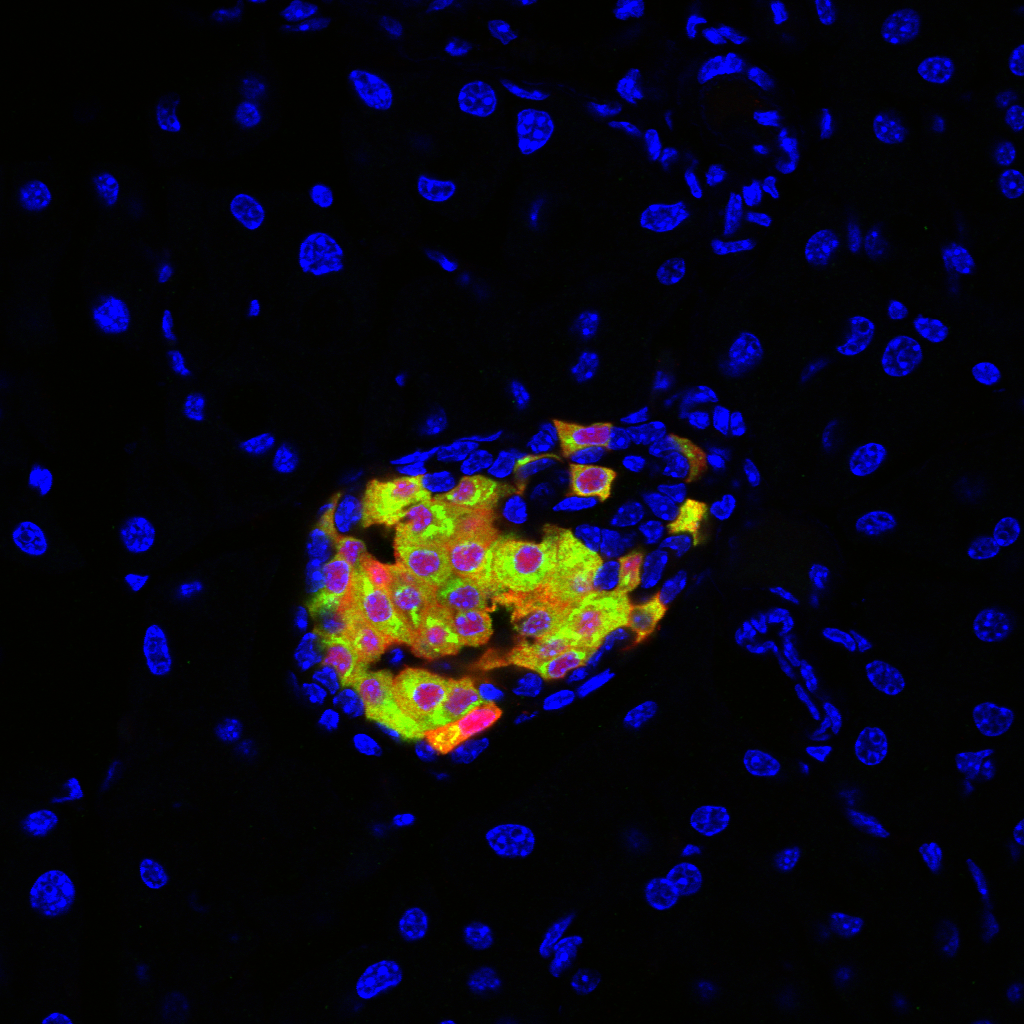

Supplement: Supplementary file 6 — Source data Fig. 4 [file 44318_2025_434_MOESM6_ESM.zip › Figure 4/4E/4E_19.tif]

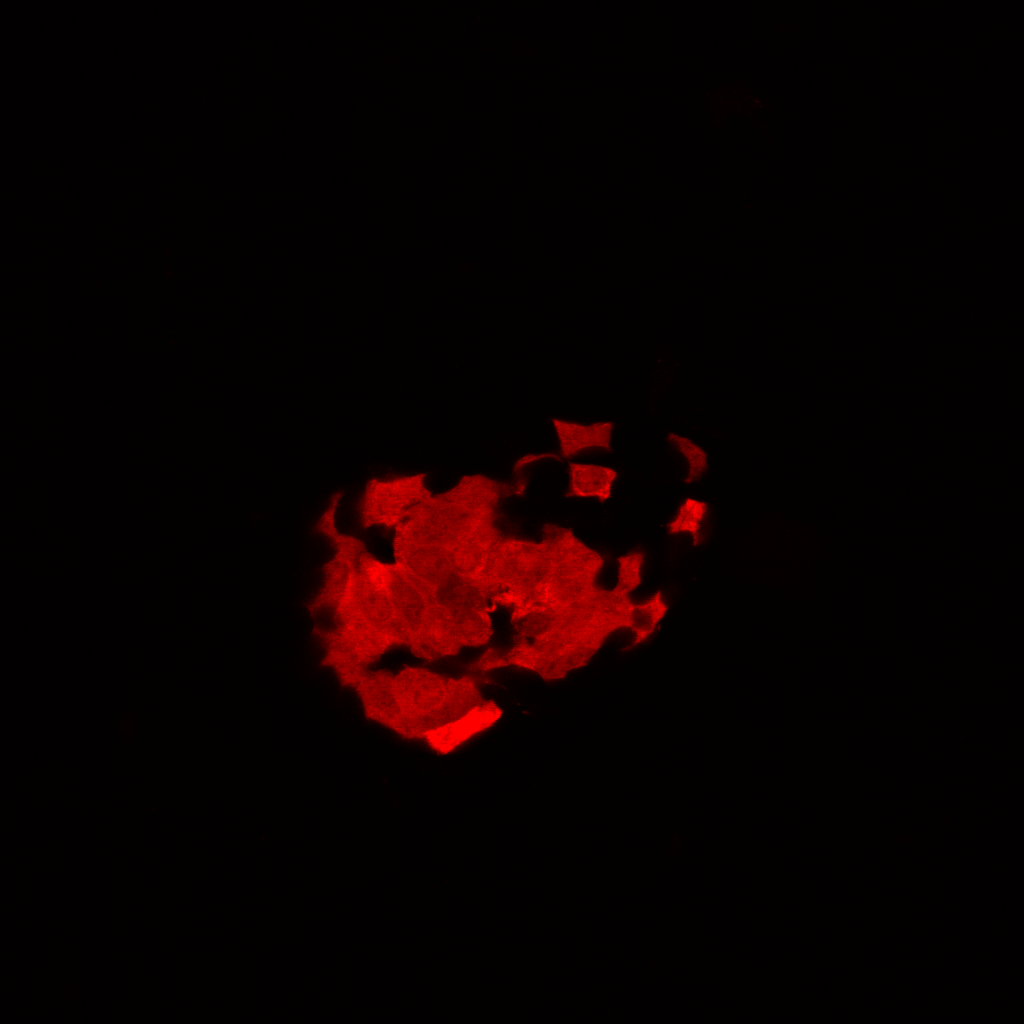

Supplement: Supplementary file 6 — Source data Fig. 4 [file 44318_2025_434_MOESM6_ESM.zip › Figure 4/4E/4E_19.tif (red).tif]

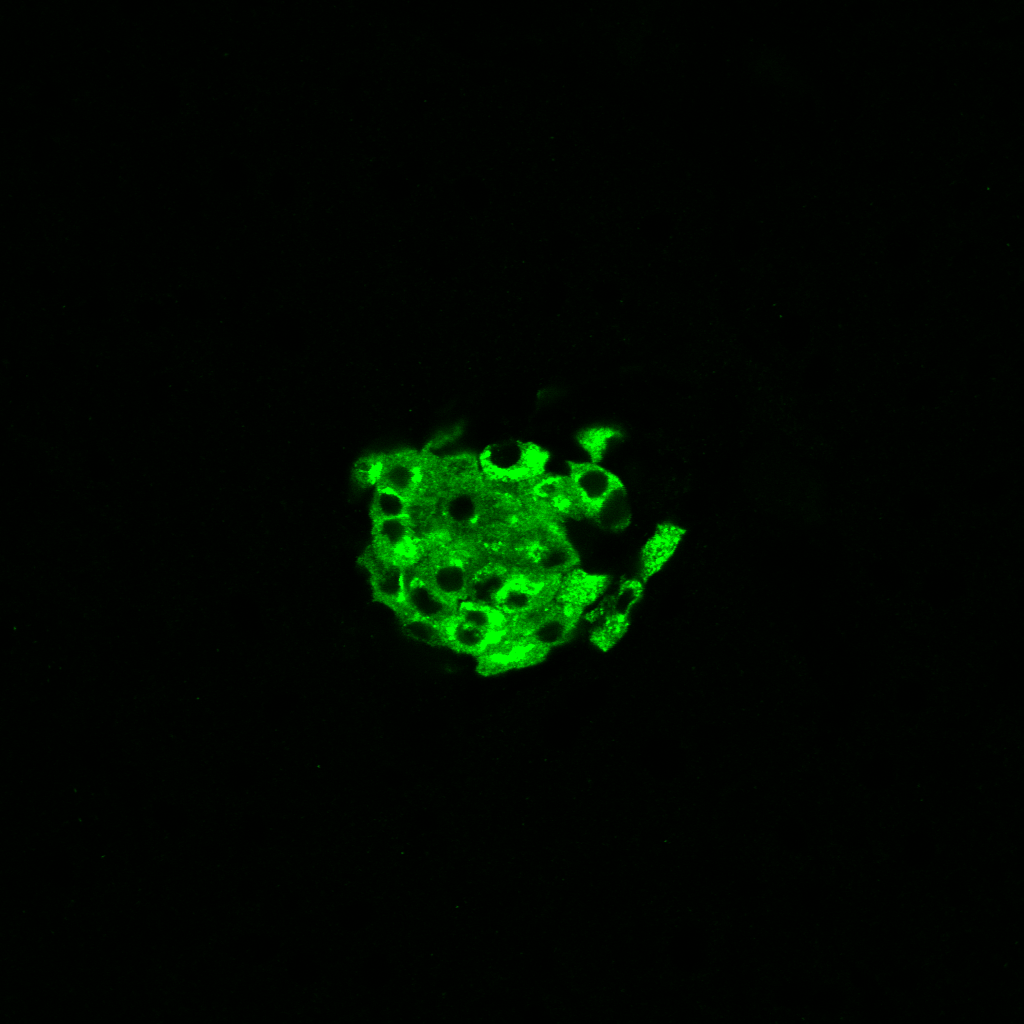

Supplement: Supplementary file 6 — Source data Fig. 4 [file 44318_2025_434_MOESM6_ESM.zip › Figure 4/4E/4E_20.tif (green).tif]

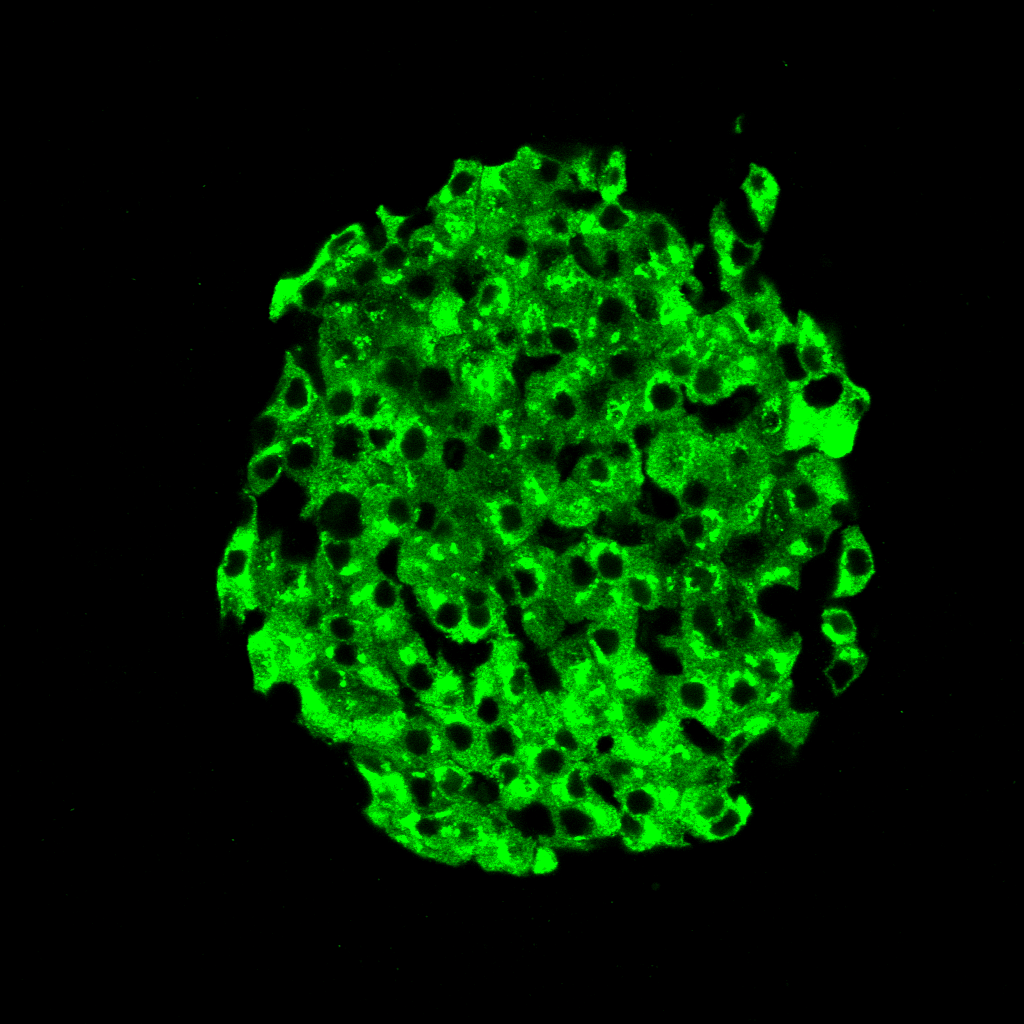

Supplement: Supplementary file 6 — Source data Fig. 4 [file 44318_2025_434_MOESM6_ESM.zip › Figure 4/4E/4E_9.tif (green).tif]

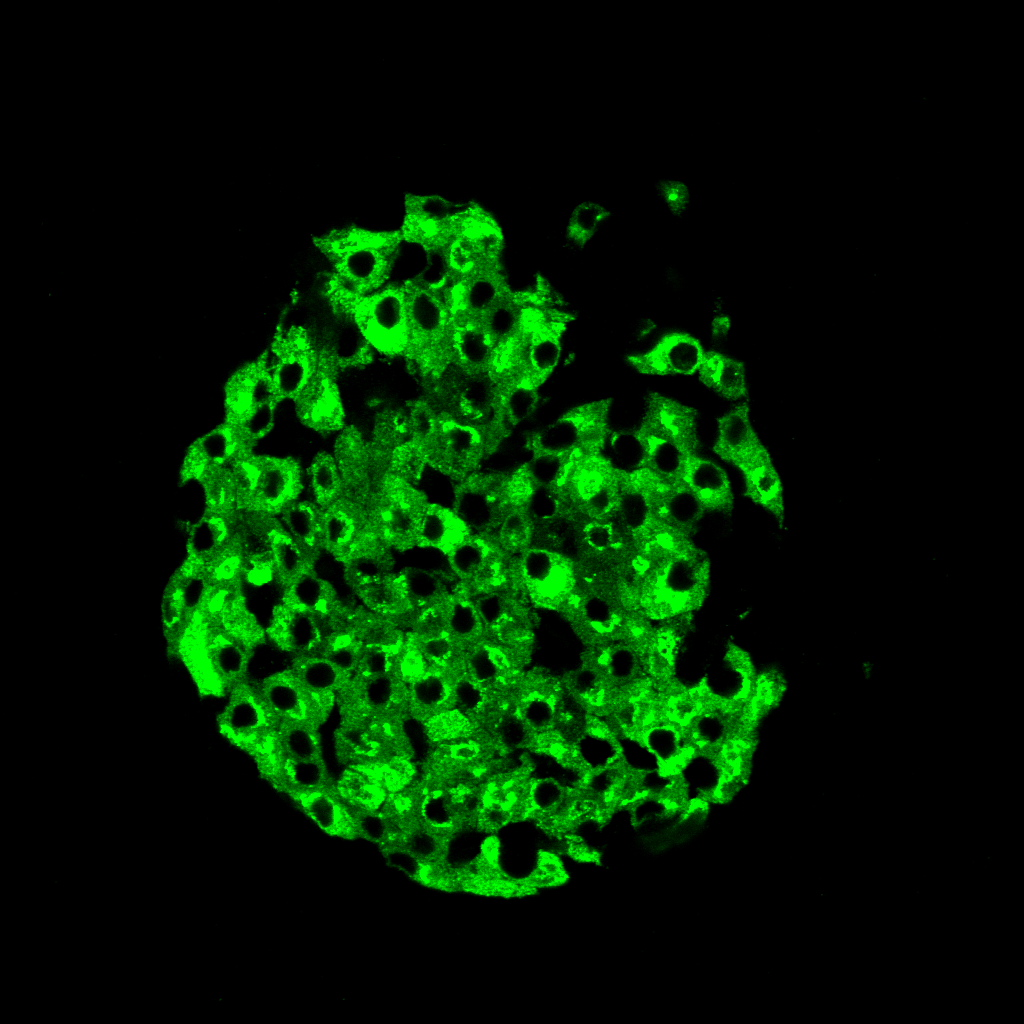

Supplement: Supplementary file 6 — Source data Fig. 4 [file 44318_2025_434_MOESM6_ESM.zip › Figure 4/4E/4E_12.tif (green).tif]

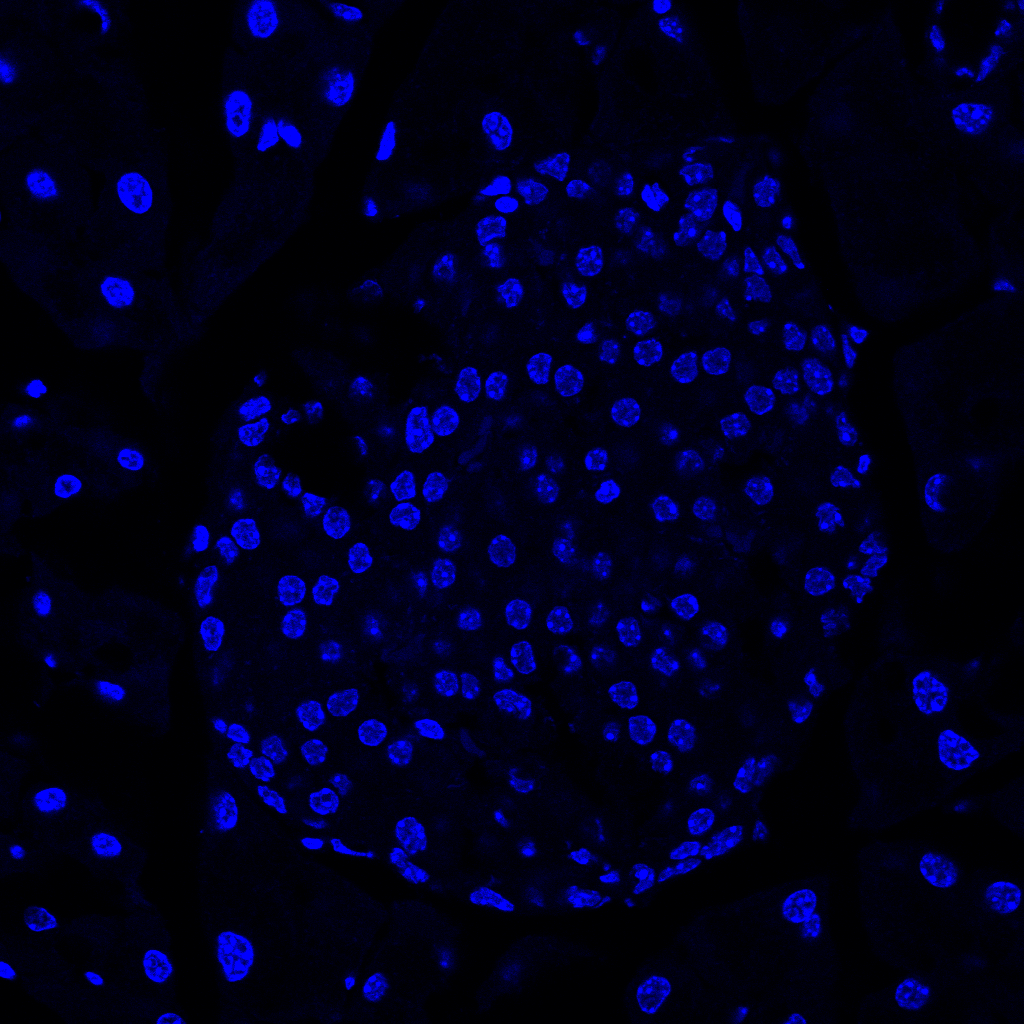

Supplement: Supplementary file 6 — Source data Fig. 4 [file 44318_2025_434_MOESM6_ESM.zip › Figure 4/4E/4E_7.tif (blue).tif]

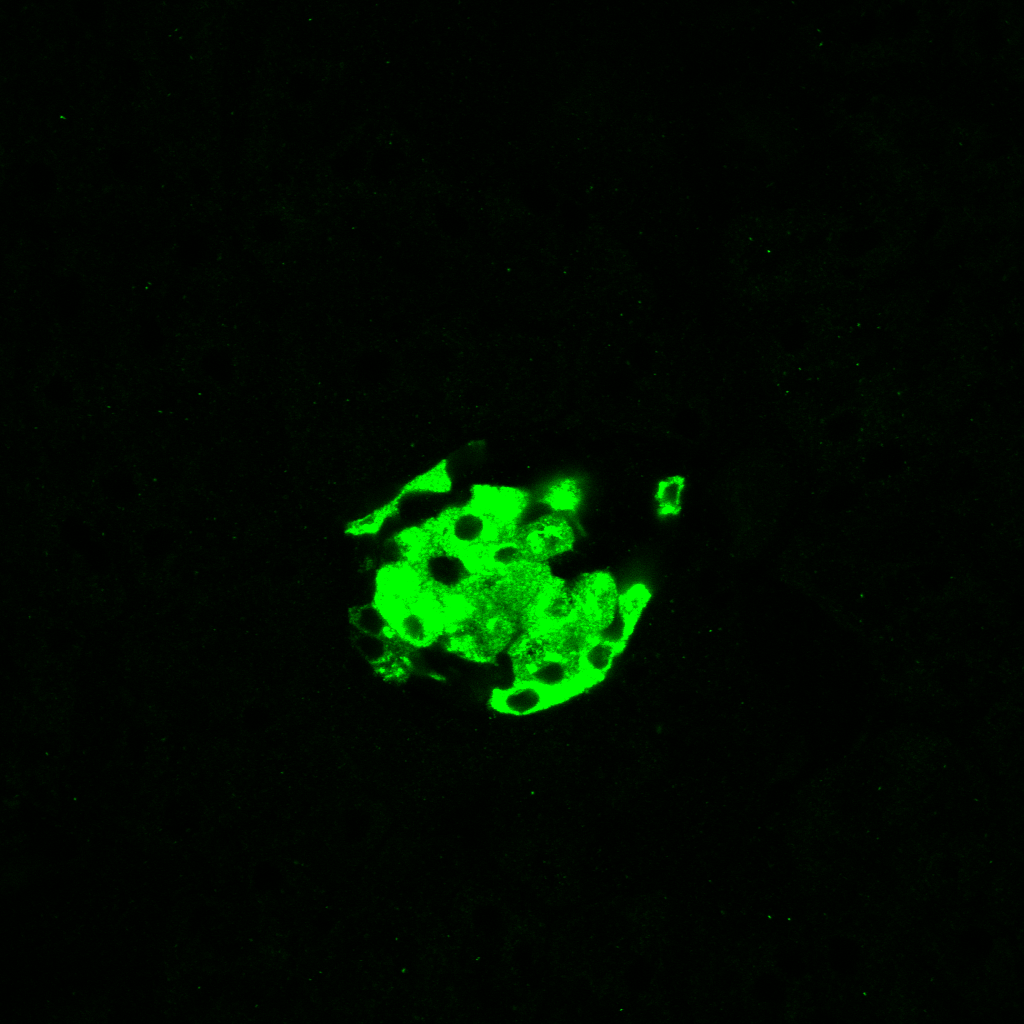

Supplement: Supplementary file 6 — Source data Fig. 4 [file 44318_2025_434_MOESM6_ESM.zip › Figure 4/4E/4E_21.tif (green).tif]

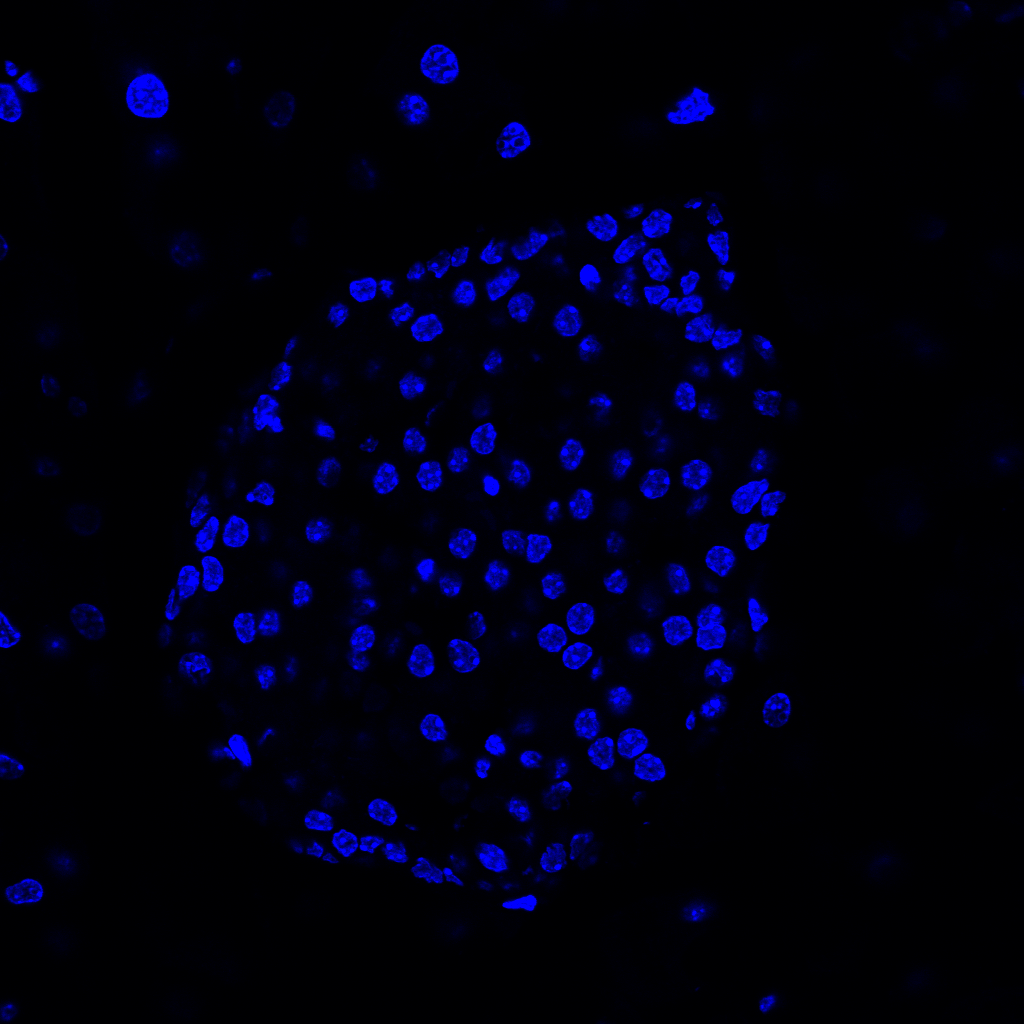

Supplement: Supplementary file 6 — Source data Fig. 4 [file 44318_2025_434_MOESM6_ESM.zip › Figure 4/4E/4E_5.tif (blue).tif]

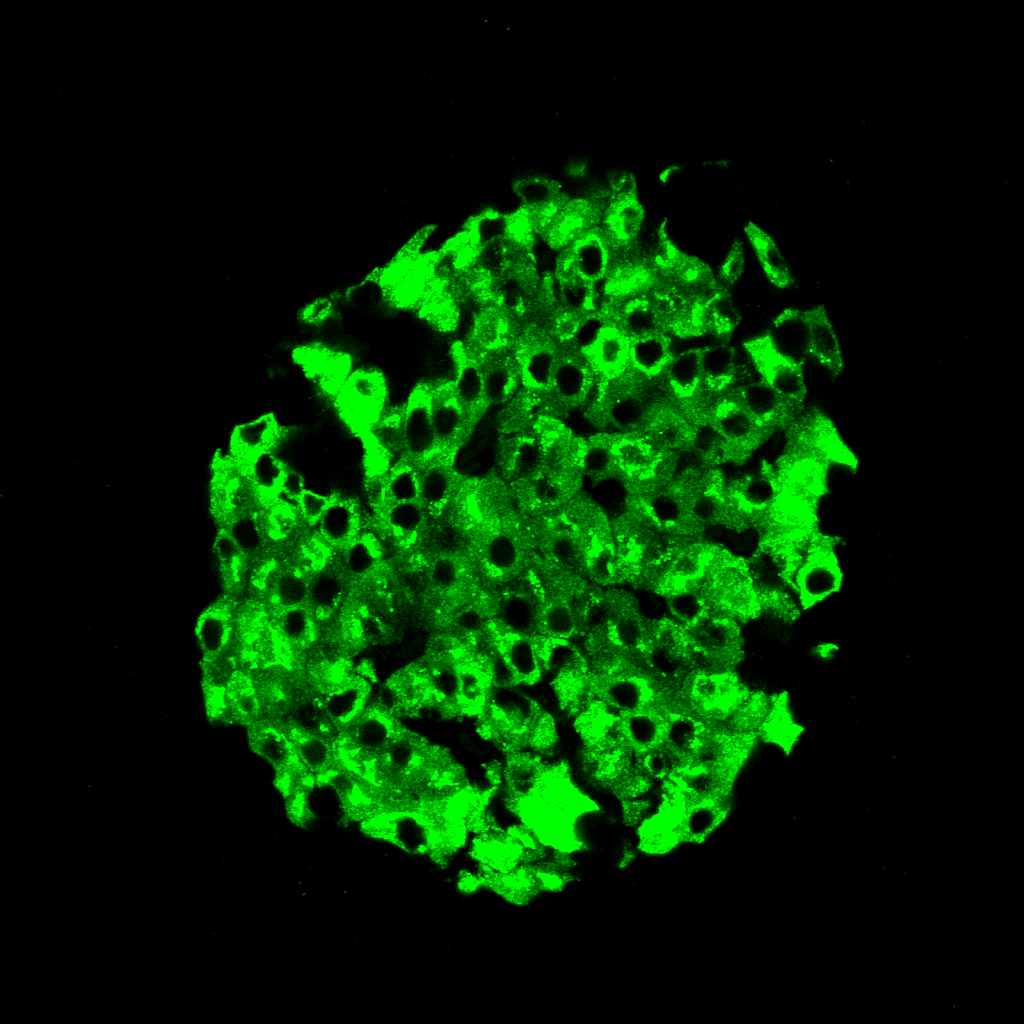

Supplement: Supplementary file 6 — Source data Fig. 4 [file 44318_2025_434_MOESM6_ESM.zip › Figure 4/4E/4E_7.tif (green).tif]

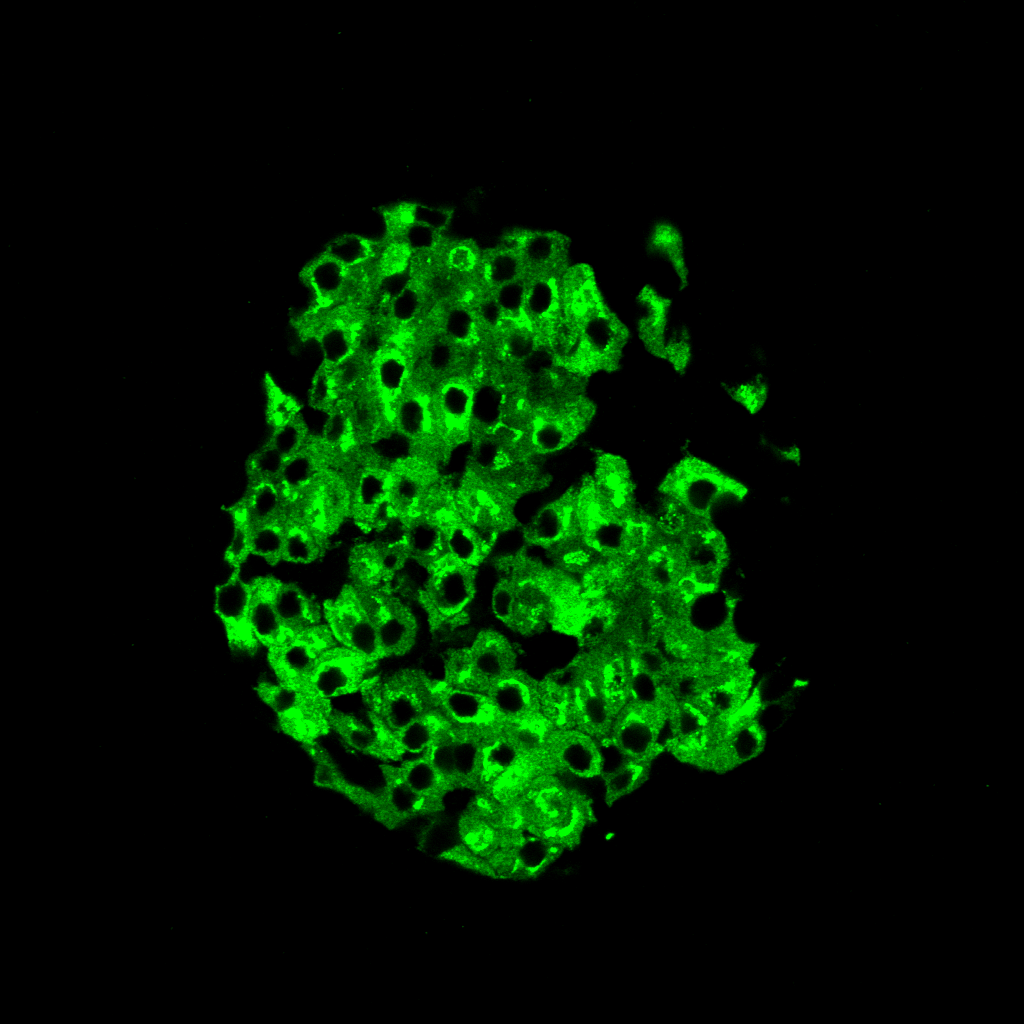

Supplement: Supplementary file 6 — Source data Fig. 4 [file 44318_2025_434_MOESM6_ESM.zip › Figure 4/4E/4E_13.tif (green).tif]

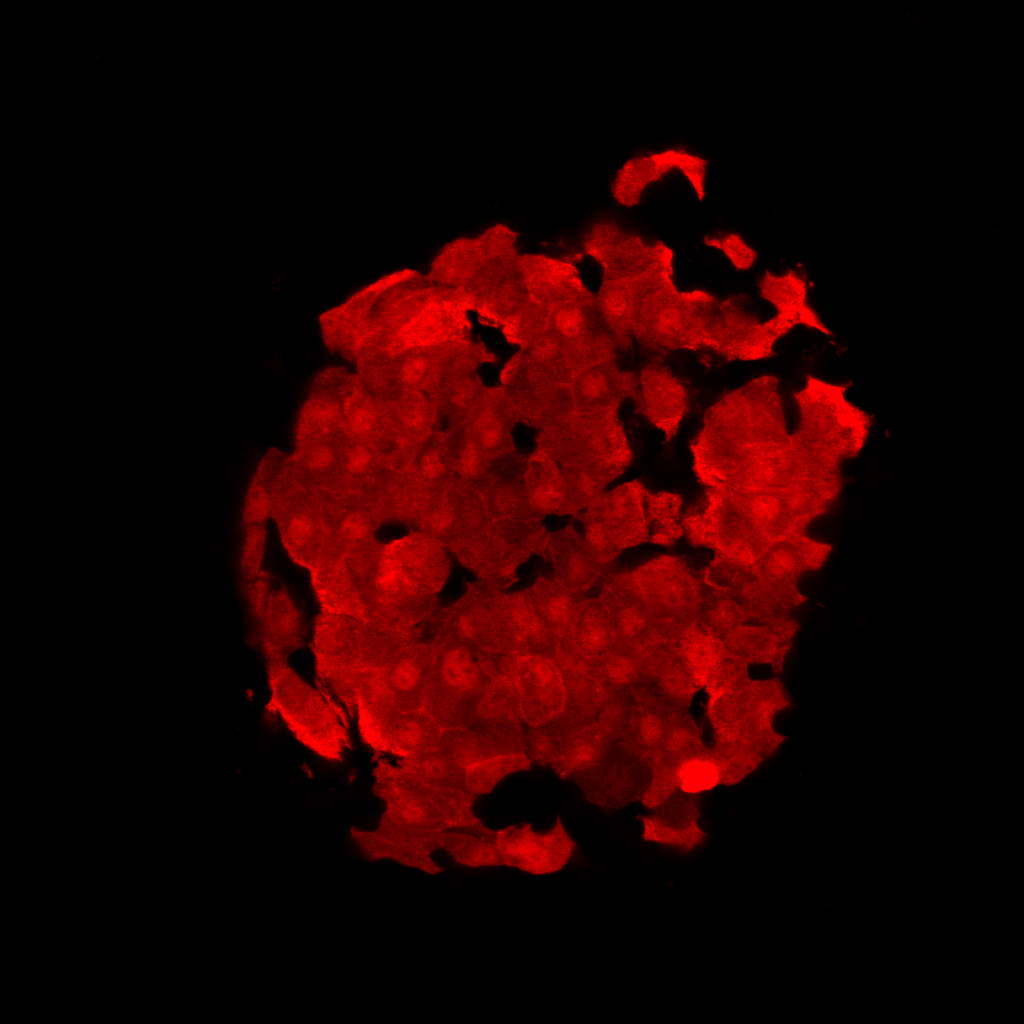

Supplement: Supplementary file 6 — Source data Fig. 4 [file 44318_2025_434_MOESM6_ESM.zip › Figure 4/4E/4E_6.tif (red).tif]

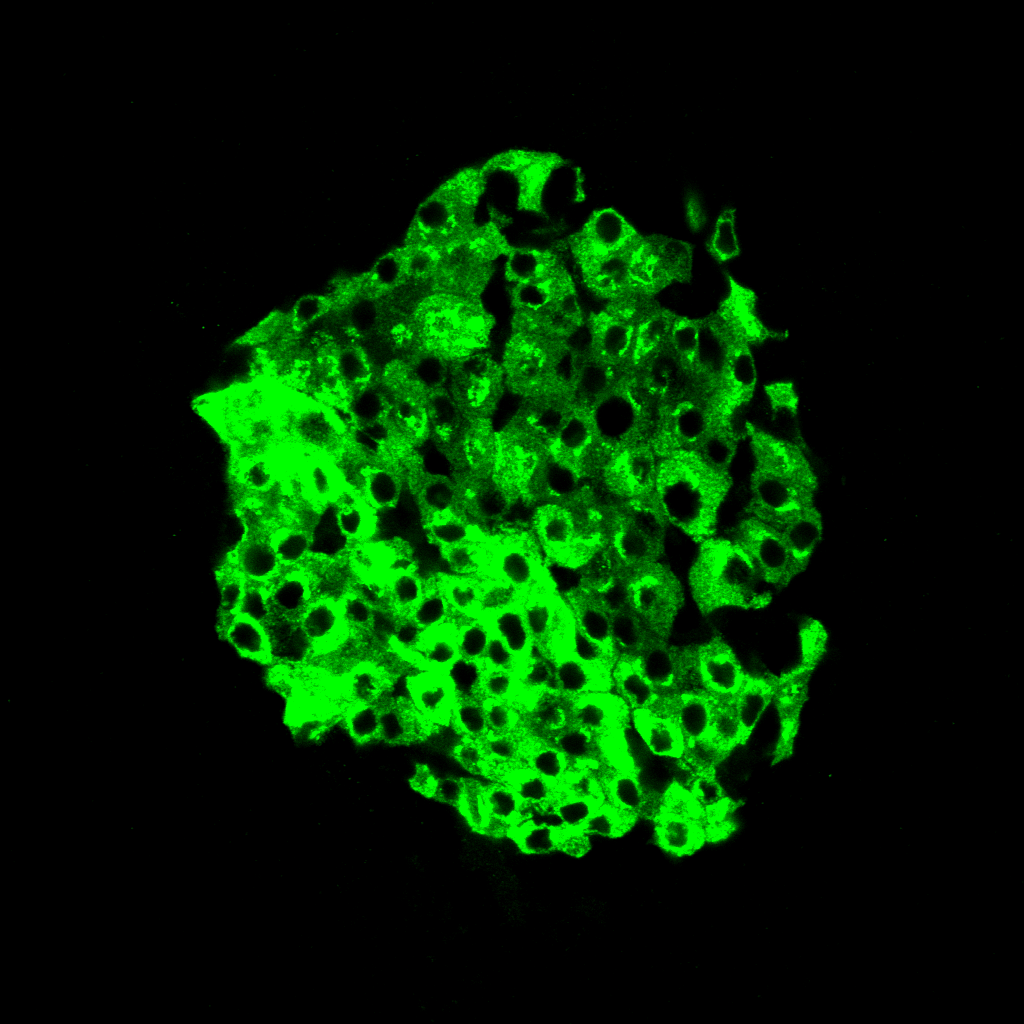

Supplement: Supplementary file 6 — Source data Fig. 4 [file 44318_2025_434_MOESM6_ESM.zip › Figure 4/4E/4E_8.tif (green).tif]

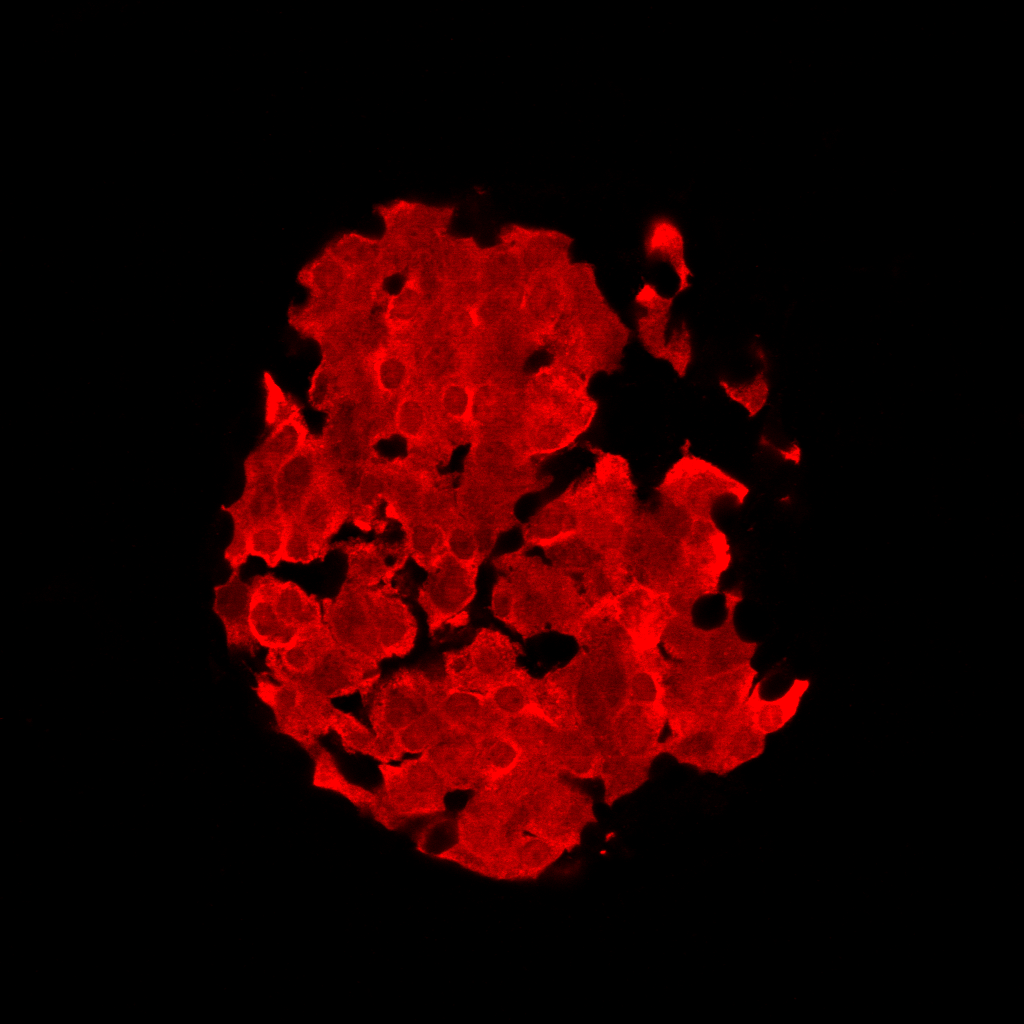

Supplement: Supplementary file 6 — Source data Fig. 4 [file 44318_2025_434_MOESM6_ESM.zip › Figure 4/4E/4E_13.tif (red).tif]

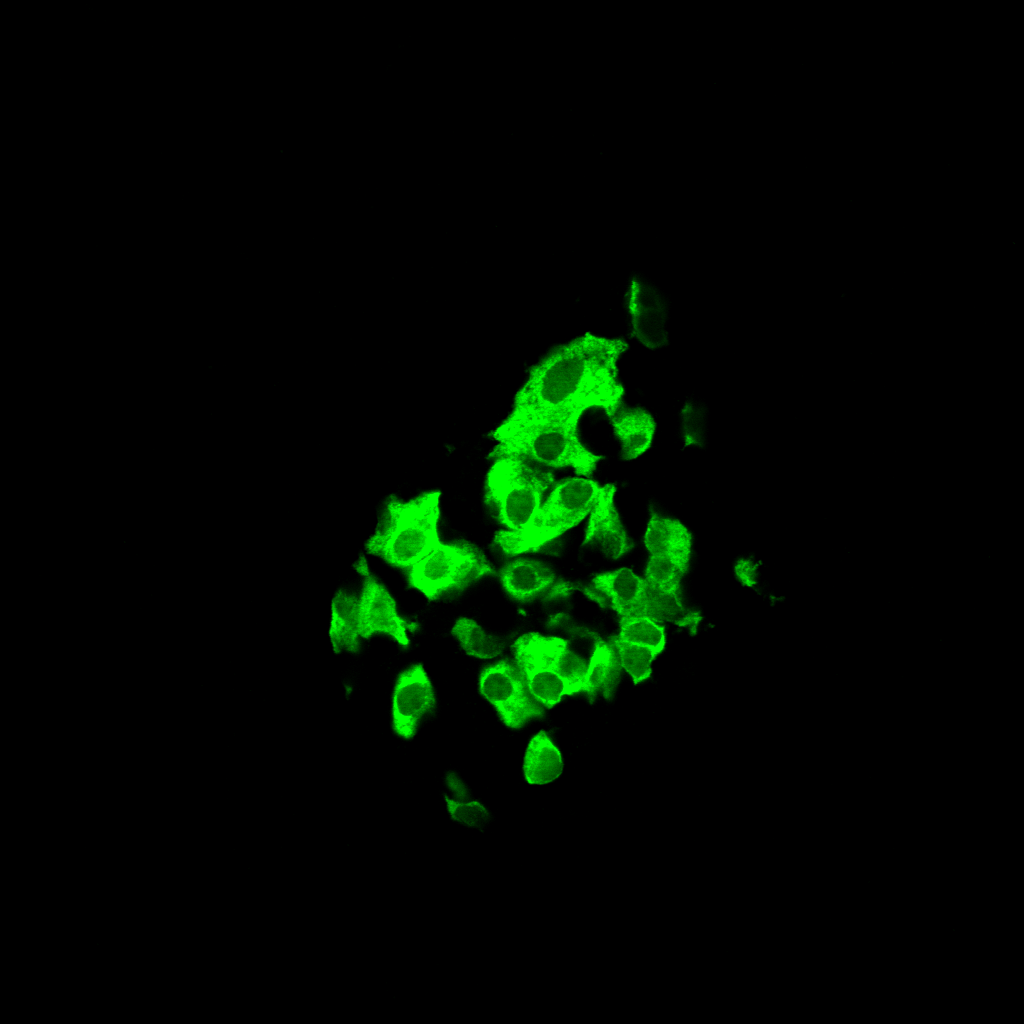

Supplement: Supplementary file 6 — Source data Fig. 4 [file 44318_2025_434_MOESM6_ESM.zip › Figure 4/4E/4E_2.tif (red).tif]

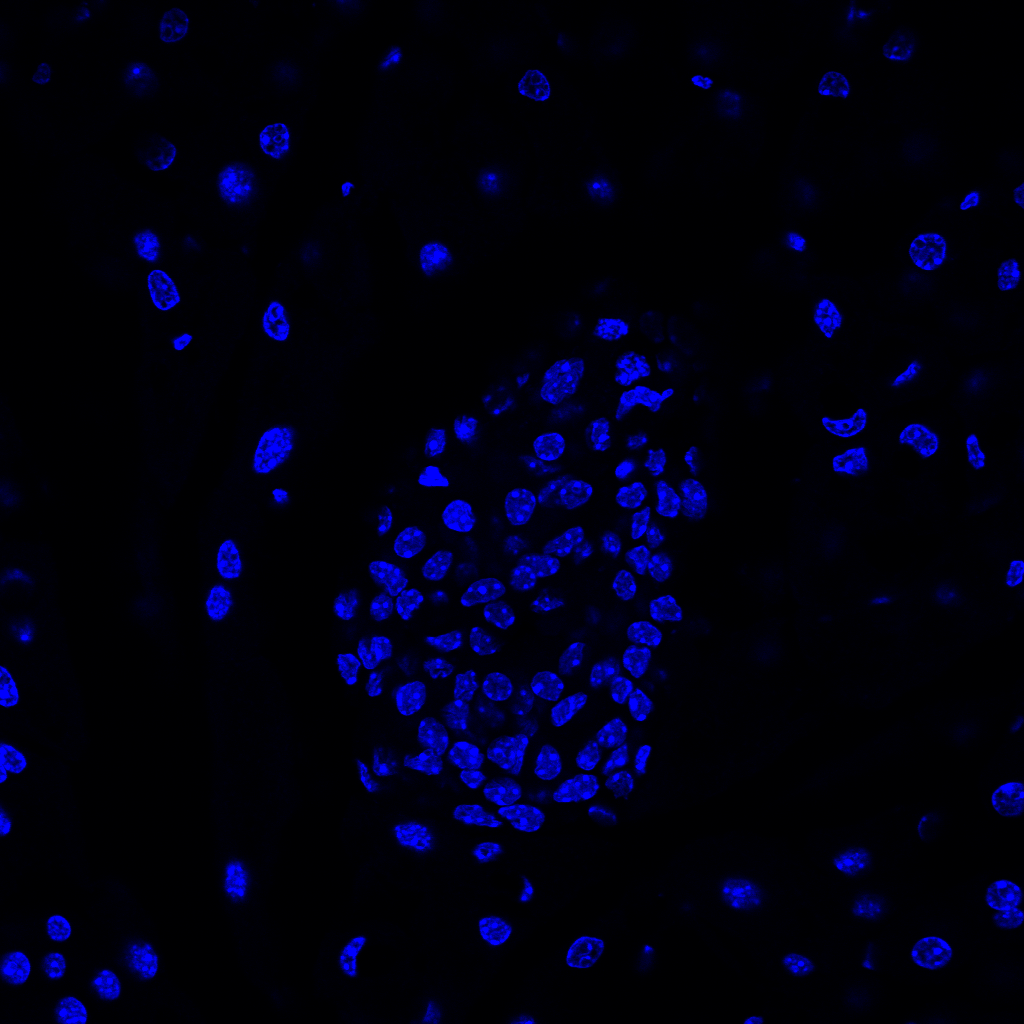

Supplement: Supplementary file 6 — Source data Fig. 4 [file 44318_2025_434_MOESM6_ESM.zip › Figure 4/4E/4E_2.tif (blue).tif]

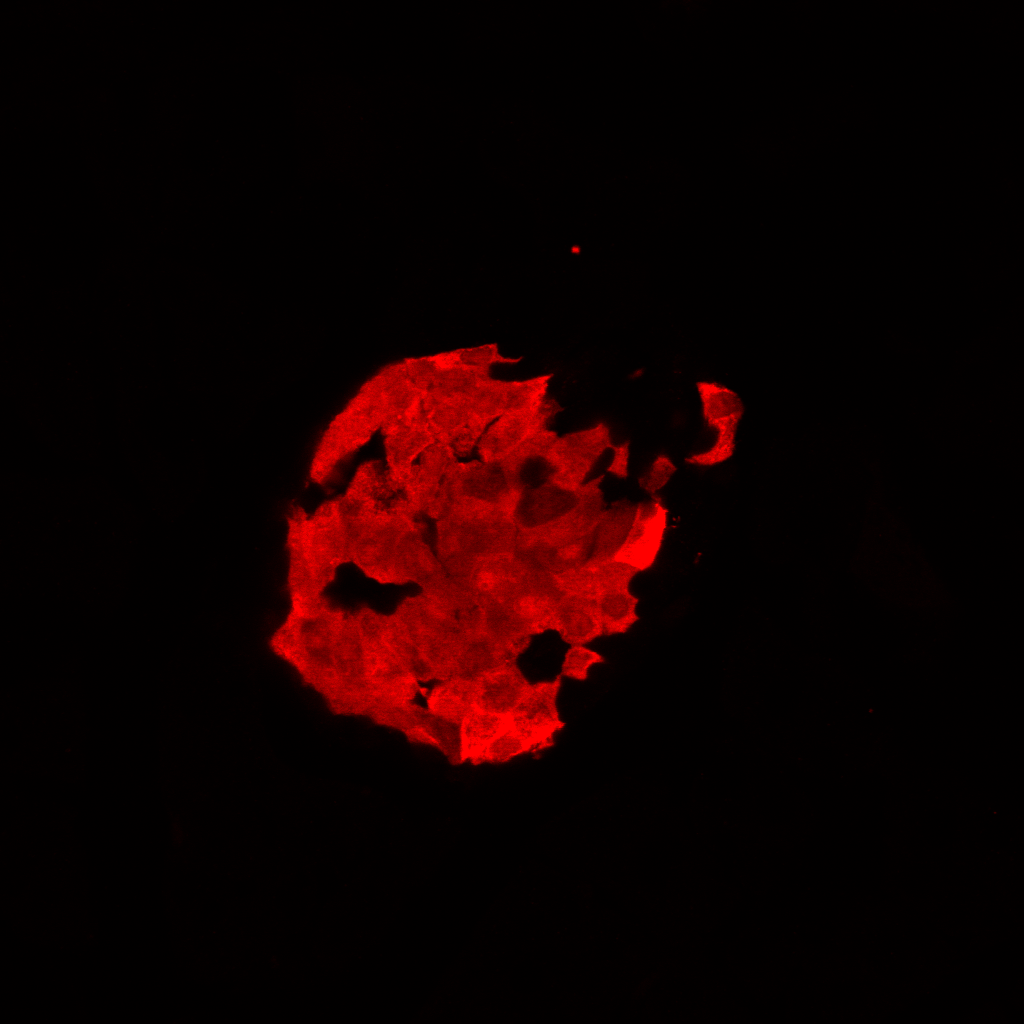

Supplement: Supplementary file 6 — Source data Fig. 4 [file 44318_2025_434_MOESM6_ESM.zip › Figure 4/4E/4E_17.tif (red).tif]
