# Supplementary figures and images for: Ductal or Ngn3+ cells do not contribute to adult pancreatic islet beta-cell neogenesis in homeostasis (part 4 of 5)
Source: EMBO J. 2025 Apr 9;44(10):2856–81. doi: 10.1038/s44318-025-00434-z (PMC12084597; doi:10.1038/s44318-025-00434-z)

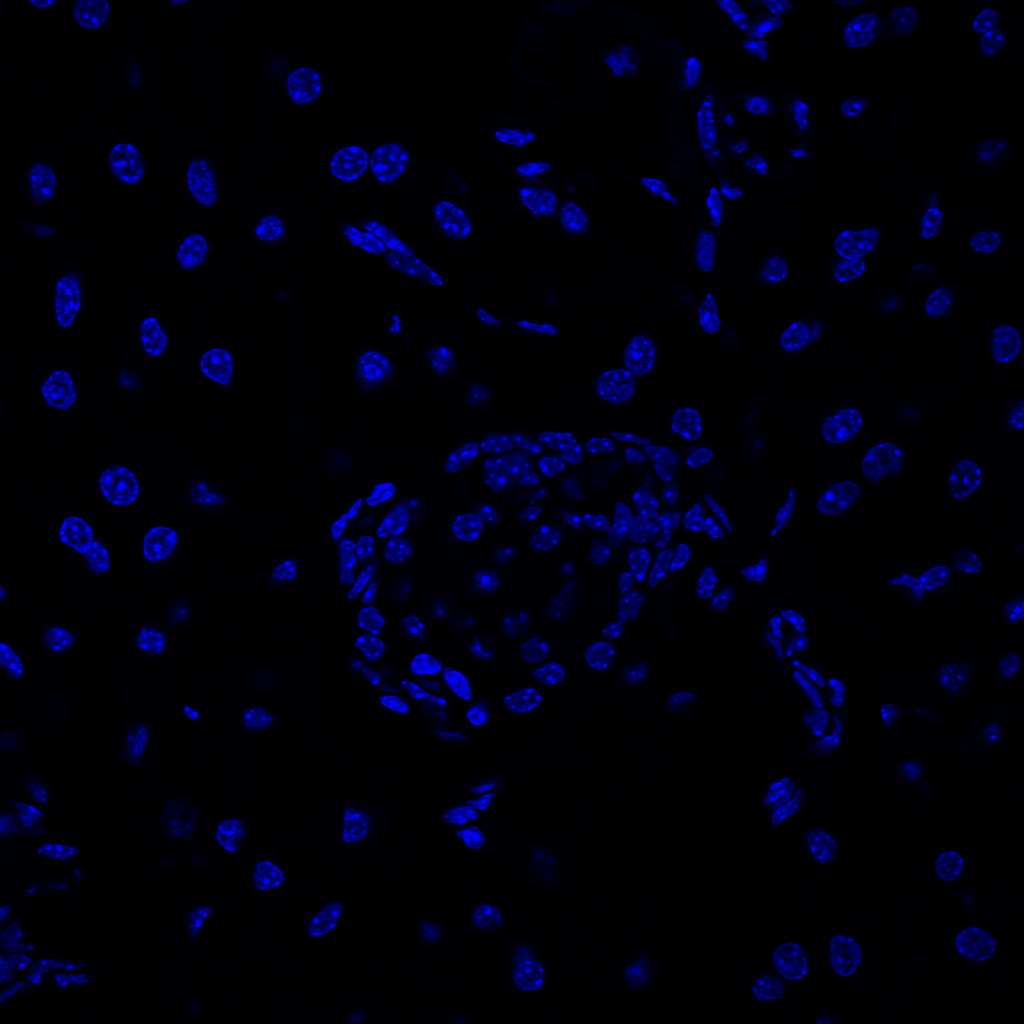

Supplement: Supplementary file 6 — Source data Fig. 4 [file 44318_2025_434_MOESM6_ESM.zip › Figure 4/4E/4E_21.tif (blue).tif]

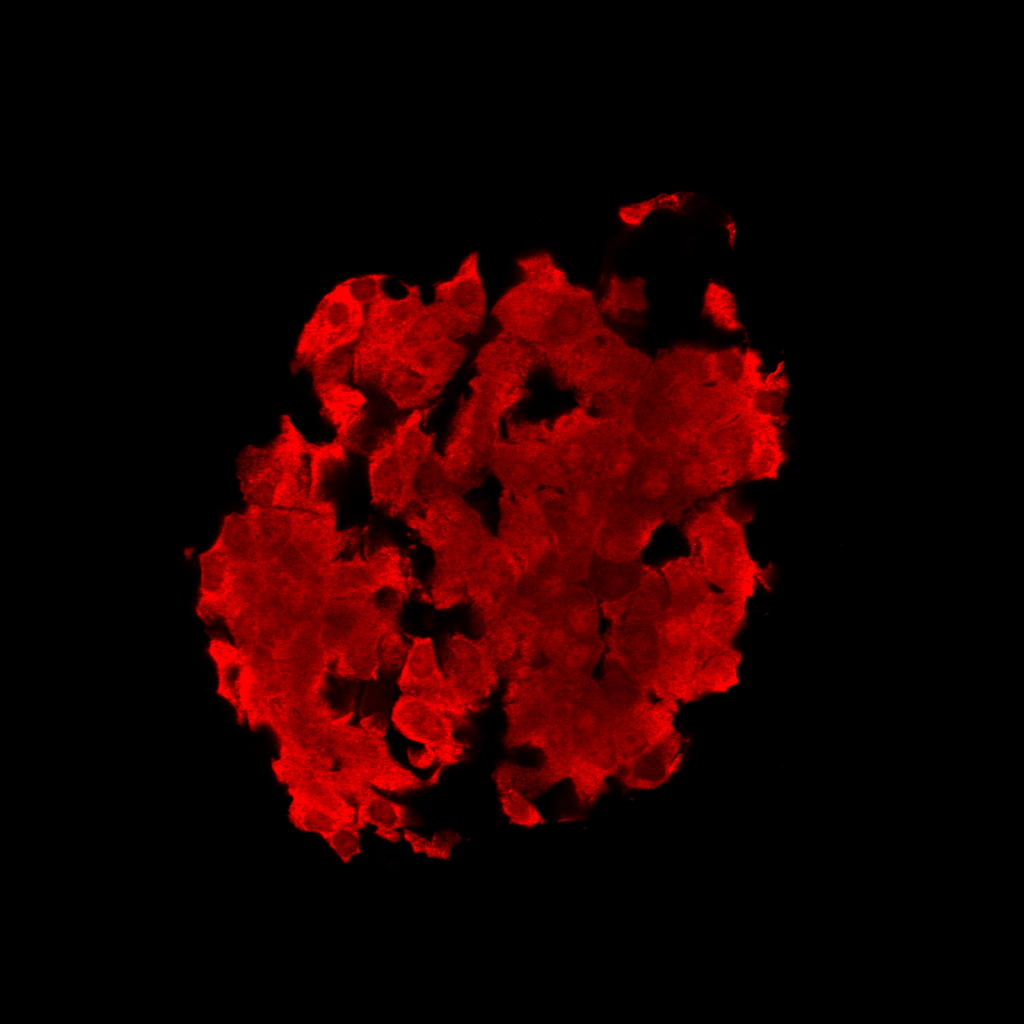

Supplement: Supplementary file 6 — Source data Fig. 4 [file 44318_2025_434_MOESM6_ESM.zip › Figure 4/4E/4E_5.tif (red).tif]

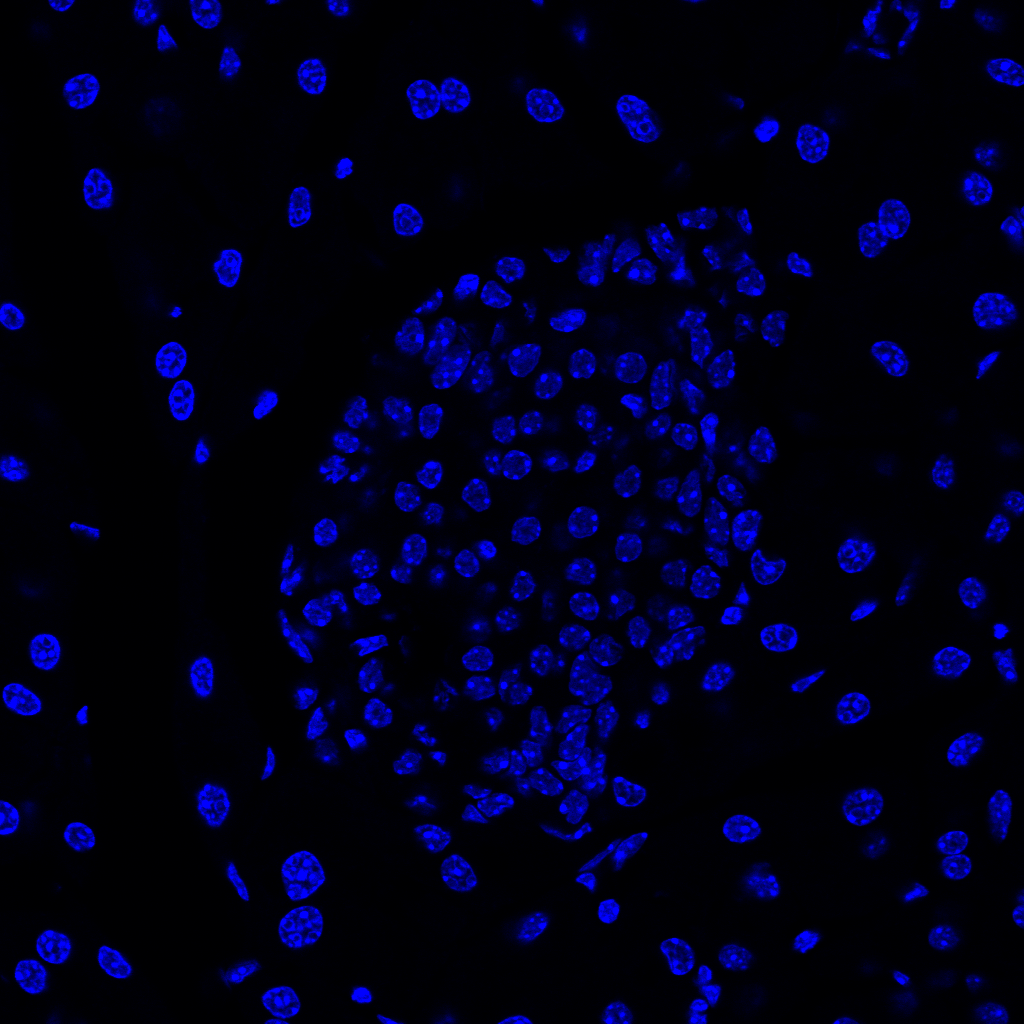

Supplement: Supplementary file 6 — Source data Fig. 4 [file 44318_2025_434_MOESM6_ESM.zip › Figure 4/4E/4E_3.tif (blue).tif]

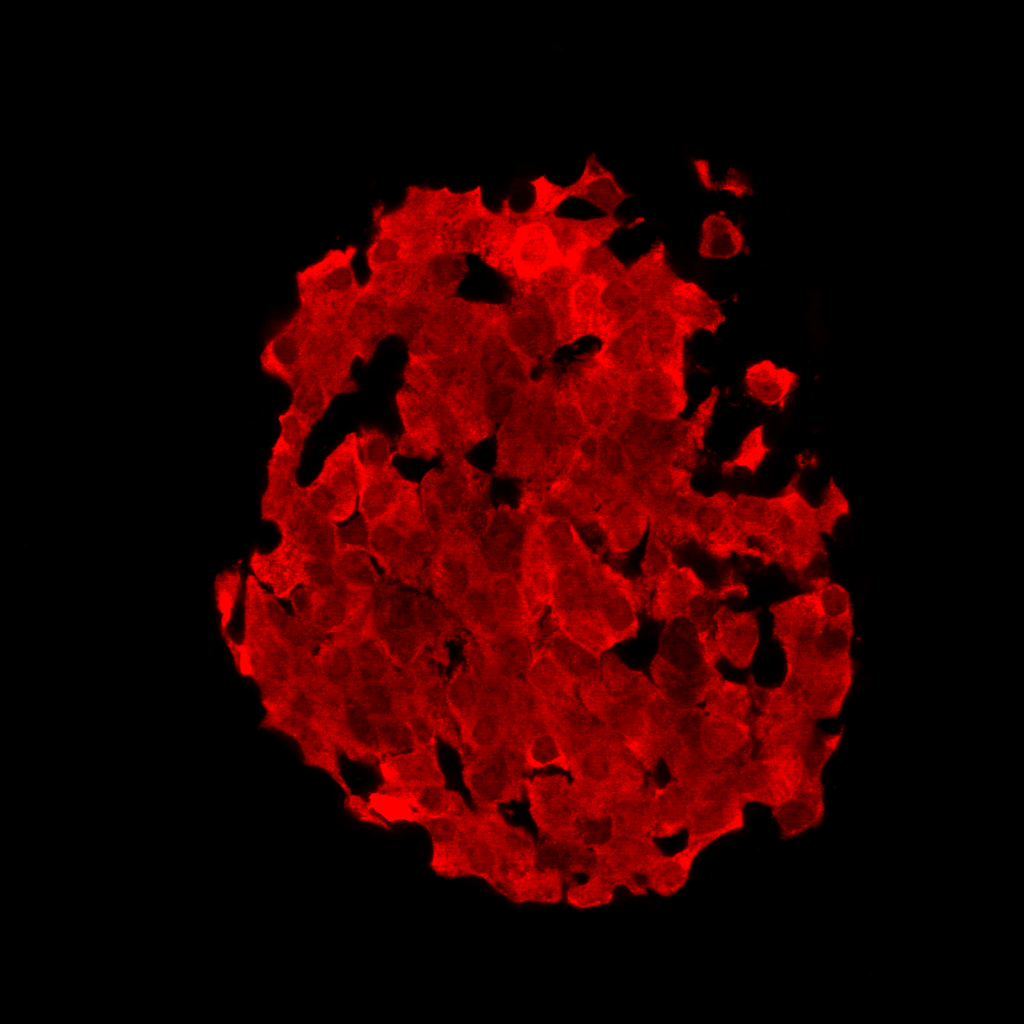

Supplement: Supplementary file 6 — Source data Fig. 4 [file 44318_2025_434_MOESM6_ESM.zip › Figure 4/4E/4E_10.tif (red).tif]

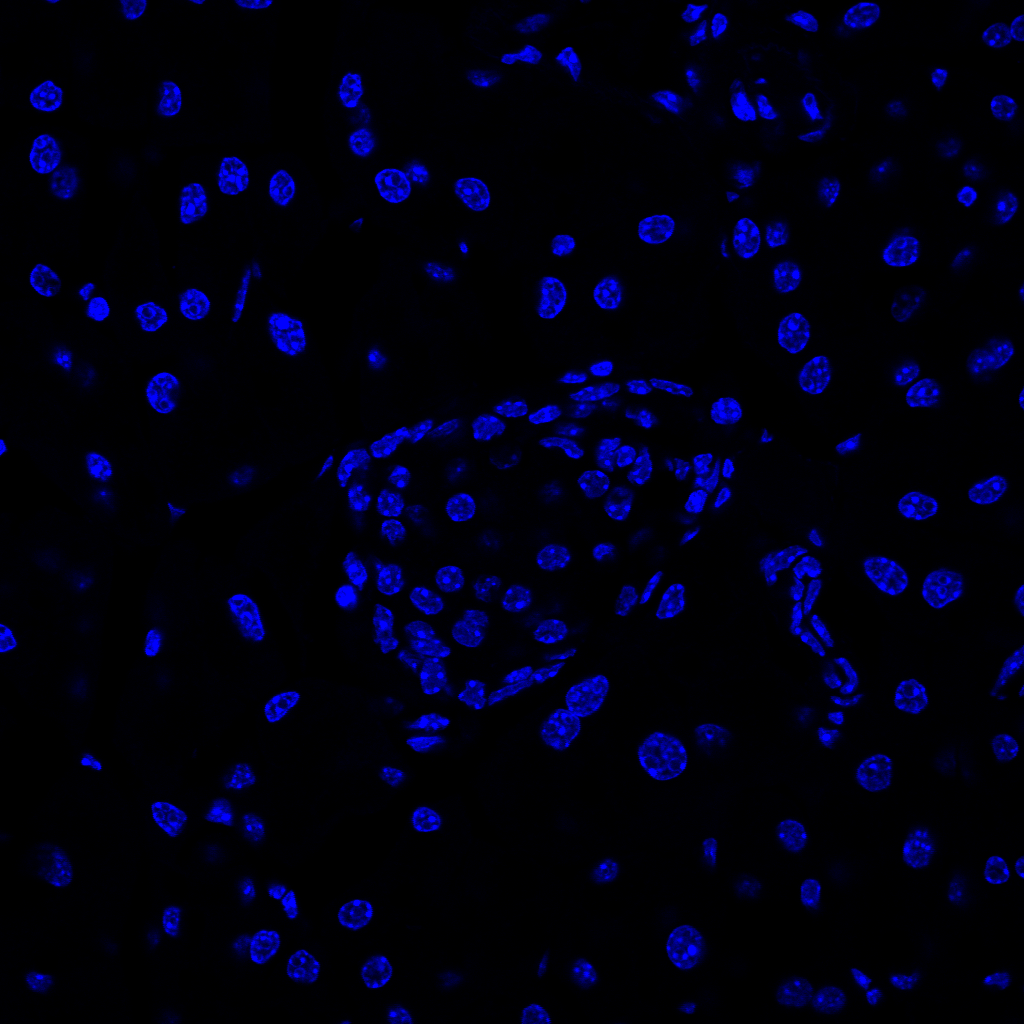

Supplement: Supplementary file 6 — Source data Fig. 4 [file 44318_2025_434_MOESM6_ESM.zip › Figure 4/4E/4E_20.tif (blue).tif]

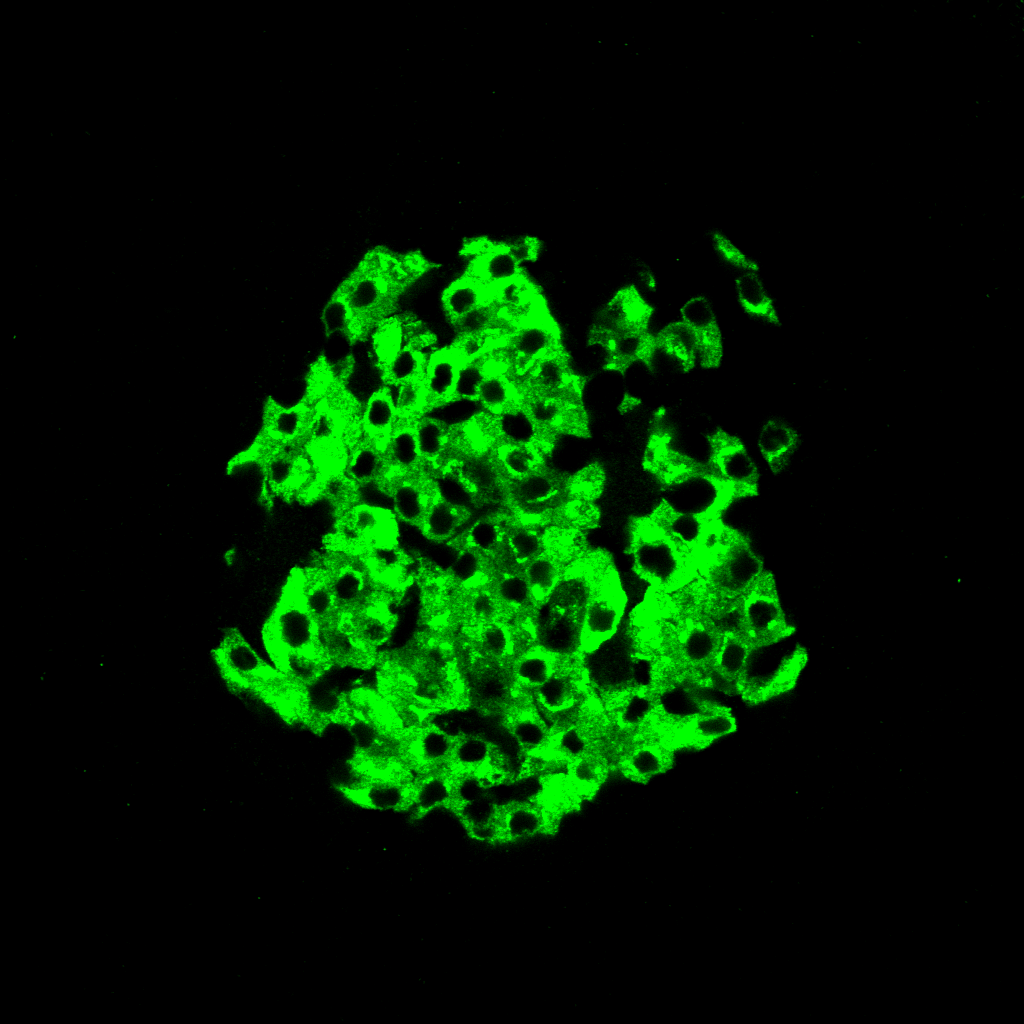

Supplement: Supplementary file 6 — Source data Fig. 4 [file 44318_2025_434_MOESM6_ESM.zip › Figure 4/4E/4E_14.tif (green).tif]

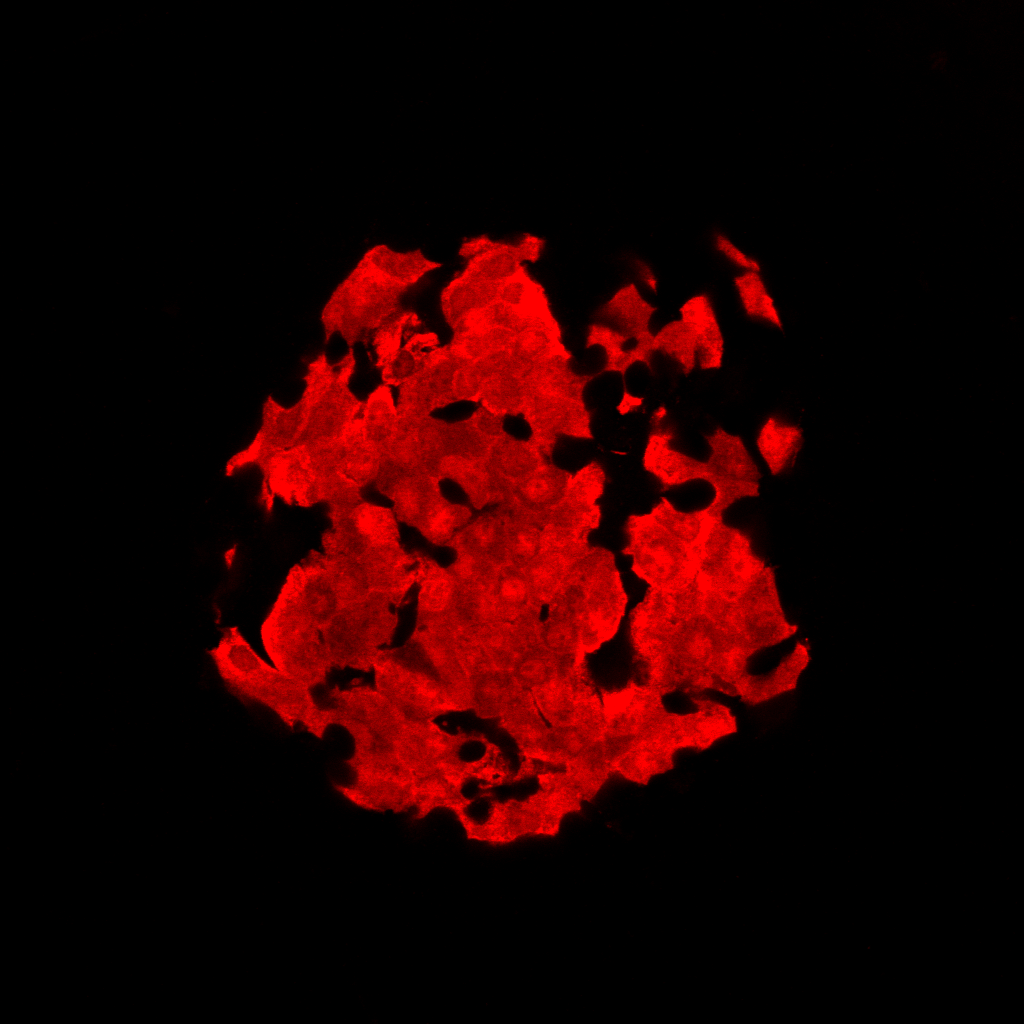

Supplement: Supplementary file 6 — Source data Fig. 4 [file 44318_2025_434_MOESM6_ESM.zip › Figure 4/4E/4E_14.tif (red).tif]

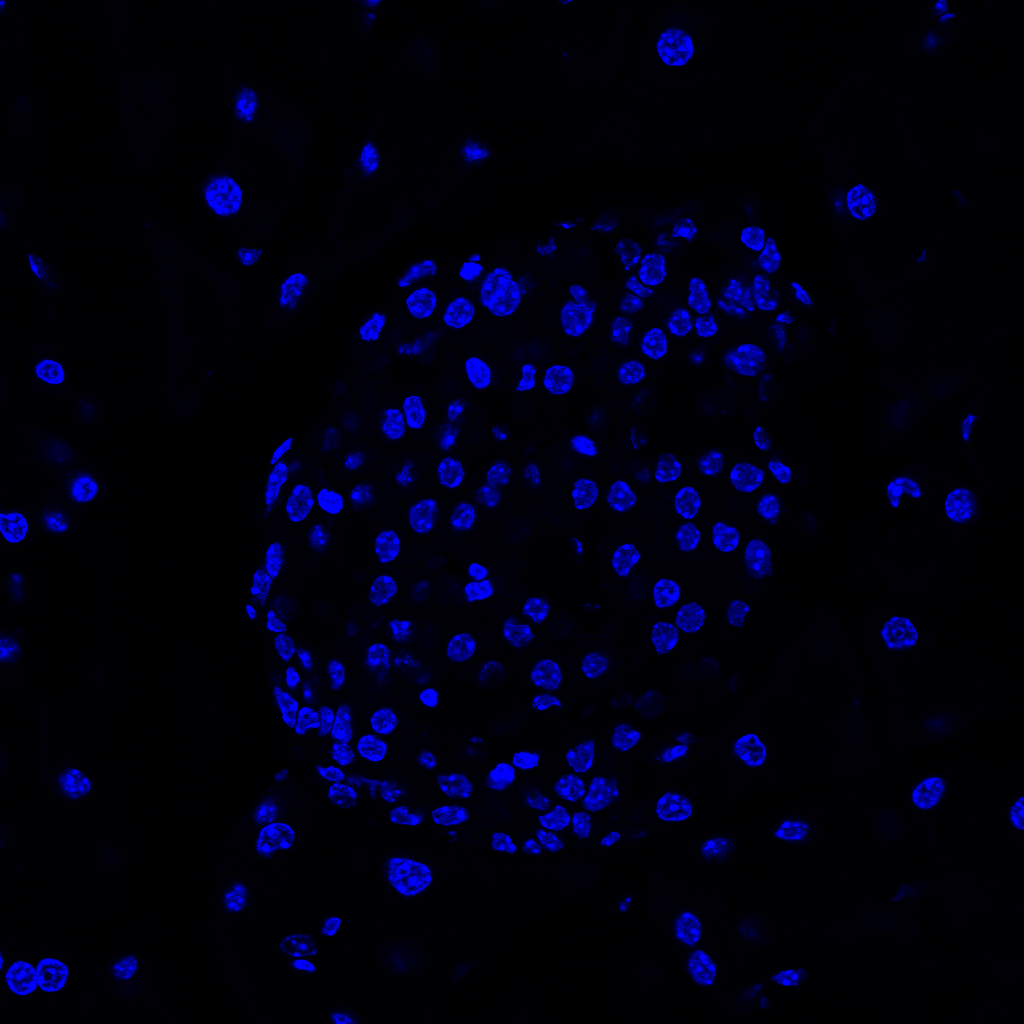

Supplement: Supplementary file 6 — Source data Fig. 4 [file 44318_2025_434_MOESM6_ESM.zip › Figure 4/4E/4E_4.tif (blue).tif]

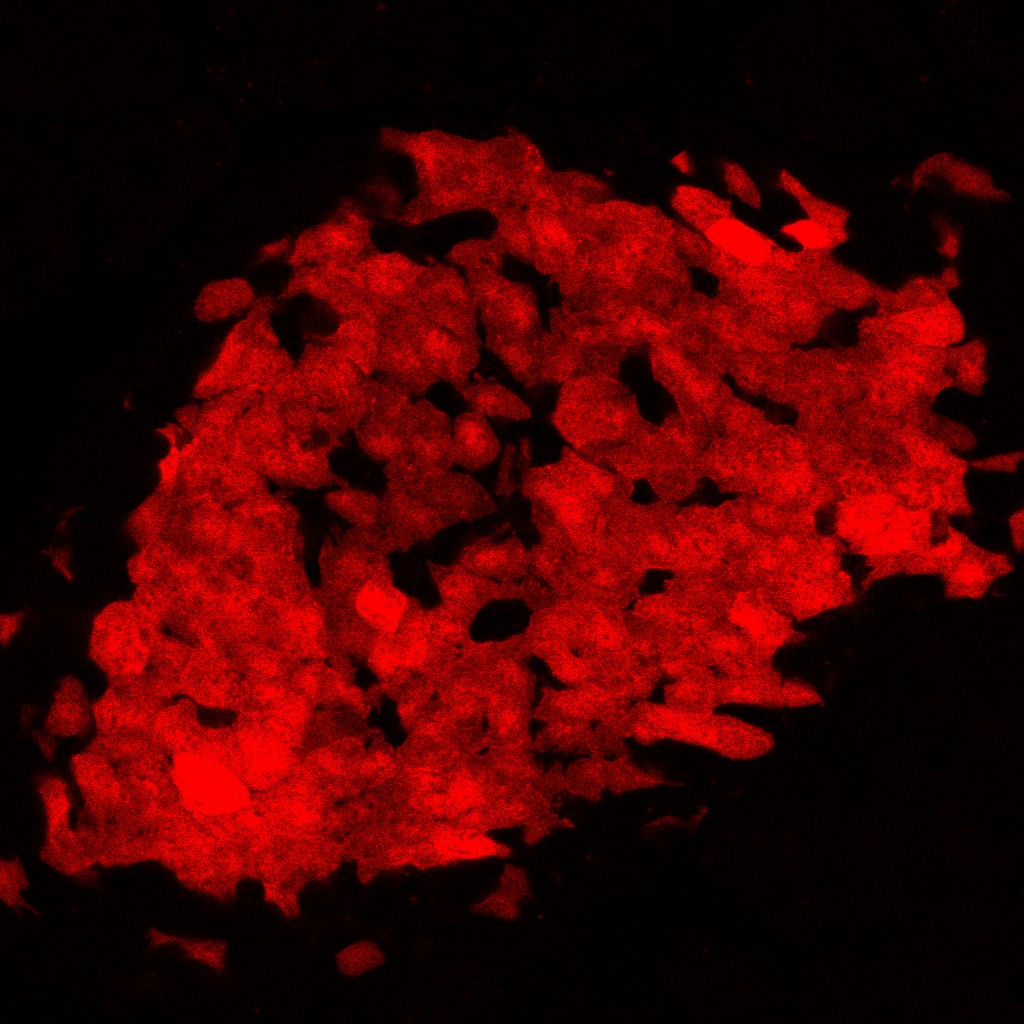

Supplement: Supplementary file 6 — Source data Fig. 4 [file 44318_2025_434_MOESM6_ESM.zip › Figure 4/4K/4K_PBS_Merge (red).tif]

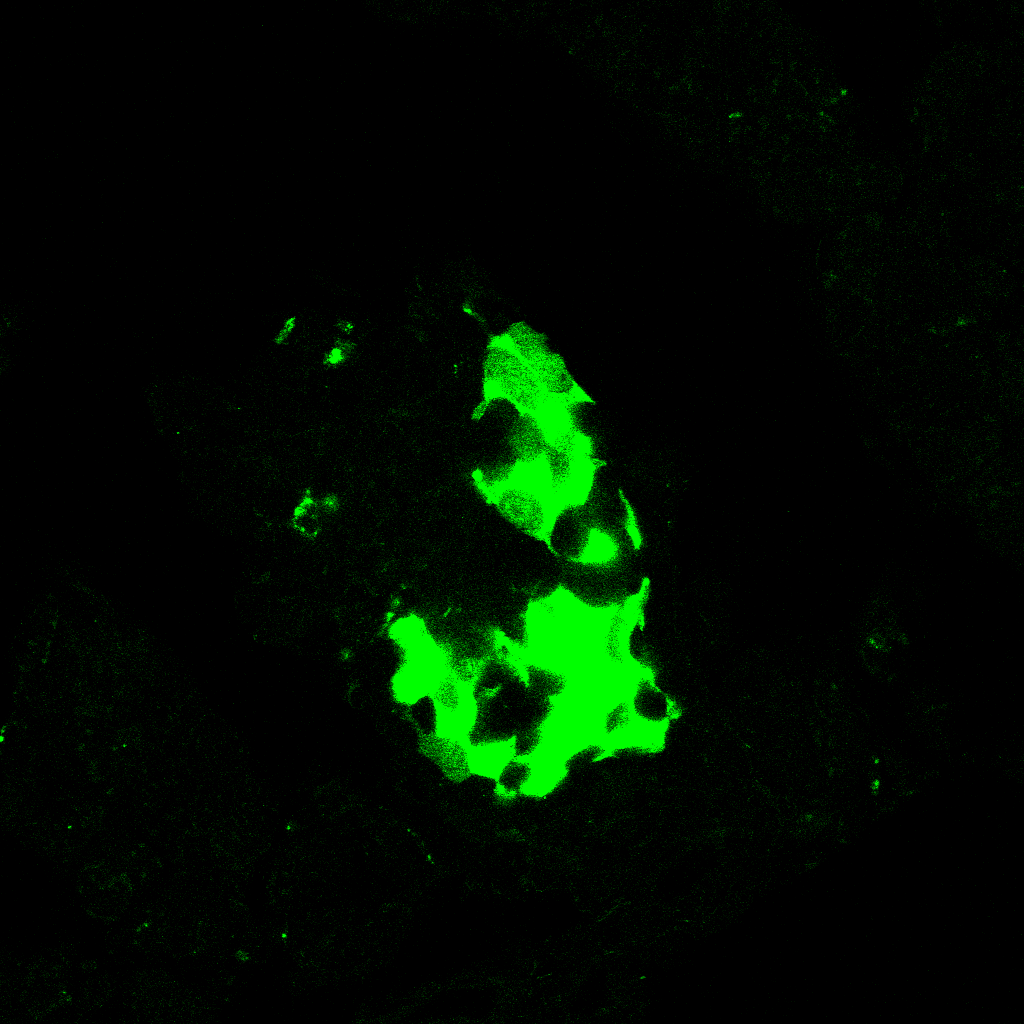

Supplement: Supplementary file 6 — Source data Fig. 4 [file 44318_2025_434_MOESM6_ESM.zip › Figure 4/4K/4K_DT_Merge (green).tif]

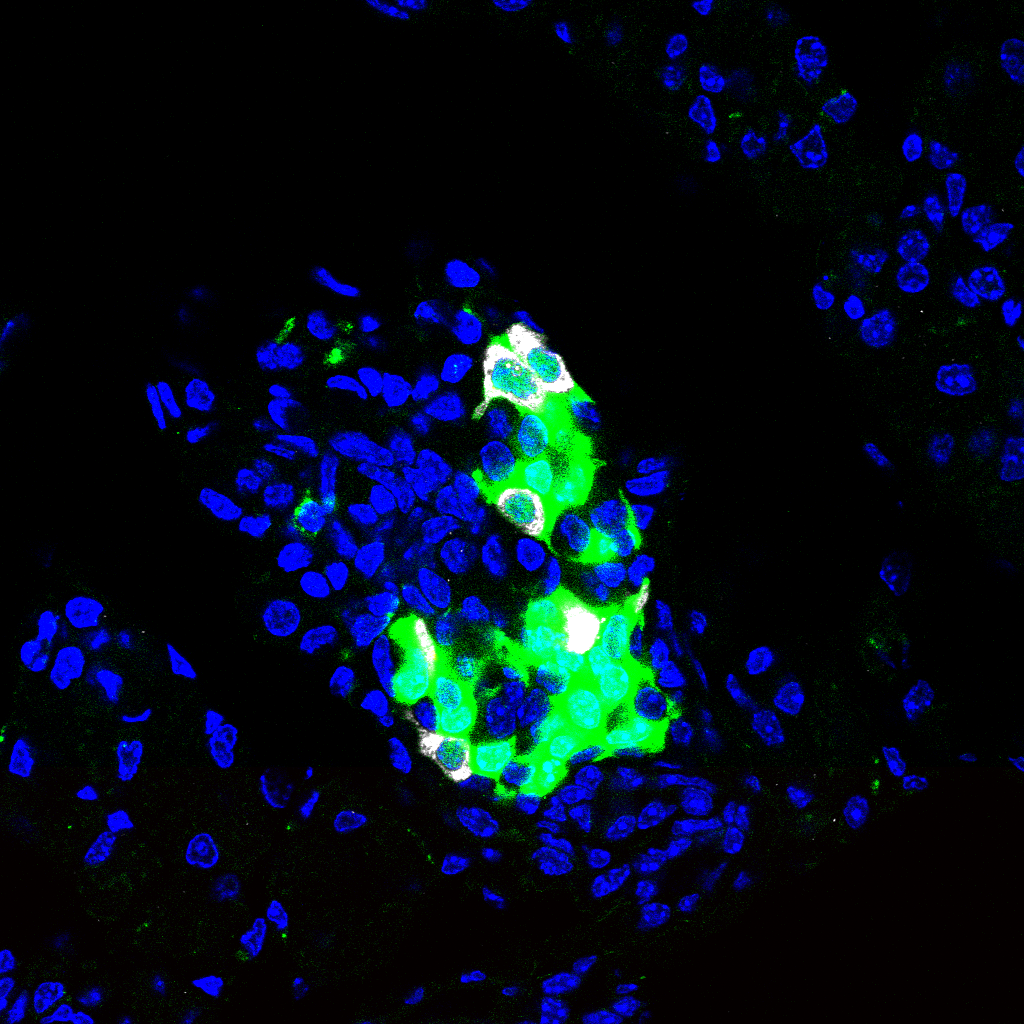

Supplement: Supplementary file 6 — Source data Fig. 4 [file 44318_2025_434_MOESM6_ESM.zip › Figure 4/4K/4K_DT_Merge.tif]

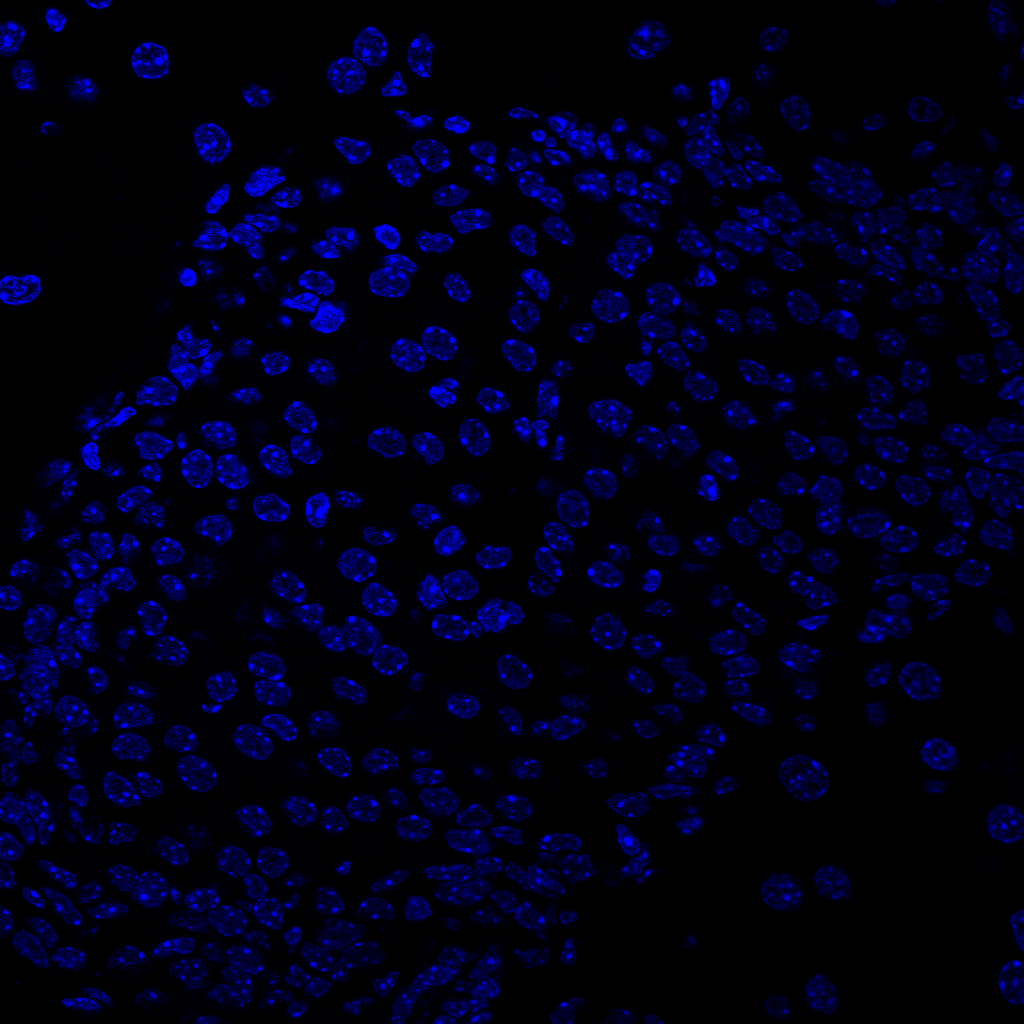

Supplement: Supplementary file 6 — Source data Fig. 4 [file 44318_2025_434_MOESM6_ESM.zip › Figure 4/4K/4K_PBS_Merge (blue).tif]

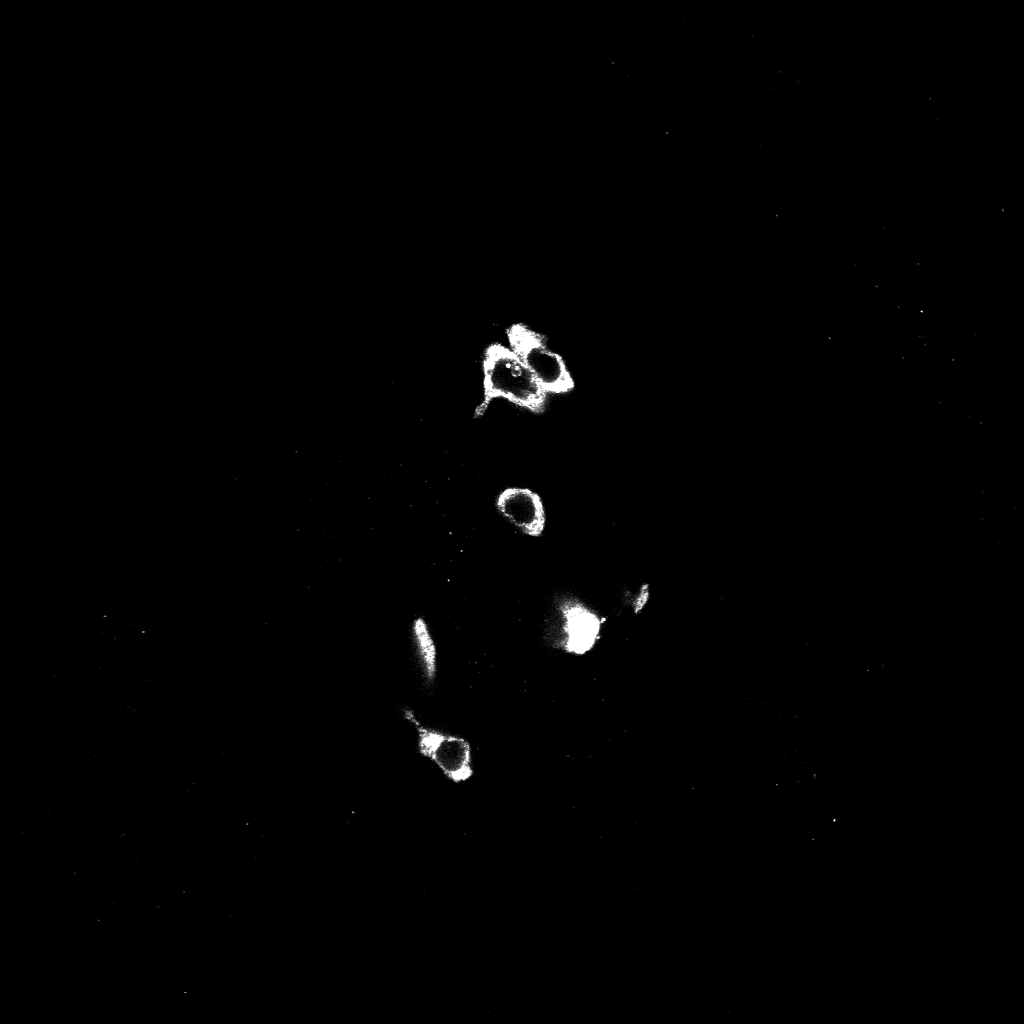

Supplement: Supplementary file 6 — Source data Fig. 4 [file 44318_2025_434_MOESM6_ESM.zip › Figure 4/4K/4K_DT_Merge (gray).tif]

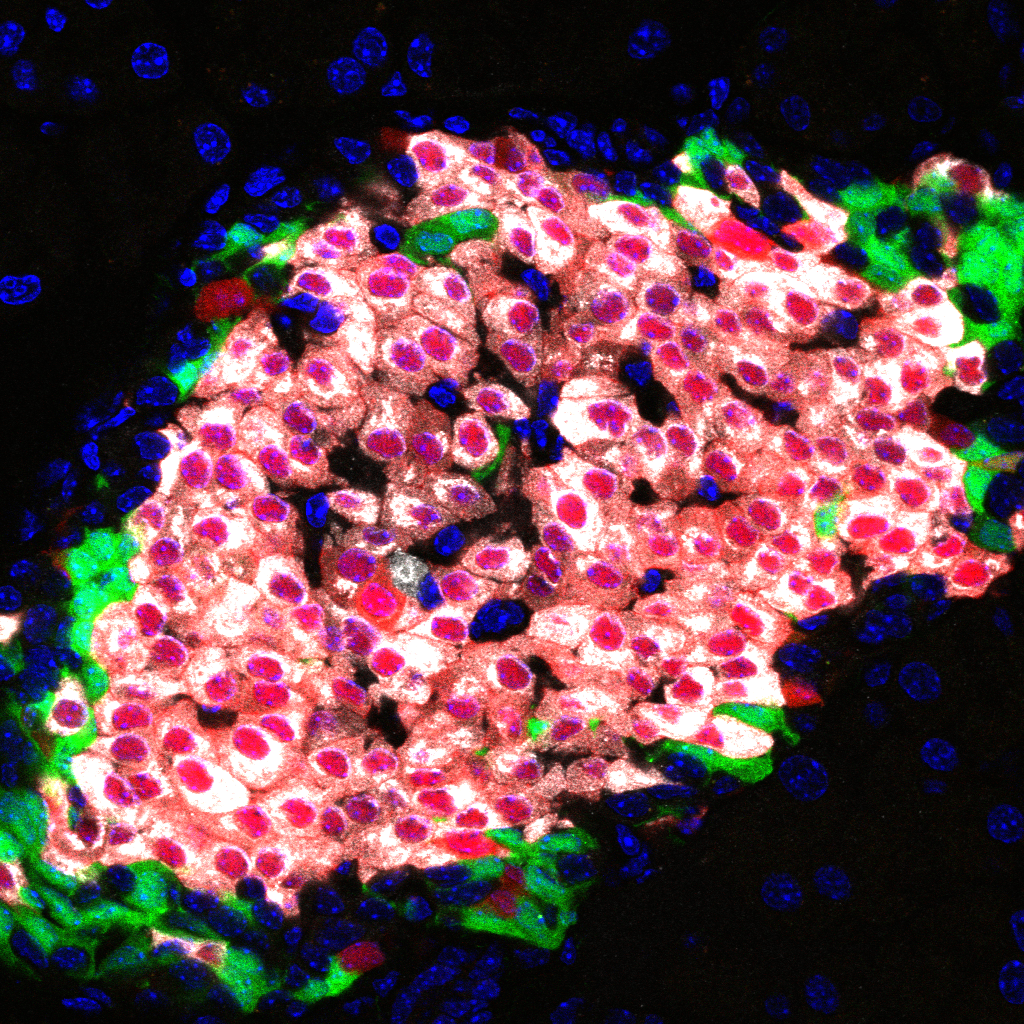

Supplement: Supplementary file 6 — Source data Fig. 4 [file 44318_2025_434_MOESM6_ESM.zip › Figure 4/4K/4K_PBS_Merge.tif]

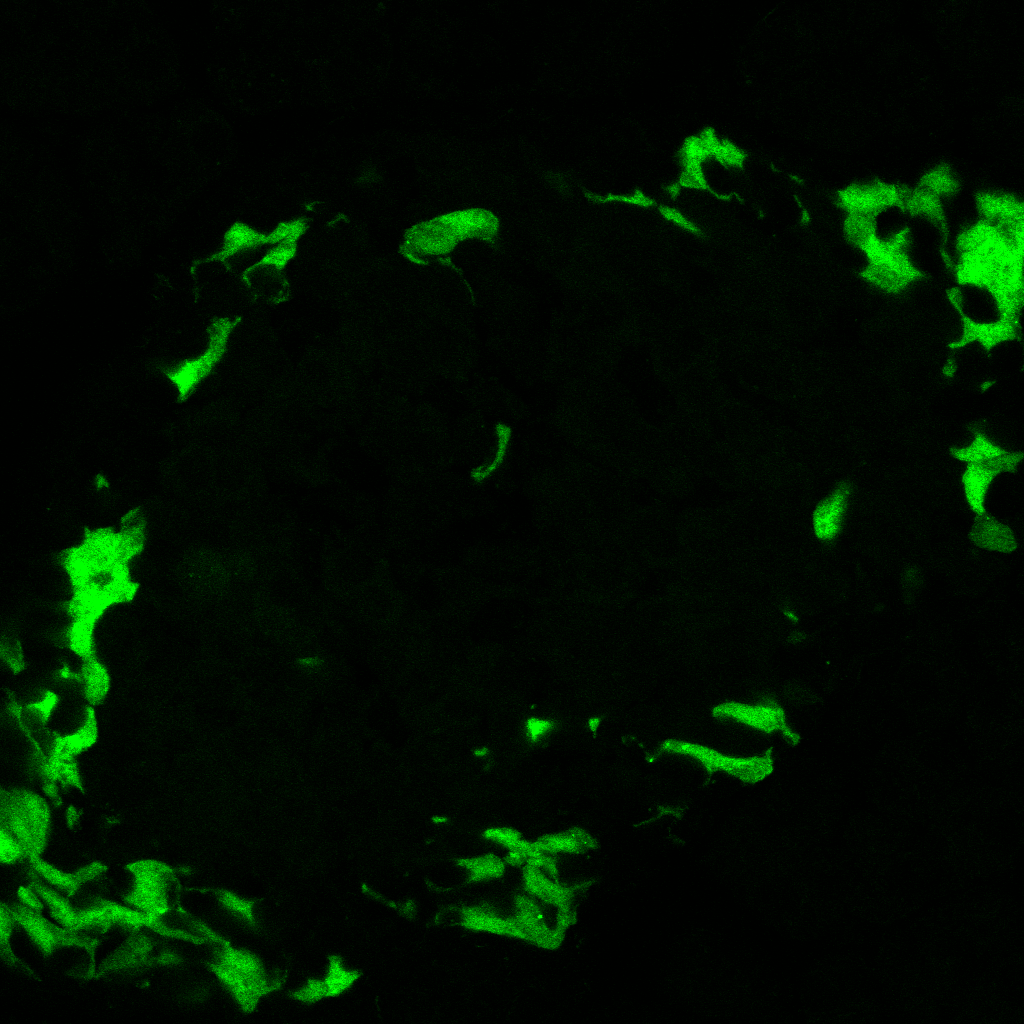

Supplement: Supplementary file 6 — Source data Fig. 4 [file 44318_2025_434_MOESM6_ESM.zip › Figure 4/4K/4K_PBS_Merge (green).tif]

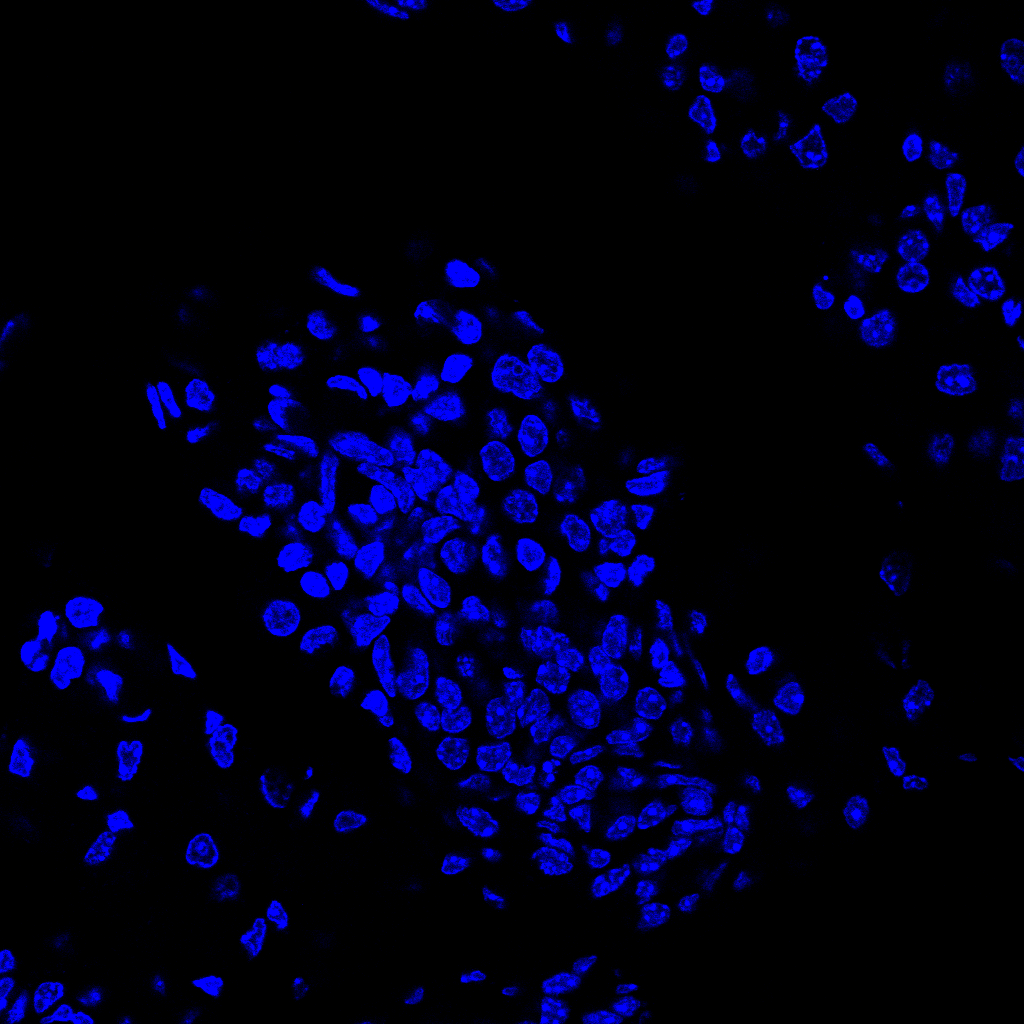

Supplement: Supplementary file 6 — Source data Fig. 4 [file 44318_2025_434_MOESM6_ESM.zip › Figure 4/4K/4K_DT_Merge (blue).tif]

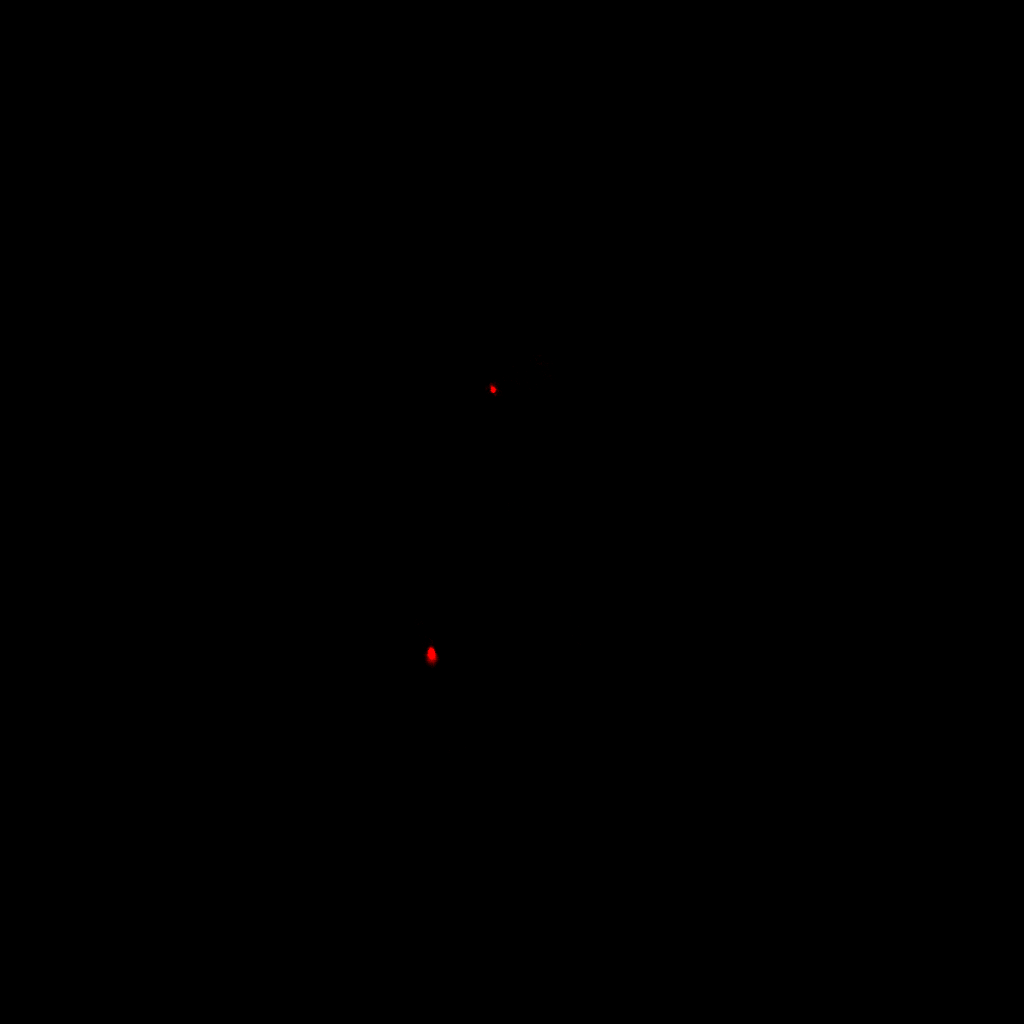

Supplement: Supplementary file 6 — Source data Fig. 4 [file 44318_2025_434_MOESM6_ESM.zip › Figure 4/4K/4K_DT_Merge (red).tif]

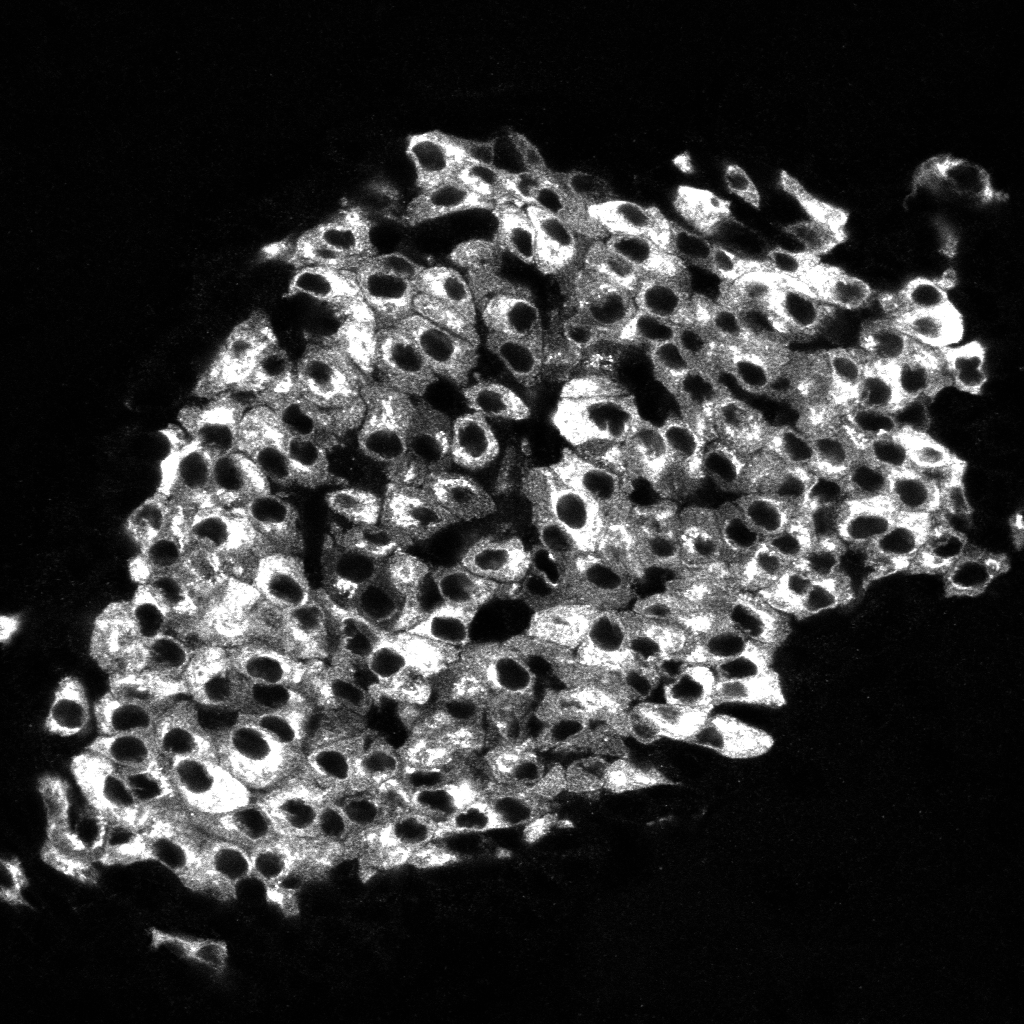

Supplement: Supplementary file 6 — Source data Fig. 4 [file 44318_2025_434_MOESM6_ESM.zip › Figure 4/4K/4K_PBS_Merge (gray).tif]

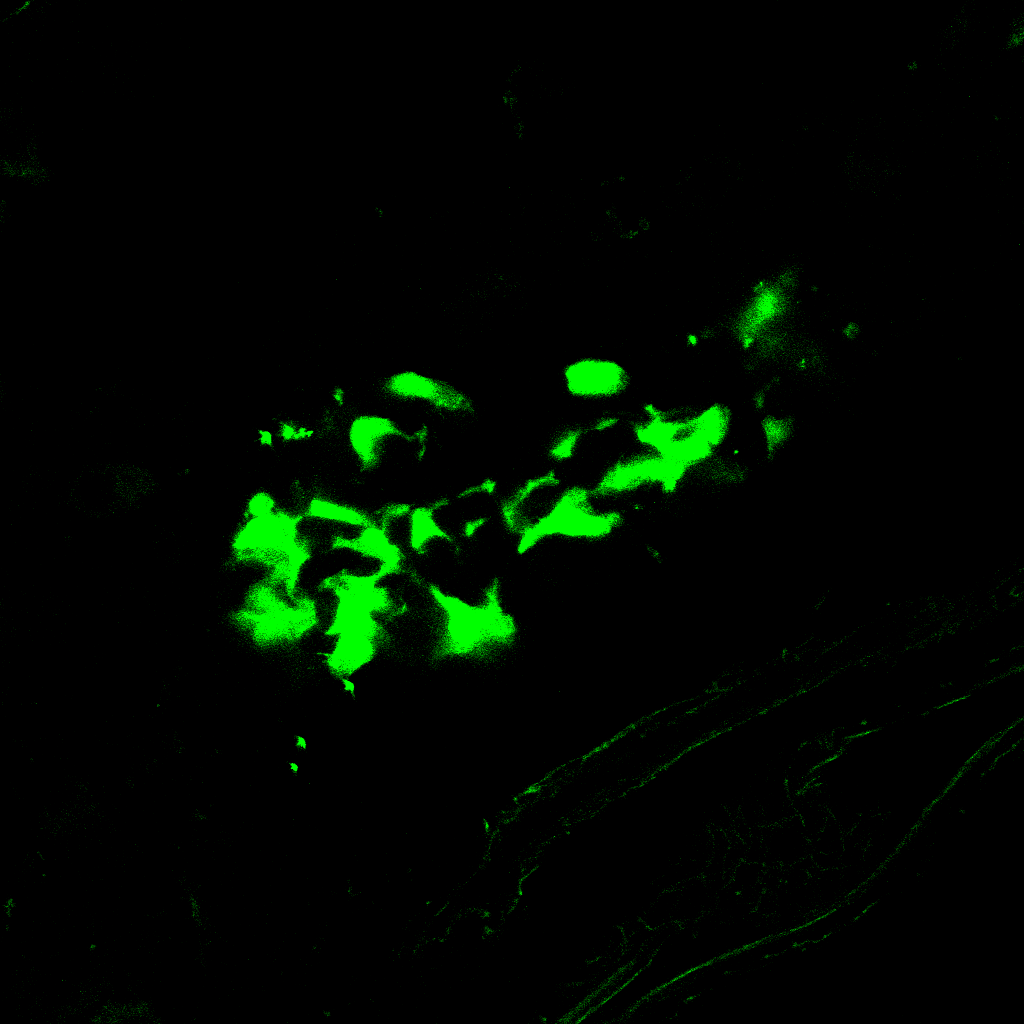

Supplement: Supplementary file 6 — Source data Fig. 4 [file 44318_2025_434_MOESM6_ESM.zip › Figure 4/4M/4M_Nkx6.1_Merge (green).tif]

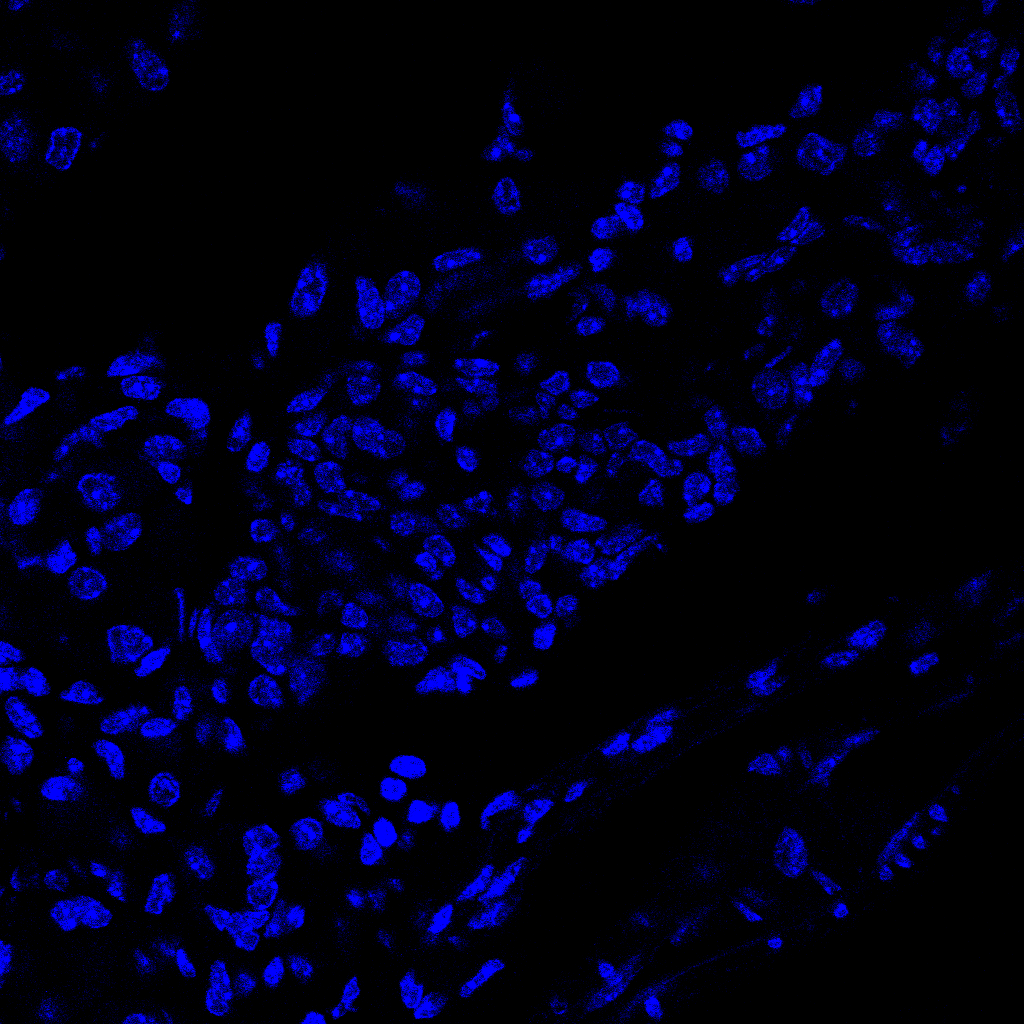

Supplement: Supplementary file 6 — Source data Fig. 4 [file 44318_2025_434_MOESM6_ESM.zip › Figure 4/4M/4M_Nkx6.1_Merge (blue).tif]

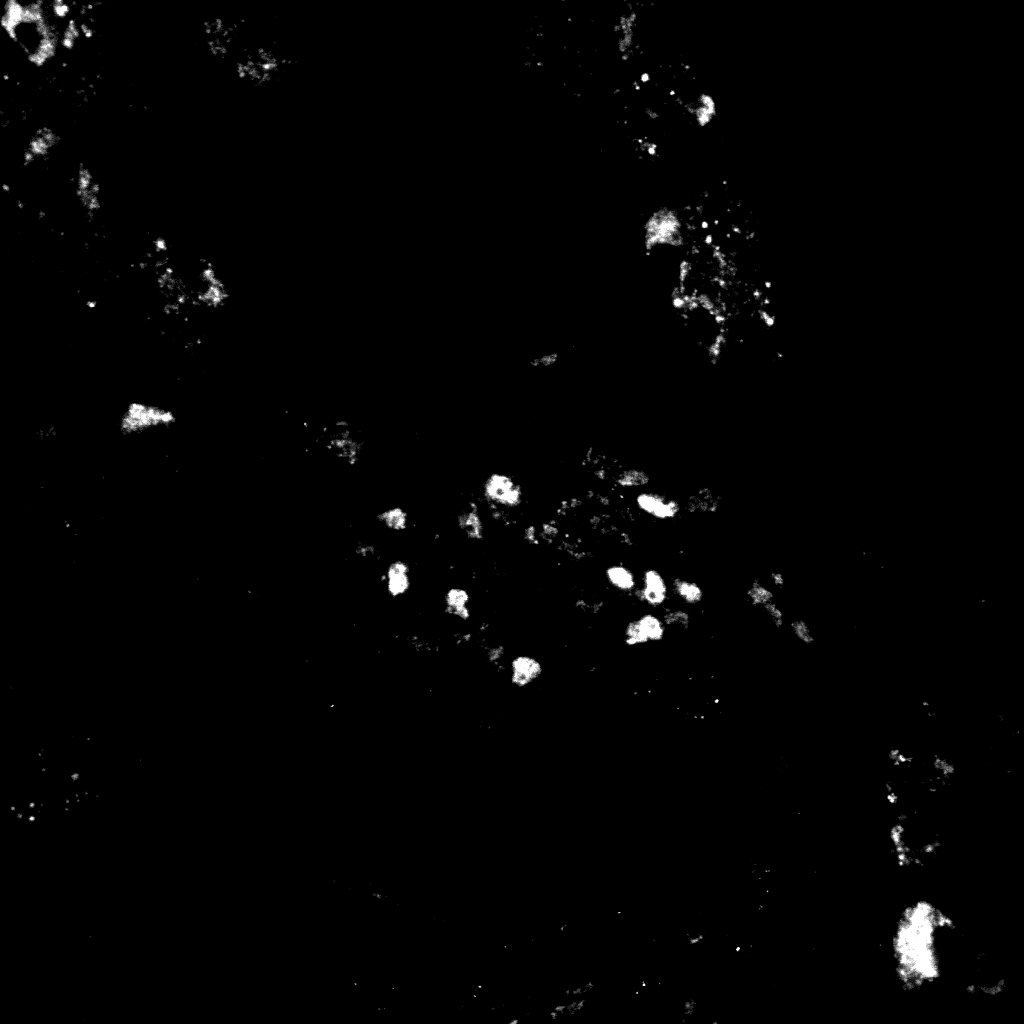

Supplement: Supplementary file 6 — Source data Fig. 4 [file 44318_2025_434_MOESM6_ESM.zip › Figure 4/4M/4M_Pdx1_Merge (gray).tif]

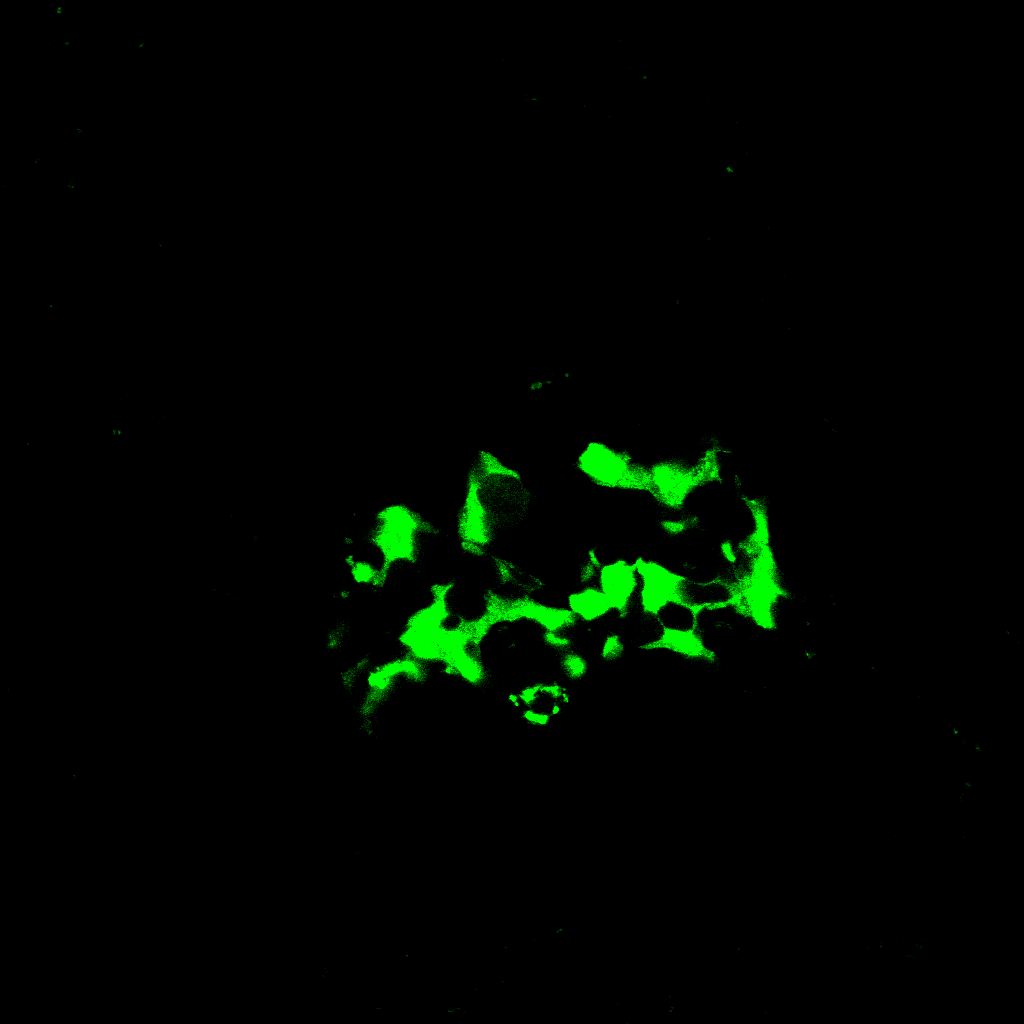

Supplement: Supplementary file 6 — Source data Fig. 4 [file 44318_2025_434_MOESM6_ESM.zip › Figure 4/4M/4M_Pdx1_Merge (green).tif]

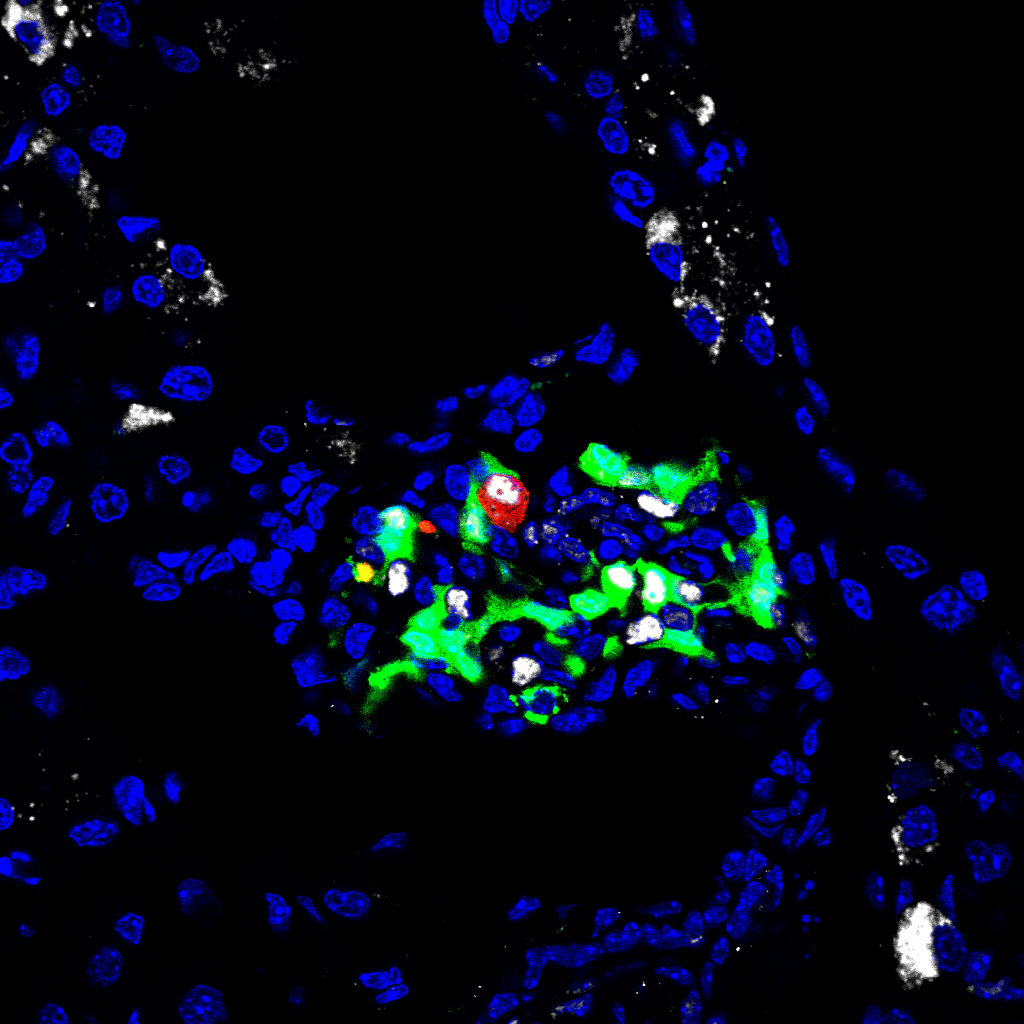

Supplement: Supplementary file 6 — Source data Fig. 4 [file 44318_2025_434_MOESM6_ESM.zip › Figure 4/4M/4M_Pdx1_Merge.tif]

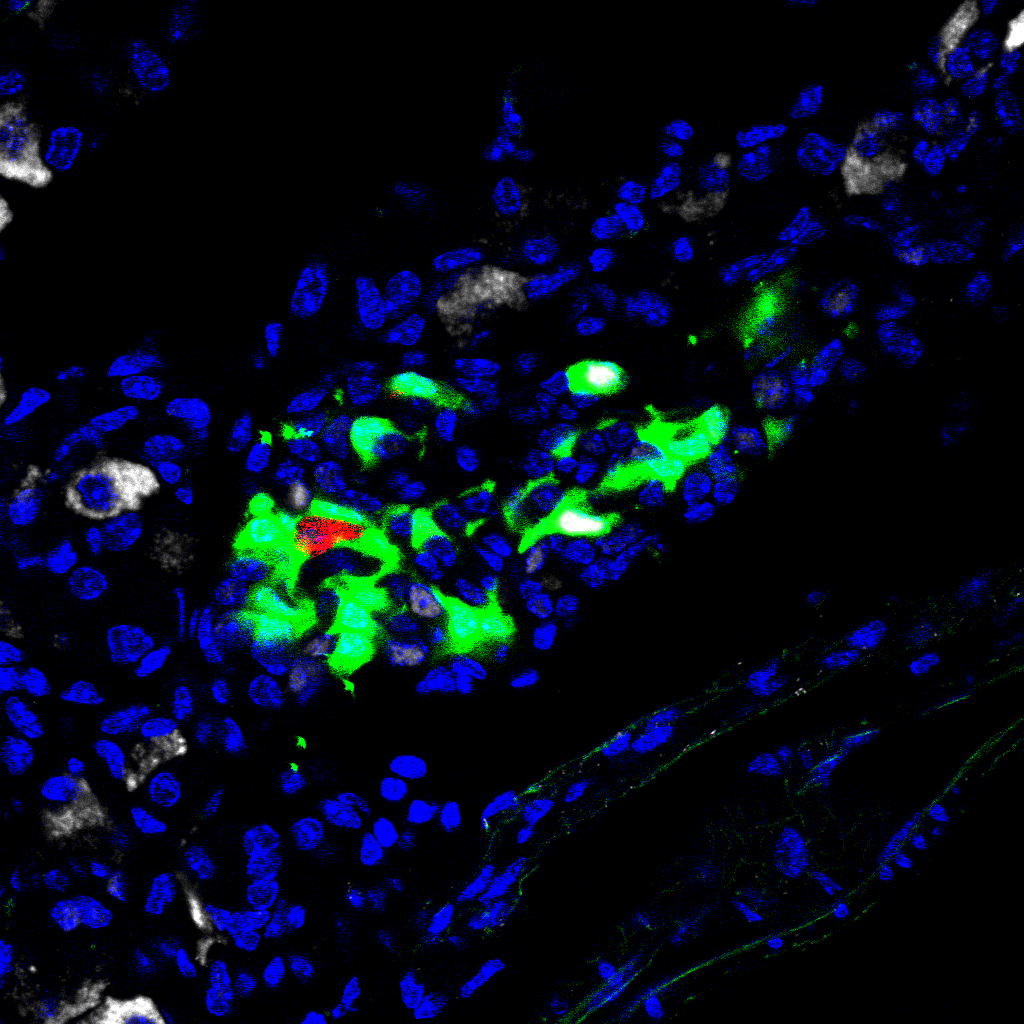

Supplement: Supplementary file 6 — Source data Fig. 4 [file 44318_2025_434_MOESM6_ESM.zip › Figure 4/4M/4M_Nkx6.1_Merge.tif]

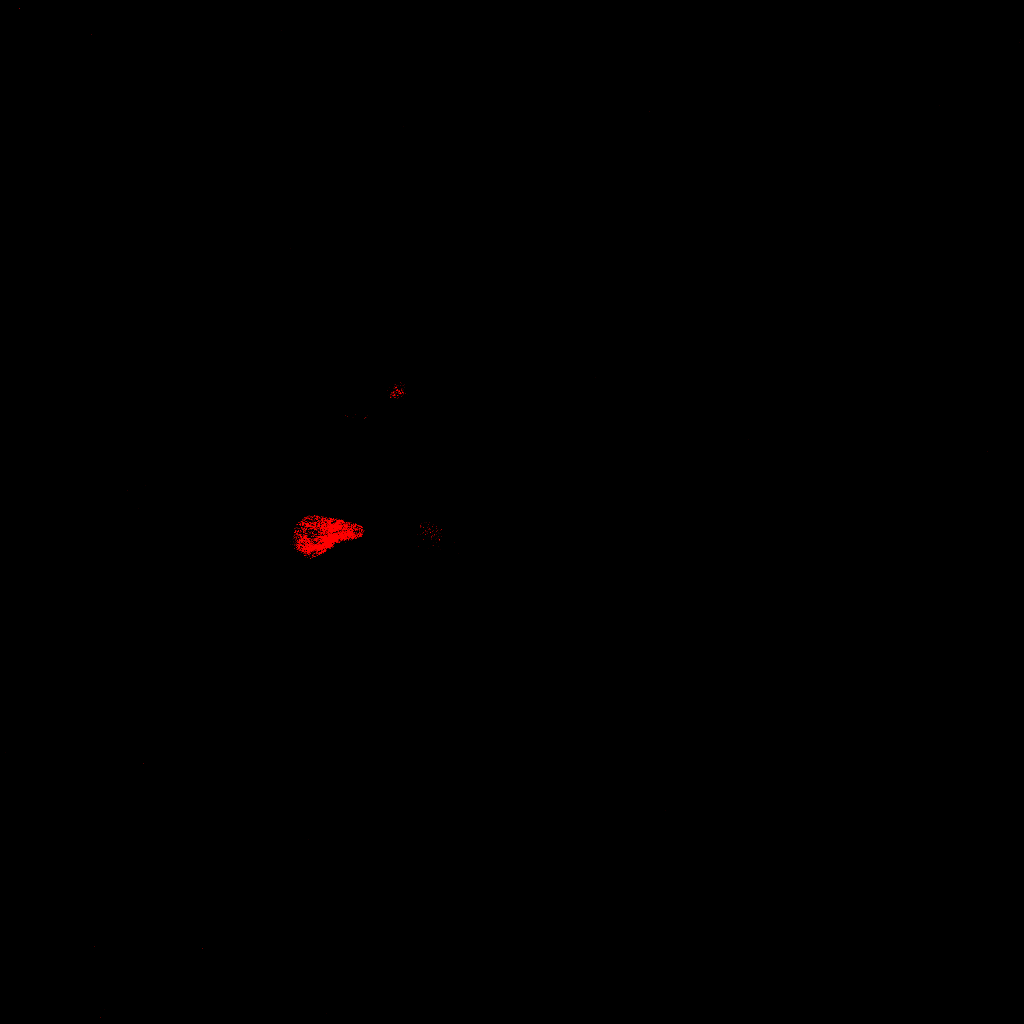

Supplement: Supplementary file 6 — Source data Fig. 4 [file 44318_2025_434_MOESM6_ESM.zip › Figure 4/4M/4M_Nkx6.1_Merge (red).tif]

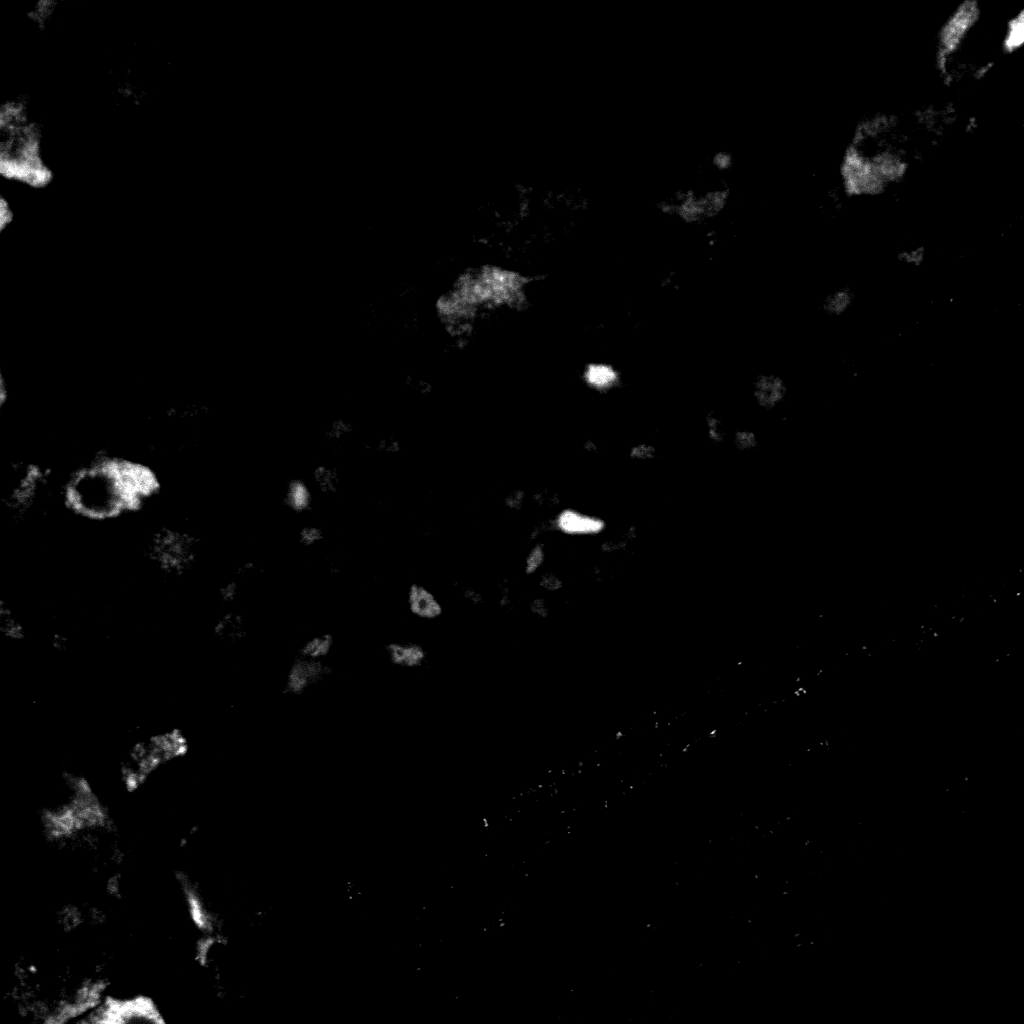

Supplement: Supplementary file 6 — Source data Fig. 4 [file 44318_2025_434_MOESM6_ESM.zip › Figure 4/4M/4M_Nkx6.1_Merge (gray).tif]

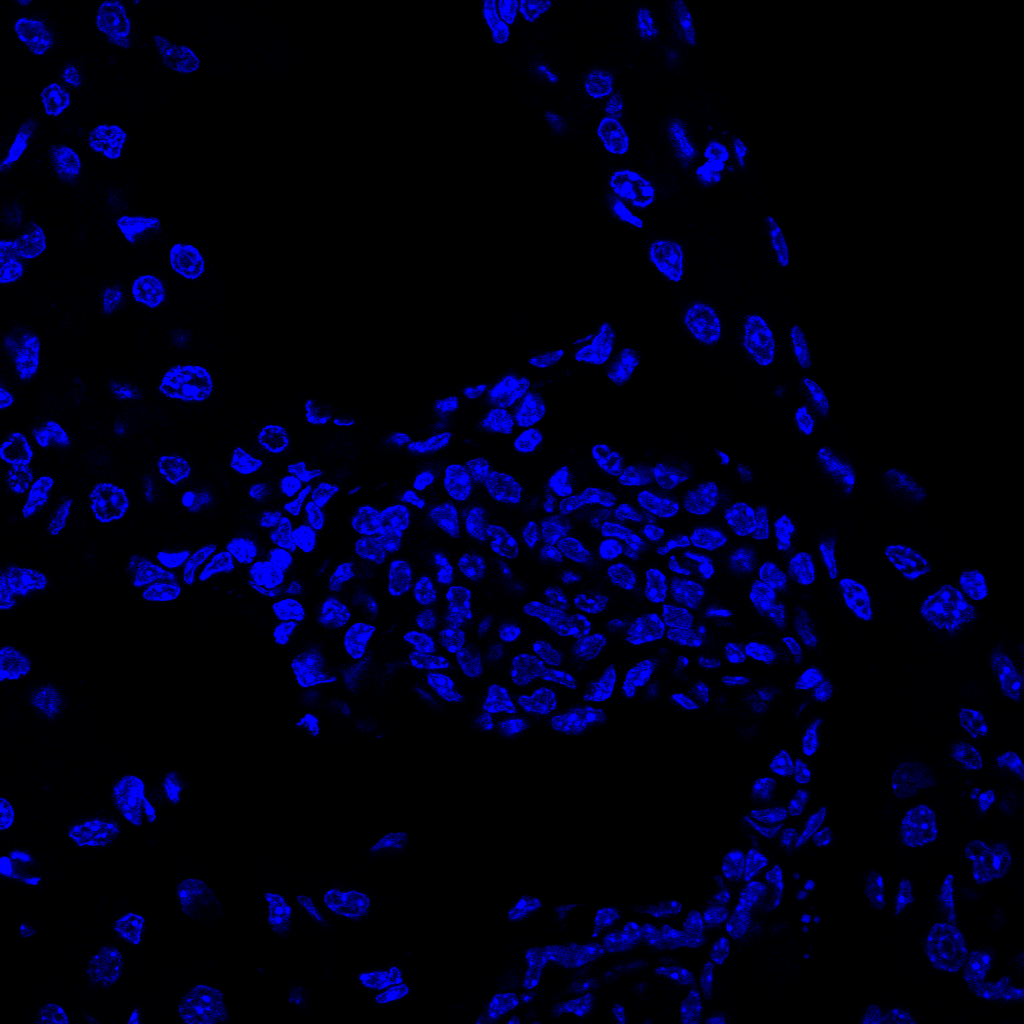

Supplement: Supplementary file 6 — Source data Fig. 4 [file 44318_2025_434_MOESM6_ESM.zip › Figure 4/4M/4M_Pdx1_Merge (blue).tif]

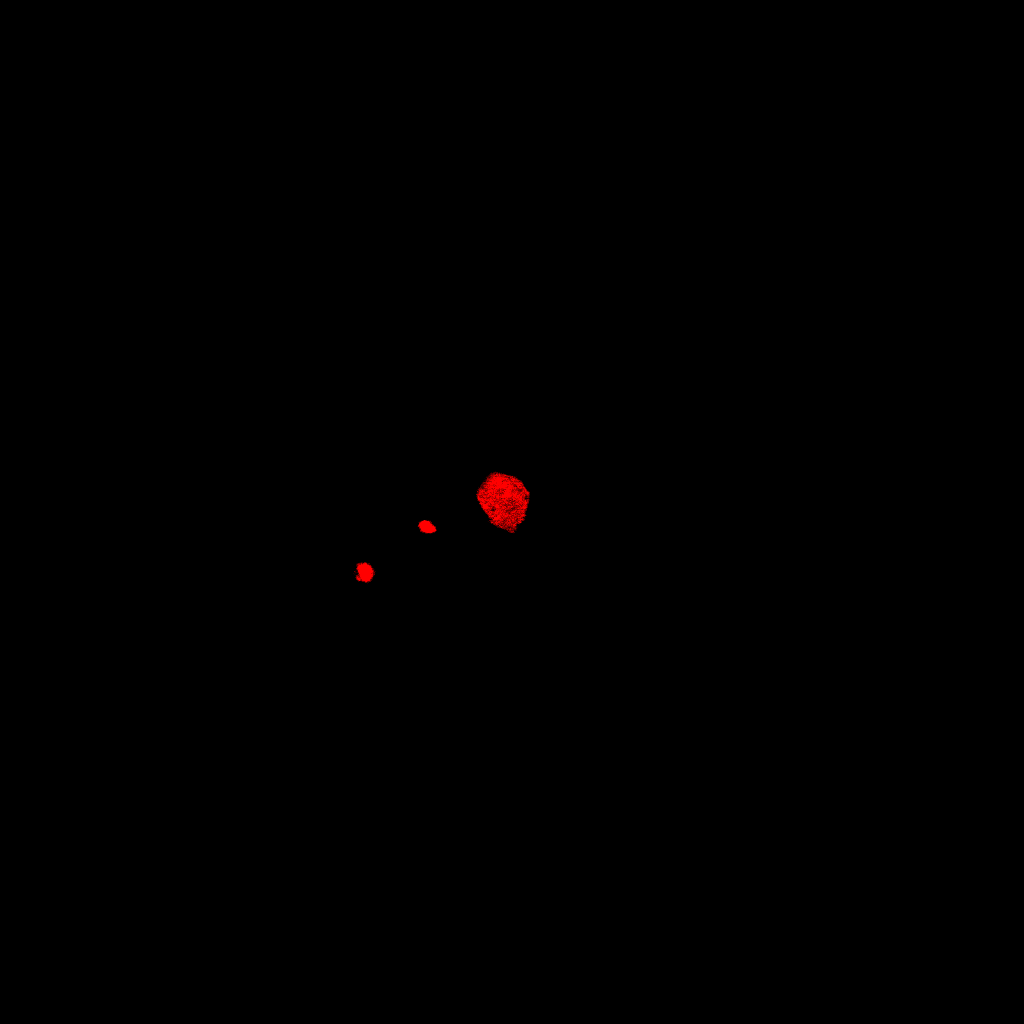

Supplement: Supplementary file 6 — Source data Fig. 4 [file 44318_2025_434_MOESM6_ESM.zip › Figure 4/4M/4M_Pdx1_Merge (red).tif]

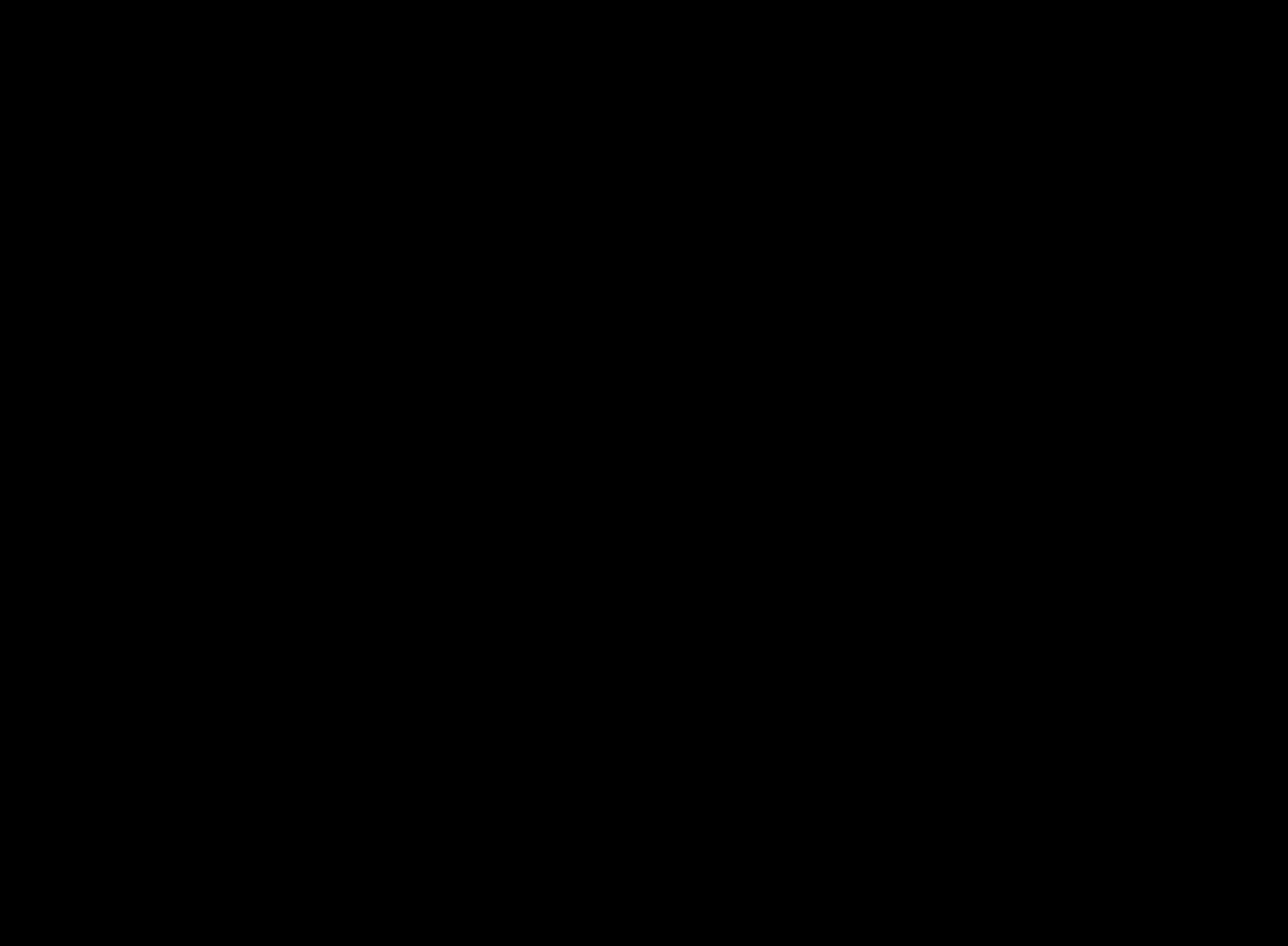

Supplement: Supplementary file 6 — Source data Fig. 4 [file 44318_2025_434_MOESM6_ESM.zip › Figure 4/4C/4C_DT_tdT.tif]

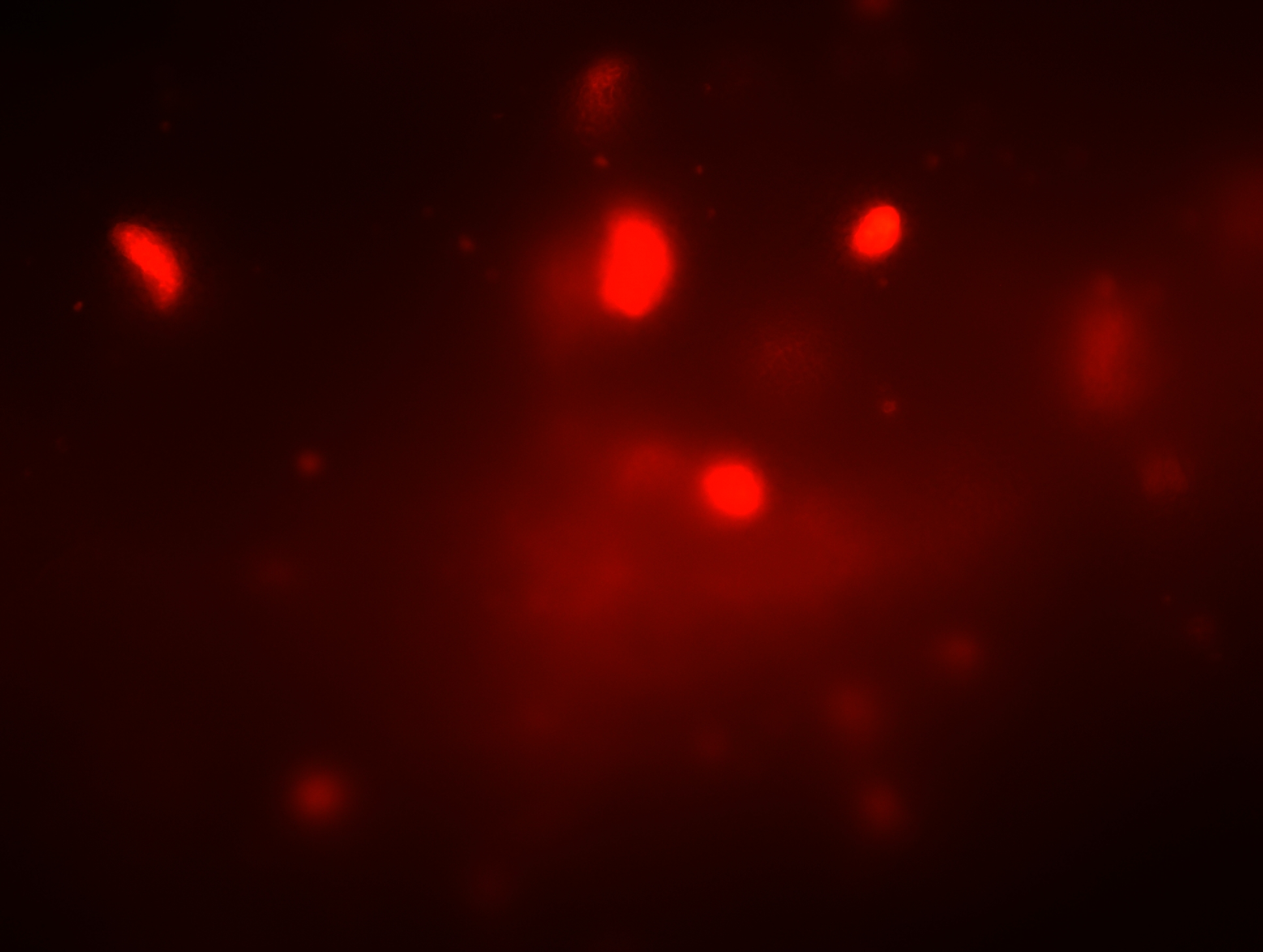

Supplement: Supplementary file 6 — Source data Fig. 4 [file 44318_2025_434_MOESM6_ESM.zip › Figure 4/4C/4C_PBS_tdT_mag.tif]

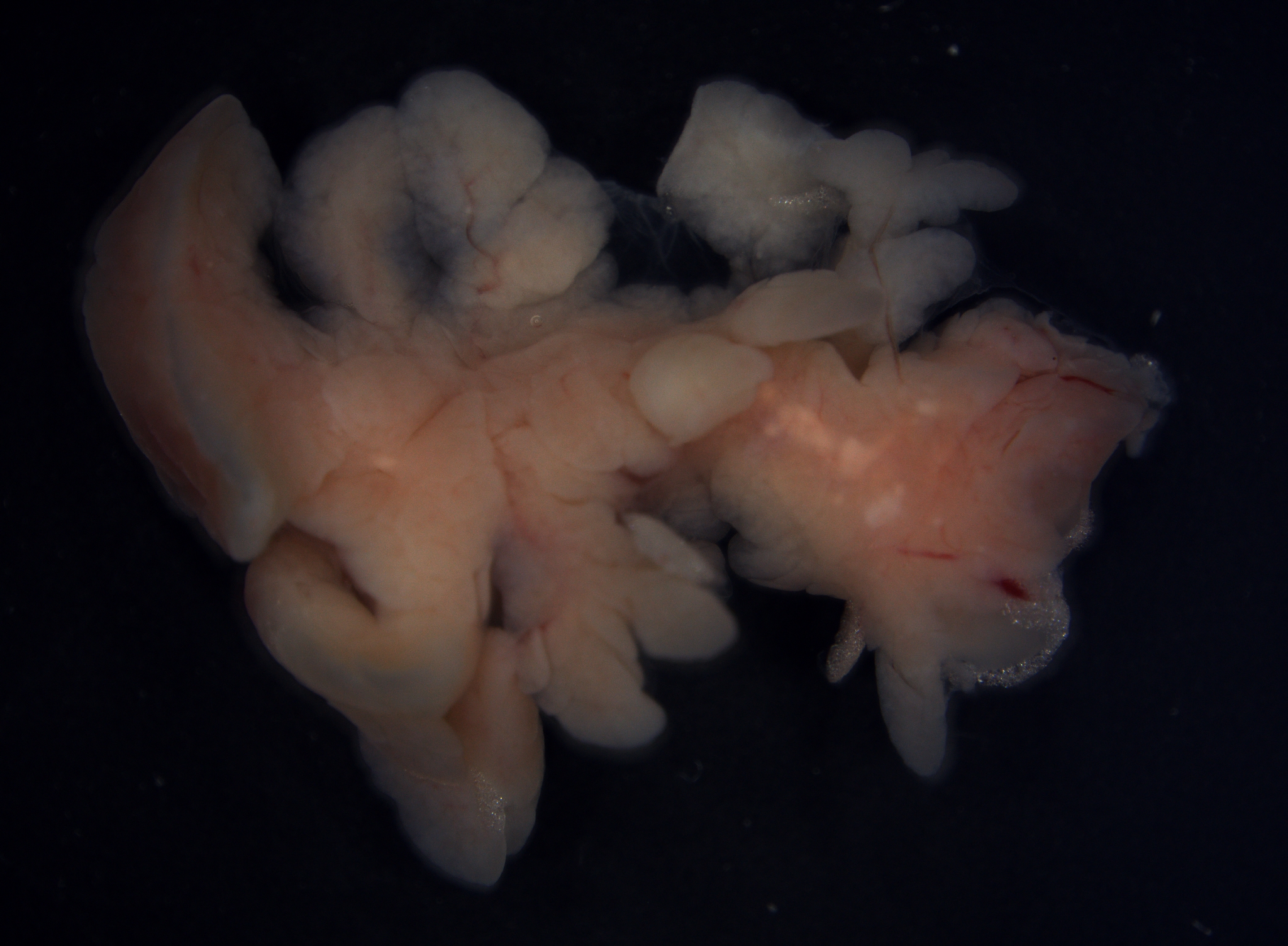

Supplement: Supplementary file 6 — Source data Fig. 4 [file 44318_2025_434_MOESM6_ESM.zip › Figure 4/4C/4C_DT_BF.tif]

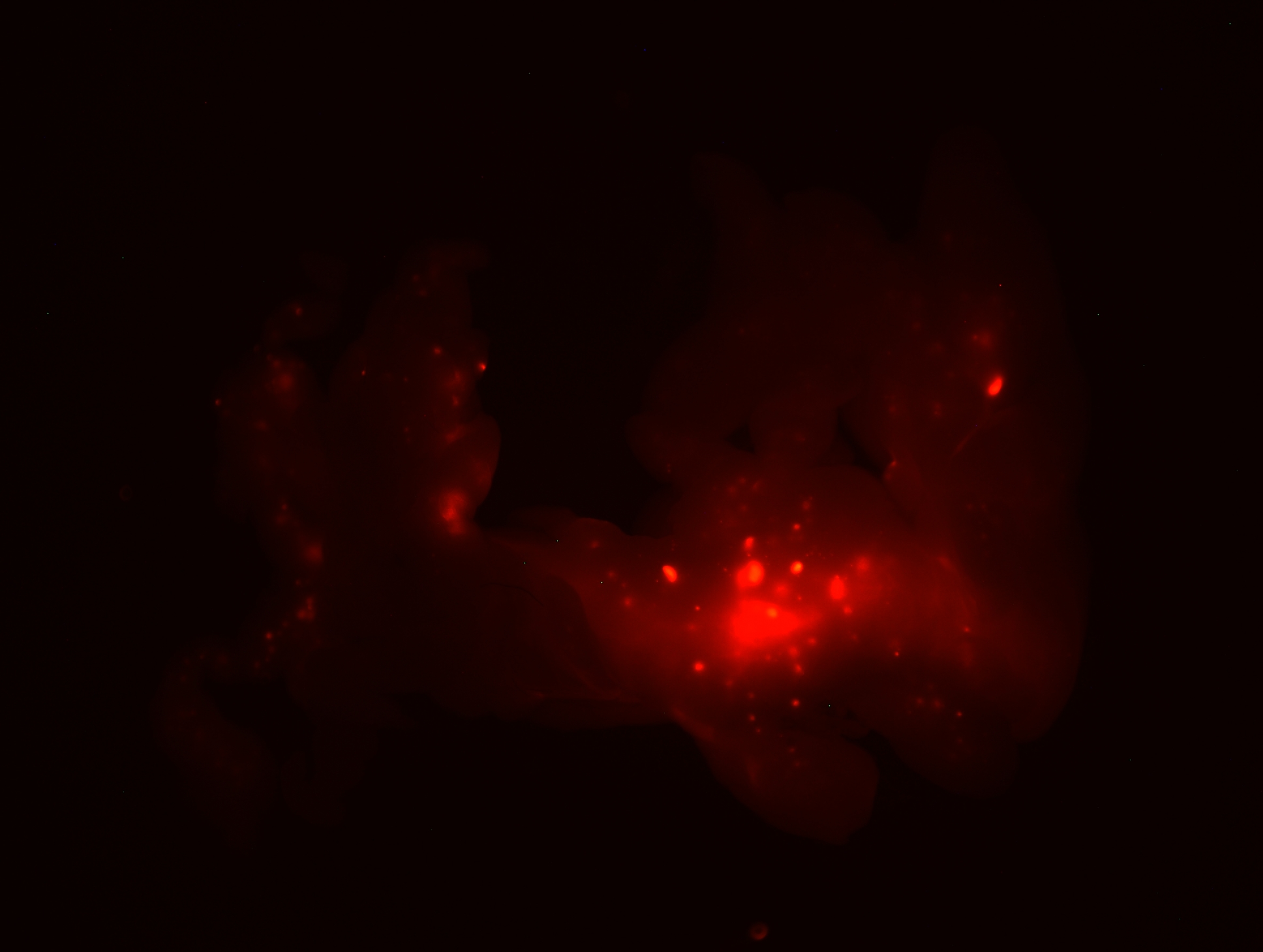

Supplement: Supplementary file 6 — Source data Fig. 4 [file 44318_2025_434_MOESM6_ESM.zip › Figure 4/4C/4C_PBS_tdT.tif]

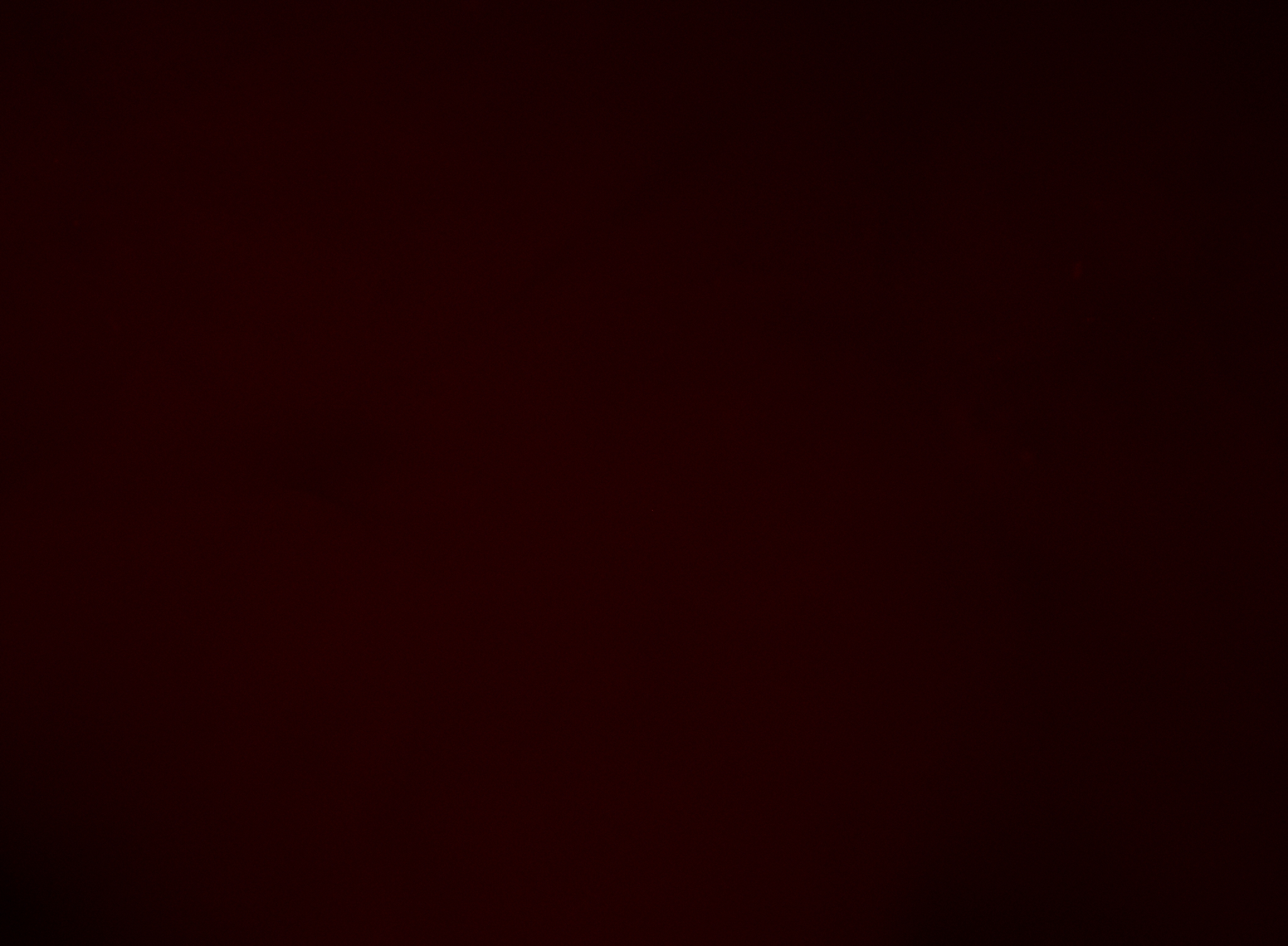

Supplement: Supplementary file 6 — Source data Fig. 4 [file 44318_2025_434_MOESM6_ESM.zip › Figure 4/4C/4C_DT_tdT_mag.tif]

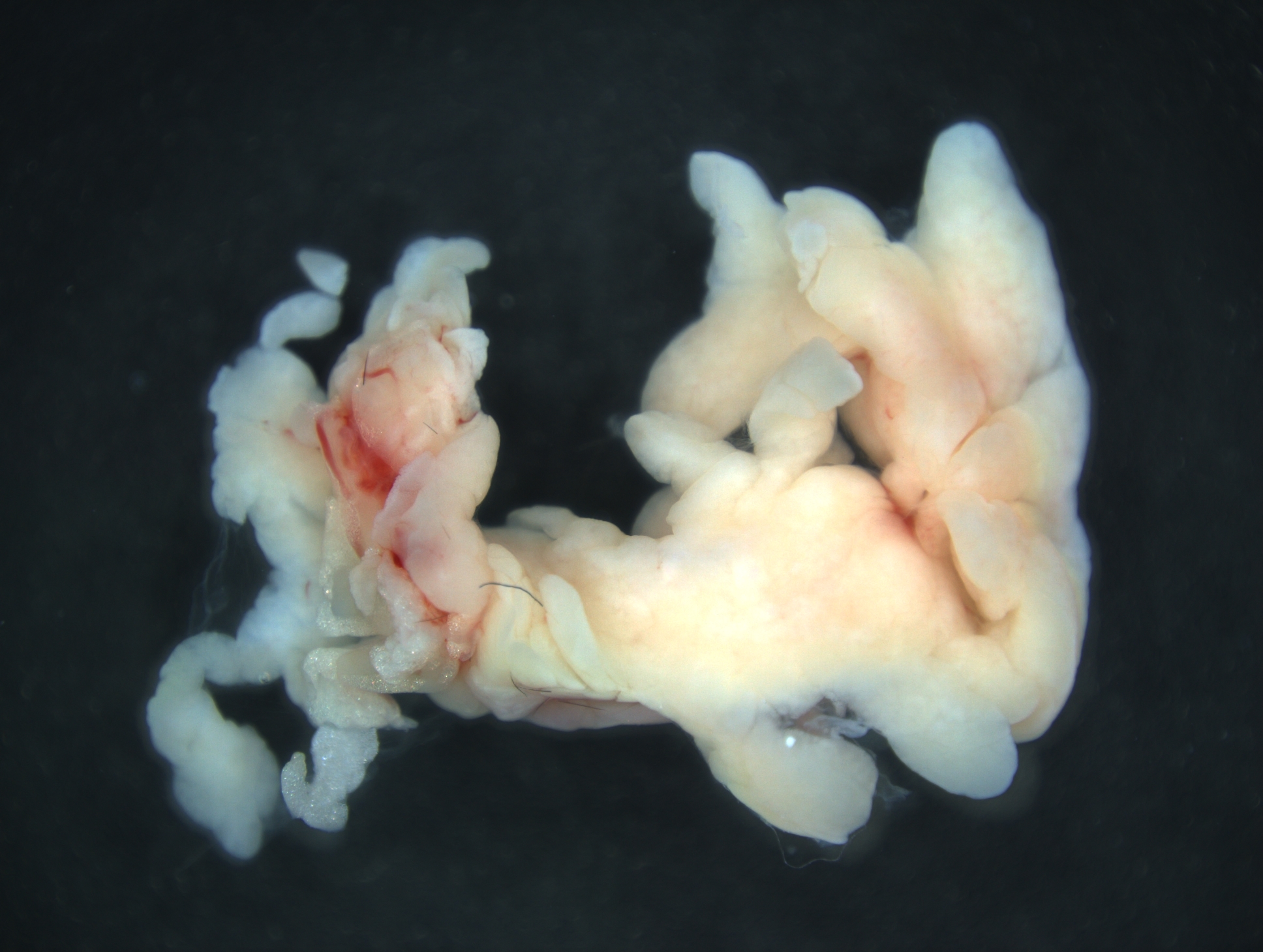

Supplement: Supplementary file 6 — Source data Fig. 4 [file 44318_2025_434_MOESM6_ESM.zip › Figure 4/4C/4C_PBS_BF.tif]

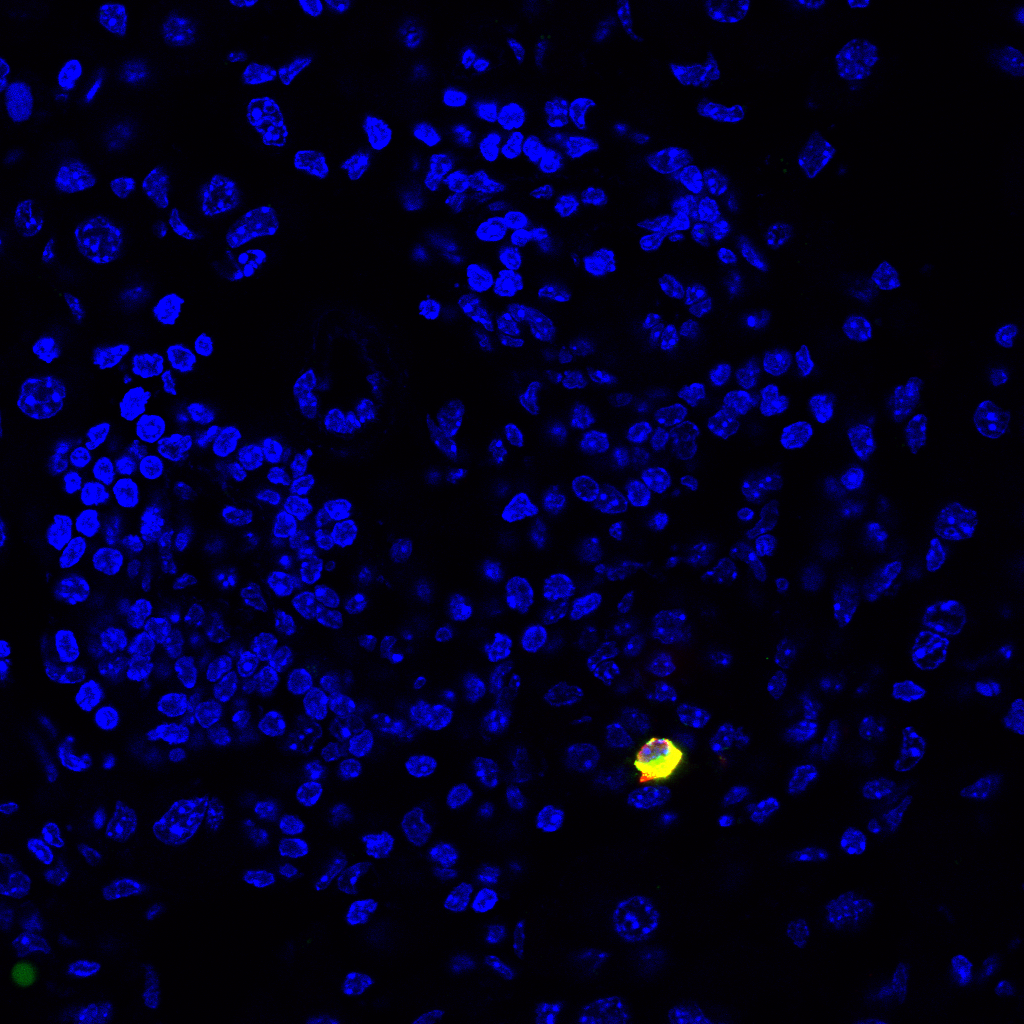

Supplement: Supplementary file 6 — Source data Fig. 4 [file 44318_2025_434_MOESM6_ESM.zip › Figure 4/4D/4D_DT.tif]

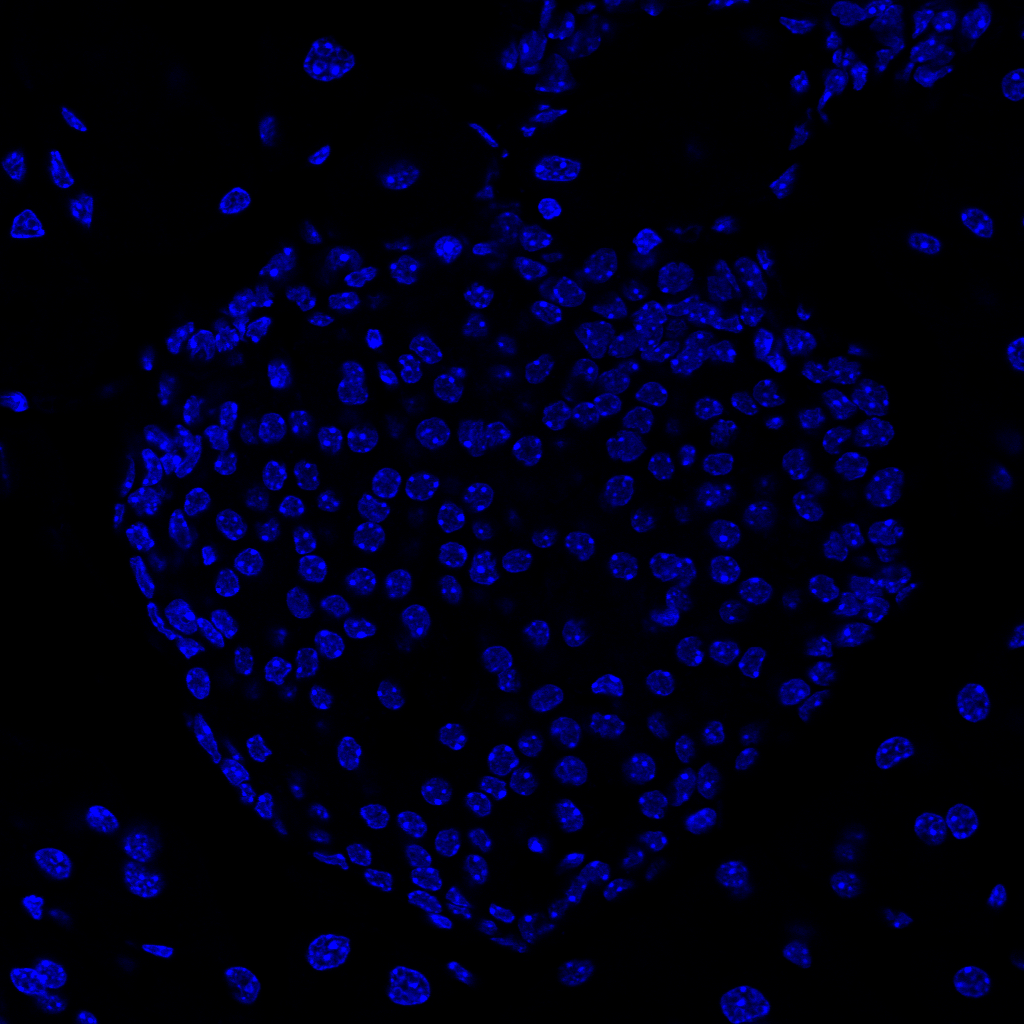

Supplement: Supplementary file 6 — Source data Fig. 4 [file 44318_2025_434_MOESM6_ESM.zip › Figure 4/4D/4D_PBS (blue).tif]

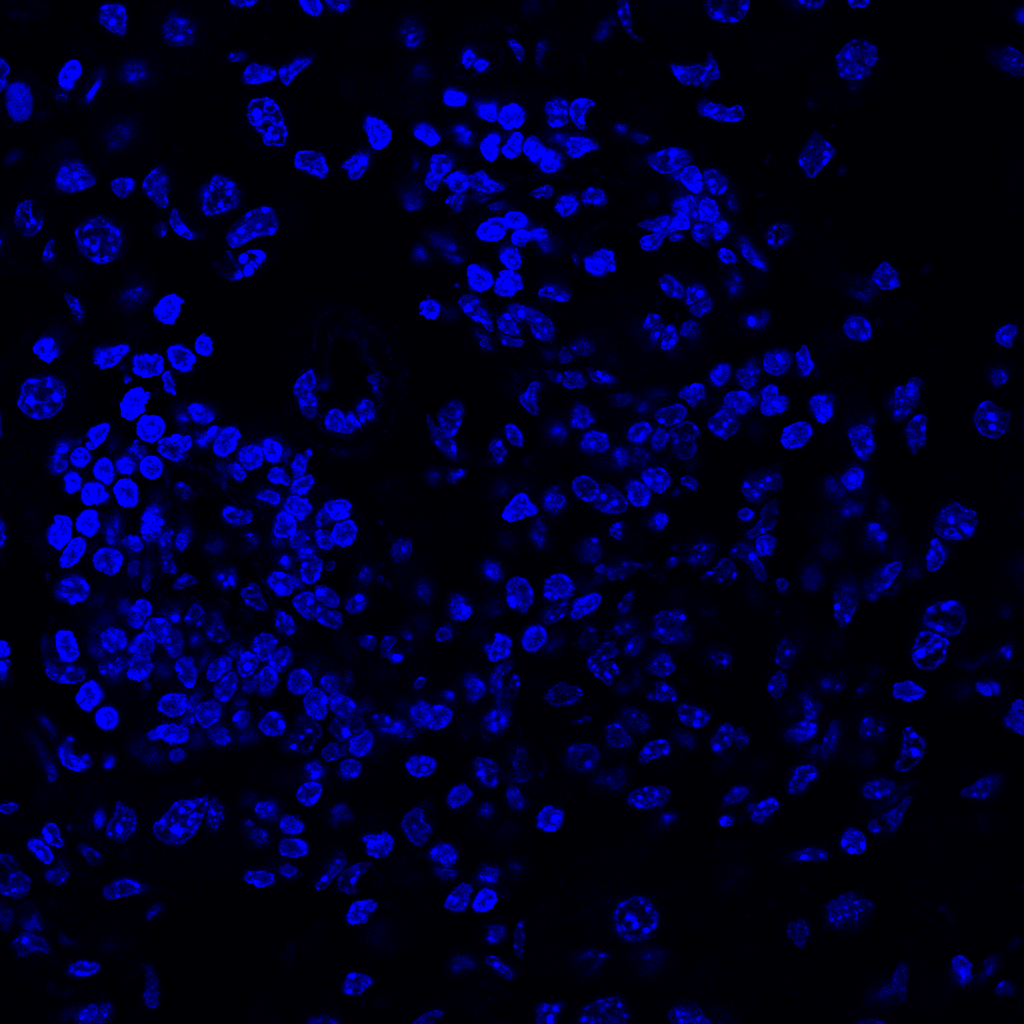

Supplement: Supplementary file 6 — Source data Fig. 4 [file 44318_2025_434_MOESM6_ESM.zip › Figure 4/4D/4D_DT (blue).tif]

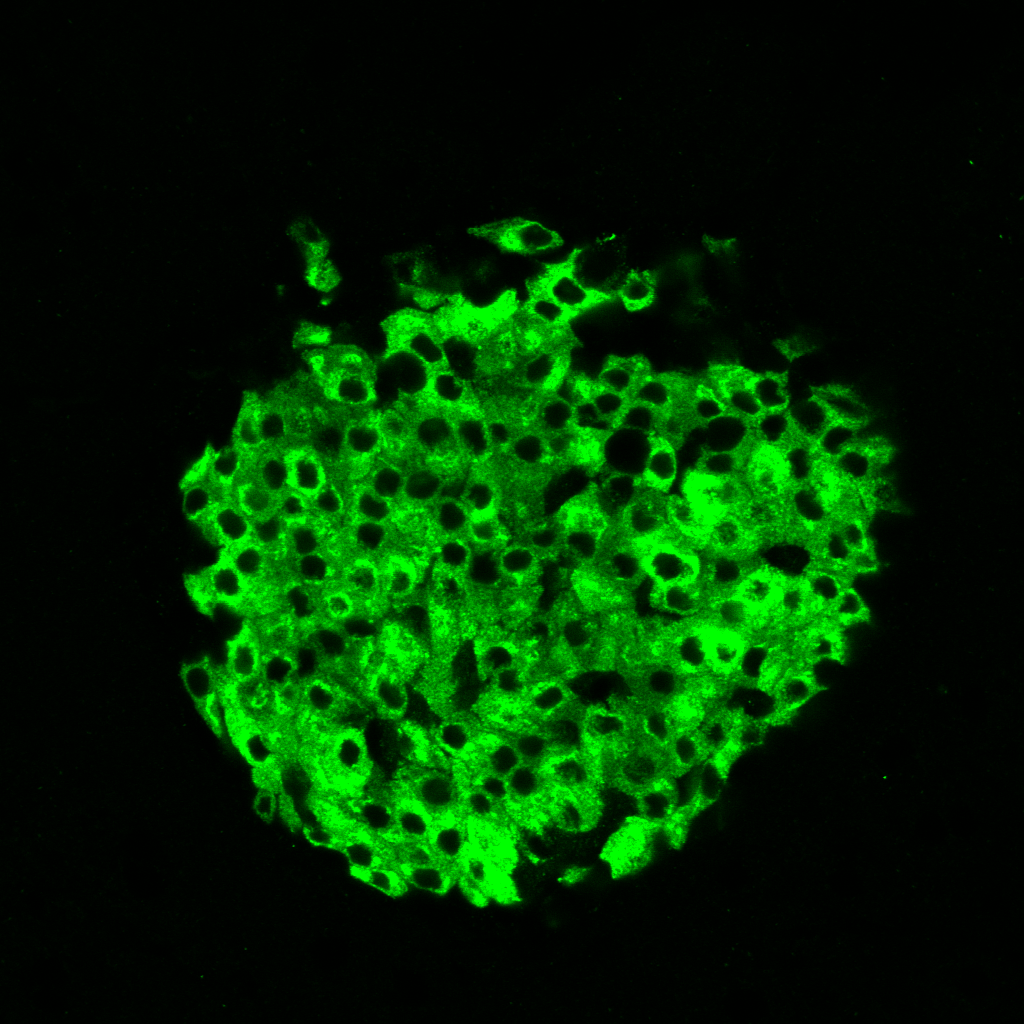

Supplement: Supplementary file 6 — Source data Fig. 4 [file 44318_2025_434_MOESM6_ESM.zip › Figure 4/4D/4D_PBS (green).tif]

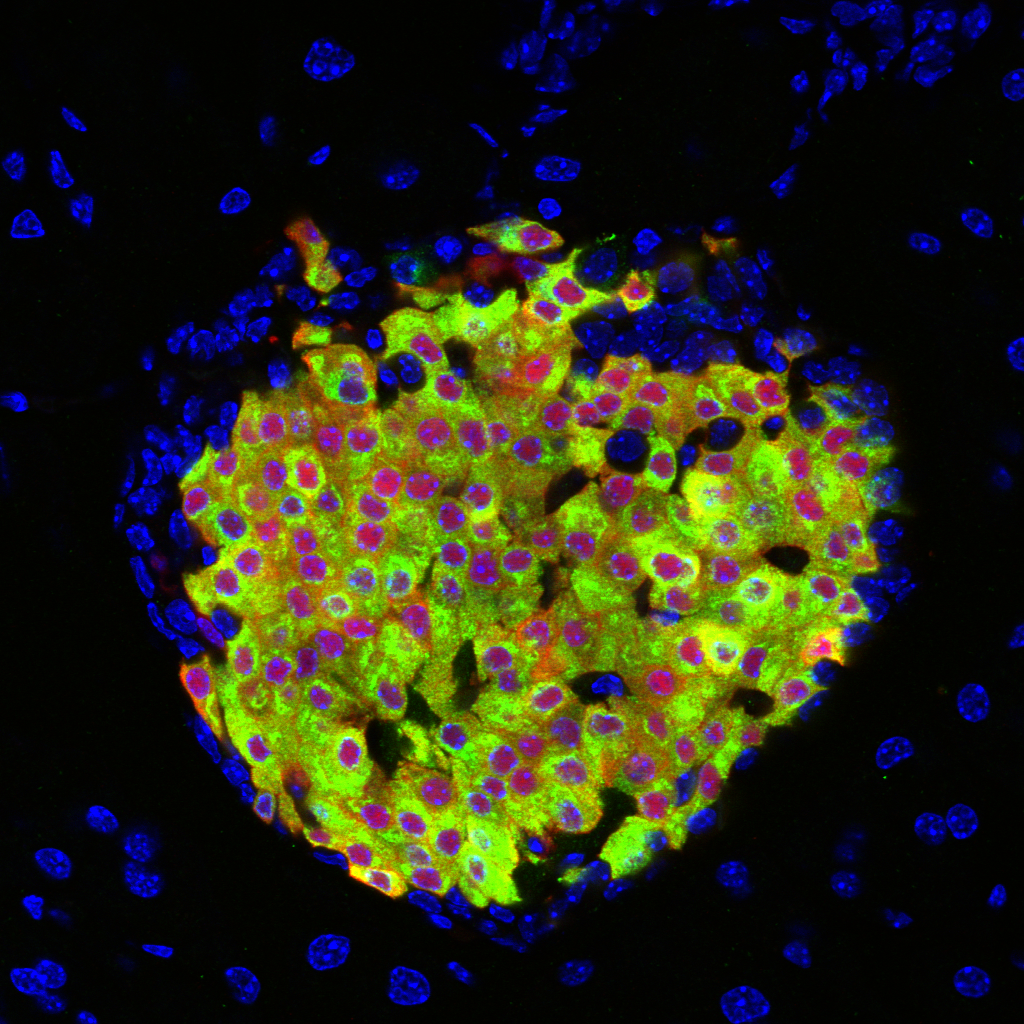

Supplement: Supplementary file 6 — Source data Fig. 4 [file 44318_2025_434_MOESM6_ESM.zip › Figure 4/4D/4D_PBS.tif]

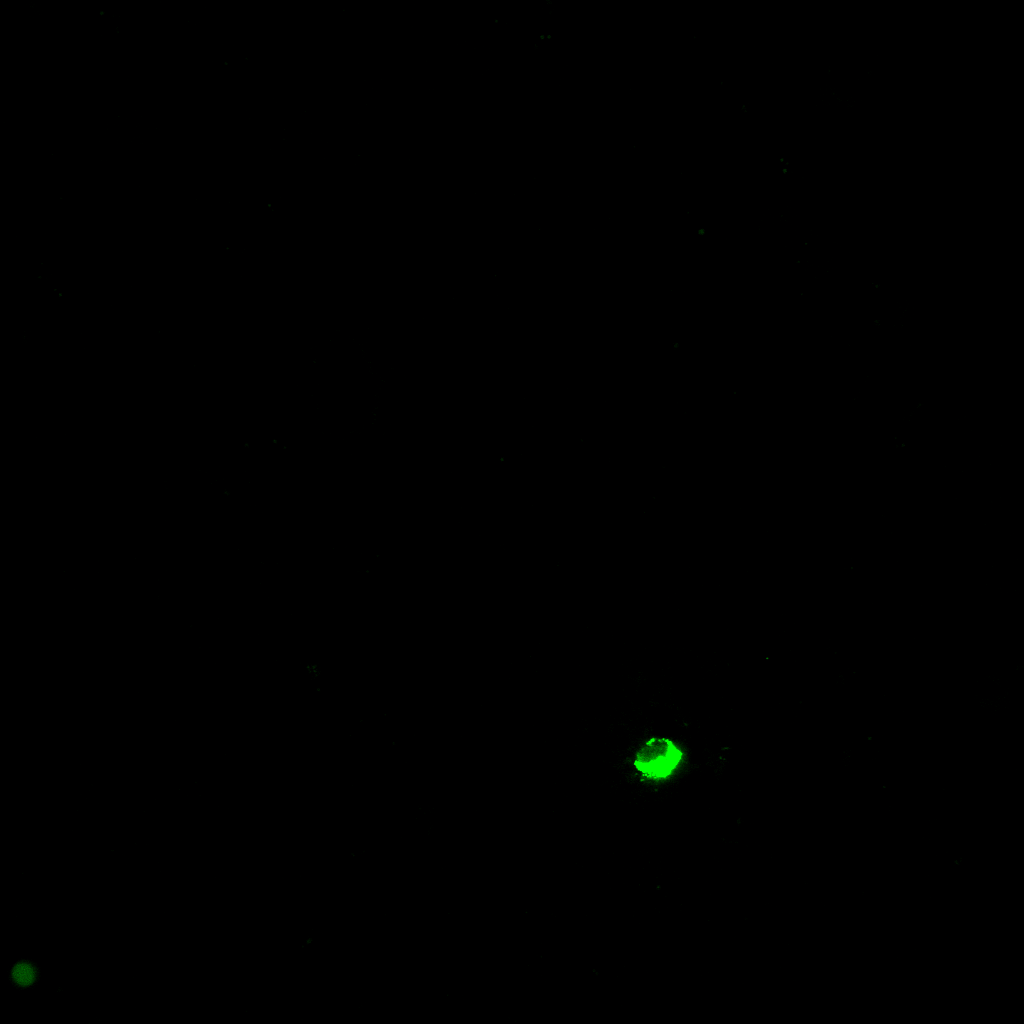

Supplement: Supplementary file 6 — Source data Fig. 4 [file 44318_2025_434_MOESM6_ESM.zip › Figure 4/4D/4D_DT (green).tif]

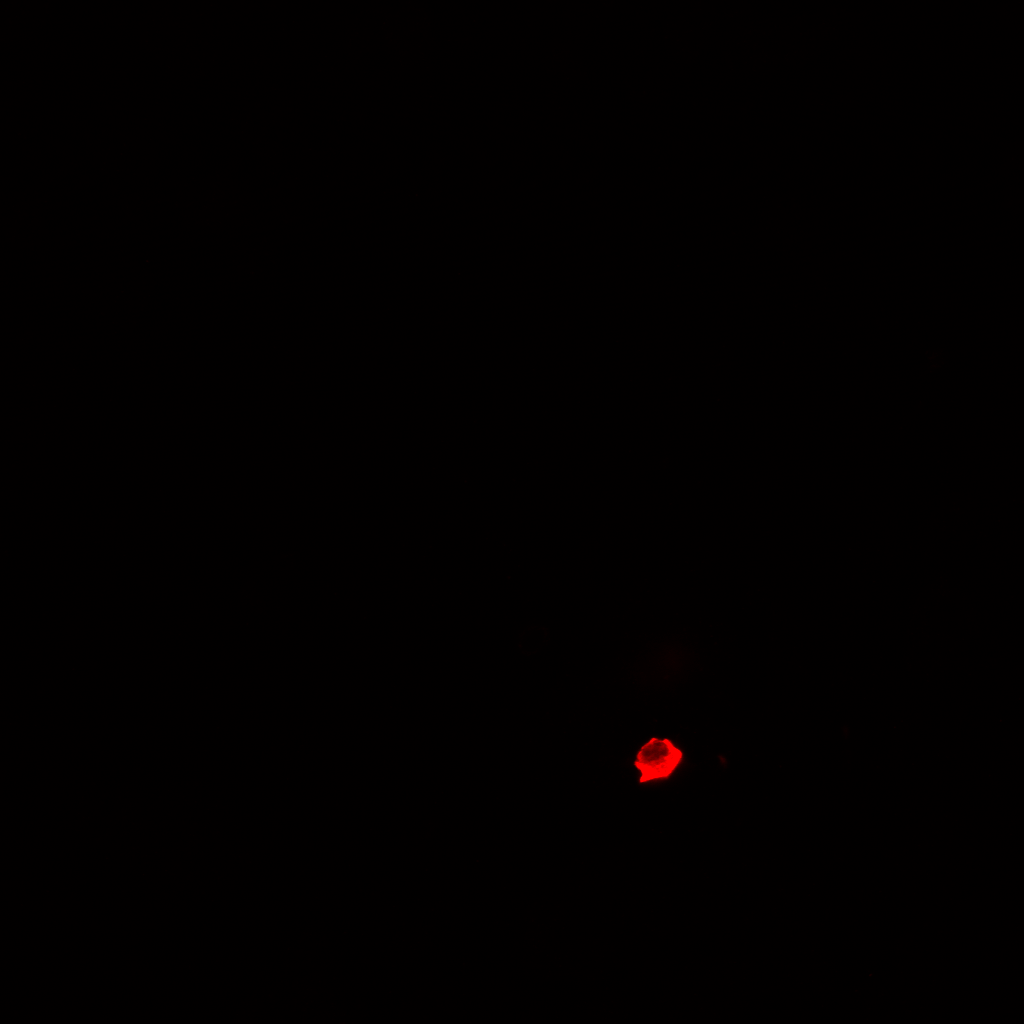

Supplement: Supplementary file 6 — Source data Fig. 4 [file 44318_2025_434_MOESM6_ESM.zip › Figure 4/4D/4D_DT (red).tif]

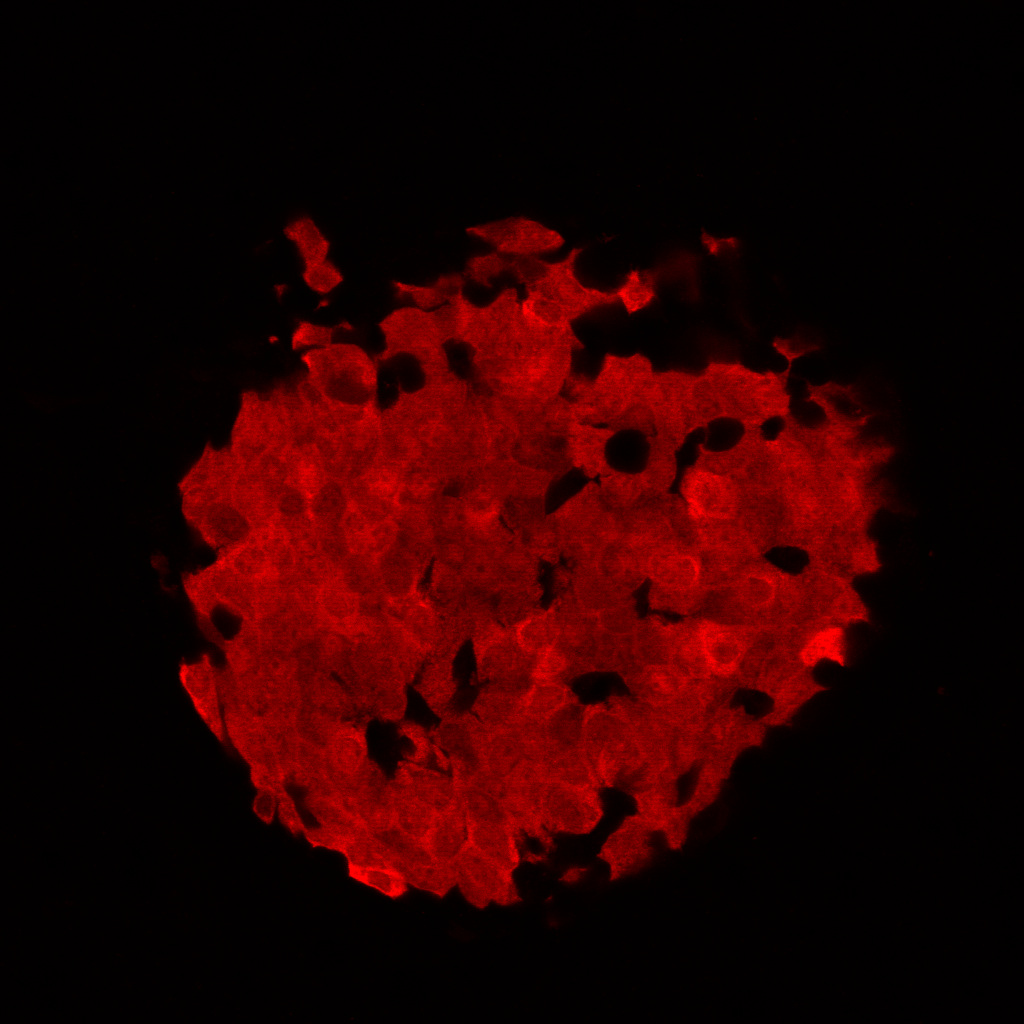

Supplement: Supplementary file 6 — Source data Fig. 4 [file 44318_2025_434_MOESM6_ESM.zip › Figure 4/4D/4D_PBS (red).tif]

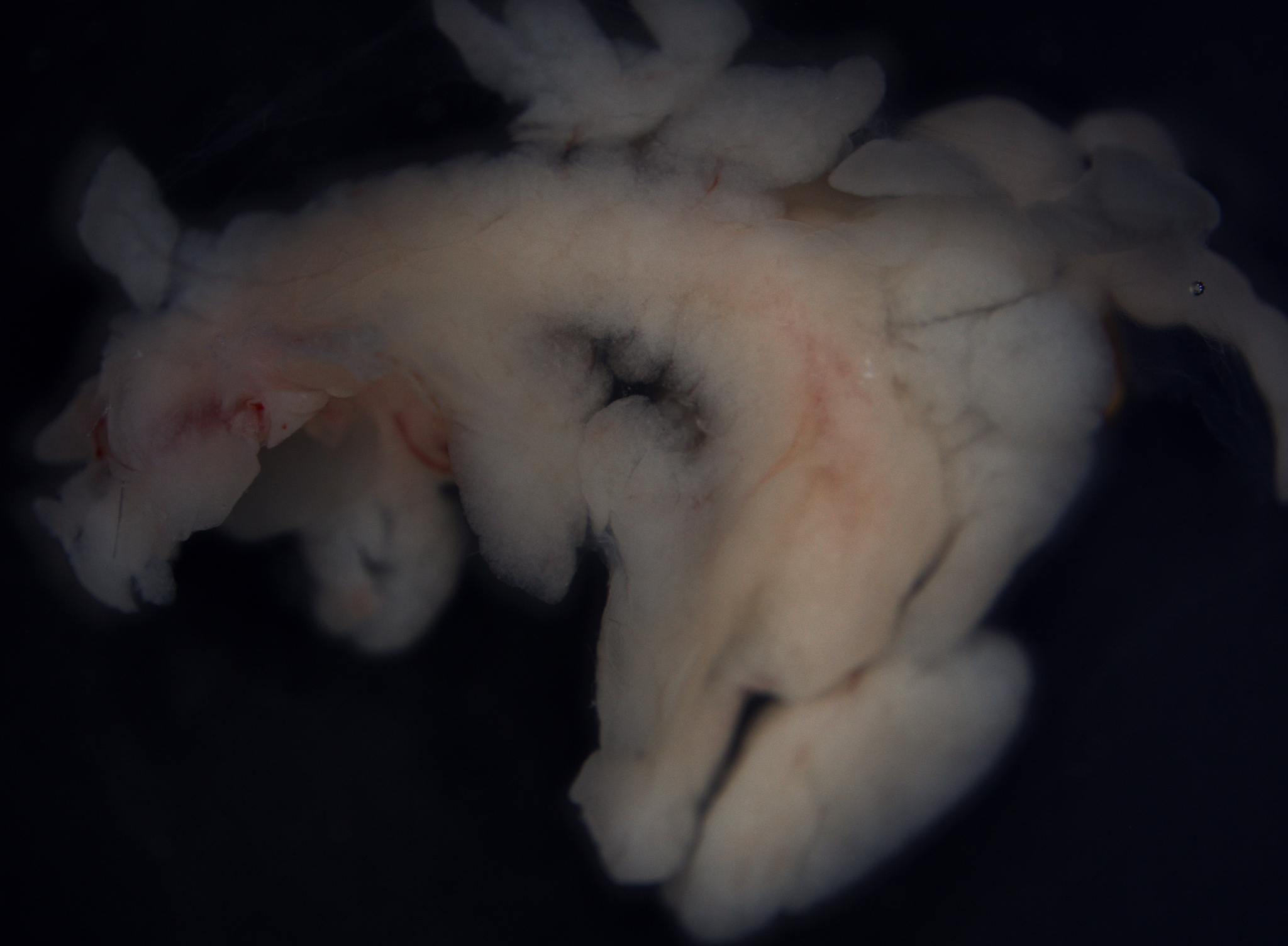

Supplement: Supplementary file 6 — Source data Fig. 4 [file 44318_2025_434_MOESM6_ESM.zip › Figure 4/4H/4H_DT_BF.tif]

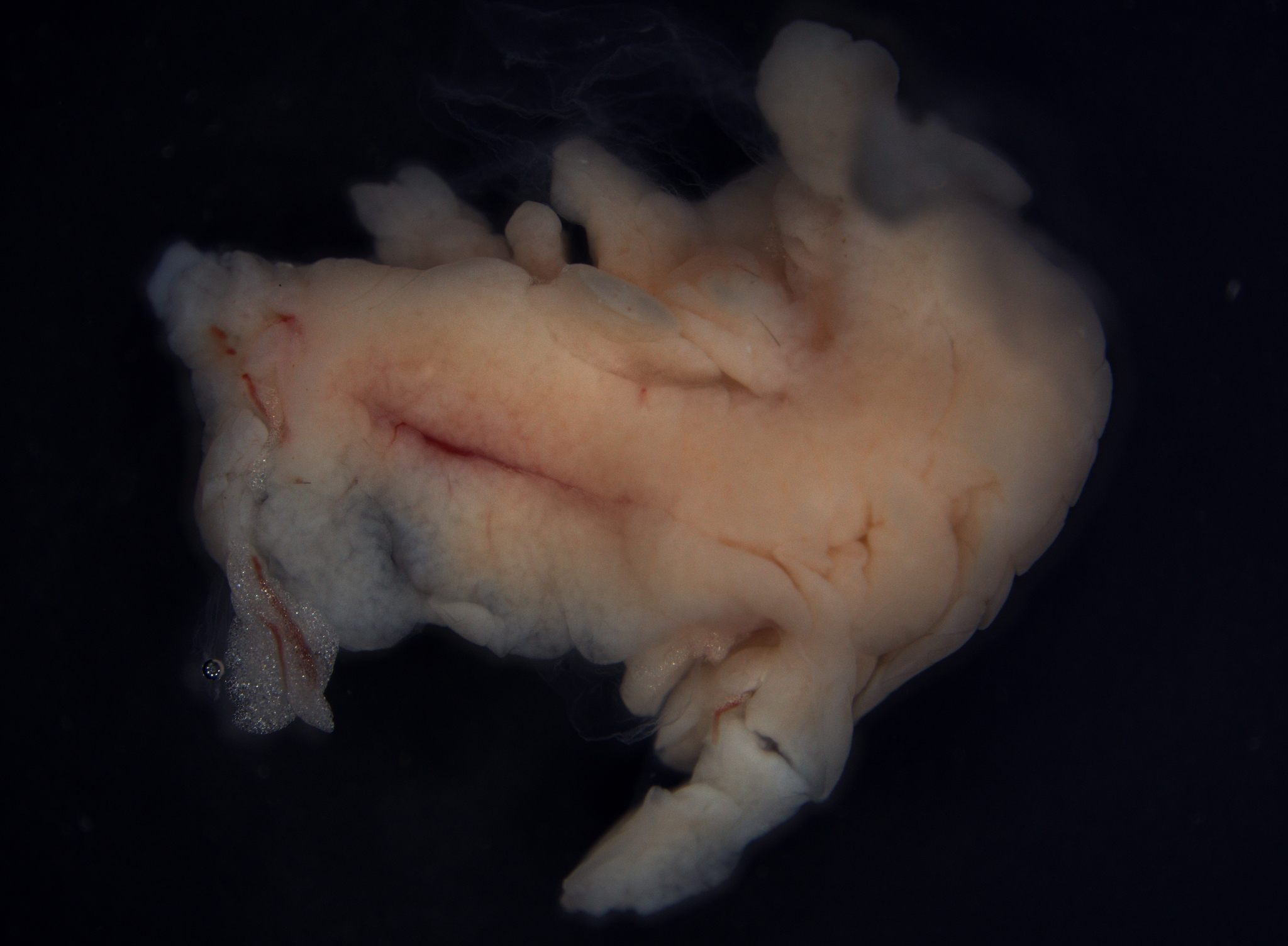

Supplement: Supplementary file 6 — Source data Fig. 4 [file 44318_2025_434_MOESM6_ESM.zip › Figure 4/4H/4H_PBS_BF.tif]

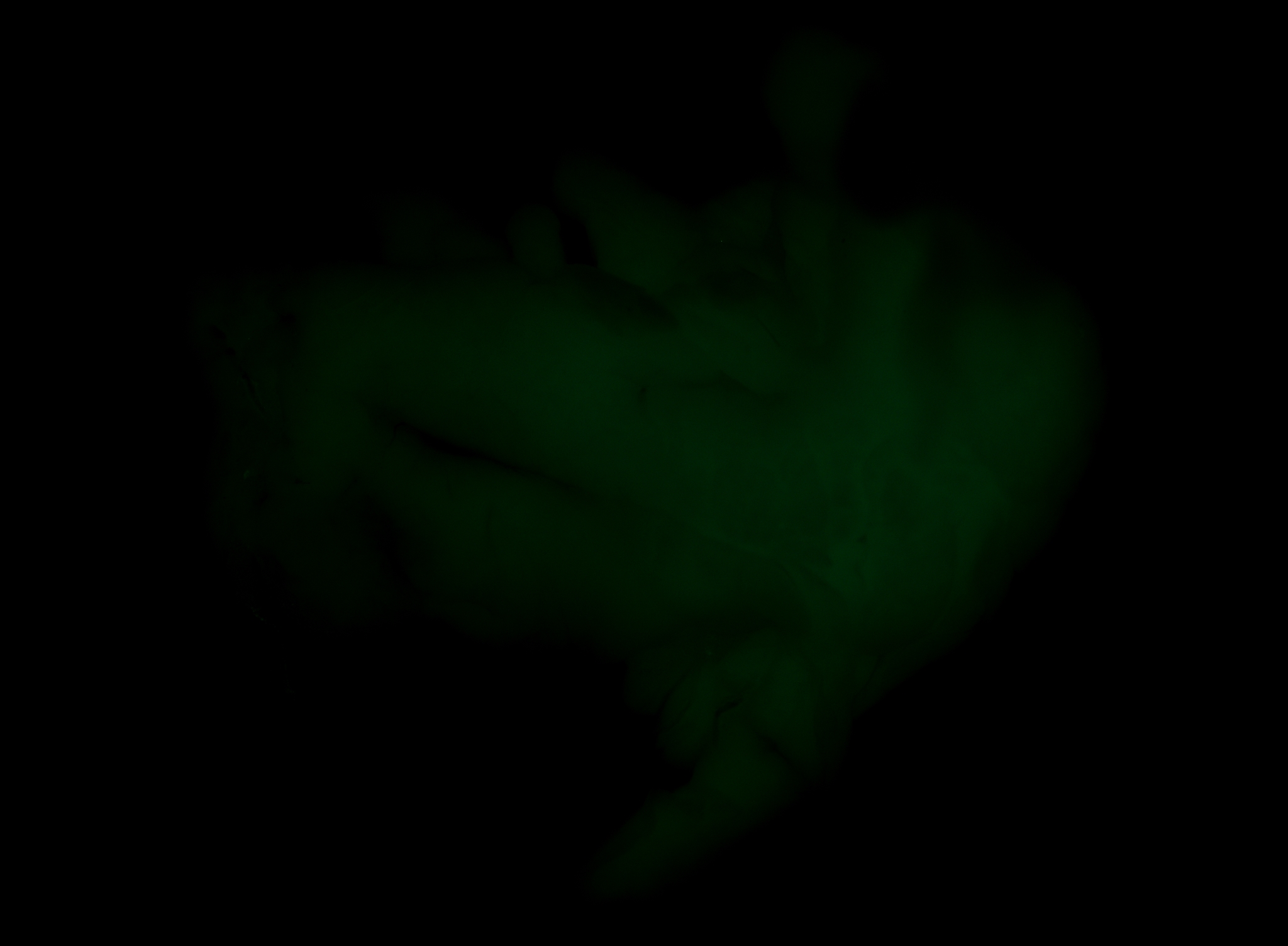

Supplement: Supplementary file 6 — Source data Fig. 4 [file 44318_2025_434_MOESM6_ESM.zip › Figure 4/4H/4H_PBS_zsGreen.tif]

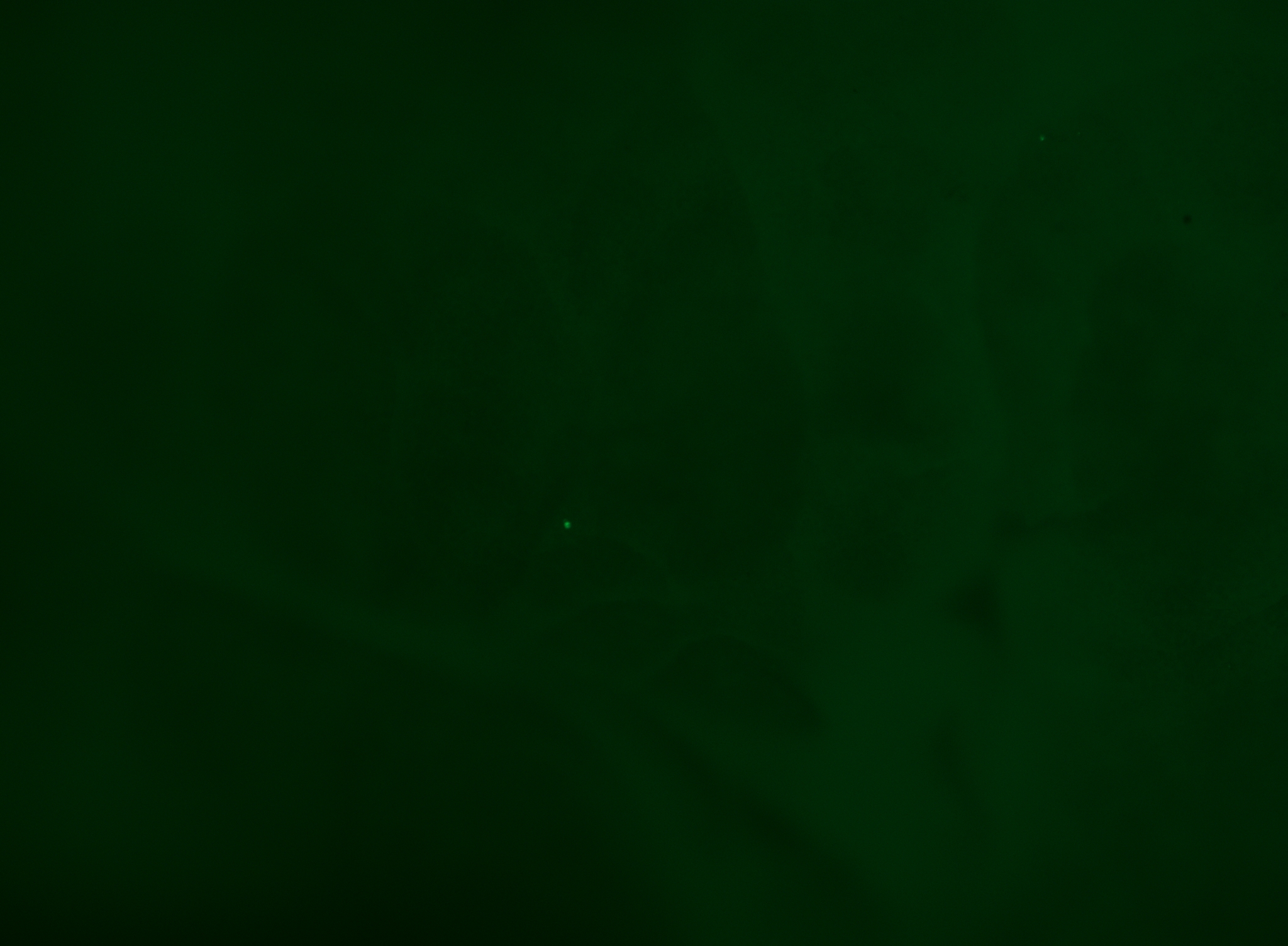

Supplement: Supplementary file 6 — Source data Fig. 4 [file 44318_2025_434_MOESM6_ESM.zip › Figure 4/4H/4H_PBS_zsGreen_mag.tif]

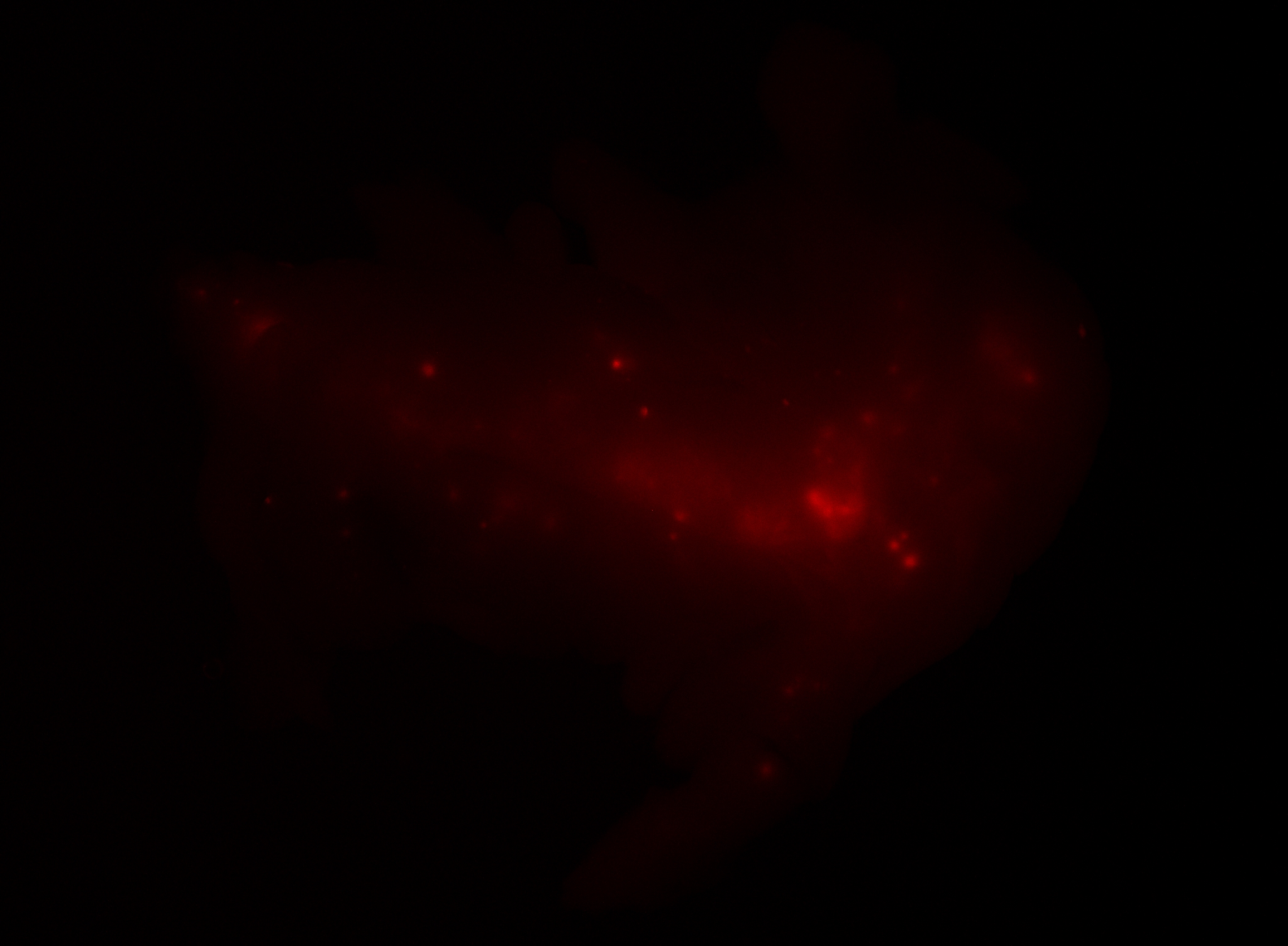

Supplement: Supplementary file 6 — Source data Fig. 4 [file 44318_2025_434_MOESM6_ESM.zip › Figure 4/4H/4H_PBS_tdT.tif]

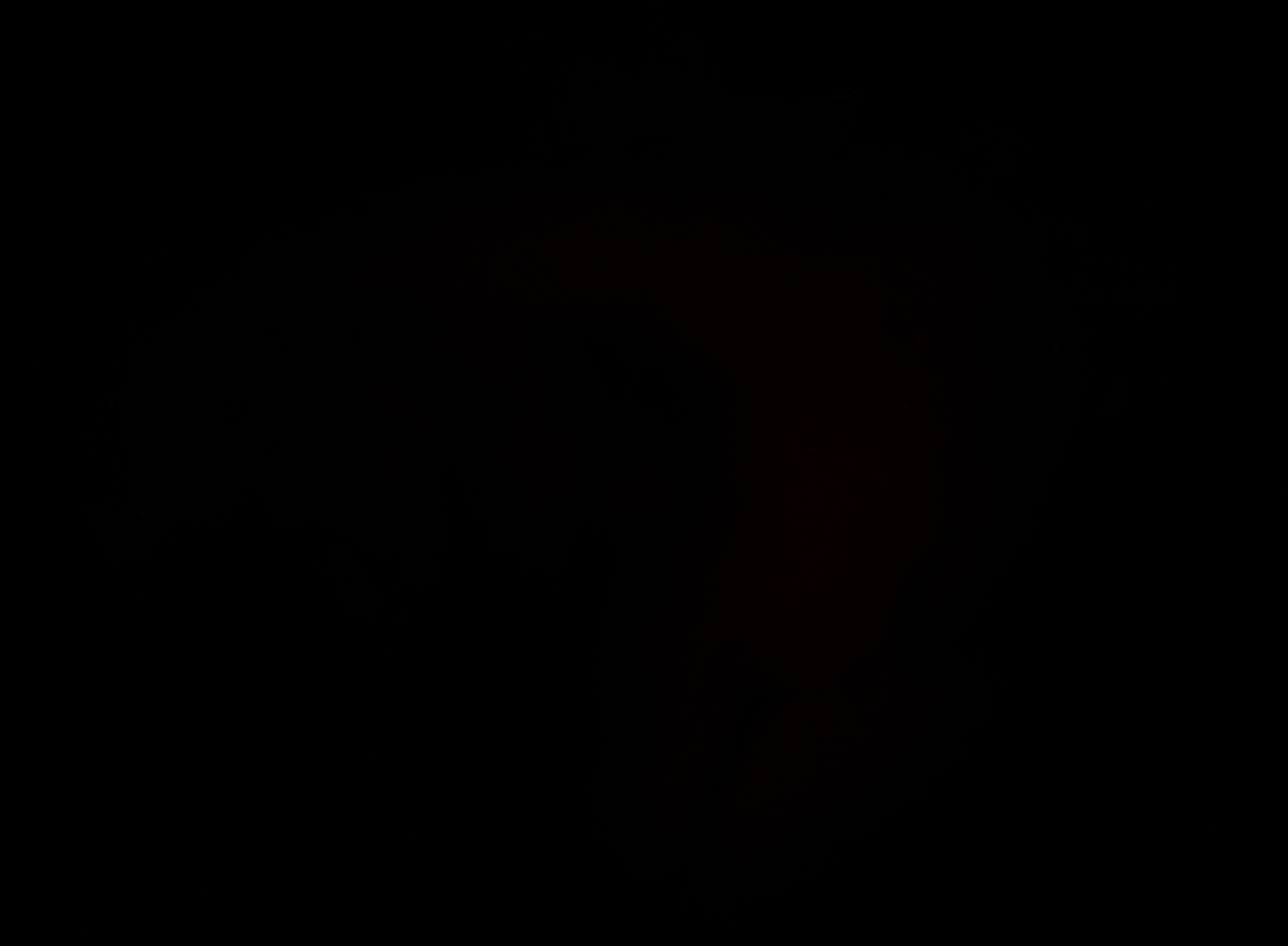

Supplement: Supplementary file 6 — Source data Fig. 4 [file 44318_2025_434_MOESM6_ESM.zip › Figure 4/4H/4H_DT_zsGreen.tif]

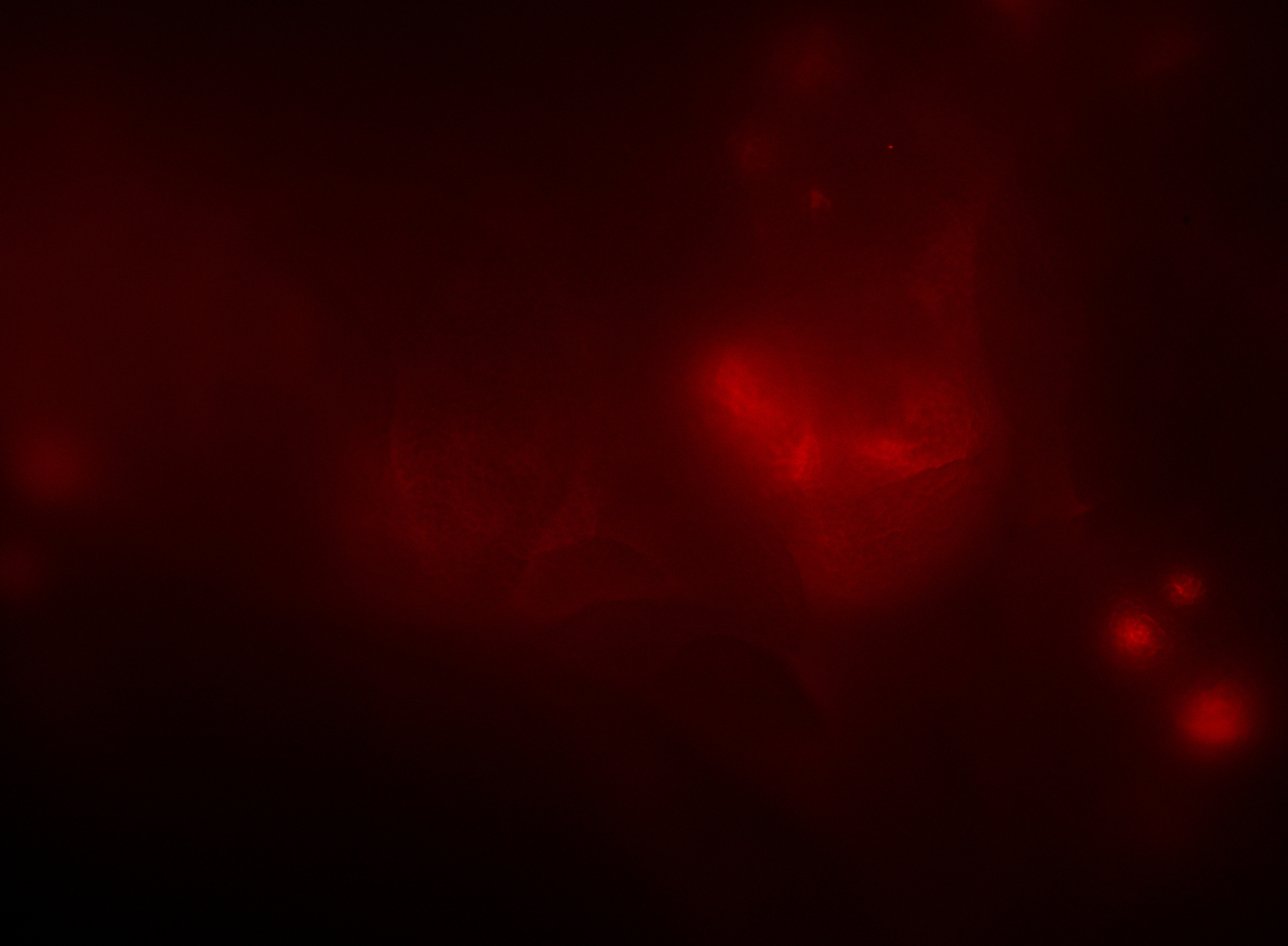

Supplement: Supplementary file 6 — Source data Fig. 4 [file 44318_2025_434_MOESM6_ESM.zip › Figure 4/4H/4H_PBS_tdT_mag.tif]

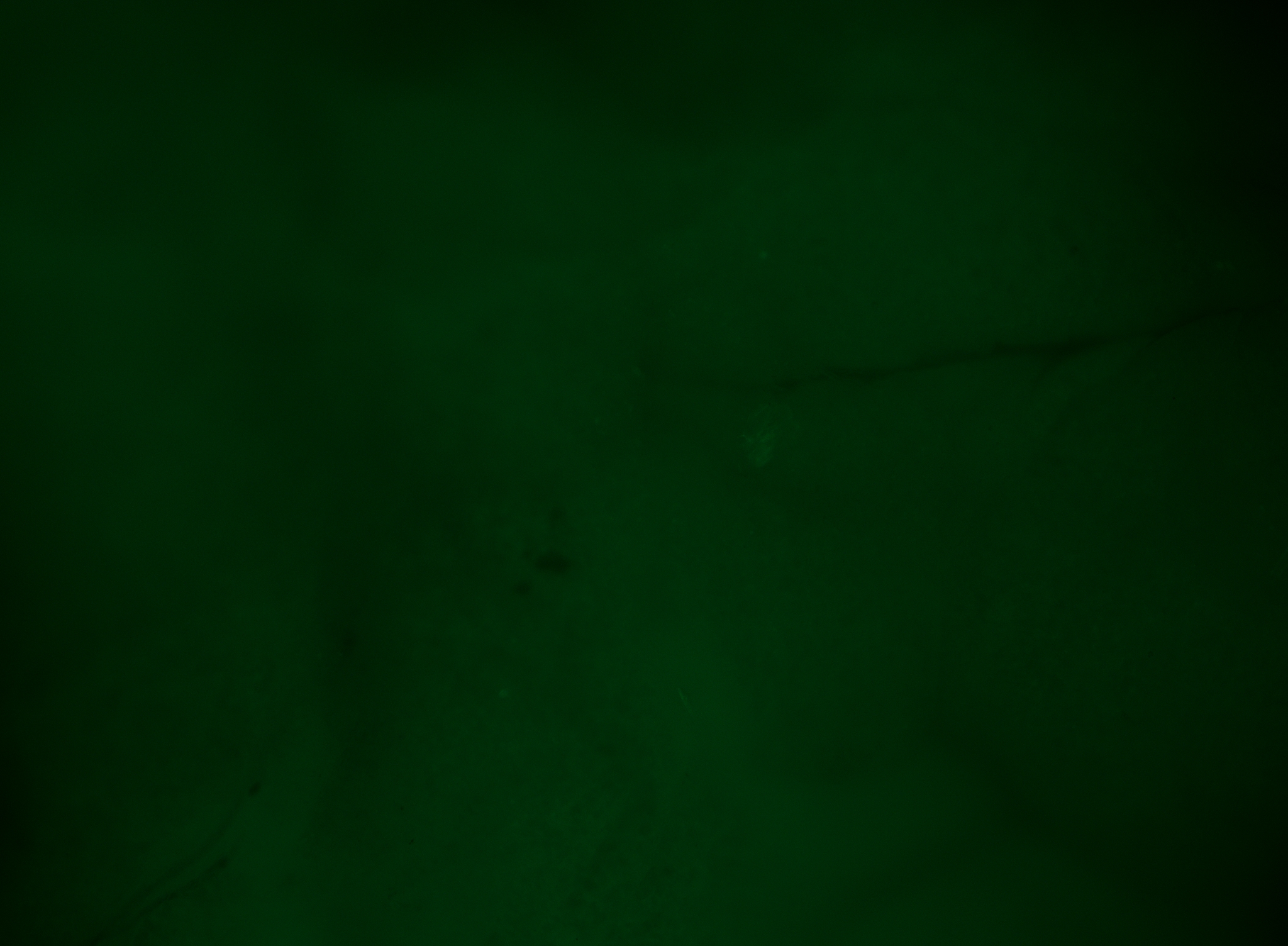

Supplement: Supplementary file 6 — Source data Fig. 4 [file 44318_2025_434_MOESM6_ESM.zip › Figure 4/4H/4H_DT_zsGreen_mag.tif]

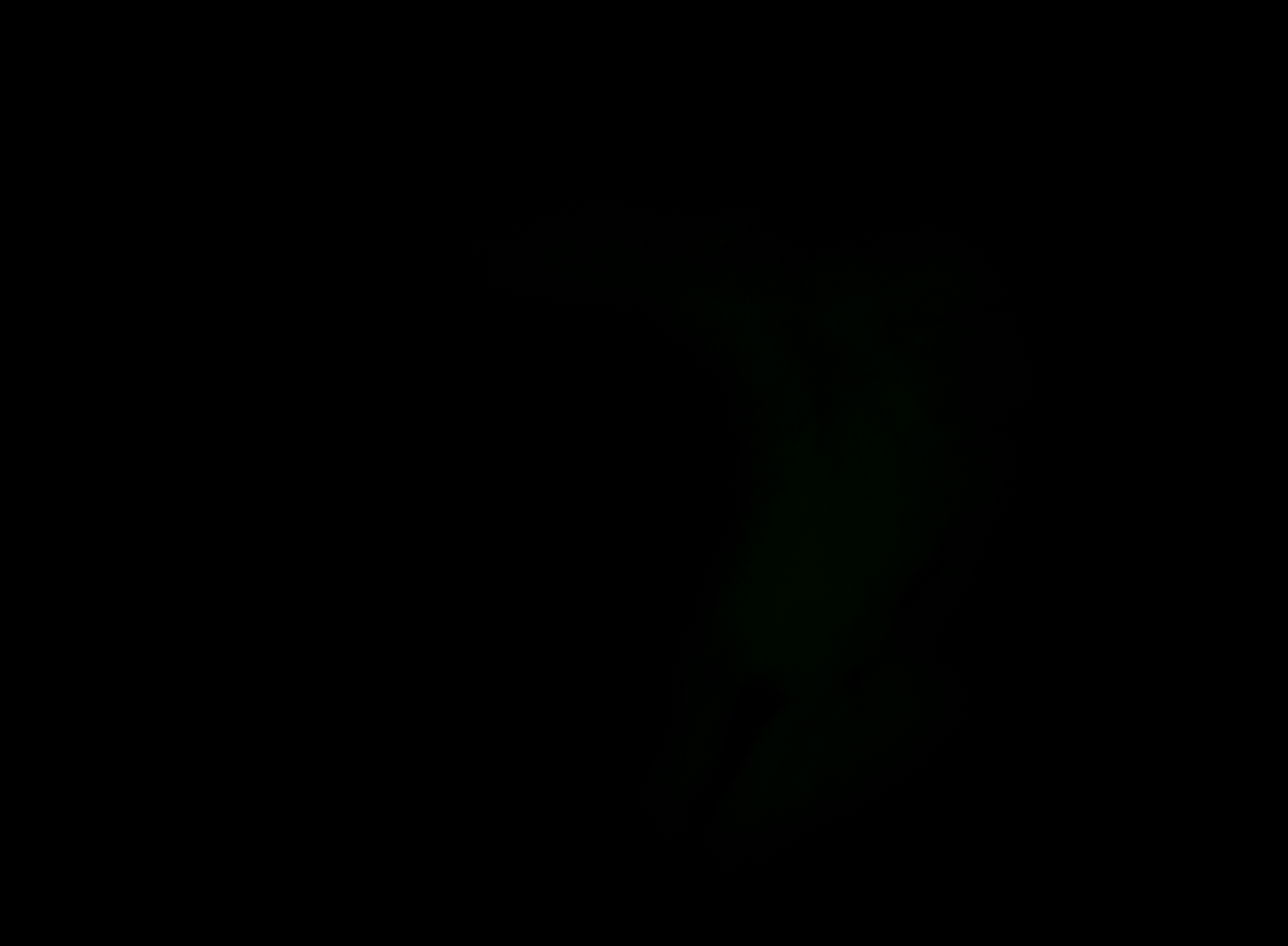

Supplement: Supplementary file 6 — Source data Fig. 4 [file 44318_2025_434_MOESM6_ESM.zip › Figure 4/4H/4H_DT_tdT.tif]

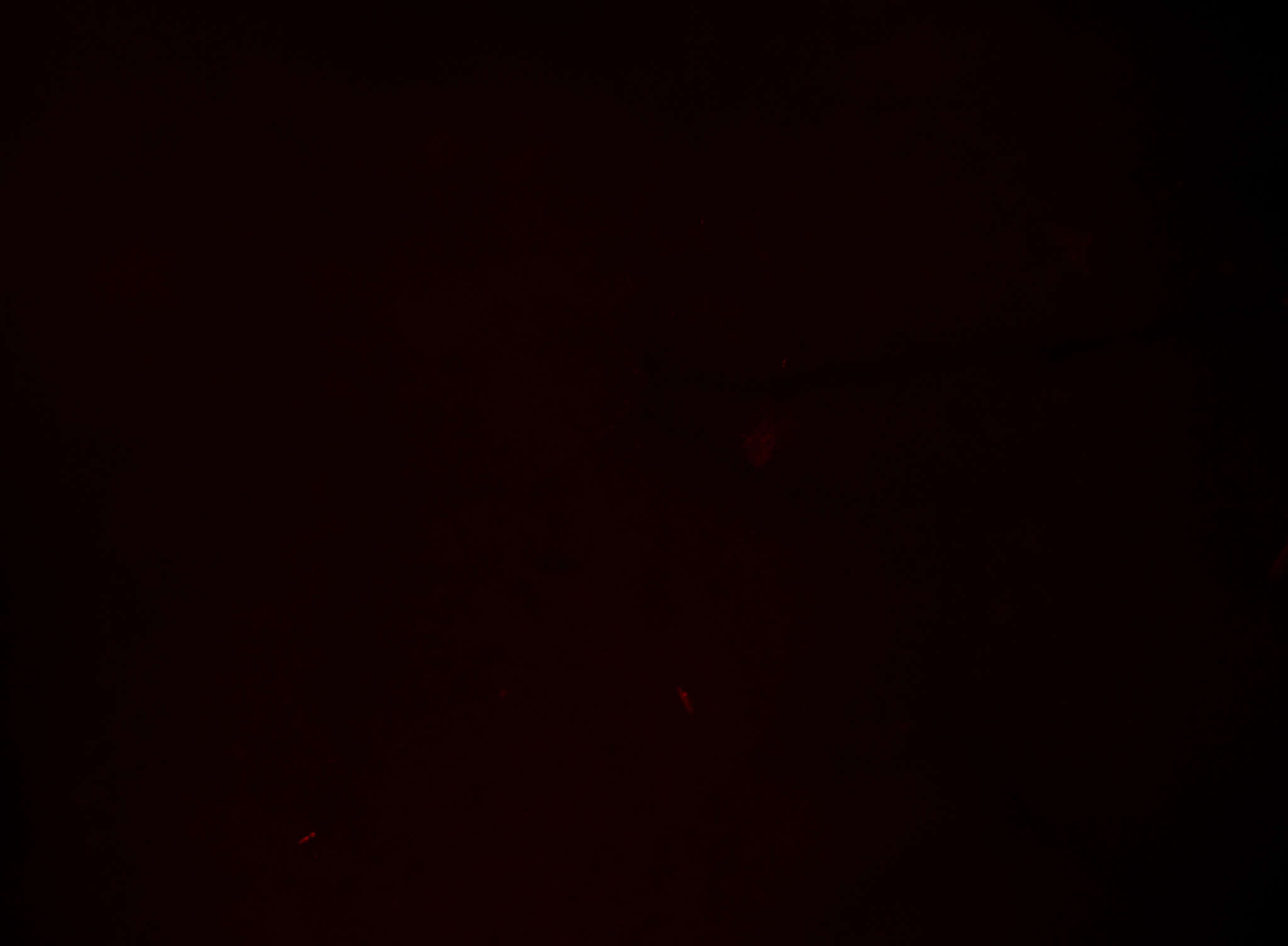

Supplement: Supplementary file 6 — Source data Fig. 4 [file 44318_2025_434_MOESM6_ESM.zip › Figure 4/4H/4H_DT_tdT_mag.tif]

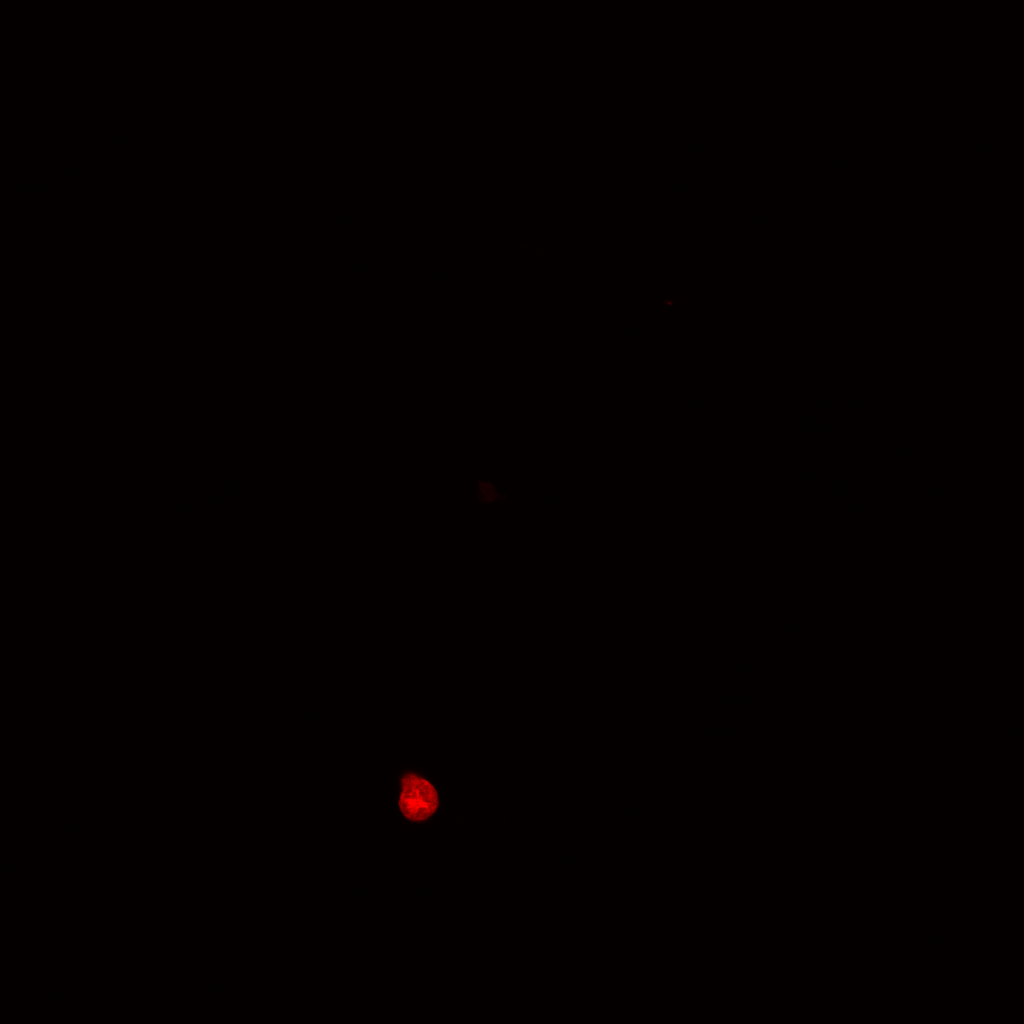

Supplement: Supplementary file 6 — Source data Fig. 4 [file 44318_2025_434_MOESM6_ESM.zip › Figure 4/4I/4I_DT_Merge (red).tif]

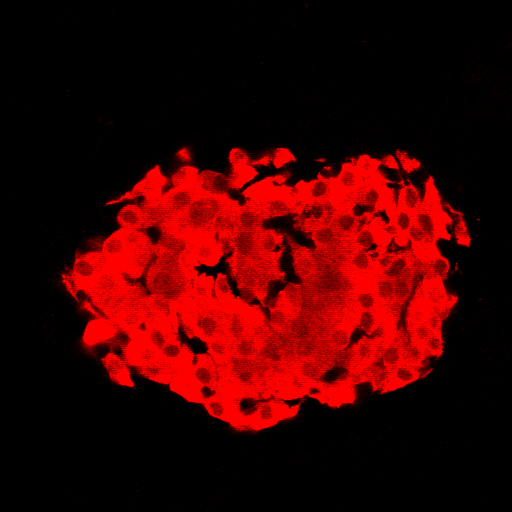

Supplement: Supplementary file 6 — Source data Fig. 4 [file 44318_2025_434_MOESM6_ESM.zip › Figure 4/4I/4I_PBS_Merge (red).tif]

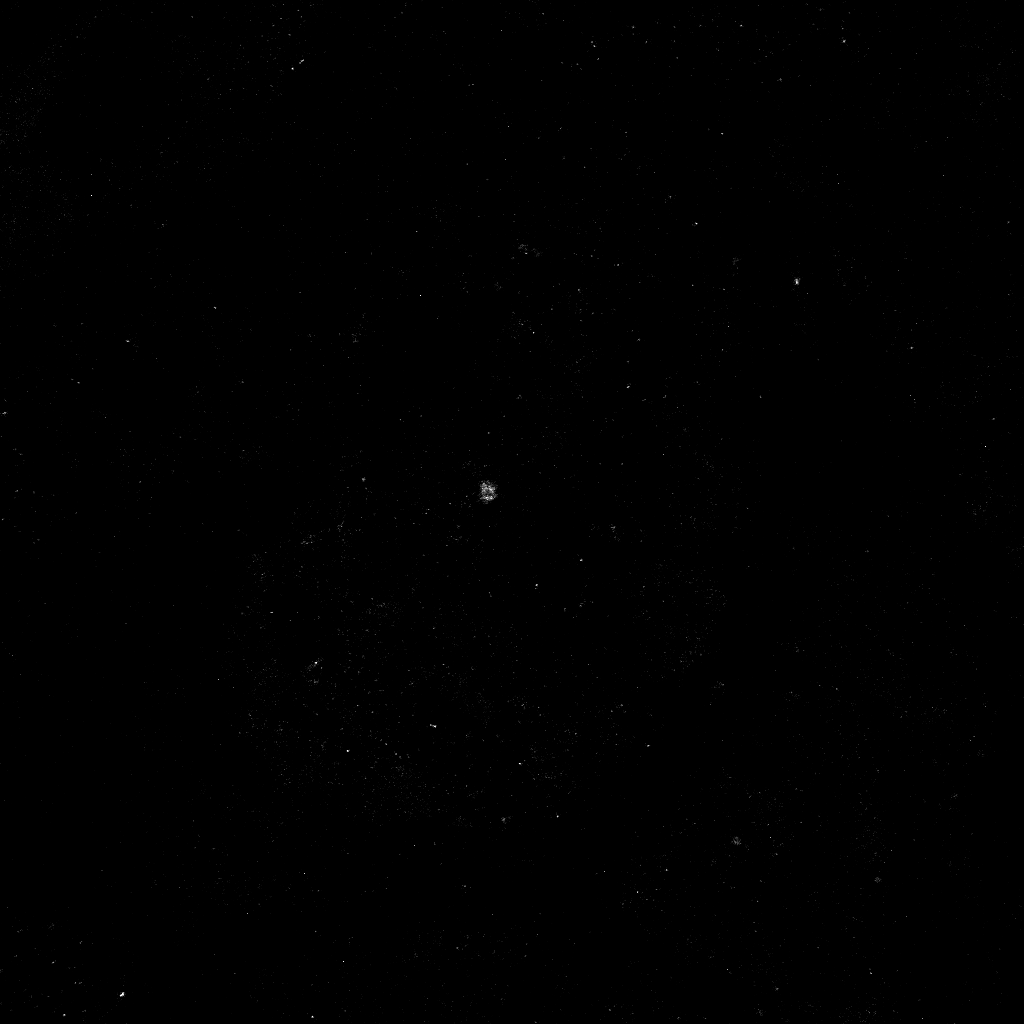

Supplement: Supplementary file 6 — Source data Fig. 4 [file 44318_2025_434_MOESM6_ESM.zip › Figure 4/4I/4I_DT_Merge (gray).tif]

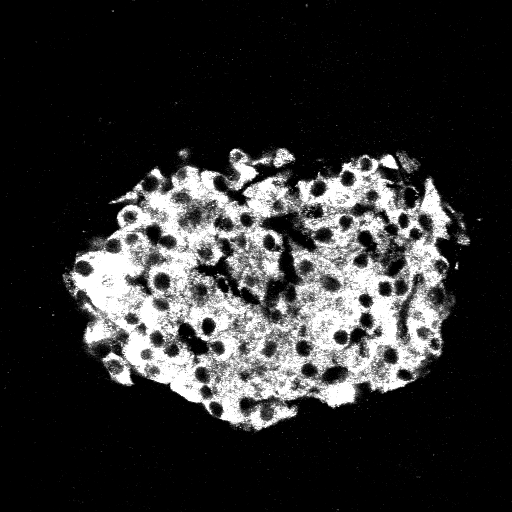

Supplement: Supplementary file 6 — Source data Fig. 4 [file 44318_2025_434_MOESM6_ESM.zip › Figure 4/4I/4I_PBS_Merge (gray).tif]

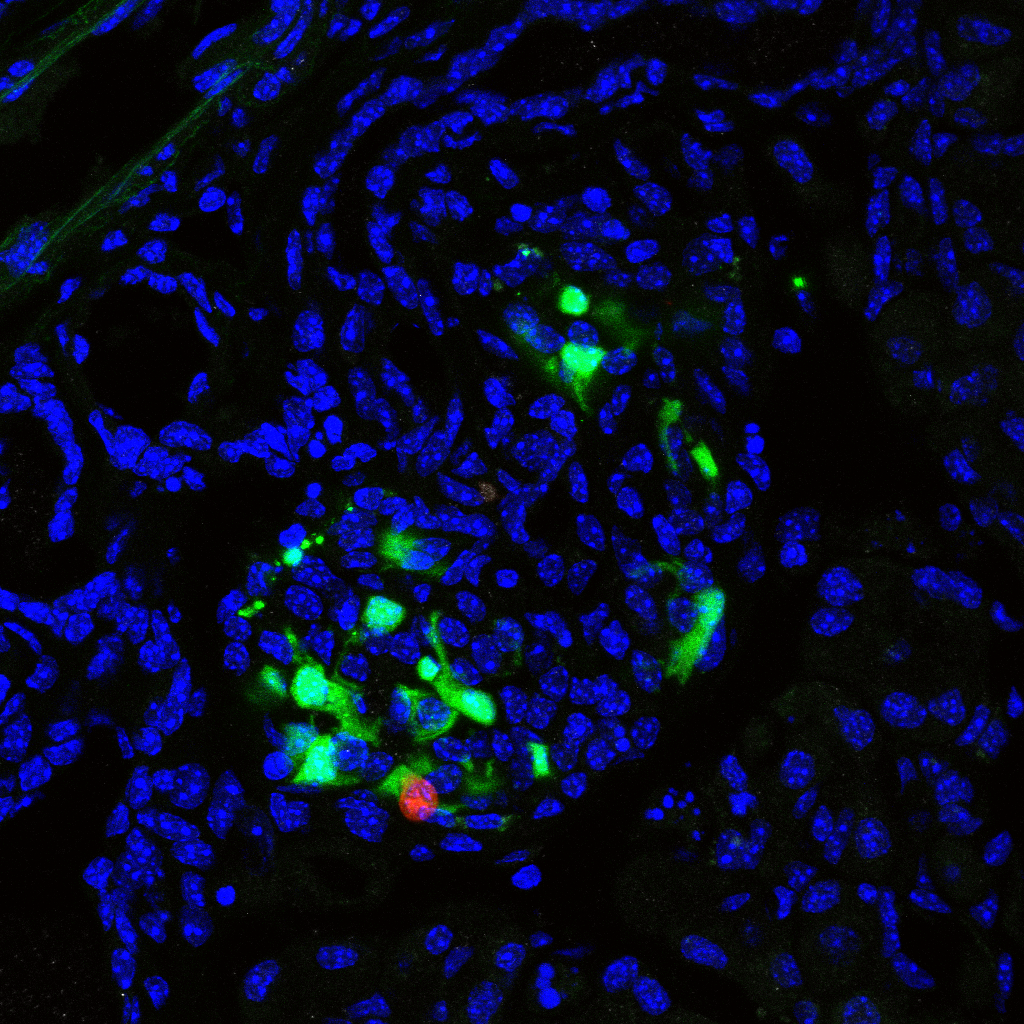

Supplement: Supplementary file 6 — Source data Fig. 4 [file 44318_2025_434_MOESM6_ESM.zip › Figure 4/4I/4I_DT_Merge.tif]

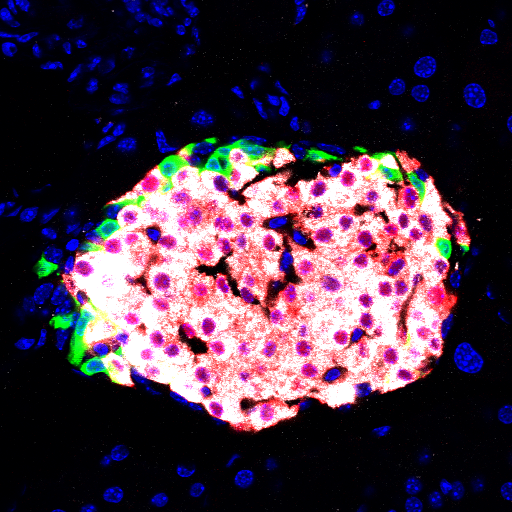

Supplement: Supplementary file 6 — Source data Fig. 4 [file 44318_2025_434_MOESM6_ESM.zip › Figure 4/4I/4I_PBS_Merge.tif]

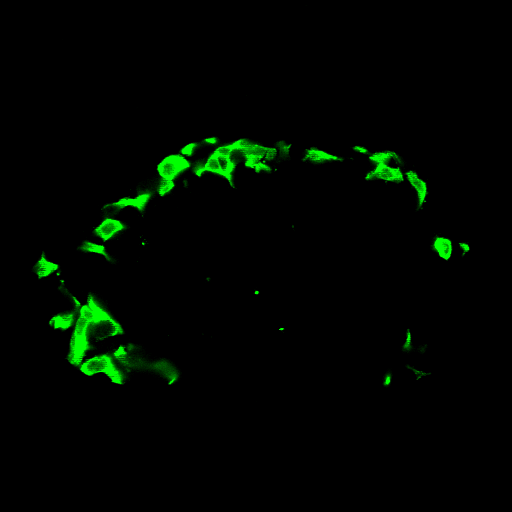

Supplement: Supplementary file 6 — Source data Fig. 4 [file 44318_2025_434_MOESM6_ESM.zip › Figure 4/4I/4I_PBS_Merge (green).tif]

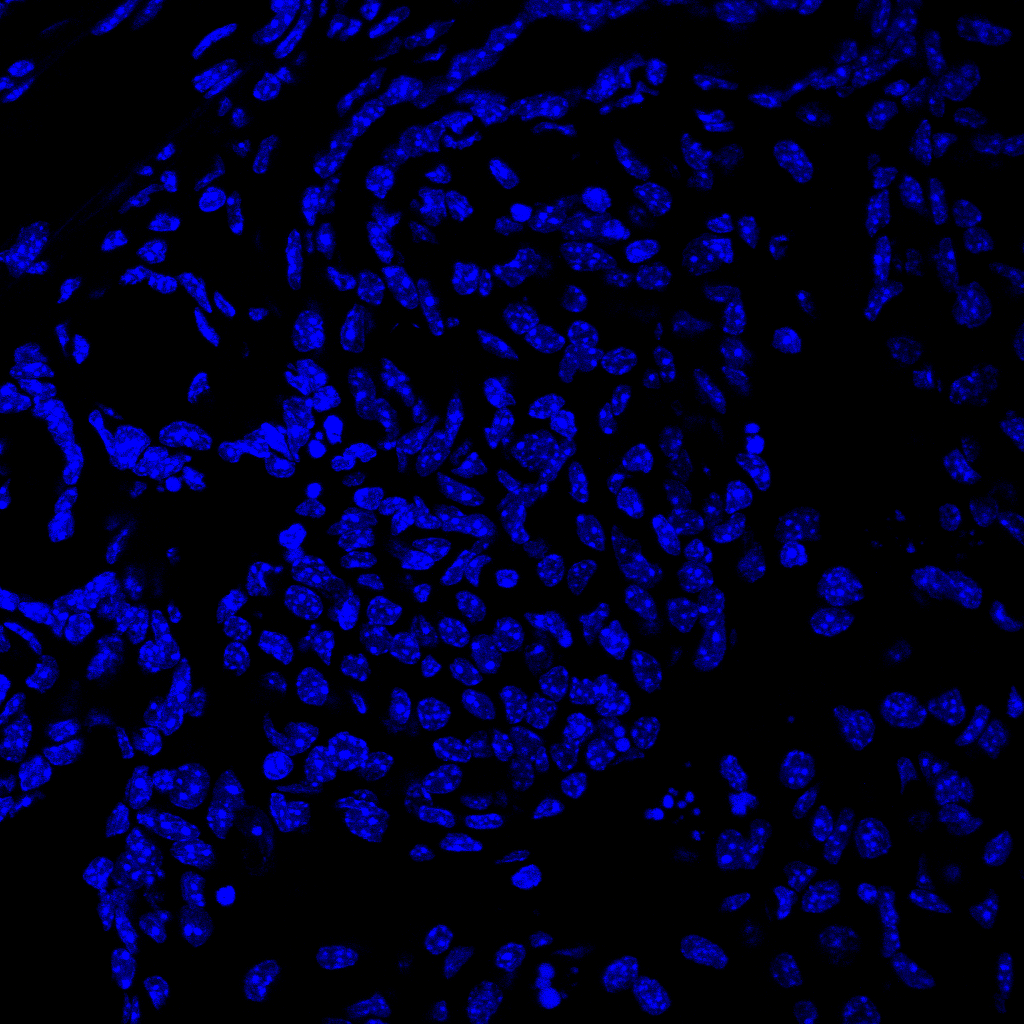

Supplement: Supplementary file 6 — Source data Fig. 4 [file 44318_2025_434_MOESM6_ESM.zip › Figure 4/4I/4I_DT_Merge (blue).tif]

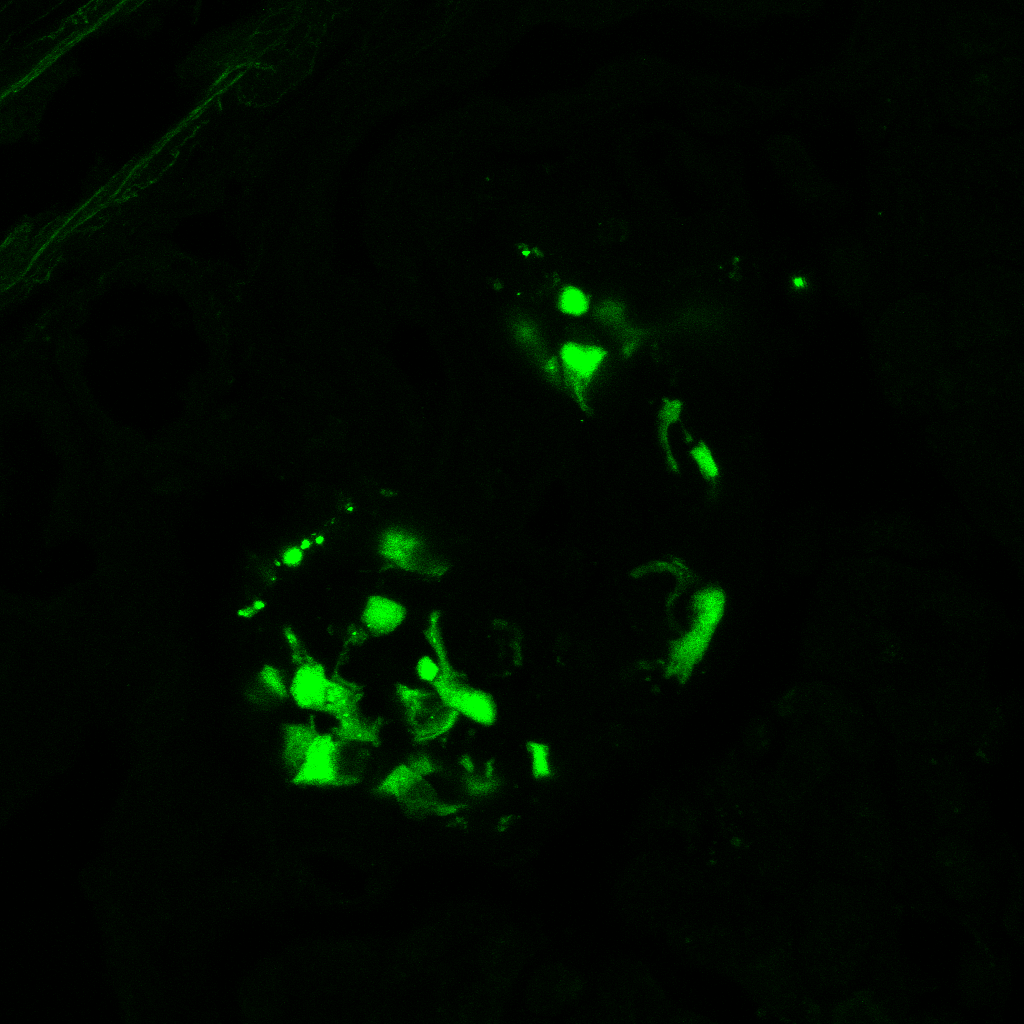

Supplement: Supplementary file 6 — Source data Fig. 4 [file 44318_2025_434_MOESM6_ESM.zip › Figure 4/4I/4I_DT_Merge (green).tif]

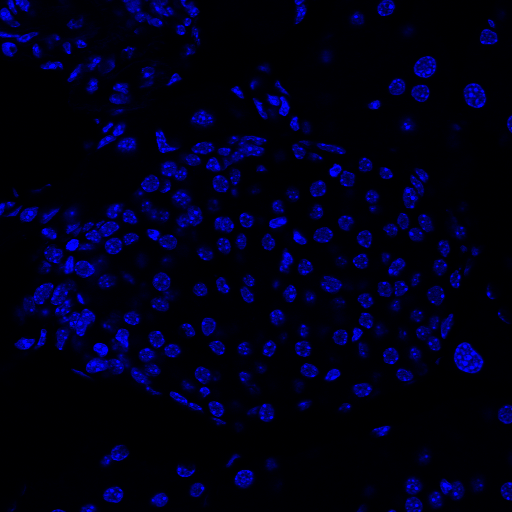

Supplement: Supplementary file 6 — Source data Fig. 4 [file 44318_2025_434_MOESM6_ESM.zip › Figure 4/4I/4I_PBS_Merge (blue).tif]

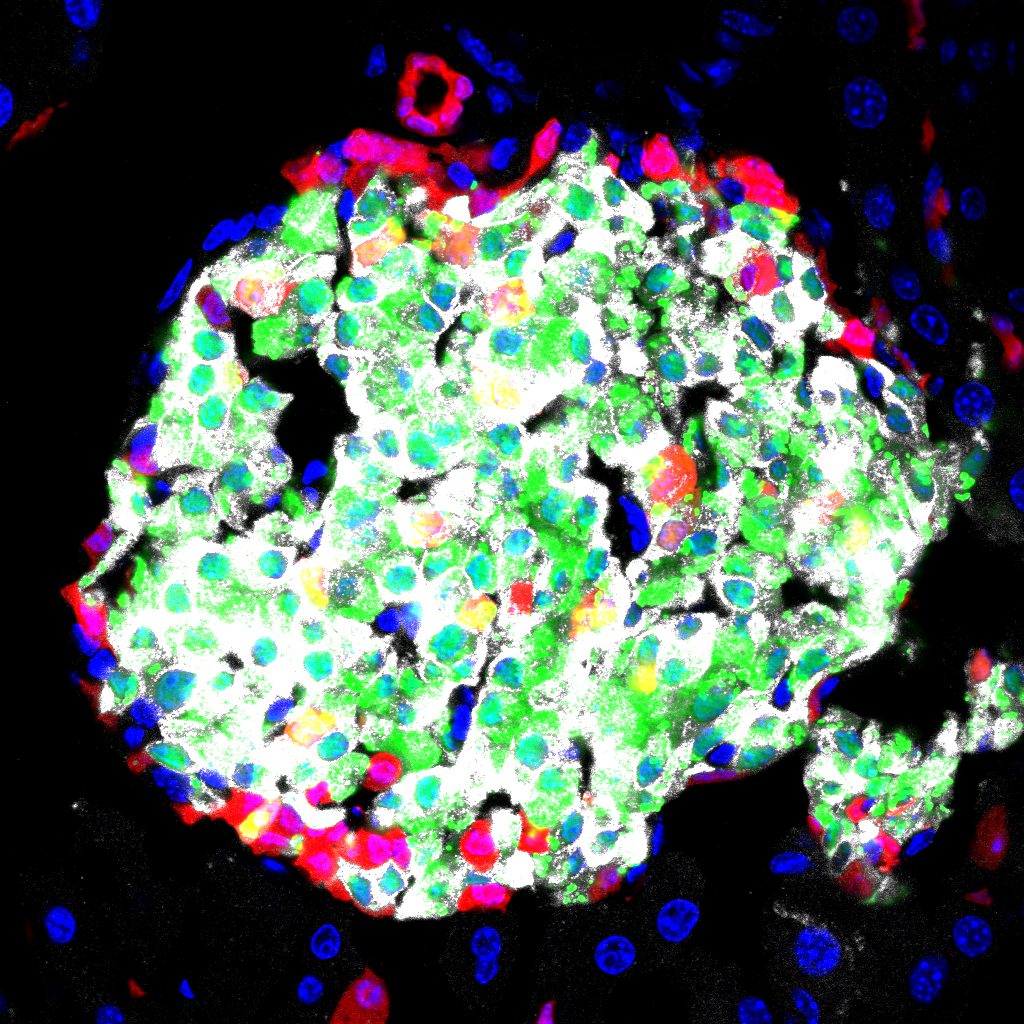

Supplement: Supplementary file 7 — Source data Fig. 5 [file 44318_2025_434_MOESM7_ESM.zip › Figure 5/5I/5I_Merge.tif]

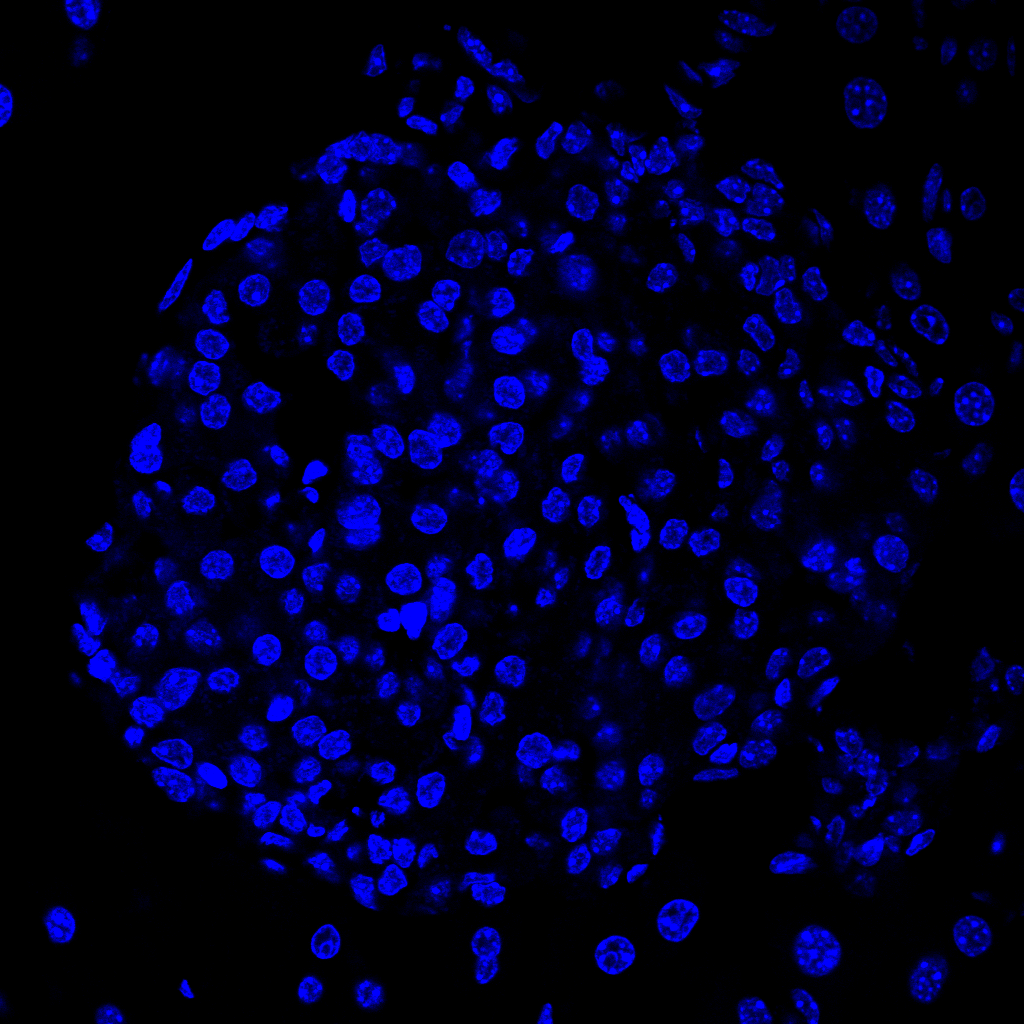

Supplement: Supplementary file 7 — Source data Fig. 5 [file 44318_2025_434_MOESM7_ESM.zip › Figure 5/5I/5I_Merge (blue).tif]

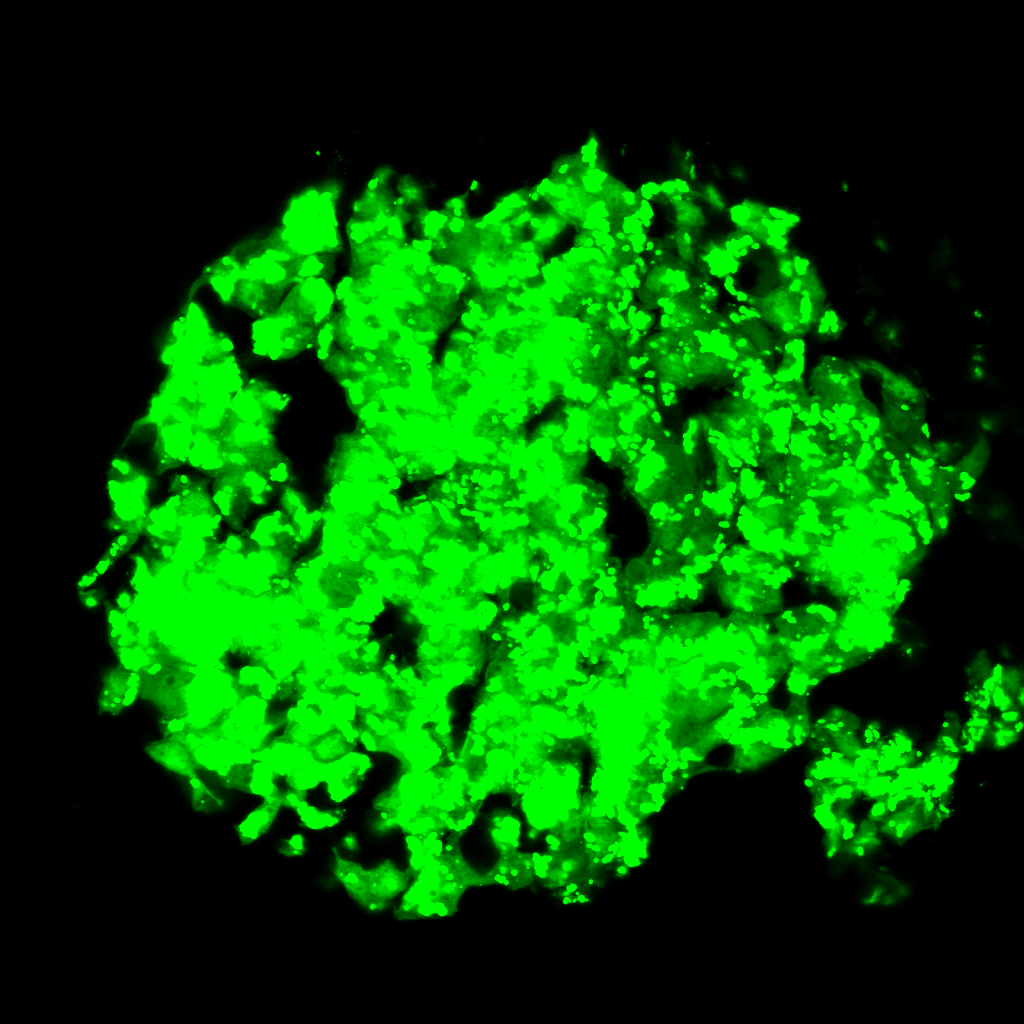

Supplement: Supplementary file 7 — Source data Fig. 5 [file 44318_2025_434_MOESM7_ESM.zip › Figure 5/5I/5I_Merge (green).tif]

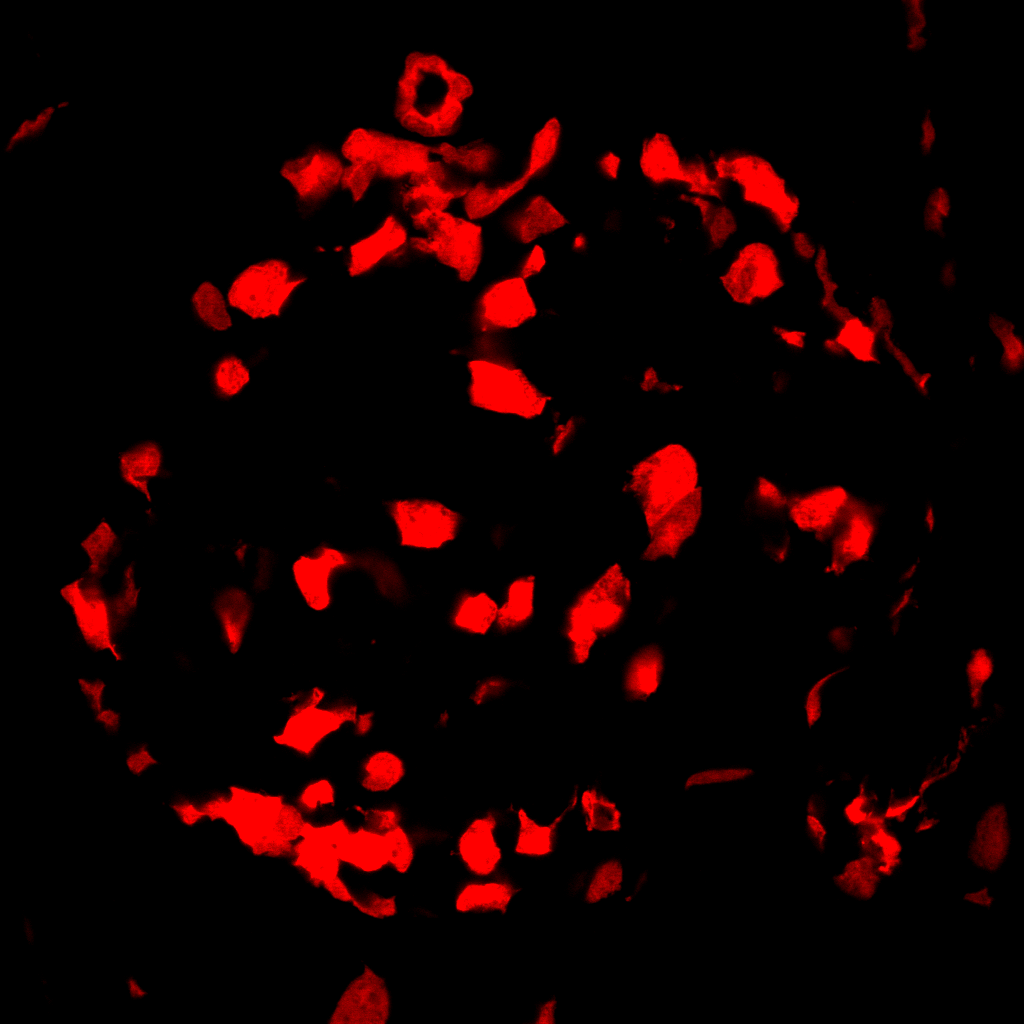

Supplement: Supplementary file 7 — Source data Fig. 5 [file 44318_2025_434_MOESM7_ESM.zip › Figure 5/5I/5I_Merge (red).tif]

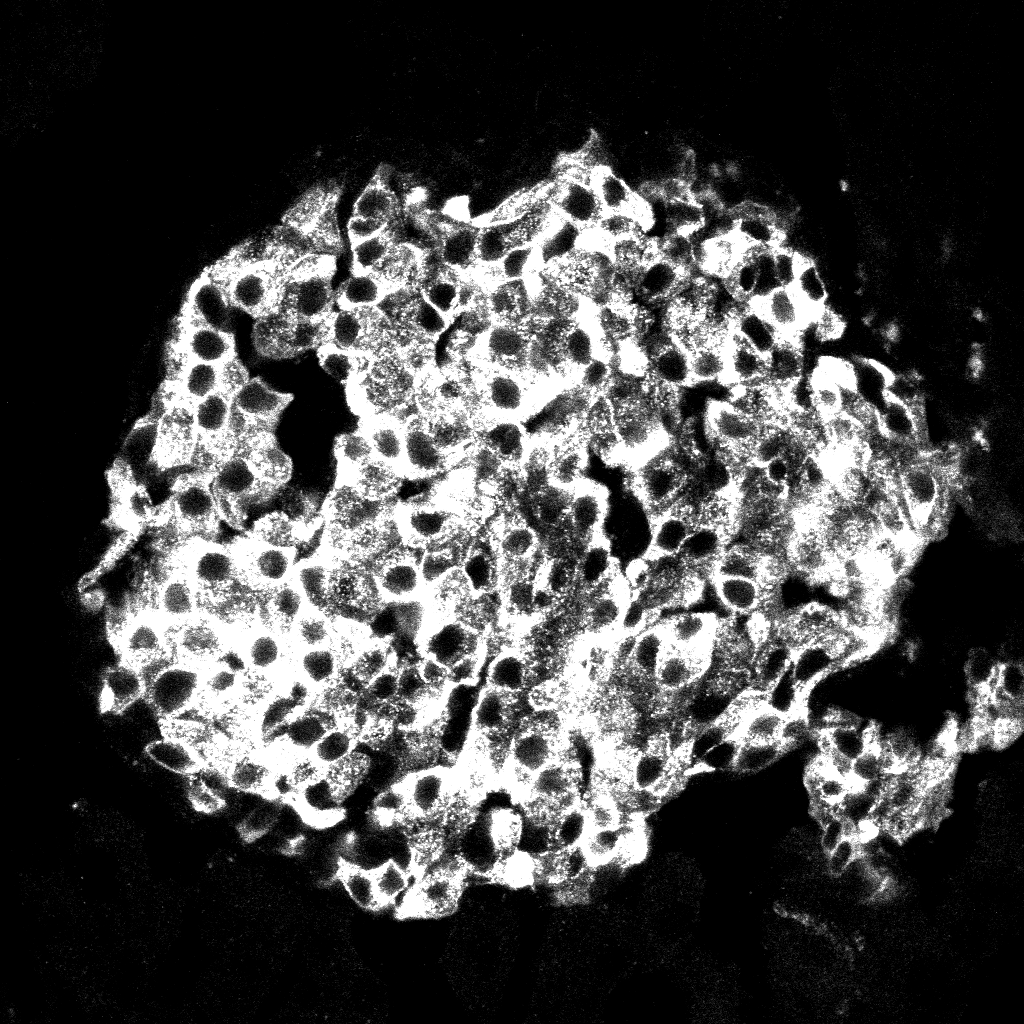

Supplement: Supplementary file 7 — Source data Fig. 5 [file 44318_2025_434_MOESM7_ESM.zip › Figure 5/5I/5I_Merge (gray).tif]

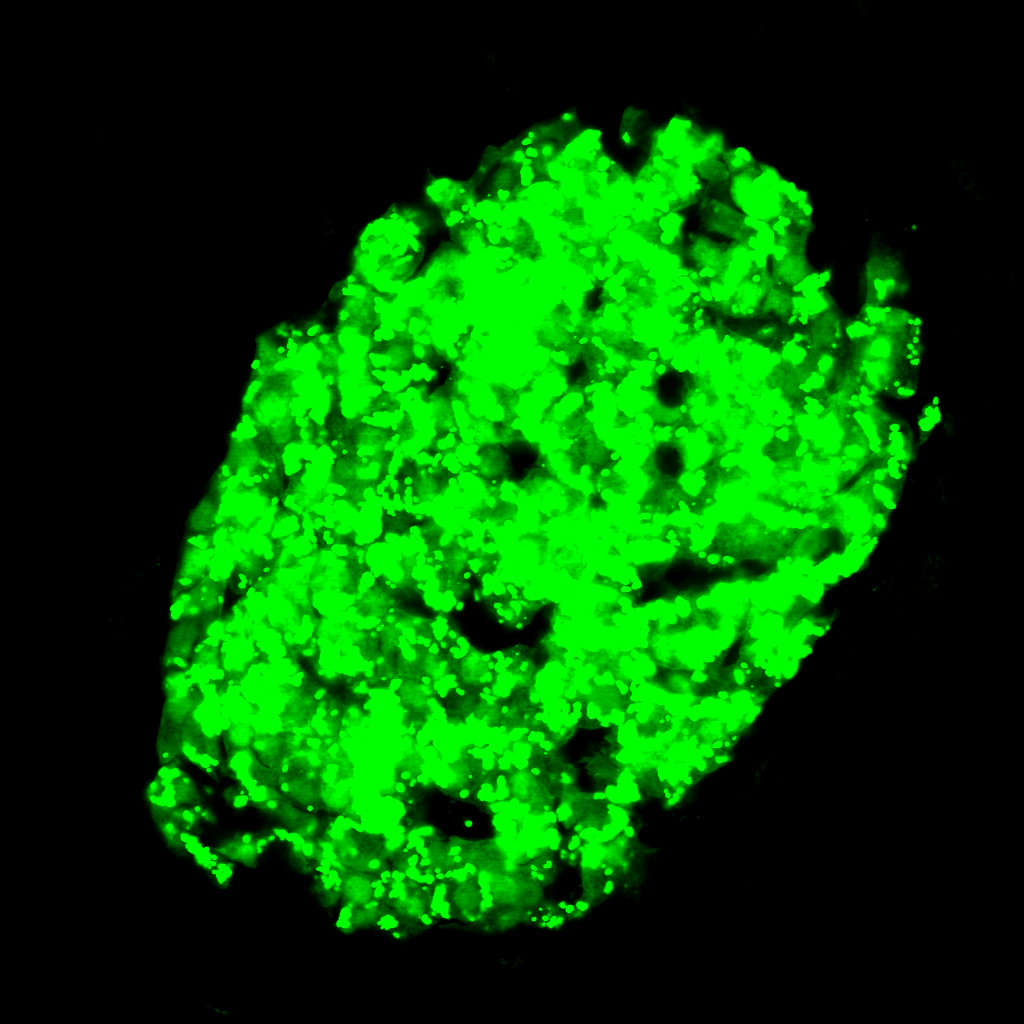

Supplement: Supplementary file 7 — Source data Fig. 5 [file 44318_2025_434_MOESM7_ESM.zip › Figure 5/5G/5G_Merge (green).tif]

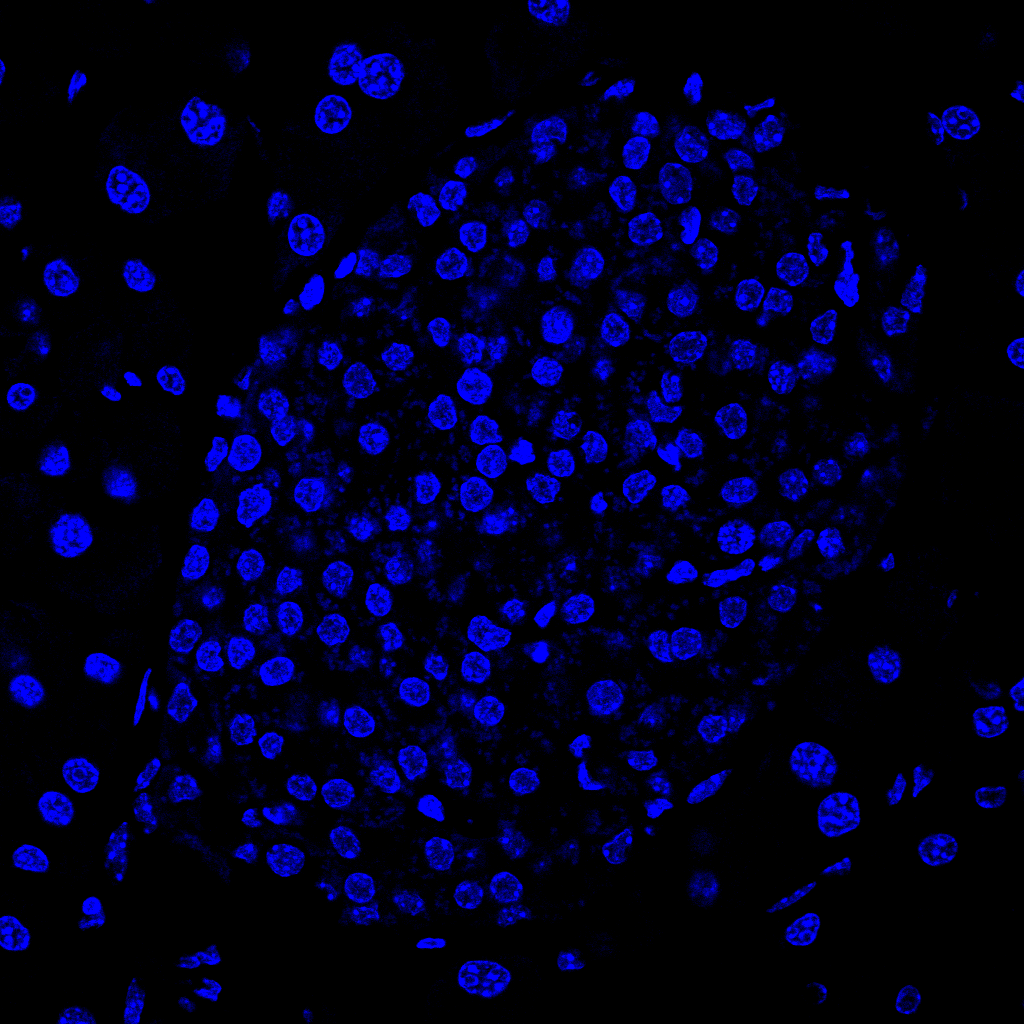

Supplement: Supplementary file 7 — Source data Fig. 5 [file 44318_2025_434_MOESM7_ESM.zip › Figure 5/5G/5G_Merge (blue).tif]

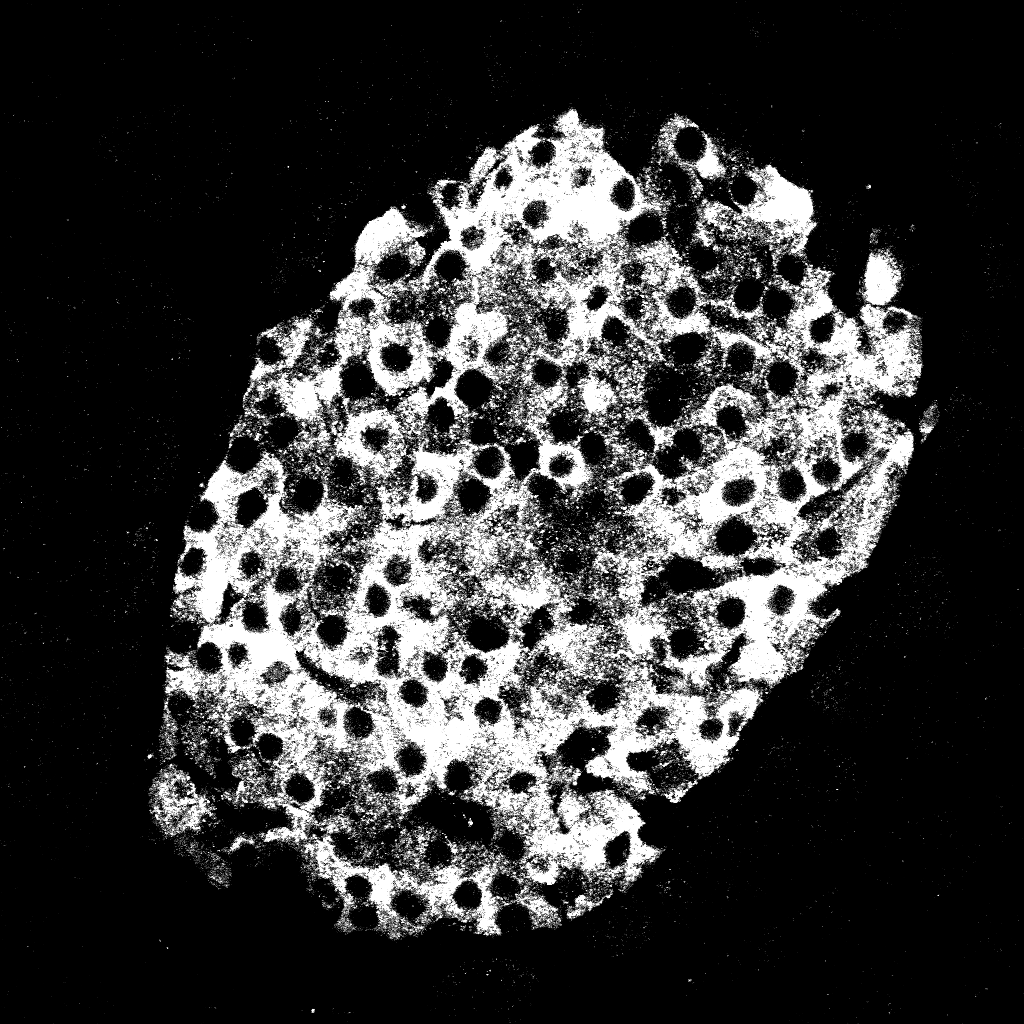

Supplement: Supplementary file 7 — Source data Fig. 5 [file 44318_2025_434_MOESM7_ESM.zip › Figure 5/5G/5G_Merge (gray).tif]

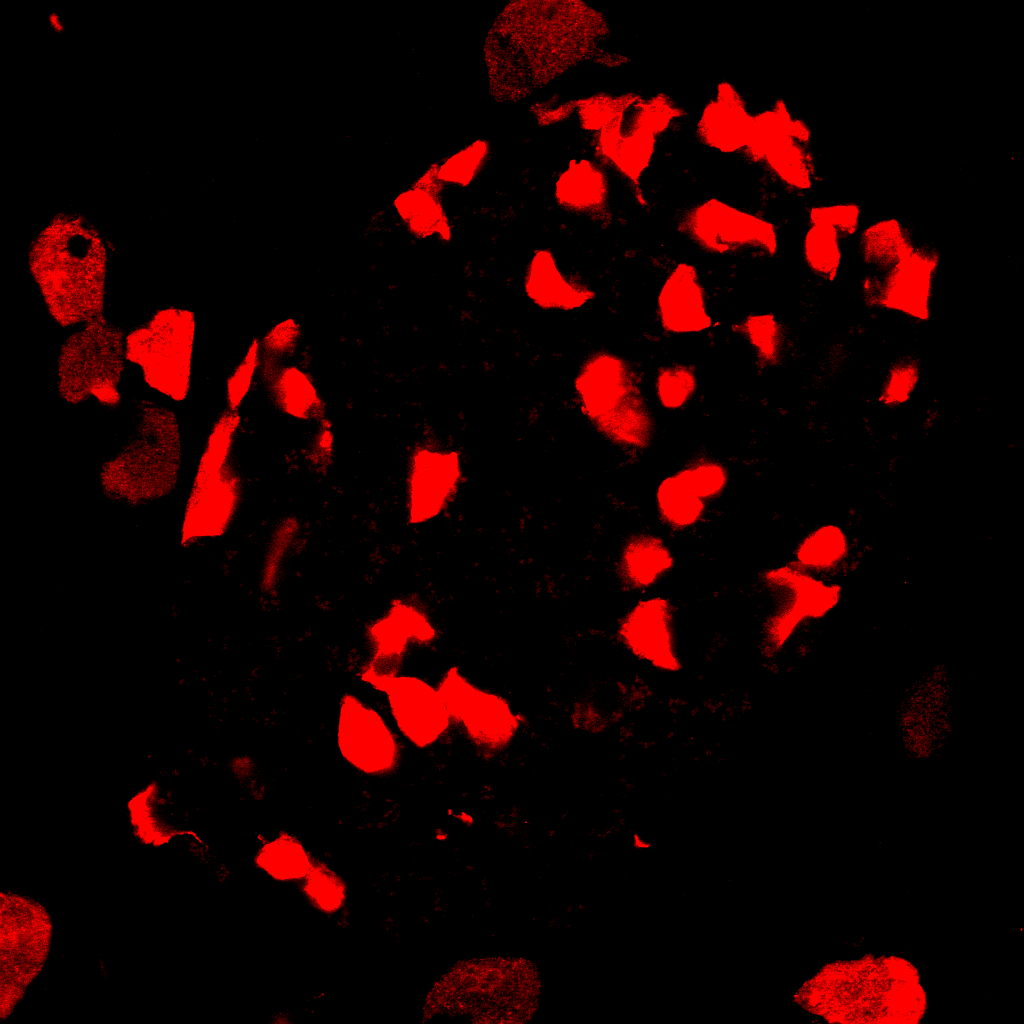

Supplement: Supplementary file 7 — Source data Fig. 5 [file 44318_2025_434_MOESM7_ESM.zip › Figure 5/5G/5G_Merge (red).tif]

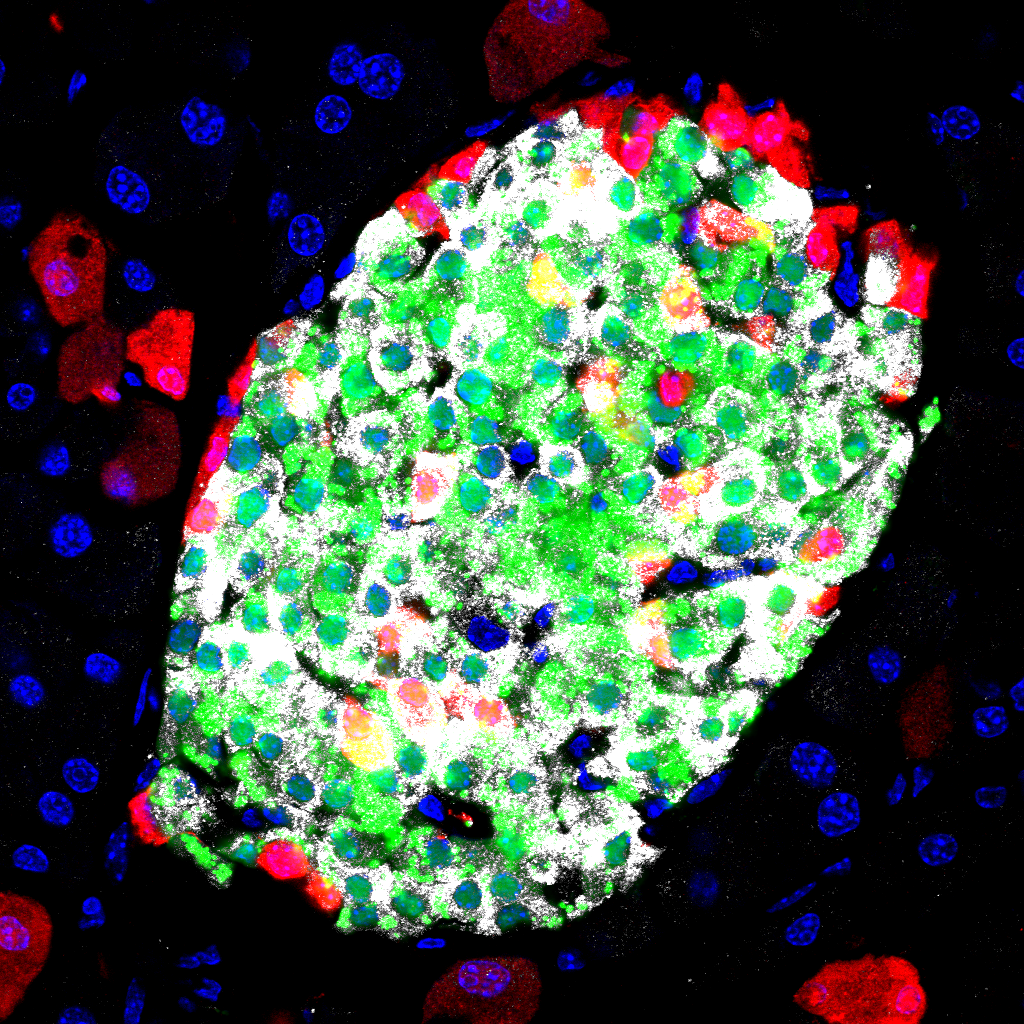

Supplement: Supplementary file 7 — Source data Fig. 5 [file 44318_2025_434_MOESM7_ESM.zip › Figure 5/5G/5G_Merge.tif]

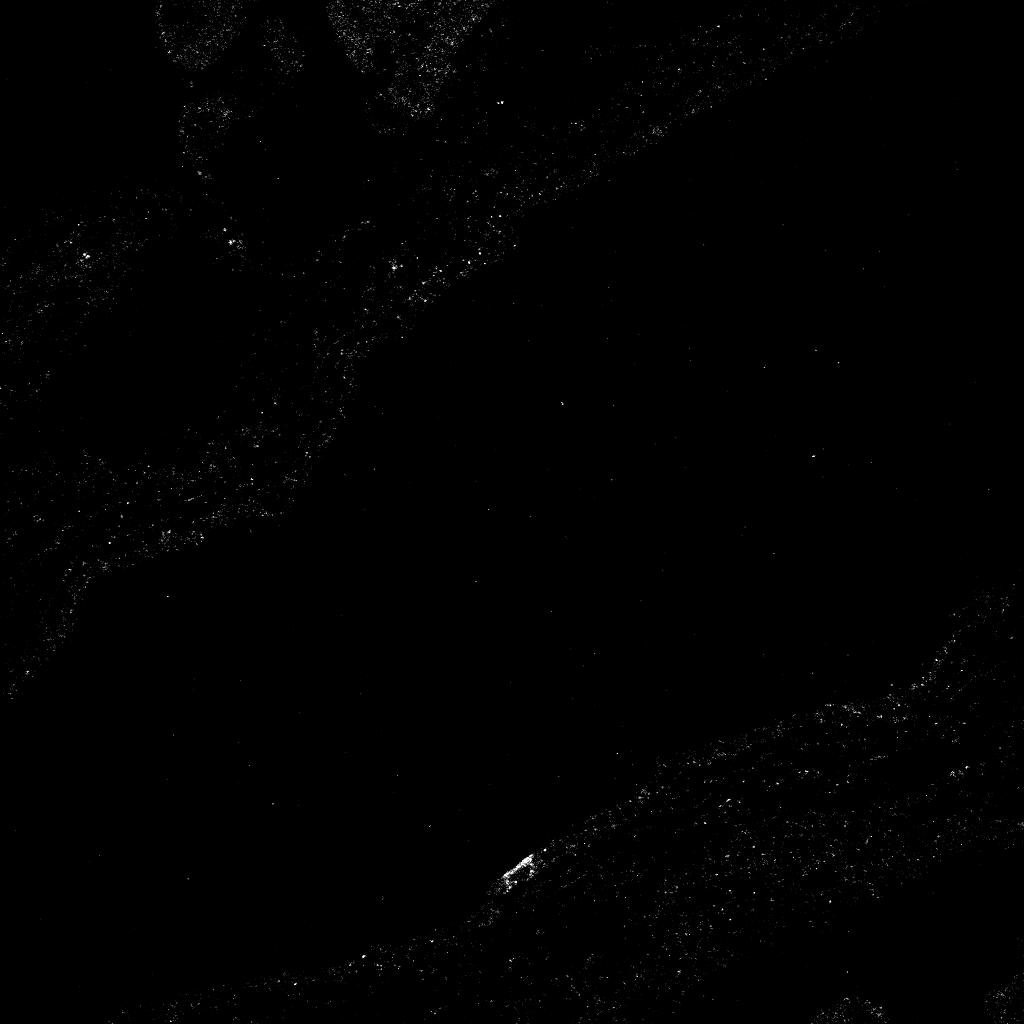

Supplement: Supplementary file 7 — Source data Fig. 5 [file 44318_2025_434_MOESM7_ESM.zip › Figure 5/5F/5F_Ins_Merge (gray).tif]

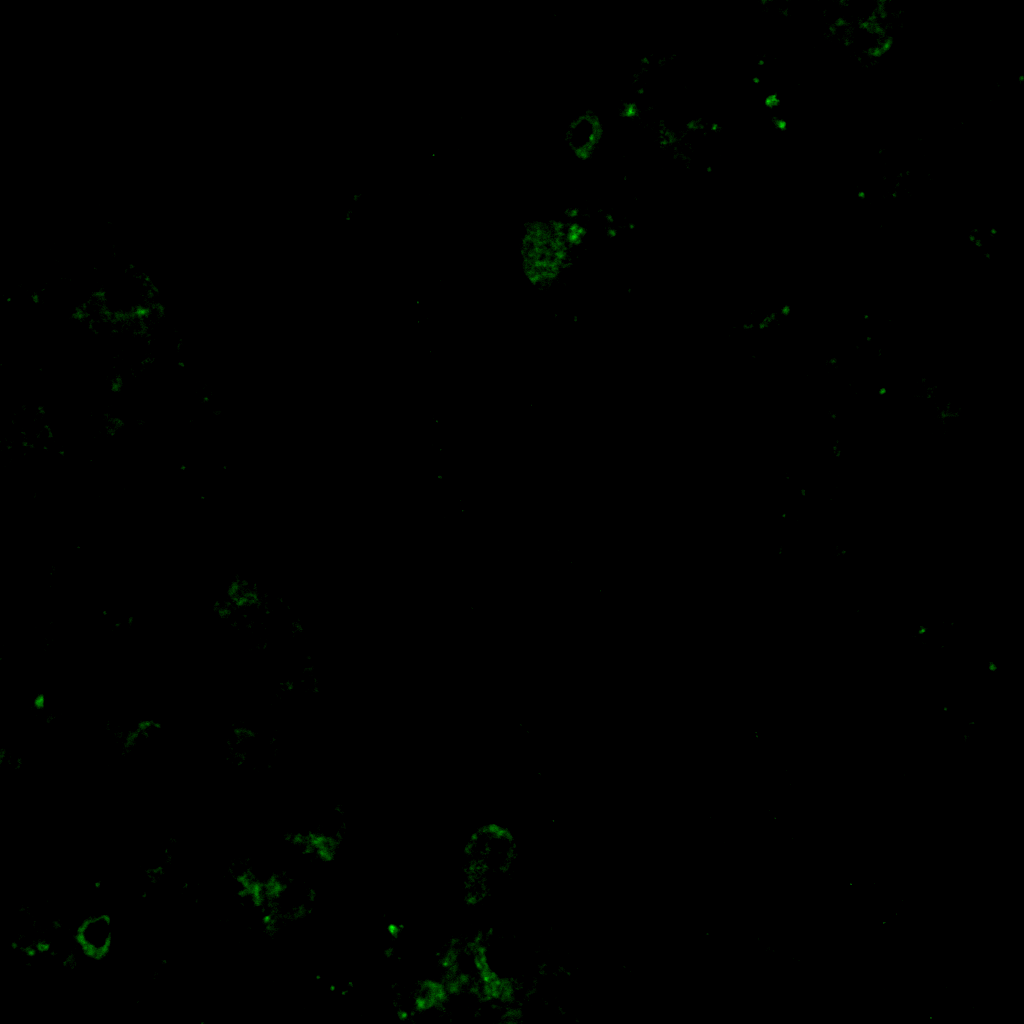

Supplement: Supplementary file 7 — Source data Fig. 5 [file 44318_2025_434_MOESM7_ESM.zip › Figure 5/5F/5F_Sst_Merge (green).tif]

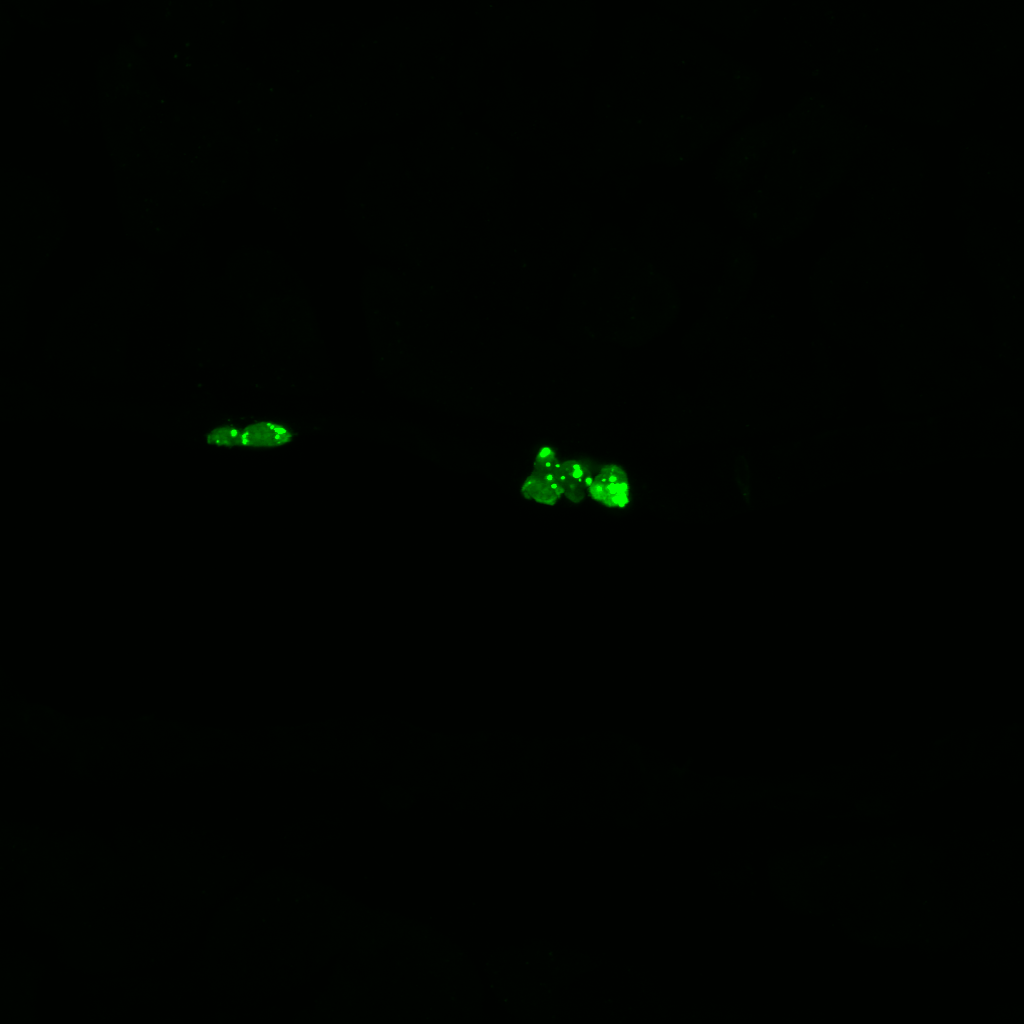

Supplement: Supplementary file 7 — Source data Fig. 5 [file 44318_2025_434_MOESM7_ESM.zip › Figure 5/5F/5F_CK19_Merge (green).tif]

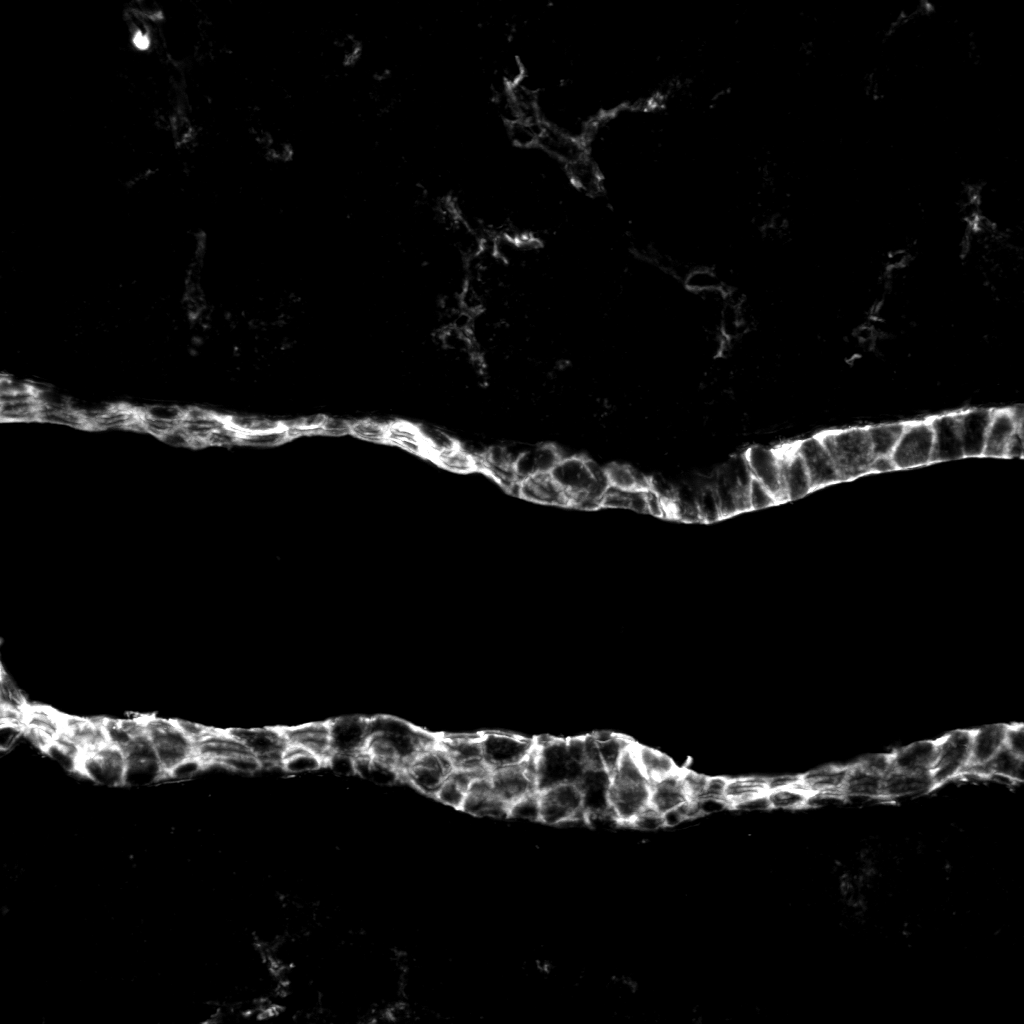

Supplement: Supplementary file 7 — Source data Fig. 5 [file 44318_2025_434_MOESM7_ESM.zip › Figure 5/5F/5F_CK19_Merge (gray).tif]

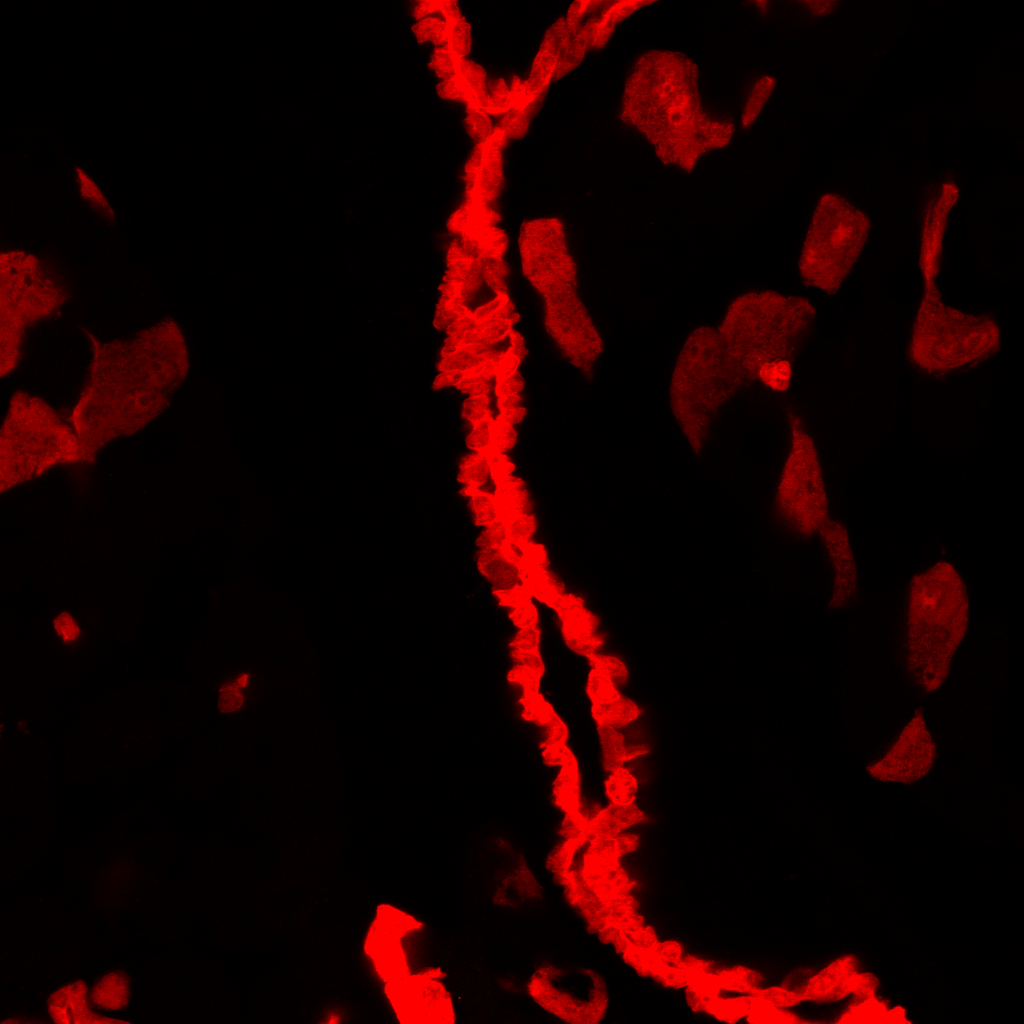

Supplement: Supplementary file 7 — Source data Fig. 5 [file 44318_2025_434_MOESM7_ESM.zip › Figure 5/5F/5F_Sst_Merge (red).tif]

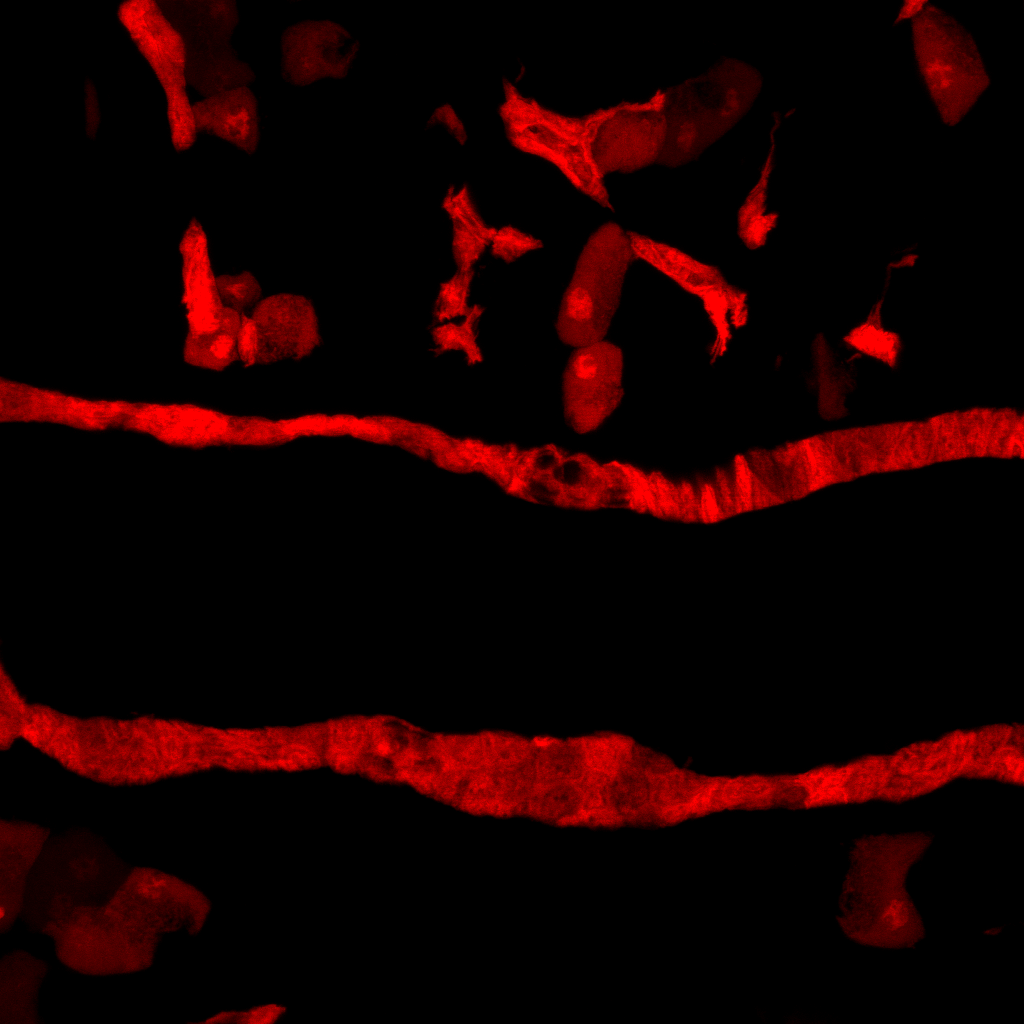

Supplement: Supplementary file 7 — Source data Fig. 5 [file 44318_2025_434_MOESM7_ESM.zip › Figure 5/5F/5F_CK19_Merge (red).tif]

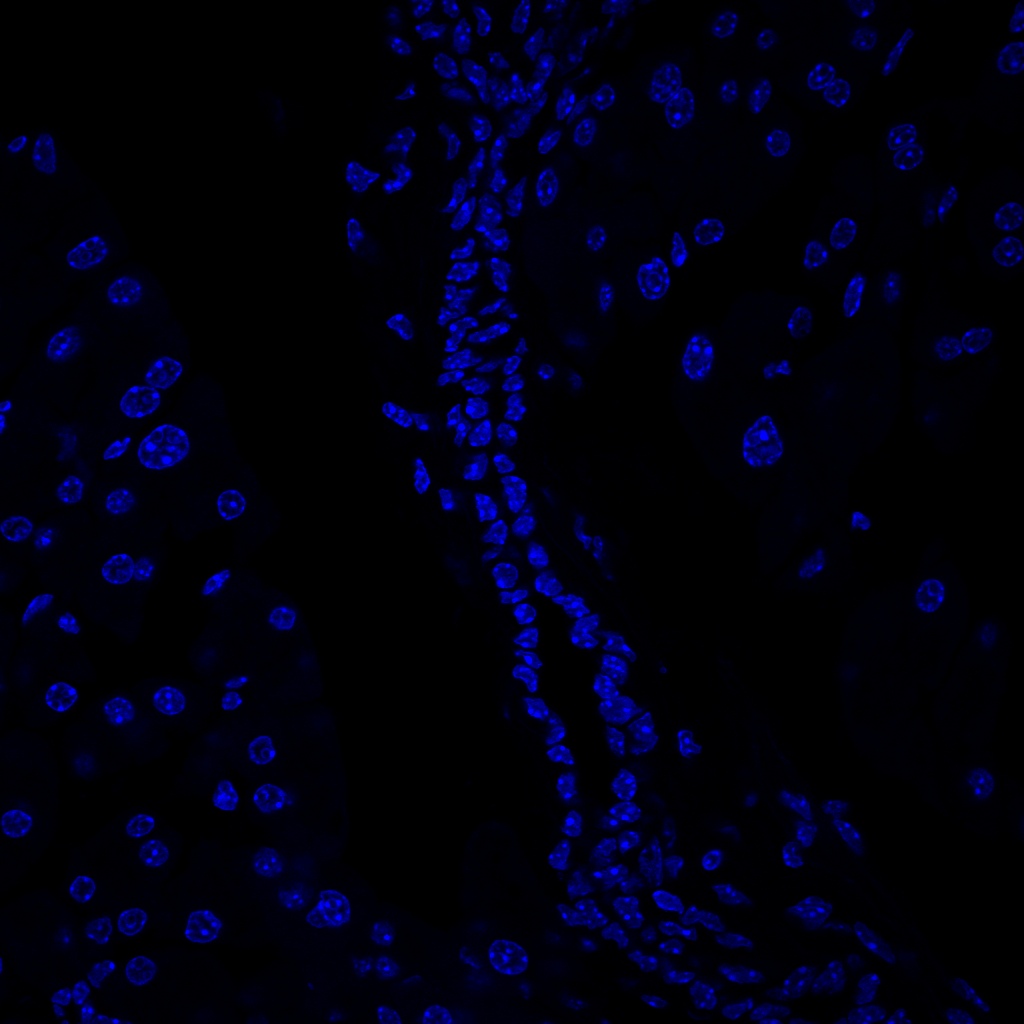

Supplement: Supplementary file 7 — Source data Fig. 5 [file 44318_2025_434_MOESM7_ESM.zip › Figure 5/5F/5F_Sst_Merge (blue).tif]

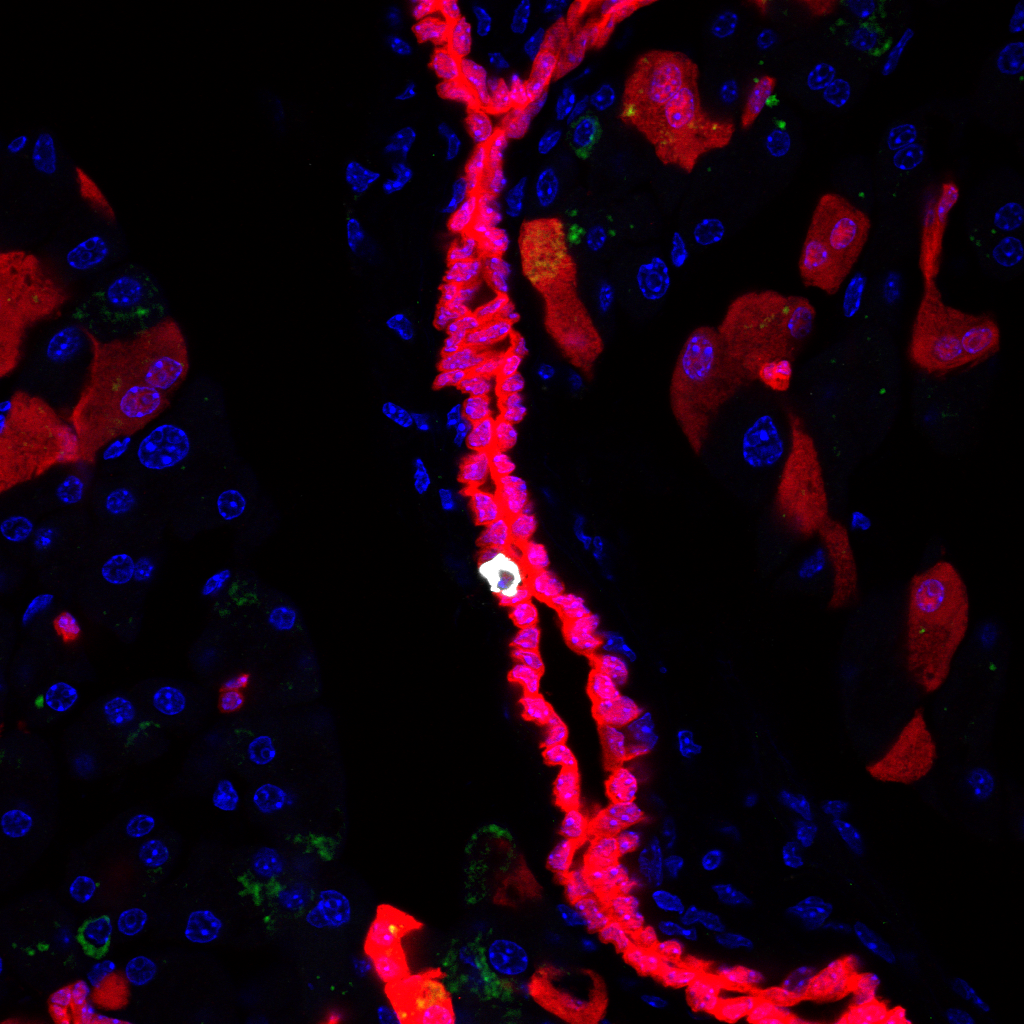

Supplement: Supplementary file 7 — Source data Fig. 5 [file 44318_2025_434_MOESM7_ESM.zip › Figure 5/5F/5F_Sst_Merge.tif]

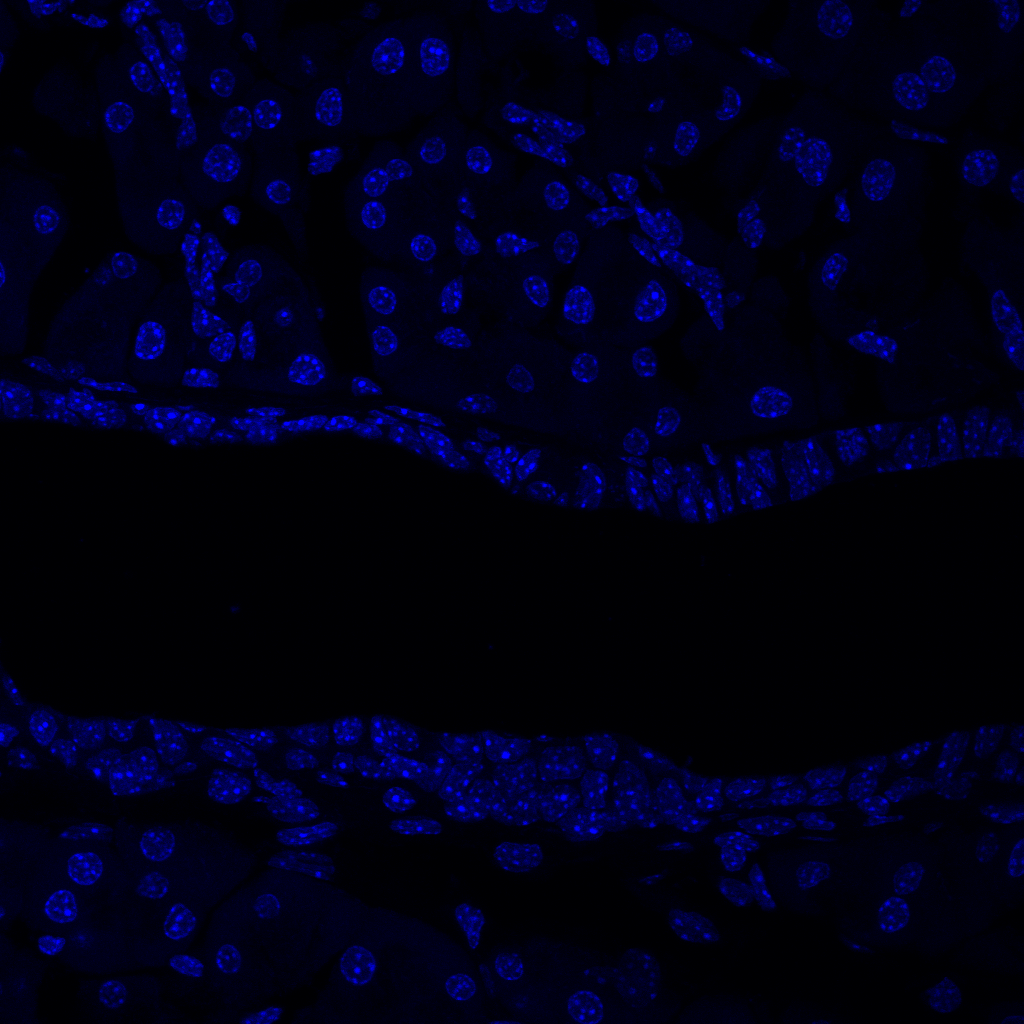

Supplement: Supplementary file 7 — Source data Fig. 5 [file 44318_2025_434_MOESM7_ESM.zip › Figure 5/5F/5F_CK19_Merge (blue).tif]

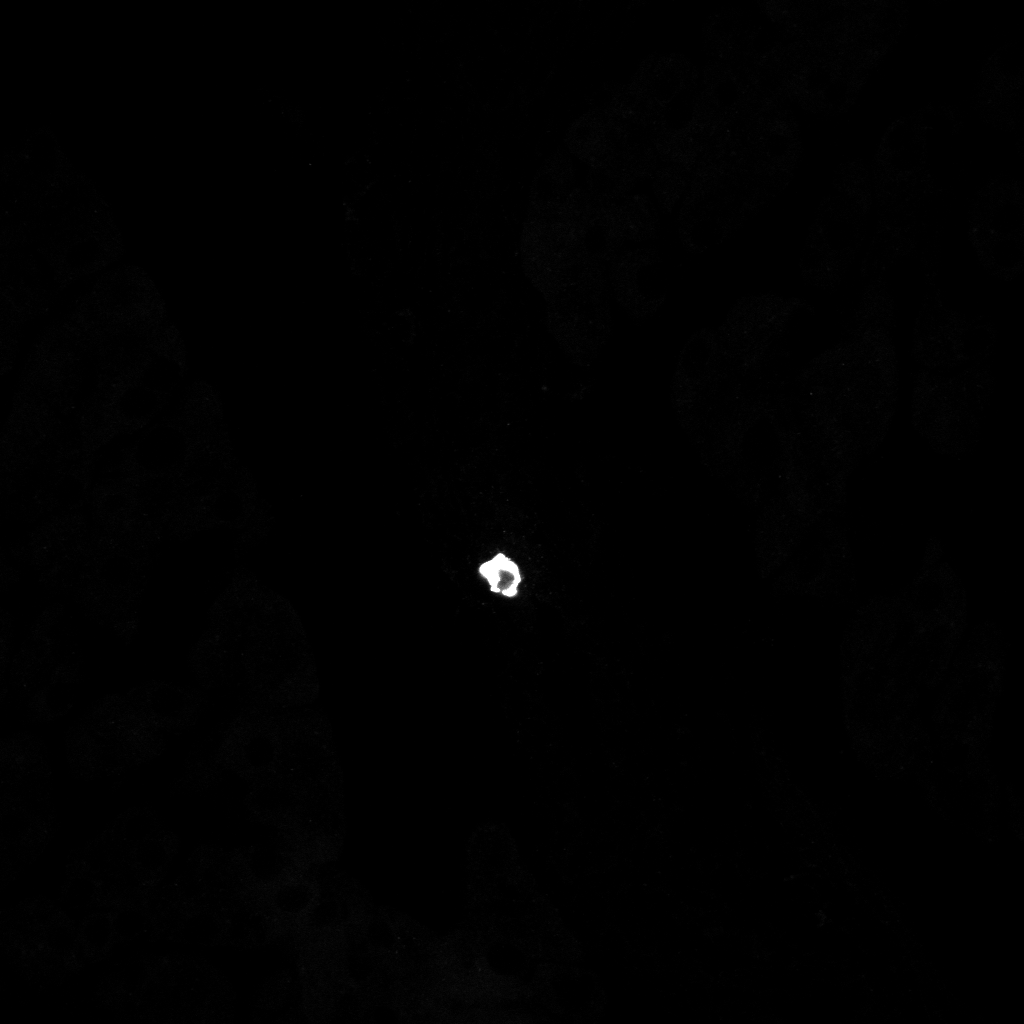

Supplement: Supplementary file 7 — Source data Fig. 5 [file 44318_2025_434_MOESM7_ESM.zip › Figure 5/5F/5F_Sst_Merge (gray).tif]

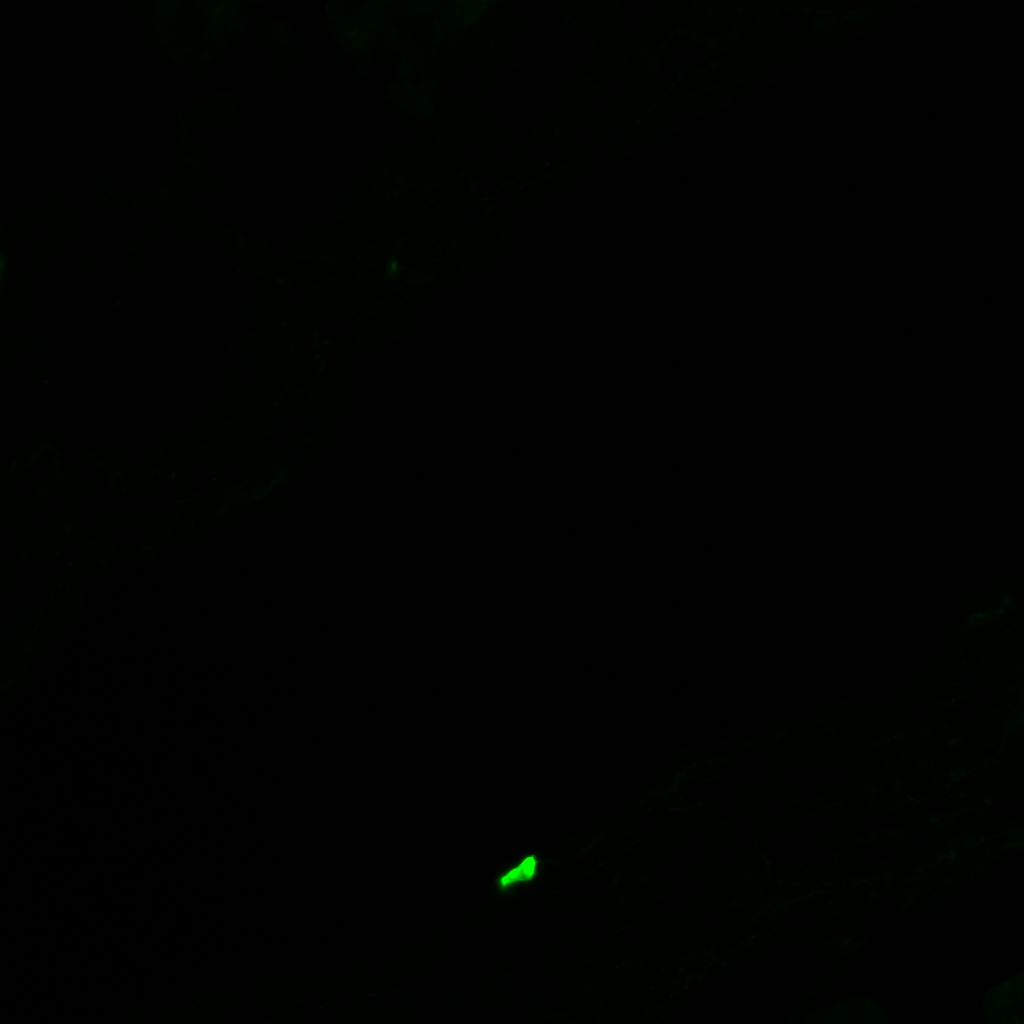

Supplement: Supplementary file 7 — Source data Fig. 5 [file 44318_2025_434_MOESM7_ESM.zip › Figure 5/5F/5F_Ins_Merge (green).tif]

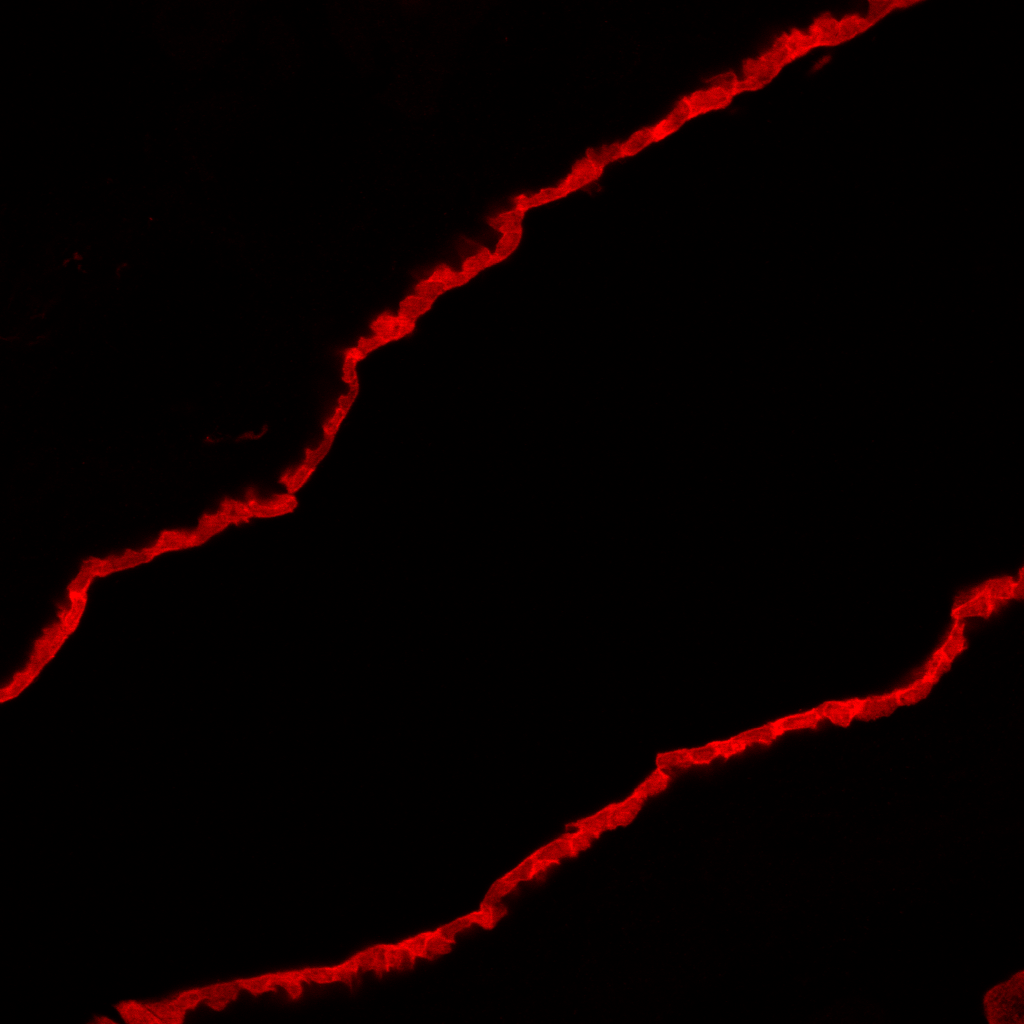

Supplement: Supplementary file 7 — Source data Fig. 5 [file 44318_2025_434_MOESM7_ESM.zip › Figure 5/5F/5F_Ins_Merge (red).tif]

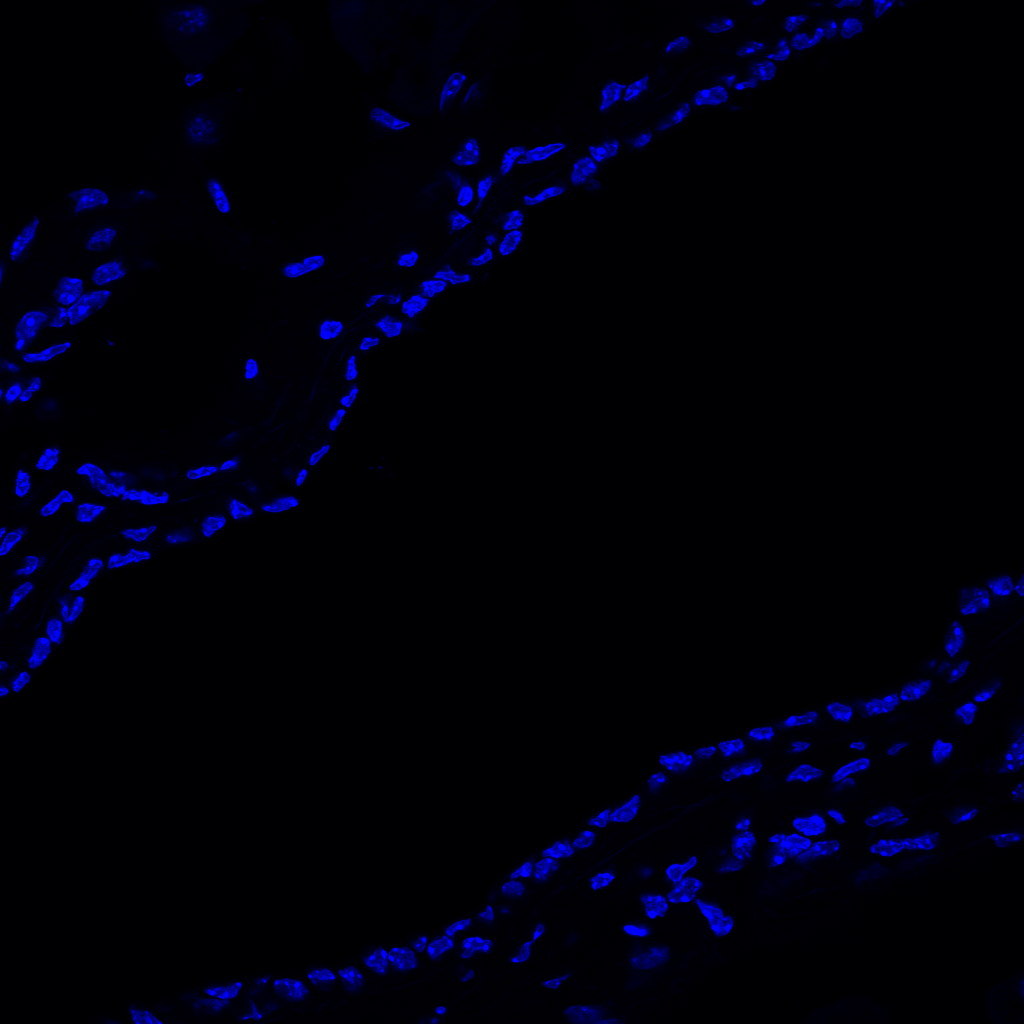

Supplement: Supplementary file 7 — Source data Fig. 5 [file 44318_2025_434_MOESM7_ESM.zip › Figure 5/5F/5F_Ins_Merge (blue).tif]

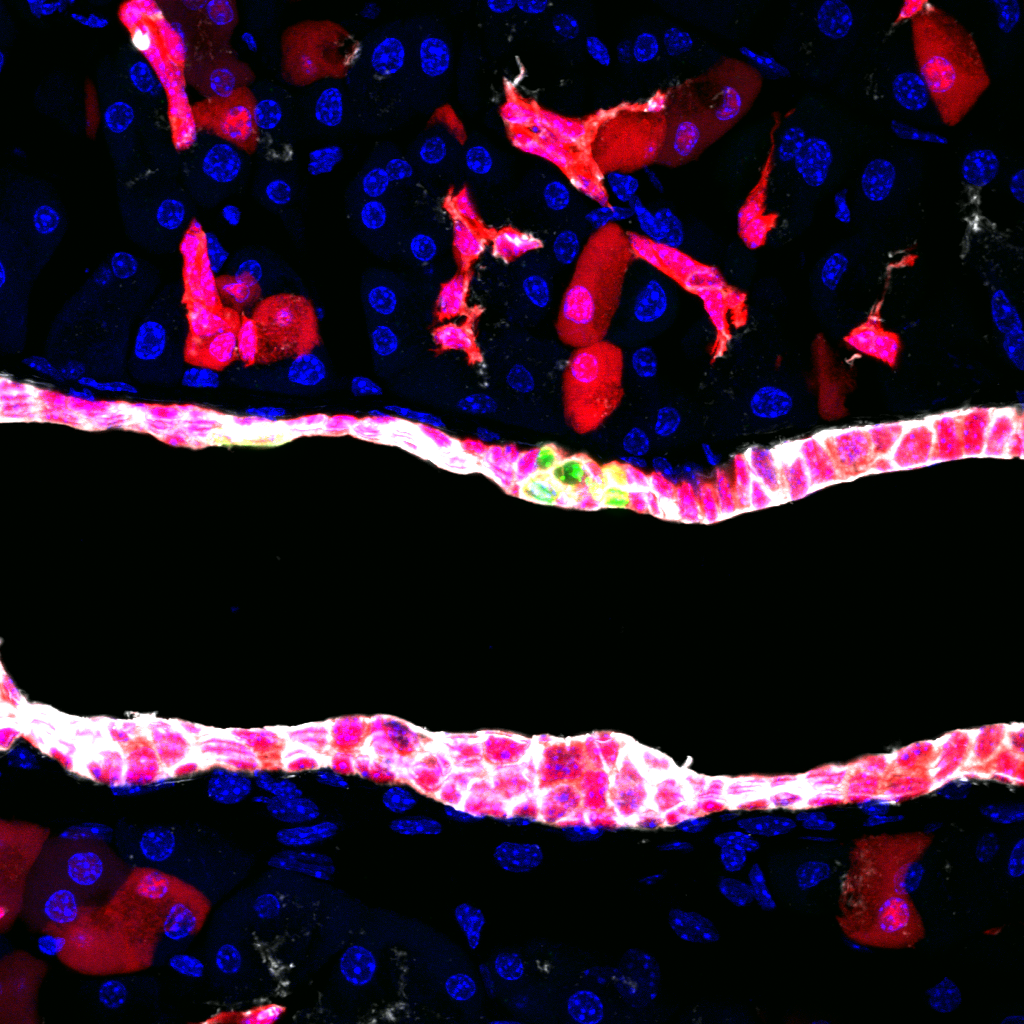

Supplement: Supplementary file 7 — Source data Fig. 5 [file 44318_2025_434_MOESM7_ESM.zip › Figure 5/5F/5F_CK19_Merge.tif]

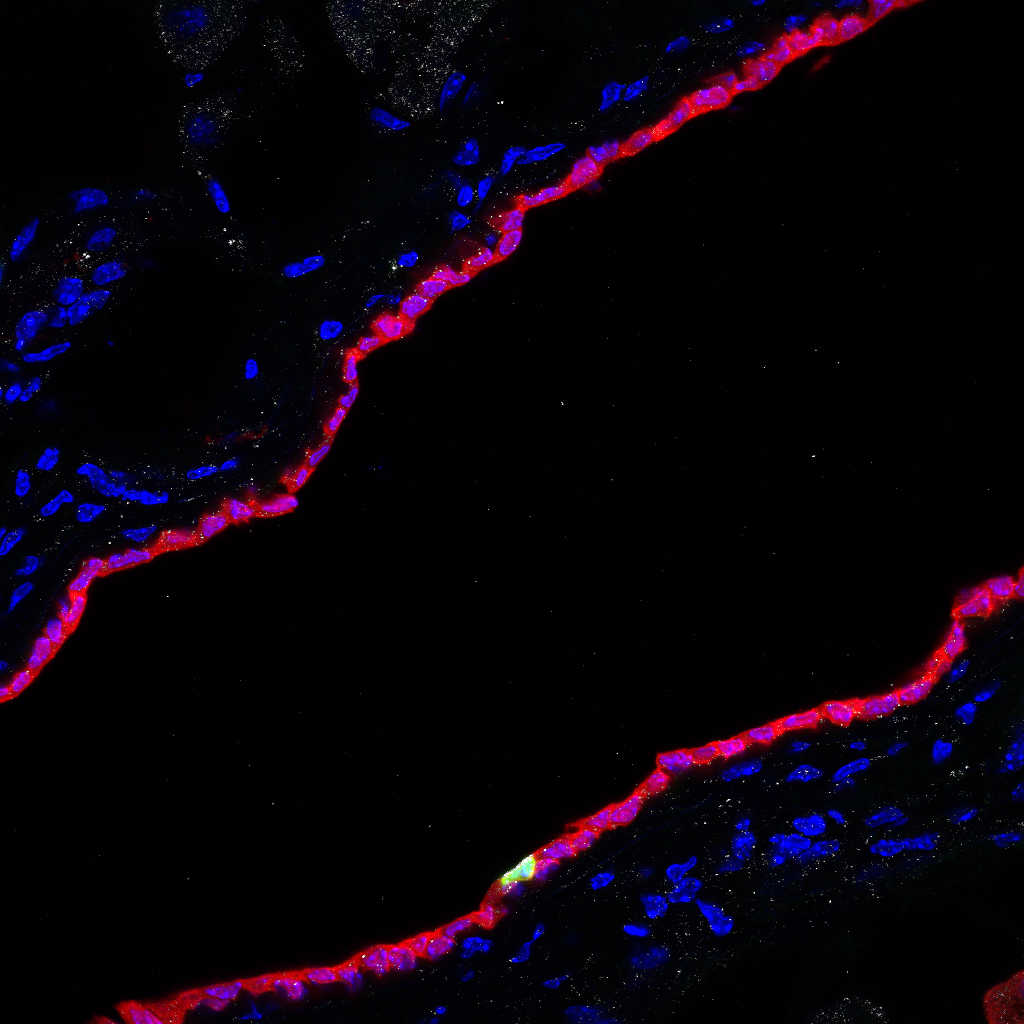

Supplement: Supplementary file 7 — Source data Fig. 5 [file 44318_2025_434_MOESM7_ESM.zip › Figure 5/5F/5F_Ins_Merge.tif]

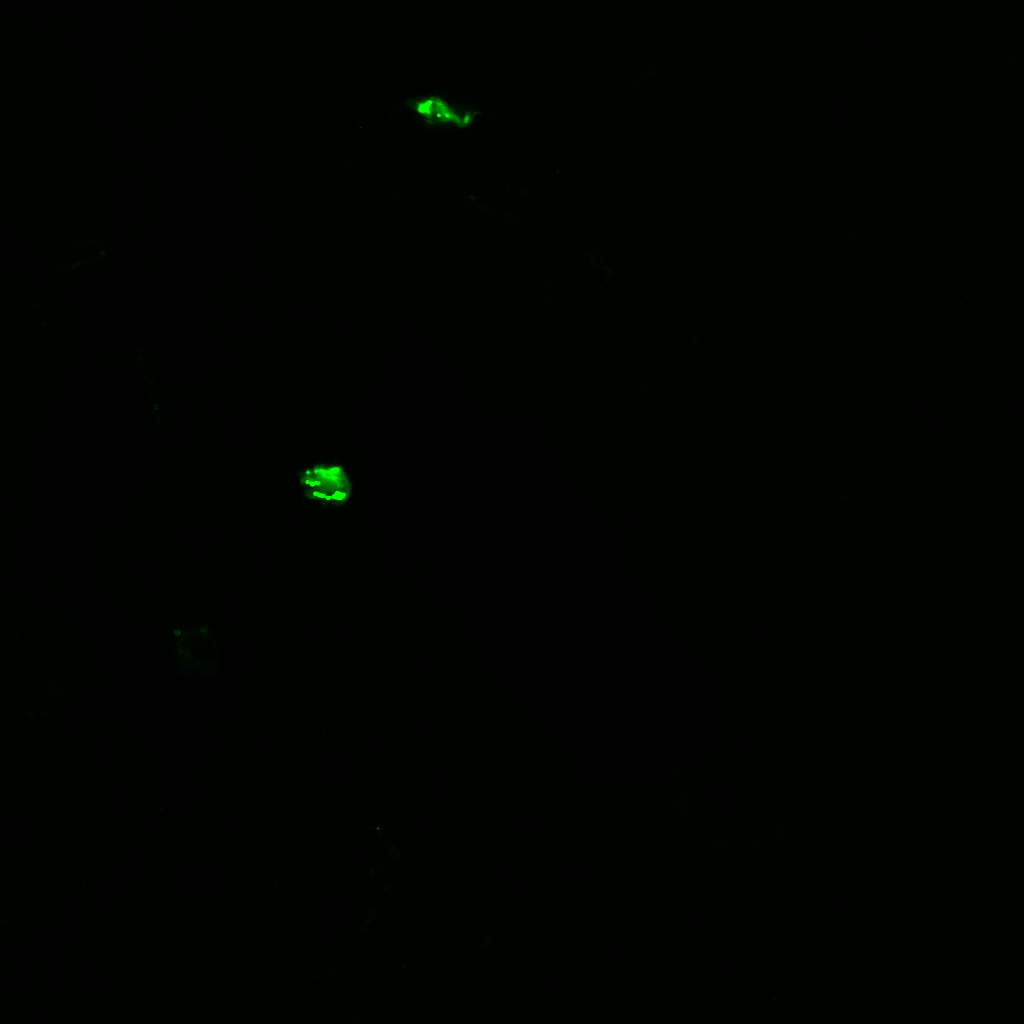

Supplement: Supplementary file 7 — Source data Fig. 5 [file 44318_2025_434_MOESM7_ESM.zip › Figure 5/5H/5H_CK19_Merge (green).tif]

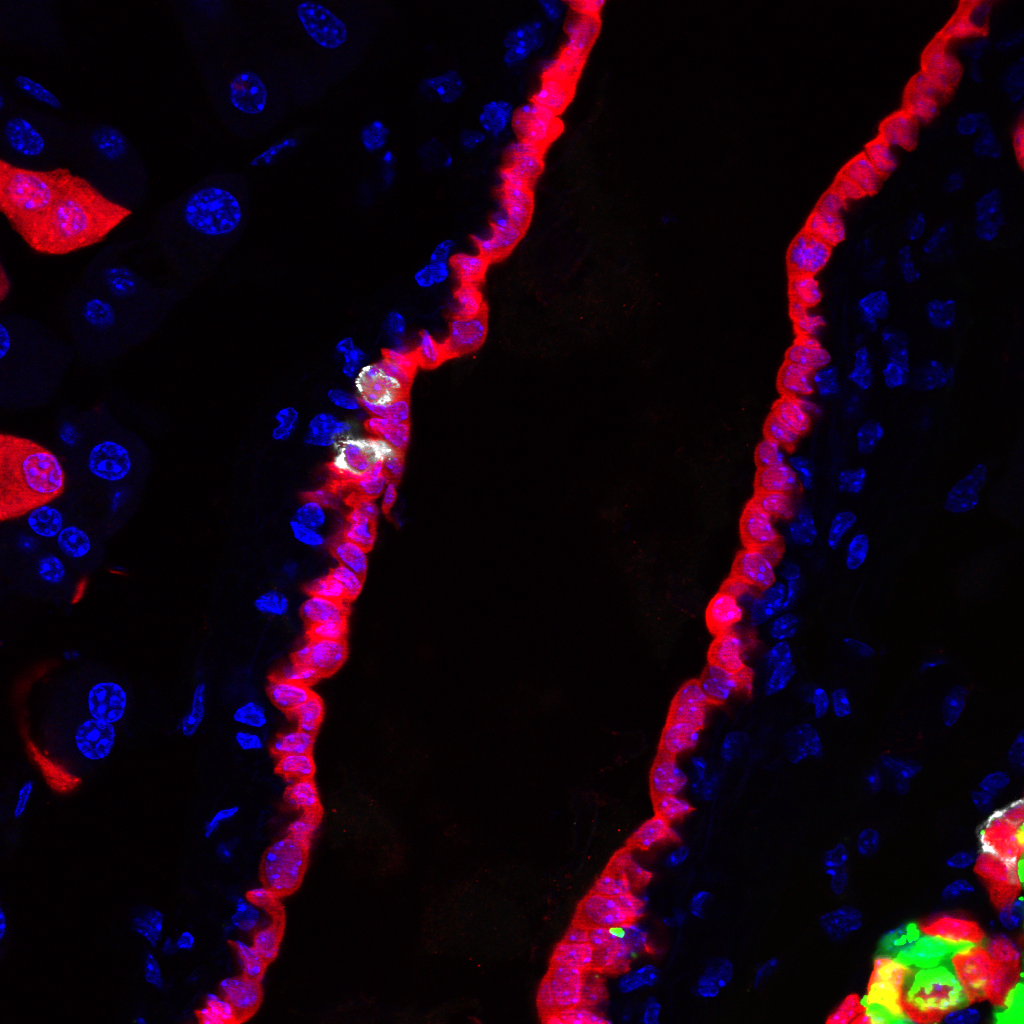

Supplement: Supplementary file 7 — Source data Fig. 5 [file 44318_2025_434_MOESM7_ESM.zip › Figure 5/5H/5H_Sst_Merge.tif]

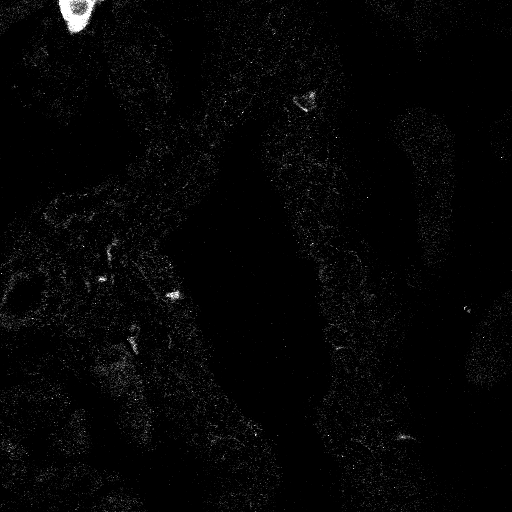

Supplement: Supplementary file 7 — Source data Fig. 5 [file 44318_2025_434_MOESM7_ESM.zip › Figure 5/5H/5H_Ins_Merge (gray).tif]

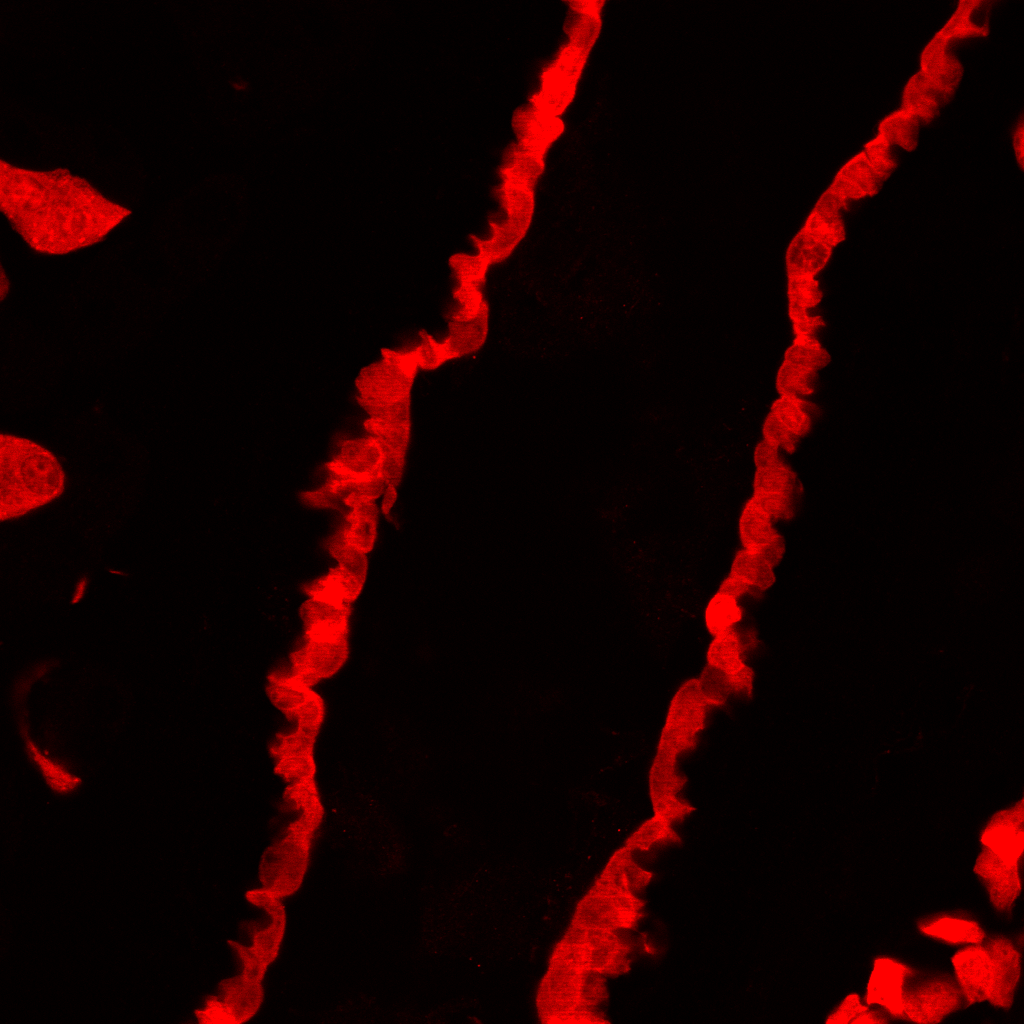

Supplement: Supplementary file 7 — Source data Fig. 5 [file 44318_2025_434_MOESM7_ESM.zip › Figure 5/5H/5H_Sst_Merge (red).tif]

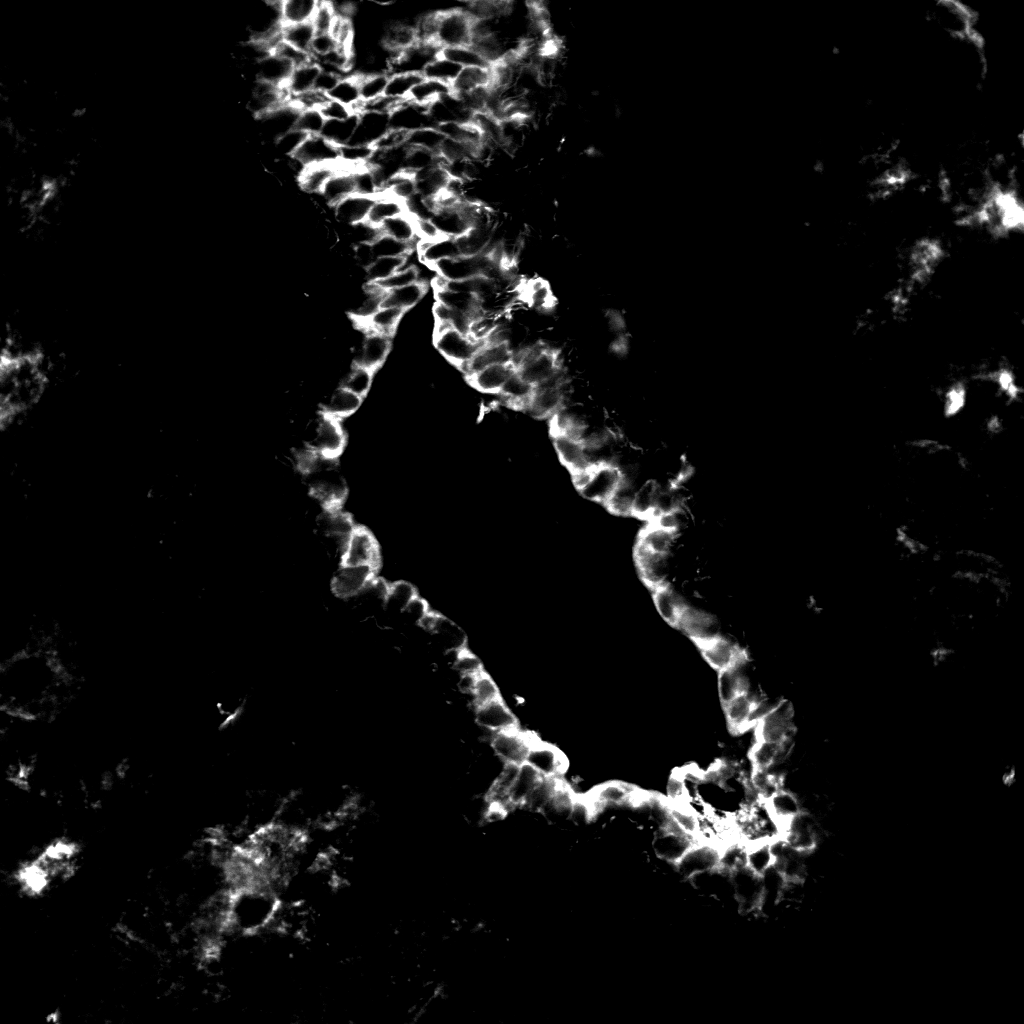

Supplement: Supplementary file 7 — Source data Fig. 5 [file 44318_2025_434_MOESM7_ESM.zip › Figure 5/5H/5H_CK19_Merge (gray).tif]

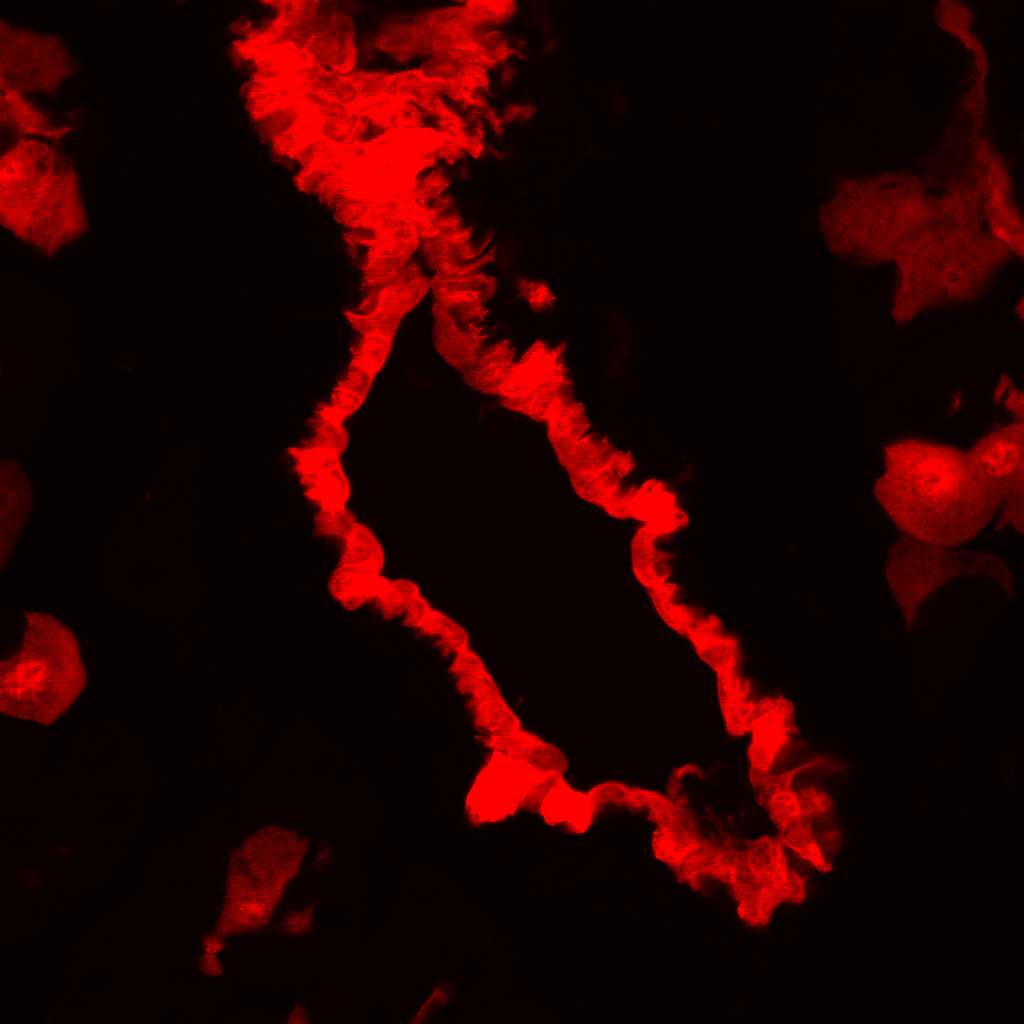

Supplement: Supplementary file 7 — Source data Fig. 5 [file 44318_2025_434_MOESM7_ESM.zip › Figure 5/5H/5H_CK19_Merge (red).tif]

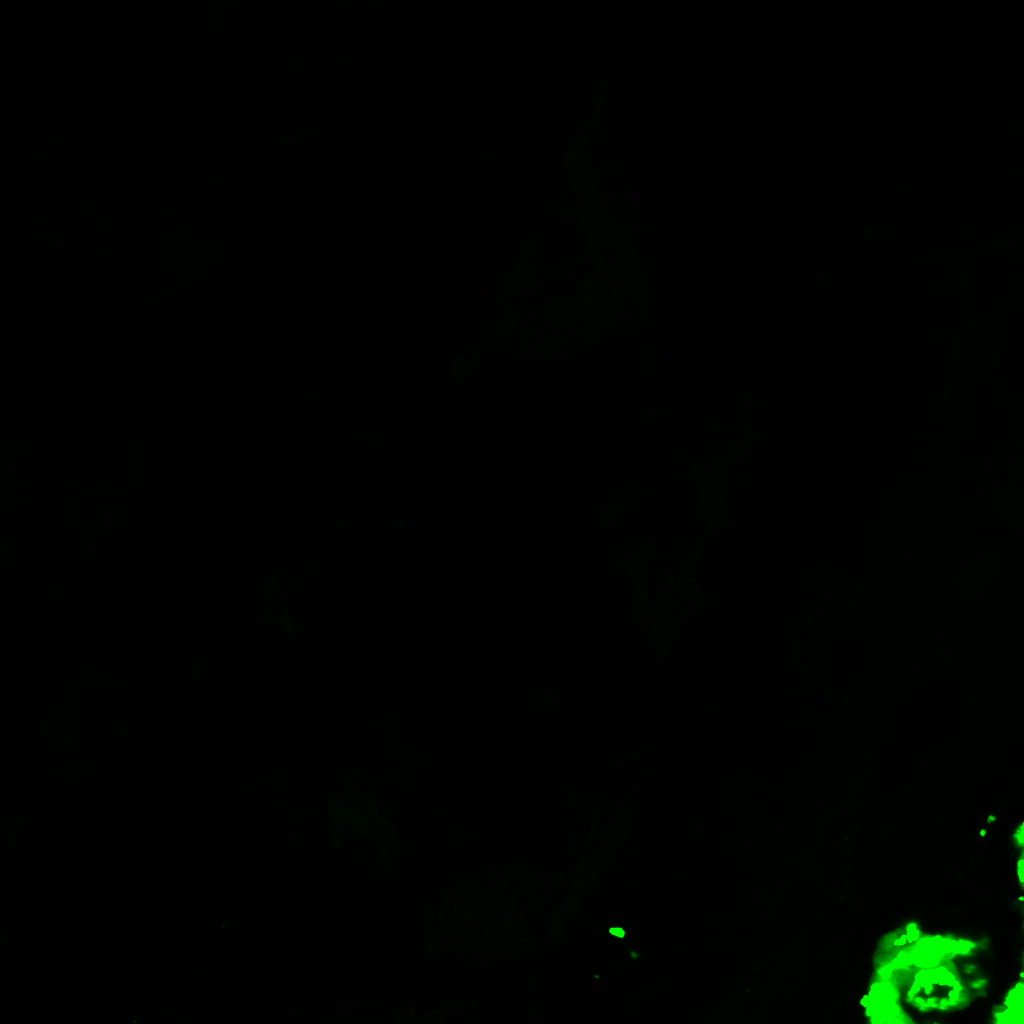

Supplement: Supplementary file 7 — Source data Fig. 5 [file 44318_2025_434_MOESM7_ESM.zip › Figure 5/5H/5H_Sst_Merge (green).tif]

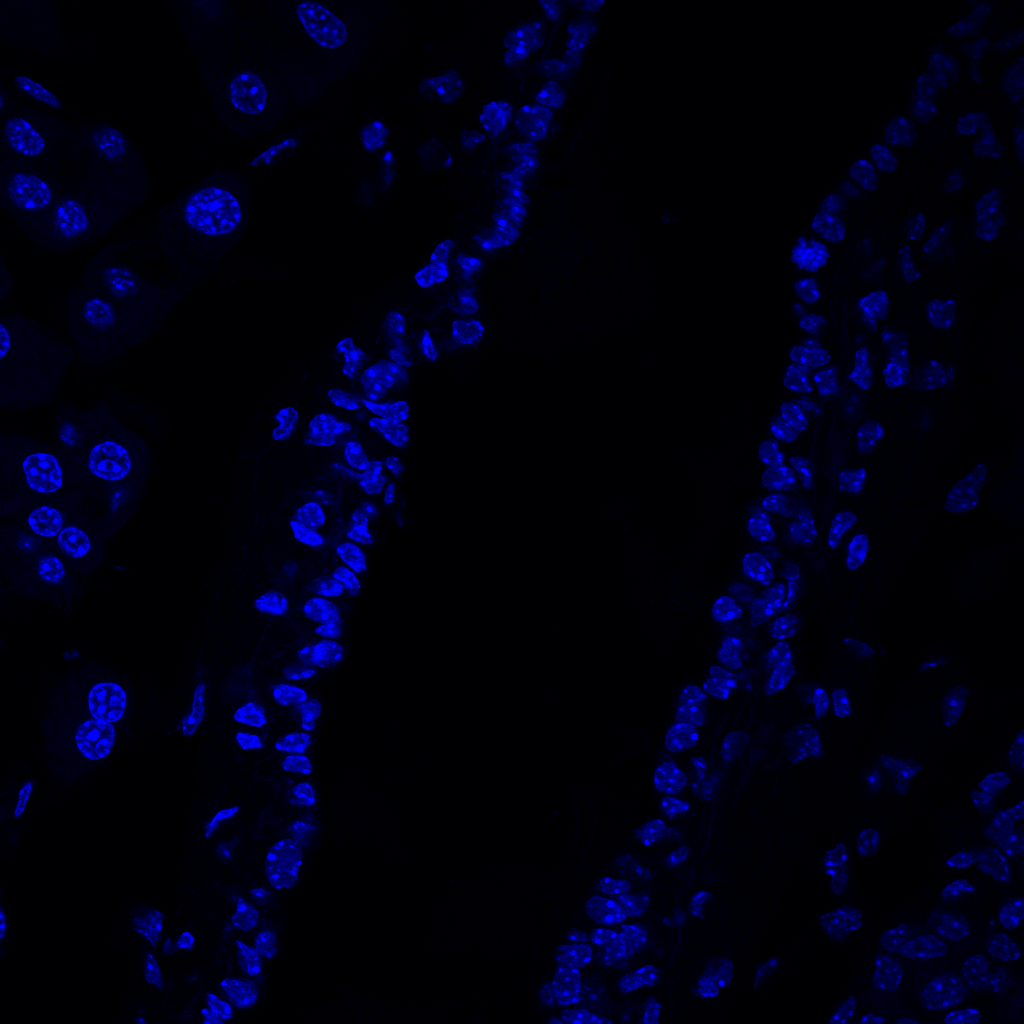

Supplement: Supplementary file 7 — Source data Fig. 5 [file 44318_2025_434_MOESM7_ESM.zip › Figure 5/5H/5H_Sst_Merge (blue).tif]

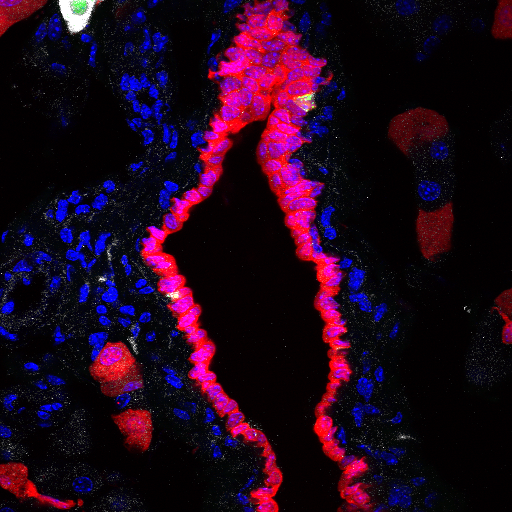

Supplement: Supplementary file 7 — Source data Fig. 5 [file 44318_2025_434_MOESM7_ESM.zip › Figure 5/5H/5H_Ins_Merge.tif]

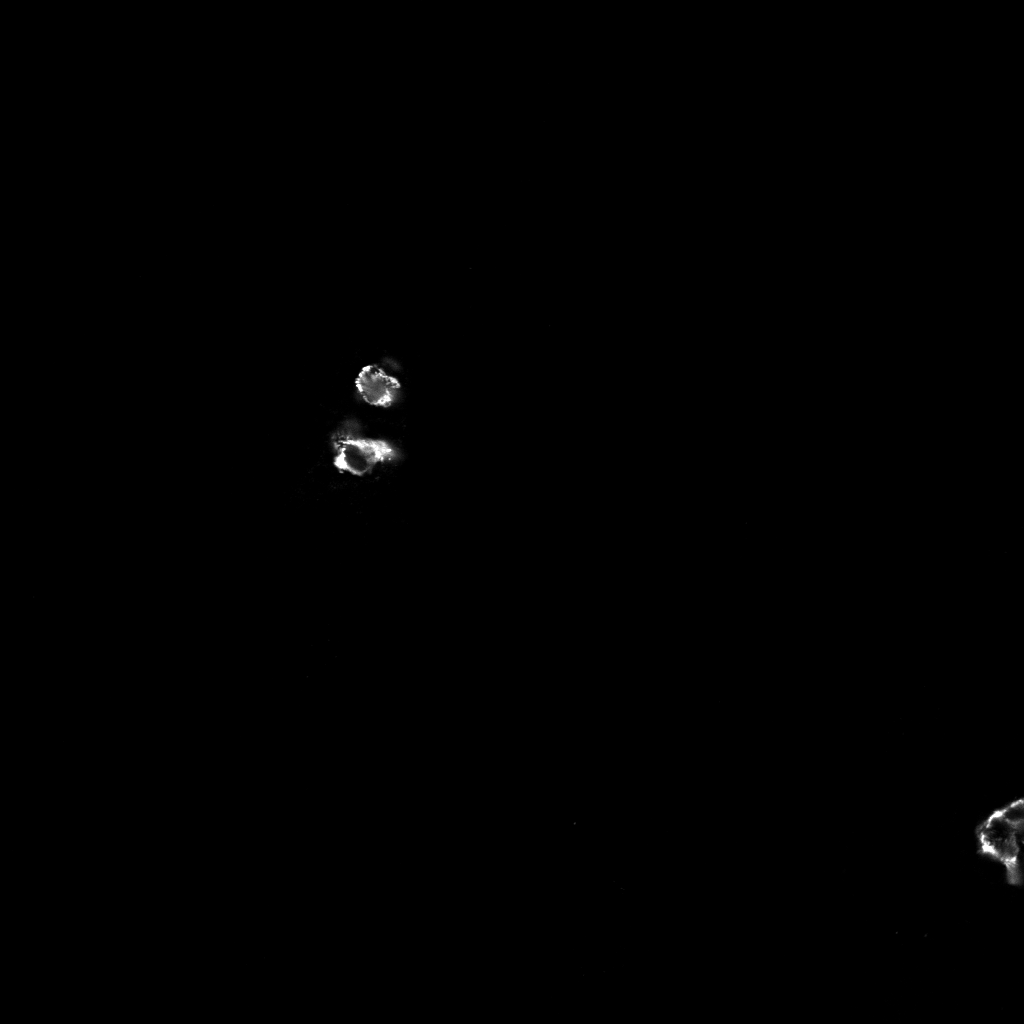

Supplement: Supplementary file 7 — Source data Fig. 5 [file 44318_2025_434_MOESM7_ESM.zip › Figure 5/5H/5H_Sst_Merge (gray).tif]

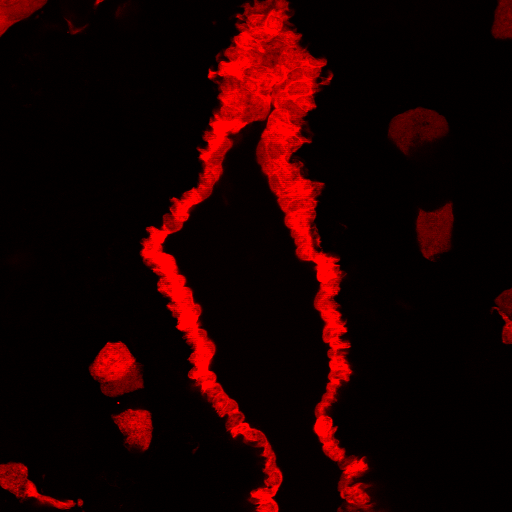

Supplement: Supplementary file 7 — Source data Fig. 5 [file 44318_2025_434_MOESM7_ESM.zip › Figure 5/5H/5H_Ins_Merge (red).tif]

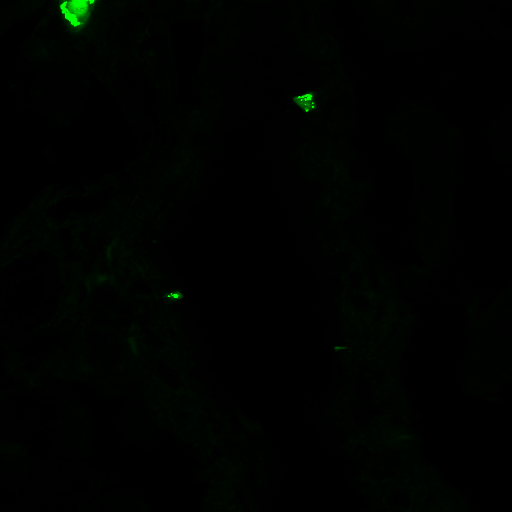

Supplement: Supplementary file 7 — Source data Fig. 5 [file 44318_2025_434_MOESM7_ESM.zip › Figure 5/5H/5H_Ins_Merge (green).tif]

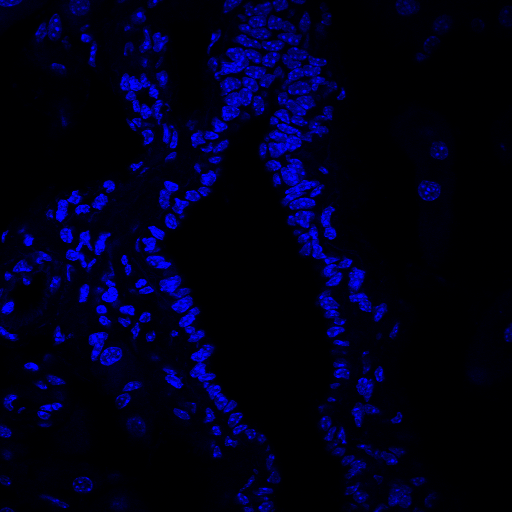

Supplement: Supplementary file 7 — Source data Fig. 5 [file 44318_2025_434_MOESM7_ESM.zip › Figure 5/5H/5H_Ins_Merge (blue).tif]
