# Supplementary figures and images for: Ductal or Ngn3+ cells do not contribute to adult pancreatic islet beta-cell neogenesis in homeostasis (part 5 of 5)
Source: EMBO J. 2025 Apr 9;44(10):2856–81. doi: 10.1038/s44318-025-00434-z (PMC12084597; doi:10.1038/s44318-025-00434-z)

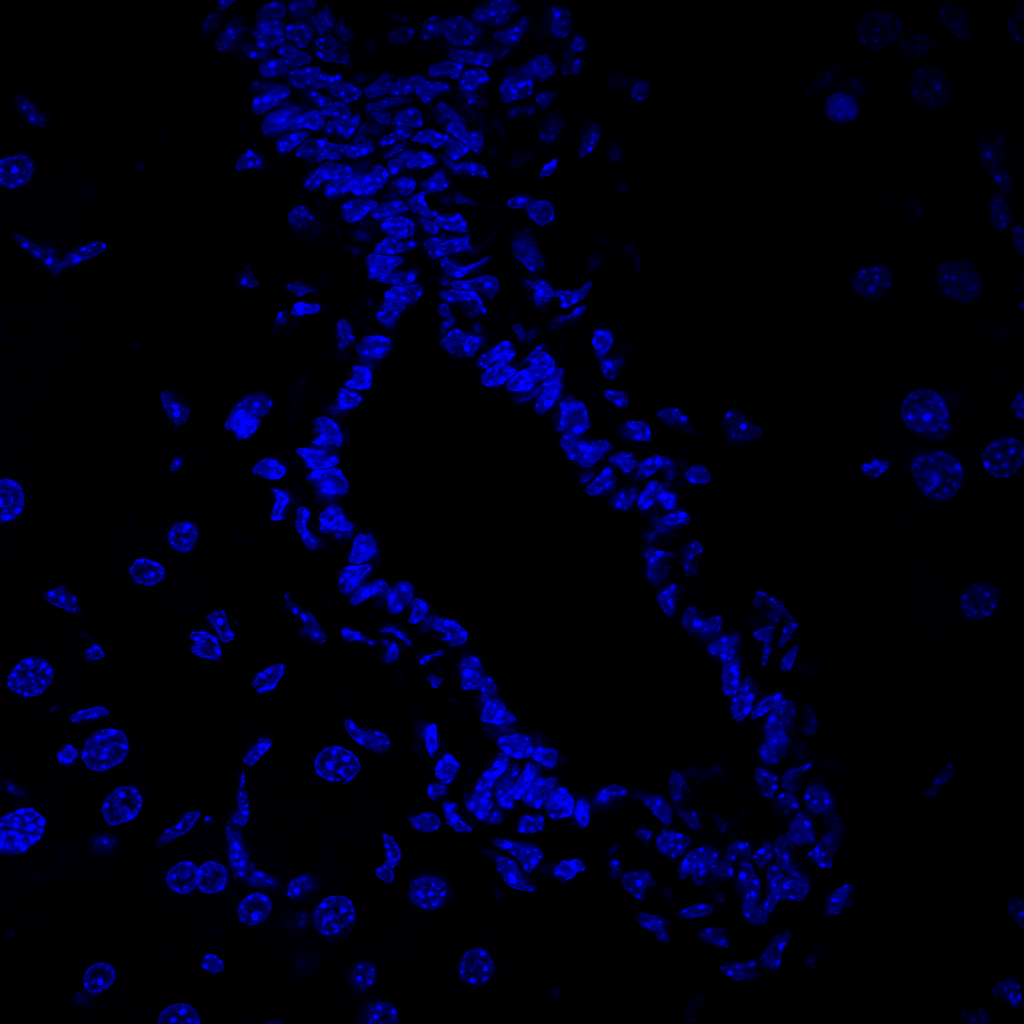

Supplement: Supplementary file 7 — Source data Fig. 5 [file 44318_2025_434_MOESM7_ESM.zip › Figure 5/5H/5H_CK19_Merge (blue).tif]

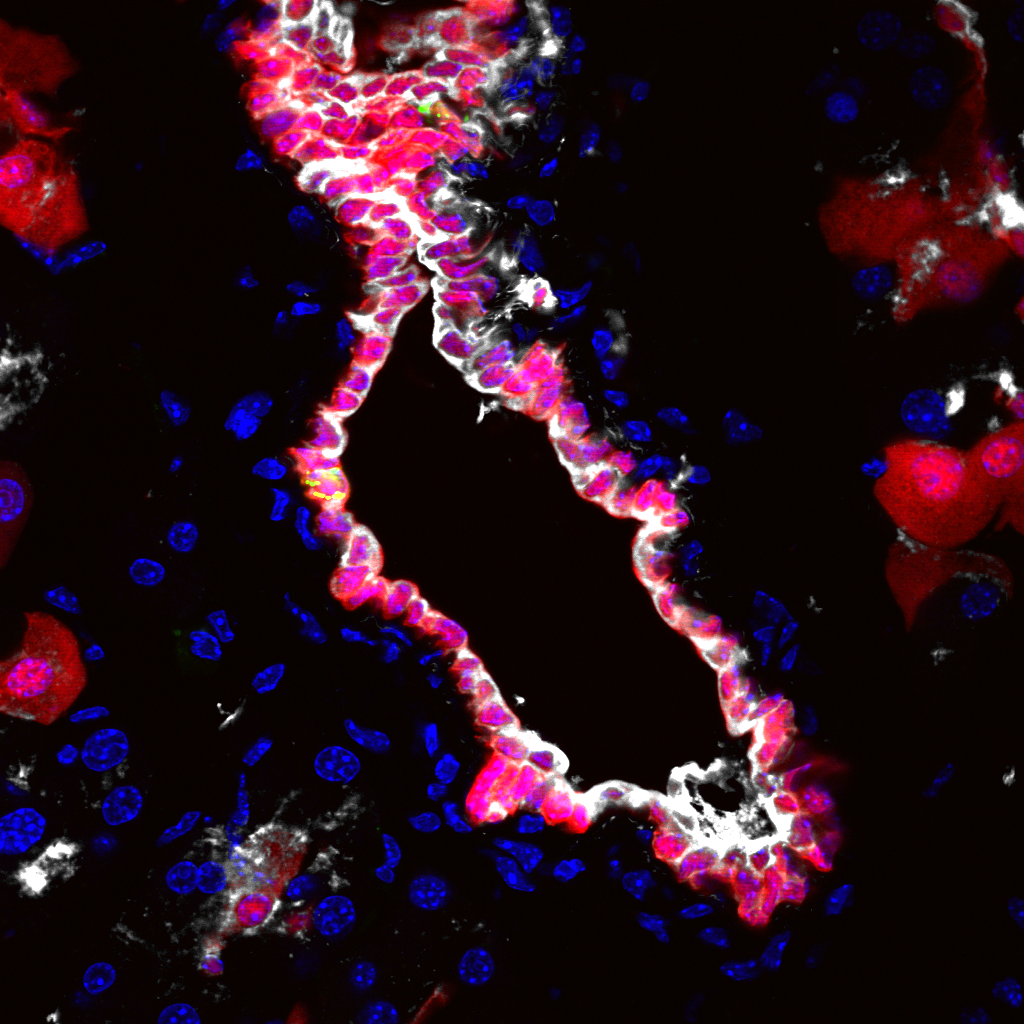

Supplement: Supplementary file 7 — Source data Fig. 5 [file 44318_2025_434_MOESM7_ESM.zip › Figure 5/5H/5H_CK19_Merge.tif]

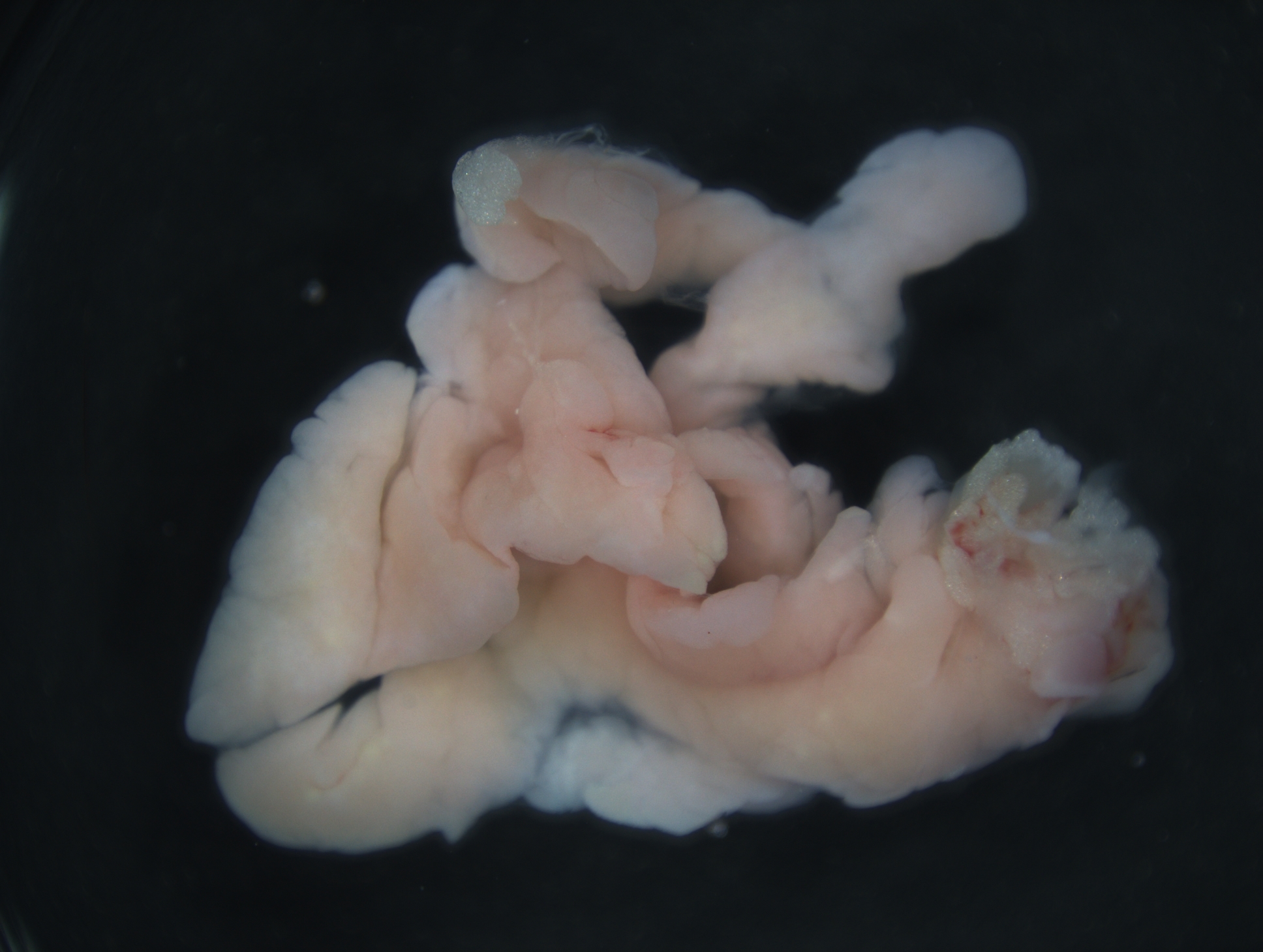

Supplement: Supplementary file 7 — Source data Fig. 5 [file 44318_2025_434_MOESM7_ESM.zip › Figure 5/5C/5C_12w_BF.tif]

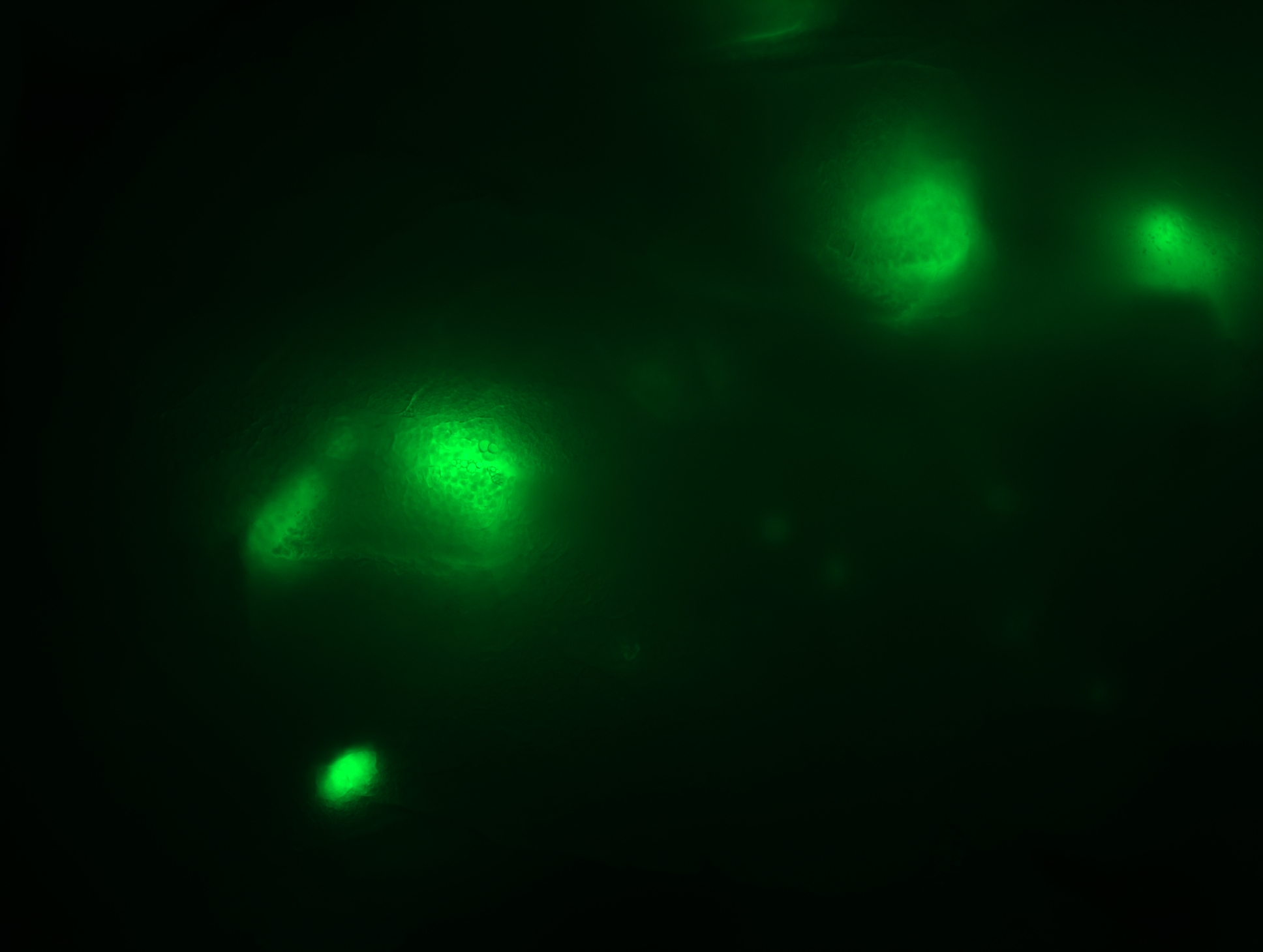

Supplement: Supplementary file 7 — Source data Fig. 5 [file 44318_2025_434_MOESM7_ESM.zip › Figure 5/5C/5C_2w_zsGreen_mag.tif]

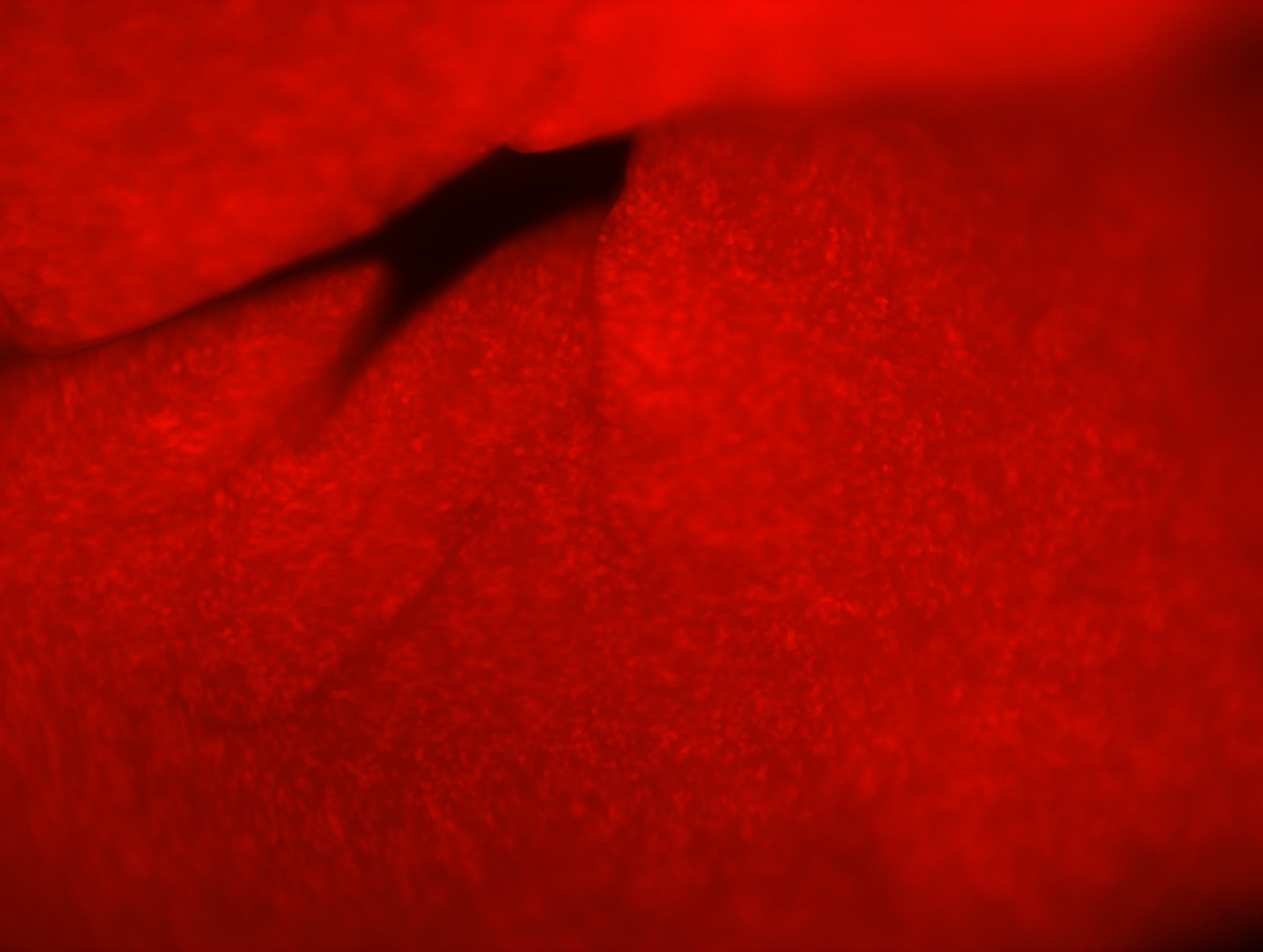

Supplement: Supplementary file 7 — Source data Fig. 5 [file 44318_2025_434_MOESM7_ESM.zip › Figure 5/5C/5C_12w_tdT_mag.tif]

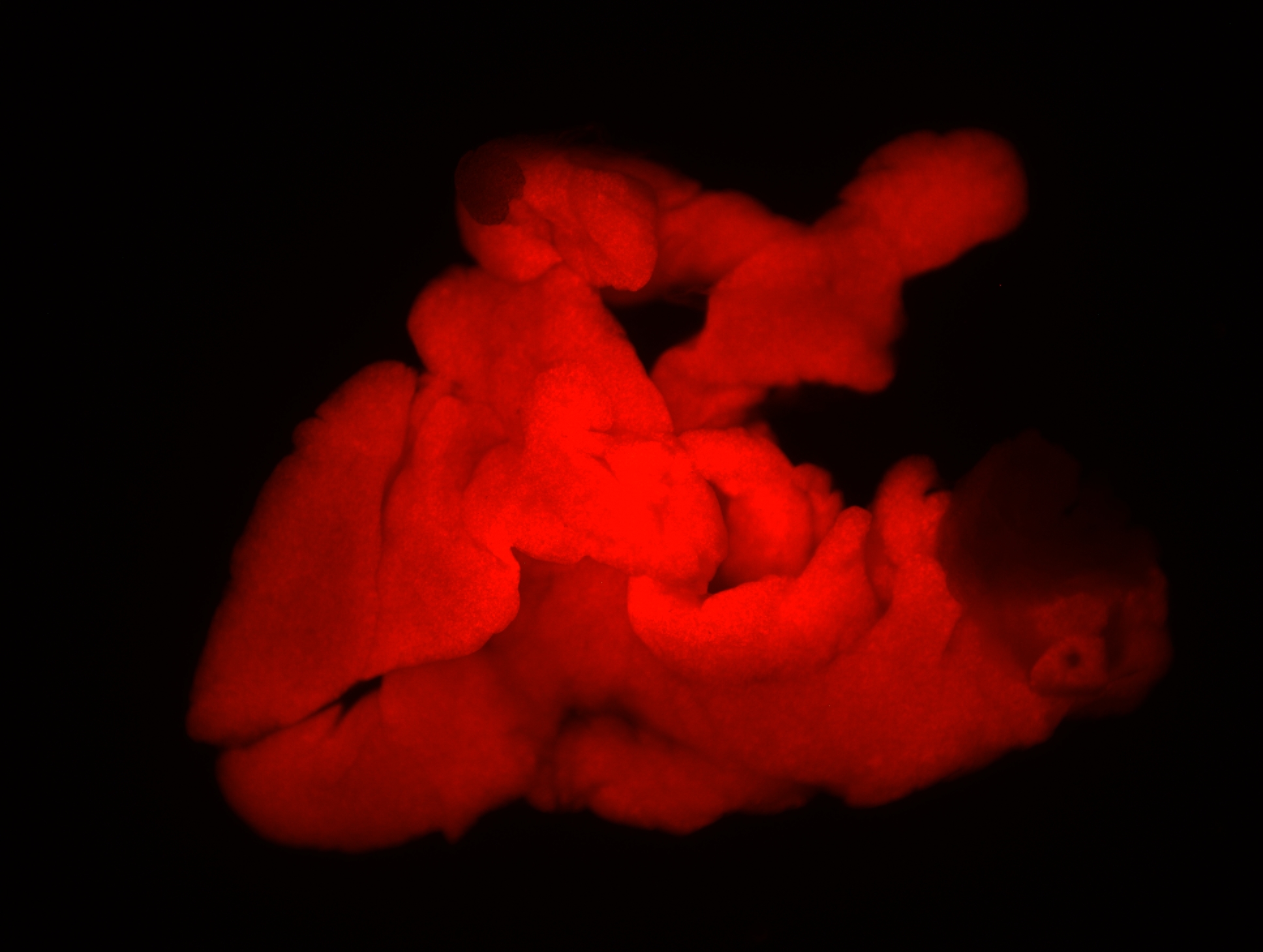

Supplement: Supplementary file 7 — Source data Fig. 5 [file 44318_2025_434_MOESM7_ESM.zip › Figure 5/5C/5C_12w_tdT.tif]

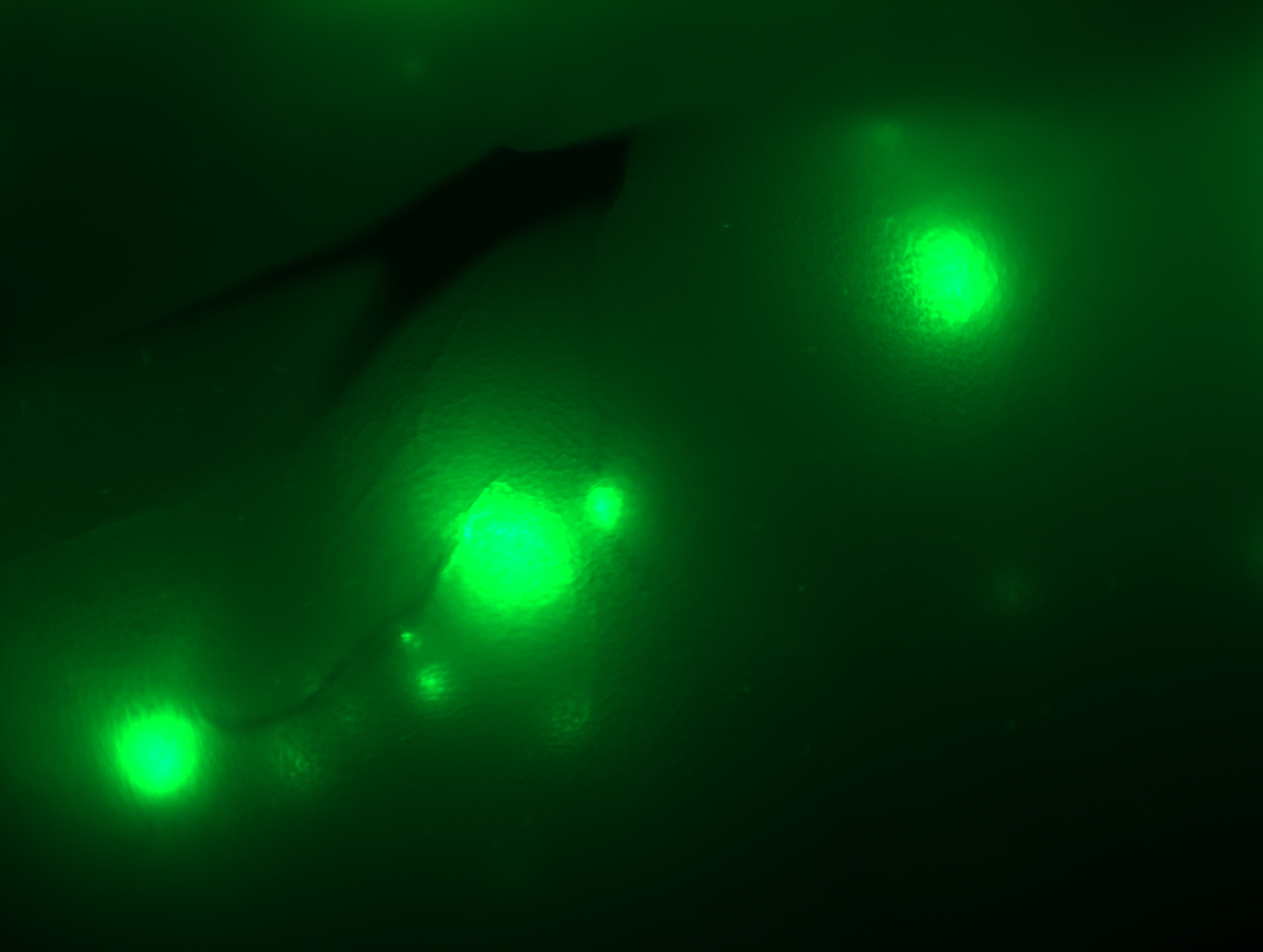

Supplement: Supplementary file 7 — Source data Fig. 5 [file 44318_2025_434_MOESM7_ESM.zip › Figure 5/5C/5C_12w_zsGreen_mag.tif]

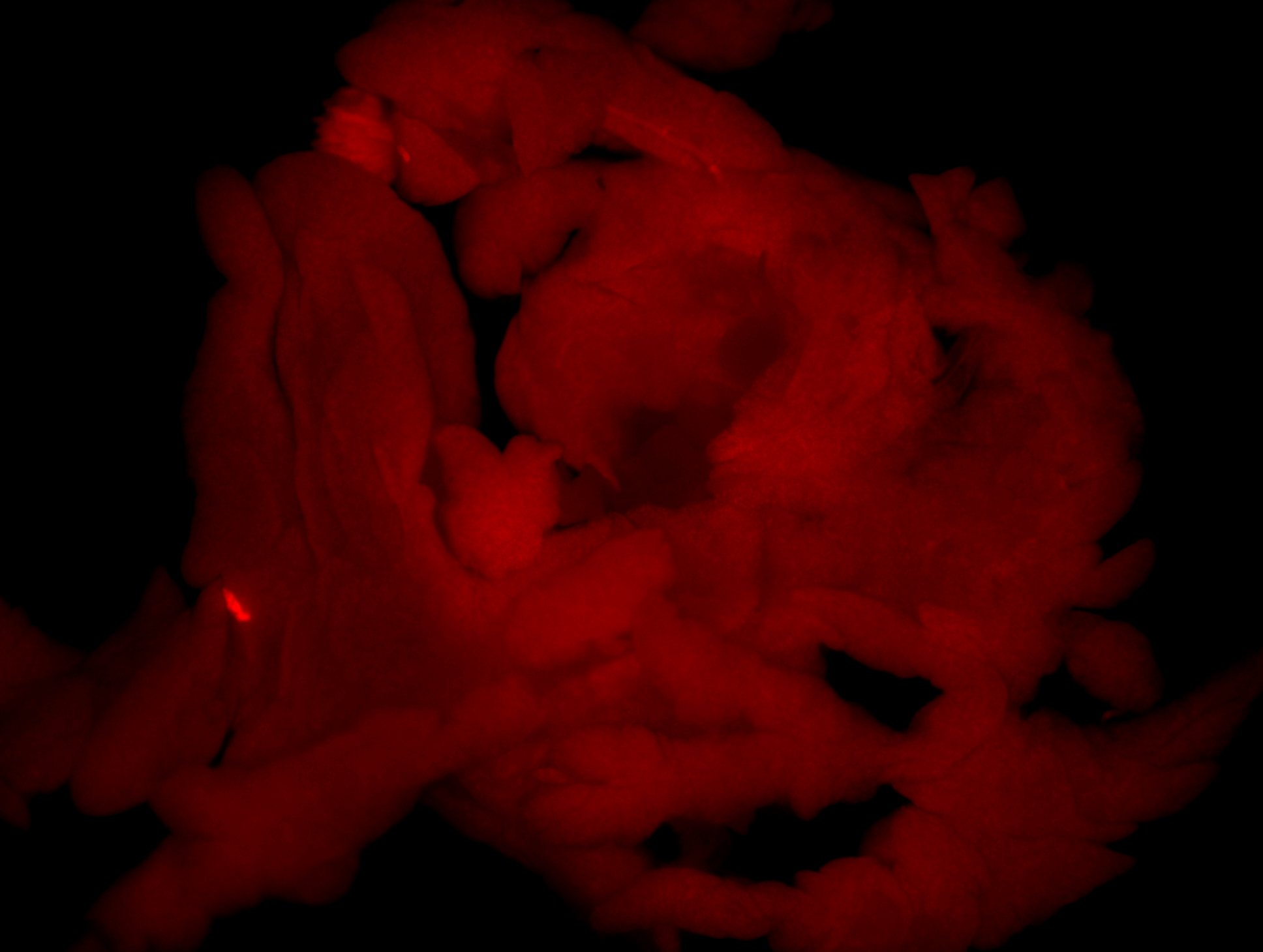

Supplement: Supplementary file 7 — Source data Fig. 5 [file 44318_2025_434_MOESM7_ESM.zip › Figure 5/5C/5C_2w_tdT.tif]

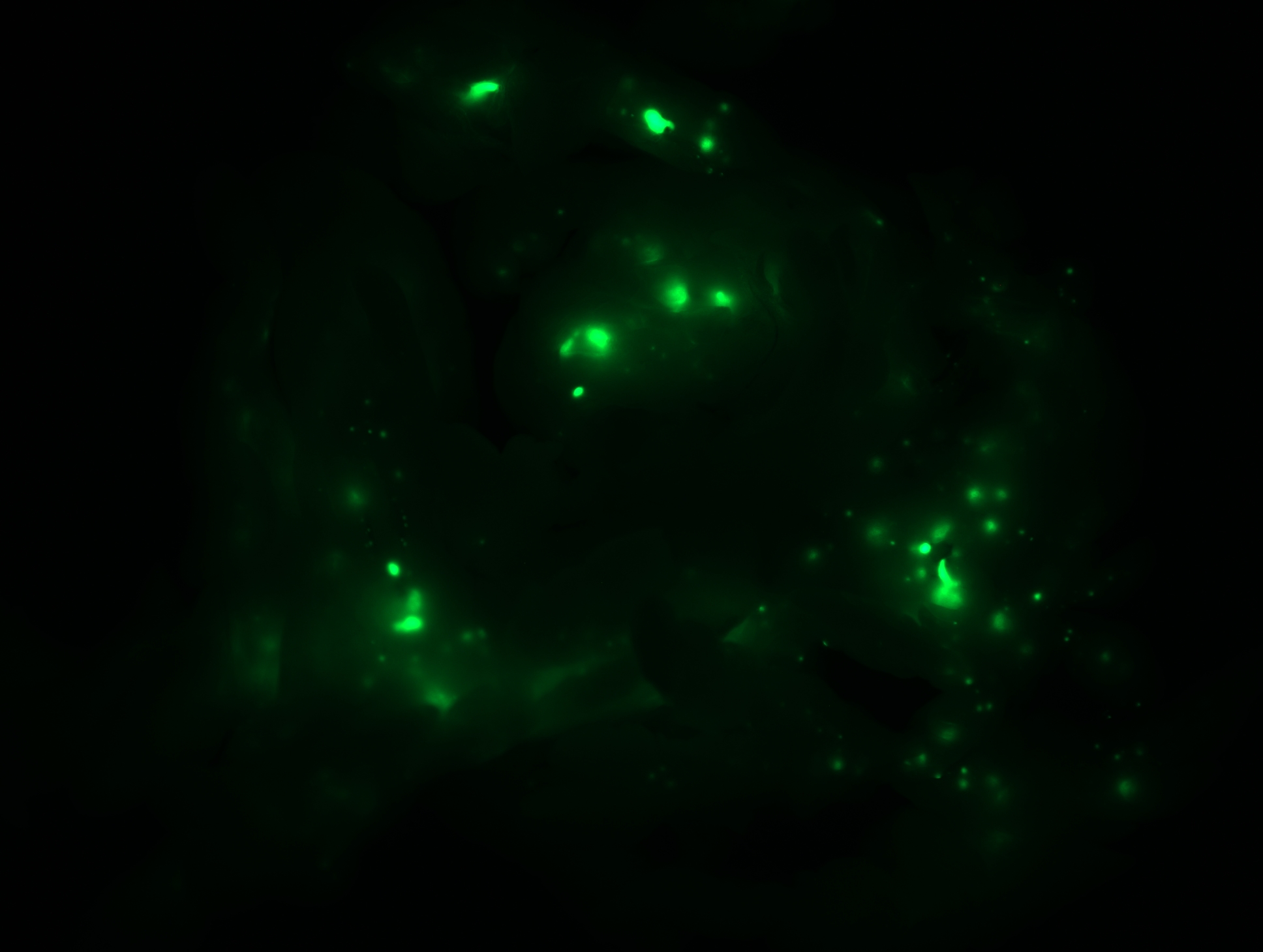

Supplement: Supplementary file 7 — Source data Fig. 5 [file 44318_2025_434_MOESM7_ESM.zip › Figure 5/5C/5C_2w_zsGreen.tif]

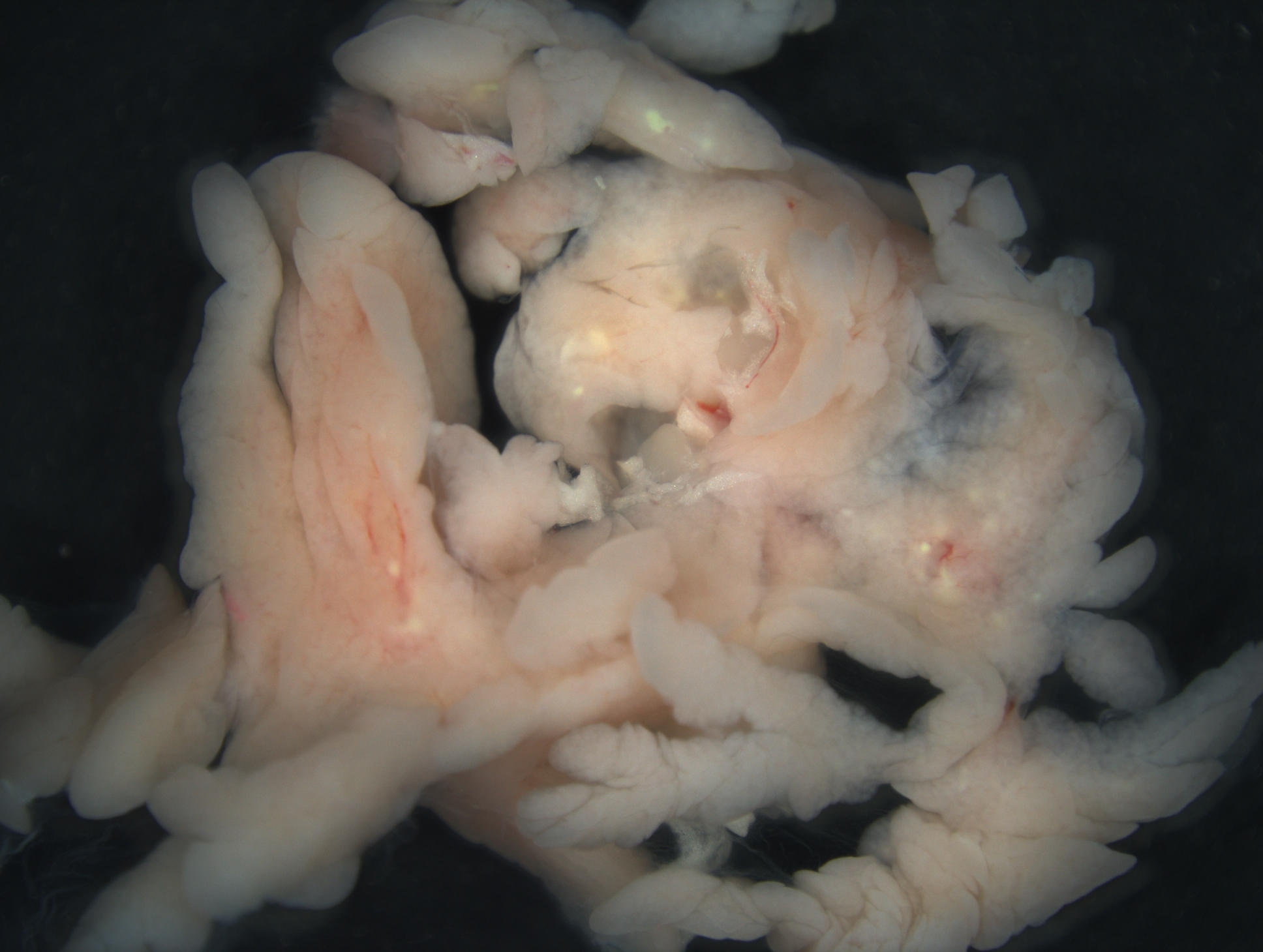

Supplement: Supplementary file 7 — Source data Fig. 5 [file 44318_2025_434_MOESM7_ESM.zip › Figure 5/5C/5C_2w_BF.tif]

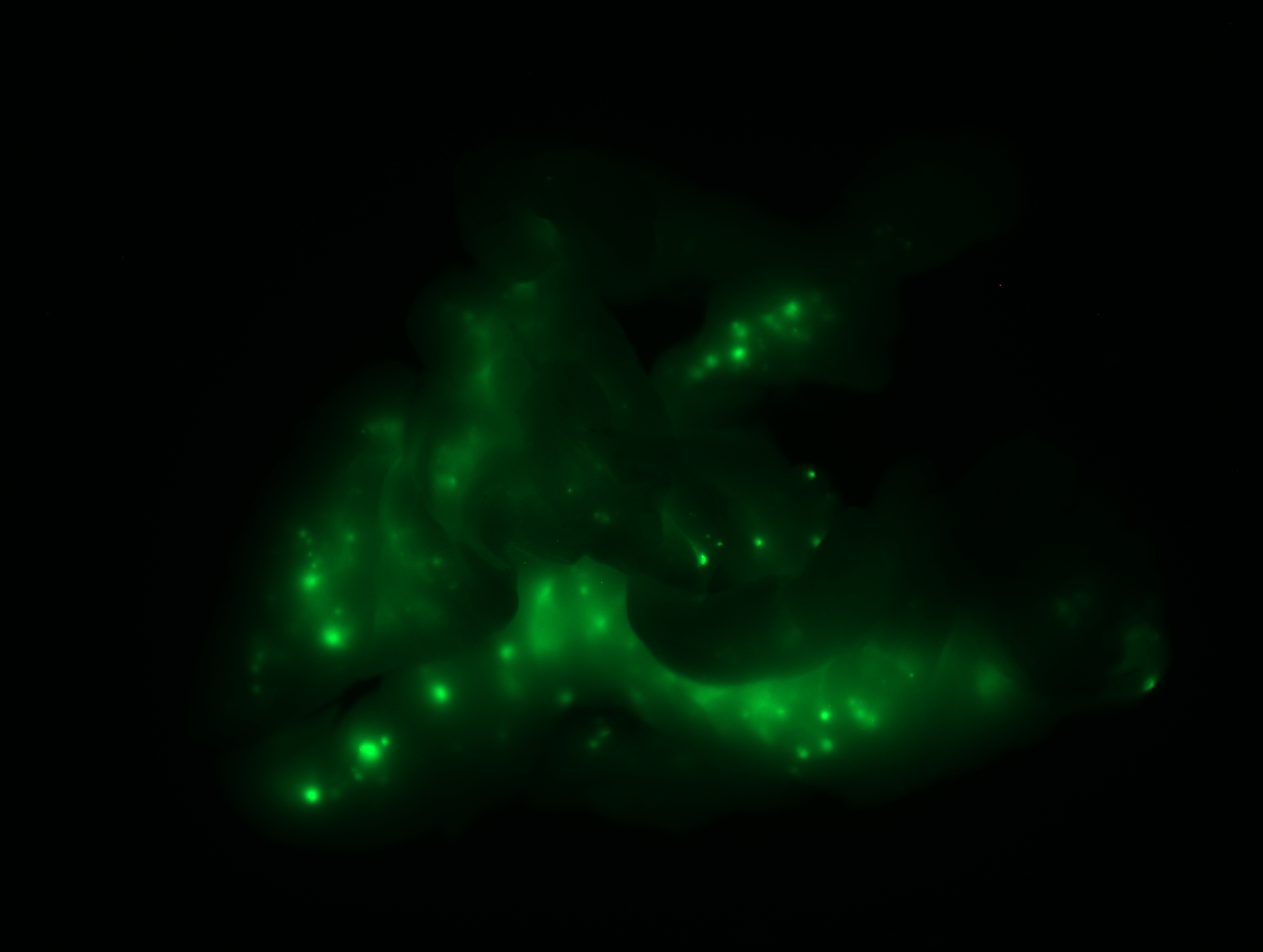

Supplement: Supplementary file 7 — Source data Fig. 5 [file 44318_2025_434_MOESM7_ESM.zip › Figure 5/5C/5C_12w_zsGreen.tif]

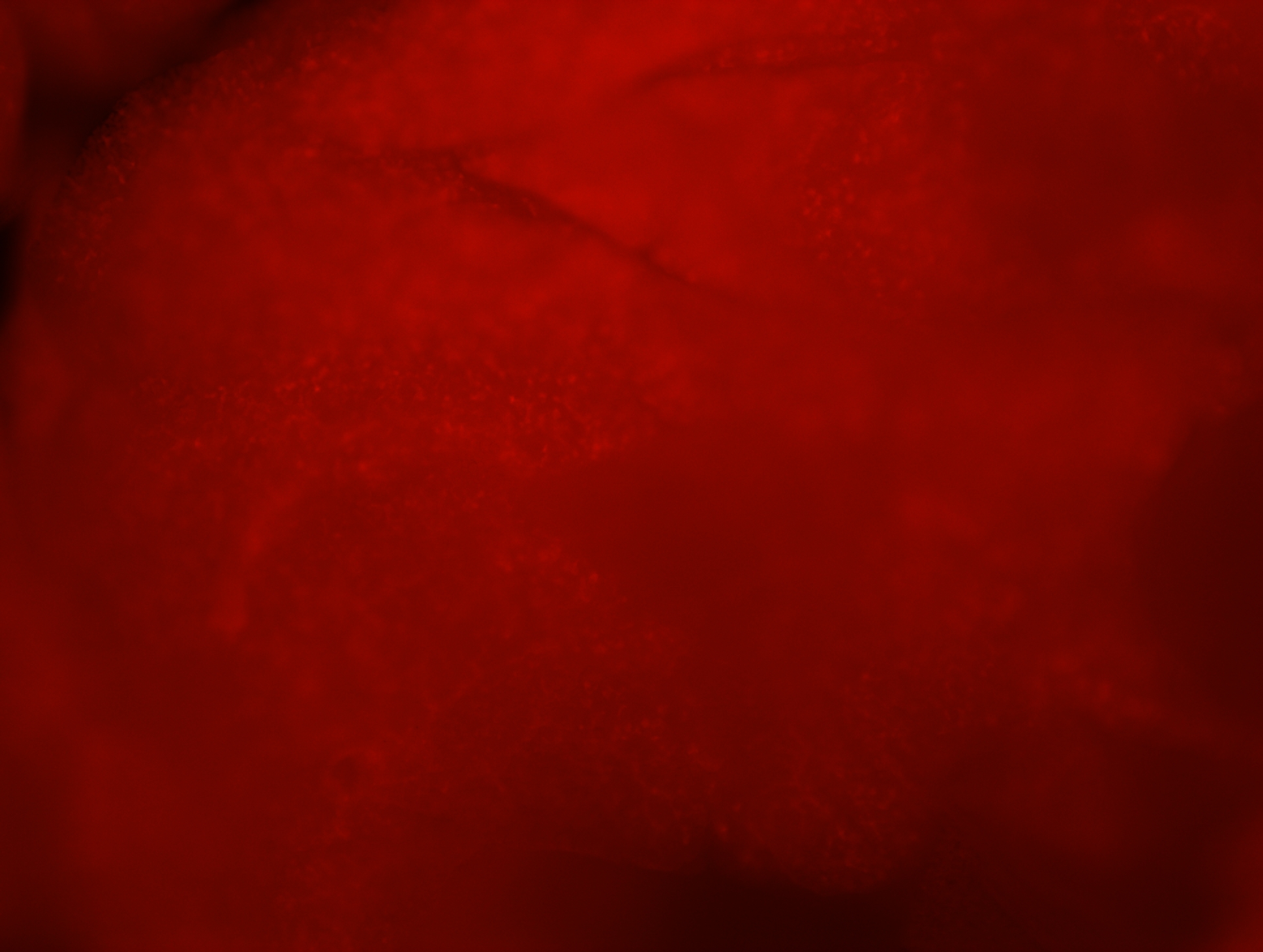

Supplement: Supplementary file 7 — Source data Fig. 5 [file 44318_2025_434_MOESM7_ESM.zip › Figure 5/5C/5C_2w_tdT_mag.tif]

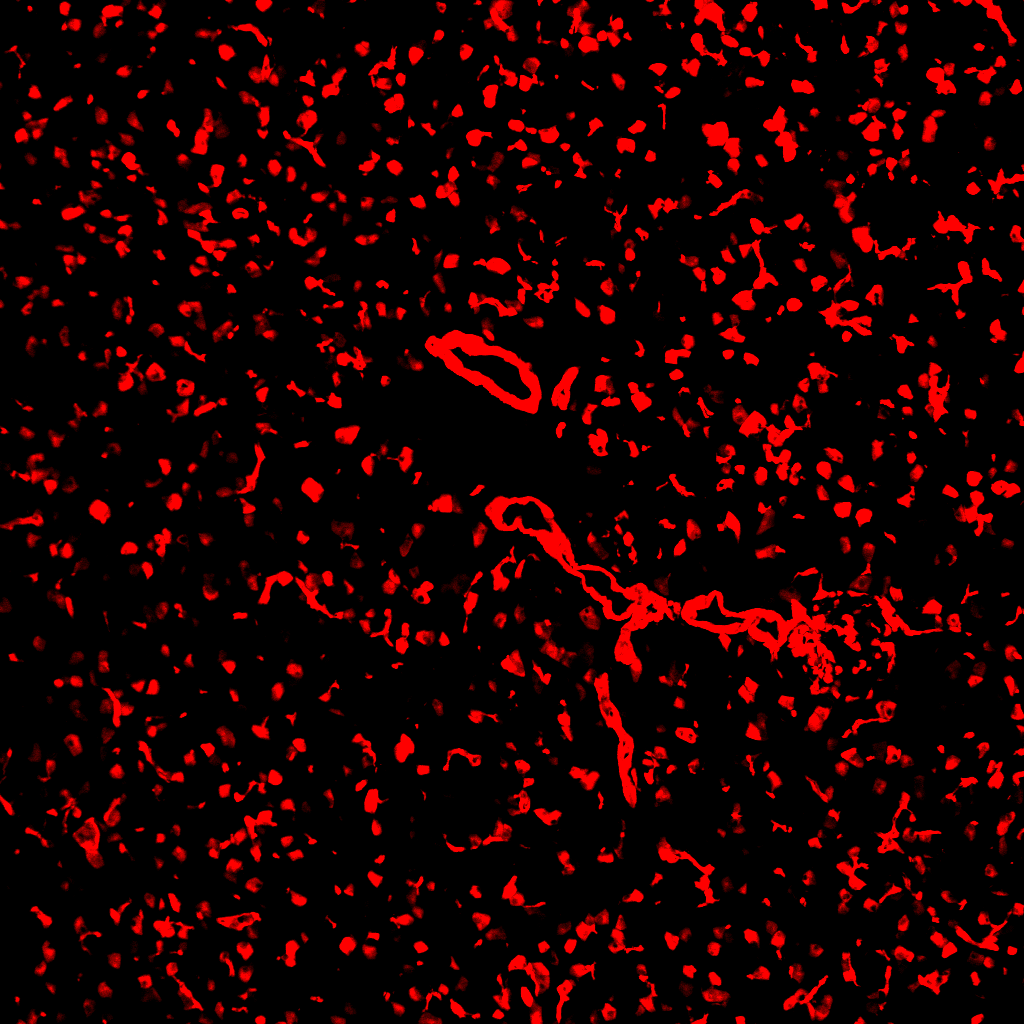

Supplement: Supplementary file 7 — Source data Fig. 5 [file 44318_2025_434_MOESM7_ESM.zip › Figure 5/5D/5D_Merge (red).tif]

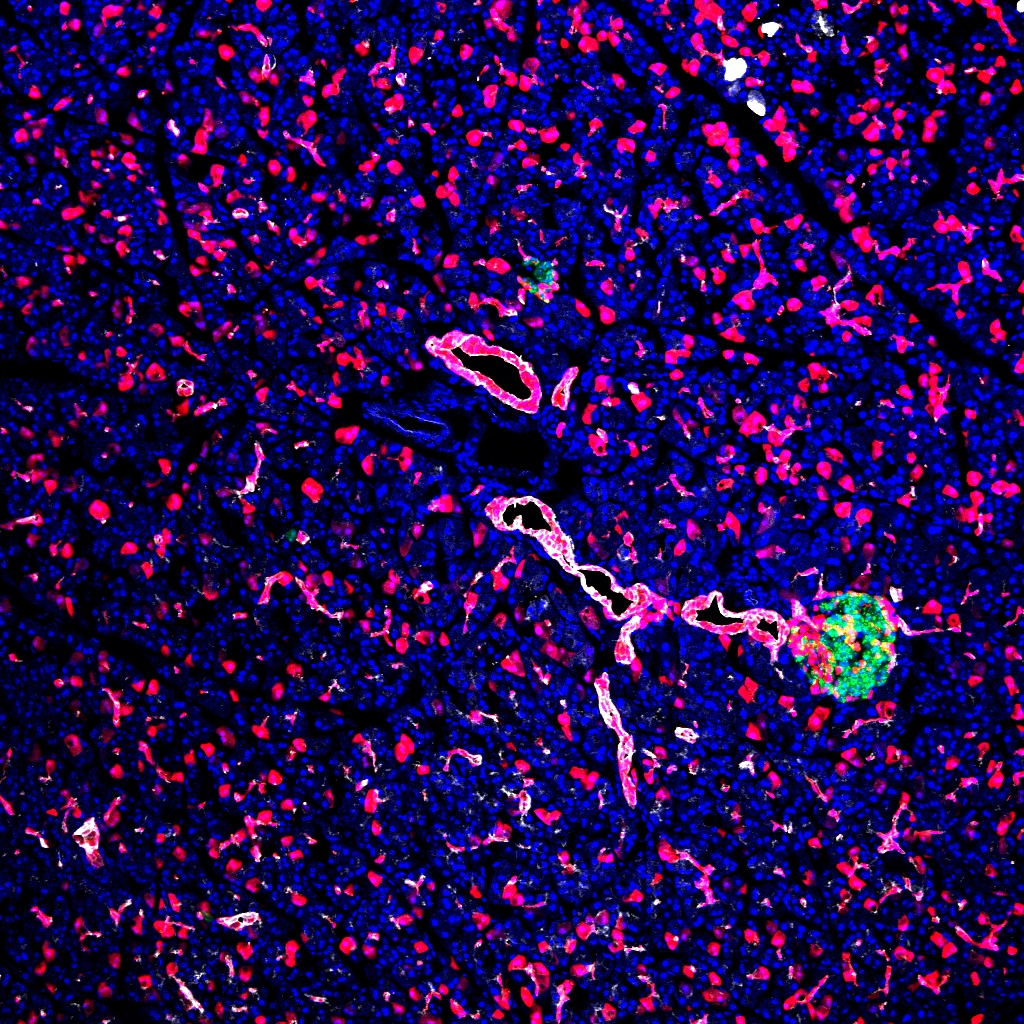

Supplement: Supplementary file 7 — Source data Fig. 5 [file 44318_2025_434_MOESM7_ESM.zip › Figure 5/5D/5D_Merge.tif]

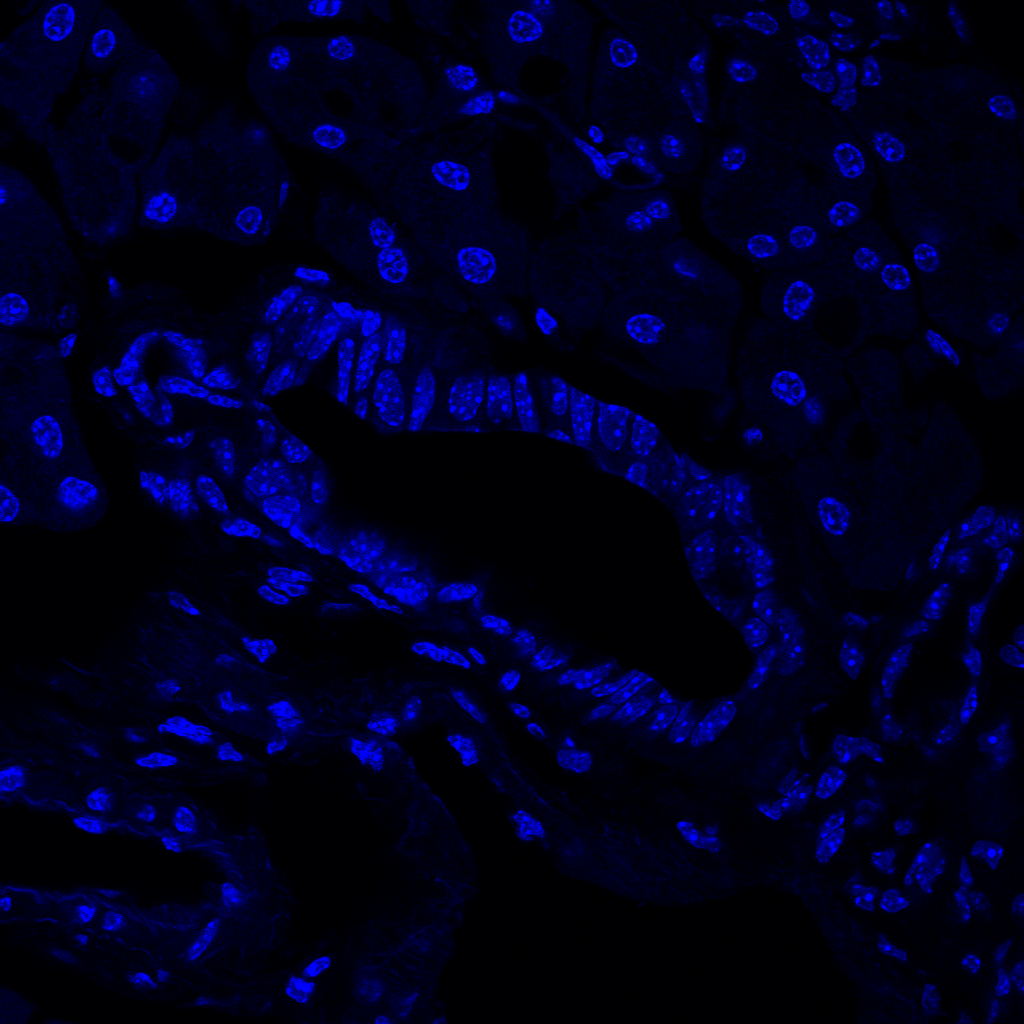

Supplement: Supplementary file 7 — Source data Fig. 5 [file 44318_2025_434_MOESM7_ESM.zip › Figure 5/5D/5D_Merge_Mag_1 (blue).tif]

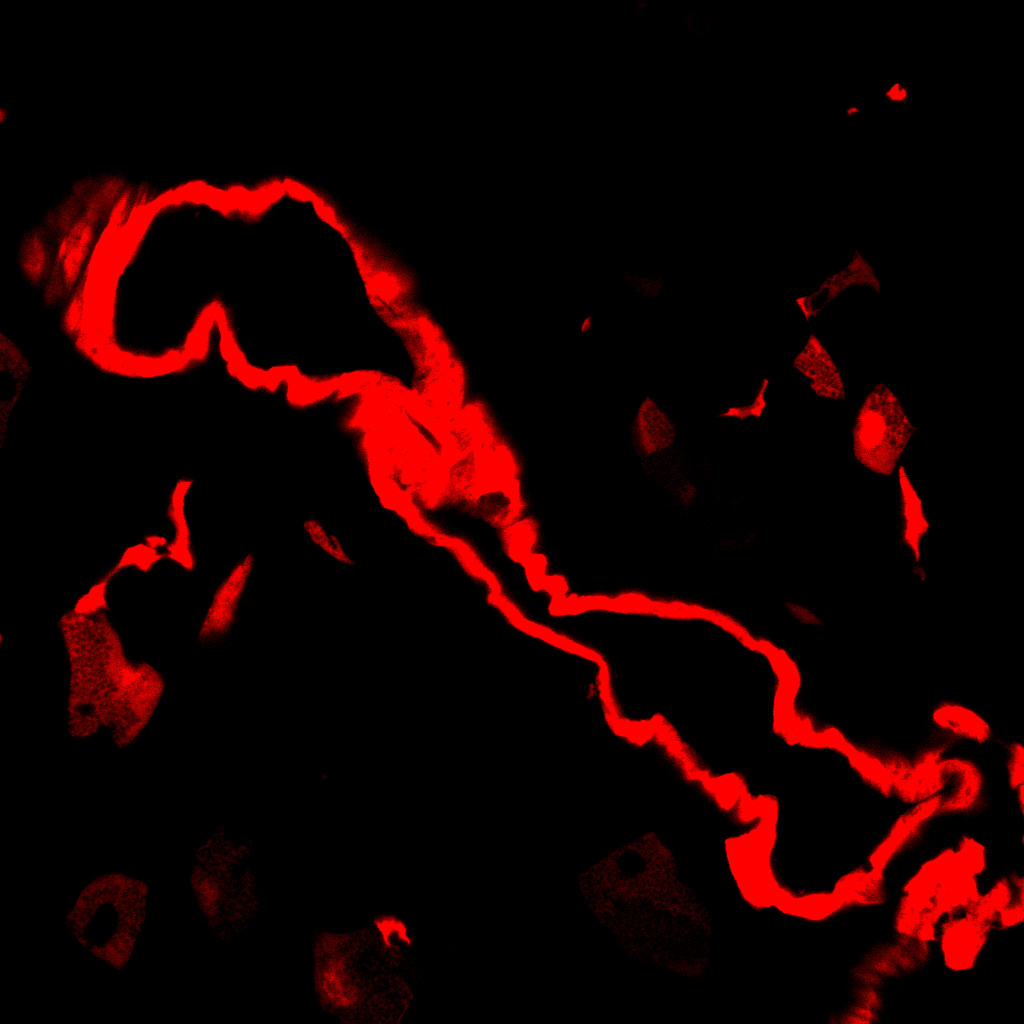

Supplement: Supplementary file 7 — Source data Fig. 5 [file 44318_2025_434_MOESM7_ESM.zip › Figure 5/5D/5D_Merge_Mag_2 (red).tif]

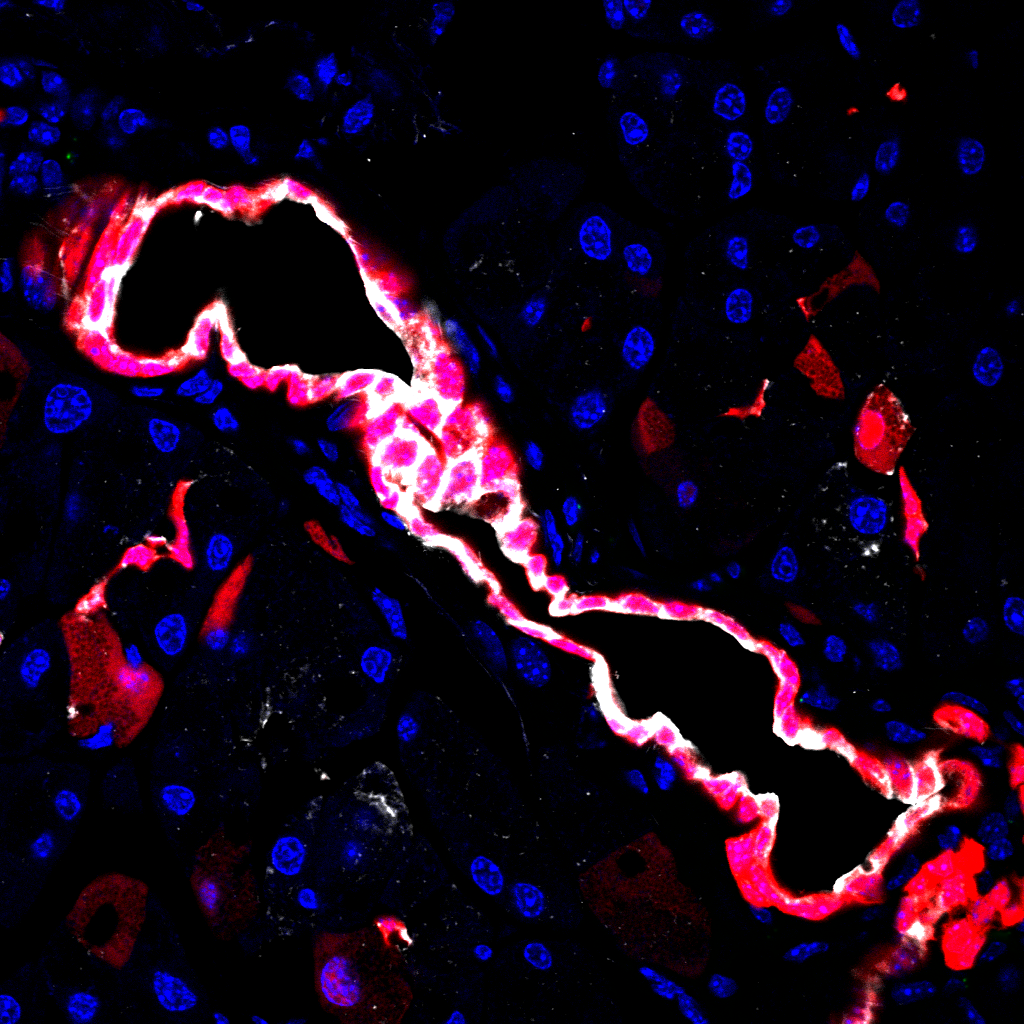

Supplement: Supplementary file 7 — Source data Fig. 5 [file 44318_2025_434_MOESM7_ESM.zip › Figure 5/5D/5D_Merge_Mag_2.tif]

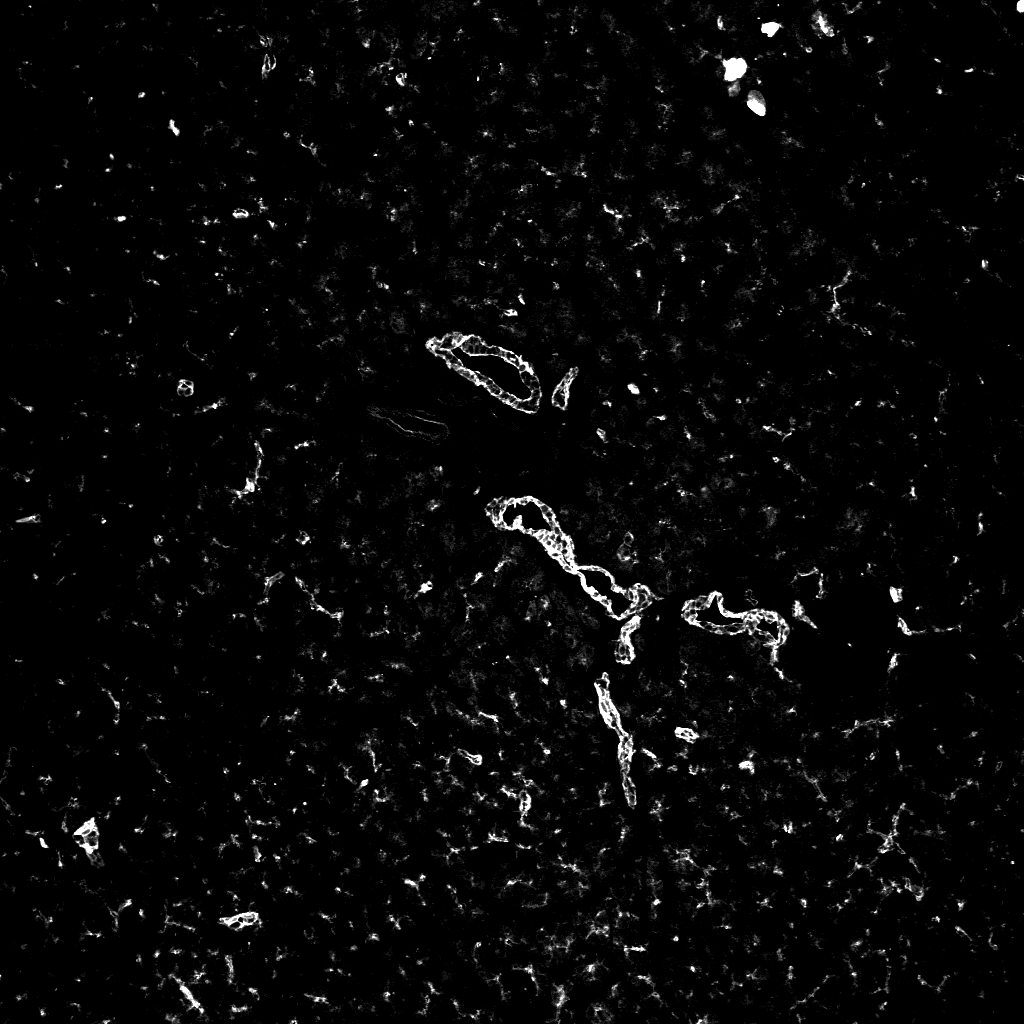

Supplement: Supplementary file 7 — Source data Fig. 5 [file 44318_2025_434_MOESM7_ESM.zip › Figure 5/5D/5D_Merge (gray).tif]

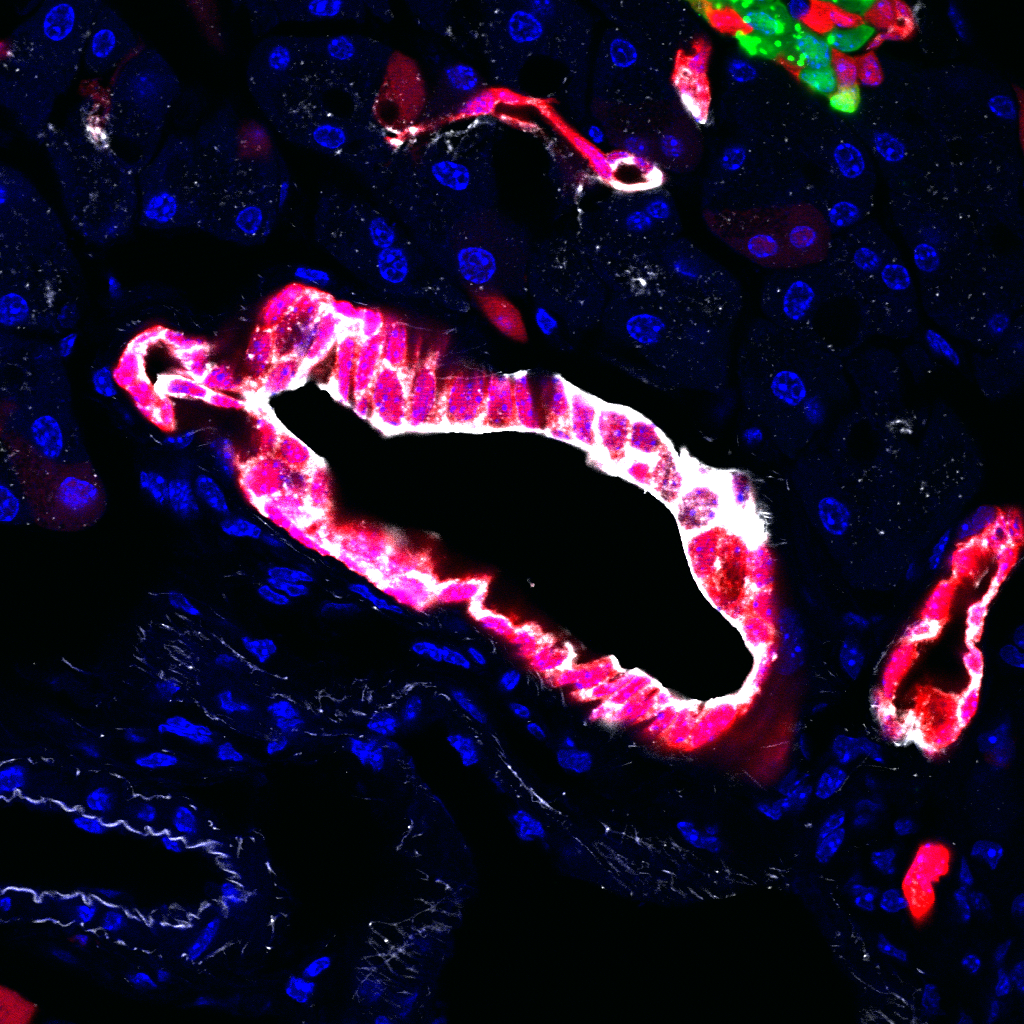

Supplement: Supplementary file 7 — Source data Fig. 5 [file 44318_2025_434_MOESM7_ESM.zip › Figure 5/5D/5D_Merge_Mag_1.tif]

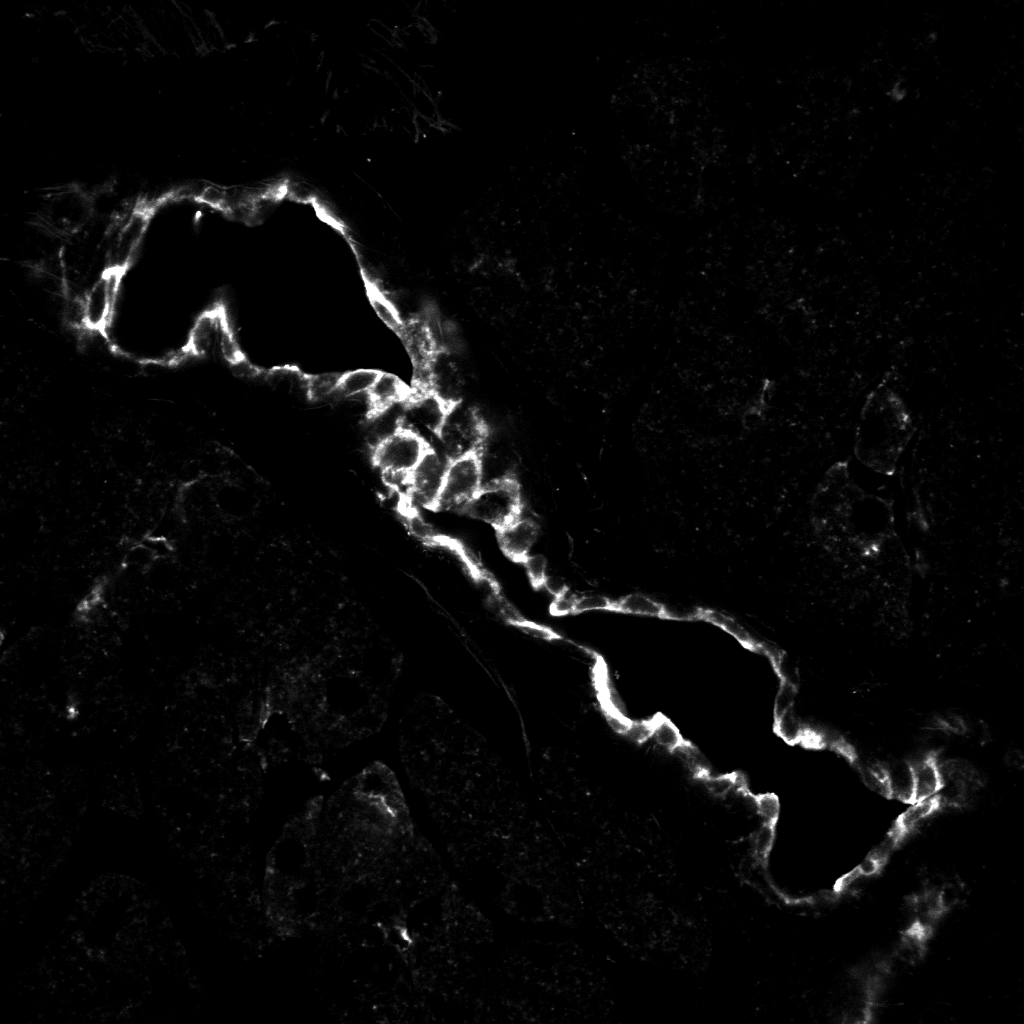

Supplement: Supplementary file 7 — Source data Fig. 5 [file 44318_2025_434_MOESM7_ESM.zip › Figure 5/5D/5D_Merge_Mag_2 (gray).tif]

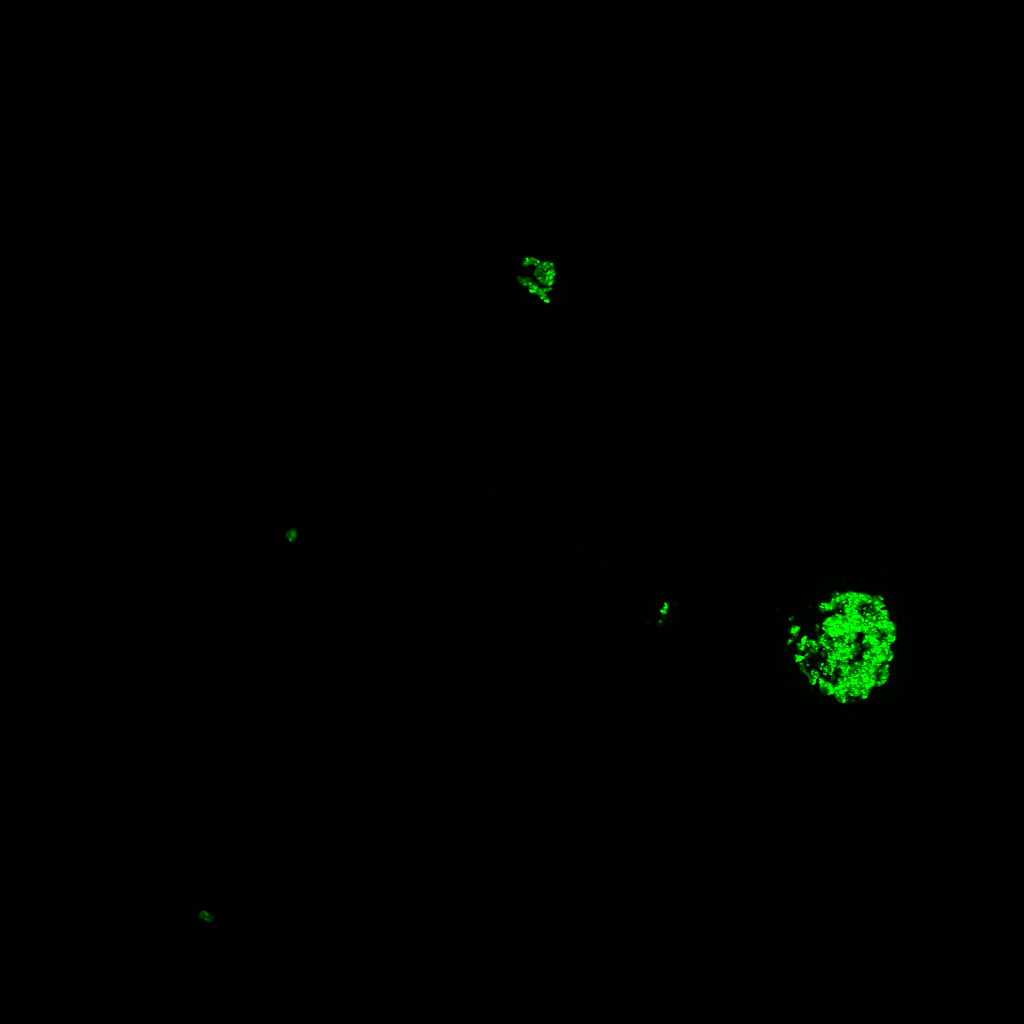

Supplement: Supplementary file 7 — Source data Fig. 5 [file 44318_2025_434_MOESM7_ESM.zip › Figure 5/5D/5D_Merge (green).tif]

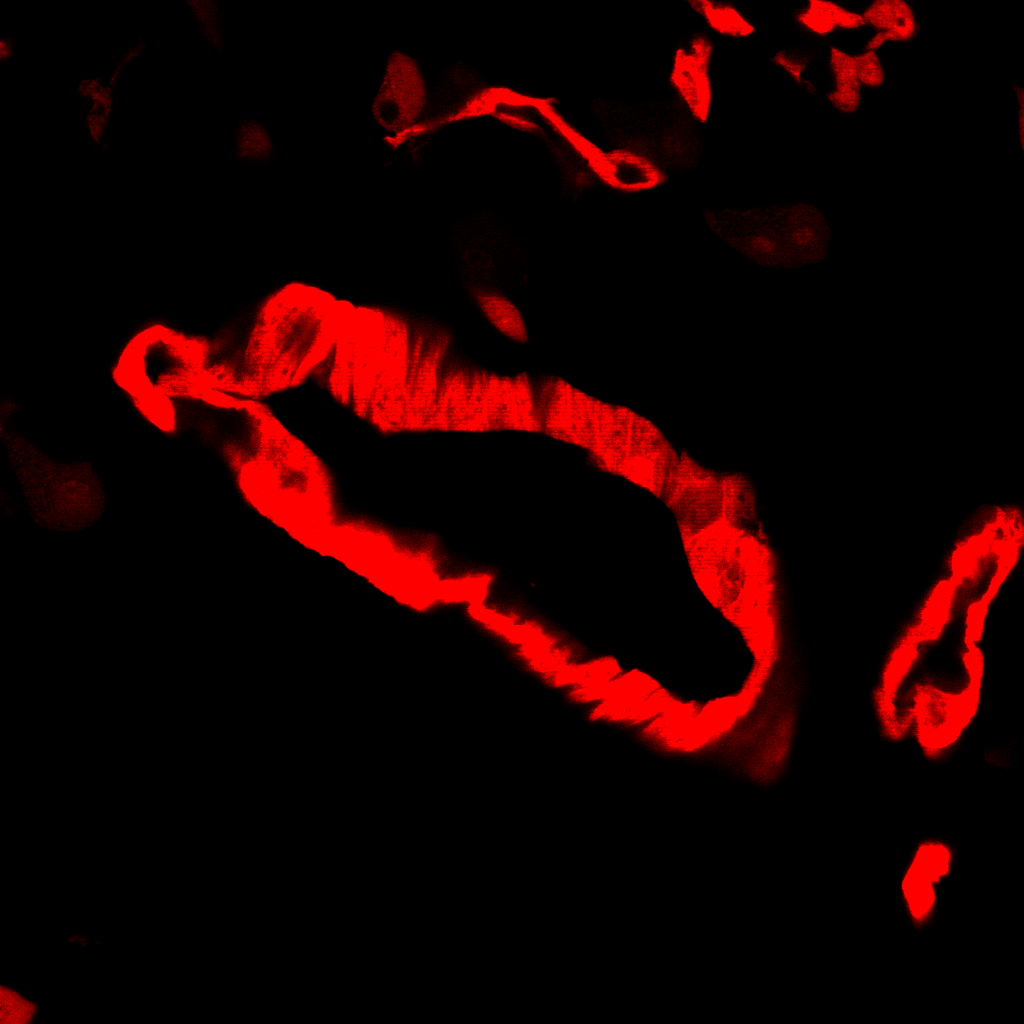

Supplement: Supplementary file 7 — Source data Fig. 5 [file 44318_2025_434_MOESM7_ESM.zip › Figure 5/5D/5D_Merge_Mag_1 (red).tif]

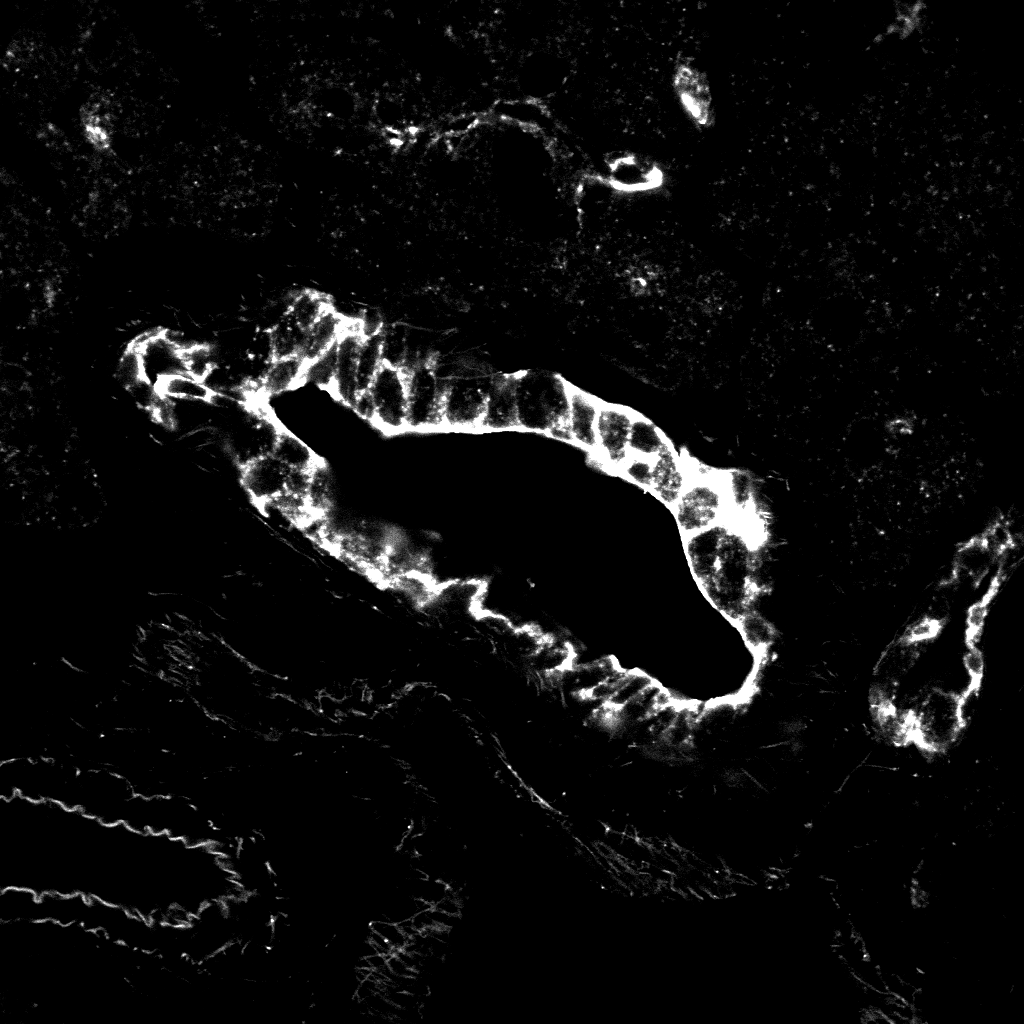

Supplement: Supplementary file 7 — Source data Fig. 5 [file 44318_2025_434_MOESM7_ESM.zip › Figure 5/5D/5D_Merge_Mag_1 (gray).tif]

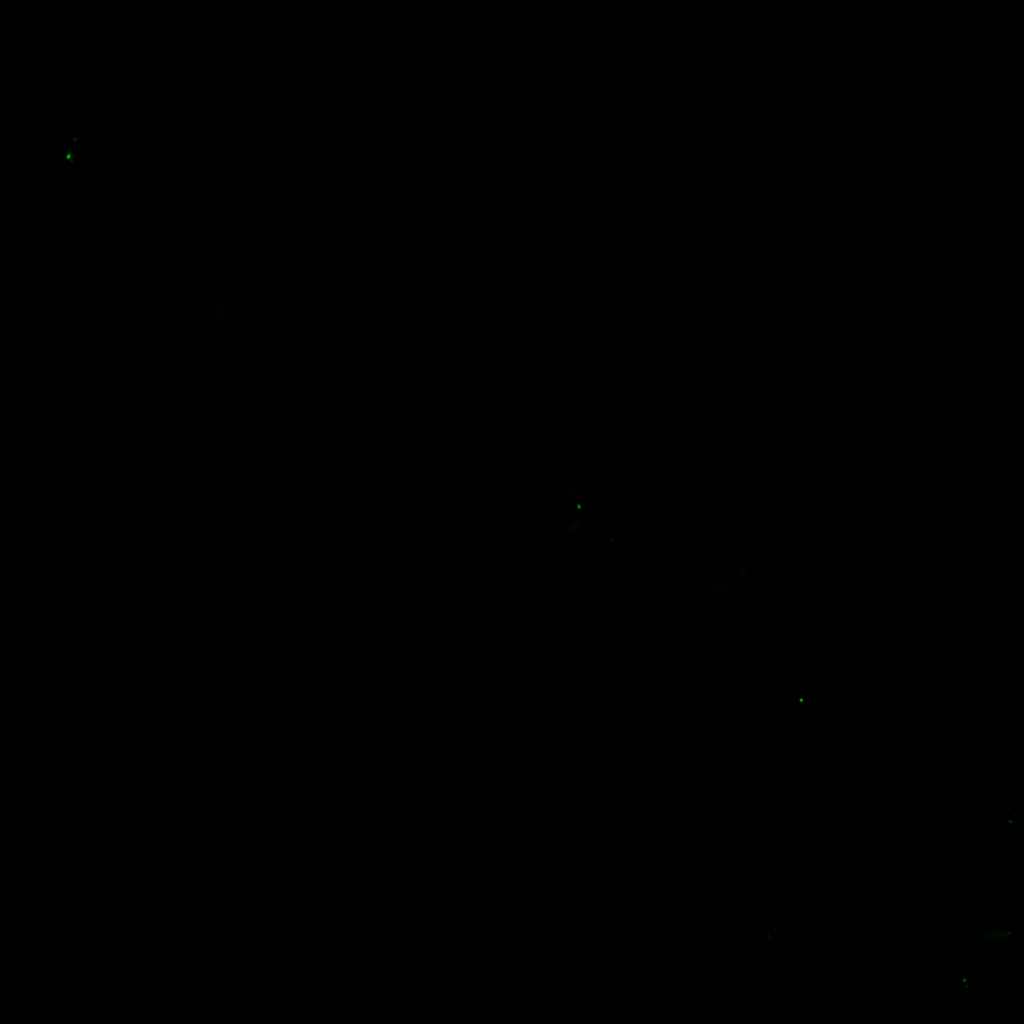

Supplement: Supplementary file 7 — Source data Fig. 5 [file 44318_2025_434_MOESM7_ESM.zip › Figure 5/5D/5D_Merge_Mag_2 (green).tif]

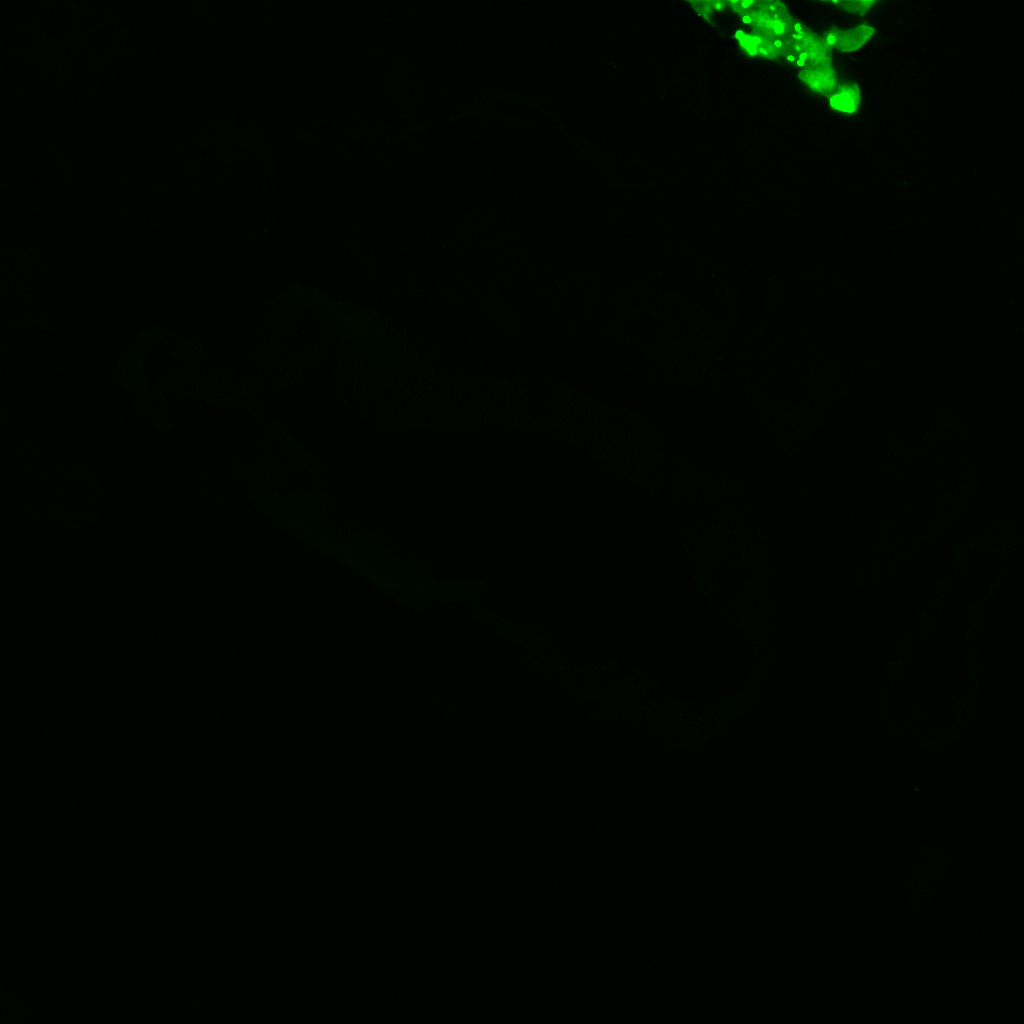

Supplement: Supplementary file 7 — Source data Fig. 5 [file 44318_2025_434_MOESM7_ESM.zip › Figure 5/5D/5D_Merge_Mag_1 (green).tif]

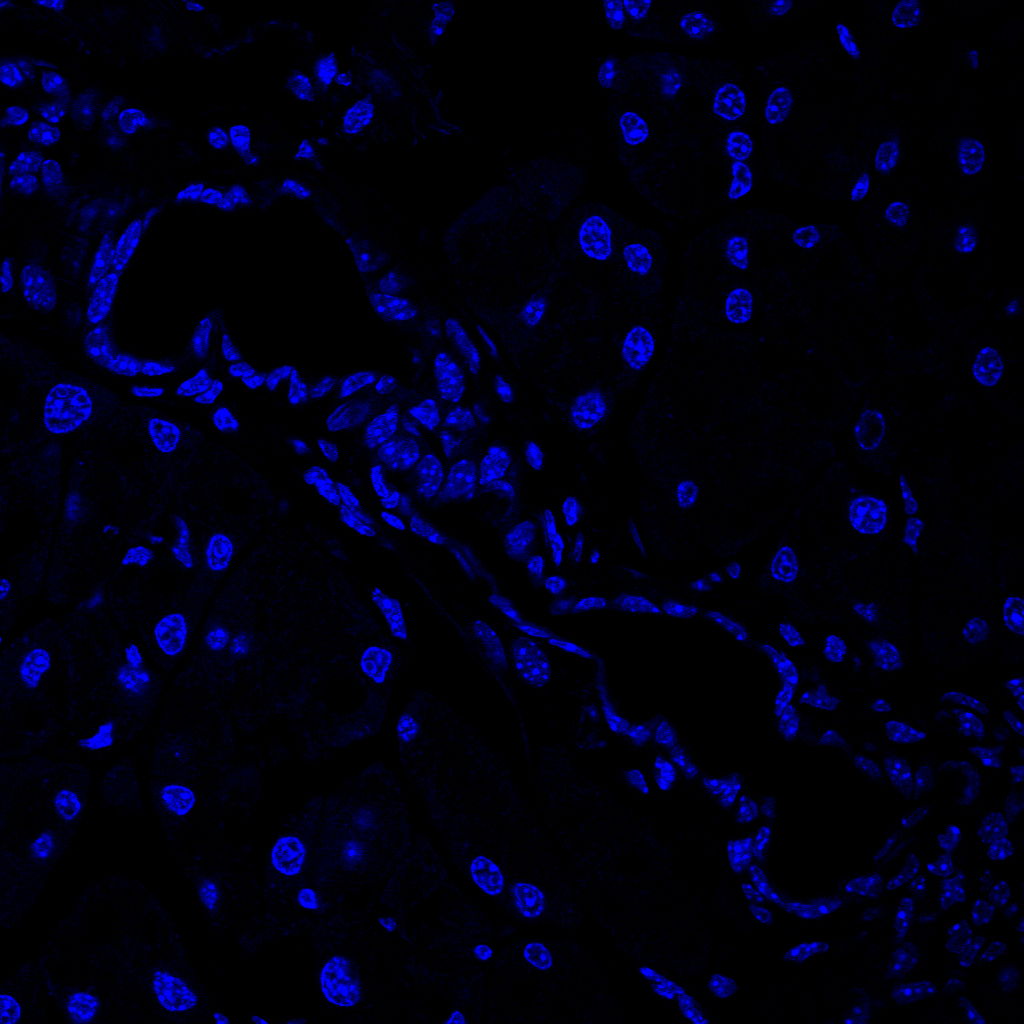

Supplement: Supplementary file 7 — Source data Fig. 5 [file 44318_2025_434_MOESM7_ESM.zip › Figure 5/5D/5D_Merge_Mag_2 (blue).tif]

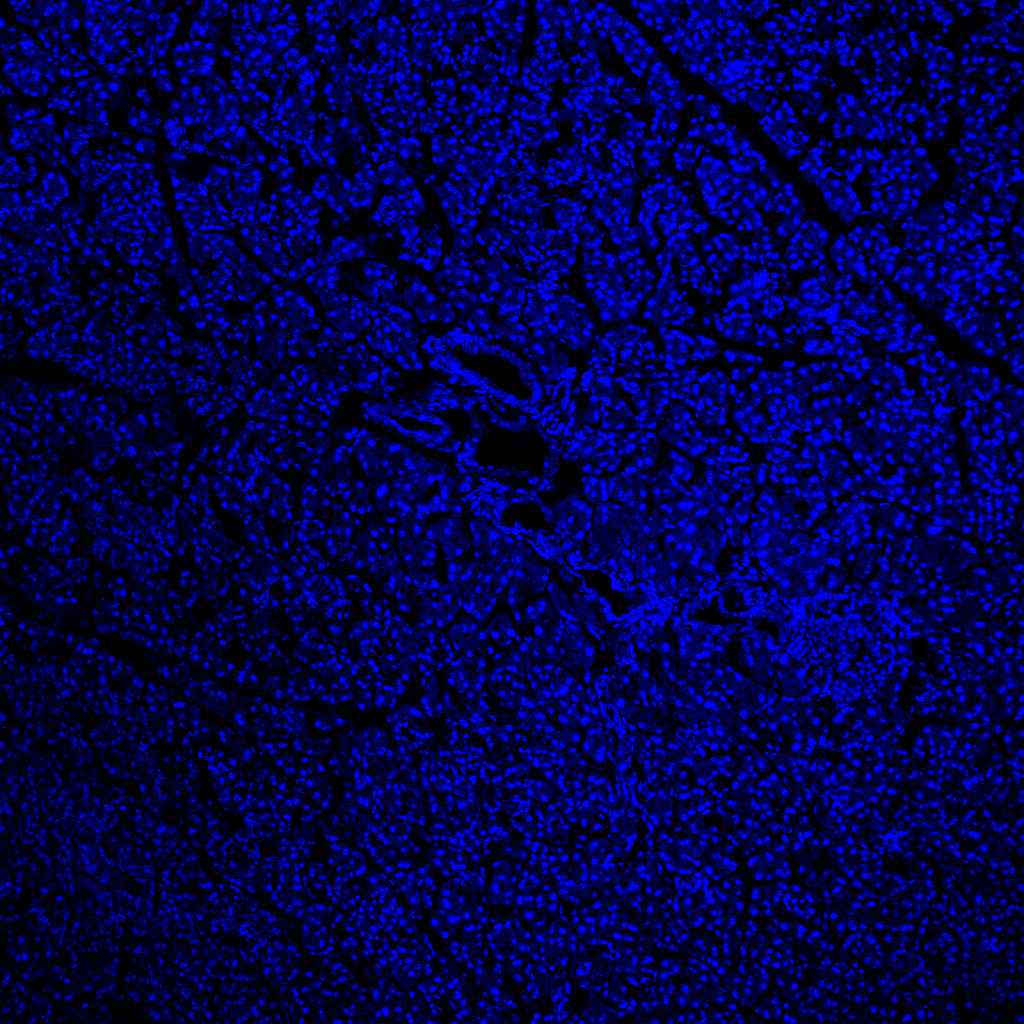

Supplement: Supplementary file 7 — Source data Fig. 5 [file 44318_2025_434_MOESM7_ESM.zip › Figure 5/5D/5D_Merge (blue).tif]

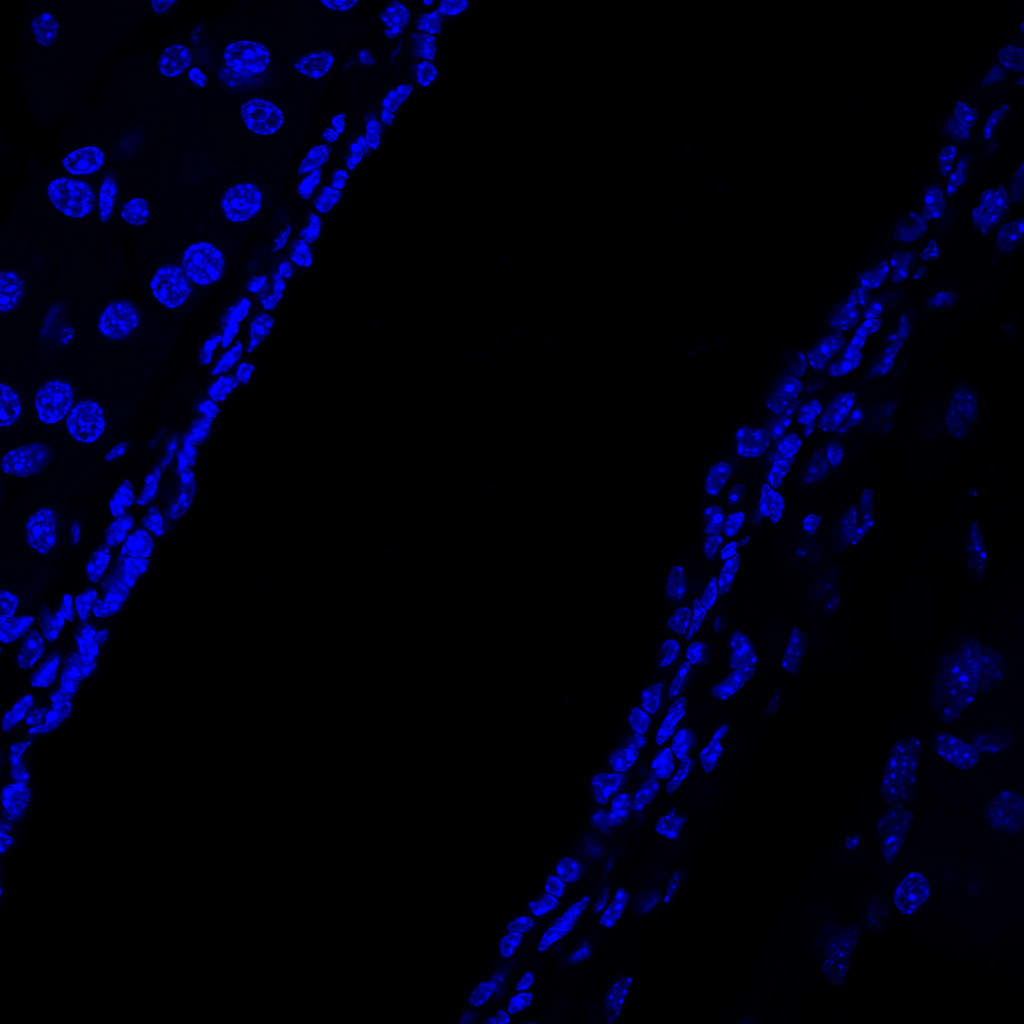

Supplement: Supplementary file 8 — Source data Fig. 6 [file 44318_2025_434_MOESM8_ESM.zip › Figure 6/6F/6F_Ins_Merge (blue).tif]

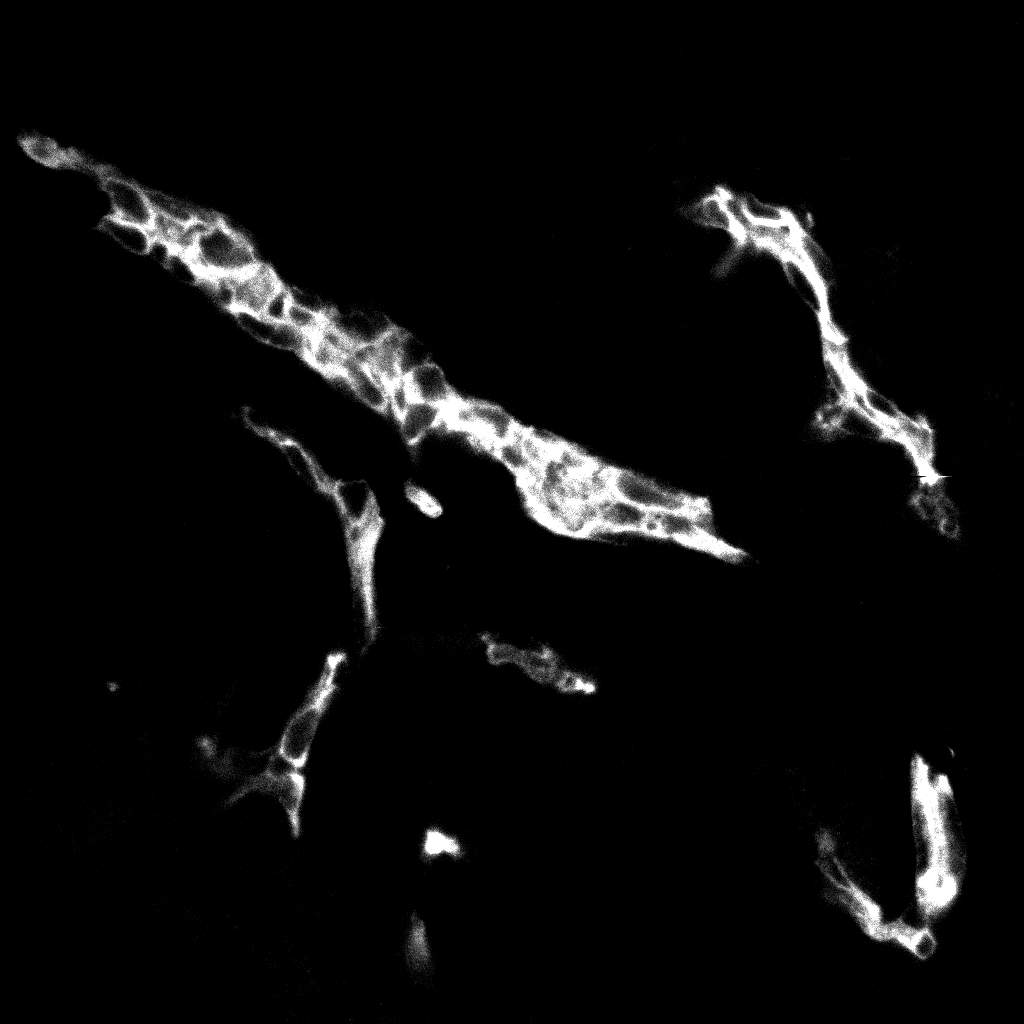

Supplement: Supplementary file 8 — Source data Fig. 6 [file 44318_2025_434_MOESM8_ESM.zip › Figure 6/6F/6F_CK19_Merge (gray).tif]

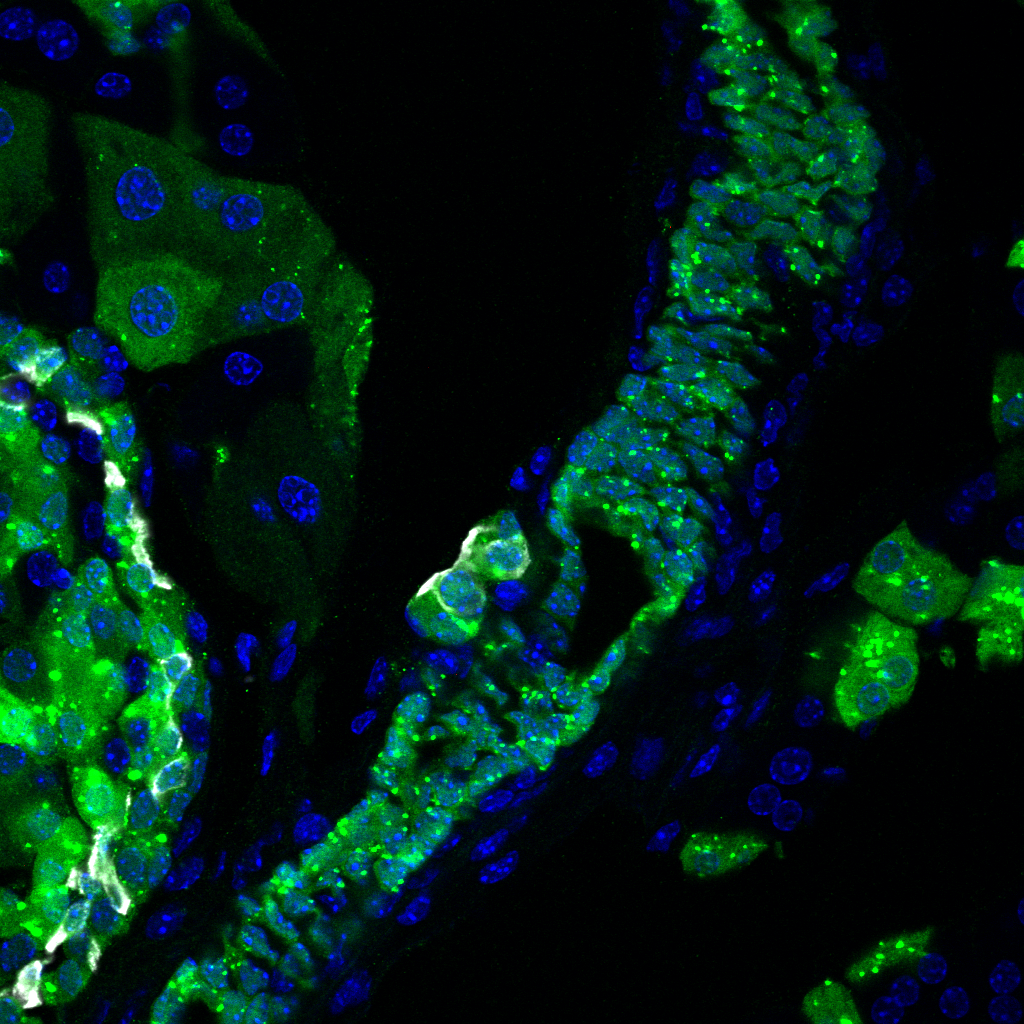

Supplement: Supplementary file 8 — Source data Fig. 6 [file 44318_2025_434_MOESM8_ESM.zip › Figure 6/6F/6F_Sst_Merge.tif]

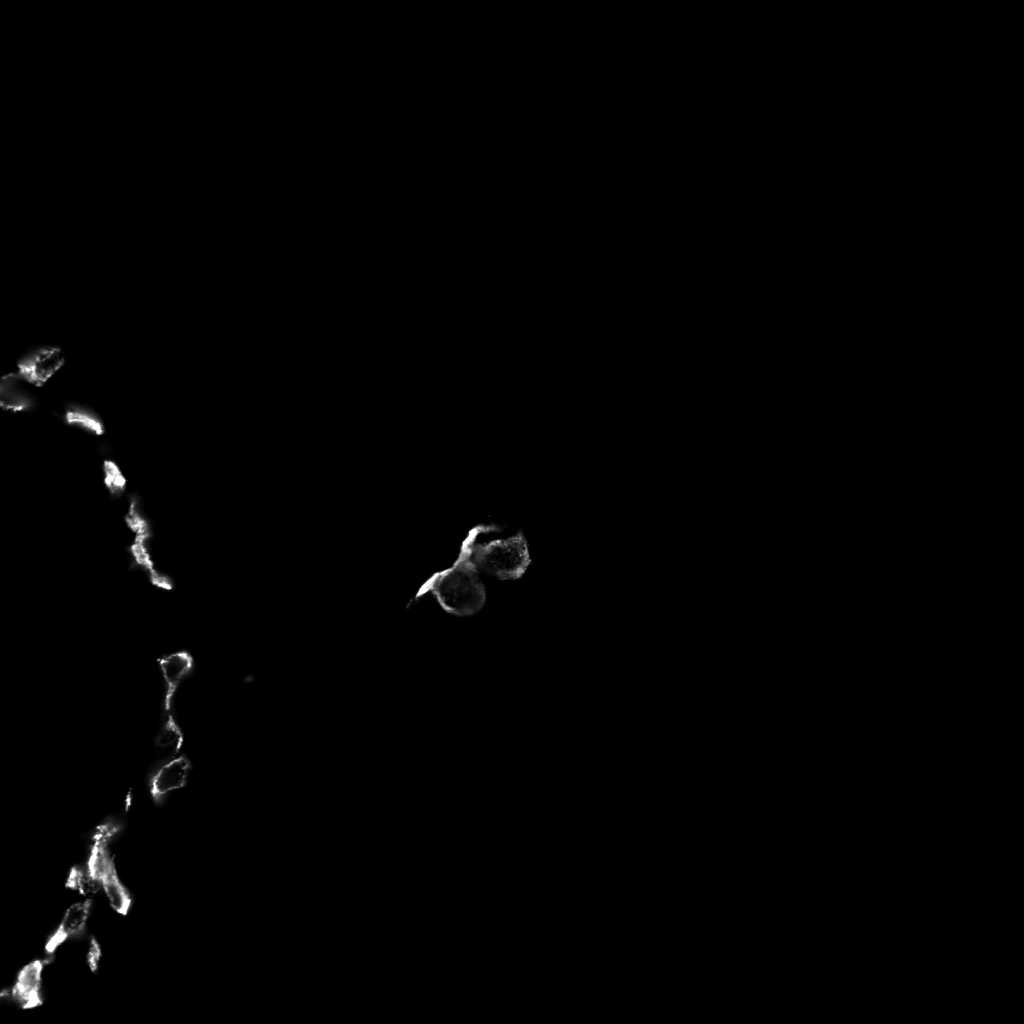

Supplement: Supplementary file 8 — Source data Fig. 6 [file 44318_2025_434_MOESM8_ESM.zip › Figure 6/6F/6F_Sst_Merge (gray).tif]

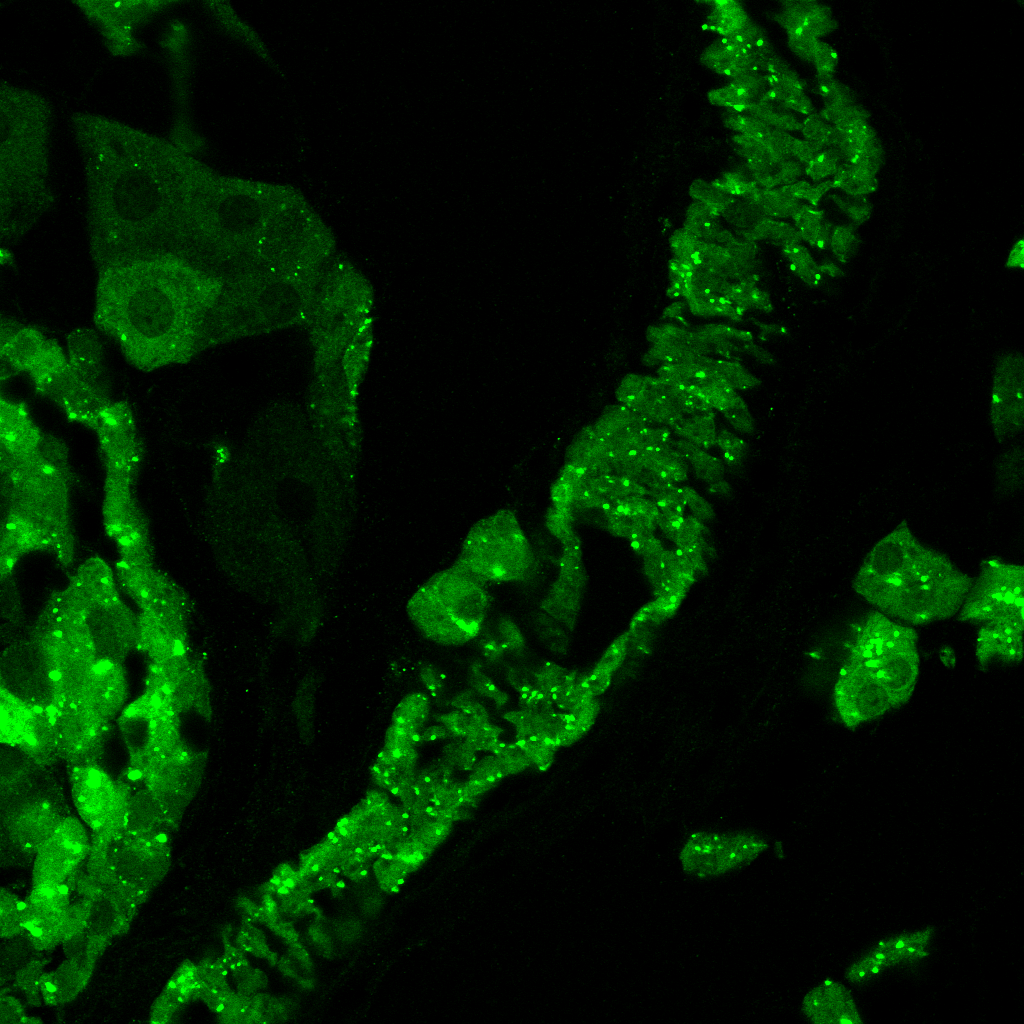

Supplement: Supplementary file 8 — Source data Fig. 6 [file 44318_2025_434_MOESM8_ESM.zip › Figure 6/6F/6F_Sst_Merge (green).tif]

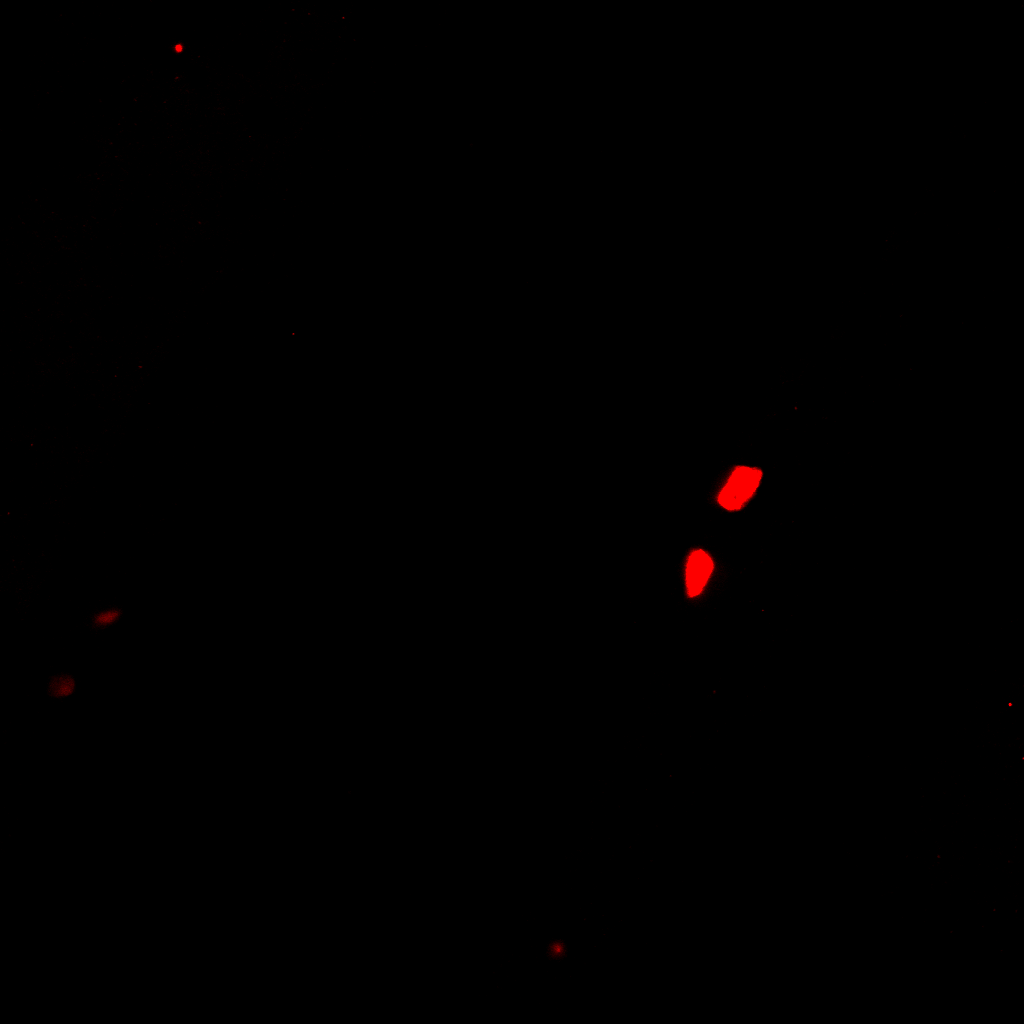

Supplement: Supplementary file 8 — Source data Fig. 6 [file 44318_2025_434_MOESM8_ESM.zip › Figure 6/6F/6F_Ins_Merge (red).tif]

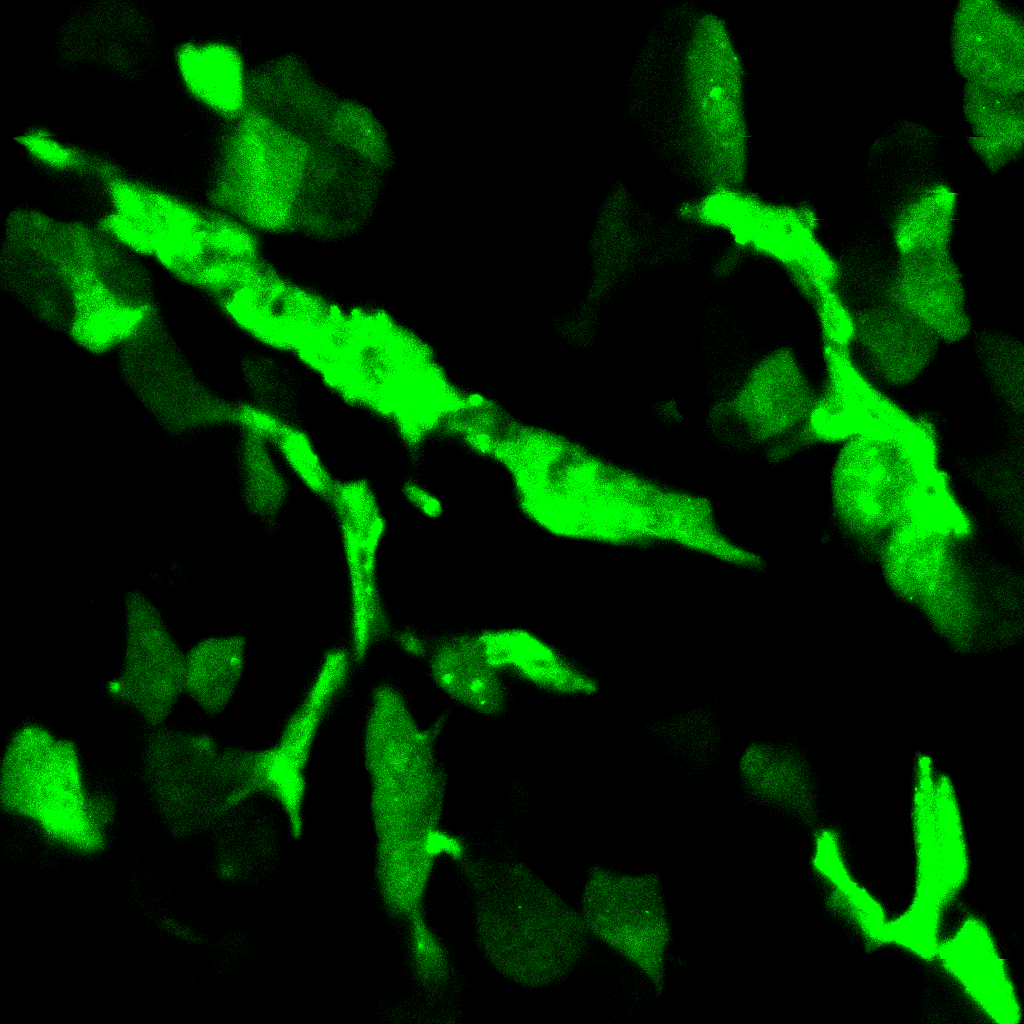

Supplement: Supplementary file 8 — Source data Fig. 6 [file 44318_2025_434_MOESM8_ESM.zip › Figure 6/6F/6F_CK19_Merge (green).tif]

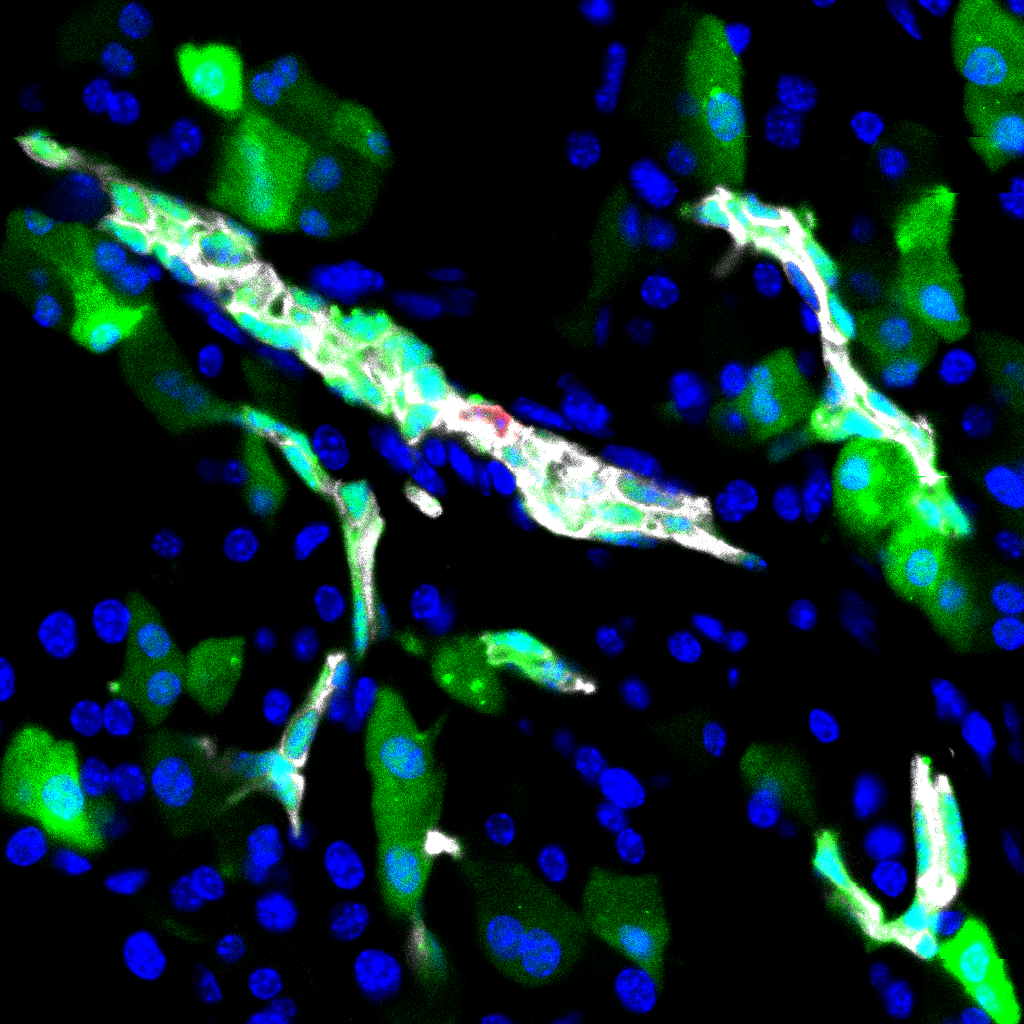

Supplement: Supplementary file 8 — Source data Fig. 6 [file 44318_2025_434_MOESM8_ESM.zip › Figure 6/6F/6F_CK19_Merge.tif]

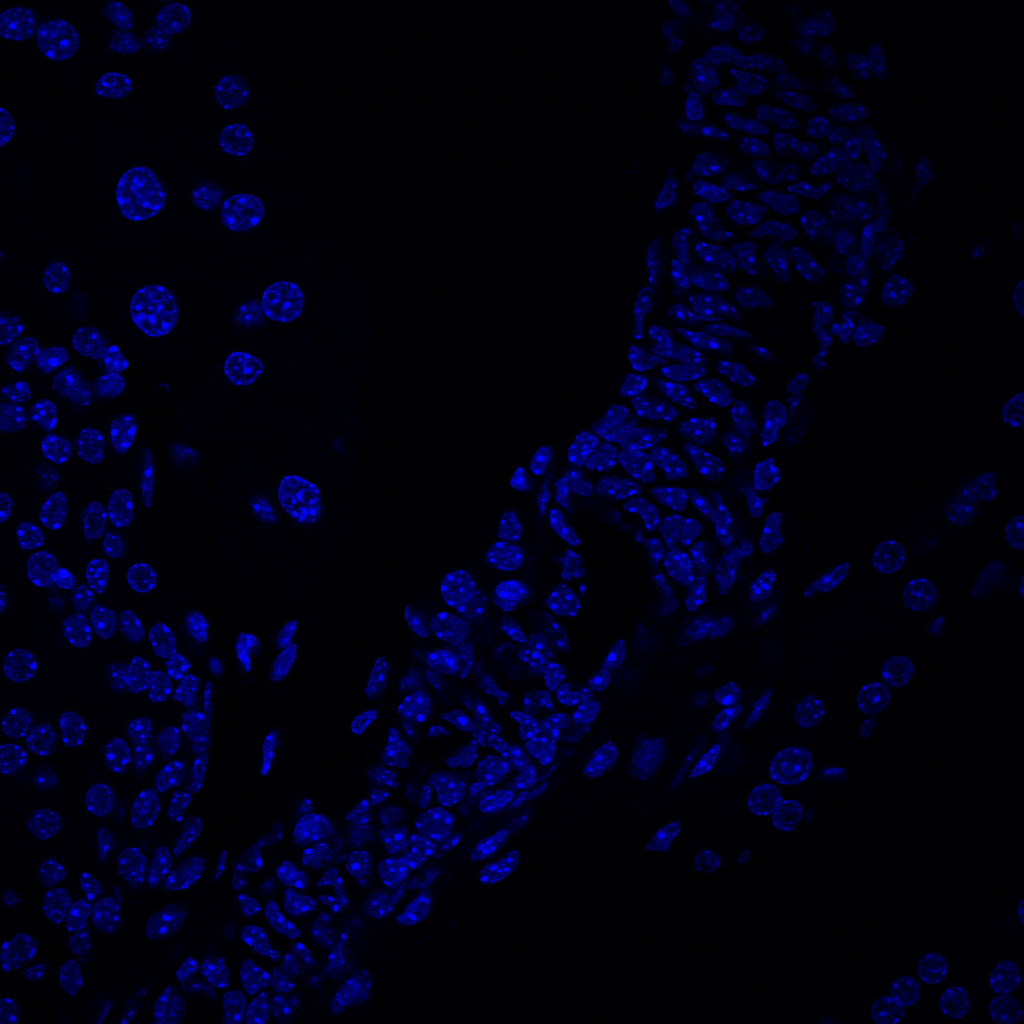

Supplement: Supplementary file 8 — Source data Fig. 6 [file 44318_2025_434_MOESM8_ESM.zip › Figure 6/6F/6F_Sst_Merge (blue).tif]

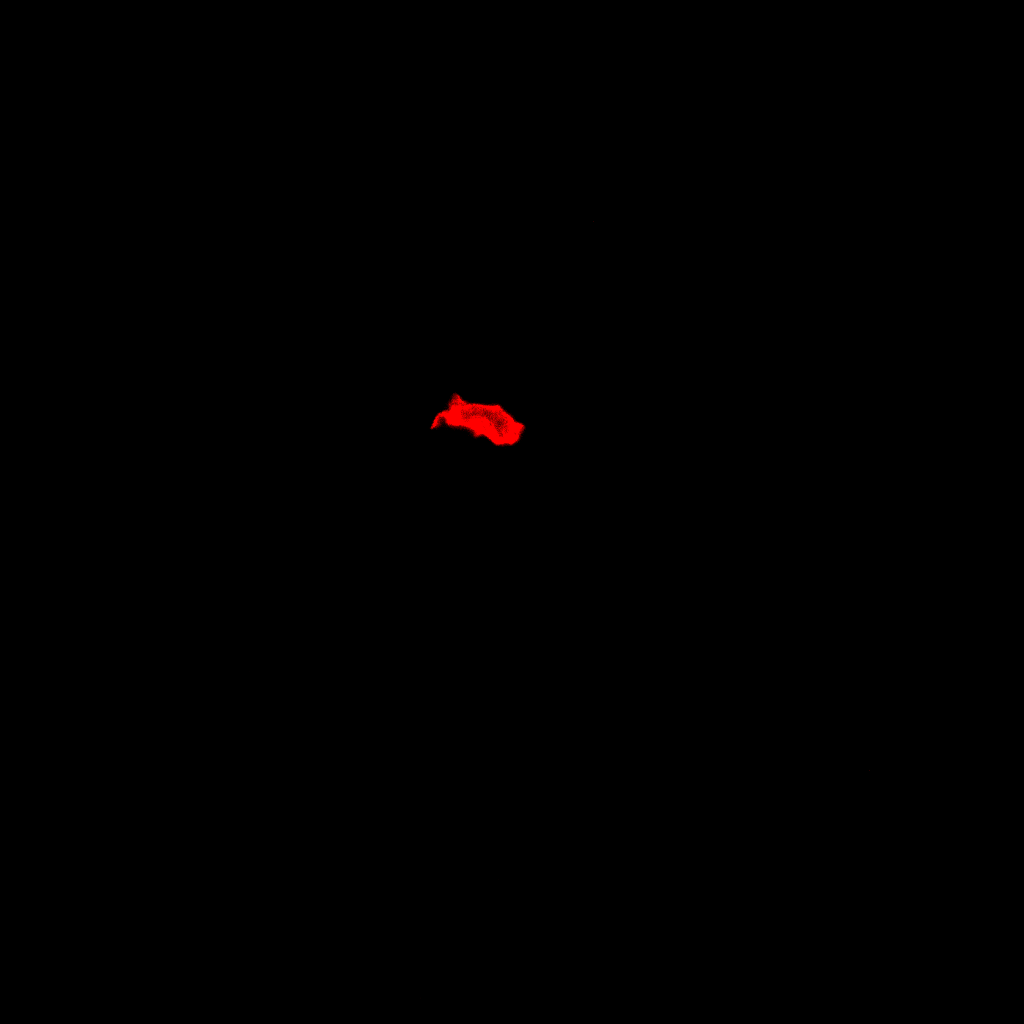

Supplement: Supplementary file 8 — Source data Fig. 6 [file 44318_2025_434_MOESM8_ESM.zip › Figure 6/6F/6F_CK19_Merge (red).tif]

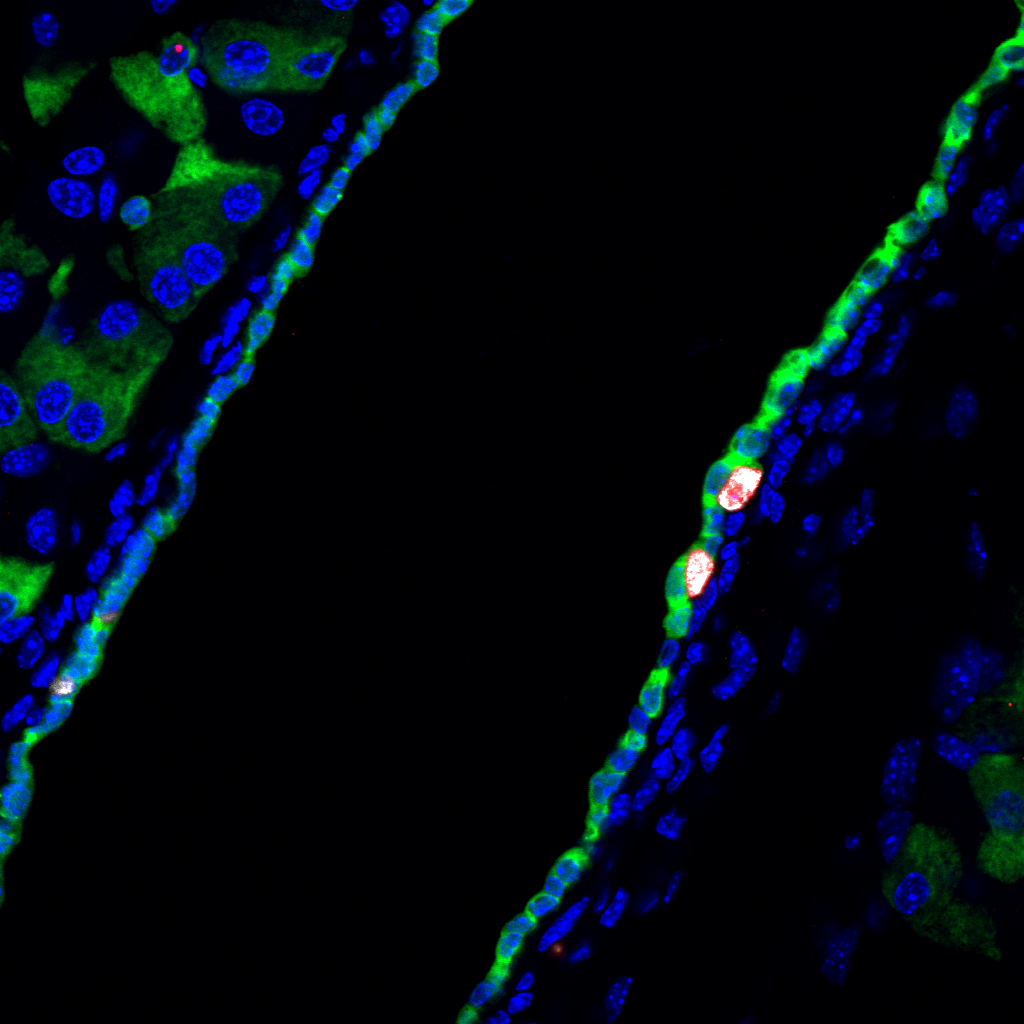

Supplement: Supplementary file 8 — Source data Fig. 6 [file 44318_2025_434_MOESM8_ESM.zip › Figure 6/6F/6F_Ins_Merge.tif]

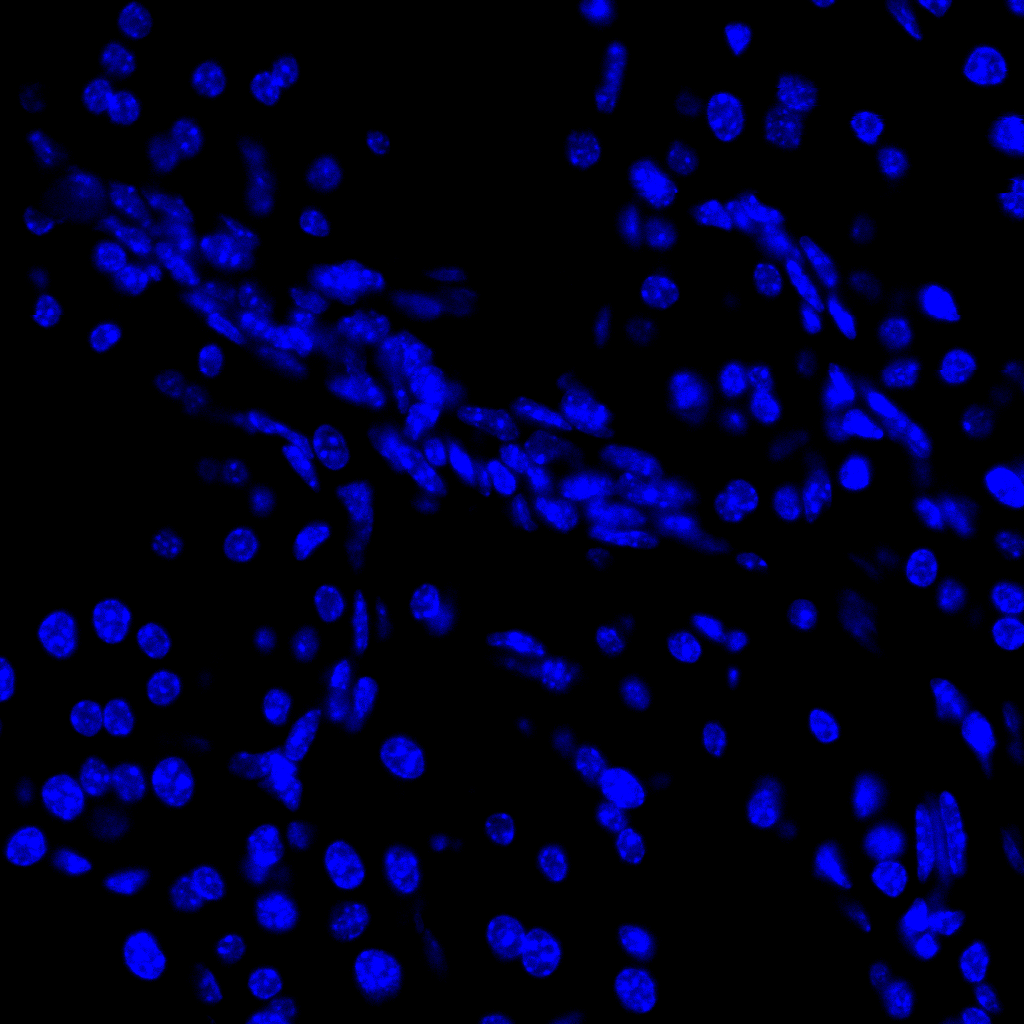

Supplement: Supplementary file 8 — Source data Fig. 6 [file 44318_2025_434_MOESM8_ESM.zip › Figure 6/6F/6F_CK19_Merge (blue).tif]

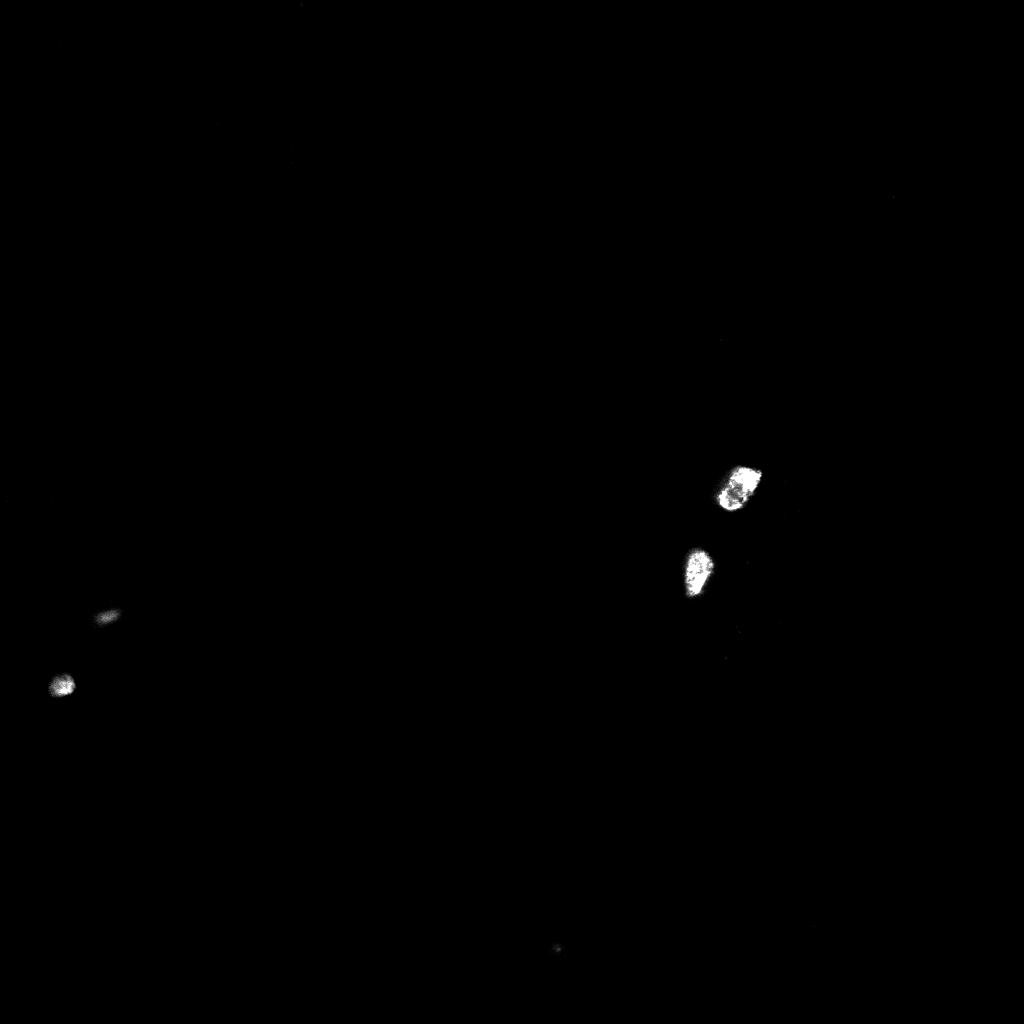

Supplement: Supplementary file 8 — Source data Fig. 6 [file 44318_2025_434_MOESM8_ESM.zip › Figure 6/6F/6F_Ins_Merge (gray).tif]

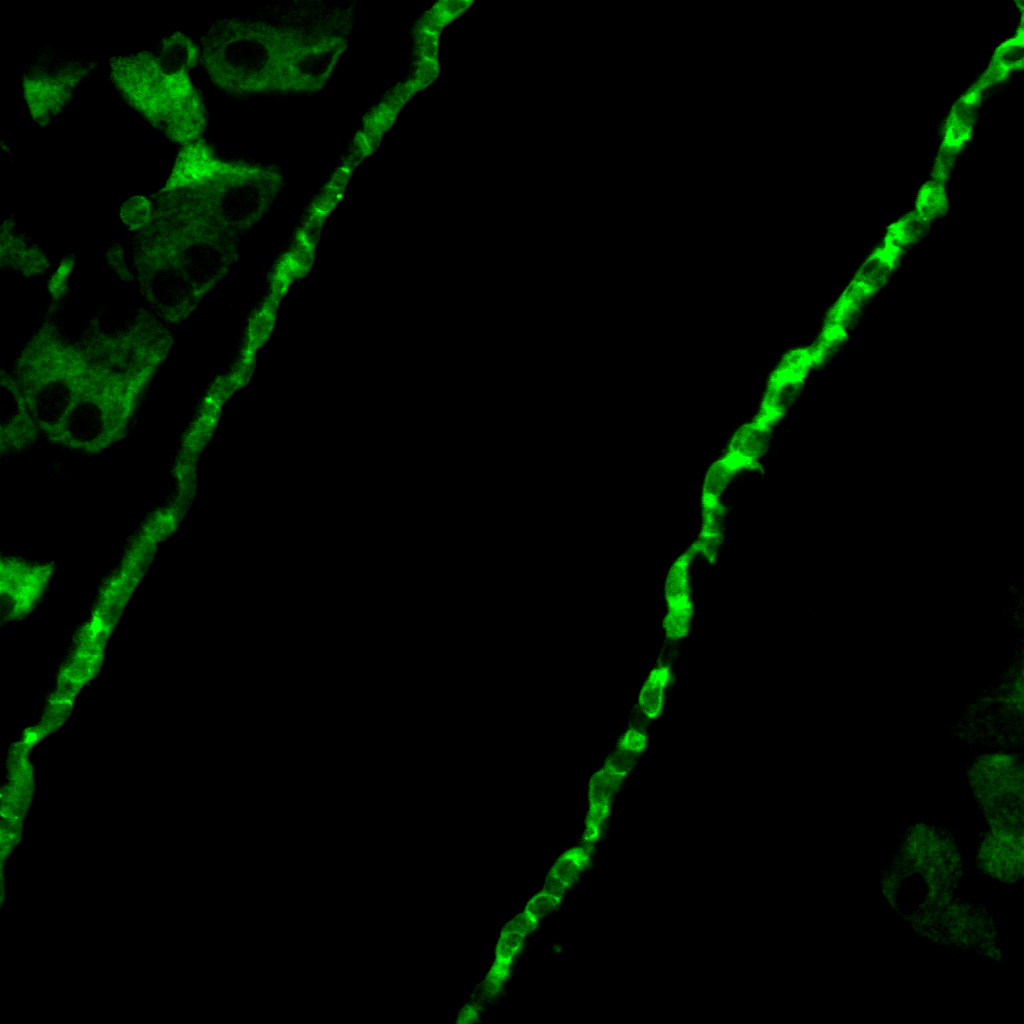

Supplement: Supplementary file 8 — Source data Fig. 6 [file 44318_2025_434_MOESM8_ESM.zip › Figure 6/6F/6F_Ins_Merge (green).tif]

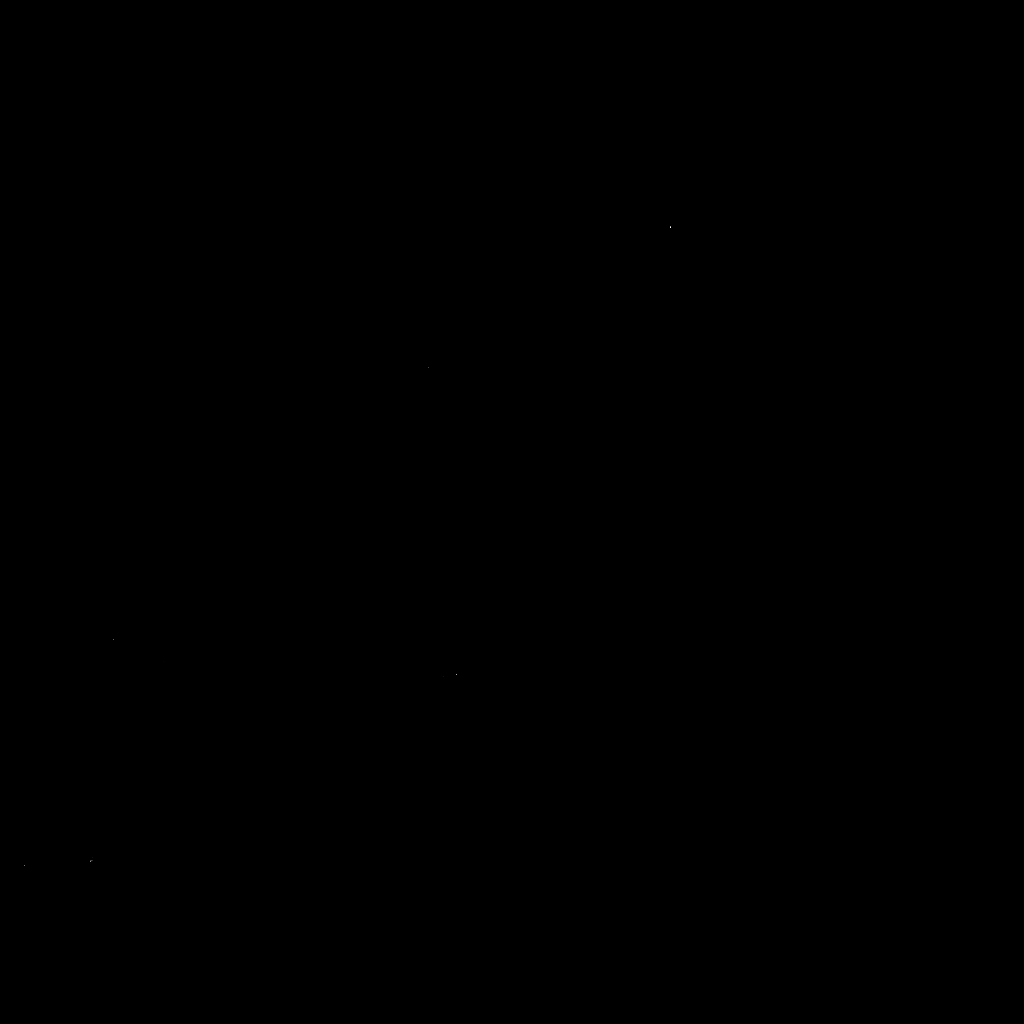

Supplement: Supplementary file 8 — Source data Fig. 6 [file 44318_2025_434_MOESM8_ESM.zip › Figure 6/6F/6F_Sst_Merge (red).tif]

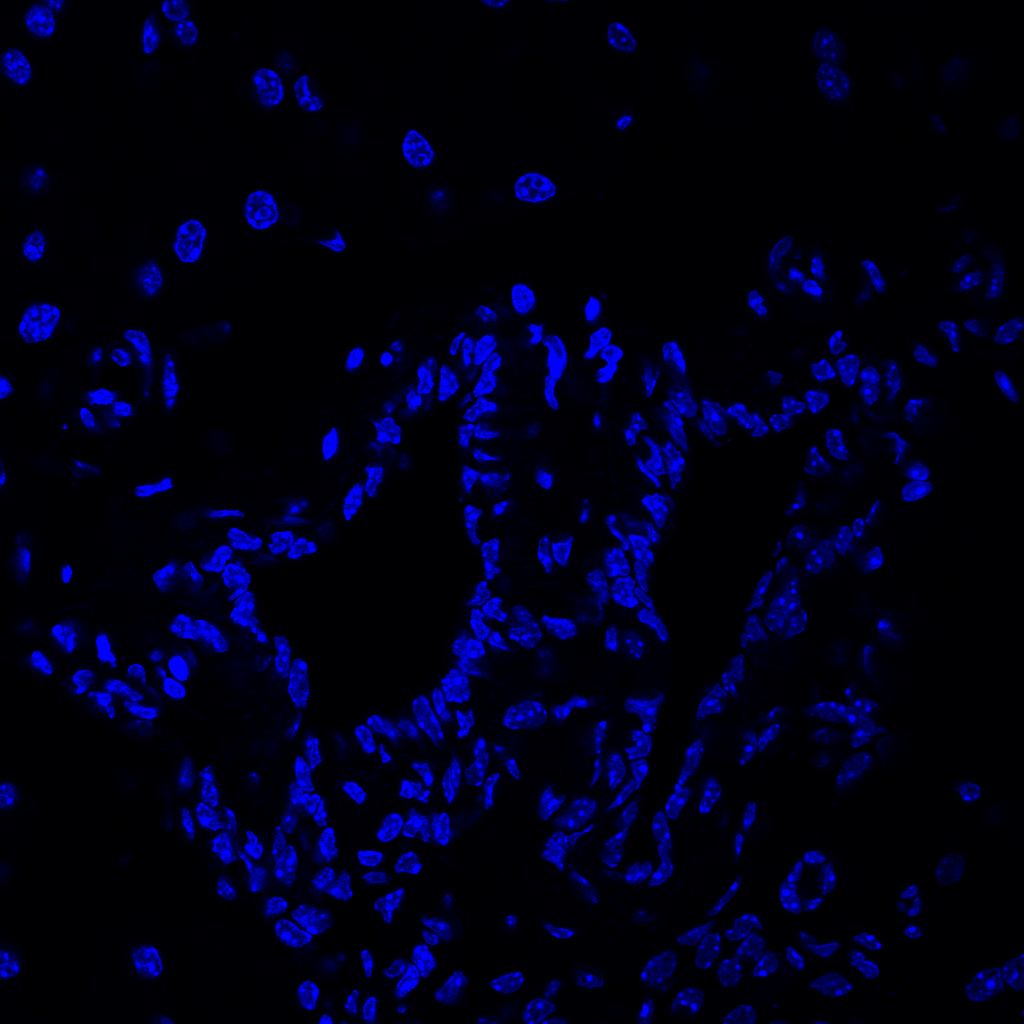

Supplement: Supplementary file 8 — Source data Fig. 6 [file 44318_2025_434_MOESM8_ESM.zip › Figure 6/6H/6H_12w_Ins (blue).tif]

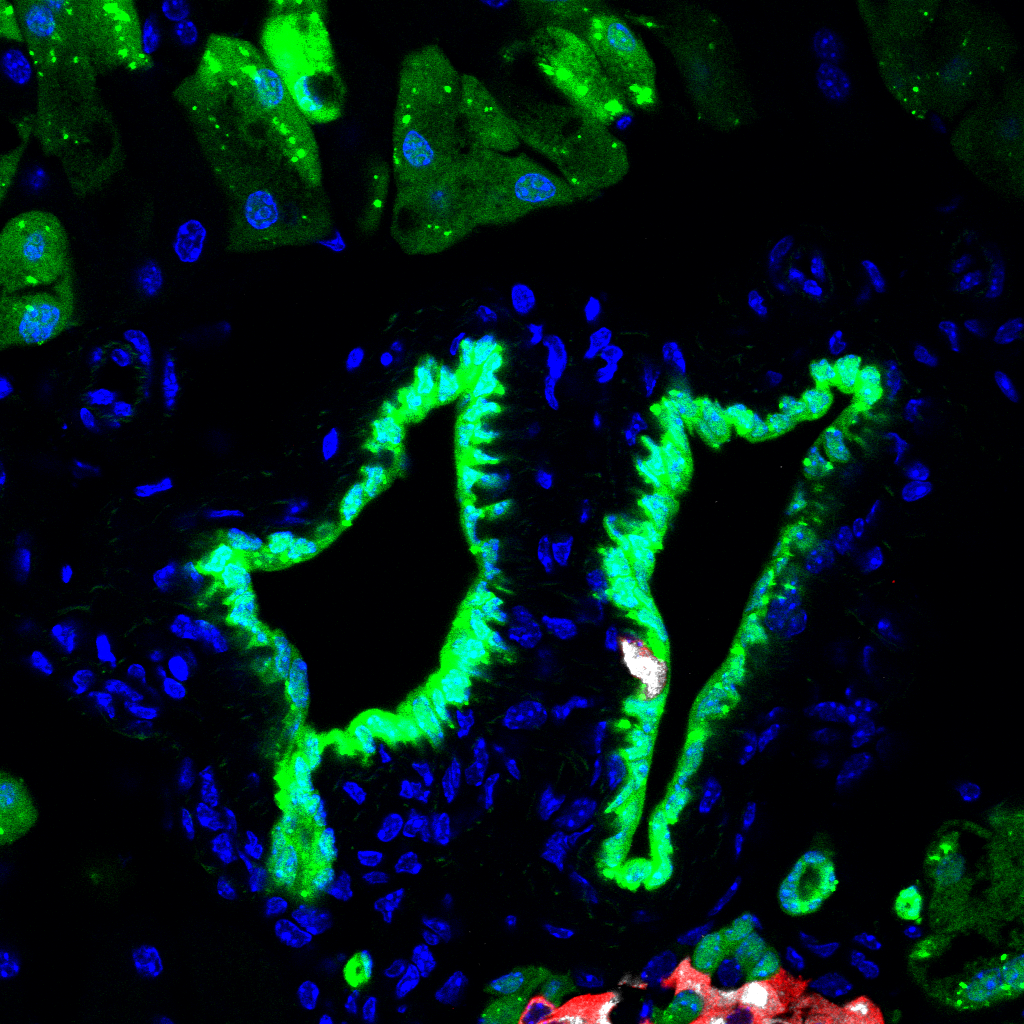

Supplement: Supplementary file 8 — Source data Fig. 6 [file 44318_2025_434_MOESM8_ESM.zip › Figure 6/6H/6H_12w_Ins.tif]

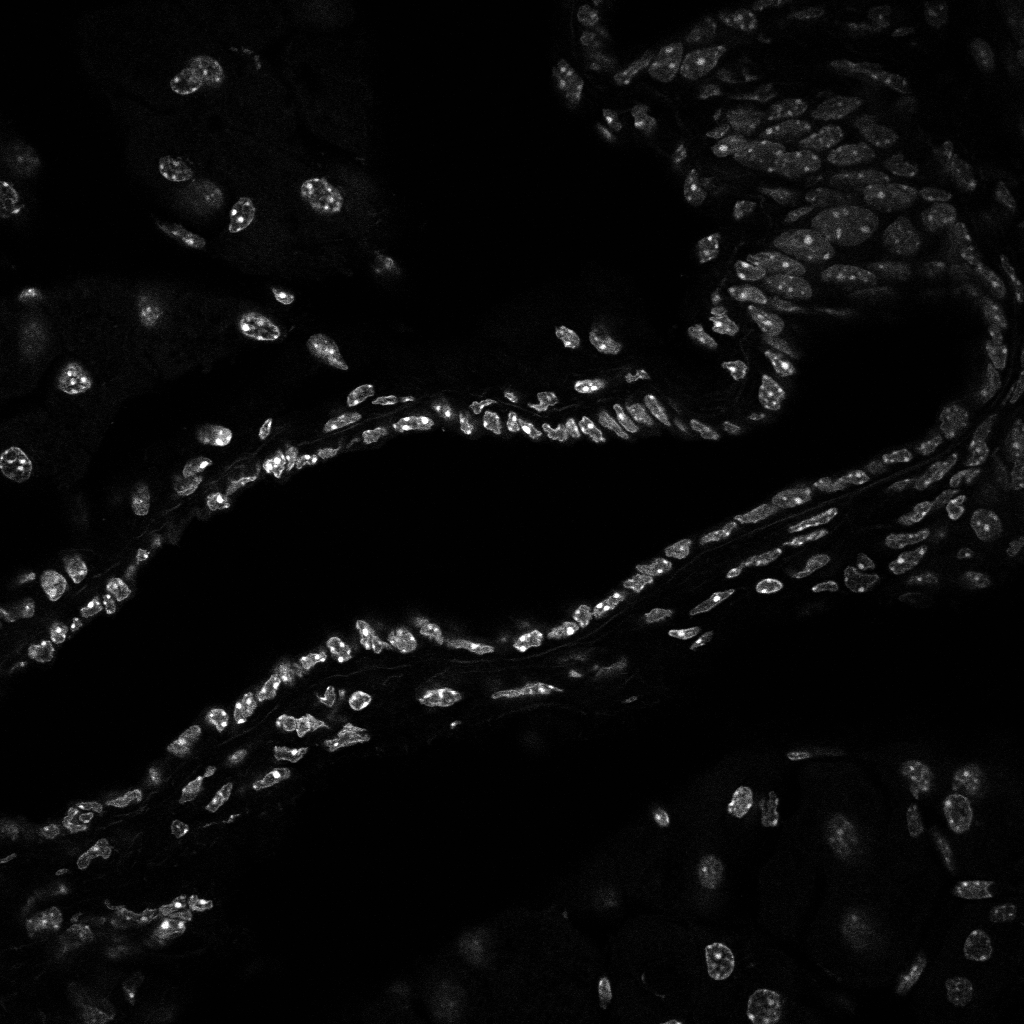

Supplement: Supplementary file 8 — Source data Fig. 6 [file 44318_2025_434_MOESM8_ESM.zip › Figure 6/6H/6H_12w_CK19 (blue).tif]

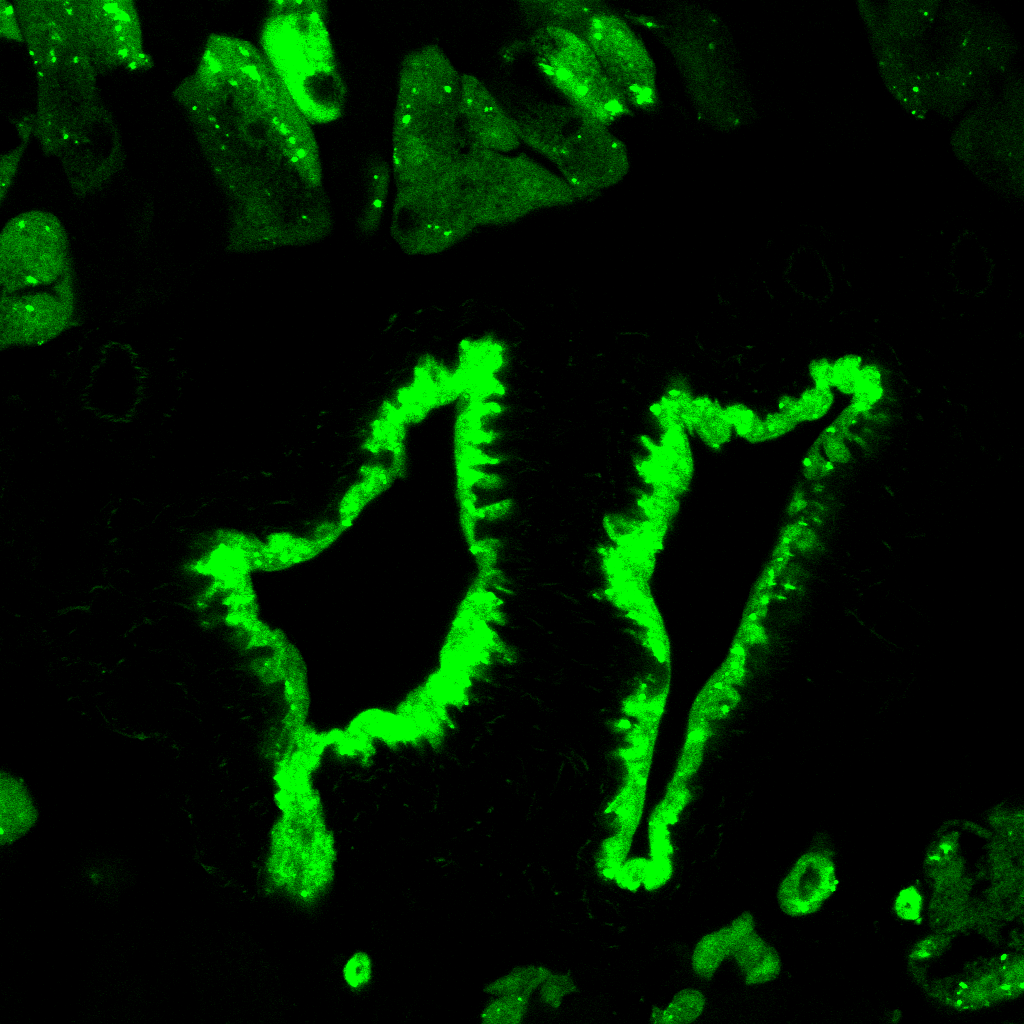

Supplement: Supplementary file 8 — Source data Fig. 6 [file 44318_2025_434_MOESM8_ESM.zip › Figure 6/6H/6H_12w_Ins (green).tif]

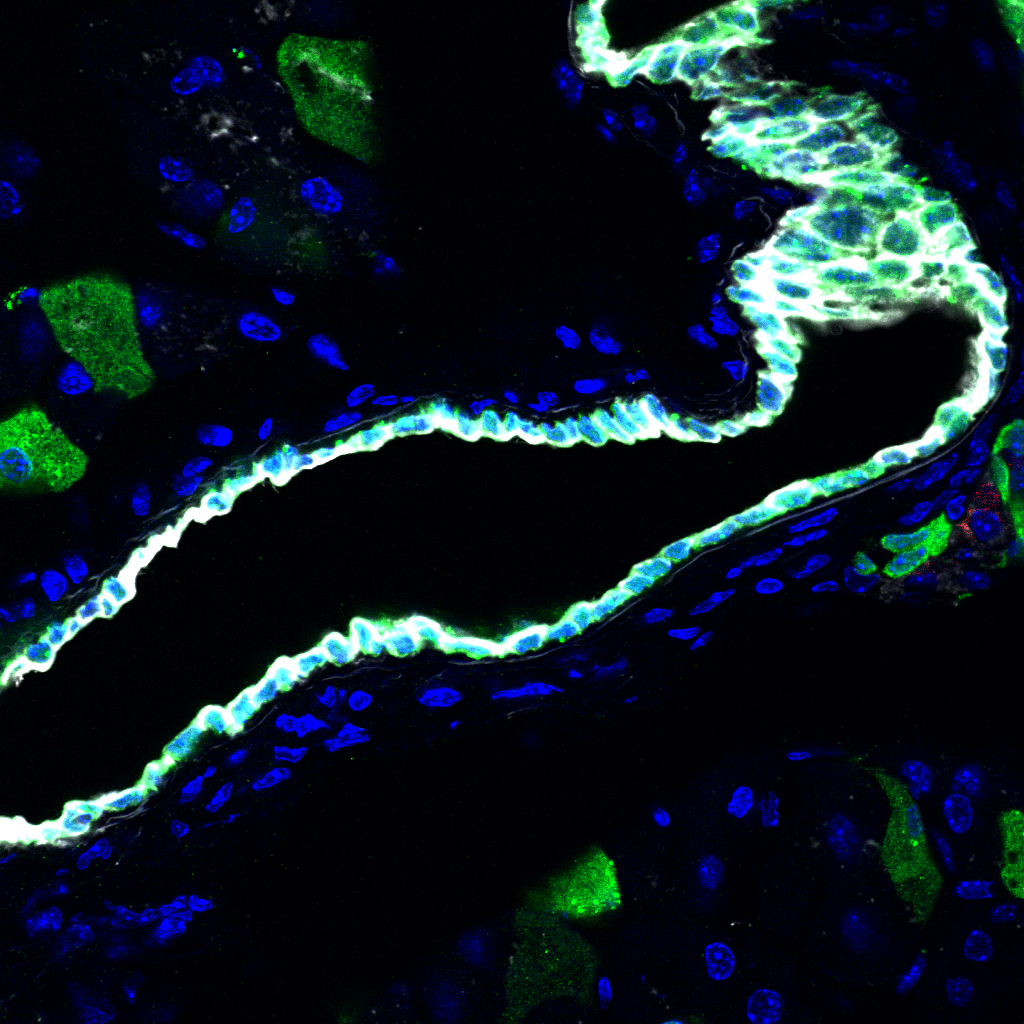

Supplement: Supplementary file 8 — Source data Fig. 6 [file 44318_2025_434_MOESM8_ESM.zip › Figure 6/6H/6H_12w_CK19.tif]

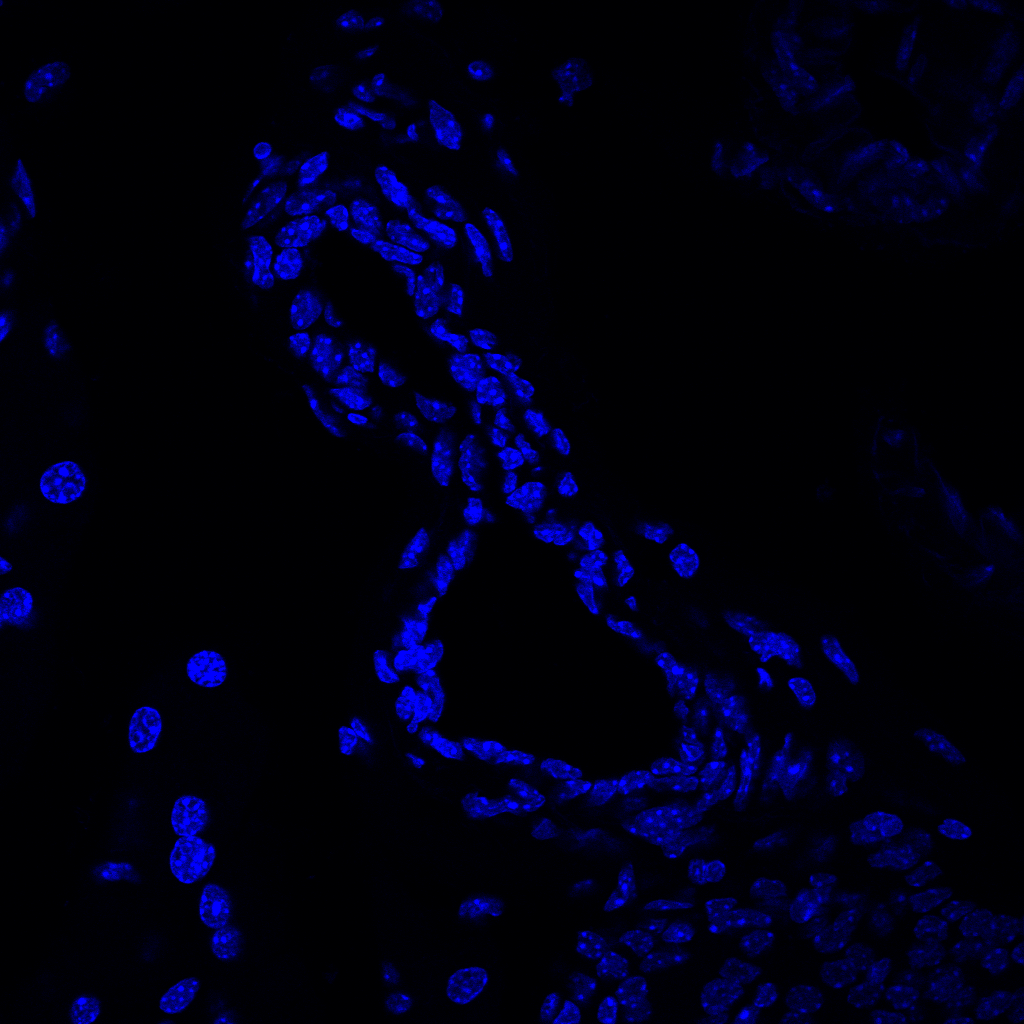

Supplement: Supplementary file 8 — Source data Fig. 6 [file 44318_2025_434_MOESM8_ESM.zip › Figure 6/6H/6H_12w_Sst (blue).tif]

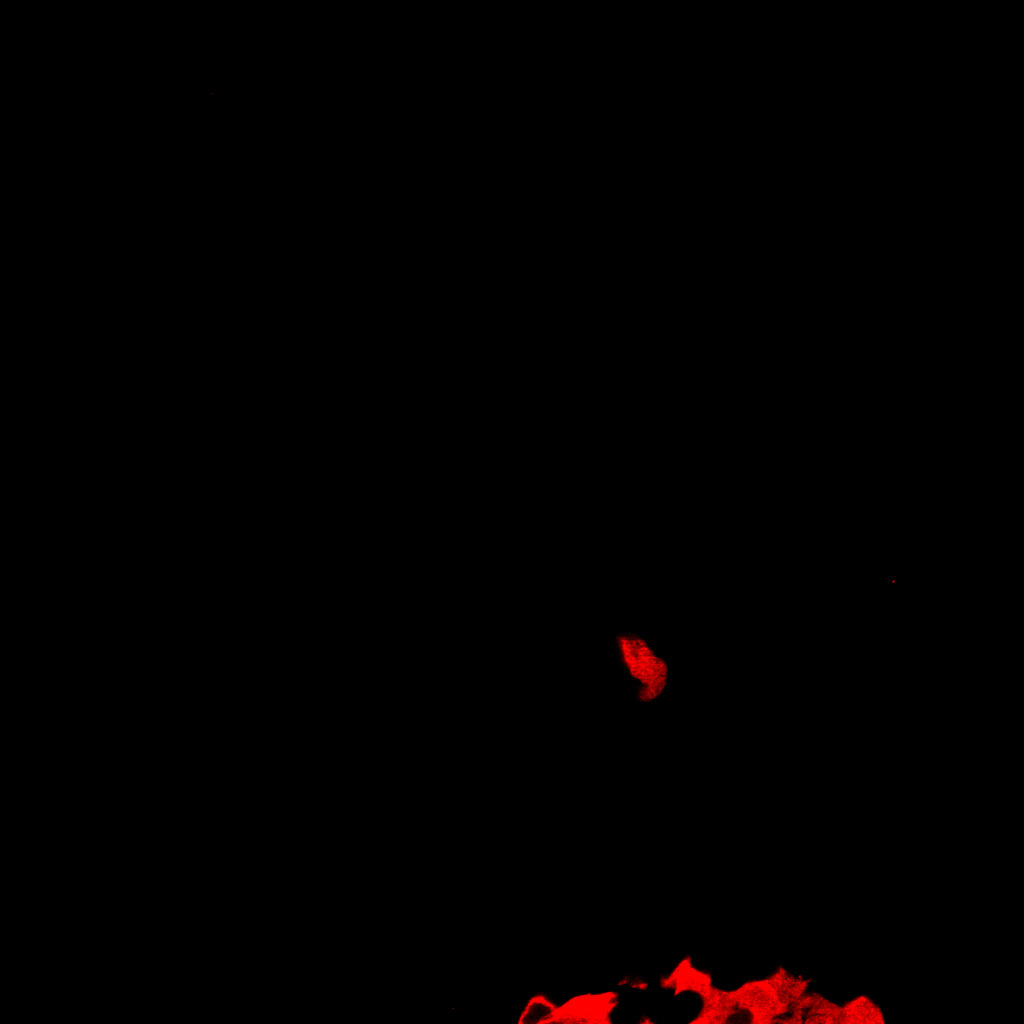

Supplement: Supplementary file 8 — Source data Fig. 6 [file 44318_2025_434_MOESM8_ESM.zip › Figure 6/6H/6H_12w_Ins (red).tif]

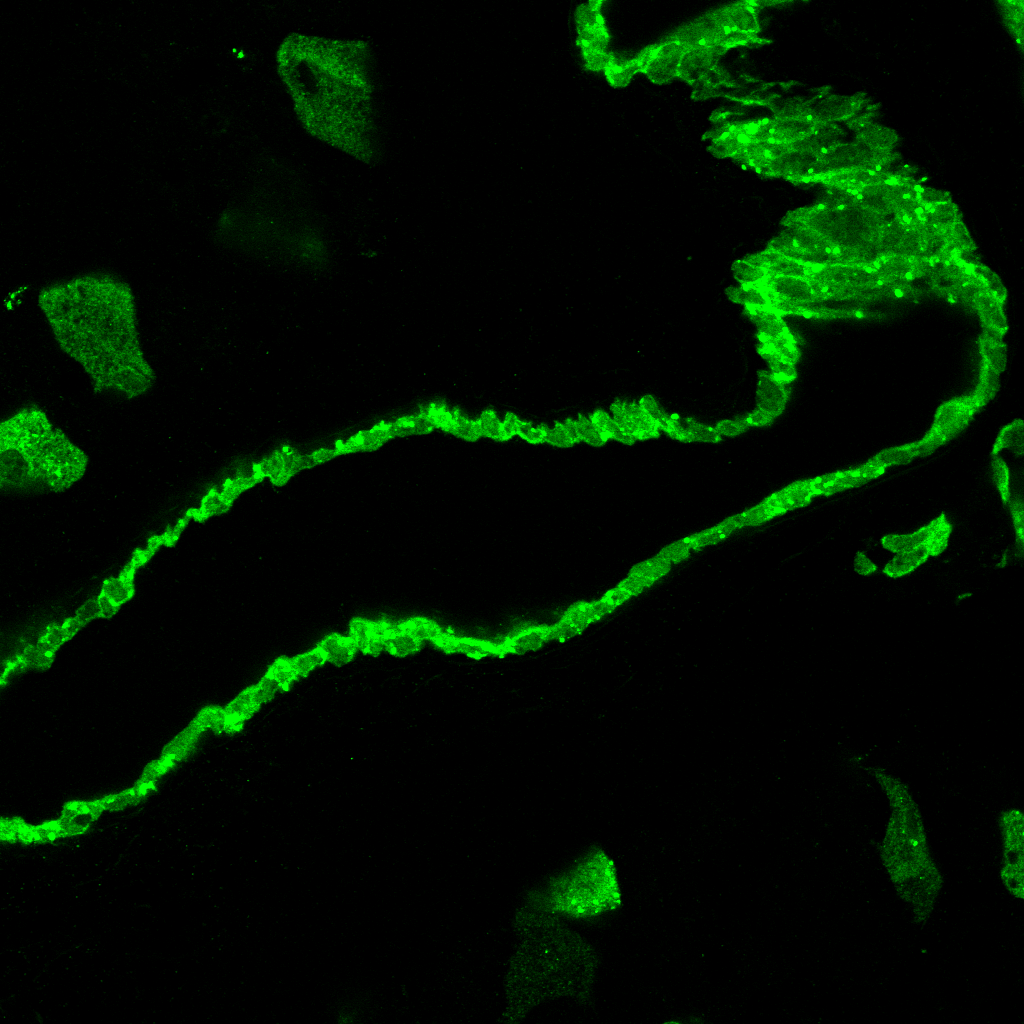

Supplement: Supplementary file 8 — Source data Fig. 6 [file 44318_2025_434_MOESM8_ESM.zip › Figure 6/6H/6H_12w_CK19 (green).tif]

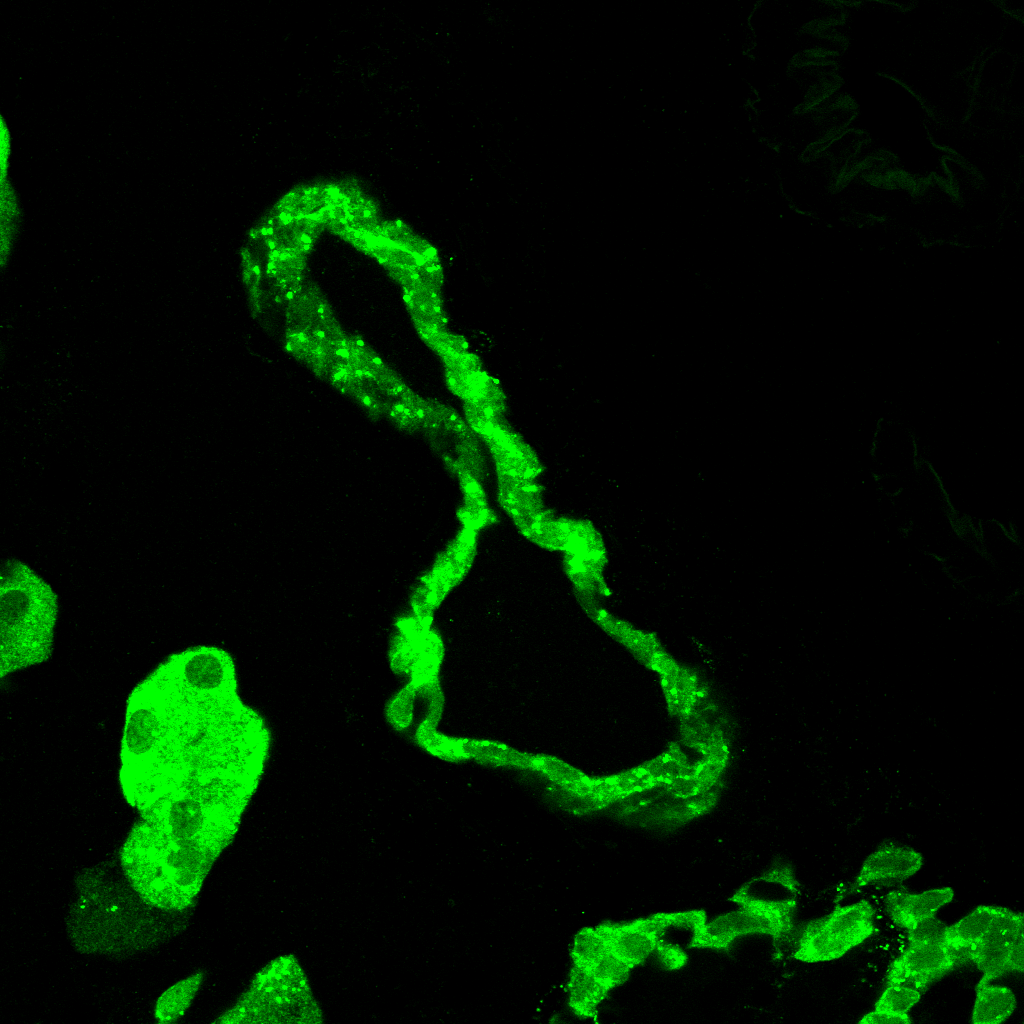

Supplement: Supplementary file 8 — Source data Fig. 6 [file 44318_2025_434_MOESM8_ESM.zip › Figure 6/6H/6H_12w_Sst (green).tif]

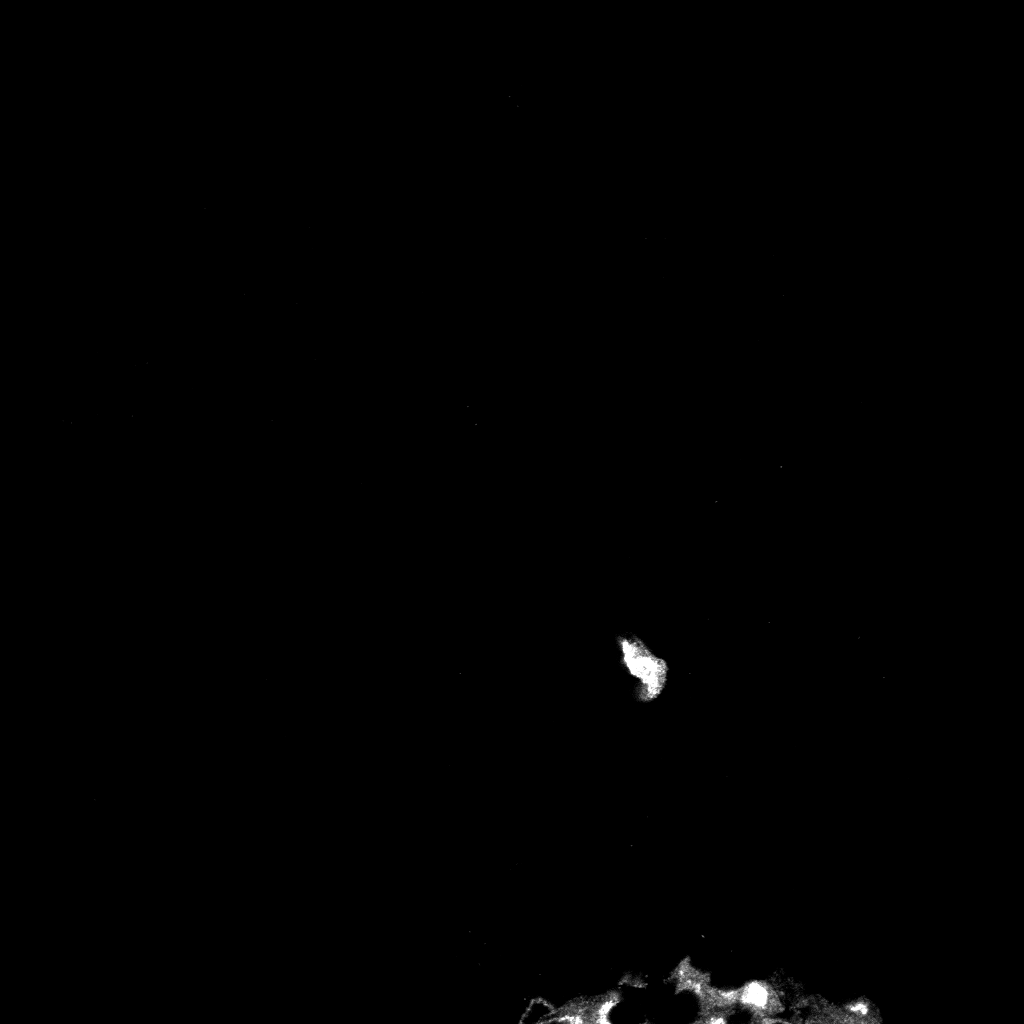

Supplement: Supplementary file 8 — Source data Fig. 6 [file 44318_2025_434_MOESM8_ESM.zip › Figure 6/6H/6H_12w_Ins (gray).tif]

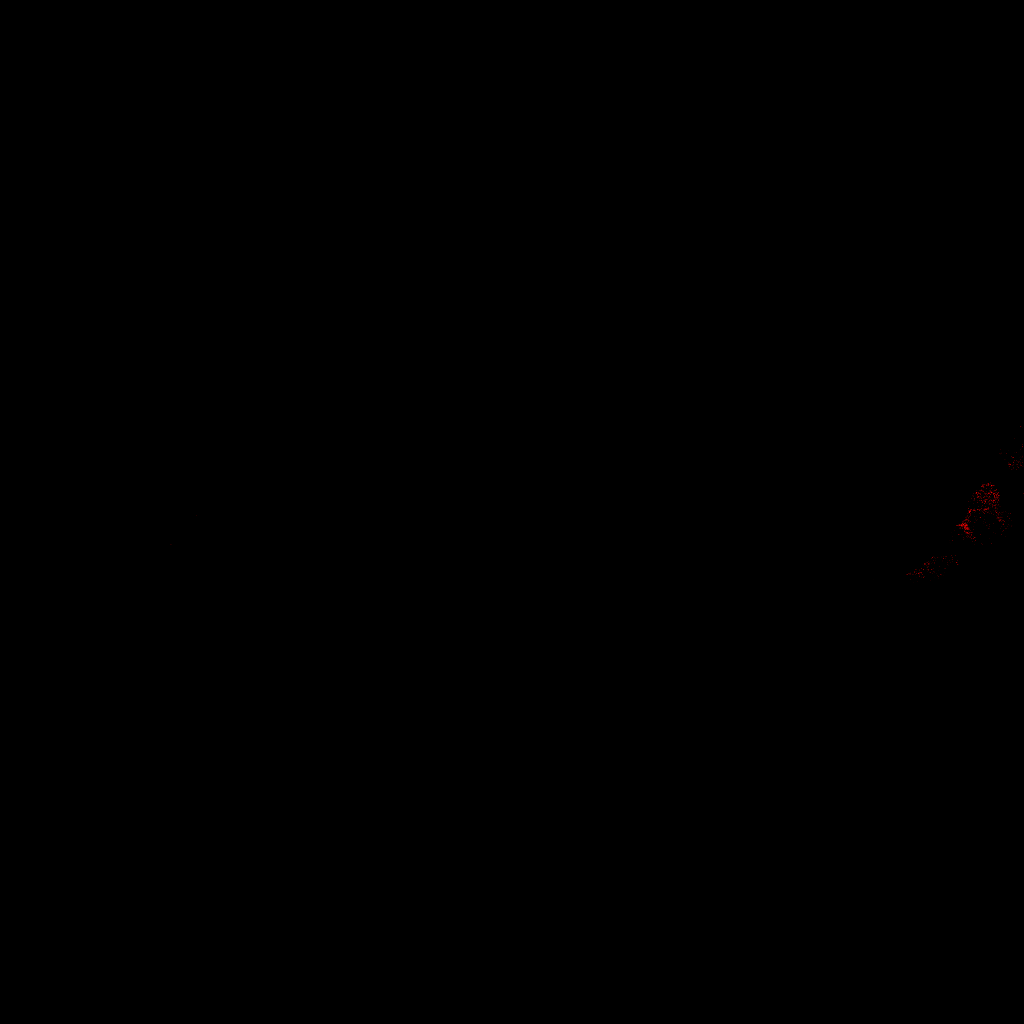

Supplement: Supplementary file 8 — Source data Fig. 6 [file 44318_2025_434_MOESM8_ESM.zip › Figure 6/6H/6H_12w_CK19 (red).tif]

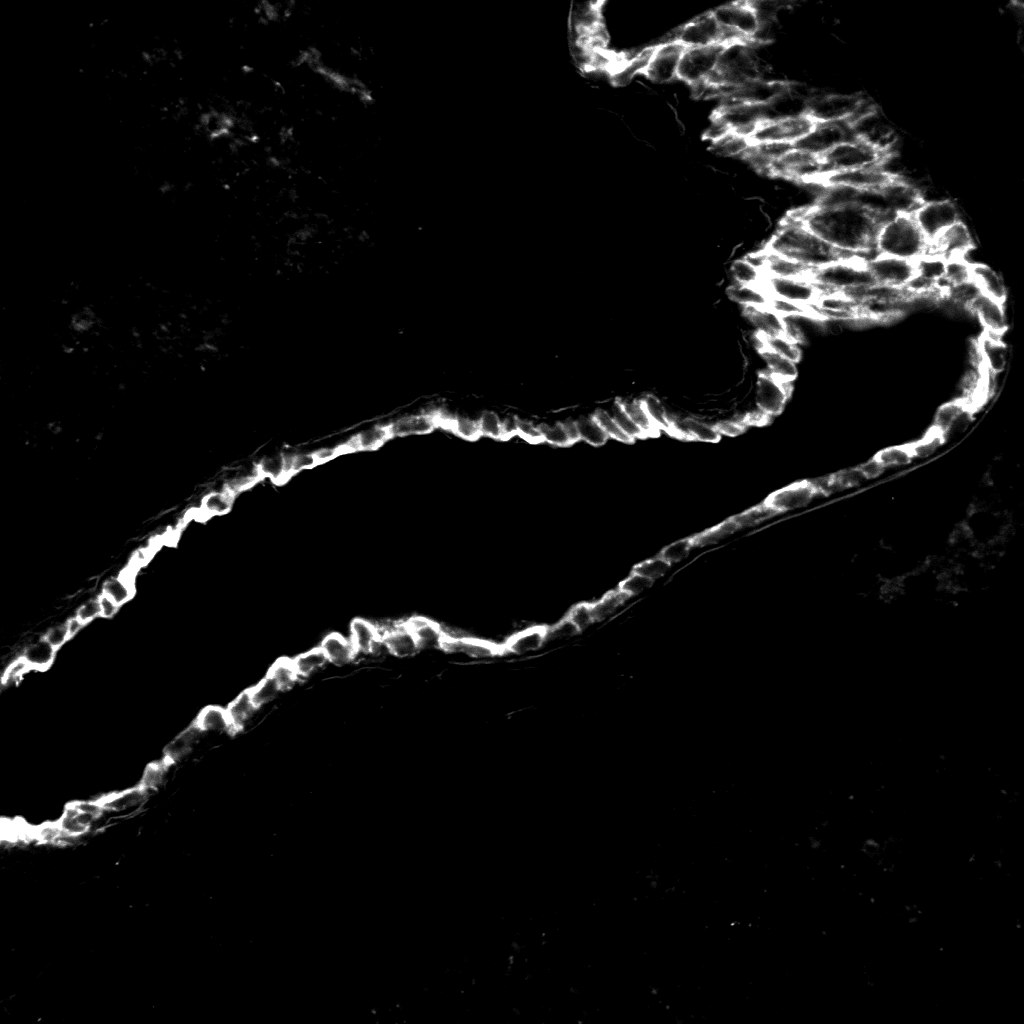

Supplement: Supplementary file 8 — Source data Fig. 6 [file 44318_2025_434_MOESM8_ESM.zip › Figure 6/6H/6H_12w_CK19 (gray).tif]

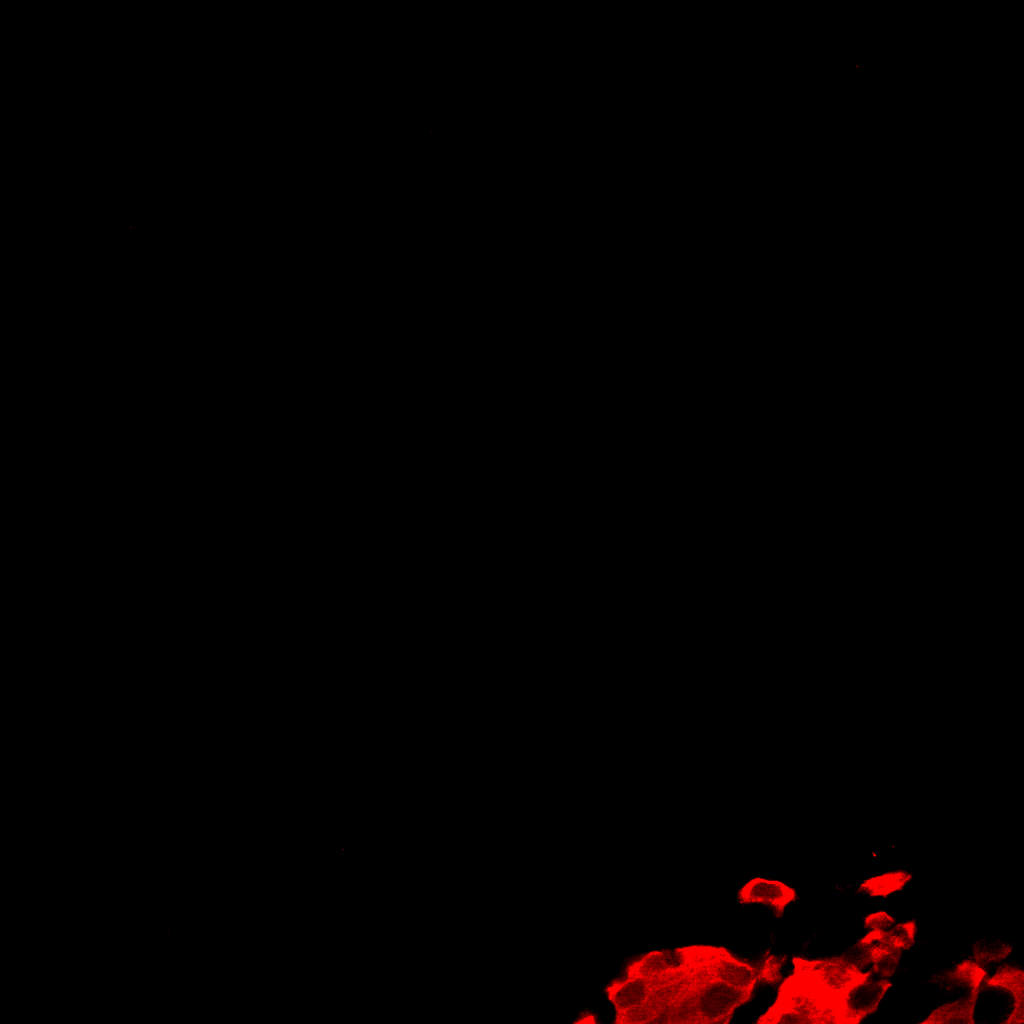

Supplement: Supplementary file 8 — Source data Fig. 6 [file 44318_2025_434_MOESM8_ESM.zip › Figure 6/6H/6H_12w_Sst (red).tif]

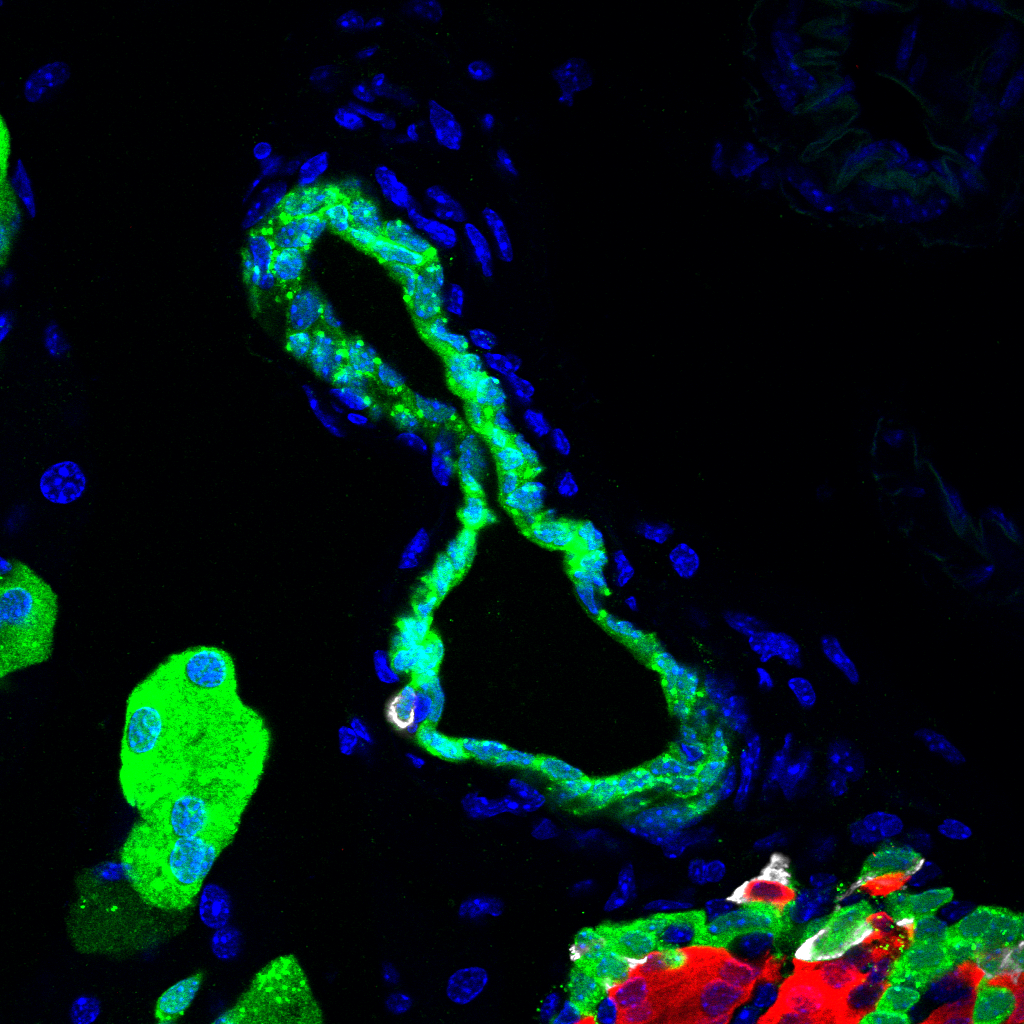

Supplement: Supplementary file 8 — Source data Fig. 6 [file 44318_2025_434_MOESM8_ESM.zip › Figure 6/6H/6H_12w_Sst.tif]

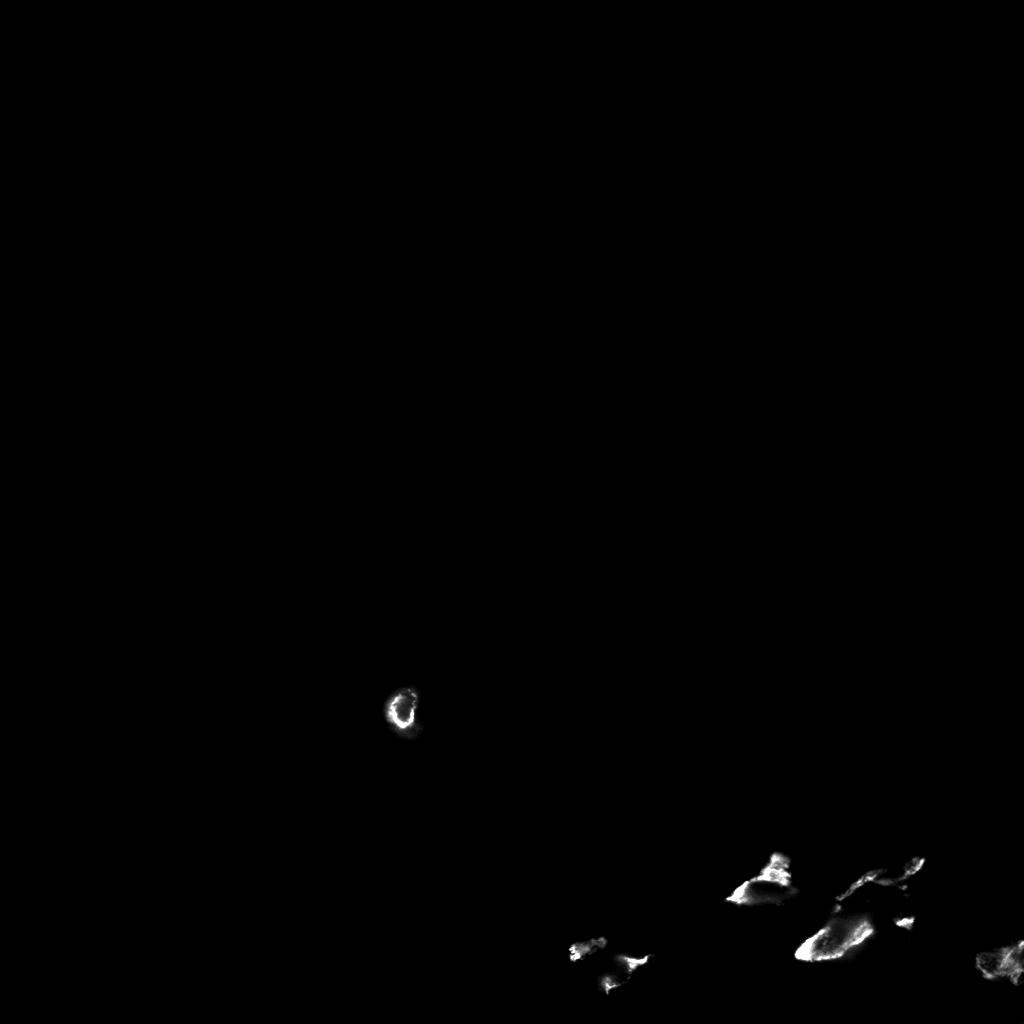

Supplement: Supplementary file 8 — Source data Fig. 6 [file 44318_2025_434_MOESM8_ESM.zip › Figure 6/6H/6H_12w_Sst (gray).tif]

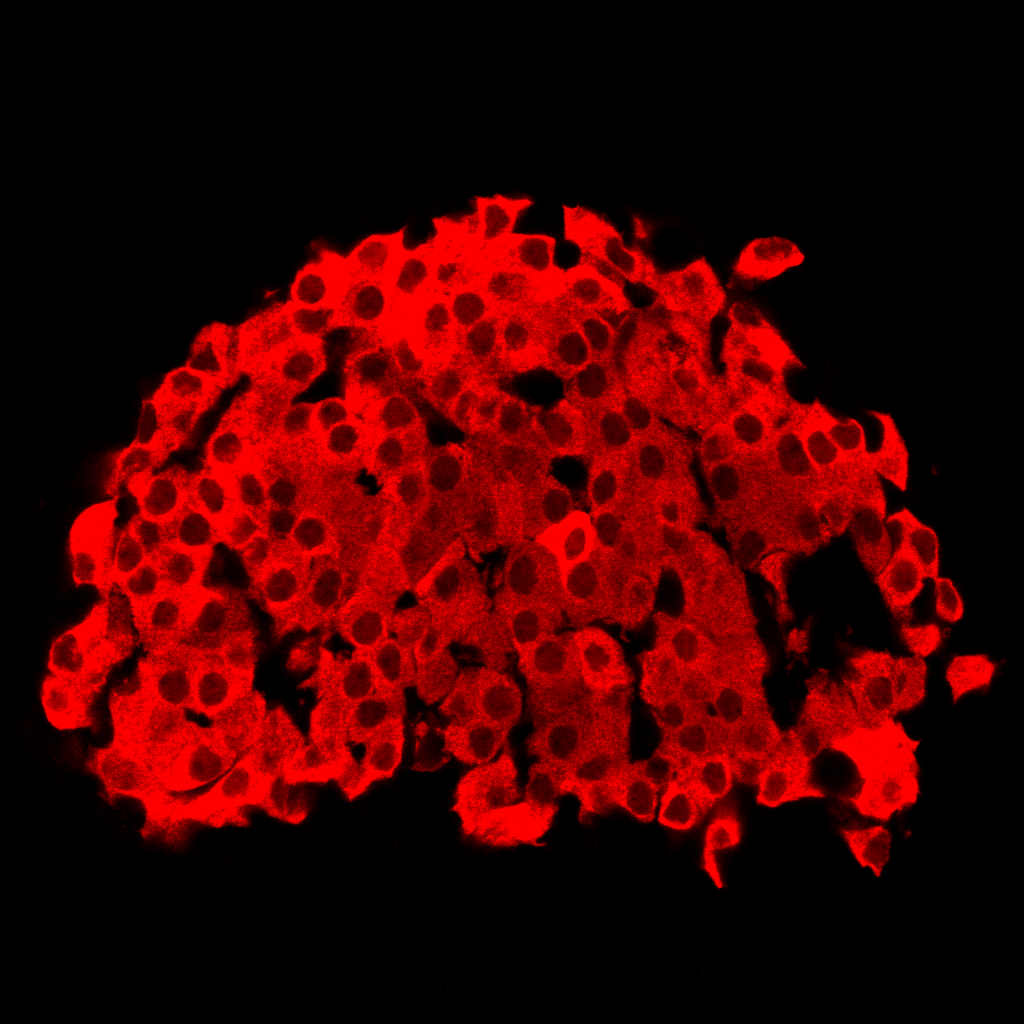

Supplement: Supplementary file 8 — Source data Fig. 6 [file 44318_2025_434_MOESM8_ESM.zip › Figure 6/6I/6I_Merge (red).tif]

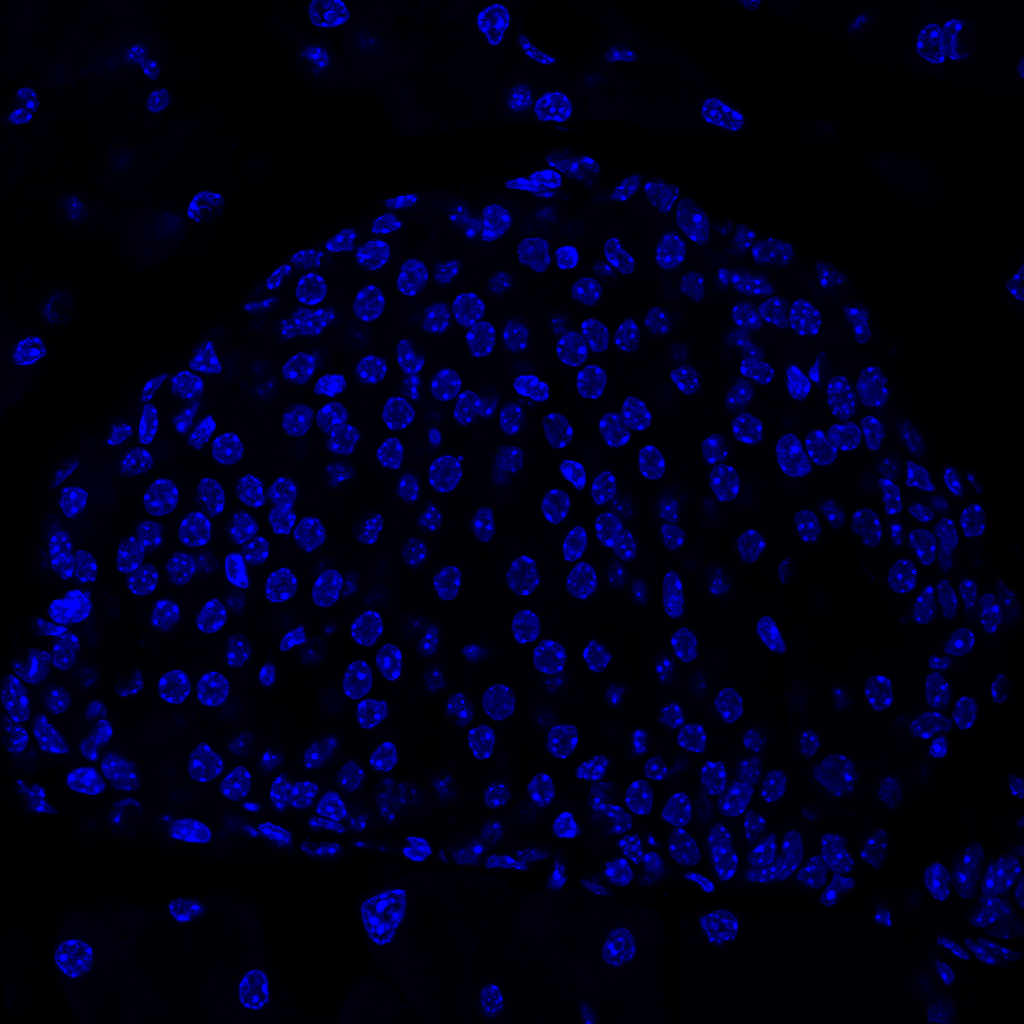

Supplement: Supplementary file 8 — Source data Fig. 6 [file 44318_2025_434_MOESM8_ESM.zip › Figure 6/6I/6I_Merge (blue).tif]

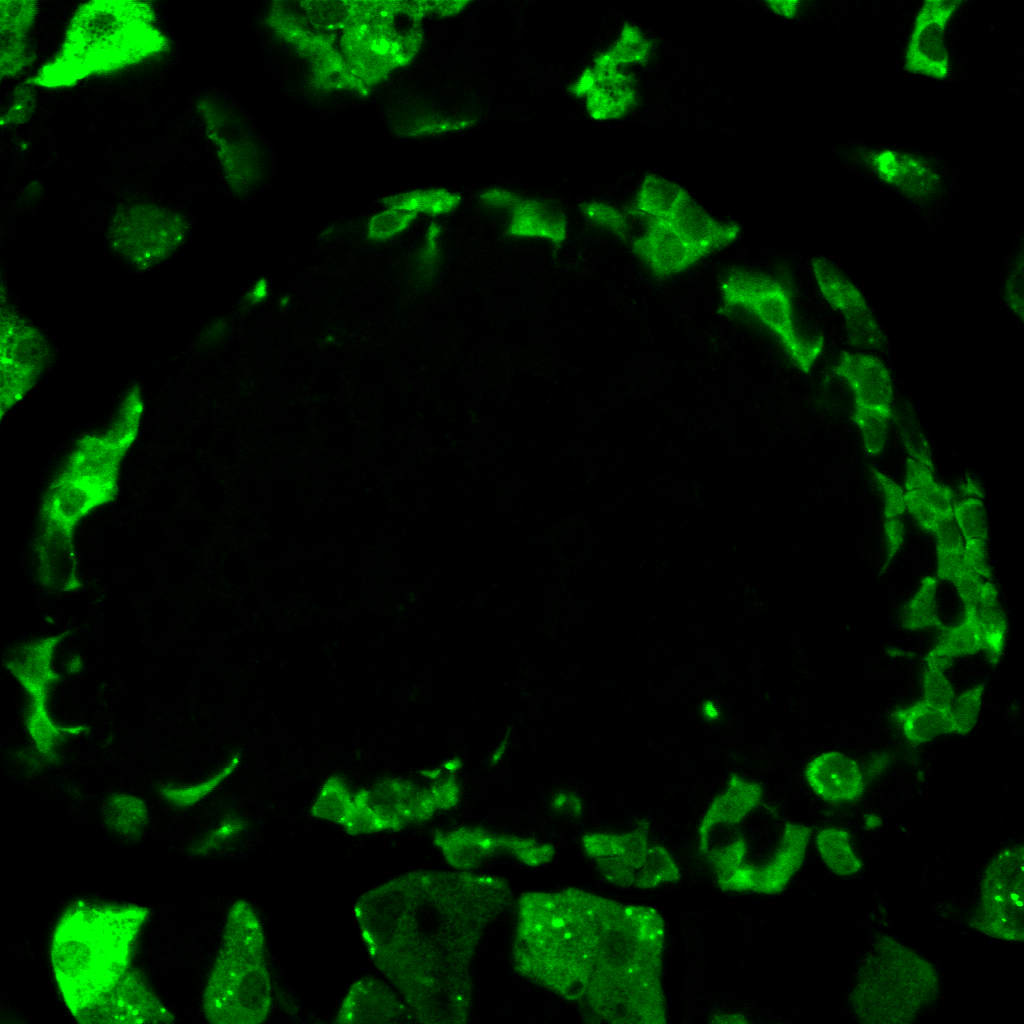

Supplement: Supplementary file 8 — Source data Fig. 6 [file 44318_2025_434_MOESM8_ESM.zip › Figure 6/6I/6I_Merge (green).tif]

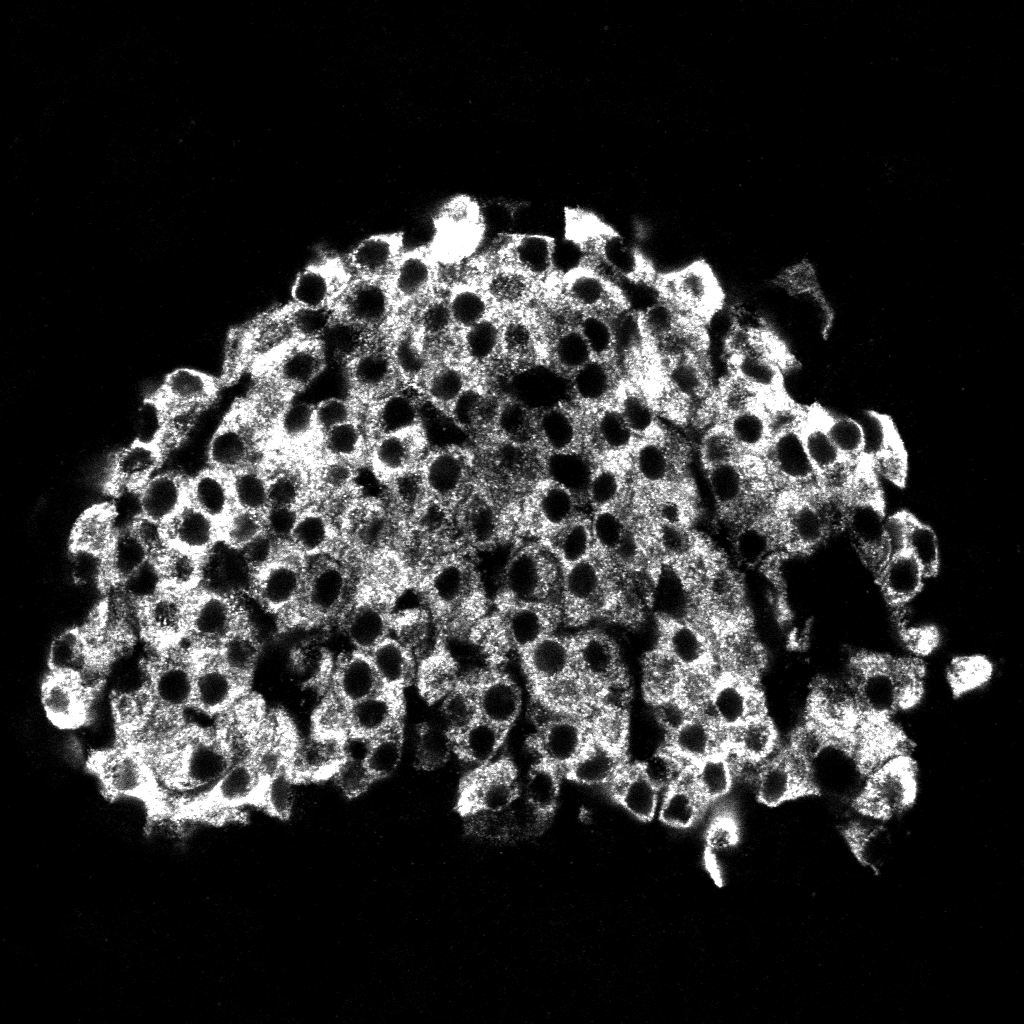

Supplement: Supplementary file 8 — Source data Fig. 6 [file 44318_2025_434_MOESM8_ESM.zip › Figure 6/6I/6I_Merge (gray).tif]

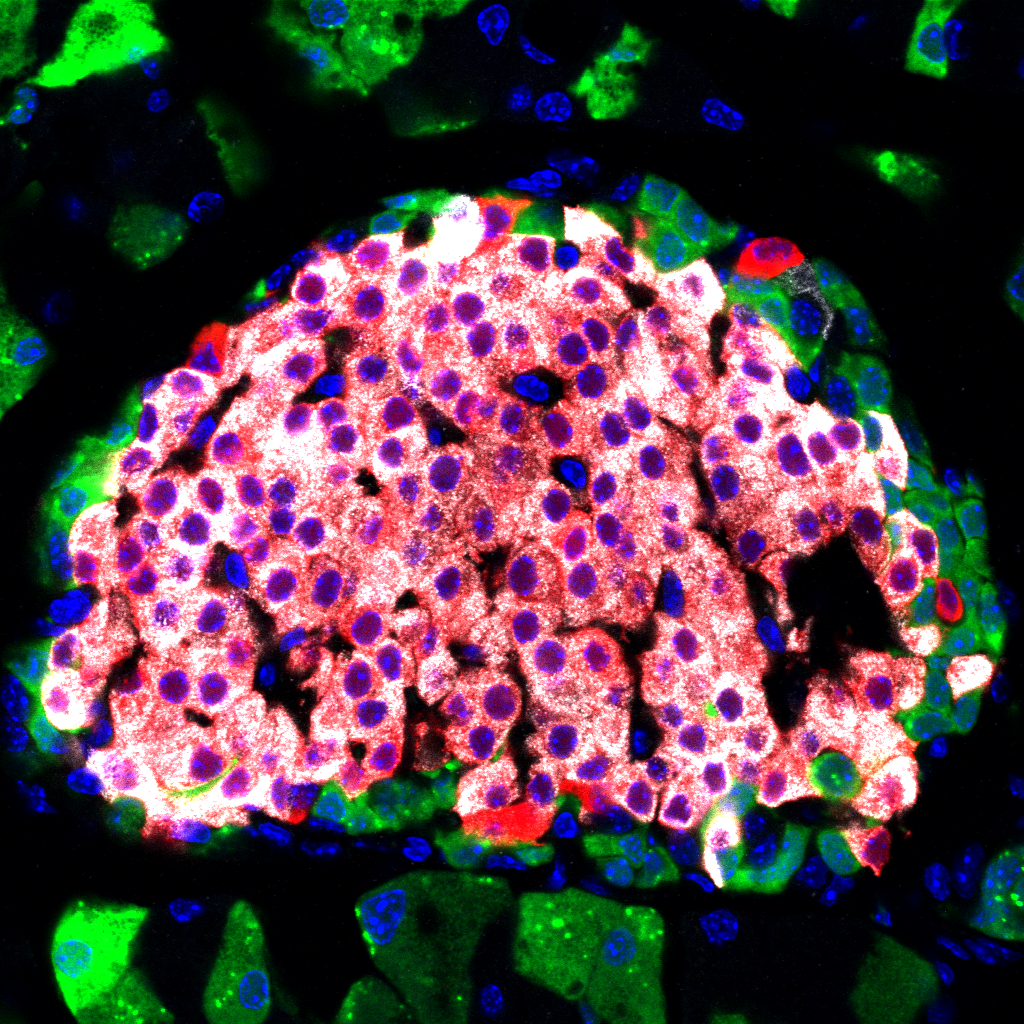

Supplement: Supplementary file 8 — Source data Fig. 6 [file 44318_2025_434_MOESM8_ESM.zip › Figure 6/6I/6I_Merge.tif]

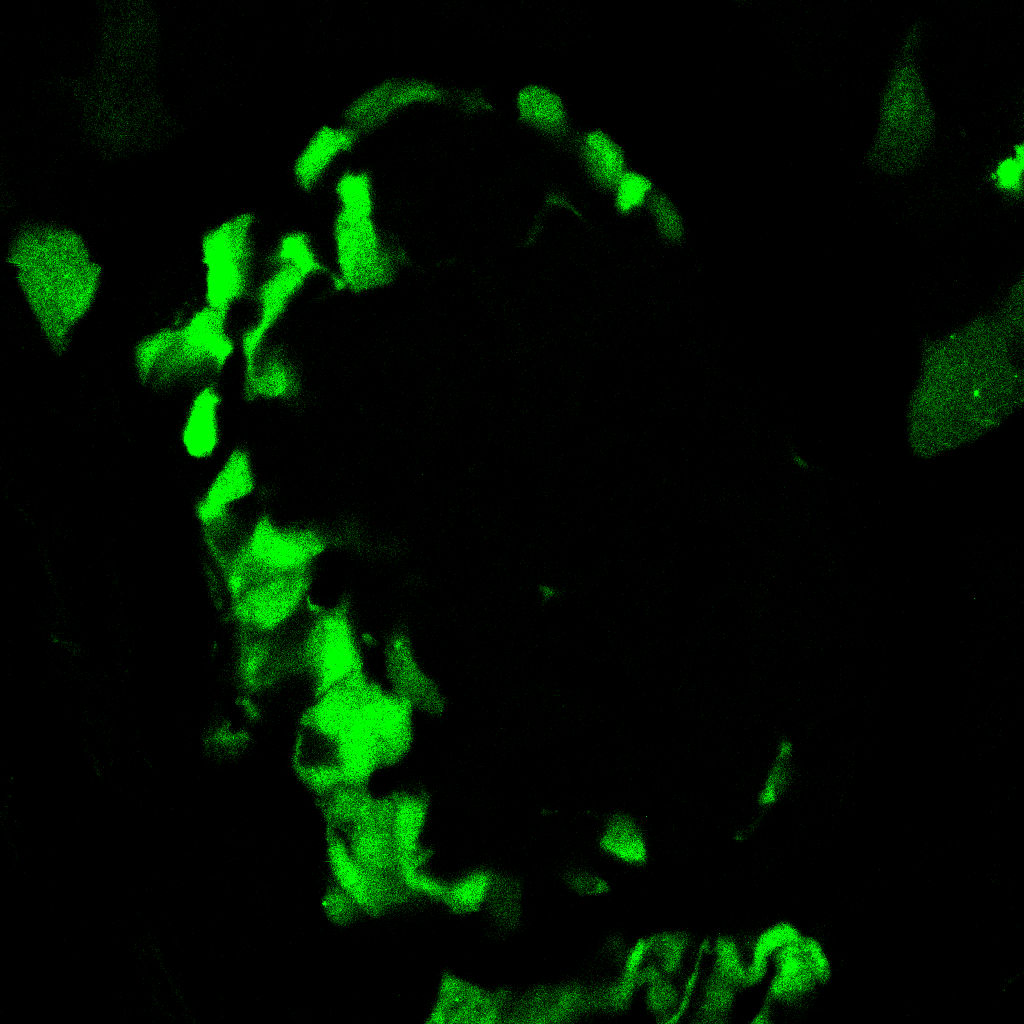

Supplement: Supplementary file 8 — Source data Fig. 6 [file 44318_2025_434_MOESM8_ESM.zip › Figure 6/6G/6G_Merge (green).tif]

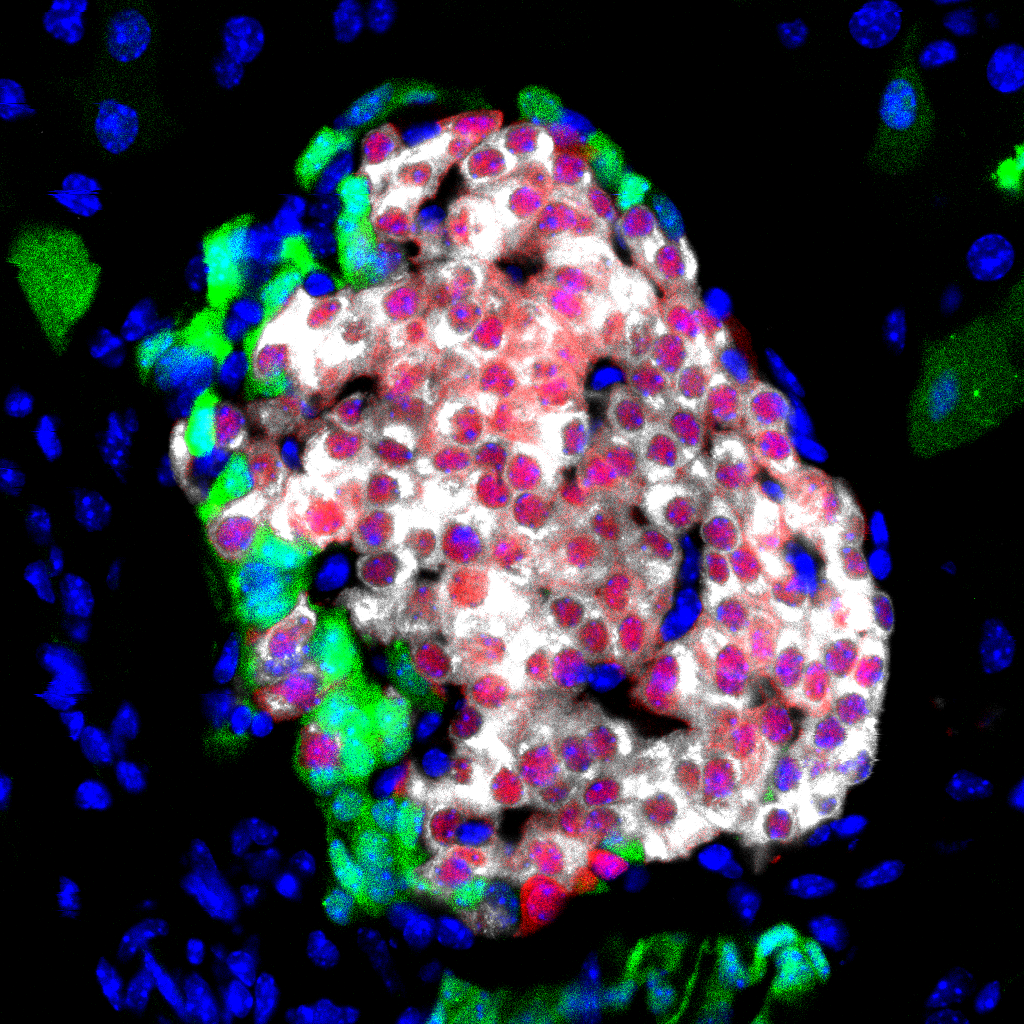

Supplement: Supplementary file 8 — Source data Fig. 6 [file 44318_2025_434_MOESM8_ESM.zip › Figure 6/6G/6G_Merge.tif]

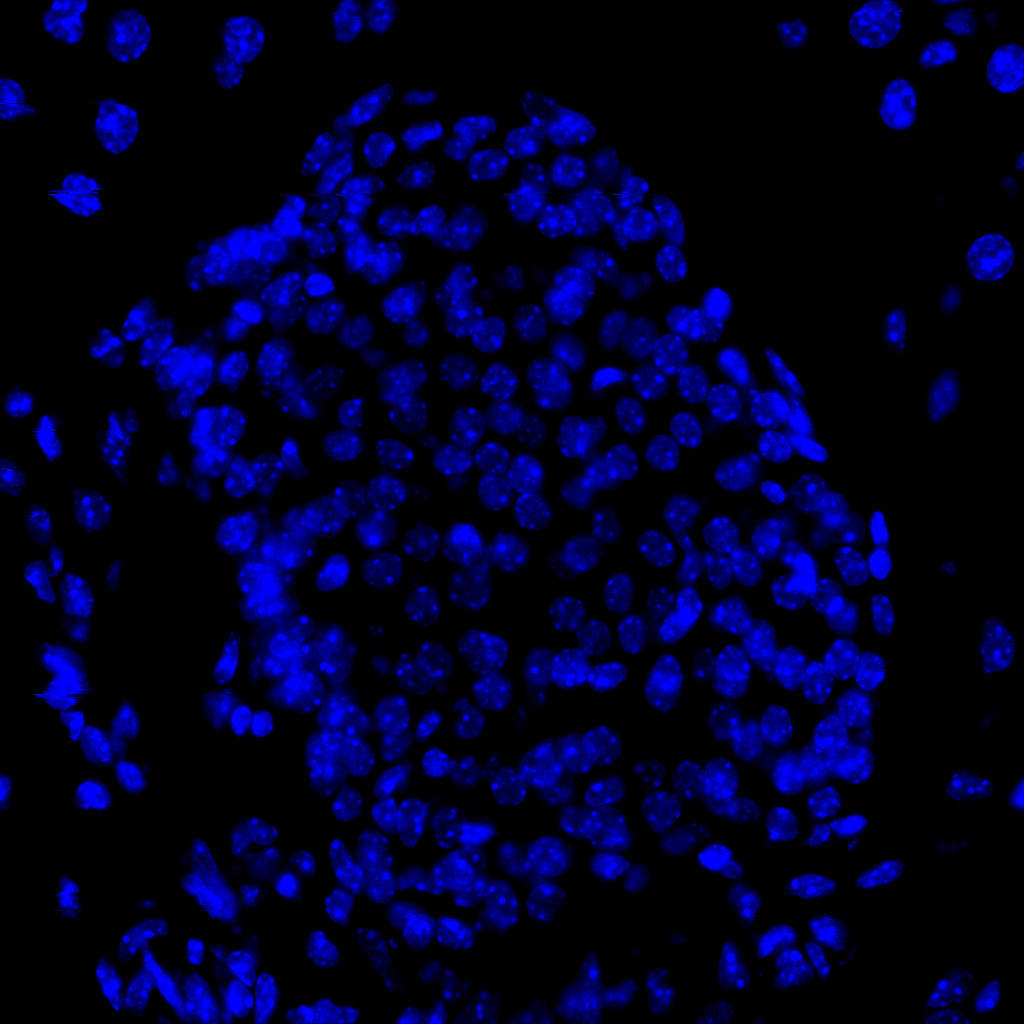

Supplement: Supplementary file 8 — Source data Fig. 6 [file 44318_2025_434_MOESM8_ESM.zip › Figure 6/6G/6G_Merge (blue).tif]

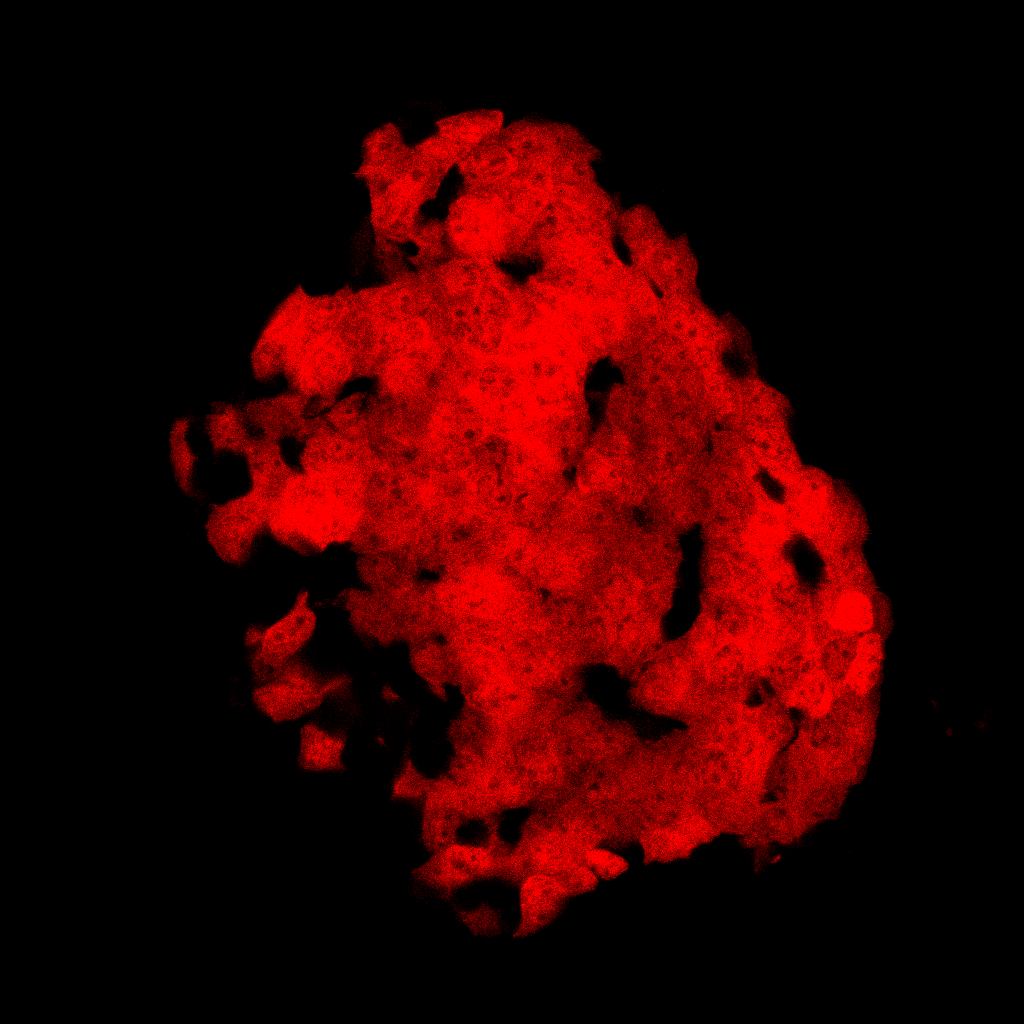

Supplement: Supplementary file 8 — Source data Fig. 6 [file 44318_2025_434_MOESM8_ESM.zip › Figure 6/6G/6G_Merge (red).tif]

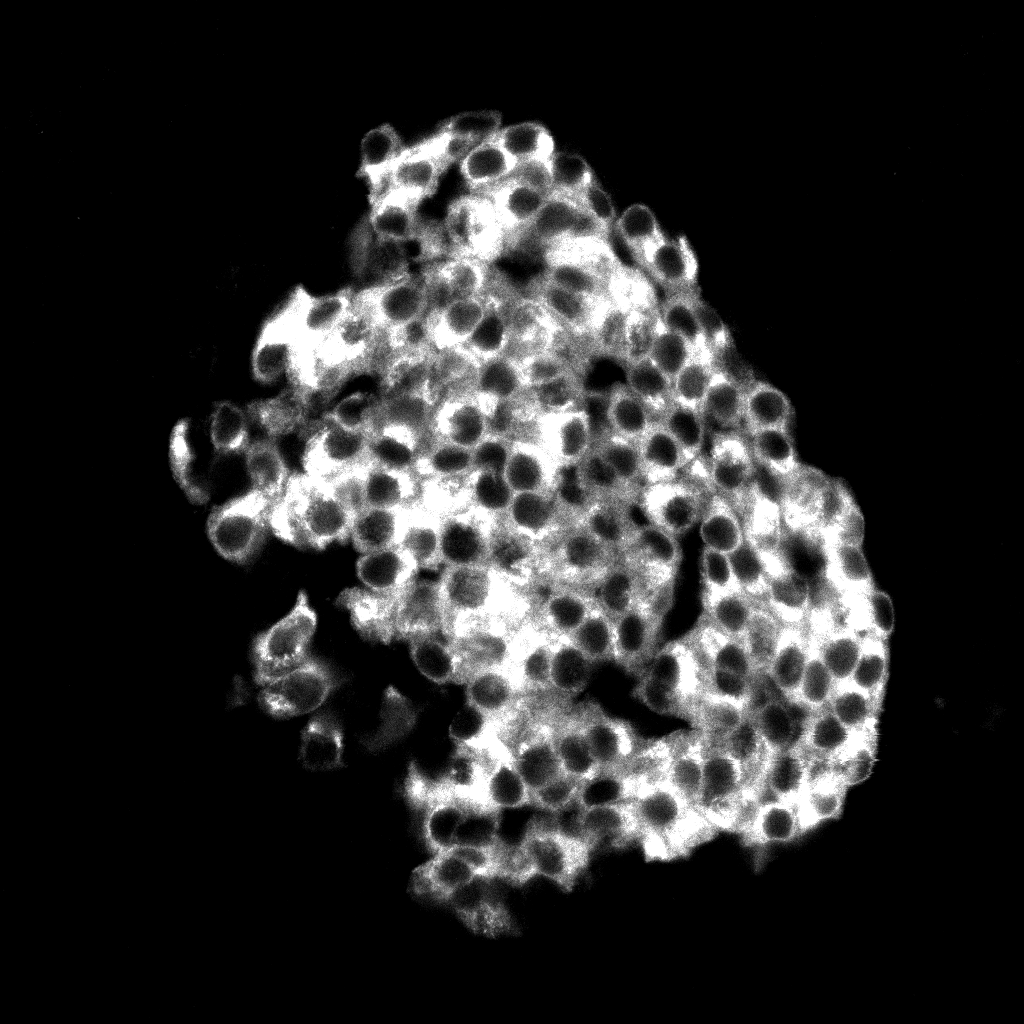

Supplement: Supplementary file 8 — Source data Fig. 6 [file 44318_2025_434_MOESM8_ESM.zip › Figure 6/6G/6G_Merge (gray).tif]

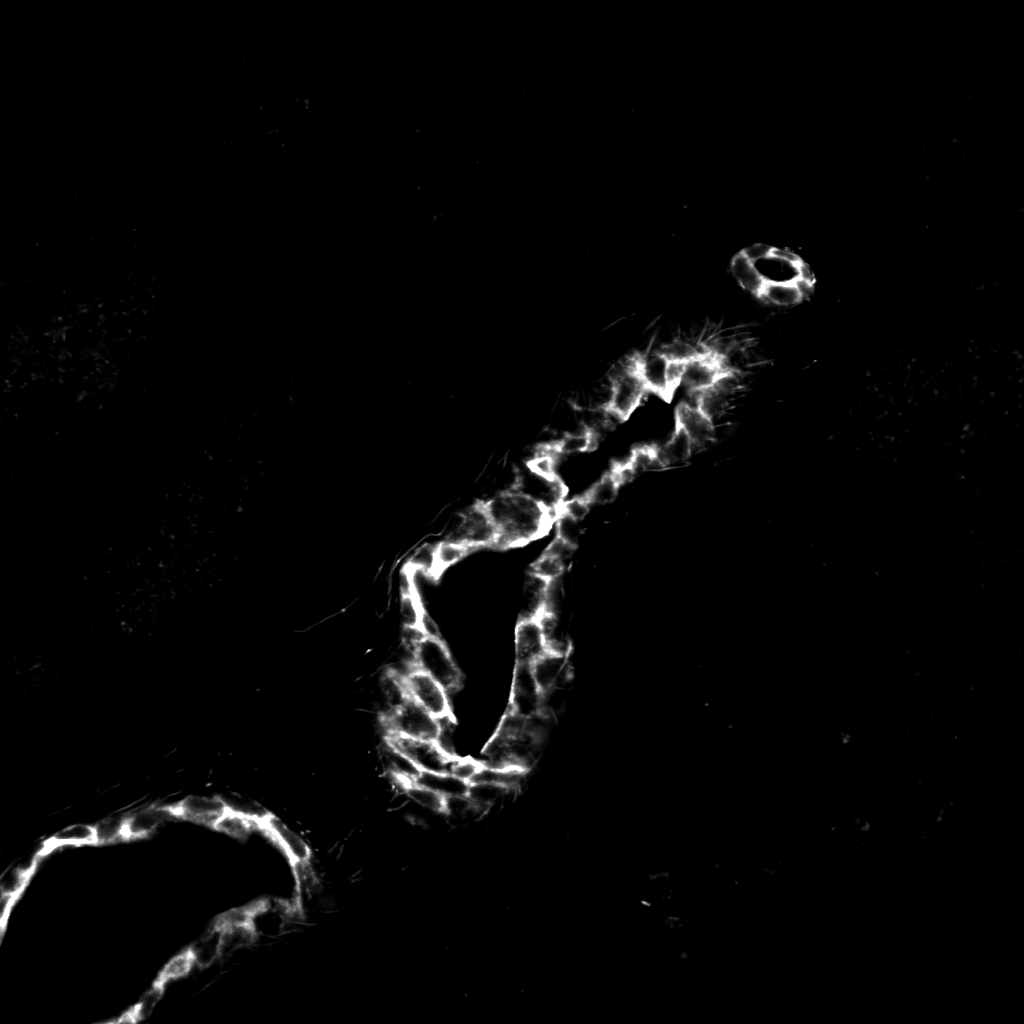

Supplement: Supplementary file 8 — Source data Fig. 6 [file 44318_2025_434_MOESM8_ESM.zip › Figure 6/6D/6D_Merge_mag_2 (gray).tif]

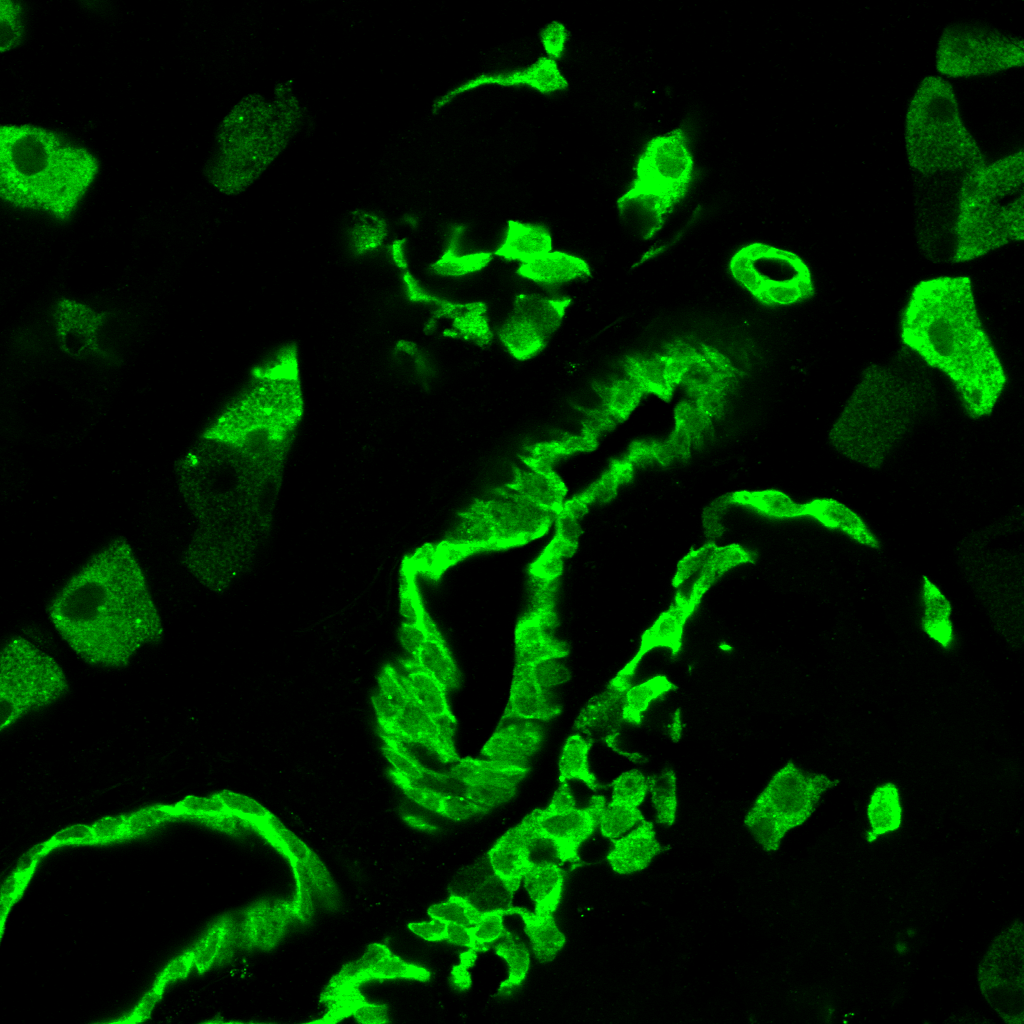

Supplement: Supplementary file 8 — Source data Fig. 6 [file 44318_2025_434_MOESM8_ESM.zip › Figure 6/6D/6D_Merge_mag_2 (green).tif]

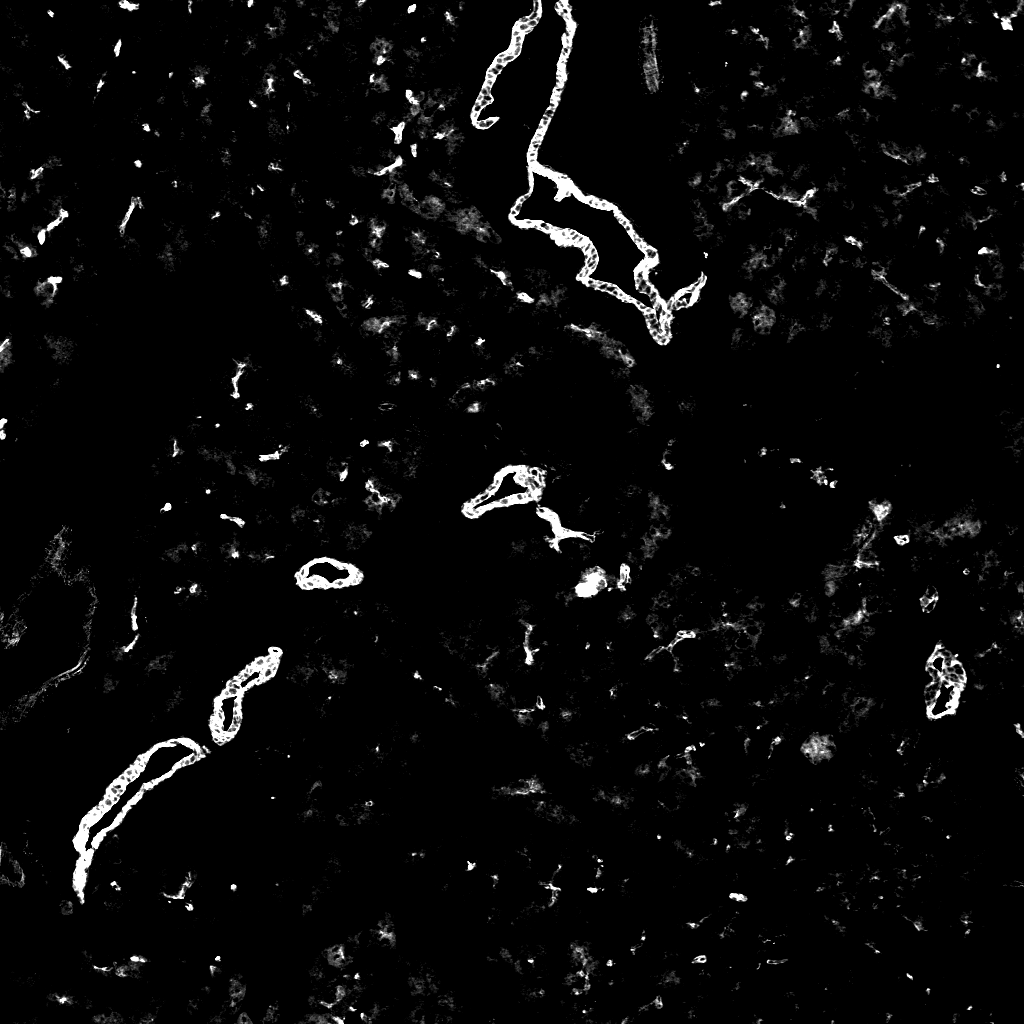

Supplement: Supplementary file 8 — Source data Fig. 6 [file 44318_2025_434_MOESM8_ESM.zip › Figure 6/6D/6D_Merge (gray).tif]

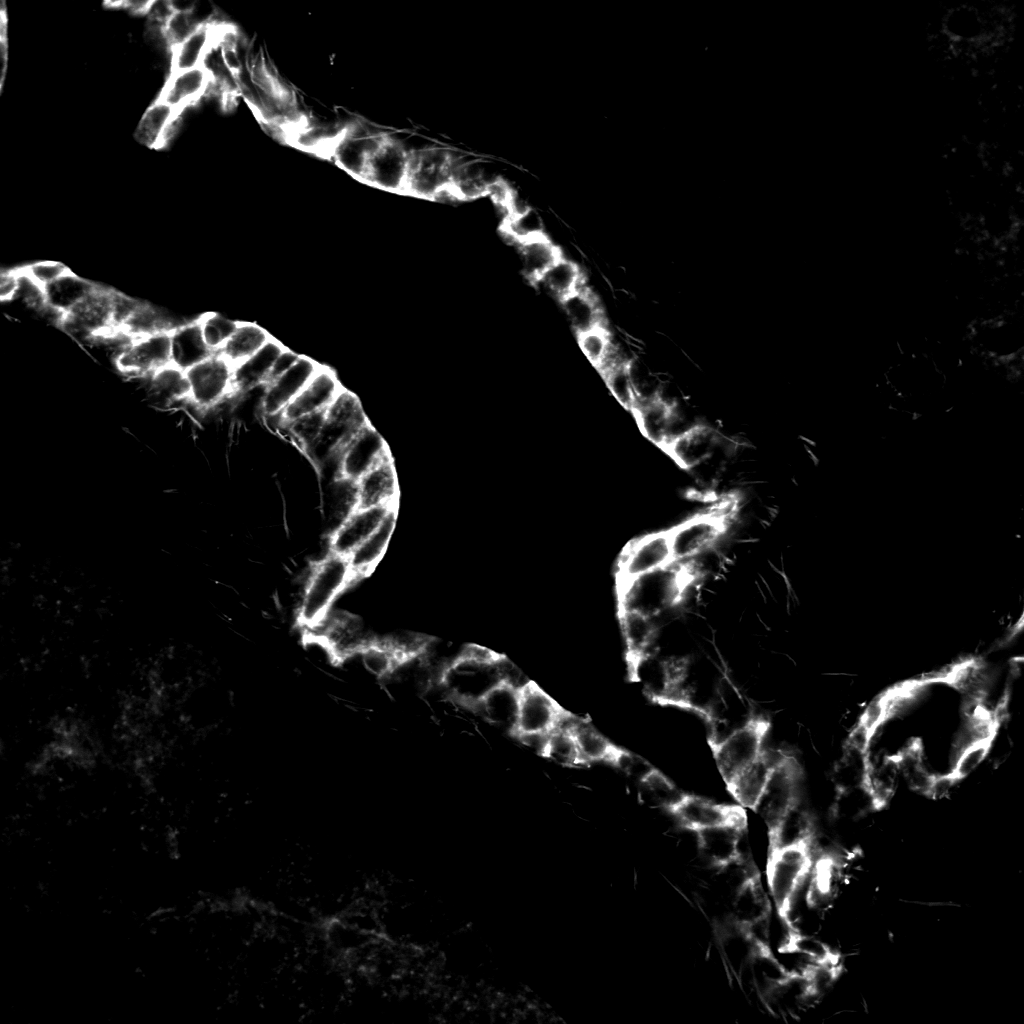

Supplement: Supplementary file 8 — Source data Fig. 6 [file 44318_2025_434_MOESM8_ESM.zip › Figure 6/6D/6D_Merge_mag_1.oib (gray).tif]

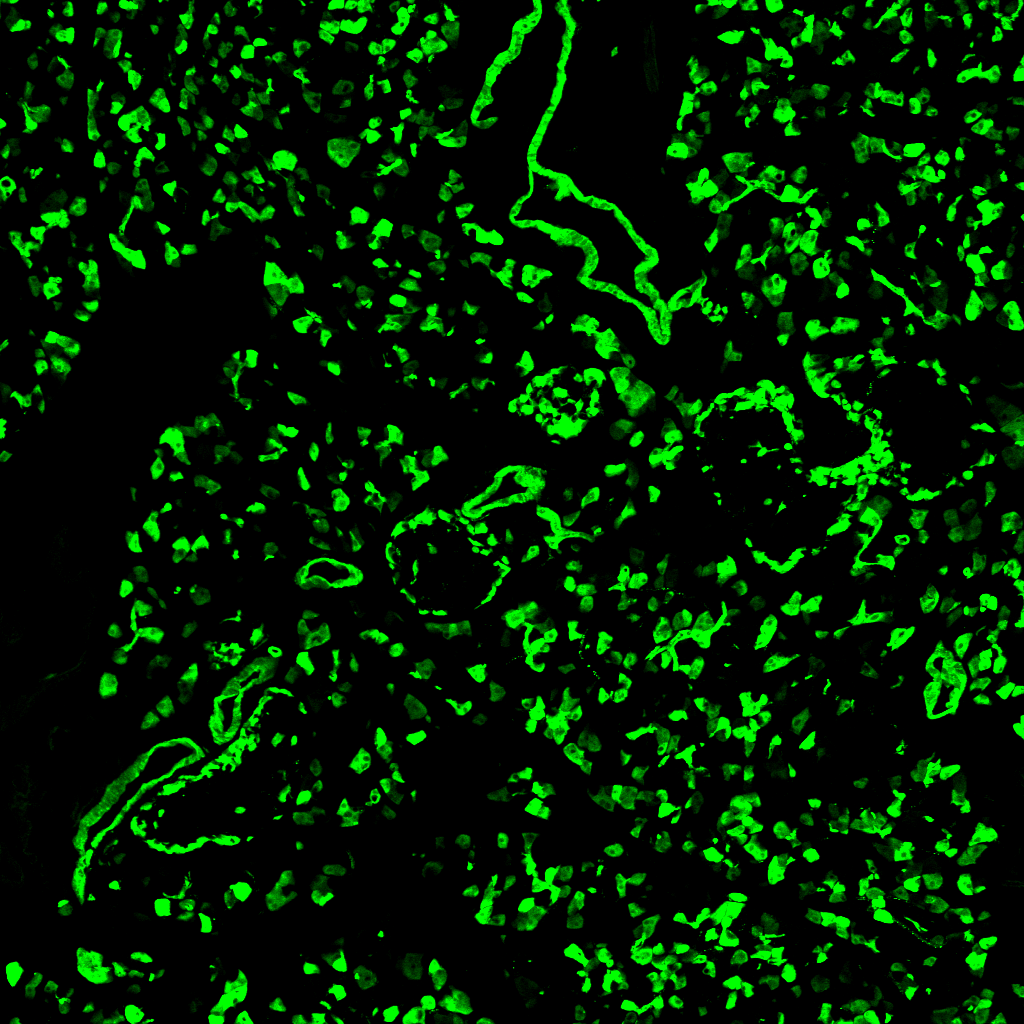

Supplement: Supplementary file 8 — Source data Fig. 6 [file 44318_2025_434_MOESM8_ESM.zip › Figure 6/6D/6D_Merge (green).tif]

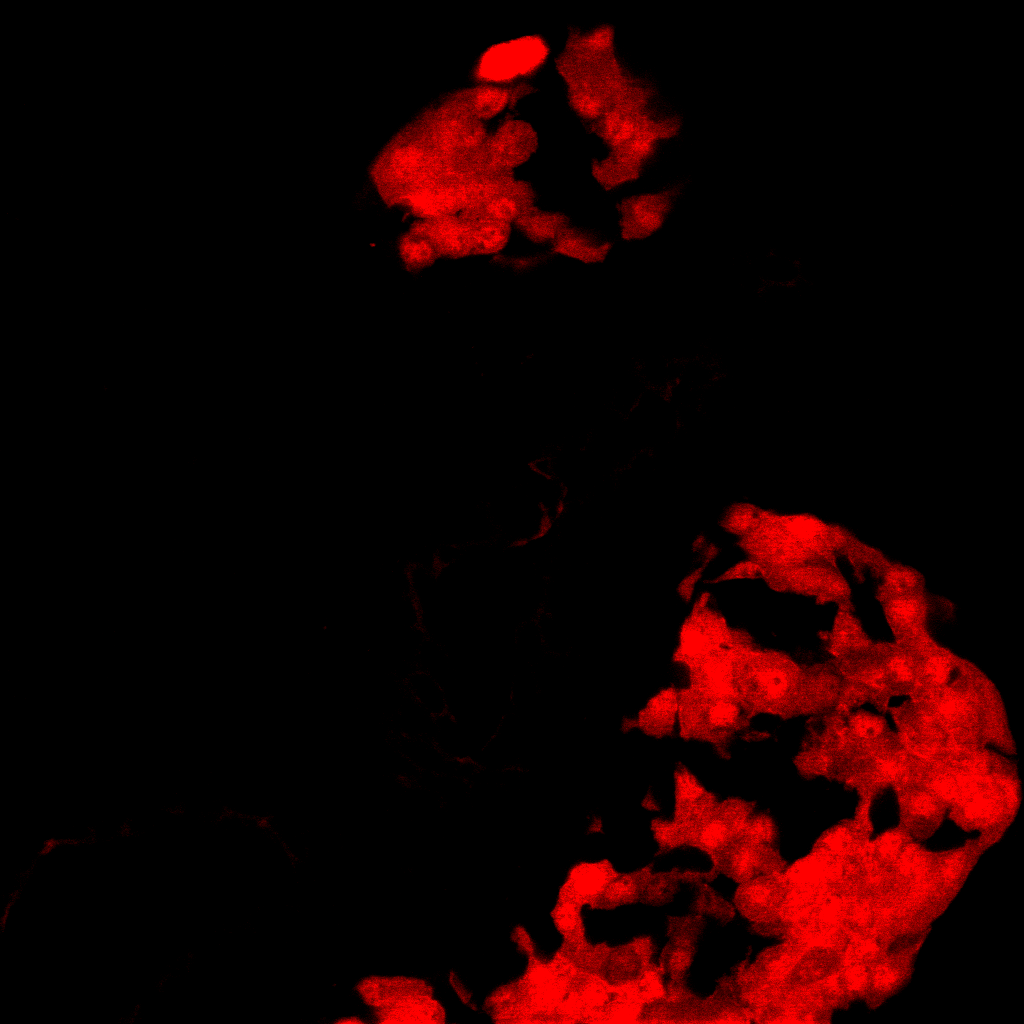

Supplement: Supplementary file 8 — Source data Fig. 6 [file 44318_2025_434_MOESM8_ESM.zip › Figure 6/6D/6D_Merge_mag_2 (red).tif]

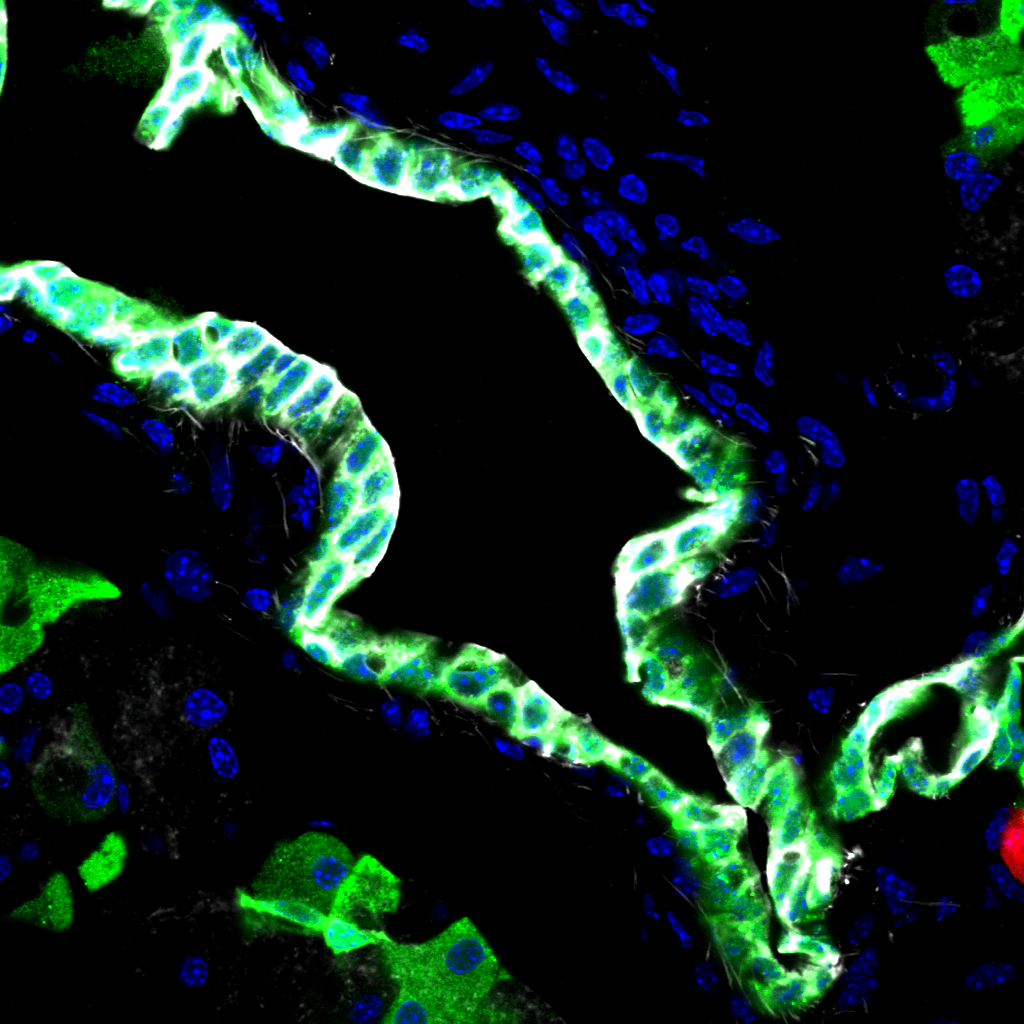

Supplement: Supplementary file 8 — Source data Fig. 6 [file 44318_2025_434_MOESM8_ESM.zip › Figure 6/6D/6D_Merge_mag_1.tif]

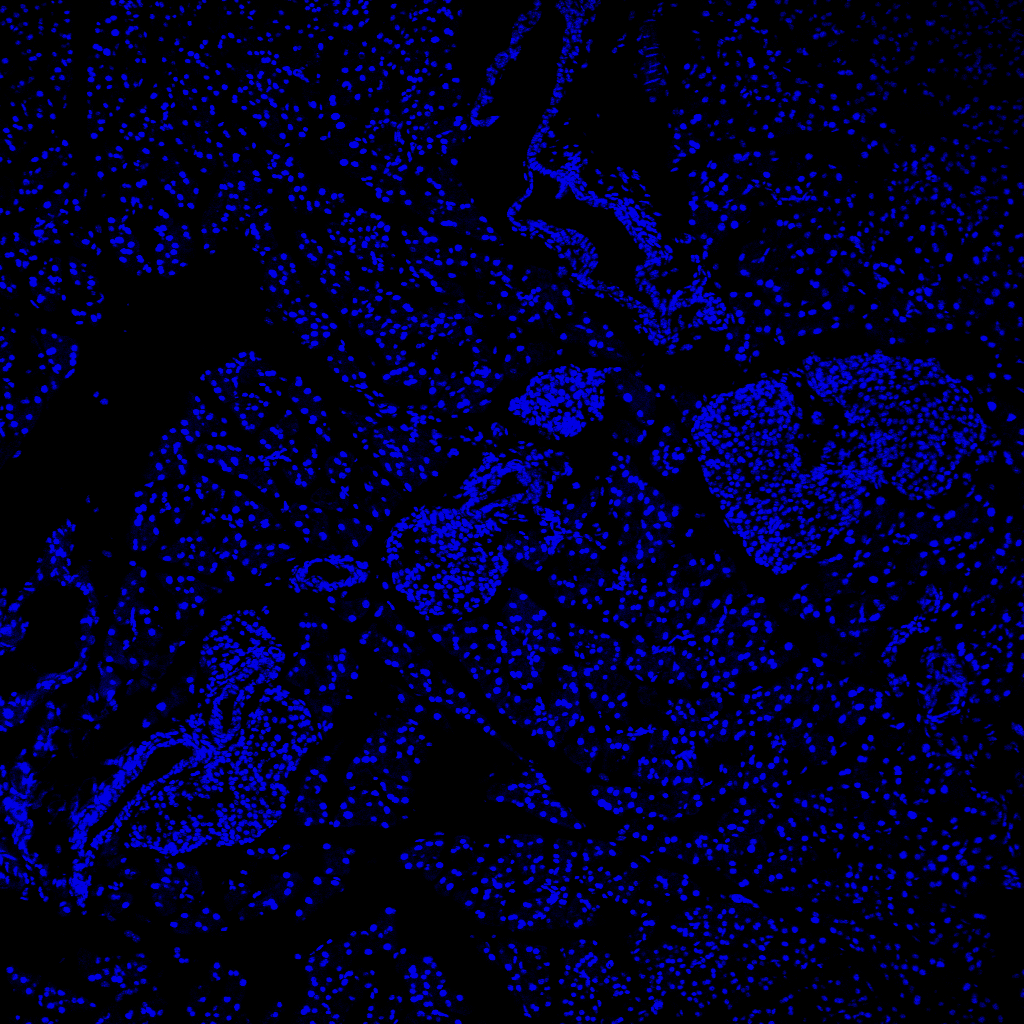

Supplement: Supplementary file 8 — Source data Fig. 6 [file 44318_2025_434_MOESM8_ESM.zip › Figure 6/6D/6D_Merge (blue).tif]

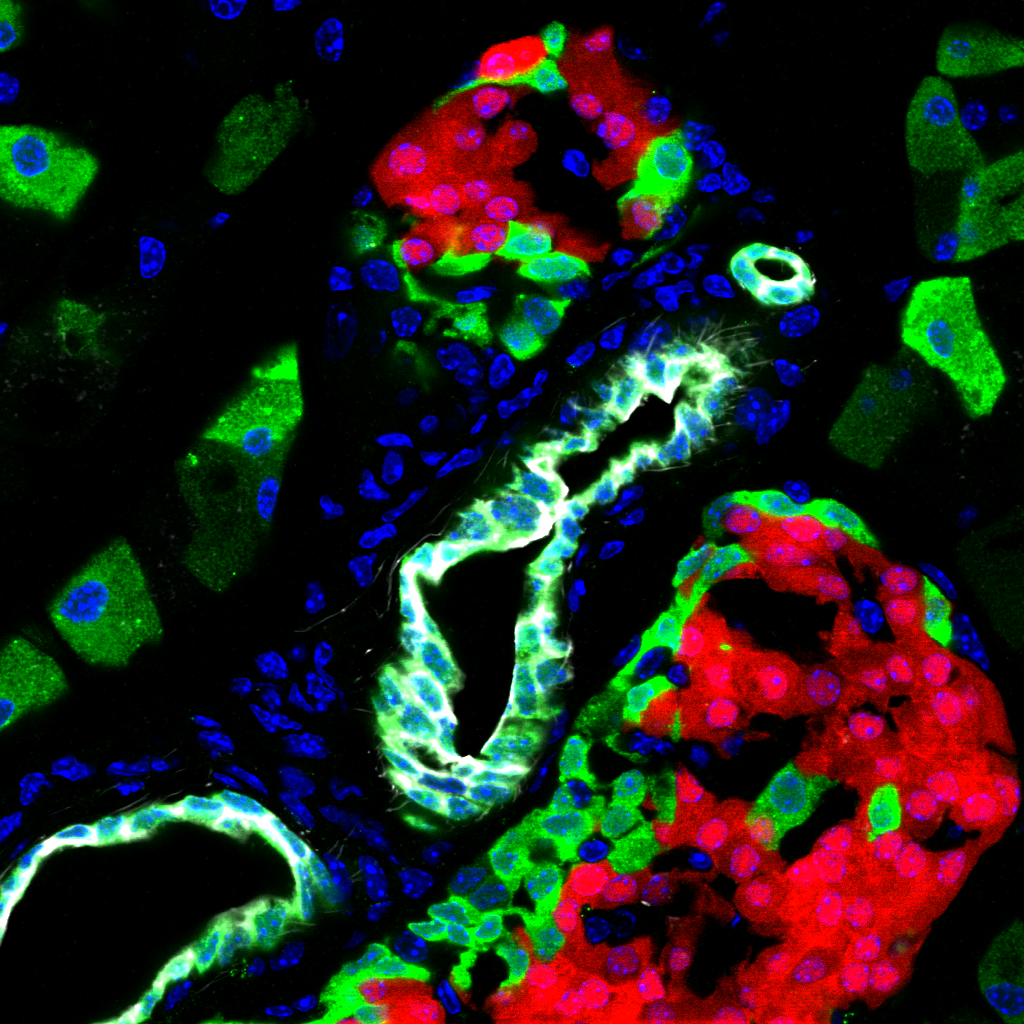

Supplement: Supplementary file 8 — Source data Fig. 6 [file 44318_2025_434_MOESM8_ESM.zip › Figure 6/6D/6D_Merge_mag_2.tif]

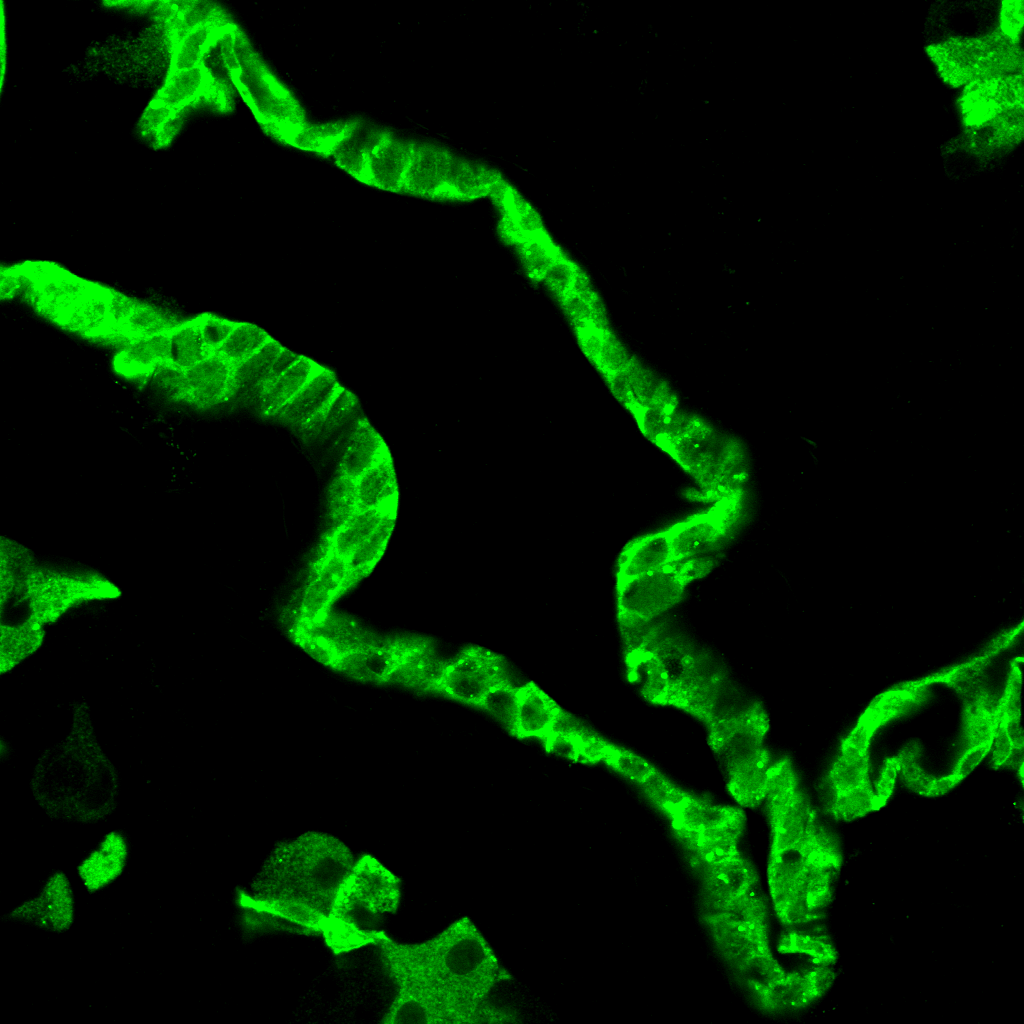

Supplement: Supplementary file 8 — Source data Fig. 6 [file 44318_2025_434_MOESM8_ESM.zip › Figure 6/6D/6D_Merge_mag_1.oib (green).tif]

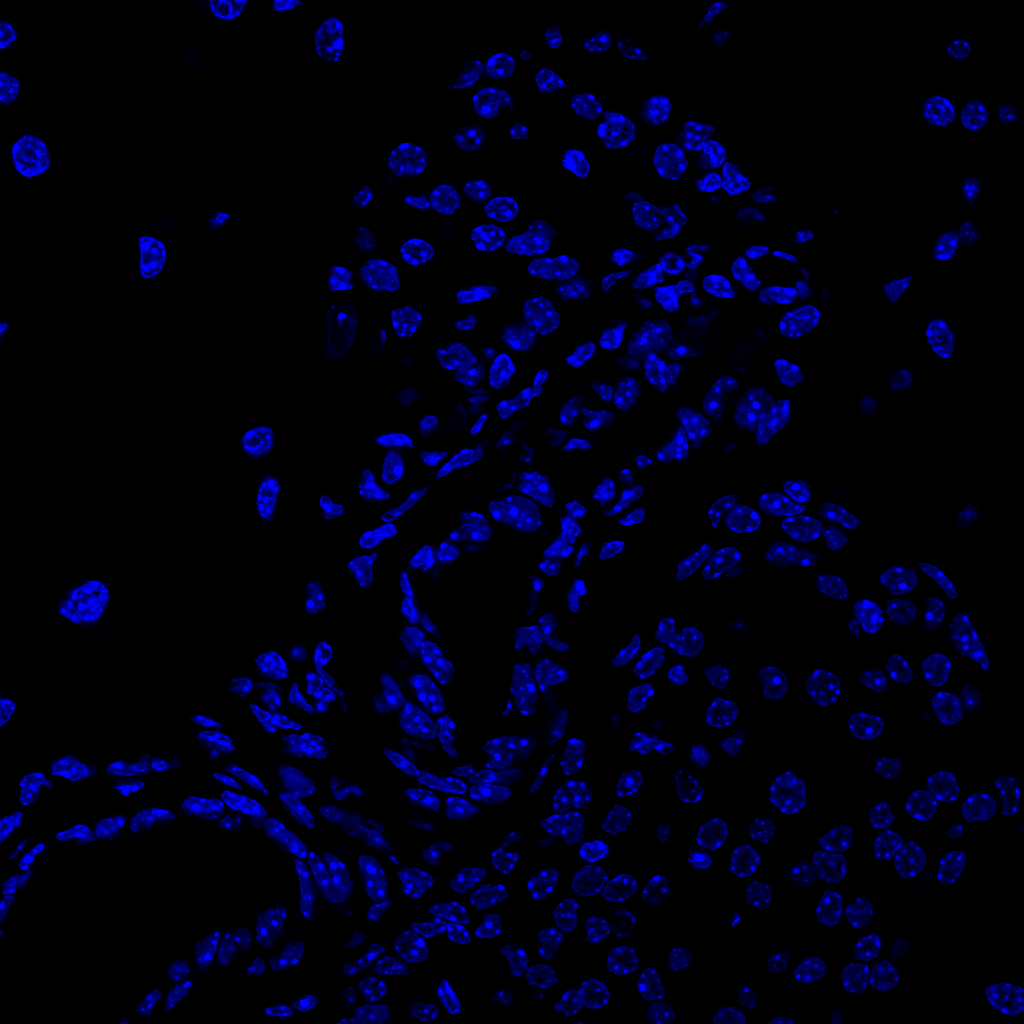

Supplement: Supplementary file 8 — Source data Fig. 6 [file 44318_2025_434_MOESM8_ESM.zip › Figure 6/6D/6D_Merge_mag_2 (blue).tif]

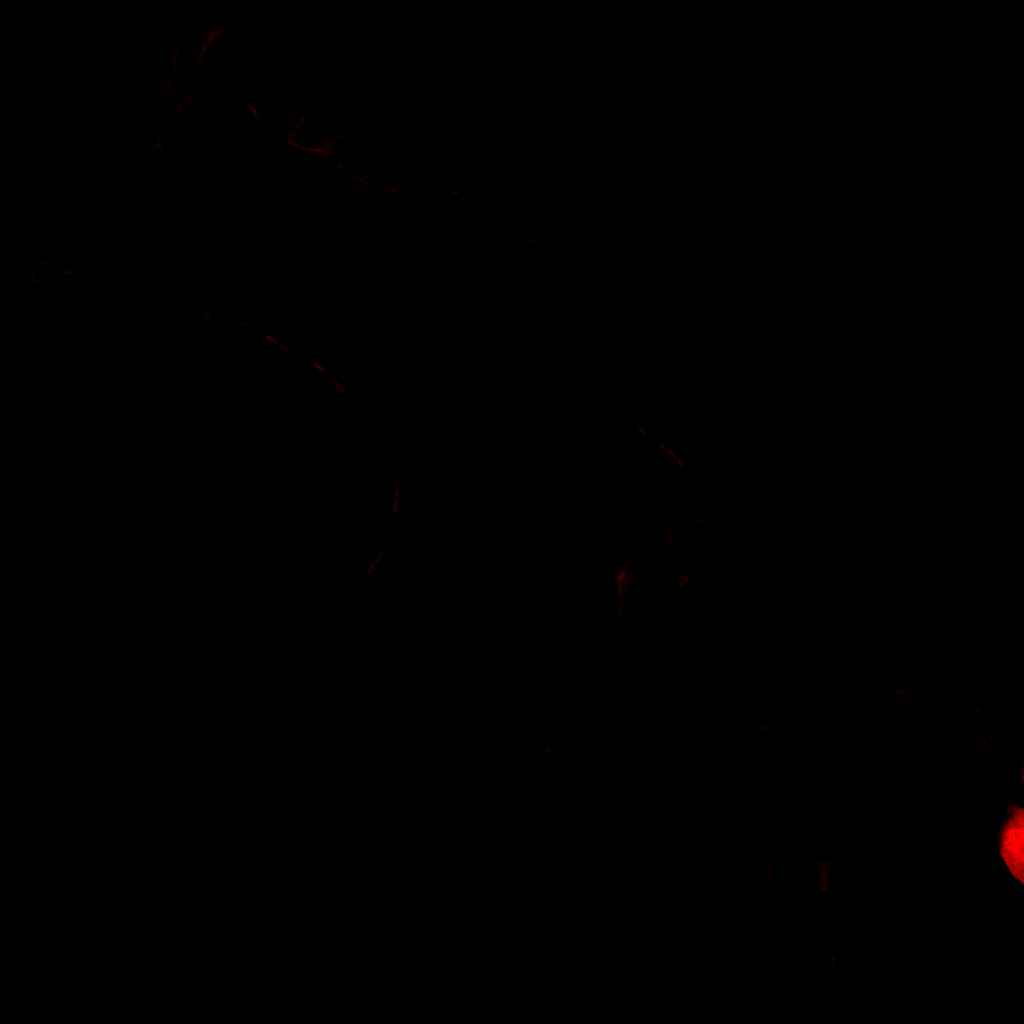

Supplement: Supplementary file 8 — Source data Fig. 6 [file 44318_2025_434_MOESM8_ESM.zip › Figure 6/6D/6D_Merge_mag_1.oib (red).tif]

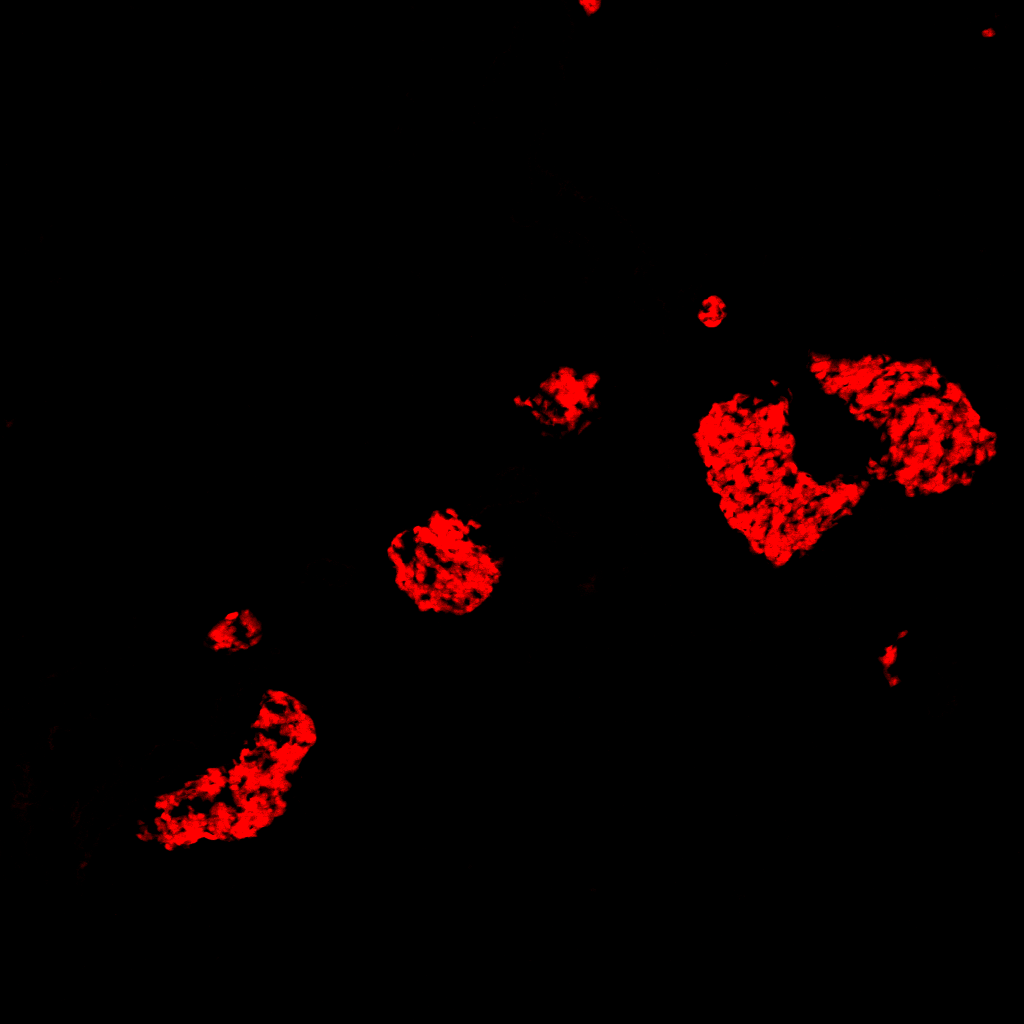

Supplement: Supplementary file 8 — Source data Fig. 6 [file 44318_2025_434_MOESM8_ESM.zip › Figure 6/6D/6D_Merge (red).tif]

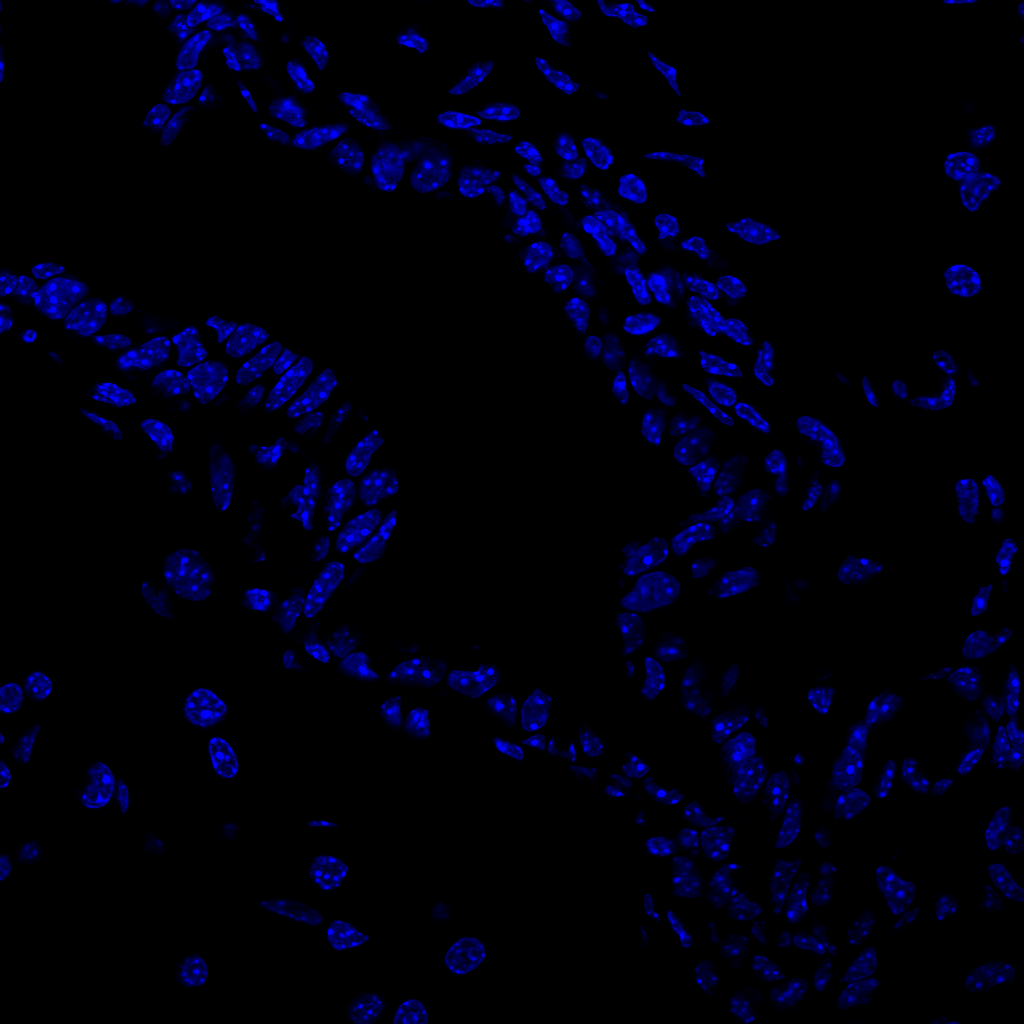

Supplement: Supplementary file 8 — Source data Fig. 6 [file 44318_2025_434_MOESM8_ESM.zip › Figure 6/6D/6D_Merge_mag_1.oib (blue).tif]

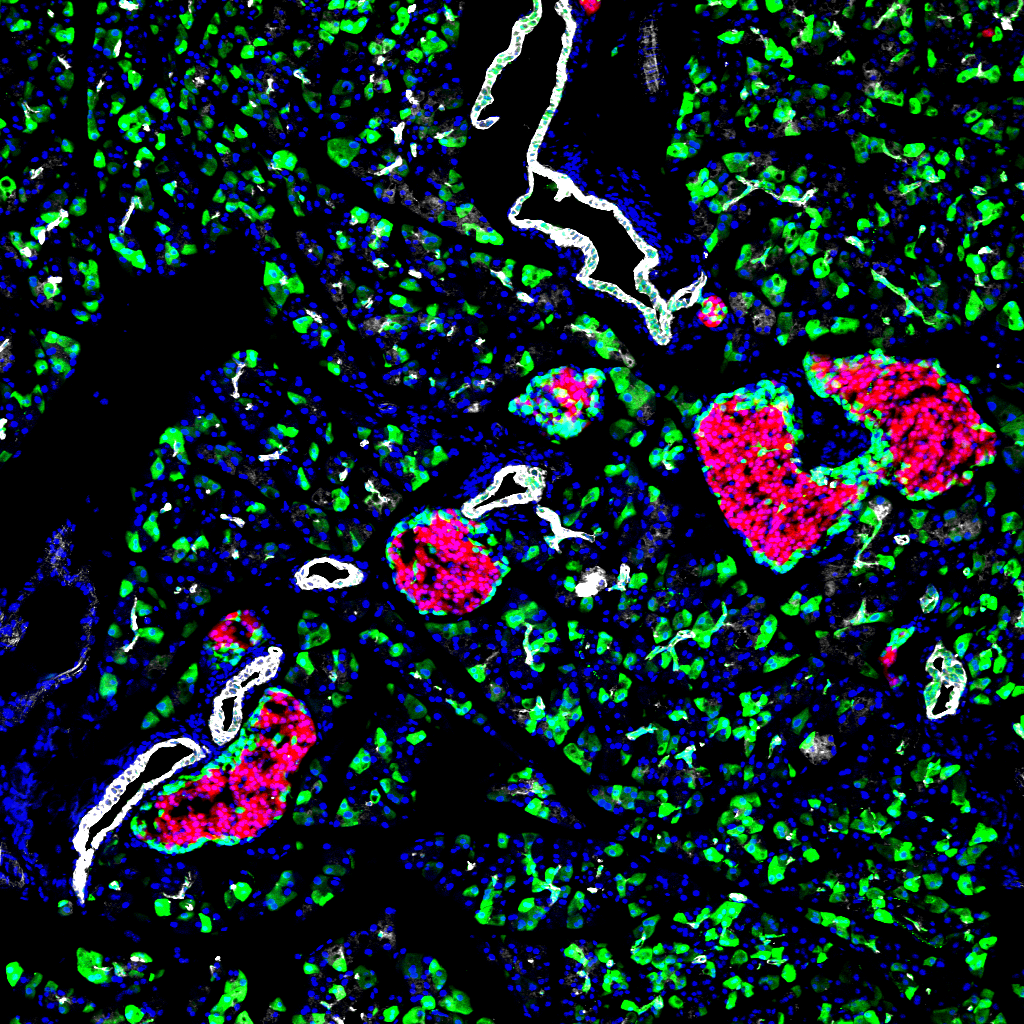

Supplement: Supplementary file 8 — Source data Fig. 6 [file 44318_2025_434_MOESM8_ESM.zip › Figure 6/6D/6D_Merge.tif]

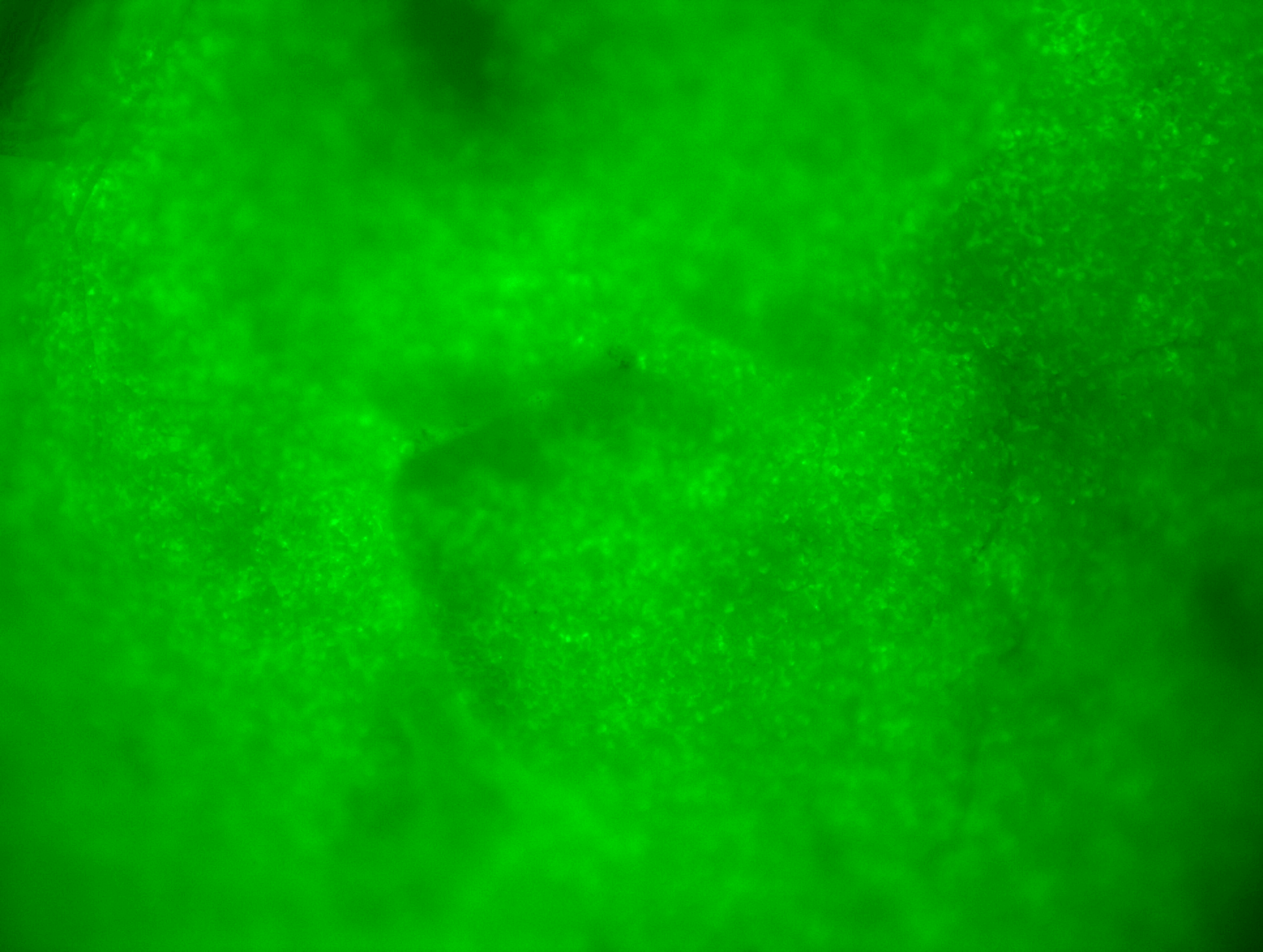

Supplement: Supplementary file 8 — Source data Fig. 6 [file 44318_2025_434_MOESM8_ESM.zip › Figure 6/6C/6C_2w_zsGreen_mag.tif]

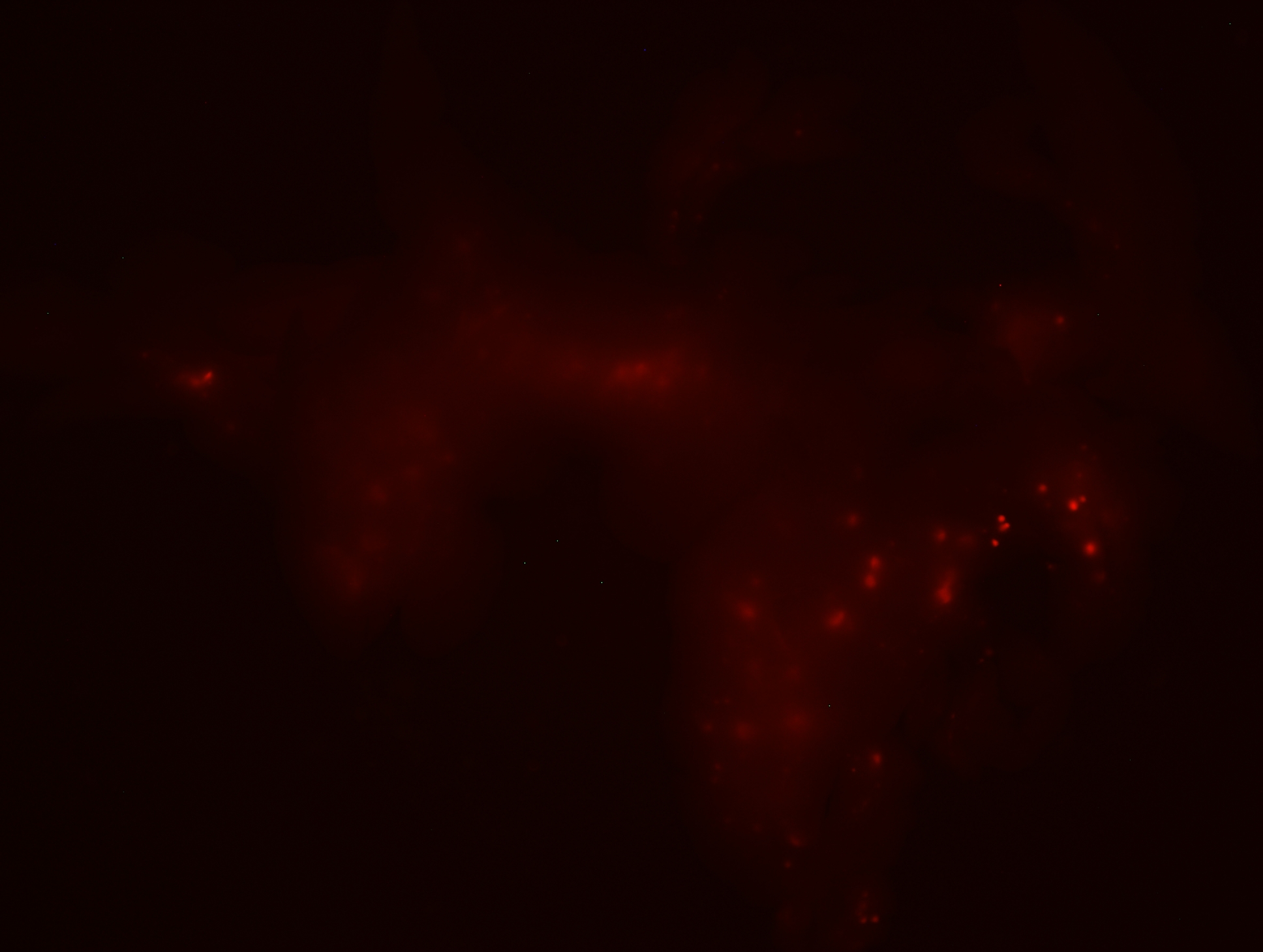

Supplement: Supplementary file 8 — Source data Fig. 6 [file 44318_2025_434_MOESM8_ESM.zip › Figure 6/6C/6C_2w_tdT.tif]

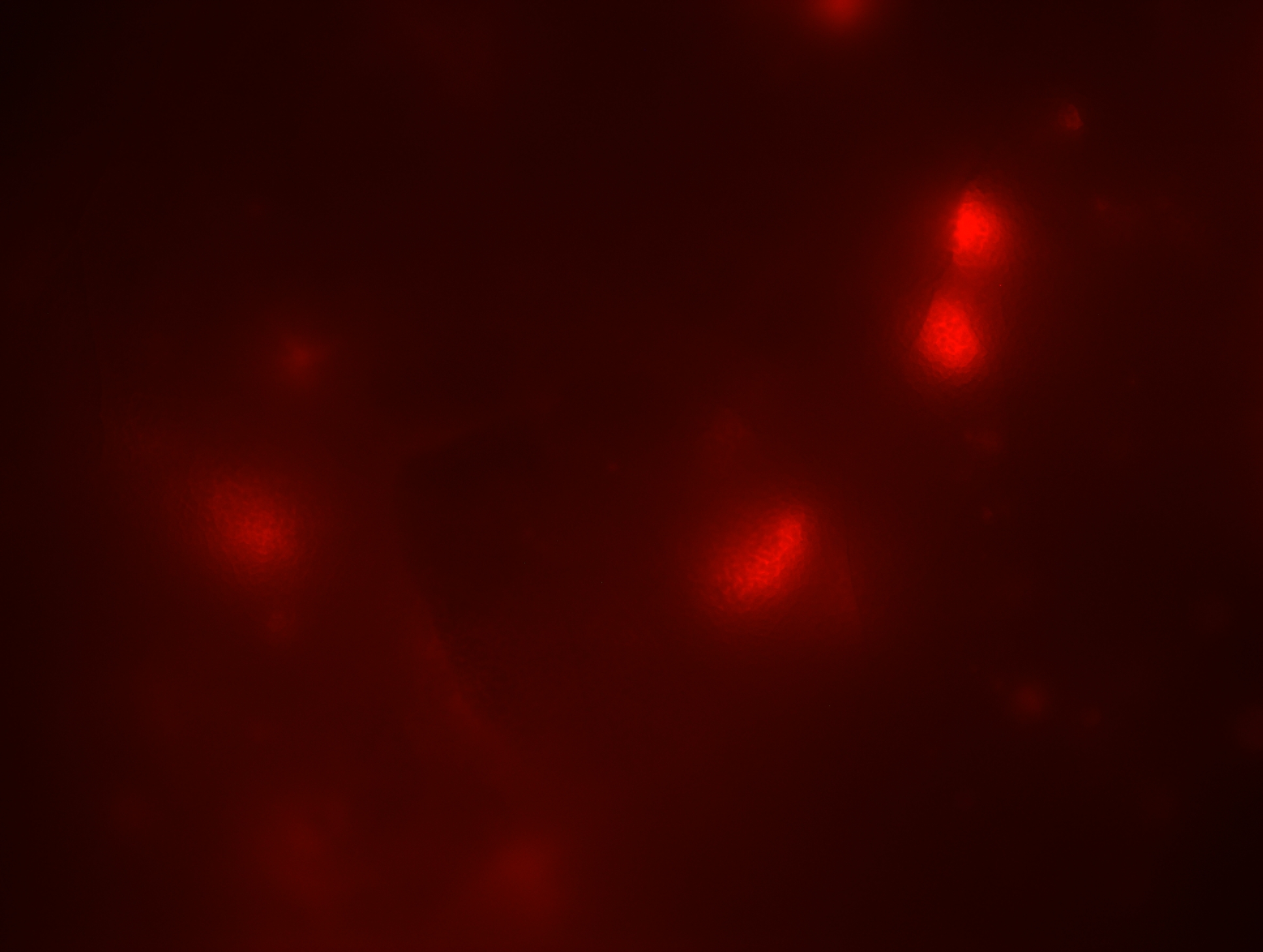

Supplement: Supplementary file 8 — Source data Fig. 6 [file 44318_2025_434_MOESM8_ESM.zip › Figure 6/6C/6C_2w_tdT_mag.tif]

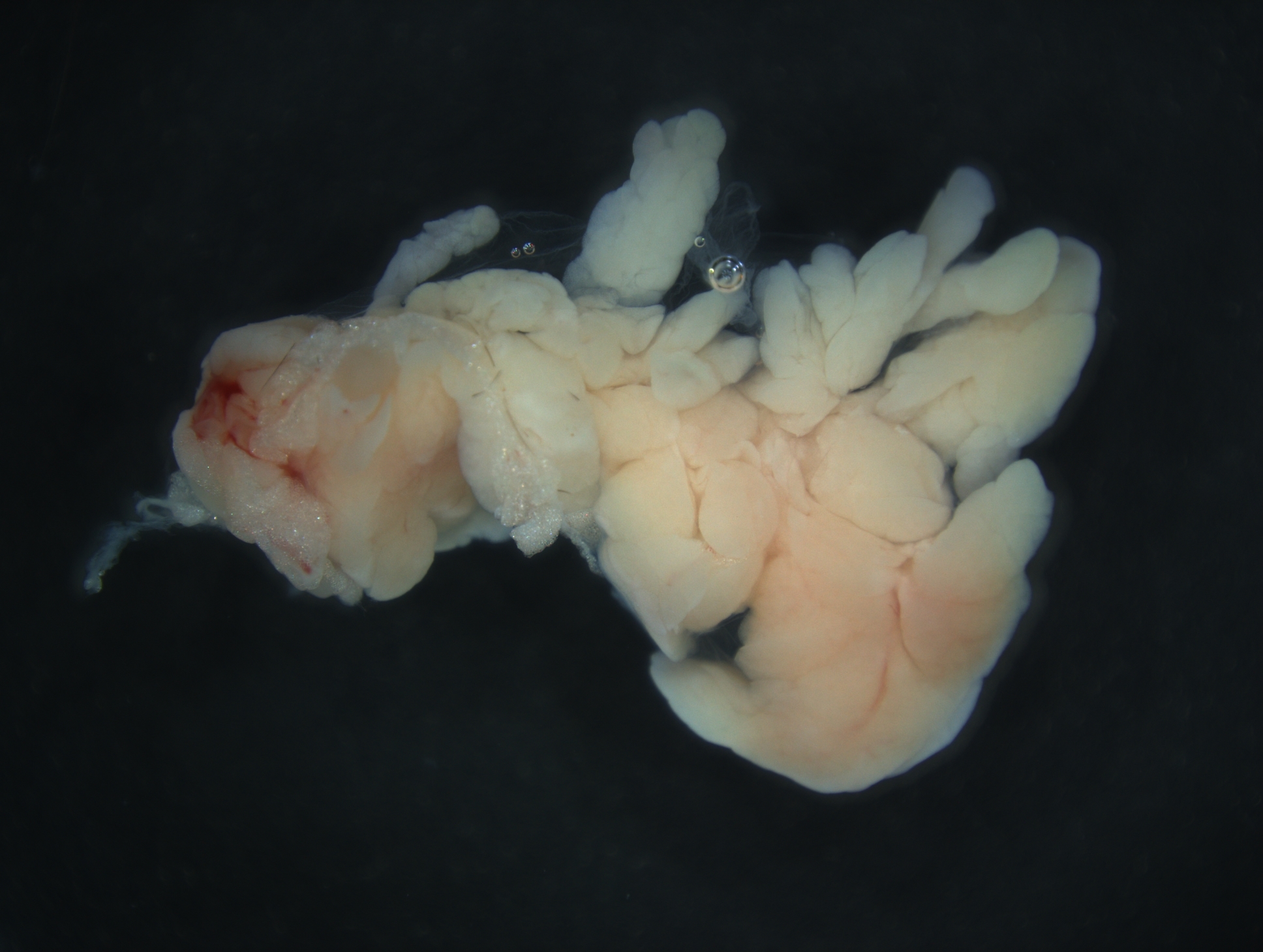

Supplement: Supplementary file 8 — Source data Fig. 6 [file 44318_2025_434_MOESM8_ESM.zip › Figure 6/6C/6C_12w_BF.tif]

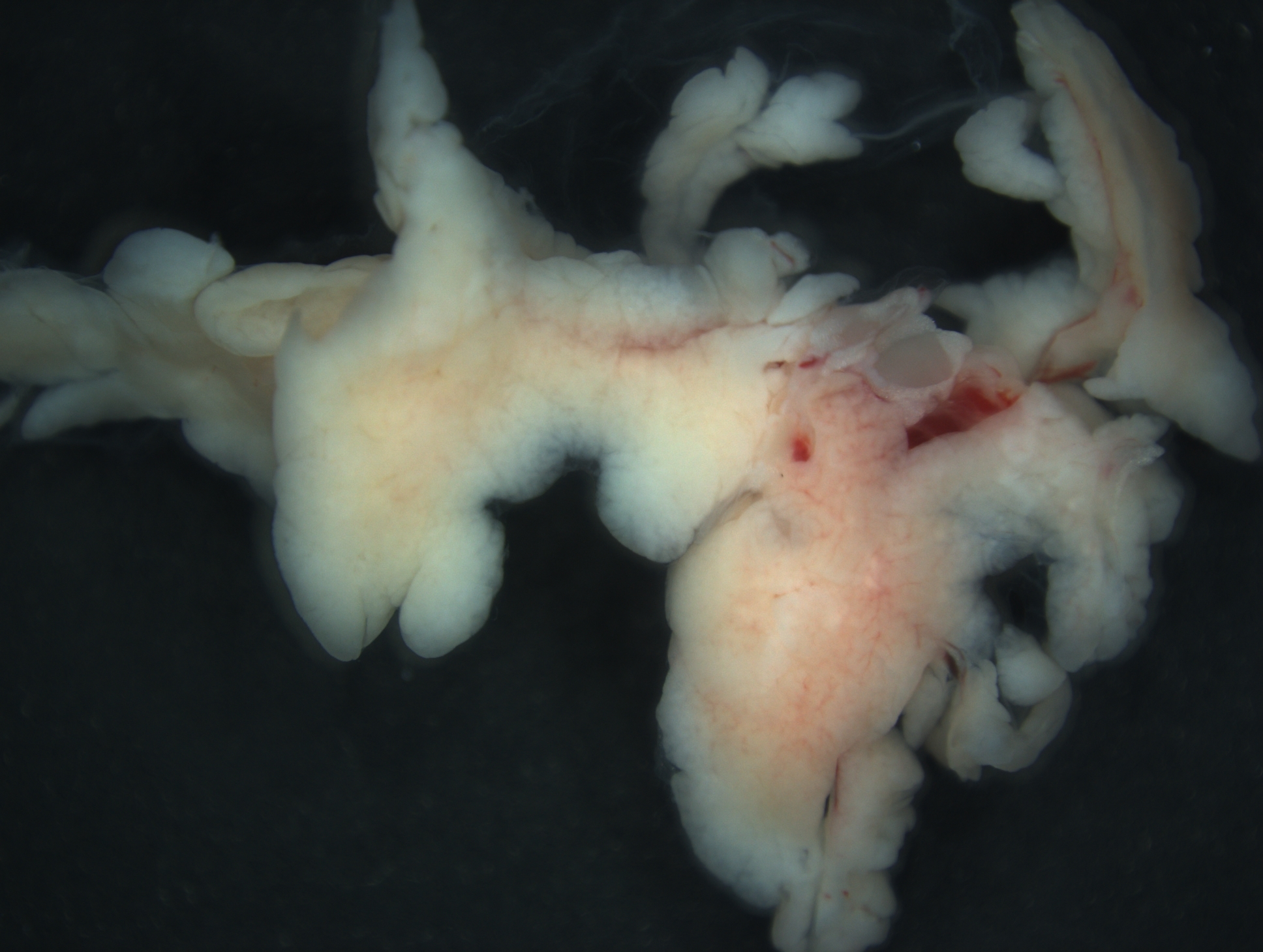

Supplement: Supplementary file 8 — Source data Fig. 6 [file 44318_2025_434_MOESM8_ESM.zip › Figure 6/6C/6C_2w_BF.tif]

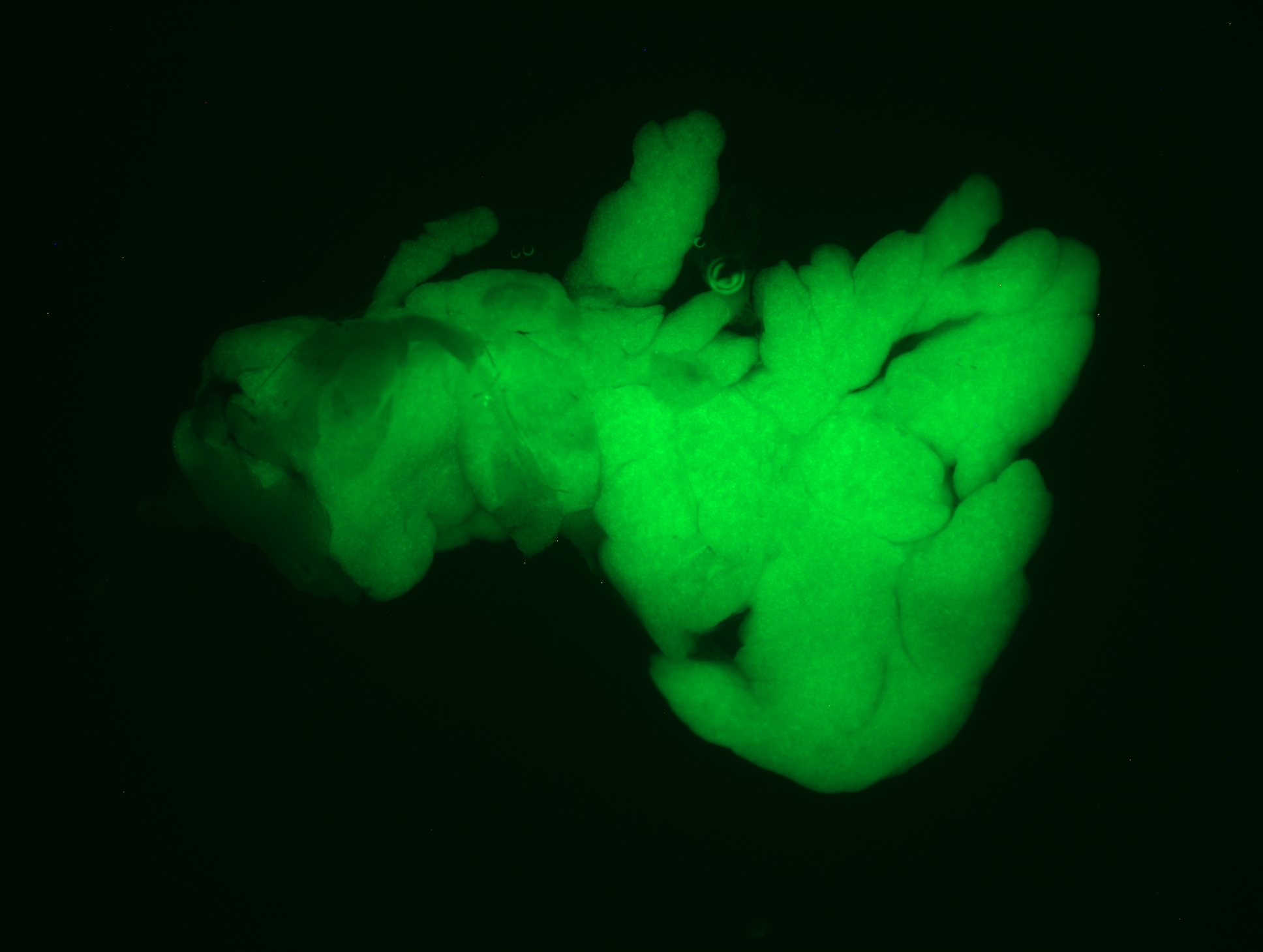

Supplement: Supplementary file 8 — Source data Fig. 6 [file 44318_2025_434_MOESM8_ESM.zip › Figure 6/6C/6C_12w_zsGreen.tif]

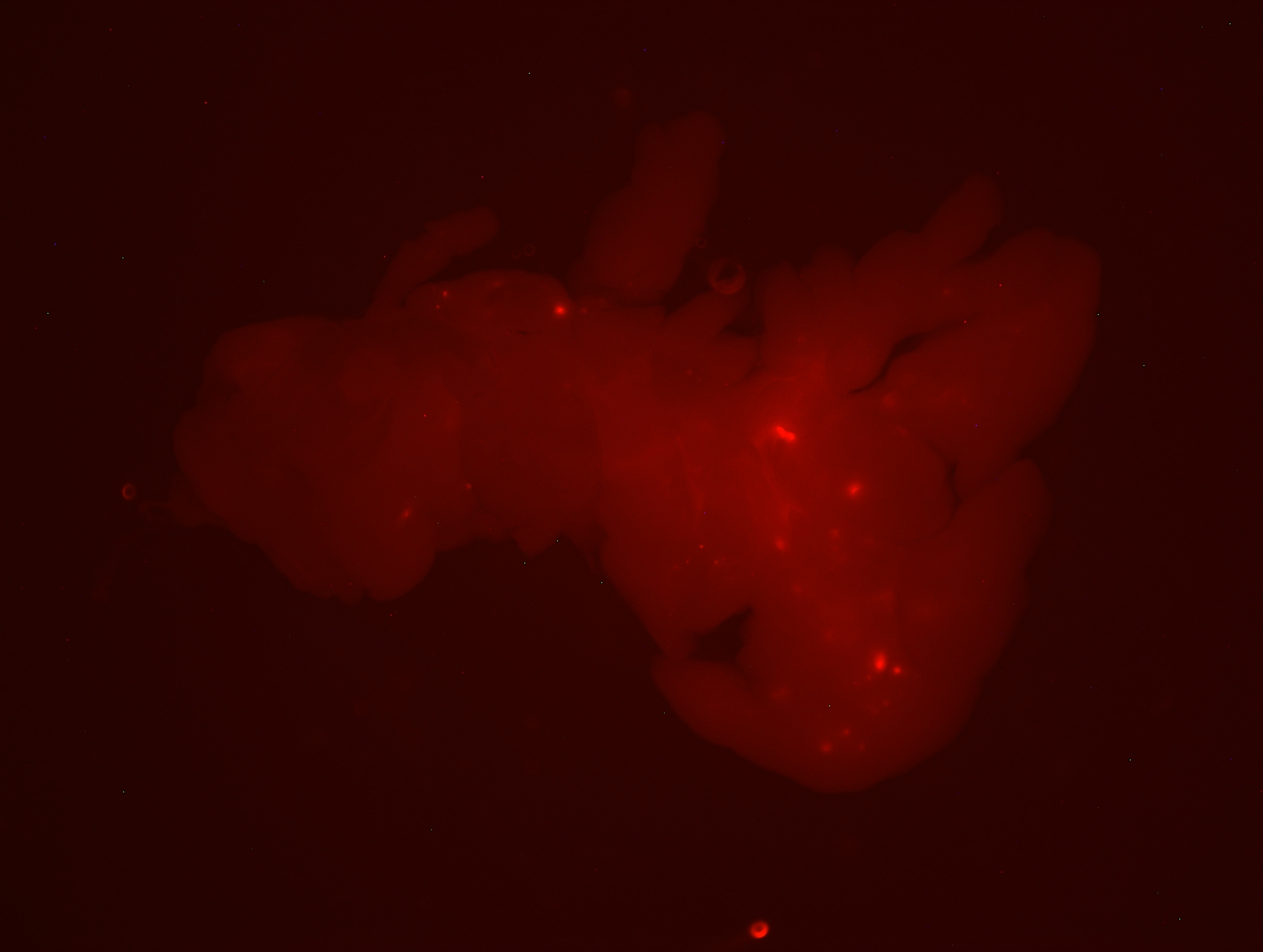

Supplement: Supplementary file 8 — Source data Fig. 6 [file 44318_2025_434_MOESM8_ESM.zip › Figure 6/6C/6C_12w_tdT.tif]

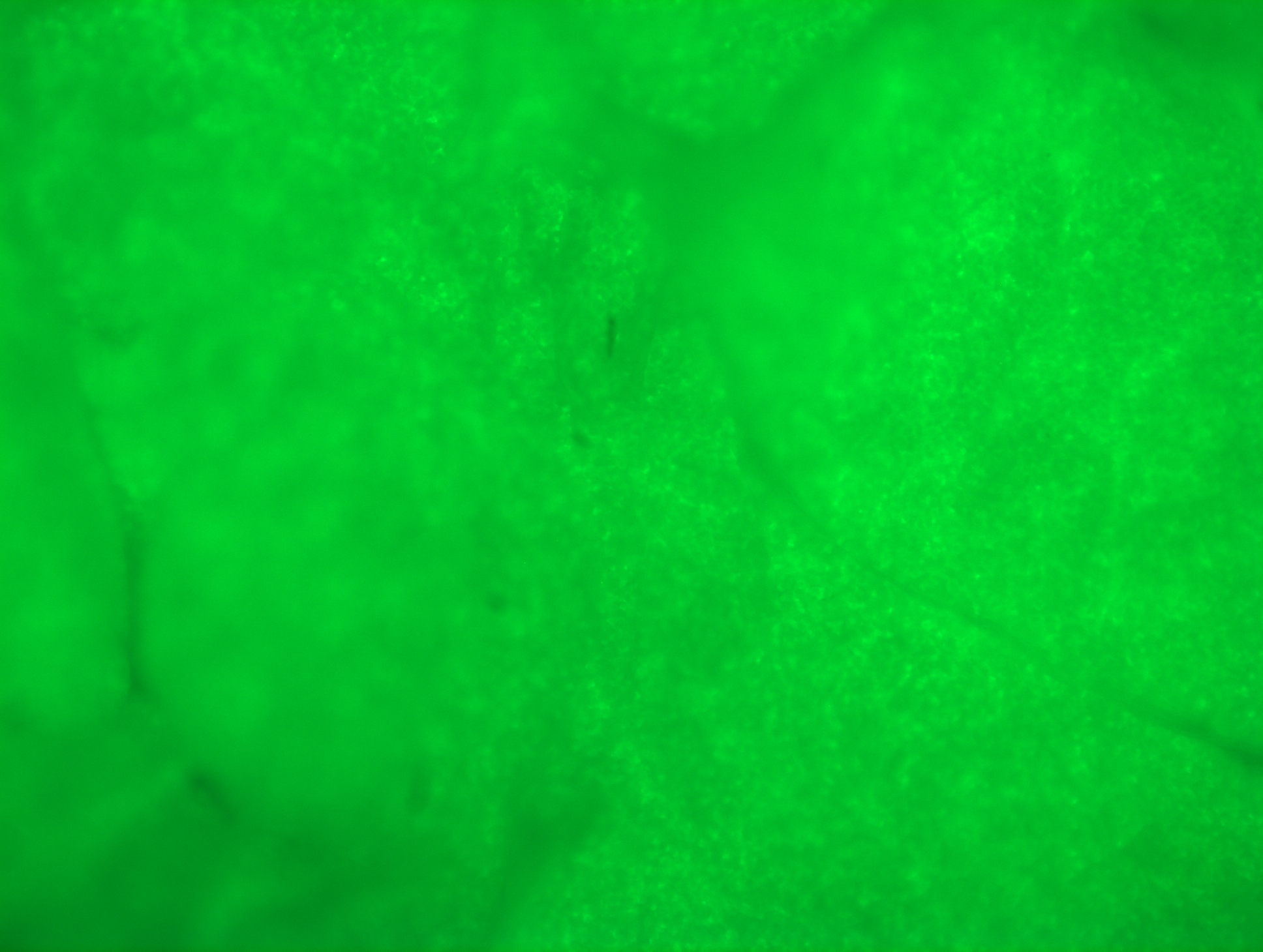

Supplement: Supplementary file 8 — Source data Fig. 6 [file 44318_2025_434_MOESM8_ESM.zip › Figure 6/6C/6C_12w_zsGreen_mag.tif]

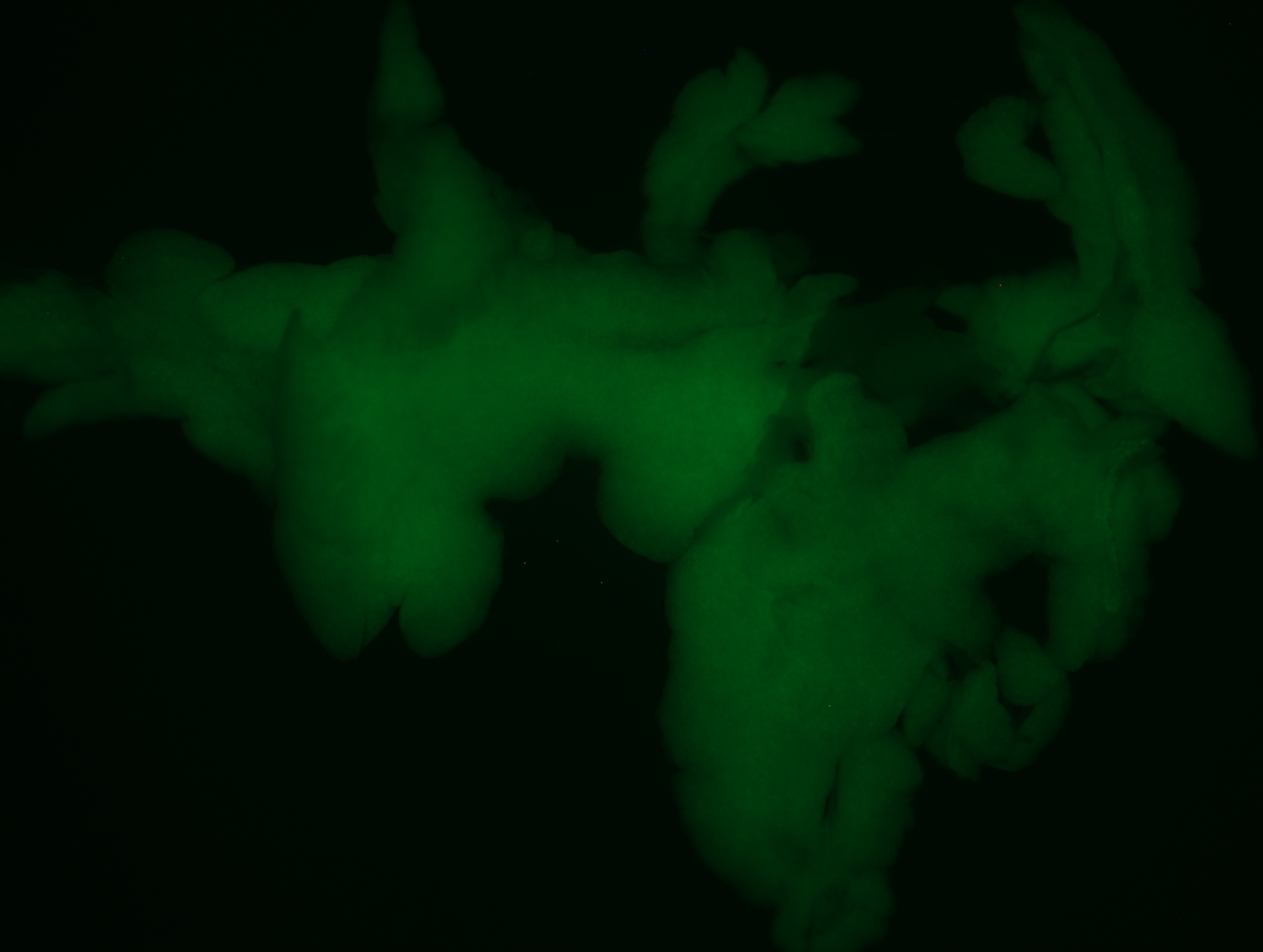

Supplement: Supplementary file 8 — Source data Fig. 6 [file 44318_2025_434_MOESM8_ESM.zip › Figure 6/6C/6C_2w_zsGreen.tif]

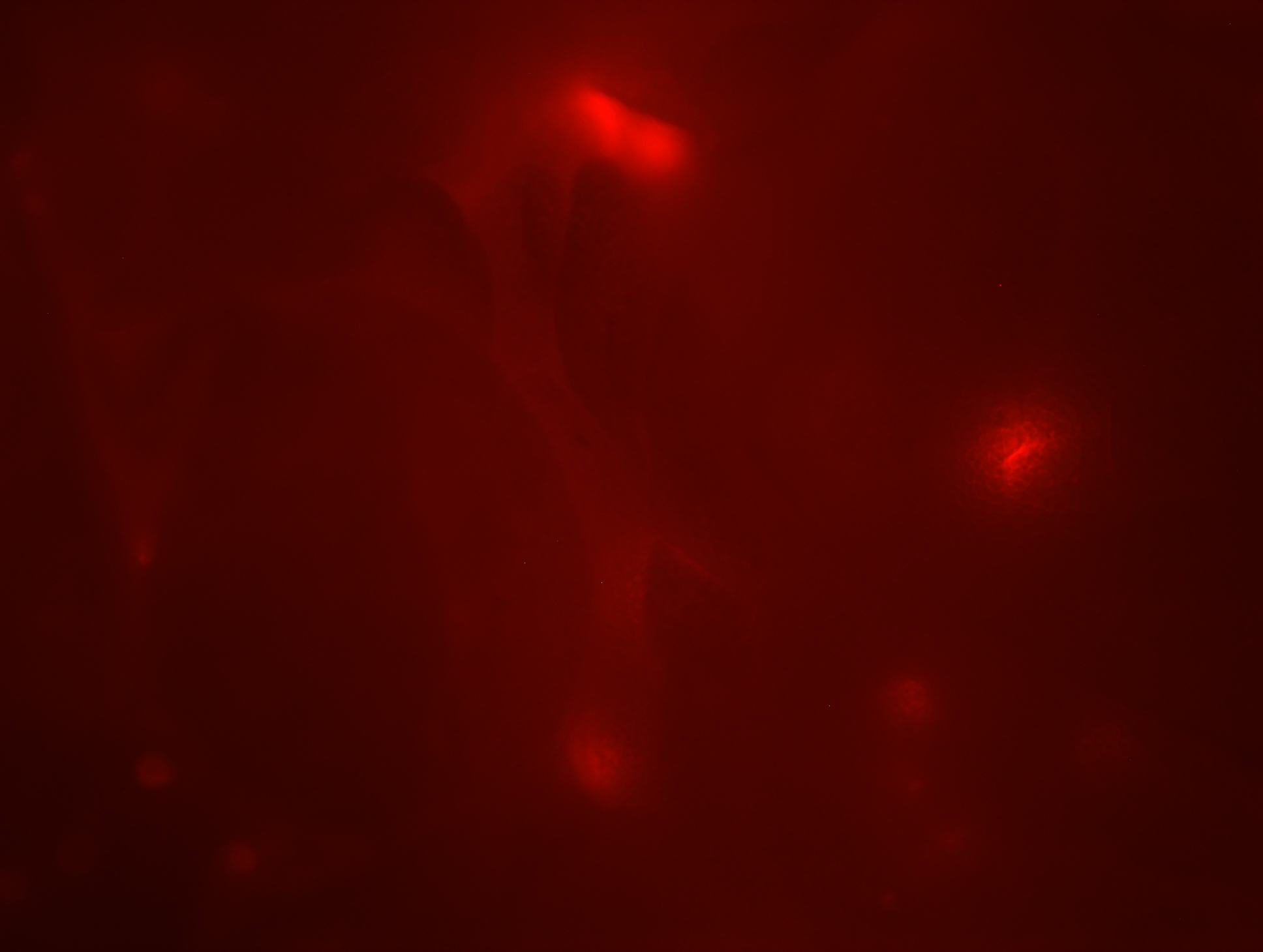

Supplement: Supplementary file 8 — Source data Fig. 6 [file 44318_2025_434_MOESM8_ESM.zip › Figure 6/6C/6C_12w_tdT_mag.tif]
