# Supplementary material for: Ductal or Ngn3+ cells do not contribute to adult pancreatic islet beta-cell neogenesis in homeostasis
Source: EMBO J. 2025 Apr 9;44(10):2856–81. doi: 10.1038/s44318-025-00434-z (PMC12084597; doi:10.1038/s44318-025-00434-z)
Supplement: Supplementary file 9 — Expanded View Figures [file 44318_2025_434_MOESM9_ESM.pdf]

Expanded View Figures

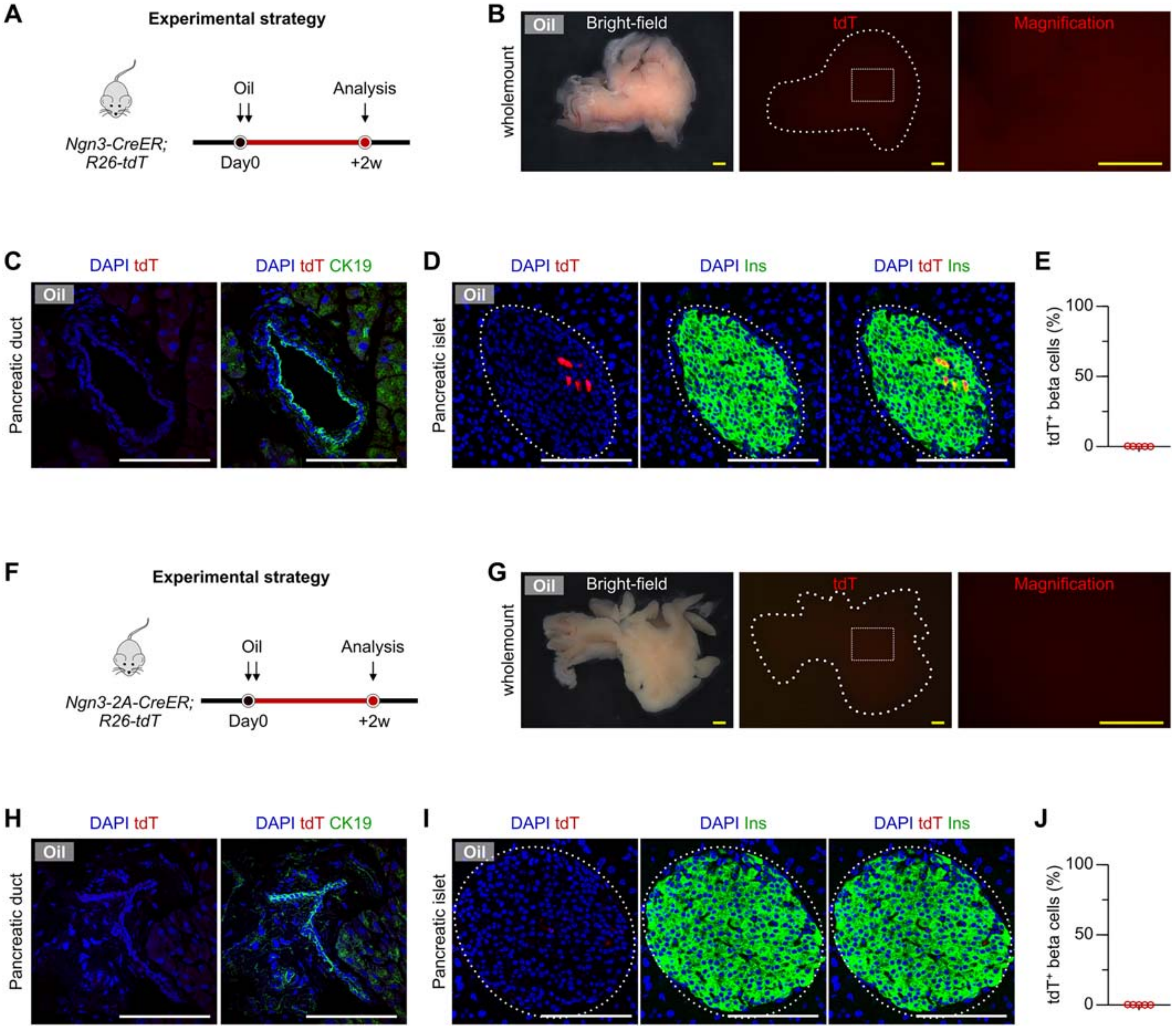

Figure EV1. Characterization of *Ngn3-CreER;R26-tdT* and *Ngn3-2A-CreER;R26-tdT* without tamoxifen treatment, related to Fig. 1.

(**A**) Schematic showing the experimental strategy of *Ngn3-CreER;R26-tdT* with oil treatment. (**B**) Whole-mount fluorescent images of pancreas collected from *Ngn3-CreER;R26-tdT* after oil treatment. (**C**, **D**) Immunostaining for tdT and CK19 (**C**) or Insulin (Ins, **D**) on pancreatic sections of *Ngn3-CreER;R26-tdT* after oil treatment. (**E**) Quantification of the percentage of tdT^+ cells in Ins^+ beta cells of *Ngn3-CreER;R26-tdT* after oil treatment. Data are mean ± SD; $n = 5$ biological replicates. In each sample, islets from 10 pancreas sections were quantified. (**F**) Schematic showing the experimental strategy of *Ngn3-2A-CreER;R26-tdT* with oil treatment. (**G**) Whole-mount fluorescent images of pancreas collected from *Ngn3-2A-CreER;R26-tdT* after oil treatment. (**H**, **I**) Immunostaining for tdT and CK19 (**H**) or Ins (**I**) on pancreatic sections of *Ngn3-2A-CreER;R26-tdT* after oil treatment. (**J**) Quantification of the percentage of tdT^+ cells in Ins^+ beta cells of *Ngn3-2A-CreER;R26-tdT* after oil treatment. Data are mean ± SD; $n = 5$ biological replicates. In each sample, islets from 10 pancreas sections were quantified. Scale bars, yellow, 1 mm; white, 100 μm. Each image is representative of 5 individual samples.

 © The Author(s)

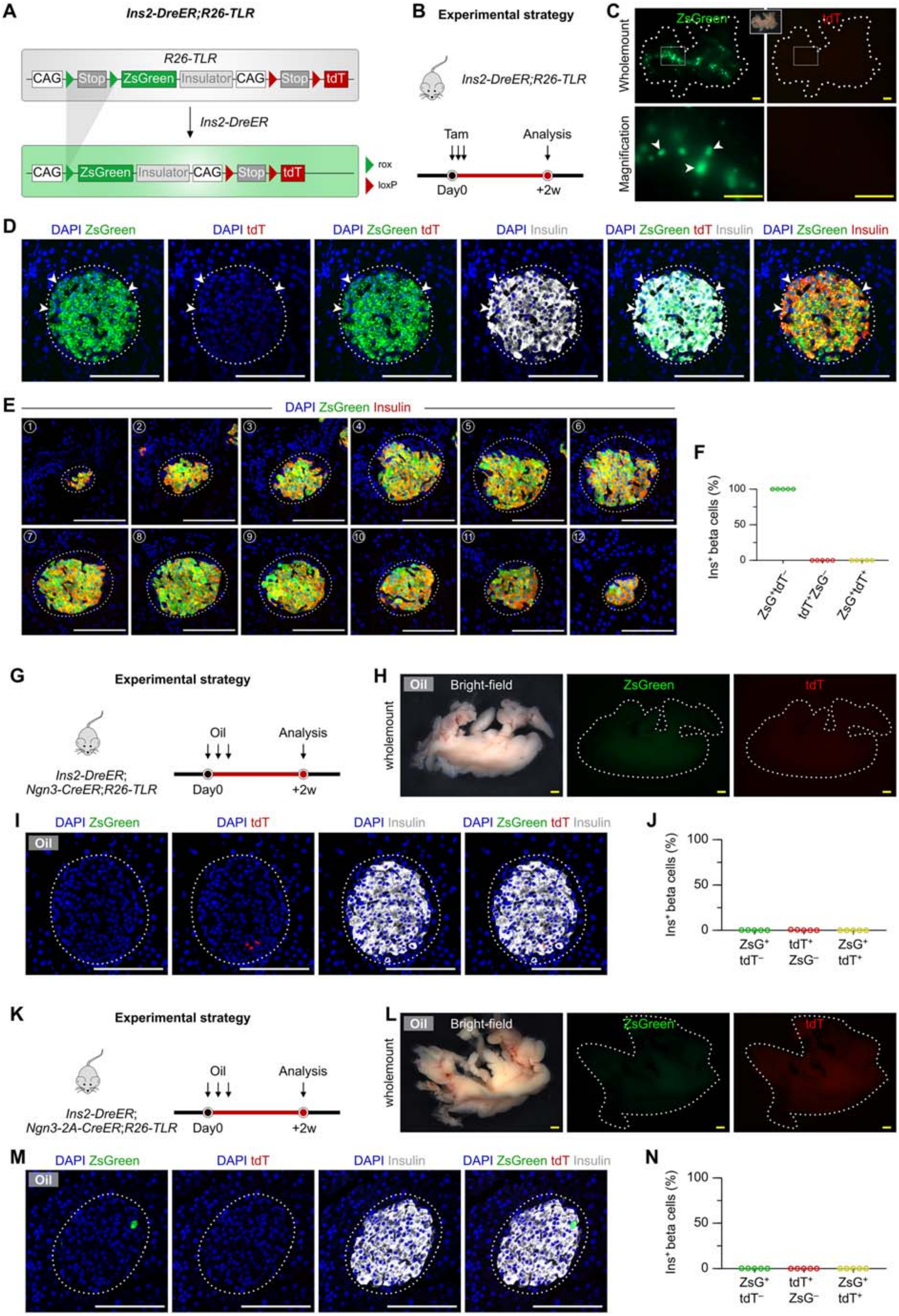
A
Ins2-DreER;R26-TLR
R26-TLR
CAG Stop ZsGreen Insulator CAG Stop tdT
Ins2-DreER
CAG ZsGreen Insulator CAG Stop tdT
rox
loxP
B
Experimental strategy
Ins2-DreER;R26-TLR
Tam
Analysis
Day0
+2w
C
Wholemount
Magnification
ZsGreen
tdT
D
DAPI ZsGreen
DAPI tdT
DAPI ZsGreen tdT
DAPI Insulin
DAPI ZsGreen tdT Insulin
DAPI ZsGreen Insulin
E
DAPI ZsGreen Insulin
F
Ins+ beta cells (%)
100
50
0
ZsG+tdT−
tdT+ZsG−
ZsG+tdT+
G
Experimental strategy
Ins2-DreER;
Ngn3-CreER;R26-TLR
Oil
Analysis
Day0
+2w
H
wholemount
Oil
Bright-field
ZsGreen
tdT
I
DAPI ZsGreen
DAPI tdT
DAPI Insulin
DAPI ZsGreen tdT Insulin
Oil
J
Ins+ beta cells (%)
100
50
0
ZsG+ tdT−
tdT+ ZsG−
ZsG+ tdT+
K
Experimental strategy
Ins2-DreER;
Ngn3-2A-CreER;R26-TLR
Oil
Analysis
Day0
+2w
L
wholemount
Oil
Bright-field
ZsGreen
tdT
M
DAPI ZsGreen
DAPI tdT
DAPI Insulin
DAPI ZsGreen tdT Insulin
Oil
N
Ins+ beta cells (%)
100
50
0
ZsG+ tdT−
tdT+ ZsG−
ZsG+ tdT+

Figure EV2. Characterization of *Ins2-DreER;R26-TLR*, and characterization of *Ins2-DreER;Ngn3-CreER;R26-TLR* and *Ins2-DreER;Ngn3-2A-CreER;R26-TLR* without tamoxifen treatment, related to Fig. 2.

(**A**) Schematic showing the labeling strategy of *Ins2-DreER;R26-TLR*. (**B**) Schematic showing the experimental strategy of *Ins2-DreER;R26-TLR* with tamoxifen (Tam) treatment. (**C**) Whole-mount fluorescent images of pancreas from *Ins2-DreER;R26-TLR*. Arrowheads, zsGreen$^+$ islets. (**D**) Immunostaining for ZsGreen, tdT and Ins on pancreatic sections of *Ins2-DreER;R26-TLR* with Tam treatment. Arrowheads, zsGreen$^+$ beta cells. (**E**) Immunostaining for ZsGreen and Ins on serial sections of one pancreatic islet from *Ins2-DreER;R26-TLR* with Tam treatment. (**F**) Quantification of the percentages of ZsGreen$^+$tdT$^-$ or tdT$^+$ZsGreen$^-$ or ZsGreen$^+$tdT$^+$ cells in Ins$^+$ pancreatic beta cells of *Ins2-DreER;R26-TLR*. Data are mean ± SD; $n = 5$ biological replicates. In each sample, islets from 10 pancreas sections were quantified. (**G**) Schematic showing the experimental strategy of *Ins2-DreER;Ngn3-CreER;R26-TLR* with oil treatment. (**H**) Whole-mount fluorescent images of pancreas from *Ins2-DreER;Ngn3-CreER;R26-TLR* after oil treatment. (**I**) Immunostaining for ZsGreen, tdT and Ins on pancreatic sections of *Ins2-DreER;Ngn3-CreER;R26-TLR* after oil treatment. (**J**) Quantification of the percentage of ZsGreen$^+$tdT$^-$ or tdT$^+$ZsGreen$^-$ or ZsGreen$^+$tdT$^+$ cells in Ins$^+$ pancreatic beta cells of *Ins2-DreER;Ngn3-CreER;R26-TLR* after oil treatment. Data are mean ± SD; $n = 5$ biological replicates. In each sample, islets from 10 pancreas sections were quantified. (**K**) Schematic showing the experimental strategy of *Ins2-DreER;Ngn3-2A-CreER;R26-TLR* with oil treatment. (**L**) Whole-mount fluorescent images of pancreas from *Ins2-DreER;Ngn3-2A-CreER;R26-TLR* after oil treatment. (**M**) Immunostaining for ZsGreen, tdT and Ins on pancreatic sections of *Ins2-DreER;Ngn3-2A-CreER;R26-TLR* after oil treatment. (**N**) Quantification of the percentage of ZsGreen$^+$ or tdT$^+$ or ZsGreen$^+$tdT$^+$ cells in Ins$^+$ pancreatic beta cells of *Ins2-DreER;Ngn3-2A-CreER;R26-TLR* after oil treatment. Data are mean ± SD; $n = 5$ biological replicates. In each sample, islets from 10 pancreas sections were quantified. Scale bars, yellow, 1 mm; white, 100 μm. Each image is representative of 5 individual samples.

 © The Author(s)

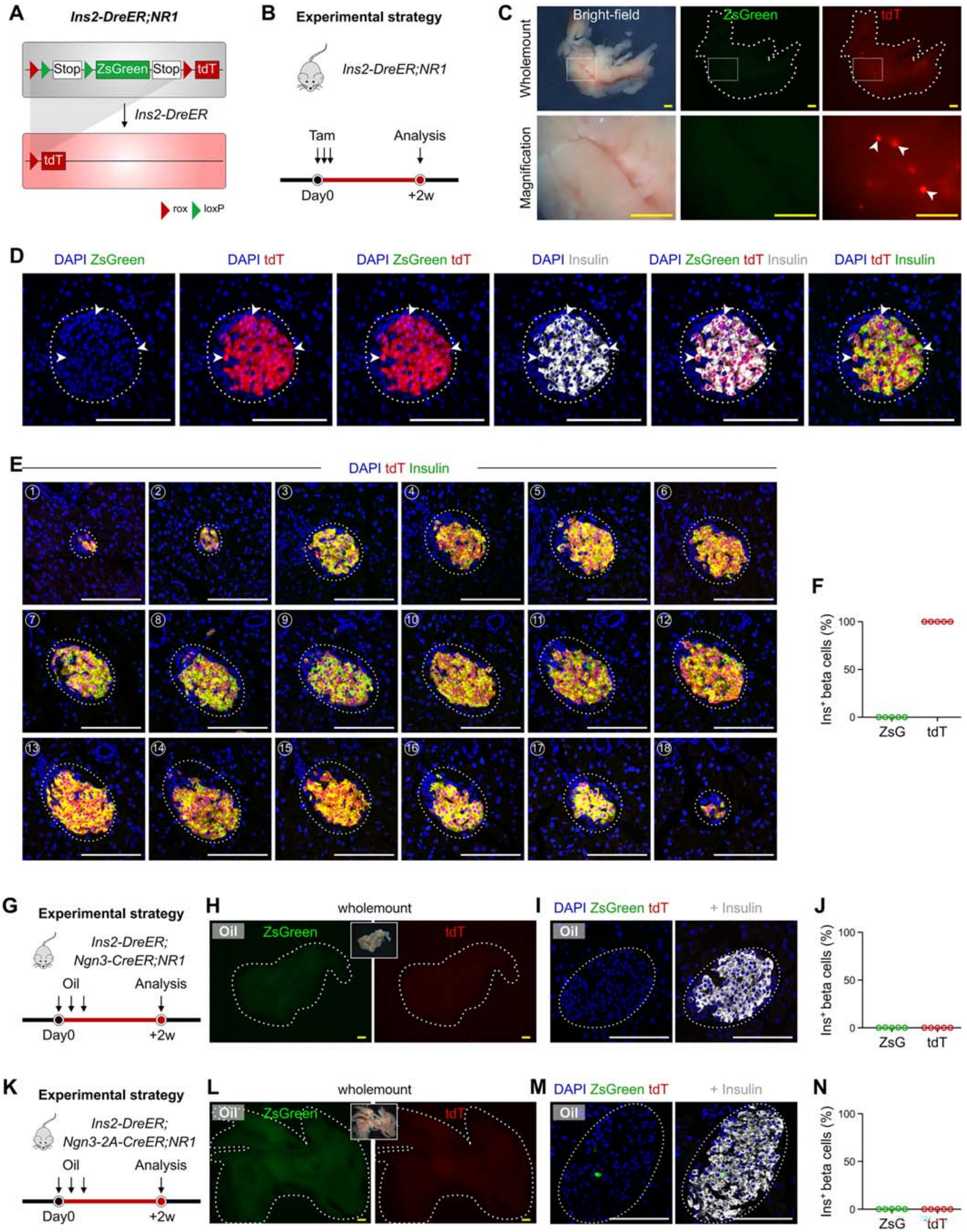
A
Ins2-DreER;NR1
Stop
ZsGreen
Stop
tdT
Ins2-DreER
tdT
rox
loxP
B
Experimental strategy
Ins2-DreER;NR1
Tam
Analysis
Day0
+2w
C
Bright-field
ZsGreen
tdT
Wholemount
Magnification
D
DAPI ZsGreen
DAPI tdT
DAPI ZsGreen tdT
DAPI Insulin
DAPI ZsGreen tdT Insulin
DAPI tdT Insulin
E
DAPI tdT Insulin
F
Ins+ beta cells (%)
100
50
0
ZsG
tdT
G
Experimental strategy
Ins2-DreER;
Ngn3-CreER;NR1
Oil
Analysis
Day0
+2w
H
wholemount
Oil
ZsGreen
tdT
I
DAPI ZsGreen tdT
+ Insulin
Oil
J
Ins+ beta cells (%)
100
50
0
ZsG
tdT
K
Experimental strategy
Ins2-DreER;
Ngn3-2A-CreER;NR1
Oil
Analysis
Day0
+2w
L
wholemount
Oil
ZsGreen
tdT
M
DAPI ZsGreen tdT
+ Insulin
Oil
N
Ins+ beta cells (%)
100
50
0
ZsG
tdT

Figure EV3. Characterization of *Ins2-DreER;NR1*, and characterization of *Ins2-DreER;Ngn3-CreER;NR1* and *Ins2-DreER;Ngn3-2A-CreER;NR1* without tamoxifen treatment, related to Fig. 3.

(**A**) Schematic showing the labeling strategy of *Ins2-DreER;NR1*. (**B**) Schematic showing the experimental strategy of *Ins2-DreER;NR1* with Tam treatment. (**C**) Whole-mount fluorescent images of pancreas from *Ins2-DreER;NR1* with Tam treatment. Arrowheads, tdT^+ islets. (**D**) Immunostaining for ZsGreen, tdT and Ins on pancreatic sections of *Ins2-DreER;NR1* with Tam treatment. Arrowheads, tdT^+ beta cells. (**E**) Immunostaining for tdT and Ins on serial sections of one pancreatic islet from *Ins2-DreER;NR1* with Tam treatment. (**F**) Quantification of the percentage of $ZsGreen^+$ or tdT^+ cells in Ins^+ pancreatic beta cells of *Ins2-DreER;NR1*. Data are mean ± SD; $n = 5$ biological replicates. In each sample, islets from 10 pancreas sections were quantified. (**G**) Schematic showing the experimental strategy of *Ins2-DreER;Ngn3-CreER;NR1* with oil treatment. (**H**) Whole-mount fluorescent images of pancreas from *Ins2-DreER;Ngn3-CreER;NR1* after oil treatment. (**I**) Immunostaining for ZsGreen, tdT and Ins on pancreatic sections of *Ins2-DreER;Ngn3-CreER;NR1* after oil treatment. (**J**) Quantification of the percentage of $ZsGreen^+$ or tdT^+ cells in Ins^+ pancreatic beta cells of *Ins2-DreER;Ngn3-CreER;NR1* after oil treatment. Data are mean ± SD; $n = 5$ biological replicates. In each sample, islets from 10 pancreas sections were quantified. (**K**) Schematic showing the experimental strategy of *Ins2-DreER;Ngn3-2A-CreER;NR1* with oil treatment. (**L**) Whole-mount fluorescent images of pancreas from *Ins2-DreER;Ngn3-2A-CreER;NR1* after oil treatment. (**M**) Immunostaining for ZsGreen, tdT and Ins on pancreatic sections of *Ins2-DreER;Ngn3-2A-CreER;NR1* after oil treatment. (**N**) Quantification of the percentage of $ZsGreen^+$ or tdT^+ cells in Ins^+ pancreatic beta cells of *Ins2-DreER;Ngn3-2A-CreER;NR1* after oil treatment. Data are mean ± SD; $n = 5$ biological replicates. In each sample, islets from 10 pancreas sections were quantified. Scale bars, yellow, 1 mm; white, 100 µm. Each image is representative of 5 individual samples.

 © The Author(s)

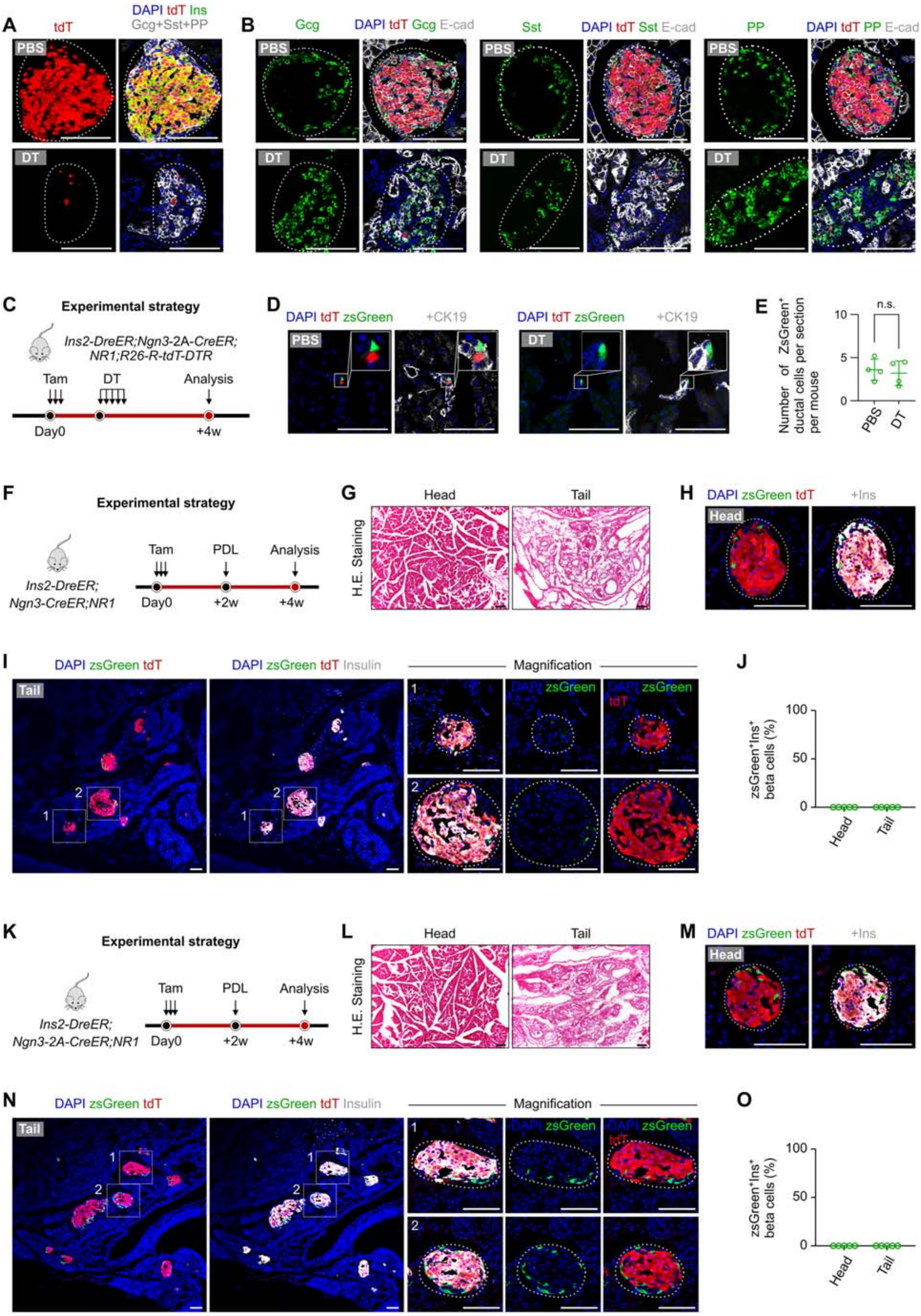
A
tdT
DAPI tdT Ins Gcg+Sst+PP
PBS
DT
B
Gcg
DAPI tdT Gcg E-cad
Sst
DAPI tdT Sst E-cad
PP
DAPI tdT PP E-cad
C
Experimental strategy
Ins2-DreER;Ngn3-2A-CreER; NR1;R26-R-tdT-DTR
Tam
DT
Analysis
Day0
+4w
D
DAPI tdT zsGreen +CK19
E
Number of ZsGreen+ ductal cells per section per mouse
n.s.
PBS
DT
F
Experimental strategy
Ins2-DreER; Ngn3-CreER;NR1
Tam
PDL
Analysis
Day0
+2w
+4w
G
H.E. Staining
Head
Tail
H
DAPI zsGreen tdT +Ins
I
DAPI zsGreen tdT
DAPI zsGreen tdT Insulin
Magnification
J
zsGreen+Ins+ beta cells (%)
K
Experimental strategy
Ins2-DreER; Ngn3-2A-CreER;NR1
L
M
N
O

Figure EV4. Characterization of *Ins2-DreER;Ngn3-CreER;NR1* and *Ins2-DreER;Ngn3-2A-CreER;NR1* with PDL injury, related to Fig. 4.

(**A**) Immunostaining for tdT, Ins, Gcg, Sst, and PP on pancreatic sections of *Ins2-DreER;R26-R-tdT*-DTR with PBS or DT treatment. (**B**) Immunostaining for tdT, Gcg, Sst, PP and E-cad on pancreatic sections of *Ins2-DreER;R26-R-tdT*-DTR with PBS or DT treatment. (**C**) Schematic showing the experimental strategy of *Ins2-DreER;Ngn3-2A-CreER;NR1;R26-R-tdT-DTR*. (**D**) Immunostaining for tdT, zsGreen and CK19 on pancreatic sections of *Ins2-DreER;Ngn3-2A-CreER;NR1;R26-R-tdT-DTR* mice with PBS or DT treatment. (**E**) Quantification of zsGreen$^+$ ductal cells per section of *Ins2-DreER;Ngn3-2A-CreER;NR1;R26-R-tdT-DTR* mice with PBS or DT treatment. Data are mean ± SD; PBS, $n = 4$ biological replicates; DT, $n = 4$ biological replicates; $P = 0.68$; n.s., non-significant. In each sample, islets from 10 pancreas sections were quantified. Two-tailed unpaired Student's *t*-tests were used for statistical comparisons and $p < 0.05$ was accepted as statistically significant. (**F**) Schematic showing the experimental strategy. (**G**) H.E. image of unligated head and ligated tail of the pancreas from *Ins2-DreER;Ngn3-CreER;NR1* mice 2 weeks after pancreatic ductal ligation (PDL) injury. (**H**) Immunostaining for tdT, zsGreen and Insulin on sections of unligated head of the pancreas from *Ins2-DreER;Ngn3-CreER;NR1* mice. (**I**) Immunostaining for tdT, zsGreen and Insulin on sections of ligated tail of the pancreas from *Ins2-DreER;Ngn3-CreER;NR1* mice. (**J**) Quantification of the percentages of ZsGreen$^+$ cells in Ins$^+$ pancreatic beta cells of unligated head and ligated tail of the pancreas from *Ins2-DreER;Ngn3-CreER;NR1* mice. Data are mean ± SD; $n = 5$ biological replicates. In each sample, islets from 10 pancreas sections were quantified. (**K**) Schematic showing the experimental strategy. (**L**) H.E. image of unligated head and ligated tail of the pancreas from *Ins2-DreER;Ngn3-2A-CreER;NR1* mice 2 weeks after PDL injury. (**M**) Immunostaining for tdT, zsGreen and Insulin on sections of unligated head of the pancreas from *Ins2-DreER;Ngn3-2A-CreER;NR1* mice. (**N**) Immunostaining for tdT, zsGreen and Insulin on sections of ligated tail of the pancreas from *Ins2-DreER;Ngn3-2A-CreER;NR1* mice. (**O**) Quantification of the percentages of ZsGreen$^+$ cells in Ins$^+$ pancreatic beta cells of unligated head and ligated tail of the pancreas from *Ins2-DreER;Ngn3-2A-CreER;NR1* mice. Data are mean ± SD; $n = 5$ biological replicates. In each sample, islets from 10 pancreas sections were quantified. Scale bars, 100 μm. Each image is representative of 4–5 individual mouse samples.

 © The Author(s)

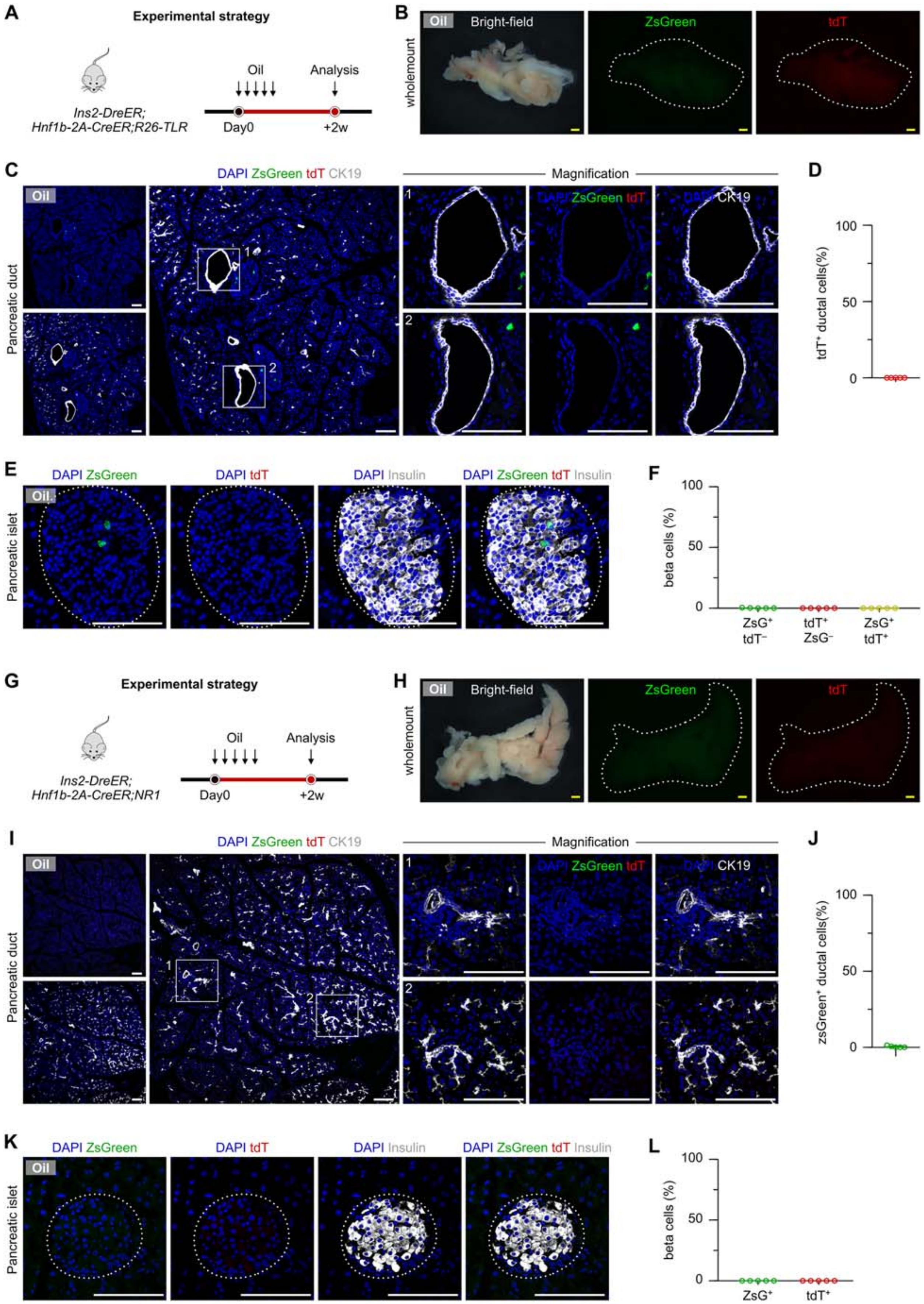
A
Experimental strategy
Oil
Analysis
Ins2-DreER;
Hnf1b-2A-CreER;R26-TLR
Day0
+2w
B
Oil
Bright-field
ZsGreen
tdT
wholemount
C
DAPI ZsGreen tdT CK19
Magnification
Oil
Pancreatic duct
1
2
D
tdT+ ductal cells(%)
100
50
0
E
DAPI ZsGreen
DAPI tdT
DAPI Insulin
DAPI ZsGreen tdT Insulin
Oil
Pancreatic islet
F
beta cells (%)
100
50
0
ZsG+ tdT−
tdT+ ZsG−
ZsG+ tdT+
G
Experimental strategy
Oil
Analysis
Ins2-DreER;
Hnf1b-2A-CreER;NR1
Day0
+2w
H
Oil
Bright-field
ZsGreen
tdT
wholemount
I
DAPI ZsGreen tdT CK19
Magnification
Oil
Pancreatic duct
1
2
J
zsGreen+ ductal cells(%)
100
50
0
K
DAPI ZsGreen
DAPI tdT
DAPI Insulin
DAPI ZsGreen tdT Insulin
Oil
Pancreatic islet
L
beta cells (%)
100
50
0
ZsG+
tdT+

Figure EV5. Characterization of *Ins2-DreER;Hnf1b-2A-CreER;R26-TLR* and *Ins2-DreER;Hnf1b-2A-CreER;NR1* without tamoxifen treatment, related to Figs. 5 and 6.

(**A**) Schematic showing the experimental strategy of *Ins2-DreER;Hnf1b-2A-CreER;R26-TLR* with oil treatment. (**B**) Whole-mount fluorescent images of pancreas from *Ins2-DreER;Hnf1b-2A-CreER;R26-TLR* after oil treatment. (**C**) Immunostaining for ZsGreen, tdT and CK19 on pancreatic sections of *Ins2-DreER;Hnf1b-2A-CreER;R26-TLR* after oil treatment. (**D**) Quantification of the percentage of tdT^+ cells in $CK19^+$ pancreatic ductal cells of *Ins2-DreER;Hnf1b-2A-CreER;R26-TLR* after oil treatment. Data are mean ± SD; $n = 5$ biological replicates. In each sample, islets from 10 pancreas sections were quantified. (**E**) Immunostaining for ZsGreen, tdT and Ins on pancreatic sections of *Ins2-DreER;Hnf1b-2A-CreER;R26-TLR* after oil treatment. (**F**) Quantification of the percentage of $ZsGreen^+tdT^-$ or $tdT^+ZsGreen^-$ or $ZsGreen^+tdT^+$ cells in Ins^+ pancreatic beta cells of *Ins2-DreER;Hnf1b-2A-CreER;R26-TLR* after oil treatment. Data are mean ± SD; $n = 5$ biological replicates. In each sample, islets from 10 pancreas sections were quantified. (**G**) Schematic showing the experimental strategy of *Ins2-DreER;Hnf1b-2A-CreER;NR1* with oil treatment. (**H**) Whole-mount fluorescent images of pancreas from *Ins2-DreER;Hnf1b-2A-CreER;NR1* after oil treatment. (**I**) Immunostaining for ZsGreen, tdT and CK19 on pancreatic sections of *Ins2-DreER;Hnf1b-2A-CreER;R26-NR1* after oil treatment. (**J**) Quantification of the percentage of $ZsGreen^+$ cells in $CK19^+$ pancreatic ductal cells of *Ins2-DreER;Hnf1b-2A-CreER;R26-NR1* after oil treatment. Data are mean ± SD; $n = 5$ biological replicates. In each sample, islets from 10 pancreas sections were quantified. (**K**) Immunostaining for ZsGreen, tdT and Ins on pancreatic sections of *Ins2-DreER;Hnf1b-2A-CreER;R26-NR1* after oil treatment. (**L**) Quantification of the percentage of $ZsGreen^+$ or tdT^+ cells in Ins^+ pancreatic beta cells of *Ins2-DreER;Hnf1b-2A-CreER;R26-NR1* after oil treatment. Data are mean ± SD; $n = 5$ biological replicates. In each sample, islets from 10 pancreas sections were quantified. Scale bars, yellow, 1 mm; white, 100 μm. Each image is representative of 5 individual samples.

© The Author(s)
